# Targeting serine hydroxymethyltransferases 1 and 2 for T-cell acute lymphoblastic leukemia therapy

**DOI:** 10.1038/s41375-021-01361-8

**Published:** 2021-08-02

**Authors:** Yana Pikman, Nicole Ocasio-Martinez, Gabriela Alexe, Boris Dimitrov, Samuel Kitara, Frances F. Diehl, Amanda L. Robichaud, Amy Saur Conway, Linda Ross, Angela Su, Frank Ling, Jun Qi, Giovanni Roti, Caroline A. Lewis, Alexandre Puissant, Matthew G. Vander Heiden, Kimberly Stegmaier

**Affiliations:** 1 Department of Pediatric Oncology, Dana-Farber Cancer Institute, and Division of Hematology/Oncology, Boston Children’s Hospital, Boston, MA USA; 2 Broad Institute of Massachusetts Institute of Technology and Harvard University, Cambridge, MA USA; 3 Graduate Program in Bioinformatics, Boston University, Boston, MA USA; 4 Koch Institute for Integrative Cancer Research at Massachusetts Institute of Technology, Massachusetts Institute of Technology, Cambridge, MA USA; 5 INSERM UMR 944, IRSL, St Louis Hospital, Paris, France; 6 Division of Cancer Biology, Dana-Farber Cancer Institute, Boston, MA USA; 7 Department of Medicine and Surgery, University of Parma, Parma, Italy; 8 Whitehead Institute for Biomedical Research, Cambridge, MA USA; 9 Department of Medical Oncology, Dana-Farber Cancer Institute, Boston, MA USA

**Keywords:** Leukaemia, Cancer metabolism

## Abstract

Despite progress in the treatment of acute lymphoblastic leukemia (ALL), T-cell ALL (T-ALL) has limited treatment options, particularly in the setting of relapsed/refractory disease. Using an unbiased genome-scale CRISPR-Cas9 screen we sought to identify pathway dependencies for T-ALL which could be harnessed for therapy development. Disruption of the one-carbon folate, purine and pyrimidine pathways scored as the top metabolic pathways required for T-ALL proliferation. We used a recently developed inhibitor of SHMT1 and SHMT2, RZ-2994, to characterize the effect of inhibiting these enzymes of the one-carbon folate pathway in T-ALL and found that T-ALL cell lines were differentially sensitive to RZ-2994, with the drug inducing a S/G2 cell cycle arrest. The effects of SHMT1/2 inhibition were rescued by formate supplementation. Loss of both SHMT1 and SHMT2 was necessary for impaired growth and cell cycle arrest, with suppression of both SHMT1 and SHMT2 inhibiting leukemia progression in vivo. RZ-2994 also decreased leukemia burden in vivo and remained effective in the setting of methotrexate resistance in vitro. This study highlights the significance of the one-carbon folate pathway in T-ALL and supports further development of SHMT inhibitors for treatment of T-ALL and other cancers.

## Introduction

Metabolic reprogramming is a hallmark of cancer. As early as the 1920s, Warburg et al. observed that tumor cells consume glucose at a high rate and do fermentation even in the presence of oxygen [1]. In the 1940s, the development of aminopterin, which was found to target dihydrofolate reductase (DHFR), a cytoplasmic enzyme involved in one-carbon folate metabolism, yielded the first remission in a child with acute lymphoblastic leukemia (ALL) [2]. Other folate derivatives, such as methotrexate, were later developed and have transformed the treatment of certain cancers.

T-cell ALL (T-ALL) is a highly aggressive cancer characterized by rapid proliferation of early lymphoid cells with immature T-cell surface markers. High NOTCH1 signaling is instructive toward normal T-cell lineage development. Early T-cell precursors express the NOTCH1 receptor and rely on expression of NOTCH1 ligand ∆-like 4 on thymic epithelial cells [3, 4]. As T cells progress through thymic development, T-cell receptor (TCR) rearrangements are coordinated with NOTCH1 signaling and rapid cell proliferation in the thymus. Moreover, T-cell activation and development rely on other coordinated pathways, including one-carbon folate, serine biosynthesis, and mitochondrial proliferation [5, 6]. T-ALL results from the accumulation of mutations affecting cell growth, proliferation and differentiation during this highly coordinated proliferative process. Over 70% of T-ALLs, for example, have mutations affecting *NOTCH1* or the NOTCH1 signaling pathway, such as *FBXW7*, highlighting the early normal proliferative signals gone awry [7].

MYC signaling is also essential for T-ALL pathogenesis, and MYC is activated by NOTCH1 [8]. As a master regulator of cell proliferation, cell cycle progression, genetic instability, and metabolism [9], MYC commonly plays a role in cancer pathogenesis. It stimulates expression of many nuclear encoded mitochondrial genes, regulates mitochondrial biogenesis [10], and is implicated in controlling the one-carbon folate pathway, especially in hypoxic conditions [11, 12]. In the context of acute myeloid leukemia (AML), we previously demonstrated that MYC binds at promoter sites of one-carbon folate pathway enzymes, such as serine hydroxymethyltransferase 2 (SHMT2), NAD-dependent mitochondrial methylenetetrahydrofolate dehydrogenase/cyclohydrolase (MTHFD2) and methylenetetrahydrofolate dehydrogenase 1 like (MTHFD1L) [13].

While cure rates for pediatric ALL have improved dramatically over the last several decades, leukemia remains the second leading cause of cancer-related death in children [14]. Adult patients with ALL continue to have poor prognosis even with intensive therapy. T-ALL, comprising 15–20% of ALL, is associated with early relapses and treatment resistance in the relapse setting [15, 16]. Moreover, while there is excitement surrounding immune-mediated therapies for B-cell ALL (B-ALL), including antibody-based and CAR T-cell therapies, these approaches are more limited for patients with T-ALL. Thus, more effective therapies are needed for T-ALL, particularly for patients with relapsed or refractory disease.

Development of one-carbon folate pathway inhibitors has yielded highly active drugs, such as methotrexate (targeting DHFR), 5-fluorouracil and pemetrexed (targeting thymidylate synthase and DHFR) and gemcitabine (a deoxycytidine analog). More recently, other de novo purine and pyrimidine synthesis inhibitors have been developed, such as dihydroorotate dehydrogenase (DHODH) inhibitors for cancer treatment [17] and plasmodial SHMT inhibitors for malaria treatment [18]. Further optimization of SHMT inhibitors has led to in vitro selective SHMT1/2 inhibitors, though testing of these compounds in vivo has been limited [19, 20].

In an unbiased CRISPR-Cas9 genome-scale screen to identify T-ALL selective pathway dependencies, we identified the one-carbon folate, purine and pyrimidine pathways as top hits. Using a combination of small molecule inhibitors and genetic suppression of SHMT, we validated the combined repression of SHMT1 and SHMT2 as a candidate therapeutic approach for T-ALL.

## Methods

### Cell culture, cell viability, and flow cytometry assays

Cell lines were maintained in RPMI 1640 (Cellgro) supplemented with 1% penicillin/streptomycin (PS) (Cellgro) and 10% FBS (Sigma-Aldrich) at 37 °C with 5% CO_2_. Viability was evaluated using the CellTiter-Glo Luminescent Cell Viability Assay (Promega) after the indicated treatment duration/concentration. Luminescence was measured using FLUOstar Omega (BMG Labtech). IC_50_ values were determined using Prism GraphPad version 8 software.

For cell cycle analysis, cells were harvested at the indicated time points, washed and fixed in ethanol and resuspended in 49 μg/mL propidium iodide (Sigma-Aldrich) and 100 μg/mL of RNase A (Qiagen). Cell death was assessed using flow cytometric analysis of Annexin V and propidium iodide staining according to the manufacturer’s instructions (eBioscience). Samples were analyzed on a FACSCanto analyzer (BD Biosciences). Data analysis used Flowjo software.

### Compounds

RZ-2994 was initially obtained from Raze Therapeutics [5] and subsequently from Medicilon. Identity was confirmed independently by liquid chromatography–mass spectrometry (LC–MS) and nuclear magnetic resonance spectroscopy performed by JQ (Dana-Farber Cancer Institute). Methotrexate was purchased from Sigma-Aldrich. Adavosertib, berzosertib, prexasertib, cytarabine and doxorubicin were purchased from Selleck. Etoposide was purchased from Cell Signaling and mercaptopurine from Santa Cruz.

### CRISPR-Cas9 screening

CRISPR-Cas9 DepMap screening was performed with the Avana library containing 73,372 guides for 18,333 genes, with an average of four guides per gene as previously described [21]. Analysis was performed using the 19Q4 version of the Gene Effect Avana data processed with the CERES algorithm [22], available at https://depmap.org/portal/. This data set contained 689 cell lines, including three T-ALL lines: SUPT1, PF382, and HSB2, and 73 other hematopoietic cell lines. See Supplementary Methods for analysis details.

### Vectors and constructs

shRNA constructs targeting SHMT1 and SHMT2 were delivered via a LT3 GEPIR (SHMT1) or REVIR (SHMT2) vectors as previously described [23] (Supplementary Table 1). For virus production, 12 μg of the above vectors with 6 μg pCMV8.9 and pCMV-VSVG packaging vectors were transfected into the 293 cells using X-tremeGENE 9 (Roche), and the resulting viral supernatants were harvested as previously described [13]. sgRNA constructs were designed using the Broad Institute’s shRNA designer tool; sequences are in Supplementary Table 1.

### RNASeq

KOPTK1 cells were grown in the presence of 2 µM RZ-2994 vs. DMSO and cells collected at 24 and 72 h of treatment, in triplicate. RNA was extracted from cells using an RNeasy Kit (Qiagen) and was sequenced using Illumina TruSeq strand specific library. The RNA-Seq data for this study is available for download from the Gene Expression Omnibus (GEO) repository https://www.ncbi.nlm.nih.gov/geo/ (GSE143176) and analyses are described in Supplementary Methods.

### Single-sample gene set enrichment analysis for T-ALL dependencies

A single-sample GSEA (ssGSEA) analysis [24, 25] was performed on the CERES dependency data across the collection of 186 KEGG pathways available from the MSigDB v7.0 database to further analyze the functional association of the amino-acid metabolic pathways with T-ALL dependencies. See Supplementary Methods for analysis details.

### Metabolite profiling and analysis

One million cells per condition in triplicate were pelleted and washed with ice cold saline. Cell pellets were resuspended in 1 mL of 80% methanol solution containing 500 nM internal standards (Metabolomics Amino Acid Mix, Cambridge Isotope Laboratories Inc.). Samples were vortexed at 4 °C, followed by centrifugation at 4 °C for 10 min. Supernatant was transferred, samples dried using a Speedvac, and resuspended in 50 μL HPLC grade water. LC–MS analysis is described in the Supplementary Methods.

### Immunoblotting

Cells were lysed in Cell Signaling Lysis Buffer (Cell Signaling Technology) [13], resolved by gel electrophoresis using Novex 4–12% Bis-Tris Gels (Invitrogen), transferred to a nitrocellulose membrane (Bio-Rad), and blocked for 1 h in 5% BSA (Sigma). Blots were incubated in primary antibody to SHMT1 (Cell Signaling, #80715), SHMT2 (Cell Signaling, #12762) or Vinculin (Cell Signaling, #13901), followed by the secondary antibodies anti-rabbit HRP (Amersham) or anti-mouse HRP (Amersham). Bound antibody was detected using the Western Lightning Chemiluminescence Reagent (Perkin Elmer).

### In vivo studies

For genetic inhibition studies, RPMI8402 cells were infected with lentivirus targeting renilla (CTL), SHMT1, SHMT2, or the combination, and cells selected. 750,000 cells were injected via tail vein into 8-week-old, female NSG mice (The Jackson Laboratory). Disease burden was followed using peripheral blood hCD45. At detection of at least 1% human cells, mice received doxycycline 2000 ppm chow and were treated for 9 days prior to disease assessment.

For the RZ-2994 study, 500,000 cells RPMI8402 luciferase-expressing cells were injected via tail vein into 8-week-old, female NSG mice (The Jackson Laboratory). Leukemia burden was assessed using bioluminescence imaging by injecting mice intraperitoneally with 75 mg/kg d-Luciferin (Promega), anesthetizing them with 2–3% isoflurane, and imaging them on an IVIS Spectrum (Caliper Life Sciences). A standardized region of interest (ROI) encompassing the entire mouse was used to determine total body bioluminescence, with data expressed as photons/s/ROI (ph/s/ROI). Upon detectable bioluminescence, mice were separated into two cohorts (RZ-2994 versus vehicle), 7 per cohort, and treated with RZ-2994100 mg/kg IP or vehicle daily for 14 days. Sample size was calculated to have 80% power to detect 1.75 SD difference between the two groups using a two-sided *t* test with *α* = 0.05. The study was not blinded. All animal studies were conducted under the auspices of protocols approved by the Dana-Farber Cancer Institute Animal Care and Use Committee.

### Drug interaction analysis

The expected dose-inhibitory fraction relationships for the combination therapy of RZ-2994 and methotrexate were assessed using the Bliss independence model [26, 27].

### Statistical analysis

Statistical significance was determined by two-tailed *t* test or Mann–Whitney test for pair-wise comparison of groups, as indicated. Statistical calculations were performed using Prism GraphPad version 8 software.

## Results

### One-carbon folate metabolism is a dependency in T-ALL

**Fig. 1:**
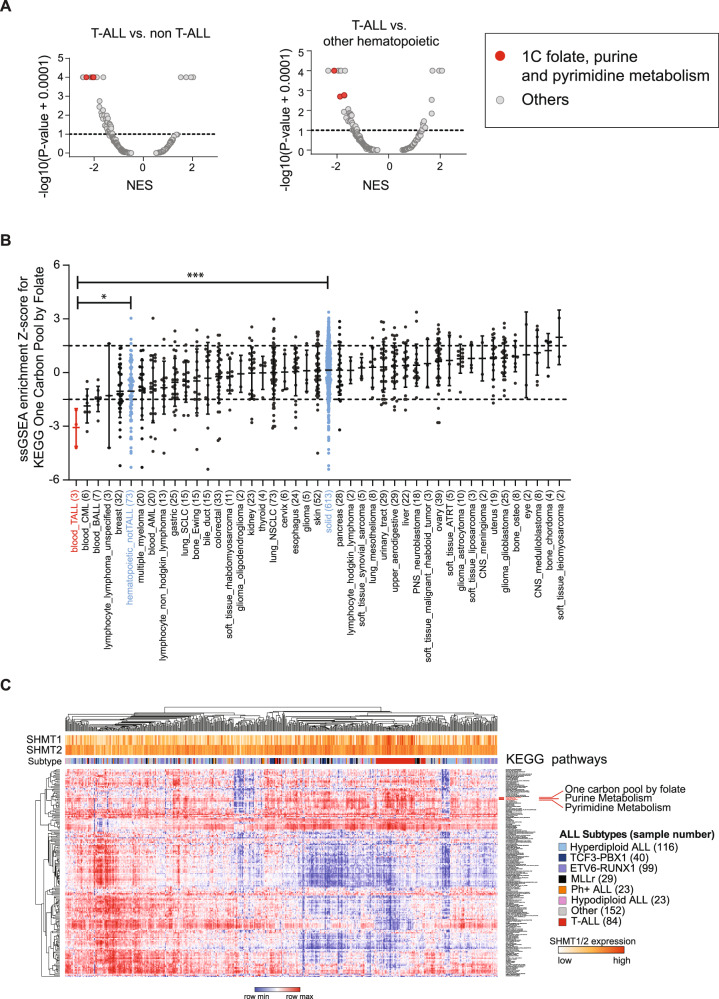
One-carbon folate metabolism is a dependency in T-ALL. **A** Volcano plots showing enrichment of KEGG pathways for 689 cell lines in the Avana 19Q4 data set. The KEGG one-carbon pool by folate, purine and pyrimidine metabolism pathways scored as most depleted in the T-ALL lineage (*n* = 3) compared to all other cell lines (*n* = 686) (left) (*P* = 0.0003, Mann–Whitney test) and to other hematopoietic cell lines (*n* = 73) (right) (*P* = 0.025, Mann–Whitney test). Normalized enrichment score (NES) shown on X-axis. **B** Graph showing the distribution of the ssGSEA Z-scores for the one-carbon pool by folate pathway across cancer cell lineages represented in the Avana 19Q4 data set. The one-carbon pool by folate pathway is significantly enriched in T-ALL vs. non-T-ALL hematopoietic (*P ≤ 0.05, Mann–Whitney test) and T-ALL vs. solid tumor (***P ≤ 0.001, Mann–Whitney test) cell lines. **C** Heatmap of ssGSEA projections for the primary ALL sample data set GSE33315 on the collection of KEGG canonical pathways. T-ALL samples are highlighted in red.

To identify selective pathway dependencies for T-ALL we used the CRISPR-Cas9 genome-scale screening data of 689 cancer cell lines from the Broad Institute’s Dependency Map [21]. Three T-ALL cell lines were screened in version 19Q4, in addition to 73 non-T-ALL hematopoietic and 613 solid tumor cell lines. GSEA was performed against 186 KEGG pathways to identify top negatively enriched pathways for T-ALL compared to other cancers. Inhibition of these pathways would be predicted to be more therapeutically effective in T-ALL compared to other tumors. Known T-ALL pathway dependencies, such as NOTCH signaling and TCR signaling, were also among the top 10 KEGG scoring pathways, serving as positive controls (Supplementary Table 2A, B). The one-carbon folate, purine and pyrimidine KEGG pathways scored among the top dependencies in T-ALL versus all other cancer and non-T-ALL hematopoietic cell lines (Fig. 1A). Each pathway was individually a significant dependency in T-ALL (Fig. 1B and Supplementary Fig. 1). To further validate this finding in a primary T-ALL data set, we performed ssGSEA on a human primary ALL gene expression data set (GSE33315, 566 ALL samples, including 84 T-ALL samples and 9 normal blood [28]) for enrichment across the 186 canonical KEGG pathways. T-ALL samples showed increased expression of genes involved in the one-carbon folate, purine and pyrimidine metabolism pathways, compared to B-ALL samples (Fig. 1C, Supplementary Table 3A, 4). We validated this finding in a second gene expression data set with 107 primary ALL samples, including 15 T-ALL (GSE13351, Supplementary Fig. 2A and Supplementary Table 3B) [29]. Both data sets showed a significant enrichment of the one-carbon folate pathway in T-ALL (*P* ≤ 0.0001, Supplementary Fig. 2B, C).

**Fig. 2:**
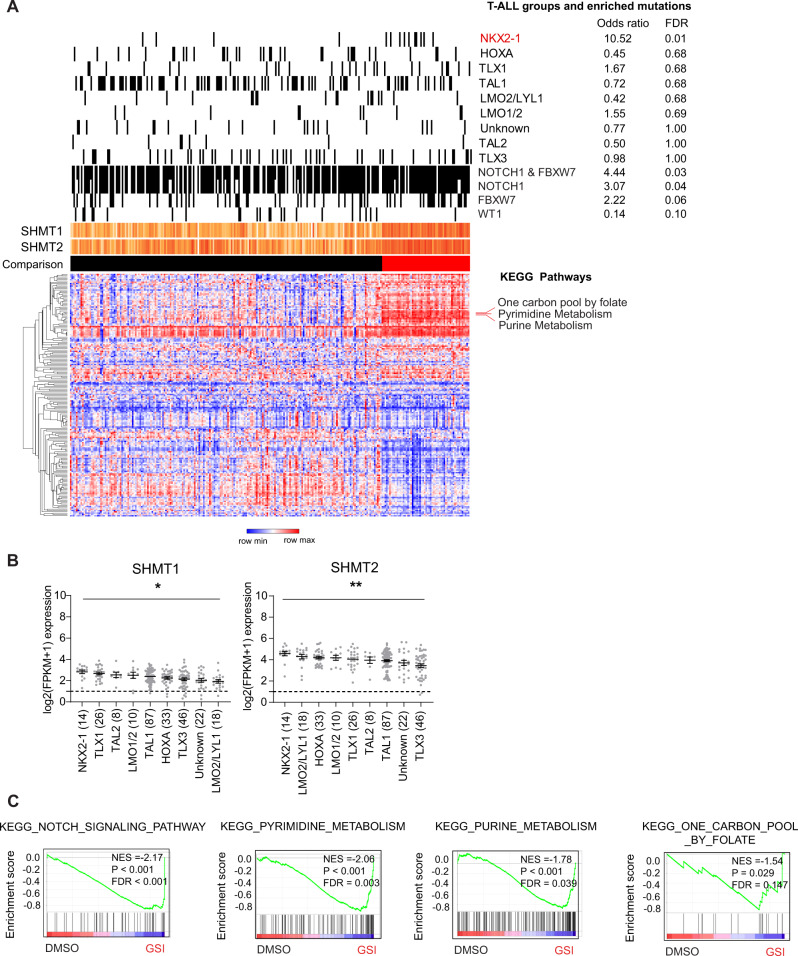
One-carbon folate metabolism in T-ALL is associated with NOTCH1 pathway mutations and the NKX2-1 group. **A** Heatmap of ssGSEA projection for the primary TARGET T-ALL sample data set on the collection of KEGG canonical pathways. T-ALL samples highlighted in red enriched for expression of the one-carbon folate, purine, and pyrimidine KEGG pathways (ssGSEA z-score > 1.5). **B** Graphs showing *SHMT1* and *SHMT2* expression across T-ALL genomic subgroups [7]. **C** Gene expression changes associated with GSI treatment of T-ALL cell lines show enrichment in NOTCH1 signaling, purine, pyrimidine, and one-carbon folate pathways.

We next probed the TARGET T-ALL data set for association between expression of the one-carbon folate, purine and pyrimidine pathways with previously defined T-ALL mutations or the eight gene expression-defined subgroups [7]. T-ALL with higher expression of these three pathways were enriched for *NOTCH1* and *FBXW7* mutations and anti-correlated with *WT1* mutations (Fig. 2A and Supplementary Table 3C). This subset of samples was also enriched for the NKX2-1 T-ALL subgroup (Fig. 2A) and had higher expression of *SHMT1* and *SHMT2* (Fig. 2B and Supplementary Fig. 3A). Both *SHMT1* and *SHMT2* are expressed in most T-ALL samples and all T-ALL cell lines profiled in CCLE (Supplementary Fig. 3B, C). Within T-ALL, expression of *SHMT1* and *SHMT2* was positively correlated within the GSE13351 and TARGET T-ALL data sets (Supplementary Fig. 3D). NOTCH1 signaling in T-ALL can be inhibited using gamma secretase inhibitors (GSI). Gene expression changes associated with GSI treatment of 10 T-ALL cell lines [30, 31] were also enriched for purine, pyrimidine and one-carbon folate pathways (Fig. 2C and Supplementary Table 5).

**Fig. 3:**
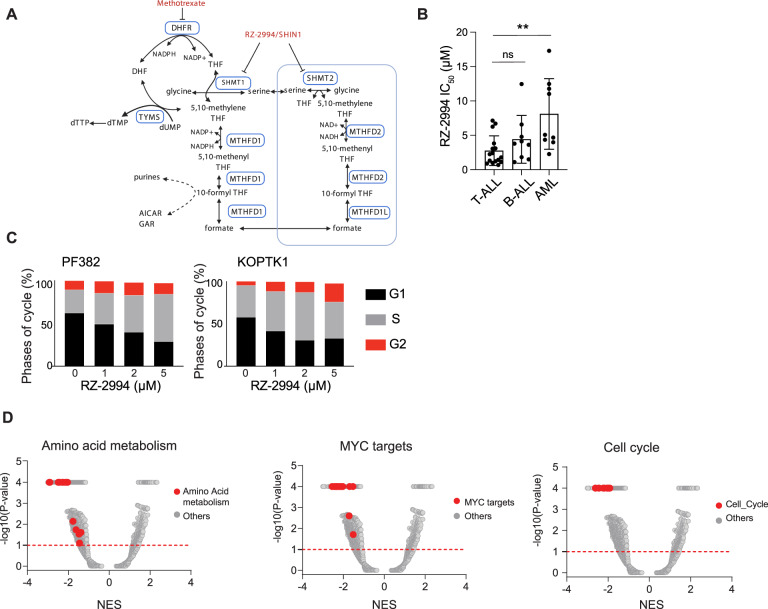
Enzymatic inhibition of SHMT1 and SHMT2 results in T-ALL arrest and gene expression changes. **A** Simplified schematic of the one-carbon folate pathway highlighting the targets for RZ-2994 and methotrexate. **B** T-ALL (*n* = 16), B-ALL (*n* = 9), and AML (*n* = 9) cell lines were treated with RZ-2994 in a range of concentrations, in quadruplicate for 6 days. Bar graph showing the average IC_50_ per lineage, with each dot representing the IC_50_ in a cell line. **P ≤ 0.01 using one-way ANOVA with post hoc multiple comparisons test. **C** Cell cycle analysis in T-ALL cells treated with increasing concentrations of RZ-2994. **D** RNAseq was performed for the KOPTK1 cell line treated with RZ-2994. Volcano plots showing quantitative comparison of gene sets from MSigDB v7.0 using ssGSEA. Volcano plots compare DMSO versus RZ-2994 after 3 days of treatment. All data sets above the dashed red line have *P* value ≤ 0.05.

Inhibitors of the one-carbon folate pathway, such as methotrexate and mercaptopurine, have formed the backbone of ALL therapy. There was significant correlation between expression of the purine or pyrimidine pathways versus one-carbon folate pathway across three primary leukemia sample and one cell line data sets (Supplementary Fig. 4A–D). In primary T-ALL data sets, expression of *SHMT1* and *SHMT2* consistently correlated with expression of the one-carbon folate pathway (Supplementary Fig. 4E–G). Given the selective dependency of T-ALL on the one-carbon folate pathway, strong correlation of SHMT1 and SHMT2 expression with enrichment for the one-carbon folate pathway in T-ALL, and availability of a novel inhibitor, we evaluated targeting this pathway using RZ-2994 [5] (also known as SHIN1 [19]). RZ-2994 is an inhibitor of cytoplasmic SHMT1 and mitochondrial SHMT2 serine hydroxymethyltransferases (Fig. 3A) [5, 19]. For comparison to other hematopoietic cell lines, we tested AML, B-ALL, and T-ALL cells for sensitivity to RZ-2994. The average IC_50_ of RZ-2994 in T-ALL was 2.8 μM, compared to 4.4 μM for B-ALL and 8.1 μM for AML cell lines (Fig. 3B and Supplementary Table 6). There was a strong correlation between the RZ-2994 dose response and IC_50_ in two T-ALL cell lines when measured using an ATP-based CellTiter-Glo assay and cell counting (Supplementary Fig. 5). Treatment of four T-ALL cell lines resulted in accumulation of cells in the S and G2 cell cycle phases with minimal apoptosis (Fig. 3C, D and Supplementary Fig. 6). We next evaluated the effects of SHMT1/2 inhibition on gene expression. We treated KOPTK1 cells with RZ-2994 for 1 and 3 days and performed RNA sequencing. RZ-2994 treatment resulted in changes in pathways associated with amino acid metabolism, MYC targets and cell cycle arrest (Fig. 3D and Supplementary Fig. 7, Supplementary Table 7A–D). These transcriptional changes were more pronounced after 3 days of treatment. There was no change in MYC levels (Supplementary Fig. 8). MYC gene signatures were enriched in genes down-regulated by RZ-2994 treatment, with about 30% of MYC target genes involved in nucleotide metabolism and mitochondrial pathways down-regulated by RZ-2994 (Supplementary Fig. 9).

**Fig. 4:**
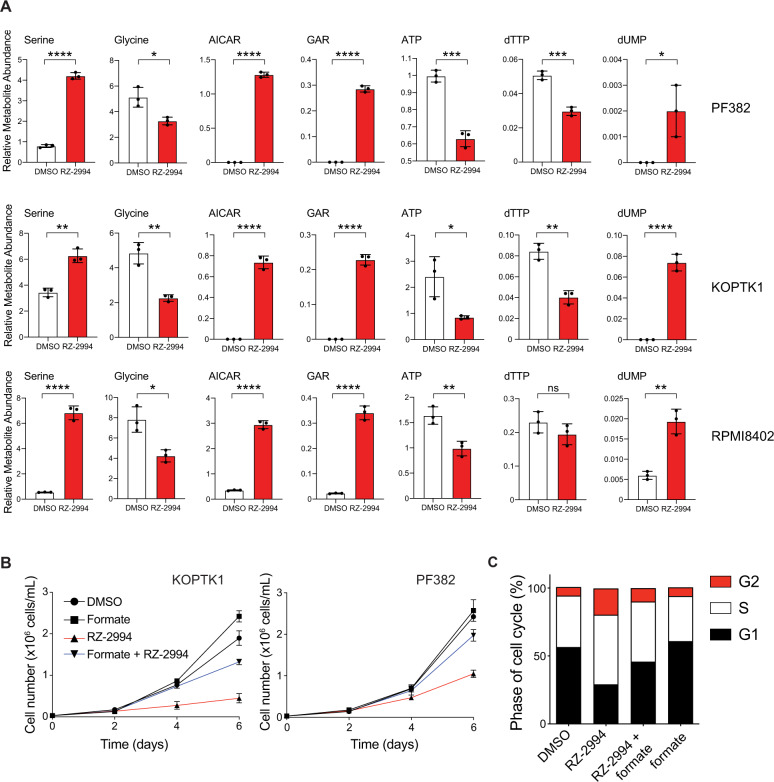
RZ-2994 causes metabolic changes in T-ALL, and its antiproliferative effects can be rescued with formate supplementation. **A** Bar graphs showing changes in metabolites associated with one-carbon folate metabolism following treatment with RZ-2994 in 3 cell lines. Cell lines were treated with 2 µM RZ-2994 for 3 days, metabolites extracted and profiled using LC–MS. Raw peak areas were normalized to internal standards. *P ≤ 0.05; **P ≤ 0.01; ***P ≤ 0.001; ****P ≤ 0.0001 using unpaired *t* test. **B** RZ-2994 leads to a decreased cell growth in T-ALL cell lines, and this growth defect can be rescued with supplementation of 1 mM formate. Graphs depict cell number as measured by trypan blue exclusion. Shown are the means ± SD of three replicates. **C** Cell cycle analysis in T-ALL cells treated with DMSO, RZ-2994 (2 µM), formate (1 mM), or the combination of RZ-2994 with formate.

Inhibition of SHMT1 and SHMT2 impairs glycine and formate synthesis, which in turn can impede nucleotide production [5, 19]. We performed metabolite profiling of PF382, KOPTK1, and RPMI8402 cell lines treated with RZ-2994 for 3 days and observed changes in intermediates that involve the one-carbon folate pathway (Fig. 4A and Supplementary Fig. 10). We focused on metabolic changes that were common among the three cell lines, as those are more likely to contribute to the RZ-2994-related effect on cell growth. Consistent with SHMT1 and SHMT2 inhibition, we found glycine levels were decreased with an increase in serine levels. We also observed increases in the purine precursors AICAR and GAR (Fig. 4A), which are upstream of steps in purine synthesis where one-carbon units are incorporated (Fig. 3A). There was also a decrease in ATP and dTTP. Thymidylate synthase requires 5,10-methylenetetrahydrofolate to synthesize dTMP from dUMP. Consistent with possible depletion of THF by SHMT1 and SHMT2 inhibition, there was an increase in dUMP (Fig. 4A). Addition of 1 mM formate rescued the proliferation of T-ALL cell lines in the presence of RZ-2994 (Fig. 4B and Supplementary Fig. 11), including rescue of cell cycle arrest (Fig. 4C).

### Loss of both SHMT1 and SHMT2 is necessary to impair proliferation of T-ALL

**Fig. 5:**
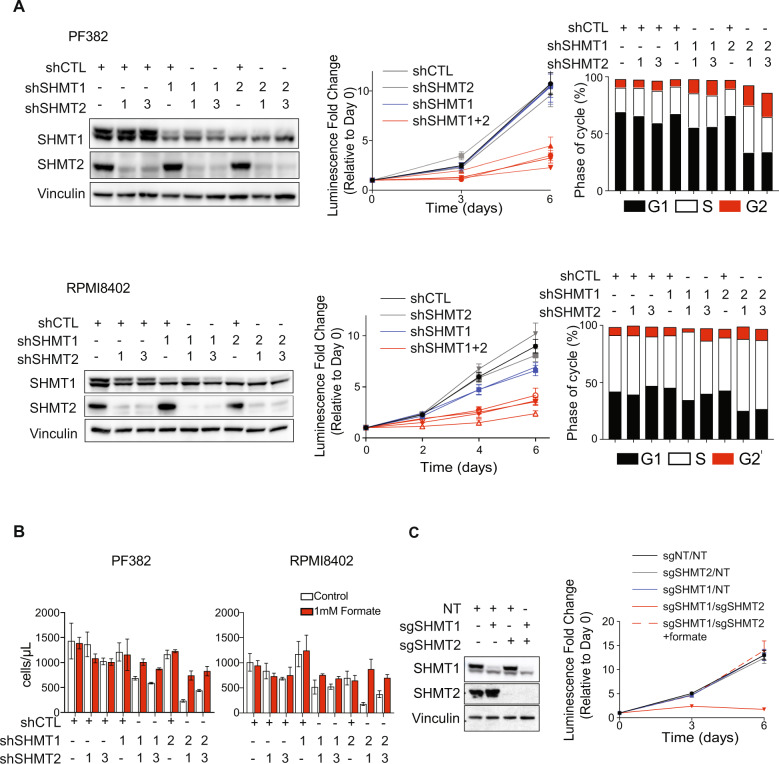
Repression of both SHMT1 and SHMT2 is required for T-ALL cell cycle arrest. **A** SHMT1 and SHMT2 targeting hairpins were used to knockdown SHMT1, SHMT2, or both in PF382 or RPMI8402 cells. On the left, western blot showing knockdown using shSHMT1-1 or shSHMT1-2 (labeled 1 or 2 in the SHMT1 row), or shSHMT2-1 or shSHMT2-3 (labeled 1 or 3 in the SHMT2 row). Addition of control vectors (shCTL) shown with +. In the middle images, cells were grown for 6 days and viability assessed by an ATP-based assay. Graphs depict luminescence fold change relative to Day 0. shSHMT2-1 and shSHMT2-3 are both labeled “shSHMT2” and colored gray. shSHMT1-1 and shSHMT1-2 are both labeled “shSHMT1” and colored blue. Cells with knockdown of both SHMT1 and SHMT2 are shown in red. Shown are the means ± SD of four replicates. On the right, bar graph showing cell cycle analysis after inducible shRNA knockdown. **B** PF382 or RPMI8402 cells were transduced with hairpins targeting SHMT1, SHMT2, or the combination and grown in media with or without formate supplementation for 6 days. Bar graphs show the means ± SD of three replicates. **C** Western blot showing knockout of *SHMT1*, *SHMT2*, or both using CRISPR guides in PF382 cells. Cells were grown over the course of 6 days and viability assessed by an ATP-based assay. Graphs depict luminescence fold change per cell line condition relative to Day 0.

Enzymes of the mitochondrial one-carbon folate pathway are highly expressed in cancer [32, 33], and their expression has been associated with poor survival [33]. We have previously shown MTHFD2 and enzymes of the one-carbon folate pathway to be highly expressed in AML, with inhibition of MTHFD2 leading to a decrease in AML viability in vitro and in vivo [13]. Redundancy of SHMT1 and SHMT2 enzymes has been shown in HEK293T and HCT-116 cells, though it is unclear if this occurs in leukemia [34]. Given that RZ-2994 inhibits both the cytoplasmic and mitochondrial SHMT enzymes, we evaluated if both need to be inhibited to impair proliferation of T-ALL cells. We used shRNA to knockdown *SHMT1* and *SHMT2* individually or both genes together. Repression of either *SHMT1* (Supplementary Fig. 12A) or *SHMT2* (Supplementary Fig. 12B) was not sufficient to impair cell proliferation. SHMT1 or SHMT2 individually were not dependencies in the DepMap (Supplementary Fig. 13). Instead, loss of both *SHMT1* and *SHMT2* was necessary for a full effect on proliferation and cell cycle (Fig. 5A). Supplementation with formate rescued the antiproliferative effect caused by repression of SHMT1 and SHMT2 (Fig. 5B). We also used CRISPR-Cas9 to knockout *SHMT1*, *SHMT2* or the combination, with an antiproliferative effect observed only upon loss of both *SHMT1* and *SHMT2*, and this effect was also rescued by formate supplementation (Fig. 5C).

### SHMT inhibition has in vivo efficacy in T-ALL

**Fig. 6:**
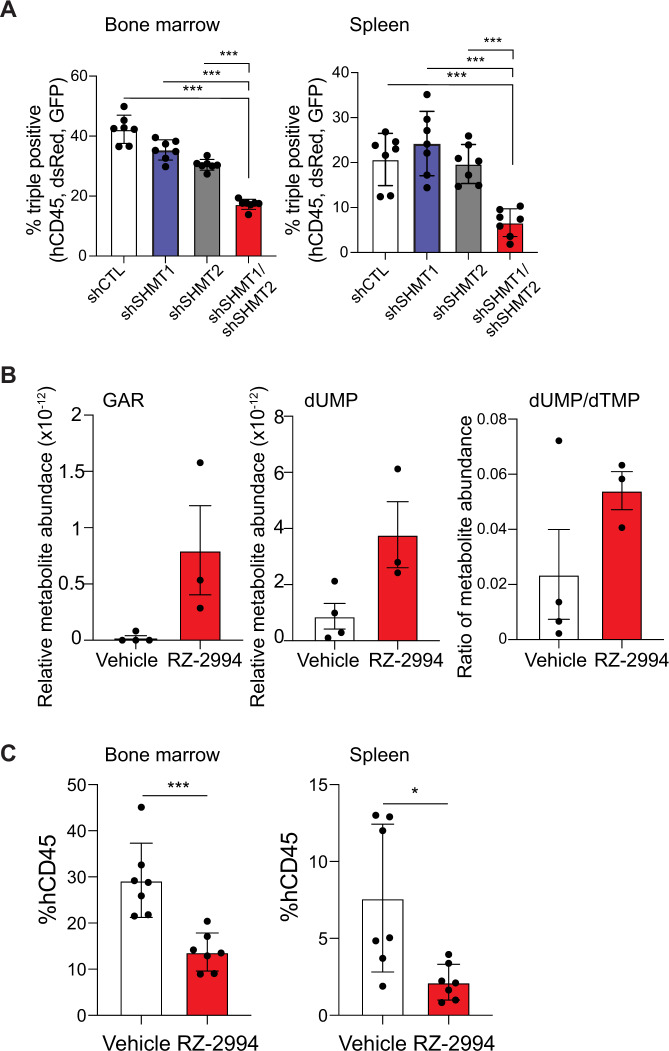
Knockdown and enzymatic inhibition of SHMT1 and SHMT2 are effective for T-ALL therapy in vivo. **A** Bar graph depicting percent of triple positive (hCD45+, GFP+, and dsRed+) cells in bone marrow and spleen. Shown is average with SD, *n* = 7 per group. *P ≤ 0.05; ***P ≤ 0.001 using Mann–Whitney test. **B** Polar metabolites were extracted from spleens of mice treated with RZ-2994 for 1 week and targeted profiling done using LC–MS. Bar graph shows relative metabolites compared to internal controls. Shown is average with SD, *n* = 4 for vehicle samples, and *n* = 3 for RZ-2994 samples. **C** Irradiated NSG mice were injected with RPMI8402-lucNeo cells. After disease was established, mice were treated with RZ-2994. Bar graph showing percent of hCD45+ cells in bone marrow and spleen after treatment with RZ-2994 100 mg/kg daily for 2 weeks. *P ≤ 0.05; ***P ≤ 0.001 using Mann–Whitney test.

In order to study the effects of SHMT1 and SHMT2 suppression after the development of T-ALL in vivo, we deployed a doxycycline-inducible shRNA system directed against SHMT1 and SHMT2 in RPMI8402. Leukemia establishment in NSG mice was confirmed by hCD45 detection in peripheral blood, and then mice were treated with doxycycline containing chow for 9 days until disease progression. Cells induced for SHMT1 repression (or its control) become GFP+, while SHMT2 repression (or its control) resulted in DsRed expression (Supplementary Fig. 14A). At time of disease evaluation, GFP+ and dsRed+ hCD45+ cells were selected by flow cytometry (Supplementary Fig. 14B). Suppression of both SHMT1 and SHMT2 led to a competitive disadvantage, with a decrease in these double knockdown cells compared to controls (Fig. 6A).

RZ-2994 was reported to have limited stability in liver microsome assays [19], though related pyrazolopyrans have shown modest efficacy in vivo for malaria treatment with oral dosing [18]. We thus performed pharmacokinetic analysis of RZ-2994 to assess its bioavailability for use as a tool compound in vivo. After injection of RZ-2994 20 mg/kg IP (intraperitoneally), blood levels were measured over time, with a *t*_1/2_ = 5.9 h (Supplementary Fig. 14C). In a dose-escalation study treatment up to 100 mg/kg for 1 week in NSG mice was tolerated without overt toxicity. To assess target inhibition, we evaluated the impact of RZ-2994 on metabolic targets in an orthotopic T-ALL model. In line with the in vitro data, RZ-2994 led to a trend toward increased GAR and dUMP, and an increase in the dUMP/dTMP ratio, consistent with disrupting one-carbon folate metabolism in vivo (Fig. 6B).

We next investigated the in vivo efficacy of RZ-2994 in a T-ALL animal model. Luciferase- expressing RPMI8402 cells were injected via tail vein into irradiated NSG mice. Leukemia establishment was determined using bioluminescent imaging, and mice were randomized into two groups, vehicle versus RZ-2994 treatment once disease was established. Mice were treated with 100 mg/kg IP daily for 2 weeks and disease burden evaluated. The drug was well tolerated, with stable weights in both cohorts (Supplementary Fig. 14D). RZ-2994 treatment led to a decrease in leukemia burden in the bone marrow and spleen (Fig. 6C), supporting further compound optimization and preclinical evaluation of this pathway in T-ALL.

### SHMT inhibition is efficacious in the setting of methotrexate resistance

**Fig. 7:**
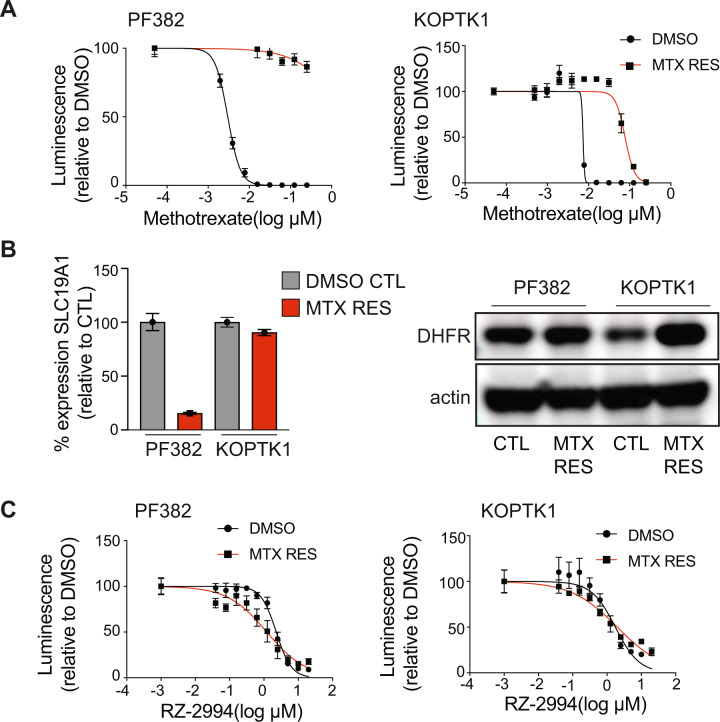
RZ-2994 is effective in the setting of methotrexate resistance. **A** PF382 and KOPTK1 cells were grown to methotrexate resistance. Parental and methotrexate resistant cells were tested with a range of methotrexate concentrations and viability evaluated at day 6 by an ATP-based assay as the percentage of viable cells relative to a DMSO control. Shown are the mean ± SD of four replicates. **B** On the left, bar graph showing *SLC19A1* expression in methotrexate resistant PF382 and KOPTK1 cells compared to controls as mean expressions ± SD of four replicates. On the right, western blot showing level of expression of DHFR in methotrexate resistant cells compared to parental cell lines. **C** Methotrexate resistant PF382 and KOPTK1 cells were tested for sensitivity to RZ-2994 with a range of concentrations and viability evaluated at day 6 by an ATP-based assay as the percentage of viable cells relative to a DMSO control. Shown are the mean ± SD of four replicates.

Methotrexate is a backbone of ALL treatment. Although testing of methotrexate sensitivity is not done routinely, ALL at the time of relapse has been shown to be relatively methotrexate resistant [35, 36]. Although highly effective in upfront therapy, inhibitors of the one-carbon folate pathway are not typically used at the time of relapse. We thus addressed whether targeting of SHMT1 and SHMT2 with RZ-2994 can be effective in the setting of methotrexate resistance. We developed methotrexate resistant cell lines by growing PF382 and KOPTK1 in increasing concentrations of methotrexate over several months (Fig. 7A). We explored which possible mechanisms of methotrexate resistance were elicited in these cells. Methotrexate resistant PF382 cells had decreased expression of *SLC19A1*, the major transporter of folate and methotrexate into the cell, while KOPTK1 cells had increased levels of DHFR, the target of methotrexate (Fig. 7B). Despite different mechanisms of methotrexate resistance, both cell lines remained sensitive to RZ-2994 (Fig. 7C).

Targeting a pathway at two different nodes can be clinically efficacious as demonstrated using the combination of methotrexate with mercaptopurine for treatment of ALL. We thus tested the combination of methotrexate with RZ-2994. We treated the PF382, KOPTK1, RPMI8402, and HSB2 cells with RZ-2994 in combination with methotrexate concurrently across a range of drug concentrations for 3 and 6 days. The combination of RZ-2994 with methotrexate was antagonistic at the highest concentration of methotrexate, including around the IC_50_, but showed synergy at lower concentrations of methotrexate (Supplementary Fig. 15) using the Bliss independence model of synergy. Given this mixed concentration-dependent response, caution would be needed in bringing this combination into a clinical setting.

**Fig. 8:**
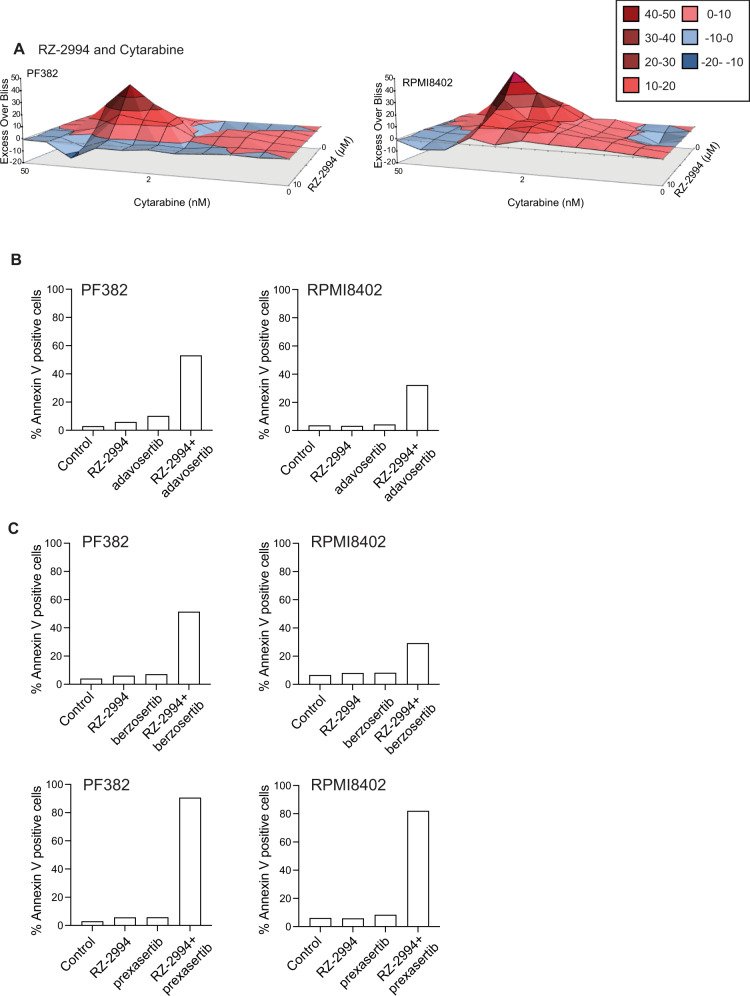
RZ-2994 is synergistic with cytarabine and inhibitors of the G2/M checkpoint. **A** Excess over Bliss analysis for the combination of RZ-2994 with cytarabine in PF382 and RPMI8402 cell lines. PF382 and RPMI8402 cells were treated with RZ-2994 (2 µM), adavosertib (125 nM), berzosertib (50 nM), prexasertib (5 nM), or the indicated combinations for 6 days and analyzed for apoptosis by flow cytometry. Bar graphs showing percent Annexin V positive cells for the combination of RZ-2994 with adavosertib (**B**), RZ-2994 with berzosertib or RZ-2994 with prexasertib (**C**). Shown is data from a representative experiment.

Given the mixed response with methotrexate, we tested the combination of RZ-2994 with other chemotherapy used to treat T-ALL. The combination of RZ-2994 with mercaptopurine, etoposide and doxorubicin were generally additive, while there was synergy when RZ-2994 was combined with cytarabine (Supplementary Fig. 16 and Fig. 8A). Since RZ-2994 contributes to S-phase arrest we hypothesized that the G2/M DNA damage checkpoint may be critical to cells treated with RZ-2994. We thus tested the combination of RZ-2994 with the WEE1 inhibitor, adavosertib. The combination of RZ-2994 was synergistic with adavosertib in T-ALL cell lines (Supplementary Fig. 17), with an increase in apoptosis (Fig. 8B). These findings also extended to the combination of RZ-2994 with other inhibitors of the S phase and G2/M DNA damage checkpoint, prexasertib, a CHK1 inhibitor and berzosertib, an ATR inhibitor (Fig. 8C). These studies may inform future effective therapy combinations with RZ-2994.

## Discussion

We identified the one-carbon folate pathway as an enriched pathway dependency in T-ALL. One-carbon folate metabolism is critical for nucleotide synthesis, support of cellular methylation reactions via methionine, and s-adenosyl methionine (SAM) production, redox regulation, and support of lipid metabolism [37]. The role of one-carbon folate metabolism in the mitochondrial compartment, and its potential contribution to cancer metabolic reprogramming, has only recently come to light with the finding that glycine, serine, and glutamine metabolism can play roles in oncogenesis [33, 38–40]. Although cytoplasmic components of the one-carbon folate pathway have long been targeted for cancer therapy, classically with drugs such as methotrexate and mercaptopurine, targeting the mitochondrial enzymes in this pathway has been understudied. Two such mitochondrial proteins, MTHFD2 and SHMT2, are among the most differentially expressed metabolic enzymes in cancer cells compared to normal cells [41, 42]. Both *MTHFD2* and *SHMT2* overexpression have been associated with tumor pathogenesis and poor survival [33, 43, 44].

Although acute leukemias are typically highly proliferative, the effect of one-carbon folate pathway inhibition using a novel inhibitor of SHMT1 and SHMT2, RZ-2994, was greater in T-ALL compared to AML. Over 70% of T-ALL have mutations leading to activation of NOTCH1 signaling, and this is associated with *MYC* overexpression. MYC controls critical metabolic processes, including the one-carbon folate pathway. In fact, inhibition of SHMT1 and SHMT2 recapitulated gene expression changes associated with MYC inhibition and may contribute to the differential sensitivity of T-ALL to one-carbon folate pathway inhibition.

The mechanistic role of SHMT1 and SHMT2 specific to T-ALL pathogenesis remains unknown. MTHFD2 is described as contributing to one-carbon unit production through conversion of serine to glycine by SHMT2 and production of formate as a product of MTHFD2 and MTHFD1L activity. The cytoplasmic arm of this pathway relies on SHMT1 and can also produce glycine and formate [34] intermediates contributing to the synthesis of purines/pyrimidines, as well as to the methionine and glutathione cycles. The direction of one-carbon and electron flow through this pathway, and the contribution of the cytoplasmic versus mitochondrial pathways, have been debated and may be different in cancer cells and under variable nutrient conditions [34, 42]. In T-ALL cell lines, formate supplementation rescued the effects of RZ-2994, as well combined SHMT1/SHMT2 knockdown. This contrasts with data in DLBCL, where a defect in exogenous glycine import impacts formate’s ability to rescue SHMT inhibition [19].

Inhibitors of the one-carbon folate pathway are used for treatment of cancers including ALL, osteosarcoma, breast, lung and many others. Resistance, however, is a key factor in treatment failure. There are numerous mechanisms of resistance to methotrexate, an inhibitor of the one-carbon folate pathway, including decrease in expression of the reduced folate carrier, increase in methotrexate polyglutamylation or increase in the expression of the drug target [35, 36, 45, 46]. We showed that T-ALL cell lines resistant to methotrexate, by two different mechanisms, remain sensitive to another inhibitor of the one-carbon folate pathway, RZ-2994. In fact, PF382 methotrexate resistant cells had a decreased expression of *SLC19A* but a twofold decrease in IC_50_ of RZ-2994. This may be related to a decrease in folate entry and increased dependency on SHMT1 [47]. Thus, it is possible that resistance to inhibition of the one-carbon folate pathway may be overcome with novel inhibitors of this pathway though further in vivo testing is necessary.

RZ-2994 was reported to have poor stability in liver microsome assays [19]. We performed a PK study, however, that showed a *t*_1/2_ = 5.9 h. Recently, an optimized derivative of RZ-2994, SHIN2, was shown to have efficacy in T-ALL models [20]. RZ-2994 causes cell cycle arrest and combinations with other drugs that cause cell death may be necessary to maximize efficacy. Often new drugs are combined with standard chemotherapy for treatment of patients with leukemia. However, the combination of RZ-2994 with standard chemotherapy, which is most toxic to rapidly proliferating cells, may be antagonistic. We found the combination of RZ-2994 to be highly synergistic with multiple inhibitors of the G2/M checkpoint and this may inform further preclinical testing of an optimized SHMT1/2 inhibitor in combination with inhibitors of this class, such as WEE1, CHK1, or ATR inhibitors.

In summary, the combination of unbiased genome-wide screening identifying the one-carbon folate pathway as a dependency in T-ALL, as well as the preclinical efficacy of SHMT1 and SHMT2 inhibition using both chemical and genetic approaches, support further optimization of SHMT1/2 inhibitors. Given the efficacy of other one-carbon folate pathway targeting drugs in cancer, novel inhibitors of this pathway are likely to be effective in other disease types, both at the time of diagnosis as well as at the time of relapse and development of drug resistance.

## Supplementary information

Supplementary Figure 1

Supplementary Figure 2

Supplementary Figure 3

Supplementary Figure 4

Supplementary Figure 5

Supplementary Figure 6

Supplementary Figure 7

Supplementary Figure 8

Supplementary Figure 9

Supplementary Figure 10

Supplementary Figure 11

Supplementary Figure 12

Supplementary Figure 13

Supplementary Figure 14

Supplementary Figure 15

Supplementary Figure 16

Supplementary Figure 17

Supplementary Figure Legends

Supplementary Methods

Supplementary Table 1

Supplementary Table 2

Supplementary Table 3

Supplementary Table 4

Supplementary Table 5

Supplementary Table 6

Supplementary Table 7
