# Supplementary figures and images for: Targeting serine hydroxymethyltransferases 1 and 2 for T-cell acute lymphoblastic leukemia therapy
Source: Leukemia. 2021 Aug 2;36(2):348–60. doi: 10.1038/s41375-021-01361-8 (PMC8807390; doi:10.1038/s41375-021-01361-8)

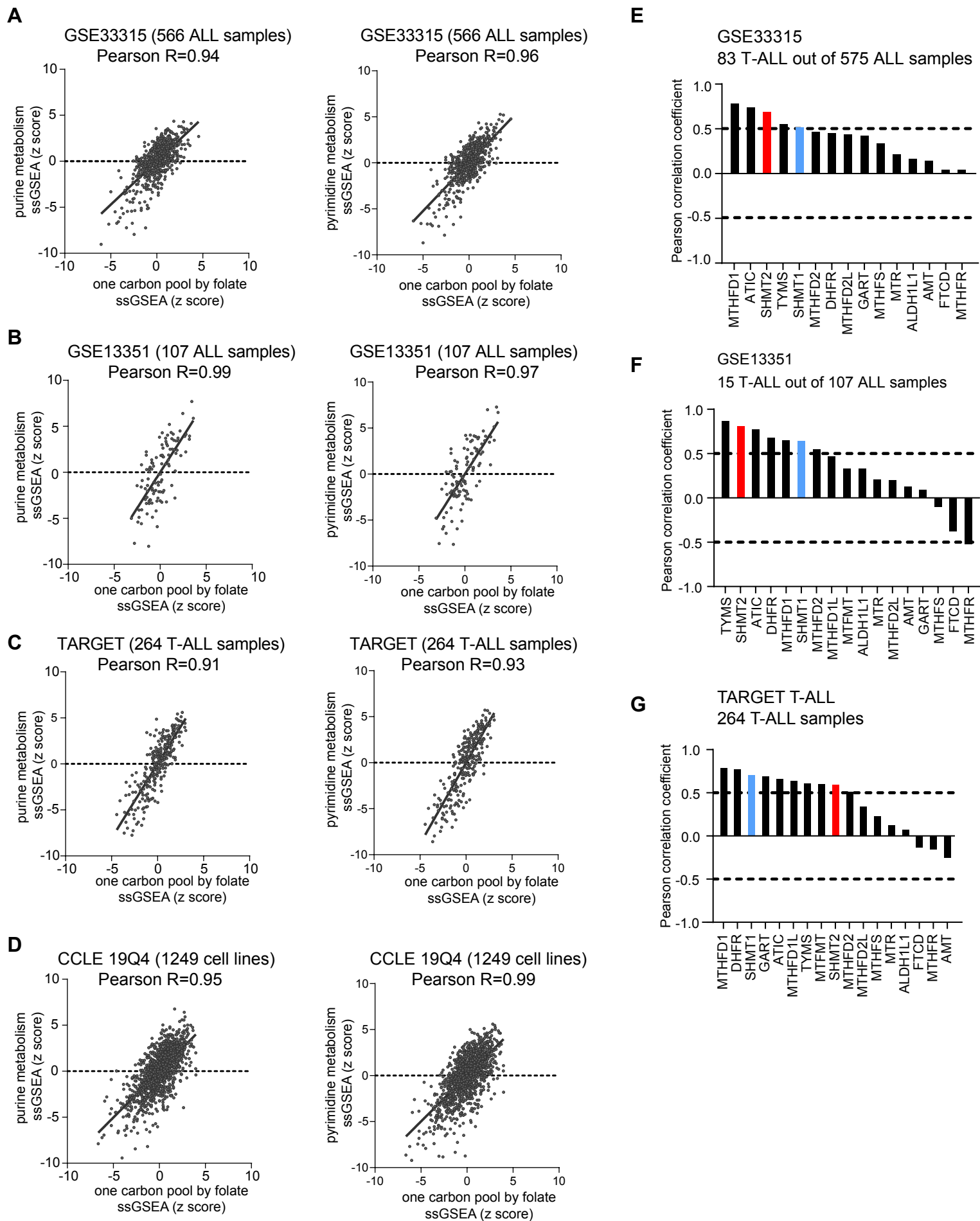

Supplementary Fig. 4

Supplement: Supplementary file 4 — Supplementary Figure 4 [file 41375_2021_1361_MOESM4_ESM.pdf]

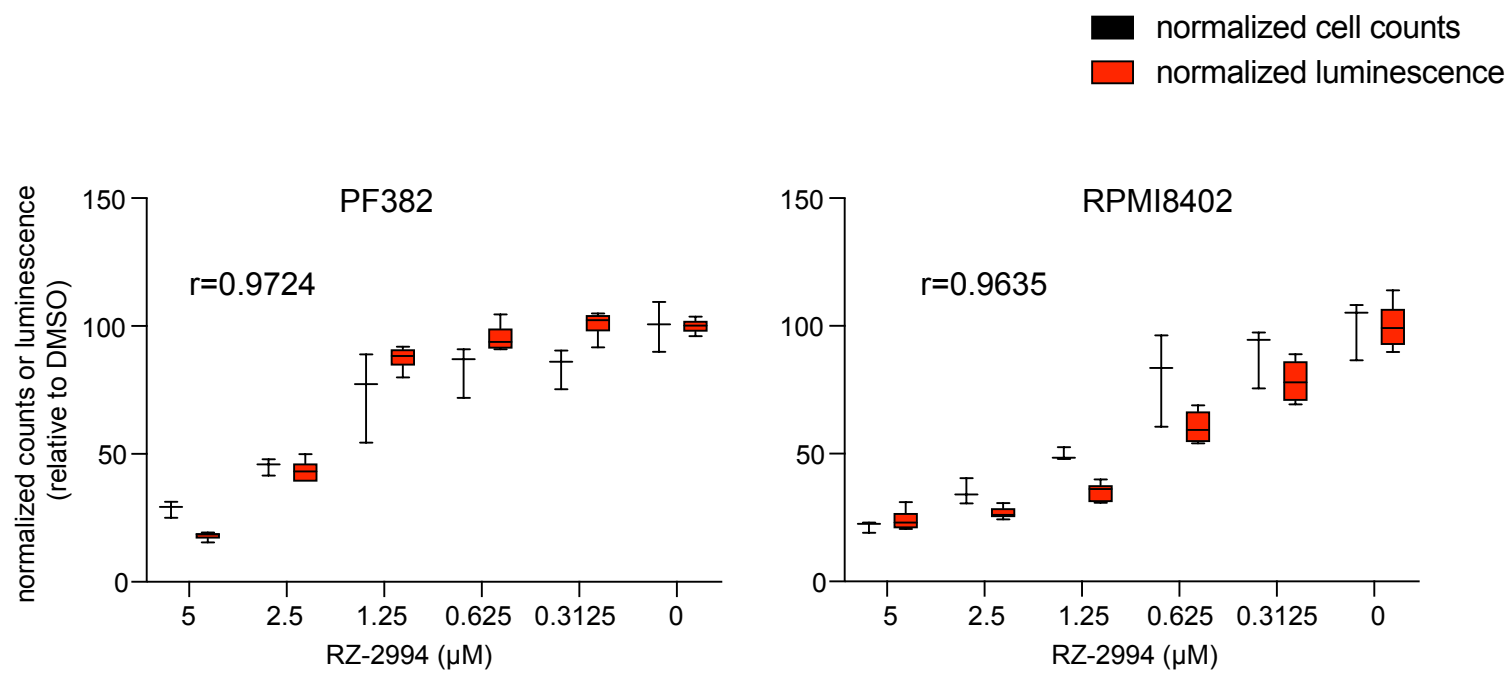

**Supplementary Fig. 5**

Supplement: Supplementary file 5 — Supplementary Figure 5 [file 41375_2021_1361_MOESM5_ESM.pdf]
