# Supplementary material for: Targeting serine hydroxymethyltransferases 1 and 2 for T-cell acute lymphoblastic leukemia therapy
Source: Leukemia. 2021 Aug 2;36(2):348–60. doi: 10.1038/s41375-021-01361-8 (PMC8807390; doi:10.1038/s41375-021-01361-8)
Supplement: Supplementary file 18 — Supplementary Figure Legends [file 41375_2021_1361_MOESM18_ESM.docx]

**Supplementary Figure Legends**

**Supplementary Figure 1:** Graphs showing distribution of the ssGSEA Z-scores for the purine and pyrimidine metabolism pathways across cancer cell lineages represented in the Avana 19Q4 data set. A) The purine metabolism pathway is significantly enriched in T-ALL vs non-T-ALL hematopoietic (**P ≤ 0.01, Mann-Whitney test) and T-ALL vs solid tumor (***P ≤ 0.001, Mann-Whitney test). B) The pyrimidine metabolism pathway is significantly enriched in T-ALL vs non-T-ALL hematopoietic (***P ≤ 0.001, Mann-Whitney test) and T-ALL vs solid tumor (***P ≤ 0.001, Mann-Whitney test).

**Supplementary Figure 2:** A) Heatmap of ssGSEA projection Z-scores for the primary ALL dataset GSE13351 on the collection of KEGG canonical pathways. T-ALL samples are highlighted in red. Graphs showing the distribution of the ssGSEA Z-scores for the one-carbon folate pathway in the GSE33315 (B) and GSE13351 (C) data sets (***P ≤ 0.001, Mann-Whitney test).

**Supplementary Figure 3:** A) Graph showing *SHMT1* and *SHMT2* expression in the T-ALL samples with one-carbon folate high group versus other T-ALL within the TARGET data set. B) *SHMT1* and *SHMT2* are expressed in most T-ALL samples within the TARGET data set and in all T-ALL cell lines available from CCLE (https://portals.broadinstitute.org/ccle) (C). D) Scatter plots showing Pearson correlation between expression of *SHMT1* and *SHMT2* in two T-ALL datasets.

**Supplementary Figure 4:** Expression of purine or pyrimidine KEGG pathways are highly correlated across datasets. Scatter plots showing Pearson correlation between ssGSEA Z-scores of purine metabolism or pyrimidine metabolism versus one-carbon folate metabolism for A) GSE33315, B) GSE13351, C) TARGET T-ALL, D) CCLE cell lines. Bar plots depicting the correlation between one-carbon folate pathway ssGSEA Z-scores versus individual gene expression for genes of the one-carbon folate pathway in T-ALL in: E) GSE33315 (83 T-ALL out of 575 ALL samples, Affy U133), F) GSE13351 (15 T-ALL of 107 ALL samples, Affy U133 Plus 2), and G) TARGET T-ALL (264 T-ALL samples, RNA-Seq). Shown are the Pearson correlation (R) coefficients for 1C ssGSEA Z-scores vs. log2(RMA) gene expression (GSE33315 and GSE13351) and for 1C ssGSEA Z-scores vs. log2(FPKM+1) gene expression (TARGET T-ALL). Cutoffs: positive correlation ≥ 0.5, negative correlation ≤ -0.5. SHMT2 is highlighted in red and SHMT1 is highlighted in blue.

**Supplementary Figure 5:** Graphs showing dose response of PF382 and RPMI8402 cells to RZ-2994. Cells were grown with increasing concentrations of RZ-2994 at indicated doses for 6 days. Data points are normalized to cell counts or luminescence of DMSO treated cells.

**Supplementary Figure 6:** (A) Cell cycle analysis in T-ALL cells treated with increasing concentrations of RZ-2994. (B) Bar graph showing percent Annexin V positive cells with increasing concentrations of RZ-2994 in T-ALL cell lines. Shown are the mean ± standard deviation (SD) of 3 replicates.

**Supplementary Figure 7:** RNAseq was performed for KOPTK1 cell line treated with RZ-2994 for 1 or 3 days. Gene expression changes associated with 3-day RZ-2994 treatment show enrichment for A) amino acid metabolism, B) MYC targets and C) cell cycle pathways. Cells were also profiled after 1-day of RZ-2994. Volcano plots showing quantitative comparison of gene sets from MSigDB v7.0 using ssGSEA. Volcano plots compare DMSO versus RZ-2994 after 1 day of treatment. All datasets above dashed red line have P-value ≤ 0.05. Gene expression changes associated with 1-day RZ-2994 treatment show enrichment for D) amino acid metabolism, E) MYC targets and F) cell cycle pathways. Top scoring GSEA plots are shown below the associated volcano plots.

**Supplementary Figure 8:** A) RNAseq was performed for KOPTK1 cell line treated with RZ-2994. Bar graphs showing *MYC* expression in KOPTK1 cells treated with RZ-2994 for 1 or 3 days. Expression shown as log2(TPM+1) from RNA-Seq experiment.

**Supplementary Figure 9:** Gene expression changes associated with RZ-2994 correlated with defined MYC and metabolic pathways. A) Heatmap showing top genes down regulated by RZ-2994 treatment in KOPTK1 cell line and enrichment of leading edge MYC pathways. ~30% of genes are represented by metabolic pathways, including purine and pyrimidine metabolism. B) Top metabolic pathways associated with MYC leading edge gene analysis.

**Supplementary Figure 10:** Bar graphs showing metabolites that were significantly changed with RZ-2994 treatment across 3 cell lines. Cell lines were treated with 2 µM RZ-2994 for 3 days, metabolites extracted and profiled using LC-MS. Raw peak areas were normalized to internal standards. *P ≤ 0.05, **P ≤ 0.01, ***P ≤ 0.001, ****P≤0.0001 using unpaired t-test.

**Supplementary Figure 11:** T-ALL cell lines were grown in a range of RZ-2994 concentrations, in regular media or supplemented with 1 mM formate, and viability evaluated at day 6 by an ATP-based assay as the percentage of viable cells relative to a DMSO control. Shown are the mean ± SD of 4 replicates.

**Supplementary Figure 12:** A) Western blot evaluating knockdown of SHMT1 in a PF382 cell line with five unique doxycycline-inducible shRNAs (shSHMT1-1, shSHMT1-2, shSHMT1-3, shSHMT1-4 and shSHMT1-5) compared to a control shRNA (shControl). Vinculin is used as a loading control. Cells were grown over the course of 6 days and viability assessed by an ATP-based assay. Graphs depict luminescence fold change per cell line condition relative to Day 0. Shown are the means ±SD of 4 replicates. B) Western blot evaluating knockdown of SHMT2 in the PF382 cell line with four unique doxycycline-inducible shRNAs (shSHMT2-1, shSHMT2-2, shSHMT2-3 and shSHMT2-5) compared to a control shRNA (shControl). Vinculin is used as a loading control. Cells were grown over the course of 6 days and viability assessed by an ATP-based assay. Graphs depict luminescence fold change per cell line condition relative to Day 0. Shown are the means ±SD of 4 replicates.

**Supplementary Figure 13:** Volcano plots showing effect size for *SHMT1* or *SHMT2* knockout in 689 cell lines in the Avana 19Q4 dataset. The differential dependency gene level scores for the T-ALL lineage were determined for T-ALL vs. all other non T-ALL cell lines (A), and also for T-ALL vs. all other non T-ALL hematopoietic cell lines (B), in order to eliminate the bias induced by the hematopoietic lineage. Graph showing CERES dependency score versus dependency probability for *SHMT1* (C) or *SHMT2* (D) knockout in 689 cancer cell lines screened as part of the Avana 19Q4 data set. T-ALL cell lines are indicated in red. Dotted lines indicate level of significant dependency (CERES score<-0.5) or proliferative advantage (CERES score >0.5). Dependency score greater than 0.5 is defined as a “dependency”, representing greater than 50% chance of being a dependency.

**Supplementary Figure 14:** A) Schematic showing SHMT1 and SHMT2 targeting doxycycline inducible constructs which were used for the cell line and mouse experiments. B) Sample flow plot showing schema for cell selection with both SHMT1 and SHMT2 hairpins (or associated controls) for the *in vivo* study. C) Graph showing blood RZ-2994 concentrations over time after a single dose of 20 mg/kg IP. Shown is the average ± SD, n=3 per group. D) Irradiated NSG mice were injected with RPMI8402-lucNeo cells. After disease was established, mice were treated with RZ-2994. Graph showing weights relative to Day 0 of treatment. Shown is average with SD, n=7 per group. After 2 weeks of treatment, leukemia burden was assessed.

**Supplementary Figure 15:** A) Excess over Bliss analysis for the combination of RZ-2994 with methotrexate in PF382, KOPTK1, RPMI8402 and HSB2 cells treated for 6 days in replicates of 4.

**Supplementary Figure 16:** Excess over Bliss analysis for the combination of RZ-2994 with mercaptopurine (A), doxorubicin (B) or etoposide (C) in PF382 and RPMI8402 cell lines.

**Supplementary Figure 17:** A) Excess over Bliss analysis for the combination of RZ-2994 with adavosertib in PF382 and RPMI8402 cell lines.
