# Supplementary material for: Targeting serine hydroxymethyltransferases 1 and 2 for T-cell acute lymphoblastic leukemia therapy
Source: Leukemia. 2021 Aug 2;36(2):348–60. doi: 10.1038/s41375-021-01361-8 (PMC8807390; doi:10.1038/s41375-021-01361-8)
Supplement: Supplementary file 19 — Supplementary Methods [file 41375_2021_1361_MOESM19_ESM.docx]

**Supplementary Methods**

**Cell Culture**

The human cell lines MOLT16 and RCH-ACV were purchased from Leibniz-Institut DSMZ-German collection of microorganisms and cell cultures. Loucy, MOLM-13, U937, HL-60, KG1, REH cell lines were purchased from American Type Culture Collection (ATCC). Human cell lines DND41, KOPTK1, MOLT4, ALL-SIL, P12-Ichikawa, CCRF-CEM, SUPT-1, RPMI8402 and PF382 were provided by Dr. Jon Aster. SKW3, SUPT11 and HSB2 were provided by Dr. James Bradner, and Jurkat cells were provided by Dr. Nicholas Haining. NOTCH1 activation for cell lines HSB2, SUPT11 and Jurkat was confirmed by western blotting. SKW3 identify was confirmed by fluorescent in situ hybridization (FISH) for the known MYC-TCR translocation. THP-1, MV4-11, 697, SEM, SEMK2, NALM6, SUPB15 and MOLM-14 cells were provided by Scott Armstrong. HB11;19 and KOPN-8 cells were provided by Dr. Sarah Tasian. NOMO-1 and NB4 cells were provided by Dr. Ross Levine. All cell lines were verified using STR profiling and tested for mycoplasma.

**CRISPR-Cas9 Screening**

Initially, cancer cell lines were transduced with Cas9 using a lentiviral system. Cell lines that met quality criteria, including acceptable Cas9 measured ability to knockout transduced GFP, appropriate growth properties and other parameters, were then screened with the Avana library. A pool of guides was transduced into a population of cells. The cells were cultured for 21 days *in vitro*, and at the end of the assay, barcodes for each guide were sequenced for each cell line in replicate.

The sgRNA read count data were deconvoluted from sequence reads by using the PoolQ public software (<https://portals.broadinstitute.org/gpp/public/software/poolq>). A series of quality control pre-processing steps was performed to remove samples with poor replicate reproducibility, as well as guides that have low representation in the initial plasmid pool, as described by Dempster et al^1^. The raw read counts were summed up by replicate and guide and the log2-fold-change from pDNA counts for each replicate was computed. The sgRNAs with suspected off-target activity and the guides with pDNA counts less than one millionth of the pDNA pool were removed. The replicates that failed fingerprinting and the replicates with less than 15 million reads were removed, and then the replicate read counts were scaled to 1 million total reads per replicate. The replicates with the null-normalized mean difference (NNMD) greater then -1.0 were filtered out. Those replicates that did not have a sufficiently high Pearson coefficient (> 0.61) with at least one other replicate for the line when looking at genes with the highest variance (top 3%) in gene effect across cell lines were also removed. Then NNMD was computed again for each cell line after averaging remaining replicates, and the cell lines with NNMD > -1.0 were filtered out.

For quality control and normalization, exogenously defined nonessential genes^2^ were used as negative controls, and common essential genes^3,4^ were used as positive controls. The gene level dependency scores were inferred by running the computational tool CERES^5^. CERES was developed to computationally correct the copy-number effect and to infer true underlying effect of gene knockout. CERES models the observed normalized log-fold change for each sgRNA and cell line as the linear combination of gene-knockout and copy-number effects with coefficients giving the guide activities. Copy-number effects are fit with a linear piece-wise model in each cell line. Once all parameters have been fit, the inferred gene scores and guide activity scores are extracted and reported.

The CERES gene dependency data were further scaled to the -1 value of the median of common essential genes in each cell line, and then transformed into z-scores so that each gene had mean = 0 and variance = 1. Next, the first five principal components of the resulting data were removed, the prior means of genes were restored, and the data were scaled again so the median of common essentials in each cell lines was -1. The pan-dependent genes were identified as those genes for whom 90% of cell lines rank the gene above a dependency cutoff determined from the central minimum in a histogram of gene ranks in their 90th percentile least dependent line. For each CERES gene score, the probability that the score represents a true dependency or not was inferred based on the expectation-maximization algorithm.

The differential dependency gene level scores for the T-ALL lineage were determined for T-ALL vs. all other non T-ALL cell lines, and also for T-ALL vs. all other non T-ALL hematopoietic cell lines, in order to eliminate the bias induced by the hematopoietic lineage itself. The analysis was performed based on the empirical Bayes (eBayes) statistics available from the limma package^6^ (Bioconductor v3.10 <https://www.bioconductor.org/packages/release/bioc/html/limma.html>) with the significance cutoffs: abs(size effect) ≥ 0.3, P-value ≤ 0.05, adjusted P-value ≤ 0.10.

**RNAseq**

Quality control tests for the 75 bp single-end mapped reads were performed using the FASTQC software (www.bioinformatics.babraham.ac.uk/projects/fastqc/). The reads were aligned to the GRCh37/hg19 human genes by using STAR v2.7-2b^7^. Quality control tests for the aligned reads and for replicate consistency were performed by using the qualimap v2.2.1^8^ and the SARTools^9^ pipelines.

Gene level reads and gene level expression estimated as log2(1+TPM) scores – where TPM stands for Transcripts Per Million - were computed using the Feature Counts method implemented in the Bioconductor v3.10 RSubread package^10^. The overall significance of differential expression between the control (DMSO) and treatment (RZ-2994) phenotypes at each time point was estimated by using the apeglm method^11^ available from the DESeq2 library^12^ (Bioconductor v3.10). The transcriptional dynamics induced by RZ-2994 treatment show a weak effect at day 1 and a stronger effect at day 3. Consequently, the significance cut-offs were adjusted as follows: *relaxed* for day 1: abs(shrinkage fold change) ≥ 1.2, adjusted P-value ≤ 0.10, and *standard* for day 3: abs(shrinkage fold change) ≥ 1.5, adjusted P-value ≤ 0.10.

**Gene Set Enrichment Analysis (GSEA) for T-ALL Dependencies**

The GSEA v4.0.3 software^13,14^ was utilized to identify the Kyoto Encyclopedia of Genes and Genomes (KEGG) canonical pathways with a significant overlap with genes showing a differential dependency for T-ALL vs. non-T-ALL cell lines and for the T-ALL vs. non-T-ALL hematopoietic cell lines in the Avana 19Q4 DepMap data. Genes were ranked in decreasing order based on the T-ALL differential dependency scores. The Pre-Rank GSEA module was run across the collection of 186 KEGG pathways available in the MSigDB v7.0 database^13,15,16^ with the significance cut-offs nominal P-value ≤ 0.10 and FDR ≤ 0.25 for the Kolmogorov-Smirnov enrichment test. The significantly enriched pathways with the Normalized Enrichment Score (NES) ≤ -1.5 were annotated for “Depletion” in T-ALL and those with NES ≥ 1.5 were annotated for “Proliferation” in T-ALL. The KEGG pathways identified as significantly enriched in dependency genes for T-ALL were further manually annotated as related to the amino-acid metabolic functional category.

**Single-sample Gene Set Enrichment Analysis for** **T-ALL Dependencies**

ssGSEA is a variant of the GSEA method that assigns to each individual sample, represented as a ranked list of genes, an Enrichment Score (ES) with respect to each gene set in a given collection of pathways. The ssGSEA ES is calculated as a running sum statistic by walking down across the ranked list of genes, increasing the sum when encountering genes in the gene set and decreasing it when encountering genes not in the gene set. The significance of the ES is estimated based on a permutation P-value and adjusted for multiple hypotheses testing through FDR. A positive ES denotes a significant overlap of the signature gene set with groups of genes at the top of the ranked list, while a negative ES denotes a significant overlap of the signature gene set with groups of genes at the bottom of the ranked list.

For each sample, the ES is further transformed into a Z-score by subtracting the average of the ES’s assigned to all other samples and by dividing the result to their standard deviation. While GSEA generates a gene set’s enrichment score with respect to phenotypic differences across a collection of samples within a dataset, ssGSEA calculates a separate enrichment score for each pairing of sample and gene set, independent of phenotype labeling. In this manner, ssGSEA transforms a single sample's dependency profile to a gene set enrichment profile. A gene set's enrichment score represents the activity level of the biological process in which the gene set's members are coordinately scoring up or down. The ssGSEA gene set representation has an unsupervised biological interpretability and can be further analyzed with statistical and machine learning methods.

**Metabolite Profiling and Analysis**

LC-MS was performed using a QExactive orbitrap mass spectrometer using an Ion Max source and heated electro-spray ionization (HESI) probe coupled to a Dionex Ultimate 3000 UPLC system (Thermo Fisher Scientific). External mass calibration was performed every 7 days. Typically, samples were separated by chromatography by injecting 2 μL of sample on a SeQuant ZIC-pHILIC 2.1 mm x 150 mm (5 μm particle size) column. Samples were run at multiple dilutions to ensure linearity of all metabolites measured. Flow rate was set to 150 mL/min. and temperatures were set to 25°C for the column compartment and 4°C for the autosampler tray. Mobile phase A was 20 mM ammonium carbonate, 0.1% ammonium hydroxide. Mobile phase B was 100% acetonitrile. The chromatographic gradient was: 0–20 min.: linear gradient from 80% to 20% mobile phase B; 20–20.5 min.: linear gradient from 20% to 80% mobile phase B; 20.5 to 28 min.: hold at 80% mobile phase B. The mass spectrometer was operated in full scan, polarity-switching mode and the spray voltage was set to 3.0 kV, the heated capillary held at 275°C, and the HESI probe was held at 350C. The sheath gas flow rate was 40 units, the auxiliary gas flow was 15 units and the sweep gas flow was one unit. The MS data acquisition was performed in a range of 70–1000 m/z, and an additional narrow-range scan (220-700 m/z) was included in negative mode to enhance the detection of nucleotides. The resolution was set at 70,000, the AGC target at 1x106, and the maximum injection time at 20 msec. Relative quantitation of polar metabolites was performed with TraceFinder 4.1™ (Thermo Fisher Scientific) using a 5 ppm mass tolerance and referencing an in-house library of chemical standards. Peak areas were normalized to internal standards and cell number. We analyzed 20 metabolites relevant to the one-carbon folate pathway.

**References:**

1. Dempster, J.M.*, et al.* Extracting Biological Insights from the Project Achilles Genome-Scale CRISPR Screens in Cancer Cell Lines. (2019).

2. Hart, T., Brown, K.R., Sircoulomb, F., Rottapel, R. & Moffat, J. Measuring error rates in genomic perturbation screens: gold standards for human functional genomics. *Mol Syst Biol* **10**, 733 (2014).

3. Blomen, V.A.*, et al.* Gene essentiality and synthetic lethality in haploid human cells. *Science* **350**, 1092-1096 (2015).

4. Hart, T.*, et al.* High-Resolution CRISPR Screens Reveal Fitness Genes and Genotype-Specific Cancer Liabilities. *Cell* **163**, 1515-1526 (2015).

5. Meyers, R.M.*, et al.* Computational correction of copy number effect improves specificity of CRISPR-Cas9 essentiality screens in cancer cells. *Nat Genet* **49**, 1779-1784 (2017).

6. Ritchie, M.E.*, et al.* limma powers differential expression analyses for RNA-sequencing and microarray studies. *Nucleic Acids Res* **43**, e47 (2015).

7. Dobin, A.*, et al.* STAR: ultrafast universal RNA-seq aligner. *Bioinformatics* **29**, 15-21 (2013).

8. Okonechnikov, K., Conesa, A. & Garcia-Alcalde, F. Qualimap 2: advanced multi-sample quality control for high-throughput sequencing data. *Bioinformatics* **32**, 292-294 (2016).

9. Varet, H., Brillet-Gueguen, L., Coppee, J.Y. & Dillies, M.A. SARTools: A DESeq2- and EdgeR-Based R Pipeline for Comprehensive Differential Analysis of RNA-Seq Data. *PLoS One* **11**, e0157022 (2016).

10. Liao, Y., Smyth, G.K. & Shi, W. The R package Rsubread is easier, faster, cheaper and better for alignment and quantification of RNA sequencing reads. *Nucleic Acids Res* **47**, e47 (2019).

11. Zhu, A., Ibrahim, J.G. & Love, M.I. Heavy-tailed prior distributions for sequence count data: removing the noise and preserving large differences. *Bioinformatics* **35**, 2084-2092 (2019).

12. Love, M.I., Huber, W. & Anders, S. Moderated estimation of fold change and dispersion for RNA-seq data with DESeq2. *Genome Biol* **15**, 550 (2014).

13. Subramanian, A.*, et al.* Gene set enrichment analysis: a knowledge-based approach for interpreting genome-wide expression profiles. *Proceedings of the National Academy of Sciences of the United States of America* **102**, 15545-15550 (2005).

14. Mootha, V.K.*, et al.* PGC-1alpha-responsive genes involved in oxidative phosphorylation are coordinately downregulated in human diabetes. *Nature Genetics* **34**, 267-273 (2003).

15. Liberzon, A.*, et al.* The Molecular Signatures Database (MSigDB) hallmark gene set collection. *Cell Syst* **1**, 417-425 (2015).

16. Liberzon, A.*, et al.* Molecular signatures database (MSigDB) 3.0. *Bioinformatics* **27**, 1739-1740 (2011).
