# Supplementary material for: Targeting serine hydroxymethyltransferases 1 and 2 for T-cell acute lymphoblastic leukemia therapy
Source: Leukemia. 2021 Aug 2;36(2):348–60. doi: 10.1038/s41375-021-01361-8 (PMC8807390; doi:10.1038/s41375-021-01361-8)
Supplement: Supplementary file 21 — Supplementary Table 2 [file 41375_2021_1361_MOESM21_ESM.pdf]

Supplementary table 2A: Genome-wide gene set enrichment results for Avana 19Q4 CERES dependency of T-ALL vs. nonT-ALL cell lines across the collection of KEGG pathways (MSigDB v7). Shown for each gene set are the normalized enrichment score (NES), the nominal P-value, the Benjamini-Hochberg False Discovery Rate (FDR), the size and the dependency status (depletion - negatively enriched, proliferation - positively enriched). Significance cutoffs: abs(NES) >1.5, nominal P-value < 0.10, FDR < 0.25.

#	Gene set enrichment for CERES dependency of T-ALL vs. all other nonT-ALL cell lines	NES	P-value	FDR	Size gene set	Status
1	KEGG_SYSTEMIC_LUPUS_ERYTHEMATOSUS	-2.46	0.0000	0.0000	111	Depletion
2	KEGG_RIBOSOME	-2.44	0.0000	0.0000	85	Depletion
3	KEGG_PYRIMIDINE_METABOLISM	-2.31	0.0000	0.0000	93	Depletion
4	KEGG_SPLICEOSOME	-2.14	0.0000	0.0010	124	Depletion
5	KEGG_RNA_POLYMERASE	-2.09	0.0000	0.0008	26	Depletion
6	KEGG_NOTCH_SIGNALING_PATHWAY	-2.07	0.0000	0.0008	47	Depletion
7	KEGG_PURINE_METABOLISM	-2.06	0.0000	0.0007	151	Depletion
8	KEGG_ONE_CARBON_POOL_BY_FOLATE	-2.02	0.0000	0.0016	17	Depletion
9	KEGG_MTOR_SIGNALING_PATHWAY	-1.98	0.0000	0.0032	52	Depletion
10	KEGG_T_CELL_RECEPTOR_SIGNALING_PATHWAY	-1.89	0.0000	0.0079	107	Depletion
11	KEGG_PRIMARY_IMMUNODEFICIENCY	-1.79	0.0036	0.0248	35	Depletion
12	KEGG_PROTEASOME	-1.78	0.0017	0.0253	44	Depletion
13	KEGG_NICOTINATE_AND_NICOTINAMIDE_METABOLISM	-1.71	0.0072	0.0463	24	Depletion
14	KEGG_CITRATE_CYCLE_TCA_CYCLE	-1.69	0.0104	0.0520	30	Depletion
15	KEGG_CIRCADIAN_RHYTHM_MAMMAL	-1.64	0.0154	0.0753	12	Depletion
16	KEGG_CHRONIC_MYELOID_LEUKEMIA	-1.63	0.0053	0.0729	73	Depletion
17	KEGG_AMINO_SUGAR_AND_NUCLEOTIDE_SUGAR_METABOLISM	-1.63	0.0055	0.0716	43	Depletion
18	KEGG_INSULIN_SIGNALING_PATHWAY	-1.63	0.0000	0.0692	135	Depletion
19	KEGG_GLUTATHIONE_METABOLISM	-1.59	0.0103	0.0935	47	Depletion
20	KEGG_ALANINE_ASPARTATE_AND_GLUTAMATE_METABOLISM	-1.56	0.0351	0.1096	32	Depletion
21	KEGG_PROGESTERONE_MEDIATED_OOCYTE_MATURATION	-1.52	0.0134	0.1426	85	Depletion
22	KEGG_GLYCOLYSIS_GLUCONEOGENESIS	-1.51	0.0173	0.1545	60	Depletion
23	KEGG_SELENOAMINO_ACID_METABOLISM	-1.50	0.0407	0.1507	26	Depletion
24	KEGG_NEUROTROPHIN_SIGNALING_PATHWAY	-1.48	0.0103	0.1725	124	ns
25	KEGG_PANTOTHENATE_AND_COA_BIOSYNTHESIS	-1.48	0.0514	0.1682	16	ns
26	KEGG_ACUTE_MYELOID_LEUKEMIA	-1.48	0.0227	0.1641	57	ns
27	KEGG_ADIPOCYTOKINE_SIGNALING_PATHWAY	-1.46	0.0230	0.1862	67	ns
28	KEGG_FOLATE_BIOSYNTHESIS	-1.44	0.0718	0.2008	11	ns
29	KEGG_APOPTOSIS	-1.44	0.0149	0.1983	86	ns
30	KEGG_AMINOACYL_TRNA_BIOSYNTHESIS	-1.39	0.0653	0.2630	41	ns
31	KEGG_NON_HOMOLOGOUS_END_JOINING	-1.38	0.1103	0.2651	12	ns
32	KEGG_CELL_CYCLE	-1.38	0.0283	0.2664	123	ns
33	KEGG_CARDIAC_MUSCLE_CONTRACTION	-1.37	0.0429	0.2715	73	ns
34	KEGG_VALINE_LEUCINE_AND_ISOLEUCINE_BIOSYNTHESIS	-1.37	0.1209	0.2645	11	ns
35	KEGG_ENDOCYTOSIS	-1.36	0.0289	0.2675	173	ns
36	KEGG_TYPE_II_DIABETES_MELLITUS	-1.35	0.0771	0.2806	47	ns
37	KEGG_COLORECTAL_CANCER	-1.35	0.0678	0.2774	62	ns
38	KEGG_TIGHT_JUNCTION	-1.34	0.0331	0.2855	130	ns
39	KEGG_ENDOMETRIAL_CANCER	-1.34	0.0672	0.2806	52	ns
40	KEGG_AMYOTROPHIC_LATERAL_SCLEROSIS_ALS	-1.34	0.0914	0.2819	52	ns
41	KEGG_SPHINGOLIPID_METABOLISM	-1.32	0.0908	0.3046	39	ns
42	KEGG_WNT_SIGNALING_PATHWAY	-1.31	0.0397	0.3185	149	ns
43	KEGG_JAK_STAT_SIGNALING_PATHWAY	-1.30	0.0497	0.3229	154	ns
44	KEGG_NON_SMALL_CELL_LUNG_CANCER	-1.29	0.1028	0.3489	54	ns
45	KEGG_VIBRIO_CHOLERAE_INFECTION	-1.28	0.1194	0.3466	53	ns
46	KEGG_ARACHIDONIC_ACID_METABOLISM	-1.27	0.1171	0.3753	56	ns
47	KEGG_SMALL_CELL_LUNG_CANCER	-1.26	0.0989	0.3818	84	ns
48	KEGG_LEUKOCYTE_TRANSENDOTHELIAL_MIGRATION	-1.26	0.1015	0.3834	114	ns
49	KEGG_OOCYTE_MEIOSIS	-1.25	0.0929	0.3781	112	ns
50	KEGG_PROXIMAL_TUBULE_BICARBONATE_RECLAMATION	-1.25	0.1624	0.3807	22	ns
51	KEGG_VEGF_SIGNALING_PATHWAY	-1.24	0.1350	0.3930	74	ns
52	KEGG_GLIOMA	-1.23	0.1507	0.4167	65	ns
53	KEGG_CYTOSOLIC_DNA_SENSING_PATHWAY	-1.21	0.1664	0.4463	55	ns
54	KEGG_TERPENOID_BACKBONE_BIOSYNTHESIS	-1.20	0.2336	0.4565	15	ns
55	KEGG_TYPE_I_DIABETES_MELLITUS	-1.20	0.2199	0.4616	23	ns
56	KEGG_RIG_I_LIKE_RECEPTOR_SIGNALING_PATHWAY	-1.19	0.1807	0.4670	70	ns
57	KEGG_HEMATOPOIETIC_CELL_LINEAGE	-1.19	0.1794	0.4624	82	ns
58	KEGG_PRION_DISEASES	-1.18	0.2331	0.4808	34	ns
59	KEGG_GLYCOSPHINGOLIPID_BIOSYNTHESIS_GANGLIO_SERIES	-1.17	0.2613	0.5102	15	ns
60	KEGG_RENAL_CELL_CARCINOMA	-1.17	0.2174	0.5041	70	ns
61	KEGG_PROPANOATE_METABOLISM	-1.16	0.2542	0.4984	31	ns
62	KEGG_BASE_EXCISION_REPAIR	-1.16	0.2384	0.4971	33	ns
63	KEGG_FC_EPSILON_RI_SIGNALING_PATHWAY	-1.16	0.1868	0.4913	76	ns

#	Gene set enrichment for CERES dependency of T-ALL vs. all other nonT-ALL cell lines	NES	P-value	FDR	Size gene set	Status
64	KEGG_PANCREATIC_CANCER	-1.15	0.2164	0.5010	70	ns
65	KEGG_DNA_REPLICATION	-1.14	0.2381	0.5273	34	ns
66	KEGG_PENTOSE_PHOSPHATE_PATHWAY	-1.14	0.2676	0.5253	26	ns
67	KEGG_EPITHELIAL_CELL_SIGNALING_IN_HELICOBACTER_PYLORI_INF	-1.12	0.2542	0.5636	67	ns
68	KEGG_GNRH_SIGNALING_PATHWAY	-1.12	0.2541	0.5704	99	ns
69	KEGG_INTESTINAL_IMMUNE_NETWORK_FOR_IGA_PRODUCTION	-1.11	0.3114	0.5629	31	ns
70	KEGG_PORPHYRIN_AND_CHLOROPHYLL_METABOLISM	-1.11	0.2855	0.5566	41	ns
71	KEGG_PATHWAYS_IN_CANCER	-1.11	0.1982	0.5532	323	ns
72	KEGG_P53_SIGNALING_PATHWAY	-1.11	0.2694	0.5498	67	ns
73	KEGG_BASAL_CELL_CARCINOMA	-1.10	0.2974	0.5594	55	ns
74	KEGG_LYSOSOME	-1.09	0.2913	0.5804	121	ns
75	KEGG_THYROID_CANCER	-1.09	0.3363	0.5827	29	ns
76	KEGG_NATURAL_KILLER_CELL_MEDIATED_CYTOTOXICITY	-1.08	0.3019	0.5881	126	ns
77	KEGG_PHOSPHATIDYLINOSITOL_SIGNALING_SYSTEM	-1.08	0.3092	0.6031	74	ns
78	KEGG_HISTIDINE_METABOLISM	-1.08	0.3482	0.5963	28	ns
79	KEGG_OTHER_GLYCAN_DEGRADATION	-1.07	0.3783	0.5912	16	ns
80	KEGG_TOLL_LIKE_RECEPTOR_SIGNALING_PATHWAY	-1.07	0.3149	0.5976	102	ns
81	KEGG_FC_GAMMA_R_MEDIATED_PHAGOCYTOSIS	-1.06	0.3413	0.6050	94	ns
82	KEGG_NEUROACTIVE_LIGAND_RECEPTOR_INTERACTION	-1.06	0.3201	0.6137	269	ns
83	KEGG_ERBB_SIGNALING_PATHWAY	-1.04	0.3719	0.6535	87	ns
84	KEGG_DORSO_VENTRAL_AXIS_FORMATION	-1.03	0.3943	0.6661	24	ns
85	KEGG_PROSTATE_CANCER	-1.02	0.4235	0.6857	87	ns
86	KEGG_VALINE_LEUCINE_AND_ISOLEUCINE_DEGRADATION	-1.02	0.4556	0.6907	43	ns
87	KEGG_AXON_GUIDANCE	-1.02	0.4078	0.6849	127	ns
88	KEGG_VASCULAR_SMOOTH_MUSCLE_CONTRACTION	-1.01	0.4277	0.7037	113	ns
89	KEGG_GLYOXYLATE_AND_DICARBOXYLATE_METABOLISM	-1.01	0.4597	0.6977	16	ns
90	KEGG_UBIQUITIN_MEDIATED_PROTEOLYSIS	-1.00	0.4531	0.7052	133	ns
91	KEGG_CYSTEINE_AND_METHIONINE_METABOLISM	-1.00	0.4590	0.7129	34	ns
92	KEGG_DRUG_METABOLISM_OTHER_ENZYMES	-0.99	0.4983	0.7243	51	ns
93	KEGG_CHEMOKINE_SIGNALING_PATHWAY	-0.98	0.5215	0.7505	184	ns
94	KEGG_ALDOSTERONE_REGULATED_SODIUM_REABSORPTION	-0.98	0.5000	0.7449	42	ns
95	KEGG_ECM_RECEPTOR_INTERACTION	-0.97	0.5358	0.7436	84	ns
96	KEGG_LONG_TERM_POTENTIATION	-0.97	0.5120	0.7419	70	ns
97	KEGG_NOD_LIKE_RECEPTOR_SIGNALING_PATHWAY	-0.97	0.5050	0.7389	62	ns
98	KEGG_MELANOGENESIS	-0.95	0.5653	0.7798	101	ns
99	KEGG_INOSITOL_PHOSPHATE_METABOLISM	-0.94	0.5529	0.7923	53	ns
100	KEGG_FOCAL_ADHESION	-0.92	0.6578	0.8381	196	ns
101	KEGG_OLFACTORY_TRANSDUCTION	-0.91	0.7401	0.8733	354	ns
102	KEGG_NUCLEOTIDE_EXCISION_REPAIR	-0.90	0.6380	0.8921	44	ns
103	KEGG_PRIMARY_BILE_ACID_BIOSYNTHESIS	-0.89	0.6104	0.8863	16	ns
104	KEGG_MAPK_SIGNALING_PATHWAY	-0.89	0.7450	0.8779	262	ns
105	KEGG_REGULATION_OF_ACTIN_CYTOSKELETON	-0.89	0.7530	0.8891	207	ns
106	KEGG_FRUCTOSE_AND_MANNOSE_METABOLISM	-0.88	0.6383	0.8912	33	ns
107	KEGG_ARRHYTHMOGENIC_RIGHT_VENTRICULAR_CARDIOMYOPATHY_	-0.88	0.6972	0.8974	73	ns
108	KEGG_VIRAL_MYOCARDITIS	-0.87	0.6986	0.8976	50	ns
109	KEGG_GLYCOSAMINOGLYCAN_BIOSYNTHESIS_CHONDROITIN_SULFAT	-0.85	0.6885	0.9388	22	ns
110	KEGG_ASTHMA	-0.85	0.6892	0.9420	16	ns
111	KEGG_GLYCEROLIPID_METABOLISM	-0.84	0.7110	0.9422	48	ns
112	KEGG_CELL_ADHESION_MOLECULES_CAMS	-0.84	0.7909	0.9384	110	ns
113	KEGG_CALCIUM_SIGNALING_PATHWAY	-0.84	0.8644	0.9367	176	ns
114	KEGG_DILATED_CARDIOMYOPATHY	-0.84	0.7905	0.9306	90	ns
115	KEGG_HEDGEHOG_SIGNALING_PATHWAY	-0.84	0.7483	0.9235	56	ns
116	KEGG_GALACTOSE_METABOLISM	-0.84	0.7104	0.9169	26	ns
117	KEGG_GLYCOSAMINOGLYCAN_DEGRADATION	-0.84	0.6954	0.9092	21	ns
118	KEGG_STARCH_AND_SUCROSE_METABOLISM	-0.83	0.7553	0.9210	48	ns
119	KEGG_PPAR_SIGNALING_PATHWAY	-0.81	0.8141	0.9454	69	ns
120	KEGG_PATHOGENIC_ESCHERICHIA_COLI_INFECTION	-0.80	0.8307	0.9490	55	ns
121	KEGG_TGF_BETA_SIGNALING_PATHWAY	-0.80	0.8385	0.9447	84	ns
122	KEGG_GAP_JUNCTION	-0.79	0.8711	0.9620	89	ns
123	KEGG_HYPERTROPHIC_CARDIOMYOPATHY_HCM	-0.77	0.9158	0.9819	83	ns
124	KEGG_PYRUVATE_METABOLISM	-0.76	0.8442	0.9861	40	ns
125	KEGG_GLYCEROPHOSPHOLIPID_METABOLISM	-0.75	0.9005	0.9856	75	ns
126	KEGG_LEISHMANIA_INFECTION	-0.74	0.9054	0.9938	57	ns
127	KEGG_ETHER_LIPID_METABOLISM	-0.74	0.8668	0.9891	31	ns
128	KEGG_COMPLEMENT_AND_COAGULATION_CASCADES	-0.73	0.9174	0.9874	67	ns
129	KEGG_GLYCINE_SERINE_AND_THREONINE_METABOLISM	-0.73	0.8521	0.9801	31	ns
130	KEGG_TAURINE_AND_HYPOTAURINE_METABOLISM	-0.73	0.8296	0.9778	10	ns
131	KEGG_GLYCOSPHINGOLIPID_BIOSYNTHESIS_GLOBO_SERIES	-0.71	0.8481	0.9849	14	ns
132	KEGG_LINOLEIC_ACID_METABOLISM	-0.69	0.9052	0.9968	27	ns
133	KEGG_GLYCOSPHINGOLIPID_BIOSYNTHESIS_LACTO_AND_NEOLACTO	-0.66	0.9345	1.0000	25	ns
134	KEGG_RIBOFLAVIN_METABOLISM	-0.65	0.9168	1.0000	16	ns

	#	Gene set enrichment for CERES dependency of T-ALL vs. all other nonT-ALL cell lines	NES	P-value	FDR	Size gene set	Status
	135	KEGG_ALLOGRAFT_REJECTION	-0.64	0.9358	1.0000	17	ns
	136	KEGG_CYTOKINE_CYTOKINE_RECEPTOR_INTERACTION	-0.63	1.0000	1.0000	260	ns
	137	KEGG_PHENYLALANINE_METABOLISM	-0.58	0.9551	1.0000	17	ns
	138	KEGG_O_GLYCAN_BIOSYNTHESIS	-0.56	0.9915	1.0000	30	ns
	139	KEGG_GLYCOSAMINOGLYCAN_BIOSYNTHESIS_KERATAN_SULFATE	-0.54	0.9646	0.9998	15	ns
	140	KEGG_LIMONENE_AND_PINENE_DEGRADATION	-0.49	0.9943	0.9975	10	ns
	141	KEGG_AUTOIMMUNE_THYROID_DISEASE	0.52	0.9930	0.9957	32	ns
	142	KEGG_TASTE_TRANSDUCTION	0.56	0.9953	1.0000	51	ns
	143	KEGG_BETA_ALANINE_METABOLISM	0.60	0.9720	1.0000	22	ns
	144	KEGG_GRAFT_VERSUS_HOST_DISEASE	0.63	0.9080	1.0000	19	ns
	145	KEGG_MATURITY_ONSET_DIABETES_OF_THE_YOUNG	0.64	0.9473	1.0000	23	ns
	146	KEGG_TYROSINE_METABOLISM	0.68	0.9455	1.0000	40	ns
	147	KEGG_ALPHA_LINOLENIC_ACID_METABOLISM	0.72	0.8650	1.0000	17	ns
	148	KEGG_GLYCOSYLPHOSPHATIDYLINOSITOL_GPI_ANCHOR_BIOSYNTHE	0.77	0.8283	1.0000	25	ns
	149	KEGG_STEROID_HORMONE_BIOSYNTHESIS	0.79	0.8614	1.0000	54	ns
	150	KEGG_PROTEIN_EXPORT	0.81	0.7115	1.0000	23	ns
	151	KEGG_N_GLYCAN_BIOSYNTHESIS	0.82	0.7978	1.0000	46	ns
	152	KEGG_BASAL_TRANSCRIPTION_FACTORS	0.82	0.7387	1.0000	35	ns
	153	KEGG_BUTANOATE_METABOLISM	0.82	0.7581	1.0000	34	ns
	154	KEGG_RENIN_ANGIOTENSIN_SYSTEM	0.86	0.6591	1.0000	16	ns
	155	KEGG_REGULATION_OF_AUTOPHAGY	0.86	0.6697	1.0000	34	ns
	156	KEGG_VASOPRESSIN_REGULATED_WATER_REABSORPTION	0.87	0.7002	1.0000	43	ns
	157	KEGG_SNARE_INTERACTIONS_IN_VESICULAR_TRANSPORT	0.87	0.6895	1.0000	37	ns
	158	KEGG_PENTOSE_AND_GLUCURONATE_INTERCONVERSIONS	0.90	0.6253	1.0000	28	ns
	159	KEGG_LYSINE_DEGRADATION	0.92	0.6250	1.0000	44	ns
	160	KEGG_TRYPTOPHAN_METABOLISM	0.92	0.5763	1.0000	39	ns
	161	KEGG_LONG_TERM_DEPRESSION	0.94	0.5682	1.0000	68	ns
	162	KEGG_ADHERENS_JUNCTION	0.94	0.5801	1.0000	72	ns
	163	KEGG_SULFUR_METABOLISM	0.96	0.5000	1.0000	12	ns
	164	KEGG_ABC_TRANSPORTERS	0.98	0.4766	0.9965	44	ns
	165	KEGG_ARGININE_AND_PROLINE_METABOLISM	0.99	0.4813	0.9982	53	ns
	166	KEGG_FATTY_ACID_METABOLISM	1.02	0.4165	0.9145	41	ns
	167	KEGG_BLADDER_CANCER	1.03	0.4034	0.9043	42	ns
	168	KEGG_RNA_DEGRADATION	1.06	0.3399	0.8568	54	ns
	169	KEGG_ASCORBATE_AND_ALDARATE_METABOLISM	1.06	0.3479	0.8732	25	ns
	170	KEGG_DRUG_METABOLISM_CYTOCHROME_P450	1.07	0.3017	0.9077	68	ns
	171	KEGG_B_CELL_RECEPTOR_SIGNALING_PATHWAY	1.08	0.3077	0.9187	74	ns
	172	KEGG_MELANOMA	1.08	0.2738	0.9502	70	ns
	173	KEGG_PEROXISOME	1.10	0.2602	0.9324	78	ns
	174	KEGG_METABOLISM_OF_XENOBIOTICS_BY_CYTOCHROME_P450	1.11	0.2389	0.9652	66	ns
	175	KEGG_GLYCOSAMINOGLYCAN_BIOSYNTHESIS_HEPARAN_SULFATE	1.12	0.2908	0.9690	26	ns
	176	KEGG_STEROID_BIOSYNTHESIS	1.13	0.2712	1.0000	17	ns
	177	KEGG_RETINOL_METABOLISM	1.16	0.1953	0.9746	63	ns
	178	KEGG_BIOSYNTHESIS_OF_UNSATURATED_FATTY_ACIDS	1.17	0.2362	1.0000	22	ns
	179	KEGG_HOMOLOGOUS_RECOMBINATION	1.21	0.1956	0.9569	28	ns
	180	KEGG_ANTIGEN_PROCESSING_AND_PRESENTATION	1.24	0.1359	0.9202	62	ns
	181	KEGG_NITROGEN_METABOLISM	1.34	0.1050	0.5734	23	ns
	182	KEGG_MISMATCH_REPAIR	1.38	0.1062	0.5539	23	ns
	183	KEGG_HUNTINGTONS_DISEASE	1.52	0.0000	0.2548	169	ns
	184	KEGG_ALZHEIMERS_DISEASE	1.74	0.0000	0.0549	157	ns
	185	KEGG_OXIDATIVE_PHOSPHORYLATION	1.93	0.0000	0.0091	116	Proliferation
	186	KEGG_PARKINSONS_DISEASE	2.03	0.0000	0.0056	112	Proliferation

Supplementary table 2B: Genome-wide gene set enrichment results for Avana 19Q4 CERES dependency of T-ALL vs. nonT-ALL hematopoietic cell lines across the collection of KEGG pathways (MSigDB v7). Shown for each gene set are the normalized enrichment score (NES), the nominal P-value, the Benjamini-Hochberg False Discovery Rate (FDR), the size and the dependency status (depletion - negatively enriched, proliferation - positively enriched). Significance cutoffs: abs(NES) >1.5, nominal P-value < 0.10, FDR < 0.25.

#	Gene set enrichment for CERES dependency of T-ALL vs. other hematopoietic cell lines	NES	P-value	FDR	Size gene set	Status
1	KEGG_RIBOSOME	-2.34	0.0000	0.0000	85	Depletion
2	KEGG_SYSTEMIC_LUPUS_ERYTHEMATOSUS	-2.34	0.0000	0.0000	111	Depletion
3	KEGG_PYRIMIDINE_METABOLISM	-2.11	0.0000	0.0009	93	Depletion
4	KEGG_NOTCH_SIGNALING_PATHWAY	-2.10	0.0000	0.0010	47	Depletion
5	KEGG_T_CELL_RECEPTOR_SIGNALING_PATHWAY	-1.95	0.0000	0.0055	107	Depletion
6	KEGG_RNA_POLYMERASE	-1.94	0.0000	0.0054	26	Depletion
7	KEGG_ONE_CARBON_POOL_BY_FOLATE	-1.88	0.0019	0.0105	17	Depletion
8	KEGG_PRIMARY_IMMUNODEFICIENCY	-1.86	0.0000	0.0111	35	Depletion
9	KEGG_SPLICEOSOME	-1.84	0.0000	0.0125	124	Depletion
10	KEGG_NON_HOMOLOGOUS_END_JOINING	-1.73	0.0118	0.0462	12	Depletion
11	KEGG_PURINE_METABOLISM	-1.72	0.0016	0.0442	151	Depletion
12	KEGG_ENDOMETRIAL_CANCER	-1.71	0.0017	0.0461	52	Depletion
13	KEGG_INSULIN_SIGNALING_PATHWAY	-1.69	0.0000	0.0547	135	Depletion
14	KEGG_CIRCADIAN_RHYTHM_MAMMAL	-1.64	0.0236	0.0776	12	Depletion
15	KEGG_VALINE_LEUCINE_AND_ISOLEUCINE_BIOSYNTHESIS	-1.56	0.0348	0.1423	11	Depletion
16	KEGG_GLYCOLYSIS_GLUCONEOGENESIS	-1.56	0.0085	0.1344	60	Depletion
17	KEGG_NON_SMALL_CELL_LUNG_CANCER	-1.52	0.0137	0.1755	54	Depletion
18	KEGG_TYPE_I_DIABETES_MELLITUS	-1.50	0.0366	0.1841	23	Depletion
19	KEGG_NEUROTROPHIN_SIGNALING_PATHWAY	-1.48	0.0144	0.2087	124	ns
20	KEGG_CHRONIC_MYELOID_LEUKEMIA	-1.48	0.0179	0.2011	73	ns
21	KEGG_MTOR_SIGNALING_PATHWAY	-1.45	0.0342	0.2349	52	ns
22	KEGG_TIGHT_JUNCTION	-1.45	0.0164	0.2318	130	ns
23	KEGG_LEUKOCYTE_TRANSENDOTHELIAL_MIGRATION	-1.43	0.0179	0.2533	114	ns
24	KEGG_PROGESTERONE_MEDIATED_OOCYTE_MATURATION	-1.41	0.0429	0.2705	85	ns
25	KEGG_GLYCOSPHINGOLIPID_BIOSYNTHESIS_GANGLIO_SERIES	-1.40	0.0960	0.2812	15	ns
26	KEGG_RENAL_CELL_CARCINOMA	-1.39	0.0524	0.2986	70	ns
27	KEGG_DORSO_VENTRAL_AXIS_FORMATION	-1.35	0.0982	0.3651	24	ns
28	KEGG_VEGF_SIGNALING_PATHWAY	-1.34	0.0755	0.3838	74	ns
29	KEGG_SPHINGOLIPID_METABOLISM	-1.33	0.0930	0.3840	39	ns
30	KEGG_AMYOTROPHIC_LATERAL_SCLEROSIS_ALS	-1.32	0.0914	0.4045	52	ns
31	KEGG_PROPANOATE_METABOLISM	-1.30	0.1278	0.4408	31	ns
32	KEGG_COLORECTAL_CANCER	-1.29	0.0809	0.4510	62	ns
33	KEGG_NATURAL_KILLER_CELL_MEDIATED_CYTOTOXICITY	-1.28	0.0625	0.4577	126	ns
34	KEGG_PENTOSE_PHOSPHATE_PATHWAY	-1.28	0.1687	0.4532	26	ns
35	KEGG_ERBB_SIGNALING_PATHWAY	-1.27	0.1007	0.4593	87	ns
36	KEGG_AMINO_SUGAR_AND_NUCLEOTIDE_SUGAR_METABOLISM	-1.27	0.1376	0.4579	43	ns
37	KEGG_BASE_EXCISION_REPAIR	-1.26	0.1472	0.4564	33	ns
38	KEGG_ARRHYTHMOGENIC_RIGHT_VENTRICULAR_CARDIOMYOPATHY_ARVC	-1.25	0.1191	0.4675	73	ns
39	KEGG_THYROID_CANCER	-1.25	0.1715	0.4579	29	ns
40	KEGG_WNT_SIGNALING_PATHWAY	-1.24	0.0937	0.4885	149	ns
41	KEGG_FOLATE_BIOSYNTHESIS	-1.24	0.2025	0.4813	11	ns
42	KEGG_ENDOCYTOSIS	-1.23	0.0869	0.4856	173	ns
43	KEGG_HEMATOPOIETIC_CELL_LINEAGE	-1.22	0.1254	0.4874	82	ns
44	KEGG_GNRH_SIGNALING_PATHWAY	-1.22	0.1299	0.4819	99	ns
45	KEGG_ACUTE_MYELOID_LEUKEMIA	-1.22	0.1809	0.4856	57	ns
46	KEGG_PATHOGENIC_ESCHERICHIA_COLI_INFECTION	-1.21	0.1536	0.4829	55	ns
47	KEGG_TYPE_II_DIABETES_MELLITUS	-1.21	0.1827	0.4892	47	ns
48	KEGG_AMINOACYL_TRNA_BIOSYNTHESIS	-1.19	0.1940	0.5296	41	ns
49	KEGG_APOPTOSIS	-1.19	0.1734	0.5226	86	ns
50	KEGG_PROTEASOME	-1.18	0.2313	0.5363	44	ns
51	KEGG_CITRATE_CYCLE_TCA_CYCLE	-1.18	0.2297	0.5340	30	ns
52	KEGG_VIBRIO_CHOLERAE_INFECTION	-1.17	0.2190	0.5490	53	ns
53	KEGG_EPITHELIAL_CELL_SIGNALING_IN_HELICOBACTER_PYLORI_INFECTION	-1.16	0.2117	0.5596	67	ns
54	KEGG_CELL_ADHESION_MOLECULES_CAMS	-1.15	0.2149	0.5779	110	ns
55	KEGG_FOCAL_ADHESION	-1.15	0.1837	0.5747	196	ns
56	KEGG_PANTOTHENATE_AND_COA_BIOSYNTHESIS	-1.15	0.3112	0.5675	16	ns
57	KEGG_GLYCOSAMINOGLYCAN_BIOSYNTHESIS_CHONDROITIN_SULFATE	-1.14	0.2673	0.5646	22	ns
58	KEGG_BASAL_CELL_CARCINOMA	-1.14	0.2309	0.5578	55	ns
59	KEGG_CARDIAC_MUSCLE_CONTRACTION	-1.11	0.2751	0.6365	73	ns
60	KEGG_NEUROACTIVE_LIGAND_RECEPTOR_INTERACTION	-1.11	0.1991	0.6322	269	ns
61	KEGG_FC_EPSILON_RI_SIGNALING_PATHWAY	-1.11	0.2813	0.6290	76	ns
62	KEGG_DILATED_CARDIOMYOPATHY	-1.10	0.2768	0.6286	90	ns
63	KEGG_ARACHIDONIC_ACID_METABOLISM	-1.10	0.2913	0.6411	56	ns
64	KEGG_GLIOMA	-1.10	0.2830	0.6334	65	ns
65	KEGG_HISTIDINE_METABOLISM	-1.09	0.3299	0.6386	28	ns
66	KEGG_UBIQUITIN_MEDIATED_PROTEOLYSIS	-1.09	0.3026	0.6336	133	ns
67	KEGG_SMALL_CELL_LUNG_CANCER	-1.09	0.2893	0.6242	84	ns
68	KEGG_OOCYTE_MEIOSIS	-1.09	0.2750	0.6177	112	ns
69	KEGG_REGULATION_OF_ACTIN_CYTOSKELETON	-1.09	0.2540	0.6120	207	ns
70	KEGG_JAK_STAT_SIGNALING_PATHWAY	-1.08	0.2809	0.6134	154	ns
71	KEGG_PRION_DISEASES	-1.08	0.3351	0.6105	34	ns
72	KEGG_PATHWAYS_IN_CANCER	-1.08	0.2707	0.6060	323	ns
73	KEGG_FC_GAMMA_R_MEDIATED_PHAGOCYTOSIS	-1.08	0.3072	0.6093	94	ns
74	KEGG_OTHER_GLYCAN_DEGRADATION	-1.07	0.3766	0.6122	16	ns
75	KEGG_ADHERENS_JUNCTION	-1.07	0.3226	0.6080	72	ns
76	KEGG_INTESTINAL_IMMUNE_NETWORK_FOR_IGA_PRODUCTION	-1.06	0.3849	0.6177	31	ns

77	KEGG_GLYCOSYLPHOSPHATIDYLINOSITOL_GPI_ANCHOR_BIOSYNTHESIS	-1.06	0.3689	0.6313	25	ns
78	KEGG_GAP_JUNCTION	-1.04	0.3748	0.6684	89	ns
79	KEGG_STARCH_AND_SUCROSE_METABOLISM	-1.02	0.4271	0.7049	48	ns
80	KEGG_INOSITOL_PHOSPHATE_METABOLISM	-1.02	0.4278	0.7037	53	ns
81	KEGG_DNA_REPLICATION	-1.02	0.4403	0.7015	34	ns
82	KEGG_NICOTINATE_AND_NICOTINAMIDE_METABOLISM	-1.02	0.4317	0.6945	24	ns
83	KEGG_VIRAL_MYOCARDITIS	-1.02	0.4273	0.6896	50	ns
84	KEGG_ECM_RECEPTOR_INTERACTION	-1.01	0.4202	0.6941	84	ns
85	KEGG_RNA_DEGRADATION	-1.00	0.4541	0.7126	54	ns
86	KEGG_ALDOSTERONE_REGULATED_SODIUM_REABSORPTION	-1.00	0.4744	0.7113	42	ns
87	KEGG_SELENOAMINO_ACID_METABOLISM	-1.00	0.4729	0.7126	26	ns
88	KEGG_PHOSPHATIDYLINOSITOL_SIGNALING_SYSTEM	-0.98	0.4749	0.7353	74	ns
89	KEGG_HYPERTROPHIC_CARDIOMYOPATHY_HCM	-0.98	0.4737	0.7308	83	ns
90	KEGG_DRUG_METABOLISM_OTHER_ENZYMES	-0.97	0.5050	0.7454	51	ns
91	KEGG_CALCIUM_SIGNALING_PATHWAY	-0.97	0.4907	0.7377	176	ns
92	KEGG_PPAR_SIGNALING_PATHWAY	-0.97	0.5315	0.7355	69	ns
93	KEGG_CHEMOKINE_SIGNALING_PATHWAY	-0.96	0.5357	0.7488	184	ns
94	KEGG_VALINE_LEUCINE_AND_ISOLEUCINE_DEGRADATION	-0.96	0.5068	0.7433	43	ns
95	KEGG_ALANINE_ASPARTATE_AND_GLUTAMATE_METABOLISM	-0.96	0.5288	0.7515	32	ns
96	KEGG_LYSOSOME	-0.95	0.5556	0.7570	121	ns
97	KEGG_RIG_I_LIKE_RECEPTOR_SIGNALING_PATHWAY	-0.95	0.5497	0.7634	70	ns
98	KEGG_GLYCEROLIPID_METABOLISM	-0.94	0.5423	0.7644	48	ns
99	KEGG_GLUTATHIONE_METABOLISM	-0.94	0.5538	0.7644	47	ns
100	KEGG_CYTOSOLIC_DNA_SENSING_PATHWAY	-0.93	0.5960	0.7943	55	ns
101	KEGG_VASCULAR_SMOOTH_MUSCLE_CONTRACTION	-0.92	0.6353	0.7925	113	ns
102	KEGG_LONG_TERM_POTENTIATION	-0.90	0.6623	0.8411	70	ns
103	KEGG_GLYCOSAMINOGLYCAN_DEGRADATION	-0.89	0.6474	0.8619	21	ns
104	KEGG_MELANOGENESIS	-0.89	0.7107	0.8567	101	ns
105	KEGG_AXON_GUIDANCE	-0.88	0.7351	0.8661	127	ns
106	KEGG_GALACTOSE_METABOLISM	-0.88	0.6445	0.8657	26	ns
107	KEGG_TOLL_LIKE_RECEPTOR_SIGNALING_PATHWAY	-0.87	0.7324	0.8823	102	ns
108	KEGG_MAPK_SIGNALING_PATHWAY	-0.86	0.8655	0.8921	262	ns
109	KEGG_PRIMARY_BILE_ACID_BIOSYNTHESIS	-0.85	0.6705	0.9128	16	ns
110	KEGG_HEDGEHOG_SIGNALING_PATHWAY	-0.84	0.7374	0.9090	56	ns
111	KEGG_COMPLEMENT_AND_COAGULATION_CASCADES	-0.83	0.7785	0.9250	67	ns
112	KEGG_FRUCTOSE_AND_MANNOSE_METABOLISM	-0.83	0.7569	0.9300	33	ns
113	KEGG_GLYCOSAMINOGLYCAN_BIOSYNTHESIS_KERATAN_SULFATE	-0.82	0.7253	0.9349	15	ns
114	KEGG_CYSTEINE_AND_METHIONINE_METABOLISM	-0.80	0.8007	0.9557	34	ns
115	KEGG_NOD_LIKE_RECEPTOR_SIGNALING_PATHWAY	-0.80	0.8223	0.9567	62	ns
116	KEGG_PYRUVATE_METABOLISM	-0.79	0.8304	0.9605	40	ns
117	KEGG_GLYCOSAMINOGLYCAN_BIOSYNTHESIS_HEPARAN_SULFATE	-0.78	0.7969	0.9710	26	ns
118	KEGG_PROXIMAL_TUBULE_BICARBONATE_RECLAMATION	-0.76	0.8301	0.9950	22	ns
119	KEGG_TERPENOID_BACKBONE_BIOSYNTHESIS	-0.76	0.8007	0.9878	15	ns
120	KEGG_ASTHMA	-0.76	0.8103	0.9799	16	ns
121	KEGG_LINOLEIC_ACID_METABOLISM	-0.75	0.8175	0.9800	27	ns
122	KEGG_PROTEIN_EXPORT	-0.74	0.8316	0.9810	23	ns
123	KEGG_OLFACTORY_TRANSDUCTION	-0.72	1.0000	0.9973	354	ns
124	KEGG_GLYCOSPHINGOLIPID_BIOSYNTHESIS_GLOBO_SERIES	-0.71	0.8606	0.9986	14	ns
125	KEGG_TAURINE_AND_HYPOTAURINE_METABOLISM	-0.68	0.8495	1.0000	10	ns
126	KEGG_MATURITY_ONSET_DIABETES_OF_THE_YOUNG	-0.67	0.9180	1.0000	23	ns
127	KEGG_LIMONENE_AND_PINENE_DEGRADATION	-0.64	0.8916	1.0000	10	ns
128	KEGG_CYTOKINE_CYTOKINE_RECEPTOR_INTERACTION	-0.63	1.0000	1.0000	260	ns
129	KEGG_O_GLYCAN_BIOSYNTHESIS	-0.62	0.9654	1.0000	30	ns
130	KEGG_PHENYLALANINE_METABOLISM	-0.59	0.9468	1.0000	17	ns
131	KEGG_GLYCOSPHINGOLIPID_BIOSYNTHESIS_LACTO_AND_NEOLACTO_SERIES	-0.58	0.9583	1.0000	25	ns
132	KEGG_ALLOGRAFT_REJECTION	-0.57	0.9484	1.0000	17	ns
133	KEGG_ETHER_LIPID_METABOLISM	-0.48	0.9930	1.0000	31	ns
134	KEGG_AUTOIMMUNE_THYROID_DISEASE	-0.43	1.0000	0.9994	32	ns
135	KEGG_GRAFT_VERSUS_HOST_DISEASE	0.54	0.9882	0.9935	19	ns
136	KEGG_ALPHA_LINOLENIC_ACID_METABOLISM	0.55	0.9750	1.0000	17	ns
137	KEGG_REGULATION_OF_AUTOPHAGY	0.59	0.9900	1.0000	34	ns
138	KEGG_BETA_ALANINE_METABOLISM	0.62	0.9497	1.0000	22	ns
139	KEGG_TASTE_TRANSDUCTION	0.64	0.9747	1.0000	51	ns
140	KEGG_TYROSINE_METABOLISM	0.66	0.9500	1.0000	40	ns
141	KEGG_GLYOXYLATE_AND_DICARBOXYLATE_METABOLISM	0.67	0.8901	1.0000	16	ns
142	KEGG_GLYCINE_SERINE_AND_THREONINE_METABOLISM	0.67	0.9310	1.0000	31	ns
143	KEGG_SULFUR_METABOLISM	0.70	0.8531	1.0000	12	ns
144	KEGG_RENIN_ANGIOTENSIN_SYSTEM	0.73	0.8562	1.0000	16	ns
145	KEGG_BIOSYNTHESIS_OF_UNSATURATED_FATTY_ACIDS	0.77	0.7894	1.0000	22	ns
146	KEGG_BUTANOATE_METABOLISM	0.79	0.7799	1.0000	34	ns
147	KEGG_TRYPTOPHAN_METABOLISM	0.82	0.7917	1.0000	39	ns
148	KEGG_STEROID_HORMONE_BIOSYNTHESIS	0.82	0.7735	1.0000	54	ns
149	KEGG_LEISHMANIA_INFECTION	0.84	0.7767	1.0000	57	ns
150	KEGG_RIBOFLAVIN_METABOLISM	0.87	0.6625	1.0000	16	ns
151	KEGG_GLYCEROPHOSPHOLIPID_METABOLISM	0.89	0.7062	0.9775	75	ns
152	KEGG_NUCLEOTIDE_EXCISION_REPAIR	0.91	0.6115	0.9255	44	ns
153	KEGG_ABC_TRANSPORTERS	0.93	0.5925	0.9104	44	ns
154	KEGG_DRUG_METABOLISM_CYTOCHROME_P450	0.93	0.5873	0.9260	68	ns
155	KEGG_SNARE_INTERACTIONS_IN_VESICULAR_TRANSPORT	0.94	0.5760	0.9259	37	ns
156	KEGG_LONG_TERM_DEPRESSION	0.94	0.5895	0.9531	68	ns
157	KEGG_PENTOSE_AND_GLUCURONATE_INTERCONVERSIONS	0.95	0.5249	0.9466	28	ns
158	KEGG_BLADDER_CANCER	0.97	0.5286	0.9271	42	ns
159	KEGG_FATTY_ACID_METABOLISM	1.00	0.4679	0.8255	41	ns
160	KEGG_ASCORBATE_AND_ALDARATE_METABOLISM	1.01	0.4289	0.8481	25	ns
161	KEGG_N_GLYCAN_BIOSYNTHESIS	1.01	0.4069	0.8787	46	ns
162	KEGG_MELANOMA	1.02	0.4179	0.8702	70	ns
163	KEGG_P53_SIGNALING_PATHWAY	1.06	0.3302	0.7390	67	ns
164	KEGG_METABOLISM_OF_XENOBIOTICS_BY_CYTOCHROME_P450	1.08	0.2965	0.7037	66	ns

165	KEGG_HOMOLOGOUS_RECOMBINATION	1.13	0.2873	0.5962	28	ns
166	KEGG_ADIPOCYTOKINE_SIGNALING_PATHWAY	1.15	0.2214	0.5667	67	ns
167	KEGG_VASOPRESSIN_REGULATED_WATER_REABSORPTION	1.16	0.2350	0.5652	43	ns
168	KEGG_PROSTATE_CANCER	1.16	0.1830	0.5816	87	ns
169	KEGG_PEROXISOME	1.16	0.2075	0.6092	78	ns
170	KEGG_TGF_BETA_SIGNALING_PATHWAY	1.21	0.1342	0.4991	84	ns
171	KEGG_PORPHYRIN_AND_CHLOROPHYLL_METABOLISM	1.21	0.1627	0.5257	41	ns
172	KEGG_BASAL_TRANSCRIPTION_FACTORS	1.23	0.1736	0.4982	35	ns
173	KEGG_ARGININE_AND_PROLINE_METABOLISM	1.24	0.1214	0.5172	53	ns
174	KEGG_STEROID_BIOSYNTHESIS	1.24	0.1827	0.5539	17	ns
175	KEGG_RETINOL_METABOLISM	1.26	0.1148	0.5258	63	ns
176	KEGG_LYSINE_DEGRADATION	1.29	0.1065	0.4945	44	ns
177	KEGG_ANTIGEN_PROCESSING_AND_PRESENTATION	1.31	0.0878	0.4806	62	ns
178	KEGG_B_CELL_RECEPTOR_SIGNALING_PATHWAY	1.33	0.0469	0.4640	74	ns
179	KEGG_PANCREATIC_CANCER	1.35	0.0624	0.4647	70	ns
180	KEGG_CELL_CYCLE	1.37	0.0351	0.4796	123	ns
181	KEGG_NITROGEN_METABOLISM	1.42	0.0728	0.4092	23	ns
182	KEGG_ALZHEIMERS_DISEASE	1.67	0.0028	0.0758	157	Proliferation
183	KEGG_MISMATCH_REPAIR	1.67	0.0142	0.0919	23	Proliferation
184	KEGG_HUNTINGTONS_DISEASE	1.70	0.0000	0.0925	169	Proliferation
185	KEGG_OXIDATIVE_PHOSPHORYLATION	1.96	0.0000	0.0130	116	Proliferation
186	KEGG_PARKINSONS_DISEASE	2.07	0.0000	0.0056	112	Proliferation
