# Supplementary material for: Targeting serine hydroxymethyltransferases 1 and 2 for T-cell acute lymphoblastic leukemia therapy
Source: Leukemia. 2021 Aug 2;36(2):348–60. doi: 10.1038/s41375-021-01361-8 (PMC8807390; doi:10.1038/s41375-021-01361-8)
Supplement: Supplementary file 22 — Supplementary Table 3 [file 41375_2021_1361_MOESM22_ESM.pdf]

Supplemental Table 3A: List of ranked KEGG pathways as shown in the heatmap of GSE33315 data in Figure 1D. ONE CARBON POOL BY FOLATE, PURINE and PYRIMIDINE pathways are highlighted red.

Rank heatmap GSE33315	KEGG pathway
1	KEGG_GLYCOLYSIS_GLUCONEOGENESIS
2	KEGG_PENTOSE_PHOSPHATE_PATHWAY
3	KEGG_FRUCTOSE_AND_MANNOSE_METABOLISM
4	KEGG_GALACTOSE_METABOLISM
5	KEGG_AMINO_SUGAR_AND_NUCLEOTIDE_SUGAR_METABOLISM
6	KEGG_ALANINE_ASPARTATE_AND_GLUTAMATE_METABOLISM
7	KEGG_ARGININE_AND_PROLINE_METABOLISM
8	KEGG_GLUTATHIONE_METABOLISM
9	KEGG_FATTY_ACID_METABOLISM
10	KEGG_BUTANOATE_METABOLISM
11	KEGG_VALINE_LEUCINE_AND_ISOLEUCINE_DEGRADATION
12	KEGG_PROPANOATE_METABOLISM
13	KEGG_LYSINE_DEGRADATION
14	KEGG_BETA_ALANINE_METABOLISM
15	KEGG_LIMONENE_AND_PINENE_DEGRADATION
16	KEGG_NICOTINATE_AND_NICOTINAMIDE_METABOLISM
17	KEGG_PANTOTHENATE_AND_COA_BIOSYNTHESIS
18	KEGG_STEROID_BIOSYNTHESIS
19	KEGG_TERPENOID_BACKBONE_BIOSYNTHESIS
20	KEGG_BIOSYNTHESIS_OF_UNSATURATED_FATTY_ACIDS
21	KEGG_VALINE_LEUCINE_AND_ISOLEUCINE_BIOSYNTHESIS
22	KEGG_CITRATE_CYCLE_TCA_CYCLE
23	KEGG_PYRUVATE_METABOLISM
24	KEGG_CYSTEINE_AND_METHIONINE_METABOLISM
25	KEGG_GLYOXYLATE_AND_DICARBOXYLATE_METABOLISM
26	KEGG_ONE_CARBON_POOL_BY_FOLATE
27	KEGG_PURINE_METABOLISM
28	KEGG_PYRIMIDINE_METABOLISM
29	KEGG_RNA_POLYMERASE
30	KEGG_BASE_EXCISION_REPAIR
31	KEGG_HOMOLOGOUS_RECOMBINATION
32	KEGG_DNA_REPLICATION
33	KEGG_MISMATCH_REPAIR
34	KEGG_NUCLEOTIDE_EXCISION_REPAIR
35	KEGG_CELL_CYCLE
36	KEGG_NON_HOMOLOGOUS_END_JOINING
37	KEGG_GLYCOSYLPHOSPHATIDYLINOSITOL_GPI_ANCHOR_BIOSYNTHESIS
38	KEGG_SELENOAMINO_ACID_METABOLISM
39	KEGG_PEROXISOME
40	KEGG_BASAL_TRANSCRIPTION_FACTORS
41	KEGG_UBIQUITIN_MEDIATED_PROTEOLYSIS
42	KEGG_P53_SIGNALING_PATHWAY
43	KEGG_OOCYTE_MEIOSIS
44	KEGG_PROGESTERONE_MEDIATED_OOCYTE_MATURATION
45	KEGG_RIBOSOME
46	KEGG_SPLICEOSOME
47	KEGG_PROTEIN_EXPORT
48	KEGG_AMINOACYL_TRNA_BIOSYNTHESIS
49	KEGG_RNA_DEGRADATION
50	KEGG_PROTEASOME
51	KEGG_OXIDATIVE_PHOSPHORYLATION
52	KEGG_PARKINSONS_DISEASE
53	KEGG_HUNTINGTONS_DISEASE
54	KEGG_ALZHEIMERS_DISEASE
55	KEGG_CARDIAC_MUSCLE_CONTRACTION

56	KEGG_PATHOGENIC_ESCHERICHIA_COLI_INFECTION
57	KEGG_GLYCOSAMINOGLYCAN_BIOSYNTHESIS_CHONDROITIN_SULFATE
58	KEGG_GLYCOSAMINOGLYCAN_BIOSYNTHESIS_HEPARAN_SULFATE
59	KEGG_SULFUR_METABOLISM
60	KEGG_PENTOSE_AND_GLUCURONATE_INTERCONVERSIONS
61	KEGG_ASCORBATE_AND_ALDARATE_METABOLISM
62	KEGG_PORPHYRIN_AND_CHLOROPHYLL_METABOLISM
63	KEGG_GLYCOSAMINOGLYCAN_BIOSYNTHESIS_KERATAN_SULFATE
64	KEGG_O_GLYCAN_BIOSYNTHESIS
65	KEGG_GLYCOSPHINGOLIPID_BIOSYNTHESIS_GLOBO_SERIES
66	KEGG_GLYCOSPHINGOLIPID_BIOSYNTHESIS_GANGLIO_SERIES
67	KEGG_FOLATE_BIOSYNTHESIS
68	KEGG_RIBOFLAVIN_METABOLISM
69	KEGG_GLYCINE_SERINE_AND_THREONINE_METABOLISM
70	KEGG_TYROSINE_METABOLISM
71	KEGG_PHENYLALANINE_METABOLISM
72	KEGG_PRIMARY_BILE_ACID_BIOSYNTHESIS
73	KEGG_HISTIDINE_METABOLISM
74	KEGG_TRYPTOPHAN_METABOLISM
75	KEGG_GLYCEROLIPID_METABOLISM
76	KEGG_STARCH_AND_SUCROSE_METABOLISM
77	KEGG_DRUG_METABOLISM_OTHER_ENZYMES
78	KEGG_NITROGEN_METABOLISM
79	KEGG_PROXIMAL_TUBULE_BICARBONATE_RECLAMATION
80	KEGG_GLYCEROPHOSPHOLIPID_METABOLISM
81	KEGG_ARACHIDONIC_ACID_METABOLISM
82	KEGG_LINOLEIC_ACID_METABOLISM
83	KEGG_ETHER_LIPID_METABOLISM
84	KEGG_ALPHA_LINOLENIC_ACID_METABOLISM
85	KEGG_RENIN_ANGIOTENSIN_SYSTEM
86	KEGG_TAURINE_AND_HYPOTAURINE_METABOLISM
87	KEGG_GLYCOSPHINGOLIPID_BIOSYNTHESIS_LACTO_AND_NEOLACTO_SERIES
88	KEGG_ABC_TRANSPORTERS
89	KEGG_PPAR_SIGNALING_PATHWAY
90	KEGG_STEROID_HORMONE_BIOSYNTHESIS
91	KEGG_RETINOL_METABOLISM
92	KEGG_METABOLISM_OF_XENOBIOTICS_BY_CYTOCHROME_P450
93	KEGG_DRUG_METABOLISM_CYTOCHROME_P450
94	KEGG_COMPLEMENT_AND_COAGULATION_CASCADES
95	KEGG_CALCIUM_SIGNALING_PATHWAY
96	KEGG_NEUROACTIVE_LIGAND_RECEPTOR_INTERACTION
97	KEGG_OLFACTORY_TRANSDUCTION
98	KEGG_CYTOKINE_CYTOKINE_RECEPTOR_INTERACTION
99	KEGG_JAK_STAT_SIGNALING_PATHWAY
100	KEGG_FOCAL_ADHESION
101	KEGG_ECM_RECEPTOR_INTERACTION
102	KEGG_ARRHYTHMOGENIC_RIGHT_VENTRICULAR_CARDIOMYOPATHY_ARVC
103	KEGG_HYPERTROPHIC_CARDIOMYOPATHY_HCM
104	KEGG_DILATED_CARDIOMYOPATHY
105	KEGG_MATURITY_ONSET_DIABETES_OF_THE_YOUNG
106	KEGG_HEMATOPOIETIC_CELL_LINEAGE
107	KEGG_SPHINGOLIPID_METABOLISM
108	KEGG_OTHER_GLYCAN_DEGRADATION
109	KEGG_LYSOSOME
110	KEGG_N_GLYCAN_BIOSYNTHESIS
111	KEGG_SNARE_INTERACTIONS_IN_VESICULAR_TRANSPORT
112	KEGG_VASOPRESSIN_REGULATED_WATER_REABSORPTION
113	KEGG_VIBRIO_CHOLERAE_INFECTION
114	KEGG_GLYCOSAMINOGLYCAN_DEGRADATION
115	KEGG_REGULATION_OF_AUTOPHAGY
116	KEGG_ADIPOCYTOKINE_SIGNALING_PATHWAY
117	KEGG_ENDOCYTOSIS

118	KEGG_CHEMOKINE_SIGNALING_PATHWAY
119	KEGG_MAPK_SIGNALING_PATHWAY
120	KEGG_REGULATION_OF_ACTIN_CYTOSKELETON
121	KEGG_AXON_GUIDANCE
122	KEGG_VEGF_SIGNALING_PATHWAY
123	KEGG_FC_EPSILON_RI_SIGNALING_PATHWAY
124	KEGG_INSULIN_SIGNALING_PATHWAY
125	KEGG_TOLL_LIKE_RECEPTOR_SIGNALING_PATHWAY
126	KEGG_RIG_I_LIKE_RECEPTOR_SIGNALING_PATHWAY
127	KEGG_CYTOSOLIC_DNA_SENSING_PATHWAY
128	KEGG_CELL_ADHESION_MOLECULES_CAMS
129	KEGG_LEUKOCYTE_TRANSENDOTHELIAL_MIGRATION
130	KEGG_NATURAL_KILLER_CELL_MEDIATED_CYTOTOXICITY
131	KEGG_NOD_LIKE_RECEPTOR_SIGNALING_PATHWAY
132	KEGG_EPITHELIAL_CELL_SIGNALING_IN_HELICOBACTER_PYLORI_INFECTION
133	KEGG_AMYOTROPHIC_LATERAL_SCLEROSIS_ALS
134	KEGG_PRION_DISEASES
135	KEGG_LEISHMANIA_INFECTION
136	KEGG_INTESTINAL_IMMUNE_NETWORK_FOR_IGA_PRODUCTION
137	KEGG_AUTOIMMUNE_THYROID_DISEASE
138	KEGG_ASTHMA
139	KEGG_ANTIGEN_PROCESSING_AND_PRESENTATION
140	KEGG_TYPE_I_DIABETES_MELLITUS
141	KEGG_ALLOGRAFT_REJECTION
142	KEGG_GRAFT_VERSUS_HOST_DISEASE
143	KEGG_VIRAL_MYOCARDITIS
144	KEGG_SYSTEMIC_LUPUS_ERYTHEMATOSUS
145	KEGG_INOSITOL_PHOSPHATE_METABOLISM
146	KEGG_PHOSPHATIDYLINOSITOL_SIGNALING_SYSTEM
147	KEGG_NOTCH_SIGNALING_PATHWAY
148	KEGG_GAP_JUNCTION
149	KEGG_VASCULAR_SMOOTH_MUSCLE_CONTRACTION
150	KEGG_GNRH_SIGNALING_PATHWAY
151	KEGG_LONG_TERM_DEPRESSION
152	KEGG_LONG_TERM_POTENTIATION
153	KEGG_ADHERENS_JUNCTION
154	KEGG_TGF_BETA_SIGNALING_PATHWAY
155	KEGG_TYPE_II_DIABETES_MELLITUS
156	KEGG_TIGHT_JUNCTION
157	KEGG_TASTE_TRANSDUCTION
158	KEGG_HEDGEHOG_SIGNALING_PATHWAY
159	KEGG_MELANOGENESIS
160	KEGG_BASAL_CELL_CARCINOMA
161	KEGG_ALDOSTERONE_REGULATED_SODIUM_REABSORPTION
162	KEGG_WNT_SIGNALING_PATHWAY
163	KEGG_SMALL_CELL_LUNG_CANCER
164	KEGG_DORSO_VENTRAL_AXIS_FORMATION
165	KEGG_THYROID_CANCER
166	KEGG_MTOR_SIGNALING_PATHWAY
167	KEGG_ENDOMETRIAL_CANCER
168	KEGG_ERBB_SIGNALING_PATHWAY
169	KEGG_GLIOMA
170	KEGG_NON_SMALL_CELL_LUNG_CANCER
171	KEGG_PATHWAYS_IN_CANCER
172	KEGG_MELANOMA
173	KEGG_PROSTATE_CANCER
174	KEGG_BLADDER_CANCER
175	KEGG_APOPTOSIS
176	KEGG_B_CELL_RECEPTOR_SIGNALING_PATHWAY
177	KEGG_FC_GAMMA_R_MEDIATED_PHAGOCYTOSIS
178	KEGG_NEUROTROPHIN_SIGNALING_PATHWAY
179	KEGG_RENAL_CELL_CARCINOMA

180	KEGG_PANCREATIC_CANCER
181	KEGG_CHRONIC_MYELOID_LEUKEMIA
182	KEGG_ACUTE_MYELOID_LEUKEMIA
183	KEGG_COLORECTAL_CANCER
184	KEGG_T_CELL_RECEPTOR_SIGNALING_PATHWAY
185	KEGG_PRIMARY_IMMUNODEFICIENCY
186	KEGG_CIRCADIAN_RHYTHM_MAMMAL

Supplemental Table 3B: List of ranked KEGG pathways as shown in the heatmap of GSE13351 data in Supplementary Figure 2A. ONE CARBON POOL BY FOLATE, PURINE and PYRIMIDINE pathways are

Rank heatmap GSE13351	KEGG pathway
1	KEGG_RIBOSOME
2	KEGG_TAURINE_AND_HYPOTAURINE_METABOLISM
3	KEGG_ETHER_LIPID_METABOLISM
4	KEGG_NITROGEN_METABOLISM
5	KEGG_MATURITY_ONSET_DIABETES_OF_THE_YOUNG
6	KEGG_RETINOL_METABOLISM
7	KEGG_LINOLEIC_ACID_METABOLISM
8	KEGG_SPLICEOSOME
9	KEGG_PROTEIN_EXPORT
10	KEGG_STEROID_BIOSYNTHESIS
11	KEGG_PROXIMAL_TUBULE_BICARBONATE_RECLAMATION
12	KEGG_GLYCINE_SERINE_AND_THREONINE_METABOLISM
13	KEGG_SULFUR_METABOLISM
14	KEGG_PPAR_SIGNALING_PATHWAY
15	KEGG_ALPHA_LINOLENIC_ACID_METABOLISM
16	KEGG_NICOTINATE_AND_NICOTINAMIDE_METABOLISM
17	KEGG_PRIMARY_BILE_ACID_BIOSYNTHESIS
18	KEGG_RIBOFLAVIN_METABOLISM
19	KEGG_PANTOTHENATE_AND_COA_BIOSYNTHESIS
20	KEGG_SELENOAMINO_ACID_METABOLISM
21	KEGG_TERPENOID_BACKBONE_BIOSYNTHESIS
22	KEGG_ALANINE_ASPARTATE_AND_GLUTAMATE_METABOLISM
23	KEGG_STEROID_HORMONE_BIOSYNTHESIS
24	KEGG_GLYCOSYLPHOSPHATIDYLINOSITOL_GPI_ANCHOR_BIOSYNTHESIS
25	KEGG_TYROSINE_METABOLISM
26	KEGG_PHENYLALANINE_METABOLISM
27	KEGG_STARCH_AND_SUCROSE_METABOLISM
28	KEGG_PORPHYRIN_AND_CHLOROPHYLL_METABOLISM
29	KEGG_GLUTATHIONE_METABOLISM
30	KEGG_VALINE_LEUCINE_AND_ISOLEUCINE_BIOSYNTHESIS
31	KEGG_CYSTEINE_AND_METHIONINE_METABOLISM
32	KEGG_PEROXISOME
33	KEGG_PENTOSE_AND_GLUCURONATE_INTERCONVERSIONS
34	KEGG_TRYPTOPHAN_METABOLISM
35	KEGG_VALINE_LEUCINE_AND_ISOLEUCINE_DEGRADATION
36	KEGG_PYRUVATE_METABOLISM
37	KEGG_PROPANOATE_METABOLISM
38	KEGG_BETA_ALANINE_METABOLISM
39	KEGG_BUTANOATE_METABOLISM
40	KEGG_FATTY_ACID_METABOLISM
41	KEGG_PENTOSE_PHOSPHATE_PATHWAY
42	KEGG_GLYCOLYSIS_GLUCONEOGENESIS
43	KEGG_FRUCTOSE_AND_MANNOSE_METABOLISM
44	KEGG_AMINO_SUGAR_AND_NUCLEOTIDE_SUGAR_METABOLISM
45	KEGG_GALACTOSE_METABOLISM
46	KEGG_GLYOXYLATE_AND_DICARBOXYLATE_METABOLISM
47	KEGG_CITRATE_CYCLE_TCA_CYCLE
48	KEGG_LYSINE_DEGRADATION
49	KEGG_NON_HOMOLOGOUS_END_JOINING
50	KEGG_RNA_POLYMERASE
51	KEGG_RNA_DEGRADATION
52	KEGG_PYRIMIDINE_METABOLISM
53	KEGG_PURINE_METABOLISM
54	KEGG_NUCLEOTIDE_EXCISION_REPAIR
55	KEGG_MISMATCH_REPAIR
56	KEGG_DNA_REPLICATION
57	KEGG_BASE_EXCISION_REPAIR
58	KEGG_HOMOLOGOUS_RECOMBINATION
59	KEGG_CELL_CYCLE
60	KEGG_PROTEASOME
61	KEGG_PARKINSONS_DISEASE

62	KEGG_OXIDATIVE_PHOSPHORYLATION
63	KEGG_HUNTINGTONS_DISEASE
64	KEGG_ALZHEIMERS_DISEASE
65	KEGG_CARDIAC_MUSCLE_CONTRACTION
66	KEGG_HISTIDINE_METABOLISM
67	KEGG_GLYCEROLIPID_METABOLISM
68	KEGG_LIMONENE_AND_PINENE_DEGRADATION
69	KEGG_ASCORBATE_AND_ALDARATE_METABOLISM
70	KEGG_ONE_CARBON_POOL_BY_FOLATE
71	KEGG_AMINOACYL_TRNA_BIOSYNTHESIS
72	KEGG_DRUG_METABOLISM_OTHER_ENZYMES
73	KEGG_METABOLISM_OF_XENOBIOTICS_BY_CYTOCHROME_P450
74	KEGG_DRUG_METABOLISM_CYTOCHROME_P450
75	KEGG_ARACHIDONIC_ACID_METABOLISM
76	KEGG_BIOSYNTHESIS_OF_UNSATURATED_FATTY_ACIDS
77	KEGG_P53_SIGNALING_PATHWAY
78	KEGG_SNARE_INTERACTIONS_IN_VESICULAR_TRANSPORT
79	KEGG_GLYCEROPHOSPHOLIPID_METABOLISM
80	KEGG_N_GLYCAN_BIOSYNTHESIS
81	KEGG_VIBRIO_CHOLERAE_INFECTION
82	KEGG_AMYOTROPHIC_LATERAL_SCLEROSIS_ALS
83	KEGG_PROGESTERONE_MEDIATED_OOCYTE_MATURATION
84	KEGG_OOCYTE_MEIOSIS
85	KEGG_BASAL_TRANSCRIPTION_FACTORS
86	KEGG_FOLATE_BIOSYNTHESIS
87	KEGG_ARGININE_AND_PROLINE_METABOLISM
88	KEGG_SYSTEMIC_LUPUS_ERYTHEMATOSUS
89	KEGG_OLFACTORY_TRANSDUCTION
90	KEGG_NEUROACTIVE_LIGAND_RECEPTOR_INTERACTION
91	KEGG_T_CELL_RECEPTOR_SIGNALING_PATHWAY
92	KEGG_PHOSPHATIDYLINOSITOL_SIGNALING_SYSTEM
93	KEGG_INOSITOL_PHOSPHATE_METABOLISM
94	KEGG_PRIMARY_IMMUNODEFICIENCY
95	KEGG_ABC_TRANSPORTERS
96	KEGG_RENIN_ANGIOTENSIN_SYSTEM
97	KEGG_HEMATOPOIETIC_CELL_LINEAGE
98	KEGG_COMPLEMENT_AND_COAGULATION_CASCADES
99	KEGG_O_GLYCAN_BIOSYNTHESIS
100	KEGG_OTHER_GLYCAN_DEGRADATION
101	KEGG_LYSOSOME
102	KEGG_GLYCOSAMINOGLYCAN_DEGRADATION
103	KEGG_GLYCOSPHINGOLIPID_BIOSYNTHESIS_GLOBO_SERIES
104	KEGG_GLYCOSPHINGOLIPID_BIOSYNTHESIS_GANGLIO_SERIES
105	KEGG_TASTE_TRANSDUCTION
106	KEGG_HEDGEHOG_SIGNALING_PATHWAY
107	KEGG_BASAL_CELL_CARCINOMA
108	KEGG_REGULATION_OF_AUTOPHAGY
109	KEGG_THYROID_CANCER
110	KEGG_VASOPRESSIN_REGULATED_WATER_REABSORPTION
111	KEGG_UBIQUITIN_MEDIATED_PROTEOLYSIS
112	KEGG_TGF_BETA_SIGNALING_PATHWAY
113	KEGG_TYPE_II_DIABETES_MELLITUS
114	KEGG_AXON_GUIDANCE
115	KEGG_ALDOSTERONE_REGULATED_SODIUM_REABSORPTION
116	KEGG_NOTCH_SIGNALING_PATHWAY
117	KEGG_MELANOGENESIS
118	KEGG_LONG_TERM_POTENTIATION
119	KEGG_MAPK_SIGNALING_PATHWAY
120	KEGG_GNRH_SIGNALING_PATHWAY
121	KEGG_LONG_TERM_DEPRESSION
122	KEGG_GAP_JUNCTION
123	KEGG_VASCULAR_SMOOTH_MUSCLE_CONTRACTION
124	KEGG_MTOR_SIGNALING_PATHWAY
125	KEGG_LEUKOCYTE_TRANSENDOTHELIAL_MIGRATION
126	KEGG_VEGF_SIGNALING_PATHWAY
127	KEGG_FC_EPSILON_RI_SIGNALING_PATHWAY
128	KEGG_REGULATION_OF_ACTIN_CYTOSKELETON
129	KEGG_FOCAL_ADHESION

130	KEGG_FC_GAMMA_R_MEDIATED_PHAGOCYTOSIS
131	KEGG_CHEMOKINE_SIGNALING_PATHWAY
132	KEGG_B_CELL_RECEPTOR_SIGNALING_PATHWAY
133	KEGG_ENDOCYTOSIS
134	KEGG_WNT_SIGNALING_PATHWAY
135	KEGG_ADHERENS_JUNCTION
136	KEGG_SMALL_CELL_LUNG_CANCER
137	KEGG_PATHWAYS_IN_CANCER
138	KEGG_PANCREATIC_CANCER
139	KEGG_NON_SMALL_CELL_LUNG_CANCER
140	KEGG_GLIOMA
141	KEGG_ENDOMETRIAL_CANCER
142	KEGG_COLORECTAL_CANCER
143	KEGG_PROSTATE_CANCER
144	KEGG_RENAL_CELL_CARCINOMA
145	KEGG_NEUROTROPHIN_SIGNALING_PATHWAY
146	KEGG_CHRONIC_MYELOID_LEUKEMIA
147	KEGG_ACUTE_MYELOID_LEUKEMIA
148	KEGG_INSULIN_SIGNALING_PATHWAY
149	KEGG_ERBB_SIGNALING_PATHWAY
150	KEGG_MELANOMA
151	KEGG_BLADDER_CANCER
152	KEGG_EPITHELIAL_CELL_SIGNALING_IN_HELICOBACTER_PYLORI_INFECTION
153	KEGG_PRION_DISEASES
154	KEGG_SPHINGOLIPID_METABOLISM
155	KEGG_RIG_I_LIKE_RECEPTOR_SIGNALING_PATHWAY
156	KEGG_NOD_LIKE_RECEPTOR_SIGNALING_PATHWAY
157	KEGG_NATURAL_KILLER_CELL_MEDIATED_CYTOTOXICITY
158	KEGG_TOLL_LIKE_RECEPTOR_SIGNALING_PATHWAY
159	KEGG_APOPTOSIS
160	KEGG_JAK_STAT_SIGNALING_PATHWAY
161	KEGG_CYTOKINE_CYTOKINE_RECEPTOR_INTERACTION
162	KEGG_ADIPOCYTOKINE_SIGNALING_PATHWAY
163	KEGG_CYTOSOLIC_DNA_SENSING_PATHWAY
164	KEGG_INTESTINAL_IMMUNE_NETWORK_FOR_IGA_PRODUCTION
165	KEGG_LEISHMANIA_INFECTION
166	KEGG_VIRAL_MYOCARDITIS
167	KEGG_GRAFT_VERSUS_HOST_DISEASE
168	KEGG_TYPE_I_DIABETES_MELLITUS
169	KEGG_ALLOGRAFT_REJECTION
170	KEGG_AUTOIMMUNE_THYROID_DISEASE
171	KEGG_ASTHMA
172	KEGG_ANTIGEN_PROCESSING_AND_PRESENTATION
173	KEGG_CELL_ADHESION_MOLECULES_CAMS
174	KEGG_TIGHT_JUNCTION
175	KEGG_PATHOGENIC_ESCHERICHIA_COLI_INFECTION
176	KEGG_ECM_RECEPTOR_INTERACTION
177	KEGG_HYPERTROPHIC_CARDIOMYOPATHY_HCM
178	KEGG_DILATED_CARDIOMYOPATHY
179	KEGG_ARRHYTHMOGENIC_RIGHT_VENTRICULAR_CARDIOMYOPATHY_ARVC
180	KEGG_CALCIUM_SIGNALING_PATHWAY
181	KEGG_GLYCOSPHINGOLIPID_BIOSYNTHESIS_LACTO_AND_NEOLACTO_SERIES
182	KEGG_GLYCOSAMINOGLYCAN_BIOSYNTHESIS_KERATAN_SULFATE
183	KEGG_DORSO_VENTRAL_AXIS_FORMATION
184	KEGG_CIRCADIAN_RHYTHM_MAMMAL
185	KEGG_GLYCOSAMINOGLYCAN_BIOSYNTHESIS_HEPARAN_SULFATE
186	KEGG_GLYCOSAMINOGLYCAN_BIOSYNTHESIS_CHONDROITIN_SULFATE

Supplemental Table 3C: List of ranked KEGG pathways as shown in the heatmap of TARGET T-ALL data in Figure 2A. ONE CARBON POOL BY FOLATE, PURINE and PYRIMIDINE pathways are highlighted red.

Rank heatmap TARGET T-ALL	KEGG pathway
1	KEGG_PENTOSE_PHOSPHATE_PATHWAY
2	KEGG_FRUCTOSE_AND_MANNOSE_METABOLISM
3	KEGG_GALACTOSE_METABOLISM
4	KEGG_AMINO_SUGAR_AND_NUCLEOTIDE_SUGAR_METABOLISM
5	KEGG_PORPHYRIN_AND_CHLOROPHYLL_METABOLISM
6	KEGG_DRUG_METABOLISM_OTHER_ENZYMES
7	KEGG_GLYCOSYLPHOSPHATIDYLINOSITOL_GPI_ANCHOR_BIOSYNTHESIS
8	KEGG_LYSINE_DEGRADATION
9	KEGG_CYSTEINE_AND_METHIONINE_METABOLISM
10	KEGG_SELENOAMINO_ACID_METABOLISM
11	KEGG_ALANINE_ASPARTATE_AND_GLUTAMATE_METABOLISM
12	KEGG_BETA_ALANINE_METABOLISM
13	KEGG_HISTIDINE_METABOLISM
14	KEGG_ARGININE_AND_PROLINE_METABOLISM
15	KEGG_GLUTATHIONE_METABOLISM
16	KEGG_GLYCOLYSIS_GLUCONEOGENESIS
17	KEGG_PYRUVATE_METABOLISM
18	KEGG_TRYPTOPHAN_METABOLISM
19	KEGG_ASCORBATE_AND_ALDARATE_METABOLISM
20	KEGG_LIMONENE_AND_PINENE_DEGRADATION
21	KEGG_VALINE_LEUCINE_AND_ISOLEUCINE_DEGRADATION
22	KEGG_PROPANOATE_METABOLISM
23	KEGG_BUTANOATE_METABOLISM
24	KEGG_FATTY_ACID_METABOLISM
25	KEGG_PEROXISOME
26	KEGG_VALINE_LEUCINE_AND_ISOLEUCINE_BIOSYNTHESIS
27	KEGG_AMINOACYL_TRNA_BIOSYNTHESIS
28	KEGG_PENTOSE_AND_GLUCURONATE_INTERCONVERSIONS
29	KEGG_ONE_CARBON_POOL_BY_FOLATE
30	KEGG_PYRIMIDINE_METABOLISM
31	KEGG_PURINE_METABOLISM
32	KEGG_RNA_POLYMERASE
33	KEGG_GLYOXYLATE_AND_DICARBOXYLATE_METABOLISM
34	KEGG_BASE_EXCISION_REPAIR
35	KEGG_DNA_REPLICATION
36	KEGG_MISMATCH_REPAIR
37	KEGG_NUCLEOTIDE_EXCISION_REPAIR
38	KEGG_HOMOLOGOUS_RECOMBINATION
39	KEGG_TYROSINE_METABOLISM
40	KEGG_PHENYLALANINE_METABOLISM
41	KEGG_RIBOSOME
42	KEGG_N_GLYCAN_BIOSYNTHESIS
43	KEGG_CITRATE_CYCLE_TCA_CYCLE
44	KEGG_PROTEASOME
45	KEGG_OXIDATIVE_PHOSPHORYLATION
46	KEGG_PARKINSONS_DISEASE
47	KEGG_HUNTINGTONS_DISEASE
48	KEGG_ALZHEIMERS_DISEASE
49	KEGG_CARDIAC_MUSCLE_CONTRACTION
50	KEGG_STEROID_HORMONE_BIOSYNTHESIS
51	KEGG_METABOLISM_OF_XENOBIOTICS_BY_CYTOCHROME_P450
52	KEGG_DRUG_METABOLISM_CYTOCHROME_P450
53	KEGG_FOLATE_BIOSYNTHESIS
54	KEGG_STEROID_BIOSYNTHESIS
55	KEGG_TERPENOID_BACKBONE_BIOSYNTHESIS
56	KEGG_BIOSYNTHESIS_OF_UNSATURATED_FATTY_ACIDS
57	KEGG_PROXIMAL_TUBULE_BICARBONATE_RECLAMATION

58	KEGG_NON_HOMOLOGOUS_END_JOINING
59	KEGG_PRIMARY_IMMUNODEFICIENCY
60	KEGG_STARCH_AND_SUCROSE_METABOLISM
61	KEGG_GLYCEROLIPID_METABOLISM
62	KEGG_GLYCEROPHOSPHOLIPID_METABOLISM
63	KEGG_PPAR_SIGNALING_PATHWAY
64	KEGG_NICOTINATE_AND_NICOTINAMIDE_METABOLISM
65	KEGG_SULFUR_METABOLISM
66	KEGG_GLYCOSAMINOGLYCAN_BIOSYNTHESIS_CHONDROITIN_SULFATE
67	KEGG_GLYCOSAMINOGLYCAN_BIOSYNTHESIS_HEPARAN_SULFATE
68	KEGG_GLYCOSPHINGOLIPID_BIOSYNTHESIS_GLOBO_SERIES
69	KEGG_AMYOTROPHIC_LATERAL_SCLEROSIS_ALS
70	KEGG_PRION_DISEASES
71	KEGG_VIBRIO_CHOLERAE_INFECTION
72	KEGG_GLYCINE_SERINE_AND_THREONINE_METABOLISM
73	KEGG_RIBOFLAVIN_METABOLISM
74	KEGG_PROTEIN_EXPORT
75	KEGG_BASAL_TRANSCRIPTION_FACTORS
76	KEGG_RNA_DEGRADATION
77	KEGG_SPLICEOSOME
78	KEGG_P53_SIGNALING_PATHWAY
79	KEGG_CELL_CYCLE
80	KEGG_OOCYTE_MEIOSIS
81	KEGG_PROGESTERONE_MEDIATED_OOCYTE_MATURATION
82	KEGG_PATHOGENIC_ESCHERICHIA_COLI_INFECTION
83	KEGG_NITROGEN_METABOLISM
84	KEGG_ARACHIDONIC_ACID_METABOLISM
85	KEGG_LINOLEIC_ACID_METABOLISM
86	KEGG_ALPHA_LINOLENIC_ACID_METABOLISM
87	KEGG_RETINOL_METABOLISM
88	KEGG_PRIMARY_BILE_ACID_BIOSYNTHESIS
89	KEGG_OLFACTORY_TRANSDUCTION
90	KEGG_TASTE_TRANSDUCTION
91	KEGG_PANTOTHENATE_AND_COA_BIOSYNTHESIS
92	KEGG_SNARE_INTERACTIONS_IN_VESICULAR_TRANSPORT
93	KEGG_REGULATION_OF_AUTOPHAGY
94	KEGG_O_GLYCAN_BIOSYNTHESIS
95	KEGG_GLYCOSAMINOGLYCAN_BIOSYNTHESIS_KERATAN_SULFATE
96	KEGG_GLYCOSPHINGOLIPID_BIOSYNTHESIS_LACTO_AND_NEOLACTO_SERIES
97	KEGG_GLYCOSPHINGOLIPID_BIOSYNTHESIS_GANGLIO_SERIES
98	KEGG_SPHINGOLIPID_METABOLISM
99	KEGG_GLYCOSAMINOGLYCAN_DEGRADATION
100	KEGG_OTHER_GLYCAN_DEGRADATION
101	KEGG_LYSOSOME
102	KEGG_NEUROACTIVE_LIGAND_RECEPTOR_INTERACTION
103	KEGG_ECM_RECEPTOR_INTERACTION
104	KEGG_COMPLEMENT_AND_COAGULATION_CASCADES
105	KEGG_RENIN_ANGIOTENSIN_SYSTEM
106	KEGG_CYTOSOLIC_DNA_SENSING_PATHWAY
107	KEGG_HEMATOPOIETIC_CELL_LINEAGE
108	KEGG_ANTIGEN_PROCESSING_AND_PRESENTATION
109	KEGG_CELL_ADHESION_MOLECULES_CAMS
110	KEGG_LEISHMANIA_INFECTION
111	KEGG_INTESTINAL_IMMUNE_NETWORK_FOR_IGA_PRODUCTION
112	KEGG_ASTHMA
113	KEGG_TYPE_I_DIABETES_MELLITUS
114	KEGG_ALLOGRAFT_REJECTION
115	KEGG_GRAFT_VERSUS_HOST_DISEASE
116	KEGG_AUTOIMMUNE_THYROID_DISEASE
117	KEGG_VIRAL_MYOCARDITIS
118	KEGG_SYSTEMIC_LUPUS_ERYTHEMATOSUS
119	KEGG_TAURINE_AND_HYPOTAURINE_METABOLISM
120	KEGG_ETHER_LIPID_METABOLISM
121	KEGG_DORSO_VENTRAL_AXIS_FORMATION

122	KEGG_UBIQUITIN_MEDIATED_PROTEOLYSIS
123	KEGG_TGF_BETA_SIGNALING_PATHWAY
124	KEGG_WNT_SIGNALING_PATHWAY
125	KEGG_ADHERENS_JUNCTION
126	KEGG_TIGHT_JUNCTION
127	KEGG_NOTCH_SIGNALING_PATHWAY
128	KEGG_CIRCADIAN_RHYTHM_MAMMAL
129	KEGG_ABC_TRANSPORTERS
130	KEGG_INOSITOL_PHOSPHATE_METABOLISM
131	KEGG_PHOSPHATIDYLINOSITOL_SIGNALING_SYSTEM
132	KEGG_CALCIUM_SIGNALING_PATHWAY
133	KEGG_VASCULAR_SMOOTH_MUSCLE_CONTRACTION
134	KEGG_ARRHYTHMOGENIC_RIGHT_VENTRICULAR_CARDIOMYOPATHY_ARVC
135	KEGG_HYPERTROPHIC_CARDIOMYOPATHY_HCM
136	KEGG_DILATED_CARDIOMYOPATHY
137	KEGG_AXON_GUIDANCE
138	KEGG_GAP_JUNCTION
139	KEGG_MELANOGENESIS
140	KEGG_ENDOCYTOSIS
141	KEGG_FC_GAMMA_R_MEDIATED_PHAGOCYTOSIS
142	KEGG_CYTOKINE_CYTOKINE_RECEPTOR_INTERACTION
143	KEGG_CHEMOKINE_SIGNALING_PATHWAY
144	KEGG_NOD_LIKE_RECEPTOR_SIGNALING_PATHWAY
145	KEGG_TOLL_LIKE_RECEPTOR_SIGNALING_PATHWAY
146	KEGG_JAK_STAT_SIGNALING_PATHWAY
147	KEGG_APOPTOSIS
148	KEGG_B_CELL_RECEPTOR_SIGNALING_PATHWAY
149	KEGG_LEUKOCYTE_TRANSENDOTHELIAL_MIGRATION
150	KEGG_NATURAL_KILLER_CELL_MEDIATED_CYTOTOXICITY
151	KEGG_EPITHELIAL_CELL_SIGNALING_IN_HELICOBACTER_PYLORI_INFECTION
152	KEGG_ADIPOCYTOKINE_SIGNALING_PATHWAY
153	KEGG_RIG_I_LIKE_RECEPTOR_SIGNALING_PATHWAY
154	KEGG_T_CELL_RECEPTOR_SIGNALING_PATHWAY
155	KEGG_LONG_TERM_POTENTIATION
156	KEGG_LONG_TERM_DEPRESSION
157	KEGG_MAPK_SIGNALING_PATHWAY
158	KEGG_FC_EPSILON_RI_SIGNALING_PATHWAY
159	KEGG_GNRH_SIGNALING_PATHWAY
160	KEGG_VEGF_SIGNALING_PATHWAY
161	KEGG_ACUTE_MYELOID_LEUKEMIA
162	KEGG_MTOR_SIGNALING_PATHWAY
163	KEGG_ERBB_SIGNALING_PATHWAY
164	KEGG_NEUROTROPHIN_SIGNALING_PATHWAY
165	KEGG_RENAL_CELL_CARCINOMA
166	KEGG_INSULIN_SIGNALING_PATHWAY
167	KEGG_ENDOMETRIAL_CANCER
168	KEGG_CHRONIC_MYELOID_LEUKEMIA
169	KEGG_COLORECTAL_CANCER
170	KEGG_PROSTATE_CANCER
171	KEGG_REGULATION_OF_ACTIN_CYTOSKELETON
172	KEGG_FOCAL_ADHESION
173	KEGG_PATHWAYS_IN_CANCER
174	KEGG_PANCREATIC_CANCER
175	KEGG_MELANOMA
176	KEGG_GLIOMA
177	KEGG_NON_SMALL_CELL_LUNG_CANCER
178	KEGG_SMALL_CELL_LUNG_CANCER
179	KEGG_TYPE_II_DIABETES_MELLITUS
180	KEGG_ALDOSTERONE_REGULATED_SODIUM_REABSORPTION
181	KEGG_THYROID_CANCER
182	KEGG_BLADDER_CANCER
183	KEGG_VASOPRESSIN_REGULATED_WATER_REABSORPTION
184	KEGG_HEDGEHOG_SIGNALING_PATHWAY
185	KEGG_BASAL_CELL_CARCINOMA

186 KEGG_MATURITY_ONSET_DIABETES_OF_THE_YOUNG
