# Supplementary material for: Targeting serine hydroxymethyltransferases 1 and 2 for T-cell acute lymphoblastic leukemia therapy
Source: Leukemia. 2021 Aug 2;36(2):348–60. doi: 10.1038/s41375-021-01361-8 (PMC8807390; doi:10.1038/s41375-021-01361-8)
Supplement: Supplementary file 23 — Supplementary Table 4 [file 41375_2021_1361_MOESM23_ESM.pdf]

KEGG_ONE_CARBON_POOL_BY_FOLATE	KEGG_PURINE_METABOLISM	KEGG_PYRIMIDINE_METABOLISM
http://www.gsea-msigdb.org/gsea/msigdb/cards/KEGG_ONE_CARBON_POOL_BY_FOLATE	http://www.gsea-msigdb.org/gsea/msigdb/cards/KEGG_PURINE_METABOLISM	http://www.gsea-msigdb.org/gsea/msigdb/cards/KEGG_PYRIMIDINE_METABOLISM
MTHFD2	POLR2G	NT5C2
GART	NT5C2	POLR2G
TYMS	POLR2H	POLR2H
FTCD	ENPP3	POLR2E
ALDH1L1	POLR2E	POLR2F
MTHFS	POLR2F	POLR2I
MTFMT	ENPP1	POLR2J
AMT	XDH	POLE3
MTHFD2L	POLR2I	TYMS
DHFR	POLR2J	TXNRD1
SHMT1	POLE3	NME6
MTHFD1L	ADSS2	POLR1D
MTHFD1	PRPS2	PNPT1
MTR	ADSL	POLR2K
ATIC	NME6	DHODH
MTHFR	POLR1D	POLD3
SHMT2	PNPT1	POLR2L
	POLR2K	NME1-NME2
	PDE11A	POLA2
	POLD3	UPRT
	POLR2L	CANT1
	AK1	NME4
	NME1-NME2	NME1
	AK2	NME2
	AK4	NME3
	POLA2	POLR3C
	CANT1	POLR3G
	AMPD1	POLR3F
	AMPD3	NUDT2
	AMPD2	PRIM1
	NME4	PRIM2
	GUCY2D	NME5
	NME1	POLD4
	ATIC	DTYMK
	NME2	CTPS1
	DGUOK	POLA1
	POLR3C	ENTPD8
	NME3	POLE2
	POLR3G	POLD1
	POLR3F	POLD2
	PRUNE1	POLE
	ADPRM	CAD
	PDE4B	CMPK2
	PDE4C	POLR1E

PDE3B
PDE4A
NUDT2
GMPR2
PDE6C
PDE6D
PDE4D
PDE6A
PRIM1
PRIM2
AK5
GMPR
NME5
PDE7B
PDE5A
POLD4
GUCY1A2
FHIT
ADCY3
ADCY2
ADCY1
POLA1
PDE3A
PDE2A
PDE1C
ADSS1
ADA
ENTPD8
POLE2
PDE8A
PDE9A
PDE1B
NUDT9
POLD1
GDA
POLD2
POLE
PDE6B
PDE7A
PRPS1
POLR1E
PAICS
NT5C1A
POLR2B
POLR3A
PDE6H
POLR2A
PDE6G
POLR2D
DCK
POLR2C
NT5C1A
POLR3A
DCTD
POLR2B
POLR2A
DCK
POLR2D
POLR2C
UCK1
UPB1
TXNRD2
CMPK1
POLR3K
RRM2B
PNP
POLR1B
UPP2
CTPS2
POLR2J2
CDA
NT5M
DUT
POLR3H
UCKL1
POLR2J3
POLR1A
POLR3B
ENTPD4
ITPA
POLR1C
POLE4
RRM1
RRM2
POLR3D
NT5C3A
DPYS
DPYD
TYMP
UMPS
UCK2
POLR3GL
TK1
NT5E
TK2
UPP1
ENTPD6
ENTPD5
ENTPD3
ENTPD1
NT5C1B
NT5C

ADCY8
ADCY9
ADCY6
AK7
ADCY7
ADCY5
GMPS
ADCY10
POLR3K
POLR1B
PNP
RRM2B
PDE1A
POLR2J2
HPRT1
GART
PAPSS1
PAPSS2
NT5M
ADCY4
IMPDH1
IMPDH2
POLR3H
POLR2J3
POLR1A
POLR3B
GUCY1B1
PKLR
GUCY2C
ENTPD4
PKM
GUCY1A1
GUK1
GUCY2F
ADK
ITPA
POLR1C
URAD
POLE4
RRM1
POLR3D
RRM2
NT5C3A
PDE8B
POLR3GL
NT5E
NPR2
NPR1
PDE10A
ENTPD6
ENTPD2
NME7
AK3
ZNRD1

ENTPD5
ENTPD3
NUDT5
ENTPD1
PFAS
NT5C1B
APRT
PRPS1L1
PPAT
NME7
NT5C
ALLC
ZNRD1
