# Supplementary material for: Targeting serine hydroxymethyltransferases 1 and 2 for T-cell acute lymphoblastic leukemia therapy
Source: Leukemia. 2021 Aug 2;36(2):348–60. doi: 10.1038/s41375-021-01361-8 (PMC8807390; doi:10.1038/s41375-021-01361-8)
Supplement: Supplementary file 26 — Supplementary Table 7 [file 41375_2021_1361_MOESM26_ESM.pdf]

Supplementary Table 7. Transcriptional effect induced by RZ-2994 vs. DMSO treatment at Day 1 and at Day 3 on KOPTK1 cells. This data is summarized in Figure 3 and Supplementary Figure 6.

Spreadsheet tabs

A Gene set enrichment for the transcriptional changes induced by RZ-2994 at Day 3 on KOPTK1 cells.
B Gene set enrichment for the transcriptional changes induced by RZ-2994 at Day 1 on KOPTK1 cells.
C Gene expression changes induced by RZ-2994 vs. DMSO at Day 3 on KOPTK1 cells.
D Gene expression changes induced by RZ-2994 vs. DMSO at Day 1 on KOPTK1 cells.

Supplementary Table 7A. Gene set enrichment for the transcriptional changes induced by RZ-2994 at Day 3 on KOPTK1 cells. MSigDB v7.0 collection, significance -log10(P-value+0.0001)>1. Effect of RZ-2994 on pathway based on Normalized Enrichment Score (NES): NES < -1.5 Repressed, NES > 1.5 Enhanced.

#	Gene set	Normalized Enrichment Score (NES) at Day 3	-log10 (P-value+0.0001) at Day 3	Amino Acid gene set signatures (1=yes, 0=no)	MYC gene set signatures (1=yes, 0=no)	Cell Cycle gene set signatures (1=yes, 0=no)	RZ-2994 vs. DMSO transcriptional effect on pathway at Day 3
1	MTOR_UP.N4.V1_UP	-3.01	4.00	0	0	0	Repressed
2	PENG_RAPAMYCIN_RESPONSE_DN	-2.97	4.00	0	0	0	Repressed
3	PENG_LEUCINE_DEPRIVATION_DN	-2.93	4.00	1	0	0	Repressed
4	PENG_GLUTAMINE_DEPRIVATION_DN	-2.90	4.00	1	0	0	Repressed
5	HALLMARK_MTORC1_SIGNALING	-2.74	4.00	0	0	0	Repressed
6	GO_INNER_MITOCHONDRIAL_MEMBRANE_PROTEIN_COMPLEX	-2.73	4.00	0	0	0	Repressed
7	GO_MITOCHONDRIAL_PROTEIN_COMPLEX	-2.73	4.00	0	0	0	Repressed
8	GARY_CD5_TARGETS_DN	-2.66	4.00	0	0	0	Repressed
9	RHODES_UNDIFFERENTIATED_CANCER	-2.63	4.00	0	0	0	Repressed
10	GO_RESPIRATORY_CHAIN_COMPLEX	-2.62	4.00	0	0	0	Repressed
11	MANALO_HYPOXIA_DN	-2.61	4.00	0	0	0	Repressed
12	REACTOME_THE_ROLE_OF_GTSE1_IN_G2_M_PROGRESSION_AFTER_G	-2.61	4.00	0	0	1	Repressed
13	TARTE_PLASMA_CELL_VS_PLASMABLAST_DN	-2.61	4.00	0	0	0	Repressed
14	REACTOME_RESPIRATORY_ELECTRON_TRANSPORT	-2.60	4.00	0	0	0	Repressed
15	TIEN_INTESTINE_PROBIOTICS_24HR_UP	-2.60	4.00	0	0	0	Repressed
16	SOTIRIOU_BREAST_CANCER_GRADE_1_VS_3_UP	-2.59	4.00	0	0	0	Repressed
17	GO_OXIDATIVE_PHOSPHORYLATION	-2.58	4.00	0	0	0	Repressed
18	GO_ELECTRON_TRANSPORT_CHAIN	-2.57	4.00	0	0	0	Repressed
19	MOOTHA_VOXPHOS	-2.56	4.00	0	0	0	Repressed
20	GO_MITOCHONDRIAL_MEMBRANE_PART	-2.55	4.00	0	0	0	Repressed
21	GO_RESPIRATORY_ELECTRON_TRANSPORT_CHAIN	-2.55	4.00	0	0	0	Repressed
22	WEI_MYCN_TARGETS_WITH_E_BOX	-2.55	4.00	0	1	0	Repressed
23	KRIGE_RESPONSE_TO_TOSEDOSTAT_24HR_DN	-2.54	4.00	0	0	0	Repressed
24	KEGG_PROTEASOME	-2.50	4.00	0	0	0	Repressed
25	GO_REGULATION_OF_CELLULAR_AMINO_ACID_METABOLIC_PROCESS	-2.49	4.00	0	0	0	Repressed
26	GO_RESPIRASOME	-2.48	4.00	0	0	0	Repressed
27	REACTOME_ACTIVATION_OF_APC_C_AND_APC_C:CDC20_MEDIATED_DE	-2.48	4.00	0	0	0	Repressed
28	REACTOME_REGULATION_OF_MITOTIC_CELL_CYCLE	-2.48	4.00	0	0	0	Repressed
29	GO_ATP_SYNTHESIS_COUPLED_ELECTRON_TRANSPORT	-2.47	4.00	0	0	0	Repressed
30	GO_REGULATION_OF_CELLULAR_AMINE_METABOLIC_PROCESS	-2.47	4.00	0	0	0	Repressed
31	GO_ANAPHASE_PROMOTING_COMPLEX_DEPENDENT_CATABOLIC_PROC	-2.46	4.00	0	0	0	Repressed
32	REACTOME_APC_C:CDH1_MEDIATED_DEGRADATION_OF_CDC20_AND_	-2.46	4.00	0	0	0	Repressed
33	REACTOME_METABOLISM_OF_POLYAMINES	-2.46	4.00	0	0	0	Repressed
34	REACTOME_RESPIRATORY_ELECTRON_TRANSPORT_ATP_SYNTHESIS_	-2.46	4.00	0	0	0	Repressed
35	YAO_TEMPORAL_RESPONSE_TO_PROGESTERONE_CLUSTER_11	-2.46	4.00	0	0	0	Repressed
36	HALLMARK_MYC_TARGETS_V2	-2.45	4.00	0	1	0	Repressed
37	REACTOME_MITOTIC_METAPHASE_AND_ANAPHASE	-2.45	4.00	0	0	1	Repressed
38	SCHLOSSER_MYC_TARGETS_AND_SERUM_RESPONSE_DN	-2.45	4.00	0	1	0	Repressed
39	GO_TRANSLATIONAL_TERMINATION	-2.44	4.00	0	0	0	Repressed
40	REICHERT_MITOSIS_LIN9_TARGETS	-2.44	4.00	0	0	1	Repressed
41	GO_MITOCHONDRIAL_RESPIRATORY_CHAIN_COMPLEX_I	-2.43	4.00	0	0	0	Repressed
42	GO_OXIDOREDUCTASE_ACTIVITY_ACTING_ON_NAD_P_H_QUINONE_OR_	-2.43	4.00	0	0	0	Repressed
43	REACTOME_CROSS_PRESENTATION_OF_SOLUBLE_EXOGENOUS_ANTIG	-2.43	4.00	0	0	0	Repressed
44	REACTOME_TRNA_AMINOACYLATION	-2.43	4.00	0	0	0	Repressed
45	SHIPP_DLBCL_VS_FOLLICULAR_LYMPHOMA_UP	-2.43	4.00	0	0	0	Repressed
46	GO_MITOCHONDRIAL_ELECTRON_TRANSPORT_NADH_TO_UBIQUINONE	-2.42	4.00	0	0	0	Repressed
47	GO_MITOCHONDRIAL_RESPIRATORY_CHAIN_COMPLEX_ASSEMBLY	-2.42	4.00	0	0	0	Repressed
48	GO_NADH_DEHYDROGENASE_ACTIVITY	-2.42	4.00	0	0	0	Repressed
49	KRIGE_RESPONSE_TO_TOSEDOSTAT_6HR_DN	-2.42	4.00	0	0	0	Repressed
50	REACTOME_COMPLEX_I_BIOGENESIS	-2.42	4.00	0	0	0	Repressed
51	REACTOME_DEGRADATION_OF_GLI1_BY_THE_PROTEASOME	-2.41	4.00	0	0	0	Repressed
52	REACTOME_SIGNALING_BY_THE_B_CELL_RECEPTOR_BCR	-2.41	4.00	0	0	0	Repressed
53	GO_PRERIBOSOME	-2.40	4.00	0	0	0	Repressed
54	GO_ORGANELLAR_RIBOSOME	-2.39	4.00	0	0	0	Repressed
55	KARLSSON_TGFB1_TARGETS_UP	-2.39	4.00	0	0	0	Repressed
56	REACTOME_FBXL7_DOWN_REGULATES_AURKA_DURING_MITOTIC_ENTR	-2.39	4.00	0	0	0	Repressed
57	REACTOME_HOST_INTERACTIONS_OF_HIV_FACTORS	-2.39	4.00	0	0	0	Repressed
58	GO_NADH_DEHYDROGENASE_COMPLEX_ASSEMBLY	-2.38	4.00	0	0	0	Repressed
59	BERENJENO_TRANSFORMED_BY_RHOA_UP	-2.37	4.00	0	0	0	Repressed
60	COLLER_MYC_TARGETS_UP	-2.37	4.00	0	1	0	Repressed
61	FOURNIER_ACINAR_DEVELOPMENT_LATE_2	-2.37	4.00	0	0	0	Repressed
62	GO_ELECTRON_TRANSFER_ACTIVITY	-2.37	4.00	0	0	0	Repressed
63	GO_MITOCHONDRIAL_GENE_EXPRESSION	-2.37	4.00	0	0	0	Repressed
64	REACTOME_DOWNSTREAM_SIGNALING_EVENTS_OF_B_CELL_RECEPTO	-2.36	4.00	0	0	0	Repressed
65	REACTOME_MITOCHONDRIAL_TRANSLATION	-2.36	4.00	0	0	0	Repressed
66	ELVIDGE_HYPOXIA_DN	-2.35	4.00	0	0	0	Repressed
67	GO_MITOCHONDRIAL_TRANSLATIONAL_TERMINATION	-2.35	4.00	0	0	0	Repressed
68	LEE_BMP2_TARGETS_DN	-2.35	4.00	0	0	0	Repressed
69	REACTOME_HIV_INFECTION	-2.35	4.00	0	0	0	Repressed
70	REACTOME_REGULATION_OF_RAS_BY_GAPS	-2.35	4.00	0	0	0	Repressed
71	REACTOME_THE_CITRIC_ACID_TCA_CYCLE_AND_RESPIRATORY_ELECT	-2.34	4.00	0	0	0	Repressed
72	SCHUHMACHER_MYC_TARGETS_UP	-2.34	4.00	0	1	0	Repressed
73	GO_ANTIGEN_PROCESSING_AND_PRESENTATION_OF_EXOGENOUS_PE	-2.33	4.00	0	0	0	Repressed
74	GO_ENDOPEPTIDASE_COMPLEX	-2.33	4.00	0	0	0	Repressed
75	HALLMARK_MYC_TARGETS_V1	-2.33	4.00	0	1	0	Repressed
76	REACTOME_ACTIVATION_OF_NF_KAPPAB_IN_B_CELLS	-2.33	4.00	0	0	0	Repressed
77	WONG_EMBRYONIC_STEM_CELL_CORE	-2.33	4.00	0	0	0	Repressed
78	HALLMARK_OXIDATIVE_PHOSPHORYLATION	-2.32	4.00	0	0	0	Repressed
79	REACTOME_VIF_MEDIATED_DEGRADATION_OF_APOBEC3G	-2.32	4.00	0	0	0	Repressed
80	GO_GENERATION_OF_PRECURSOR_METABOLITES_AND_ENERGY	-2.31	4.00	0	0	0	Repressed
81	PID_MYC_ACTIV_PATHWAY	-2.31	4.00	0	1	0	Repressed
82	REACTOME_REGULATION_OF_APOPTOSIS	-2.31	4.00	0	0	0	Repressed
83	YU_MYC_TARGETS_UP	-2.31	4.00	0	1	0	Repressed
84	GO_CELLULAR_RESPIRATION	-2.30	4.00	0	0	0	Repressed
85	GO_MITOCHONDRIAL_TRANSLATION	-2.30	4.00	0	0	0	Repressed
86	GO_PEPTIDASE_COMPLEX	-2.30	4.00	0	0	0	Repressed
87	NAKAMURA_CANCER_MICROENVIRONMENT_DN	-2.30	4.00	0	0	0	Repressed
88	NAKAYAMA_SOFT_TISSUE_TUMORS_PCA2_UP	-2.30	4.00	0	0	0	Repressed
89	REACTOME_REGULATION_OF_RUNX2_EXPRESSION_AND_ACTIVITY	-2.30	4.00	0	0	0	Repressed
90	REACTOME_RRNA_MODIFICATION_IN_THE_NUCLEUS_AND_CYTOSOL	-2.30	4.00	0	0	0	Repressed
91	ROSTY_CERVICAL_CANCER_PROLIFERATION_CLUSTER	-2.30	4.00	0	0	0	Repressed
92	TANG_SENESCENCE_TP53_TARGETS_DN	-2.30	4.00	0	0	0	Repressed
93	WINTER_HYPOXIA_UP	-2.30	4.00	0	0	0	Repressed
94	REACTOME_CYTOSOLIC_TRNA_AMINOACYLATION	-2.29	4.00	0	0	0	Repressed
95	REACTOME_NEGATIVE_REGULATION_OF_NOTCH4_SIGNALING	-2.29	4.00	0	0	0	Repressed
96	SHEDDEN_LUNG_CANCER_POOR_SURVIVAL_A6	-2.29	4.00	0	0	0	Repressed
97	BASAKI_YBX1_TARGETS_UP	-2.28	4.00	0	0	0	Repressed

98	GO_PROTEASOME_ACCESSORY_COMPLEX	-2.28	4.00	0	0	0	Repressed
99	RAHMAN_TP53_TARGETS_PHOSPHORYLATED	-2.28	4.00	0	0	0	Repressed
100	GO_TRNA_METABOLIC_PROCESS	-2.27	4.00	1	0	0	Repressed
101	GO_ANTIGEN_PROCESSING_AND_PRESENTATION_OF_PEPTIDE_ANTIGEI	-2.26	4.00	0	0	0	Repressed
102	GO_MITOCHONDRIAL_MATRIX	-2.26	4.00	0	0	0	Repressed
103	GO_MITOCHONDRIAL_PART	-2.26	4.00	0	0	0	Repressed
104	GO_OXIDOREDUCTASE_COMPLEX	-2.26	4.00	0	0	0	Repressed
105	KEGG_AMINOACYL_TRNA_BIOSYNTHESIS	-2.26	4.00	1	0	0	Repressed
106	MOOTHA_HUMAN_MITODB_6_2002	-2.26	4.00	0	0	0	Repressed
107	GO_OXIDOREDUCTASE_ACTIVITY_ACTING_ON_NAD_P_H	-2.25	4.00	0	0	0	Repressed
108	GRAHAM_CML_DIVIDING_VS_NORMAL_QUIESCENT_UP	-2.25	4.00	0	0	0	Repressed
109	REACTOME_REGULATION_OF_RUNX3_EXPRESSION_AND_ACTIVITY	-2.25	4.00	0	0	0	Repressed
110	WONG_MITOCHONDRIA_GENE_MODULE	-2.25	4.00	0	0	0	Repressed
111	GO_CATALYTIC_ACTIVITY_ACTING_ON_A_TRNA	-2.24	4.00	0	0	0	Repressed
112	GO_SMALL_NUCLEOLAR_RIBONUCLEOPROTEIN_COMPLEX	-2.24	4.00	0	0	0	Repressed
113	LY_AGING_OLD_DN	-2.24	4.00	0	0	0	Repressed
114	SEMENZA_HIF1_TARGETS	-2.24	4.00	0	0	0	Repressed
115	YAO_TEMPORAL_RESPONSE_TO_PROGESTERONE_CLUSTER_13	-2.24	4.00	0	0	0	Repressed
116	GO_SMALL_SUBUNIT_PROCESSOME	-2.23	4.00	0	0	0	Repressed
117	KEGG_OXIDATIVE_PHOSPHORYLATION	-2.23	4.00	0	0	0	Repressed
118	MORI_LARGE_PRE_BII_LYMPHOCYTE_UP	-2.23	4.00	0	0	0	Repressed
119	PUJANA_CHEK2_PCC_NETWORK	-2.23	4.00	0	0	1	Repressed
120	REACTOME_CELLULAR_RESPONSE_TO_HYPOXIA	-2.23	4.00	0	0	0	Repressed
121	ZHOU_CELL_CYCLE_GENES_IN_IR_RESPONSE_24HR	-2.23	4.00	0	0	1	Repressed
122	GO_REGULATION_OF_TRANSCRIPTION_FROM_RNA_POLYMERASE_II_PR	-2.22	4.00	0	0	0	Repressed
123	MENSSEN_MYC_TARGETS	-2.22	4.00	0	1	0	Repressed
124	ODONNELL_TARGETS_OF_MYC_AND_TFRC_DN	-2.22	4.00	0	0	0	Repressed
125	REACTOME_DOWNSTREAM_TCR_SIGNALING	-2.22	4.00	0	0	0	Repressed
126	GO_ORGANELLAR_LARGE_RIBOSOMAL_SUBUNIT	-2.21	4.00	0	0	0	Repressed
127	ONO_FOXP3_TARGETS_UP	-2.21	4.00	0	0	0	Repressed
128	REACTOME_DEGRADATION_OF_AXIN	-2.21	4.00	0	0	0	Repressed
129	ZHAN_MULTIPLE_MYELOMA_CD1_VS_CD2_UP	-2.21	4.00	0	0	0	Repressed
130	ELVIDGE_HIF1A_TARGETS_UP	-2.20	4.00	0	0	0	Repressed
131	GO_MITOCHONDRIAL_ENVELOPE	-2.20	4.00	0	0	0	Repressed
132	REACTOME_AUF1_HNRNP_D0_BINDS_AND_DESTABILIZES_MRNA	-2.19	4.00	0	0	0	Repressed
133	WINNEPENNINCKX_MELANOMA_METASTASIS_UP	-2.19	4.00	0	0	0	Repressed
134	FISCHER_G2_M_CELL_CYCLE	-2.18	4.00	0	0	1	Repressed
135	KANG_GIST_WITH_PDGFRA_UP	-2.18	4.00	0	0	0	Repressed
136	REACTOME_DECTIN_1_MEDIATED_NONCANONICAL_NF_KB_SIGNALING	-2.18	4.00	0	0	0	Repressed
137	REACTOME_DEGRADATION_OF_DVL	-2.18	4.00	0	0	0	Repressed
138	REACTOME_GLUCOSE_METABOLISM	-2.18	4.00	0	0	0	Repressed
139	BOYAULT_LIVER_CANCER_SUBCLASS_G23_UP	-2.17	4.00	0	0	0	Repressed
140	ELVIDGE_HYPOXIA_BY_DMOG_DN	-2.17	4.00	0	0	0	Repressed
141	GAVIN_FOXP3_TARGETS_CLUSTER_P6	-2.17	4.00	0	0	0	Repressed
142	GO_90S_PRERIBOSOME	-2.17	4.00	0	0	0	Repressed
143	REACTOME_ASYMMETRIC_LOCALIZATION_OF_PCP_PROTEINS	-2.17	4.00	0	0	0	Repressed
144	CROONQUIST_IL6_DEPRIVATION_DN	-2.16	4.00	0	0	0	Repressed
145	FARMER_BREAST_CANCER_CLUSTER_2	-2.16	4.00	0	0	0	Repressed
146	GO_AMINO_ACID_ACTIVATION	-2.16	4.00	0	0	0	Repressed
147	KIM_MYC_AMPLIFICATION_TARGETS_UP	-2.16	4.00	0	1	0	Repressed
148	CHANG_CORE_SERUM_RESPONSE_UP	-2.15	4.00	0	0	0	Repressed
149	GO_RNA_LOCALIZATION	-2.15	4.00	0	0	0	Repressed
150	GO_TRNA_METHYLATION	-2.15	4.00	0	0	0	Repressed
151	ODONNELL_TFRC_TARGETS_DN	-2.15	4.00	0	0	0	Repressed
152	REACTOME_CDK_MEDIATED_PHOSPHORYLATION_AND_REMOVAL_OF_C	-2.15	4.00	0	0	0	Repressed
153	REACTOME_GLYCOLYSIS	-2.15	4.00	0	0	0	Repressed
154	REACTOME_TRNA_PROCESSING	-2.15	4.00	0	0	0	Repressed
155	WILLIAMS_ESR1_TARGETS_UP	-2.15	4.00	0	0	0	Repressed
156	BASSO_B_LYMPHOCYTE_NETWORK	-2.14	4.00	0	0	0	Repressed
157	CASORELLI_ACUTE_PROMYELOCYTIC_LEUKEMIA_DN	-2.14	4.00	0	0	0	Repressed
158	GO_CYTOKINE_RECEPTOR_ACTIVITY	-2.14	4.00	0	0	0	Repressed
159	GO_NCRNA_METABOLIC_PROCESS	-2.14	4.00	0	0	0	Repressed
160	KRIGE_AMINO_ACID_DEPRIVATION	-2.14	4.00	0	0	0	Repressed
161	MOOTHA_GLUCONEOGENESIS	-2.14	4.00	0	0	0	Repressed
162	RODRIGUES_THYROID_CARCINOMA_POORLY_DIFFERENTIATED_UP	-2.14	4.00	0	0	0	Repressed
163	GO_NCRNA_PROCESSING	-2.13	4.00	0	0	0	Repressed
164	KEGG_GLYCOLYSIS_GLUCONEOGENESIS	-2.13	4.00	0	0	0	Repressed
165	NAKAMURA_TUMOR_ZONE_PERIPHERAL_VS_CENTRAL_UP	-2.13	4.00	0	0	0	Repressed
166	GO_ANAPHASE_PROMOTING_COMPLEX_BINDING	-2.12	4.00	0	0	0	Repressed
167	HALLMARK_UNFOLDED_PROTEIN_RESPONSE	-2.12	4.00	0	0	0	Repressed
168	BURTON_ADIPOGENESIS_PEAK_AT_24HR	-2.11	4.00	0	0	0	Repressed
169	GO_ORGANELLE_INNER_MEMBRANE	-2.11	4.00	0	0	0	Repressed
170	GO_TRNA_METHYLTRANSFERASE_ACTIVITY	-2.11	4.00	0	0	0	Repressed
171	MUELLER_PLURINET	-2.11	4.00	0	0	0	Repressed
172	REACTOME_FCERI_MEDIATED_NF_KB_ACTIVATION	-2.11	4.00	0	0	0	Repressed
173	REACTOME_SCF_SKP2_MEDIATED_DEGRADATION_OF_P27_P21	-2.11	4.00	0	0	0	Repressed
174	REACTOME_TCR_SIGNALING	-2.11	4.00	0	0	0	Repressed
175	REACTOME_UCH_PROTEINASES	-2.11	4.00	0	0	0	Repressed
176	AMUNDSON_GAMMA_RADIATION_RESPONSE	-2.10	4.00	0	0	0	Repressed
177	GO_CELLULAR_PROTEIN_COMPLEX_DISASSEMBLY	-2.10	4.00	0	0	0	Repressed
178	GO_LIGASE_ACTIVITY_FORMING_CARBON_OXYGEN_BONDS	-2.10	4.00	0	0	0	Repressed
179	GO_RNA_MODIFICATION	-2.10	4.00	0	0	0	Repressed
180	REACTOME_MAPK6_MAPK4_SIGNALING	-2.10	4.00	0	0	0	Repressed
181	WEST_ADRENOCORTICAL_TUMOR_UP	-2.10	4.00	0	0	0	Repressed
182	GO_INTRINSIC_COMPONENT_OF_MITOCHONDRIAL_INNER_MEMBRANE	-2.09	4.00	0	0	0	Repressed
183	GO_REGULATION_OF_DNA_TEMPLATED_TRANSCRIPTION_IN_RESPONSE	-2.09	4.00	0	0	0	Repressed
184	HOFFMANN_LARGE_TO_SMALL_PRE_BII_LYMPHOCYTE_UP	-2.09	4.00	0	0	0	Repressed
185	KEGG_FRUCTOSE_AND_MANNOSE_METABOLISM	-2.09	4.00	0	0	0	Repressed
186	MOOTHA_MITOCHONDRIA	-2.09	4.00	0	0	0	Repressed
187	MOREAUX_B_LYMPHOCYTE_MATURATION_BY_TACI_DN	-2.09	4.00	0	0	0	Repressed
188	REACTOME_FORMATION_OF_TUBULIN_FOLDING_INTERMEDIATES_BY_C	-2.09	4.00	0	0	0	Repressed
189	SIMBULAN_PARP1_TARGETS_DN	-2.09	4.00	0	0	0	Repressed
190	VECCHI_GASTRIC_CANCER_EARLY_UP	-2.09	4.00	0	0	0	Repressed
191	GO_AMINE_METABOLIC_PROCESS	-2.08	4.00	0	0	0	Repressed
192	GO_CELLULAR_AMINO_ACID_METABOLIC_PROCESS	-2.08	4.00	0	0	0	Repressed
193	GO_CYTOCHROME_COMPLEX	-2.08	4.00	0	0	0	Repressed
194	KEGG_PARKINSONS_DISEASE	-2.08	4.00	0	0	0	Repressed
195	KORKOLA_TERATOMA	-2.08	4.00	0	0	0	Repressed
196	LI_DCP2_BOUND_MRNA	-2.08	4.00	0	0	0	Repressed
197	MOOTHA_PGC	-2.08	4.00	0	0	0	Repressed
198	REACTOME_ANTIGEN_PROCESSING_CROSS_PRESENTATION	-2.08	4.00	0	0	0	Repressed
199	SCHLOSSER_MYC_TARGETS_REPRESSED_BY_SERUM	-2.08	4.00	0	1	0	Repressed
200	YAO_TEMPORAL_RESPONSE_TO_PROGESTERONE_CLUSTER_14	-2.08	4.00	0	0	0	Repressed
201	BURTON_ADIPOGENESIS_5	-2.07	4.00	0	0	0	Repressed
202	GO_ENERGY_DERIVATION_BY_OXIDATION_OF_ORGANIC_COMPOUNDS	-2.07	4.00	0	0	0	Repressed
203	GO_INNATE_IMMUNE_RESPONSE_ACTIVATING_CELL_SURFACE_RECEPT	-2.07	4.00	0	0	0	Repressed
204	GO_MITOCHONDRION	-2.07	4.00	0	0	0	Repressed
205	HONMA_DOCETAXEL_RESISTANCE	-2.07	4.00	0	0	0	Repressed
206	REACTOME_BETA_CATENIN_INDEPENDENT_WNT_SIGNALING	-2.07	4.00	0	0	0	Repressed

207	REACTOME_HEDGEHOG_LIGAND_BIOGENESIS	-2.07	4.00	0	0	0	Repressed
208	REACTOME_PCP_CE_PATHWAY	-2.07	4.00	0	0	0	Repressed
209	REACTOME_STABILIZATION_OF_P53	-2.07	4.00	0	0	0	Repressed
210	XU_RESPONSE_TO_TRETINOIN_AND_NSC682994_DN	-2.07	2.60	0	0	0	Repressed
211	BIOCARTA_IL12_PATHWAY	-2.06	4.00	0	0	0	Repressed
212	BMI1_DN.V1_DN	-2.06	4.00	0	0	0	Repressed
213	CONCANNON_APOPTOSIS_BY_EPOXOMICIN_DN	-2.06	4.00	0	0	0	Repressed
214	CSR_EARLY_UP.V1_UP	-2.06	4.00	0	0	0	Repressed
215	GO_ESTABLISHMENT_OF_RNA_LOCALIZATION	-2.06	4.00	0	0	0	Repressed
216	GO_REGULATION_OF_CARDIAC_CONDUCTION	-2.06	4.00	0	0	0	Repressed
217	GO_TRNA_MODIFICATION	-2.06	4.00	0	0	0	Repressed
218	REACTOME_SIGNALING_BY_NOTCH4	-2.06	4.00	0	0	0	Repressed
219	SERVITJA_LIVER_HNF1A_TARGETS_UP	-2.06	4.00	0	0	0	Repressed
220	SHAFFER_IRF4_TARGETS_IN_ACTIVATED_B_LYMPHOCYTE	-2.06	4.00	0	0	0	Repressed
221	GO_INTRINSIC_COMPONENT_OF_MITOCHONDRIAL_MEMBRANE	-2.05	4.00	0	0	0	Repressed
222	GO_REGULATION_OF_CELLULAR_KETONE_METABOLIC_PROCESS	-2.05	4.00	0	0	0	Repressed
223	HESS_TARGETS_OF_HOXA9_AND_MEIS1_UP	-2.05	4.00	0	0	0	Repressed
224	REACTOME_DEGRADATION_OF_BETA_CATENIN_BY_THE_DESTRUCTION	-2.05	4.00	0	0	0	Repressed
225	REACTOME_HEDGEHOG_OFF_STATE	-2.05	4.00	0	0	0	Repressed
226	REACTOME_RESOLUTION_OF_SISTER_CHROMATID_COHESION	-2.05	4.00	0	0	0	Repressed
227	GO_TRNA_PROCESSING	-2.04	4.00	0	0	0	Repressed
228	HALLMARK_G2M_CHECKPOINT	-2.04	4.00	0	0	1	Repressed
229	KEGG_ALZHEIMERS_DISEASE	-2.04	4.00	0	0	0	Repressed
230	KOBAYASHI_EGFR_SIGNALING_24HR_DN	-2.04	4.00	0	0	0	Repressed
231	MOOTHA_GLYCOLYSIS	-2.04	4.00	0	0	0	Repressed
232	OUELLET_OVARIAN_CANCER_INVASIVE_VS_LMP_UP	-2.04	4.00	0	0	0	Repressed
233	REACTOME_MITOCHONDRIAL_PROTEIN_IMPORT	-2.04	4.00	0	0	0	Repressed
234	RODRIGUES_THYROID_CARCINOMA_ANAPLASTIC_UP	-2.04	4.00	0	0	0	Repressed
235	CHR17Q24	-2.03	4.00	0	0	0	Repressed
236	GO_ANTIGEN_PROCESSING_AND_PRESENTATION_OF_PEPTIDE_ANTIGE	-2.03	4.00	0	0	0	Repressed
237	GO_POSITIVE_REGULATION_OF_TELOMERASE_RNA_LOCALIZATION_TO	-2.03	4.00	0	0	0	Repressed
238	REACTOME_ABC_TRANSPORTER_DISORDERS	-2.03	4.00	0	0	0	Repressed
239	RHODES_CANCER_META_SIGNATURE	-2.03	4.00	0	0	0	Repressed
240	SCHLOSSER_MYC_AND_SERUM_RESPONSE_SYNERGY	-2.03	4.00	0	1	0	Repressed
241	TOOKER_GEMCITABINE_RESISTANCE_DN	-2.03	4.00	0	0	0	Repressed
242	WINTER_HYPOXIA_METAGENE	-2.03	4.00	0	0	0	Repressed
243	CHEN_LUNG_CANCER_SURVIVAL	-2.02	4.00	0	0	0	Repressed
244	FLORIO_NEOCORTEX_BASAL_RADIAL_GLIA_DN	-2.02	4.00	0	0	0	Repressed
245	GO_CELLULAR_KETONE_METABOLIC_PROCESS	-2.02	4.00	0	0	0	Repressed
246	GO_NEUTROPHIL_HOMEOSTASIS	-2.02	4.00	0	0	0	Repressed
247	GO_SNORNA_BINDING	-2.02	4.00	0	0	0	Repressed
248	REACTOME_CELL_CYCLE_CHECKPOINTS	-2.02	4.00	0	0	1	Repressed
249	REACTOME_METABOLISM_OF_NON_CODING_RNA	-2.02	4.00	0	0	0	Repressed
250	SCIAN_CELL_CYCLE_TARGETS_OF_TP53_AND_TP73_DN	-2.02	4.00	0	0	0	Repressed
251	WANG_SMARCE1_TARGETS_DN	-2.02	4.00	0	0	0	Repressed
252	FOURNIER_ACINAR_DEVELOPMENT_LATE_DN	-2.02	2.62	0	0	0	Repressed
253	GO_RNA_METHYLTRANSFERASE_ACTIVITY	-2.01	4.00	0	0	0	Repressed
254	RB_P107_DN.V1_DN	-2.01	4.00	0	0	0	Repressed
255	REACTOME_HIV_LIFE_CYCLE	-2.01	4.00	0	0	0	Repressed
256	REACTOME_REGULATION_OF_PTEN_STABILITY_AND_ACTIVITY	-2.01	4.00	0	0	0	Repressed
257	GO_REACTIVE_OXYGEN_SPECIES_METABOLIC_PROCESS	-2.00	4.00	0	0	0	Repressed
258	GO_REGULATION_OF_RIBOSOME_BIOGENESIS	-2.00	4.00	0	0	0	Repressed
259	GO_RIBOSOME_BIOGENESIS	-2.00	4.00	0	0	0	Repressed
260	KEGG_HUNTINGTONS_DISEASE	-2.00	4.00	0	0	0	Repressed
261	MALONEY_RESPONSE_TO_17AAG_DN	-2.00	4.00	0	0	0	Repressed
262	REACTOME_COOPERATION_OF_PREFOLDIN_AND_TRIC_CCT_IN_ACTIN_	-2.00	4.00	0	0	0	Repressed
263	REACTOME_PREFOLDIN_MEDIATED_TRANSFER_OF_SUBSTRATE_TO_C	-2.00	4.00	0	0	0	Repressed
264	YAO_TEMPORAL_RESPONSE_TO_PROGESTERONE_CLUSTER_17	-2.00	4.00	0	0	0	Repressed
265	GO_GLYCOLYTIC_PROCESS_THROUGH_FRUCTOSE_6_PHOSPHATE	-1.99	4.00	0	0	0	Repressed
266	GO_SCF_DEPENDENT_PROTEASOMAL_UBIQUITIN_DEPENDENT_PROTEI	-1.99	4.00	0	0	0	Repressed
267	GOLDRATH_ANTIGEN_RESPONSE	-1.99	4.00	0	0	0	Repressed
268	NABA_ECM_AFFILIATED	-1.99	4.00	0	0	0	Repressed
269	REACTOME_HEDGEHOG_ON_STATE	-1.99	4.00	0	0	0	Repressed
270	REACTOME_HSP90_CHAPERONE_CYCLE_FOR_STEROID_HORMONE_RE	-1.99	4.00	0	0	0	Repressed
271	REACTOME_M_PHASE	-1.99	4.00	0	0	1	Repressed
272	REACTOME_METABOLISM_OF_COFACTORS	-1.99	4.00	0	0	0	Repressed
273	REACTOME_TRANSCRIPTIONAL_REGULATION_BY_RUNX2	-1.99	4.00	0	0	0	Repressed
274	REACTOME_TRANSCRIPTIONAL_REGULATION_BY_RUNX3	-1.99	4.00	0	0	0	Repressed
275	WANG_ADIPOGENIC_GENES_REPRESSED_BY_SIRT1	-1.99	4.00	0	0	0	Repressed
276	KEGG_PROXIMAL_TUBULE_BICARBONATE_RECLAMATION	-1.99	2.63	0	0	0	Repressed
277	GO_RNA_EXPORT_FROM_NUCLEUS	-1.98	4.00	0	0	0	Repressed
278	REACTOME_RNA_POLYMERASE_II_TRANSCRIPTION_PRE_INITIATION_AN	-1.98	4.00	0	0	0	Repressed
279	REACTOME_TNFR2_NON_CANONICAL_NF_KB_PATHWAY	-1.98	4.00	0	0	0	Repressed
280	REACTOME_TRANSCRIPTION_OF_THE_HIV_GENOME	-1.98	4.00	0	0	0	Repressed
281	WANG_TUMOR_INVASIVENESS_UP	-1.98	4.00	0	0	0	Repressed
282	YAO_TEMPORAL_RESPONSE_TO_PROGESTERONE_CLUSTER_10	-1.98	4.00	0	0	0	Repressed
283	BHATI_G2M_ARREST_BY_2METHOXYESTRADIOL_UP	-1.97	4.00	0	0	0	Repressed
284	GO_SARCOPLASMIC_RETICULUM_CALCIUM_ION_TRANSPORT	-1.97	4.00	0	0	0	Repressed
285	GO_TRNA_GUANINE_METHYLTRANSFERASE_ACTIVITY	-1.97	4.00	0	0	0	Repressed
286	KEGG_PPAR_SIGNALING_PATHWAY	-1.97	4.00	0	0	0	Repressed
287	MORI_IMMATURE_B_LYMPHOCYTE_DN	-1.97	4.00	0	0	0	Repressed
288	REACTOME_G_PROTEIN_MEDIATED_EVENTS	-1.97	4.00	0	0	0	Repressed
289	REACTOME_GENE_AND_PROTEIN_EXPRESSION_BY_JAK_STAT_SIGNALI	-1.97	4.00	0	0	0	Repressed
290	REACTOME_REGULATION_OF_MRNA_STABILITY_BY_PROTEINS_THAT_B	-1.97	4.00	0	0	0	Repressed
291	BOYAULT_LIVER_CANCER_SUBCLASS_G3_UP	-1.96	4.00	0	0	0	Repressed
292	CHAUHAN_RESPONSE_TO_METHOXYESTRADIOL_UP	-1.96	4.00	0	0	0	Repressed
293	GO_CYTOSOLIC_PROTEASOME_COMPLEX	-1.96	4.00	0	0	0	Repressed
294	GO_TRANSLATIONAL_ELONGATION	-1.96	4.00	0	0	0	Repressed
295	GO_TRNA_BINDING	-1.96	4.00	0	0	0	Repressed
296	GRUETZMANN_PANCREATIC_CANCER_UP	-1.96	4.00	0	0	0	Repressed
297	HORIUCHI_WTAP_TARGETS_DN	-1.96	4.00	0	0	0	Repressed
298	LY_AGING_MIDDLE_DN	-1.96	4.00	0	0	0	Repressed
299	REACTOME_MITOTIC_G2_G2_M_PHASES	-1.96	4.00	0	0	1	Repressed
300	REACTOME_MITOTIC_SPINDLE_CHECKPOINT	-1.96	4.00	0	0	1	Repressed
301	REACTOME_ORC1_REMOVAL_FROM_CHROMATIN	-1.96	4.00	0	0	0	Repressed
302	REACTOME_REGULATION_OF_CHOLESTEROL_BIOSYNTHESIS_BY_SREB	-1.96	4.00	0	0	0	Repressed
303	REACTOME_SUMOYLATION_OF_DNA_REPLICATION_PROTEINS	-1.96	4.00	0	0	0	Repressed
304	SHETH_LIVER_CANCER_VS_TXNIP_LOSS_PAM3	-1.96	4.00	0	0	0	Repressed
305	TONKS_TARGETS_OF_RUNX1_RUNX1T1_FUSION_HSC_DN	-1.96	4.00	0	0	0	Repressed
306	GO_MITOCHONDRIAL_RNA_PROCESSING	-1.96	2.61	0	0	0	Repressed
307	GO_RNA_POLYMERASE_II_GENERAL_TRANSCRIPTION_INITIATION_FACT	-1.96	2.61	0	0	0	Repressed
308	VALK_AML_CLUSTER_15	-1.96	2.33	0	0	0	Repressed
309	GO_ION_CHANNEL_BINDING	-1.95	4.00	0	0	0	Repressed
310	GO_MITOCHONDRIAL_RNA_METABOLIC_PROCESS	-1.95	4.00	0	0	0	Repressed
311	GO_ORGANELLE_ENVELOPE_LUMEN	-1.95	4.00	0	0	0	Repressed
312	ICHIBA_GRAFT_VERSUS_HOST_DISEASE_35D_UP	-1.95	4.00	0	0	0	Repressed
313	PID_AURORA_B_PATHWAY	-1.95	4.00	0	0	0	Repressed
314	POOLA_INVASIVE_BREAST_CANCER_UP	-1.95	4.00	0	0	0	Repressed
315	REACTOME_DEFECTIVE_CFTR_CAUSES_CYSTIC_FIBROSIS	-1.95	4.00	0	0	0	Repressed

316	REACTOME_RNA_POLYMERASE_II_PRE_TRANSCRIPTION_EVENTS	-1.95	4.00	0	0	0	Repressed
317	REACTOME_TRNA_PROCESSING_IN_THE_NUCLEUS	-1.95	4.00	0	0	0	Repressed
318	WONG_PROTEASOME_GENE_MODULE	-1.95	4.00	0	0	0	Repressed
319	GO_RELEASE_OF_SEQUESTERED_CALCIUM_ION_INTO_CYTOSOL_BY_E	-1.95	2.61	0	0	0	Repressed
320	BENPORATH_PROLIFERATION	-1.94	4.00	0	0	0	Repressed
321	BIOCARTA_NO2IL12_PATHWAY	-1.94	4.00	0	0	0	Repressed
322	GO_ENVELOPE	-1.94	4.00	0	0	0	Repressed
323	GO_RRNA_METABOLIC_PROCESS	-1.94	4.00	0	0	0	Repressed
324	HALLMARK_E2F_TARGETS	-1.94	4.00	0	0	0	Repressed
325	KUMAMOTO_RESPONSE_TO_NUTLIN_3A_DN	-1.94	4.00	0	0	0	Repressed
326	MOHANKUMAR_HOXA1_TARGETS_UP	-1.94	4.00	0	0	0	Repressed
327	REACTOME_PEPTIDE_LIGAND_BINDING_RECEPTORS	-1.94	4.00	0	0	0	Repressed
328	SHEPARD_CRUSH_AND_BURN_MUTANT_UP	-1.94	4.00	0	0	0	Repressed
329	YANG_BREAST_CANCER_ESR1_LASER_DN	-1.94	4.00	0	0	0	Repressed
330	WU_APOPTOSIS_BY_CDKN1A_VIA_TP53	-1.94	2.55	0	0	0	Repressed
331	GO_MYELOID_CELL_APOPTOTIC_PROCESS	-1.93	4.00	0	0	0	Repressed
332	GO_PLASMINOGEN_ACTIVATION	-1.93	4.00	0	0	0	Repressed
333	HALLMARK_GLYCOLYSIS	-1.93	4.00	0	0	0	Repressed
334	KINSEY_TARGETS_OF_EWSR1_FLII_FUSION_UP	-1.93	4.00	0	0	0	Repressed
335	PACHER_TARGETS_OF_IGF1_AND_IGF2_UP	-1.93	4.00	0	0	0	Repressed
336	PELLICCIOTTA_HDAC_IN_ANTIGEN_PRESENTATION_DN	-1.93	4.00	0	0	0	Repressed
337	REACTOME_GLUCONEOGENESIS	-1.93	4.00	0	0	0	Repressed
338	REACTOME_ION_HOMEOSTASIS	-1.93	4.00	0	0	0	Repressed
339	SAKAI_TUMOR_INFILTRATING_MONOCYTES_DN	-1.93	4.00	0	0	0	Repressed
340	BLALOCK_ALZHEIMERS_DISEASE_DN	-1.92	4.00	0	0	0	Repressed
341	GO_ADP_BINDING	-1.92	4.00	0	0	0	Repressed
342	GO_CATALYTIC_ACTIVITY_ACTING_ON_RNA	-1.92	4.00	0	0	0	Repressed
343	GO_HEMATOPOIETIC_STEM_CELL_DIFFERENTIATION	-1.92	4.00	0	0	0	Repressed
344	GO_LYMPHOCYTE_CHEMOTAXIS	-1.92	4.00	0	0	0	Repressed
345	GO_PROTEIN_HETEROTETRAMERIZATION	-1.92	4.00	0	0	0	Repressed
346	GO_RESPIRATORY_CHAIN_COMPLEX_IV_ASSEMBLY	-1.92	4.00	0	0	0	Repressed
347	GRADE_COLON_AND_RECTAL_CANCER_UP	-1.92	4.00	0	0	0	Repressed
348	HWANG_PROSTATE_CANCER_MARKERS	-1.92	4.00	0	0	0	Repressed
349	MATTIOLI_MULTIPLE_MYELOMA_SUBGROUPS	-1.92	4.00	0	0	0	Repressed
350	REACTOME_ASSEMBLY_OF_THE_PRE_REPLICATIVE_COMPLEX	-1.92	4.00	0	0	0	Repressed
351	REACTOME_EARLY_PHASE_OF_HIV_LIFE_CYCLE	-1.92	4.00	0	0	0	Repressed
352	SESTO_RESPONSE_TO_UV_C0	-1.92	4.00	0	0	0	Repressed
353	WHITFIELD_CELL_CYCLE_M_G1	-1.92	4.00	0	0	1	Repressed
354	COLIN_PILOCYTIC_ASTROCYTOMA_VS_GLIOBLASTOMA_DN	-1.91	4.00	0	0	0	Repressed
355	CROONQUIST_NRAS_SIGNALING_DN	-1.91	4.00	0	0	0	Repressed
356	FARDIN_HYPOXIA_11	-1.91	4.00	0	0	0	Repressed
357	GILDEA_METASTASIS	-1.91	4.00	0	0	0	Repressed
358	GO_FC_EPSILON_RECEPTOR_SIGNALING_PATHWAY	-1.91	4.00	0	0	0	Repressed
359	GO_GENERAL_TRANSCRIPTION_INITIATION_FACTOR_BINDING	-1.91	4.00	0	0	0	Repressed
360	GO_ORGANELLAR_SMALL_RIBOSOMAL_SUBUNIT	-1.91	4.00	0	0	0	Repressed
361	GO_RESPONSE_TO_KETONE	-1.91	4.00	0	0	0	Repressed
362	GO_THIOESTER_METABOLIC_PROCESS	-1.91	4.00	0	0	0	Repressed
363	PELLICCIOTTA_HDAC_IN_ANTIGEN_PRESENTATION_UP	-1.91	4.00	0	0	0	Repressed
364	REACTOME_CLEC7A_DECTIN_1_SIGNALING	-1.91	4.00	0	0	0	Repressed
365	REACTOME_DISORDERS_OF_TRANSMEMBRANE_TRANSPORTERS	-1.91	4.00	0	0	0	Repressed
366	REACTOME_SIGNALING_BY_HEDGEHOG	-1.91	4.00	0	0	0	Repressed
367	ZHAN_MULTIPLE_MYELOMA_SUBGROUPS	-1.91	4.00	0	0	0	Repressed
368	GO_SYNAPSE_MATURATION	-1.91	2.36	0	0	0	Repressed
369	DAIRKEE_CANCER_PRONE_RESPONSE_BPA	-1.90	4.00	0	0	0	Repressed
370	GO_CYTOCHROME_COMPLEX_ASSEMBLY	-1.90	4.00	0	0	0	Repressed
371	GO_INTERLEUKIN_1_MEDIATED_SIGNALING_PATHWAY	-1.90	4.00	0	0	0	Repressed
372	GO_NUCLEAR_EXPORT	-1.90	4.00	0	0	0	Repressed
373	GO_PRERIBOSOME_LARGE_SUBUNIT_PRECURSOR	-1.90	4.00	0	0	0	Repressed
374	GO_TRANSFERASE_ACTIVITY_TRANSFERRING_ALKYL_OR_ARYL_OTHEF	-1.90	4.00	0	0	0	Repressed
375	REACTOME_COOPERATION_OF_PDCL_PHLP1_AND_TRIC_CCT_IN_G_PR	-1.90	4.00	0	0	0	Repressed
376	REACTOME_METABOLISM_OF_RNA	-1.90	4.00	0	0	0	Repressed
377	REACTOME_POST_CHAPERONIN_TUBULIN_FOLDING_PATHWAY	-1.90	4.00	0	0	0	Repressed
378	REACTOME_SWITCHING_OF_ORIGINS_TO_A_POST_REPLICATIVE_STATE	-1.90	4.00	0	0	0	Repressed
379	STARK_PREFRONTAL_CORTEX_22Q11_DELETION_DN	-1.90	4.00	0	0	0	Repressed
380	ZHONG_SECRETOME_OF_LUNG_CANCER_AND_FIBROBLAST	-1.90	4.00	0	0	0	Repressed
381	REACTOME_KINESINS	-1.90	2.58	0	0	0	Repressed
382	GO_POSITIVE_REGULATION_OF_UBIQUITIN_PROTEIN_LIGASE_ACTIVITY	-1.90	2.36	0	0	0	Repressed
383	BOSCO_EPITHELIAL_DIFFERENTIATION_MODULE	-1.90	2.33	0	0	0	Repressed
384	DAIRKEE_CANCER_PRONE_RESPONSE_BPA_E2	-1.89	4.00	0	0	0	Repressed
385	GO_NCRNA_TRANSCRIPTION	-1.89	4.00	0	0	0	Repressed
386	MORI_PRE_BI_LYMPHOCYTE_UP	-1.89	4.00	0	0	0	Repressed
387	REACTOME_TRANSPORT_OF_CONNEXONS_TO_THE_PLASMA_MEMBRAN	-1.89	4.00	0	0	0	Repressed
388	GO_PROTEASOME_REGULATORY_PARTICLE_BASE_SUBCOMPLEX	-1.89	2.62	0	0	0	Repressed
389	GO_TIM23_MITOCHONDRIAL_IMPORT_INNER_MEMBRANE_TRANSLOCASE	-1.89	2.61	0	0	0	Repressed
390	GO_CALCIUM_DEPENDENT_PROTEIN_BINDING	-1.89	2.57	0	0	0	Repressed
391	ZHAN_MULTIPLE_MYELOMA_DN	-1.89	2.33	0	0	0	Repressed
392	CHANG_CYCLING_GENES	-1.88	4.00	0	0	0	Repressed
393	CHIANG_LIVER_CANCER_SUBCLASS_UNANNOTATED_DN	-1.88	4.00	0	0	0	Repressed
394	ELVIDGE_HIF1A_AND_HIF2A_TARGETS_UP	-1.88	4.00	0	0	0	Repressed
395	GO_CELLULAR_RESPONSE_TO_GLUCAGON_STIMULUS	-1.88	4.00	0	0	0	Repressed
396	GO_HEART_PROCESS	-1.88	4.00	0	0	0	Repressed
397	GO_LIPASE_INHIBITOR_ACTIVITY	-1.88	4.00	0	0	0	Repressed
398	GO_POSITIVE_REGULATION_OF_RIBOSOME_BIOGENESIS	-1.88	4.00	0	0	0	Repressed
399	GO_REGULATION_OF_CHOLESTEROL_BIOSYNTHETIC_PROCESS	-1.88	4.00	0	0	0	Repressed
400	GO_RESPONSE_TO_GLUCAGON	-1.88	4.00	0	0	0	Repressed
401	GO_RNA_BINDING	-1.88	4.00	0	0	0	Repressed
402	KANG_DOXORUBICIN_RESISTANCE_UP	-1.88	4.00	0	0	0	Repressed
403	KEGG_OOCYTE_MEIOSIS	-1.88	4.00	0	0	0	Repressed
404	MOHANKUMAR_TLX1_TARGETS_UP	-1.88	4.00	0	0	0	Repressed
405	REACTOME_PHOSPHORYLATION_OF_EMI1	-1.88	4.00	0	0	0	Repressed
406	RPS14_DN.V1_DN	-1.88	4.00	0	0	0	Repressed
407	REACTOME_INWARDLY_RECTIFYING_KPLUS_CHANNELS	-1.88	2.64	0	0	0	Repressed
408	GO_PODOSOME_ASSEMBLY	-1.88	2.32	0	0	0	Repressed
409	REACTOME_INTERLEUKIN_10_SIGNALING	-1.88	2.32	0	0	0	Repressed
410	GO_SPINDLE_MICROTUBULE	-1.88	2.29	0	0	0	Repressed
411	DELLA_RESPONSE_TO_TSA_AND_BUTYRATE	-1.87	4.00	0	0	0	Repressed
412	DOANE_BREAST_CANCER_ESR1_DN	-1.87	4.00	0	0	0	Repressed
413	GO_ANTIGEN_PROCESSING_AND_PRESENTATION	-1.87	4.00	0	0	0	Repressed
414	GO_NEGATIVE_REGULATION_OF_CELL_CYCLE_G2_M_PHASE_TRANSITI(	-1.87	4.00	0	0	0	Repressed
415	GO_REGULATION_OF_ESTABLISHMENT_OF_PLANAR_POLARITY	-1.87	4.00	0	0	0	Repressed
416	GO_STEROL_BIOSYNTHETIC_PROCESS	-1.87	4.00	0	0	0	Repressed
417	KAAB_HEART_ATRIUM_VS_VENTRICLE_DN	-1.87	4.00	0	0	0	Repressed
418	KORKOLA_SEMINOMA_UP	-1.87	4.00	0	0	0	Repressed
419	LI_WILMS_TUMOR_VS_FETAL_KIDNEY_1_DN	-1.87	4.00	0	0	0	Repressed
420	QI_PLASMACYTOMA_UP	-1.87	4.00	0	0	0	Repressed
421	REACTOME_PROGRAMMED_CELL_DEATH	-1.87	4.00	0	0	0	Repressed
422	REACTOME_RUNX1_REGULATES_TRANSCRIPTION_OF_GENES_INVOLVEI	-1.87	4.00	0	0	0	Repressed
423	NAKAYAMA_SOFT_TISSUE_TUMORS_PCA2_DN	-1.87	2.61	0	0	0	Repressed
424	PID_RXR_VDR_PATHWAY	-1.87	2.61	0	0	0	Repressed

425	REACTOME_ACTIVATION_OF_NIMA_KINASES_NEK9_NEK6_NEK7	-1.87	2.34	0	0	0	Repressed
426	CYCLIN_D1_UP.V1_UP	-1.86	4.00	0	0	0	Repressed
427	DIAZ_CHRONIC_MEYLOGENOUS_LEUKEMIA_UP	-1.86	4.00	0	0	0	Repressed
428	GO_CHROMOSOME_MOVEMENT_TOWARDS_SPINDLE_POLE	-1.86	4.00	0	0	0	Repressed
429	GO_VITAMIN_TRANSMEMBRANE_TRANSPORT	-1.86	4.00	0	0	0	Repressed
430	JAEGER_METASTASIS_UP	-1.86	4.00	0	0	0	Repressed
431	JISON_SICKLE_CELL_DISEASE_UP	-1.86	4.00	0	0	0	Repressed
432	KEGG_SPLICEOSOME	-1.86	4.00	0	0	0	Repressed
433	REACTOME_ATF6_ATF6_ALPHA_ACTIVATES_CHAPERONE_GENES	-1.86	4.00	0	0	0	Repressed
434	REACTOME_C_TYPE_LECTIN_RECEPTORS_CLRS	-1.86	4.00	0	0	0	Repressed
435	REACTOME_CELLULAR_RESPONSES_TO_STRESS	-1.86	4.00	0	0	0	Repressed
436	REACTOME_G2_M_CHECKPOINTS	-1.86	4.00	0	0	0	Repressed
437	REACTOME_HOST_INTERACTIONS_WITH_INFLUENZA_FACTORS	-1.86	4.00	0	0	0	Repressed
438	REACTOME_NUCLEAR_PORE_COMPLEX_NPC_DISASSEMBLY	-1.86	4.00	0	0	0	Repressed
439	REACTOME_REGULATION_OF_HSF1_MEDIATED_HEAT_SHOCK_RESPONS	-1.86	4.00	0	0	0	Repressed
440	TAVOR_CEBPA_TARGETS_UP	-1.86	4.00	0	0	0	Repressed
441	VEGF_A_UP.V1_DN	-1.86	4.00	0	0	0	Repressed
442	WANG_RESPONSE_TO_GSK3_INHIBITOR_SB216763_DN	-1.86	4.00	0	0	0	Repressed
443	WHITFIELD_CELL_CYCLE_G2_M	-1.86	4.00	0	0	0	Repressed
444	FUNG_IL2_SIGNALING_1	-1.86	2.62	0	0	0	Repressed
445	GO_RNA_CAPPING	-1.86	2.60	0	0	0	Repressed
446	KEGG_CITRATE_CYCLE_TCA_CYCLE	-1.86	2.58	0	0	0	Repressed
447	BHAT_ESR1_TARGETS_NOT_VIA_AKT1_UP	-1.85	4.00	0	0	0	Repressed
448	GO_OXIDOREDUCTASE_ACTIVITY_ACTING_ON_A_HEME_GROUP_OF_DO	-1.85	4.00	0	0	0	Repressed
449	GO_RESPONSE_TO_CORTICOSTEROID	-1.85	4.00	0	0	0	Repressed
450	GO_T_CELL_CHEMOTAXIS	-1.85	4.00	0	0	0	Repressed
451	KLEIN_PRIMARY_EFFUSION_LYMPHOMA_DN	-1.85	4.00	0	0	0	Repressed
452	LABBE_TARGETS_OF_TGFB1_AND_WNT3A_UP	-1.85	4.00	0	0	0	Repressed
453	MEINHOLD_OVARIAN_CANCER_LOW_GRADE_DN	-1.85	4.00	0	0	0	Repressed
454	MEL18_DN.V1_DN	-1.85	4.00	0	0	0	Repressed
455	PUJANA_BRCA1_PCC_NETWORK	-1.85	4.00	0	0	0	Repressed
456	REACTOME_DNA_REPLICATION_PRE_INITIATION	-1.85	4.00	0	0	0	Repressed
457	REACTOME_G1_S_DNA_DAMAGE_CHECKPOINTS	-1.85	4.00	0	0	0	Repressed
458	REACTOME_INHIBITION_OF_THE_PROTEOLYTIC_ACTIVITY_OF_APC_C_R	-1.85	4.00	0	0	0	Repressed
459	REACTOME_INTERLEUKIN_1_SIGNALING	-1.85	4.00	0	0	0	Repressed
460	WHITFIELD_CELL_CYCLE_LITERATURE	-1.85	4.00	0	0	0	Repressed
461	GREENBAUM_E2A_TARGETS_UP	-1.85	2.61	0	0	0	Repressed
462	FURUKAWA_DUSP6_TARGETS_PCI35_DN	-1.85	2.56	0	0	0	Repressed
463	GO_TOXIN_TRANSPORT	-1.85	2.31	0	0	0	Repressed
464	GO_MITOCHONDRIAL_RESPIRATORY_CHAIN_COMPLEX_IV_ASSEMBLY	-1.85	2.15	0	0	0	Repressed
465	GO_RESPONSE_TO_MINERALOCORTICOID	-1.85	2.02	0	0	0	Repressed
466	CAIRO_HEPATOBLASTOMA_CLASSES_UP	-1.84	4.00	0	0	0	Repressed
467	GO_CELLULAR_RESPONSE_TO_HEAT	-1.84	4.00	0	0	0	Repressed
468	GO_OXIDOREDUCTASE_ACTIVITY	-1.84	4.00	0	0	0	Repressed
469	LABBE_WNT3A_TARGETS_UP	-1.84	4.00	0	0	0	Repressed
470	MIKKELSEN_MEF_LCP_WITH_H3K4ME3	-1.84	4.00	0	0	0	Repressed
471	REACTOME_UBIQUINOL_BIOSYNTHESIS	-1.84	4.00	0	0	0	Repressed
472	ZHONG_SECRETOME_OF_LUNG_CANCER_AND_MACROPHAGE	-1.84	4.00	0	0	0	Repressed
473	REACTOME_CHEMOKINE_RECEPTORS_BIND_CHEMOKINES	-1.84	2.64	0	0	0	Repressed
474	REACTOME_PHOSPHORYLATION_OF_THE_APC_C	-1.84	2.62	0	0	0	Repressed
475	NAGASHIMA_EGF_SIGNALING_UP	-1.84	2.59	0	0	0	Repressed
476	GO_NUCLEOSIDE_BISPHOSPHATE_METABOLIC_PROCESS	-1.84	2.55	0	0	0	Repressed
477	REACTOME_GAP_JUNCTION_ASSEMBLY	-1.84	2.35	0	0	0	Repressed
478	GO_RESPONSE_TO_AMINE	-1.84	2.29	0	0	0	Repressed
479	BORCZUK_MALIGNANT_MESOTHELIOMA_UP	-1.83	4.00	0	0	0	Repressed
480	BURTON_ADIPOGENESIS_6	-1.83	4.00	0	0	0	Repressed
481	GO_MITOCHONDRION_MORPHOGENESIS	-1.83	4.00	0	0	0	Repressed
482	GO_MULTI_ORGANISM_MEMBRANE_ORGANIZATION	-1.83	4.00	0	0	0	Repressed
483	GO_RIBONUCLEOPROTEIN_COMPLEX_BIOGENESIS	-1.83	4.00	0	0	0	Repressed
484	GO_SNRNA_PROCESSING	-1.83	4.00	0	0	0	Repressed
485	GO_SPLICEOSOMAL_SNRNP_ASSEMBLY	-1.83	4.00	0	0	0	Repressed
486	GO_TELOMERASE_RNA_LOCALIZATION	-1.83	4.00	0	0	0	Repressed
487	LE_EGR2_TARGETS_UP	-1.83	4.00	0	0	0	Repressed
488	PID_HIF1_TFPATHWAY	-1.83	4.00	0	0	0	Repressed
489	REACTOME_INTERACTIONS_OF_REV_WITH_HOST_CELLULAR_PROTEINS	-1.83	4.00	0	0	0	Repressed
490	REACTOME_INTERLEUKIN_12_SIGNALING	-1.83	4.00	0	0	0	Repressed
491	SESTO_RESPONSE_TO_UV_C7	-1.83	4.00	0	0	0	Repressed
492	GO_PHOSPHOLIPASE_INHIBITOR_ACTIVITY	-1.83	2.68	0	0	0	Repressed
493	BIOCARTA_MHC_PATHWAY	-1.83	2.66	0	0	0	Repressed
494	GO_VENTRICULAR_CARDIAC_MUSCLE_CELL_DIFFERENTIATION	-1.83	2.62	0	0	0	Repressed
495	GO_TBP_CLASS_PROTEIN_BINDING	-1.83	2.60	0	0	0	Repressed
496	FINETTI_BREAST_CANCER_KINOME_RED	-1.83	2.59	0	0	0	Repressed
497	GO_ESTABLISHMENT_OF_PROTEIN_LOCALIZATION_TO_MITOCHONDRIAL	-1.83	2.16	0	0	0	Repressed
498	BLUM_RESPONSE_TO_SALIRASIB_DN	-1.82	4.00	0	0	0	Repressed
499	CAFFAREL_RESPONSE_TO_THC_8HR_3_DN	-1.82	4.00	0	0	0	Repressed
500	GO_CYTOKINE_BINDING	-1.82	4.00	0	0	0	Repressed
501	GO_MORPHOGENESIS_OF_A_POLARIZED_EPITHELIUM	-1.82	4.00	0	0	0	Repressed
502	GO_RESPIRATORY_CHAIN_COMPLEX_IV	-1.82	4.00	0	0	0	Repressed
503	GO_RNA_5_END_PROCESSING	-1.82	4.00	0	0	0	Repressed
504	REACTOME_MRNA_CAPPING	-1.82	4.00	0	0	0	Repressed
505	VANTVEER_BREAST_CANCER_METASTASIS_DN	-1.82	4.00	0	0	0	Repressed
506	DASU_IL6_SIGNALING_SCAR_UP	-1.82	2.64	0	0	0	Repressed
507	BIOCARTA_MPR_PATHWAY	-1.82	2.62	0	0	0	Repressed
508	MAGRANGEAS_MULTIPLE_MYELOMA_IGG_VS_IGA_UP	-1.82	2.62	0	0	0	Repressed
509	VANDESLUIS_COMMD1_TARGETS_GROUP_2_UP	-1.82	2.62	0	0	0	Repressed
510	REACTOME_ACTIVATION_OF_GENE_EXPRESSION_BY_SREBF_SREBP	-1.82	2.57	0	0	0	Repressed
511	LEE_NEURAL_CREST_STEM_CELL_UP	-1.82	2.56	0	0	0	Repressed
512	REACTOME_VIRAL_MESSENGER_RNA_SYNTHESIS	-1.82	2.55	0	0	0	Repressed
513	GO_G_PROTEIN_COUPLED_CHEMOATTRACTANT_RECEPTOR_ACTIVITY	-1.82	2.13	0	0	0	Repressed
514	BIDUS_METASTASIS_UP	-1.81	4.00	0	0	0	Repressed
515	CAHOY_ASTROGLIAL	-1.81	4.00	0	0	0	Repressed
516	CHIANG_LIVER_CANCER_SUBCLASS_PROLIFERATION_UP	-1.81	4.00	0	0	0	Repressed
517	GO_NUCLEOBASE_CONTAINING_COMPOUND_TRANSPORT	-1.81	4.00	0	0	0	Repressed
518	GO_ORGANIC_ACID_METABOLIC_PROCESS	-1.81	4.00	0	0	0	Repressed
519	GO_OXIDATION_REDUCTION_PROCESS	-1.81	4.00	0	0	0	Repressed
520	KIM_WT1_TARGETS_12HR_DN	-1.81	4.00	0	0	0	Repressed
521	REACTOME_ABORTIVE_ELONGATION_OF_HIV_1_TRANSCRIPT_IN_THE_A	-1.81	4.00	0	0	0	Repressed
522	REACTOME_G_ALPHA_I_SIGNALLING_EVENTS	-1.81	4.00	0	0	0	Repressed
523	REACTOME_MHC_CLASS_II_ANTIGEN_PRESENTATION	-1.81	4.00	0	0	0	Repressed
524	FARDIN_HYPOXIA_9	-1.81	2.64	0	0	0	Repressed
525	IVANOV_MUTATED_IN_COLON_CANCER	-1.81	2.64	0	0	0	Repressed
526	PID_CD8_TCR_DOWNSTREAM_PATHWAY	-1.81	2.28	0	0	0	Repressed
527	GO_SIRNA_BINDING	-1.81	2.17	0	0	0	Repressed
528	GO_RESPONSE_TO_MUSCLE_STRETCH	-1.81	2.16	0	0	0	Repressed
529	GO_RNA_POLYMERASE_III_COMPLEX	-1.81	2.04	0	0	0	Repressed
530	DAZARD_RESPONSE_TO_UV_SCC_UP	-1.80	4.00	0	0	0	Repressed
531	GO_AEROBIC_RESPIRATION	-1.80	4.00	0	0	0	Repressed
532	GO_CHAPERONE_MEDIATED_PROTEIN_FOLDING	-1.80	4.00	0	0	0	Repressed
533	GO_MRNA_EXPORT_FROM_NUCLEUS	-1.80	4.00	0	0	0	Repressed

534	GO_MULTI_ORGANISM_MEMBRANE_FUSION	-1.80	4.00	0	0	0	Repressed
535	GO_ORGANIC_ACID_BIOSYNTHETIC_PROCESS	-1.80	4.00	0	0	0	Repressed
536	GO_REGULATION_OF_SMALL_MOLECULE_METABOLIC_PROCESS	-1.80	4.00	0	0	0	Repressed
537	GO_RNA_METABOLIC_PROCESS	-1.80	4.00	0	0	0	Repressed
538	GO_SARCOPLASM	-1.80	4.00	0	0	0	Repressed
539	REACTOME_FORMATION_OF_THE_EARLY_ELONGATION_COMPLEX	-1.80	4.00	0	0	0	Repressed
540	REACTOME_INTEGRATION_OF_ENERGY_METABOLISM	-1.80	4.00	0	0	0	Repressed
541	REACTOME_RESPONSE_TO_ELEVATED_PLATELET_CYTOSOLIC_CA2PLU	-1.80	4.00	0	0	0	Repressed
542	REACTOME_TRNA_MODIFICATION_IN_THE_NUCLEUS_AND_CYTOSOL	-1.80	4.00	0	0	0	Repressed
543	REN_ALVEOLAR_RHABDOMYOSARCOMA_DN	-1.80	4.00	0	0	0	Repressed
544	WHITEFORD_PEDIATRIC_CANCER_MARKERS	-1.80	4.00	0	0	0	Repressed
545	GO_ADENYLATE_CYCLASE_BINDING	-1.80	2.17	0	0	0	Repressed
546	DORN_ADENOVIRUS_INFECTION_12HR_DN	-1.80	2.15	0	0	0	Repressed
547	LIU_IL13_MEMORY_MODEL_UP	-1.80	2.14	0	0	0	Repressed
548	REACTOME_STING_MEDIATED_INDUCTION_OF_HOST_IMMUNE_RESPONS	-1.80	1.94	0	0	0	Repressed
549	GO_INTERACTION_WITH_SYMBIONT	-1.79	4.00	0	0	0	Repressed
550	GO_MRNA_TRANSPORT	-1.79	4.00	0	0	0	Repressed
551	GO_PEPTIDASE_ACTIVATOR_ACTIVITY	-1.79	4.00	0	0	0	Repressed
552	GO_PROTEIN_FOLDING	-1.79	4.00	0	0	0	Repressed
553	GO_RESPONSE_TO_ESTRADIOL	-1.79	4.00	0	0	0	Repressed
554	GO_RESPONSE_TO_INTERLEUKIN_12	-1.79	4.00	0	0	0	Repressed
555	REACTOME_CYCLIN_A:CDK2_ASSOCIATED_EVENTS_AT_S_PHASE_ENTR	-1.79	4.00	0	0	0	Repressed
556	REACTOME_NUCLEAR_ENVELOPE_BREAKDOWN	-1.79	4.00	0	0	0	Repressed
557	SHETH_LIVER_CANCER_VS_TXNIP_LOSS_PAM2	-1.79	4.00	0	0	0	Repressed
558	ZHANG_RESPONSE_TO_CANTHARIDIN_DN	-1.79	4.00	0	0	0	Repressed
559	GO_2_IRON_2_SULFUR_CLUSTER_BINDING	-1.79	2.62	0	0	0	Repressed
560	GO_THREONINE_TYPE_PEPTIDASE_ACTIVITY	-1.79	2.60	0	0	0	Repressed
561	REACTOME_RECYCLING_PATHWAY_OF_L1	-1.79	2.57	0	0	0	Repressed
562	SIG_CHEMOTAXIS	-1.79	2.56	0	0	0	Repressed
563	JEON_SMAD6_TARGETS_DN	-1.79	2.36	0	0	0	Repressed
564	BIOCARTA_RANMS_PATHWAY	-1.79	2.33	0	0	0	Repressed
565	GO_PROTEASOME_CORE_COMPLEX	-1.79	2.31	0	0	0	Repressed
566	GO_MATURATION_OF_5_8S_RRNA	-1.79	2.03	0	0	0	Repressed
567	GO_CADHERIN_BINDING_INVOLVED_IN_CELL_CELL_ADHESION	-1.79	1.86	0	0	0	Repressed
568	GO_POSITIVE_REGULATION_BY_HOST_OF_VIRAL_PROCESS	-1.79	1.69	0	0	0	Repressed
569	DODD_NASOPHARYNGEAL_CARCINOMA_DN	-1.78	4.00	0	0	0	Repressed
570	GO_ANIMAL_ORGAN_REGENERATION	-1.78	4.00	0	0	0	Repressed
571	GO_ISOMERASE_ACTIVITY	-1.78	4.00	0	0	0	Repressed
572	GO_MITOCHONDRION_ORGANIZATION	-1.78	4.00	0	0	0	Repressed
573	GO_SUPEROXIDE_METABOLIC_PROCESS	-1.78	4.00	0	0	0	Repressed
574	GO_VESICLE_LUMEN	-1.78	4.00	0	0	0	Repressed
575	LI_INDUCED_T_TO_NATURAL_KILLER_UP	-1.78	4.00	0	0	0	Repressed
576	ONDER_CDH1_TARGETS_1_DN	-1.78	4.00	0	0	0	Repressed
577	REACTOME_FC_EPSILON_RECEPTOR_FCERI_SIGNALING	-1.78	4.00	0	0	0	Repressed
578	REACTOME_MITOTIC_PROPHASE	-1.78	4.00	0	0	0	Repressed
579	REACTOME_MUSCLE_CONTRACTION	-1.78	4.00	0	0	0	Repressed
580	REACTOME_PROCESSING_OF_CAPPED_INTRON_CONTAINING_PRE_MRN	-1.78	4.00	0	0	0	Repressed
581	GO_ANATOMICAL_STRUCTURE_ARRANGEMENT	-1.78	2.64	0	0	0	Repressed
582	BIOCARTA_HIF_PATHWAY	-1.78	2.61	0	0	0	Repressed
583	KORKOLA_YOLK_SAC_TUMOR_UP	-1.78	2.61	0	0	0	Repressed
584	REACTOME_TRANSPORT_OF_THE_SLBP_DEPENDANT_MATURE_MRNA	-1.78	2.61	0	0	0	Repressed
585	SPIRA_SMOKERS_LUNG_CANCER_UP	-1.78	2.61	0	0	0	Repressed
586	GO_PROTEASOMAL_UBIQUITIN_INDEPENDENT_PROTEIN_CATABOLIC_PR	-1.78	2.60	0	0	0	Repressed
587	REACTOME_ABC_FAMILY_PROTEINS_MEDIATED_TRANSPORT	-1.78	2.51	0	0	0	Repressed
588	GO_CHEMOKINE_BINDING	-1.78	2.30	0	0	0	Repressed
589	GO_INNER_MITOCHONDRIAL_MEMBRANE_ORGANIZATION	-1.78	2.29	0	0	0	Repressed
590	HOFFMANN_SMALL_PRE_BII_TO_IMMATURE_B_LYMPHOCYTE_UP	-1.78	2.29	0	0	0	Repressed
591	REACTOME_APC_CDC20_MEDIATED_DEGRADATION_OF_NEK2A	-1.78	2.28	0	0	0	Repressed
592	GO_ENDOCYTIC_VESICLE_LUMEN	-1.78	2.19	0	0	0	Repressed
593	GARGALOVIC_RESPONSE_TO_OXIDIZED_PHOSPHOLIPIDS_RED_UP	-1.78	2.14	0	0	0	Repressed
594	GO_REGULATION_OF_SUPEROXIDE_METABOLIC_PROCESS	-1.78	2.12	0	0	0	Repressed
595	GO_DENDRITIC_CELL_MIGRATION	-1.78	2.05	0	0	0	Repressed
596	GO_CALCIUM_DEPENDENT_PHOSPHOLIPID_BINDING	-1.78	2.00	0	0	0	Repressed
597	GO_PROTEIN_IMPORT_INTO_MITOCHONDRIAL_MATRIX	-1.78	1.94	0	0	0	Repressed
598	GO_C_C_CHEMOKINE_BINDING	-1.78	1.78	0	0	0	Repressed
599	AMIT_EGF_RESPONSE_240_MCF10A	-1.77	4.00	0	0	0	Repressed
600	BHAT_ESR1_TARGETS_VIA_AKT1_UP	-1.77	4.00	0	0	0	Repressed
601	FERREIRA_EWINGS_SARCOMA_UNSTABLE_VS_STABLE_UP	-1.77	4.00	0	0	0	Repressed
602	GO_PROTEIN_CONTAINING_COMPLEX_DISASSEMBLY	-1.77	4.00	0	0	0	Repressed
603	GO_SARCOLEMMA	-1.77	4.00	0	0	0	Repressed
604	KEGG_ANTIGEN_PROCESSING_AND_PRESENTATION	-1.77	4.00	0	0	0	Repressed
605	REACTOME_MRNA_SPLICING	-1.77	4.00	0	0	0	Repressed
606	REACTOME_RHO_GTPASES_ACTIVATE_FORMINS	-1.77	4.00	0	0	0	Repressed
607	SASAI_TARGETS_OF_CXCR6_AND_PTCH1_UP	-1.77	2.66	0	0	0	Repressed
608	GO_SMN_SM_PROTEIN_COMPLEX	-1.77	2.65	0	0	0	Repressed
609	GRATIAS_RETINOBLASTOMA_16Q24	-1.77	2.62	0	0	0	Repressed
610	GO_CARTILAGE_DEVELOPMENT_INVOLVED_IN_ENDOCHONDRAL_BONE_	-1.77	2.60	0	0	0	Repressed
611	ZHAN_MULTIPLE_MYELOMA_CD2_DN	-1.77	2.59	0	0	0	Repressed
612	FU_INTERACT_WITH_ALKBH8	-1.77	2.33	0	0	0	Repressed
613	GO_RESPONSE_TO_CHEMOKINE	-1.77	2.33	0	0	0	Repressed
614	GO_PROTEASOME_REGULATORY_PARTICLE_LID_SUBCOMPLEX	-1.77	2.18	0	0	0	Repressed
615	REACTOME_ACTIVATION_OF_AMPK_DOWNSTREAM_OF_NMDARS	-1.77	2.15	0	0	0	Repressed
616	KEGG_VALINE_LEUCINE_AND_ISOLEUCINE_BIOSYNTHESIS	-1.77	2.14	1	0	0	Repressed
617	REACTOME_ANTIMICROBIAL_PEPTIDES	-1.77	2.14	0	0	0	Repressed
618	REACTOME_MET_ACTIVATES_PTK2_SIGNALING	-1.77	1.98	0	0	0	Repressed
619	REACTOME_GOLGI_CISTERNAE_PERICENTRIOLAR_STACK_REORGANIZA	-1.77	1.92	0	0	0	Repressed
620	REACTOME_RHO_GTPASES_ACTIVATE_IQGAPS	-1.77	1.90	0	0	0	Repressed
621	NGO_MALIGNANT_GLIOMA_1P_LOH	-1.77	1.86	0	0	0	Repressed
622	ALFANO_MYC_TARGETS	-1.76	4.00	0	0	0	Repressed
623	SESTO_RESPONSE_TO_UV_C1	-1.76	4.00	0	0	0	Repressed
624	TAKAO_RESPONSE_TO_UVB_RADIATION_DN	-1.76	4.00	0	0	0	Repressed
625	TONKS_TARGETS_OF_RUNX1_RUNX1T1_FUSION_MONOCYTE_UP	-1.76	4.00	0	0	0	Repressed
626	REACTOME_COPI_INDEPENDENT_GOLGI_TO_ER_RETROGRADE_TRAFF	-1.76	2.58	0	0	0	Repressed
627	GO_DNA_TEMPLATED_TRANSCRIPTION_TERMINATION	-1.76	2.53	0	0	0	Repressed
628	DUNNE_TARGETS_OF_AML1_MTG8_FUSION_DN	-1.76	2.32	0	0	0	Repressed
629	MOOTHA_TCA	-1.76	2.32	0	0	0	Repressed
630	CHR5P15	-1.76	2.28	0	0	0	Repressed
631	GO_MITOCHONDRIAL_TRANSMEMBRANE_TRANSPORT	-1.76	2.28	0	0	0	Repressed
632	BIOCARTA_NPC_PATHWAY	-1.76	2.16	0	0	0	Repressed
633	GO_ARP2_3_PROTEIN_COMPLEX	-1.76	2.15	0	0	0	Repressed
634	GO_INNER_EAR_RECEPTOR_CELL_STEREOCILIUM_ORGANIZATION	-1.76	2.04	0	0	0	Repressed
635	GO_POSITIVE_REGULATION_OF_CATION_CHANNEL_ACTIVITY	-1.76	2.01	0	0	0	Repressed
636	GO_OXIDOREDUCTASE_ACTIVITY_ACTING_ON_PEROXIDE_AS_ACCEPTO	-1.76	1.99	0	0	0	Repressed
637	GO_NEUTRAL_AMINO_ACID_TRANSMEMBRANE_TRANSPORTER_ACTIVITY	-1.76	1.93	0	0	0	Repressed
638	OXFORD_RALA_OR_RALB_TARGETS_DN	-1.76	1.92	0	0	0	Repressed
639	GO_RESPONSE_TO_HYPEROXIA	-1.76	1.87	0	0	0	Repressed
640	CHICAS_RB1_TARGETS_GROWING	-1.75	4.00	0	0	0	Repressed
641	FARMER_BREAST_CANCER_APOCRINE_VS_BASAL	-1.75	4.00	0	0	0	Repressed
642	GO_BOX_C_D_SNORNP_COMPLEX	-1.75	4.00	0	0	0	Repressed

643	GO_DNA_TEMPLATED_TRANSCRIPTION_INITIATION	-1.75	4.00	0	0	0	Repressed
644	GO_POSITIVE_REGULATION_OF_OXIDOREDUCTASE_ACTIVITY	-1.75	4.00	0	0	0	Repressed
645	GO_REGULATION_OF_REACTIVE_OXYGEN_SPECIES_METABOLIC_PROCI	-1.75	4.00	0	0	0	Repressed
646	LAIHO_COLORECTAL_CANCER_SERRATED_UP	-1.75	4.00	0	0	0	Repressed
647	PEART_HDAC_PROLIFERATION_CLUSTER_DN	-1.75	4.00	0	0	0	Repressed
648	PUIFFE_INVASION_INHIBITED_BY_ASCITES_UP	-1.75	4.00	0	0	0	Repressed
649	REACTOME_BINDING_AND_UPTAKE_OF_LIGANDS_BY_SCAVENGER_REC	-1.75	4.00	0	0	0	Repressed
650	REACTOME_TRANSCRIPTIONAL_REGULATION_BY_SMALL_RNAS	-1.75	4.00	0	0	0	Repressed
651	GO_PEPTIDE_RECEPTOR_ACTIVITY	-1.75	2.59	0	0	0	Repressed
652	GO_TRNA_TRANSPORT	-1.75	2.56	0	0	0	Repressed
653	REACTOME_THE_CANONICAL_RETINOID_CYCLE_IN_RODS_TWILIGHT_VIS	-1.75	2.36	0	0	0	Repressed
654	GO_NUCLEAR_NUCLEOSOME	-1.75	2.31	0	0	0	Repressed
655	REACTOME_MRNA_SPLICING_MINOR_PATHWAY	-1.75	2.27	0	0	0	Repressed
656	GO_PEPTIDYL_ARGININE_MODIFICATION	-1.75	2.16	0	0	0	Repressed
657	GO_VITAMIN_TRANSPORT	-1.75	2.13	0	0	0	Repressed
658	KEGG_PROPANOATE_METABOLISM	-1.75	2.12	0	0	0	Repressed
659	REACTOME_EXPORT_OF_VIRAL_RIBONUCLEOPROTEINS_FROM_NUCLEU	-1.75	2.10	0	0	0	Repressed
660	REACTOME_VASOPRESSIN_REGULATES_RENAL_WATER_HOMEOSTASIS	-1.75	2.00	0	0	0	Repressed
661	GO_ADENYL_NUCLEOTIDE_EXCHANGE_FACTOR_ACTIVITY	-1.75	1.97	0	0	0	Repressed
662	ITO_PTTG1_TARGETS_DN	-1.75	1.96	0	0	0	Repressed
663	GO_REGULATION_OF_PROTON_TRANSPORT	-1.75	1.85	0	0	0	Repressed
664	LEE_INTRATHYMIC_T_PROGENITOR	-1.75	1.81	0	0	0	Repressed
665	SMID_BREAST_CANCER_LUMINAL_A_DN	-1.75	1.77	0	0	0	Repressed
666	DANG_MYC_TARGETS_UP	-1.74	4.00	0	0	0	Repressed
667	GO_S_ADENOSYLMETHIONINE_DEPENDENT_METHYLTRANSFERASE_ACT	-1.74	4.00	0	0	0	Repressed
668	GO_SEQUESTERING_OF_CALCIUM_ION	-1.74	4.00	0	0	0	Repressed
669	MULLIGAN_NTF3_SIGNALING_VIA_INSR_AND_IGF1R_UP	-1.74	4.00	0	0	0	Repressed
670	PAL_PRMT5_TARGETS_UP	-1.74	4.00	0	0	0	Repressed
671	REACTOME_CELLULAR_RESPONSE_TO_HEAT_STRESS	-1.74	4.00	0	0	0	Repressed
672	REACTOME_TP53_REGULATES_METABOLIC_GENES	-1.74	4.00	0	0	0	Repressed
673	WANG_TARGETS_OF_MLL_CBP_FUSION_DN	-1.74	4.00	0	0	0	Repressed
674	GO_METHYLOSOME	-1.74	2.62	0	0	0	Repressed
675	CERVERA_SDHB_TARGETS_2	-1.74	2.55	0	0	0	Repressed
676	GO_TRANSCRIPTION_INITIATION_FROM_RNA_POLYMERASE_II_PROMOTE	-1.74	2.48	0	0	0	Repressed
677	GO_CELL_PROLIFERATION_INVOLVED_IN_METANEPHROS_DEVELOPMEN	-1.74	2.37	0	0	0	Repressed
678	GAJATE_RESPONSE_TO_TRABECTEDIN_DN	-1.74	2.33	0	0	0	Repressed
679	GO_MEMBRANE_RAFT_ASSEMBLY	-1.74	2.19	0	0	0	Repressed
680	WANG_NFKB_TARGETS	-1.74	2.18	0	0	0	Repressed
681	REACTOME_GLUCAGON_LIKE_PEPTIDE_1_GLP1_REGULATES_INSULIN_S	-1.74	2.13	0	0	0	Repressed
682	GO_WATER_SOLUBLE_VITAMIN_BIOSYNTHETIC_PROCESS	-1.74	2.05	0	0	0	Repressed
683	GO_CELLULAR_RESPONSE_TO_DEXAMETHASONE_STIMULUS	-1.74	2.02	0	0	0	Repressed
684	GO_LOW_DENSITY_LIPOPROTEIN_PARTICLE_CLEARANCE	-1.74	1.93	0	0	0	Repressed
685	GROSS_HIF1A_TARGETS_DN	-1.74	1.93	0	0	0	Repressed
686	BOQUEST_STEM_CELL_CULTURED_VS_FRESH_UP	-1.73	4.00	0	0	0	Repressed
687	GO_REGULATION_OF_HEMATOPOIETIC_PROGENITOR_CELL_DIFFERENT	-1.73	4.00	0	0	0	Repressed
688	HALLMARK_FATTY_ACID_METABOLISM	-1.73	4.00	0	0	0	Repressed
689	JUBAN_TARGETS_OF_SPI1_AND_FLI1_DN	-1.73	4.00	0	0	0	Repressed
690	NIELSEN_GIST_AND_SYNOVIAL_SARCOMA_DN	-1.73	4.00	0	0	0	Repressed
691	REACTOME_DNA_REPLICATION	-1.73	4.00	0	0	0	Repressed
692	REACTOME_INTERLEUKIN_1_FAMILY_SIGNALING	-1.73	4.00	0	0	0	Repressed
693	REACTOME_PLATELET_ACTIVATION_SIGNALING_AND_AGGREGATION	-1.73	4.00	0	0	0	Repressed
694	VANTVEER_BREAST_CANCER_ESR1_DN	-1.73	4.00	0	0	0	Repressed
695	WELCSH_BRCA1_TARGETS_DN	-1.73	4.00	0	0	0	Repressed
696	ZHU_CMV_ALL_UP	-1.73	4.00	0	0	0	Repressed
697	GO_POSITIVE_REGULATION_OF_RYANODINE_SENSITIVE_CALCIUM_RELE	-1.73	2.66	0	0	0	Repressed
698	GO_AMINO_ACID_BINDING	-1.73	2.56	0	0	0	Repressed
699	SASAKI_ADULT_T_CELL_LEUKEMIA	-1.73	2.46	0	0	0	Repressed
700	KUROZUMI_RESPONSE_TO_ONCOCYTIC_VIRUS	-1.73	2.34	0	0	0	Repressed
701	GO_ACTIVATION_OF_ADENYLATE_CYCLASE_ACTIVITY	-1.73	2.32	0	0	0	Repressed
702	GO_REGULATION_OF_SECONDARY_METABOLIC_PROCESS	-1.73	2.32	0	0	0	Repressed
703	BASSO_HAIRY_CELL_LEUKEMIA_UP	-1.73	2.28	0	0	0	Repressed
704	REACTOME_OPIOID_SIGNALLING	-1.73	2.27	0	0	0	Repressed
705	GO_SNRNA_TRANSCRIPTION	-1.73	2.24	0	0	0	Repressed
706	XU_HGF_SIGNALING_NOT_VIA_AKT1_48HR_DN	-1.73	2.15	0	0	0	Repressed
707	GO_CELLULAR_RESPONSE_TO_ESTRADIOL_STIMULUS	-1.73	2.01	0	0	0	Repressed
708	HORTON_SREBF_TARGETS	-1.73	2.01	0	0	0	Repressed
709	KORKOLA_EMBRYONIC_CARCINOMA_VS_SEMINOMA_DN	-1.73	1.97	0	0	0	Repressed
710	LINDGREN_BLADDER_CANCER_HIGH_RECURRENCE	-1.73	1.96	0	0	0	Repressed
711	REACTOME_ATF6_ATF6_ALPHA_ACTIVATES_CHAPERONES	-1.73	1.88	0	0	0	Repressed
712	REACTOME_SIGNALING_BY_FGFR2_IIIA_TM	-1.73	1.85	0	0	0	Repressed
713	GO_NEGATIVE_REGULATION_OF_LYASE_ACTIVITY	-1.73	1.81	0	0	0	Repressed
714	GO_POSITIVE_REGULATION_OF_VIRAL_PROCESS	-1.72	4.00	0	0	0	Repressed
715	GO_REGULATION_OF_LIPID_BIOSYNTHETIC_PROCESS	-1.72	4.00	0	0	0	Repressed
716	JOHANSSON_GLIOMAGENESIS_BY_PDGFB_UP	-1.72	4.00	0	0	0	Repressed
717	SCHLOSSER_MYC_TARGETS_AND_SERUM_RESPONSE_UP	-1.72	2.60	0	1	0	Repressed
718	GO_ENDOPLASMIC_RETICULUM_CHAPERONE_COMPLEX	-1.72	2.34	0	0	0	Repressed
719	GO_AMIDE_TRANSMEMBRANE_TRANSPORTER_ACTIVITY	-1.72	2.30	0	0	0	Repressed
720	SARTIPY_NORMAL_AT_INSULIN_RESISTANCE_UP	-1.72	2.30	0	0	0	Repressed
721	GO_MICROBODY_LUMEN	-1.72	2.29	0	0	0	Repressed
722	JIANG_AGING_CEREBRAL_CORTEX_DN	-1.72	2.29	0	0	0	Repressed
723	GO_PODOSOME	-1.72	2.28	0	0	0	Repressed
724	GO_NCRNA_3_END_PROCESSING	-1.72	2.13	0	0	0	Repressed
725	KEGG_FATTY_ACID_METABOLISM	-1.72	2.13	0	0	0	Repressed
726	GESERICK_TERT_TARGETS_DN	-1.72	2.04	0	0	0	Repressed
727	GO_REGULATION_OF_LOW_DENSITY_LIPOPROTEIN_PARTICLE_CLEARAI	-1.72	2.04	0	0	0	Repressed
728	GO_RIBONUCLEASE_P_COMPLEX	-1.72	2.03	0	0	0	Repressed
729	REACTOME_RNA_POLYMERASE_II_TRANSCRIPTION_ELONGATION	-1.72	1.88	0	0	0	Repressed
730	GO_DEFENSE_RESPONSE_TO_FUNGUS	-1.72	1.85	0	0	0	Repressed
731	GO_COENZYME_METABOLIC_PROCESS	-1.71	4.00	0	0	0	Repressed
732	GO_GLYCOLYTIC_PROCESS	-1.71	4.00	0	0	0	Repressed
733	GO_POSITIVE_REGULATION_OF_TRANSPORTER_ACTIVITY	-1.71	4.00	0	0	0	Repressed
734	GO_REGULATION_OF_MRNA_CATABOLIC_PROCESS	-1.71	4.00	0	0	0	Repressed
735	GO_REGULATION_OF_STEM_CELL_DIFFERENTIATION	-1.71	4.00	0	0	0	Repressed
736	MOREAUX_MULTIPLE_MYELOMA_BY_TACI_DN	-1.71	4.00	0	0	0	Repressed
737	PID_ILK_PATHWAY	-1.71	4.00	0	0	0	Repressed
738	REACTOME_VISUAL_PHOTOTRANSDUCTION	-1.71	4.00	0	0	0	Repressed
739	SANA_RESPONSE_TO_IFNG_DN	-1.71	4.00	0	0	0	Repressed
740	STEIN_ESRRA_TARGETS_UP	-1.71	4.00	0	0	0	Repressed
741	WIELAND_UP_BY_HBV_INFECTION	-1.71	4.00	0	0	0	Repressed
742	GO_STRUCTURAL_CONSTITUENT_OF_CYTOSKELETON	-1.71	2.59	0	0	0	Repressed
743	ZHONG_SECRETOME_OF_LUNG_CANCER_AND_ENDOTHELIUM	-1.71	2.59	0	0	0	Repressed
744	LIANG_HEMATOPOIESIS_STEM_CELL_NUMBER_LARGE_VS_TINY_UP	-1.71	2.58	0	0	0	Repressed
745	GRAHAM_CML_QUIESCENT_VS_NORMAL_QUIESCENT_UP	-1.71	2.57	0	0	0	Repressed
746	GO_RNA_METHYLATION	-1.71	2.55	0	0	0	Repressed
747	REACTOME_RNA_POLYMERASE_II_TRANSCRIBES_SNRNA_GENES	-1.71	2.53	0	0	0	Repressed
748	GO_DOUBLE_STRANDED_RNA_BINDING	-1.71	2.52	0	0	0	Repressed
749	GRAHAM_NORMAL_QUIESCENT_VS_NORMAL_DIVIDING_DN	-1.71	2.51	0	0	0	Repressed
750	HOLLEMAN_PREDNISOLONE_RESISTANCE_ALL_DN	-1.71	2.36	0	0	0	Repressed
751	ZHAN_MULTIPLE_MYELOMA_PR_UP	-1.71	2.28	0	0	0	Repressed

752	REACTOME_APC_C:CDC20_MEDIATED_DEGRADATION_OF_CYCLIN_B	-1.71	2.27	0	0	0	Repressed
753	GO_NOSE_DEVELOPMENT	-1.71	2.22	0	0	0	Repressed
754	GO_INFLAMMATORY_CELL_APOPTOTIC_PROCESS	-1.71	2.18	0	0	0	Repressed
755	GO_PROTEASOME_ACTIVATING_ATPASE_ACTIVITY	-1.71	2.17	0	0	0	Repressed
756	GO_PARASYMPATHETIC_NERVOUS_SYSTEM_DEVELOPMENT	-1.71	2.16	0	0	0	Repressed
757	REACTOME_RNA_POLYMERASE_III_TRANSCRIPTION_INITIATION_FROM_T	-1.71	2.12	0	0	0	Repressed
758	GO_INTERFERON_BETA_BIOSYNTHETIC_PROCESS	-1.71	2.06	0	0	0	Repressed
759	PEDERSEN_METASTASIS_BY_ERBB2_ISOFORM_6	-1.71	2.03	0	0	0	Repressed
760	GO_VITAMIN_BIOSYNTHETIC_PROCESS	-1.71	2.02	0	0	0	Repressed
761	GO_DIGESTION	-1.71	1.98	0	0	0	Repressed
762	GO_BOX_H_ACA_RNP_COMPLEX	-1.71	1.97	0	0	0	Repressed
763	REACTOME_FORMATION_OF_HIV_ELONGATION_COMPLEX_IN_THE_ABSE	-1.71	1.97	0	0	0	Repressed
764	KEGG_BETA_ALANINE_METABOLISM	-1.71	1.94	0	0	0	Repressed
765	RODRIGUES_NTN1_TARGETS_UP	-1.71	1.86	0	0	0	Repressed
766	GO_RIBOSOMAL_LARGE_SUBUNIT_EXPORT_FROM_NUCLEUS	-1.71	1.84	0	0	0	Repressed
767	LOPEZ_MESOTHELIOMA_SURVIVAL_OVERALL_DN	-1.71	1.82	0	0	0	Repressed
768	GO_TRNA_5_END_PROCESSING	-1.71	1.80	0	0	0	Repressed
769	REACTOME_RECEPTOR_TYPE_TYROSINE_PROTEIN_PHOSPHATASES	-1.71	1.80	0	0	0	Repressed
770	GO_SEH1_ASSOCIATED_COMPLEX	-1.71	1.57	0	0	0	Repressed
771	BLALOCK_ALZHEIMERS_DISEASE_INCIPIENT_DN	-1.70	4.00	0	0	0	Repressed
772	DELYS_THYROID_CANCER_UP	-1.70	4.00	0	0	0	Repressed
773	GO_TELOMERE_MAINTENANCE_VIA_TELOMERE_LENGTHENING	-1.70	4.00	0	0	0	Repressed
774	IVANOVA_HEMATOPOIESIS_INTERMEDIATE_PROGENITOR	-1.70	4.00	0	0	0	Repressed
775	LEI_MYB_TARGETS	-1.70	4.00	0	0	0	Repressed
776	PID_PLK1_PATHWAY	-1.70	4.00	0	0	0	Repressed
777	SMITH_TERT_TARGETS_UP	-1.70	4.00	0	0	0	Repressed
778	REACTOME_NUCLEAR_IMPORT_OF_REV_PROTEIN	-1.70	2.57	0	0	0	Repressed
779	HARRIS_HYPOXIA	-1.70	2.55	0	0	0	Repressed
780	LENAOUR_DENDRITIC_CELL_MATURATION_DN	-1.70	2.52	0	0	0	Repressed
781	EGUCHI_CELL_CYCLE_RB1_TARGETS	-1.70	2.31	0	0	0	Repressed
782	GO_FEMALE_GENITALIA_DEVELOPMENT	-1.70	2.19	0	0	0	Repressed
783	SMID_BREAST_CANCER_RELAPSE_IN_PLEURA_DN	-1.70	2.19	0	0	0	Repressed
784	GO_ACTIN_FILAMENT_BUNDLE	-1.70	2.15	0	0	0	Repressed
785	REACTOME_CRISTAE_FORMATION	-1.70	2.05	0	0	0	Repressed
786	REACTOME_CA_DEPENDENT_EVENTS	-1.70	2.00	0	0	0	Repressed
787	GO_NEGATIVE_REGULATION_OF_GLIAL_CELL_PROLIFERATION	-1.70	1.97	0	0	0	Repressed
788	GO_MITOTIC_NUCLEAR_ENVELOPE_DISASSEMBLY	-1.70	1.96	0	0	0	Repressed
789	REACTOME_FOLDING_OF_ACTIN_BY_CCT_TRIC	-1.70	1.96	0	0	0	Repressed
790	GO_NITRIC_OXIDE_SYNTHASE_BINDING	-1.70	1.95	0	0	0	Repressed
791	GO_BONE_GROWTH	-1.70	1.93	0	0	0	Repressed
792	REACTOME_GAP_JUNCTION_TRAFFICKING_AND_REGULATION	-1.70	1.93	0	0	0	Repressed
793	BIOCARTA_S1P_PATHWAY	-1.70	1.90	0	0	0	Repressed
794	GO_MITOCHONDRIAL_ELECTRON_TRANSPORT_UBIQUINOL_TO_CYTOCH	-1.70	1.87	0	0	0	Repressed
795	GO_TRNA_CYTOSINE_METHYLTRANSFERASE_ACTIVITY	-1.70	1.83	1	0	0	Repressed
796	GO_LONG_CHAIN_FATTY_ACID_COA_LIGASE_ACTIVITY	-1.70	1.74	0	0	0	Repressed
797	GO_MITOCHONDRIAL_CALCIUM_ION_HOMEOSTASIS	-1.70	1.69	0	0	0	Repressed
798	BILD_MYC_ONCOGENIC_SIGNATURE	-1.69	4.00	0	1	0	Repressed
799	GO_POSITIVE_REGULATION_OF_ION_TRANSPORT	-1.69	4.00	0	0	0	Repressed
800	GO_SMALL_MOLECULE_BIOSYNTHETIC_PROCESS	-1.69	4.00	0	0	0	Repressed
801	REACTOME_SIGNALING_BY_WNT	-1.69	4.00	0	0	0	Repressed
802	REACTOME_TRANSPORT_OF_MATURE_MRNAS_DERIVED_FROM_INTROI	-1.69	4.00	0	0	0	Repressed
803	REACTOME_TRANSPORT_OF_MATURE_TRANSCRIPT_TO_CYTOPLASM	-1.69	4.00	0	0	0	Repressed
804	ELVIDGE_HIF1A_AND_HIF2A_TARGETS_DN	-1.69	2.55	0	0	0	Repressed
805	REACTOME_CYTOSOLIC_SENSORS_OF_PATHOGEN_ASSOCIATED_DNA	-1.69	2.54	0	0	0	Repressed
806	DANG_REGULATED_BY_MYC_UP	-1.69	2.53	0	0	0	Repressed
807	GO_DIAPHRAGM_DEVELOPMENT	-1.69	2.37	0	0	0	Repressed
808	GO_CHEMOATTRACTANT_ACTIVITY	-1.69	2.34	0	0	0	Repressed
809	GO_NCRNA_EXPORT_FROM_NUCLEUS	-1.69	2.28	0	0	0	Repressed
810	GO_OXIDOREDUCTASE_ACTIVITY_OXIDIZING_METAL_IONS	-1.69	2.20	0	0	0	Repressed
811	GO_REGENERATION	-1.69	2.20	0	0	0	Repressed
812	REACTOME_RRNA_PROCESSING_IN_THE_MITOCHONDRION	-1.69	2.15	0	0	0	Repressed
813	GRADE_METASTASIS_DN	-1.69	2.10	0	0	0	Repressed
814	GO_LOCOMOTOR_RHYTHM	-1.69	2.05	0	0	0	Repressed
815	GO_NEUROTRANSMITTER_BINDING	-1.69	2.04	0	0	0	Repressed
816	REACTOME_TRNA_MODIFICATION_IN_THE_MITOCHONDRION	-1.69	2.03	0	0	0	Repressed
817	REACTOME_CONVERSION_FROM_APC_C:CDC20_TO_APC_C:CDH1_IN_L/	-1.69	1.93	0	0	0	Repressed
818	GO_MACROLIDE_BINDING	-1.69	1.87	0	0	0	Repressed
819	GO_REGULATION_OF_CARDIAC_MUSCLE_CONTRACTION_BY_REGULATI	-1.69	1.86	0	0	0	Repressed
820	DORMOY_ELAVL1_TARGETS	-1.69	1.85	0	0	0	Repressed
821	GO_POSITIVE_REGULATION_OF_POTASSIUM_ION_TRANSMEMBRANE_TR	-1.69	1.80	0	0	0	Repressed
822	OXFORD_RALA_TARGETS_UP	-1.69	1.80	0	0	0	Repressed
823	PID_HNF3B_PATHWAY	-1.69	1.74	0	0	0	Repressed
824	GO_INTEGRATOR_COMPLEX	-1.69	1.70	0	0	0	Repressed
825	GO_MITOTIC_SPINDLE_ORGANIZATION	-1.68	4.00	0	0	0	Repressed
826	GO_REGULATION_OF_MORPHOGENESIS_OF_AN_EPITHELIUM	-1.68	4.00	0	0	0	Repressed
827	HALLMARK_PI3K_AKT_MTOR_SIGNALING	-1.68	4.00	0	0	0	Repressed
828	LASTOWSKA_NEUROBLASTOMA_COPY_NUMBER_UP	-1.68	4.00	0	0	0	Repressed
829	MARTORIATI_MDM4_TARGETS_NEUROEPITHELIUM_UP	-1.68	4.00	0	0	0	Repressed
830	ROSS_LEUKEMIA_WITH_MLL_FUSIONS	-1.68	4.00	0	0	0	Repressed
831	GO_REGULATION_OF_SYNAPSE_MATURATION	-1.68	2.65	0	0	0	Repressed
832	REACTOME_RNA_POLYMERASE_III_TRANSCRIPTION	-1.68	2.56	0	0	0	Repressed
833	RAMASWAMY_METASTASIS_UP	-1.68	2.55	0	0	0	Repressed
834	GO_BRUSH_BORDER	-1.68	2.53	0	0	0	Repressed
835	ZHU_CMV_24_HR_UP	-1.68	2.52	0	0	0	Repressed
836	GO_POSITIVE_REGULATION_OF_ASPARTIC_TYPE_PEPTIDASE_ACTIVITY	-1.68	2.35	0	0	0	Repressed
837	FARMER_BREAST_CANCER_CLUSTER_1	-1.68	2.26	0	0	0	Repressed
838	WEST_ADRENOCORTICAL_TUMOR_MARKERS_UP	-1.68	2.15	0	0	0	Repressed
839	REACTOME_PYRUVATE_METABOLISM_AND_CITRIC_ACID_TCA_CYCLE	-1.68	2.10	0	0	0	Repressed
840	REACTOME_ERKS_ARE_INACTIVATED	-1.68	2.05	0	0	0	Repressed
841	GO_FRUCTOSE_METABOLIC_PROCESS	-1.68	2.02	0	0	0	Repressed
842	GO_GROWTH_INVOLVED_IN_SYMBIOTIC_INTERACTION	-1.68	1.96	0	0	0	Repressed
843	GO_AMINOACYL_TRNA_SYNTHETASE_MULTIENZYME_COMPLEX	-1.68	1.94	0	0	0	Repressed
844	BIOCARTA_NDKDYNAMIN_PATHWAY	-1.68	1.93	0	0	0	Repressed
845	KANG_GLIS3_TARGETS	-1.68	1.85	0	0	0	Repressed
846	LANDIS_ERBB2_BREAST_TUMORS_65_UP	-1.68	1.85	0	0	0	Repressed
847	GO_CONDENSED_CHROMOSOME_OUTER_KINETOCHORE	-1.68	1.82	0	0	0	Repressed
848	GO_PROTEIN_ARGININE_N_METHYLTRANSFERASE_ACTIVITY	-1.68	1.80	0	0	0	Repressed
849	GO_TRNA_5_LEADER_REMOVAL	-1.68	1.76	0	0	0	Repressed
850	GO_REGULATION_OF_PROTEIN_FOLDING	-1.68	1.74	0	0	0	Repressed
851	GO_RESPONSE_TO_REDOX_STATE	-1.68	1.73	0	0	0	Repressed
852	BOYAULT_LIVER_CANCER_SUBCLASS_G2	-1.68	1.67	0	0	0	Repressed
853	LOPEZ_MESOTELIOMA_SURVIVAL_TIME_UP	-1.68	1.63	0	0	0	Repressed
854	CONCANNON_APOPTOSIS_BY_EPOXOMICIN_UP	-1.67	4.00	0	0	0	Repressed
855	GO_AZUROPHIL_GRANULE_LUMEN	-1.67	4.00	0	0	0	Repressed
856	GO_CELLULAR_COMPONENT_DISASSEMBLY	-1.67	4.00	0	0	0	Repressed
857	GO_COFACTOR_METABOLIC_PROCESS	-1.67	4.00	0	0	0	Repressed
858	GO_NUCLEAR_TRANSPORT	-1.67	4.00	0	0	0	Repressed
859	GO_NUCLEOLAR_PART	-1.67	4.00	0	0	0	Repressed
860	GO_RIBONUCLEOPROTEIN_COMPLEX_BINDING	-1.67	4.00	0	0	0	Repressed

861	LINDGREN_BLADDER_CANCER_CLUSTER_3_UP	-1.67	4.00	0	0	0	Repressed
862	WATANABE_RECTAL_CANCER_RADIOTHERAPY_RESPONSIVE_DN	-1.67	4.00	0	0	0	Repressed
863	ZHANG_TLX_TARGETS_36HR_DN	-1.67	4.00	0	0	0	Repressed
864	FERRANDO_T_ALL_WITH_MLL_ENL_FUSION_DN	-1.67	2.54	0	0	0	Repressed
865	MORI_SMALL_PRE_BII_LYMPHOCYTE_DN	-1.67	2.53	0	0	0	Repressed
866	GO_MONOCARBOXYLIC_ACID_BIOSYNTHETIC_PROCESS	-1.67	2.51	0	0	0	Repressed
867	GO_EUKARYOTIC_TRANSLATION_INITIATION_FACTOR_2B_COMPLEX	-1.67	2.35	0	0	0	Repressed
868	CROMER_METASTASIS_DN	-1.67	2.30	0	0	0	Repressed
869	ALK_DN.V1_UP	-1.67	2.28	0	0	0	Repressed
870	ONO_AML1_TARGETS_DN	-1.67	2.13	0	0	0	Repressed
871	REACTOME_MITOCHONDRIAL_TRNA_AMINOACYLATION	-1.67	2.04	0	0	0	Repressed
872	BURTON_ADIPOGENESIS_4	-1.67	1.99	0	0	0	Repressed
873	GO_MODULATION_OF_GROWTH_OF_SYMBIONT_INVOLVED_IN_INTERAC	-1.67	1.94	0	0	0	Repressed
874	GO_ANTIBIOTIC_CATABOLIC_PROCESS	-1.67	1.91	0	0	0	Repressed
875	GO_FATTY_ACID_DERIVATIVE_BIOSYNTHETIC_PROCESS	-1.67	1.91	0	0	0	Repressed
876	GO_NEGATIVE_REGULATION_OF_ADENYLATE_CYCLASE_ACTIVITY	-1.67	1.87	0	0	0	Repressed
877	GO_MEMBRANE_DISASSEMBLY	-1.67	1.85	0	0	0	Repressed
878	BIOCARTA_SM_PATHWAY	-1.67	1.82	0	0	0	Repressed
879	GO_TFIID_CLASS_TRANSCRIPTION_FACTOR_COMPLEX_BINDING	-1.67	1.82	0	0	0	Repressed
880	BIOCARTA_GCR_PATHWAY	-1.67	1.80	0	0	0	Repressed
881	LOPEZ_MBD_TARGETS_IMPRINTED_AND_X_LINKED	-1.67	1.77	0	0	0	Repressed
882	REACTOME_GLUCAGON_SIGNALING_IN_METABOLIC_REGULATION	-1.67	1.75	0	0	0	Repressed
883	REACTOME_CITRIC_ACID_CYCLE_TCA_CYCLE	-1.67	1.72	0	0	0	Repressed
884	GO_ENDOLYSOSOME_MEMBRANE	-1.67	1.69	0	0	0	Repressed
885	GO_GATOR2_COMPLEX	-1.67	1.69	0	0	0	Repressed
886	GO_PROTEIN_COFACTOR_LINKAGE	-1.67	1.68	0	0	0	Repressed
887	GO_REGULATION_OF_APOPTOTIC_DNA_FRAGMENTATION	-1.67	1.66	0	0	0	Repressed
888	YAO_TEMPORAL_RESPONSE_TO_PROGESTERONE_CLUSTER_5	-1.67	1.63	0	0	0	Repressed
889	GO_CELLULAR_RESPONSE_TO_OXYGEN_LEVELS	-1.66	4.00	0	0	0	Repressed
890	GO_NUCLEOTIDYLTRANSFERASE_ACTIVITY	-1.66	4.00	0	0	0	Repressed
891	GO_RESPONSE_TO_ORGANIC_CYCLIC_COMPOUND	-1.66	4.00	0	0	0	Repressed
892	GO_NUCLEOSIDE_BISPHOSPHATE_BIOSYNTHETIC_PROCESS	-1.66	2.56	0	0	0	Repressed
893	GO_NUCLEAR_PORE	-1.66	2.55	0	0	0	Repressed
894	GOTZMANN_EPITHELIAL_TO_MESENCHYMAL_TRANSITION_DN	-1.66	2.48	0	0	0	Repressed
895	HALLMARK_ADIPOGENESIS	-1.66	2.47	0	0	0	Repressed
896	WANG_CISPLATIN_RESPONSE_AND_XPC_UP	-1.66	2.46	0	0	0	Repressed
897	GO_TELOMERASE_RNA_BINDING	-1.66	2.31	0	0	0	Repressed
898	KEGG_RNA_POLYMERASE	-1.66	2.31	0	0	0	Repressed
899	GO_STRIATED_MUSCLE_CONTRACTION	-1.66	2.26	0	0	0	Repressed
900	REACTOME_ANTIVIRAL_MECHANISM_BY_IFN_STIMULATED_GENES	-1.66	2.25	0	0	0	Repressed
901	GO_CHROMOSOME_PASSENGER_COMPLEX	-1.66	2.06	0	0	0	Repressed
902	GO_BOX_H_ACA_SNORNA_BINDING	-1.66	2.04	0	0	0	Repressed
903	GO_ATP_DEPENDENT_5_3_DNA_HELICASE_ACTIVITY	-1.66	1.97	0	0	0	Repressed
904	GO_REGULATION_OF_CALCIUM_TRANSPORTING_ATPASE_ACTIVITY	-1.66	1.95	0	0	0	Repressed
905	GO_GLOMERULUS_DEVELOPMENT	-1.66	1.92	0	0	0	Repressed
906	PID_HDAC_CLASSIII_PATHWAY	-1.66	1.92	0	0	0	Repressed
907	REACTOME_DEFECTIVE_TPR_MAY_CONFER_SUSCEPTIBILITY_TOWARD	-1.66	1.90	0	0	0	Repressed
908	CAIRO_PML_TARGETS_BOUND_BY_MYC_UP	-1.66	1.86	0	0	0	Repressed
909	AMIT_SERUM_RESPONSE_240_MCF10A	-1.66	1.85	0	0	0	Repressed
910	GO_CHONDROITIN_SULFATE_BINDING	-1.66	1.84	0	0	0	Repressed
911	GO_RESPONSE_TO_DEXAMETHASONE	-1.66	1.80	0	0	0	Repressed
912	REACTOME_REGULATION_OF_GLYCOLYSIS_BY_FRUCTOSE_2_6_BISPH	-1.66	1.79	0	0	0	Repressed
913	BIOCARTA_G2_PATHWAY	-1.66	1.75	0	0	0	Repressed
914	BIOCARTA_EGFR_SMRTE_PATHWAY	-1.66	1.74	0	0	0	Repressed
915	GO_SERINE_FAMILY_AMINO_ACID_METABOLIC_PROCESS	-1.66	1.72	0	0	0	Repressed
916	KEGG_PRION_DISEASES	-1.66	1.72	0	0	0	Repressed
917	NIKOLSKY_OVERCONNECTED_IN_BREAST_CANCER	-1.66	1.72	0	0	0	Repressed
918	GO_POTASSIUM_ION_BINDING	-1.66	1.68	0	0	0	Repressed
919	GO_REGULATION_OF_LIPOPROTEIN_PARTICLE_CLEARANCE	-1.66	1.58	0	0	0	Repressed
920	GO_PEPTIDYL_ARGININE_N_METHYLATION	-1.66	1.55	0	0	0	Repressed
921	AFFAR_YY1_TARGETS_DN	-1.65	4.00	0	0	0	Repressed
922	GO_CADHERIN_BINDING	-1.65	4.00	0	0	0	Repressed
923	GO_CATALYTIC_STEP_2_SPLICEOSOME	-1.65	4.00	0	0	0	Repressed
924	GO_REGULATION_OF_CATION_TRANSMEMBRANE_TRANSPORT	-1.65	4.00	0	0	0	Repressed
925	GO_REGULATION_OF_POSTTRANSCRIPTIONAL_GENE_SILENCING	-1.65	4.00	0	0	0	Repressed
926	GO_REGULATION_OF_STEROID_BIOSYNTHETIC_PROCESS	-1.65	4.00	0	0	0	Repressed
927	GO_RESPONSE_TO_HEAT	-1.65	4.00	0	0	0	Repressed
928	GO_RESPONSE_TO_TEMPERATURE_STIMULUS	-1.65	4.00	0	0	0	Repressed
929	GO_SMALL_MOLECULE_METABOLIC_PROCESS	-1.65	4.00	0	0	0	Repressed
930	HELLER_HDAC_TARGETS_DN	-1.65	4.00	0	0	0	Repressed
931	LI_WILMS_TUMOR_VS_FETAL_KIDNEY_1_UP	-1.65	4.00	0	0	0	Repressed
932	REACTOME_UB_SPECIFIC_PROCESSING_PROTEASES	-1.65	4.00	0	0	0	Repressed
933	GO_PROTEIN_LOCALIZATION_TO_CELL_SURFACE	-1.65	2.58	0	0	0	Repressed
934	REACTOME_RNA_POLYMERASE_III_TRANSCRIPTION_INITIATION_FROM_T	-1.65	2.58	0	0	0	Repressed
935	MARTINEZ_RESPONSE_TO_TRABECTEDIN_UP	-1.65	2.55	0	0	0	Repressed
936	MARZEC_IL2_SIGNALING_UP	-1.65	2.54	0	0	0	Repressed
937	TARTE_PLASMA_CELL_VS_B_LYMPHOCYTE_UP	-1.65	2.54	0	0	0	Repressed
938	GO_ORGANIC_ACID_BINDING	-1.65	2.53	0	0	0	Repressed
939	ANDERSEN_CHOLANGIOCARCINOMA_CLASS2	-1.65	2.26	0	0	0	Repressed
940	GO_NEUTROPHIL_APOPTOTIC_PROCESS	-1.65	2.18	0	0	0	Repressed
941	GO_REGULATION_OF_CHONDROCYTE_DIFFERENTIATION	-1.65	2.18	0	0	0	Repressed
942	GO_MOLECULAR_CARRIER_ACTIVITY	-1.65	2.13	0	0	0	Repressed
943	SERVITJA_ISLET_HNF1A_TARGETS_DN	-1.65	2.12	0	0	0	Repressed
944	REACTOME_INTRAFLAGELLAR_TRANSPORT	-1.65	2.10	0	0	0	Repressed
945	DACOSTA_LOW_DOSE_UV_RESPONSE_VIA_ERCC3_XPCS_DN	-1.65	2.05	0	0	0	Repressed
946	GO_MATURATION_OF_SSU_RRNA_FROM_TRICISTRONIC_RRNA_TRANSC	-1.65	1.99	0	0	0	Repressed
947	GO_RRNA_TRANSCRIPTION	-1.65	1.98	0	0	0	Repressed
948	KEGG_BASAL_TRANSCRIPTION_FACTORS	-1.65	1.97	0	0	0	Repressed
949	GO_RRNA_PSEUDOURIDINE_SYNTHESIS	-1.65	1.95	0	0	0	Repressed
950	REACTOME_G_PROTEIN_ACTIVATION	-1.65	1.93	0	0	0	Repressed
951	BIOCARTA_NKT_PATHWAY	-1.65	1.82	0	0	0	Repressed
952	REACTOME_SCAVENGING_BY_CLASS_F_RECEPTORS	-1.65	1.82	0	0	0	Repressed
953	GO_NADH_METABOLIC_PROCESS	-1.65	1.81	0	0	0	Repressed
954	GO_NITRIC_OXIDE_SYNTHASE_REGULATOR_ACTIVITY	-1.65	1.76	0	0	0	Repressed
955	GO_C_X_C_CHEMOKINE_RECEPTOR_ACTIVITY	-1.65	1.73	0	0	0	Repressed
956	CHR6P24	-1.65	1.72	0	0	0	Repressed
957	GO_ATF6_MEDIATED_UNFOLDED_PROTEIN_RESPONSE	-1.65	1.69	0	0	0	Repressed
958	GO_COLLAGEN_FIBRIL_ORGANIZATION	-1.65	1.69	0	0	0	Repressed
959	GO_OLFACTORY_LOBE_DEVELOPMENT	-1.65	1.69	0	0	0	Repressed
960	KEGG_PHENYLALANINE_METABOLISM	-1.65	1.69	0	0	0	Repressed
961	REACTOME_RNA_POLYMERASE_III_CHAIN_ELONGATION	-1.65	1.59	0	0	0	Repressed
962	GO_INTRACELLULAR_STEROL_TRANSPORT	-1.65	1.51	0	0	0	Repressed
963	BENPORATH_ES_1	-1.64	4.00	0	0	0	Repressed
964	BURTON_ADIPOGENESIS_3	-1.64	4.00	0	0	0	Repressed
965	DUTERTRE_ESTRADIOL_RESPONSE_6HR_UP	-1.64	4.00	0	0	0	Repressed
966	FLECHNER_BIOPSY_KIDNEY_TRANSPLANT_REJECTED_VS_OK_DN	-1.64	4.00	0	0	0	Repressed
967	GO_CELL_CYCLE_G2_M_PHASE_TRANSITION	-1.64	4.00	0	0	0	Repressed
968	GO_CONTRACTILE_FIBER	-1.64	4.00	0	0	0	Repressed
969	GO_FICOLIN_1_RICH_GRANULE	-1.64	4.00	0	0	0	Repressed

970	GO_REGULATION_OF_METAL_ION_TRANSPORT	-1.64	4.00	0	0	0	Repressed
971	GO_RESPONSE_TO_STEROID_HORMONE	-1.64	4.00	0	0	0	Repressed
972	GO_RNA_3_END_PROCESSING	-1.64	4.00	0	0	0	Repressed
973	GO_SULFUR_COMPOUND_METABOLIC_PROCESS	-1.64	4.00	0	0	0	Repressed
974	LINDGREN_BLADDER_CANCER_CLUSTER_1_DN	-1.64	4.00	0	0	0	Repressed
975	REACTOME_CELL_CYCLE_MITOTIC	-1.64	4.00	0	0	0	Repressed
976	REACTOME_PROTEIN_FOLDING	-1.64	4.00	0	0	0	Repressed
977	REACTOME_PROTEIN_LOCALIZATION	-1.64	4.00	0	0	0	Repressed
978	GO_DIGESTIVE_SYSTEM_PROCESS	-1.64	2.29	0	0	0	Repressed
979	REACTOME_REGULATION_OF_INSULIN_SECRETION	-1.64	2.29	0	0	0	Repressed
980	GO_NUCLEAR_RECEPTOR_TRANSCRIPTION_COACTIVATOR_ACTIVITY	-1.64	2.27	0	0	0	Repressed
981	GO_MATURATION_OF_SSU_RRNA	-1.64	2.02	0	0	0	Repressed
982	GO_REGULATION_OF_RESPONSE_TO_REACTIVE_OXYGEN_SPECIES	-1.64	2.01	0	0	0	Repressed
983	GO_TRICARBOXYLIC_ACID_CYCLE	-1.64	1.98	0	0	0	Repressed
984	GO_HISTONE_SERINE_KINASE_ACTIVITY	-1.64	1.89	0	0	0	Repressed
985	HADDAD_T_LYMPHOCYTE_AND_NK_PROGENITOR_DN	-1.64	1.89	0	0	0	Repressed
986	GO_CHROMATOID_BODY	-1.64	1.82	0	0	0	Repressed
987	PEDERSEN_METASTASIS_BY_ERBB2_ISOFORM_1	-1.64	1.79	0	0	0	Repressed
988	REACTOME_AQUAPORIN_MEDIATED_TRANSPORT	-1.64	1.78	0	0	0	Repressed
989	GO_AEROBIC_ELECTRON_TRANSPORT_CHAIN	-1.64	1.75	0	0	0	Repressed
990	CHR8P23	-1.64	1.73	0	0	0	Repressed
991	DAVIES_MULTIPLE_MYELOMA_VS_MGUS_DN	-1.64	1.73	0	0	0	Repressed
992	GO_MITOCHONDRIAL_TRNA_PROCESSING	-1.64	1.73	0	0	0	Repressed
993	GO_PEROXIREDOXIN_ACTIVITY	-1.64	1.73	0	0	0	Repressed
994	GO_GOLGI_LOCALIZATION	-1.64	1.72	0	0	0	Repressed
995	GO_NEGATIVE_REGULATION_OF_MRNA_SPLICING_VIA_SPLICEOSOME	-1.64	1.72	0	0	0	Repressed
996	REACTOME_RNA_POLYMERASE_III_TRANSCRIPTION_TERMINATION	-1.64	1.68	0	0	0	Repressed
997	GO_GROWTH_PLATE_CARTILAGE_DEVELOPMENT	-1.64	1.67	0	0	0	Repressed
998	GO_PSEUDOURIDINE_SYNTHESIS	-1.64	1.67	0	0	0	Repressed
999	GO_PEPTIDE_DISULFIDE_OXIDOREDUCTASE_ACTIVITY	-1.64	1.66	0	0	0	Repressed
1000	KEGG_TERPENOID_BACKBONE_BIOSYNTHESIS	-1.64	1.63	0	0	0	Repressed
1001	GO_NEGATIVE_REGULATION_OF_MEGAKARYOCYTE_DIFFERENTIATION	-1.64	1.62	0	0	0	Repressed
1002	REACTOME_IRF3_MEDIATED_INDUCTION_OF_TYPE_I_IFN	-1.64	1.60	0	0	0	Repressed
1003	KANG_FLUOROURACIL_RESISTANCE_DN	-1.64	1.59	0	0	0	Repressed
1004	GO_POSITIVE_REGULATION_OF_PODOSOME_ASSEMBLY	-1.64	1.58	0	0	0	Repressed
1005	REACTOME_FGFR2_MUTANT_RECEPTOR_ACTIVATION	-1.64	1.56	0	0	0	Repressed
1006	GO_HISTONE_ARGININE_N_METHYLTRANSFERASE_ACTIVITY	-1.64	1.52	0	0	0	Repressed
1007	BIOCARTA_ETC_PATHWAY	-1.64	1.47	0	0	0	Repressed
1008	DAZARD_RESPONSE_TO_UV_NHEK_UP	-1.63	4.00	0	0	0	Repressed
1009	GO_MICROTUBULE_CYTOSKELETON_ORGANIZATION_INVOLVED_IN_MITO	-1.63	4.00	0	0	0	Repressed
1010	GO_POSITIVE_REGULATION_OF_MULTI_ORGANISM_PROCESS	-1.63	4.00	0	0	0	Repressed
1011	REACTOME_CYTOKINE_SIGNALING_IN_IMMUNE_SYSTEM	-1.63	4.00	0	0	0	Repressed
1012	RICKMAN_METASTASIS_DN	-1.63	4.00	0	0	0	Repressed
1013	ZHANG_BREAST_CANCER_PROGENITORS_UP	-1.63	4.00	0	0	0	Repressed
1014	KIM_MYCN_AMPLIFICATION_TARGETS_UP	-1.63	2.58	0	0	0	Repressed
1015	JAZAERI_BREAST_CANCER_BRCA1_VS_BRCA2_UP	-1.63	2.56	0	0	0	Repressed
1016	REACTOME_SUMOYLATION_OF_UBIQUITINYLATION_PROTEINS	-1.63	2.56	0	0	0	Repressed
1017	REACTOME_ACTIVATION_OF_HOX_GENES_DURING_DIFFERENTIATION	-1.63	2.54	0	0	0	Repressed
1018	GO_SM_LIKE_PROTEIN_FAMILY_COMPLEX	-1.63	2.52	0	0	0	Repressed
1019	REACTOME_COPI_DEPENDENT_GOLGI_TO_ER_RETROGRADE_TRAFFIC	-1.63	2.48	0	0	0	Repressed
1020	SU_TESTIS	-1.63	2.26	0	0	0	Repressed
1021	WAESCH_ANAPHASE_PROMOTING_COMPLEX	-1.63	2.15	0	0	0	Repressed
1022	GO_CARDIAC_MUSCLE_CONTRACTION	-1.63	2.10	0	0	0	Repressed
1023	TSENG_IRS1_TARGETS_UP	-1.63	2.08	0	0	0	Repressed
1024	GO_CELLULAR_RESPONSE_TO_UV_B	-1.63	1.91	0	0	0	Repressed
1025	GO_BOX_H_ACA_TELOMERASE_RNP_COMPLEX	-1.63	1.90	0	0	0	Repressed
1026	GO_REGULATION_OF_CALCIUM_ION_TRANSMEMBRANE_TRANSPORTER	-1.63	1.88	0	0	0	Repressed
1027	GO_SPINDLE_LOCALIZATION	-1.63	1.88	0	0	0	Repressed
1028	LI_CISPLATIN_RESISTANCE_DN	-1.63	1.88	0	0	0	Repressed
1029	GO_MODULATION_BY_HOST_OF_VIRAL_PROCESS	-1.63	1.86	0	0	0	Repressed
1030	HERNANDEZ_ABERRANT_MITOSIS_BY_DOCETACEL_4NM_DN	-1.63	1.82	0	0	0	Repressed
1031	GO_TOLL_LIKE_RECEPTOR_9_SIGNALING_PATHWAY	-1.63	1.80	0	0	0	Repressed
1032	GROSS_ELK3_TARGETS_UP	-1.63	1.80	0	0	0	Repressed
1033	GO_HYDROGEN_PEROXIDE_CATABOLIC_PROCESS	-1.63	1.77	0	0	0	Repressed
1034	REACTOME_G_BETA:GAMMA_SIGNALLING_THROUGH_CDC42	-1.63	1.75	0	0	0	Repressed
1035	GO_DEAMINASE_ACTIVITY	-1.63	1.73	0	0	0	Repressed
1036	BIOCARTA_CTL_PATHWAY	-1.63	1.72	0	0	0	Repressed
1037	GO_REGULATION_OF_CARTILAGE_DEVELOPMENT	-1.63	1.72	0	0	0	Repressed
1038	BIOCARTA_MCALPAIN_PATHWAY	-1.63	1.70	0	0	0	Repressed
1039	GO_CELLULAR_RESPONSE_TO_CORTICOSTEROID_STIMULUS	-1.63	1.69	0	0	0	Repressed
1040	GO_SPLICEOSOMAL_TRI_SNRNP_COMPLEX	-1.63	1.69	0	0	0	Repressed
1041	MASRI_RESISTANCE_TO_TAMOXIFEN_AND_AROMATASE_INHIBITORS_UP	-1.63	1.60	0	0	0	Repressed
1042	AMIT_DELAYED_EARLY_GENES	-1.63	1.59	0	0	0	Repressed
1043	MURAKAMI_UV_RESPONSE_6HR_DN	-1.63	1.42	0	0	0	Repressed
1044	GO_NEGATIVE_REGULATION_OF_WNT_SIGNALING_PATHWAY	-1.62	4.00	0	0	0	Repressed
1045	GO_POSTTRANSCRIPTIONAL_REGULATION_OF_GENE_EXPRESSION	-1.62	4.00	0	0	0	Repressed
1046	GO_REGULATION_OF_CELL_CYCLE_G2_M_PHASE_TRANSITION	-1.62	4.00	0	0	0	Repressed
1047	GO_REGULATION_OF_TRANSPORTER_ACTIVITY	-1.62	4.00	0	0	0	Repressed
1048	GO_RIBOSE_PHOSPHATE_BIOSYNTHETIC_PROCESS	-1.62	4.00	0	0	0	Repressed
1049	GO_RNA_DEPENDENT_DNA_BIOSYNTHETIC_PROCESS	-1.62	4.00	0	0	0	Repressed
1050	GO_TRANSFERASE_ACTIVITY_TRANSFERRING_ONE_CARBON_GROUPS	-1.62	4.00	0	0	0	Repressed
1051	MARTORIATI_MDM4_TARGETS_FETAL_LIVER_UP	-1.62	4.00	0	0	0	Repressed
1052	SENESE_HDAC1_TARGETS_UP	-1.62	4.00	0	0	0	Repressed
1053	SMID_BREAST_CANCER_BASAL_UP	-1.62	4.00	0	0	0	Repressed
1054	WAMUNYOKOLI_OVARIAN_CANCER_LMP_UP	-1.62	4.00	0	0	0	Repressed
1055	GO_CARDIOCYTE_DIFFERENTIATION	-1.62	2.55	0	0	0	Repressed
1056	GO_CHANNEL_REGULATOR_ACTIVITY	-1.62	2.53	0	0	0	Repressed
1057	GO_CLUSTER_OF_ACTIN_BASED_CELL_PROJECTIONS	-1.62	2.52	0	0	0	Repressed
1058	FLECHNER_BIOPSY_KIDNEY_TRANSPLANT_REJECTED_VS_OK_UP	-1.62	2.26	0	0	0	Repressed
1059	GO_REGULATION_OF_TELOMERE_MAINTENANCE_VIA_TELOMERE_LENG	-1.62	2.24	0	0	0	Repressed
1060	GO_LYASE_ACTIVITY	-1.62	2.22	0	0	0	Repressed
1061	BROWN_MYELOID_CELL_DEVELOPMENT_DN	-1.62	2.21	0	0	0	Repressed
1062	KEGG_CARDIAC_MUSCLE_CONTRACTION	-1.62	1.99	0	0	0	Repressed
1063	MARCHINI_TRABECTEDIN_RESISTANCE_DN	-1.62	1.98	0	0	0	Repressed
1064	GO_PROTEIN_TRANSPORTER_ACTIVITY	-1.62	1.96	0	0	0	Repressed
1065	GO_SUBSTANTIA_NIGRA_DEVELOPMENT	-1.62	1.92	0	0	0	Repressed
1066	GO_SECONDARY_METABOLIC_PROCESS	-1.62	1.83	0	0	0	Repressed
1067	LEE_LIVER_CANCER_E2F1_UP	-1.62	1.82	0	0	0	Repressed
1068	GO_REGULATION_OF_DNA_METHYLATION	-1.62	1.78	0	0	0	Repressed
1069	BIOCARTA_BCR_PATHWAY	-1.62	1.76	0	0	0	Repressed
1070	KEGG_ARGININE_AND_PROLINE_METABOLISM	-1.62	1.74	0	0	0	Repressed
1071	BOHN_PRIMARY_IMMUNODEFICIENCY_SYNDROM_DN	-1.62	1.67	0	0	0	Repressed
1072	GO_REGULATION_OF_OXIDATIVE_PHOSPHORYLATION	-1.62	1.67	0	0	0	Repressed
1073	GO_INTRACELLULAR_LIPID_TRANSPORT	-1.62	1.66	0	0	0	Repressed
1074	GO_NUCLEOCYTOPLASMIC_CARRIER_ACTIVITY	-1.62	1.62	0	0	0	Repressed
1075	PARK_HSC_VS_MULTIPOTENT_PROGENITORS_DN	-1.62	1.55	0	0	0	Repressed
1076	REACTOME_GABA_B_RECEPTOR_ACTIVATION	-1.62	1.55	0	0	0	Repressed
1077	AMIT_SERUM_RESPONSE_20_MCF10A	-1.62	1.53	0	0	0	Repressed
1078	GO_SUPEROXIDE_ANION_GENERATION	-1.62	1.53	0	0	0	Repressed

1079	REACTOME_ADRENALINE_NORADRENALINE_INHIBITS_INSULIN_SECRETIC	-1.62	1.50	0	0	0	Repressed
1080	GO_PYRUVATE_METABOLIC_PROCESS	-1.62	1.49	0	0	0	Repressed
1081	BIOCARTA_CCR5_PATHWAY	-1.62	1.47	0	0	0	Repressed
1082	GARGALOVIC_RESPONSE_TO_OXIDIZED_PHOSPHOLIPIDS_BROWN_UP	-1.62	1.45	0	0	0	Repressed
1083	GO_MONOCARBOXYLIC_ACID_METABOLIC_PROCESS	-1.61	4.00	0	0	0	Repressed
1084	GO_POSITIVE_REGULATION_OF_ION_TRANSMEMBRANE_TRANSPORT	-1.61	4.00	0	0	0	Repressed
1085	GO_REGULATION_OF_ANIMAL_ORGAN_MORPHOGENESIS	-1.61	4.00	0	0	0	Repressed
1086	GO_REGULATION_OF_CHROMOSOME_SEGREGATION	-1.61	4.00	0	0	0	Repressed
1087	HOELZEL_NF1_TARGETS_UP	-1.61	4.00	0	0	0	Repressed
1088	MATTIOLI_MGUS_VS_PCL	-1.61	4.00	0	0	0	Repressed
1089	SARRIO_EPITHELIAL_MESENCHYMAL_TRANSITION_UP	-1.61	4.00	0	0	0	Repressed
1090	GO_LEUKOCYTE_CHEMOTAXIS	-1.61	2.55	0	0	0	Repressed
1091	GO_REGULATION_OF_LEUKOCYTE_APOPTOTIC_PROCESS	-1.61	2.55	0	0	0	Repressed
1092	GO_RESPONSE_TO_INTERLEUKIN_1	-1.61	2.50	0	0	0	Repressed
1093	GO_PURINE_CONTAINING_COMPOUND_BIOSYNTHETIC_PROCESS	-1.61	2.49	0	0	0	Repressed
1094	GO_REGULATION_OF_MUSCLE_SYSTEM_PROCESS	-1.61	2.49	0	0	0	Repressed
1095	SNF5_DN.V1_UP	-1.61	2.49	0	0	0	Repressed
1096	GO_ACTOMYOSIN	-1.61	2.25	0	0	0	Repressed
1097	NAGASHIMA_NRG1_SIGNALING_UP	-1.61	2.18	0	0	0	Repressed
1098	RICKMAN_TUMOR_DIFFERENTIATED_WELL_VS_MODERATELY_DN	-1.61	2.12	0	0	0	Repressed
1099	LEF1_UP.V1_UP	-1.61	2.08	0	0	0	Repressed
1100	YAO_TEMPORAL_RESPONSE_TO_PROGESTERONE_CLUSTER_7	-1.61	1.97	0	0	0	Repressed
1101	GO_REGULATION_OF_SISTER_CHROMATID_SEGREGATION	-1.61	1.95	0	0	0	Repressed
1102	GO_BOX_H_ACA_SNORNP_COMPLEX	-1.61	1.89	0	0	0	Repressed
1103	GO_NAD_METABOLIC_PROCESS	-1.61	1.85	0	0	0	Repressed
1104	GO_ESTABLISHMENT_OF_EPITHELIAL_CELL_POLARITY	-1.61	1.83	0	0	0	Repressed
1105	GO_MRNA_PSEUDOURIDINE_SYNTHESIS	-1.61	1.81	0	0	0	Repressed
1106	GARGALOVIC_RESPONSE_TO_OXIDIZED_PHOSPHOLIPIDS_TURQUOISE_[	-1.61	1.80	0	0	0	Repressed
1107	TERAMOTO_OPN_TARGETS_CLUSTER_1	-1.61	1.80	0	0	0	Repressed
1108	GARGALOVIC_RESPONSE_TO_OXIDIZED_PHOSPHOLIPIDS_GREEN_UP	-1.61	1.77	0	0	0	Repressed
1109	GO_CRD_MEDIATED_MRNA_STABILITY_COMPLEX	-1.61	1.76	0	0	0	Repressed
1110	REACTOME_DAG_AND_IP3_SIGNALING	-1.61	1.76	0	0	0	Repressed
1111	GO_RNA_POLYMERASE_ACTIVITY	-1.61	1.75	0	0	0	Repressed
1112	PID_CXCR3_PATHWAY	-1.61	1.75	0	0	0	Repressed
1113	KEGG_RENIN_ANGIOTENSIN_SYSTEM	-1.61	1.71	0	0	0	Repressed
1114	LIANG_HEMATOPOIESIS_STEM_CELL_NUMBER_SMALL_VS_HUGE_UP	-1.61	1.69	0	0	0	Repressed
1115	GO_RRNA_MODIFICATION	-1.61	1.66	0	0	0	Repressed
1116	GO_UBIQUITIN_PROTEIN_TRANSFERASE_ACTIVATOR_ACTIVITY	-1.61	1.65	0	0	0	Repressed
1117	REACTOME_CYCLIN_A_B1_B2_ASSOCIATED_EVENTS_DURING_G2_M_TF	-1.61	1.65	0	0	0	Repressed
1118	GO_REGULATION_OF_MAST_CELL_CHEMOTAXIS	-1.61	1.63	0	0	0	Repressed
1119	REACTOME_FGFR2_ALTERNATIVE_SPLICING	-1.61	1.61	0	0	0	Repressed
1120	GO_RNA_POLYMERASE_III_ACTIVITY	-1.61	1.60	0	0	0	Repressed
1121	GO_DOUBLE_STRANDED_METHYLATED_DNA_BINDING	-1.61	1.59	0	0	0	Repressed
1122	GO_PROTEIN_DISULFIDE_ISOMERASE_ACTIVITY	-1.61	1.54	0	0	0	Repressed
1123	BILANGES_SERUM_SENSITIVE_VIA_TSC1	-1.61	1.53	0	0	0	Repressed
1124	GO_RRNA_CONTAINING_RIBONUCLEOPROTEIN_COMPLEX_EXPORT_FRO	-1.61	1.52	0	0	0	Repressed
1125	BIOCARTA_RARRXR_PATHWAY	-1.61	1.51	0	0	0	Repressed
1126	GO_POSTTRANSLATIONAL_PROTEIN_TARGETING_TO_ENDOPLASMIC_RI	-1.61	1.45	0	0	0	Repressed
1127	ALCALAY_AML_BY_NPM1_LOCALIZATION_DN	-1.60	4.00	0	0	0	Repressed
1128	BENPORATH_MYC_MAX_TARGETS	-1.60	4.00	0	0	0	Repressed
1129	ERBB2_UP.V1_DN	-1.60	4.00	0	0	0	Repressed
1130	GO_MITOTIC_SISTER_CHROMATID_SEGREGATION	-1.60	4.00	0	0	0	Repressed
1131	GO_PROTEIN_TETRAMERIZATION	-1.60	4.00	0	0	0	Repressed
1132	IGLESIAS_E2F_TARGETS_UP	-1.60	4.00	0	0	0	Repressed
1133	KENNY_CTNNB1_TARGETS_UP	-1.60	4.00	0	0	0	Repressed
1134	MTOR_UP.V1_UP	-1.60	4.00	0	0	0	Repressed
1135	REACTOME_TCF_DEPENDENT_SIGNALING_IN_RESPONSE_TO_WNT	-1.60	4.00	0	0	0	Repressed
1136	ZHANG_TLX_TARGETS_60HR_DN	-1.60	4.00	0	0	0	Repressed
1137	GO_POSITIVE_REGULATION_OF_LIPID_BIOSYNTHETIC_PROCESS	-1.60	2.27	0	0	0	Repressed
1138	GO_MYELIN_SHEATH_ADAXONAL_REGION	-1.60	2.17	0	0	0	Repressed
1139	GO_CELLULAR_RESPONSE_TO_AMINO_ACID_STIMULUS	-1.60	1.91	0	0	0	Repressed
1140	GO_NEURAL_NUCLEUS_DEVELOPMENT	-1.60	1.87	0	0	0	Repressed
1141	GO_NUCLEOSOMAL_DNA_BINDING	-1.60	1.80	0	0	0	Repressed
1142	REACTOME_ANTIGEN_PRESENTATION:_FOLDING_ASSEMBLY_AND_PEPT	-1.60	1.78	0	0	0	Repressed
1143	GO_NEGATIVE_REGULATION_OF_LEUKOCYTE_APOPTOTIC_PROCESS	-1.60	1.76	0	0	0	Repressed
1144	GO_SCAVENGER_RECEPTOR_ACTIVITY	-1.60	1.72	0	0	0	Repressed
1145	REACTOME_SUMOYLATION_OF_SUMOYLATION_PROTEINS	-1.60	1.71	0	0	0	Repressed
1146	GO_CHAPERONIN_CONTAINING_T_COMPLEX	-1.60	1.69	0	0	0	Repressed
1147	BIOCARTA_TCAPOPTOSIS_PATHWAY	-1.60	1.61	0	0	0	Repressed
1148	GO_TRNA_3_END_PROCESSING	-1.60	1.60	0	0	0	Repressed
1149	GRAHAM_CML_QUIESCENT_VS_NORMAL_QUIESCENT_DN	-1.60	1.60	0	0	0	Repressed
1150	GO_CATALYTIC_ACTIVITY_ACTING_ON_A_RRNA	-1.60	1.59	0	0	0	Repressed
1151	GO_NUCLEAR_RECEPTOR_ACTIVITY	-1.60	1.57	0	0	0	Repressed
1152	GO_POSITIVE_REGULATION_OF_SODIUM_ION_TRANSMEMBRANE_TRANS	-1.60	1.57	0	0	0	Repressed
1153	SUZUKI_RESPONSE_TO_TSA	-1.60	1.56	0	0	0	Repressed
1154	GO_POSITIVE_REGULATION_OF_SODIUM_ION_TRANSMEMBRANE_TRANS	-1.60	1.55	0	0	0	Repressed
1155	GO_REGULATION_OF_TRANSLATIONAL_TERMINATION	-1.60	1.55	0	0	0	Repressed
1156	REACTOME_TIE2_SIGNALING	-1.60	1.55	0	0	0	Repressed
1157	GO_MITOCHONDRIAL_RNA_MODIFICATION	-1.60	1.54	0	0	0	Repressed
1158	MANN_RESPONSE_TO_AMIFOSTINE_DN	-1.60	1.54	0	0	0	Repressed
1159	REACTOME_ETHANOL_OXIDATION	-1.60	1.54	0	0	0	Repressed
1160	GO_MITOCHONDRIAL_TRANSCRIPTION	-1.60	1.50	0	0	0	Repressed
1161	TURJANSKI_MAPK8_AND_MAPK9_TARGETS	-1.60	1.49	0	0	0	Repressed
1162	GO_METHYL_CPG_BINDING	-1.60	1.47	0	0	0	Repressed
1163	GO_CELL_ADHESION_MOLECULE_BINDING	-1.59	4.00	0	0	0	Repressed
1164	GO_CYTOKINE_MEDIATED_SIGNALING_PATHWAY	-1.59	4.00	0	0	0	Repressed
1165	GO_LIGASE_ACTIVITY	-1.59	4.00	0	0	0	Repressed
1166	GO_RESPONSE_TO_OXYGEN_LEVELS	-1.59	4.00	0	0	0	Repressed
1167	GO_RNA_SPLICING_VIA_TRANSESTERIFICATION_REACTIONS	-1.59	4.00	0	0	0	Repressed
1168	HALLMARK_HYPOXIA	-1.59	4.00	0	0	0	Repressed
1169	GO_OTHER_ORGANISM	-1.59	2.53	0	0	0	Repressed
1170	GO_PROTEIN_SERINE_THREONINE_PHOSPHATASE_COMPLEX	-1.59	2.30	0	0	0	Repressed
1171	GO_FATTY_ACID_BIOSYNTHETIC_PROCESS	-1.59	2.23	0	0	0	Repressed
1172	REACTOME_COPI_MEDIATED_ANTEROGRADE_TRANSPORT	-1.59	2.23	0	0	0	Repressed
1173	GO_FATTY_ACID_BETA_OXIDATION	-1.59	2.09	0	0	0	Repressed
1174	LEONARD_HYPOXIA	-1.59	2.08	0	0	0	Repressed
1175	GO_PROTEIN_TARGETING_TO_MITOCHONDRION	-1.59	2.05	0	0	0	Repressed
1176	MOHANKUMAR_TLX1_TARGETS_DN	-1.59	1.98	0	0	0	Repressed
1177	BIOCARTA_CELL2CELL_PATHWAY	-1.59	1.83	0	0	0	Repressed
1178	GO_METAPHASE_ANAPHASE_TRANSITION_OF_CELL_CYCLE	-1.59	1.79	0	0	0	Repressed
1179	SMITH_LIVER_CANCER	-1.59	1.75	0	0	0	Repressed
1180	REACTOME_MAP2K_AND_MAPK_ACTIVATION	-1.59	1.74	0	0	0	Repressed
1181	GO_ENUCLEATE_ERYTHROCYTE_DIFFERENTIATION	-1.59	1.73	0	0	0	Repressed
1182	GO_REGULATION_OF_TRIGLYCERIDE_BIOSYNTHETIC_PROCESS	-1.59	1.70	0	0	0	Repressed
1183	GO_RNA_POLYMERASE_II_CTD_HEPTAPEPTIDE_REPEAT_KINASE_ACTIV	-1.59	1.70	0	0	0	Repressed
1184	GO_REGULATION_OF_TELOMERASE_ACTIVITY	-1.59	1.69	0	0	0	Repressed
1185	BIOCARTA_CARM1_PATHWAY	-1.59	1.67	0	0	0	Repressed
1186	GO_TRNA_SPLICING_LIGASE_COMPLEX	-1.59	1.66	0	0	0	Repressed
1187	FARMER_BREAST_CANCER_CLUSTER_5	-1.59	1.63	0	0	0	Repressed

1188	GO_NUCLEOBASE_BIOSYNTHETIC_PROCESS	-1.59	1.63	0	0	0	Repressed
1189	GO_POSITIVE_REGULATION_OF_TRANSCRIPTION_OF_NUCLEOLAR_LAR(	-1.59	1.60	0	0	0	Repressed
1190	GO_REGULATION_OF_FATTY_ACID_BIOSYNTHETIC_PROCESS	-1.59	1.60	0	0	0	Repressed
1191	MCBRYAN_TERMINAL_END_BUD_DN	-1.59	1.56	0	0	0	Repressed
1192	VALK_AML_CLUSTER_16	-1.59	1.53	0	0	0	Repressed
1193	GO_PHOSPHATIDYLINOSITOL_3_KINASE_BINDING	-1.59	1.51	0	0	0	Repressed
1194	MACLACHLAN_BRCA1_TARGETS_UP	-1.59	1.49	0	0	0	Repressed
1195	VILIMAS_NOTCH1_TARGETS_DN	-1.59	1.49	0	0	0	Repressed
1196	GO_MICOS_COMPLEX	-1.59	1.48	0	0	0	Repressed
1197	GO_ESTABLISHMENT_OF_MITOTIC_SPINDLE_LOCALIZATION	-1.59	1.47	0	0	0	Repressed
1198	GO_GEMINI_OF_COILED_BODIES	-1.59	1.47	0	0	0	Repressed
1199	REACTOME_REGULATION_OF_CYTOSKELETAL_REMODELING_AND_CEL	-1.59	1.47	0	0	0	Repressed
1200	GO_RESPONSE_TO_FUNGUS	-1.59	1.46	0	0	0	Repressed
1201	GO_TAT_PROTEIN_BINDING	-1.59	1.44	0	0	0	Repressed
1202	GO_REGULATION_OF_TRANSLATIONAL_ELONGATION	-1.59	1.43	0	0	0	Repressed
1203	GO_TRNA_THREONYLCARBAMOYLADENOSINE_METABOLIC_PROCESS	-1.59	1.43	1	0	0	Repressed
1204	GO_PHOSPHOFRUCTOKINASE_ACTIVITY	-1.59	1.41	0	0	0	Repressed
1205	GO_SODIUM_CHANNEL_REGULATOR_ACTIVITY	-1.59	1.39	0	0	0	Repressed
1206	CHUANG_OXIDATIVE_STRESS_RESPONSE_DN	-1.59	1.34	0	0	0	Repressed
1207	GO_N6_METHYLADENOSINE_CONTAINING_RNA_BINDING	-1.59	1.34	0	0	0	Repressed
1208	GO_MULTIMERIC_RIBONUCLEASE_P_COMPLEX	-1.59	1.30	0	0	0	Repressed
1209	GO_MONOCYTE_CHEMOTAXIS	-1.59	1.22	0	0	0	Repressed
1210	DITTMER_PTHLH_TARGETS_UP	-1.58	4.00	0	0	0	Repressed
1211	FISCHER_DREAM_TARGETS	-1.58	4.00	0	0	0	Repressed
1212	GO_REGULATION_OF_SYMBIOSIS_ENCOMPASSING_MUTUALISM_THROU(	-1.58	4.00	0	0	0	Repressed
1213	GO_RESPONSE_TO_HORMONE	-1.58	4.00	0	0	0	Repressed
1214	GO_T_CELL_RECEPTOR_SIGNALING_PATHWAY	-1.58	4.00	0	0	0	Repressed
1215	QI_HYPOXIA	-1.58	4.00	0	0	0	Repressed
1216	REACTOME_CELL_CYCLE	-1.58	4.00	0	0	0	Repressed
1217	REACTOME_RHO_GTPASE_EFFECTORS	-1.58	4.00	0	0	0	Repressed
1218	SMIRNOV_RESPONSE_TO_IR_6HR_DN	-1.58	4.00	0	0	0	Repressed
1219	GO_OXIDOREDUCTASE_ACTIVITY_ACTING_ON_CH_OH_GROUP_OF_DON	-1.58	2.51	0	0	0	Repressed
1220	GO_POSITIVE_REGULATION_OF_RNA_METABOLIC_PROCESS	-1.58	2.22	0	0	0	Repressed
1221	GO_COFACTOR_BIOSYNTHETIC_PROCESS	-1.58	2.17	0	0	0	Repressed
1222	GO_MONOCARBOXYLIC_ACID_TRANSPORT	-1.58	2.05	0	0	0	Repressed
1223	GO_POSITIVE_REGULATION_OF_VIRAL_LIFE_CYCLE	-1.58	2.01	0	0	0	Repressed
1224	LU_IL4_SIGNALING	-1.58	1.96	0	0	0	Repressed
1225	GAURNIER_PSMD4_TARGETS	-1.58	1.84	0	0	0	Repressed
1226	HOFFMANN_IMMATURE_TO_MATURE_B_LYMPHOCYTE_DN	-1.58	1.84	0	0	0	Repressed
1227	GO_NUCLEOID	-1.58	1.78	0	0	0	Repressed
1228	GO_EXTRACELLULAR_MATRIX_BINDING	-1.58	1.72	0	0	0	Repressed
1229	HESS_TARGETS_OF_HOXA9_AND_MEIS1_DN	-1.58	1.72	0	0	0	Repressed
1230	GO_OXALOACETATE_METABOLIC_PROCESS	-1.58	1.71	0	0	0	Repressed
1231	GO_HORMONE_ACTIVITY	-1.58	1.64	0	0	0	Repressed
1232	GO_NEGATIVE_REGULATION_OF_LIPOPROTEIN_PARTICLE_CLEARANCE	-1.58	1.63	0	0	0	Repressed
1233	GO_DEFENSE_RESPONSE_TO_GRAM_NEGATIVE_BACTERIUM	-1.58	1.61	0	0	0	Repressed
1234	GO_STAT_FAMILY_PROTEIN_BINDING	-1.58	1.61	0	0	0	Repressed
1235	KEGG_LIMONENE_AND_PINENE_DEGRADATION	-1.58	1.61	0	0	0	Repressed
1236	GO_VENTRICULAR_CARDIAC_MUSCLE_CELL_DEVELOPMENT	-1.58	1.58	0	0	0	Repressed
1237	FARMER_BREAST_CANCER_CLUSTER_4	-1.58	1.55	0	0	0	Repressed
1238	GO_CELL_PROLIFERATION_INVOLVED_IN_HEART_MORPHOGENESIS	-1.58	1.53	0	0	0	Repressed
1239	GO_POSITIVE_REGULATION_OF_REACTIVE_OXYGEN_SPECIES_METABO	-1.58	1.53	0	0	0	Repressed
1240	GO_RNA_POLYMERASE_II_PREINITIATION_COMPLEX_ASSEMBLY	-1.58	1.53	0	0	0	Repressed
1241	SCHUHMACHER_MYC_TARGETS_DN	-1.58	1.52	0	0	0	Repressed
1242	REACTOME_ANTIGEN_ACTIVATES_B_CELL_RECEPTOR_BCR_LEADING_T	-1.58	1.50	0	0	0	Repressed
1243	GO_CYSTEINE_METABOLIC_PROCESS	-1.58	1.49	0	0	0	Repressed
1244	KEGG_GALACTOSE_METABOLISM	-1.58	1.48	0	0	0	Repressed
1245	GO_RESPIRATORY_CHAIN_COMPLEX_III	-1.58	1.47	0	0	0	Repressed
1246	GO_TISSUE_REGENERATION	-1.58	1.47	0	0	0	Repressed
1247	GO_ENDOPEPTIDASE_ACTIVATOR_ACTIVITY	-1.58	1.46	0	0	0	Repressed
1248	GO_POSITIVE_REGULATION_OF_FATTY_ACID_BIOSYNTHETIC_PROCESS	-1.58	1.44	0	0	0	Repressed
1249	GO_POSITIVE_REGULATION_OF_STEROID_METABOLIC_PROCESS	-1.58	1.42	0	0	0	Repressed
1250	GO_ESTABLISHMENT_OR_MAINTENANCE_OF_TRANSMEMBRANE_ELECTI	-1.58	1.41	0	0	0	Repressed
1251	HANN_RESISTANCE_TO_BCL2_INHIBITOR_DN	-1.58	1.39	0	0	0	Repressed
1252	GO_PROTEIN_LOCALIZATION_TO_NUCLEAR_ENVELOPE	-1.58	1.37	0	0	0	Repressed
1253	GO_CELLULAR_RESPONSE_TO_FLUID_SHEAR_STRESS	-1.58	1.31	0	0	0	Repressed
1254	MONTERO_THYROID_CANCER_POOR_SURVIVAL_UP	-1.58	1.31	0	0	0	Repressed
1255	ACEVEDO_LIVER_TUMOR_VS_NORMAL_ADJACENT_TISSUE_UP	-1.57	4.00	0	0	0	Repressed
1256	CHICAS_RB1_TARGETS_CONFLUENT	-1.57	4.00	0	0	0	Repressed
1257	GO_UNFOLDED_PROTEIN_BINDING	-1.57	4.00	0	0	0	Repressed
1258	JOHNSTONE_PARVB_TARGETS_3_DN	-1.57	4.00	0	0	0	Repressed
1259	RB_DN.V1_DN	-1.57	4.00	0	0	0	Repressed
1260	REACTOME_S_PHASE	-1.57	4.00	0	0	0	Repressed
1261	GO_VESICLE_BUDDING_FROM_MEMBRANE	-1.57	2.54	0	0	0	Repressed
1262	GO_MULTI_ORGANISM_LOCALIZATION	-1.57	2.53	0	0	0	Repressed
1263	LEE_LIVER_CANCER_SURVIVAL_UP	-1.57	2.52	0	0	0	Repressed
1264	GO_REGULATION_OF_TELOMERE_MAINTENANCE	-1.57	2.50	0	0	0	Repressed
1265	GO_REGULATION_OF_BLOOD_CIRCULATION	-1.57	2.48	0	0	0	Repressed
1266	GO_REGULATION_OF_CELLULAR_RESPONSE_TO_HEAT	-1.57	2.09	0	0	0	Repressed
1267	ALCALAY_AML_BY_NPM1_LOCALIZATION_UP	-1.57	1.96	0	0	0	Repressed
1268	CHR10Q23	-1.57	1.91	0	0	0	Repressed
1269	GO_CARDIAC_CONDUCTION	-1.57	1.88	0	0	0	Repressed
1270	REACTOME_SIGNALING_BY_RAS_MUTANTS	-1.57	1.80	0	0	0	Repressed
1271	SABATES_COLORECTAL_ADENOMA_UP	-1.57	1.80	0	0	0	Repressed
1272	GENTILE_UV_LOW_DOSE_DN	-1.57	1.74	0	0	0	Repressed
1273	GO_COFACTOR_CATABOLIC_PROCESS	-1.57	1.74	0	0	0	Repressed
1274	GO_CARDIAC_MUSCLE_CELL_DIFFERENTIATION	-1.57	1.73	0	0	0	Repressed
1275	GO_PROTEIN_KINASE_A_BINDING	-1.57	1.73	0	0	0	Repressed
1276	GO_POSITIVE_REGULATION_OF_VOLTAGE_GATED_POTASSIUM_CHANNI	-1.57	1.72	0	0	0	Repressed
1277	GO_STRUCTURAL_CONSTITUENT_OF_NUCLEAR_PORE	-1.57	1.72	0	0	0	Repressed
1278	REACTOME_CONDENSATION_OF_PROPHASE_CHROMOSOMES	-1.57	1.71	0	0	0	Repressed
1279	KONDO_HYPOXIA	-1.57	1.63	0	0	0	Repressed
1280	GO_DE_NOVO_PROTEIN_FOLDING	-1.57	1.62	0	0	0	Repressed
1281	REACTOME_METABOLISM_OF_INGESTED_SEMET_SEC_MESEC_INTO_H2	-1.57	1.62	0	0	0	Repressed
1282	REACTOME_HSF1_ACTIVATION	-1.57	1.59	0	0	0	Repressed
1283	PID_RETINOIC_ACID_PATHWAY	-1.57	1.58	0	0	0	Repressed
1284	CHR3P14	-1.57	1.57	0	0	0	Repressed
1285	LEE_LIVER_CANCER_DENA_UP	-1.57	1.56	0	0	0	Repressed
1286	GO_MATURE_RIBOSOME_ASSEMBLY	-1.57	1.55	0	0	0	Repressed
1287	GO_P_P_BOND_HYDROLYSIS_DRIVEN_PROTEIN_TRANSMEMBRANE_TRA	-1.57	1.55	0	0	0	Repressed
1288	GO_REGULATION_OF_SUPEROXIDE_ANION_GENERATION	-1.57	1.54	0	0	0	Repressed
1289	GO_POSITIVE_REGULATION_OF_MITOCHONDRIAL_CALCIUM_ION_CONCE	-1.57	1.52	0	0	0	Repressed
1290	GO_COMPACT_MYELIN	-1.57	1.51	0	0	0	Repressed
1291	REACTOME_ADP_SIGNALLING_THROUGH_P2Y_PURINOCEPTOR_12	-1.57	1.49	0	0	0	Repressed
1292	REACTOME_SEMA3A_PAK_DEPENDENT_AXON_REPULSION	-1.57	1.49	0	0	0	Repressed
1293	GO_POSITIVE_REGULATION_OF_STEROID_BIOSYNTHETIC_PROCESS	-1.57	1.48	0	0	0	Repressed
1294	REACTOME_ASPARTATE_AND_ASPARAGINE_METABOLISM	-1.57	1.48	0	0	0	Repressed
1295	GO_PEPTIDYL_ARGININE_METHYLATION	-1.57	1.47	0	0	0	Repressed
1296	REACTOME_CELLULAR_HEXOSE_TRANSPORT	-1.57	1.47	0	0	0	Repressed

1297	GO_THIOREDOXIN_PEROXIDASE_ACTIVITY	-1.57	1.46	0	0	0	Repressed
1298	PIEPOLI_LGI1_TARGETS_UP	-1.57	1.46	0	0	0	Repressed
1299	SUMI_HNF4A_TARGETS	-1.57	1.45	0	0	0	Repressed
1300	GO_POLY_G_BINDING	-1.57	1.44	0	0	0	Repressed
1301	GO_PSEUDOPODIUM	-1.57	1.44	0	0	0	Repressed
1302	GO_I_KAPPAB_PHOSPHORYLATION	-1.57	1.42	0	0	0	Repressed
1303	GO_L_SERINE_METABOLIC_PROCESS	-1.57	1.41	0	0	0	Repressed
1304	REACTOME_CHK1_CHK2_CDS1_MEDIATED_INACTIVATION_OF_CYCLIN_E	-1.57	1.41	0	0	0	Repressed
1305	GO_SHORT_CHAIN_FATTY_ACID_CATABOLIC_PROCESS	-1.57	1.38	0	0	0	Repressed
1306	DALESSIO_TSA_RESPONSE	-1.57	1.35	0	0	0	Repressed
1307	GO_RESPONSE_TO_CORTICOSTERONE	-1.57	1.30	0	0	0	Repressed
1308	FLECHNER_BIOPSY_KIDNEY_TRANSPLANT_OK_VS_DONOR_UP	-1.56	4.00	0	0	0	Repressed
1309	GO_RESPONSE_TO_CYTOKINE	-1.56	4.00	0	0	0	Repressed
1310	GROSS_HYPOXIA_VIA_ELK3_UP	-1.56	4.00	0	0	0	Repressed
1311	MULLIGHAN_MLL_SIGNATURE_2_UP	-1.56	4.00	0	0	0	Repressed
1312	RICKMAN_TUMOR_DIFFERENTIATED_WELL_VS_POORLY_DN	-1.56	4.00	0	0	0	Repressed
1313	SCHUETZ_BREAST_CANCER_DUCTAL_INVASIVE_UP	-1.56	4.00	0	0	0	Repressed
1314	SHEN_SMARCA2_TARGETS_DN	-1.56	4.00	0	0	0	Repressed
1315	HUANG_GATA2_TARGETS_DN	-1.56	2.55	0	0	0	Repressed
1316	GO_COENZYME_BIOSYNTHETIC_PROCESS	-1.56	2.50	0	0	0	Repressed
1317	HALLMARK_ESTROGEN_RESPONSE_EARLY	-1.56	2.50	0	0	0	Repressed
1318	CYCLIN_D1_KE_.V1_UP	-1.56	2.49	0	0	0	Repressed
1319	HALLMARK_ALLOGRAFT_REJECTION	-1.56	2.48	0	0	0	Repressed
1320	HALLMARK_ESTROGEN_RESPONSE_LATE	-1.56	2.48	0	0	0	Repressed
1321	CAHOY_ASTROCYTIC	-1.56	2.26	0	0	0	Repressed
1322	GO_RIBOSOMAL_LARGE_SUBUNIT_BIOGENESIS	-1.56	2.09	0	0	0	Repressed
1323	KEGG_CYTOKINE_CYTOKINE_RECEPTOR_INTERACTION	-1.56	2.07	0	0	0	Repressed
1324	HIRSCH_CELLULAR_TRANSFORMATION_SIGNATURE_DN	-1.56	2.06	0	0	0	Repressed
1325	DUNNE_TARGETS_OF_AML1_MTG8_FUSION_UP	-1.56	1.97	0	0	0	Repressed
1326	REACTOME_RNA_POLYMERASE_II_TRANSCRIPTION_TERMINATION	-1.56	1.81	0	0	0	Repressed
1327	GO_SPINDLE_MIDZONE	-1.56	1.78	0	0	0	Repressed
1328	GO_ENDOPEPTIDASE_REGULATOR_ACTIVITY	-1.56	1.76	0	0	0	Repressed
1329	GO_DNA_REPLICATION_DEPENDENT_NUCLEOSOME_ORGANIZATION	-1.56	1.69	0	0	0	Repressed
1330	HUANG_DASATINIB_RESISTANCE_UP	-1.56	1.69	0	0	0	Repressed
1331	GO_REGULATION_OF_RESPONSE_TO_OXIDATIVE_STRESS	-1.56	1.68	0	0	0	Repressed
1332	GO_REGULATION_OF_INTRACELLULAR_LIPID_TRANSPORT	-1.56	1.65	0	0	0	Repressed
1333	GO_MATURATION_OF_5_8S_RRNA_FROM_TRICISTRONIC_RRNA_TRANS(	-1.56	1.62	0	0	0	Repressed
1334	TURASHVILI_BREAST_DUCTAL_CARCINOMA_VS_DUCTAL_NORMAL_UP	-1.56	1.62	0	0	0	Repressed
1335	GO_ENDOCHONDRAL_BONE_MORPHOGENESIS	-1.56	1.60	0	0	0	Repressed
1336	BIOCARTA_CALCINEURIN_PATHWAY	-1.56	1.58	0	0	0	Repressed
1337	GO_REGULATION_OF_VESICLE_FUSION	-1.56	1.58	0	0	0	Repressed
1338	LEIN_NEURON_MARKERS	-1.56	1.58	0	0	0	Repressed
1339	GO_NEGATIVE_REGULATION_OF_INSULIN_SECRETION	-1.56	1.54	0	0	0	Repressed
1340	GO_FRUCTOSE_1_6_BISPHOSPHATE_METABOLIC_PROCESS	-1.56	1.51	0	0	0	Repressed
1341	GO_PROSTATE_GLAND_DEVELOPMENT	-1.56	1.51	0	0	0	Repressed
1342	HE_PTEN_TARGETS_UP	-1.56	1.51	0	0	0	Repressed
1343	GO_NEGATIVE_REGULATION_OF_SUBSTRATE_ADHESION_DEPENDENT_(	-1.56	1.48	0	0	0	Repressed
1344	GO_PEBOW_COMPLEX	-1.56	1.48	0	0	0	Repressed
1345	GO_REGULATION_OF_T_HELPER_1_TYPE_IMMUNE_RESPONSE	-1.56	1.48	0	0	0	Repressed
1346	MARCINIAK_ER_STRESS_RESPONSE_VIA_CHOP	-1.56	1.48	0	0	0	Repressed
1347	LEE_LIVER_CANCER_E2F1_DN	-1.56	1.47	0	0	0	Repressed
1348	GO_DNA_PROTECTION	-1.56	1.44	0	0	0	Repressed
1349	GO_POSITIVE_REGULATION_OF_SUPEROXIDE_ANION_GENERATION	-1.56	1.44	0	0	0	Repressed
1350	KEGG_PRIMARY_BILE_ACID_BIOSYNTHESIS	-1.56	1.43	0	0	0	Repressed
1351	SUH_COEXPRESSED_WITH_ID1_AND_ID2_UP	-1.56	1.43	0	0	0	Repressed
1352	GO_FACIAL_NERVE_MORPHOGENESIS	-1.56	1.42	0	0	0	Repressed
1353	GO_MEDIUM_CHAIN_FATTY_ACID_COA_LIGASE_ACTIVITY	-1.56	1.42	0	0	0	Repressed
1354	GO_NEUTRAL_LIPID_BIOSYNTHETIC_PROCESS	-1.56	1.42	0	0	0	Repressed
1355	SCHURINGA_STAT5A_TARGETS_UP	-1.56	1.41	0	0	0	Repressed
1356	GO_CENTROMERIC_SISTER_CHROMATID_COHESION	-1.56	1.39	0	0	0	Repressed
1357	GO_CHOLESTEROL_CATABOLIC_PROCESS	-1.56	1.39	0	0	0	Repressed
1358	GO_MYOBLAST_MIGRATION	-1.56	1.39	0	0	0	Repressed
1359	GO_CARBONYL_REDUCTASE_NADPH_ACTIVITY	-1.56	1.36	0	0	0	Repressed
1360	GO_GDP_DISSOCIATION_INHIBITOR_ACTIVITY	-1.56	1.28	0	0	0	Repressed
1361	GO_CALCIUM_CHANNEL_INHIBITOR_ACTIVITY	-1.56	1.25	0	0	0	Repressed
1362	GO_FC_RECEPTOR_SIGNALING_PATHWAY	-1.55	4.00	0	0	0	Repressed
1363	GO_SPINDLE_POLE	-1.55	4.00	0	0	0	Repressed
1364	LEE_LIVER_CANCER_SURVIVAL_DN	-1.55	4.00	0	0	0	Repressed
1365	MONNIER_POSTRADIATION_TUMOR_ESCAPE_UP	-1.55	4.00	0	0	0	Repressed
1366	REACTOME_CELLULAR_RESPONSES_TO_EXTERNAL_STIMULI	-1.55	4.00	0	0	0	Repressed
1367	REACTOME_MITOTIC_PROMETAPHASE	-1.55	4.00	0	0	0	Repressed
1368	SWEET_LUNG_CANCER_KRAS_UP	-1.55	4.00	0	0	0	Repressed
1369	ZHENG_GLIOBLASTOMA_PLASTICITY_UP	-1.55	2.45	0	0	0	Repressed
1370	GO_RESPIRATORY_SYSTEM_DEVELOPMENT	-1.55	2.23	0	0	0	Repressed
1371	HELLER_SILENCED_BY_METHYLATION_DN	-1.55	2.08	0	0	0	Repressed
1372	HALLMARK_PEROXISOME	-1.55	2.07	0	0	0	Repressed
1373	GO_HEPATICOBILIARY_SYSTEM_DEVELOPMENT	-1.55	2.06	0	0	0	Repressed
1374	GO_RIBOSOMAL_SMALL_SUBUNIT_BIOGENESIS	-1.55	2.06	0	0	0	Repressed
1375	LIN_APC_TARGETS	-1.55	1.96	0	0	0	Repressed
1376	HOFFMANN_SMALL_PRE_BII_TO_IMMATURE_B_LYMPHOCYTE_DN	-1.55	1.90	0	0	0	Repressed
1377	GO_POSITIVE_REGULATION_OF_TYPE_I_INTERFERON_PRODUCTION	-1.55	1.77	0	0	0	Repressed
1378	JOSEPH_RESPONSE_TO_SODIUM_BUTYRATE_DN	-1.55	1.70	0	0	0	Repressed
1379	SCHMIDT_POR_TARGETS_IN_LIMB_BUD_UP	-1.55	1.68	0	0	0	Repressed
1380	GO_STEREOCILIUM_TIP	-1.55	1.67	0	0	0	Repressed
1381	GO_EXTRACELLULAR_MATRIX_CONSTITUENT_CONFERRING_ELASTICITY	-1.55	1.63	0	0	0	Repressed
1382	LI_WILMS_TUMOR_ANAPLASTIC_UP	-1.55	1.63	0	0	0	Repressed
1383	GO_CELLULAR_RESPONSE_TO_FATTY_ACID	-1.55	1.62	0	0	0	Repressed
1384	GO_POSITIVE_REGULATION_OF_HORMONE_BIOSYNTHETIC_PROCESS	-1.55	1.58	0	0	0	Repressed
1385	GO_KINETOCHORE_BINDING	-1.55	1.54	0	0	0	Repressed
1386	GO_RNA_POLYMERASE_II_ACTIVITY	-1.55	1.53	0	0	0	Repressed
1387	NGUYEN_NOTCH1_TARGETS_UP	-1.55	1.53	0	0	0	Repressed
1388	CHNG_MULTIPLE_MYELOMA_HYPERPLOID_DN	-1.55	1.49	0	0	0	Repressed
1389	WUNDER_INFLAMMATORY_RESPONSE_AND_CHOLESTEROL_UP	-1.55	1.49	0	0	0	Repressed
1390	GO_MITOCHONDRIAL_RNA_3_END_PROCESSING	-1.55	1.46	0	0	0	Repressed
1391	GO_REGULATION_OF_CARDIAC_MUSCLE_CONTRACTION_BY_CALCIUM_	-1.55	1.46	0	0	0	Repressed
1392	REACTOME_DETOXIFICATION_OF_REACTIVE_OXYGEN_SPECIES	-1.55	1.44	0	0	0	Repressed
1393	KAPOSI_LIVER_CANCER_MET_UP	-1.55	1.43	0	0	0	Repressed
1394	GO_SCHMIDT_LANTERMAN_INCISURE	-1.55	1.42	0	0	0	Repressed
1395	FUNG_IL2_TARGETS_WITH_STAT5_BINDING_SITES	-1.55	1.40	0	0	0	Repressed
1396	REACTOME_NOTCH2_INTRACELLULAR_DOMAIN_REGULATES_TRANSCRI	-1.55	1.40	0	0	0	Repressed
1397	REACTOME_PKA_MEDIATED_PHOSPHORYLATION_OF_CREB	-1.55	1.40	0	0	0	Repressed
1398	COLIN_PILOCYTIC_ASTROCYTOMA_VS_GLIOBLASTOMA_UP	-1.55	1.39	0	0	0	Repressed
1399	LIU_IL13_PRIMING_MODEL	-1.55	1.39	0	0	0	Repressed
1400	GO_CELLULAR_RESPONSE_TO_OXYGEN_RADICAL	-1.55	1.38	0	0	0	Repressed
1401	GO_PEPTIDE_CATABOLIC_PROCESS	-1.55	1.38	0	0	0	Repressed
1402	GO_PRERIBOSOME_SMALL_SUBUNIT_PRECURSOR	-1.55	1.37	0	0	0	Repressed
1403	GO_REGULATION_OF_PROTEIN_LOCALIZATION_TO_CHROMOSOME_TEL	-1.55	1.37	0	0	0	Repressed
1404	GO_NEGATIVE_REGULATION_OF_RESPONSE_TO_DRUG	-1.55	1.36	0	0	0	Repressed
1405	GO_NEGATIVE_REGULATION_OF_T_CELL_APOPTOTIC_PROCESS	-1.55	1.36	0	0	0	Repressed

1406	GO_PULMONARY_VALVE_DEVELOPMENT	-1.55	1.36	0	0	0	Repressed
1407	GO_EYELID_DEVELOPMENT_IN_CAMERA_TYPE_EYE	-1.55	1.34	0	0	0	Repressed
1408	GO_N_TERMINAL_PROTEIN_ACETYLTRANSFERASE_COMPLEX	-1.55	1.33	0	0	0	Repressed
1409	REACTOME_INTEGRATION_OF_PROVIRUS	-1.55	1.31	0	0	0	Repressed
1410	GO_PULMONARY_VALVE_MORPHOGENESIS	-1.55	1.30	0	0	0	Repressed
1411	GO_NEGATIVE_REGULATION_OF_TELOMERASE_ACTIVITY	-1.55	1.28	0	0	0	Repressed
1412	GO_POSITIVE_REGULATION_OF_OXIDATIVE_PHOSPHORYLATION	-1.55	1.27	0	0	0	Repressed
1413	GO_REGULATION_OF_UNSATURATED_FATTY_ACID_BIOSYNTHETIC_PRC	-1.55	1.21	0	0	0	Repressed
1414	GO_RESPONSE_TO_LIPID	-1.54	4.00	0	0	0	Repressed
1415	MULLIGHAN_MLL_SIGNATURE_1_UP	-1.54	4.00	0	0	0	Repressed
1416	REACTOME_INTERFERON_SIGNALING	-1.54	4.00	0	0	0	Repressed
1417	ELVIDGE_HIF1A_TARGETS_DN	-1.54	2.25	0	0	0	Repressed
1418	GO_COLLAGEN_CONTAINING_EXTRACELLULAR_MATRIX	-1.54	2.20	0	0	0	Repressed
1419	GRABARCZYK_BCL11B_TARGETS_UP	-1.54	2.07	0	0	0	Repressed
1420	LANDIS_ERBB2_BREAST_TUMORS_324_DN	-1.54	2.06	0	0	0	Repressed
1421	GO_TRANSCRIPTION_BY_RNA_POLYMERASE_I	-1.54	1.88	0	0	0	Repressed
1422	GO_SUBSTRATE_ADHESION_DEPENDENT_CELL_SPREADING	-1.54	1.87	0	0	0	Repressed
1423	GO_RESPONSE_TO_AMINO_ACID	-1.54	1.86	0	0	0	Repressed
1424	VANHARANTA_UTERINE_FIBROID_WITH_7Q_DELETION_UP	-1.54	1.79	0	0	0	Repressed
1425	BRACHAT_RESPONSE_TO_CAMPTOTHECIN_DN	-1.54	1.77	0	0	0	Repressed
1426	GO_VENTRICULAR_CARDIAC_MUSCLE_TISSUE_DEVELOPMENT	-1.54	1.75	0	0	0	Repressed
1427	GO_MITOTIC_SPINDLE_ASSEMBLY	-1.54	1.72	0	0	0	Repressed
1428	KEGG_TYROSINE_METABOLISM	-1.54	1.55	0	0	0	Repressed
1429	GO_MYELIN_SHEATH	-1.54	1.54	0	0	0	Repressed
1430	GO_DICARBOXYLIC_ACID_BIOSYNTHETIC_PROCESS	-1.54	1.49	0	0	0	Repressed
1431	GO_NEGATIVE_REGULATION_OF_MRNA_PROCESSING	-1.54	1.49	0	0	0	Repressed
1432	SIG_REGULATION_OF_THE_ACTIN_CYTOSKELETON_BY_RHO_GTPASES	-1.54	1.49	0	0	0	Repressed
1433	CHR12Q22	-1.54	1.48	0	0	0	Repressed
1434	GO_OXIDATIVE_RNA_DEMETHYLASE_ACTIVITY	-1.54	1.48	0	0	0	Repressed
1435	GO_U2_TYPE_CATALYTIC_STEP_2_SPLICEOSOME	-1.54	1.47	0	0	0	Repressed
1436	GO_OXIDATIVE_RNA_DEMETHYLATION	-1.54	1.46	0	0	0	Repressed
1437	GO_HYDROLASE_ACTIVITY_ACTING_ON_CARBON_NITROGEN_BUT_NOT	-1.54	1.45	0	0	0	Repressed
1438	GO_NECROPTOTIC_SIGNALING_PATHWAY	-1.54	1.45	0	0	0	Repressed
1439	NIELSEN_SCHWANNOMA_UP	-1.54	1.43	0	0	0	Repressed
1440	BIOCARTA_PRION_PATHWAY	-1.54	1.42	0	0	0	Repressed
1441	GO_POSITIVE_REGULATION_OF_POTASSIUM_ION_TRANSPORT	-1.54	1.41	0	0	0	Repressed
1442	GO_EXTRACELLULAR_MATRIX_COMPONENT	-1.54	1.40	0	0	0	Repressed
1443	GO_POLYADENYLATION_DEPENDENT_RNA_CATABOLIC_PROCESS	-1.54	1.40	0	0	0	Repressed
1444	GO_TRACHEA_DEVELOPMENT	-1.54	1.39	0	0	0	Repressed
1445	REACTOME_IMPORT_OF_PALMITOYL_COA_INTO_THE_MITOCHONDRIAL_	-1.54	1.39	0	0	0	Repressed
1446	BIOCARTA_CDMAC_PATHWAY	-1.54	1.38	0	0	0	Repressed
1447	BIOCARTA_RAN_PATHWAY	-1.54	1.37	0	0	0	Repressed
1448	GO_SPECTRIN_BINDING	-1.54	1.34	0	0	0	Repressed
1449	BIOCARTA_TID_PATHWAY	-1.54	1.33	0	0	0	Repressed
1450	DAZARD_UV_RESPONSE_CLUSTER_G4	-1.54	1.33	0	0	0	Repressed
1451	GO_5_3_DNA_HELICASE_ACTIVITY	-1.54	1.32	0	0	0	Repressed
1452	GO_POSITIVE_REGULATION_OF_G_PROTEIN_COUPLED_RECEPTOR_SIG	-1.54	1.29	0	0	0	Repressed
1453	REACTOME_NEF_MEDIATES_DOWN_MODULATION_OF_CELL_SURFACE_	-1.54	1.29	0	0	0	Repressed
1454	GO_CALCIUM_ION_EXPORT	-1.54	1.28	0	0	0	Repressed
1455	GO_VITAMIN_TRANSMEMBRANE_TRANSPORTER_ACTIVITY	-1.54	1.28	0	0	0	Repressed
1456	GO_EXONUCLEOLYTIC_TRIMMING_INVOLVED_IN_RRNA_PROCESSING	-1.54	1.24	0	0	0	Repressed
1457	CHEN_HOXA5_TARGETS_6HR_UP	-1.54	1.23	0	0	0	Repressed
1458	PLASARI_NFIC_TARGETS_BASAL_DN	-1.54	1.23	0	0	0	Repressed
1459	REACTOME_TETRAHYDROBIOPTERIN_BH4_SYNTHESIS_RECYCLING_SAL	-1.54	1.21	0	0	0	Repressed
1460	GO_ENDOLYSOSOME	-1.54	1.20	0	0	0	Repressed
1461	GO_U5_SNRNP	-1.54	1.19	0	0	0	Repressed
1462	GO_NIK_NF_KAPPAB_SIGNALING	-1.53	4.00	0	0	0	Repressed
1463	GO_SISTER_CHROMATID_SEGREGATION	-1.53	4.00	0	0	0	Repressed
1464	KRIEG_KDM3A_TARGETS_NOT_HYPOXIA	-1.53	4.00	0	0	0	Repressed
1465	MARKEY_RB1_ACUTE_LOF_UP	-1.53	4.00	0	0	0	Repressed
1466	MATSUDA_NATURAL_KILLER_DIFFERENTIATION	-1.53	4.00	0	0	0	Repressed
1467	REACTOME_MAPK_FAMILY_SIGNALING_CASCADES	-1.53	4.00	0	0	0	Repressed
1468	REACTOME_SIGNALING_BY_INTERLEUKINS	-1.53	4.00	0	0	0	Repressed
1469	SANSOM_APC_TARGETS_REQUIRE_MYC	-1.53	4.00	0	1	0	Repressed
1470	SERVITJA_ISLET_HNF1A_TARGETS_UP	-1.53	2.52	0	0	0	Repressed
1471	GO_PROTEIN_LOCALIZATION_TO_MITOCHONDRION	-1.53	2.51	0	0	0	Repressed
1472	EIF4E_UP	-1.53	2.22	0	0	0	Repressed
1473	GO_LEUKOCYTE_APOPTOTIC_PROCESS	-1.53	2.22	0	0	0	Repressed
1474	GO_STEROID_BIOSYNTHETIC_PROCESS	-1.53	2.20	0	0	0	Repressed
1475	REACTOME_GOLGI_TO_ER_RETROGRADE_TRANSPORT	-1.53	1.99	0	0	0	Repressed
1476	HU_ANGIOGENESIS_DN	-1.53	1.91	0	0	0	Repressed
1477	GO_CELLULAR_RESPONSE_TO_KETONE	-1.53	1.85	0	0	0	Repressed
1478	BOYAULT_LIVER_CANCER_SUBCLASS_G6_UP	-1.53	1.70	0	0	0	Repressed
1479	GO_CELLULAR_DETOXIFICATION	-1.53	1.68	0	0	0	Repressed
1480	CROONQUIST_NRAS_VS_STROMAL_STIMULATION_UP	-1.53	1.61	0	0	0	Repressed
1481	HALLMARK_CHOLESTEROL_HOMEOSTASIS	-1.53	1.61	0	0	0	Repressed
1482	KEGG_PENTOSE_PHOSPHATE_PATHWAY	-1.53	1.49	0	0	0	Repressed
1483	GO_GENITALIA_DEVELOPMENT	-1.53	1.46	0	0	0	Repressed
1484	GO_POSITIVE_REGULATION_OF_CALCIUM_ION_TRANSMEMBRANE_TRAN	-1.53	1.44	0	0	0	Repressed
1485	WILSON_PROTEASES_AT_TUMOR_BONE_INTERFACE_UP	-1.53	1.44	0	0	0	Repressed
1486	GO_CELLULAR_DEFENSE_RESPONSE	-1.53	1.42	0	0	0	Repressed
1487	GO_NEGATIVE_REGULATION_OF_RELEASE_OF_CYTOCHROME_C_FROM	-1.53	1.42	0	0	0	Repressed
1488	REACTOME_THE_ROLE_OF_NEF_IN_HIV_1_REPLICATION_AND_DISEASE_	-1.53	1.42	0	0	0	Repressed
1489	GO_ETHANOL_OXIDATION	-1.53	1.41	0	0	0	Repressed
1490	MARIADASON_RESPONSE_TO_CURCUMIN_SULINDAC_5	-1.53	1.41	0	0	0	Repressed
1491	GO_ALDITOL_METABOLIC_PROCESS	-1.53	1.40	0	0	0	Repressed
1492	GO_MUSCLE_FILAMENT_SLIDING	-1.53	1.40	0	0	0	Repressed
1493	GO_REGULATION_OF_SUBSTRATE_ADHESION_DEPENDENT_CELL_SPRE	-1.53	1.40	0	0	0	Repressed
1494	GO_MITOCHONDRIAL_RESPIRATORY_CHAIN_COMPLEX_IV	-1.53	1.38	0	0	0	Repressed
1495	BIOCARTA_ARENRF2_PATHWAY	-1.53	1.36	0	0	0	Repressed
1496	GO_AMP_BINDING	-1.53	1.36	0	0	0	Repressed
1497	GO_NEGATIVE_REGULATION_OF_PEPTIDE_HORMONE_SECRETION	-1.53	1.36	0	0	0	Repressed
1498	KIM_HYPOXIA	-1.53	1.36	0	0	0	Repressed
1499	REACTOME_PROSTACYCLIN_SIGNALLING_THROUGH_PROSTACYCLIN_RI	-1.53	1.36	0	0	0	Repressed
1500	GO_POSITIVE_REGULATION_OF_VESICLE_FUSION	-1.53	1.34	0	0	0	Repressed
1501	GO_MITOCHONDRIAL_TRNA_METHYLATION	-1.53	1.33	0	0	0	Repressed
1502	GO_REGULATION_OF_RYANODINE_SENSITIVE_CALCIUM_RELEASE_CHAN	-1.53	1.32	0	0	0	Repressed
1503	NIELSEN_GIST_VS_SYNOVIAL_SARCOMA_DN	-1.53	1.32	0	0	0	Repressed
1504	GO_TRANSLATION_TERMINATION_FACTOR_ACTIVITY	-1.53	1.31	0	0	0	Repressed
1505	REACTOME_TNFS_BIND_THEIR_PHYSIOLOGICAL_RECEPTORS	-1.53	1.31	0	0	0	Repressed
1506	GO_ACYLGLYCEROL_HOMEOSTASIS	-1.53	1.29	0	0	0	Repressed
1507	YIH_RESPONSE_TO_ARSENITE_C1	-1.53	1.29	0	0	0	Repressed
1508	GO_BASE_CONVERSION_OR_SUBSTITUTION_EDITING	-1.53	1.28	0	0	0	Repressed
1509	GO_T_CELL_EXTRAVASATION	-1.53	1.27	0	0	0	Repressed
1510	LANDIS_ERBB2_BREAST_PRENEOPLASTIC_UP	-1.53	1.27	0	0	0	Repressed
1511	GO_SMN_COMPLEX	-1.53	1.25	0	0	0	Repressed
1512	GO_NEGATIVE_REGULATION_OF_CELLULAR_RESPONSE_TO_DRUG	-1.53	1.23	0	0	0	Repressed
1513	MCCLUNG_DELTA_FOSB_TARGETS_2WK	-1.53	1.22	0	0	0	Repressed
1514	LE_NEURONAL_DIFFERENTIATION_DN	-1.53	1.19	0	0	0	Repressed

1515	GO_RIBONUCLEOPROTEIN_COMPLEX	-1.52	4.00	0	0	0	Repressed
1516	GOBERT_OLIGODENDROCYTE_DIFFERENTIATION_UP	-1.52	4.00	0	0	0	Repressed
1517	RHEIN_ALL_GLUCOCORTICOID_THERAPY_DN	-1.52	4.00	0	0	0	Repressed
1518	MYC_UP.V1_UP	-1.52	2.49	0	0	0	Repressed
1519	FARMER_BREAST_CANCER_BASAL_VS_LULMINAL	-1.52	2.38	0	0	0	Repressed
1520	GO_OXIDOREDUCTASE_ACTIVITY_ACTING_ON_A_SULFUR_GROUP_OF_D	-1.52	1.99	0	0	0	Repressed
1521	GO_HEAT_SHOCK_PROTEIN_BINDING	-1.52	1.95	0	0	0	Repressed
1522	GO_PEPTIDASE_REGULATOR_ACTIVITY	-1.52	1.95	0	0	0	Repressed
1523	GO_NON_CANONICAL_WNT_SIGNALING_PATHWAY	-1.52	1.91	0	0	0	Repressed
1524	OUELLET_CULTURED_OVARIAN_CANCER_INVASIVE_VS_LMP_UP	-1.52	1.88	0	0	0	Repressed
1525	GO_REGULATION_OF_CHROMOSOME_SEPARATION	-1.52	1.86	0	0	0	Repressed
1526	DAZARD_UV_RESPONSE_CLUSTER_G1	-1.52	1.73	0	0	0	Repressed
1527	GO_ALPHA_AMINO_ACID_BIOSYNTHETIC_PROCESS	-1.52	1.73	0	0	0	Repressed
1528	KEGG_PEROXISOME	-1.52	1.73	0	0	0	Repressed
1529	ACOSTA_PROLIFERATION_INDEPENDENT_MYC_TARGETS_UP	-1.52	1.71	0	1	0	Repressed
1530	GO_NEGATIVE_REGULATION_OF_RESPONSE_TO_WOUNDING	-1.52	1.69	0	0	0	Repressed
1531	PID_AURORA_A_PATHWAY	-1.52	1.65	0	0	0	Repressed
1532	BERENJENO_ROCK_SIGNALING_NOT_VIA_RHOA_DN	-1.52	1.51	0	0	0	Repressed
1533	GO_MAINTENANCE_OF_DNA_REPEAT_ELEMENTS	-1.52	1.48	0	0	0	Repressed
1534	GO_RELAXATION_OF_SMOOTH_MUSCLE	-1.52	1.44	0	0	0	Repressed
1535	TSUNODA_CISPLATIN_RESISTANCE_DN	-1.52	1.43	0	0	0	Repressed
1536	KEGG_SELENOAMINO_ACID_METABOLISM	-1.52	1.39	0	0	0	Repressed
1537	GO_REGULATION_OF_PANCREATIC_JUICE_SECRETION	-1.52	1.37	0	0	0	Repressed
1538	GO_NEGATIVE_REGULATION_OF_INTERLEUKIN_8_SECRETION	-1.52	1.36	0	0	0	Repressed
1539	CREIGHTON_AKT1_SIGNALING_VIA_MTOR_DN	-1.52	1.35	0	0	0	Repressed
1540	NAKAJIMA_EOSINOPHIL	-1.52	1.35	0	0	0	Repressed
1541	GO_CAMP_DEPENDENT_PROTEIN_KINASE_INHIBITOR_ACTIVITY	-1.52	1.34	0	0	0	Repressed
1542	BAFNA_MUC4_TARGETS_UP	-1.52	1.28	0	0	0	Repressed
1543	GO_HETEROTRIMERIC_G_PROTEIN_COMPLEX	-1.52	1.28	0	0	0	Repressed
1544	GO_NUCLEOLAR_LARGE_RRNA_TRANSCRIPTION_BY_RNA_POLYMERASE	-1.52	1.26	0	0	0	Repressed
1545	GO_IMMUNOLOGICAL_SYNAPSE_FORMATION	-1.52	1.24	0	0	0	Repressed
1546	GO_REGULATION_OF_RIBONUCLEASE_ACTIVITY	-1.52	1.24	0	0	0	Repressed
1547	GO_S100_PROTEIN_BINDING	-1.52	1.23	0	0	0	Repressed
1548	GO_PROTEIN_FOLDING_IN_ENDOPLASMIC_RETICULUM	-1.52	1.20	0	0	0	Repressed
1549	SMID_BREAST_CANCER_RELAPSE_IN_LUNG_UP	-1.52	1.19	0	0	0	Repressed
1550	GO_CELL_CYCLE_PHASE_TRANSITION	-1.51	4.00	0	0	0	Repressed
1551	GO_CIRCULATORY_SYSTEM_PROCESS	-1.51	4.00	0	0	0	Repressed
1552	GO_REGULATION_OF_GENERATION_OF_PRECURSOR_METABOLITES_AN	-1.51	4.00	0	0	0	Repressed
1553	HOSHIDA_LIVER_CANCER_SUBCLASS_S1	-1.51	4.00	0	0	0	Repressed
1554	GO_MODIFICATION_OF_MORPHOLOGY_OR_PHYSIOLOGY_OF_OTHER_C	-1.51	2.53	0	0	0	Repressed
1555	GO_CELLULAR_RESPONSE_TO_BIOTIC_STIMULUS	-1.51	2.49	0	0	0	Repressed
1556	GO_MODIFICATION_OF_MORPHOLOGY_OR_PHYSIOLOGY_OF_OTHER_C	-1.51	2.22	0	0	0	Repressed
1557	GO_SKELETAL_MUSCLE_ORGAN_DEVELOPMENT	-1.51	2.22	0	0	0	Repressed
1558	HELLER_SILENCED_BY_METHYLATION_UP	-1.51	2.17	0	0	0	Repressed
1559	SMIRNOV_CIRCULATING_ENDOTHELIOCYTES_IN_CANCER_UP	-1.51	2.04	0	0	0	Repressed
1560	GO_CELLULAR_GLUCOSE_HOMEOSTASIS	-1.51	2.03	0	0	0	Repressed
1561	GO_PLATELET_DEGRANULATION	-1.51	1.97	0	0	0	Repressed
1562	MIKKELSEN_ES_LCP_WITH_H3K4ME3	-1.51	1.95	0	0	0	Repressed
1563	ATF2_S_UP.V1_UP	-1.51	1.83	0	0	0	Repressed
1564	BHATTACHARYA_EMBRYONIC_STEM_CELL	-1.51	1.76	0	0	0	Repressed
1565	PETROVA_ENDOTHELIUM_LYMPHATIC_VS_BLOOD_UP	-1.51	1.73	0	0	0	Repressed
1566	GO_REGULATION_OF_PROTEIN_COMPLEX_DISASSEMBLY	-1.51	1.70	0	0	0	Repressed
1567	GROSS_HYPOXIA_VIA_HIF1A_UP	-1.51	1.56	0	0	0	Repressed
1568	GO_IMP_BIOSYNTHETIC_PROCESS	-1.51	1.52	0	0	0	Repressed
1569	DELPUECH_FOXO3_TARGETS_DN	-1.51	1.50	0	0	0	Repressed
1570	PID_PI3KCI_AKT_PATHWAY	-1.51	1.50	0	0	0	Repressed
1571	REACTOME_PHASE_II_CONJUGATION_OF_COMPOUNDS	-1.51	1.47	0	0	0	Repressed
1572	ASTIER_INTEGRIN_SIGNALING	-1.51	1.43	0	0	0	Repressed
1573	REACTOME_PROCESSING_OF_INTRONLESS_PRE_MRNAS	-1.51	1.41	0	0	0	Repressed
1574	GO_GLYCINE_BINDING	-1.51	1.40	0	0	0	Repressed
1575	GO_POSTTRANSLATIONAL_PROTEIN_TARGETING_TO_MEMBRANE_TRAN	-1.51	1.38	0	0	0	Repressed
1576	GO_SARCOPLASMIC_RETICULUM_MEMBRANE	-1.51	1.37	0	0	0	Repressed
1577	REACTOME_INTERACTIONS_OF_VPR_WITH_HOST_CELLULAR_PROTEINS	-1.51	1.36	0	0	0	Repressed
1578	MCMURRAY_TP53_HRAS_COOPERATION_RESPONSE_DN	-1.51	1.34	0	0	0	Repressed
1579	GO_POTASSIUM_TRANSPORTING_ATPASE_ACTIVITY	-1.51	1.33	0	0	0	Repressed
1580	CAMPS_COLON_CANCER_COPY_NUMBER_DN	-1.51	1.31	0	0	0	Repressed
1581	GO_PROTEIN_PHOSPHATASE_TYPE_2A_COMPLEX	-1.51	1.30	0	0	0	Repressed
1582	AMIT_EGF_RESPONSE_40_MCF10A	-1.51	1.29	0	0	0	Repressed
1583	GO_RESPONSE_TO_THYROID_HORMONE	-1.51	1.29	0	0	0	Repressed
1584	GO_PROTEASOME_CORE_COMPLEX_BETA_SUBUNIT_COMPLEX	-1.51	1.28	0	0	0	Repressed
1585	GO_VESICLE_CARGO_LOADING	-1.51	1.28	0	0	0	Repressed
1586	PID_RANBP2_PATHWAY	-1.51	1.28	0	0	0	Repressed
1587	KERLEY_RESPONSE_TO_CISPLATIN_UP	-1.51	1.27	0	0	0	Repressed
1588	GO_PROTEIN_LIPOYLATION	-1.51	1.26	0	0	0	Repressed
1589	GO_REGULATION_OF_UBIQUITIN_PROTEIN_LIGASE_ACTIVITY	-1.51	1.24	0	0	0	Repressed
1590	GO_CARBOHYDRATE_PHOSPHATASE_ACTIVITY	-1.51	1.23	0	0	0	Repressed
1591	XU_RESPONSE_TO_TRETINOIN_DN	-1.51	1.22	0	0	0	Repressed
1592	GO_LEUCINE_BINDING	-1.51	1.20	0	0	0	Repressed
1593	GO_OLIGOPEPTIDE_BINDING	-1.51	1.18	0	0	0	Repressed
1594	REACTOME_NEF_MEDIATED_CD4_DOWN_REGULATION	-1.51	1.16	0	0	0	Repressed
1595	KARAKAS_TGFB1_SIGNALING	-1.51	1.15	0	0	0	Repressed
1596	GO_ANATOMICAL_STRUCTURE_HOMEOSTASIS	-1.50	4.00	0	0	0	Repressed
1597	GO_CATALYTIC_COMPLEX	-1.50	4.00	0	0	0	Repressed
1598	GO_CELLULAR_RESPONSE_TO_OXIDATIVE_STRESS	-1.50	4.00	0	0	0	Repressed
1599	GO_COENZYME_BINDING	-1.50	4.00	0	0	0	Repressed
1600	GO_GTPASE_ACTIVITY	-1.50	4.00	0	0	0	Repressed
1601	GO_MITOCHONDRIAL_TRANSPORT	-1.50	4.00	0	0	0	Repressed
1602	GO_SUPRAMOLECULAR_COMPLEX	-1.50	4.00	0	0	0	Repressed
1603	KIM_ALL_DISORDERS_OLIGODENDROCYTE_NUMBER_CORR_UP	-1.50	4.00	0	0	0	Repressed
1604	SPIELMAN_LYMPHOBLAST_EUROPEAN_VS_ASIAN_DN	-1.50	4.00	0	0	0	Repressed
1605	GO_TUMOR_NECROSIS_FACTOR_MEDIATED_SIGNALING_PATHWAY	-1.50	2.49	0	0	0	Repressed
1606	REACTOME_ER_TO_GOLGI_ANTEROGRADE_TRANSPORT	-1.50	2.48	0	0	0	Repressed
1607	PGF_UP.V1_UP	-1.50	2.46	0	0	0	Repressed
1608	GO_CELLULAR_RESPONSE_TO_XENOBIOTIC_STIMULUS	-1.50	2.22	0	0	0	Repressed
1609	BOYLAN_MULTIPLE_MYELOMA_C_D_UP	-1.50	2.21	0	0	0	Repressed
1610	REACTOME_MITOTIC_G1_G1_S_PHASES	-1.50	2.18	0	0	0	Repressed
1611	GO_U2_TYPE_SPLICEOSOMAL_COMPLEX	-1.50	1.93	0	0	0	Repressed
1612	CAIRO_LIVER_DEVELOPMENT_DN	-1.50	1.90	0	0	0	Repressed
1613	REACTOME_DUAL_INCISION_IN_TC_NER	-1.50	1.77	0	0	0	Repressed
1614	KAYO_CALORIE_RESTRICTION_MUSCLE_UP	-1.50	1.76	0	0	0	Repressed
1615	GO_LIPID_OXIDATION	-1.50	1.71	0	0	0	Repressed
1616	MUNSHI_MULTIPLE_MYELOMA_UP	-1.50	1.71	0	0	0	Repressed
1617	SAKAI_CHRONIC_HEPATITIS_VS_LIVER_CANCER_UP	-1.50	1.69	0	0	0	Repressed
1618	REACTOME_HIV_ELONGATION_ARREST_AND_RECOVERY	-1.50	1.67	0	0	0	Repressed
1619	GO_PROTEIN_SUMOYLATION	-1.50	1.66	0	0	0	Repressed
1620	REACTOME_FORMATION_OF_TC_NER_PRE_INCISION_COMPLEX	-1.50	1.60	0	0	0	Repressed
1621	GO_NEGATIVE_REGULATION_OF_VASCULATURE_DEVELOPMENT	-1.50	1.59	0	0	0	Repressed
1622	GO_THIOESTER_BIOSYNTHETIC_PROCESS	-1.50	1.49	0	0	0	Repressed
1623	LINDSTEDT_DENDRITIC_CELL_MATURATION_A	-1.50	1.46	0	0	0	Repressed

1624	GO_FACE_DEVELOPMENT	-1.50	1.45	0	0	0	Repressed
1625	GO_REGULATION_OF_GLYCOPROTEIN_METABOLIC_PROCESS	-1.50	1.45	0	0	0	Repressed
1626	PLASARI_TGFB1_TARGETS_1HR_UP	-1.50	1.45	0	0	0	Repressed
1627	GO_POSITIVE_REGULATION_OF_VASCULAR_ENDOTHELIAL_GROWTH_F/	-1.50	1.42	0	0	0	Repressed
1628	GO_INTRAMOLECULAR_TRANSFERASE_ACTIVITY	-1.50	1.38	0	0	0	Repressed
1629	GO_RAN_GTPASE_BINDING	-1.50	1.37	0	0	0	Repressed
1630	WU_SILENCED_BY_METHYLATION_IN_BLADDER_CANCER	-1.50	1.37	0	0	0	Repressed
1631	GO_RIBONUCLEASE_MRP_COMPLEX	-1.50	1.36	0	0	0	Repressed
1632	CHEOK_RESPONSE_TO_MERCAPTOPURINE_DN	-1.50	1.31	0	0	0	Repressed
1633	GO_POSITIVE_REGULATION_OF_MYELOID_CELL_APOPTOTIC_PROCESS	-1.50	1.30	0	0	0	Repressed
1634	HALLMARK_PANCREAS_BETA_CELLS	-1.50	1.29	0	0	0	Repressed
1635	GO POSITIVE REGULATION OF ESTABLISHMENT OF PROTEIN LOCALI	-1.50	1.28	0	0	0	Repressed
1636	ZEMBUTSU_SENSITIVITY_TO_DOXORUBICIN	-1.50	1.28	0	0	0	Repressed
1637	FARMER_BREAST_CANCER_CLUSTER_7	-1.50	1.27	0	0	0	Repressed
1638	BOYAULT_LIVER_CANCER_SUBCLASS_G12_DN	-1.50	1.26	0	0	0	Repressed
1639	GO_ACID_THIOL_LIGASE_ACTIVITY	-1.50	1.26	0	0	0	Repressed
1640	GO_REGULATION_OF_TRANSCRIPTION_BY_RNA_POLYMERASE_I	-1.50	1.26	0	0	0	Repressed
1641	PID_IL12_STAT4_PATHWAY	-1.50	1.26	0	0	0	Repressed
1642	GO_REGULATION_OF_PODOSOME_ASSEMBLY	-1.50	1.22	0	0	0	Repressed
1643	GO_RESPONSE_TO_TUMOR_CELL	-1.50	1.22	0	0	0	Repressed
1644	GO_UBIQUITIN_RECYCLING	-1.50	1.21	0	0	0	Repressed
1645	GO_NLRP3_INFLAMMASOME_COMPLEX_ASSEMBLY	-1.50	1.20	0	0	0	Repressed
1646	GO_POSITIVE_REGULATION_OF_CHEMOKINE_PRODUCTION	-1.50	1.20	0	0	0	Repressed
1647	GO_PURINE_NUCLEOBASE_BIOSYNTHETIC_PROCESS	-1.50	1.20	0	0	0	Repressed
1648	GO_REGULATION_OF_HORMONE_BIOSYNTHETIC_PROCESS	-1.50	1.17	0	0	0	Repressed
1649	GO_PROTEASOME_CORE_COMPLEX_ALPHA_SUBUNIT_COMPLEX	-1.50	1.14	0	0	0	Repressed
1650	SANDERSON_PPARA_TARGETS	-1.50	1.14	0	0	0	Repressed
1651	GO ORGANIC HYDROXY COMPOUND TRANSMEMBRANE TRANSPORTEI	-1.50	1.08	0	0	0	Repressed
1652	ACEVEDO_NORMAL_TISSUE_ADJACENT_TO_LIVER_TUMOR_DN	-1.49	4.00	0	0	0	not significant
1653	FARMER_BREAST_CANCER_APOCRINE_VS_LUMINAL	-1.49	4.00	0	0	0	not significant
1654	GO_ACTIVATION_OF_INNATE_IMMUNE_RESPONSE	-1.49	4.00	0	0	0	not significant
1655	GO_ANTIGEN_RECEPTOR_MEDIATED_SIGNALING_PATHWAY	-1.49	4.00	0	0	0	not significant
1656	GO_CELLULAR_AMIDE_METABOLIC_PROCESS	-1.49	4.00	0	0	0	not significant
1657	GO_PROTEIN_CONTAINING_COMPLEX_BINDING	-1.49	4.00	0	0	0	not significant
1658	GO_RNA_SPLICING	-1.49	4.00	0	0	0	not significant
1659	REACTOME_ADAPTIVE_IMMUNE_SYSTEM	-1.49	4.00	0	0	0	not significant
1660	RUTELLA_RESPONSE_TO_HGF_VS_CSF2RB_AND_IL4_UP	-1.49	4.00	0	0	0	not significant
1661	GO_RESPONSE_TO_MECHANICAL_STIMULUS	-1.49	2.21	0	0	0	not significant
1662	GO_RESPONSE_TO_MOLECULE_OF_BACTERIAL_ORIGIN	-1.49	2.17	0	0	0	not significant
1663	GO_POSITIVE_REGULATION_OF_TRANSMEMBRANE_TRANSPORT	-1.49	2.03	0	0	0	not significant
1664	GO_RNA_POLYMERASE_COMPLEX	-1.49	2.01	0	0	0	not significant
1665	GO_RESPONSE_TO_TYPE_I_INTERFERON	-1.49	1.94	0	0	0	not significant
1666	GO_SINGLE_STRANDED_RNA_BINDING	-1.49	1.85	0	0	0	not significant
1667	ROSS_AML_OF_FAB_M7_TYPE	-1.49	1.72	0	0	0	not significant
1668	GOZGIT_ESR1_TARGETS_UP	-1.49	1.63	0	0	0	not significant
1669	LANDIS_ERBB2_BREAST_PRENEOPLASTIC_DN	-1.49	1.61	0	0	0	not significant
1670	NADERI_BREAST_CANCER_PROGNOSIS_UP	-1.49	1.57	0	0	0	not significant
1671	GO_ION_CHANNEL_REGULATOR_ACTIVITY	-1.49	1.55	0	0	0	not significant
1672	GO_MITOTIC_METAPHASE_PLATE_CONGRESSION	-1.49	1.50	0	0	0	not significant
1673	GO_RUFFLE_ORGANIZATION	-1.49	1.48	0	0	0	not significant
1674	BYSTROEM_CORRELATED_WITH_IL5_UP	-1.49	1.46	0	0	0	not significant
1675	CORDENONSI_YAP_CONSERVED_SIGNATURE	-1.49	1.44	0	0	0	not significant
1676	KRAS.LUNG_UP.V1_DN	-1.49	1.43	0	0	0	not significant
1677	GO_MITOCHONDRIAL_CALCIUM_ION_TRANSMEMBRANE_TRANSPORT	-1.49	1.42	0	0	0	not significant
1678	REACTOME_SIGNAL_AMPLIFICATION	-1.49	1.42	0	0	0	not significant
1679	GO_REGULATION_OF_ER_TO_GOLGI_VESICLE_MEDIATED_TRANSPORT	-1.49	1.41	0	0	0	not significant
1680	GO_NEGATIVE_REGULATION_OF_HORMONE_SECRETION	-1.49	1.40	0	0	0	not significant
1681	MUELLER_COMMON_TARGETS_OF_AML_FUSIONS_DN	-1.49	1.36	0	0	0	not significant
1682	GO_POSITIVE_REGULATION_OF_GLYCOPROTEIN_METABOLIC_PROCESS	-1.49	1.35	0	0	0	not significant
1683	GO_CHAPERONE_COMPLEX	-1.49	1.30	0	0	0	not significant
1684	GO_POSITIVE_REGULATION_OF_ANOIKIS	-1.49	1.30	0	0	0	not significant
1685	REACTOME FOXO MEDIATED TRANSCRIPTION OF OXIDATIVE STRESS	-1.49	1.28	0	0	0	not significant
1686	GO_PHOSPHOLIPASE_C_ACTIVITY	-1.49	1.25	0	0	0	not significant
1687	SHANK_TAL1_TARGETS_DN	-1.49	1.22	0	0	0	not significant
1688	GO_OUTER_MITOCHONDRIAL_MEMBRANE_ORGANIZATION	-1.49	1.21	0	0	0	not significant
1689	GO_RESPONSE_TO_LAMINAR_FLUID_SHEAR_STRESS	-1.49	1.21	0	0	0	not significant
1690	GO_STRUCTURAL_MOLECULE_ACTIVITY_CONFERRING_ELASTICITY	-1.49	1.21	0	0	0	not significant
1691	GO_ADENYLATE_KINASE_ACTIVITY	-1.49	1.20	0	0	0	not significant
1692	GO_ALDO_KETO_REDUCTASE_NADP_ACTIVITY	-1.49	1.20	0	0	0	not significant
1693	REACTOME_SCAVENGING_BY_CLASS_A_RECEPTORS	-1.49	1.20	0	0	0	not significant
1694	BIOCARTA_CPSF_PATHWAY	-1.49	1.17	0	0	0	not significant
1695	GO_3_PHOSPHOADENOSINE_5_PHOSPHOSULFATE_BIOSYNTHETIC_PRO	-1.49	1.16	0	0	0	not significant
1696	GO_POSITIVE_REGULATION_OF_SODIUM_ION_TRANSPORT	-1.49	1.16	0	0	0	not significant
1697	GO_REGULATION_OF_MICROVILLUS_ORGANIZATION	-1.49	1.16	0	0	0	not significant
1698	GO_POSITIVE_REGULATION_OF_CHEMOKINE_BIOSYNTHETIC_PROCESS	-1.49	1.15	0	0	0	not significant
1699	GO_CELLULAR_RESPONSE_TO_LAMINAR_FLUID_SHEAR_STRESS	-1.49	1.14	0	0	0	not significant
1700	GO_PURINE_CONTAINING_COMPOUND_SALVAGE	-1.49	1.14	0	0	0	not significant
1701	REACTOME_TRAFFICKING_AND_PROCESSING_OF_ENDOSOMAL_TLR	-1.49	1.13	0	0	0	not significant
1702	GO_PEPTIDE_CROSS_LINKING	-1.49	1.11	0	0	0	not significant
1703	BERENJENO_TRANSFORMED_BY_RHOA_FOREVER_DN	-1.49	1.06	0	0	0	not significant
1704	BUYTAERT_PHOTODYNAMIC_THERAPY_STRESS_DN	-1.48	4.00	0	0	0	not significant
1705	DEURIG_T_CELL_PROLYMPHOCYTIC_LEUKEMIA_UP	-1.48	4.00	0	0	0	not significant
1706	ENK_UV_RESPONSE_KERATINOCYTE_UP	-1.48	4.00	0	0	0	not significant
1707	GO_RESPONSE_TO_TUMOR_NECROSIS_FACTOR	-1.48	4.00	0	0	0	not significant
1708	GO_TRANSMEMBRANE_TRANSPORT	-1.48	4.00	0	0	0	not significant
1709	JAATINEN_HEMATOPOIETIC_STEM_CELL_DN	-1.48	4.00	0	0	0	not significant
1710	RUTELLA_RESPONSE_TO_HGF_UP	-1.48	4.00	0	0	0	not significant
1711	CSR_LATE_UP.V1_UP	-1.48	2.50	0	0	0	not significant
1712	GO_TELOMERE_ORGANIZATION	-1.48	2.48	0	0	0	not significant
1713	GO_ACTIN_FILAMENT_BUNDLE_ORGANIZATION	-1.48	2.23	0	0	0	not significant
1714	MCBRYAN_PUBERTAL_BREAST_4_5WK_DN	-1.48	2.20	0	0	0	not significant
1715	GO_ORGANIC_ACID_TRANSPORT	-1.48	2.19	0	0	0	not significant
1716	GO_CARBOHYDRATE_HOMEOSTASIS	-1.48	2.17	0	0	0	not significant
1717	UDAYAKUMAR_MED1_TARGETS_UP	-1.48	2.05	0	0	0	not significant
1718	GO_PIGMENT_GRANULE	-1.48	1.78	0	0	0	not significant
1719	KEGG_PROGESTERONE_MEDIATED_OOCYTE_MATURATION	-1.48	1.77	0	0	0	not significant
1720	GO_LONG_CHAIN_FATTY_ACID_TRANSPORT	-1.48	1.64	0	0	0	not significant
1721	MOHANKUMAR_HOXA1_TARGETS_DN	-1.48	1.61	0	0	0	not significant
1722	GO_CELLULAR_AMINO_ACID_BIOSYNTHETIC_PROCESS	-1.48	1.60	0	0	0	not significant
1723	BOYAULT_LIVER_CANCER_SUBCLASS_G1_DN	-1.48	1.55	0	0	0	not significant
1724	GO_REGULATION_OF_ALCOHOL_BIOSYNTHETIC_PROCESS	-1.48	1.55	0	0	0	not significant
1725	GO_RESPONSE_TO_CAMP	-1.48	1.55	0	0	0	not significant
1726	KEGG_PYRUVATE_METABOLISM	-1.48	1.54	0	0	0	not significant
1727	KEGG_VALINE_LEUCINE_AND_ISOLEUCINE_DEGRADATION	-1.48	1.53	0	0	0	not significant
1728	RUAN_RESPONSE_TO_TNF_DN	-1.48	1.50	0	0	0	not significant
1729	PENG_GLUCOSE_DEPRIVATION_UP	-1.48	1.43	0	0	0	not significant
1730	GO_TELOMERASE_HOLOENZYME_COMPLEX	-1.48	1.29	0	0	0	not significant
1731	PETRETTO_BLOOD_PRESSURE_UP	-1.48	1.27	0	0	0	not significant
1732	GO_PYRIMIDINE_RIBONUCLEOSIDE_CATABOLIC_PROCESS	-1.48	1.26	0	0	0	not significant

1733	PETRETTO_CARDIAC_HYPERTROPHY	-1.48	1.26	0	0	0	not significant
1734	GO_CYSTEINE_TYPE_ENDOPEPTIDASE_INHIBITOR_ACTIVITY	-1.48	1.25	0	0	0	not significant
1735	GO_MULTICELLULAR_ORGANISM_AGING	-1.48	1.24	0	0	0	not significant
1736	GO_NEGATIVE_REGULATION_OF_PROTEIN_LOCALIZATION_TO_CELL_PE	-1.48	1.22	0	0	0	not significant
1737	GO_SNRNA_3_END_PROCESSING	-1.48	1.22	0	0	0	not significant
1738	GO_GLUTAMINE_FAMILY_AMINO_ACID_BIOSYNTHETIC_PROCESS	-1.48	1.21	0	0	0	not significant
1739	MCBRYAN_TERMINAL_END_BUD_UP	-1.48	1.20	0	0	0	not significant
1740	GO_HEART_FORMATION	-1.48	1.16	0	0	0	not significant
1741	GO_PROTEIN_LOCALIZATION_TO_NUCLEOLUS	-1.48	1.16	0	0	0	not significant
1742	GO_T_HELPER_1_CELL_DIFFERENTIATION	-1.48	1.14	0	0	0	not significant
1743	YANG_MUC2_TARGETS_DUODENUM_6MO_DN	-1.48	1.13	0	0	0	not significant
1744	GO_REGULATION_OF_RECEPTOR_CATABOLIC_PROCESS	-1.48	1.10	0	0	0	not significant
1745	GO POSITIVE REGULATION OF T HELPER 1 TYPE IMMUNE RESPONS	-1.48	1.06	0	0	0	not significant
1746	GAL_LEUKEMIC_STEM_CELL_DN	-1.47	4.00	0	0	0	not significant
1747	GO_INNATE_IMMUNE_RESPONSE	-1.47	4.00	0	0	0	not significant
1748	GO_REGULATION_OF_ION_TRANSMEMBRANE_TRANSPORT	-1.47	4.00	0	0	0	not significant
1749	GO_REGULATION_OF_LIPID_METABOLIC_PROCESS	-1.47	4.00	0	0	0	not significant
1750	GO_RESPONSE_TO_DRUG	-1.47	4.00	0	0	0	not significant
1751	MILI_PSEUDOPODIA_HAPTOTAXIS_UP	-1.47	4.00	0	0	0	not significant
1752	ONDER_CDH1_TARGETS_2_DN	-1.47	4.00	0	0	0	not significant
1753	REACTOME_INNATE_IMMUNE_SYSTEM	-1.47	4.00	0	0	0	not significant
1754	GO_REGULATION_OF_VASCULATURE_DEVELOPMENT	-1.47	2.44	0	0	0	not significant
1755	CAMP_UP.V1_UP	-1.47	2.43	0	0	0	not significant
1756	MCLACHLAN_DENTAL_CARIES_UP	-1.47	2.21	0	0	0	not significant
1757	PID_ERBB1_DOWNSTREAM_PATHWAY	-1.47	2.02	0	0	0	not significant
1758	SHEPARD_BMYB_MORPHOLINO_DN	-1.47	2.02	0	0	0	not significant
1759	REACTOME_SIGNALING_BY_NUCLEAR_RECEPTORS	-1.47	1.96	0	0	0	not significant
1760	GO_PROTON_TRANSMEMBRANE_TRANSPORT	-1.47	1.89	0	0	0	not significant
1761	WILCOX_RESPONSE_TO_PROGESTERONE_UP	-1.47	1.89	0	0	0	not significant
1762	WOOD_EBV_EBNA1_TARGETS_UP	-1.47	1.75	0	0	0	not significant
1763	GO_MONOSACCHARIDE_BIOSYNTHETIC_PROCESS	-1.47	1.70	0	0	0	not significant
1764	GO_CARBON_CARBON_LYASE_ACTIVITY	-1.47	1.57	0	0	0	not significant
1765	VANTVEER_BREAST_CANCER_BRCA1_UP	-1.47	1.57	0	0	0	not significant
1766	GO_NUCLEAR_ENVELOPE_ORGANIZATION	-1.47	1.55	0	0	0	not significant
1767	ZHOU_TNF_SIGNALING_30MIN	-1.47	1.55	0	0	0	not significant
1768	ZHAN_MULTIPLE_MYELOMA_CD1_UP	-1.47	1.49	0	0	0	not significant
1769	GRAESSMANN_RESPONSE_TO_MC_AND_SERUM_DEPRIVATION_DN	-1.47	1.46	0	0	0	not significant
1770	REACTOME_FATTY_ACYL_COA_BIOSYNTHESIS	-1.47	1.46	0	0	0	not significant
1771	REACTOME_RMTS_METHYLATE_HISTONE_ARGININES	-1.47	1.46	0	0	0	not significant
1772	GO_ENDONUCLEASE_COMPLEX	-1.47	1.38	0	0	0	not significant
1773	GO_REGULATORY_RNA_BINDING	-1.47	1.36	0	0	0	not significant
1774	GO_CHANNEL_INHIBITOR_ACTIVITY	-1.47	1.35	0	0	0	not significant
1775	LEE_LIVER_CANCER_MYC_E2F1_UP	-1.47	1.35	0	0	0	not significant
1776	GO_UNSATURATED_FATTY_ACID_BIOSYNTHETIC_PROCESS	-1.47	1.33	0	0	0	not significant
1777	LY_AGING_PREMATURE_DN	-1.47	1.32	0	0	0	not significant
1778	GO_RESPONSE_TO_DSRNA	-1.47	1.29	0	0	0	not significant
1779	GO_TRNA_SPECIFIC_RIBONUCLEASE_ACTIVITY	-1.47	1.29	0	0	0	not significant
1780	GO_RNA_POLYMERASE_II_CORE_COMPLEX	-1.47	1.25	0	0	0	not significant
1781	GO_CELLULAR_RESPONSE_TO_GONADOTROPIN_STIMULUS	-1.47	1.24	0	0	0	not significant
1782	GO_RESPONSE_TO_IRON_III_ION	-1.47	1.24	0	0	0	not significant
1783	GO_FIBRINOLYSIS	-1.47	1.23	0	0	0	not significant
1784	GO_LEUCINE_CATABOLIC_PROCESS	-1.47	1.23	0	0	0	not significant
1785	LIANG_SILENCED_BY_METHYLATION_UP	-1.47	1.23	0	0	0	not significant
1786	GO_ACTIN_CROSSLINK_FORMATION	-1.47	1.20	0	0	0	not significant
1787	GO_ADENOSINE_RECEPTOR_SIGNALING_PATHWAY	-1.47	1.20	0	0	0	not significant
1788	GO_ADENYLATE_CYCLASE_ACTIVATOR_ACTIVITY	-1.47	1.20	0	0	0	not significant
1789	GO_LONG_CHAIN_FATTY_ACID_TRANSPORTER_ACTIVITY	-1.47	1.20	0	0	0	not significant
1790	GO SINGLE STRANDED VIRAL RNA REPLICATION VIA DOUBLE STRAN	-1.47	1.20	0	0	0	not significant
1791	BIOCARTA_NUCLEARRS_PATHWAY	-1.47	1.17	0	0	0	not significant
1792	GO_GLYCINE_TRANSPORT	-1.47	1.16	0	0	0	not significant
1793	GO POSITIVE REGULATION OF AMYLOID PRECURSOR PROTEIN CATA	-1.47	1.16	0	0	0	not significant
1794	SCHAEFFER_SOX9_TARGETS_IN_PROSTATE_DEVELOPMENT_UP	-1.47	1.16	0	0	0	not significant
1795	REACTOME_TRNA_PROCESSING_IN_THE_MITOCHONDRION	-1.47	1.14	0	0	0	not significant
1796	GO POSITIVE REGULATION OF TUMOR NECROSIS FACTOR BIOSYNTH	-1.47	1.13	0	0	0	not significant
1797	GO_REGULATION_OF_ASTROCYTE_DIFFERENTIATION	-1.47	1.13	0	0	0	not significant
1798	ZHAN_V1_LATE_DIFFERENTIATION_GENES_DN	-1.47	1.13	0	0	0	not significant
1799	GO_FOREBRAIN_NEURON_DEVELOPMENT	-1.47	1.11	0	0	0	not significant
1800	ZERBINI_RESPONSE_TO_SULINDAC_DN	-1.47	1.11	0	0	0	not significant
1801	GO_PINOSOME	-1.47	1.08	0	0	0	not significant
1802	GO POSITIVE REGULATION OF DNA TEMPLATED TRANSCRIPTION INIT	-1.47	1.08	0	0	0	not significant
1803	YANG_MUC2_TARGETS_DUODENUM_3MO_DN	-1.47	1.08	0	0	0	not significant
1804	GO_RENAL_ABSORPTION	-1.47	1.07	0	0	0	not significant
1805	GO_MAINTENANCE_OF_GASTROINTESTINAL_EPITHELIUM	-1.47	1.06	0	0	0	not significant
1806	GO_ACTIN_CYTOSKELETON	-1.46	4.00	0	0	0	not significant
1807	GO_CELLULAR_PROTEIN_CONTAINING_COMPLEX_ASSEMBLY	-1.46	4.00	0	0	0	not significant
1808	GO_REGULATION_OF_SYSTEM_PROCESS	-1.46	4.00	0	0	0	not significant
1809	GO_RESPONSE_TO_ABIOTIC_STIMULUS	-1.46	4.00	0	0	0	not significant
1810	REACTOME_TRANSLATION	-1.46	4.00	0	0	0	not significant
1811	MEK_UP.V1_DN	-1.46	2.43	0	0	0	not significant
1812	KIM_WT1_TARGETS_DN	-1.46	2.25	0	0	0	not significant
1813	GO_CYTOSOLIC_CALCIUM_ION_TRANSPORT	-1.46	2.24	0	0	0	not significant
1814	RAMALHO_STEMNESS_UP	-1.46	2.10	0	0	0	not significant
1815	REACTOME_METABOLISM_OF_STEROIDS	-1.46	2.02	0	0	0	not significant
1816	EPPERT_PROGENITOR	-1.46	2.00	0	0	0	not significant
1817	REACTOME_AURKA_ACTIVATION_BY_TPX2	-1.46	1.94	0	0	0	not significant
1818	MACAEVA_PBMC_RESPONSE_TO_IR	-1.46	1.84	0	0	0	not significant
1819	REACTOME_CARDIAC_CONDUCTION	-1.46	1.75	0	0	0	not significant
1820	GO_I_BAND	-1.46	1.74	0	0	0	not significant
1821	REACTOME_INTERLEUKIN_12_FAMILY_SIGNALING	-1.46	1.73	0	0	0	not significant
1822	GO_REGULATION_OF_OXIDOREDUCTASE_ACTIVITY	-1.46	1.68	0	0	0	not significant
1823	ADDYA_ERYTHROID_DIFFERENTIATION_BY_HEMIN	-1.46	1.63	0	0	0	not significant
1824	REACTOME_TRANSCRIPTIONAL_ACTIVATION_OF_MITOCHONDRIAL_BIOG	-1.46	1.63	0	0	0	not significant
1825	BROWNE_HCMV_INFECTION_20HR_DN	-1.46	1.60	0	0	0	not significant
1826	GO_MUSCLE_CELL_DEVELOPMENT	-1.46	1.57	0	0	0	not significant
1827	GO_ORGANIC_ACID_TRANSMEMBRANE_TRANSPORT	-1.46	1.57	0	0	0	not significant
1828	GO_RESPONSE_TO_FATTY_ACID	-1.46	1.53	0	0	0	not significant
1829	HOFFMANN_PRE_BI_TO_LARGE_PRE_BII_LYMPHOCYTE_DN	-1.46	1.48	0	0	0	not significant
1830	GO_CELLULAR_RESPONSE_TO_MECHANICAL_STIMULUS	-1.46	1.44	0	0	0	not significant
1831	NAKAMURA_METASTASIS_MODEL_UP	-1.46	1.44	0	0	0	not significant
1832	REACTOME_MITOCHONDRIAL_FATTY_ACID_BETA_OXIDATION	-1.46	1.41	0	0	0	not significant
1833	PID_BCR_5PATHWAY	-1.46	1.40	0	0	0	not significant
1834	REACTOME_GPVI_MEDIATED_ACTIVATION_CASCADE	-1.46	1.33	0	0	0	not significant
1835	GRAHAM_NORMAL_QUIESCENT_VS_NORMAL_DIVIDING_UP	-1.46	1.31	0	0	0	not significant
1836	GO_CIS_TRANS_ISOMERASE_ACTIVITY	-1.46	1.30	0	0	0	not significant
1837	KEGG_CYSTEINE_AND_METHIONINE_METABOLISM	-1.56	1.30	1	0	0	Repressed
1838	GENTILE_UV_RESPONSE_CLUSTER_D5	-1.46	1.29	0	0	0	not significant
1839	GO_SODIUM_POTASSIUM_EXCHANGING_ATPASE_COMPLEX	-1.46	1.29	0	0	0	not significant
1840	AMIT_EGF_RESPONSE_60_HELA	-1.46	1.27	0	0	0	not significant
1841	GO_SMALL_NUCLEOLAR_RIBONUCLEOPROTEIN_COMPLEX_ASSEMBLY	-1.46	1.22	0	0	0	not significant

1842	REACTOME_PKA_ACTIVATION_IN_GLUCAGON_SIGNALLING	-1.46	1.21	0	0	0	not significant
1843	GO_POSITIVE_REGULATION_OF_ASTROCYTE_DIFFERENTIATION	-1.46	1.19	0	0	0	not significant
1844	GO_EXON_EXON_JUNCTION_COMPLEX	-1.46	1.18	0	0	0	not significant
1845	GO_QUINONE_BIOSYNTHETIC_PROCESS	-1.46	1.18	0	0	0	not significant
1846	MARCHINI_TRABECTEDIN_RESISTANCE_UP	-1.46	1.18	0	0	0	not significant
1847	STAMBOLSKY_BOUND_BY_MUTATED_TP53	-1.46	1.18	0	0	0	not significant
1848	GO_FATTY_ACYL_COA_BIOSYNTHETIC_PROCESS	-1.46	1.17	0	0	0	not significant
1849	GO_PWP2P_CONTAINING_SUBCOMPLEX_OF_90S_PRERIBOSOME	-1.46	1.16	0	0	0	not significant
1850	GO_DENDRITIC_TRANSPORT	-1.46	1.15	0	0	0	not significant
1851	KOBAYASHI_EGFR_SIGNALING_6HR_DN	-1.46	1.15	0	0	0	not significant
1852	REACTOME_G2_M_DNA_REPLICATION_CHECKPOINT	-1.46	1.15	0	0	0	not significant
1853	GO_CHAPERONE_MEDIATED_PROTEIN_COMPLEX_ASSEMBLY	-1.46	1.14	0	0	0	not significant
1854	GO ENDONUCLEOLYTIC CLEAVAGE IN 5 ETS OF TRICISTRONIC RRN/	-1.46	1.14	0	0	0	not significant
1855	GO_NEGATIVE_REGULATION_OF_CENTROSOME_CYCLE	-1.46	1.14	0	0	0	not significant
1856	GO_POSITIVE_REGULATION_OF_NAD_P_H_OXIDASE_ACTIVITY	-1.46	1.13	0	0	0	not significant
1857	SARTIPY_BLUNTED_BY_INSULIN_RESISTANCE_UP	-1.46	1.13	0	0	0	not significant
1858	GO_AMINOACYL_TRNA_EDITING_ACTIVITY	-1.46	1.12	0	0	0	not significant
1859	GO_POSITIVE_REGULATION_OF_MUSCLE_HYPERTROPHY	-1.46	1.12	0	0	0	not significant
1860	GO AMINOACYL TRNA METABOLISM INVOLVED IN TRANSLATIONAL FII	-1.46	1.11	0	0	0	not significant
1861	GO_NUCLEAR_EXPORT_SIGNAL_RECEPTOR_ACTIVITY	-1.46	1.11	0	0	0	not significant
1862	GO NEGATIVE REGULATION OF MULTICELLULAR ORGANISM GROWTH	-1.46	1.10	0	0	0	not significant
1863	HERNANDEZ_MITOTIC_ARREST_BY_DOCETAXEL_1_UP	-1.46	1.08	0	0	0	not significant
1864	HOWLIN_CITED1_TARGETS_2_DN	-1.46	1.08	0	0	0	not significant
1865	STEGER_ADIPOGENESIS_UP	-1.46	1.08	0	0	0	not significant
1866	GO OXIDOREDUCTASE ACTIVITY ACTING ON DIPHENOLS AND RELATI	-1.46	1.05	0	0	0	not significant
1867	DANG_BOUND_BY_MYC	-1.45	4.00	0	0	0	not significant
1868	GO_REGULATION_OF_TRANSMEMBRANE_TRANSPORT	-1.45	4.00	0	0	0	not significant
1869	KYNG_WERNER_SYNDROM_AND_NORMAL_AGING_DN	-1.45	4.00	0	0	0	not significant
1870	LOPEZ_MBD_TARGETS	-1.45	4.00	0	0	0	not significant
1871	SPIELMAN_LYMPHOBLAST_EUROPEAN_VS_ASIAN_UP	-1.45	4.00	0	0	0	not significant
1872	GO_SPLICEOSOMAL_COMPLEX	-1.45	2.40	0	0	0	not significant
1873	GO_MUSCLE_SYSTEM_PROCESS	-1.45	2.16	0	0	0	not significant
1874	GOLDRATH_HOMEOSTATIC_PROLIFERATION	-1.45	1.99	0	0	0	not significant
1875	PROVENZANI_METASTASIS_DN	-1.45	1.97	0	0	0	not significant
1876	GO_MICROBODY_PART	-1.45	1.96	0	0	0	not significant
1877	MISSIAGLIA_REGULATED_BY_METHYLATION_DN	-1.45	1.75	0	0	0	not significant
1878	CAFFAREL_RESPONSE_TO_THC_24HR_5_DN	-1.45	1.63	0	0	0	not significant
1879	FOSTER_TOLERANT_MACROPHAGE_UP	-1.45	1.62	0	0	0	not significant
1880	JAIN_NFKB_SIGNALING	-1.45	1.62	0	0	0	not significant
1881	REACTOME_RNA_POLYMERASE_I_PROMOTER_ESCAPE	-1.45	1.61	0	0	0	not significant
1882	GO_POSITIVE_REGULATION_OF_CALCIUM_ION_TRANSPORT	-1.45	1.60	0	0	0	not significant
1883	REACTOME POSITIVE EPIGENETIC REGULATION OF RRNA EXPRESSIC	-1.45	1.60	0	0	0	not significant
1884	REACTOME_FORMATION_OF_THE_BETA_CATENIN:TCF_TRANSACTIVATII	-1.45	1.57	0	0	0	not significant
1885	REACTOME_INTERFERON_ALPHA_BETA_SIGNALING	-1.45	1.57	0	0	0	not significant
1886	GO_NAD_BINDING	-1.45	1.53	0	0	0	not significant
1887	FAELT_B_CLL_WITH_VH3_21_UP	-1.45	1.46	0	0	0	not significant
1888	ABRAMSON_INTERACT_WITH_AIRE	-1.45	1.44	0	0	0	not significant
1889	GO_PROTEIN_K11_LINKED_UBIQUITINATION	-1.45	1.39	0	0	0	not significant
1890	NAKAMURA_METASTASIS	-1.45	1.38	0	0	0	not significant
1891	SENGUPTA_NASOPHARYNGEAL_CARCINOMA_WITH_LMP1_DN	-1.45	1.38	0	0	0	not significant
1892	GO_MACROMOLECULE_TRANSMEMBRANE_TRANSPORTER_ACTIVITY	-1.45	1.36	0	0	0	not significant
1893	GO_ESTABLISHMENT_OF_SPINDLE_ORIENTATION	-1.45	1.35	0	0	0	not significant
1894	JIANG_AGING_HYPOTHALAMUS_UP	-1.45	1.35	0	0	0	not significant
1895	REACTOME_NUCLEAR_RECEPTOR_TRANSCRIPTION_PATHWAY	-1.45	1.31	0	0	0	not significant
1896	ROY_WOUND_BLOOD_VESSEL_UP	-1.45	1.27	0	0	0	not significant
1897	GO_FAD_BINDING	-1.45	1.26	0	0	0	not significant
1898	REACTOME_MET_PROMOTES_CELL_MOTILITY	-1.45	1.26	0	0	0	not significant
1899	ZHAN_VARIABLE_EARLY_DIFFERENTIATION_GENES_DN	-1.45	1.26	0	0	0	not significant
1900	GO_PROTEIN_SERINE_THREONINE_KINASE_ACTIVATOR_ACTIVITY	-1.45	1.25	0	0	0	not significant
1901	GNATENKO_PLATELET_SIGNATURE	-1.45	1.24	0	0	0	not significant
1902	GO_ANTIBACTERIAL_HUMORAL_RESPONSE	-1.45	1.22	0	0	0	not significant
1903	GO_POSITIVE_REGULATION_OF_TRANSLATIONAL_INITIATION	-1.45	1.22	0	0	0	not significant
1904	GO_PYRIDINE_CONTAINING_COMPOUND_METABOLIC_PROCESS	-1.45	1.22	0	0	0	not significant
1905	GO_SULFURTRANSFERASE_ACTIVITY	-1.45	1.17	0	0	0	not significant
1906	GO_CRISTAE_FORMATION	-1.45	1.16	0	0	0	not significant
1907	GO_PROTEIN_LOCALIZATION_TO_NUCLEOPLASM	-1.45	1.16	0	0	0	not significant
1908	GO_RNA_POLYMERASE_I_ACTIVITY	-1.45	1.15	0	0	0	not significant
1909	COLLIS_PRKDC_REGULATORS	-1.45	1.14	0	0	0	not significant
1910	GO_POSITIVE_REGULATION_OF_LAMELLIPODIUM_MORPHOGENESIS	-1.45	1.12	0	0	0	not significant
1911	ZHANG_GATA6_TARGETS_UP	-1.45	1.12	0	0	0	not significant
1912	BIOCARTA_MTA3_PATHWAY	-1.45	1.11	0	0	0	not significant
1913	GO_CELLULAR_LIPID_BIOSYNTHETIC_PROCESS	-1.45	1.10	0	0	0	not significant
1914	GO_PURINE_NUCLEOTIDE_SALVAGE	-1.45	1.10	0	0	0	not significant
1915	KEGG_FOLATE_BIOSYNTHESIS	-1.45	1.10	0	0	0	not significant
1916	LEE_METASTASIS_AND_RNA_PROCESSING_UP	-1.45	1.10	0	0	0	not significant
1917	WU_HBX_TARGETS_3_DN	-1.45	1.10	0	0	0	not significant
1918	GO_INFLAMMATORY_RESPONSE_TO_WOUNDING	-1.45	1.08	0	0	0	not significant
1919	CROSBY_E2F4_TARGETS	-1.45	1.07	0	0	0	not significant
1920	KRAS.50_UP.V1_UP	-1.45	1.07	0	0	0	not significant
1921	BIOCARTA_MEF2D_PATHWAY	-1.45	1.06	0	0	0	not significant
1922	GO_PYRAMIDAL_NEURON_DIFFERENTIATION	-1.45	1.06	0	0	0	not significant
1923	GO_RESPONSE_TO_GONADOTROPIN	-1.45	1.06	0	0	0	not significant
1924	GO_RETROGRADE_AXONAL_TRANSPORT	-1.45	1.06	0	0	0	not significant
1925	REACTOME_POLO_LIKE_KINASE_MEDIATED_EVENTS	-1.45	1.05	0	0	0	not significant
1926	GO_LEUKOCYTE_ACTIVATION_INVOLVED_IN_INFLAMMATORY_RESPONSI	-1.45	1.04	0	0	0	not significant
1927	GO_PEPTIDYL_ARGININE_METHYLATION_TO_ASYMMETRICAL_DIMETHYL	-1.45	1.04	0	0	0	not significant
1928	GO EPITHELIAL TUBE BRANCHING INVOLVED IN LUNG MORPHOGENES	-1.45	1.03	0	0	0	not significant
1929	REACTOME BINDING OF TCF LEF:CTNNB1 TO TARGET GENE PROMC	-1.45	1.03	0	0	0	not significant
1930	GO_REGULATION_OF_VITAMIN_METABOLIC_PROCESS	-1.45	1.01	0	0	0	not significant
1931	YAMANAKA_GLIOBLASTOMA_SURVIVAL_UP	-1.45	1.01	0	0	0	not significant
1932	GO_HEART_TRABECULA_FORMATION	-1.45	0.99	0	0	0	not significant
1933	GO_HEPARAN_SULFATE_PROTEOGLYCAN_BINDING	-1.45	0.99	0	0	0	not significant
1934	GO_POSITIVE_REGULATION_OF_CELLULAR_RESPIRATION	-1.45	0.98	0	0	0	not significant
1935	GO_NEGATIVE_REGULATION_OF_DIGESTIVE_SYSTEM_PROCESS	-1.45	0.96	0	0	0	not significant
1936	COLINA_TARGETS_OF_4EBP1_AND_4EBP2	-1.44	4.00	0	0	0	not significant
1937	GO_AMIDE_BIOSYNTHETIC_PROCESS	-1.44	4.00	0	0	0	not significant
1938	GO_HYDROLASE_ACTIVITY_ACTING_ON_ACID_ANHYDRIDES	-1.44	4.00	0	0	0	not significant
1939	REACTOME_PTEN_REGULATION	-1.44	4.00	0	0	0	not significant
1940	KONG_E2F3_TARGETS	-1.44	2.52	0	0	0	not significant
1941	GO_ENZYME_INHIBITOR_ACTIVITY	-1.44	2.44	0	0	0	not significant
1942	IVANOVA_HEMATOPOIESIS_LATE_PROGENITOR	-1.44	2.31	0	0	0	not significant
1943	GO_RESPONSE_TO_CARBOHYDRATE	-1.44	2.19	0	0	0	not significant
1944	GO_FATTY_ACID_CATABOLIC_PROCESS	-1.44	2.07	0	0	0	not significant
1945	GO_RESPONSE_TO_XENOBIOTIC_STIMULUS	-1.44	2.02	0	0	0	not significant
1946	BROWNE_HCMV_INFECTION_14HR_UP	-1.44	1.99	0	0	0	not significant
1947	GO_NUCLEUS_ORGANIZATION	-1.44	1.90	0	0	0	not significant
1948	REACTOME_METABOLISM_OF_VITAMINS_AND_COFACTORS	-1.44	1.88	0	0	0	not significant
1949	ACEVEDO_NORMAL_TISSUE_ADJACENT_TO_LIVER_TUMOR_UP	-1.44	1.80	0	0	0	not significant
1950	GO_CALCIUM_MEDIATED_SIGNALING	-1.44	1.80	0	0	0	not significant

1951	GO_NEPHRON_DEVELOPMENT	-1.44	1.57	0	0	0	not significant
1952	MORI_MATURE_B_LYMPHOCYTE_DN	-1.44	1.56	0	0	0	not significant
1953	CRX_DN.V1_UP	-1.44	1.55	0	0	0	not significant
1954	CHR1P32	-1.44	1.46	0	0	0	not significant
1955	GO_HORMONE_BINDING	-1.44	1.44	0	0	0	not significant
1956	GO_POSITIVE_REGULATION_OF_VIRAL_TRANSCRIPTION	-1.44	1.41	0	0	0	not significant
1957	GO_REGULATION_OF_PROTEIN_DEPOLYMERIZATION	-1.44	1.39	0	0	0	not significant
1958	PID_FOXM1_PATHWAY	-1.44	1.39	0	0	0	not significant
1959	KEGG_PATHOGENIC_ESCHERICHIA_COLI_INFECTION	-1.44	1.33	0	0	0	not significant
1960	BOYAULT_LIVER_CANCER_SUBCLASS_G123_DN	-1.44	1.32	0	0	0	not significant
1961	KLEIN_PRIMARY_EFFUSION_LYMPHOMA_UP	-1.44	1.32	0	0	0	not significant
1962	CUI_GLUCOSE_DEPRIVATION	-1.44	1.31	0	0	0	not significant
1963	GO_REGULATION_OF_SISTER_CHROMATID_COHESION	-1.44	1.24	0	0	0	not significant
1964	GO_TERMINATION_OF_RNA_POLYMERASE_I_TRANSCRIPTION	-1.44	1.24	0	0	0	not significant
1965	GO_NEUROMUSCULAR_SYNAPTIC_TRANSMISSION	-1.44	1.22	0	0	0	not significant
1966	GO_CELLULAR_RESPONSE_TO_CAMP	-1.44	1.21	0	0	0	not significant
1967	GO_MATURATION_OF_LSU_RRNA	-1.44	1.21	0	0	0	not significant
1968	KEGG_GLYCINE_SERINE_AND_THREONINE_METABOLISM	-1.44	1.21	0	0	0	not significant
1969	GO_ATP_DEPENDENT_MICROTUBULE_MOTOR_ACTIVITY_PLUS_END_DIF	-1.44	1.19	0	0	0	not significant
1970	GO_GLANDULAR_EPITHELIAL_CELL_DIFFERENTIATION	-1.44	1.19	0	0	0	not significant
1971	CROMER_TUMORIGENESIS_UP	-1.44	1.18	0	0	0	not significant
1972	GO_ANAPHASE_PROMOTING_COMPLEX	-1.44	1.18	0	0	0	not significant
1973	HAHTOLA_MYCOSIS_FUNGOIDES_SKIN_DN	-1.44	1.18	0	0	0	not significant
1974	GO_MONOCARBOXYLIC_ACID_BINDING	-1.44	1.17	0	0	0	not significant
1975	GO_KETONE_BIOSYNTHETIC_PROCESS	-1.44	1.16	0	0	0	not significant
1976	KEGG_PORPHYRIN_AND_CHLOROPHYLL_METABOLISM	-1.44	1.15	0	0	0	not significant
1977	REACTOME_CHOLESTEROL_BIOSYNTHESIS	-1.44	1.15	0	0	0	not significant
1978	GO_REGULATION_OF_LYASE_ACTIVITY	-1.44	1.14	0	0	0	not significant
1979	GO_REGULATION_OF_SYSTEMIC_ARTERIAL_BLOOD_PRESSURE_BY_HO	-1.44	1.14	0	0	0	not significant
1980	GO_NEGATIVE_REGULATION_OF_MACROPHAGE_DERIVED_FOAM_CELL_	-1.44	1.12	0	0	0	not significant
1981	GO_POSITIVE_REGULATION_OF_NOTCH_SIGNALING_PATHWAY	-1.44	1.12	0	0	0	not significant
1982	WANG_RESPONSE_TO_FORSKOLIN_UP	-1.44	1.12	0	0	0	not significant
1983	GO_REGULATION_OF_VOLTAGE_GATED_CALCIUM_CHANNEL_ACTIVITY	-1.44	1.11	0	0	0	not significant
1984	GO_CARDIAC_MUSCLE_CELL_MEMBRANE_REPOLARIZATION	-1.44	1.08	0	0	0	not significant
1985	GO_CRANIAL_NERVE_MORPHOGENESIS	-1.44	1.08	0	0	0	not significant
1986	GO_INNATE_IMMUNE_RESPONSE_IN_MUCOSA	-1.44	1.08	0	0	0	not significant
1987	MURAKAMI_UV_RESPONSE_24HR	-1.44	1.07	0	0	0	not significant
1988	REACTOME_BETA_OXIDATION_OF_VERY_LONG_CHAIN_FATTY_ACIDS	-1.44	1.07	0	0	0	not significant
1989	GO_POSITIVE_REGULATION_OF_MRNA_3_END_PROCESSING	-1.44	1.06	0	0	0	not significant
1990	GO_PROTEIN_INSERTION_INTO_MITOCHONDRIAL_OUTER_MEMBRANE	-1.44	1.04	0	0	0	not significant
1991	REACTOME_PURINE_SALVAGE	-1.44	1.04	0	0	0	not significant
1992	GO_CYTIDINE_TO_URIDINE_EDITING	-1.44	1.03	0	0	0	not significant
1993	GO_EMBRYONIC_CLEAVAGE	-1.44	1.02	0	0	0	not significant
1994	GO_TROPHECTODERMAL_CELL_DIFFERENTIATION	-1.44	1.01	0	0	0	not significant
1995	GO_POSITIVE_REGULATION_OF_NUCLEOBASE_CONTAINING_COMPOUNI	-1.44	0.98	0	0	0	not significant
1996	GO_COFACTOR_BINDING	-1.43	4.00	0	0	0	not significant
1997	GO_NUCLEOSIDE_PHOSPHATE_BIOSYNTHETIC_PROCESS	-1.43	4.00	0	0	0	not significant
1998	GO_PROTEIN_CONTAINING_COMPLEX_ASSEMBLY	-1.43	4.00	0	0	0	not significant
1999	GO_PROTEIN_CONTAINING_COMPLEX_LOCALIZATION	-1.43	4.00	0	0	0	not significant
2000	GO_REGULATION_OF_ION_TRANSPORT	-1.43	4.00	0	0	0	not significant
2001	ONKEN_UVEAL_MELANOMA_UP	-1.43	4.00	0	0	0	not significant
2002	STEIN_ESRRA_TARGETS	-1.43	4.00	0	0	0	not significant
2003	YOSHIMURA_MAPK8_TARGETS_DN	-1.43	4.00	0	0	0	not significant
2004	DAVICIONI_TARGETS_OF_PAX_FOXO1_FUSIONS_UP	-1.43	2.46	0	0	0	not significant
2005	IL2_UP.V1_UP	-1.43	2.18	0	0	0	not significant
2006	REACTOME_SIGNALING_BY_NOTCH	-1.43	1.98	0	0	0	not significant
2007	GO_PROTEIN_HETEROOLIGOMERIZATION	-1.43	1.92	0	0	0	not significant
2008	REACTOME_ESTROGEN_DEPENDENT_GENE_EXPRESSION	-1.43	1.89	0	0	0	not significant
2009	REACTOME_NUCLEOTIDE_EXCISION_REPAIR	-1.43	1.88	0	0	0	not significant
2010	CHIARADONNA_NEOPLASTIC_TRANSFORMATION_CDC25_DN	-1.43	1.86	0	0	0	not significant
2011	GO_RESPONSE_TO_PURINE_CONTAINING_COMPOUND	-1.43	1.85	0	0	0	not significant
2012	GO_EXTRACELLULAR_MATRIX	-1.43	1.78	0	0	0	not significant
2013	GO_MONOCARBOXYLIC_ACID_CATABOLIC_PROCESS	-1.43	1.72	0	0	0	not significant
2014	GO_CELLULAR_RESPONSE_TO_ACID_CHEMICAL	-1.43	1.65	0	0	0	not significant
2015	GO_CELL_REDOX_HOMEOSTASIS	-1.43	1.60	0	0	0	not significant
2016	HALLMARK_UV_RESPONSE_UP	-1.43	1.58	0	0	0	not significant
2017	GO_RNA_POLYMERASE_II_HOLOENZYME	-1.43	1.50	0	0	0	not significant
2018	VECCHI_GASTRIC_CANCER_ADVANCED_VS_EARLY_DN	-1.43	1.46	0	0	0	not significant
2019	TURASHVILI_BREAST_DUCTAL_CARCINOMA_VS_DUCTAL_NORMAL_DN	-1.43	1.44	0	0	0	not significant
2020	GO_CHEMOKINE_PRODUCTION	-1.43	1.43	0	0	0	not significant
2021	KANG_IMMORTALIZED_BY_TERT_UP	-1.43	1.41	0	0	0	not significant
2022	APPIERTO_RESPONSE_TO_FENRETINIDE_DN	-1.43	1.35	0	0	0	not significant
2023	ZHAN_EARLY_DIFFERENTIATION_GENES_DN	-1.43	1.34	0	0	0	not significant
2024	MASSARWEH_RESPONSE_TO_ESTRADIOL	-1.43	1.33	0	0	0	not significant
2025	GO_PRECATALYTIC_SPLICEOSOME	-1.43	1.29	0	0	0	not significant
2026	REACTOME_PROCESSING_OF_CAPPED_INTRONLESS_PRE_MRNA	-1.43	1.29	0	0	0	not significant
2027	REACTOME_SIGNALING_BY_MODERATE_KINASE_ACTIVITY_BRAF_MUTAI	-1.43	1.29	0	0	0	not significant
2028	CHR2P22	-1.43	1.26	0	0	0	not significant
2029	PAPASPYRIDONOS_UNSTABLE_ATEROSCLEROTIC_PLAQUE_UP	-1.43	1.26	0	0	0	not significant
2030	SESTO_RESPONSE_TO_UV_C4	-1.43	1.24	0	0	0	not significant
2031	GO_REGULATION_OF_DEFENSE_RESPONSE_TO_VIRUS_BY_VIRUS	-1.43	1.23	0	0	0	not significant
2032	YAMASHITA_LIVER_CANCER_STEM_CELL_UP	-1.43	1.22	0	0	0	not significant
2033	GO_SARCOMERE_ORGANIZATION	-1.43	1.17	0	0	0	not significant
2034	KEGG_PROTEIN_EXPORT	-1.43	1.17	0	0	0	not significant
2035	REACTOME_MOLECULES_ASSOCIATED_WITH_ELASTIC_FIBRES	-1.43	1.17	0	0	0	not significant
2036	GO_REGULATION_OF_CARDIAC_MUSCLE_CONTRACTION	-1.43	1.14	0	0	0	not significant
2037	HU_GENOTOXIC_DAMAGE_4HR	-1.43	1.14	0	0	0	not significant
2038	GO_REGULATION_OF_NAD_P_H_OXIDASE_ACTIVITY	-1.43	1.13	0	0	0	not significant
2039	GO_SERINE_TRANSMEMBRANE_TRANSPORTER_ACTIVITY	-1.43	1.13	0	0	0	not significant
2040	BYSTRYKH_HEMATOPOIESIS_STEM_CELL_IL3RA	-1.43	1.12	0	0	0	not significant
2041	GO_ATPASE_ACTIVITY_COUPLED_TO_TRANSMEMBRANE_MOVEMENT_O	-1.43	1.12	0	0	0	not significant
2042	REACTOME_POTASSIUM_CHANNELS	-1.43	1.12	0	0	0	not significant
2043	GO_COA_HYDROLASE_ACTIVITY	-1.43	1.11	0	0	0	not significant
2044	GO_AZOLE_TRANSMEMBRANE_TRANSPORTER_ACTIVITY	-1.43	1.10	0	0	0	not significant
2045	GO_MATURATION_OF_LSU_RRNA_FROM_TRICISTRONIC_RRNA_TRANSC	-1.43	1.10	0	0	0	not significant
2046	ROY_WOUND_BLOOD_VESSEL_DN	-1.43	1.10	0	0	0	not significant
2047	GO_DETECTION_OF_MECHANICAL_STIMULUS_INVOLVED_IN_SENSORY_F	-1.43	1.09	0	0	0	not significant
2048	GO_GLYCINE_METABOLIC_PROCESS	-1.43	1.08	0	0	0	not significant
2049	GO_NUCLEAR_NCRNA_SURVEILLANCE	-1.43	1.08	0	0	0	not significant
2050	GO_REGULATION_OF_TYPE_2_IMMUNE_RESPONSE	-1.43	1.08	0	0	0	not significant
2051	GO_MYELIN_ASSEMBLY	-1.43	1.07	0	0	0	not significant
2052	GO_STEREOCILIUM_BUNDLE	-1.43	1.07	0	0	0	not significant
2053	GO_GLUTATHIONE_TRANSFERASE_ACTIVITY	-1.43	1.06	0	0	0	not significant
2054	GO_POSITIVE_REGULATION_OF_PROTEIN_LOCALIZATION_TO_CELL_SUI	-1.43	1.06	0	0	0	not significant
2055	BIOCARTA_ECM_PATHWAY	-1.43	1.05	0	0	0	not significant
2056	GO_MULTI_ORGANISM_CELLULAR_LOCALIZATION	-1.43	1.05	0	0	0	not significant
2057	GO_REGULATION_OF_T_HELPER_1_CELL_DIFFERENTIATION	-1.43	1.05	0	0	0	not significant
2058	GO_CARBOHYDRATE_TRANSMEMBRANE_TRANSPORTER_ACTIVITY	-1.43	1.04	0	0	0	not significant
2059	GO_POTASSIUM_ION_IMPORT_ACROSS_PLASMA_MEMBRANE	-1.43	1.04	0	0	0	not significant

2060	GO_GLUCOSE_6_PHOSPHATE_METABOLIC_PROCESS	-1.43	1.03	0	0	0	not significant
2061	GO_NUCLEAR_PORE_CENTRAL_TRANSPORT_CHANNEL	-1.43	1.03	0	0	0	not significant
2062	GO_PSEUDOURIDINE_SYNTHASE_ACTIVITY	-1.43	1.02	0	0	0	not significant
2063	GO_CENTRAL_NERVOUS_SYSTEM_NEURON_AXONOGENESIS	-1.43	1.01	0	0	0	not significant
2064	FIGUEROA_AML_METHYLATION_CLUSTER_4_DN	-1.43	1.00	0	0	0	not significant
2065	CUI_TCF21_TARGETS_UP	-1.43	0.98	0	0	0	not significant
2066	GO_SUGAR_TRANSMEMBRANE_TRANSPORTER_ACTIVITY	-1.43	0.98	0	0	0	not significant
2067	GO_DNA_CYTOSINE_DEAMINATION	-1.43	0.97	0	0	0	not significant
2068	GO_NEGATIVE_REGULATION_OF_SMOOTH_MUSCLE_CELL_MIGRATION	-1.43	0.94	0	0	0	not significant
2069	GO_SYNAPTIC_VESICLE_BUDDING	-1.43	0.94	0	0	0	not significant
2070	GO_DNA_DEAMINATION	-1.43	0.91	0	0	0	not significant
2071	ENK_UV_RESPONSE_KERATINOCYTE_DN	-1.42	4.00	0	0	0	not significant
2072	GO_CELL_DIVISION	-1.42	4.00	0	0	0	not significant
2073	GO_CELLULAR_RESPONSE_TO_EXTERNAL_STIMULUS	-1.42	4.00	0	0	0	not significant
2074	GO_ENZYME_REGULATOR_ACTIVITY	-1.42	4.00	0	0	0	not significant
2075	GO_MORPHOGENESIS_OF_AN_EPITHELIUM	-1.42	4.00	0	0	0	not significant
2076	REACTOME_SIGNALING_BY_RHO_GTPASES	-1.42	4.00	0	0	0	not significant
2077	WIERENGA_STAT5A_TARGETS_DN	-1.42	2.49	0	0	0	not significant
2078	REACTOME_OTHER_INTERLEUKIN_SIGNALING	-1.42	2.43	0	0	0	not significant
2079	DEBIASI_APOPTOSIS_BY_REOVIRUS_INFECTION_DN	-1.42	2.37	0	0	0	not significant
2080	GO_REGULATION_OF_CALCIUM_ION_TRANSPORT	-1.42	2.20	0	0	0	not significant
2081	SHETH_LIVER_CANCER_VS_TXNIP_LOSS_PAM1	-1.42	2.18	0	0	0	not significant
2082	REACTOME_NEDDYLATION	-1.42	2.13	0	0	0	not significant
2083	GO_MEMBRANE_FUSION	-1.42	1.88	0	0	0	not significant
2084	GO_REGULATION_OF_CYTOKINESIS	-1.42	1.79	0	0	0	not significant
2085	KAAB_FAILED_HEART_ATRIUM_DN	-1.42	1.77	0	0	0	not significant
2086	RASHI_RESPONSE_TO_IONIZING_RADIATION_5	-1.42	1.74	0	0	0	not significant
2087	GO_MITOCHONDRIAL_MEMBRANE_ORGANIZATION	-1.42	1.59	0	0	0	not significant
2088	REACTOME_MITOCHONDRIAL_BIOGENESIS	-1.42	1.54	0	0	0	not significant
2089	AMUNDSON_POOR_SURVIVAL_AFTER_GAMMA_RADIATION_8G	-1.42	1.47	0	0	0	not significant
2090	GO_REGULATION_OF_STEROID_METABOLIC_PROCESS	-1.42	1.44	0	0	0	not significant
2091	KEGG_SYSTEMIC_LUPUS_ERYTHEMATOSUS	-1.42	1.43	0	0	0	not significant
2092	LEE_AGING_NEOCORTEX_DN	-1.42	1.43	0	0	0	not significant
2093	GO_NEGATIVE_REGULATION_OF_PROTEIN_BINDING	-1.42	1.42	0	0	0	not significant
2094	GO_METAL_CLUSTER_BINDING	-1.42	1.40	0	0	0	not significant
2095	CHR14Q12	-1.42	1.39	0	0	0	not significant
2096	GO_TRANSCRIPTION_ELONGATION_FROM_RNA_POLYMERASE_II_PROM(	-1.42	1.39	0	0	0	not significant
2097	GO_REGULATION_OF_MUSCLE_ADAPTATION	-1.42	1.38	0	0	0	not significant
2098	GO_MUSCLE_ADAPTATION	-1.42	1.36	0	0	0	not significant
2099	GO_NEGATIVE_REGULATION_OF_RNA_METABOLIC_PROCESS	-1.42	1.31	0	0	0	not significant
2100	GO_PROTEIN_PEPTIDYL_PROLYL_ISOMERIZATION	-1.42	1.30	0	0	0	not significant
2101	QUINTENS_EMBRYONIC_BRAIN_RESPONSE_TO_IR	-1.42	1.29	0	0	0	not significant
2102	REACTOME_RAF_ACTIVATION	-1.42	1.29	0	0	0	not significant
2103	SMID_BREAST_CANCER_ERBB2_UP	-1.42	1.28	0	0	0	not significant
2104	REACTOME_SMOOTH_MUSCLE_CONTRACTION	-1.42	1.27	0	0	0	not significant
2105	PID_EPHB_FWD_PATHWAY	-1.42	1.23	0	0	0	not significant
2106	GO_TERMINATION_OF_RNA_POLYMERASE_II_TRANSCRIPTION	-1.42	1.22	0	0	0	not significant
2107	CHR2Q32	-1.42	1.21	0	0	0	not significant
2108	GO_POSITIVE_REGULATION_OF_TELOMERASE_ACTIVITY	-1.42	1.21	0	0	0	not significant
2109	LI_LUNG_CANCER	-1.42	1.21	0	0	0	not significant
2110	CORRE_MULTIPLE_MYELOMA_UP	-1.42	1.20	0	0	0	not significant
2111	GO_POSITIVE_REGULATION_OF_INTERLEUKIN_2_PRODUCTION	-1.42	1.20	0	0	0	not significant
2112	GO_POSITIVE_REGULATION_OF_TRANSCRIPTION_BY_RNA_POLYMERAS	-1.42	1.19	0	0	0	not significant
2113	TRACEY_RESISTANCE_TO_IFNA2_DN	-1.42	1.18	0	0	0	not significant
2114	GO_SECRETORY_GRANULE_ORGANIZATION	-1.42	1.17	0	0	0	not significant
2115	LINDSTEDT_DENDRITIC_CELL_MATURATION_D	-1.42	1.17	0	0	0	not significant
2116	REACTOME_STRIATED_MUSCLE_CONTRACTION	-1.42	1.17	0	0	0	not significant
2117	GO_LIGASE_ACTIVITY_FORMING_CARBON_SULFUR_BONDS	-1.42	1.16	0	0	0	not significant
2118	GO_RESPONSE_TO_ISCHEMIA	-1.42	1.16	0	0	0	not significant
2119	GO_REGULATION_OF_B_CELL_DIFFERENTIATION	-1.42	1.15	0	0	0	not significant
2120	REACTOME_TRANSPORT_AND_SYNTHESIS_OF_PAPS	-1.42	1.15	0	0	0	not significant
2121	GO_POSITIVE_REGULATION_OF_FAT_CELL_DIFFERENTIATION	-1.42	1.14	0	0	0	not significant
2122	GO_TOLL_LIKE_RECEPTOR_7_SIGNALING_PATHWAY	-1.42	1.14	0	0	0	not significant
2123	PID_DELTA_NP63_PATHWAY	-1.42	1.12	0	0	0	not significant
2124	JIANG_TIP30_TARGETS_DN	-1.42	1.11	0	0	0	not significant
2125	GO_ZYMOGEN_ACTIVATION	-1.42	1.10	0	0	0	not significant
2126	KAMIKUBO_MYELOID_CEBPA_NETWORK	-1.42	1.10	0	0	0	not significant
2127	PARK_APL_PATHOGENESIS_UP	-1.42	1.10	0	0	0	not significant
2128	REACTOME_MITOTIC_TELOPHASE_CYTOKINESIS	-1.42	1.10	0	0	0	not significant
2129	BARRIER_CANCER_RELAPSE_NORMAL_SAMPLE_UP	-1.42	1.09	0	0	0	not significant
2130	GO_REGULATION_OF_T_CELL_CYTOKINE_PRODUCTION	-1.42	1.09	0	0	0	not significant
2131	ZWANG_EGF_PERSISTENTLY_UP	-1.42	1.09	0	0	0	not significant
2132	DAZARD_UV_RESPONSE_CLUSTER_G2	-1.42	1.08	0	0	0	not significant
2133	GO_PROTEASOME_ASSEMBLY	-1.42	1.08	0	0	0	not significant
2134	ZUCCHI_METASTASIS_DN	-1.42	1.08	0	0	0	not significant
2135	GO_COENZYME_A_BIOSYNTHETIC_PROCESS	-1.42	1.07	0	0	0	not significant
2136	GO_POSITIVE_REGULATION_OF_GLIAL_CELL_DIFFERENTIATION	-1.42	1.07	0	0	0	not significant
2137	GO_RESPONSE_TO_ANGIOTENSIN	-1.42	1.07	0	0	0	not significant
2138	AKL_HTLV1_INFECTION_UP	-1.42	1.06	0	0	0	not significant
2139	GO_POSITIVE_REGULATION_OF_CALCINEURIN_MEDIATED_SIGNALING	-1.42	1.06	0	0	0	not significant
2140	MA_PITUITARY_FETAL_VS_ADULT_DN	-1.42	1.06	0	0	0	not significant
2141	GO_CELLULAR_RESPONSE_TO_PURINE_CONTAINING_COMPOUND	-1.42	1.05	0	0	0	not significant
2142	GO_POSITIVE_REGULATION_OF_OXIDATIVE_STRESS_INDUCED_INTRINSI	-1.42	1.05	0	0	0	not significant
2143	GO_ISOPRENOID_BINDING	-1.42	1.04	0	0	0	not significant
2144	GO_CYTIDINE_DEAMINASE_ACTIVITY	-1.42	1.03	0	0	0	not significant
2145	GO_MONONUCLEAR_CELL_MIGRATION	-1.42	1.03	0	0	0	not significant
2146	REACTOME_RECYCLING_OF_EIF2:GDP	-1.42	1.02	0	0	0	not significant
2147	LIANG_HEMATOPOIESIS_STEM_CELL_NUMBER_QTL	-1.42	1.01	0	0	0	not significant
2148	KUROKAWA_LIVER_CANCER_EARLY_RECURRENCE_DN	-1.42	1.00	0	0	0	not significant
2149	GO_CELL_COMMUNICATION_BY_ELECTRICAL_COUPLING_INVOLVED_IN_(	-1.42	0.99	0	0	0	not significant
2150	GO_HISTONE_SERINE_PHOSPHORYLATION	-1.42	0.99	0	0	0	not significant
2151	BUCKANOVICH_T_LYMPHOCYTE_HOMING_ON_TUMOR_DN	-1.42	0.98	0	0	0	not significant
2152	WIEMANN_TELOMERE_SHORTENING_AND_CHRONIC_LIVER_DAMAGE_UP	-1.42	0.98	0	0	0	not significant
2153	GO_NUCLEOLAR_RIBONUCLEASE_P_COMPLEX	-1.42	0.97	0	0	0	not significant
2154	GO_PROTEIN_K6_LINKED_UBIQUITINATION	-1.42	0.97	0	0	0	not significant
2155	GO_GLYCEROL_ETHER_BIOSYNTHETIC_PROCESS	-1.42	0.95	0	0	0	not significant
2156	BIOCARTA_UCALPAIN_PATHWAY	-1.42	0.94	0	0	0	not significant
2157	GO_RIBONUCLEASE_P_ACTIVITY	-1.42	0.93	0	0	0	not significant
2158	GO_DRUG_BINDING	-1.41	4.00	0	0	0	not significant
2159	GO_MITOTIC_CELL_CYCLE	-1.41	4.00	0	0	0	not significant
2160	GO_NEGATIVE_REGULATION_OF_CELL_CYCLE_PHASE_TRANSITION	-1.41	4.00	0	0	0	not significant
2161	GO_RESPONSE_TO_EXTRACELLULAR_STIMULUS	-1.41	4.00	0	0	0	not significant
2162	GO_RESPONSE_TO_OXIDATIVE_STRESS	-1.41	4.00	0	0	0	not significant
2163	GO_ACTIN_FILAMENT_BINDING	-1.41	2.47	0	0	0	not significant
2164	GO_NUCLEOSIDE_PHOSPHATE_METABOLIC_PROCESS	-1.41	2.46	0	0	0	not significant
2165	REACTOME_DISEASES_OF_SIGNAL_TRANSDUCTION	-1.41	2.35	0	0	0	not significant
2166	CREIGHTON_ENDOCRINE_THERAPY_RESISTANCE_1	-1.41	2.29	0	0	0	not significant
2167	GO_CELLULAR_RESPONSE_TO_STEROID_HORMONE_STIMULUS	-1.41	2.17	0	0	0	not significant
2168	GO_POSITIVE_REGULATION_OF_NUCLEOBASE_CONTAINING_COMPOUNI	-1.41	2.15	0	0	0	not significant

2169	GO_UROGENITAL_SYSTEM_DEVELOPMENT	-1.41	2.14	0	0	0	not significant
2170	REACTOME_DEUBIQUITINATION	-1.41	2.05	0	0	0	not significant
2171	HOSHIDA_LIVER_CANCER_SUBCLASS_S3	-1.41	2.01	0	0	0	not significant
2172	SMID_BREAST_CANCER_LUMINAL_B_UP	-1.41	1.67	0	0	0	not significant
2173	DARWICHE_SQUAMOUS_CELL_CARCINOMA_UP	-1.41	1.63	0	0	0	not significant
2174	MENSE_HYPOXIA_UP	-1.41	1.59	0	0	0	not significant
2175	GO_POSITIVE_REGULATION_OF_LEUKOCYTE_PROLIFERATION	-1.41	1.57	0	0	0	not significant
2176	WANG_ESOPHAGUS_CANCER_VS_NORMAL_DN	-1.41	1.57	0	0	0	not significant
2177	HSIAO_LIVER_SPECIFIC_GENES	-1.41	1.56	0	0	0	not significant
2178	IL21_UP.V1_UP	-1.41	1.52	0	0	0	not significant
2179	REACTOME_CLASS_A_1_RHODOPSIN_LIKE_RECEPTORS	-1.41	1.49	0	0	0	not significant
2180	ICHIBA_GRAFT_VERSUS_HOST_DISEASE_D7_UP	-1.41	1.46	0	0	0	not significant
2181	GO_ORGANIC_HYDROXY_COMPOUND_CATABOLIC_PROCESS	-1.41	1.43	0	0	0	not significant
2182	GO_REGULATION_OF_MUSCLE_CONTRACTION	-1.41	1.40	0	0	0	not significant
2183	GO_RESPONSE_TO_AMMONIUM_ION	-1.41	1.40	0	0	0	not significant
2184	KEGG_GAP_JUNCTION	-1.41	1.37	0	0	0	not significant
2185	HALLMARK_REACTIVE_OXYGEN_SPECIES_PATHWAY	-1.41	1.36	0	0	0	not significant
2186	PID_CDC42_PATHWAY	-1.41	1.35	0	0	0	not significant
2187	SHEPARD_BMYB_TARGETS	-1.41	1.35	0	0	0	not significant
2188	GO_CHROMOSOME_SEPARATION	-1.41	1.34	0	0	0	not significant
2189	GO_REGULATION_OF_SODIUM_ION_TRANSMEMBRANE_TRANSPORT	-1.41	1.32	0	0	0	not significant
2190	REACTOME_TRANSLOCATION_OF_SLC2A4_GLUT4_TO_THE_PLASMA_MI	-1.41	1.29	0	0	0	not significant
2191	GO_CARDIAC_CELL_DEVELOPMENT	-1.41	1.28	0	0	0	not significant
2192	AMIT_EGF_RESPONSE_480_MCF10A	-1.41	1.27	0	0	0	not significant
2193	REACTOME_EPH_EPHRIN_SIGNALING	-1.41	1.27	0	0	0	not significant
2194	GO_BODY_MORPHOGENESIS	-1.41	1.26	0	0	0	not significant
2195	PID_IL12_2PATHWAY	-1.41	1.25	0	0	0	not significant
2196	GO_ALCOHOL_CATABOLIC_PROCESS	-1.41	1.21	0	0	0	not significant
2197	GO_REGULATION_OF_PROTEIN_MATURATION	-1.41	1.21	0	0	0	not significant
2198	BROWNE_HCMV_INFECTION_6HR_UP	-1.41	1.20	0	0	0	not significant
2199	BURTON_ADIPOGENESIS_PEAK_AT_8HR	-1.41	1.19	0	0	0	not significant
2200	NAKAYAMA_SOFT_TISSUE_TUMORS_PCA1_UP	-1.41	1.19	0	0	0	not significant
2201	GO_NUCLEAR_ENVELOPE_REASSEMBLY	-1.41	1.18	0	0	0	not significant
2202	GO_POSITIVE_REGULATION_OF_UBIQUITIN_PROTEIN_TRANSFERASE_AC	-1.41	1.18	0	0	0	not significant
2203	REACTOME_BMAL1:CLOCK_NPAS2_ACTIVATES_CIRCADIAN_GENE_EXPR	-1.41	1.17	0	0	0	not significant
2204	BIOCARTA_AKAP95_PATHWAY	-1.41	1.15	0	0	0	not significant
2205	GO_MICROVILLUS_ORGANIZATION	-1.41	1.15	0	0	0	not significant
2206	GO_INTRINSIC_APOPTOTIC_SIGNALING_PATHWAY_IN_RESPONSE_TO_O)	-1.41	1.14	0	0	0	not significant
2207	GO_SKELETAL_MUSCLE_TISSUE_REGENERATION	-1.41	1.12	0	0	0	not significant
2208	KYNG_ENVIRONMENTAL_STRESS_RESPONSE_NOT_BY_4NQO_IN_WS	-1.41	1.10	0	0	0	not significant
2209	GO_PROTEIN_KINASE_A_REGULATORY_SUBUNIT_BINDING	-1.41	1.08	0	0	0	not significant
2210	GO_RESPONSE_TO_MORPHINE	-1.41	1.08	0	0	0	not significant
2211	HOFMANN_CELL_LYMPHOMA_DN	-1.41	1.08	0	0	0	not significant
2212	DACOSTA_ERCC3_ALLELE_XPCS_VS_TTD_UP	-1.41	1.05	0	0	0	not significant
2213	GO_NUCLEAR_LOCALIZATION_SEQUENCE_BINDING	-1.41	1.05	0	0	0	not significant
2214	BIOCARTA_PPARG_PATHWAY	-1.41	1.04	0	0	0	not significant
2215	GO_CARBON_NITROGEN_LYASE_ACTIVITY	-1.41	1.03	0	0	0	not significant
2216	GO_NEGATIVE_REGULATION_OF_MYELOID_CELL_APOPTOTIC_PROCES:	-1.41	1.02	0	0	0	not significant
2217	GO_NEGATIVE_REGULATION_OF_PROTEIN_LOCALIZATION_TO_MEMBRA	-1.41	1.02	0	0	0	not significant
2218	GO_POSITIVE_REGULATION_OF_MONOOXYGENASE_ACTIVITY	-1.41	1.02	0	0	0	not significant
2219	PID_INTEGRIN_A9B1_PATHWAY	-1.41	1.01	0	0	0	not significant
2220	GO_NUCLEAR_POLYADENYLATION_DEPENDENT_TRNA_CATABOLIC_PRC	-1.41	0.99	0	0	0	not significant
2221	GO_ADULT_HEART_DEVELOPMENT	-1.41	0.97	0	0	0	not significant
2222	GO_MITOCHONDRIAL_ACETYL_COA_BIOSYNTHETIC_PROCESS_FROM_P	-1.41	0.97	0	0	0	not significant
2223	GO_REGULATION_OF_CARDIAC_MUSCLE_ADAPTATION	-1.41	0.97	0	0	0	not significant
2224	GO_POSITIVE_REGULATION_OF_MONOCYTE_CHEMOTAXIS	-1.41	0.96	0	0	0	not significant
2225	OXFORD_RALA_TARGETS_DN	-1.41	0.96	0	0	0	not significant
2226	SCHAEFFER_PROSTATE_DEVELOPMENT_12HR_DN	-1.41	0.96	0	0	0	not significant
2227	GO_TOXIN_METABOLIC_PROCESS	-1.41	0.94	0	0	0	not significant
2228	OHASHI_AURKA_TARGETS	-1.41	0.93	0	0	0	not significant
2229	GO_FEMALE_MEIOSIS_I	-1.41	0.92	0	0	0	not significant
2230	KEGG_ASCORBATE_AND_ALDARATE_METABOLISM	-1.41	0.92	0	0	0	not significant
2231	REACTOME_WAX_AND_PLASMALOGEN_BIOSYNTHESIS	-1.41	0.92	0	0	0	not significant
2232	GO_HISTONE_H4_R3_METHYLATION	-1.41	0.90	0	0	0	not significant
2233	ACEVEDO_LIVER_CANCER_UP	-1.40	4.00	0	0	0	not significant
2234	CUI_TCF21_TARGETS_2_UP	-1.40	4.00	0	0	0	not significant
2235	GO_CELLULAR_RESPONSE_TO_ORGANIC_CYCLIC_COMPOUND	-1.40	4.00	0	0	0	not significant
2236	GO_MRNA_METABOLIC_PROCESS	-1.40	4.00	0	0	0	not significant
2237	YANG_BCL3_TARGETS_UP	-1.40	2.43	0	0	0	not significant
2238	NABA_MATRISOME_ASSOCIATED	-1.40	2.41	0	0	0	not significant
2239	ENK_UV_RESPONSE_EPIDERMIS_UP	-1.40	2.40	0	0	0	not significant
2240	SMID_BREAST_CANCER_RELAPSE_IN_BONE_DN	-1.40	2.00	0	0	0	not significant
2241	GO_PURINE_CONTAINING_COMPOUND_METABOLIC_PROCESS	-1.40	1.80	0	0	0	not significant
2242	REACTOME_REGULATION_OF_LIPID_METABOLISM_BY_PEROXISOME_PR	-1.40	1.73	0	0	0	not significant
2243	SMITH_TERT_TARGETS_DN	-1.40	1.58	0	0	0	not significant
2244	EBAUER_TARGETS_OF_PAX3_FOXO1_FUSION_UP	-1.40	1.53	0	0	0	not significant
2245	GO_ANTIGEN_PROCESSING_AND_PRESENTATION_OF_PEPTIDE_OR_POI	-1.40	1.53	0	0	0	not significant
2246	DER_IFN_ALPHA_RESPONSE_UP	-1.40	1.52	0	0	0	not significant
2247	GO_VESICLE_TARGETING_TO_FROM_OR_WITHIN_GOLGI	-1.40	1.44	0	0	0	not significant
2248	SUNG_METASTASIS_STROMA_UP	-1.40	1.42	0	0	0	not significant
2249	GO_DIGESTIVE_SYSTEM_DEVELOPMENT	-1.40	1.39	0	0	0	not significant
2250	GO_CELL_CELL_ADHERENS_JUNCTION	-1.40	1.37	0	0	0	not significant
2251	GO_NEGATIVE_REGULATION_OF_RNA_CATABOLIC_PROCESS	-1.40	1.36	0	0	0	not significant
2252	PID_HEDGEHOG_GLI_PATHWAY	-1.40	1.34	0	0	0	not significant
2253	MORI_LARGE_PRE_BII_LYMPHOCYTE_DN	-1.40	1.27	0	0	0	not significant
2254	BRUINS_UVC_RESPONSE_VIA_TP53_GROUP_C	-1.40	1.26	0	0	0	not significant
2255	REACTOME_G_ALPHA_Z_SIGNALLING_EVENTS	-1.40	1.23	0	0	0	not significant
2256	GO_CARBOHYDRATE_KINASE_ACTIVITY	-1.40	1.22	0	0	0	not significant
2257	KORKOLA_EMBRYONAL_CARCINOMA_UP	-1.40	1.22	0	0	0	not significant
2258	GO_HOMOTYPIC_CELL_CELL_ADHESION	-1.40	1.21	0	0	0	not significant
2259	KAMMINGA_EZH2_TARGETS	-1.40	1.17	0	0	0	not significant
2260	REACTOME_MICRORNA_MIRNA_BIOGENESIS	-1.40	1.14	0	0	0	not significant
2261	BORLAK_LIVER_CANCER_EGF_UP	-1.40	1.13	0	0	0	not significant
2262	GO_MEMBRANE_REPOLARIZATION	-1.40	1.12	0	0	0	not significant
2263	GO_RESPONSE_TO_PROGESTERONE	-1.40	1.12	0	0	0	not significant
2264	OUYANG_PROSTATE_CANCER_PROGRESSION_DN	-1.40	1.12	0	0	0	not significant
2265	GO_MAIN_AXON	-1.40	1.11	0	0	0	not significant
2266	GO_POTASSIUM_CHANNEL_ACTIVITY	-1.40	1.11	0	0	0	not significant
2267	WANG_METASTASIS_OF_BREAST_CANCER_ESR1_UP	-1.40	1.10	0	0	0	not significant
2268	BIOCARTA_RHO_PATHWAY	-1.40	1.09	0	0	0	not significant
2269	PETRETTO_HEART_MASS_QTL_CIS_DN	-1.40	1.06	0	0	0	not significant
2270	RUAN_RESPONSE_TO_TROGLITAZONE_UP	-1.40	1.06	0	0	0	not significant
2271	MAGRANGEAS_MULTIPLE_MYELOMA_IGG_VS_IGA_DN	-1.40	1.05	0	0	0	not significant
2272	REACTOME_ATTENUATION_PHASE	-1.40	1.05	0	0	0	not significant
2273	GO_SEMI_LUNAR_VALVE_DEVELOPMENT	-1.40	1.04	0	0	0	not significant
2274	NEBEN_AML_WITH_FLT3_OR_NRAS_UP	-1.40	1.03	0	0	0	not significant
2275	GO_CALCIUM_IMPORT_INTO_THE_MITOCHONDRION	-1.40	1.02	0	0	0	not significant
2276	GO_SPINDLE_POLE_CENTROSOME	-1.40	1.02	0	0	0	not significant
2277	GO_ALDEHYDE_LYASE_ACTIVITY	-1.40	1.01	0	0	0	not significant

2278	HOFMANN_MYELODYSPLASTIC_SYNDROM_LOW_RISK_DN	-1.40	1.01	0	0	0	not significant
2279	GO_ESTABLISHMENT_OR_MAINTENANCE_OF_MONOPOLAR_CELL_POLAI	-1.40	1.00	0	0	0	not significant
2280	MAHADEVAN_RESPONSE_TO_MP470_UP	-1.40	1.00	0	0	0	not significant
2281	GO_REGULATION_OF_T_HELPER_2_CELL_DIFFERENTIATION	-1.40	0.99	0	0	0	not significant
2282	NIELSEN_LEIOMYOSARCOMA_UP	-1.40	0.99	0	0	0	not significant
2283	GO_CARBON_NITROGEN_LIGASE_ACTIVITY_WITH_GLUTAMINE_AS_AMID	-1.40	0.98	0	0	0	not significant
2284	HERNANDEZ_ABERRANT_MITOSIS_BY_DOCETACEL_2NM_DN	-1.40	0.97	0	0	0	not significant
2285	TSAI_DNAJB4_TARGETS_UP	-1.40	0.97	0	0	0	not significant
2286	GAVIN_IL2_RESPONSIVE_FOXP3_TARGETS_UP	-1.40	0.96	0	0	0	not significant
2287	GO_PROTEIN_LOCALIZATION_TO_CYTOPLASMIC_STRESS_GRANULE	-1.40	0.96	0	0	0	not significant
2288	GO_NEGATIVE_REGULATION_OF_AUTOPHAGOSOME_ASSEMBLY	-1.40	0.95	0	0	0	not significant
2289	MYLLYKANGAS_AMPLIFICATION_HOT_SPOT_15	-1.40	0.95	0	0	0	not significant
2290	GO_NEGATIVE_REGULATION_OF_CHOLESTEROL_STORAGE	-1.40	0.94	0	0	0	not significant
2291	REACTOME_P130CAS_LINKAGE_TO_MAPK_SIGNALING_FOR_INTEGRINS	-1.40	0.94	0	0	0	not significant
2292	GO_DE_NOVO_PYRIMIDINE_NUCLEOBASE_BIOSYNTHETIC_PROCESS	-1.40	0.93	0	0	0	not significant
2293	GO_POSITIVE_REGULATION_OF_BEHAVIOR	-1.40	0.93	0	0	0	not significant
2294	GO_NUCLEAR_IMPORT_SIGNAL_RECEPTOR_ACTIVITY	-1.40	0.92	0	0	0	not significant
2295	GO_PTW_PP1_PHOSPHATASE_COMPLEX	-1.40	0.92	0	0	0	not significant
2296	SUZUKI_RESPONSE_TO_TSA_AND_DECITABINE_1B	-1.40	0.92	0	0	0	not significant
2297	BIOCARTA_SALMONELLA_PATHWAY	-1.40	0.91	0	0	0	not significant
2298	GO_PROTEIN_INSERTION_INTO_MITOCHONDRIAL_INNER_MEMBRANE	-1.40	0.91	0	0	0	not significant
2299	GO_REGULATION_OF_SECONDARY_HEART_FIELD_CARDIOBLAST_PROL	-1.40	0.90	0	0	0	not significant
2300	CHEN_METABOLIC_SYNDROM_NETWORK	-1.39	4.00	0	0	0	not significant
2301	GO_METAL_ION_TRANSPORT	-1.39	4.00	0	0	0	not significant
2302	GO_PEPTIDE_BIOSYNTHETIC_PROCESS	-1.39	4.00	0	0	0	not significant
2303	GO_RESPONSE_TO_ENDOGENOUS_STIMULUS	-1.39	4.00	0	0	0	not significant
2304	GO_RESPONSE_TO_OXYGEN_CONTAINING_COMPOUND	-1.39	4.00	0	0	0	not significant
2305	REACTOME_HEMOSTASIS	-1.39	4.00	0	0	0	not significant
2306	GCNP_SHH_UP_LATE.V1_UP	-1.39	2.16	0	0	0	not significant
2307	GRAESSMANN_RESPONSE_TO_MC_AND_SERUM_DEPRIVATION_UP	-1.39	2.03	0	0	0	not significant
2308	GO_HEMATOPOIETIC_PROGENITOR_CELL_DIFFERENTIATION	-1.39	1.89	0	0	0	not significant
2309	GO_GENE_SILENCING_BY_RNA	-1.39	1.88	0	0	0	not significant
2310	LTE2_UP.V1_DN	-1.39	1.88	0	0	0	not significant
2311	GO_RESPONSE_TO_INTERFERON_GAMMA	-1.39	1.81	0	0	0	not significant
2312	GO_ALCOHOL_BIOSYNTHETIC_PROCESS	-1.39	1.80	0	0	0	not significant
2313	HALLMARK_XENOBIOTIC_METABOLISM	-1.39	1.79	0	0	0	not significant
2314	WAMUNYOKOLI_OVARIAN_CANCER_GRADES_1_2_UP	-1.39	1.73	0	0	0	not significant
2315	GO_CELLULAR_RESPONSE_TO_STARVATION	-1.39	1.72	0	0	0	not significant
2316	REACTOME_REGULATION_OF_PLK1_ACTIVITY_AT_G2_M_TRANSITION	-1.39	1.62	0	0	0	not significant
2317	TAKAO_RESPONSE_TO_UVB_RADIATION_UP	-1.39	1.57	0	0	0	not significant
2318	KIM_WT1_TARGETS_12HR_UP	-1.39	1.54	0	0	0	not significant
2319	GO_ACTIVATION_OF_CYSTEINE_TYPE_ENDOPEPTIDASE_ACTIVITY_INVO	-1.39	1.40	0	0	0	not significant
2320	GO_TRANSCRIPTION_FACTOR_TFIID_COMPLEX	-1.39	1.37	0	0	0	not significant
2321	FLECHNER_PBL_KIDNEY_TRANSPLANT_REJECTED_VS_OK_UP	-1.39	1.31	0	0	0	not significant
2322	KEGG_B_CELL_RECEPTOR_SIGNALING_PATHWAY	-1.39	1.25	0	0	0	not significant
2323	GO_REGULATION_OF_SODIUM_ION_TRANSPORT	-1.39	1.23	0	0	0	not significant
2324	IRITANI_MAD1_TARGETS_DN	-1.39	1.21	0	0	0	not significant
2325	JOHANSSON_BRAIN_CANCER_EARLY_VS_LATE_DN	-1.39	1.12	0	0	0	not significant
2326	GO_EXOSOME_RNASE_COMPLEX_	-1.39	1.08	0	0	0	not significant
2327	GO_STEROL_HOMEOSTASIS	-1.39	1.07	0	0	0	not significant
2328	WANG_BARRETTS_ESOPHAGUS_AND_ESOPHAGUS_CANCER_DN	-1.39	1.07	0	0	0	not significant
2329	GO_INNER_EAR_RECEPTOR_CELL_DEVELOPMENT	-1.39	1.04	0	0	0	not significant
2330	REACTOME_SYNTHESIS_OF_BILE_ACIDS_AND_BILE_SALTS	-1.39	1.04	0	0	0	not significant
2331	GO_XENOBIOTIC_CATABOLIC_PROCESS	-1.39	1.03	0	0	0	not significant
2332	GO_CORECEPTOR_ACTIVITY	-1.39	1.02	0	0	0	not significant
2333	REACTOME_BASIGIN_INTERACTIONS	-1.39	1.02	0	0	0	not significant
2334	GO_NUCLEAR_PORE_OUTER_RING	-1.39	1.01	0	0	0	not significant
2335	GO_OXIDOREDUCTASE_ACTIVITY_ACTING_ON_THE_CH_NH_GROUP_OF	-1.39	1.00	0	0	0	not significant
2336	GO_NEGATIVE_REGULATION_OF_NECROTIC_CELL_DEATH	-1.39	0.99	0	0	0	not significant
2337	GO_RIBONUCLEOSIDE_CATABOLIC_PROCESS	-1.39	0.98	0	0	0	not significant
2338	KYNG_DNA_DAMAGE_BY_4NQO_OR_GAMMA_RADIATION	-1.39	0.97	0	0	0	not significant
2339	GO_CARNITINE_SHUTTLE	-1.39	0.96	0	0	0	not significant
2340	GO_CHONDROCYTE_DIFFERENTIATION_INVOLVED_IN_ENDOCHONDRAL_	-1.39	0.96	0	0	0	not significant
2341	GO_F_ACTIN_CAPPING_PROTEIN_COMPLEX	-1.39	0.96	0	0	0	not significant
2342	GO_POSITIVE_REGULATION_OF_MONONUCLEAR_CELL_MIGRATION	-1.39	0.96	0	0	0	not significant
2343	GO_GLUCOSE_BINDING	-1.39	0.95	0	0	0	not significant
2344	NAKAYAMA_FGF2_TARGETS	-1.39	0.95	0	0	0	not significant
2345	GO_POSITIVE_REGULATION_OF_PHOSPHOLIPID_BIOSYNTHETIC_PROCES	-1.39	0.93	0	0	0	not significant
2346	GO_PRONUCLEUS	-1.39	0.93	0	0	0	not significant
2347	GO_VESICLE_FUSION_WITH_ENDOPLASMIC_RETICULUM_GOLGI_INTERM	-1.39	0.93	0	0	0	not significant
2348	HO_LIVER_CANCER_VASCULAR_INVASION	-1.39	0.92	0	0	0	not significant
2349	REACTOME_TRIGLYCERIDE_CATABOLISM	-1.39	0.92	0	0	0	not significant
2350	GO_PML_BODY_ORGANIZATION	-1.39	0.91	0	0	0	not significant
2351	GO_CYTOCHROME_B5_REDUCTASE_ACTIVITY_ACTING_ON_NAD_P_H	-1.39	0.90	0	0	0	not significant
2352	GO_SNRNA_MODIFICATION	-1.39	0.90	0	0	0	not significant
2353	REACTOME_TRANSLOCATION_OF_ZAP_70_TO_IMMUNOLOGICAL_SYNAP	-1.39	0.89	0	0	0	not significant
2354	GO_PYRIMIDINE_NUCLEOBASE_BIOSYNTHETIC_PROCESS	-1.39	0.86	0	0	0	not significant
2355	BRUINS_UVC_RESPONSE_LATE	-1.38	4.00	0	0	0	not significant
2356	GO_CELLULAR_RESPONSE_TO_HORMONE_STIMULUS	-1.38	4.00	0	0	0	not significant
2357	GO_DIVALENT_INORGANIC_CATION_TRANSPORT	-1.38	4.00	0	0	0	not significant
2358	GO_REGULATION_OF_CELL_CYCLE_PHASE_TRANSITION	-1.38	4.00	0	0	0	not significant
2359	GRYDER_PAX3FOXO1_ENHANCERS_IN_TADS	-1.38	4.00	0	0	0	not significant
2360	KIM_ALL_DISORDERS_CALB1_CORR_UP	-1.38	4.00	0	0	0	not significant
2361	MARSON_BOUND_BY_E2F4_UNSTIMULATED	-1.38	4.00	0	0	0	not significant
2362	GO_ORGANIC_HYDROXY_COMPOUND_BIOSYNTHETIC_PROCESS	-1.38	2.49	0	0	0	not significant
2363	GO_PHAGOCYTOSIS	-1.38	2.43	0	0	0	not significant
2364	SMID_BREAST_CANCER_LUMINAL_B_DN	-1.38	2.40	0	0	0	not significant
2365	KIM_ALL_DISORDERS_DURATION_CORR_DN	-1.38	2.21	0	0	0	not significant
2366	HALLMARK_MITOTIC_SPINDLE	-1.38	1.73	0	0	0	not significant
2367	GO_REGULATION_OF_VIRAL_LIFE_CYCLE	-1.38	1.70	0	0	0	not significant
2368	GO_REGULATION_OF_CELL_DIVISION	-1.38	1.69	0	0	0	not significant
2369	P53_DN.V1_DN	-1.38	1.64	0	0	0	not significant
2370	KEEN_RESPONSE_TO_ROSIGLITAZONE_DN	-1.38	1.61	0	0	0	not significant
2371	KEGG_PURINE_METABOLISM	-1.51	1.61	1	0	0	Repressed
2372	GO_CARBOHYDRATE_CATABOLIC_PROCESS	-1.38	1.58	0	0	0	not significant
2373	REACTOME_FATTY_ACID_METABOLISM	-1.38	1.58	0	0	0	not significant
2374	GO_CARBON_OXYGEN_LYASE_ACTIVITY	-1.38	1.38	0	0	0	not significant
2375	JOHNSTONE_PARVB_TARGETS_2_UP	-1.38	1.33	0	0	0	not significant
2376	DORN_ADENOVIRUS_INFECTION_24HR_DN	-1.38	1.28	0	0	0	not significant
2377	ZAMORA_NOS2_TARGETS_DN	-1.38	1.27	0	0	0	not significant
2378	GO_REGULATION_OF_PEPTIDYL_THREONINE_PHOSPHORYLATION	-1.38	1.26	0	0	0	not significant
2379	GO_ANTIOXIDANT_ACTIVITY	-1.38	1.24	0	0	0	not significant
2380	GO_TELOMERIC_DNA_BINDING	-1.38	1.22	0	0	0	not significant
2381	PEDERSEN_METASTASIS_BY_ERBB2_ISOFORM_4	-1.38	1.22	0	0	0	not significant
2382	DOANE_BREAST_CANCER_CLASSES_UP	-1.38	1.21	0	0	0	not significant
2383	CHR15Q22	-1.38	1.19	0	0	0	not significant
2384	HU_GENOTOXIC_DAMAGE_24HR	-1.38	1.19	0	0	0	not significant
2385	CHIBA_RESPONSE_TO_TSA	-1.38	1.13	0	0	0	not significant
2386	SU_PANCREAS	-1.38	1.13	0	0	0	not significant

2387	ONGUSAHA_TP53_TARGETS	-1.38	1.09	0	0	0	not significant
2388	GO_STEROID_HORMONE_RECEPTOR_ACTIVITY	-1.38	1.08	0	0	0	not significant
2389	REACTOME_PYRUVATE_METABOLISM	-1.38	1.08	0	0	0	not significant
2390	GO_SIGNAL_SEQUENCE_BINDING	-1.38	1.07	0	0	0	not significant
2391	GO_PINOCYTOSIS	-1.38	1.06	0	0	0	not significant
2392	GO_FEEDING_BEHAVIOR	-1.38	1.05	0	0	0	not significant
2393	GO_POSITIVE_REGULATION_OF_LIPID_LOCALIZATION	-1.38	1.05	0	0	0	not significant
2394	IIZUKA_LIVER_CANCER_PROGRESSION_G1_G2_DN	-1.38	1.05	0	0	0	not significant
2395	LAMB_CCND1_TARGETS	-1.38	1.04	0	0	0	not significant
2396	PID_SYNDECAN_2_PATHWAY	-1.38	1.04	0	0	0	not significant
2397	GO_NEGATIVE_REGULATION_OF_EXOCYTOSIS	-1.38	1.03	0	0	0	not significant
2398	HOUSTIS_ROS	-1.38	1.03	0	0	0	not significant
2399	GO_ATP_BIOSYNTHETIC_PROCESS	-1.38	1.02	0	0	0	not significant
2400	GO_NEUROMUSCULAR_PROCESS_CONTROLLING_BALANCE	-1.38	1.02	0	0	0	not significant
2401	SEIDEN_MET_SIGNALING	-1.38	1.02	0	0	0	not significant
2402	GO_ENTRAINMENT_OF_CIRCADIAN_CLOCK_BY_PHOTOPERIOD	-1.38	1.01	0	0	0	not significant
2403	GO_MRNA_MODIFICATION	-1.38	1.01	0	0	0	not significant
2404	GO_PORE_COMPLEX	-1.38	1.00	0	0	0	not significant
2405	GO_ORGAN_OR_TISSUE_SPECIFIC_IMMUNE_RESPONSE	-1.38	0.99	0	0	0	not significant
2406	NOUSHMEHR_GBM_SOMATIC_MUTATED	-1.38	0.99	0	0	0	not significant
2407	CHR2Q36	-1.38	0.98	0	0	0	not significant
2408	GO_FACE_MORPHOGENESIS	-1.38	0.98	0	0	0	not significant
2409	NIKOLSKY_BREAST_CANCER_5P15_AMPLICON	-1.38	0.98	0	0	0	not significant
2410	GO_POSITIVE_REGULATION_OF_TRANSCRIPTION_FROM_RNA_POLYMEF	-1.38	0.97	0	0	0	not significant
2411	GO_REGULATION_OF_RNA_POLYMERASE_II_TRANSCRIPTIONAL_PREINIT	-1.38	0.97	0	0	0	not significant
2412	GO_NEGATIVE_REGULATION_OF_ENDOPLASMIC_RETICULUM_STRESS_I	-1.38	0.96	0	0	0	not significant
2413	GO_NUCLEOLUS_ORGANIZATION	-1.38	0.96	0	0	0	not significant
2414	GO_RENAL_SYSTEM_VASCULATURE_DEVELOPMENT	-1.38	0.95	0	0	0	not significant
2415	REACTOME_GDP_FUCOSE_BIOSYNTHESIS	-1.38	0.95	0	0	0	not significant
2416	STONER_ESOPHAGEAL_CARCINOGENESIS_DN	-1.38	0.95	0	0	0	not significant
2417	GO_POSITIVE_REGULATION_OF_VACUOLE_ORGANIZATION	-1.38	0.94	0	0	0	not significant
2418	GO_DNA_TOPOISOMERASE_ACTIVITY	-1.38	0.93	0	0	0	not significant
2419	GO_REGULATION_OF_SYSTEMIC_ARTERIAL_BLOOD_PRESSURE_BY_CIF	-1.38	0.93	0	0	0	not significant
2420	PID_CONE_PATHWAY	-1.38	0.92	0	0	0	not significant
2421	GO_CATION_TRANSPORTING_ATPASE_COMPLEX	-1.38	0.91	0	0	0	not significant
2422	GO_INTESTINAL_ABSORPTION	-1.38	0.91	0	0	0	not significant
2423	GO_TYPE_I_INTERFERON_BIOSYNTHETIC_PROCESS	-1.38	0.91	0	0	0	not significant
2424	REACTOME_CREB1_PHOSPHORYLATION_THROUGH_THE_ACTIVATION_C	-1.38	0.91	0	0	0	not significant
2425	REACTOME_TELOMERE_EXTENSION_BY_TELOMERASE	-1.38	0.91	0	0	0	not significant
2426	GO_ENOYL_COA_HYDRATASE_ACTIVITY	-1.38	0.90	0	0	0	not significant
2427	GO_FATTY_ACID_BINDING	-1.38	0.89	0	0	0	not significant
2428	GO_ALKALI_METAL_ION_BINDING	-1.38	0.88	0	0	0	not significant
2429	GO_REGULATION_OF_ISOMERASE_ACTIVITY	-1.38	0.88	0	0	0	not significant
2430	REACTOME_CLEC7A_DECTIN_1_INDUCES_NFAT_ACTIVATION	-1.38	0.88	0	0	0	not significant
2431	GO_U4_SNRNP	-1.38	0.87	0	0	0	not significant
2432	REACTOME_DNA_DAMAGE_REVERSAL	-1.38	0.87	0	0	0	not significant
2433	GO_GLOMERULAR_MESANGIUM_DEVELOPMENT	-1.38	0.81	0	0	0	not significant
2434	GO_ADENYL_NUCLEOTIDE_BINDING	-1.37	4.00	0	0	0	not significant
2435	GO_ATPASE_ACTIVITY_COUPLED	-1.37	4.00	0	0	0	not significant
2436	GO_HOMEOSTATIC_PROCESS	-1.37	4.00	0	0	0	not significant
2437	GO_POSITIVE_REGULATION_OF_IMMUNE_RESPONSE	-1.37	4.00	0	0	0	not significant
2438	GO_REGULATION_OF_IMMUNE_RESPONSE	-1.37	4.00	0	0	0	not significant
2439	REACTOME_METABOLISM_OF_CARBOHYDRATES	-1.37	4.00	0	0	0	not significant
2440	MITSIADES_RESPONSE_TO_APLIDIN_DN	-1.37	1.92	0	0	0	not significant
2441	HALLMARK_IL2_STAT5_SIGNALING	-1.37	1.80	0	0	0	not significant
2442	DOANE_RESPONSE_TO_ANDROGEN_UP	-1.37	1.75	0	0	0	not significant
2443	GO_RESPONSE_TO_NUTRIENT	-1.37	1.73	0	0	0	not significant
2444	GO_SPINDLE_ORGANIZATION	-1.37	1.73	0	0	0	not significant
2445	HAMAI_APOPTOSIS_VIA_TRAIL_DN	-1.37	1.73	0	0	0	not significant
2446	GO_NEGATIVE_REGULATION_OF_BINDING	-1.37	1.70	0	0	0	not significant
2447	GO_STEM_CELL_DIFFERENTIATION	-1.37	1.69	0	0	0	not significant
2448	GO_RENAL_SYSTEM_DEVELOPMENT	-1.37	1.64	0	0	0	not significant
2449	LEE_EARLY_T_LYMPHOCYTE_UP	-1.37	1.64	0	0	0	not significant
2450	REACTOME_RNA_POLYMERASE_I_TRANSCRIPTION	-1.37	1.63	0	0	0	not significant
2451	SCHAEFFER_PROSTATE_DEVELOPMENT_6HR_UP	-1.37	1.56	0	0	0	not significant
2452	E2F3_UP.V1_DN	-1.37	1.44	0	0	0	not significant
2453	GO_REGULATION_OF_TRANSLATIONAL_INITIATION	-1.37	1.42	0	0	0	not significant
2454	MCCLUNG_CREB1_TARGETS_UP	-1.37	1.30	0	0	0	not significant
2455	GO_REGULATION_OF_WOUND_HEALING	-1.37	1.27	0	0	0	not significant
2456	GO_MACROPHAGE_ACTIVATION	-1.37	1.22	0	0	0	not significant
2457	BURTON_ADIPOGENESIS_2	-1.37	1.18	0	0	0	not significant
2458	GO_BASAL_TRANSCRIPTION_MACHINERY_BINDING	-1.37	1.17	0	0	0	not significant
2459	WARTERS_IR_RESPONSE_5GY	-1.37	1.15	0	0	0	not significant
2460	GO_MRNA_CIS_SPLICING_VIA_SPLICEOSOME	-1.37	1.13	0	0	0	not significant
2461	GO_PLATELET_AGGREGATION	-1.37	1.10	0	0	0	not significant
2462	VILLANUEVA_LIVER_CANCER_KRT19_DN	-1.37	1.10	0	0	0	not significant
2463	GO_ESTABLISHMENT_OF_PROTEIN_LOCALIZATION_TO_TELOMERE	-1.37	1.09	0	0	0	not significant
2464	GO_POSITIVE_REGULATION_OF_LIPID_TRANSPORT	-1.37	1.07	0	0	0	not significant
2465	GO_REGULATION_OF_CELLULAR_RESPIRATION	-1.37	1.05	0	0	0	not significant
2466	REACTOME_TP53_REGULATES_TRANSCRIPTION_OF_GENES_INVOLVED	-1.37	1.05	0	0	0	not significant
2467	GO_REGULATION_OF_T_CELL_CHEMOTAXIS	-1.37	1.04	0	0	0	not significant
2468	GO_CALCIUM_CHANNEL_REGULATOR_ACTIVITY	-1.37	1.03	0	0	0	not significant
2469	GO_REGULATION_OF_MITOCHONDRIAL_TRANSLATION	-1.37	1.03	0	0	0	not significant
2470	BYSTRYKH_HEMATOPOIESIS_STEM_CELL_SCP2_QTL_TRANS	-1.37	1.02	0	0	0	not significant
2471	GO_ESTABLISHMENT_OF_PROTEIN_LOCALIZATION_TO_CHROMOSOME	-1.37	1.01	0	0	0	not significant
2472	GO_LAMELLIPODIUM_MORPHOGENESIS	-1.37	1.01	0	0	0	not significant
2473	GO_PROTEIN_LOCALIZATION_TO_CHROMOSOME_TELOMERIC_REGION	-1.37	1.01	0	0	0	not significant
2474	JOSEPH_RESPONSE_TO_SODIUM_BUTYRATE_UP	-1.37	1.01	0	0	0	not significant
2475	GO_REGULATION_OF_MYELINATION	-1.37	1.00	0	0	0	not significant
2476	GO_MHC_CLASS_I_PEPTIDE_LOADING_COMPLEX	-1.37	0.99	0	0	0	not significant
2477	GO_NEGATIVE_REGULATION_OF_DENDRITE_DEVELOPMENT	-1.37	0.99	0	0	0	not significant
2478	GO_BRANCHED_CHAIN_AMINO_ACID_CATABOLIC_PROCESS	-1.37	0.98	0	0	0	not significant
2479	GO_REGULATION_OF_MACROPHAGE_ACTIVATION	-1.37	0.97	0	0	0	not significant
2480	GO_CENTROSOME_SEPARATION	-1.37	0.96	0	0	0	not significant
2481	GO_OXIDOREDUCTASE_ACTIVITY_ACTING_ON_THE_CH_CH_GROUP_OF_	-1.37	0.96	0	0	0	not significant
2482	TERAO_AOX4_TARGETS_SKIN_DN	-1.37	0.96	0	0	0	not significant
2483	GO_HAIR_CELL_DIFFERENTIATION	-1.37	0.95	0	0	0	not significant
2484	GO_TRNA_WOBBLE_BASE_MODIFICATION	-1.37	0.95	0	0	0	not significant
2485	GO_VASCULAR_ENDOTHELIAL_GROWTH_FACTOR_RECEPTOR_BINDING	-1.37	0.95	0	0	0	not significant
2486	MOOTHA_GLYCOGEN_METABOLISM	-1.37	0.95	0	0	0	not significant
2487	BIOCARTA_NO1_PATHWAY	-1.37	0.94	0	0	0	not significant
2488	GO_POSITIVE_REGULATION_OF_CARTILAGE_DEVELOPMENT	-1.37	0.94	0	0	0	not significant
2489	GO_RIBOSOMAL_SMALL_SUBUNIT_BINDING	-1.37	0.94	0	0	0	not significant
2490	GO_U4_SNRNA_BINDING	-1.37	0.94	0	0	0	not significant
2491	THUM_MIR21_TARGETS_HEART_DISEASE_DN	-1.37	0.93	0	0	0	not significant
2492	GO_REGULATION_OF_SYSTEMIC_ARTERIAL_BLOOD_PRESSURE_BY_RE	-1.37	0.92	0	0	0	not significant
2493	GO_RNA_POLYMERASE_I_COMPLEX	-1.37	0.92	0	0	0	not significant
2494	ZHAN_MULTIPLE_MYELOMA_SPIKED	-1.37	0.92	0	0	0	not significant
2495	GO_CARDIOBLAST_PROLIFERATION	-1.37	0.91	0	0	0	not significant

2496	GO_COMMITMENT_COMPLEX	-1.37	0.90	0	0	0	not significant
2497	GO_PHOSPHATIDYLINOSITOL_3_KINASE_REGULATORY_SUBUNIT_BINDIN	-1.37	0.90	0	0	0	not significant
2498	GO_EMBRYONIC_CAMERA_TYPE_EYE_FORMATION	-1.37	0.89	0	0	0	not significant
2499	GO_POSITIVE_REGULATION_OF_RNA_POLYMERASE_II_TRANSCRIPTION	-1.37	0.89	0	0	0	not significant
2500	CHEN_ETV5_TARGETS_SERTOLI	-1.37	0.88	0	0	0	not significant
2501	GO_PEPTIDE_HORMONE_PROCESSING	-1.37	0.87	0	0	0	not significant
2502	GO_TRNA_CYTOSINE_5__METHYLTRANSFERASE_ACTIVITY	-1.37	0.87	0	0	0	not significant
2503	MIKKELSEN_ES_ICP_WITH_H3K27ME3	-1.37	0.87	0	0	0	not significant
2504	GO_NEGATIVE_REGULATION_OF_MACROPHAGE_ACTIVATION	-1.37	0.86	0	0	0	not significant
2505	FEVR_CTNNB1_TARGETS_DN	-1.36	4.00	0	0	0	not significant
2506	GO_NEGATIVE_REGULATION_OF_CELL_CYCLE_PROCESS	-1.36	4.00	0	0	0	not significant
2507	GO_REGULATION_OF_INNATE_IMMUNE_RESPONSE	-1.36	4.00	0	0	0	not significant
2508	GO_RESPONSE_TO_NITROGEN_COMPOUND	-1.36	4.00	0	0	0	not significant
2509	GO_RIBONUCLEOTIDE_BINDING	-1.36	4.00	0	0	0	not significant
2510	HAMAI_APOPTOSIS_VIA_TRAIL_UP	-1.36	4.00	0	0	0	not significant
2511	KIM_BIPOLAR_DISORDER_OLIGODENDROCYTE_DENSITY_CORR_UP	-1.36	4.00	0	0	0	not significant
2512	PATIL_LIVER_CANCER	-1.36	4.00	0	0	0	not significant
2513	REACTOME_INFECTIOUS_DISEASE	-1.36	4.00	0	0	0	not significant
2514	REACTOME_TRANSPORT_OF_SMALL_MOLECULES	-1.36	4.00	0	0	0	not significant
2515	NABA_MATRISOME	-1.36	1.91	0	0	0	not significant
2516	VERHAAK_GLIOBLASTOMA_MESENCHYMAL	-1.36	1.86	0	0	0	not significant
2517	UEDA_PERIFERAL_CLOCK	-1.36	1.74	0	0	0	not significant
2518	MARKEY_RB1_ACUTE_LOF_DN	-1.36	1.61	0	0	0	not significant
2519	CHR11P15	-1.36	1.60	0	0	0	not significant
2520	GO_RESPONSE_TO_ACID_CHEMICAL	-1.36	1.60	0	0	0	not significant
2521	GO_PEPTIDE_BINDING	-1.36	1.59	0	0	0	not significant
2522	CHEMNITZ_RESPONSE_TO_PROSTAGLANDIN_E2_UP	-1.36	1.55	0	0	0	not significant
2523	GO_KINETOCHORE	-1.36	1.54	0	0	0	not significant
2524	OISHI_CHOLANGIOMA_STEM_CELL_LIKE_DN	-1.36	1.53	0	0	0	not significant
2525	GO_CIRCADIAN_RHYTHM	-1.36	1.50	0	0	0	not significant
2526	REACTOME_PROTEIN_UBIQUITINATION	-1.36	1.48	0	0	0	not significant
2527	GO_REGULATION_OF_CATION_CHANNEL_ACTIVITY	-1.36	1.46	0	0	0	not significant
2528	JIANG_HYPOXIA_CANCER	-1.36	1.35	0	0	0	not significant
2529	REACTOME_DISEASES_OF_GLYCOSYLATION	-1.36	1.30	0	0	0	not significant
2530	GO_CELL_DEATH_IN_RESPONSE_TO_OXIDATIVE_STRESS	-1.36	1.29	0	0	0	not significant
2531	GO_METAPHASE_PLATE_CONGRESSION	-1.36	1.22	0	0	0	not significant
2532	GO_ALPHA_AMINO_ACID_CATABOLIC_PROCESS	-1.36	1.21	0	0	0	not significant
2533	GO_CELLULAR_RESPONSE_TO_AMMONIUM_ION	-1.36	1.17	0	0	0	not significant
2534	PID_RAS_PATHWAY	-1.36	1.14	0	0	0	not significant
2535	GO_NEGATIVE_REGULATION_OF_ERK1_AND_ERK2_CASCADE	-1.36	1.13	0	0	0	not significant
2536	GO_TRANSCRIPTION_INITIATION_FROM_RNA_POLYMERASE_I_PROMOTE	-1.36	1.13	0	0	0	not significant
2537	TBK1.DN.48HRS_UP	-1.36	1.12	0	0	0	not significant
2538	GO_CELLULAR_RESPONSE_TO_GLUCOSE_STARVATION	-1.36	1.09	0	0	0	not significant
2539	ZHANG_ANTIVIRAL_RESPONSE_TO_RIBAVIRIN_DN	-1.36	1.08	0	0	0	not significant
2540	DAVICIONI_RHABDOMYOSARCOMA_PAX_FOXO1_FUSION_UP	-1.36	1.07	0	0	0	not significant
2541	GO_NEUTRAL_AMINO_ACID_TRANSPORT	-1.36	1.06	0	0	0	not significant
2542	GO_REGULATION_OF_ATTACHMENT_OF_SPINDLE_MICROTUBULES_TO_	-1.36	1.05	0	0	0	not significant
2543	SASAI_RESISTANCE_TO_NEOPLASTIC_TRANSFROMATION	-1.36	1.02	0	0	0	not significant
2544	EPPERT_CE_HSC_LSC	-1.36	1.01	0	0	0	not significant
2545	GO_COPPER_ION_TRANSPORT	-1.36	0.98	0	0	0	not significant
2546	GO_T_CELL_RECEPTOR_COMPLEX	-1.36	0.98	0	0	0	not significant
2547	GO_INTERFERON_ALPHA_PRODUCTION	-1.36	0.96	0	0	0	not significant
2548	REACTOME_GLUTATHIONE_CONJUGATION	-1.36	0.96	0	0	0	not significant
2549	REACTOME_PHOSPHORYLATION_SITE_MUTANTS_OF_CTNNB1_ARE_NOT	-1.36	0.95	0	0	0	not significant
2550	ALONSO_METASTASIS_EMT_UP	-1.36	0.93	0	0	0	not significant
2551	GAZDA_DIAMOND_BLACKFAN_ANEMIA_ERYTHROID_UP	-1.36	0.93	0	0	0	not significant
2552	GO_REGULATION_OF_MACROPHAGE_DERIVED_FOAM_CELL_DIFFERENT	-1.36	0.93	0	0	0	not significant
2553	MIKKELSEN_MEF_LCP_WITH_H3K27ME3	-1.36	0.93	0	0	0	not significant
2554	GO_EMBRYONIC_HINDLIMB_MORPHOGENESIS	-1.36	0.92	0	0	0	not significant
2555	KEGG_TRYPTOPHAN_METABOLISM	-1.36	0.92	0	0	0	not significant
2556	STOSSI_RESPONSE_TO_ESTRADIOL	-1.36	0.92	0	0	0	not significant
2557	GLINSKY_CANCER_DEATH_UP	-1.36	0.91	0	0	0	not significant
2558	GO_NEGATIVE_REGULATION_OF_PEPTIDYL_THREONINE_PHOSPHORYLA	-1.36	0.91	0	0	0	not significant
2559	CHR5Q32	-1.36	0.90	0	0	0	not significant
2560	GO_MAINTENANCE_OF_ORGANELLE_LOCATION	-1.36	0.90	0	0	0	not significant
2561	REACTOME_CYTOSOLIC_SULFONATION_OF_SMALL_MOLECULES	-1.36	0.90	0	0	0	not significant
2562	REACTOME_SYNTHESIS_OF_BILE_ACIDS_AND_BILE_SALTS_VIA_7ALPHA	-1.36	0.90	0	0	0	not significant
2563	GO_APOPTOTIC_DNA_FRAGMENTATION	-1.36	0.89	0	0	0	not significant
2564	GO_NEGATIVE_REGULATION_OF_LIPASE_ACTIVITY	-1.36	0.89	0	0	0	not significant
2565	IIZUKA_LIVER_CANCER_PROGRESSION_L0_L1_DN	-1.36	0.89	0	0	0	not significant
2566	REACTOME_CALCINEURIN_ACTIVATES_NFAT	-1.36	0.89	0	0	0	not significant
2567	GO_PYRUVATE_DEHYDROGENASE_ACTIVITY	-1.36	0.88	0	0	0	not significant
2568	GO_ASTROCYTE_PROJECTION	-1.36	0.87	0	0	0	not significant
2569	GO_CELLULAR_RESPONSE_TO_NICOTINE	-1.36	0.87	0	0	0	not significant
2570	GO_INDUCTION_OF_POSITIVE_CHEMOTAXIS	-1.36	0.87	0	0	0	not significant
2571	GO_DE_NOVO_IMP_BIOSYNTHETIC_PROCESS	-1.36	0.86	0	0	0	not significant
2572	LEI_HOXC8_TARGETS_DN	-1.36	0.86	0	0	0	not significant
2573	GO_ESTABLISHMENT_OF_EPITHELIAL_CELL_APICAL_BASAL_POLARITY	-1.36	0.85	0	0	0	not significant
2574	GO_TROPHOBLAST_CELL_MIGRATION	-1.36	0.85	0	0	0	not significant
2575	GO_AMIDINE_LYASE_ACTIVITY	-1.36	0.84	0	0	0	not significant
2576	GO_POSITIVE_REGULATION_OF_CELLULAR_EXTRAVASATION	-1.36	0.83	0	0	0	not significant
2577	REACTOME_G_PROTEIN_GATED_POTASSIUM_CHANNELS	-1.36	0.81	0	0	0	not significant
2578	GO_POSITIVE_REGULATION_OF_IMMUNE_SYSTEM_PROCESS	-1.35	4.00	0	0	0	not significant
2579	GO_REGULATION_OF_PROTEOLYSIS	-1.35	4.00	0	0	0	not significant
2580	GO_PROTEIN_DEPHOSPHORYLATION	-1.35	2.41	0	0	0	not significant
2581	REACTOME_CLASS_I_MHC_MEDIATED_ANTIGEN_PROCESSING_PRESENT	-1.35	2.35	0	0	0	not significant
2582	GO_CELLULAR_RESPONSE_TO_LIPID	-1.35	2.31	0	0	0	not significant
2583	REACTOME_NEUTROPHIL_DEGRANULATION	-1.35	2.30	0	0	0	not significant
2584	NFE2L2.V2	-1.35	2.16	0	0	0	not significant
2585	GO_CELL_CORTEX	-1.35	2.12	0	0	0	not significant
2586	GO_PHOSPHATASE_ACTIVITY	-1.35	1.85	0	0	0	not significant
2587	GO_MUSCLE_CONTRACTION	-1.35	1.63	0	0	0	not significant
2588	GO_EXTERNAL_SIDE_OF_PLASMA_MEMBRANE	-1.35	1.51	0	0	0	not significant
2589	GO_NEGATIVE_REGULATION_OF_SECRETION	-1.35	1.51	0	0	0	not significant
2590	AKT_UP.V1_UP	-1.35	1.49	0	0	0	not significant
2591	GO_LEARNING	-1.35	1.48	0	0	0	not significant
2592	DER_IFN_BETA_RESPONSE_UP	-1.35	1.46	0	0	0	not significant
2593	SENESE_HDAC2_TARGETS_UP	-1.35	1.46	0	0	0	not significant
2594	RUIZ_TNC_TARGETS_DN	-1.35	1.43	0	0	0	not significant
2595	WANG_ESOPHAGUS_CANCER_VS_NORMAL_UP	-1.35	1.37	0	0	0	not significant
2596	GO_CHROMOSOME_LOCALIZATION	-1.35	1.31	0	0	0	not significant
2597	APRELIKOVA_BRCA1_TARGETS	-1.35	1.20	0	0	0	not significant
2598	GO_TRANSCRIPTION_BY_RNA_POLYMERASE_III	-1.35	1.18	0	0	0	not significant
2599	LEE_LIVER_CANCER_MYC_TGFA_UP	-1.35	1.15	0	0	0	not significant
2600	GO_MUSCLE_HYPERTROPHY	-1.35	1.13	0	0	0	not significant
2601	GO_PEPTIDYL_PROLINE_MODIFICATION	-1.35	1.12	0	0	0	not significant
2602	GO_CARGO_RECEPTOR_ACTIVITY	-1.35	1.11	0	0	0	not significant
2603	NATSUME_RESPONSE_TO_INTERFERON_BETA_DN	-1.35	1.11	0	0	0	not significant
2604	PID_CDC42_REG_PATHWAY	-1.35	1.11	0	0	0	not significant

2605	GO_PLASMA_LIPOPROTEIN_PARTICLE_CLEARANCE	-1.35	1.09	0	0	0	not significant
2606	MCBRYAN_PUBERTAL_BREAST_6_7WK_DN	-1.35	1.08	0	0	0	not significant
2607	WHITEHURST_PACLITAXEL_SENSITIVITY	-1.35	1.08	0	0	0	not significant
2608	GO_PEPTIDE_TRANSMEMBRANE_TRANSPORTER_ACTIVITY	-1.35	1.06	0	0	0	not significant
2609	YAMASHITA_LIVER_CANCER_STEM_CELL_DN	-1.35	1.04	0	0	0	not significant
2610	GO_CLEAVAGE_INVOLVED_IN_RRNA_PROCESSING	-1.35	1.01	0	0	0	not significant
2611	GO_INNER_EAR_AUDITORY_RECEPTOR_CELL_DIFFERENTIATION	-1.35	0.99	0	0	0	not significant
2612	CAFFAREL_RESPONSE_TO_THC_DN	-1.35	0.98	0	0	0	not significant
2613	GO_CONDENSED_NUCLEAR_CHROMOSOME_CENTROMERIC_REGION	-1.35	0.98	0	0	0	not significant
2614	NIELSEN_SYNOVIAL_SARCOMA_DN	-1.35	0.98	0	0	0	not significant
2615	REACTOME_G_PROTEIN_BETA:GAMMA_SIGNALLING	-1.35	0.97	0	0	0	not significant
2616	GO_CELL_DIFFERENTIATION_INVOLVED_IN_KIDNEY_DEVELOPMENT	-1.35	0.95	0	0	0	not significant
2617	GO_HISTONE_KINASE_ACTIVITY	-1.35	0.95	0	0	0	not significant
2618	KIM_ALL_DISORDERS_CALB1_CORR_DN	-1.35	0.95	0	0	0	not significant
2619	KASLER_HDAC7_TARGETS_2_DN	-1.35	0.94	0	0	0	not significant
2620	GO_UREA_CYCLE	-1.35	0.92	0	0	0	not significant
2621	GO_MRNA_5_SPLICE_SITE_RECOGNITION	-1.35	0.91	0	0	0	not significant
2622	REACTOME_BILE_ACID_AND_BILE_SALT_METABOLISM	-1.35	0.91	0	0	0	not significant
2623	GO_DETERMINATION_OF_ADULT_LIFESPAN	-1.35	0.89	0	0	0	not significant
2624	HOLLEMAN_DAUNORUBICIN_ALL_UP	-1.35	0.89	0	0	0	not significant
2625	XU_HGF_TARGETS_INDUCED_BY_AKT1_48HR_DN	-1.35	0.89	0	0	0	not significant
2626	GLI1_UP.V1_DN	-1.35	0.88	0	0	0	not significant
2627	GO_CONDENSED_NUCLEAR_CHROMOSOME_KINETOCHORE	-1.35	0.88	0	0	0	not significant
2628	GO_NUCLEAR_PORE_NUCLEAR_BASKET	-1.35	0.88	0	0	0	not significant
2629	GO_PLATELET_DENSE_TUBULAR_NETWORK_MEMBRANE	-1.35	0.88	0	0	0	not significant
2630	TURASHVILI_BREAST_NORMAL_DUCTAL_VS_LOBULAR_DN	-1.35	0.88	0	0	0	not significant
2631	GO_FATTY_ACID_LIGASE_ACTIVITY	-1.35	0.87	0	0	0	not significant
2632	GO_POSITIVE_REGULATION_OF_CELL_PROLIFERATION_INVOLVED_IN_H	-1.35	0.87	0	0	0	not significant
2633	GO_POSITIVE_REGULATION_OF_CHONDROCYTE_DIFFERENTIATION	-1.35	0.87	0	0	0	not significant
2634	GO_UV_PROTECTION	-1.35	0.87	0	0	0	not significant
2635	GO_CELLULAR_RESPONSE_TO_CAFFEINE	-1.35	0.86	0	0	0	not significant
2636	GO_NEURON_NEURON_SYNAPTIC_TRANSMISSION	-1.35	0.86	0	0	0	not significant
2637	GO_PROTEIN_OXIDATION	-1.35	0.86	0	0	0	not significant
2638	SETLUR_PROSTATE_CANCER_TMPRSS2_ERG_FUSION_DN	-1.35	0.86	0	0	0	not significant
2639	GO_CHEMOKINE_BIOSYNTHETIC_PROCESS	-1.35	0.85	0	0	0	not significant
2640	GO_MITOCHONDRIAL_INTERMEMBRANE_SPACE_PROTEIN_TRANSPORTE	-1.35	0.85	0	0	0	not significant
2641	GO_POSITIVE_REGULATION_OF_NON_CANONICAL_WNT_SIGNALING_PAT	-1.35	0.84	0	0	0	not significant
2642	GO_ER_MEMBRANE_INSERTION_COMPLEX	-1.35	0.83	0	0	0	not significant
2643	GO_DEPYRIMIDINATION	-1.35	0.81	0	0	0	not significant
2644	GO_POSITIVE_REGULATION_OF_HUMORAL_IMMUNE_RESPONSE	-1.35	0.79	0	0	0	not significant
2645	GO_CATION_TRANSMEMBRANE_TRANSPORT	-1.34	4.00	0	0	0	not significant
2646	GO_CELLULAR_RESPONSE_TO_OXYGEN_CONTAINING_COMPOUND	-1.34	4.00	0	0	0	not significant
2647	GO_POSITIVE_REGULATION_OF_DEFENSE_RESPONSE	-1.34	4.00	0	0	0	not significant
2648	GO_REGULATION_OF_MITOTIC_CELL_CYCLE	-1.34	4.00	0	0	0	not significant
2649	GRADE_COLON_CANCER_UP	-1.34	4.00	0	0	0	not significant
2650	MARTENS_TRETINOIN_RESPONSE_UP	-1.34	1.97	0	0	0	not significant
2651	GO_PHOSPHOPROTEIN_PHOSPHATASE_ACTIVITY	-1.34	1.80	0	0	0	not significant
2652	GO_DNA_TEMPLATED_TRANSCRIPTION_ELONGATION	-1.34	1.72	0	0	0	not significant
2653	GO_CELLULAR_RESPONSE_TO_CARBOHYDRATE_STIMULUS	-1.34	1.43	0	0	0	not significant
2654	REACTOME_NEGATIVE_EPIGENETIC_REGULATION_OF_RRNA_EXPRESSI(	-1.34	1.41	0	0	0	not significant
2655	HALLMARK_PROTEIN_SECRETION	-1.34	1.37	0	0	0	not significant
2656	GO_CONDENSED_CHROMOSOME_CENTROMERIC_REGION	-1.34	1.33	0	0	0	not significant
2657	GO_CELLULAR_COMPONENT_ASSEMBLY_INVOLVED_IN_MORPHOGENES	-1.34	1.25	0	0	0	not significant
2658	PEART_HDAC_PROLIFERATION_CLUSTER_UP	-1.34	1.22	0	0	0	not significant
2659	GO_VESICLE_TARGETING	-1.34	1.21	0	0	0	not significant
2660	LIM_MAMMARY_LUMINAL_MATURE_UP	-1.34	1.21	0	0	0	not significant
2661	GO_REGULATION_OF_VIRAL_TRANSCRIPTION	-1.34	1.20	0	0	0	not significant
2662	GO_VESICLE_COAT	-1.34	1.19	0	0	0	not significant
2663	GO_KINASE_ACTIVATOR_ACTIVITY	-1.34	1.18	0	0	0	not significant
2664	OHGUCHI_LIVER_HNF4A_TARGETS_DN	-1.34	1.17	0	0	0	not significant
2665	REACTOME_RHO_GTPASES_ACTIVATE_PKNS	-1.34	1.15	0	0	0	not significant
2666	DURCHDEWALD_SKIN_CARCINOGENESIS_UP	-1.34	1.13	0	0	0	not significant
2667	PUJANA_BREAST_CANCER_WITH_BRCA1_MUTATED_UP	-1.34	1.11	0	0	0	not significant
2668	BOYLAN_MULTIPLE_MYELOMA_PCA1_UP	-1.34	1.10	0	0	0	not significant
2669	GO_HIPPOCAMPUS_DEVELOPMENT	-1.34	1.10	0	0	0	not significant
2670	GO_PROTEIN_TRANSMEMBRANE_IMPORT_INTO_INTRACELLULAR_ORGA	-1.34	1.10	0	0	0	not significant
2671	GO_RIBOSOME_BINDING	-1.34	1.09	0	0	0	not significant
2672	GO_POSITIVE_REGULATION_OF_TELOMERE_MAINTENANCE	-1.34	1.08	0	0	0	not significant
2673	HEIDENBLAD_AMPLICON_12P11_12_UP	-1.34	1.08	0	0	0	not significant
2674	BIOCARTA_VEGF_PATHWAY	-1.34	1.06	0	0	0	not significant
2675	GO_RESPONSE_TO_AMINO_ACID_STARVATION	-1.34	1.06	0	0	0	not significant
2676	REACTOME_EPHB_MEDIATED_FORWARD_SIGNALING	-1.34	1.03	0	0	0	not significant
2677	GO_POSITIVE_REGULATION_OF_GLIOGENESIS	-1.34	1.01	0	0	0	not significant
2678	GEISS_RESPONSE_TO_DSRNA_UP	-1.34	1.00	0	0	0	not significant
2679	REACTOME_SIGNALING_BY_RETINOIC_ACID	-1.34	0.98	0	0	0	not significant
2680	BIOCARTA_FMLP_PATHWAY	-1.34	0.96	0	0	0	not significant
2681	GO_MHC_PROTEIN_COMPLEX_BINDING	-1.34	0.96	0	0	0	not significant
2682	GO_RESPONSE_TO_LITHIUM_ION	-1.34	0.95	0	0	0	not significant
2683	GO_A_BAND	-1.34	0.93	0	0	0	not significant
2684	GO_RESPONSE_TO_INCREASED_OXYGEN_LEVELS	-1.34	0.92	0	0	0	not significant
2685	KEGG_OLFACTORY_TRANSDUCTION	-1.34	0.92	0	0	0	not significant
2686	GO_REGULATION_OF_HORMONE_METABOLIC_PROCESS	-1.34	0.91	0	0	0	not significant
2687	GO_RESPONSE_TO_FLUID_SHEAR_STRESS	-1.34	0.91	0	0	0	not significant
2688	CAFFAREL_RESPONSE_TO_THC_24HR_3_DN	-1.34	0.89	0	0	0	not significant
2689	GO_ACTIN_MONOMER_BINDING	-1.34	0.89	0	0	0	not significant
2690	GO_REGULATION_OF_HIGH_VOLTAGE_GATED_CALCIUM_CHANNEL_ACT	-1.34	0.89	0	0	0	not significant
2691	GO_CELL_PROLIFERATION_INVOLVED_IN_KIDNEY_DEVELOPMENT	-1.34	0.88	0	0	0	not significant
2692	GO_EPITHELIAL_STRUCTURE_MAINTENANCE	-1.34	0.88	0	0	0	not significant
2693	GO_NEGATIVE_REGULATION_OF_ADHERENS_JUNCTION_ORGANIZATION	-1.34	0.88	0	0	0	not significant
2694	GO_NEGATIVE_REGULATION_OF_EMBRYONIC_DEVELOPMENT	-1.34	0.88	0	0	0	not significant
2695	GO_RELAXATION_OF_MUSCLE	-1.34	0.88	0	0	0	not significant
2696	BAKER_HEMATOPOIESIS_STAT3_TARGETS	-1.34	0.87	0	0	0	not significant
2697	GO_NONRIBOSOMAL_PEPTIDE_BIOSYNTHETIC_PROCESS	-1.34	0.87	0	0	0	not significant
2698	GO_PROTEASOME_BINDING	-1.34	0.87	0	0	0	not significant
2699	MATTHEWS_SKIN_CARCINOGENESIS_VIA_JUN	-1.34	0.87	0	0	0	not significant
2700	GO_REGULATION_OF_POTASSIUM_ION_IMPORT	-1.34	0.86	0	0	0	not significant
2701	HUNSBERGER_EXERCISE_REGULATED_GENES	-1.34	0.86	0	0	0	not significant
2702	GO_CHOLESTEROL_STORAGE	-1.34	0.85	0	0	0	not significant
2703	GO_NUCLEOTIDE_DIPHOSPHATASE_ACTIVITY	-1.34	0.84	0	0	0	not significant
2704	GO_POSITIVE_REGULATION_OF_TRANSCRIPTION_INITIATION_FROM_RNA	-1.34	0.84	0	0	0	not significant
2705	GO_DSRNA_TRANSPORT	-1.34	0.83	0	0	0	not significant
2706	GO_MAST_CELL_GRANULE	-1.34	0.83	0	0	0	not significant
2707	GO_POSITIVE_REGULATION_OF_SPINDLE_CHECKPOINT	-1.34	0.83	0	0	0	not significant
2708	GO_RECEPTOR_SERINE_THREONINE_KINASE_BINDING	-1.34	0.83	0	0	0	not significant
2709	REACTOME_HUR_ELAVL1_BINDS_AND_STABILIZES_MRNA	-1.34	0.83	0	0	0	not significant
2710	GO_ATP_DEPENDENT_PEPTIDASE_ACTIVITY	-1.34	0.82	0	0	0	not significant
2711	GO_GLUTATHIONE_DISULFIDE_OXIDOREDUCTASE_ACTIVITY	-1.34	0.82	0	0	0	not significant
2712	GO_NEGATIVE_REGULATION_OF_TYPE_2_IMMUNE_RESPONSE	-1.34	0.82	0	0	0	not significant
2713	GO_CELLULAR_RESPONSE_TO_LITHIUM_ION	-1.34	0.81	0	0	0	not significant

2714	GO_MEIOTIC_CELL_CYCLE_CHECKPOINT	-1.34	0.81	0	0	0	not significant
2715	GO_CALCINEURIN_COMPLEX	-1.34	0.80	0	0	0	not significant
2716	GO_COSTAMERE	-1.34	0.80	0	0	0	not significant
2717	MATZUK_IMPLANTATION_AND_UTERINE	-1.34	0.80	0	0	0	not significant
2718	GO_MIRNA_CATABOLIC_PROCESS	-1.34	0.79	0	0	0	not significant
2719	GO_MITOTIC_SPINDLE_MIDZONE_ASSEMBLY	-1.34	0.79	0	0	0	not significant
2720	MIKKELSEN_MCV6_LCP_WITH_H3K27ME3	-1.34	0.77	0	0	0	not significant
2721	GO_ANIMAL_ORGAN_MORPHOGENESIS	-1.33	4.00	0	0	0	not significant
2722	GO_EPITHELIUM_DEVELOPMENT	-1.33	4.00	0	0	0	not significant
2723	GO_TISSUE_MORPHOGENESIS	-1.33	4.00	0	0	0	not significant
2724	GO_TUBE_DEVELOPMENT	-1.33	2.26	0	0	0	not significant
2725	GO_RESPONSE_TO_REACTIVE_OXYGEN_SPECIES	-1.33	2.00	0	0	0	not significant
2726	SHAFFER_IRF4_TARGETS_IN_MYELOMA_VS_MATURE_B_LYMPHOCYTE	-1.33	1.59	0	0	0	not significant
2727	PENG_LEUCINE_DEPRIVATION_UP	-1.33	1.37	0	0	0	not significant
2728	KAN_RESPONSE_TO_ARSENIC_TRIOXIDE	-1.33	1.32	0	0	0	not significant
2729	REACTOME_GENE_SILENCING_BY_RNA	-1.33	1.31	0	0	0	not significant
2730	BMI1_DN_MEL18_DN.V1_DN	-1.33	1.25	0	0	0	not significant
2731	PTEN_DN.V1_UP	-1.33	1.25	0	0	0	not significant
2732	GO_COPII_COATED_VESICLE_BUDDING	-1.33	1.23	0	0	0	not significant
2733	GO_TRANSCRIPTION_COUPLED_NUCLEOTIDE_EXCISION_REPAIR	-1.33	1.20	0	0	0	not significant
2734	TAKEDA_TARGETS_OF_NUP98_HOXA9_FUSION_10D_DN	-1.33	1.17	0	0	0	not significant
2735	GO_DNA_GEOMETRIC_CHANGE	-1.33	1.16	0	0	0	not significant
2736	GO_REGULATION_OF_PROTEIN_LOCALIZATION_TO_PLASMA_MEMBRANI	-1.33	1.16	0	0	0	not significant
2737	LABBE_TARGETS_OF_TGFB1_AND_WNT3A_DN	-1.33	1.15	0	0	0	not significant
2738	GO_INTRACELLULAR_PROTEIN_TRANSMEMBRANE_TRANSPORT	-1.33	1.13	0	0	0	not significant
2739	ANDERSEN_LIVER_CANCER_KRT19_DN	-1.33	1.09	0	0	0	not significant
2740	GO_FATTY_ACID_TRANSPORT	-1.33	1.08	0	0	0	not significant
2741	REACTOME_DNA_METHYLATION	-1.33	1.08	0	0	0	not significant
2742	OUILLETTE_CLL_13Q14_DELETION_DN	-1.33	1.07	0	0	0	not significant
2743	GO_REGULATION_OF_STRIATED_MUSCLE_CONTRACTION	-1.33	1.06	0	0	0	not significant
2744	BILANGES_SERUM_SENSITIVE_VIA_TSC2	-1.33	1.00	0	0	0	not significant
2745	GO_GLIAL_CELL_ACTIVATION	-1.33	0.99	0	0	0	not significant
2746	BEIER_GLIOMA_STEM_CELL_UP	-1.33	0.98	0	0	0	not significant
2747	GO_ACTIN_FILAMENT_DEPOLYMERIZATION	-1.33	0.97	0	0	0	not significant
2748	GO_RESPONSE_TO_TESTOSTERONE	-1.33	0.96	0	0	0	not significant
2749	KEEN_RESPONSE_TO_ROSIGLITAZONE_UP	-1.33	0.95	0	0	0	not significant
2750	GO_POSITIVE_REGULATION_OF_BMP_SIGNALING_PATHWAY	-1.33	0.94	0	0	0	not significant
2751	BARIS_THYROID_CANCER_UP	-1.33	0.93	0	0	0	not significant
2752	GO_LOW_DENSITY_LIPOPROTEIN_RECEPTOR_PARTICLE_METABOLIC_P	-1.33	0.91	0	0	0	not significant
2753	EBAUER_MYOGENIC_TARGETS_OF_PAX3_FOXO1_FUSION	-1.33	0.90	0	0	0	not significant
2754	REACTOME_ELASTIC_FIBRE_FORMATION	-1.33	0.90	0	0	0	not significant
2755	GO_LUNG_EPITHELIUM_DEVELOPMENT	-1.33	0.89	0	0	0	not significant
2756	GO_PEPTIDE_ANTIGEN_BINDING	-1.33	0.89	0	0	0	not significant
2757	GO_PHOTOPERIODISM	-1.33	0.88	0	0	0	not significant
2758	HASLINGER_B_CLL_WITH_17P13_DELETION	-1.33	0.88	0	0	0	not significant
2759	GO_REGULATION_OF_ANOIKIS	-1.33	0.87	0	0	0	not significant
2760	GO_AZOLE_TRANSPORT	-1.33	0.86	0	0	0	not significant
2761	GO_SERINE_FAMILY_AMINO_ACID_BIOSYNTHETIC_PROCESS	-1.33	0.86	0	0	0	not significant
2762	GO_SWR1_COMPLEX	-1.33	0.86	0	0	0	not significant
2763	RAMPON_ENRICHED_LEARNING_ENVIRONMENT_LATE_UP	-1.33	0.84	0	0	0	not significant
2764	GO_LONG_CHAIN_FATTY_ACYL_COA_BIOSYNTHETIC_PROCESS	-1.33	0.83	0	0	0	not significant
2765	KOINUMA_COLON_CANCER_MSI_UP	-1.33	0.83	0	0	0	not significant
2766	GO_BOX_C_D_SNORNP_ASSEMBLY	-1.33	0.82	0	0	0	not significant
2767	GO_IMMUNE_RESPONSE_TO_TUMOR_CELL	-1.33	0.82	0	0	0	not significant
2768	GO_LOW_DENSITY_LIPOPROTEIN_PARTICLE_RECEPTOR_CATABOLIC_P	-1.33	0.82	0	0	0	not significant
2769	GO_RENAL_SODIUM_ION_TRANSPORT	-1.33	0.82	0	0	0	not significant
2770	GALI_TP53_TARGETS_APOPTOTIC_DN	-1.33	0.81	0	0	0	not significant
2771	GO_KINETOCHORE_MICROTUBULE	-1.33	0.81	0	0	0	not significant
2772	GO_REGULATION_OF_GROWTH_RATE	-1.33	0.81	0	0	0	not significant
2773	MACLACHLAN_BRCA1_TARGETS_DN	-1.33	0.81	0	0	0	not significant
2774	GO_CYTOSKELETAL_ADAPTOR_ACTIVITY	-1.33	0.80	0	0	0	not significant
2775	GO_PROTEIN_SERINE_THREONINE_PHOSPHATASE_INHIBITOR_ACTIVITY	-1.33	0.80	0	0	0	not significant
2776	MAINA_VHL_TARGETS_DN	-1.33	0.80	0	0	0	not significant
2777	GO_RESPONSE_TO_GRANULOCYTE_MACROPHAGE_COLONY_STIMULAT	-1.33	0.79	0	0	0	not significant
2778	GO_POSITIVE_REGULATION_OF_VIRAL_ENTRY_INTO_HOST_CELL	-1.33	0.78	0	0	0	not significant
2779	GO_INTRINSIC_APOPTOTIC_SIGNALING_PATHWAY_IN_RESPONSE_TO_HY	-1.33	0.75	0	0	0	not significant
2780	SCIAN_CELL_CYCLE_TARGETS_OF_TP53_AND_TP73_UP	-1.33	0.73	0	0	0	not significant
2781	GO_CYTOSKELETAL_PROTEIN_BINDING	-1.32	4.00	0	0	0	not significant
2782	GO_NUCLEOLUS	-1.32	4.00	0	0	0	not significant
2783	ROME_INSULIN_TARGETS_IN_MUSCLE_UP	-1.32	4.00	0	0	0	not significant
2784	GO_DEFENSE_RESPONSE_TO_OTHER_ORGANISM	-1.32	2.39	0	0	0	not significant
2785	GO_ATPASE_ACTIVITY	-1.32	2.35	0	0	0	not significant
2786	GO_RESPONSE_TO_WOUNDING	-1.32	2.32	0	0	0	not significant
2787	GO_TUBE_MORPHOGENESIS	-1.32	2.28	0	0	0	not significant
2788	GO_REGULATION_OF_CHROMOSOME_ORGANIZATION	-1.32	2.07	0	0	0	not significant
2789	GO_GUANYL_NUCLEOTIDE_BINDING	-1.32	2.04	0	0	0	not significant
2790	GO_SPINDLE	-1.32	1.94	0	0	0	not significant
2791	GO_REGULATION_OF_PEPTIDASE_ACTIVITY	-1.32	1.93	0	0	0	not significant
2792	GO_GLAND_DEVELOPMENT	-1.32	1.82	0	0	0	not significant
2793	HEDENFALK_BREAST_CANCER_BRCA1_VS_BRCA2	-1.32	1.77	0	0	0	not significant
2794	GO_CELL_SURFACE	-1.32	1.71	0	0	0	not significant
2795	GO_AMIDE_BINDING	-1.32	1.69	0	0	0	not significant
2796	CAIRO_HEPATOBLASTOMA_DN	-1.32	1.62	0	0	0	not significant
2797	LINDGREN_BLADDER_CANCER_WITH_LOH_IN_CHR9Q	-1.32	1.58	0	0	0	not significant
2798	KEGG_CHEMOKINE_SIGNALING_PATHWAY	-1.32	1.55	0	0	0	not significant
2799	GO_STRIATED_MUSCLE_CELL_DIFFERENTIATION	-1.32	1.48	0	0	0	not significant
2800	CHUNG_BLISTER_CYTOTOXICITY_UP	-1.32	1.40	0	0	0	not significant
2801	ESC_J1_UP_LATE.V1_DN	-1.32	1.37	0	0	0	not significant
2802	GO_POSITIVE_REGULATION_OF_WNT_SIGNALING_PATHWAY	-1.32	1.35	0	0	0	not significant
2803	GO_SINGLE_STRANDED_DNA_BINDING	-1.32	1.34	0	0	0	not significant
2804	BROWNE_HCMV_INFECTION_48HR_UP	-1.32	1.29	0	0	0	not significant
2805	REACTOME_TRANSCRIPTION_COUPLED_NUCLEOTIDE_EXCISION_REPAIR	-1.32	1.28	0	0	0	not significant
2806	GO_REGULATION_OF_PROTEIN_LOCALIZATION_TO_CELL_PERIPHERY	-1.32	1.20	0	0	0	not significant
2807	DER_IFN_GAMMA_RESPONSE_UP	-1.32	1.17	0	0	0	not significant
2808	TBK1.DN.48HRS_DN	-1.32	1.13	0	0	0	not significant
2809	GO_LYMPHOCYTE_MIGRATION	-1.32	1.09	0	0	0	not significant
2810	FLECHNER_PBL_KIDNEY_TRANSPLANT_OK_VS_DONOR_DN	-1.32	1.06	0	0	0	not significant
2811	GO_RESPONSE_TO_BRONCHODILATOR	-1.32	1.05	0	0	0	not significant
2812	COATES_MACROPHAGE_M1_VS_M2_DN	-1.32	1.04	0	0	0	not significant
2813	GO_UBIQUITIN_LIKE_PROTEIN_CONJUGATING_ENZYME_ACTIVITY	-1.32	0.98	0	0	0	not significant
2814	REACTOME_PINK_PARKIN_MEDIATED_MITOPHAGY	-1.32	0.97	0	0	0	not significant
2815	KEGG_MTOR_SIGNALING_PATHWAY	-1.32	0.96	0	0	0	not significant
2816	REACTOME_AMINO_ACID_TRANSPORT_ACROSS_THE_PLASMA_MEMBRA	-1.32	0.96	0	0	0	not significant
2817	GO_DISULFIDE_OXIDOREDUCTASE_ACTIVITY	-1.32	0.95	0	0	0	not significant
2818	GO_RNA_STABILIZATION	-1.32	0.95	0	0	0	not significant
2819	REACTOME_MITOCHONDRIAL_CALCIUM_ION_TRANSPORT	-1.32	0.94	0	0	0	not significant
2820	CEBALLOS_TARGETS_OF_TP53_AND_MYC_UP	-1.32	0.93	0	0	0	not significant
2821	GO_NEGATIVE_REGULATION_OF_PEPTIDYL_SERINE_PHOSPHORYLATIOI	-1.32	0.91	0	0	0	not significant
2822	GO_RNA_POLYMERASE_II_GENERAL_TRANSCRIPTION_INITIATION_FACT(	-1.32	0.91	0	0	0	not significant

2823	GO_GOLGI_LUMEN	-1.32	0.90	0	0	0	not significant
2824	NOUSHMEHR_GBM_SILENCED_BY_METHYLATION	-1.32	0.90	0	0	0	not significant
2825	GO_FATTY_ACID_DERIVATIVE_BINDING	-1.32	0.89	0	0	0	not significant
2826	GO_CULLIN_FAMILY_PROTEIN_BINDING	-1.32	0.88	0	0	0	not significant
2827	HALMOS_CEBPA_TARGETS_DN	-1.32	0.88	0	0	0	not significant
2828	REACTOME_BRANCHED_CHAIN_AMINO_ACID_CATABOLISM	-1.32	0.88	0	0	0	not significant
2829	REACTOME_VEGFR2_MEDIATED_CELL_PROLIFERATION	-1.32	0.88	0	0	0	not significant
2830	GO_OXIDOREDUCTASE_ACTIVITY_ACTING_ON_THE_CH_NH_GROUP_OF	-1.32	0.87	0	0	0	not significant
2831	GO_REGULATION_OF_CELLULAR_EXTRAVASATION	-1.32	0.87	0	0	0	not significant
2832	BERENJENO_TRANSFORMED_BY_RHOA_REVERSIBLY_DN	-1.32	0.86	0	0	0	not significant
2833	GO_POSITIVE_REGULATION_OF_LIPID_STORAGE	-1.32	0.86	0	0	0	not significant
2834	GO_POSITIVE_REGULATION_OF_TRIGLYCERIDE_BIOSYNTHETIC_PROCE	-1.32	0.86	0	0	0	not significant
2835	GO_RIBOSOMAL_LARGE_SUBUNIT_BINDING	-1.32	0.85	0	0	0	not significant
2836	GO_ANTIGEN_PROCESSING_AND_PRESENTATION_OF_PEPTIDE_ANTIGEI	-1.32	0.84	0	0	0	not significant
2837	GO_PRENYLTRANSFERASE_ACTIVITY	-1.32	0.84	0	0	0	not significant
2838	CHUANG_OXIDATIVE_STRESS_RESPONSE_UP	-1.32	0.83	0	0	0	not significant
2839	DEMAGALHAES_AGING_DN	-1.32	0.83	0	0	0	not significant
2840	GO_LIPOPHAGY	-1.32	0.83	0	0	0	not significant
2841	GO_SECONDARY_METABOLITE_BIOSYNTHETIC_PROCESS	-1.32	0.82	0	0	0	not significant
2842	BIOCARTA_CDC25_PATHWAY	-1.32	0.81	0	0	0	not significant
2843	GO_CELLULAR_RESPONSE_TO_NITROGEN_STARVATION	-1.32	0.81	0	0	0	not significant
2844	GO_DNA_TOPOLOGICAL_CHANGE	-1.32	0.81	0	0	0	not significant
2845	GO_NEGATIVE_REGULATION_OF_CAMP_DEPENDENT_PROTEIN_KINASE_	-1.32	0.81	0	0	0	not significant
2846	BIOCARTA_FREE_PATHWAY	-1.32	0.80	0	0	0	not significant
2847	GO_TRANSFERASE_ACTIVITY_TRANSFERRING_NITROGENOUS_GROUPS	-1.32	0.80	0	0	0	not significant
2848	BIOCARTA_CACAM_PATHWAY	-1.32	0.79	0	0	0	not significant
2849	GO_NEPHRON_TUBULE_EPITHELIAL_CELL_DIFFERENTIATION	-1.32	0.79	0	0	0	not significant
2850	GO_S_ADENOSYL_L_METHIONINE_BINDING	-1.32	0.79	0	0	0	not significant
2851	PHESSE_TARGETS_OF_APC_AND_MBD2_DN	-1.32	0.78	0	0	0	not significant
2852	GO_EPITHELIAL_CELL_DIFFERENTIATION_INVOLVED_IN_PROSTATE_GLA	-1.32	0.77	0	0	0	not significant
2853	GO_PRIMARY_ALCOHOL_CATABOLIC_PROCESS	-1.32	0.77	0	0	0	not significant
2854	REACTOME_SHC_RELATED_EVENTS_TRIGGERED_BY_IGF1R	-1.32	0.77	0	0	0	not significant
2855	WANG_CLASSIC_ADIPOGENIC_TARGETS_OF_PPARG	-1.32	0.77	0	0	0	not significant
2856	GO_DEPHOSPHORYLATION_OF_RNA_POLYMERASE_II_C_TERMINAL_DOI	-1.32	0.74	0	0	0	not significant
2857	WU_APOPTOSIS_BY_CDKN1A_NOT_VIA_TP53	-1.32	0.74	0	0	0	not significant
2858	GO_CIRCULATORY_SYSTEM_DEVELOPMENT	-1.31	4.00	0	0	0	not significant
2859	GO_ESTABLISHMENT_OF_ORGANELLE_LOCALIZATION	-1.31	4.00	0	0	0	not significant
2860	GO_MOLECULAR_FUNCTION_REGULATOR	-1.31	4.00	0	0	0	not significant
2861	GO_MYELOID_LEUKOCYTE_ACTIVATION	-1.31	4.00	0	0	0	not significant
2862	GO_RESPONSE_TO_BIOTIC_STIMULUS	-1.31	4.00	0	0	0	not significant
2863	GO_CALCIUM_ION_BINDING	-1.31	2.35	0	0	0	not significant
2864	REACTOME_ESR_MEDIATED_SIGNALING	-1.31	1.98	0	0	0	not significant
2865	GO_RUFFLE	-1.31	1.75	0	0	0	not significant
2866	GO_FIBRILLAR_CENTER	-1.31	1.66	0	0	0	not significant
2867	GO_RESPONSE_TO_STARVATION	-1.31	1.57	0	0	0	not significant
2868	GO_ESTABLISHMENT_OR_MAINTENANCE_OF_CELL_POLARITY	-1.31	1.55	0	0	0	not significant
2869	GO_INTERACTION_WITH_HOST	-1.31	1.55	0	0	0	not significant
2870	GO_FATTY_ACID_METABOLIC_PROCESS	-1.31	1.44	0	0	0	not significant
2871	MEK_UP.V1_UP	-1.31	1.43	0	0	0	not significant
2872	SHETH_LIVER_CANCER_VS_TXNIP_LOSS_PAM4	-1.31	1.43	0	0	0	not significant
2873	DARWICHE_SKIN_TUMOR_PROMOTER_UP	-1.31	1.35	0	0	0	not significant
2874	GO_CALCIUM_ION_TRANSMEMBRANE_IMPORT_INTO_CYTOSOL	-1.31	1.35	0	0	0	not significant
2875	GO_RNA_PHOSPHODIESTER_BOND_HYDROLYSIS	-1.31	1.35	0	0	0	not significant
2876	GO_SULFUR_COMPOUND_BINDING	-1.31	1.30	0	0	0	not significant
2877	BROWN_MYELOID_CELL_DEVELOPMENT_UP	-1.31	1.23	0	0	0	not significant
2878	REACTOME_INTERFERON_GAMMA_SIGNALING	-1.31	1.21	0	0	0	not significant
2879	FOSTER_KDM1A_TARGETS_UP	-1.31	1.20	0	0	0	not significant
2880	REACTOME_NONHOMOLOGOUS_END_JOINING_NHEJ	-1.31	1.17	0	0	0	not significant
2881	UEDA_CENTRAL_CLOCK	-1.31	1.17	0	0	0	not significant
2882	GO_RESPONSE_TO_ALKALOID	-1.31	1.14	0	0	0	not significant
2883	ZHU_CMV_ALL_DN	-1.31	1.13	0	0	0	not significant
2884	GO_ATP_DEPENDENT_CHROMATIN_REMODELING	-1.31	1.10	0	0	0	not significant
2885	GO_DNA_PACKAGING_COMPLEX	-1.31	1.10	0	0	0	not significant
2886	HOOI_ST7_TARGETS_DN	-1.31	1.06	0	0	0	not significant
2887	GO_BONE_MORPHOGENESIS	-1.31	1.05	0	0	0	not significant
2888	GO_RENAL_TUBULE_DEVELOPMENT	-1.31	1.05	0	0	0	not significant
2889	GO_POSITIVE_REGULATION_OF_T_CELL_PROLIFERATION	-1.31	1.04	0	0	0	not significant
2890	BROWNE_INTERFERON_RESPONSIVE_GENES	-1.31	1.03	0	0	0	not significant
2891	GO_REGULATION_OF_SPINDLE_ORGANIZATION	-1.31	1.03	0	0	0	not significant
2892	REACTOME_FORMATION_OF_INCISION_COMPLEX_IN_GG_NER	-1.31	1.03	0	0	0	not significant
2893	HAHTOLA_SEZARY_SYNDROM_UP	-1.31	1.02	0	0	0	not significant
2894	GENTILE_UV_RESPONSE_CLUSTER_D4	-1.31	1.01	0	0	0	not significant
2895	HU_GENOTOXIN_ACTION_DIRECT_VS_INDIRECT_24HR	-1.31	1.00	0	0	0	not significant
2896	MEISSNER_NPC_HCP_WITH_H3K4ME3_AND_H3K27ME3	-1.31	0.98	0	0	0	not significant
2897	GO_POSITIVE_REGULATION_OF_REPRODUCTIVE_PROCESS	-1.31	0.95	0	0	0	not significant
2898	VANDESLUIS_COMMD1_TARGETS_GROUP_3_UP	-1.31	0.95	0	0	0	not significant
2899	GO_REGULATION_OF_MONOOXYGENASE_ACTIVITY	-1.31	0.94	0	0	0	not significant
2900	GUTIERREZ_MULTIPLE_MYELOMA_UP	-1.31	0.94	0	0	0	not significant
2901	GO_EMBRYONIC_CAMERA_TYPE_EYE_DEVELOPMENT	-1.31	0.93	0	0	0	not significant
2902	GO_NEGATIVE_REGULATION_OF_LYMPHOCYTE_APOPTOTIC_PROCESS	-1.31	0.93	0	0	0	not significant
2903	MODY_HIPPOCAMPUS_NEONATAL	-1.31	0.93	0	0	0	not significant
2904	GO_NUCLEOTIDE_EXCISION_REPAIR_PREINCISION_COMPLEX_STABILIZAT	-1.31	0.92	0	0	0	not significant
2905	GO_REGULATION_OF_VASCULAR_ENDOTHELIAL_GROWTH_FACTOR_RE	-1.31	0.92	0	0	0	not significant
2906	PID_VEGFR1_PATHWAY	-1.31	0.92	0	0	0	not significant
2907	CHEOK_RESPONSE_TO_MERCAPTOPURINE_AND_HD_MTX_DN	-1.31	0.91	0	0	0	not significant
2908	GO_ANTIBIOTIC_METABOLIC_PROCESS	-1.31	0.91	0	0	0	not significant
2909	WU_HBX_TARGETS_3_UP	-1.31	0.91	0	0	0	not significant
2910	GO_CELL_DEATH_IN_RESPONSE_TO_HYDROGEN_PEROXIDE	-1.31	0.89	0	0	0	not significant
2911	GO_REGULATION_OF_STEROL_TRANSPORT	-1.31	0.88	0	0	0	not significant
2912	GO_CALCIUM_RELEASE_CHANNEL_ACTIVITY	-1.31	0.87	0	0	0	not significant
2913	GO_POSITIVE_REGULATION_OF_NITRIC_OXIDE_SYNTHASE_ACTIVITY	-1.31	0.87	0	0	0	not significant
2914	GO_RETINOIC_ACID_RECEPTOR_SIGNALING_PATHWAY	-1.31	0.87	0	0	0	not significant
2915	GO_REGULATION_OF_ESTABLISHMENT_OF_PROTEIN_LOCALIZATION_TO	-1.31	0.84	0	0	0	not significant
2916	REACTOME_UREA_CYCLE	-1.31	0.83	0	0	0	not significant
2917	REACTOME_MASTL_FACILITATES_MITOTIC_PROGRESSION	-1.31	0.81	0	0	0	not significant
2918	REACTOME_RUNX1_REGULATES_ESTROGEN_RECEPTOR_MEDIATED_TI	-1.31	0.80	0	0	0	not significant
2919	SA_TRKA_RECEPTOR	-1.31	0.80	0	0	0	not significant
2920	GO_DETECTION_OF_CALCIUM_ION	-1.31	0.79	0	0	0	not significant
2921	GO_ESTABLISHMENT_OR_MAINTENANCE_OF_CYTOSKELETON_POLARIT	-1.31	0.79	0	0	0	not significant
2922	GO_FATTY_ACID_HOMEOSTASIS	-1.31	0.79	0	0	0	not significant
2923	GO_NUCLEOTIDE_SALVAGE	-1.31	0.79	0	0	0	not significant
2924	GO_RETINOL_DEHYDROGENASE_ACTIVITY	-1.31	0.79	0	0	0	not significant
2925	KEGG_PANTOTHENATE_AND_COA_BIOSYNTHESIS	-1.31	0.79	0	0	0	not significant
2926	GO_OXIDATIVE_DNA_DEMETHYLATION	-1.31	0.78	0	0	0	not significant
2927	GO_VINCULIN_BINDING	-1.31	0.78	0	0	0	not significant
2928	REACTOME_PRESYNAPTIC_FUNCTION_OF_KAINATE_RECEPTORS	-1.31	0.78	0	0	0	not significant
2929	GO_POSITIVE_REGULATION_OF_CELL_MIGRATION_INVOLVED_IN_SPROU	-1.31	0.77	0	0	0	not significant
2930	GO_REVERSE_CHOLESTEROL_TRANSPORT	-1.31	0.77	0	0	0	not significant
2931	GO_RIBOSOMAL_PROTEIN_S6_KINASE_ACTIVITY	-1.31	0.77	0	0	0	not significant

2932	GO_SEQUENCE_SPECIFIC_MRNA_BINDING	-1.31	0.77	0	0	0	not significant
2933	GO_DYSTROGLYCAN_BINDING	-1.31	0.76	0	0	0	not significant
2934	GO_POSITIVE_REGULATION_OF_DEACETYLASE_ACTIVITY	-1.31	0.76	0	0	0	not significant
2935	SHIN_B_CELL_LYMPHOMA_CLUSTER_3	-1.31	0.76	0	0	0	not significant
2936	GO_ARACHIDONIC_ACID_SECRETION	-1.31	0.75	0	0	0	not significant
2937	GO_EMBRYONIC_DIGESTIVE_TRACT_DEVELOPMENT	-1.31	0.75	0	0	0	not significant
2938	TSUDA_ALVEOLAR_SOFT_PART_SARCOMA	-1.31	0.75	0	0	0	not significant
2939	GO_7S_RNA_BINDING	-1.31	0.74	0	0	0	not significant
2940	CHR4Q23	-1.31	0.73	0	0	0	not significant
2941	GO_ACTIN_DEPENDENT_ATPASE_ACTIVITY	-1.31	0.73	0	0	0	not significant
2942	GO_REGULATION_OF_REMOVAL_OF_SUPEROXIDE_RADICALS	-1.31	0.73	0	0	0	not significant
2943	REACTOME_ACTIVATION_OF_RAC1_DOWNSTREAM_OF_NMDARS	-1.31	0.73	0	0	0	not significant
2944	GO_ACTIN_FILAMENT_BASED_PROCESS	-1.30	4.00	0	0	0	not significant
2945	GO_DEFENSE_RESPONSE	-1.30	4.00	0	0	0	not significant
2946	GO_ION_TRANSMEMBRANE_TRANSPORT	-1.30	4.00	0	0	0	not significant
2947	GO_REGULATION_OF_CELLULAR_CATABOLIC_PROCESS	-1.30	4.00	0	0	0	not significant
2948	GO_REGULATION_OF_IMMUNE_SYSTEM_PROCESS	-1.30	4.00	0	0	0	not significant
2949	LIU_SOX4_TARGETS_DN	-1.30	2.37	0	0	0	not significant
2950	GO_SUPRAMOLECULAR_FIBER_ORGANIZATION	-1.30	2.26	0	0	0	not significant
2951	WANG_CLIM2_TARGETS_DN	-1.30	1.76	0	0	0	not significant
2952	GO_MUSCLE_TISSUE_DEVELOPMENT	-1.30	1.73	0	0	0	not significant
2953	GO_RESPONSE_TO_PEPTIDE_HORMONE	-1.30	1.71	0	0	0	not significant
2954	SHEPARD_BMYB_MORPHOLINO_UP	-1.30	1.60	0	0	0	not significant
2955	CREIGHTON_ENDOCRINE_THERAPY_RESISTANCE_4	-1.30	1.56	0	0	0	not significant
2956	HALLMARK_APICAL_JUNCTION	-1.30	1.51	0	0	0	not significant
2957	WHITFIELD_CELL_CYCLE_G2	-1.30	1.48	0	0	0	not significant
2958	ALONSO_METASTASIS_UP	-1.30	1.46	0	0	0	not significant
2959	GO_TISSUE_MIGRATION	-1.30	1.42	0	0	0	not significant
2960	GO_PROTEIN_IMPORT	-1.30	1.34	0	0	0	not significant
2961	GROSS_HYPOXIA_VIA_ELK3_AND_HIF1A_UP	-1.30	1.33	0	0	0	not significant
2962	GO_REGULATION_OF_GENE_SILENCING	-1.30	1.31	0	0	0	not significant
2963	ELVIDGE_HYPOXIA_BY_DMOG_UP	-1.30	1.26	0	0	0	not significant
2964	REACTOME_SUMOYLATION	-1.30	1.26	0	0	0	not significant
2965	GO_MRNA_3_END_PROCESSING	-1.30	1.23	0	0	0	not significant
2966	GO_PROTEIN_TYROSINE_PHOSPHATASE_ACTIVITY	-1.30	1.19	0	0	0	not significant
2967	GO_POSITIVE_REGULATION_OF_CANONICAL_WNT_SIGNALING_PATHWAY	-1.30	1.18	0	0	0	not significant
2968	HALLMARK_MYOGENESIS	-1.30	1.18	0	0	0	not significant
2969	GO_NEGATIVE_REGULATION_OF_PEPTIDE_SECRETION	-1.30	1.17	0	0	0	not significant
2970	GO_POSITIVE_REGULATION_OF_PROTEIN_MODIFICATION_BY_SMALL_PI	-1.30	1.17	0	0	0	not significant
2971	DARWICHE_PAPILLOMA_RISK_LOW_UP	-1.30	1.16	0	0	0	not significant
2972	GO_ORGANELLE_MEMBRANE_FUSION	-1.30	1.12	0	0	0	not significant
2973	REACTOME_TELOMERE_MAINTENANCE	-1.30	1.10	0	0	0	not significant
2974	GO_PROTEIN_DEPOLYMERIZATION	-1.30	1.09	0	0	0	not significant
2975	KEGG_ADIPOCYTOKINE_SIGNALING_PATHWAY	-1.30	1.08	0	0	0	not significant
2976	GO_REGULATION_OF_PLASMA_LIPOPROTEIN_PARTICLE_LEVELS	-1.30	1.06	0	0	0	not significant
2977	GO_POTASSIUM_ION_TRANSMEMBRANE_TRANSPORTER_ACTIVITY	-1.30	1.05	0	0	0	not significant
2978	LEE_AGING_CEREBELLUM_DN	-1.30	1.05	0	0	0	not significant
2979	REACTOME_SUMOYLATION_OF_CHROMATIN_ORGANIZATION_PROTEINS	-1.30	1.05	0	0	0	not significant
2980	REACTOME_TP53_REGULATES_TRANSCRIPTION_OF_DNA_REPAIR_GENI	-1.30	1.05	0	0	0	not significant
2981	ESC_J1_UP_LATE.V1_UP	-1.30	1.04	0	0	0	not significant
2982	CHR7Q32	-1.30	0.97	0	0	0	not significant
2983	REN_BOUND_BY_E2F	-1.30	0.97	0	0	0	not significant
2984	REACTOME_SYNTHESIS_OF_ACTIVE_UBIQUITIN:_ROLES_OF_E1_AND_E2	-1.30	0.96	0	0	0	not significant
2985	REACTOME_RNA_POLYMERASE_I_TRANSCRIPTION_TERMINATION	-1.30	0.95	0	0	0	not significant
2986	GO_TERTIARY_GRANULE_MEMBRANE	-1.30	0.94	0	0	0	not significant
2987	GO_KINESIN_COMPLEX	-1.30	0.93	0	0	0	not significant
2988	GO_MECHANORECEPTOR_DIFFERENTIATION	-1.30	0.92	0	0	0	not significant
2989	GO_RECEPTOR_CATABOLIC_PROCESS	-1.30	0.91	0	0	0	not significant
2990	PID_HIF2PATHWAY	-1.30	0.90	0	0	0	not significant
2991	LIANG_SILENCED_BY_METHYLATION_2	-1.30	0.89	0	0	0	not significant
2992	GO_POSITIVE_REGULATION_OF_VIRAL_GENOME_REPLICATION	-1.30	0.88	0	0	0	not significant
2993	KRASNOSELSKAYA_ILF3_TARGETS_DN	-1.30	0.88	0	0	0	not significant
2994	SCIAN_INVERSED_TARGETS_OF_TP53_AND_TP73_DN	-1.30	0.88	0	0	0	not significant
2995	YAO_HOXA10_TARGETS_VIA_PROGESTERONE_UP	-1.30	0.88	0	0	0	not significant
2996	GO_BETA_CATENIN_TCF_COMPLEX_ASSEMBLY	-1.30	0.87	0	0	0	not significant
2997	GO_SULFUR_COMPOUND_TRANSPORT	-1.30	0.87	0	0	0	not significant
2998	PID_ECADHERIN_STABILIZATION_PATHWAY	-1.30	0.87	0	0	0	not significant
2999	HAHTOLA_SEZARY_SYNDROM_DN	-1.30	0.86	0	0	0	not significant
3000	GO_ICOSANOID_BIOSYNTHETIC_PROCESS	-1.30	0.82	0	0	0	not significant
3001	GO_POSITIVE_REGULATION_OF_INTERFERON_ALPHA_PRODUCTION	-1.30	0.82	0	0	0	not significant
3002	GO_RHYTHMIC_BEHAVIOR	-1.30	0.82	0	0	0	not significant
3003	GO_NEGATIVE_REGULATION_OF_TELOMERE_MAINTENANCE_VIA_TELOM	-1.30	0.81	0	0	0	not significant
3004	GO_PROTON_TRANSPORTING_TWO_SECTOR_ATPASE_COMPLEX_CATA	-1.30	0.81	0	0	0	not significant
3005	GO_U1_SNRNP	-1.30	0.81	0	0	0	not significant
3006	KIM_LRRC3B_TARGETS	-1.30	0.81	0	0	0	not significant
3007	REACTOME_REGULATION_OF_GENE_EXPRESSION_IN_LATE_STAGE_BR/	-1.30	0.81	0	0	0	not significant
3008	REACTOME_SYNTHESIS_OF_VERY_LONG_CHAIN_FATTY_ACYL_COAS	-1.30	0.81	0	0	0	not significant
3009	GHO_ATF5_TARGETS_DN	-1.30	0.80	0	0	0	not significant
3010	GO_DIGESTIVE_TRACT_MORPHOGENESIS	-1.30	0.80	0	0	0	not significant
3011	GO_INCLUSION_BODY_ASSEMBLY	-1.30	0.80	0	0	0	not significant
3012	REACTOME_CELL_EXTRACELLULAR_MATRIX_INTERACTIONS	-1.30	0.80	0	0	0	not significant
3013	GO_DNA_TOPOISOMERASE_BINDING	-1.30	0.79	0	0	0	not significant
3014	GO_NEGATIVE_REGULATION_OF_STRIATED_MUSCLE_CELL_APOPTOTIC	-1.30	0.79	0	0	0	not significant
3015	GO_SIGNAL_RECOGNITION_PARTICLE_ENDOPLASMIC_RETICULUM_TARC	-1.30	0.78	0	0	0	not significant
3016	BIOCARTA_DREAM_PATHWAY	-1.30	0.77	0	0	0	not significant
3017	GO_NOTCH_BINDING	-1.30	0.77	0	0	0	not significant
3018	GO_OXIDOREDUCTASE_ACTIVITY_ACTING_ON_A_SULFUR_GROUP_OF_I	-1.30	0.77	0	0	0	not significant
3019	GO_VASCULAR_ENDOTHELIAL_GROWTH_FACTOR_RECEPTOR_2_BINDIN	-1.30	0.77	0	0	0	not significant
3020	KIM_PTEN_TARGETS_UP	-1.30	0.77	0	0	0	not significant
3021	REACTOME_NEF_MEDIATED_DOWNREGULATION_OF_MHC_CLASS_I_CO	-1.30	0.77	0	0	0	not significant
3022	REACTOME_LOSS_OF_MECP2_BINDING_ABILITY_TO_THE_NCOR_SMRT_	-1.30	0.76	0	0	0	not significant
3023	REACTOME_SYNTHESIS_OF_PYROPHOSPHATES_IN_THE_CYTOSOL	-1.30	0.76	0	0	0	not significant
3024	GO_AXO_DENDRITIC_PROTEIN_TRANSPORT	-1.30	0.75	0	0	0	not significant
3025	GO_CELLULAR_RESPONSE_TO_ALDEHYDE	-1.30	0.75	0	0	0	not significant
3026	GO_NATURAL_KILLER_CELL_DEGRANULATION	-1.30	0.75	0	0	0	not significant
3027	GO_PRE_SNORNP_COMPLEX	-1.30	0.75	0	0	0	not significant
3028	GO_REGULATION_OF_SKELETAL_MUSCLE_ADAPTATION	-1.30	0.75	0	0	0	not significant
3029	KRISHNAN_FURIN_TARGETS_DN	-1.30	0.75	0	0	0	not significant
3030	YAO_TEMPORAL_RESPONSE_TO_PROGESTERONE_CLUSTER_4	-1.30	0.75	0	0	0	not significant
3031	GO_ANNULATE_LAMELLAE	-1.30	0.74	0	0	0	not significant
3032	GO_NEGATIVE_REGULATION_OF_DENDRITIC_SPINE_DEVELOPMENT	-1.30	0.74	0	0	0	not significant
3033	GO_REGULATION_OF_TOLL_LIKE_RECEPTOR_9_SIGNALING_PATHWAY	-1.30	0.74	0	0	0	not significant
3034	REACTOME_GLUTAMATE_NEUROTRANSMITTER_RELEASE_CYCLE	-1.30	0.74	0	0	0	not significant
3035	GO_ESTABLISHMENT_OF_GOLGI_LOCALIZATION	-1.30	0.73	0	0	0	not significant
3036	GO_LACTATE_METABOLIC_PROCESS	-1.30	0.73	0	0	0	not significant
3037	LIU_CDX2_TARGETS_UP	-1.30	0.73	0	0	0	not significant
3038	GO_TRANSCRIPTION_FACTOR_TFIIH_CORE_COMPLEX	-1.30	0.72	0	0	0	not significant
3039	HOLLEMAN_DAUNORUBICIN_B_ALL_UP	-1.30	0.72	0	0	0	not significant
3040	GO_EMBRYONIC_VISCEROCRANIUM_MORPHOGENESIS	-1.30	0.69	0	0	0	not significant

3041	GO_MEMBRANE_ORGANIZATION	-1.29	4.00	0	0	0	not significant
3042	GO_PROTEIN_DIMERIZATION_ACTIVITY	-1.29	4.00	0	0	0	not significant
3043	GO_PROTEIN_HOMODIMERIZATION_ACTIVITY	-1.29	4.00	0	0	0	not significant
3044	GO_MYELOID_LEUKOCYTE_MEDIATED_IMMUNITY	-1.29	1.97	0	0	0	not significant
3045	GO NUCLEOBASE CONTAINING SMALL MOLECULE METABOLIC PROCE	-1.29	1.94	0	0	0	not significant
3046	GO_RESPONSE_TO_BACTERIUM	-1.29	1.86	0	0	0	not significant
3047	HUTTMANN_B_CLL_POOR_SURVIVAL_UP	-1.29	1.73	0	0	0	not significant
3048	GO_DEPHOSPHORYLATION	-1.29	1.67	0	0	0	not significant
3049	BERTUCCI_MEDULLARY_VS_DUCTAL_BREAST_CANCER_UP	-1.29	1.65	0	0	0	not significant
3050	GO_RHYTHMIC_PROCESS	-1.29	1.51	0	0	0	not significant
3051	GO_PROTEIN_HOMOOLIGOMERIZATION	-1.29	1.49	0	0	0	not significant
3052	GO_CELLULAR_RESPONSE_TO_EXTRACELLULAR_STIMULUS	-1.29	1.39	0	0	0	not significant
3053	GO_CYTOKINESIS	-1.29	1.30	0	0	0	not significant
3054	GO_REGULATION_OF_PROTEIN_DEPHOSPHORYLATION	-1.29	1.20	0	0	0	not significant
3055	ACEVEDO_LIVER_CANCER_WITH_H3K9ME3_DN	-1.29	1.18	0	0	0	not significant
3056	RPS14_DN.V1_UP	-1.29	1.18	0	0	0	not significant
3057	REACTOME_BIOLOGICAL_OXIDATIONS	-1.29	1.17	0	0	0	not significant
3058	REACTOME_G2_M_DNA_DAMAGE_CHECKPOINT	-1.29	1.17	0	0	0	not significant
3059	PID_P73PATHWAY	-1.29	1.15	0	0	0	not significant
3060	GO_REGULATION_OF_DNA_BIOSYNTHETIC_PROCESS	-1.29	1.13	0	0	0	not significant
3061	GO_RESPONSE_TO_ORGANOPHOSPHORUS	-1.29	1.10	0	0	0	not significant
3062	ZHOU_CELL_CYCLE_GENES_IN_IR_RESPONSE_6HR	-1.29	1.10	0	0	0	not significant
3063	JAZAG_TGFB1_SIGNALING_VIA_SMAD4_UP	-1.29	1.04	0	0	0	not significant
3064	PRAMOONJAGO_SOX4_TARGETS_DN	-1.29	1.04	0	0	0	not significant
3065	CHR12Q23	-1.29	1.03	0	0	0	not significant
3066	GAJATE_RESPONSE_TO_TRABECTEDIN_UP	-1.29	1.02	0	0	0	not significant
3067	BIOCARTA_TCR_PATHWAY	-1.29	0.94	0	0	0	not significant
3068	CERVERA_SDHB_TARGETS_1_UP	-1.29	0.94	0	0	0	not significant
3069	GO_CENTRAL_NERVOUS_SYSTEM_NEURON_DEVELOPMENT	-1.29	0.93	0	0	0	not significant
3070	GO_NEGATIVE_REGULATION_OF_CHROMOSOME_SEGREGATION	-1.29	0.93	0	0	0	not significant
3071	GO_OVULATION_CYCLE	-1.29	0.90	0	0	0	not significant
3072	GO_SKELETAL_MUSCLE_CELL_DIFFERENTIATION	-1.29	0.88	0	0	0	not significant
3073	REACTOME_DISEASES_ASSOCIATED_WITH_O_GLYCOSYLATION_OF_PR	-1.29	0.88	0	0	0	not significant
3074	REACTOME_SIGNALING_BY_FGFR2_IN_DISEASE	-1.29	0.88	0	0	0	not significant
3075	BIOCARTA_INTEGRIN_PATHWAY	-1.29	0.87	0	0	0	not significant
3076	GO_MODULATION_BY_SYMBIONT_OF_HOST_CELLULAR_PROCESS	-1.29	0.87	0	0	0	not significant
3077	PETROVA_PROX1_TARGETS_DN	-1.29	0.87	0	0	0	not significant
3078	GO_POSITIVE_REGULATION_OF_STEROL_TRANSPORT	-1.29	0.85	0	0	0	not significant
3079	ICHIBA_GRAFT_VERSUS_HOST_DISEASE_35D_DN	-1.29	0.85	0	0	0	not significant
3080	XU_AKT1_TARGETS_6HR	-1.29	0.85	0	0	0	not significant
3081	GO_RESPONSE_TO_INTERFERON_BETA	-1.29	0.82	0	0	0	not significant
3082	GO_ATP_DEPENDENT_MICROTUBULE_MOTOR_ACTIVITY	-1.29	0.81	0	0	0	not significant
3083	GRANDVAUX_IRF3_TARGETS_UP	-1.29	0.81	0	0	0	not significant
3084	SINGH_NFE2L2_TARGETS	-1.29	0.81	0	0	0	not significant
3085	GO_KILLING_OF_CELLS_OF_OTHER_ORGANISM	-1.29	0.80	0	0	0	not significant
3086	GO_RENAL_WATER_HOMEOSTASIS	-1.29	0.80	0	0	0	not significant
3087	REACTOME_SEMA3A_PLEXIN_REPULSION_SIGNALING_BY_INHIBITING_INT	-1.29	0.80	0	0	0	not significant
3088	VERRECCHIA_RESPONSE_TO_TGFB1_C3	-1.29	0.79	0	0	0	not significant
3089	MATTIOLI_MGUS_VS_MULTIPLE_MYELOMA	-1.29	0.78	0	0	0	not significant
3090	GO_MUSCLE_HYPERTROPHY_IN_RESPONSE_TO_STRESS	-1.29	0.77	0	0	0	not significant
3091	BIOCARTA_EFP_PATHWAY	-1.29	0.76	0	0	0	not significant
3092	CHR14Q13	-1.29	0.76	0	0	0	not significant
3093	EHRLICH_ICF_SYNDROM_DN	-1.29	0.75	0	0	0	not significant
3094	GO_INTEGRIN_ACTIVATION	-1.29	0.75	0	0	0	not significant
3095	GO_PANCREATIC_JUICE_SECRETION	-1.29	0.75	0	0	0	not significant
3096	JOHANSSON_BRAIN_CANCER_EARLY_VS_LATE_UP	-1.29	0.75	0	0	0	not significant
3097	REACTOME RUNX1 REGULATES TRANSCRIPTION OF GENES INVOLVEI	-1.29	0.75	0	0	0	not significant
3098	SHIN_B_CELL_LYMPHOMA_CLUSTER_9	-1.29	0.75	0	0	0	not significant
3099	WANG_RECURRENT_LIVER_CANCER_DN	-1.29	0.75	0	0	0	not significant
3100	GO_SNRNP_BINDING	-1.29	0.74	0	0	0	not significant
3101	KEGG_GRAFT_VERSUS_HOST_DISEASE	-1.29	0.74	0	0	0	not significant
3102	GO_G_PROTEIN_BETA_GAMMA_SUBUNIT_COMPLEX_BINDING	-1.29	0.73	0	0	0	not significant
3103	GO_NEGATIVE_REGULATION_OF_SINGLE_STRANDED_VIRAL_RNA_REPL	-1.29	0.73	0	0	0	not significant
3104	GO_PEPTIDE_ALPHA_N_ACETYLTRANSFERASE_ACTIVITY	-1.29	0.73	0	0	0	not significant
3105	GO_REGULATION_OF_TAU_PROTEIN_KINASE_ACTIVITY	-1.29	0.73	0	0	0	not significant
3106	BIOCARTA_EIF2_PATHWAY	-1.29	0.72	0	0	0	not significant
3107	GO_BARBED_END_ACTIN_FILAMENT_CAPPING	-1.29	0.72	0	0	0	not significant
3108	GO_WD40_REPEAT_DOMAIN_BINDING	-1.29	0.70	0	0	0	not significant
3109	MURAKAMI_UV_RESPONSE_1HR_UP	-1.29	0.70	0	0	0	not significant
3110	DOUGLAS_BMI1_TARGETS_UP	-1.28	4.00	0	0	0	not significant
3111	GO_CATION_TRANSPORT	-1.28	4.00	0	0	0	not significant
3112	GO_CHEMICAL_HOMEOSTASIS	-1.28	4.00	0	0	0	not significant
3113	GO_EXOCYTOSIS	-1.28	4.00	0	0	0	not significant
3114	REACTOME_DISEASE	-1.28	4.00	0	0	0	not significant
3115	GO_REGULATION_OF_CELL_CYCLE_PROCESS	-1.28	2.12	0	0	0	not significant
3116	GO_RESPONSE_TO_TOXIC_SUBSTANCE	-1.28	2.06	0	0	0	not significant
3117	GO_NEGATIVE_REGULATION_OF_MITOTIC_CELL_CYCLE	-1.28	1.88	0	0	0	not significant
3118	HELLER_HDAC_TARGETS_SILENCED_BY_METHYLATION_UP	-1.28	1.68	0	0	0	not significant
3119	BENPORATH_MYC_TARGETS_WITH_EBOX	-1.28	1.65	0	0	0	not significant
3120	GO_NEGATIVE_REGULATION_OF_TRANSPORT	-1.28	1.61	0	0	0	not significant
3121	GO_AMEBOIDAL_TYPE_CELL_MIGRATION	-1.28	1.43	0	0	0	not significant
3122	GO_CHROMOSOME_CENTROMERIC_REGION	-1.28	1.37	0	0	0	not significant
3123	GO_ACTIN_BINDING	-1.28	1.31	0	0	0	not significant
3124	EGFR_UP.V1_UP	-1.28	1.30	0	0	0	not significant
3125	GO_CELL_CHEMOTAXIS	-1.28	1.30	0	0	0	not significant
3126	GO_MULTI_MULTICELLULAR_ORGANISM_PROCESS	-1.28	1.28	0	0	0	not significant
3127	KYNG_DNA_DAMAGE_DN	-1.28	1.28	0	0	0	not significant
3128	GO_POSITIVE_REGULATION_OF_PEPTIDASE_ACTIVITY	-1.28	1.25	0	0	0	not significant
3129	GO_NEGATIVE_REGULATION_OF_ESTABLISHMENT_OF_PROTEIN_LOCAL	-1.28	1.17	0	0	0	not significant
3130	GO_PEPTIDYL_TYROSINE_DEPHOSPHORYLATION	-1.28	1.17	0	0	0	not significant
3131	DARWICHE_PAPILLOMA_RISK_HIGH_UP	-1.28	1.16	0	0	0	not significant
3132	YAO_TEMPORAL_RESPONSE_TO_PROGESTERONE_CLUSTER_12	-1.28	1.07	0	0	0	not significant
3133	GROSS_HYPOXIA_VIA_ELK3_AND_HIF1A_DN	-1.28	1.06	0	0	0	not significant
3134	GO_REGULATION_OF_CALCIUM_ION_TRANSMEMBRANE_TRANSPORT	-1.28	1.04	0	0	0	not significant
3135	GO_REGULATION_OF_GLIOGENESIS	-1.28	1.04	0	0	0	not significant
3136	GO_NEURON_PROJECTION_CYTOPLASM	-1.28	1.03	0	0	0	not significant
3137	REACTOME_HDACS_DEACETYLATE_HISTONES	-1.28	1.02	0	0	0	not significant
3138	REACTOME_SUMOYLATION_OF_RNA_BINDING_PROTEINS	-1.28	1.00	0	0	0	not significant
3139	GO_ER_TO_GOLGI_TRANSPORT_VESICLE_MEMBRANE	-1.28	0.99	0	0	0	not significant
3140	GO_RESPONSE_TO_ANTINEOPLASTIC_AGENT	-1.28	0.99	0	0	0	not significant
3141	MODY_HIPPOCAMPUS_POSTNATAL	-1.28	0.98	0	0	0	not significant
3142	CHR6Q21	-1.28	0.95	0	0	0	not significant
3143	CHR1P22	-1.28	0.94	0	0	0	not significant
3144	DASU_IL6_SIGNALING_UP	-1.28	0.93	0	0	0	not significant
3145	REACTOME_HSF1_DEPENDENT_TRANSACTIVATION	-1.28	0.92	0	0	0	not significant
3146	NIKOLSKY_BREAST_CANCER_12Q13_Q21_AMPLICON	-1.28	0.91	0	0	0	not significant
3147	STAMBOLSKY_TARGETS_OF_MUTATED_TP53_UP	-1.28	0.91	0	0	0	not significant
3148	GO_AMINO_ACID_TRANSMEMBRANE_TRANSPORTER_ACTIVITY	-1.28	0.90	0	0	0	not significant
3149	GO_EXTRACELLULAR_MATRIX_STRUCTURAL_CONSTITUENT	-1.28	0.90	0	0	0	not significant

3150	BIOCARTA_GPCR_PATHWAY	-1.28	0.89	0	0	0	not significant
3151	GERHOLD_ADIPOGENESIS_UP	-1.28	0.89	0	0	0	not significant
3152	GO_PLATELET_ALPHA_GRANULE_LUMEN	-1.28	0.88	0	0	0	not significant
3153	GO_REGULATION_OF_LAMELLIPODIUM_ORGANIZATION	-1.28	0.88	0	0	0	not significant
3154	EINAV_INTERFERON_SIGNATURE_IN_CANCER	-1.28	0.86	0	0	0	not significant
3155	GO_POSITIVE_REGULATION_OF_TELOMERE_MAINTENANCE_VIA_TELOM	-1.28	0.86	0	0	0	not significant
3156	HEIDENBLAD_AMPLICON_8Q24_UP	-1.28	0.85	0	0	0	not significant
3157	GO_REGULATION_OF_CYTOPLASMIC_TRANSLATION	-1.28	0.84	0	0	0	not significant
3158	REACTOME_SIGNALING_BY_WNT_IN_CANCER	-1.28	0.84	0	0	0	not significant
3159	GO_TRANSCRIPTION_PREINITIATION_COMPLEX_ASSEMBLY	-1.28	0.83	0	0	0	not significant
3160	SU_LIVER	-1.28	0.83	0	0	0	not significant
3161	WANG_RECURRENT_LIVER_CANCER_UP	-1.28	0.83	0	0	0	not significant
3162	GO_CLATHRIN_VESICLE_COAT	-1.28	0.82	0	0	0	not significant
3163	REACTOME_THROMBOXANE_SIGNALLING_THROUGH_TP_RECEPTOR	-1.28	0.81	0	0	0	not significant
3164	CEBALLOS_TARGETS_OF_TP53_AND_MYC_DN	-1.28	0.80	0	0	0	not significant
3165	BIOCARTA_NGF_PATHWAY	-1.28	0.79	0	0	0	not significant
3166	GO_INACTIVATION_OF_MAPK_ACTIVITY	-1.28	0.79	0	0	0	not significant
3167	GO_MUSCLE_FIBER_DEVELOPMENT	-1.28	0.78	0	0	0	not significant
3168	GO_CRANIAL_NERVE_DEVELOPMENT	-1.28	0.77	0	0	0	not significant
3169	GO_FEMALE_MEIOTIC_NUCLEAR_DIVISION	-1.28	0.77	0	0	0	not significant
3170	GO_PHOTORECEPTOR_INNER_SEGMENT	-1.28	0.77	0	0	0	not significant
3171	LI_ADIPOGENESIS_BY_ACTIVATED_PPARG	-1.28	0.77	0	0	0	not significant
3172	YANG_BREAST_CANCER_ESR1_BULK_DN	-1.28	0.77	0	0	0	not significant
3173	GO_MICRO_RIBONUCLEOPROTEIN_COMPLEX	-1.28	0.76	0	0	0	not significant
3174	GO_MYELIN_MAINTENANCE	-1.28	0.76	0	0	0	not significant
3175	WANG_LSD1_TARGETS_UP	-1.28	0.76	0	0	0	not significant
3176	KEGG_BIOSYNTHESIS_OF_UNSATURATED_FATTY_ACIDS	-1.28	0.75	0	0	0	not significant
3177	WHITESIDE_CISPLATIN_RESISTANCE_UP	-1.28	0.75	0	0	0	not significant
3178	GO_MITOCHONDRIAL_RNA_CATABOLIC_PROCESS	-1.28	0.74	0	0	0	not significant
3179	VALK_AML_CLUSTER_13	-1.28	0.74	0	0	0	not significant
3180	GO_ESTABLISHMENT_OF_PROTEIN_LOCALIZATION_TO_CHROMATIN	-1.28	0.73	0	0	0	not significant
3181	GO_MAP_KINASE_PHOSPHATASE_ACTIVITY	-1.28	0.73	0	0	0	not significant
3182	GO_REGULATION_OF_DIGESTIVE_SYSTEM_PROCESS	-1.28	0.73	0	0	0	not significant
3183	GO_TRNA_SPECIFIC_ADENOSINE_DEAMINASE_ACTIVITY	-1.28	0.73	0	0	0	not significant
3184	GO_WIDE_PORE_CHANNEL_ACTIVITY	-1.28	0.73	0	0	0	not significant
3185	REACTOME_CREB1_PHOSPHORYLATION_THROUGH_THE_ACTIVATION_C	-1.28	0.73	0	0	0	not significant
3186	GO_EOSINOPHIL_ACTIVATION	-1.28	0.72	0	0	0	not significant
3187	KUROKAWA_LIVER_CANCER_EARLY_RECURRENCE_UP	-1.28	0.72	0	0	0	not significant
3188	GO_NEGATIVE_REGULATION_OF_GLUCOSE_TRANSMEMBRANE_TRANSP	-1.28	0.71	0	0	0	not significant
3189	GO_SMOOTH_MUSCLE_TISSUE_DEVELOPMENT	-1.28	0.70	0	0	0	not significant
3190	CERVERA_SDHB_TARGETS_1_DN	-1.28	0.69	0	0	0	not significant
3191	GO_BEHAVIORAL_RESPONSE_TO_COCAINE	-1.28	0.69	0	0	0	not significant
3192	REACTOME_SYNTHESIS_OF_KETONE_BODIES	-1.28	0.69	0	0	0	not significant
3193	GO_REGULATION_OF_MICROGLIAL_CELL_ACTIVATION	-1.28	0.68	0	0	0	not significant
3194	GO_REGULATION_BY_VIRUS_OF_VIRAL_PROTEIN_LEVELS_IN_HOST_CEI	-1.28	0.67	0	0	0	not significant
3195	FULCHER_INFLAMMATORY_RESPONSE_LECTIN_VS_LPS_UP	-1.27	4.00	0	0	0	not significant
3196	GO_ION_TRANSPORT	-1.27	4.00	0	0	0	not significant
3197	REACTOME_TRANSCRIPTIONAL_REGULATION_BY_TP53	-1.27	2.03	0	0	0	not significant
3198	MEISSNER_BRAIN_HCP_WITH_H3K4ME3_AND_H3K27ME3	-1.27	2.00	0	0	0	not significant
3199	RUTELLA_RESPONSE_TO_CSF2RB_AND_IL4_DN	-1.27	1.88	0	0	0	not significant
3200	GO_IMMUNE_EFFECTOR_PROCESS	-1.27	1.74	0	0	0	not significant
3201	DAIRKEE_TERT_TARGETS_UP	-1.27	1.67	0	0	0	not significant
3202	GO_CELLULAR_RESPONSE_TO_DRUG	-1.27	1.66	0	0	0	not significant
3203	GO_MITOTIC_NUCLEAR_DIVISION	-1.27	1.55	0	0	0	not significant
3204	GO_PHOSPHORIC_ESTER_HYDROLASE_ACTIVITY	-1.27	1.37	0	0	0	not significant
3205	GRAESSMANN_APOPTOSIS_BY_SERUM_DEPRIVATION_DN	-1.27	1.34	0	0	0	not significant
3206	GO_MIDBODY	-1.27	1.23	0	0	0	not significant
3207	GO_REGULATION_OF_ENDOTHELIAL_CELL_MIGRATION	-1.27	1.23	0	0	0	not significant
3208	GO_REGULATION_OF_EPITHELIAL_CELL_MIGRATION	-1.27	1.21	0	0	0	not significant
3209	PRC2_EZH2_UP.V1_UP	-1.27	1.20	0	0	0	not significant
3210	SASSON_RESPONSE_TO_FORSKOLIN_UP	-1.27	1.20	0	0	0	not significant
3211	GO_ORGANELLE_FUSION	-1.27	1.12	0	0	0	not significant
3212	GO_ENDOTHELIAL_CELL_MIGRATION	-1.27	1.11	0	0	0	not significant
3213	GO_HISTONE_DEACETYLASE_BINDING	-1.27	1.10	0	0	0	not significant
3214	WIERENGA_STAT5A_TARGETS_GROUP1	-1.27	1.05	0	0	0	not significant
3215	P53_DN.V2_DN	-1.27	1.03	0	0	0	not significant
3216	TOOKER_GEMCITABINE_RESISTANCE_UP	-1.27	1.00	0	0	0	not significant
3217	GO_POSITIVE_REGULATION_OF_COLD_INDUCED_THERMOGENESIS	-1.27	0.99	0	0	0	not significant
3218	CHR9Q33	-1.27	0.98	0	0	0	not significant
3219	COLDREN_GEFITINIB_RESISTANCE_UP	-1.27	0.97	0	0	0	not significant
3220	KAUFFMANN_MELANOMA_RELAPSE_UP	-1.27	0.96	0	0	0	not significant
3221	GO_NEUROMUSCULAR_PROCESS	-1.27	0.94	0	0	0	not significant
3222	PTEN_DN.V1_DN	-1.27	0.94	0	0	0	not significant
3223	GO_MICROBODY_MEMBRANE	-1.27	0.93	0	0	0	not significant
3224	LUCAS_HNF4A_TARGETS_UP	-1.27	0.92	0	0	0	not significant
3225	KEGG_AMINO_SUGAR_AND_NUCLEOTIDE_SUGAR_METABOLISM	-1.27	0.89	0	0	0	not significant
3226	ZHU_CMV_24_HR_DN	-1.27	0.89	0	0	0	not significant
3227	BARRIER_COLON_CANCER_RECURRENCE_UP	-1.27	0.88	0	0	0	not significant
3228	GO_HEAD_MORPHOGENESIS	-1.27	0.86	0	0	0	not significant
3229	GO_POSITIVE_REGULATION_OF_TRANSCRIPTION_FROM_RNA_POLYMEF	-1.27	0.86	0	0	0	not significant
3230	GO_POSITIVE_REGULATION_OF_CALCIUM_ION_TRANSMEMBRANE_TRAN	-1.27	0.85	0	0	0	not significant
3231	PLASARI_TGFB1_SIGNALING_VIA_NFIC_1HR_UP	-1.27	0.84	0	0	0	not significant
3232	CHR12Q14	-1.27	0.83	0	0	0	not significant
3233	GO_POSITIVE_REGULATION_OF_IMMUNOGLOBULIN_PRODUCTION	-1.27	0.83	0	0	0	not significant
3234	GO_REGULATION_OF_DNA_TEMPLATED_TRANSCRIPTION_INITIATION	-1.27	0.83	0	0	0	not significant
3235	KEGG_CYTOSOLIC_DNA_SENSING_PATHWAY	-1.27	0.83	0	0	0	not significant
3236	GO_FATTY_ACYL_COA_BINDING	-1.27	0.81	0	0	0	not significant
3237	GO_MEMBRANE_RAFT_ORGANIZATION	-1.27	0.81	0	0	0	not significant
3238	GO_TRANSCRIPTION_ELONGATION_FROM_RNA_POLYMERASE_I_PROMC	-1.27	0.81	0	0	0	not significant
3239	GO_POTASSIUM_CHANNEL_COMPLEX	-1.27	0.80	0	0	0	not significant
3240	OSADA_ASCL1_TARGETS_UP	-1.27	0.80	0	0	0	not significant
3241	GO_NEURON_CELLULAR_HOMEOSTASIS	-1.27	0.79	0	0	0	not significant
3242	GO_POSITIVE_REGULATION_OF_SUBSTRATE_ADHESION_DEPENDENT_C	-1.27	0.79	0	0	0	not significant
3243	GO_SUMO_BINDING	-1.27	0.79	0	0	0	not significant
3244	REACTOME_NON_INTEGRIN_MEMBRANE_ECM_INTERACTIONS	-1.27	0.79	0	0	0	not significant
3245	GO_NCRNA_CATABOLIC_PROCESS	-1.27	0.78	0	0	0	not significant
3246	GO_ADENYLYLTRANSFERASE_ACTIVITY	-1.27	0.77	0	0	0	not significant
3247	GO_REGULATION_OF_CELLULAR_RESPONSE_TO_DRUG	-1.27	0.76	0	0	0	not significant
3248	GO_OUTER_MITOCHONDRIAL_MEMBRANE_PROTEIN_COMPLEX	-1.27	0.75	0	0	0	not significant
3249	GO_MITOCHONDRIAL_OUTER_MEMBRANE_TRANSLOCASE_COMPLEX	-1.27	0.74	0	0	0	not significant
3250	GO_POSITIVE_REGULATION_OF_PEPTIDYL_THREONINE_PHOSPHORYLA	-1.27	0.74	0	0	0	not significant
3251	GO_POSITIVE_REGULATION_OF_SEQUESTERING_OF_CALCIUM_ION	-1.27	0.74	0	0	0	not significant
3252	GO_PTERIDINE_CONTAINING_COMPOUND_BIOSYNTHETIC_PROCESS	-1.27	0.74	0	0	0	not significant
3253	GO_AP_2_ADAPTOR_COMPLEX_BINDING	-1.27	0.73	0	0	0	not significant
3254	PODAR_RESPONSE_TO_ADAPHOSTIN_DN	-1.27	0.73	0	0	0	not significant
3255	REACTOME_RNF_MUTANTS_SHOW_ENHANCED_WNT_SIGNALING_AND_P	-1.27	0.73	0	0	0	not significant
3256	GO_COPII_COATED_VESICLE_CARGO_LOADING	-1.27	0.72	0	0	0	not significant
3257	GO_REGULATION_OF_PINOCYTOSIS	-1.27	0.72	0	0	0	not significant
3258	KUROZUMI_RESPONSE_TO_ONCOCYTIC_VIRUS_AND_CYCLIC_RGD	-1.27	0.72	0	0	0	not significant

3259	GO_REGULATION_OF_HOMOTYPIC_CELL_CELL_ADHESION	-1.27	0.71	0	0	0	not significant
3260	REACTOME_2_LTR_CIRCLE_FORMATION	-1.27	0.71	0	0	0	not significant
3261	REACTOME_METHYLATION	-1.27	0.71	0	0	0	not significant
3262	GO_EPITHELIAL_CELL_FATE_COMMITMENT	-1.27	0.70	0	0	0	not significant
3263	GO_CDP_DIACYLGLYCEROL_BIOSYNTHETIC_PROCESS	-1.27	0.69	0	0	0	not significant
3264	GO_NEGATIVE_REGULATION_OF_DENDRITE_MORPHOGENESIS	-1.27	0.69	0	0	0	not significant
3265	GO_SPINDLE_MIDZONE_ASSEMBLY	-1.27	0.69	0	0	0	not significant
3266	GO_BRANCH_ELONGATION_OF_AN_EPITHELIUM	-1.27	0.68	0	0	0	not significant
3267	GO_MEIOTIC_SPINDLE	-1.27	0.68	0	0	0	not significant
3268	GO_SMALL_PROTEIN_ACTIVATING_ENZYME_BINDING	-1.27	0.68	0	0	0	not significant
3269	REACTOME_ACTIVATION_OF_RAS_IN_B_CELLS	-1.27	0.68	0	0	0	not significant
3270	GO_MACROPHAGE_APOPTOTIC_PROCESS	-1.27	0.67	0	0	0	not significant
3271	GO_NEGATIVE_REGULATION_OF_INTERLEUKIN_17_PRODUCTION	-1.27	0.67	0	0	0	not significant
3272	GO_RAGE_RECEPTOR_BINDING	-1.27	0.67	0	0	0	not significant
3273	GO_RESPONSE_TO_ISOLATION_STRESS	-1.27	0.67	0	0	0	not significant
3274	GO_PROTEIN_NITROSYLATION	-1.27	0.66	0	0	0	not significant
3275	GO_TRANSCRIPTIONAL_PREINITIATION_COMPLEX	-1.27	0.63	0	0	0	not significant
3276	GO_NEGATIVE_REGULATION_OF_MOLECULAR_FUNCTION	-1.26	4.00	0	0	0	not significant
3277	GO_INORGANIC_ION_TRANSMEMBRANE_TRANSPORT	-1.26	2.01	0	0	0	not significant
3278	RODWELL_AGING_KIDNEY_UP	-1.26	1.78	0	0	0	not significant
3279	GO_POLYMERIC_CYTOSKELETAL_FIBER	-1.26	1.76	0	0	0	not significant
3280	GO_BLOOD_VESSEL_MORPHOGENESIS	-1.26	1.56	0	0	0	not significant
3281	RIGGI_EWING_SARCOMA_PROGENITOR_UP	-1.26	1.42	0	0	0	not significant
3282	GO_MEMBRANE_REGION	-1.26	1.41	0	0	0	not significant
3283	GO_CELLULAR_RESPONSE_TO_REACTIVE_OXYGEN_SPECIES	-1.26	1.37	0	0	0	not significant
3284	YAMAZAKI_TCEB3_TARGETS_DN	-1.26	1.37	0	0	0	not significant
3285	WALLACE_PROSTATE_CANCER_RACE_UP	-1.26	1.27	0	0	0	not significant
3286	CHR3P21	-1.26	1.25	0	0	0	not significant
3287	PANGAS_TUMOR_SUPPRESSION_BY_SMAD1_AND_SMAD5_DN	-1.26	1.25	0	0	0	not significant
3288	REACTOME_ONCOGENIC_MAPK_SIGNALING	-1.26	1.08	0	0	0	not significant
3289	GO_MULTICELLULAR_ORGANISMAL_SIGNALING	-1.26	1.05	0	0	0	not significant
3290	MILI_PSEUDOPODIA_CHEMOTAXIS_UP	-1.26	1.05	0	0	0	not significant
3291	SEIDEN_ONCOGENESIS_BY_MET	-1.26	1.03	0	0	0	not significant
3292	LANDIS_ERBB2_BREAST_TUMORS_324_UP	-1.26	1.00	0	0	0	not significant
3293	GO_CYTOSKELETON_DEPENDENT_CYTOKINESIS	-1.26	0.98	0	0	0	not significant
3294	GO_POSITIVE_REGULATION_OF_PRODUCTION_OF_MOLECULAR_MEDIAT	-1.26	0.98	0	0	0	not significant
3295	MAYBURD_RESPONSE_TO_L663536_DN	-1.26	0.98	0	0	0	not significant
3296	GO_INTRAMOLECULAR_OXIDOREDUCTASE_ACTIVITY	-1.26	0.94	0	0	0	not significant
3297	GO_ATPASE_REGULATOR_ACTIVITY	-1.26	0.93	0	0	0	not significant
3298	PID_THROMBIN_PAR1_PATHWAY	-1.26	0.93	0	0	0	not significant
3299	BERNARD_PPAPDC1B_TARGETS_DN	-1.26	0.92	0	0	0	not significant
3300	IZADPANAH_STEM_CELL_ADIPOSE_VS_BONE_DN	-1.26	0.92	0	0	0	not significant
3301	KEGG_TYPE_II_DIABETES_MELLITUS	-1.26	0.91	0	0	0	not significant
3302	GAUSSMANN_MLL_AF4_FUSION_TARGETS_A_DN	-1.26	0.89	0	0	0	not significant
3303	GO_HISTONE_EXCHANGE	-1.26	0.83	0	0	0	not significant
3304	GO_NEGATIVE_REGULATION_OF_TOR_SIGNALING	-1.26	0.83	0	0	0	not significant
3305	HINATA_NFKB_TARGETS_KERATINOCYTE_UP	-1.26	0.83	0	0	0	not significant
3306	GALLUZZI_PERMEABILIZE_MITOCHONDRIA	-1.26	0.82	0	0	0	not significant
3307	GO_NEGATIVE_REGULATION_OF_RNA_SPLICING	-1.26	0.81	0	0	0	not significant
3308	GO_PROTEIN_REFOLDING	-1.26	0.81	0	0	0	not significant
3309	YIH_RESPONSE_TO_ARSENITE_C3	-1.26	0.81	0	0	0	not significant
3310	BROWNE_HCMV_INFECTION_1HR_UP	-1.26	0.80	0	0	0	not significant
3311	GO_REGULATION_OF_CELL_MIGRATION_INVOLVED_IN_SPROUTING_ANG	-1.26	0.80	0	0	0	not significant
3312	SCHLESINGER_METHYLATED_DE_NOVO_IN_CANCER	-1.26	0.80	0	0	0	not significant
3313	GO_ENDOCRINE_PANCREAS_DEVELOPMENT	-1.26	0.79	0	0	0	not significant
3314	REACTOME_SYNDECAN_INTERACTIONS	-1.26	0.78	0	0	0	not significant
3315	VALK_AML_CLUSTER_11	-1.26	0.78	0	0	0	not significant
3316	CERIBELLI_PROMOTERS_INACTIVE_AND_BOUND_BY_NFY	-1.26	0.77	0	0	0	not significant
3317	GO_REGULATION_OF_PROTEIN_LOCALIZATION_TO_CELL_SURFACE	-1.26	0.77	0	0	0	not significant
3318	GO_REGULATION_OF_OXIDATIVE_STRESS_INDUCED_INTRINSIC_APOPTC	-1.26	0.76	0	0	0	not significant
3319	HOFMANN_MYELODYSPLASTIC_SYNDROM_RISK_UP	-1.26	0.76	0	0	0	not significant
3320	TURASHVILI_BREAST_DUCTAL_CARCINOMA_VS_LOBULAR_NORMAL_DN	-1.26	0.76	0	0	0	not significant
3321	GO_MYOFIBRIL_ASSEMBLY	-1.26	0.75	0	0	0	not significant
3322	GO_POSITIVE_REGULATION_OF_TOLL_LIKE_RECEPTOR_SIGNALING_PA	-1.26	0.75	0	0	0	not significant
3323	REACTOME_RORA_ACTIVATES_GENE_EXPRESSION	-1.26	0.75	0	0	0	not significant
3324	GO_ACETYLCHOLINE_GATED_CHANNEL_COMPLEX	-1.26	0.74	0	0	0	not significant
3325	GO_FOREBRAIN_NEURON_DIFFERENTIATION	-1.26	0.74	0	0	0	not significant
3326	GO_PSEUDOPODIUM_ORGANIZATION	-1.26	0.74	0	0	0	not significant
3327	GO_POLYAMINE_BIOSYNTHETIC_PROCESS	-1.26	0.73	0	0	0	not significant
3328	GO_TRNA_CATABOLIC_PROCESS	-1.26	0.73	0	0	0	not significant
3329	GO_RESPONSE_TO_CAFFEINE	-1.26	0.72	0	0	0	not significant
3330	GO_MODIFIED_AMINO_ACID_TRANSMEMBRANE_TRANSPORTER_ACTIVIT	-1.26	0.71	0	0	0	not significant
3331	GO_RESPONSE_TO_L_GLUTAMATE	-1.26	0.71	0	0	0	not significant
3332	KUROKAWA_LIVER_CANCER_CHEMOTHERAPY_UP	-1.26	0.71	0	0	0	not significant
3333	ZIRN_TRETINOIN_RESPONSE_UP	-1.26	0.70	0	0	0	not significant
3334	GO_CHEMOKINE_C_C_MOTIF_LIGAND_2_SECRETION	-1.26	0.69	0	0	0	not significant
3335	GO_OXIDOREDUCTASE_ACTIVITY_ACTING_ON_NAD_P_H_HEME_PROTEI	-1.26	0.69	0	0	0	not significant
3336	GO_PROSTANOID_BIOSYNTHETIC_PROCESS	-1.26	0.69	0	0	0	not significant
3337	REACTOME_SHC1_EVENTS_IN_EGFR_SIGNALING	-1.26	0.69	0	0	0	not significant
3338	GO_MITOTIC_NUCLEAR_ENVELOPE_REASSEMBLY	-1.26	0.68	0	0	0	not significant
3339	GO_NEGATIVE_REGULATION_OF_PROGRAMMED_NECROTIC_CELL_DEA	-1.26	0.68	0	0	0	not significant
3340	GO_REGULATION_OF_PROTEIN_LOCALIZATION_TO_NUCLEOLUS	-1.26	0.68	0	0	0	not significant
3341	GO_POSITIVE_REGULATION_OF_MUSCLE_ADAPTATION	-1.26	0.67	0	0	0	not significant
3342	REACTOME_KETONE_BODY_METABOLISM	-1.26	0.67	0	0	0	not significant
3343	GO_REGULATION_OF_PROTEIN_HETERODIMERIZATION_ACTIVITY	-1.26	0.66	0	0	0	not significant
3344	GO_SODIUM_ION_EXPORT_ACROSS_PLASMA_MEMBRANE	-1.26	0.65	0	0	0	not significant
3345	GO_MAINTENANCE_OF_CENTROSOME_LOCATION	-1.26	0.64	0	0	0	not significant
3346	REACTOME_ACTIVATED_NTRK3_SIGNALS_THROUGH_RAS	-1.26	0.63	0	0	0	not significant
3347	REACTOME_P75NTR_REGULATES_AXONOGENESIS	-1.26	0.63	0	0	0	not significant
3348	GO_L_PHENYLALANINE_CATABOLIC_PROCESS	-1.26	0.62	0	0	0	not significant
3349	GO_REGULATION_OF_CATABOLIC_PROCESS	-1.25	4.00	0	0	0	not significant
3350	MARTINEZ_RB1_TARGETS_UP	-1.25	4.00	0	0	0	not significant
3351	GO_NEGATIVE_REGULATION_OF_CATALYTIC_ACTIVITY	-1.25	2.29	0	0	0	not significant
3352	GO_METHYLATION	-1.25	1.68	0	0	0	not significant
3353	GO_MICROTUBULE	-1.25	1.68	0	0	0	not significant
3354	GO_ACTIVATION_OF_IMMUNE_RESPONSE	-1.25	1.60	0	0	0	not significant
3355	GO_CHROMOSOMAL_REGION	-1.25	1.58	0	0	0	not significant
3356	GO_ORGANIC_HYDROXY_COMPOUND_METABOLIC_PROCESS	-1.25	1.56	0	0	0	not significant
3357	GO_POSITIVE_REGULATION_OF_PROTEOLYSIS	-1.25	1.53	0	0	0	not significant
3358	GO_IMMUNE_RESPONSE_REGULATING_CELL_SURFACE_RECEPTOR_SIG	-1.25	1.52	0	0	0	not significant
3359	GO_VIRAL_LIFE_CYCLE	-1.25	1.49	0	0	0	not significant
3360	GO_PROTEIN_MODIFICATION_BY_SMALL_PROTEIN_REMOVAL	-1.25	1.48	0	0	0	not significant
3361	GO_CALCIUM_ION_TRANSMEMBRANE_TRANSPORT	-1.25	1.32	0	0	0	not significant
3362	GO_MRNA_BINDING	-1.25	1.28	0	0	0	not significant
3363	GO_RESPONSE_TO_ALCOHOL	-1.25	1.18	0	0	0	not significant
3364	MASSARWEH_TAMOXIFEN_RESISTANCE_DN	-1.25	1.18	0	0	0	not significant
3365	SHEPARD_CRUSH_AND_BURN_MUTANT_DN	-1.25	1.16	0	0	0	not significant
3366	HALLMARK_KRAS_SIGNALING_UP	-1.25	1.15	0	0	0	not significant
3367	KUMAR_PATHOGEN_LOAD_BY_MACROPHAGES	-1.25	1.12	0	0	0	not significant

3368	RODRIGUES_NTN1_TARGETS_DN	-1.25	1.11	0	0	0	not significant
3369	REACTOME_FACTORS_INVOLVED_IN_MEGAKARYOCYTE_DEVELOPMENT	-1.25	1.10	0	0	0	not significant
3370	WIERENGA_STAT5A_TARGETS_UP	-1.25	1.10	0	0	0	not significant
3371	GUO_TARGETS_OF_IRS1_AND_IRS2	-1.25	1.08	0	0	0	not significant
3372	GO_POSITIVE_REGULATION_OF_VASCULATURE_DEVELOPMENT	-1.25	1.05	0	0	0	not significant
3373	YAMAZAKI_TCEB3_TARGETS_UP	-1.25	1.04	0	0	0	not significant
3374	GO_TISSUE_HOMEOSTASIS	-1.25	1.02	0	0	0	not significant
3375	CHENG_IMPRINTED_BY_ESTRADIOL	-1.25	0.98	0	0	0	not significant
3376	GO_PROTEIN_LOCALIZATION_TO_CHROMOSOME	-1.25	0.98	0	0	0	not significant
3377	KEGG_VASCULAR_SMOOTH_MUSCLE_CONTRACTION	-1.25	0.97	0	0	0	not significant
3378	GO_REGULATION_OF_REPRODUCTIVE_PROCESS	-1.25	0.96	0	0	0	not significant
3379	GO_NUCLEOSOME_BINDING	-1.25	0.94	0	0	0	not significant
3380	GRAHAM_CML_DIVIDING_VS_NORMAL_QUIESCENT_DN	-1.25	0.92	0	0	0	not significant
3381	GO_NEPHRON_EPITHELIUM_DEVELOPMENT	-1.25	0.91	0	0	0	not significant
3382	ISHIDA_E2F_TARGETS	-1.25	0.86	0	0	0	not significant
3383	LIEN_BREAST_CARCINOMA_METAPLASTIC_VS_DUCTAL_UP	-1.25	0.86	0	0	0	not significant
3384	GO_SISTER_CHROMATID_COHESION	-1.25	0.85	0	0	0	not significant
3385	GO_PROTEIN_TYROSINE_SERINE_THREONINE_PHOSPHATASE_ACTIVITY	-1.25	0.84	0	0	0	not significant
3386	REACTOME_CA2PLUS_PATHWAY	-1.25	0.84	0	0	0	not significant
3387	GO_REGULATION_OF_NITRIC_OXIDE_SYNTHASE_ACTIVITY	-1.25	0.83	0	0	0	not significant
3388	MALONEY_RESPONSE_TO_17AAG_UP	-1.25	0.83	0	0	0	not significant
3389	TURASHVILI_BREAST_LOBULAR_CARCINOMA_VS_LOBULAR_NORMAL_UF	-1.25	0.83	0	0	0	not significant
3390	ZHU_CMV_8_HR_UP	-1.25	0.83	0	0	0	not significant
3391	TURASHVILI_BREAST_LOBULAR_CARCINOMA_VS_DUCTAL_NORMAL_UP	-1.25	0.82	0	0	0	not significant
3392	PID_RET_PATHWAY	-1.25	0.80	0	0	0	not significant
3393	DORN_ADENOVIRUS_INFECTION_48HR_DN	-1.25	0.79	0	0	0	not significant
3394	GO_ANCHORED_COMPONENT_OF_PLASMA_MEMBRANE	-1.25	0.79	0	0	0	not significant
3395	GO_POSITIVE_CHEMOTAXIS	-1.25	0.79	0	0	0	not significant
3396	GO_RESPONSE_TO_ANESTHETIC	-1.25	0.78	0	0	0	not significant
3397	REACTOME_SUMOYLATION_OF_INTRACELLULAR_RECEPTORS	-1.25	0.78	0	0	0	not significant
3398	KEGG_BUTANOATE_METABOLISM	-1.25	0.77	0	0	0	not significant
3399	GO_POSITIVE_REGULATION_OF_LAMELLIPODIUM_ORGANIZATION	-1.25	0.76	0	0	0	not significant
3400	GO_REGULATION_OF_HELICASE_ACTIVITY	-1.25	0.76	0	0	0	not significant
3401	REACTOME_CARBOXYTERMINAL_POST_TRANSLATIONAL_MODIFICATION	-1.25	0.76	0	0	0	not significant
3402	REACTOME_RHO_GTPASES_ACTIVATE_ROCKS	-1.25	0.76	0	0	0	not significant
3403	ANASTASSIOU_CANCER_MESENCHYMAL_TRANSITION_SIGNATURE	-1.25	0.75	0	0	0	not significant
3404	GO_RESPONSE_TO_VITAMIN_D	-1.25	0.75	0	0	0	not significant
3405	GO_ALPHA_LINOLENIC_ACID_METABOLIC_PROCESS	-1.25	0.73	0	0	0	not significant
3406	GO_ENTRAINMENT_OF_CIRCADIAN_CLOCK	-1.25	0.73	0	0	0	not significant
3407	GO_NEGATIVE_REGULATION_OF_ALPHA_BETA_T_CELL_DIFFERENTIATI(	-1.25	0.73	0	0	0	not significant
3408	GO_CELL_COMMUNICATION_BY_ELECTRICAL_COUPLING	-1.25	0.72	0	0	0	not significant
3409	GO_EYE_PHOTORECEPTOR_CELL_DEVELOPMENT	-1.25	0.72	0	0	0	not significant
3410	GO_NEGATIVE_REGULATION_OF_SIGNAL_TRANSDUCTION_IN_ABSENCE_	-1.25	0.72	0	0	0	not significant
3411	GO_REGULATION_OF_MICROTUBULE_DEPOLYMERIZATION	-1.25	0.72	0	0	0	not significant
3412	REACTOME_ZINC_INFLUX_INTO_CELLS_BY_THE_SLC39_GENE_FAMILY	-1.25	0.71	0	0	0	not significant
3413	GO_PROTEIN_KINASE_A_CATALYTIC_SUBUNIT_BINDING	-1.25	0.70	0	0	0	not significant
3414	GO_RESPONSE_TO_PLATELET_DERIVED_GROWTH_FACTOR	-1.25	0.70	0	0	0	not significant
3415	GO_RESPONSE_TO_OLEIC_ACID	-1.25	0.69	0	0	0	not significant
3416	GO_NEGATIVE_REGULATION_OF_CARTILAGE_DEVELOPMENT	-1.25	0.68	0	0	0	not significant
3417	GO_NEGATIVE_REGULATION_OF_CHONDROCYTE_DIFFERENTIATION	-1.25	0.68	0	0	0	not significant
3418	GO_PROTEIN_CONTAINING_COMPLEX_REMODELING	-1.25	0.68	0	0	0	not significant
3419	GARGALOVIC_RESPONSE_TO_OXIDIZED_PHOSPHOLIPIDS_BLACK_DN	-1.25	0.67	0	0	0	not significant
3420	GAUTSCHI_SRC_SIGNALING	-1.25	0.67	0	0	0	not significant
3421	GO_POSITIVE_REGULATION_OF_MONOCYTE_DIFFERENTIATION	-1.25	0.67	0	0	0	not significant
3422	GO_POSITIVE_REGULATION_OF_NITRIC_OXIDE_SYNTHASE_BIOSYNTHET	-1.25	0.67	0	0	0	not significant
3423	BIOCARTA_RB_PATHWAY	-1.25	0.66	0	0	0	not significant
3424	GO_S_SHAPED_BODY_MORPHOGENESIS	-1.25	0.65	0	0	0	not significant
3425	WORSCHECH_TUMOR_REJECTION_DN	-1.25	0.65	0	0	0	not significant
3426	GO_REGULATION_OF_DNA_CATABOLIC_PROCESS	-1.25	0.64	0	0	0	not significant
3427	REACTOME_PHENYLALANINE_AND_TYROSINE_METABOLISM	-1.25	0.64	0	0	0	not significant
3428	GO_ACTIVATION_INDUCED_CELL_DEATH_OF_T_CELLS	-1.25	0.63	0	0	0	not significant
3429	GO_REGULATION_OF_MITOCHONDRIAL_ATP_SYNTHESIS_COUPLED_ELE	-1.25	0.63	0	0	0	not significant
3430	GO_HIGH_DENSITY_LIPOPROTEIN_PARTICLE_REMODELING	-1.25	0.62	0	0	0	not significant
3431	REACTOME_ACTIVATED_NTRK2_SIGNALS_THROUGH_RAS	-1.25	0.62	0	0	0	not significant
3432	GO_ENDOCYTIC_ADAPTOR_ACTIVITY	-1.25	0.60	0	0	0	not significant
3433	PILON_KLF1_TARGETS_DN	-1.24	4.00	0	0	0	not significant
3434	GO_MEMBRANE_PROTEIN_COMPLEX	-1.24	2.14	0	0	0	not significant
3435	GO_REGULATION_OF_ANATOMICAL_STRUCTURE_MORPHOGENESIS	-1.24	2.14	0	0	0	not significant
3436	GO_SECRETION	-1.24	1.92	0	0	0	not significant
3437	WANG_LMO4_TARGETS_DN	-1.24	1.61	0	0	0	not significant
3438	GO_ACTIN_FILAMENT_ORGANIZATION	-1.24	1.52	0	0	0	not significant
3439	GO_REGULATION_OF_BINDING	-1.24	1.50	0	0	0	not significant
3440	GO_SMALL_MOLECULE_CATABOLIC_PROCESS	-1.24	1.43	0	0	0	not significant
3441	GO_REGULATION_OF_MULTI_ORGANISM_PROCESS	-1.24	1.42	0	0	0	not significant
3442	RUTELLA_RESPONSE_TO_CSF2RB_AND_IL4_UP	-1.24	1.42	0	0	0	not significant
3443	GO_MUSCLE_ORGAN_DEVELOPMENT	-1.24	1.31	0	0	0	not significant
3444	GO_PROTEIN_DNA_COMPLEX_SUBUNIT_ORGANIZATION	-1.24	1.26	0	0	0	not significant
3445	GO_REGULATION_OF_PROTEIN_STABILITY	-1.24	1.26	0	0	0	not significant
3446	HAHTOLA_MYCOSIS_FUNGOIDES_SKIN_UP	-1.24	1.22	0	0	0	not significant
3447	ACEVEDO_FGFR1_TARGETS_IN_PROSTATE_CANCER_MODEL_UP	-1.24	1.21	0	0	0	not significant
3448	FLECHNER_PBL_KIDNEY_TRANSPLANT_OK_VS_DONOR_UP	-1.24	1.19	0	0	0	not significant
3449	GO_ORGANIC_ANION_TRANSPORT	-1.24	1.18	0	0	0	not significant
3450	GO_POSITIVE_REGULATION_OF_IMMUNE_EFFECTOR_PROCESS	-1.24	1.15	0	0	0	not significant
3451	ESC_J1_UP_EARLY.V1_DN	-1.24	1.10	0	0	0	not significant
3452	GO_CELLULAR_RESPONSE_TO_TOXIC_SUBSTANCE	-1.24	1.10	0	0	0	not significant
3453	TIEN_INTESTINE_PROBIOTICS_6HR_DN	-1.24	1.10	0	0	0	not significant
3454	GO_LIPID_HOMEOSTASIS	-1.24	1.01	0	0	0	not significant
3455	LU_AGING_BRAIN_DN	-1.24	1.00	0	0	0	not significant
3456	KIM_RESPONSE_TO_TSA_AND_DECITABINE_UP	-1.24	0.94	0	0	0	not significant
3457	GO_PERIKARYON	-1.24	0.93	0	0	0	not significant
3458	ACOSTA_PROLIFERATION_INDEPENDENT_MYC_TARGETS_DN	-1.24	0.92	0	0	0	not significant
3459	GO_INTEGRIN_BINDING	-1.24	0.89	0	0	0	not significant
3460	GO_REGULATION_OF_CALCIUM_ION_TRANSPORT_INTO_CYTOSOL	-1.24	0.89	0	0	0	not significant
3461	GO_MONOSACCHARIDE_BINDING	-1.24	0.88	0	0	0	not significant
3462	GO_ACID_SECRETION	-1.24	0.86	0	0	0	not significant
3463	GO_DRUG_CATABOLIC_PROCESS	-1.24	0.86	0	0	0	not significant
3464	REACTOME_NEGATIVE_REGULATION_OF_MAPK_PATHWAY	-1.24	0.86	0	0	0	not significant
3465	BOSCO_TH1_CYTOTOXIC_MODULE	-1.24	0.84	0	0	0	not significant
3466	GO_INTERLEUKIN_1_PRODUCTION	-1.24	0.84	0	0	0	not significant
3467	REACTOME_DNA_DOUBLE_STRAND_BREAK_RESPONSE	-1.24	0.84	0	0	0	not significant
3468	GO_CARDIAC_VENTRICLE_DEVELOPMENT	-1.24	0.82	0	0	0	not significant
3469	HUTTMANN_B_CLL_POOR_SURVIVAL_DN	-1.24	0.82	0	0	0	not significant
3470	GO_SOMATIC_STEM_CELL_POPULATION_MAINTENANCE	-1.24	0.81	0	0	0	not significant
3471	SESTO_RESPONSE_TO_UV_C2	-1.24	0.81	0	0	0	not significant
3472	GO_CHROMATIN_SILENCING_AT_RDNA	-1.24	0.80	0	0	0	not significant
3473	GO_POSITIVE_REGULATION_OF_GENE_EXPRESSION_EPIGENETIC	-1.24	0.80	0	0	0	not significant
3474	CHR7P15	-1.24	0.79	0	0	0	not significant
3475	GO_RELEASE_OF_CYTOCHROME_C_FROM_MITOCHONDRIA	-1.24	0.79	0	0	0	not significant
3476	REACTOME_BASE_EXCISION_REPAIR_AP_SITE_FORMATION	-1.24	0.79	0	0	0	not significant

3477	REACTOME_THROMBIN_SIGNALLING_THROUGH_PROTEINASE_ACTIVATE	-1.24	0.79	0	0	0	not significant
3478	LABBE_TGFB1_TARGETS_UP	-1.24	0.78	0	0	0	not significant
3479	GO_INORGANIC_ION_IMPORT_ACROSS_PLASMA_MEMBRANE	-1.24	0.77	0	0	0	not significant
3480	BOYAULT_LIVER_CANCER_SUBCLASS_G123_UP	-1.24	0.76	0	0	0	not significant
3481	GO_CELLULAR_RESPONSE_TO_ALKALOID	-1.24	0.76	0	0	0	not significant
3482	GO_DITERPENOID_METABOLIC_PROCESS	-1.24	0.76	0	0	0	not significant
3483	GO_VOLTAGE_GATED_POTASSIUM_CHANNEL_ACTIVITY	-1.24	0.75	0	0	0	not significant
3484	WEINMANN_ADAPTATION_TO_HYPOXIA_DN	-1.24	0.74	0	0	0	not significant
3485	GO_NEGATIVE_REGULATION_OF_TELOMERE_MAINTENANCE_VIA_TELOM	-1.24	0.73	0	0	0	not significant
3486	TOMIDA_METASTASIS_UP	-1.24	0.73	0	0	0	not significant
3487	GO_COPPER_ION_HOMEOSTASIS	-1.24	0.72	0	0	0	not significant
3488	GO_COPII_VESICLE_COAT	-1.24	0.71	0	0	0	not significant
3489	GO_POSITIVE_REGULATION_OF_MRNA_PROCESSING	-1.24	0.71	0	0	0	not significant
3490	GO_VITAMIN_B6_BINDING	-1.24	0.71	0	0	0	not significant
3491	REACTOME_EPHA_MEDIATED_GROWTH_CONE_COLLAPSE	-1.24	0.71	0	0	0	not significant
3492	BIOCARTA_PML_PATHWAY	-1.24	0.70	0	0	0	not significant
3493	GO_VASCULAR_ENDOTHELIAL_GROWTH_FACTOR_PRODUCTION	-1.24	0.70	0	0	0	not significant
3494	VALK_AML_CLUSTER_9	-1.24	0.70	0	0	0	not significant
3495	GO_CELL_CORTEX_REGION	-1.24	0.69	0	0	0	not significant
3496	GO_GLUTATHIONE_PEROXIDASE_ACTIVITY	-1.24	0.69	0	0	0	not significant
3497	GO_M_BAND	-1.24	0.69	0	0	0	not significant
3498	GO_MUSCLE_CELL_CELLULAR_HOMEOSTASIS	-1.24	0.69	0	0	0	not significant
3499	GO_REGULATION_OF_SYSTEMIC_ARTERIAL_BLOOD_PRESSURE_MEDIAT	-1.24	0.69	0	0	0	not significant
3500	GO_NEGATIVE_REGULATION_OF_CD4_POSITIVE_ALPHA_BETA_T_CELL_	-1.24	0.68	0	0	0	not significant
3501	GO_REGULATION_OF_NUCLEOBASE_CONTAINING_COMPOUND_TRANSP	-1.24	0.68	0	0	0	not significant
3502	GO_SEMAPHORIN_PLEXIN_SIGNALING_PATHWAY	-1.24	0.68	0	0	0	not significant
3503	GO_CELLULAR_RESPONSE_TO_THYROID_HORMONE_STIMULUS	-1.24	0.67	0	0	0	not significant
3504	GO_POSITIVE_REGULATION_OF_ACTIN_FILAMENT_DEPOLYMERIZATION	-1.24	0.67	0	0	0	not significant
3505	GO_REGULATION_OF_ASPARTIC_TYPE_PEPTIDASE_ACTIVITY	-1.24	0.67	0	0	0	not significant
3506	LIANG_SILENCED_BY_METHYLATION_DN	-1.24	0.67	0	0	0	not significant
3507	REACTOME_GLUTAMATE_AND_GLUTAMINE_METABOLISM	-1.24	0.67	0	0	0	not significant
3508	GO_TRABECULA_FORMATION	-1.24	0.66	0	0	0	not significant
3509	REACTOME_SYNTHESIS_OF_IP2_IP_AND_INS_IN_THE_CYTOSOL	-1.24	0.66	0	0	0	not significant
3510	IWANAGA_E2F1_TARGETS_NOT_INDUCED_BY_SERUM	-1.24	0.65	0	0	0	not significant
3511	REACTOME_ADP_SIGNALLING_THROUGH_P2Y_PURINOCEPTOR_1	-1.24	0.65	0	0	0	not significant
3512	REACTOME_ALPHA_LINOLENIC_OMEGA3_AND_LINOLEIC_OMEGA6_ACID_	-1.24	0.65	0	0	0	not significant
3513	GO_CELLULAR_RESPONSE_TO_MANGANESE_ION	-1.24	0.64	0	0	0	not significant
3514	GO_G_PROTEIN_COUPLED_PURINERGIC_RECEPTOR_SIGNALING_PATHW	-1.24	0.64	0	0	0	not significant
3515	GO_POSITIVE_REGULATION_OF_METANEPHROS_DEVELOPMENT	-1.24	0.64	0	0	0	not significant
3516	GO_TRNA_THIO_MODIFICATION	-1.24	0.64	0	0	0	not significant
3517	BIOCARTA_DNAFRAGMENT_PATHWAY	-1.24	0.63	0	0	0	not significant
3518	GO_CYTIDYLYLTRANSFERASE_ACTIVITY	-1.24	0.63	0	0	0	not significant
3519	GO_REGULATION_OF_MACROPHAGE_APOPTOTIC_PROCESS	-1.24	0.63	0	0	0	not significant
3520	GO_IMPORTIN_ALPHA_FAMILY_PROTEIN_BINDING	-1.24	0.62	0	0	0	not significant
3521	REACTOME_APOBEC3G_MEDIATED_RESISTANCE_TO_HIV_1_INFECTION	-1.24	0.62	0	0	0	not significant
3522	GO_AMP_METABOLIC_PROCESS	-1.24	0.61	0	0	0	not significant
3523	GO_SULFUR_AMINO_ACID_CATABOLIC_PROCESS	-1.24	0.61	0	0	0	not significant
3524	GO_ANATOMICAL_STRUCTURE_REGRESSION	-1.24	0.59	0	0	0	not significant
3525	GO_CELLULAR_HOMEOSTASIS	-1.23	4.00	0	0	0	not significant
3526	GO_ORGANONITROGEN_COMPOUND_BIOSYNTHETIC_PROCESS	-1.23	4.00	0	0	0	not significant
3527	WAKABAYASHI_ADIPOGENESIS_PPARG_RXRA_BOUND_8D	-1.23	2.15	0	0	0	not significant
3528	GO_CELL_SUBSTRATE_JUNCTION	-1.23	2.06	0	0	0	not significant
3529	GRYDER_PAX3FOXO1_TOP_ENHANCERS	-1.23	1.84	0	0	0	not significant
3530	MARTENS_BOUND_BY_PML_RARA_FUSION	-1.23	1.66	0	0	0	not significant
3531	LOCKWOOD_AMPLIFIED_IN_LUNG_CANCER	-1.23	1.51	0	0	0	not significant
3532	GO_DEFENSE_RESPONSE_TO_VIRUS	-1.23	1.47	0	0	0	not significant
3533	GO_WOUND_HEALING	-1.23	1.41	0	0	0	not significant
3534	REACTOME_ANTIGEN_PROCESSING:_UBIQUITINATION_PROTEASOME_DE	-1.23	1.39	0	0	0	not significant
3535	GO_NEURON_DEATH	-1.23	1.34	0	0	0	not significant
3536	GO_LEUKOCYTE_MIGRATION	-1.23	1.32	0	0	0	not significant
3537	BROWNE_HCMV_INFECTION_14HR_DN	-1.23	1.25	0	0	0	not significant
3538	REACTOME_RRNA_PROCESSING	-1.23	1.21	0	0	0	not significant
3539	SWEET_LUNG_CANCER_KRAS_DN	-1.23	1.17	0	0	0	not significant
3540	PDGF_UP.V1_UP	-1.23	1.06	0	0	0	not significant
3541	REACTOME_EPIGENETIC_REGULATION_OF_GENE_EXPRESSION	-1.23	1.03	0	0	0	not significant
3542	MIKKELSEN_MCV6_LCP_WITH_H3K4ME3	-1.23	1.02	0	0	0	not significant
3543	GO_ALPHA_AMINO_ACID_METABOLIC_PROCESS	-1.23	1.01	0	0	0	not significant
3544	GO_REGULATION_OF_CELL_CYCLE_G1_S_PHASE_TRANSITION	-1.23	1.00	0	0	0	not significant
3545	WARTERS_RESPONSE_TO_IR_SKIN	-1.23	1.00	0	0	0	not significant
3546	BRUECKNER_TARGETS_OF_MIRLET7A3_UP	-1.23	0.96	0	0	0	not significant
3547	PLASARI_TGFB1_TARGETS_10HR_UP	-1.23	0.96	0	0	0	not significant
3548	GO_REGULATION_OF_CALCIUM_MEDIATED_SIGNALING	-1.23	0.94	0	0	0	not significant
3549	REACTOME_GPCR_LIGAND_BINDING	-1.23	0.94	0	0	0	not significant
3550	SRC_UP.V1_DN	-1.23	0.93	0	0	0	not significant
3551	GO_DNA_DEPENDENT_ATPASE_ACTIVITY	-1.23	0.92	0	0	0	not significant
3552	REACTOME_GLOBAL_GENOME_NUCLEOTIDE_EXCISION_REPAIR_GG_NE	-1.23	0.89	0	0	0	not significant
3553	KEGG_TIGHT_JUNCTION	-1.23	0.88	0	0	0	not significant
3554	BREDEMEYER_RAG_SIGNALING_NOT_VIA_ATM_UP	-1.23	0.87	0	0	0	not significant
3555	GO_TRANSPORT_OF_VIRUS	-1.23	0.87	0	0	0	not significant
3556	REACTOME_CIRCADIAN_CLOCK	-1.23	0.87	0	0	0	not significant
3557	GO_CAJAL_BODY	-1.23	0.86	0	0	0	not significant
3558	GO_DRUG_TRANSMEMBRANE_TRANSPORT	-1.23	0.86	0	0	0	not significant
3559	GO_FLAVIN_ADENINE_DINUCLEOTIDE_BINDING	-1.23	0.86	0	0	0	not significant
3560	GO_PROTEIN_TRANSMEMBRANE_TRANSPORT	-1.23	0.86	0	0	0	not significant
3561	GO_GDP_BINDING	-1.23	0.85	0	0	0	not significant
3562	GO_REGULATION_OF_MITOCHONDRIAL_MEMBRANE_POTENTIAL	-1.23	0.84	0	0	0	not significant
3563	LIN_NPAS4_TARGETS_DN	-1.23	0.84	0	0	0	not significant
3564	GO_INOSITOL_PHOSPHATE_MEDIATED_SIGNALING	-1.23	0.83	0	0	0	not significant
3565	GO_REGULATION_OF_OXIDATIVE_STRESS_INDUCED_CELL_DEATH	-1.23	0.83	0	0	0	not significant
3566	REACTOME_REGULATION_OF_ACTIN_DYNAMICS_FOR_PHAGOCYTIC_CU	-1.23	0.82	0	0	0	not significant
3567	BRUECKNER_TARGETS_OF_MIRLET7A3_DN	-1.23	0.81	0	0	0	not significant
3568	GO_CELL_CELL_RECOGNITION	-1.23	0.80	0	0	0	not significant
3569	GO_MIDBRAIN_DEVELOPMENT	-1.23	0.79	0	0	0	not significant
3570	GO_STRIATED_MUSCLE_ADAPTATION	-1.23	0.78	0	0	0	not significant
3571	NADLER_OBESITY_DN	-1.23	0.78	0	0	0	not significant
3572	GO_REGULATION_OF_SODIUM_ION_TRANSMEMBRANE_TRANSPORTER_	-1.23	0.77	0	0	0	not significant
3573	GOTZMANN_EPITHELIAL_TO_MESENCHYMAL_TRANSITION_UP	-1.23	0.77	0	0	0	not significant
3574	GO_REGULATION_OF_MUSCLE_HYPERTROPHY	-1.23	0.76	0	0	0	not significant
3575	GO_HOMEOSTASIS_OF_NUMBER_OF_CELLS_WITHIN_A_TISSUE	-1.23	0.75	0	0	0	not significant
3576	GO_RETINA_HOMEOSTASIS	-1.23	0.75	0	0	0	not significant
3577	ROSS_AML_WITH_CBFB_MYH11_FUSION	-1.23	0.75	0	0	0	not significant
3578	GO_METANEPHROS_DEVELOPMENT	-1.23	0.74	0	0	0	not significant
3579	GO_LIVER_REGENERATION	-1.23	0.72	0	0	0	not significant
3580	HOFMANN_MYELODYSPLASTIC_SYNDROM_LOW_RISK_UP	-1.23	0.72	0	0	0	not significant
3581	GO_POSITIVE_REGULATION_OF_CYTOKINESIS	-1.23	0.71	0	0	0	not significant
3582	TIAN_TNF_SIGNALING_NOT_VIA_NFKB	-1.23	0.71	0	0	0	not significant
3583	YORDY_RECIPROCAL_REGULATION_BY_ETS1_AND_SP100_UP	-1.23	0.70	0	0	0	not significant
3584	BIOCARTA_CSK_PATHWAY	-1.23	0.69	0	0	0	not significant
3585	GO_AUTONOMIC_NERVOUS_SYSTEM_DEVELOPMENT	-1.23	0.69	0	0	0	not significant

3586	GO_DNA_DEALKYLATION	-1.23	0.69	0	0	0	not significant
3587	GO_REGULATION_OF_GLIAL_CELL_PROLIFERATION	-1.23	0.69	0	0	0	not significant
3588	GO_EPITHELIAL_CELL_DIFFERENTIATION_INVOLVED_IN_KIDNEY_DEVELO	-1.23	0.67	0	0	0	not significant
3589	REACTOME_NUCLEAR_ENVELOPE_REASSEMBLY	-1.23	0.67	0	0	0	not significant
3590	REACTOME_TRAF6_MEDIATED_IRF7_ACTIVATION_IN_TLR7_8_OR_9_SIG	-1.23	0.67	0	0	0	not significant
3591	GO_MEIOTIC_SPINDLE_ORGANIZATION	-1.23	0.66	0	0	0	not significant
3592	GO_PLATELET_MORPHOGENESIS	-1.23	0.66	0	0	0	not significant
3593	RAMPON_ENRICHED_LEARNING_ENVIRONMENT_EARLY_DN	-1.23	0.66	0	0	0	not significant
3594	BIOCARTA_PTC1_PATHWAY	-1.23	0.65	0	0	0	not significant
3595	GO_C_ACETYLTRANSFERASE_ACTIVITY	-1.23	0.65	0	0	0	not significant
3596	REACTOME_REDUCTION_OF_CYTOSOLIC_CAPLUSPLUS_LEVELS	-1.23	0.65	0	0	0	not significant
3597	GO_AGGREPHAGY	-1.23	0.64	0	0	0	not significant
3598	GO_CORONARY_VASCULATURE_MORPHOGENESIS	-1.23	0.64	0	0	0	not significant
3599	GO_MEMBRANE_DEPOLARIZATION_DURING_CARDIAC_MUSCLE_CELL_AC	-1.23	0.64	0	0	0	not significant
3600	GO_OXIDOREDUCTASE_ACTIVITY_ACTING_ON_PAIRED_DONORS_WITH_	-1.23	0.64	0	0	0	not significant
3601	GO_PROTEOGLYCAN_BINDING	-1.23	0.64	0	0	0	not significant
3602	GO_REGULATION_OF_MEMBRANE_REPOLARIZATION	-1.23	0.64	0	0	0	not significant
3603	GO_SNORNA_3_END_PROCESSING	-1.23	0.64	0	0	0	not significant
3604	HOFMANN_MYELODYSPLASTIC_SYNDROM_HIGH_RISK_DN	-1.23	0.64	0	0	0	not significant
3605	REACTOME_VLDLR_INTERNALISATION_AND_DEGRADATION	-1.23	0.64	0	0	0	not significant
3606	GO_DOLICHYL_DIPHOSPHATE_BIOSYNTHETIC_PROCESS	-1.23	0.63	0	0	0	not significant
3607	GO_POTASSIUM_CHANNEL_INHIBITOR_ACTIVITY	-1.23	0.63	0	0	0	not significant
3608	REACTOME_GRB2:SOS_PROVIDES_LINKAGE_TO_MAPK_SIGNALING_FOR	-1.23	0.63	0	0	0	not significant
3609	WANG_ESOPHAGUS_CANCER_PROGRESSION_UP	-1.23	0.63	0	0	0	not significant
3610	GO_NEGATIVE_REGULATION_OF_HELICASE_ACTIVITY	-1.23	0.62	0	0	0	not significant
3611	GO_REGULATION_OF_DNA_DUPLEX_UNWINDING	-1.23	0.62	0	0	0	not significant
3612	FRIDMAN_SENESCENCE_DN	-1.23	0.61	0	0	0	not significant
3613	GO_CAMERA_TYPE_EYE_PHOTORECEPTOR_CELL_DIFFERENTIATION	-1.23	0.61	0	0	0	not significant
3614	GO_MITOCHONDRIAL_RESPIRATORY_CHAIN_COMPLEX_III_ASSEMBLY	-1.23	0.61	0	0	0	not significant
3615	GO_POSITIVE_REGULATION_OF_IMMUNOGLOBULIN_SECRETION	-1.23	0.60	0	0	0	not significant
3616	GO_SPERMATOPROTEASOME_COMPLEX	-1.23	0.60	0	0	0	not significant
3617	REACTOME_SHC1_EVENTS_IN_ERBB4_SIGNALING	-1.23	0.60	0	0	0	not significant
3618	GO_IDENTICAL_PROTEIN_BINDING	-1.22	4.00	0	0	0	not significant
3619	GO_LEUKOCYTE_MEDIATED_IMMUNITY	-1.22	4.00	0	0	0	not significant
3620	GRAESSMANN_RESPONSE_TO_MC_AND_DOXORUBICIN_DN	-1.22	4.00	0	0	0	not significant
3621	SCHLOSSER_SERUM_RESPONSE_DN	-1.22	1.82	0	0	0	not significant
3622	GO_HEART_DEVELOPMENT	-1.22	1.63	0	0	0	not significant
3623	GO_IMMUNE_RESPONSE_REGULATING_SIGNALING_PATHWAY	-1.22	1.63	0	0	0	not significant
3624	DELACROIX_RARG_BOUND_MEF	-1.22	1.31	0	0	0	not significant
3625	GO_REGULATION_OF_CELL_ADHESION	-1.22	1.31	0	0	0	not significant
3626	REACTOME_TRANSCRIPTIONAL_REGULATION_BY_RUNX1	-1.22	1.31	0	0	0	not significant
3627	HOLLMANN_APOPTOSIS_VIA_CD40_UP	-1.22	1.29	0	0	0	not significant
3628	GO_EPITHELIAL_TUBE_MORPHOGENESIS	-1.22	1.23	0	0	0	not significant
3629	MEISSNER_NPC_HCP_WITH_H3K4ME2	-1.22	1.23	0	0	0	not significant
3630	REACTOME_SIGNALING_BY_GPCR	-1.22	1.23	0	0	0	not significant
3631	GO_DNA_CONFORMATION_CHANGE	-1.22	1.19	0	0	0	not significant
3632	PURBEY_TARGETS_OF_CTBP1_NOT_SATB1_UP	-1.22	1.15	0	0	0	not significant
3633	GO_PROTEIN_MATURATION	-1.22	1.13	0	0	0	not significant
3634	REACTOME_NEURONAL_SYSTEM	-1.22	1.12	0	0	0	not significant
3635	GO_REGULATION_OF_RNA_METABOLIC_PROCESS	-1.22	1.09	0	0	0	not significant
3636	GO_CONNECTIVE_TISSUE_DEVELOPMENT	-1.22	1.08	0	0	0	not significant
3637	GO_REGULATION_OF_CYSTEINE_TYPE_ENDOPEPTIDASE_ACTIVITY	-1.22	1.08	0	0	0	not significant
3638	GO_REGULATION_OF_PROTEASOMAL_PROTEIN_CATABOLIC_PROCESS	-1.22	1.06	0	0	0	not significant
3639	GRUETZMANN_PANCREATIC_CANCER_DN	-1.22	1.04	0	0	0	not significant
3640	GARGALOVIC_RESPONSE_TO_OXIDIZED_PHOSPHOLIPIDS_BLUE_UP	-1.22	1.00	0	0	0	not significant
3641	GO_VACUOLAR_LUMEN	-1.22	1.00	0	0	0	not significant
3642	ACEVEDO_LIVER_CANCER_WITH_H3K27ME3_DN	-1.22	0.99	0	0	0	not significant
3643	GO_MICROTUBULE_ASSOCIATED_COMPLEX	-1.22	0.98	0	0	0	not significant
3644	KIM_WT1_TARGETS_8HR_UP	-1.22	0.97	0	0	0	not significant
3645	GO_MYELOID_CELL_HOMEOSTASIS	-1.22	0.90	0	0	0	not significant
3646	GO_ORGANIC_ACID_TRANSMEMBRANE_TRANSPORTER_ACTIVITY	-1.22	0.90	0	0	0	not significant
3647	GO_PURINE_NTP_DEPENDENT_HELICASE_ACTIVITY	-1.22	0.90	0	0	0	not significant
3648	GO_PROTEIN_SERINE_THREONINE_PHOSPHATASE_ACTIVITY	-1.22	0.89	0	0	0	not significant
3649	GO_REGULATION_OF_RESPONSE_TO_WOUNDING	-1.22	0.89	0	0	0	not significant
3650	CHR10Q22	-1.22	0.88	0	0	0	not significant
3651	GO_GOLGI_VESICLE_BUDDING	-1.22	0.86	0	0	0	not significant
3652	CHANG_IMMORTALIZED_BY_HPV31_UP	-1.22	0.85	0	0	0	not significant
3653	GO_PLATELET_ALPHA_GRANULE	-1.22	0.84	0	0	0	not significant
3654	HUMMERICH_SKIN_CANCER_PROGRESSION_UP	-1.22	0.84	0	0	0	not significant
3655	SCIBETTA_KDM5B_TARGETS_DN	-1.22	0.84	0	0	0	not significant
3656	REACTOME_ACTIVATED_PKN1_STIMULATES_TRANSCRIPTION_OF_AR_A	-1.22	0.83	0	0	0	not significant
3657	NUTT_GBM_VS_AO_GLIOMA_UP	-1.22	0.81	0	0	0	not significant
3658	GO_IMPORT_ACROSS_PLASMA_MEMBRANE	-1.22	0.80	0	0	0	not significant
3659	MA_MYELOID_DIFFERENTIATION_UP	-1.22	0.80	0	0	0	not significant
3660	MOLENAAR_TARGETS_OF_CCND1_AND_CDK4_DN	-1.22	0.79	0	0	0	not significant
3661	REACTOME_IRE1ALPHA_ACTIVATES_CHAPERONES	-1.22	0.79	0	0	0	not significant
3662	GO_POSITIVE_REGULATION_OF_FIBROBLAST_PROLIFERATION	-1.22	0.78	0	0	0	not significant
3663	LINDVALL_IMMORTALIZED_BY_TERT_DN	-1.22	0.78	0	0	0	not significant
3664	PID_HIV_NEF_PATHWAY	-1.22	0.77	0	0	0	not significant
3665	GO_REGULATION_OF_T_CELL_APOPTOTIC_PROCESS	-1.22	0.76	0	0	0	not significant
3666	ZHAN_MULTIPLE_MYELOMA_MF_UP	-1.22	0.75	0	0	0	not significant
3667	GO_NEUROTROPHIN_SIGNALING_PATHWAY	-1.22	0.74	0	0	0	not significant
3668	GAVIN_FOXP3_TARGETS_CLUSTER_P2	-1.22	0.71	0	0	0	not significant
3669	GO_DNA_DEMETHYLATION	-1.22	0.71	0	0	0	not significant
3670	GO_DNA_HELICASE_COMPLEX	-1.22	0.71	0	0	0	not significant
3671	GO_NUCLEOSIDE_MONOPHOSPHATE_KINASE_ACTIVITY	-1.22	0.71	0	0	0	not significant
3672	GO_POSITIVE_REGULATION_OF_MULTICELLULAR_ORGANISM_GROWTH	-1.22	0.71	0	0	0	not significant
3673	WILCOX_RESPONSE_TO_PROGESTERONE_DN	-1.22	0.70	0	0	0	not significant
3674	GO_REGULATION_OF_MEMBRANE_DEPOLARIZATION	-1.22	0.69	0	0	0	not significant
3675	PID_UPA_UPAR_PATHWAY	-1.22	0.69	0	0	0	not significant
3676	GO_DENDRITE_CYTOPLASM	-1.22	0.67	0	0	0	not significant
3677	GO_FOAM_CELL_DIFFERENTIATION	-1.22	0.67	0	0	0	not significant
3678	GO_POSITIVE_REGULATION_OF_ALCOHOL_BIOSYNTHETIC_PROCESS	-1.22	0.67	0	0	0	not significant
3679	GO_POSITIVE_REGULATION_OF_HEART_GROWTH	-1.22	0.67	0	0	0	not significant
3680	GO_PROTEIN_HOMOTRIMERIZATION	-1.22	0.67	0	0	0	not significant
3681	BIOCARTA_TCRA_PATHWAY	-1.22	0.66	0	0	0	not significant
3682	GO_GLUTATHIONE_DERIVATIVE_BIOSYNTHETIC_PROCESS	-1.22	0.66	0	0	0	not significant
3683	GO_ANKYRIN_BINDING	-1.22	0.64	0	0	0	not significant
3684	GO_GLUTAMINE_FAMILY_AMINO_ACID_CATABOLIC_PROCESS	-1.22	0.64	0	0	0	not significant
3685	GO_ESTABLISHMENT_OR_MAINTENANCE_OF_ACTIN_CYTOSKELETON_PO	-1.22	0.63	0	0	0	not significant
3686	GO_ESTROUS_CYCLE	-1.22	0.63	0	0	0	not significant
3687	GO_MHC_CLASS_I_PROTEIN_COMPLEX	-1.22	0.63	0	0	0	not significant
3688	GO_TRNA_AMINOACYLATION_FOR_MITOCHONDRIAL_PROTEIN_TRANSLA	-1.22	0.63	0	0	0	not significant
3689	OHASHI_AURKB_TARGETS	-1.22	0.62	0	0	0	not significant
3690	VALK_AML_CLUSTER_5	-1.22	0.62	0	0	0	not significant
3691	GO_ALANINE_TRANSMEMBRANE_TRANSPORTER_ACTIVITY	-1.22	0.61	0	0	0	not significant
3692	GO_CYTOPLASMIC_TRANSLATIONAL_ELONGATION	-1.22	0.61	0	0	0	not significant
3693	GO_GALACTOSE_CATABOLIC_PROCESS_VIA_UDP_GALACTOSE	-1.22	0.61	0	0	0	not significant
3694	GO_SPERM_MIDPIECE	-1.22	0.61	0	0	0	not significant

3695	GO_NEGATIVE_REGULATION_OF_TRANSLATION_IN_RESPONSE_TO_STR	-1.22	0.60	0	0	0	not significant
3696	GO_PRESYNAPTIC_ACTIVE_ZONE_CYTOPLASMIC_COMPONENT	-1.22	0.60	0	0	0	not significant
3697	MYLLYKANGAS_AMPLIFICATION_HOT_SPOT_27	-1.22	0.60	0	0	0	not significant
3698	REACTOME_PP2A_MEDIATED_DEPHOSPHORYLATION_OF_KEY_METABO	-1.22	0.60	0	0	0	not significant
3699	GO_L_ALANINE_TRANSPORT	-1.22	0.58	0	0	0	not significant
3700	GO_POSITIVE_REGULATION_OF_URINE_VOLUME	-1.22	0.57	0	0	0	not significant
3701	MISHRA_CARCINOMA_ASSOCIATED_FIBROBLAST_UP	-1.22	0.57	0	0	0	not significant
3702	GO_PHOSPHATIDYLCHOLINE_CATABOLIC_PROCESS	-1.22	0.56	0	0	0	not significant
3703	GO_ZONA_PELLUCIDA_RECEPTOR_COMPLEX	-1.22	0.55	0	0	0	not significant
3704	GO_NEGATIVE_REGULATION_OF_CELL_DEATH	-1.21	2.14	0	0	0	not significant
3705	GO_CELL_ACTIVATION_INVOLVED_IN_IMMUNE_RESPONSE	-1.21	1.95	0	0	0	not significant
3706	GO_PROTEASOMAL_PROTEIN_CATABOLIC_PROCESS	-1.21	1.94	0	0	0	not significant
3707	GO_CELLULAR_RESPONSE_TO_ENDOGENOUS_STIMULUS	-1.21	1.78	0	0	0	not significant
3708	GO_PROTEIN_COMPLEX_OLIGOMERIZATION	-1.21	1.70	0	0	0	not significant
3709	GO_ENZYME_ACTIVATOR_ACTIVITY	-1.21	1.56	0	0	0	not significant
3710	GO_CYTOPLASMIC_REGION	-1.21	1.54	0	0	0	not significant
3711	GO_LIPID_BIOSYNTHETIC_PROCESS	-1.21	1.51	0	0	0	not significant
3712	MARTINEZ_RESPONSE_TO_TRABECTEDIN_DN	-1.21	1.36	0	0	0	not significant
3713	FORTSCHEGGER_PHF8_TARGETS_UP	-1.21	1.20	0	0	0	not significant
3714	WELCSH_BRCA1_TARGETS_UP	-1.21	1.12	0	0	0	not significant
3715	GO_REGULATION_OF_CELL_SHAPE	-1.21	1.03	0	0	0	not significant
3716	GO_NEGATIVE_REGULATION_OF_MULTI_ORGANISM_PROCESS	-1.21	1.01	0	0	0	not significant
3717	GO_MICROBODY	-1.21	0.98	0	0	0	not significant
3718	GO_ERK1_AND_ERK2_CASCADE	-1.21	0.97	0	0	0	not significant
3719	BOSCO_ALLERGEN_INDUCED_TH2_ASSOCIATED_MODULE	-1.21	0.96	0	0	0	not significant
3720	GO_SULFUR_COMPOUND_BIOSYNTHETIC_PROCESS	-1.21	0.96	0	0	0	not significant
3721	GO_NUCLEAR_CHROMOSOME_TELOMERIC_REGION	-1.21	0.92	0	0	0	not significant
3722	GO_SERINE_HYDROLASE_ACTIVITY	-1.21	0.88	0	0	0	not significant
3723	GO_ORGAN_GROWTH	-1.21	0.85	0	0	0	not significant
3724	GO_ACTIN_FILAMENT	-1.21	0.84	0	0	0	not significant
3725	GO_REGULATION_OF_VIRAL_GENOME_REPLICATION	-1.21	0.84	0	0	0	not significant
3726	GEORGES_CELL_CYCLE_MIR192_TARGETS	-1.21	0.81	0	0	0	not significant
3727	GO_INTRINSIC_APOPTOTIC_SIGNALING_PATHWAY_IN_RESPONSE_TO_EN	-1.21	0.81	0	0	0	not significant
3728	GO_AXON_CYTOPLASM	-1.21	0.80	0	0	0	not significant
3729	LEE_EARLY_T_LYMPHOCYTE_DN	-1.21	0.80	0	0	0	not significant
3730	CHR11Q14	-1.21	0.79	0	0	0	not significant
3731	LEE_CALORIE_RESTRICTION_NEOCORTEX_DN	-1.21	0.79	0	0	0	not significant
3732	PID_NFAT_3PATHWAY	-1.21	0.77	0	0	0	not significant
3733	GO_RUFFLE_ASSEMBLY	-1.21	0.76	0	0	0	not significant
3734	JIANG_AGING_CEREBRAL_CORTEX_UP	-1.21	0.76	0	0	0	not significant
3735	SMIRNOV_RESPONSE_TO_IR_2HR_UP	-1.21	0.75	0	0	0	not significant
3736	GO_POSITIVE_REGULATION_OF_ATPASE_ACTIVITY	-1.21	0.74	0	0	0	not significant
3737	GO_COLUMNAR_CUBOIDAL_EPITHELIAL_CELL_DIFFERENTIATION	-1.21	0.73	0	0	0	not significant
3738	GO_HYDRO_LYASE_ACTIVITY	-1.21	0.73	0	0	0	not significant
3739	GO_T_CELL_MIGRATION	-1.21	0.72	0	0	0	not significant
3740	GO_TIGHT_JUNCTION_ORGANIZATION	-1.21	0.72	0	0	0	not significant
3741	GO_CARDIAC_MUSCLE_TISSUE_MORPHOGENESIS	-1.21	0.71	0	0	0	not significant
3742	PID_ARF6_PATHWAY	-1.21	0.71	0	0	0	not significant
3743	GO_MEMBRANE_BIOGENESIS	-1.21	0.70	0	0	0	not significant
3744	WESTON_VEGFA_TARGETS_3HR	-1.21	0.69	0	0	0	not significant
3745	GO_LIPOPROTEIN_PARTICLE_RECEPTOR_BINDING	-1.21	0.68	0	0	0	not significant
3746	GO_REGULATION_OF_VIRAL_ENTRY_INTO_HOST_CELL	-1.21	0.68	0	0	0	not significant
3747	GO_BILE_ACID_BIOSYNTHETIC_PROCESS	-1.21	0.67	0	0	0	not significant
3748	GO_REGULATION_OF_HISTONE_UBIQUITINATION	-1.21	0.66	0	0	0	not significant
3749	VISALA_RESPONSE_TO_HEAT_SHOCK_AND_AGING_DN	-1.21	0.65	0	0	0	not significant
3750	GO_ANNEALING_ACTIVITY	-1.21	0.64	0	0	0	not significant
3751	GO_CARBOHYDRATE_PHOSPHORYLATION	-1.21	0.64	0	0	0	not significant
3752	ONDER_CDH1_TARGETS_3_UP	-1.21	0.64	0	0	0	not significant
3753	REACTOME_NEF_MEDIATED_CD8_DOWN_REGULATION	-1.21	0.64	0	0	0	not significant
3754	REACTOME_RUNX3_REGULATES_NOTCH_SIGNALING	-1.21	0.64	0	0	0	not significant
3755	GO_CYTOPLASMIC_EXOSOME_RNASE_COMPLEX_	-1.21	0.63	0	0	0	not significant
3756	HERNANDEZ_ABERRANT_MITOSIS_BY_DOCETACEL_4NM_UP	-1.21	0.63	0	0	0	not significant
3757	REACTOME_PROCESSING_OF_SMDT1	-1.21	0.63	0	0	0	not significant
3758	GO_ASPARTATE_FAMILY_AMINO_ACID_BIOSYNTHETIC_PROCESS	-1.21	0.62	0	0	0	not significant
3759	GO_MHC_PROTEIN_COMPLEX	-1.21	0.62	0	0	0	not significant
3760	CHEN_LVAD_SUPPORT_OF_FAILING_HEART_DN	-1.21	0.61	0	0	0	not significant
3761	GO_INTRAMOLECULAR_OXIDOREDUCTASE_ACTIVITY_TRANSPOSING_C	-1.21	0.60	0	0	0	not significant
3762	GO_NEGATIVE_REGULATION_OF_CELL_GROWTH_INVOLVED_IN_CARDIA	-1.21	0.60	0	0	0	not significant
3763	GO_NOTOCHORD_DEVELOPMENT	-1.21	0.60	0	0	0	not significant
3764	GO_PROTEIN_MATURATION_BY_PROTEIN_FOLDING	-1.21	0.60	0	0	0	not significant
3765	GO_MITOCHONDRIAL_DNA_REPLICATION	-1.21	0.59	0	0	0	not significant
3766	GO_NEGATIVE_REGULATION_OF_ENDOPLASMIC_RETICULUM_CALCIUM_	-1.21	0.59	0	0	0	not significant
3767	GO_POSITIVE_REGULATION_OF_EXTRINSIC_APOPTOTIC_SIGNALING_PA	-1.21	0.59	0	0	0	not significant
3768	GO_HISTONE_METHYLTRANSFERASE_BINDING	-1.21	0.58	0	0	0	not significant
3769	GO_REGULATION_OF_INTEGRIN_MEDIATED_SIGNALING_PATHWAY	-1.21	0.58	0	0	0	not significant
3770	GO_REGULATION_OF_MONOCYTE_CHEMOTAXIS	-1.21	0.58	0	0	0	not significant
3771	GO_WASH_COMPLEX	-1.21	0.58	0	0	0	not significant
3772	REACTOME_CAMK_IV_MEDIATED_PHOSPHORYLATION_OF_CREB	-1.21	0.58	0	0	0	not significant
3773	REACTOME_COENZYME_A_BIOSYNTHESIS	-1.21	0.58	0	0	0	not significant
3774	GO_ALDITOL_CATABOLIC_PROCESS	-1.21	0.57	0	0	0	not significant
3775	GO_CLEAVAGE_FURROW_FORMATION	-1.21	0.57	0	0	0	not significant
3776	GO_L_GLUTAMATE_IMPORT	-1.21	0.57	0	0	0	not significant
3777	GO_PORIN_ACTIVITY	-1.21	0.57	0	0	0	not significant
3778	GO_NEGATIVE_REGULATION_OF_RNA_POLYMERASE_II_REGULATORY_F	-1.21	0.56	0	0	0	not significant
3779	GO_CELL_CYCLE_PROCESS	-1.20	4.00	0	0	0	not significant
3780	CHARAFE_BREAST_CANCER_LUMINAL_VS_MESENCHYMAL_DN	-1.20	1.30	0	0	0	not significant
3781	GO_CELL_LEADING_EDGE	-1.20	1.29	0	0	0	not significant
3782	GO_CELL_CYCLE_G1_S_PHASE_TRANSITION	-1.20	1.24	0	0	0	not significant
3783	CHYLA_CBFA2T3_TARGETS_UP	-1.20	1.06	0	0	0	not significant
3784	REACTOME_INTRA_GOLGI_AND_RETROGRADE_GOLGI_TO_ER_TRAFFIC	-1.20	1.05	0	0	0	not significant
3785	GO_DRUG_METABOLIC_PROCESS	-1.20	1.01	0	0	0	not significant
3786	GO_HELICASE_ACTIVITY	-1.20	0.97	0	0	0	not significant
3787	GO_CARDIAC_MUSCLE_TISSUE_DEVELOPMENT	-1.20	0.94	0	0	0	not significant
3788	GO_OSTEOBLAST_DIFFERENTIATION	-1.20	0.89	0	0	0	not significant
3789	P53_DN.V1_UP	-1.20	0.89	0	0	0	not significant
3790	GO_POSITIVE_REGULATION_OF_LIPID_METABOLIC_PROCESS	-1.20	0.88	0	0	0	not significant
3791	BURTON_ADIPOGENESIS_8	-1.20	0.84	0	0	0	not significant
3792	GO_NEGATIVE_REGULATION_OF_CELLULAR_PROTEIN_LOCALIZATION	-1.20	0.84	0	0	0	not significant
3793	CHR2P13	-1.20	0.82	0	0	0	not significant
3794	REACTOME_COPII_MEDIATED_VESICLE_TRANSPORT	-1.20	0.82	0	0	0	not significant
3795	BASSO_CD40_SIGNALING_DN	-1.20	0.81	0	0	0	not significant
3796	GROSS_HYPOXIA_VIA_HIF1A_DN	-1.20	0.81	0	0	0	not significant
3797	HOEBEKE_LYMPHOID_STEM_CELL_DN	-1.20	0.81	0	0	0	not significant
3798	BURTON_ADIPOGENESIS_PEAK_AT_16HR	-1.20	0.78	0	0	0	not significant
3799	GO_DNA_HELICASE_ACTIVITY	-1.20	0.78	0	0	0	not significant
3800	POMEROY_MEDULLOBLASTOMA_DESMOPLASIC_VS_CLASSIC_UP	-1.20	0.77	0	0	0	not significant
3801	GO_ATP_DEPENDENT_DNA_HELICASE_ACTIVITY	-1.20	0.74	0	0	0	not significant
3802	GO_NUCLEOSIDE_TRIPHOSPHATE_BIOSYNTHETIC_PROCESS	-1.20	0.74	0	0	0	not significant
3803	GO_NERVE_DEVELOPMENT	-1.20	0.73	0	0	0	not significant

3804	REACTOME_PEROXISOMAL_PROTEIN_IMPORT	-1.20	0.72	0	0	0	not significant
3805	GO_MUSCLE_CELL_MIGRATION	-1.20	0.71	0	0	0	not significant
3806	GO_TERTIARY_GRANULE_LUMEN	-1.20	0.71	0	0	0	not significant
3807	HOSHIDA_LIVER_CANCER_LATE_RECURRENCE_UP	-1.20	0.71	0	0	0	not significant
3808	BECKER_TAMOXIFEN_RESISTANCE_UP	-1.20	0.70	0	0	0	not significant
3809	GO_BLOOD_MICROPARTICLE	-1.20	0.70	0	0	0	not significant
3810	GO_NEURAL_CREST_CELL_DIFFERENTIATION	-1.20	0.69	0	0	0	not significant
3811	GAZDA_DIAMOND_BLACKFAN_ANEMIA_PROGENITOR_UP	-1.20	0.68	0	0	0	not significant
3812	GO_U12_TYPE_SPLICEOSOMAL_COMPLEX	-1.20	0.68	0	0	0	not significant
3813	BANDRES_RESPONSE_TO_CARMUSTIN_WITHOUT_MGMT_48HR_DN	-1.20	0.67	0	0	0	not significant
3814	GO_CELLULAR_RESPONSE_TO_GAMMA_RADIATION	-1.20	0.67	0	0	0	not significant
3815	GO_ONE_CARBON_METABOLIC_PROCESS	-1.20	0.67	0	0	0	not significant
3816	REACTOME_MITOPHAGY	-1.20	0.67	0	0	0	not significant
3817	GO_CELLULAR_RESPONSE_TO_DSRNA	-1.20	0.66	0	0	0	not significant
3818	REACTOME_MRNA_DECAY_BY_3_TO_5_EXORIBONUCLEASE	-1.20	0.66	0	0	0	not significant
3819	APPIERTO_RESPONSE_TO_FENRETINIDE_UP	-1.20	0.65	0	0	0	not significant
3820	GO_FATTY_ACID_TRANSMEMBRANE_TRANSPORT	-1.20	0.65	0	0	0	not significant
3821	GO_SPLICEOSOMAL_TRI_SNRNP_COMPLEX_ASSEMBLY	-1.20	0.65	0	0	0	not significant
3822	MORI_EMU_MYC_LYMPHOMA_BY_ONSET_TIME_DN	-1.20	0.65	0	0	0	not significant
3823	CHOI_ATL_CHRONIC_VS_ACUTE_DN	-1.20	0.64	0	0	0	not significant
3824	LIAO_HAVE_SOX4_BINDING_SITES	-1.20	0.64	0	0	0	not significant
3825	GO_T_CELL_CYTOKINE_PRODUCTION	-1.20	0.63	0	0	0	not significant
3826	REACTOME_GENERATION_OF_SECOND_MESSENGER_MOLECULES	-1.20	0.63	0	0	0	not significant
3827	REACTOME_REGULATION_OF_FZD_BY_UBIQUITINATION	-1.20	0.63	0	0	0	not significant
3828	DEN_INTERACT_WITH_LCA5	-1.20	0.62	0	0	0	not significant
3829	GO_GOLGI_TO_VACUOLE_TRANSPORT	-1.20	0.62	0	0	0	not significant
3830	GO_LINOLEIC_ACID_METABOLIC_PROCESS	-1.20	0.62	0	0	0	not significant
3831	GO_NEGATIVE_REGULATION_OF_PROTEIN_KINASE_ACTIVITY_BY_REGUI	-1.20	0.62	0	0	0	not significant
3832	GO_PROTEIN_DENEDDYLATION	-1.20	0.62	0	0	0	not significant
3833	HAHTOLA_CTCL_PATHOGENESIS	-1.20	0.62	0	0	0	not significant
3834	GO_TRNA_WOBBLE_URIDINE_MODIFICATION	-1.20	0.61	0	0	0	not significant
3835	GO_VENTRICULAR_CARDIAC_MUSCLE_CELL_MEMBRANE_REPOLARIZAT	-1.20	0.61	0	0	0	not significant
3836	REACTOME_REGULATION_OF_TP53_ACTIVITY_THROUGH_ASSOCIATION_	-1.20	0.61	0	0	0	not significant
3837	GO_POLYAMINE_METABOLIC_PROCESS	-1.20	0.60	0	0	0	not significant
3838	GO_POSITIVE_REGULATION_OF_MITOCHONDRIAL_TRANSLATION	-1.20	0.60	0	0	0	not significant
3839	KEGG_RETINOL_METABOLISM	-1.20	0.60	0	0	0	not significant
3840	ANASTASSIOU_MULTICANCER_INVASIVENESS_SIGNATURE	-1.20	0.59	0	0	0	not significant
3841	GO_HEPATOCYTE_DIFFERENTIATION	-1.20	0.59	0	0	0	not significant
3842	GO_POSITIVE_REGULATION_OF_GONAD_DEVELOPMENT	-1.20	0.59	0	0	0	not significant
3843	GO_SUCCINATE_METABOLIC_PROCESS	-1.20	0.58	0	0	0	not significant
3844	NIELSEN_MALIGNAT_FIBROUS_HISTIOCYTOMA_UP	-1.20	0.58	0	0	0	not significant
3845	REACTOME_INCRETIN_SYNTHESIS_SECRETION_AND_INACTIVATION	-1.20	0.58	0	0	0	not significant
3846	SHIRAISHI_PLZF_TARGETS_UP	-1.20	0.58	0	0	0	not significant
3847	GO_ANGIOTENSIN_ACTIVATED_SIGNALING_PATHWAY	-1.20	0.57	0	0	0	not significant
3848	GO_OXIDOREDUCTASE_ACTIVITY_ACTING_ON_NAD_P_H_OXYGEN_AS_A	-1.20	0.57	0	0	0	not significant
3849	GO_PURINE_RIBONUCLEOSIDE_SALVAGE	-1.20	0.57	0	0	0	not significant
3850	GO_RIBOSOMAL_SMALL_SUBUNIT_EXPORT_FROM_NUCLEUS	-1.20	0.56	0	0	0	not significant
3851	GO_SERINE_TRANSPORT	-1.20	0.56	0	0	0	not significant
3852	CHR2Q13	-1.20	0.55	0	0	0	not significant
3853	GO_NEGATIVE_REGULATION_OF_SKELETAL_MUSCLE_TISSUE_DEVELOF	-1.20	0.55	0	0	0	not significant
3854	GO_POSITIVE_REGULATION_OF_ATTACHMENT_OF_SPINDLE_MICROTUB	-1.20	0.54	0	0	0	not significant
3855	GO_RESPONSE_TO_MUSCLE_INACTIVITY	-1.20	0.54	0	0	0	not significant
3856	GRAESSMANN_APOPTOSIS_BY_DOXORUBICIN_DN	-1.19	4.00	0	0	0	not significant
3857	GO_STRUCTURAL_MOLECULE_ACTIVITY	-1.19	1.45	0	0	0	not significant
3858	GO_REGULATION_OF_CELLULAR_AMIDE_METABOLIC_PROCESS	-1.19	1.33	0	0	0	not significant
3859	MARTINEZ_TP53_TARGETS_UP	-1.19	1.30	0	0	0	not significant
3860	GO_NEGATIVE_REGULATION_OF_ORGANELLE_ORGANIZATION	-1.19	1.25	0	0	0	not significant
3861	GO_CHROMOSOME_SEGREGATION	-1.19	1.16	0	0	0	not significant
3862	GO_NUCLEAR_CHROMOSOME_SEGREGATION	-1.19	1.05	0	0	0	not significant
3863	GO_POSITIVE_REGULATION_OF_LYMPHOCYTE_ACTIVATION	-1.19	1.05	0	0	0	not significant
3864	REACTOME_TRANSPORT_TO_THE_GOLGI_AND_SUBSEQUENT_MODIFIC/	-1.19	0.96	0	0	0	not significant
3865	GO_TELENCEPHALON_DEVELOPMENT	-1.19	0.95	0	0	0	not significant
3866	GO_REGULATION_OF_LEUKOCYTE_PROLIFERATION	-1.19	0.94	0	0	0	not significant
3867	KRAS.DF.V1_UP	-1.19	0.94	0	0	0	not significant
3868	GO_REGULATION_OF_PROTEIN_LOCALIZATION_TO_NUCLEUS	-1.19	0.93	0	0	0	not significant
3869	GO_VIRAL_GENOME_REPLICATION	-1.19	0.89	0	0	0	not significant
3870	ELVIDGE_HYPOXIA_UP	-1.19	0.88	0	0	0	not significant
3871	GO_EPIDERMAL_CELL_DIFFERENTIATION	-1.19	0.88	0	0	0	not significant
3872	GO_POSITIVE_REGULATION_OF_CYSTEINE_TYPE_ENDOPEPTIDASE_ACT	-1.19	0.88	0	0	0	not significant
3873	RODWELL_AGING_KIDNEY_NO_BLOOD_DN	-1.19	0.88	0	0	0	not significant
3874	GO_TRANSLATION_REGULATOR_ACTIVITY_NUCLEIC_ACID_BINDING	-1.19	0.85	0	0	0	not significant
3875	GALE_APL_WITH_FLT3_MUTATED_UP	-1.19	0.80	0	0	0	not significant
3876	GO_NUCLEOSIDE_PHOSPHATE_CATABOLIC_PROCESS	-1.19	0.80	0	0	0	not significant
3877	GO_REGULATION_OF_LIPID_LOCALIZATION	-1.19	0.80	0	0	0	not significant
3878	GO_NEGATIVE_REGULATION_OF_NUCLEAR_DIVISION	-1.19	0.78	0	0	0	not significant
3879	HADDAD_T_LYMPHOCYTE_AND_NK_PROGENITOR_UP	-1.19	0.75	0	0	0	not significant
3880	GO_NECROTIC_CELL_DEATH	-1.19	0.74	0	0	0	not significant
3881	GO_POSITIVE_REGULATION_OF_ACTIN_FILAMENT_BUNDLE_ASSEMBLY	-1.19	0.74	0	0	0	not significant
3882	GO_POSITIVE_REGULATION_OF_DNA_BINDING	-1.19	0.74	0	0	0	not significant
3883	CHR11Q23	-1.19	0.73	0	0	0	not significant
3884	GO_WATER_SOLUBLE_VITAMIN_METABOLIC_PROCESS	-1.19	0.72	0	0	0	not significant
3885	THEILGAARD_NEUTROPHIL_AT_SKIN_WOUND_UP	-1.19	0.72	0	0	0	not significant
3886	LINDVALL_IMMORTALIZED_BY_TERT_UP	-1.19	0.71	0	0	0	not significant
3887	GO_REGULATION_OF_LIPID_TRANSPORT	-1.19	0.70	0	0	0	not significant
3888	GO_TRANSLATION_INITIATION_FACTOR_ACTIVITY	-1.19	0.70	0	0	0	not significant
3889	PID_ERBB1_INTERNALIZATION_PATHWAY	-1.19	0.70	0	0	0	not significant
3890	GO_CELLULAR_AMINO_ACID_CATABOLIC_PROCESS	-1.19	0.69	0	0	0	not significant
3891	GO_NADP_BINDING	-1.19	0.69	0	0	0	not significant
3892	HALLMARK_NOTCH_SIGNALING	-1.19	0.69	0	0	0	not significant
3893	ASTON_MAJOR_DEPRESSIVE_DISORDER_UP	-1.19	0.68	0	0	0	not significant
3894	GO_CELLULAR_MODIFIED_AMINO_ACID_BIOSYNTHETIC_PROCESS	-1.19	0.68	0	0	0	not significant
3895	GO_EXTRINSIC_COMPONENT_OF_ORGANELLE_MEMBRANE	-1.19	0.68	0	0	0	not significant
3896	GO_NEGATIVE_REGULATION_OF_METAPHASE_ANAPHASE_TRANSITION_	-1.19	0.68	0	0	0	not significant
3897	GO_RESPONSE_TO_COLD	-1.19	0.68	0	0	0	not significant
3898	BIOCARTA_FCER1_PATHWAY	-1.19	0.67	0	0	0	not significant
3899	ZHANG_GATA6_TARGETS_DN	-1.19	0.67	0	0	0	not significant
3900	BROWNE_HCMV_INFECTION_4HR_UP	-1.19	0.66	0	0	0	not significant
3901	GO_NUCLEOBASE_CONTAINING_SMALL_MOLECULE_INTERCONVERSION	-1.19	0.66	0	0	0	not significant
3902	GO_POSITIVE_REGULATION_OF_CALCIUM_MEDIATED_SIGNALING	-1.19	0.66	0	0	0	not significant
3903	GO_REGULATION_OF_TRANSLATIONAL_FIDELITY	-1.19	0.66	0	0	0	not significant
3904	MCCLUNG_DELTA_FOSB_TARGETS_8WK	-1.19	0.65	0	0	0	not significant
3905	CHR5Q12	-1.19	0.64	0	0	0	not significant
3906	DORN_ADENOVIRUS_INFECTION_32HR_DN	-1.19	0.64	0	0	0	not significant
3907	GO_RESPONSE_TO_X_RAY	-1.19	0.64	0	0	0	not significant
3908	PID_LIS1_PATHWAY	-1.19	0.64	0	0	0	not significant
3909	GO_EMBRYONIC_EYE_MORPHOGENESIS	-1.19	0.63	0	0	0	not significant
3910	GO_MHC_CLASS_II_PROTEIN_COMPLEX_BINDING	-1.19	0.63	0	0	0	not significant
3911	GO_NEGATIVE_REGULATION_OF_NUCLEOCYTOPLASMIC_TRANSPORT	-1.19	0.63	0	0	0	not significant
3912	GRADE_COLON_CANCER_DN	-1.19	0.63	0	0	0	not significant

3913	GO_ASYMMETRIC_CELL_DIVISION	-1.19	0.62	0	0	0	not significant
3914	GO_MONOCARBOXYLIC_ACID_TRANSMEMBRANE_TRANSPORTER_ACTIV	-1.19	0.62	0	0	0	not significant
3915	LI_WILMS_TUMOR_VS_FETAL_KIDNEY_2_DN	-1.19	0.62	0	0	0	not significant
3916	GO_DYNEIN_INTERMEDIATE_CHAIN_BINDING	-1.19	0.61	0	0	0	not significant
3917	GO_FATTY_ACID_DERIVATIVE_CATABOLIC_PROCESS	-1.19	0.61	0	0	0	not significant
3918	GO_REGULATION_OF_CELL_MATURATION	-1.19	0.61	0	0	0	not significant
3919	REACTOME_SYNTHESIS_SECRETION_AND_INACTIVATION_OF_GLUCOSE_	-1.19	0.61	0	0	0	not significant
3920	WENG_POR_TARGETS_GLOBAL_DN	-1.19	0.60	0	0	0	not significant
3921	GO_POSITIVE_REGULATION_OF_ENDOPLASMIC_RETICULUM_UNFOLDED	-1.19	0.59	0	0	0	not significant
3922	GO_SNORNA_PROCESSING	-1.19	0.59	0	0	0	not significant
3923	GO_G_QUADRUPLEX_DNA_BINDING	-1.19	0.58	0	0	0	not significant
3924	GO_JUN_KINASE_KINASE_KINASE_ACTIVITY	-1.19	0.58	0	0	0	not significant
3925	GO_NUCLEAR_CYCLIN_DEPENDENT_PROTEIN_KINASE_HOLOENZYME_CO	-1.19	0.58	0	0	0	not significant
3926	GO_RESPONSE_TO_MANGANESE_ION	-1.19	0.58	0	0	0	not significant
3927	GO_TRANSLOCATION_OF_MOLECULES_INTO_HOST	-1.19	0.58	0	0	0	not significant
3928	GOLUB_ALL_VS_AML_DN	-1.19	0.58	0	0	0	not significant
3929	KEGG_STEROID_HORMONE_BIOSYNTHESIS	-1.19	0.58	0	0	0	not significant
3930	MIKKELSEN_IPS_WITH_HCP_H3K27ME3	-1.19	0.58	0	0	0	not significant
3931	GO_EXTRINSIC_COMPONENT_OF_ENDOSOME_MEMBRANE	-1.19	0.57	0	0	0	not significant
3932	GO_KINETOCHORE_ASSEMBLY	-1.19	0.57	0	0	0	not significant
3933	GO_STEROL_IMPORT	-1.19	0.57	0	0	0	not significant
3934	GO_STRESS_INDUCED_PREMATURE_SENESCENCE	-1.19	0.57	0	0	0	not significant
3935	GO_UTERUS_DEVELOPMENT	-1.19	0.57	0	0	0	not significant
3936	REACTOME_ROBO_RECEPTORS_BIND_AKAP5	-1.19	0.57	0	0	0	not significant
3937	REACTOME_SYNTHESIS_OF_BILE_ACIDS_AND_BILE_SALTS_VIA_27_HYD	-1.19	0.57	0	0	0	not significant
3938	GO_SECRETION_OF_LYSOSOMAL_ENZYMES	-1.19	0.56	0	0	0	not significant
3939	KUWANO_RNA_STABILIZED_BY_NO	-1.19	0.56	0	0	0	not significant
3940	DELASERNA_TARGETS_OF_MYOD_AND_SMARCA4	-1.19	0.55	0	0	0	not significant
3941	GO_ESTABLISHMENT_OF_CENTROSOME_LOCALIZATION	-1.19	0.55	0	0	0	not significant
3942	GO_POSITIVE_REGULATION_OF_RECEPTOR_BIOSYNTHETIC_PROCESS	-1.19	0.55	0	0	0	not significant
3943	GO_SEX_CHROMATIN	-1.19	0.55	0	0	0	not significant
3944	GO_SIGNAL_PEPTIDASE_COMPLEX	-1.19	0.55	0	0	0	not significant
3945	GO_SUCCINYL_COA_METABOLIC_PROCESS	-1.19	0.55	0	0	0	not significant
3946	GO_EARLY_ENDOSOME_TO_GOLGI_TRANSPORT	-1.19	0.54	0	0	0	not significant
3947	GO_NEGATIVE_REGULATION_OF_PEPTIDYL_CYSTEINE_S_NITROSYLATIO	-1.19	0.54	0	0	0	not significant
3948	GO_PERICHROMATIN_FIBRILS	-1.19	0.54	0	0	0	not significant
3949	GO_POSITIVE_REGULATION_OF_NEURON_PROJECTION_REGENERATION	-1.19	0.54	0	0	0	not significant
3950	GO_REGULATION_OF_DEVELOPMENT_HETEROCHRONIC	-1.19	0.54	0	0	0	not significant
3951	GO_REGULATION_OF_NEUROMUSCULAR_JUNCTION_DEVELOPMENT	-1.19	0.54	0	0	0	not significant
3952	GO_REGULATION_OF_WNT_SIGNALING_PATHWAY_PLANAR_CELL_POLA	-1.19	0.54	0	0	0	not significant
3953	GO_U1_SNRNP_BINDING	-1.19	0.54	0	0	0	not significant
3954	REACTOME_TYSND1_CLEAVES_PEROXISOMAL_PROTEINS	-1.19	0.53	0	0	0	not significant
3955	GO_MECHANOSENSORY_BEHAVIOR	-1.19	0.52	0	0	0	not significant
3956	GO_FEMALE_MEIOSIS_CHROMOSOME_SEGREGATION	-1.19	0.51	0	0	0	not significant
3957	GO_POSITIVE_REGULATION_OF_ER_ASSOCIATED_UBIQUITIN_DEPENDEN	-1.19	0.51	0	0	0	not significant
3958	GO_SECRETORY_GRANULE	-1.18	1.96	0	0	0	not significant
3959	GO_REGULATION_OF_CELL_DEATH	-1.18	1.91	0	0	0	not significant
3960	PUJANA_ATM_PCC_NETWORK	-1.18	1.82	0	0	0	not significant
3961	GO_SECRETORY_VESICLE	-1.18	1.68	0	0	0	not significant
3962	BENPORATH_CYCLING_GENES	-1.18	1.59	0	0	0	not significant
3963	GO_CARDIOVASCULAR_SYSTEM_DEVELOPMENT	-1.18	1.46	0	0	0	not significant
3964	GO_SIGNAL_TRANSDUCTION_BY_PROTEIN_PHOSPHORYLATION	-1.18	1.36	0	0	0	not significant
3965	GO_IMPORT_INTO_CELL	-1.18	1.34	0	0	0	not significant
3966	GO_CELLULAR_CHEMICAL_HOMEOSTASIS	-1.18	1.29	0	0	0	not significant
3967	GO_NUCLEAR_ENVELOPE	-1.18	1.29	0	0	0	not significant
3968	GO_REGULATION_OF_DEFENSE_RESPONSE	-1.18	1.25	0	0	0	not significant
3969	GO_MULTICELLULAR_ORGANISMAL_HOMEOSTASIS	-1.18	1.20	0	0	0	not significant
3970	GO_RESPONSE_TO_PEPTIDE	-1.18	1.18	0	0	0	not significant
3971	GO_EPITHELIAL_CELL_DIFFERENTIATION	-1.18	1.17	0	0	0	not significant
3972	REACTOME_METABOLISM_OF_AMINO_ACIDS_AND_DERIVATIVES	-1.18	1.15	0	0	0	not significant
3973	GO_MONOVALENT_INORGANIC_CATION_TRANSPORT	-1.18	1.07	0	0	0	not significant
3974	REACTOME_INTRACELLULAR_SIGNALING_BY_SECOND_MESSENGERS	-1.18	1.07	0	0	0	not significant
3975	GO_PROTEIN_LOCALIZATION_TO_NUCLEUS	-1.18	1.04	0	0	0	not significant
3976	GO_SECOND_MESSENGER_MEDIATED_SIGNALING	-1.18	0.98	0	0	0	not significant
3977	GO_NEGATIVE_REGULATION_OF_CELL_MOTILITY	-1.18	0.97	0	0	0	not significant
3978	HOXA9_DN.V1_DN	-1.18	0.97	0	0	0	not significant
3979	GO_DNA_BIOSYNTHETIC_PROCESS	-1.18	0.96	0	0	0	not significant
3980	GO_NEGATIVE_REGULATION_OF_CHROMOSOME_ORGANIZATION	-1.18	0.95	0	0	0	not significant
3981	SANSOM_APC_TARGETS	-1.18	0.92	0	0	0	not significant
3982	GO_REGULATION_OF_PROTEIN_BINDING	-1.18	0.91	0	0	0	not significant
3983	GO_REGULATION_OF_CYTOSOLIC_CALCIUM_ION_CONCENTRATION	-1.18	0.89	0	0	0	not significant
3984	GO_ADAPTIVE_THERMOGENESIS	-1.18	0.88	0	0	0	not significant
3985	GO_TEMPERATURE_HOMEOSTASIS	-1.18	0.87	0	0	0	not significant
3986	CHR14Q24	-1.18	0.85	0	0	0	not significant
3987	GO_ESTABLISHMENT_OF_CELL_POLARITY	-1.18	0.83	0	0	0	not significant
3988	ROESSLER_LIVER_CANCER_METASTASIS_UP	-1.18	0.83	0	0	0	not significant
3989	GO_ENTRY_INTO_OTHER_ORGANISM_INVOLVED_IN_SYMBIOTIC_INTERA(	-1.18	0.81	0	0	0	not significant
3990	GO_MICROTUBULE_MOTOR_ACTIVITY	-1.18	0.80	0	0	0	not significant
3991	GO_SPINDLE_ASSEMBLY	-1.18	0.78	0	0	0	not significant
3992	PICCALUGA_ANGIOIMMUNOBLASTIC_LYMPHOMA_UP	-1.18	0.76	0	0	0	not significant
3993	GO_CHAPERONE_BINDING	-1.18	0.75	0	0	0	not significant
3994	GO_KIDNEY_EPITHELIUM_DEVELOPMENT	-1.18	0.75	0	0	0	not significant
3995	REACTOME_RECRUITMENT_OF_NUMA_TO_MITOTIC_CENTROSOMES	-1.18	0.75	0	0	0	not significant
3996	CARD_MIR302A_TARGETS	-1.18	0.74	0	0	0	not significant
3997	REACTOME_NUCLEOSOME_ASSEMBLY	-1.18	0.74	0	0	0	not significant
3998	RASHI_RESPONSE_TO_IONIZING_RADIATION_6	-1.18	0.73	0	0	0	not significant
3999	BRUNO_HEMATOPOIESIS	-1.18	0.71	0	0	0	not significant
4000	DOANE_BREAST_CANCER_ESR1_UP	-1.18	0.70	0	0	0	not significant
4001	GO_ALCOHOL_BINDING	-1.18	0.70	0	0	0	not significant
4002	GO_RESPONSE_TO_NERVE_GROWTH_FACTOR	-1.18	0.69	0	0	0	not significant
4003	GO_DICARBOXYLIC_ACID_TRANSPORT	-1.18	0.68	0	0	0	not significant
4004	NADLER_OBESITY_UP	-1.18	0.68	0	0	0	not significant
4005	YORDY_RECIPROCAL_REGULATION_BY_ETS1_AND_SP100_DN	-1.18	0.68	0	0	0	not significant
4006	LANDIS_BREAST_CANCER_PROGRESSION_UP	-1.18	0.66	0	0	0	not significant
4007	LEE_CALORIE_RESTRICTION_MUSCLE_UP	-1.18	0.65	0	0	0	not significant
4008	PID_AR_NONGENOMIC_PATHWAY	-1.18	0.65	0	0	0	not significant
4009	REACTOME_DAP12_INTERACTIONS	-1.18	0.65	0	0	0	not significant
4010	TERAO_AOX4_TARGETS_HG_UP	-1.18	0.65	0	0	0	not significant
4011	GO_NEGATIVE_REGULATION_OF_REPRODUCTIVE_PROCESS	-1.18	0.64	0	0	0	not significant
4012	GO_OLIGOSACCHARIDE_LIPID_INTERMEDIATE_BIOSYNTHETIC_PROCESS	-1.18	0.64	0	0	0	not significant
4013	GO_VIRAL_RNA_GENOME_REPLICATION	-1.18	0.64	0	0	0	not significant
4014	SEITZ_NEOPLASTIC_TRANSFORMATION_BY_8P_DELETION_DN	-1.18	0.64	0	0	0	not significant
4015	GO_MAMMARY_GLAND_MORPHOGENESIS	-1.18	0.63	0	0	0	not significant
4016	GO_PROTEIN_DISULFIDE_OXIDOREDUCTASE_ACTIVITY	-1.18	0.63	0	0	0	not significant
4017	GO_REGULATION_OF_ACTIN_CYTOSKELETON_REORGANIZATION	-1.18	0.63	0	0	0	not significant
4018	GO_REGULATION_OF_CYCLASE_ACTIVITY	-1.18	0.63	0	0	0	not significant
4019	CHR6Q16	-1.18	0.62	0	0	0	not significant
4020	GAUSSMANN_MLL_AF4_FUSION_TARGETS_F_DN	-1.18	0.62	0	0	0	not significant
4021	SA_CASPASE_CASCADE	-1.18	0.62	0	0	0	not significant

4022	GO_NUCLEAR_PORE_ORGANIZATION	-1.18	0.61	0	0	0	not significant
4023	GO_POSITIVE_REGULATION_OF_INTERFERON_BETA_PRODUCTION	-1.18	0.61	0	0	0	not significant
4024	REACTOME_PENTOSE_PHOSPHATE_PATHWAY	-1.18	0.61	0	0	0	not significant
4025	CHEOK_RESPONSE_TO_HD_MTX_UP	-1.18	0.60	0	0	0	not significant
4026	GO_NUCLEAR_BODY_ORGANIZATION	-1.18	0.60	0	0	0	not significant
4027	WATANABE_ULCERATIVE_COLITIS_WITH_CANCER_DN	-1.18	0.60	0	0	0	not significant
4028	GO_CLATHRIN_COAT_OF_ENDOCYTIC_VESICLE	-1.18	0.59	0	0	0	not significant
4029	REACTOME_REGULATION_OF_LOCALIZATION_OF_FOXO_TRANSCRIPTIC	-1.18	0.59	0	0	0	not significant
4030	SILIGAN_TARGETS_OF_EWS_FLI1_FUSION_UP	-1.18	0.59	0	0	0	not significant
4031	GO_HOST_CELL_PART	-1.18	0.58	0	0	0	not significant
4032	GO_REGULATION_OF_ADENYLATE_CYCLASE_ACTIVITY	-1.18	0.58	0	0	0	not significant
4033	KYNG_ENVIRONMENTAL_STRESS_RESPONSE_DN	-1.18	0.58	0	0	0	not significant
4034	SA_G2_AND_M_PHASES	-1.18	0.58	0	0	0	not significant
4035	GO_ACTIVATION_OF_PROTEIN_KINASE_A_ACTIVITY	-1.18	0.57	0	0	0	not significant
4036	GO_MOTILE_CILIUM_ASSEMBLY	-1.18	0.57	0	0	0	not significant
4037	GO_ALCOHOL_DEHYDROGENASE_NADPPLUS_ACTIVITY	-1.18	0.56	0	0	0	not significant
4038	GO_CELLULAR_RESPONSE_TO_EXOGENOUS_DSRNA	-1.18	0.56	0	0	0	not significant
4039	GO_HOST_INTRACELLULAR_PART	-1.18	0.56	0	0	0	not significant
4040	GO_NEGATIVE_REGULATION_OF_MYOBLAST_DIFFERENTIATION	-1.18	0.56	0	0	0	not significant
4041	GO_NITRIC_OXIDE_SYNTHASE_BIOSYNTHETIC_PROCESS	-1.18	0.56	0	0	0	not significant
4042	GO_POSITIVE_REGULATION_OF_AMYLOID_BETA_FORMATION	-1.18	0.56	0	0	0	not significant
4043	GO_TPR_DOMAIN_BINDING	-1.18	0.56	0	0	0	not significant
4044	GO_TRACHEA_MORPHOGENESIS	-1.18	0.56	0	0	0	not significant
4045	GO_TROPOMYOSIN_BINDING	-1.18	0.56	0	0	0	not significant
4046	GO_POSITIVE_REGULATION_OF_MACROPHAGE_DIFFERENTIATION	-1.18	0.55	0	0	0	not significant
4047	REACTOME_CONDENSATION_OF_PROMETAPHASE_CHROMOSOMES	-1.18	0.55	0	0	0	not significant
4048	BIOCARTA_TSP1_PATHWAY	-1.18	0.54	0	0	0	not significant
4049	GO_NERVE_GROWTH_FACTOR_SIGNALING_PATHWAY	-1.18	0.54	0	0	0	not significant
4050	LOPEZ_MESOTHELIOMA_SURVIVAL_WORST_VS_BEST_UP	-1.18	0.54	0	0	0	not significant
4051	REACTOME_DECTIN_2_FAMILY	-1.18	0.54	0	0	0	not significant
4052	GO_EUKARYOTIC_INITIATION_FACTOR_4E_BINDING	-1.18	0.53	0	0	0	not significant
4053	GO_REGULATION_OF_MACROPHAGE_DIFFERENTIATION	-1.18	0.53	0	0	0	not significant
4054	GO_NEGATIVE_REGULATION_OF_FIBROBLAST_APOPTOTIC_PROCESS	-1.18	0.51	0	0	0	not significant
4055	GO_REGULATION_OF_VITAMIN_D_RECEPTOR_SIGNALING_PATHWAY	-1.18	0.51	0	0	0	not significant
4056	GO_HEART_FIELD_SPECIFICATION	-1.18	0.50	0	0	0	not significant
4057	GO_HYDROLASE_ACTIVITY_ACTING_ON_ESTER_BONDS	-1.17	2.21	0	0	0	not significant
4058	GO_POSITIVE_REGULATION_OF_CATALYTIC_ACTIVITY	-1.17	1.97	0	0	0	not significant
4059	REACTOME_POST_TRANSLATIONAL_PROTEIN_MODIFICATION	-1.17	1.92	0	0	0	not significant
4060	GO_REGULATION_OF_HYDROLASE_ACTIVITY	-1.17	1.76	0	0	0	not significant
4061	GO_APOPTOTIC_PROCESS	-1.17	1.74	0	0	0	not significant
4062	MARTINEZ_RB1_AND_TP53_TARGETS_UP	-1.17	1.50	0	0	0	not significant
4063	SENESE_HDAC3_TARGETS_UP	-1.17	1.15	0	0	0	not significant
4064	GO_CELL_BODY	-1.17	1.11	0	0	0	not significant
4065	DUTERTRE_ESTRADIOL_RESPONSE_24HR_UP	-1.17	1.03	0	0	0	not significant
4066	HALLMARK_INTERFERON_GAMMA_RESPONSE	-1.17	0.98	0	0	0	not significant
4067	GO_CANONICAL_WNT_SIGNALING_PATHWAY	-1.17	0.94	0	0	0	not significant
4068	KEGG_UBIQUITIN_MEDIATED_PROTEOLYSIS	-1.17	0.93	0	0	0	not significant
4069	AMUNDSON_POOR_SURVIVAL_AFTER_GAMMA_RADIATION_2G	-1.17	0.92	0	0	0	not significant
4070	FLORIO_NEOCORTEX_BASAL_RADIAL_GLIA_UP	-1.17	0.91	0	0	0	not significant
4071	JIANG_HYPOXIA_NORMAL	-1.17	0.88	0	0	0	not significant
4072	IWANAGA_CARCINOGENESIS_BY_KRAS_UP	-1.17	0.87	0	0	0	not significant
4073	FERNANDEZ_BOUND_BY_MYC	-1.17	0.83	0	0	0	not significant
4074	GO_T_CELL_MEDIATED_IMMUNITY	-1.17	0.80	0	0	0	not significant
4075	GO_POSITIVE_REGULATION_OF_EPITHELIAL_CELL_MIGRATION	-1.17	0.77	0	0	0	not significant
4076	XU_GH1_AUTOCRINE_TARGETS_UP	-1.17	0.76	0	0	0	not significant
4077	GO_GLUTAMINE_FAMILY_AMINO_ACID_METABOLIC_PROCESS	-1.17	0.74	0	0	0	not significant
4078	KEGG_RNA_DEGRADATION	-1.17	0.74	0	0	0	not significant
4079	GO_LEUKOCYTE_HOMEOSTASIS	-1.17	0.73	0	0	0	not significant
4080	GO_NEGATIVE_REGULATION_OF_PROTEIN_COMPLEX_DISASSEMBLY	-1.17	0.73	0	0	0	not significant
4081	GO_POSITIVE_REGULATION_OF_SMALL_MOLECULE_METABOLIC_PROCI	-1.17	0.73	0	0	0	not significant
4082	GO_POSITIVE_REGULATION_OF_PROTEIN_LOCALIZATION_TO_CELL_PEI	-1.17	0.71	0	0	0	not significant
4083	STARK_HYPPOCAMPUS_22Q11_DELETION_UP	-1.17	0.69	0	0	0	not significant
4084	ROVERSI_GLIOMA_COPY_NUMBER_DN	-1.17	0.68	0	0	0	not significant
4085	GO_PEROXISOMAL_TRANSPORT	-1.17	0.67	0	0	0	not significant
4086	GO_POLYOL_METABOLIC_PROCESS	-1.17	0.67	0	0	0	not significant
4087	GO_POSITIVE_REGULATION_OF_G1_S_TRANSITION_OF_MITOTIC_CELL_	-1.17	0.67	0	0	0	not significant
4088	GO_REGULATION_OF_RELEASE_OF_CYTOCHROME_C_FROM_MITOCHO	-1.17	0.67	0	0	0	not significant
4089	BOYAULT_LIVER_CANCER_SUBCLASS_G3_DN	-1.17	0.66	0	0	0	not significant
4090	GO_REGULATION_OF_SPROUTING_ANGIOGENESIS	-1.17	0.66	0	0	0	not significant
4091	JAERVINEN_AMPLIFIED_IN_LARYNGEAL_CANCER	-1.17	0.66	0	0	0	not significant
4092	GO_9PLUS2_MOTILE_CILIUM	-1.17	0.65	0	0	0	not significant
4093	GO_INTERLEUKIN_12_PRODUCTION	-1.17	0.64	0	0	0	not significant
4094	MA_MYELOID_DIFFERENTIATION_DN	-1.17	0.64	0	0	0	not significant
4095	GO_REGULATION_OF_LEUKOCYTE_DEGRANULATION	-1.17	0.63	0	0	0	not significant
4096	MOREIRA_RESPONSE_TO_TSA_UP	-1.17	0.63	0	0	0	not significant
4097	NEMETH_INFLAMMATORY_RESPONSE_LPS_DN	-1.17	0.63	0	0	0	not significant
4098	REACTOME_INTERCONVERSION_OF_NUCLEOTIDE_DI_AND_TRIPHOSPHA	-1.17	0.62	0	0	0	not significant
4099	GO_NEGATIVE_REGULATION_OF_CALCIUM_ION_TRANSPORT	-1.17	0.61	0	0	0	not significant
4100	GO_POSITIVE_REGULATION_OF_INTERLEUKIN_12_PRODUCTION	-1.17	0.61	0	0	0	not significant
4101	GO_NUCLEAR_EXOSOME_RNASE_COMPLEX_	-1.17	0.60	0	0	0	not significant
4102	NAKAMURA_METASTASIS_MODEL_DN	-1.17	0.60	0	0	0	not significant
4103	GO_ACETYL_COA_BIOSYNTHETIC_PROCESS	-1.17	0.59	0	0	0	not significant
4104	GO_CHONDROCYTE_DEVELOPMENT	-1.17	0.59	0	0	0	not significant
4105	GO_LOW_DENSITY_LIPOPROTEIN_PARTICLE_RECEPTOR_BINDING	-1.17	0.59	0	0	0	not significant
4106	GO_MAP_KINASE_KINASE_ACTIVITY	-1.17	0.59	0	0	0	not significant
4107	GO_NUCLEAR_TRANSCRIBED_MRNA_CATABOLIC_PROCESS_EXONUCLE(	-1.17	0.59	0	0	0	not significant
4108	GO_PHAGOCYTIC_CUP	-1.17	0.59	0	0	0	not significant
4109	GO_POSITIVE_REGULATION_OF_TRANSCRIPTION_FROM_RNA_POLYMEF	-1.17	0.59	0	0	0	not significant
4110	GO_PROTEIN_DESTABILIZATION	-1.17	0.59	0	0	0	not significant
4111	GO_CELLULAR_MODIFIED_AMINO_ACID_CATABOLIC_PROCESS	-1.17	0.58	0	0	0	not significant
4112	GO_REGULATION_OF_LYMPHOCYTE_CHEMOTAXIS	-1.17	0.58	0	0	0	not significant
4113	GROSS_ELK3_TARGETS_DN	-1.17	0.58	0	0	0	not significant
4114	GO_CELLULAR_RESPONSE_TO_LEUCINE_STARVATION	-1.17	0.56	0	0	0	not significant
4115	GO_FATTY_ACID_BETA_OXIDATION_USING_ACYL_COA_OXIDASE	-1.17	0.55	0	0	0	not significant
4116	GO_KERATINOCYTE_MIGRATION	-1.17	0.55	0	0	0	not significant
4117	GO_NEGATIVE_REGULATION_OF_RYANODINE_SENSITIVE_CALCIUM_REL	-1.17	0.55	0	0	0	not significant
4118	GO_POSITIVE_REGULATION_OF_T_HELPER_CELL_DIFFERENTIATION	-1.17	0.55	0	0	0	not significant
4119	GO_SPERM_PRINCIPAL_PIECE	-1.17	0.55	0	0	0	not significant
4120	GO_VITAMIN_D_RECEPTOR_SIGNALING_PATHWAY	-1.17	0.55	0	0	0	not significant
4121	GO_ACETYLCHOLINE_GATED_CATION_SELECTIVE_CHANNEL_ACTIVITY	-1.17	0.54	0	0	0	not significant
4122	REACTOME_ABC_TRANSPORTERS_IN_LIPID_HOMEOSTASIS	-1.17	0.54	0	0	0	not significant
4123	REACTOME_GP1B_IX_V_ACTIVATION_SIGNALLING	-1.17	0.54	0	0	0	not significant
4124	GO_NEGATIVE_REGULATION_OF_METANEPHROS_DEVELOPMENT	-1.17	0.53	0	0	0	not significant
4125	GO_REGULATION_OF_WOUND_HEALING_SPREADING_OF_EPIDERMAL_C	-1.17	0.53	0	0	0	not significant
4126	GO_SYNAPTIC_TRANSMISSION_DOPAMINERGIC	-1.17	0.53	0	0	0	not significant
4127	GO_TETRAHYDROBIOPTERIN_BIOSYNTHETIC_PROCESS	-1.17	0.53	0	0	0	not significant
4128	REACTOME_ACETYLCHOLINE_BINDING_AND_DOWNSTREAM_EVENTS	-1.17	0.53	0	0	0	not significant
4129	CHR5Q34	-1.17	0.52	0	0	0	not significant
4130	GO_KETONE_BODY_BIOSYNTHETIC_PROCESS	-1.17	0.52	0	0	0	not significant

4131	GO_MODULATION_OF_MOLECULAR_FUNCTION_IN_OTHER_ORGANISM	-1.17	0.52	0	0	0	not significant
4132	GO_SERINE_FAMILY_AMINO_ACID_CATABOLIC_PROCESS	-1.17	0.52	0	0	0	not significant
4133	GO_PRE_MIRNA_BINDING	-1.17	0.51	0	0	0	not significant
4134	GO_SECONDARY_HEART_FIELD_SPECIFICATION	-1.17	0.51	0	0	0	not significant
4135	GO_SERINE_TYPE_EXOPEPTIDASE_ACTIVITY	-1.17	0.51	0	0	0	not significant
4136	GO_NEGATIVE_REGULATION_OF_PROTEIN_LOCALIZATION_TO_CELL_SU	-1.17	0.50	0	0	0	not significant
4137	GO_POSITIVE_REGULATION_OF_MOLECULAR_FUNCTION	-1.16	1.96	0	0	0	not significant
4138	GO_INTERSPECIES_INTERACTION_BETWEEN_ORGANISMS	-1.16	1.75	0	0	0	not significant
4139	GO_REGULATION_OF_RESPONSE_TO_STRESS	-1.16	1.71	0	0	0	not significant
4140	GO_CELL_ACTIVATION	-1.16	1.69	0	0	0	not significant
4141	GO_GROWTH	-1.16	1.52	0	0	0	not significant
4142	GO_POSITIVE_REGULATION_OF_MULTICELLULAR_ORGANISMAL_PROCE	-1.16	1.47	0	0	0	not significant
4143	GO_POST_TRANSLATIONAL_PROTEIN_MODIFICATION	-1.16	1.17	0	0	0	not significant
4144	GO_REGULATION_OF_CYTOSKELETON_ORGANIZATION	-1.16	1.15	0	0	0	not significant
4145	LIM_MAMMARY_STEM_CELL_UP	-1.16	0.99	0	0	0	not significant
4146	GO_COGNITION	-1.16	0.92	0	0	0	not significant
4147	GO_ORGANIC_ACID_CATABOLIC_PROCESS	-1.16	0.88	0	0	0	not significant
4148	WANG_TUMOR_INVASIVENESS_DN	-1.16	0.88	0	0	0	not significant
4149	GO_PROTEIN_STABILIZATION	-1.16	0.87	0	0	0	not significant
4150	GO_RESPONSE_TO_VIRUS	-1.16	0.87	0	0	0	not significant
4151	REACTOME_TOLL_LIKE_RECEPTOR_CASCADES	-1.16	0.84	0	0	0	not significant
4152	GO_REGULATION_OF_PROTEIN_LOCALIZATION_TO_MEMBRANE	-1.16	0.83	0	0	0	not significant
4153	GO_NUCLEOTIDE_EXCISION_REPAIR	-1.16	0.79	0	0	0	not significant
4154	KEGG_CELL_CYCLE	-1.16	0.79	0	0	0	not significant
4155	MIYAGAWA_TARGETS_OF_EWSR1_ETS_FUSIONS_DN	-1.16	0.79	0	0	0	not significant
4156	GO_CHROMATIN_DNA_BINDING	-1.16	0.78	0	0	0	not significant
4157	GO_RIBONUCLEASE_ACTIVITY	-1.16	0.78	0	0	0	not significant
4158	REACTOME_CHROMOSOME_MAINTENANCE	-1.16	0.77	0	0	0	not significant
4159	REACTOME_SENESCENCE_ASSOCIATED_SECRETORY_PHENOTYPE_SAS	-1.16	0.77	0	0	0	not significant
4160	GO_CARBOHYDRATE_TRANSMEMBRANE_TRANSPORT	-1.16	0.76	0	0	0	not significant
4161	GO_IMPORT_INTO_NUCLEUS	-1.16	0.76	0	0	0	not significant
4162	NGUYEN_NOTCH1_TARGETS_DN	-1.16	0.76	0	0	0	not significant
4163	BMI1_DN_MEL18_DN.V1_UP	-1.16	0.75	0	0	0	not significant
4164	GO_AMINO_ACID_TRANSPORT	-1.16	0.75	0	0	0	not significant
4165	GO_REGULATION_OF_MRNA_PROCESSING	-1.16	0.75	0	0	0	not significant
4166	GO_NUCLEOBASE_CONTAINING_SMALL_MOLECULE_BIOSYNTHETIC_PRC	-1.16	0.74	0	0	0	not significant
4167	GO_TRANSLATION_FACTOR_ACTIVITY_RNA_BINDING	-1.16	0.74	0	0	0	not significant
4168	GROSS_HYPOXIA_VIA_ELK3_DN	-1.16	0.73	0	0	0	not significant
4169	DE_YY1_TARGETS_DN	-1.16	0.72	0	0	0	not significant
4170	GO_N_METHYLTRANSFERASE_ACTIVITY	-1.16	0.71	0	0	0	not significant
4171	GO_CARDIAC_CHAMBER_MORPHOGENESIS	-1.16	0.69	0	0	0	not significant
4172	GO_EPHRIN_RECEPTOR_SIGNALING_PATHWAY	-1.16	0.69	0	0	0	not significant
4173	KAYO_CALORIE_RESTRICTION_MUSCLE_DN	-1.16	0.69	0	0	0	not significant
4174	ZHANG_PROLIFERATING_VS_QUIESCENT	-1.16	0.69	0	0	0	not significant
4175	BMI1_DN.V1_UP	-1.16	0.68	0	0	0	not significant
4176	DIRMEIER_LMP1_RESPONSE_LATE_UP	-1.16	0.68	0	0	0	not significant
4177	GO_POSITIVE_REGULATION_OF_TRANSMEMBRANE_RECEPTOR_PROTEI	-1.16	0.68	0	0	0	not significant
4178	GO_PROTEIN_HOMOTETRAMERIZATION	-1.16	0.68	0	0	0	not significant
4179	GO_MODIFICATION_BY_SYMBIONT_OF_HOST_MORPHOLOGY_OR_PHYSI	-1.16	0.67	0	0	0	not significant
4180	PID_RAC1_PATHWAY	-1.16	0.66	0	0	0	not significant
4181	GO_EXOGENOUS_PROTEIN_BINDING	-1.16	0.65	0	0	0	not significant
4182	GO_HISTONE_PHOSPHORYLATION	-1.16	0.65	0	0	0	not significant
4183	GO_NUCLEOTIDE_EXCISION_REPAIR_DNA_INCISION	-1.16	0.65	0	0	0	not significant
4184	GO_T_CELL_APOPTOTIC_PROCESS	-1.16	0.65	0	0	0	not significant
4185	MARSON_FOXP3_TARGETS_UP	-1.16	0.65	0	0	0	not significant
4186	GAUSSMANN_MLL_AF4_FUSION_TARGETS_E_UP	-1.16	0.64	0	0	0	not significant
4187	GO_ACIDIC_AMINO_ACID_TRANSPORT	-1.16	0.63	0	0	0	not significant
4188	GUO_HEX_TARGETS_DN	-1.16	0.63	0	0	0	not significant
4189	PID_RHOA_PATHWAY	-1.16	0.63	0	0	0	not significant
4190	CHR12P12	-1.16	0.62	0	0	0	not significant
4191	COLLIS_PRKDC_SUBSTRATES	-1.16	0.62	0	0	0	not significant
4192	WILLERT_WNT_SIGNALING	-1.16	0.62	0	0	0	not significant
4193	GO_POSITIVE_REGULATION_OF_PROTEIN_COMPLEX_DISASSEMBLY	-1.16	0.61	0	0	0	not significant
4194	KEGG_GLUTATHIONE_METABOLISM	-1.16	0.61	0	0	0	not significant
4195	CROONQUIST_NRAS_SIGNALING_UP	-1.16	0.60	0	0	0	not significant
4196	GO_CELLULAR_RESPONSE_TO_VASCULAR_ENDOTHELIAL_GROWTH_FA	-1.16	0.60	0	0	0	not significant
4197	GO_NEGATIVE_REGULATION_OF_VIRAL_GENOME_REPLICATION	-1.16	0.60	0	0	0	not significant
4198	HARRIS_BRAIN_CANCER_PROGENITORS	-1.16	0.60	0	0	0	not significant
4199	STEIN_ESRRA_TARGETS_RESPONSIVE_TO_ESTROGEN_DN	-1.16	0.60	0	0	0	not significant
4200	BERENJENO_ROCK_SIGNALING_NOT_VIA_RHOA_UP	-1.16	0.59	0	0	0	not significant
4201	GUILLAUMOND_KLF10_TARGETS_DN	-1.16	0.59	0	0	0	not significant
4202	GENTILE_UV_LOW_DOSE_UP	-1.16	0.58	0	0	0	not significant
4203	GO_ANTERIOR_POSTERIOR_AXIS_SPECIFICATION	-1.16	0.58	0	0	0	not significant
4204	GO_ANTIMICROBIAL_HUMORAL_IMMUNE_RESPONSE_MEDIATED_BY_ANT	-1.16	0.58	0	0	0	not significant
4205	REACTOME_PROTEIN_METHYLATION	-1.16	0.58	0	0	0	not significant
4206	VARELA_ZMPSTE24_TARGETS_DN	-1.16	0.58	0	0	0	not significant
4207	GO_ASTROCYTE_DEVELOPMENT	-1.16	0.57	0	0	0	not significant
4208	GO_HISTONE_H2A_K63_LINKED_UBIQUITINATION	-1.16	0.57	0	0	0	not significant
4209	GO_RESPONSE_TO_OXYGEN_RADICAL	-1.16	0.57	0	0	0	not significant
4210	BIOCARTA_RANBP2_PATHWAY	-1.16	0.56	0	0	0	not significant
4211	GO_REGULATION_OF_METANEPHROS_DEVELOPMENT	-1.16	0.56	0	0	0	not significant
4212	SILIGAN_TARGETS_OF_EWS_FLI1_FUSION_DN	-1.16	0.56	0	0	0	not significant
4213	DE_YY1_TARGETS_UP	-1.16	0.55	0	0	0	not significant
4214	GO_REGULATION_OF_MEMBRANE_PROTEIN_ECTODOMAIN_PROTEOLYS	-1.16	0.55	0	0	0	not significant
4215	RAY_TARGETS_OF_P210_BCR_ABL_FUSION_UP	-1.16	0.55	0	0	0	not significant
4216	REACTOME_OAS_ANTIVIRAL_RESPONSE	-1.16	0.55	0	0	0	not significant
4217	XU_HGF_TARGETS_INDUCED_BY_AKT1_6HR	-1.16	0.55	0	0	0	not significant
4218	GO_POSITIVE_REGULATION_OF_LYMPHOCYTE_CHEMOTAXIS	-1.16	0.54	0	0	0	not significant
4219	GO_POSITIVE_REGULATION_OF_MEMBRANE_PROTEIN_ECTODOMAIN_PI	-1.16	0.54	0	0	0	not significant
4220	BIOCARTA_SRCRPTP_PATHWAY	-1.16	0.53	0	0	0	not significant
4221	GO_ADENOSINE_DEAMINASE_ACTIVITY	-1.16	0.53	0	0	0	not significant
4222	GO_DEATH_DOMAIN_BINDING	-1.16	0.53	0	0	0	not significant
4223	GO_GERM_CELL_NUCLEUS	-1.16	0.53	0	0	0	not significant
4224	GO_LUNG_CELL_DIFFERENTIATION	-1.16	0.53	0	0	0	not significant
4225	GO_MAST_CELL_MIGRATION	-1.16	0.53	0	0	0	not significant
4226	GO_L_SERINE_BIOSYNTHETIC_PROCESS	-1.16	0.52	0	0	0	not significant
4227	GO_MORPHOGENESIS_OF_AN_EPITHELIAL_FOLD	-1.16	0.52	0	0	0	not significant
4228	GO_OBSERVATIONAL_LEARNING	-1.16	0.51	0	0	0	not significant
4229	GO_PLATELET_DENSE_GRANULE_MEMBRANE	-1.16	0.51	0	0	0	not significant
4230	GO_LSM1_7_PAT1_COMPLEX	-1.16	0.50	0	0	0	not significant
4231	GO_POSITIVE_REGULATION_OF_INTERFERON_GAMMA_BIOSYNTHETIC_I	-1.16	0.50	0	0	0	not significant
4232	GO_TELOMERASE_ACTIVITY	-1.16	0.50	0	0	0	not significant
4233	GO_L_GLUTAMATE_TRANSMEMBRANE_TRANSPORTER_ACTIVITY	-1.16	0.49	0	0	0	not significant
4234	GO_POSITIVE_REGULATION_OF_HISTONE_UBIQUITINATION	-1.16	0.48	0	0	0	not significant
4235	INAMURA_LUNG_CANCER_SCC_DN	-1.16	0.48	0	0	0	not significant
4236	GO_CELL_CYCLE	-1.15	4.00	0	0	0	not significant
4237	GO_REGULATION_OF_CELL_CYCLE	-1.15	4.00	0	0	0	not significant
4238	GO_CYTOPLASMIC_VESICLE_PART	-1.15	1.60	0	0	0	not significant
4239	GO_POSITIVE_REGULATION_OF_TRANSPORT	-1.15	1.39	0	0	0	not significant

4240	GO_NEGATIVE_REGULATION_OF_CELLULAR_COMPONENT_ORGANIZATI(	-1.15	1.25	0	0	0	not significant
4241	KOINUMA_TARGETS_OF_SMAD2_OR_SMAD3	-1.15	1.25	0	0	0	not significant
4242	GO_ORGANOPHOSPHATE_BIOSYNTHETIC_PROCESS	-1.15	1.23	0	0	0	not significant
4243	GO_REGULATION_OF_NUCLEOBASE_CONTAINING_COMPOUND_METABO	-1.15	1.22	0	0	0	not significant
4244	TOYOTA_TARGETS_OF_MIR34B_AND_MIR34C	-1.15	1.19	0	0	0	not significant
4245	GO_MUSCLE_STRUCTURE_DEVELOPMENT	-1.15	1.18	0	0	0	not significant
4246	GO_DEVELOPMENTAL_GROWTH	-1.15	1.14	0	0	0	not significant
4247	SMID_BREAST_CANCER_NORMAL_LIKE_UP	-1.15	1.05	0	0	0	not significant
4248	GO_REGULATION_OF_BODY_FLUID_LEVELS	-1.15	0.95	0	0	0	not significant
4249	GO_RIBONUCLEOPROTEIN_COMPLEX_SUBUNIT_ORGANIZATION	-1.15	0.95	0	0	0	not significant
4250	GO_RESPONSE_TO_ANTIBIOTIC	-1.15	0.90	0	0	0	not significant
4251	E2F1_UP.V1_UP	-1.15	0.85	0	0	0	not significant
4252	GO_REGULATION_OF_CELLULAR_PROTEIN_CATABOLIC_PROCESS	-1.15	0.85	0	0	0	not significant
4253	GO_OUTER_MEMBRANE	-1.15	0.84	0	0	0	not significant
4254	WANG_MLL_TARGETS	-1.15	0.83	0	0	0	not significant
4255	HALLMARK_DNA_REPAIR	-1.15	0.81	0	0	0	not significant
4256	PENG_GLUCOSE_DEPRIVATION_DN	-1.15	0.77	0	0	0	not significant
4257	ZHANG_TLX_TARGETS_DN	-1.15	0.76	0	0	0	not significant
4258	GO_REGULATION_OF_LEUKOCYTE_MIGRATION	-1.15	0.75	0	0	0	not significant
4259	SAFFORD_T_LYMPHOCYTE_ANERGY	-1.15	0.72	0	0	0	not significant
4260	REACTOME_RNA_POLYMERASE_I_TRANSCRIPTION_INITIATION	-1.15	0.70	0	0	0	not significant
4261	GO_PROTON_TRANSMEMBRANE_TRANSPORTER_ACTIVITY	-1.15	0.69	0	0	0	not significant
4262	HALLMARK_BILE_ACID_METABOLISM	-1.15	0.69	0	0	0	not significant
4263	GO_EYE_MORPHOGENESIS	-1.15	0.68	0	0	0	not significant
4264	GO_REGULATION_OF_UBIQUITIN_PROTEIN_TRANSFERASE_ACTIVITY	-1.15	0.68	0	0	0	not significant
4265	REACTOME_FCGAMMA_RECEPTOR_FCGR_DEPENDENT_PHAGOCYTOSIS	-1.15	0.68	0	0	0	not significant
4266	ROSS_ACUTE_MYELOID_LEUKEMIA_CBF	-1.15	0.68	0	0	0	not significant
4267	GAVIN_FOXP3_TARGETS_CLUSTER_T4	-1.15	0.67	0	0	0	not significant
4268	RICKMAN_TUMOR_DIFFERENTIATED_MODERATELY_VS_POORLY_UP	-1.15	0.67	0	0	0	not significant
4269	ROSS_AML_WITH_MLL_FUSIONS	-1.15	0.67	0	0	0	not significant
4270	GO_LYMPHOCYTE_APOPTOTIC_PROCESS	-1.15	0.66	0	0	0	not significant
4271	GO_FIBROBLAST_PROLIFERATION	-1.15	0.65	0	0	0	not significant
4272	GO_IRE1_MEDIATED_UNFOLDED_PROTEIN_RESPONSE	-1.15	0.65	0	0	0	not significant
4273	REACTOME_INOSITOL_PHOSPHATE_METABOLISM	-1.15	0.65	0	0	0	not significant
4274	REACTOME_DNA_DAMAGE_RECOGNITION_IN_GG_NER	-1.15	0.62	0	0	0	not significant
4275	SEITZ_NEOPLASTIC_TRANSFORMATION_BY_8P_DELETION_UP	-1.15	0.62	0	0	0	not significant
4276	GO_REGULATION_OF_POTASSIUM_ION_TRANSMEMBRANE_TRANSPORTI	-1.15	0.61	0	0	0	not significant
4277	REACTOME_SIGNALING_BY_FGFR2	-1.15	0.61	0	0	0	not significant
4278	GO_CHAPERONE_COFACTOR_DEPENDENT_PROTEIN_REFOLDING	-1.15	0.59	0	0	0	not significant
4279	GO_MOVEMENT_IN_ENVIRONMENT_OF_OTHER_ORGANISM_INVOLVED_II	-1.15	0.59	0	0	0	not significant
4280	GO_OXIDOREDUCTASE_ACTIVITY_ACTING_ON_THE_ALDEHYDE_OR_OX(	-1.15	0.59	0	0	0	not significant
4281	REACTOME_GABA_RECEPTOR_ACTIVATION	-1.15	0.59	0	0	0	not significant
4282	GO_GLANDULAR_EPITHELIAL_CELL_DEVELOPMENT	-1.15	0.58	0	0	0	not significant
4283	GO_QUINONE_BINDING	-1.15	0.58	0	0	0	not significant
4284	GO_RESPONSE_TO_AUDITORY_STIMULUS	-1.15	0.58	0	0	0	not significant
4285	VALK_AML_WITH_FLT3_ITD	-1.15	0.58	0	0	0	not significant
4286	VANASSE_BCL2_TARGETS_DN	-1.15	0.58	0	0	0	not significant
4287	ZHU_CMV_8_HR_DN	-1.15	0.58	0	0	0	not significant
4288	BURTON_ADIPOGENESIS_PEAK_AT_2HR	-1.15	0.57	0	0	0	not significant
4289	GO_NEGATIVE_REGULATION_OF_CELL_JUNCTION_ASSEMBLY	-1.15	0.57	0	0	0	not significant
4290	GO_NEGATIVE_REGULATION_OF_GLIOGENESIS	-1.15	0.57	0	0	0	not significant
4291	GO_RESPONSE_TO_AMPHETAMINE	-1.15	0.57	0	0	0	not significant
4292	TURASHVILI_BREAST_CARCINOMA_DUCTAL_VS_LOBULAR_UP	-1.15	0.57	0	0	0	not significant
4293	CHR2Q21	-1.15	0.56	0	0	0	not significant
4294	GO_ANTIGEN_PROCESSING_AND_PRESENTATION_OF_ENDOGENOUS_PE	-1.15	0.56	0	0	0	not significant
4295	GO_MELANIN_BIOSYNTHETIC_PROCESS	-1.15	0.56	0	0	0	not significant
4296	GO_NEGATIVE_REGULATION_OF_DNA_BIOSYNTHETIC_PROCESS	-1.15	0.56	0	0	0	not significant
4297	GO_POSITIVE_REGULATION_OF_B_CELL_DIFFERENTIATION	-1.15	0.56	0	0	0	not significant
4298	GO_DEATH_RECEPTOR_BINDING	-1.15	0.55	0	0	0	not significant
4299	GO_EXTRINSIC_COMPONENT_OF_MITOCHONDRIAL_INNER_MEMBRANE	-1.15	0.55	0	0	0	not significant
4300	GO_RESPONSE_TO_NICOTINE	-1.15	0.55	0	0	0	not significant
4301	GO_MITOCHONDRIAL_FRAGMENTATION_INVOLVED_IN_APOPTOTIC_PRO	-1.15	0.54	0	0	0	not significant
4302	TERAMOTO_OPN_TARGETS_CLUSTER_6	-1.15	0.54	0	0	0	not significant
4303	FIGUEROA_AML_METHYLATION_CLUSTER_5_UP	-1.15	0.53	0	0	0	not significant
4304	GO_BETA_CATENIN_DESTRUCTION_COMPLEX	-1.15	0.53	0	0	0	not significant
4305	GO_CELLULAR_RESPONSE_TO_INTERFERON_BETA	-1.15	0.53	0	0	0	not significant
4306	GO_NADH_PYROPHOSPHATASE_ACTIVITY	-1.15	0.53	0	0	0	not significant
4307	GO_PHOTORECEPTOR_CONNECTING_CILIUM	-1.15	0.53	0	0	0	not significant
4308	GO_PRESPLICEOSOME	-1.15	0.53	0	0	0	not significant
4309	GO_EXTRINSIC_COMPONENT_OF_POSTSYNAPTIC_MEMBRANE	-1.15	0.52	0	0	0	not significant
4310	GO_NEGATIVE_REGULATION_OF_T_HELPER_CELL_DIFFERENTIATION	-1.15	0.52	0	0	0	not significant
4311	GO_POSITIVE_REGULATION_OF_BLOOD_PRESSURE	-1.15	0.52	0	0	0	not significant
4312	PID_S1P_S1P4_PATHWAY	-1.15	0.52	0	0	0	not significant
4313	XU_HGF_TARGETS_INDUCED_BY_AKT1_48HR_UP	-1.15	0.52	0	0	0	not significant
4314	GO_CELLULAR_SODIUM_ION_HOMEOSTASIS	-1.15	0.51	0	0	0	not significant
4315	PHONG_TNF_TARGETS_DN	-1.15	0.51	0	0	0	not significant
4316	REACTOME_LINOLEIC_ACID_LA_METABOLISM	-1.15	0.51	0	0	0	not significant
4317	FUKUSHIMA_TNFSF11_TARGETS	-1.15	0.50	0	0	0	not significant
4318	GO_CHROMO_SHADOW_DOMAIN_BINDING	-1.15	0.50	0	0	0	not significant
4319	GO_NEGATIVE_REGULATION_OF_MEMBRANE_POTENTIAL	-1.15	0.50	0	0	0	not significant
4320	GO_REGULATION_OF_TOLL_LIKE_RECEPTOR_4_SIGNALING_PATHWAY	-1.15	0.50	0	0	0	not significant
4321	GO_U4_SNRNA_3_END_PROCESSING	-1.15	0.50	0	0	0	not significant
4322	REICHERT_G1S_REGULATORS_AS_PI3K_TARGETS	-1.15	0.50	0	0	0	not significant
4323	GO_ASTROCYTE_CELL_MIGRATION	-1.15	0.49	0	0	0	not significant
4324	KIM_RESPONSE_TO_TSA_AND_DECITABINE_DN	-1.15	0.49	0	0	0	not significant
4325	GO_POSITIVE_REGULATION_OF_PROTEIN_MONOUBIQUITINATION	-1.15	0.48	0	0	0	not significant
4326	GO_PURINE_NUCLEOSIDE_DIPHOSPHATE_BIOSYNTHETIC_PROCESS	-1.15	0.48	0	0	0	not significant
4327	GO_REGULATION_OF_CATECHOLAMINE_METABOLIC_PROCESS	-1.15	0.48	0	0	0	not significant
4328	GO_REGULATION_OF_PEPTIDYL_CYSTEINE_S_NITROSYLATION	-1.15	0.48	0	0	0	not significant
4329	REACTOME_INTERLEUKIN_1_PROCESSING	-1.15	0.48	0	0	0	not significant
4330	GO_INTERFERON_GAMMA_BIOSYNTHETIC_PROCESS	-1.15	0.47	0	0	0	not significant
4331	BENPORATH_NANOG_TARGETS	-1.14	1.72	0	0	0	not significant
4332	GO_CELL_MOTILITY	-1.14	1.72	0	0	0	not significant
4333	GO_ORGANIC_CYCLIC_COMPOUND_CATABOLIC_PROCESS	-1.14	1.17	0	0	0	not significant
4334	REACTOME_DNA_REPAIR	-1.14	1.13	0	0	0	not significant
4335	PHONG_TNF_RESPONSE_NOT_VIA_P38	-1.14	1.10	0	0	0	not significant
4336	GO_RESPONSE_TO_INORGANIC_SUBSTANCE	-1.14	1.04	0	0	0	not significant
4337	GRYDER_PAX3FOXO1_ENHANCERS_KO_DOWN	-1.14	1.00	0	0	0	not significant
4338	GO_REGULATION_OF_HEMOPOIESIS	-1.14	0.94	0	0	0	not significant
4339	GO_SIDE_OF_MEMBRANE	-1.14	0.93	0	0	0	not significant
4340	GO_MACROMOLECULE_METHYLATION	-1.14	0.92	0	0	0	not significant
4341	GO_REGULATION_OF_ACTIN_FILAMENT_BASED_PROCESS	-1.14	0.92	0	0	0	not significant
4342	SENGUPTA_NASOPHARYNGEAL_CARCINOMA_WITH_LMP1_UP	-1.14	0.91	0	0	0	not significant
4343	CHARAFE_BREAST_CANCER_LUMINAL_VS_BASAL_DN	-1.14	0.89	0	0	0	not significant
4344	GO_POSITIVE_REGULATION_OF_CELL_ACTIVATION	-1.14	0.86	0	0	0	not significant
4345	GO_AGING	-1.14	0.83	0	0	0	not significant
4346	OSWALD_HEMATOPOIETIC_STEM_CELL_IN_COLLAGEN_GEL_UP	-1.14	0.82	0	0	0	not significant
4347	SRC_UP.V1_UP	-1.14	0.77	0	0	0	not significant
4348	GO_RECEPTOR_MEDIATED_ENDOCYTOSIS	-1.14	0.76	0	0	0	not significant

4349	GO_REGULATION_OF_DEPHOSPHORYLATION	-1.14	0.74	0	0	0	not significant
4350	GO_POSITIVE_REGULATION_OF_LEUKOCYTE_DIFFERENTIATION	-1.14	0.73	0	0	0	not significant
4351	LI_AMPLIFIED_IN_LUNG_CANCER	-1.14	0.73	0	0	0	not significant
4352	IWANAGA_CARCINOGENESIS_BY_KRAS_PTEN_UP	-1.14	0.72	0	0	0	not significant
4353	KEGG_PYRIMIDINE_METABOLISM	-1.14	0.70	0	0	0	not significant
4354	TSAI_RESPONSE_TO_IONIZING_RADIATION	-1.14	0.70	0	0	0	not significant
4355	CROONQUIST_NRAS_VS_STROMAL_STIMULATION_DN	-1.14	0.69	0	0	0	not significant
4356	GO_MRNA_3_UTR_BINDING	-1.14	0.69	0	0	0	not significant
4357	GO_VITAMIN_METABOLIC_PROCESS	-1.14	0.69	0	0	0	not significant
4358	WATANABE_RECTAL_CANCER_RADIOTHERAPY_RESPONSIVE_UP	-1.14	0.69	0	0	0	not significant
4359	GO_NEGATIVE_REGULATION_OF_EPITHELIAL_CELL_PROLIFERATION	-1.14	0.66	0	0	0	not significant
4360	GO_CARDIAC_CHAMBER_DEVELOPMENT	-1.14	0.65	0	0	0	not significant
4361	REACTOME_PI_METABOLISM	-1.14	0.65	0	0	0	not significant
4362	GO_ACTIN_MEDIATED_CELL_CONTRACTION	-1.14	0.64	0	0	0	not significant
4363	GO_NEGATIVE_REGULATION_OF_MAP_KINASE_ACTIVITY	-1.14	0.64	0	0	0	not significant
4364	GO_POSITIVE_REGULATION_OF_INFLAMMATORY_RESPONSE	-1.14	0.64	0	0	0	not significant
4365	LEE_NEURAL_CREST_STEM_CELL_DN	-1.14	0.64	0	0	0	not significant
4366	REACTOME_TRANSCRIPTIONAL_REGULATION_OF_WHITE_ADIPOCYTE_D	-1.14	0.64	0	0	0	not significant
4367	BIOCARTA_HIVNEF_PATHWAY	-1.14	0.63	0	0	0	not significant
4368	GO_ACTIN_FILAMENT_BASED_MOVEMENT	-1.14	0.63	0	0	0	not significant
4369	PID_CASPASE_PATHWAY	-1.14	0.63	0	0	0	not significant
4370	PTEN_DN.V2_UP	-1.14	0.63	0	0	0	not significant
4371	GO_POSITIVE_REGULATION_OF_CYTOKINE_BIOSYNTHETIC_PROCESS	-1.14	0.62	0	0	0	not significant
4372	GO_PROTEIN_TRANSPORT_ALONG_MICROTUBULE	-1.14	0.62	0	0	0	not significant
4373	GUTIERREZ_CHRONIC_LYMPHOCYTIC_LEUKEMIA_DN	-1.14	0.62	0	0	0	not significant
4374	NEMETH_INFLAMMATORY_RESPONSE_LPS_UP	-1.14	0.62	0	0	0	not significant
4375	FONTAINE_PAPILLARY_THYROID_CARCINOMA_UP	-1.14	0.61	0	0	0	not significant
4376	GO_T_CELL_MEDIATED_CYTOTOXICITY	-1.14	0.61	0	0	0	not significant
4377	GO_VISUAL_BEHAVIOR	-1.14	0.61	0	0	0	not significant
4378	BROWNE_HCMV_INFECTION_2HR_DN	-1.14	0.60	0	0	0	not significant
4379	GO_REGULATION_OF_BEHAVIOR	-1.14	0.60	0	0	0	not significant
4380	GO_REGULATION_OF_HEART_GROWTH	-1.14	0.60	0	0	0	not significant
4381	GO_REGULATION_OF_LIPID_STORAGE	-1.14	0.60	0	0	0	not significant
4382	GO_DICARBOXYLIC_ACID_METABOLIC_PROCESS	-1.14	0.59	0	0	0	not significant
4383	GO_MHC_PROTEIN_BINDING	-1.14	0.59	0	0	0	not significant
4384	REACTOME_PERK_REGULATES_GENE_EXPRESSION	-1.14	0.59	0	0	0	not significant
4385	GO_DOPAMINE_RECEPTOR_SIGNALING_PATHWAY	-1.14	0.58	0	0	0	not significant
4386	GO_L_AMINO_ACID_TRANSMEMBRANE_TRANSPORTER_ACTIVITY	-1.14	0.58	0	0	0	not significant
4387	GO_REGULATION_OF_LAMELLIPODIUM_ASSEMBLY	-1.14	0.58	0	0	0	not significant
4388	BIOCARTA_TNFR1_PATHWAY	-1.14	0.57	0	0	0	not significant
4389	BRACHAT_RESPONSE_TO_METHOTREXATE_DN	-1.14	0.57	0	0	0	not significant
4390	GO_PROTEIN_LOCALIZATION_TO_CHROMATIN	-1.14	0.57	0	0	0	not significant
4391	GO_REGULATION_OF_MONONUCLEAR_CELL_MIGRATION	-1.14	0.57	0	0	0	not significant
4392	CHR17Q22	-1.14	0.56	0	0	0	not significant
4393	HUANG_FOXA2_TARGETS_DN	-1.14	0.56	0	0	0	not significant
4394	YAO_TEMPORAL_RESPONSE_TO_PROGESTERONE_CLUSTER_15	-1.14	0.56	0	0	0	not significant
4395	CHR4Q25	-1.14	0.55	0	0	0	not significant
4396	GO_INTERCALATED_DISC	-1.14	0.55	0	0	0	not significant
4397	GO_NEUROMUSCULAR_PROCESS_CONTROLLING_POSTURE	-1.14	0.55	0	0	0	not significant
4398	GO_REGULATION_OF_MRNA_3_END_PROCESSING	-1.14	0.55	0	0	0	not significant
4399	KEGG_TYPE_I_DIABETES_MELLITUS	-1.14	0.55	0	0	0	not significant
4400	LEE_LIVER_CANCER_MYC_TGFA_DN	-1.14	0.55	0	0	0	not significant
4401	PID_NECTIN_PATHWAY	-1.14	0.55	0	0	0	not significant
4402	TIMOFEEVA_GROWTH_STRESS_VIA_STAT1_DN	-1.14	0.55	0	0	0	not significant
4403	BIOCARTA_CDK5_PATHWAY	-1.14	0.54	0	0	0	not significant
4404	GO_PLASMA_MEMBRANE_TUBULATION	-1.14	0.54	0	0	0	not significant
4405	GO_NEURON_PROJECTION_MEMBRANE	-1.14	0.53	0	0	0	not significant
4406	GO_O_METHYLTRANSFERASE_ACTIVITY	-1.14	0.53	0	0	0	not significant
4407	KEGG_STEROID_BIOSYNTHESIS	-1.14	0.53	0	0	0	not significant
4408	MIKKELSEN_MEF_ICP_WITH_H3K4ME3_AND_H3K27ME3	-1.14	0.53	0	0	0	not significant
4409	PID_HIF1A_PATHWAY	-1.14	0.53	0	0	0	not significant
4410	REACTOME_DISEASES_ASSOCIATED_WITH_GLYCOSYLATION_PRECURS	-1.14	0.53	0	0	0	not significant
4411	RICKMAN_TUMOR_DIFFERENTIATED_MODERATELY_VS_POORLY_DN	-1.14	0.53	0	0	0	not significant
4412	GO_CALCIUM_MEDIATED_SIGNALING_USING_INTRACELLULAR_CALCIUM_	-1.14	0.52	0	0	0	not significant
4413	GO_N_TERMINAL_PEPTIDYL_METHIONINE_ACETYLATION	-1.14	0.52	0	0	0	not significant
4414	GO_POSITIVE_REGULATION_OF_CARDIAC_MUSCLE_CELL_PROLIFERATI	-1.14	0.52	0	0	0	not significant
4415	KEGG_RIBOFLAVIN_METABOLISM	-1.14	0.52	0	0	0	not significant
4416	REACTOME_ENOS_ACTIVATION	-1.14	0.52	0	0	0	not significant
4417	SESTO_RESPONSE_TO_UV_C3	-1.14	0.52	0	0	0	not significant
4418	GO_CHEMOKINE_SECRETION	-1.14	0.51	0	0	0	not significant
4419	GO_NEURAL_CREST_FORMATION	-1.14	0.51	0	0	0	not significant
4420	GO_PHOSPHATIDYLETHANOLAMINE_BIOSYNTHETIC_PROCESS	-1.14	0.51	0	0	0	not significant
4421	GO_FATTY_ACID_BETA_OXIDATION_USING_ACYL_COA_DEHYDROGENAS	-1.14	0.50	0	0	0	not significant
4422	GO_FRUCTOSE_6_PHOSPHATE_METABOLIC_PROCESS	-1.14	0.50	0	0	0	not significant
4423	GO_MOTOR_NEURON_APOPTOTIC_PROCESS	-1.14	0.50	0	0	0	not significant
4424	GO_REGULATION_OF_CYCLIC_NUCLEOTIDE_PHOSPHODIESTERASE_ACT	-1.14	0.50	0	0	0	not significant
4425	GO_REGULATION_OF_RECEPTOR_CLUSTERING	-1.14	0.50	0	0	0	not significant
4426	REACTOME_REGULATION_OF_GENE_EXPRESSION_BY_HYPOXIA_INDUCI	-1.14	0.50	0	0	0	not significant
4427	GO_GLOMERULAR_EPITHELIUM_DEVELOPMENT	-1.14	0.49	0	0	0	not significant
4428	GO_NEGATIVE_REGULATION_OF_LEUKOCYTE_DEGRANULATION	-1.14	0.49	0	0	0	not significant
4429	GO_NEGATIVE_REGULATION_OF_RETINOIC_ACID_RECEPTOR_SIGNALIN	-1.14	0.49	0	0	0	not significant
4430	GO_POSITIVE_REGULATION_OF_NEURON_MIGRATION	-1.14	0.49	0	0	0	not significant
4431	GO_RESPONSE_TO_NITROSATIVE_STRESS	-1.14	0.49	0	0	0	not significant
4432	GO_RNA_DIRECTED_DNA_POLYMERASE_ACTIVITY	-1.14	0.49	0	0	0	not significant
4433	BIOCARTA_DSP_PATHWAY	-1.14	0.48	0	0	0	not significant
4434	TAGHAVI_NEOPLASTIC_TRANSFORMATION	-1.14	0.48	0	0	0	not significant
4435	GO_NUCLEAR_RETENTION_OF_PRE_MRNA_AT_THE_SITE_OF_TRANSCR	-1.14	0.46	0	0	0	not significant
4436	GO_REGULATION_OF_GOLGI_TO_PLASMA_MEMBRANE_PROTEIN_TRANS	-1.14	0.44	0	0	0	not significant
4437	GO_HYDROXYMETHYL__FORMYL_AND_RELATED_TRANSFERASE_ACTIV	-1.14	0.43	0	0	0	not significant
4438	GO_POSITIVE_REGULATION_OF_PROTEIN_METABOLIC_PROCESS	-1.13	1.94	0	0	0	not significant
4439	GO_PROTEOLYSIS	-1.13	1.70	0	0	0	not significant
4440	GO_REGULATION_OF_TRANSPORT	-1.13	1.28	0	0	0	not significant
4441	GO_BIOLOGICAL_ADHESION	-1.13	1.25	0	0	0	not significant
4442	GO_ORGANELLE_LOCALIZATION	-1.13	0.99	0	0	0	not significant
4443	GO_CELLULAR_RESPONSE_TO_NITROGEN_COMPOUND	-1.13	0.98	0	0	0	not significant
4444	GO_REGULATION_OF_PROTEIN_CATABOLIC_PROCESS	-1.13	0.94	0	0	0	not significant
4445	GRAESSMANN_APOPTOSIS_BY_SERUM_DEPRIVATION_UP	-1.13	0.92	0	0	0	not significant
4446	GO_MYELOID_CELL_DIFFERENTIATION	-1.13	0.90	0	0	0	not significant
4447	GO_VESICLE_ORGANIZATION	-1.13	0.84	0	0	0	not significant
4448	GO_NUCLEIC_ACID_PHOSPHODIESTER_BOND_HYDROLYSIS	-1.13	0.83	0	0	0	not significant
4449	VANOEVELEN_MYOGENESIS_SIN3A_TARGETS	-1.13	0.79	0	0	0	not significant
4450	GO_CARBOHYDRATE_BINDING	-1.13	0.77	0	0	0	not significant
4451	GO_NEGATIVE_REGULATION_OF_CELL_ADHESION	-1.13	0.77	0	0	0	not significant
4452	YAGI_AML_FAB_MARKERS	-1.13	0.77	0	0	0	not significant
4453	GO_CELL_CELL_JUNCTION	-1.13	0.74	0	0	0	not significant
4454	GO_PHAGOCYTIC_VESICLE	-1.13	0.73	0	0	0	not significant
4455	STK33_SKM_DN	-1.13	0.73	0	0	0	not significant
4456	RIZKI_TUMOR_INVASIVENESS_3D_DN	-1.13	0.72	0	0	0	not significant
4457	GO_REGULATION_OF_CIRCADIAN_RHYTHM	-1.13	0.68	0	0	0	not significant

4458	SCHLOSSER_SERUM_RESPONSE_AUGMENTED_BY_MYC	-1.13	0.68	0	0	0	not significant
4459	GO_LEUKOCYTE_MEDIATED_CYTOTOXICITY	-1.13	0.67	0	0	0	not significant
4460	GO_HEART_MORPHOGENESIS	-1.13	0.66	0	0	0	not significant
4461	GO_P_BODY	-1.13	0.66	0	0	0	not significant
4462	REACTOME_TOLL_LIKE_RECEPTOR_9_TLR9_CASCADE	-1.13	0.66	0	0	0	not significant
4463	GO_INOSITOL_PHOSPHATE_METABOLIC_PROCESS	-1.13	0.65	0	0	0	not significant
4464	REACTOME_PROCESSING_OF_DNA_DOUBLE_STRAND_BREAK_ENDS	-1.13	0.65	0	0	0	not significant
4465	MEL18_DN.V1_UP	-1.13	0.64	0	0	0	not significant
4466	HUANG_DASATINIB_RESISTANCE_DN	-1.13	0.63	0	0	0	not significant
4467	FAELT_B_CLL_WITH_VH_REARRANGEMENTS_DN	-1.13	0.62	0	0	0	not significant
4468	GO_MEMORY	-1.13	0.62	0	0	0	not significant
4469	MULLIGHAN_NPM1_MUTATED_SIGNATURE_2_DN	-1.13	0.62	0	0	0	not significant
4470	PID_TGFBR_PATHWAY	-1.13	0.62	0	0	0	not significant
4471	GO_MICROVILLUS	-1.13	0.61	0	0	0	not significant
4472	HUMMEL_BURKITTS_LYMPHOMA_UP	-1.13	0.61	0	0	0	not significant
4473	GO_ASSOCIATIVE_LEARNING	-1.13	0.60	0	0	0	not significant
4474	GO_INTERFERON_GAMMA_MEDIATED_SIGNALING_PATHWAY	-1.13	0.60	0	0	0	not significant
4475	REACTOME_SIGNALING_BY_FGFR	-1.13	0.60	0	0	0	not significant
4476	GO_ATTACHMENT_OF_SPINDLE_MICROTUBULES_TO_KINETOCHORE	-1.13	0.59	0	0	0	not significant
4477	GO_GLYCOSYL_COMPOUND_METABOLIC_PROCESS	-1.13	0.59	0	0	0	not significant
4478	GO_PROTEIN_LOCALIZATION_TO_GOLGI_APPARATUS	-1.13	0.59	0	0	0	not significant
4479	GO_REGULATION_OF_ATPASE_ACTIVITY	-1.13	0.59	0	0	0	not significant
4480	ROSS_AML_WITH_AML1_ETO_FUSION	-1.13	0.59	0	0	0	not significant
4481	GO_BODY_FLUID_SECRETION	-1.13	0.58	0	0	0	not significant
4482	GO_CENTROMERE_COMPLEX_ASSEMBLY	-1.13	0.58	0	0	0	not significant
4483	REACTOME_SIRT1_NEGATIVELY_REGULATES_RRNA_EXPRESSION	-1.13	0.58	0	0	0	not significant
4484	CAFFAREL_RESPONSE_TO_THC_24HR_5_UP	-1.13	0.56	0	0	0	not significant
4485	STEARMAN_LUNG_CANCER_EARLY_VS_LATE_DN	-1.13	0.56	0	0	0	not significant
4486	VARELA_ZMPSTE24_TARGETS_UP	-1.13	0.56	0	0	0	not significant
4487	BURTON_ADIPOGENESIS_10	-1.13	0.55	0	0	0	not significant
4488	GO_GUANOSINE_CONTAINING_COMPOUND_METABOLIC_PROCESS	-1.13	0.55	0	0	0	not significant
4489	GO_LUNG_ALVEOLUS_DEVELOPMENT	-1.13	0.55	0	0	0	not significant
4490	PID_ATR_PATHWAY	-1.13	0.55	0	0	0	not significant
4491	REACTOME_GLUCAGON_TYPE_LIGAND_RECEPTORS	-1.13	0.55	0	0	0	not significant
4492	YAO_TEMPORAL_RESPONSE_TO_PROGESTERONE_CLUSTER_8	-1.13	0.55	0	0	0	not significant
4493	BIOCARTA_SET_PATHWAY	-1.13	0.54	0	0	0	not significant
4494	GO_ANIMAL_ORGAN_FORMATION	-1.13	0.54	0	0	0	not significant
4495	GO_BONE_CELL_DEVELOPMENT	-1.13	0.54	0	0	0	not significant
4496	GO_NEGATIVE_REGULATION_OF_REGULATED_SECRETORY_PATHWAY	-1.13	0.54	0	0	0	not significant
4497	GO_POTASSIUM_CHANNEL_REGULATOR_ACTIVITY	-1.13	0.54	0	0	0	not significant
4498	GO_REGULATION_OF_NUCLEASE_ACTIVITY	-1.13	0.54	0	0	0	not significant
4499	RUAN_RESPONSE_TO_TNF_TROGLITAZONE_DN	-1.13	0.54	0	0	0	not significant
4500	XU_RESPONSE_TO_TRETINOIN_AND_NSC682994_UP	-1.13	0.54	0	0	0	not significant
4501	YE_METASTATIC_LIVER_CANCER	-1.13	0.54	0	0	0	not significant
4502	CHR2P14	-1.13	0.53	0	0	0	not significant
4503	GO_BRANCHING_INVOLVED_IN_MAMMARY_GLAND_DUCT_MORPHOGENE	-1.13	0.53	0	0	0	not significant
4504	GO_RESPIRATORY_BURST	-1.13	0.53	0	0	0	not significant
4505	ROZANOV_MMP14_TARGETS_SUBSET	-1.13	0.53	0	0	0	not significant
4506	AZARE_STAT3_TARGETS	-1.13	0.52	0	0	0	not significant
4507	CHR2P15	-1.13	0.52	0	0	0	not significant
4508	GO_RECEPTOR_BIOSYNTHETIC_PROCESS	-1.13	0.52	0	0	0	not significant
4509	REACTOME_SYNTHESIS_OF_PIPS_AT_THE_EARLY_ENDOSOME_MEMBRA	-1.13	0.52	0	0	0	not significant
4510	YANG_BREAST_CANCER_ESR1_UP	-1.13	0.52	0	0	0	not significant
4511	CHRXQ21	-1.13	0.51	0	0	0	not significant
4512	GO_IMMUNOGLOBULIN_SECRETION	-1.13	0.51	0	0	0	not significant
4513	GO_PALMITOYL_COA_HYDROLASE_ACTIVITY	-1.13	0.51	0	0	0	not significant
4514	GO_REGULATION_OF_INCLUSION_BODY_ASSEMBLY	-1.13	0.51	0	0	0	not significant
4515	GO_REGULATION_OF_THE_FORCE_OF_HEART_CONTRACTION	-1.13	0.51	0	0	0	not significant
4516	GO_FATTY_ACID_DERIVATIVE_TRANSPORT	-1.13	0.50	0	0	0	not significant
4517	GO_NEGATIVE_REGULATION_OF_CENTRIOLE_REPLICATION	-1.13	0.50	0	0	0	not significant
4518	LIU_VAV3_PROSTATE_CARCINOGENESIS_DN	-1.13	0.50	0	0	0	not significant
4519	PID_ARF_3PATHWAY	-1.13	0.50	0	0	0	not significant
4520	REACTOME_G_BETA:GAMMA_SIGNALLING_THROUGH_PI3KGAMMA	-1.13	0.50	0	0	0	not significant
4521	GO_CYCLIN_DEPENDENT_PROTEIN_SERINE_THREONINE_KINASE_ACTIVA	-1.13	0.49	0	0	0	not significant
4522	KYNG_ENVIRONMENTAL_STRESS_RESPONSE_NOT_BY_UV_IN_WS	-1.13	0.49	0	0	0	not significant
4523	REACTOME_DARPP_32_EVENTS	-1.13	0.49	0	0	0	not significant
4524	GO_ADENOSINE_TO_INOSINE_EDITING	-1.13	0.48	0	0	0	not significant
4525	GO_BILE_ACID_AND_BILE_SALT_TRANSPORT	-1.13	0.48	0	0	0	not significant
4526	GO_CELLULAR_KETONE_BODY_METABOLIC_PROCESS	-1.13	0.48	0	0	0	not significant
4527	GO_NEGATIVE_REGULATION_OF_INTERLEUKIN_8_PRODUCTION	-1.13	0.48	0	0	0	not significant
4528	GO_REGULATION_OF_KERATINOCYTE_MIGRATION	-1.13	0.48	0	0	0	not significant
4529	BIOCARTA_MALATEX_PATHWAY	-1.13	0.47	0	0	0	not significant
4530	GO_AXONEMAL_DYNEIN_COMPLEX	-1.13	0.47	0	0	0	not significant
4531	GO_NEGATIVE_REGULATION_OF_NUCLEASE_ACTIVITY	-1.13	0.47	0	0	0	not significant
4532	GO_REGULATION_OF_DNA_TOPOISOMERASE_ATP_HYDROLYZING_ACTI	-1.13	0.47	0	0	0	not significant
4533	MEINHOLD_OVARIAN_CANCER_LOW_GRADE_UP	-1.13	0.47	0	0	0	not significant
4534	GO_RRNA_ADENINE_METHYLTRANSFERASE_ACTIVITY	-1.13	0.46	0	0	0	not significant
4535	GO_RESPONSE_TO_VITAMIN_E	-1.13	0.45	0	0	0	not significant
4536	REACTOME_SYNTHESIS_OF_DOLICHYL_PHOSPHATE	-1.13	0.45	0	0	0	not significant
4537	GO_MALE_GENITALIA_DEVELOPMENT	-1.13	0.44	0	0	0	not significant
4538	GO_NEGATIVE_REGULATION_OF_VESICLE_FUSION	-1.13	0.42	0	0	0	not significant
4539	GO_REGULATION_OF_ORGANELLE_ORGANIZATION	-1.12	1.49	0	0	0	not significant
4540	NUYTTEN_EZH2_TARGETS_UP	-1.12	1.24	0	0	0	not significant
4541	GO_POSITIVE_REGULATION_OF_HYDROLASE_ACTIVITY	-1.12	1.22	0	0	0	not significant
4542	REACTOME_METABOLISM_OF_LIPIDS	-1.12	1.21	0	0	0	not significant
4543	REACTOME_VESICLE_MEDIATED_TRANSPORT	-1.12	1.07	0	0	0	not significant
4544	GO_NEGATIVE_REGULATION_OF_CELL_CYCLE	-1.12	1.06	0	0	0	not significant
4545	GO_PERINUCLEAR_REGION_OF_CYTOPLASM	-1.12	1.02	0	0	0	not significant
4546	MASSARWEH_TAMOXIFEN_RESISTANCE_UP	-1.12	0.95	0	0	0	not significant
4547	GO_REGULATION_OF_GENE_EXPRESSION_EPIGENETIC	-1.12	0.88	0	0	0	not significant
4548	PASINI_SUZ12_TARGETS_DN	-1.12	0.84	0	0	0	not significant
4549	GENTILE_UV_HIGH_DOSE_DN	-1.12	0.79	0	0	0	not significant
4550	GO_MUSCLE_CELL_DIFFERENTIATION	-1.12	0.77	0	0	0	not significant
4551	KEGG_REGULATION_OF_ACTIN_CYTOSKELETON	-1.12	0.74	0	0	0	not significant
4552	GO_ENDOPLASMIC_RETICULUM_TO_GOLGI_VESICLE_MEDIATED_TRANS	-1.12	0.73	0	0	0	not significant
4553	GO_EXTRACELLULAR_STRUCTURE_ORGANIZATION	-1.12	0.73	0	0	0	not significant
4554	GO_NEGATIVE_REGULATION_OF_NEURON_DIFFERENTIATION	-1.12	0.72	0	0	0	not significant
4555	GO_ALCOHOL_METABOLIC_PROCESS	-1.12	0.71	0	0	0	not significant
4556	GO_POSITIVE_REGULATION_OF_CELL_CELL_ADHESION	-1.12	0.71	0	0	0	not significant
4557	ROZANOV_MMP14_TARGETS_UP	-1.12	0.71	0	0	0	not significant
4558	GO_REGULATION_OF_CELL_SUBSTRATE_ADHESION	-1.12	0.69	0	0	0	not significant
4559	GO_RNA_POLYMERASE_II_TRANSCRIPTION_FACTOR_COMPLEX	-1.12	0.69	0	0	0	not significant
4560	IGARASHI_ATF4_TARGETS_DN	-1.12	0.67	0	0	0	not significant
4561	CHR21Q22	-1.12	0.66	0	0	0	not significant
4562	GO_REGULATION_OF_NUCLEOCYTOPLASMIC_TRANSPORT	-1.12	0.66	0	0	0	not significant
4563	IL15_UP.V1_UP	-1.12	0.66	0	0	0	not significant
4564	ONDER_CDH1_TARGETS_1_UP	-1.12	0.66	0	0	0	not significant
4565	GO_REGULATION_OF_PRODUCTION_OF_MOLECULAR_MEDIATOR_OF_IM	-1.12	0.62	0	0	0	not significant
4566	GO_POTASSIUM_ION_TRANSPORT	-1.12	0.61	0	0	0	not significant

4567	GO_REGULATION_OF_CARBOHYDRATE_CATABOLIC_PROCESS	-1.12	0.61	0	0	0	not significant
4568	REACTOME_ACTIVATION_OF_NMDA_RECEPTORS_AND_POSTSYNAPTIC_	-1.12	0.61	0	0	0	not significant
4569	CHIANG_LIVER_CANCER_SUBCLASS_PROLIFERATION_DN	-1.12	0.59	0	0	0	not significant
4570	DAUER_STAT3_TARGETS_DN	-1.12	0.59	0	0	0	not significant
4571	GO_ER_NUCLEUS_SIGNALING_PATHWAY	-1.12	0.59	0	0	0	not significant
4572	KYNG_DNA_DAMAGE_BY_UV	-1.12	0.59	0	0	0	not significant
4573	MARTINEZ_RESPONSE_TO_TRABECTEDIN	-1.12	0.59	0	0	0	not significant
4574	REACTOME_E3_UBIQUITIN_LIGASES_UBIQUITINATE_TARGET_PROTEINS	-1.12	0.59	0	0	0	not significant
4575	CHR1Q42	-1.12	0.58	0	0	0	not significant
4576	GO_REGULATION_OF_PROTEIN_EXPORT_FROM_NUCLEUS	-1.12	0.58	0	0	0	not significant
4577	SETLUR_PROSTATE_CANCER_TMPRSS2_ERG_FUSION_UP	-1.12	0.58	0	0	0	not significant
4578	YAO_TEMPORAL_RESPONSE_TO_PROGESTERONE_CLUSTER_16	-1.12	0.58	0	0	0	not significant
4579	CHR1Q25	-1.12	0.57	0	0	0	not significant
4580	ZHAN_MULTIPLE_MYELOMA_LB_UP	-1.12	0.57	0	0	0	not significant
4581	GO_CIRCADIAN_REGULATION_OF_GENE_EXPRESSION	-1.12	0.56	0	0	0	not significant
4582	GO_PHAGOCYTIC_VESICLE_MEMBRANE	-1.12	0.56	0	0	0	not significant
4583	GO_CYTOKINETIC_PROCESS	-1.12	0.55	0	0	0	not significant
4584	GO_E_BOX_BINDING	-1.12	0.55	0	0	0	not significant
4585	GO_NEGATIVE_REGULATION_OF_SMOOTHENED_SIGNALING_PATHWAY	-1.12	0.55	0	0	0	not significant
4586	GO_NUCLEOSIDE_DIPHOSPHATE_METABOLIC_PROCESS	-1.12	0.55	0	0	0	not significant
4587	GO_T_TUBULE	-1.12	0.55	0	0	0	not significant
4588	REACTOME_RHO_GTPASES_ACTIVATE_WASPS_AND_WAVES	-1.12	0.55	0	0	0	not significant
4589	BIOCARTA_PYK2_PATHWAY	-1.12	0.54	0	0	0	not significant
4590	GO_REGULATION_OF_COLLAGEN_BIOSYNTHETIC_PROCESS	-1.12	0.54	0	0	0	not significant
4591	HOWLIN_PUBERTAL_MAMMARY_GLAND	-1.12	0.54	0	0	0	not significant
4592	ZHAN_LATE_DIFFERENTIATION_GENES_UP	-1.12	0.54	0	0	0	not significant
4593	GO_ESTABLISHMENT_OF_MITOCHONDRION_LOCALIZATION	-1.12	0.53	0	0	0	not significant
4594	GO_INO80_TYPE_COMPLEX	-1.12	0.53	0	0	0	not significant
4595	JAZAG_TGFB1_SIGNALING_DN	-1.12	0.53	0	0	0	not significant
4596	LEE_LIVER_CANCER_MYC_DN	-1.12	0.53	0	0	0	not significant
4597	PID_ALPHA_SYNUCLEIN_PATHWAY	-1.12	0.53	0	0	0	not significant
4598	GO_MODIFIED_AMINO_ACID_TRANSPORT	-1.12	0.52	0	0	0	not significant
4599	PID_INSULIN_GLUCOSE_PATHWAY	-1.12	0.52	0	0	0	not significant
4600	GO_RESPONSE_TO_UV_B	-1.12	0.51	0	0	0	not significant
4601	GO_TOLL_LIKE_RECEPTOR_4_SIGNALING_PATHWAY	-1.12	0.51	0	0	0	not significant
4602	REACTOME_EXTENSION_OF_TELOMERES	-1.12	0.51	0	0	0	not significant
4603	GO_NEGATIVE_REGULATION_OF_CATION_CHANNEL_ACTIVITY	-1.12	0.50	0	0	0	not significant
4604	LEE_LIVER_CANCER	-1.12	0.50	0	0	0	not significant
4605	REACTOME_NEPHRIN_FAMILY_INTERACTIONS	-1.12	0.50	0	0	0	not significant
4606	ROETH_TERT_TARGETS_UP	-1.12	0.50	0	0	0	not significant
4607	GO_RESPONSE_TO_INSECTICIDE	-1.12	0.49	0	0	0	not significant
4608	REACTOME_NOTCH3_INTRACELLULAR_DOMAIN_REGULATES_TRANSCRI	-1.12	0.49	0	0	0	not significant
4609	GO_CLATHRIN_HEAVY_CHAIN_BINDING	-1.12	0.48	0	0	0	not significant
4610	GO_NEGATIVE_REGULATION_OF_NEURAL_PRECURSOR_CELL_PROLIFE	-1.12	0.48	0	0	0	not significant
4611	GO_NEGATIVE_REGULATION_OF_PROTEIN_EXPORT_FROM_NUCLEUS	-1.12	0.48	0	0	0	not significant
4612	GO_TYPE_B_PANCREATIC_CELL_APOPTOTIC_PROCESS	-1.12	0.48	0	0	0	not significant
4613	MATZUK_OVULATION	-1.12	0.48	0	0	0	not significant
4614	OXFORD_RALB_TARGETS_DN	-1.12	0.48	0	0	0	not significant
4615	REACTOME_SYNTHESIS_OF_PE	-1.12	0.48	0	0	0	not significant
4616	GO_CAMP_DEPENDENT_PROTEIN_KINASE_COMPLEX	-1.12	0.47	0	0	0	not significant
4617	GO_DNA_REPAIR_COMPLEX_ASSEMBLY	-1.12	0.47	0	0	0	not significant
4618	GO_GLUCOSE_CATABOLIC_PROCESS	-1.12	0.47	0	0	0	not significant
4619	GO_PRESYNAPTIC_ENDOCYTIC_ZONE	-1.12	0.47	0	0	0	not significant
4620	GO_REGULATION_OF_CELL_PROLIFERATION_INVOLVED_IN_KIDNEY_DE\	-1.12	0.47	0	0	0	not significant
4621	GO_NEGATIVE_REGULATION_OF_ESTABLISHMENT_OF_PROTEIN_LOCAL	-1.12	0.46	0	0	0	not significant
4622	NIKOLSKY_BREAST_CANCER_1Q32_AMPLICON	-1.12	0.46	0	0	0	not significant
4623	GO_METANEPHRIC_EPITHELIUM_DEVELOPMENT	-1.12	0.45	0	0	0	not significant
4624	GO_POSITIVE_REGULATION_OF_TOLL_LIKE_RECEPTOR_4_SIGNALING_F	-1.12	0.45	0	0	0	not significant
4625	MOOTHA_PYR	-1.12	0.45	0	0	0	not significant
4626	REACTOME_LRR_FLII_INTERACTING_PROTEIN_1_LRRFIP1_ACTIVATES_T	-1.12	0.45	0	0	0	not significant
4627	GO_INTRINSIC_APOPTOTIC_SIGNALING_PATHWAY_IN_RESPONSE_TO_H	-1.12	0.44	0	0	0	not significant
4628	GO_MODULATION_OF_SIGNAL_TRANSDUCTION_IN_OTHER_ORGANISM	-1.12	0.43	0	0	0	not significant
4629	VICENT_METASTASIS_UP	-1.12	0.43	0	0	0	not significant
4630	GO_CYTOSKELETON_ORGANIZATION	-1.11	1.36	0	0	0	not significant
4631	YOSHIMURA_MAPK8_TARGETS_UP	-1.11	1.26	0	0	0	not significant
4632	GO_CELLULAR_PROTEIN_CATABOLIC_PROCESS	-1.11	1.15	0	0	0	not significant
4633	GO_POSITIVE_REGULATION_OF_CELLULAR_COMPONENT_ORGANIZATIC	-1.11	1.11	0	0	0	not significant
4634	GO_ORGANONITROGEN_COMPOUND_CATABOLIC_PROCESS	-1.11	1.07	0	0	0	not significant
4635	GO_ANCHORING_JUNCTION	-1.11	0.90	0	0	0	not significant
4636	MARTINEZ_RB1_AND_TP53_TARGETS_DN	-1.11	0.87	0	0	0	not significant
4637	GO_NEGATIVE_REGULATION_OF_CELL_POPULATION_PROLIFERATION	-1.11	0.80	0	0	0	not significant
4638	GO_TRANSFERASE_COMPLEX_TRANSFERRING_PHOSPHORUS_CONTAIN	-1.11	0.79	0	0	0	not significant
4639	GO_POSITIVE_REGULATION_OF_CELL_ADHESION	-1.11	0.77	0	0	0	not significant
4640	REACTOME_ORGANELLE_BIOGENESIS_AND_MAINTENANCE	-1.11	0.76	0	0	0	not significant
4641	GO_POSITIVE_REGULATION_OF_CYTOKINE_PRODUCTION	-1.11	0.75	0	0	0	not significant
4642	THEILGAARD_NEUTROPHIL_AT_SKIN_WOUND_DN	-1.11	0.73	0	0	0	not significant
4643	GO_RESPONSE_TO_INSULIN	-1.11	0.72	0	0	0	not significant
4644	GO_COATED_VESICLE_MEMBRANE	-1.11	0.69	0	0	0	not significant
4645	GO_PRODUCTION_OF_MOLECULAR_MEDIATOR_OF_IMMUNE_RESPONSE	-1.11	0.68	0	0	0	not significant
4646	GO_PROTEIN_PROCESSING	-1.11	0.68	0	0	0	not significant
4647	AKT_UP_MTOR_DN.V1_UP	-1.11	0.67	0	0	0	not significant
4648	GO_MYELOID_LEUKOCYTE_DIFFERENTIATION	-1.11	0.67	0	0	0	not significant
4649	GO_POSITIVE_REGULATION_OF_BINDING	-1.11	0.67	0	0	0	not significant
4650	DAVICIONI_MOLECULAR_ARMS_VS_ERMS_DN	-1.11	0.66	0	0	0	not significant
4651	GO_BONE_DEVELOPMENT	-1.11	0.66	0	0	0	not significant
4652	GO_RESPONSE_TO_TOPOLOGICALLY_INCORRECT_PROTEIN	-1.11	0.66	0	0	0	not significant
4653	RAF_UP.V1_DN	-1.11	0.66	0	0	0	not significant
4654	BOQUEST_STEM_CELL_DN	-1.11	0.64	0	0	0	not significant
4655	GO_TOLL_LIKE_RECEPTOR_SIGNALING_PATHWAY	-1.11	0.64	0	0	0	not significant
4656	GAVIN_FOXP3_TARGETS_CLUSTER_T7	-1.11	0.62	0	0	0	not significant
4657	GO_COATED_MEMBRANE	-1.11	0.62	0	0	0	not significant
4658	SASSON_RESPONSE_TO_GONADOTROPHINS_UP	-1.11	0.62	0	0	0	not significant
4659	ACEVEDO_LIVER_CANCER_WITH_H3K27ME3_UP	-1.11	0.60	0	0	0	not significant
4660	ATF2_UP.V1_UP	-1.11	0.60	0	0	0	not significant
4661	GO_PEROXISOME_ORGANIZATION	-1.11	0.60	0	0	0	not significant
4662	GO_REGULATION_OF_PHOSPHOPROTEIN_PHOSPHATASE_ACTIVITY	-1.11	0.60	0	0	0	not significant
4663	MARKEY_RB1_CHRONIC_LOF_UP	-1.11	0.60	0	0	0	not significant
4664	PUJANA_BRCA_CENTERED_NETWORK	-1.11	0.60	0	0	0	not significant
4665	BANDRES_RESPONSE_TO_CARMUSTIN_MGMT_48HR_DN	-1.11	0.59	0	0	0	not significant
4666	NIKOLSKY_BREAST_CANCER_17Q11_Q21_AMPLICON	-1.11	0.58	0	0	0	not significant
4667	VANLOO_SP3_TARGETS_DN	-1.11	0.58	0	0	0	not significant
4668	CHARAFE_BREAST_CANCER_BASAL_VS_MESENCHYMAL_UP	-1.11	0.57	0	0	0	not significant
4669	GO_POSITIVE_REGULATION_OF_ADAPTIVE_IMMUNE_RESPONSE	-1.11	0.56	0	0	0	not significant
4670	GO_REGULATION_OF_CELL_MATRIX_ADHESION	-1.11	0.56	0	0	0	not significant
4671	KEGG_LEUKOCYTE_TRANSENDOTHELIAL_MIGRATION	-1.11	0.56	0	0	0	not significant
4672	KEGG_NUCLEOTIDE_EXCISION_REPAIR	-1.11	0.56	0	0	0	not significant
4673	KRAS.300_UP.V1_UP	-1.11	0.56	0	0	0	not significant
4674	REACTOME_GOLGI_ASSOCIATED_VESICLE_BIOGENESIS	-1.11	0.56	0	0	0	not significant
4675	KIM_BIPOLAR_DISORDER_OLIGODENDROCYTE_DENSITY_CORR_DN	-1.11	0.55	0	0	0	not significant

4676	GO_NEGATIVE_REGULATION_OF_RESPONSE_TO_OXIDATIVE_STRESS	-1.11	0.54	0	0	0	not significant
4677	GO_OLIGODENDROCYTE_DEVELOPMENT	-1.11	0.54	0	0	0	not significant
4678	SCHAEFFER_PROSTATE_DEVELOPMENT_12HR_UP	-1.11	0.54	0	0	0	not significant
4679	GO_MRNA_CLEAVAGE_FACTOR_COMPLEX	-1.11	0.53	0	0	0	not significant
4680	GO_POSITIVE_REGULATION_OF_RESPONSE_TO_ENDOPLASMIC_RETICU	-1.11	0.53	0	0	0	not significant
4681	GO_T_HELPER_1_TYPE_IMMUNE_RESPONSE	-1.11	0.53	0	0	0	not significant
4682	PID_MYC_REPRESS_PATHWAY	-1.11	0.53	0	0	0	not significant
4683	REACTOME_THE_PHOTOTRANSDUCTION_CASCADE	-1.11	0.53	0	0	0	not significant
4684	GO_POSITIVE_REGULATION_OF_WOUND_HEALING	-1.11	0.52	0	0	0	not significant
4685	GO_PROTEIN_INSERTION_INTO_MITOCHONDRIAL_MEMBRANE	-1.11	0.52	0	0	0	not significant
4686	GO_REGULATION_OF_GLIAL_CELL_DIFFERENTIATION	-1.11	0.52	0	0	0	not significant
4687	PID_S1P_S1P2_PATHWAY	-1.11	0.52	0	0	0	not significant
4688	WANG_LSD1_TARGETS_DN	-1.11	0.52	0	0	0	not significant
4689	GO_HISTONE_MRNA_METABOLIC_PROCESS	-1.11	0.51	0	0	0	not significant
4690	GO_NEGATIVE_REGULATION_OF_COAGULATION	-1.11	0.51	0	0	0	not significant
4691	GO_PEPTIDYL_METHIONINE_MODIFICATION	-1.11	0.51	0	0	0	not significant
4692	GO_PHOTORECEPTOR_CELL_MAINTENANCE	-1.11	0.51	0	0	0	not significant
4693	GO_POSITIVE_REGULATION_OF_SPROUTING_ANGIOGENESIS	-1.11	0.51	0	0	0	not significant
4694	LEIN_LOCALIZED_TO_PROXIMAL_DENDRITES	-1.11	0.51	0	0	0	not significant
4695	REACTOME_DISEASES_ASSOCIATED_WITH_N_GLYCOSYLATION_OF_PR(	-1.11	0.51	0	0	0	not significant
4696	AMIT_SERUM_RESPONSE_480_MCF10A	-1.11	0.50	0	0	0	not significant
4697	GO_L_AMINO_ACID_TRANSPORT	-1.11	0.50	0	0	0	not significant
4698	GO_NEGATIVE_REGULATION_OF_CELLULAR_RESPONSE_TO_INSULIN_S'	-1.11	0.50	0	0	0	not significant
4699	GO_REGULATION_OF_GRANULOCYTE_DIFFERENTIATION	-1.11	0.50	0	0	0	not significant
4700	KIM_LIVER_CANCER_POOR_SURVIVAL_DN	-1.11	0.50	0	0	0	not significant
4701	PID_MAPK_TRK_PATHWAY	-1.11	0.50	0	0	0	not significant
4702	REACTOME_REGULATION_OF_INNATE_IMMUNE_RESPONSES_TO_CYTOS	-1.11	0.50	0	0	0	not significant
4703	SESTO_RESPONSE_TO_UV_C6	-1.11	0.50	0	0	0	not significant
4704	GO_DOUBLE_STRANDED_TELOMERIC_DNA_BINDING	-1.11	0.49	0	0	0	not significant
4705	GO_GLUTAMATE_SECRETION	-1.11	0.49	0	0	0	not significant
4706	GO_REGULATION_OF_ER_ASSOCIATED_UBIQUITIN_DEPENDENT_PROTE	-1.11	0.49	0	0	0	not significant
4707	REACTOME_GLUTATHIONE_SYNTHESIS_AND_RECYCLING	-1.11	0.49	0	0	0	not significant
4708	YUAN_ZNF143_PARTNERS	-1.11	0.49	0	0	0	not significant
4709	FARMER_BREAST_CANCER_CLUSTER_3	-1.11	0.48	0	0	0	not significant
4710	GO_CHEMOKINE_RECEPTOR_BINDING	-1.11	0.48	0	0	0	not significant
4711	GO_POSITIVE_REGULATION_OF_TRANSCRIPTION_OF_NOTCH_RECEPTC	-1.11	0.48	0	0	0	not significant
4712	GO_RESPONSE_TO_NITRIC_OXIDE	-1.11	0.48	0	0	0	not significant
4713	GO_RETINOIC_ACID_RECEPTOR_BINDING	-1.11	0.48	0	0	0	not significant
4714	REACTOME_MOLYBDENUM_COFACTOR_BIOSYNTHESIS	-1.11	0.48	0	0	0	not significant
4715	REACTOME_RAP1_SIGNALLING	-1.11	0.48	0	0	0	not significant
4716	ZHANG_ANTIVIRAL_RESPONSE_TO_RIBAVIRIN_UP	-1.11	0.48	0	0	0	not significant
4717	BIOCARTA_STATHMIN_PATHWAY	-1.11	0.47	0	0	0	not significant
4718	GO_RESPONSE_TO_PH	-1.11	0.47	0	0	0	not significant
4719	REACTOME_METABOLISM_OF_PORPHYRINS	-1.11	0.47	0	0	0	not significant
4720	GO_GOLGI_TRANSPORT_COMPLEX	-1.11	0.46	0	0	0	not significant
4721	TOMLINS_METASTASIS_UP	-1.11	0.46	0	0	0	not significant
4722	CAIRO_HEPATOBLASTOMA_POOR_SURVIVAL	-1.11	0.45	0	0	0	not significant
4723	GO_CARDIAC_MUSCLE_FIBER_DEVELOPMENT	-1.11	0.45	0	0	0	not significant
4724	GO_GAS_HOMEOSTASIS	-1.11	0.45	0	0	0	not significant
4725	GO_GLOMERULAR_EPITHELIAL_CELL_DIFFERENTIATION	-1.11	0.45	0	0	0	not significant
4726	GO_INTERLEUKIN_2_SECRETION	-1.11	0.45	0	0	0	not significant
4727	GO_MIDGUT_DEVELOPMENT	-1.11	0.45	0	0	0	not significant
4728	GO_REGULATION_OF_CARDIAC_MUSCLE_CELL_MEMBRANE_REPOLARIZ	-1.11	0.45	0	0	0	not significant
4729	GO_INTERFERON_RECEPTOR_ACTIVITY	-1.11	0.44	0	0	0	not significant
4730	GO_MEIOSIS_II_CELL_CYCLE_PROCESS	-1.11	0.44	0	0	0	not significant
4731	GO_REGULATION_OF_RESPIRATORY_GASEOUS_EXCHANGE_BY_NEURO	-1.11	0.43	0	0	0	not significant
4732	LU_TUMOR_VASCULATURE_DN	-1.11	0.43	0	0	0	not significant
4733	GO_ROSTROCAUDAL_NEURAL_TUBE_PATTERNING	-1.11	0.42	0	0	0	not significant
4734	NUYTTEN_EZH2_TARGETS_DN	-1.10	1.52	0	0	0	not significant
4735	GO_PROTEIN_PHOSPHORYLATION	-1.10	1.30	0	0	0	not significant
4736	GO_SIGNALING_RECEPTOR_BINDING	-1.10	0.97	0	0	0	not significant
4737	IVANOVA_HEMATOPOIESIS_EARLY_PROGENITOR	-1.10	0.94	0	0	0	not significant
4738	GO_ORGANOPHOSPHATE_METABOLIC_PROCESS	-1.10	0.90	0	0	0	not significant
4739	GO_NEGATIVE_REGULATION_OF_DEVELOPMENTAL_PROCESS	-1.10	0.83	0	0	0	not significant
4740	GO_INFLAMMATORY_RESPONSE	-1.10	0.80	0	0	0	not significant
4741	BRUINS_UVC_RESPONSE_EARLY_LATE	-1.10	0.78	0	0	0	not significant
4742	MULLIGHAN_NPM1_SIGNATURE_3_UP	-1.10	0.76	0	0	0	not significant
4743	GO_LEUKOCYTE_CELL_CELL_ADHESION	-1.10	0.72	0	0	0	not significant
4744	GO_SENSORY_SYSTEM_DEVELOPMENT	-1.10	0.71	0	0	0	not significant
4745	REACTOME_ASPARAGINE_N_LINKED_GLYCOSYLATION	-1.10	0.71	0	0	0	not significant
4746	GO_LIPID_LOCALIZATION	-1.10	0.69	0	0	0	not significant
4747	GO_REPRODUCTIVE_SYSTEM_DEVELOPMENT	-1.10	0.68	0	0	0	not significant
4748	GO_STEROID_METABOLIC_PROCESS	-1.10	0.67	0	0	0	not significant
4749	PRC2_EZH2_UP.V1_DN	-1.10	0.67	0	0	0	not significant
4750	GARCIA_TARGETS_OF_FLI1_AND_DAX1_DN	-1.10	0.66	0	0	0	not significant
4751	GO_REGULATION_OF_MEMBRANE_POTENTIAL	-1.10	0.66	0	0	0	not significant
4752	HALLMARK_P53_PATHWAY	-1.10	0.65	0	0	0	not significant
4753	GO_ENDOCYTIC_VESICLE	-1.10	0.64	0	0	0	not significant
4754	GO_REGULATION_OF_INFLAMMATORY_RESPONSE	-1.10	0.64	0	0	0	not significant
4755	REACTOME_RRNA_PROCESSING_IN_THE_NUCLEUS_AND_CYTOSOL	-1.10	0.64	0	0	0	not significant
4756	GO_MEMBRANE_DOCKING	-1.10	0.63	0	0	0	not significant
4757	KEGG_FC_GAMMA_R_MEDIATED_PHAGOCYTOSIS	-1.10	0.63	0	0	0	not significant
4758	GO_CARBOHYDRATE_TRANSPORT	-1.10	0.62	0	0	0	not significant
4759	GO_ACTOMYOSIN_STRUCTURE_ORGANIZATION	-1.10	0.61	0	0	0	not significant
4760	GO_CYTOKINE_RECEPTOR_BINDING	-1.10	0.61	0	0	0	not significant
4761	GO_CYTOKINE_SECRETION	-1.10	0.60	0	0	0	not significant
4762	BROWNE_HCMV_INFECTION_12HR_UP	-1.10	0.58	0	0	0	not significant
4763	GO_BETA_CATENIN_BINDING	-1.10	0.58	0	0	0	not significant
4764	GO_POSITIVE_REGULATION_OF_DEVELOPMENTAL_GROWTH	-1.10	0.58	0	0	0	not significant
4765	GO_REGULATION_OF_SYNAPSE_STRUCTURE_OR_ACTIVITY	-1.10	0.58	0	0	0	not significant
4766	GO_VITAMIN_BINDING	-1.10	0.58	0	0	0	not significant
4767	ONDER_CDH1_SIGNALING_VIA_CTNNB1	-1.10	0.58	0	0	0	not significant
4768	GO_EXTRINSIC_APOPTOTIC_SIGNALING_PATHWAY_VIA_DEATH_DOMAIN	-1.10	0.56	0	0	0	not significant
4769	GO_POSITIVE_REGULATION_OF_PROTEIN_LOCALIZATION_TO_NUCLEUS	-1.10	0.55	0	0	0	not significant
4770	GO_4_IRON_4_SULFUR_CLUSTER_BINDING	-1.10	0.54	0	0	0	not significant
4771	GO_CELL_ADHESION_MEDIATOR_ACTIVITY	-1.10	0.54	0	0	0	not significant
4772	GO_FEMALE_SEX_DIFFERENTIATION	-1.10	0.54	0	0	0	not significant
4773	GO_GLIAL_CELL_DEVELOPMENT	-1.10	0.54	0	0	0	not significant
4774	GO_NEGATIVE_REGULATION_OF_CELL_MORPHOGENESIS_INVOLVED_IN	-1.10	0.54	0	0	0	not significant
4775	GO_POSITIVE_REGULATION_OF_CD4_POSITIVE_ALPHA_BETA_T_CELL_/	-1.10	0.54	0	0	0	not significant
4776	GO_CHONDROCYTE_DIFFERENTIATION	-1.10	0.53	0	0	0	not significant
4777	GO_CLATHRIN_COAT	-1.10	0.53	0	0	0	not significant
4778	GO_LIPID_DROPLET	-1.10	0.53	0	0	0	not significant
4779	GO_MUSCLE_ORGAN_MORPHOGENESIS	-1.10	0.53	0	0	0	not significant
4780	GO_PHOTORECEPTOR_OUTER_SEGMENT	-1.10	0.53	0	0	0	not significant
4781	HUMMERICH_SKIN_CANCER_PROGRESSION_DN	-1.10	0.53	0	0	0	not significant
4782	REACTOME_ERCC6_CSB_AND_EHMT2_G9A_POSITIVELY_REGULATE_RR	-1.10	0.53	0	0	0	not significant
4783	LIN_MELANOMA_COPY_NUMBER_DN	-1.10	0.52	0	0	0	not significant
4784	REACTOME_DEPURINATION	-1.10	0.52	0	0	0	not significant

4785	GO_NUCLEOTIDE_EXCISION_REPAIR_DNA_DAMAGE_RECOGNITION	-1.10	0.51	0	0	0	not significant
4786	GO_RRNA_METHYLATION	-1.10	0.51	0	0	0	not significant
4787	WEIGEL_OXIDATIVE_STRESS_RESPONSE	-1.10	0.51	0	0	0	not significant
4788	CHR7P21	-1.10	0.50	0	0	0	not significant
4789	REACTOME_ACTIVATION_OF_KAINATE_RECEPTORS_UPON_GLUTAMATE	-1.10	0.50	0	0	0	not significant
4790	CHR4Q28	-1.10	0.49	0	0	0	not significant
4791	GO_INTRACELLULAR_LIGAND_GATED_ION_CHANNEL_ACTIVITY	-1.10	0.49	0	0	0	not significant
4792	GO_POSITIVE_REGULATION_OF_VIRAL_RELEASE_FROM_HOST_CELL	-1.10	0.49	0	0	0	not significant
4793	GO_REGULATION_OF_TRANSCRIPTION_INITIATION_FROM_RNA_POLYME	-1.10	0.49	0	0	0	not significant
4794	GO_REGULATION_OF_VACUOLE_ORGANIZATION	-1.10	0.49	0	0	0	not significant
4795	GREENBAUM_E2A_TARGETS_DN	-1.10	0.49	0	0	0	not significant
4796	REACTOME_REGULATION_OF_BETA_CELL_DEVELOPMENT	-1.10	0.49	0	0	0	not significant
4797	WEIGEL_OXIDATIVE_STRESS_BY_TBH_AND_H2O2	-1.10	0.49	0	0	0	not significant
4798	GO_CYSTEINE_TYPE_ENDOPEPTIDASE_ACTIVATOR_ACTIVITY_INVOLVEI	-1.10	0.48	0	0	0	not significant
4799	GO_POSITIVE_REGULATION_OF_CAMP_MEDIATED_SIGNALING	-1.10	0.48	0	0	0	not significant
4800	GO_TELENCEPHALON_GLIAL_CELL_MIGRATION	-1.10	0.48	0	0	0	not significant
4801	TRAYNOR_RETT_SYNDROM_DN	-1.10	0.48	0	0	0	not significant
4802	VERNELL_RETINOBLASTOMA_PATHWAY_DN	-1.10	0.48	0	0	0	not significant
4803	BARRIER_CANCER_RELAPSE_TUMOR_SAMPLE_UP	-1.10	0.47	0	0	0	not significant
4804	CHR1Q43	-1.10	0.47	0	0	0	not significant
4805	GO_ADRENAL_GLAND_DEVELOPMENT	-1.10	0.47	0	0	0	not significant
4806	GO_ASTROCYTE_ACTIVATION	-1.10	0.47	0	0	0	not significant
4807	GO_CELL_CELL_SIGNALING_INVOLVED_IN_CARDIAC_CONDUCTION	-1.10	0.47	0	0	0	not significant
4808	GO_POLARIZED_EPITHELIAL_CELL_DIFFERENTIATION	-1.10	0.47	0	0	0	not significant
4809	GO_PROTEIN_FOLDING_CHAPERONE	-1.10	0.47	0	0	0	not significant
4810	GO_RESPONSE_TO_FOOD	-1.10	0.47	0	0	0	not significant
4811	TANG_SENESCENCE_TP53_TARGETS_UP	-1.10	0.47	0	0	0	not significant
4812	WALLACE_PROSTATE_CANCER_UP	-1.10	0.47	0	0	0	not significant
4813	BIOCARTA_EPONFKB_PATHWAY	-1.10	0.46	0	0	0	not significant
4814	GHO_ATF5_TARGETS_UP	-1.10	0.46	0	0	0	not significant
4815	GO_GLIAL_CELL_MIGRATION	-1.10	0.46	0	0	0	not significant
4816	GO_RESPONSE_TO_INACTIVITY	-1.10	0.46	0	0	0	not significant
4817	GO_RNA_CAP_BINDING	-1.10	0.46	0	0	0	not significant
4818	NELSON_RESPONSE_TO_ANDROGEN_DN	-1.10	0.46	0	0	0	not significant
4819	RAMPON_ENRICHED_LEARNING_ENVIRONMENT_EARLY_UP	-1.10	0.46	0	0	0	not significant
4820	GO_CARDIAC_CONDUCTION_SYSTEM_DEVELOPMENT	-1.10	0.45	0	0	0	not significant
4821	GO_MATING	-1.10	0.45	0	0	0	not significant
4822	GO_NUCLEOTIDE_TRANSMEMBRANE_TRANSPORTER_ACTIVITY	-1.10	0.45	0	0	0	not significant
4823	GO_REGULATION_OF_CAMP_DEPENDENT_PROTEIN_KINASE_ACTIVITY	-1.10	0.45	0	0	0	not significant
4824	CHR13Q31	-1.10	0.44	0	0	0	not significant
4825	GO_1_PHOSPHATIDYLINOSITOL_3_KINASE_REGULATOR_ACTIVITY	-1.10	0.44	0	0	0	not significant
4826	GO_2_OXOGLUTARATE_METABOLIC_PROCESS	-1.10	0.44	0	0	0	not significant
4827	GO_DICARBOXYLIC_ACID_TRANSMEMBRANE_TRANSPORTER_ACTIVITY	-1.10	0.44	0	0	0	not significant
4828	GO_DNA_UNWINDING_INVOLVED_IN_DNA_REPLICATION	-1.10	0.44	0	0	0	not significant
4829	GO_POSITIVE_REGULATION_OF_OLIGODENDROCYTE_DIFFERENTIATION	-1.10	0.44	0	0	0	not significant
4830	GO_POSITIVE_REGULATION_OF_PROTEIN_DEPOLYMERIZATION	-1.10	0.44	0	0	0	not significant
4831	GO_REGULATION_OF_HISTONE_H4_K16_ACETYLATION	-1.10	0.44	0	0	0	not significant
4832	GO_MODULATION_OF_AGE_RELATED_BEHAVIORAL_DECLINE	-1.10	0.43	0	0	0	not significant
4833	GO_REGULATION_OF_PSEUDOPODIUM_ASSEMBLY	-1.10	0.43	0	0	0	not significant
4834	LUI_THYROID_CANCER_CLUSTER_5	-1.10	0.42	0	0	0	not significant
4835	GO_DENSE_CORE_GRANULE_TRANSPORT	-1.10	0.40	0	0	0	not significant
4836	GO_CYTOSKELETAL_PART	-1.09	1.27	0	0	0	not significant
4837	GO_CHROMOSOME	-1.09	1.05	0	0	0	not significant
4838	GO_MODIFICATION_DEPENDENT_MACROMOLECULE_CATABOLIC_PROCI	-1.09	0.92	0	0	0	not significant
4839	HSIAO_HOUSEKEEPING_GENES	-1.09	0.82	0	0	0	not significant
4840	MARTORIATI_MDM4_TARGETS_FETAL_LIVER_DN	-1.09	0.77	0	0	0	not significant
4841	GO_ENDOCYTOSIS	-1.09	0.76	0	0	0	not significant
4842	DELACROIX_RAR_BOUND_ES	-1.09	0.75	0	0	0	not significant
4843	FISCHER_DIRECT_P53_TARGETS_META_ANALYSIS	-1.09	0.73	0	0	0	not significant
4844	GO_POSITIVE_REGULATION_OF_CELLULAR_COMPONENT_BIOGENESIS	-1.09	0.73	0	0	0	not significant
4845	GO_MAINTENANCE_OF_LOCATION	-1.09	0.69	0	0	0	not significant
4846	GO_REGULATION_OF_HORMONE_LEVELS	-1.09	0.68	0	0	0	not significant
4847	GO_CHROMOSOME_TELOMERIC_REGION	-1.09	0.64	0	0	0	not significant
4848	CHR1P34	-1.09	0.61	0	0	0	not significant
4849	GO_ORGANIC_ANION_TRANSMEMBRANE_TRANSPORTER_ACTIVITY	-1.09	0.61	0	0	0	not significant
4850	SENESE_HDAC1_AND_HDAC2_TARGETS_UP	-1.09	0.61	0	0	0	not significant
4851	KAAB_HEART_ATRIUM_VS_VENTRICLE_UP	-1.09	0.59	0	0	0	not significant
4852	NOTCH_DN.V1_DN	-1.09	0.58	0	0	0	not significant
4853	GO_RESPONSE_TO_HYDROGEN_PEROXIDE	-1.09	0.55	0	0	0	not significant
4854	GO_RNA_HELICASE_ACTIVITY	-1.09	0.55	0	0	0	not significant
4855	REACTOME_TRANSCRIPTIONAL_REGULATION_OF_GRANULOPOIESIS	-1.09	0.55	0	0	0	not significant
4856	KEGG_NATURAL_KILLER_CELL_MEDIATED_CYTOTOXICITY	-1.09	0.54	0	0	0	not significant
4857	CYCLIN_D1_KE_.V1_DN	-1.09	0.53	0	0	0	not significant
4858	GO_POSITIVE_REGULATION_OF_ENDOTHELIAL_CELL_MIGRATION	-1.09	0.53	0	0	0	not significant
4859	GO_POSITIVE_REGULATION_OF_MYELOID_LEUKOCYTE_DIFFERENTIATIC	-1.09	0.52	0	0	0	not significant
4860	GO_REGULATION_OF_HEART_RATE	-1.09	0.52	0	0	0	not significant
4861	GO_REGULATION_OF_RELEASE_OF_SEQUESTERED_CALCIUM_ION_INTC	-1.09	0.52	0	0	0	not significant
4862	SESTO_RESPONSE_TO_UV_C5	-1.09	0.52	0	0	0	not significant
4863	CHR15Q25	-1.09	0.51	0	0	0	not significant
4864	GO_NLS_BEARING_PROTEIN_IMPORT_INTO_NUCLEUS	-1.09	0.51	0	0	0	not significant
4865	GO_SENSORY_PERCEPTION_OF_MECHANICAL_STIMULUS	-1.09	0.51	0	0	0	not significant
4866	HALLMARK_COAGULATION	-1.09	0.51	0	0	0	not significant
4867	CHR5Q13	-1.09	0.50	0	0	0	not significant
4868	GO_MRNA_CLEAVAGE	-1.09	0.50	0	0	0	not significant
4869	GO_POSITIVE_REGULATION_OF_SMOOTH_MUSCLE_CELL_PROLIFERATI	-1.09	0.50	0	0	0	not significant
4870	GO_RIBONUCLEOSIDE_TRIPHOSPHATE_BIOSYNTHETIC_PROCESS	-1.09	0.50	0	0	0	not significant
4871	GO_CARBOHYDRATE_DERIVATIVE_TRANSPORT	-1.09	0.49	0	0	0	not significant
4872	GO_ENDOSOME_LUMEN	-1.09	0.49	0	0	0	not significant
4873	GO_INTERLEUKIN_1_SECRETION	-1.09	0.49	0	0	0	not significant
4874	GO_MULTICELLULAR_ORGANISMAL_MOVEMENT	-1.09	0.49	0	0	0	not significant
4875	GO_NEGATIVE_REGULATION_OF_EPITHELIAL_CELL_MIGRATION	-1.09	0.49	0	0	0	not significant
4876	GO_SULFUR_COMPOUND_TRANSMEMBRANE_TRANSPORTER_ACTIVITY	-1.09	0.49	0	0	0	not significant
4877	JECHLINGER_EPITHELIAL_TO_MESENCHYMAL_TRANSITION_DN	-1.09	0.49	0	0	0	not significant
4878	LANDIS_BREAST_CANCER_PROGRESSION_DN	-1.09	0.49	0	0	0	not significant
4879	BANDRES_RESPONSE_TO_CARMUSTIN_MGMT_24HR_DN	-1.09	0.48	0	0	0	not significant
4880	GO_ANION_HOMEOSTASIS	-1.09	0.48	0	0	0	not significant
4881	GO_ISOPRENOID_BIOSYNTHETIC_PROCESS	-1.09	0.48	0	0	0	not significant
4882	GO_PRIMARY_ALCOHOL_METABOLIC_PROCESS	-1.09	0.48	0	0	0	not significant
4883	GO_REGULATION_OF_GOLGI_ORGANIZATION	-1.09	0.48	0	0	0	not significant
4884	GO_AMINO_SUGAR_METABOLIC_PROCESS	-1.09	0.47	0	0	0	not significant
4885	GO_NADPH_BINDING	-1.09	0.47	0	0	0	not significant
4886	GO_RESPONSE_TO_ARSENIC_CONTAINING_SUBSTANCE	-1.09	0.47	0	0	0	not significant
4887	GO_THIOLESTER_HYDROLASE_ACTIVITY	-1.09	0.47	0	0	0	not significant
4888	HAHTOLA_CTCL_CUTANEOUS	-1.09	0.47	0	0	0	not significant
4889	GO_AMYLOID_FIBRIL_FORMATION	-1.09	0.46	0	0	0	not significant
4890	GO_NEGATIVE_REGULATION_OF_CD4_POSITIVE_ALPHA_BETA_T_CELL_	-1.09	0.46	0	0	0	not significant
4891	GO_NUCLEOTIDE_TRANSPORT	-1.09	0.46	0	0	0	not significant
4892	TERAMOTO_OPN_TARGETS_CLUSTER_7	-1.09	0.46	0	0	0	not significant
4893	BIOCARTA_IL17_PATHWAY	-1.09	0.45	0	0	0	not significant

4894	BIOCARTA_TRKA_PATHWAY	-1.09	0.45	0	0	0	not significant
4895	GO_FATTY_ACID_ELONGATION	-1.09	0.45	0	0	0	not significant
4896	GO_NEGATIVE_REGULATION_OF_PROTEIN_LOCALIZATION_TO_NUCLEUS	-1.09	0.45	0	0	0	not significant
4897	GO_STRIATED_MUSCLE_CELL_APOPTOTIC_PROCESS	-1.09	0.45	0	0	0	not significant
4898	GO_ANTIGEN_PROCESSING_AND_PRESENTATION_OF_EXOGENOUS_PEF	-1.09	0.44	0	0	0	not significant
4899	GO_MODULATION_BY_HOST_OF_VIRAL_GENOME_REPLICATION	-1.09	0.44	0	0	0	not significant
4900	GO_NADP_RETINOL_DEHYDROGENASE_ACTIVITY	-1.09	0.44	0	0	0	not significant
4901	GO_NEGATIVE_REGULATION_OF_ATPASE_ACTIVITY	-1.09	0.44	0	0	0	not significant
4902	RICKMAN_HEAD_AND_NECK_CANCER_E	-1.09	0.44	0	0	0	not significant
4903	GO_ACROSOME_ASSEMBLY	-1.09	0.43	0	0	0	not significant
4904	GO_CAMP_DEPENDENT_PROTEIN_KINASE_ACTIVITY	-1.09	0.43	0	0	0	not significant
4905	GO_MITOCHONDRIAL_RIBOSOME_ASSEMBLY	-1.09	0.43	0	0	0	not significant
4906	GO_MYOFILAMENT	-1.09	0.43	0	0	0	not significant
4907	GO_TELENCEPHALON_REGIONALIZATION	-1.09	0.43	0	0	0	not significant
4908	REACTOME_ACETYLCHOLINE_NEUROTRANSMITTER_RELEASE_CYCLE	-1.09	0.43	0	0	0	not significant
4909	REACTOME_RHO_GTPASES_ACTIVATE_KTN1	-1.09	0.43	0	0	0	not significant
4910	REACTOME_SLBP_DEPENDENT_PROCESSING_OF_REPLICATION_DEPEN	-1.09	0.43	0	0	0	not significant
4911	WILLIAMS_ESR2_TARGETS_UP	-1.09	0.43	0	0	0	not significant
4912	GO_PEPTIDYL_HISTIDINE_MODIFICATION	-1.09	0.42	0	0	0	not significant
4913	GO_SERINE_TYPE_CARBOXYPEPTIDASE_ACTIVITY	-1.09	0.42	0	0	0	not significant
4914	NIELSEN_LEIOMYOSARCOMA_CNN1_UP	-1.09	0.42	0	0	0	not significant
4915	GO_POSTSYNAPTIC_SIGNAL_TRANSDUCTION	-1.09	0.41	0	0	0	not significant
4916	GO_PERIPHERAL_NERVOUS_SYSTEM_MYELIN_MAINTENANCE	-1.09	0.40	0	0	0	not significant
4917	GO_EXTRACELLULAR_MATRIX_CONSTITUENT_SECRETION	-1.09	0.39	0	0	0	not significant
4918	DACOSTA_UV_RESPONSE_VIA_ERCC3_DN	-1.08	1.01	0	0	0	not significant
4919	REACTOME_DEVELOPMENTAL_BIOLOGY	-1.08	0.88	0	0	0	not significant
4920	GO_RESPONSE_TO_GROWTH_FACTOR	-1.08	0.84	0	0	0	not significant
4921	GO_TRANSPORTER_ACTIVITY	-1.08	0.83	0	0	0	not significant
4922	GO_APOPTOTIC_SIGNALING_PATHWAY	-1.08	0.76	0	0	0	not significant
4923	GO_CELL_CELL_ADHESION	-1.08	0.72	0	0	0	not significant
4924	GO_NEGATIVE_REGULATION_OF_HYDROLASE_ACTIVITY	-1.08	0.68	0	0	0	not significant
4925	SANSOM_APC_TARGETS_DN	-1.08	0.68	0	0	0	not significant
4926	GO_NEGATIVE_REGULATION_OF_APOPTOTIC_SIGNALING_PATHWAY	-1.08	0.65	0	0	0	not significant
4927	GO_NEGATIVE_REGULATION_OF_LOCOMOTION	-1.08	0.64	0	0	0	not significant
4928	GO_NEGATIVE_REGULATION_OF_TRANSFERASE_ACTIVITY	-1.08	0.63	0	0	0	not significant
4929	GO_MONOVALENT_INORGANIC_CATION_TRANSMEMBRANE_TRANSPORT	-1.08	0.62	0	0	0	not significant
4930	GO_REGULATION_OF_PROTEIN_MODIFICATION_BY_SMALL_PROTEIN_CO	-1.08	0.60	0	0	0	not significant
4931	SANSOM_APC_MYC_TARGETS	-1.08	0.60	0	0	0	not significant
4932	GO_RESPONSE_TO_IONIZING_RADIATION	-1.08	0.59	0	0	0	not significant
4933	GCNP_SHH_UP_LATE.V1_DN	-1.08	0.57	0	0	0	not significant
4934	GO_KINASE_REGULATOR_ACTIVITY	-1.08	0.57	0	0	0	not significant
4935	MANALO_HYPOXIA_UP	-1.08	0.55	0	0	0	not significant
4936	REACTOME_NEUROTRANSMITTER_RECEPTORS_AND_POSTSYNAPTIC_S	-1.08	0.55	0	0	0	not significant
4937	GO_PROTEIN_DNA_COMPLEX	-1.08	0.54	0	0	0	not significant
4938	GRABARCZYK_BCL11B_TARGETS_DN	-1.08	0.54	0	0	0	not significant
4939	JAEGER_METASTASIS_DN	-1.08	0.54	0	0	0	not significant
4940	NAKAMURA_ADIPOGENESIS_LATE_UP	-1.08	0.54	0	0	0	not significant
4941	REACTOME_SLC_TRANSPORTER_DISORDERS	-1.08	0.54	0	0	0	not significant
4942	AKL_HTLV1_INFECTION_DN	-1.08	0.53	0	0	0	not significant
4943	PURBEY_TARGETS_OF_CTBP1_AND_SATB1_UP	-1.08	0.53	0	0	0	not significant
4944	AMUNDSON_GENOTOXIC_SIGNATURE	-1.08	0.52	0	0	0	not significant
4945	GO_RIBOSOME_ASSEMBLY	-1.08	0.52	0	0	0	not significant
4946	GO_HORMONE_METABOLIC_PROCESS	-1.08	0.51	0	0	0	not significant
4947	GO_PLASMA_MEMBRANE_ORGANIZATION	-1.08	0.51	0	0	0	not significant
4948	GO_POSITIVE_REGULATION_OF_ERK1_AND_ERK2_CASCADE	-1.08	0.51	0	0	0	not significant
4949	GO_MULTI_ORGANISM_CELLULAR_PROCESS	-1.08	0.50	0	0	0	not significant
4950	GO_MYELOID_LEUKOCYTE_MIGRATION	-1.08	0.50	0	0	0	not significant
4951	GO_POSITIVE_REGULATION_OF_DNA_BIOSYNTHETIC_PROCESS	-1.08	0.50	0	0	0	not significant
4952	GO_REGULATION_OF_AUTOPHAGOSOME_ASSEMBLY	-1.08	0.50	0	0	0	not significant
4953	HELLEBREKERS_SILENCED_DURING_TUMOR_ANGIOGENESIS	-1.08	0.50	0	0	0	not significant
4954	REACTOME_MTOR_SIGNALLING	-1.08	0.50	0	0	0	not significant
4955	REACTOME_TGF_BETA_RECEPTOR_SIGNALING_ACTIVATES_SMADS	-1.08	0.50	0	0	0	not significant
4956	CHR2Q35	-1.08	0.49	0	0	0	not significant
4957	GO_ENDORIBONUCLEASE_ACTIVITY_PRODUCING_5_PHOSPHOMONOEST	-1.08	0.49	0	0	0	not significant
4958	GO_NEGATIVE_REGULATION_OF_INTRINSIC_APOPTOTIC_SIGNALING_PA	-1.08	0.49	0	0	0	not significant
4959	GO_POSITIVE_REGULATION_OF_CYCLIN_DEPENDENT_PROTEIN_KINASE_	-1.08	0.49	0	0	0	not significant
4960	GO_CYSTEINE_TYPE_ENDOPEPTIDASE_REGULATOR_ACTIVITY_INVOLVE	-1.08	0.48	0	0	0	not significant
4961	GO_EXTRACELLULAR_MATRIX_DISASSEMBLY	-1.08	0.48	0	0	0	not significant
4962	GO_INTERLEUKIN_1_BETA_SECRETION	-1.08	0.48	0	0	0	not significant
4963	LEE_LIVER_CANCER_DENA_DN	-1.08	0.48	0	0	0	not significant
4964	SHAFFER_IRF4_TARGETS_IN_ACTIVATED_DENDRITIC_CELL	-1.08	0.48	0	0	0	not significant
4965	GO_CARBOXY_LYASE_ACTIVITY	-1.08	0.47	0	0	0	not significant
4966	GO_CELL_CELL_ADHESION_MEDIATOR_ACTIVITY	-1.08	0.47	0	0	0	not significant
4967	GO_DEFENSE_RESPONSE_TO_GRAM_POSITIVE_BACTERIUM	-1.08	0.47	0	0	0	not significant
4968	GO_REGULATION_OF_KERATINOCYTE_DIFFERENTIATION	-1.08	0.47	0	0	0	not significant
4969	LEE_METASTASIS_AND_ALTERNATIVE_SPLICING_DN	-1.08	0.47	0	0	0	not significant
4970	REACTOME_GLYOXYLATE_METABOLISM_AND_GLYCINE_DEGRADATION	-1.08	0.47	0	0	0	not significant
4971	YU_BAP1_TARGETS	-1.08	0.47	0	0	0	not significant
4972	BIOCARTA_STRESS_PATHWAY	-1.08	0.46	0	0	0	not significant
4973	GO_MATERNAL_PLACENTA_DEVELOPMENT	-1.08	0.46	0	0	0	not significant
4974	GO_NEGATIVE_REGULATION_OF_CALCIUM_ION_TRANSMEMBRANE_TRAI	-1.08	0.46	0	0	0	not significant
4975	GO_OXIDATIVE_DEMETHYLATION	-1.08	0.46	0	0	0	not significant
4976	MYLLYKANGAS_AMPLIFICATION_HOT_SPOT_17	-1.08	0.46	0	0	0	not significant
4977	REACTOME_PEPTIDE_HORMONE_METABOLISM	-1.08	0.46	0	0	0	not significant
4978	GO_CEREBELLAR_PURKINJE_CELL_LAYER_DEVELOPMENT	-1.08	0.45	0	0	0	not significant
4979	GO_RNA_INTERFERENCE	-1.08	0.45	0	0	0	not significant
4980	GO_TELOMERE_CAP_COMPLEX	-1.08	0.45	0	0	0	not significant
4981	REACTOME_MTORC1_MEDIATED_SIGNALLING	-1.08	0.45	0	0	0	not significant
4982	REACTOME_PHOSPHORYLATION_OF_CD3_AND_TCR_ZETA_CHAINS	-1.08	0.45	0	0	0	not significant
4983	CAVARD_LIVER_CANCER_MALIGNANT_VS_BENIGN	-1.08	0.44	0	0	0	not significant
4984	GO_CILIARY_TRANSITION_FIBER	-1.08	0.44	0	0	0	not significant
4985	GO_POSITIVE_REGULATION_OF_TRANSCRIPTION_FROM_RNA_POLYMEF	-1.08	0.44	0	0	0	not significant
4986	GO_REGULATION_OF_CELL_GROWTH_INVOLVED_IN_CARDIAC_MUSCLE	-1.08	0.44	0	0	0	not significant
4987	KIM_GLIS2_TARGETS_UP	-1.08	0.44	0	0	0	not significant
4988	REACTOME_PLATELET_SENSITIZATION_BY_LDL	-1.08	0.44	0	0	0	not significant
4989	WEST_ADRENOCORTICAL_CARCINOMA_VS_ADENOMA_UP	-1.08	0.44	0	0	0	not significant
4990	GO_EPITHELIAL_CELL_MATURATION	-1.08	0.43	0	0	0	not significant
4991	GO_RETINOID_X_RECEPTOR_BINDING	-1.08	0.43	0	0	0	not significant
4992	GO_SAGA_COMPLEX	-1.08	0.43	0	0	0	not significant
4993	GO_U7_SNRNP	-1.08	0.43	0	0	0	not significant
4994	REACTOME_REGULATED_NECROSIS	-1.08	0.43	0	0	0	not significant
4995	GO_CYCLIC_NUCLEOTIDE_DEPENDENT_PROTEIN_KINASE_ACTIVITY	-1.08	0.42	0	0	0	not significant
4996	GO_NEDD8_SPECIFIC_PROTEASE_ACTIVITY	-1.08	0.42	0	0	0	not significant
4997	GO_PLATELET_DENSE_TUBULAR_NETWORK	-1.08	0.42	0	0	0	not significant
4998	GO_RECEPTOR_RECYCLING	-1.08	0.42	0	0	0	not significant
4999	REACTOME_ESTROGEN_STIMULATED_SIGNALING_THROUGH_PRKCZ	-1.08	0.42	0	0	0	not significant
5000	GO_POSITIVE_REGULATION_OF_GLIAL_CELL_MIGRATION	-1.08	0.41	0	0	0	not significant
5001	HASINA_NOL7_TARGETS_DN	-1.08	0.41	0	0	0	not significant
5002	GO_GALACTOSE_CATABOLIC_PROCESS	-1.08	0.40	0	0	0	not significant

5003	GO_POSITIVE_REGULATION_OF_CHEMOKINE_C_X_C_MOTIF_LIGAND_2_	-1.08	0.40	0	0	0	not significant
5004	REACTOME_RUNX1_REGULATES_TRANSCRIPTION_OF_GENES_INVOLVEI	-1.08	0.40	0	0	0	not significant
5005	GO_REGULATION_OF_KETONE_BIOSYNTHETIC_PROCESS	-1.08	0.39	0	0	0	not significant
5006	BYSTRYKH_HEMATOPOIESIS_STEM_CELL_QTL_TRANS	-1.07	0.81	0	0	0	not significant
5007	GO_NUCLEAR_CHROMOSOME	-1.07	0.74	0	0	0	not significant
5008	BAELDE_DIABETIC_NEPHROPATHY_DN	-1.07	0.70	0	0	0	not significant
5009	SCHAEFFER_PROSTATE_DEVELOPMENT_6HR_DN	-1.07	0.67	0	0	0	not significant
5010	SENESE_HDAC3_TARGETS_DN	-1.07	0.63	0	0	0	not significant
5011	GO_REGULATION_OF_DNA_METABOLIC_PROCESS	-1.07	0.59	0	0	0	not significant
5012	GO_CELLULAR_RESPONSE_TO_PEPTIDE_HORMONE_STIMULUS	-1.07	0.58	0	0	0	not significant
5013	GO_NUCLEOSIDE_TRIPHOSPHATASE_REGULATOR_ACTIVITY	-1.07	0.58	0	0	0	not significant
5014	MOREAUX_MULTIPLE_MYELOMA_BY_TACI_UP	-1.07	0.58	0	0	0	not significant
5015	GO_ENDOPEPTIDASE_ACTIVITY	-1.07	0.57	0	0	0	not significant
5016	GO_NEGATIVE_REGULATION_OF_CELLULAR_CATABOLIC_PROCESS	-1.07	0.56	0	0	0	not significant
5017	GO_POSITIVE_REGULATION_OF_DNA_METABOLIC_PROCESS	-1.07	0.56	0	0	0	not significant
5018	ERBB2_UP.V1_UP	-1.07	0.55	0	0	0	not significant
5019	GO_NEGATIVE_REGULATION_OF_CELL_DEVELOPMENT	-1.07	0.55	0	0	0	not significant
5020	REACTOME_TRANSMISSION_ACROSS_CHEMICAL_SYNAPSES	-1.07	0.55	0	0	0	not significant
5021	GO_CELL_CORTEX_PART	-1.07	0.54	0	0	0	not significant
5022	HALLMARK_EPITHELIAL_MESENCHYMAL_TRANSITION	-1.07	0.53	0	0	0	not significant
5023	VILLANUEVA_LIVER_CANCER_KRT19_UP	-1.07	0.53	0	0	0	not significant
5024	GO_CELL_KILLING	-1.07	0.52	0	0	0	not significant
5025	GO_LARGE_RIBOSOMAL_SUBUNIT	-1.07	0.52	0	0	0	not significant
5026	IKEDA_MIR133_TARGETS_UP	-1.07	0.52	0	0	0	not significant
5027	CHR2Q31	-1.07	0.51	0	0	0	not significant
5028	GO_AMINO_ACID_TRANSMEMBRANE_TRANSPORT	-1.07	0.51	0	0	0	not significant
5029	GO_NEGATIVE_REGULATION_OF_TRANSMEMBRANE_TRANSPORT	-1.07	0.51	0	0	0	not significant
5030	TAKEDA_TARGETS_OF_NUP98_HOXA9_FUSION_8D_DN	-1.07	0.51	0	0	0	not significant
5031	GO_ASTROCYTE_DIFFERENTIATION	-1.07	0.50	0	0	0	not significant
5032	GO_DRUG_TRANSPORT	-1.07	0.50	0	0	0	not significant
5033	KEGG_APOPTOSIS	-1.07	0.50	0	0	0	not significant
5034	GO_PHOSPHOLIPASE_ACTIVITY	-1.07	0.49	0	0	0	not significant
5035	GO_RESPONSE_TO_VITAMIN	-1.07	0.49	0	0	0	not significant
5036	KOKKINAKIS_METHIONINE_DEPRIVATION_96HR_DN	-1.07	0.49	0	0	0	not significant
5037	HINATA_NFKB_TARGETS_FIBROBLAST_UP	-1.07	0.48	0	0	0	not significant
5038	KRAS.KIDNEY_UP.V1_DN	-1.07	0.48	0	0	0	not significant
5039	REACTOME_SYNTHESIS_OF_PIPS_AT_THE_PLASMA_MEMBRANE	-1.07	0.48	0	0	0	not significant
5040	GO_BRANCHING_MORPHOGENESIS_OF_AN_EPITHELIAL_TUBE	-1.07	0.47	0	0	0	not significant
5041	GO_NEUROINFLAMMATORY_RESPONSE	-1.07	0.47	0	0	0	not significant
5042	GO_POSITIVE_REGULATION_OF_MUSCLE_TISSUE_DEVELOPMENT	-1.07	0.47	0	0	0	not significant
5043	GO_POSITIVE_REGULATION_OF_STRESS_FIBER_ASSEMBLY	-1.07	0.47	0	0	0	not significant
5044	BIOCARTA_AT1R_PATHWAY	-1.07	0.46	0	0	0	not significant
5045	CHR1P35	-1.07	0.46	0	0	0	not significant
5046	GO_GRANULOCYTE_MIGRATION	-1.07	0.46	0	0	0	not significant
5047	GO_PHOSPHOTRANSFERASE_ACTIVITY_PHOSPHATE_GROUP_AS_ACCEI	-1.07	0.46	0	0	0	not significant
5048	GO_PROGRAMMED_NECROTIC_CELL_DEATH	-1.07	0.46	0	0	0	not significant
5049	GO_REGULATION_OF_LYMPHOCYTE_APOPTOTIC_PROCESS	-1.07	0.46	0	0	0	not significant
5050	LEE_LIVER_CANCER_MYC_UP	-1.07	0.46	0	0	0	not significant
5051	LEIN_MEDULLA_MARKERS	-1.07	0.46	0	0	0	not significant
5052	REACTOME_EXTRA_NUCLEAR_ESTROGEN_SIGNALING	-1.07	0.46	0	0	0	not significant
5053	ZHAN_MULTIPLE_MYELOMA_HP_DN	-1.07	0.46	0	0	0	not significant
5054	GO_POSITIVE_REGULATION_OF_RESPONSE_TO_WOUNDING	-1.07	0.45	0	0	0	not significant
5055	PID_ANTHRAX_PATHWAY	-1.07	0.45	0	0	0	not significant
5056	REACTOME_MAPK_TARGETS_NUCLEAR_EVENTS_MEDIATED_BY_MAP_K	-1.07	0.45	0	0	0	not significant
5057	TOMIDA_METASTASIS_DN	-1.07	0.45	0	0	0	not significant
5058	GO_CCR4_NOT_COMPLEX	-1.07	0.44	0	0	0	not significant
5059	GO_PEPTIDASE_ACTIVATOR_ACTIVITY_INVOLVED_IN_APOPTOTIC_PROC	-1.07	0.44	0	0	0	not significant
5060	GO_PERK_MEDIATED_UNFOLDED_PROTEIN_RESPONSE	-1.07	0.44	0	0	0	not significant
5061	GO_REGULATION_OF_SKELETAL_MUSCLE_TISSUE_DEVELOPMENT	-1.07	0.44	0	0	0	not significant
5062	REACTOME_APOPTOSIS_INDUCED_DNA_FRAGMENTATION	-1.07	0.44	0	0	0	not significant
5063	REACTOME_PLATELET_AGGREGATION_PLUG_FORMATION	-1.07	0.44	0	0	0	not significant
5064	REACTOME_UPTAKE_AND_FUNCTION_OF_ANTHRAX_TOXINS	-1.07	0.44	0	0	0	not significant
5065	XU_HGF_SIGNALING_NOT_VIA_AKT1_6HR	-1.07	0.44	0	0	0	not significant
5066	CHIN_BREAST_CANCER_COPY_NUMBER_UP	-1.07	0.43	0	0	0	not significant
5067	GO_LUNG_MORPHOGENESIS	-1.07	0.43	0	0	0	not significant
5068	GO_NEGATIVE_REGULATION_OF_CELL_CYCLE_ARREST	-1.07	0.43	0	0	0	not significant
5069	GO_REGULATION_OF_TRANSLATION_IN_RESPONSE_TO_STRESS	-1.07	0.43	0	0	0	not significant
5070	GO_SNRNA_BINDING	-1.07	0.43	0	0	0	not significant
5071	STREICHER_LSM1_TARGETS_DN	-1.07	0.43	0	0	0	not significant
5072	ALONSO_METASTASIS_NEURAL_UP	-1.07	0.42	0	0	0	not significant
5073	BIOCARTA_CDC42RAC_PATHWAY	-1.07	0.42	0	0	0	not significant
5074	GO_ANTIGEN_PROCESSING_AND_PRESENTATION_OF_ENDOGENOUS_PE	-1.07	0.42	0	0	0	not significant
5075	GO_GMP_METABOLIC_PROCESS	-1.07	0.42	0	0	0	not significant
5076	GO_GOLGI_TO_LYSOSOME_TRANSPORT	-1.07	0.42	0	0	0	not significant
5077	GO_NEUROTROPHIN_TRK_RECEPTOR_SIGNALING_PATHWAY	-1.07	0.42	0	0	0	not significant
5078	GO_PEPTIDYL_DIPHTHAMIDE_BIOSYNTHETIC_PROCESS_FROM_PEPTIDY	-1.07	0.42	0	0	0	not significant
5079	GUTIERREZ_WALDENSTROEMS_MACROGLOBULINEMIA_1_UP	-1.07	0.42	0	0	0	not significant
5080	PARK_HSC_VS_MULTIPOTENT_PROGENITORS_UP	-1.07	0.42	0	0	0	not significant
5081	REACTOME_SHC_MEDIATED_CASCADE:FGFR3	-1.07	0.42	0	0	0	not significant
5082	VIETOR_IFRD1_TARGETS	-1.07	0.42	0	0	0	not significant
5083	GO_CELLULAR_RESPONSE_TO_EPINEPHRINE_STIMULUS	-1.07	0.41	0	0	0	not significant
5084	GO_CORNIFIED_ENVELOPE	-1.07	0.41	0	0	0	not significant
5085	GO_NONHOMOLOGOUS_END_JOINING_COMPLEX	-1.07	0.41	0	0	0	not significant
5086	GO_CHLORIDE_CHANNEL_INHIBITOR_ACTIVITY	-1.07	0.40	0	0	0	not significant
5087	GO_ELASTIC_FIBER_ASSEMBLY	-1.07	0.40	0	0	0	not significant
5088	GO_FATTY_ACID_SYNTHASE_ACTIVITY	-1.07	0.40	0	0	0	not significant
5089	GO_G_PROTEIN_BETA_SUBUNIT_BINDING	-1.07	0.40	0	0	0	not significant
5090	GO_PATTERN_RECOGNITION_RECEPTOR_ACTIVITY	-1.07	0.40	0	0	0	not significant
5091	GO_POSITIVE_REGULATION_OF_HORMONE_METABOLIC_PROCESS	-1.07	0.40	0	0	0	not significant
5092	GO_UBIQUINONE_BINDING	-1.07	0.40	0	0	0	not significant
5093	BYSTRYKH_HEMATOPOIESIS_STEM_CELL_FLI1	-1.07	0.39	0	0	0	not significant
5094	GO_PROTEIN_O_LINKED_FUCOSYLATION	-1.07	0.39	0	0	0	not significant
5095	GO_REGULATION_OF_PROTEIN_LOCALIZATION_TO_CELL_CORTEX	-1.07	0.39	0	0	0	not significant
5096	MYLLYKANGAS_AMPLIFICATION_HOT_SPOT_13	-1.07	0.39	0	0	0	not significant
5097	GO_CARNITINE_O_ACYLTRANSFERASE_ACTIVITY	-1.07	0.38	0	0	0	not significant
5098	GO_REGULATION_OF_ASPARTIC_TYPE_ENDOPEPTIDASE_ACTIVITY_INV(	-1.07	0.38	0	0	0	not significant
5099	GO_REGULATION_OF_HYALURONAN_BIOSYNTHETIC_PROCESS	-1.07	0.36	0	0	0	not significant
5100	GO_DNA_METABOLIC_PROCESS	-1.06	0.82	0	0	0	not significant
5101	GO_REGULATION_OF_CELL_DEVELOPMENT	-1.06	0.73	0	0	0	not significant
5102	GO_LIPID_METABOLIC_PROCESS	-1.06	0.72	0	0	0	not significant
5103	GO_REGULATION_OF_CELLULAR_RESPONSE_TO_STRESS	-1.06	0.70	0	0	0	not significant
5104	GO_PROTEIN_POLYUBIQUITINATION	-1.06	0.64	0	0	0	not significant
5105	GO_REGULATION_OF_GROWTH	-1.06	0.63	0	0	0	not significant
5106	GO_PROTEIN_CATABOLIC_PROCESS	-1.06	0.62	0	0	0	not significant
5107	GO_CELLULAR_RESPONSE_TO_ABIOTIC_STIMULUS	-1.06	0.60	0	0	0	not significant
5108	GO_RIBONUCLEOPROTEIN_GRANULE	-1.06	0.58	0	0	0	not significant
5109	GO_RNA_CATABOLIC_PROCESS	-1.06	0.58	0	0	0	not significant
5110	GO_LIPID_MODIFICATION	-1.06	0.56	0	0	0	not significant
5111	GO_OSSIFICATION	-1.06	0.56	0	0	0	not significant

5112	GO_POSITIVE_REGULATION_OF_CELL_CYCLE	-1.06	0.56	0	0	0	not significant
5113	GO_REGULATION_OF_VESICLE_MEDIATED_TRANSPORT	-1.06	0.56	0	0	0	not significant
5114	MULLIGHAN_NPM1_MUTATED_SIGNATURE_1_UP	-1.06	0.54	0	0	0	not significant
5115	FUJII_YBX1_TARGETS_DN	-1.06	0.53	0	0	0	not significant
5116	GO_POSITIVE_REGULATION_OF_CHROMOSOME_ORGANIZATION	-1.06	0.53	0	0	0	not significant
5117	GO_REGULATION_OF_CELL_CELL_ADHESION	-1.06	0.53	0	0	0	not significant
5118	STK33_DN	-1.06	0.53	0	0	0	not significant
5119	GO_CELL_SUBSTRATE_ADHESION	-1.06	0.52	0	0	0	not significant
5120	GO_RECEPTOR_METABOLIC_PROCESS	-1.06	0.51	0	0	0	not significant
5121	GO_REGULATION_OF_CELL_CYCLE_ARREST	-1.06	0.51	0	0	0	not significant
5122	GO_BASOLATERAL_PLASMA_MEMBRANE	-1.06	0.50	0	0	0	not significant
5123	GO_IMMUNOGLOBULIN_PRODUCTION	-1.06	0.50	0	0	0	not significant
5124	PROVENZANI_METASTASIS_UP	-1.06	0.50	0	0	0	not significant
5125	GO_PRIMARY_ACTIVE_TRANSMEMBRANE_TRANSPORTER_ACTIVITY	-1.06	0.48	0	0	0	not significant
5126	CHAUHAN_RESPONSE_TO_METHOXYESTRADIOL_DN	-1.06	0.47	0	0	0	not significant
5127	GO_ISOPRENOID_METABOLIC_PROCESS	-1.06	0.47	0	0	0	not significant
5128	GO_NUCLEOTIDE_EXCISION_REPAIR_PREINCISION_COMPLEX_ASSEMBLY	-1.06	0.47	0	0	0	not significant
5129	HALLMARK_INFLAMMATORY_RESPONSE	-1.06	0.47	0	0	0	not significant
5130	IKEDA_MIR1_TARGETS_UP	-1.06	0.46	0	0	0	not significant
5131	KYNG_ENVIRONMENTAL_STRESS_RESPONSE_UP	-1.06	0.46	0	0	0	not significant
5132	MORI_EMU_MYC_LYMPHOMA_BY_ONSET_TIME_UP	-1.06	0.46	0	0	0	not significant
5133	WANG_PROSTATE_CANCER_ANDROGEN_INDEPENDENT	-1.06	0.46	0	0	0	not significant
5134	CHESLER_BRAIN_QTL_CIS	-1.06	0.45	0	0	0	not significant
5135	GO_MITOTIC_CYTOKINESIS	-1.06	0.45	0	0	0	not significant
5136	GO_POSITIVE_REGULATION_OF_EXTRINSIC_APOPTOTIC_SIGNALING_PA	-1.06	0.45	0	0	0	not significant
5137	GO_REGULATION_OF_MULTICELLULAR_ORGANISM_GROWTH	-1.06	0.45	0	0	0	not significant
5138	GO_SIGNAL_TRANSDUCTION_INVOLVED_IN_CELL_CYCLE_CHECKPOINT	-1.06	0.45	0	0	0	not significant
5139	KEGG_LYSINE_DEGRADATION	-1.06	0.45	0	0	0	not significant
5140	RB_P130_DN.V1_UP	-1.06	0.45	0	0	0	not significant
5141	REACTOME_RET_SIGNALING	-1.06	0.45	0	0	0	not significant
5142	REACTOME_SEMAPHORIN_INTERACTIONS	-1.06	0.45	0	0	0	not significant
5143	SWEET_KRAS_TARGETS_DN	-1.06	0.45	0	0	0	not significant
5144	CHR10P15	-1.06	0.44	0	0	0	not significant
5145	GO_REGULATION_OF_POTASSIUM_ION_TRANSPORT	-1.06	0.44	0	0	0	not significant
5146	REACTOME_OTHER_SEMAPHORIN_INTERACTIONS	-1.06	0.44	0	0	0	not significant
5147	TERAO_AOX4_TARGETS_SKIN_UP	-1.06	0.44	0	0	0	not significant
5148	GO_C2H2_ZINC_FINGER_DOMAIN_BINDING	-1.06	0.43	0	0	0	not significant
5149	GO_POSITIVE_REGULATION_OF_GLYCOLYTIC_PROCESS	-1.06	0.43	0	0	0	not significant
5150	GO_REGULATION_OF_ACTOMYOSIN_STRUCTURE_ORGANIZATION	-1.06	0.43	0	0	0	not significant
5151	JOHANSSON_GLIOMAGENESIS_BY_PDGFB_DN	-1.06	0.43	0	0	0	not significant
5152	KEGG_DRUG_METABOLISM_OTHER_ENZYMES	-1.06	0.43	0	0	0	not significant
5153	REACTOME_REGULATION_OF_FOXO_TRANSCRIPTIONAL_ACTIVITY_BY_	-1.06	0.43	0	0	0	not significant
5154	ZHAN_V2_LATE_DIFFERENTIATION_GENES	-1.06	0.43	0	0	0	not significant
5155	BIOCARTA_ACTINY_PATHWAY	-1.06	0.42	0	0	0	not significant
5156	CHEN_PDGF_TARGETS	-1.06	0.42	0	0	0	not significant
5157	CHOW_RASSF1_TARGETS_UP	-1.06	0.42	0	0	0	not significant
5158	GO_COP9_SIGNALOSOME	-1.06	0.42	0	0	0	not significant
5159	GO_DISRUPTION_OF_CELLS_OF_OTHER_ORGANISM_INVOLVED_IN_SYM	-1.06	0.42	0	0	0	not significant
5160	GO_NEGATIVE_REGULATION_OF_ACTIN_FILAMENT_DEPOLYMERIZATION	-1.06	0.42	0	0	0	not significant
5161	GO_NEUROTRANSMITTER_TRANSPORTER_ACTIVITY	-1.06	0.42	0	0	0	not significant
5162	GO_PHYSIOLOGICAL_CARDIAC_MUSCLE_HYPERTROPHY	-1.06	0.42	0	0	0	not significant
5163	GO_POSITIVE_REGULATION_OF_MEMBRANE_POTENTIAL	-1.06	0.42	0	0	0	not significant
5164	GO_POSITIVE_REGULATION_OF_ORGAN_GROWTH	-1.06	0.42	0	0	0	not significant
5165	REACTOME_ECM_PROTEOGLYCANS	-1.06	0.42	0	0	0	not significant
5166	GO_CAMP_RESPONSE_ELEMENT_BINDING_PROTEIN_BINDING	-1.06	0.41	0	0	0	not significant
5167	GO_CHEMOKINE_C_X_C_MOTIF_LIGAND_2_PRODUCTION	-1.06	0.41	0	0	0	not significant
5168	GO_GUANYLATE_KINASE_ACTIVITY	-1.06	0.41	0	0	0	not significant
5169	GO_INTRINSIC_COMPONENT_OF_MITOCHONDRIAL_OUTER_MEMBRANE	-1.06	0.41	0	0	0	not significant
5170	GO_NEGATIVE_REGULATION_OF_T_CELL_RECEPTOR_SIGNALING_PATH	-1.06	0.41	0	0	0	not significant
5171	GO_REGULATION_OF_NEUTROPHIL_ACTIVATION	-1.06	0.41	0	0	0	not significant
5172	GO_TRANSCRIPTION_DEPENDENT_TETHERING_OF_RNA_POLYMERASE_	-1.06	0.41	0	0	0	not significant
5173	REACTOME_METABOLISM_OF_FAT_SOLUBLE_VITAMINS	-1.06	0.41	0	0	0	not significant
5174	GO_GROWTH_PLATE_CARTILAGE_CHONDROCYTE_DIFFERENTIATION	-1.06	0.40	0	0	0	not significant
5175	GO_HINDBRAIN_RADIAL_GLIA_GUIDED_CELL_MIGRATION	-1.06	0.40	0	0	0	not significant
5176	GO_MOLYBDOPTERIN_COFACTOR_BIOSYNTHETIC_PROCESS	-1.06	0.40	0	0	0	not significant
5177	GO_NUCLEOSIDE_CATABOLIC_PROCESS	-1.06	0.40	0	0	0	not significant
5178	GO_POSITIVE_REGULATION_OF_HELICASE_ACTIVITY	-1.06	0.40	0	0	0	not significant
5179	GO_POSITIVE_REGULATION_OF_MYELOID_LEUKOCYTE_CYTOKINE_PRO	-1.06	0.40	0	0	0	not significant
5180	GO_RRNA_TRANSPORT	-1.06	0.40	0	0	0	not significant
5181	GO_STEROID_ESTERIFICATION	-1.06	0.40	0	0	0	not significant
5182	HUPER_BREAST_BASAL_VS_LUMINAL_UP	-1.06	0.40	0	0	0	not significant
5183	REACTOME_TRIGLYCERIDE_METABOLISM	-1.06	0.40	0	0	0	not significant
5184	BIOCARTA_LONGEVITY_PATHWAY	-1.06	0.39	0	0	0	not significant
5185	GO_DOPAMINE_METABOLIC_PROCESS	-1.06	0.39	0	0	0	not significant
5186	GO_LONG_CHAIN_FATTY_ACID_BINDING	-1.06	0.39	0	0	0	not significant
5187	GO_PROTEIN_INSERTION_INTO_ER_MEMBRANE	-1.06	0.39	0	0	0	not significant
5188	GO_TETRAHYDROFOLATE_BIOSYNTHETIC_PROCESS	-1.06	0.39	0	0	0	not significant
5189	GO_POSITIVE_REGULATION_OF_CHEMOKINE_SECRETION	-1.06	0.38	0	0	0	not significant
5190	WACKER_HYPOXIA_TARGETS_OF_VHL	-1.06	0.38	0	0	0	not significant
5191	GO_ETHER_METABOLIC_PROCESS	-1.06	0.37	0	0	0	not significant
5192	GO_GLUTAMATE_CATABOLIC_PROCESS	-1.06	0.37	0	0	0	not significant
5193	GO_CDC42_PROTEIN_SIGNAL_TRANSDUCTION	-1.06	0.36	0	0	0	not significant
5194	REACTOME_PRE_NOTCH_PROCESSING_IN_THE_ENDOPLASMIC_RETICUL	-1.06	0.36	0	0	0	not significant
5195	GO_REGULATION_OF_PHOSPHORUS_METABOLIC_PROCESS	-1.05	0.79	0	0	0	not significant
5196	GO_NEGATIVE_REGULATION_OF_SIGNALING	-1.05	0.67	0	0	0	not significant
5197	MARTINEZ_TP53_TARGETS_DN	-1.05	0.64	0	0	0	not significant
5198	NUYTTEN_NIPP1_TARGETS_UP	-1.05	0.64	0	0	0	not significant
5199	MARTINEZ_RB1_TARGETS_DN	-1.05	0.56	0	0	0	not significant
5200	GO_PEPTIDASE_ACTIVITY	-1.05	0.55	0	0	0	not significant
5201	GO_LAMELLIPODIUM	-1.05	0.54	0	0	0	not significant
5202	CHR6P21	-1.05	0.53	0	0	0	not significant
5203	GO_REGULATION_OF_APOPTOTIC_SIGNALING_PATHWAY	-1.05	0.53	0	0	0	not significant
5204	GO_VESICLE_LOCALIZATION	-1.05	0.53	0	0	0	not significant
5205	KEGG_ENDOCYTOSIS	-1.05	0.53	0	0	0	not significant
5206	GO_HOMEOSTASIS_OF_NUMBER_OF_CELLS	-1.05	0.52	0	0	0	not significant
5207	GO_REGULATION_OF_WNT_SIGNALING_PATHWAY	-1.05	0.52	0	0	0	not significant
5208	YAGI_AML_WITH_11Q23_REARRANGED	-1.05	0.52	0	0	0	not significant
5209	BILD_HRAS_ONCOGENIC_SIGNATURE	-1.05	0.50	0	0	0	not significant
5210	BROWNE_HCMV_INFECTION_24HR_UP	-1.05	0.50	0	0	0	not significant
5211	THUM_SYSTOLIC_HEART_FAILURE_UP	-1.05	0.50	0	0	0	not significant
5212	GO_AZUROPHIL_GRANULE	-1.05	0.49	0	0	0	not significant
5213	KOKKINAKIS_METHIONINE_DEPRIVATION_48HR_UP	-1.05	0.49	0	0	0	not significant
5214	GO_CONDENSED_CHROMOSOME	-1.05	0.48	0	0	0	not significant
5215	GO_MONOSACCHARIDE_METABOLIC_PROCESS	-1.05	0.48	0	0	0	not significant
5216	GO_NEGATIVE_REGULATION_OF_MAPK_CASCADE	-1.05	0.48	0	0	0	not significant
5217	GO_TYPE_I_INTERFERON_PRODUCTION	-1.05	0.48	0	0	0	not significant
5218	TONKS_TARGETS_OF_RUNX1_RUNX1T1_FUSION_ERYTHROCYTE_UP	-1.05	0.48	0	0	0	not significant
5219	REACTOME_CENTROSOME_MATURATION	-1.05	0.47	0	0	0	not significant
5220	REACTOME_PRC2_METHYLATES_HISTONES_AND_DNA	-1.05	0.47	0	0	0	not significant

5221	GAVIN_FOXP3_TARGETS_CLUSTER_P3	-1.05	0.46	0	0	0	not significant
5222	GO_NEGATIVE_REGULATION_OF_PROTEOLYSIS	-1.05	0.46	0	0	0	not significant
5223	NABA_ECM_GLYCOPROTEINS	-1.05	0.46	0	0	0	not significant
5224	FONTAINE_THYROID_TUMOR_UNCERTAIN_MALIGNANCY_UP	-1.05	0.45	0	0	0	not significant
5225	GERY_CEBP_TARGETS	-1.05	0.45	0	0	0	not significant
5226	GO_KINESIN_BINDING	-1.05	0.45	0	0	0	not significant
5227	GO_TRANSLATION_REGULATOR_ACTIVITY	-1.05	0.45	0	0	0	not significant
5228	GRADE_COLON_VS_RECTAL_CANCER_UP	-1.05	0.45	0	0	0	not significant
5229	BROWNE_HCMV_INFECTION_16HR_DN	-1.05	0.44	0	0	0	not significant
5230	GO_EAR_DEVELOPMENT	-1.05	0.44	0	0	0	not significant
5231	GO_GROWTH_FACTOR_ACTIVITY	-1.05	0.44	0	0	0	not significant
5232	GO_PURINE_CONTAINING_COMPOUND_CATABOLIC_PROCESS	-1.05	0.44	0	0	0	not significant
5233	KALMA_E2F1_TARGETS	-1.05	0.44	0	0	0	not significant
5234	KEGG_ABC_TRANSPORTERS	-1.05	0.44	0	0	0	not significant
5235	KEGG_ECM_RECEPTOR_INTERACTION	-1.05	0.44	0	0	0	not significant
5236	KEGG_PHOSPHATIDYLINOSITOL_SIGNALING_SYSTEM	-1.05	0.44	0	0	0	not significant
5237	LEE_AGING_NEOCORTEX_UP	-1.05	0.44	0	0	0	not significant
5238	REACTOME_SIGNALING_BY_EGFR	-1.05	0.44	0	0	0	not significant
5239	ROSS_AML_WITH_PML_RARA_FUSION	-1.05	0.44	0	0	0	not significant
5240	GO_CYCLIN_DEPENDENT_PROTEIN_KINASE_HOLOENZYME_COMPLEX	-1.05	0.43	0	0	0	not significant
5241	GO_POSITIVE_REGULATION_OF_DEPHOSPHORYLATION	-1.05	0.43	0	0	0	not significant
5242	MARSON_FOXP3_CORE_DIRECT_TARGETS	-1.05	0.43	0	0	0	not significant
5243	BIOCARTA_CARDIACEGF_PATHWAY	-1.05	0.42	0	0	0	not significant
5244	GO_CELLULAR_COMPONENT_DISASSEMBLY_INVOLVED_IN_EXECUTION_	-1.05	0.42	0	0	0	not significant
5245	GO_EMBRYONIC_CAMERA_TYPE_EYE_MORPHOGENESIS	-1.05	0.42	0	0	0	not significant
5246	GO_H4_H2A_HISTONE_ACETYLTRANSFERASE_COMPLEX	-1.05	0.42	0	0	0	not significant
5247	GO_MITOTIC_SPINDLE_POLE	-1.05	0.42	0	0	0	not significant
5248	GO_PROTEIN_PHOSPHATASE_ACTIVATOR_ACTIVITY	-1.05	0.42	0	0	0	not significant
5249	GO_REGULATION_OF_TYPE_I_INTERFERON_MEDIATED_SIGNALING_PAT	-1.05	0.42	0	0	0	not significant
5250	RASHI_RESPONSE_TO_IONIZING_RADIATION_1	-1.05	0.42	0	0	0	not significant
5251	REACTOME_ACTIVATION_OF_BAD_AND_TRANSLOCATION_TO_MITOCHO	-1.05	0.42	0	0	0	not significant
5252	REACTOME_LDL_CLEARANCE	-1.05	0.42	0	0	0	not significant
5253	BOYAULT_LIVER_CANCER_SUBCLASS_G5_DN	-1.05	0.41	0	0	0	not significant
5254	GO_LOW_DENSITY_LIPOPROTEIN_PARTICLE_BINDING	-1.05	0.41	0	0	0	not significant
5255	GO_NEGATIVE_REGULATION_OF_MUSCLE_HYPERTROPHY	-1.05	0.41	0	0	0	not significant
5256	KUROKAWA_LIVER_CANCER_CHEMOTHERAPY_DN	-1.05	0.41	0	0	0	not significant
5257	POS_RESPONSE_TO_HISTAMINE_UP	-1.05	0.41	0	0	0	not significant
5258	RICKMAN_HEAD_AND_NECK_CANCER_C	-1.05	0.41	0	0	0	not significant
5259	BIOCARTA_VDR_PATHWAY	-1.05	0.40	0	0	0	not significant
5260	GO_GLUTATHIONE_METABOLIC_PROCESS	-1.05	0.40	0	0	0	not significant
5261	GO_NEGATIVE_REGULATION_OF_PROTEIN_MATURATION	-1.05	0.40	0	0	0	not significant
5262	GO_POSITIVE_REGULATION_OF_MRNA_SPLICING_VIA_SPLICEOSOME	-1.05	0.40	0	0	0	not significant
5263	GO_RESPONSE_TO_STEROL	-1.05	0.40	0	0	0	not significant
5264	GO_UBIQUITIN_CONJUGATING_ENZYME_COMPLEX	-1.05	0.40	0	0	0	not significant
5265	HENDRICKS_SMARCA4_TARGETS_DN	-1.05	0.40	0	0	0	not significant
5266	KORKOLA_TERATOMA_UP	-1.05	0.40	0	0	0	not significant
5267	REACTOME_METABOLISM_OF_NITRIC_OXIDE:_ENOS_ACTIVATION_AND_I	-1.05	0.40	0	0	0	not significant
5268	BIOCARTA_MITOCHONDRIA_PATHWAY	-1.05	0.39	0	0	0	not significant
5269	CHIANG_LIVER_CANCER_SUBCLASS_POLYSOMY7_DN	-1.05	0.39	0	0	0	not significant
5270	GO_3_5_CYCLIC_GMP_PHOSPHODIESTERASE_ACTIVITY	-1.05	0.39	0	0	0	not significant
5271	GO_CCR_CHEMOKINE_RECEPTOR_BINDING	-1.05	0.39	0	0	0	not significant
5272	GO_LIPID_DROPLET_ORGANIZATION	-1.05	0.39	0	0	0	not significant
5273	GO_MEGAKARYOCYTE_DEVELOPMENT	-1.05	0.39	0	0	0	not significant
5274	GO_MITOCHONDRIAL_GENOME_MAINTENANCE	-1.05	0.39	0	0	0	not significant
5275	GO_NEGATIVE_REGULATION_OF_HEMATOPOIETIC_PROGENITOR_CELL_	-1.05	0.39	0	0	0	not significant
5276	GO_PHOSPHORYLATION_OF_RNA_POLYMERASE_II_C_TERMINAL_DOMAI	-1.05	0.39	0	0	0	not significant
5277	GO_POSITIVE_REGULATION_OF_IRE1_MEDIATED_UNFOLDED_PROTEIN_	-1.05	0.39	0	0	0	not significant
5278	GO_POSITIVE_REGULATION_OF_PHOSPHOLIPID_TRANSPORT	-1.05	0.39	0	0	0	not significant
5279	GO_RESPONSE_TO_ETHER	-1.05	0.39	0	0	0	not significant
5280	HOEGERKORP_CD44_TARGETS_TEMPORAL_DN	-1.05	0.39	0	0	0	not significant
5281	MYLLYKANGAS_AMPLIFICATION_HOT_SPOT_24	-1.05	0.39	0	0	0	not significant
5282	GO_ALANINE_TRANSPORT	-1.05	0.38	0	0	0	not significant
5283	GO_CALCIUM_TRANSPORTING_ATPASE_ACTIVITY	-1.05	0.38	0	0	0	not significant
5284	GO_CLATHRIN_COMPLEX	-1.05	0.38	0	0	0	not significant
5285	GO_PHOSPHOSERINE_RESIDUE_BINDING	-1.05	0.38	0	0	0	not significant
5286	GO_REGULATION_OF_LAMELLIPODIUM_MORPHOGENESIS	-1.05	0.38	0	0	0	not significant
5287	GO_POLYNUCLEOTIDE_ADENYLYLTRANSFERASE_ACTIVITY	-1.05	0.37	0	0	0	not significant
5288	REACTOME_RECYCLING_OF_BILE_ACIDS_AND_SALTS	-1.05	0.36	0	0	0	not significant
5289	ZEILSTRA_CD44_TARGETS_DN	-1.05	0.36	0	0	0	not significant
5290	GO_REGULATION_OF_GLOMERULUS_DEVELOPMENT	-1.05	0.34	0	0	0	not significant
5291	GO_POSITIVE_REGULATION_OF_GENE_EXPRESSION	-1.04	0.86	0	0	0	not significant
5292	GO_REGULATION_OF_PROTEIN_MODIFICATION_PROCESS	-1.04	0.68	0	0	0	not significant
5293	GO_HEAD_DEVELOPMENT	-1.04	0.51	0	0	0	not significant
5294	GO_LIPID_CATABOLIC_PROCESS	-1.04	0.51	0	0	0	not significant
5295	GO_CARTILAGE_DEVELOPMENT	-1.04	0.48	0	0	0	not significant
5296	GO_POSITIVE_REGULATION_OF_PROTEIN_CATABOLIC_PROCESS	-1.04	0.48	0	0	0	not significant
5297	BONOME_OVARIAN_CANCER_SURVIVAL_OPTIMAL_DEBULKING	-1.04	0.47	0	0	0	not significant
5298	HOLLMANN_APOPTOSIS_VIA_CD40_DN	-1.04	0.47	0	0	0	not significant
5299	ZHOU_INFLAMMATORY_RESPONSE_FIMA_UP	-1.04	0.47	0	0	0	not significant
5300	GO_DNA_REPLICATION_INDEPENDENT_NUCLEOSOME_ORGANIZATION	-1.04	0.46	0	0	0	not significant
5301	GO_LAMELLIPODIUM_ORGANIZATION	-1.04	0.46	0	0	0	not significant
5302	GO_ORGANOPHOSPHATE_CATABOLIC_PROCESS	-1.04	0.46	0	0	0	not significant
5303	KEGG_VIRAL_MYOCARDITIS	-1.04	0.46	0	0	0	not significant
5304	GO_CLATHRIN_COATED_PIT	-1.04	0.45	0	0	0	not significant
5305	GO_COPII_COATED_ER_TO_GOLGI_TRANSPORT_VESICLE	-1.04	0.45	0	0	0	not significant
5306	PURBEY_TARGETS_OF_CTBP1_AND_SATB1_DN	-1.04	0.45	0	0	0	not significant
5307	CHIANG_LIVER_CANCER_SUBCLASS_CTNNB1_DN	-1.04	0.44	0	0	0	not significant
5308	DANG_REGULATED_BY_MYC_DN	-1.04	0.44	0	0	0	not significant
5309	GO_CELLULAR_RESPONSE_TO_IONIZING_RADIATION	-1.04	0.44	0	0	0	not significant
5310	GO_POSITIVE_REGULATION_OF_CELL_DIVISION	-1.04	0.44	0	0	0	not significant
5311	GO_PROTEIN_LOCALIZATION_TO_PLASMA_MEMBRANE	-1.04	0.44	0	0	0	not significant
5312	GO_REGULATION_OF_NOTCH_SIGNALING_PATHWAY	-1.04	0.44	0	0	0	not significant
5313	REACTOME_TRIF_TICAM1_MEDIATED_TLR4_SIGNALING	-1.04	0.44	0	0	0	not significant
5314	REACTOME_UNFOLDED_PROTEIN_RESPONSE_UPR	-1.04	0.44	0	0	0	not significant
5315	CREIGHTON_AKT1_SIGNALING_VIA_MTOR_UP	-1.04	0.43	0	0	0	not significant
5316	GO_DETECTION_OF_LIGHT_STIMULUS	-1.04	0.43	0	0	0	not significant
5317	GO_PROTEIN_TYROSINE_KINASE_BINDING	-1.04	0.43	0	0	0	not significant
5318	KEGG_VASOPRESSIN_REGULATED_WATER_REABSORPTION	-1.04	0.43	0	0	0	not significant
5319	LANG_MYB_FAMILY_TARGETS	-1.04	0.43	0	0	0	not significant
5320	LEE_CALORIE_RESTRICTION_MUSCLE_DN	-1.04	0.43	0	0	0	not significant
5321	REACTOME_ANCHORING_OF_THE_BASAL_BODY_TO_THE_PLASMA_MEM	-1.04	0.43	0	0	0	not significant
5322	GO_INTERFERON_BETA_PRODUCTION	-1.04	0.42	0	0	0	not significant
5323	GO_REGULATION_OF_CALCINEURIN_MEDIATED_SIGNALING	-1.04	0.42	0	0	0	not significant
5324	GO_REGULATION_OF_COFACTOR_METABOLIC_PROCESS	-1.04	0.42	0	0	0	not significant
5325	CAHOY_OLIGODENDROCUTIC	-1.04	0.41	0	0	0	not significant
5326	GENTILE_UV_RESPONSE_CLUSTER_D1	-1.04	0.41	0	0	0	not significant
5327	GO_HSP90_PROTEIN_BINDING	-1.04	0.41	0	0	0	not significant
5328	GO_MYOSIN_BINDING	-1.04	0.41	0	0	0	not significant
5329	GO_NUCLEAR_TRANSCRIBED_MRNA_CATABOLIC_PROCESS_EXONUCLE	-1.04	0.41	0	0	0	not significant

5330	GO_POSITIVE_REGULATION_OF_SKELETAL_MUSCLE_TISSUE_DEVELOPI	-1.04	0.41	0	0	0	not significant
5331	GO POSITIVE REGULATION OF TUMOR NECROSIS FACTOR SUPERFA	-1.04	0.41	0	0	0	not significant
5332	GO_REGULATION_OF_RUFFLE_ASSEMBLY	-1.04	0.41	0	0	0	not significant
5333	GO_SKELETAL_MUSCLE_CONTRACTION	-1.04	0.41	0	0	0	not significant
5334	HAN_JNK_SINGALING_DN	-1.04	0.41	0	0	0	not significant
5335	KYNG_RESPONSE_TO_H2O2_VIA_ERCC6_UP	-1.04	0.41	0	0	0	not significant
5336	SANA_TNF_SIGNALING_UP	-1.04	0.41	0	0	0	not significant
5337	GO_AXIS_ELONGATION	-1.04	0.40	0	0	0	not significant
5338	GO NEGATIVE REGULATION OF MUSCLE CELL APOPTOTIC PROCESS	-1.04	0.40	0	0	0	not significant
5339	GO_OUTFLOW_TRACT_MORPHOGENESIS	-1.04	0.40	0	0	0	not significant
5340	GO_POSITIVE_REGULATION_OF_ALPHA_BETA_T_CELL_ACTIVATION	-1.04	0.40	0	0	0	not significant
5341	GO_PROTEIN_K63_LINKED_UBIQUITINATION	-1.04	0.40	0	0	0	not significant
5342	GO_REGULATION_OF_SYNAPTIC_VESICLE_TRANSPORT	-1.04	0.40	0	0	0	not significant
5343	RASHI_RESPONSE_TO_IONIZING_RADIATION_3	-1.04	0.40	0	0	0	not significant
5344	VALK_AML_WITH_CEBPA	-1.04	0.40	0	0	0	not significant
5345	ABE_VEGFA_TARGETS_2HR	-1.04	0.39	0	0	0	not significant
5346	BIOCARTA_IGF1_PATHWAY	-1.04	0.39	0	0	0	not significant
5347	GO_NEGATIVE_REGULATION_OF_LIPID_STORAGE	-1.04	0.39	0	0	0	not significant
5348	GO_NUCLEOSIDE_DIPHOSPHATE_BIOSYNTHETIC_PROCESS	-1.04	0.39	0	0	0	not significant
5349	GO_PENTOSE_PHOSPHATE_SHUNT	-1.04	0.39	0	0	0	not significant
5350	GO_REGULATION_OF_ALKALINE_PHOSPHATASE_ACTIVITY	-1.04	0.39	0	0	0	not significant
5351	GO_RESPONSE_TO_ACIDIC_PH	-1.04	0.39	0	0	0	not significant
5352	GO_RESPONSE_TO_EPINEPHRINE	-1.04	0.39	0	0	0	not significant
5353	GO_TRANSFERRIN_RECEPTOR_BINDING	-1.04	0.39	0	0	0	not significant
5354	MCBRYAN_PUBERTAL_BREAST_3_4WK_DN	-1.04	0.39	0	0	0	not significant
5355	REACTOME_DEFECTIVE_EXT2_CAUSES_EXOSTOSES_2	-1.04	0.39	0	0	0	not significant
5356	BIOCARTA_RNA_PATHWAY	-1.04	0.38	0	0	0	not significant
5357	GO_PHOSPHATIDYLINOSITOL_PHOSPHATE_4_PHOSPHATASE_ACTIVITY	-1.04	0.38	0	0	0	not significant
5358	GO_POSITIVE_REGULATION_OF_HISTONE_DEACETYLATION	-1.04	0.38	0	0	0	not significant
5359	GO_REGULATION_OF_RESPONSE_TO_EXTRACELLULAR_STIMULUS	-1.04	0.38	0	0	0	not significant
5360	REACTOME_SIGNALING_BY_NTRK3_TRKC	-1.04	0.38	0	0	0	not significant
5361	RUAN_RESPONSE_TO_TNF_TROGLITAZONE_UP	-1.04	0.38	0	0	0	not significant
5362	GO_ANTEROGRADE_AXONAL_PROTEIN_TRANSPORT	-1.04	0.37	0	0	0	not significant
5363	GO_CELL_MIGRATION_IN_HINDBRAIN	-1.04	0.37	0	0	0	not significant
5364	GO CELLULAR RESPONSE TO BRAIN DERIVED NEUROTROPHIC FACT	-1.04	0.37	0	0	0	not significant
5365	GO_CHEMOKINE_C_C_MOTIF_LIGAND_5_PRODUCTION	-1.04	0.37	0	0	0	not significant
5366	GO_DOUBLE_STRAND_BREAK_REPAIR_VIA_SYNTHESIS_DEPENDENT_ST	-1.04	0.37	0	0	0	not significant
5367	GO PHOSPHATIDYLINOSITOL 3 5 BISPHOSPHATE PHOSPHATASE ACTI	-1.04	0.37	0	0	0	not significant
5368	GO POSITIVE REGULATION BY HOST OF VIRAL GENOME REPLICATIO	-1.04	0.37	0	0	0	not significant
5369	GO_SCARNA_LOCALIZATION_TO_CAJAL_BODY	-1.04	0.37	0	0	0	not significant
5370	BIOCARTA_AGPCR_PATHWAY	-1.04	0.36	0	0	0	not significant
5371	GO_5_3_EXODEOXYRIBONUCLEASE_ACTIVITY	-1.04	0.36	0	0	0	not significant
5372	GO_NEGATIVE_REGULATION_OF_PROTEIN_FOLDING	-1.04	0.36	0	0	0	not significant
5373	GO_PROSTAGLANDIN_TRANSPORT	-1.04	0.36	0	0	0	not significant
5374	HERNANDEZ_MITOTIC_ARREST_BY_DOCETAXEL_2_DN	-1.04	0.36	0	0	0	not significant
5375	MIKHAYLOVA_OXIDATIVE_STRESS_RESPONSE_VIA_VHL_DN	-1.04	0.36	0	0	0	not significant
5376	REACTOME_PLASMA_LIPOPROTEIN_ASSEMBLY	-1.04	0.36	0	0	0	not significant
5377	CHEOK_RESPONSE_TO_MERCAPTOPURINE_AND_HD_MTX_UP	-1.04	0.35	0	0	0	not significant
5378	GO AMYLOID BETA CLEARANCE BY CELLULAR CATABOLIC PROCESS	-1.04	0.35	0	0	0	not significant
5379	GO_RS_DOMAIN_BINDING	-1.04	0.35	0	0	0	not significant
5380	NAKAMURA_CANCER_MICROENVIRONMENT_UP	-1.04	0.35	0	0	0	not significant
5381	REACTOME_PD_1_SIGNALING	-1.04	0.35	0	0	0	not significant
5382	GO_REGULATION_OF_PROTEIN_LOCALIZATION	-1.03	0.60	0	0	0	not significant
5383	GO_POSITIVE_REGULATION_OF_PROTEIN_MODIFICATION_PROCESS	-1.03	0.53	0	0	0	not significant
5384	GO_GOLGI_VESICLE_TRANSPORT	-1.03	0.49	0	0	0	not significant
5385	GO_ANION_TRANSPORT	-1.03	0.48	0	0	0	not significant
5386	KOYAMA_SEMA3B_TARGETS_DN	-1.03	0.47	0	0	0	not significant
5387	GO_RNA_PHOSPHODIESTER_BOND_HYDROLYSIS_ENDONUCLEOLYTIC	-1.03	0.45	0	0	0	not significant
5388	LU_AGING_BRAIN_UP	-1.03	0.45	0	0	0	not significant
5389	ACEVEDO_LIVER_CANCER_DN	-1.03	0.44	0	0	0	not significant
5390	PKCA_DN.V1_DN	-1.03	0.44	0	0	0	not significant
5391	GO_APICAL_PART_OF_CELL	-1.03	0.43	0	0	0	not significant
5392	GO_INTERLEUKIN_1_BETA_PRODUCTION	-1.03	0.43	0	0	0	not significant
5393	GO_NEGATIVE_REGULATION_OF_TRANSPORTER_ACTIVITY	-1.03	0.43	0	0	0	not significant
5394	GO_RESPONSE_TO_METAL_ION	-1.03	0.43	0	0	0	not significant
5395	GO_SIGNAL_TRANSDUCTION_IN_RESPONSE_TO_DNA_DAMAGE	-1.03	0.43	0	0	0	not significant
5396	PID_AR_TF_PATHWAY	-1.03	0.43	0	0	0	not significant
5397	GO_ANTIGEN_PROCESSING_AND_PRESENTATION_VIA_MHC_CLASS_IB	-1.03	0.42	0	0	0	not significant
5398	GO_CALCIUM_DEPENDENT_PROTEIN_KINASE_ACTIVITY	-1.03	0.42	0	0	0	not significant
5399	GO_CELL_MIGRATION_INVOLVED_IN_SPROUTING_ANGIOGENESIS	-1.03	0.42	0	0	0	not significant
5400	GO_EMBRYONIC_ORGAN_MORPHOGENESIS	-1.03	0.42	0	0	0	not significant
5401	GO_G_PROTEIN_COUPLED_RECEPTOR_SIGNALING_PATHWAY_COUPLEI	-1.03	0.42	0	0	0	not significant
5402	GO_NEPHRON_MORPHOGENESIS	-1.03	0.42	0	0	0	not significant
5403	GO_NEURON_APOPTOTIC_PROCESS	-1.03	0.42	0	0	0	not significant
5404	GO_NUCLEAR_TRANSCRIPTION_FACTOR_COMPLEX	-1.03	0.42	0	0	0	not significant
5405	REACTOME_PLATELET_HOMEOSTASIS	-1.03	0.42	0	0	0	not significant
5406	GO_PEPTIDYL_THREONINE_MODIFICATION	-1.03	0.41	0	0	0	not significant
5407	GO_PYRIMIDINE_CONTAINING_COMPOUND_CATABOLIC_PROCESS	-1.03	0.41	0	0	0	not significant
5408	GO_REGULATION_OF_ERBB_SIGNALING_PATHWAY	-1.03	0.41	0	0	0	not significant
5409	GO_REGULATION_OF_HORMONE_SECRETION	-1.03	0.41	0	0	0	not significant
5410	LABBE_WNT3A_TARGETS_DN	-1.03	0.41	0	0	0	not significant
5411	LEE_AGING_CEREBELLUM_UP	-1.03	0.41	0	0	0	not significant
5412	REACTOME_SULFUR_AMINO_ACID_METABOLISM	-1.03	0.41	0	0	0	not significant
5413	TAVAZOIE_METASTASIS	-1.03	0.41	0	0	0	not significant
5414	BAE_BRCA1_TARGETS_UP	-1.03	0.40	0	0	0	not significant
5415	GO_CENTRAL_NERVOUS_SYSTEM_PROJECTION_NEURON_AXONOGENES	-1.03	0.40	0	0	0	not significant
5416	GO_MITOTIC_CYTOKINETIC_PROCESS	-1.03	0.40	0	0	0	not significant
5417	GO_PHOTOTRANSDUCTION	-1.03	0.40	0	0	0	not significant
5418	GO_RNA_SURVEILLANCE	-1.03	0.40	0	0	0	not significant
5419	HOSHIDA_LIVER_CANCER_SURVIVAL_UP	-1.03	0.40	0	0	0	not significant
5420	KEGG_FOCAL_ADHESION	-1.03	0.40	0	0	0	not significant
5421	MORI_MATURE_B_LYMPHOCYTE_UP	-1.03	0.40	0	0	0	not significant
5422	REACTOME_GAP_JUNCTION_DEGRADATION	-1.03	0.40	0	0	0	not significant
5423	REACTOME_INTERLEUKIN_4_AND_INTERLEUKIN_13_SIGNALING	-1.03	0.40	0	0	0	not significant
5424	REACTOME_VITAMIN_B5_PANTOTHENATE_METABOLISM	-1.03	0.40	0	0	0	not significant
5425	REN_ALVEOLAR_RHABDOMYOSARCOMA_UP	-1.03	0.40	0	0	0	not significant
5426	SATO_SILENCED_EPIGENETICALLY_IN_PANCREATIC_CANCER	-1.03	0.40	0	0	0	not significant
5427	ZWANG_EGF_INTERVAL_UP	-1.03	0.40	0	0	0	not significant
5428	GO_HISTONE_METHYLTRANSFERASE_ACTIVITY	-1.03	0.39	0	0	0	not significant
5429	GO_MOTOR_ACTIVITY	-1.03	0.39	0	0	0	not significant
5430	GO NEGATIVE REGULATION OF PHOSPHATIDYLINOSITOL 3 KINASE SI	-1.03	0.39	0	0	0	not significant
5431	GO_NEGATIVE_REGULATION_OF_TRANSCRIPTION_FROM_RNA_POLYME	-1.03	0.39	0	0	0	not significant
5432	GO_PHOSPHATIDYLINOSITOL_PHOSPHATE_PHOSPHATASE_ACTIVITY	-1.03	0.39	0	0	0	not significant
5433	GO_REGULATION_OF_MITOTIC_CENTROSOME_SEPARATION	-1.03	0.39	0	0	0	not significant
5434	GO_RUFFLE_MEMBRANE	-1.03	0.39	0	0	0	not significant
5435	HONRADO_BREAST_CANCER_BRCA1_VS_BRCA2	-1.03	0.39	0	0	0	not significant
5436	MCCLUNG_COCAIN_REWARD_4WK	-1.03	0.39	0	0	0	not significant
5437	OHGUCHI_LIVER_HNF4A_TARGETS_UP	-1.03	0.39	0	0	0	not significant
5438	REACTOME_TRANS_GOLGI_NETWORK_VESICLE_BUDDING	-1.03	0.39	0	0	0	not significant

5439	FINETTI_BREAST_CANCER_KINOME_GREEN	-1.03	0.38	0	0	0	not significant
5440	GO_CELL_COMMUNICATION_INVOLVED_IN_CARDIAC_CONDUCTION	-1.03	0.38	0	0	0	not significant
5441	GO_CHEMICAL_HOMEOSTASIS_WITHIN_A_TISSUE	-1.03	0.38	0	0	0	not significant
5442	GO_NEGATIVE_REGULATION_OF_MUSCLE_CONTRACTION	-1.03	0.38	0	0	0	not significant
5443	GO_PLASMA_MEMBRANE_TO_ENDOSOME_TRANSPORT	-1.03	0.38	0	0	0	not significant
5444	GO_POSITIVE_REGULATION_OF_CALCIUM_ION_TRANSPORT_INTO_CYTC	-1.03	0.38	0	0	0	not significant
5445	GO_POSITIVE_REGULATION_OF_NIK_NF_KAPPAB_SIGNALING	-1.03	0.38	0	0	0	not significant
5446	GO_REGULATION_OF_TRANSCRIPTION_ELONGATION_FROM_RNA_POLY	-1.03	0.38	0	0	0	not significant
5447	GO_RESPONSE_TO_GAMMA_RADIATION	-1.03	0.38	0	0	0	not significant
5448	MOOTHA_FFA_OXYDATION	-1.03	0.38	0	0	0	not significant
5449	REACTOME_FCERI_MEDIATED_MAPK_ACTIVATION	-1.03	0.38	0	0	0	not significant
5450	XU_HGF_TARGETS_REPRESSED_BY_AKT1_UP	-1.03	0.38	0	0	0	not significant
5451	GO_INOSITOL_PHOSPHATE_PHOSPHATASE_ACTIVITY	-1.03	0.37	0	0	0	not significant
5452	GO_INWARD_RECTIFIER_POTASSIUM_CHANNEL_ACTIVITY	-1.03	0.37	0	0	0	not significant
5453	GO_REGULATION_OF_RNA_BINDING	-1.03	0.37	0	0	0	not significant
5454	GO_REGULATION_OF_SYNAPTIC_TRANSMISSION_DOPAMINERGIC	-1.03	0.37	0	0	0	not significant
5455	GO_REGULATION_OF_URINE_VOLUME	-1.03	0.37	0	0	0	not significant
5456	GO_RIBOSOMAL_LARGE_SUBUNIT_ASSEMBLY	-1.03	0.37	0	0	0	not significant
5457	LEIN_PONS_MARKERS	-1.03	0.37	0	0	0	not significant
5458	WORSCHECH_TUMOR_EVASION_AND_TOLEROGENICITY_DN	-1.03	0.37	0	0	0	not significant
5459	GO_INTRINSIC_COMPONENT_OF_EXTERNAL_SIDE_OF_PLASMA_MEMBRA	-1.03	0.36	0	0	0	not significant
5460	GO_LAYER_FORMATION_IN_CEREBRAL_CORTEX	-1.03	0.36	0	0	0	not significant
5461	GO_REPLICATION_FORK_PROTECTION_COMPLEX	-1.03	0.36	0	0	0	not significant
5462	GO_SERTOLI_CELL_DIFFERENTIATION	-1.03	0.36	0	0	0	not significant
5463	REACTOME_MITOCHONDRIAL_IRON_SULFUR_CLUSTER_BIOGENESIS	-1.03	0.36	0	0	0	not significant
5464	BIOCARTA_HES_PATHWAY	-1.03	0.35	0	0	0	not significant
5465	CORRADETTI_MTOR_PATHWAY_REGULATORS_DN	-1.03	0.35	0	0	0	not significant
5466	GENTLES_LEUKEMIC_STEM_CELL_DN	-1.03	0.35	0	0	0	not significant
5467	GO_CORTICAL_ENDOPLASMIC_RETICULUM	-1.03	0.35	0	0	0	not significant
5468	REACTOME_ARMS_MEDIATED_ACTIVATION	-1.03	0.35	0	0	0	not significant
5469	GO_NUCLEAR_STRESS_GRANULE	-1.03	0.34	0	0	0	not significant
5470	GO_TRNA_PSEUDOURIDINE_SYNTHESIS	-1.03	0.34	0	0	0	not significant
5471	PID_P38_MK2_PATHWAY	-1.03	0.34	0	0	0	not significant
5472	GO_NEGATIVE_REGULATION_OF_PROTEIN_METABOLIC_PROCESS	-1.02	0.52	0	0	0	not significant
5473	GO_EMBRYONIC_ORGAN_DEVELOPMENT	-1.02	0.45	0	0	0	not significant
5474	GO_REGULATION_OF_CELLULAR_PROTEIN_LOCALIZATION	-1.02	0.45	0	0	0	not significant
5475	GO_CELLULAR_MACROMOLECULE_LOCALIZATION	-1.02	0.42	0	0	0	not significant
5476	GO_PEPTIDE_METABOLIC_PROCESS	-1.02	0.42	0	0	0	not significant
5477	GO_POSITIVE_REGULATION_OF_CELL_CYCLE_ARREST	-1.02	0.42	0	0	0	not significant
5478	GO_POSITIVE_REGULATION_OF_CELL_DEVELOPMENT	-1.02	0.42	0	0	0	not significant
5479	GO_HEART_GROWTH	-1.02	0.41	0	0	0	not significant
5480	GO_NEGATIVE_REGULATION_OF_NEURON_PROJECTION_DEVELOPMENT	-1.02	0.41	0	0	0	not significant
5481	GO_REGULATION_OF_CELLULAR_LOCALIZATION	-1.02	0.41	0	0	0	not significant
5482	MULLIGHAN_NPM1_MUTATED_SIGNATURE_2_UP	-1.02	0.41	0	0	0	not significant
5483	PID_IL8_CXCR2_PATHWAY	-1.02	0.41	0	0	0	not significant
5484	CHR4Q32	-1.02	0.40	0	0	0	not significant
5485	DING_LUNG_CANCER_EXPRESSION_BY_COPY_NUMBER	-1.02	0.40	0	0	0	not significant
5486	JAK2_DN.V1_UP	-1.02	0.40	0	0	0	not significant
5487	REACTOME_DNA_DAMAGE_TELOMERE_STRESS_INDUCED_SENESCENCI	-1.02	0.40	0	0	0	not significant
5488	REACTOME_SIGNALING_BY_BRAF_AND_RAF_FUSIONS	-1.02	0.40	0	0	0	not significant
5489	ATF2_UP.V1_DN	-1.02	0.39	0	0	0	not significant
5490	CHIANG_LIVER_CANCER_SUBCLASS_INTERFERON_UP	-1.02	0.39	0	0	0	not significant
5491	GO_CEREBRAL_CORTEX_RADIALLY_ORIENTED_CELL_MIGRATION	-1.02	0.39	0	0	0	not significant
5492	GO_DENDRITIC_CELL_DIFFERENTIATION	-1.02	0.39	0	0	0	not significant
5493	GO_DNA_METHYLATION_OR_DEMETHYLATION	-1.02	0.39	0	0	0	not significant
5494	GO_POSITIVE_REGULATION_OF_CHROMOSOME_SEGREGATION	-1.02	0.39	0	0	0	not significant
5495	GO_PROTEIN_METHYLTRANSFERASE_ACTIVITY	-1.02	0.39	0	0	0	not significant
5496	REACTOME_RHO_GTPASES_ACTIVATE_NADPH_OXIDASES	-1.02	0.39	0	0	0	not significant
5497	SENGUPTA_NASOPHARYNGEAL_CARCINOMA_UP	-1.02	0.39	0	0	0	not significant
5498	CHR9Q22	-1.02	0.38	0	0	0	not significant
5499	GO_CELL_ADHESION_MEDIATED_BY_INTEGRIN	-1.02	0.38	0	0	0	not significant
5500	GO_CYTOPLASMIC_STRESS_GRANULE	-1.02	0.38	0	0	0	not significant
5501	GO_FILOPODIUM_ASSEMBLY	-1.02	0.38	0	0	0	not significant
5502	GO_MICROTUBULE_DEPOLYMERIZATION	-1.02	0.38	0	0	0	not significant
5503	GO_NEGATIVE_REGULATION_OF_CALCIUM_MEDIATED_SIGNALING	-1.02	0.38	0	0	0	not significant
5504	GO_NEGATIVE_REGULATION_OF_MITOCHONDRION_ORGANIZATION	-1.02	0.38	0	0	0	not significant
5505	GO_PIGMENT_BIOSYNTHETIC_PROCESS	-1.02	0.38	0	0	0	not significant
5506	GO_PIGMENT_METABOLIC_PROCESS	-1.02	0.38	0	0	0	not significant
5507	GO_POSITIVE_REGULATION_OF_CELL_SUBSTRATE_ADHESION	-1.02	0.38	0	0	0	not significant
5508	GO_REGULATION_OF_RECEPTOR_RECYCLING	-1.02	0.38	0	0	0	not significant
5509	GO_RESPONSE_TO_ESTROGEN	-1.02	0.38	0	0	0	not significant
5510	GO_VESICLE_DOCKING	-1.02	0.38	0	0	0	not significant
5511	HOWLIN_CITED1_TARGETS_1_DN	-1.02	0.38	0	0	0	not significant
5512	PID_ARF6_DOWNSTREAM_PATHWAY	-1.02	0.38	0	0	0	not significant
5513	REACTOME_FORMATION_OF_SENESCENCE_ASSOCIATED_HETEROCHR(	-1.02	0.38	0	0	0	not significant
5514	REACTOME_NEURODEGENERATIVE_DISEASES	-1.02	0.38	0	0	0	not significant
5515	GO_CARDIAC_VENTRICLE_MORPHOGENESIS	-1.02	0.37	0	0	0	not significant
5516	GO_CLATHRIN_COAT_OF_COATED_PIT	-1.02	0.37	0	0	0	not significant
5517	GO_INSULIN_SECRETION	-1.02	0.37	0	0	0	not significant
5518	GO_NEGATIVE_REGULATION_OF_TORC1_SIGNALING	-1.02	0.37	0	0	0	not significant
5519	GO_POSITIVE_REGULATION_OF_LEUKOCYTE_ADHESION_TO_VASCULAF	-1.02	0.37	0	0	0	not significant
5520	GO_VESICLE_DOCKING_INVOLVED_IN_EXOCYTOSIS	-1.02	0.37	0	0	0	not significant
5521	REACTOME_DEADENYLATION_DEPENDENT_MRNA_DECAY	-1.02	0.37	0	0	0	not significant
5522	STANELLE_E2F1_TARGETS	-1.02	0.37	0	0	0	not significant
5523	VANDESLUIS_COMMD1_TARGETS_GROUP_4_UP	-1.02	0.37	0	0	0	not significant
5524	ZUCCHI_METASTASIS_UP	-1.02	0.37	0	0	0	not significant
5525	BIOCARTA_IGF1MTOR_PATHWAY	-1.02	0.36	0	0	0	not significant
5526	GO_ARTERY_MORPHOGENESIS	-1.02	0.36	0	0	0	not significant
5527	GO_EXOCYTIC_PROCESS	-1.02	0.36	0	0	0	not significant
5528	GO_GLUCAN_CATABOLIC_PROCESS	-1.02	0.36	0	0	0	not significant
5529	GO_REGULATION_OF_T_CELL_MEDIATED_IMMUNITY	-1.02	0.36	0	0	0	not significant
5530	GO_SEMAPHORIN_RECEPTOR_ACTIVITY	-1.02	0.36	0	0	0	not significant
5531	ICHIBA_GRAFT_VERSUS_HOST_DISEASE_D7_DN	-1.02	0.36	0	0	0	not significant
5532	REACTOME_CHREBP_ACTIVATES_METABOLIC_GENE_EXPRESSION	-1.02	0.36	0	0	0	not significant
5533	GO_METALLOCHAPERONE_ACTIVITY	-1.02	0.35	0	0	0	not significant
5534	GO_NK_T_CELL_DIFFERENTIATION	-1.02	0.35	0	0	0	not significant
5535	GO_SEMAPHORIN_PLEXIN_SIGNALING_PATHWAY_INVOLVED_IN_NEURON	-1.02	0.35	0	0	0	not significant
5536	GO_ATRIAL_CARDIAC_MUSCLE_CELL_TO_AV_NODE_CELL_SIGNALING	-1.02	0.34	0	0	0	not significant
5537	GO_ICOSANOID_RECEPTOR_ACTIVITY	-1.02	0.34	0	0	0	not significant
5538	GO_NEGATIVE_REGULATION_OF_PROTEIN_IMPORT_INTO_NUCLEUS	-1.02	0.34	0	0	0	not significant
5539	GO_POSITIVE_REGULATION_OF_CELL_MATURATION	-1.02	0.34	0	0	0	not significant
5540	LEIN_MIDBRAIN_MARKERS	-1.02	0.34	0	0	0	not significant
5541	GO_AXON_INITIAL_SEGMENT	-1.02	0.33	0	0	0	not significant
5542	GO_CARDIAC_LEFT_VENTRICLE_MORPHOGENESIS	-1.02	0.33	0	0	0	not significant
5543	GO_REGULATION_OF_NEUROTROPHIN_TRK_RECEPTOR_SIGNALING_PA	-1.02	0.33	0	0	0	not significant
5544	GO_ACTIN_BASED_CELL_PROJECTION	-1.01	0.42	0	0	0	not significant
5545	GO_TRANSMEMBRANE_TRANSPORTER_ACTIVITY	-1.01	0.42	0	0	0	not significant
5546	CHR12Q13	-1.01	0.40	0	0	0	not significant
5547	GO_REGULATION_OF_ORGAN_GROWTH	-1.01	0.40	0	0	0	not significant

5548	GO_TUMOR_NECROSIS_FACTOR_RECEPTOR_SUPERFAMILY_BINDING	-1.01	0.40	0	0	0	not significant
5549	GO_CELLULAR_MACROMOLECULE_CATABOLIC_PROCESS	-1.01	0.39	0	0	0	not significant
5550	GO_INTRINSIC_APOPTOTIC_SIGNALING_PATHWAY	-1.01	0.39	0	0	0	not significant
5551	GO_MAINTENANCE_OF_CELL_NUMBER	-1.01	0.39	0	0	0	not significant
5552	GO_MICROTUBULE_BASED_PROCESS	-1.01	0.39	0	0	0	not significant
5553	JAZAG_TGFB1_SIGNALING_UP	-1.01	0.39	0	0	0	not significant
5554	JIANG_VHL_TARGETS	-1.01	0.39	0	0	0	not significant
5555	KEGG_THYROID_CANCER	-1.01	0.39	0	0	0	not significant
5556	BYSTRYKH_HEMATOPOIESIS_STEM_CELL_AND_BRAIN_QTL_TRANS	-1.01	0.38	0	0	0	not significant
5557	GAZDA_DIAMOND_BLACKFAN_ANEMIA_MYELOID_DN	-1.01	0.38	0	0	0	not significant
5558	GO_POSITIVE_REGULATION_OF_APOPTOTIC_SIGNALING_PATHWAY	-1.01	0.38	0	0	0	not significant
5559	GO POSITIVE REGULATION OF CYTOKINE PRODUCTION INVOLVED IN	-1.01	0.38	0	0	0	not significant
5560	GO REGULATION OF AMYLOID PRECURSOR PROTEIN CATABOLIC PR	-1.01	0.38	0	0	0	not significant
5561	GO_REGULATION_OF_DEVELOPMENTAL_GROWTH	-1.01	0.38	0	0	0	not significant
5562	GO_REGULATION_OF_SUPRAMOLECULAR_FIBER_ORGANIZATION	-1.01	0.38	0	0	0	not significant
5563	GO_SKELETAL_SYSTEM_MORPHOGENESIS	-1.01	0.38	0	0	0	not significant
5564	AMIT_EGF_RESPONSE_480_HELA	-1.01	0.37	0	0	0	not significant
5565	BIOCARTA_FAS_PATHWAY	-1.01	0.37	0	0	0	not significant
5566	CROMER_TUMORIGENESIS_DN	-1.01	0.37	0	0	0	not significant
5567	GO_NEGATIVE_REGULATION_OF_RESPONSE_TO_STIMULUS	-1.01	0.37	0	0	0	not significant
5568	GO_REGION_OF_CYTOSOL	-1.01	0.37	0	0	0	not significant
5569	GO REGULATION OF EXTRINSIC APOPTOTIC SIGNALING PATHWAY IN	-1.01	0.37	0	0	0	not significant
5570	GO_REGULATION_OF_INTRACELLULAR_TRANSPORT	-1.01	0.37	0	0	0	not significant
5571	KEGG_INSULIN_SIGNALING_PATHWAY	-1.01	0.37	0	0	0	not significant
5572	KUNINGER_IGF1_VS_PDGFB_TARGETS_DN	-1.01	0.37	0	0	0	not significant
5573	OKUMURA_INFLAMMATORY_RESPONSE_LPS	-1.01	0.37	0	0	0	not significant
5574	PID_VEGFR1_2_PATHWAY	-1.01	0.37	0	0	0	not significant
5575	RHEIN_ALL_GLUCOCORTICOID_THERAPY_UP	-1.01	0.37	0	0	0	not significant
5576	RIGGINS_TAMOXIFEN_RESISTANCE_DN	-1.01	0.37	0	0	0	not significant
5577	TANAKA_METHYLATED_IN_ESOPHAGEAL_CARCINOMA	-1.01	0.37	0	0	0	not significant
5578	BIOCARTA_RAC1_PATHWAY	-1.01	0.36	0	0	0	not significant
5579	BLUM_RESPONSE_TO_SALIRASIB_UP	-1.01	0.36	0	0	0	not significant
5580	BOYLAN_MULTIPLE_MYELOMA_D_UP	-1.01	0.36	0	0	0	not significant
5581	CHENG_RESPONSE_TO_NICKEL_ACETATE	-1.01	0.36	0	0	0	not significant
5582	CHIARADONNA_NEOPLASTIC_TRANSFORMATION_KRAS_DN	-1.01	0.36	0	0	0	not significant
5583	CHR3P25	-1.01	0.36	0	0	0	not significant
5584	CHR9Q21	-1.01	0.36	0	0	0	not significant
5585	E2F1_UP.V1_DN	-1.01	0.36	0	0	0	not significant
5586	GO_CATALYTIC_ACTIVITY_ACTING_ON_DNA	-1.01	0.36	0	0	0	not significant
5587	GO_ENDOCARDIUM_DEVELOPMENT	-1.01	0.36	0	0	0	not significant
5588	GO NUCLEOBASE CONTAINING SMALL MOLECULE CATABOLIC PROCE	-1.01	0.36	0	0	0	not significant
5589	GO_NUCLEOSOME_ORGANIZATION	-1.01	0.36	0	0	0	not significant
5590	GO_POSITIVE_REGULATION_OF_PROTEIN_DEPHOSPHORYLATION	-1.01	0.36	0	0	0	not significant
5591	GO_POSITIVE_REGULATION_OF_T_CELL_MIGRATION	-1.01	0.36	0	0	0	not significant
5592	GO_REGULATION_OF_STRIATED_MUSCLE_CELL_DIFFERENTIATION	-1.01	0.36	0	0	0	not significant
5593	GO_TAU_PROTEIN_BINDING	-1.01	0.36	0	0	0	not significant
5594	REACTOME_DNA_DOUBLE_STRAND_BREAK_REPAIR	-1.01	0.36	0	0	0	not significant
5595	REACTOME TP53 REGULATES TRANSCRIPTION OF CELL CYCLE GEN	-1.01	0.36	0	0	0	not significant
5596	TSENG_IRS1_TARGETS_DN	-1.01	0.36	0	0	0	not significant
5597	YAP1_UP	-1.01	0.36	0	0	0	not significant
5598	ACEVEDO_FGFR1_TARGETS_IN_PROSTATE_CANCER_MODEL_DN	-1.01	0.35	0	0	0	not significant
5599	CASTELLANO_NRAS_TARGETS_DN	-1.01	0.35	0	0	0	not significant
5600	GO_ALPHA_ACTININ_BINDING	-1.01	0.35	0	0	0	not significant
5601	GO_COLLAGEN_BIOSYNTHETIC_PROCESS	-1.01	0.35	0	0	0	not significant
5602	GO_DISORDERED_DOMAIN_SPECIFIC_BINDING	-1.01	0.35	0	0	0	not significant
5603	GO_NUCLEAR_TRANSCRIBED_MRNA_CATABOLIC_PROCESS_DEADENYL	-1.01	0.35	0	0	0	not significant
5604	GO POSITIVE REGULATION OF ALPHA BETA T CELL DIFFERENTIATIO	-1.01	0.35	0	0	0	not significant
5605	GO_REGULATION_OF_RECEPTOR_BIOSYNTHETIC_PROCESS	-1.01	0.35	0	0	0	not significant
5606	GO_RESPONSE_TO_PROSTAGLANDIN	-1.01	0.35	0	0	0	not significant
5607	GO_SEMAPHORIN_RECEPTOR_COMPLEX	-1.01	0.35	0	0	0	not significant
5608	KANNAN_TP53_TARGETS_UP	-1.01	0.35	0	0	0	not significant
5609	LABBE_TGFB1_TARGETS_DN	-1.01	0.35	0	0	0	not significant
5610	OUYANG_PROSTATE_CANCER_MARKERS	-1.01	0.35	0	0	0	not significant
5611	PHONG_TNF_RESPONSE_VIA_P38_COMPLETE	-1.01	0.35	0	0	0	not significant
5612	CHR8P21	-1.01	0.34	0	0	0	not significant
5613	GCNP_SHH_UP_EARLY.V1_DN	-1.01	0.34	0	0	0	not significant
5614	GO_COENZYME_CATABOLIC_PROCESS	-1.01	0.34	0	0	0	not significant
5615	GO_ENZYME_LINKED_RECEPTOR_PROTEIN_SIGNALING_PATHWAY	-1.01	0.34	0	0	0	not significant
5616	GO_MATING_BEHAVIOR	-1.01	0.34	0	0	0	not significant
5617	GO NEGATIVE REGULATION OF PROTEASOMAL UBIQUITIN DEPENDEN	-1.01	0.34	0	0	0	not significant
5618	GO_NEUROPEPTIDE_RECEPTOR_BINDING	-1.01	0.34	0	0	0	not significant
5619	GO_OXIDOREDUCTASE_ACTIVITY_ACTING_ON_THE_CH_CH_GROUP_OF_	-1.01	0.34	0	0	0	not significant
5620	GO_POSITIVE_REGULATION_OF_RNA_BINDING	-1.01	0.34	0	0	0	not significant
5621	GO_POSITIVE_REGULATION_OF_RUFFLE_ASSEMBLY	-1.01	0.34	0	0	0	not significant
5622	GO_REGULATION_OF_OOCYTE_MATURATION	-1.01	0.34	0	0	0	not significant
5623	GO_SMOOTH_MUSCLE_CELL_PROLIFERATION	-1.01	0.34	0	0	0	not significant
5624	REACTOME_INTERLEUKIN_17_SIGNALING	-1.01	0.34	0	0	0	not significant
5625	REACTOME NUCLEAR EVENTS KINASE AND TRANSCRIPTION FACTOR	-1.01	0.34	0	0	0	not significant
5626	GO_CHLORIDE_ION_BINDING	-1.01	0.33	0	0	0	not significant
5627	GO_POSITIVE_REGULATION_OF_TYPE_2_IMMUNE_RESPONSE	-1.01	0.33	0	0	0	not significant
5628	GO_QUINONE_METABOLIC_PROCESS	-1.01	0.33	0	0	0	not significant
5629	JOHNSTONE_PARVB_TARGETS_2_DN	-1.01	0.33	0	0	0	not significant
5630	KANG_GIST_WITH_PDGFRA_DN	-1.01	0.33	0	0	0	not significant
5631	REACTOME_SYNTHESIS_SECRETION_AND_DEACYLATION_OF_GHRELIN	-1.01	0.33	0	0	0	not significant
5632	GO_POSITIVE_REGULATION_OF_DENDRITIC_CELL_CYTOKINE_PRODUCT	-1.01	0.32	0	0	0	not significant
5633	REACTOME_CGMP_EFFECTS	-1.01	0.32	0	0	0	not significant
5634	EPPERT_LSC_R	-1.01	0.31	0	0	0	not significant
5635	MATZUK_LUTEAL_GENES	-1.01	0.30	0	0	0	not significant
5636	GO_DNA_DEALKYLATION_INVOLVED_IN_DNA_REPAIR	-1.00	0.37	0	0	0	not significant
5637	GO_POSITIVE_REGULATION_OF_BIOSYNTHETIC_PROCESS	-1.00	0.37	0	0	0	not significant
5638	GO_REGULATION_OF_INSULIN_SECRETION	-1.00	0.37	0	0	0	not significant
5639	GO_REGULATION_OF_LONG_TERM_NEURONAL_SYNAPTIC_PLASTICITY	-1.00	0.37	0	0	0	not significant
5640	KIM_WT1_TARGETS_8HR_DN	-1.00	0.37	0	0	0	not significant
5641	BIOCARTA_MTOR_PATHWAY	-1.00	0.36	0	0	0	not significant
5642	GO_ACTIVIN_RECEPTOR_SIGNALING_PATHWAY	-1.00	0.36	0	0	0	not significant
5643	GO_APOPTOTIC_MITOCHONDRIAL_CHANGES	-1.00	0.36	0	0	0	not significant
5644	GO_CEREBELLAR_CORTEX_DEVELOPMENT	-1.00	0.36	0	0	0	not significant
5645	GO_L_GLUTAMATE_TRANSMEMBRANE_TRANSPORT	-1.00	0.36	0	0	0	not significant
5646	GO_MEMBRANE_FISSION	-1.00	0.36	0	0	0	not significant
5647	GO_NEURONAL_STEM_CELL_POPULATION_MAINTENANCE	-1.00	0.36	0	0	0	not significant
5648	GO_PHOSPHATIDYLINOSITOL_3_KINASE_COMPLEX	-1.00	0.36	0	0	0	not significant
5649	GO_REGULATION_OF_MICROTUBULE_BASED_PROCESS	-1.00	0.36	0	0	0	not significant
5650	GO_TRANSCRIPTION_FACTOR_TFTC_COMPLEX	-1.00	0.36	0	0	0	not significant
5651	GO_TRANSLATION_REPRESSOR_ACTIVITY	-1.00	0.36	0	0	0	not significant
5652	LU_EZH2_TARGETS_UP	-1.00	0.36	0	0	0	not significant
5653	PID_ENDOTHELIN_PATHWAY	-1.00	0.36	0	0	0	not significant
5654	REACTOME_DISEASES_OF_METABOLISM	-1.00	0.36	0	0	0	not significant
5655	REACTOME_METABOLIC_DISORDERS_OF_BIOLOGICAL_OXIDATION_ENZ	-1.00	0.36	0	0	0	not significant
5656	REACTOME_SIGNALING_BY_VEGF	-1.00	0.36	0	0	0	not significant

5657	REACTOME_ZBP1_DAI_MEDIATED_INDUCTION_OF_TYPE_I_IFNS	-1.00	0.36	0	0	0	not significant
5658	RIZ_ERYTHROID_DIFFERENTIATION_CCNE1	-1.00	0.36	0	0	0	not significant
5659	CHR2Q12	-1.00	0.35	0	0	0	not significant
5660	GO_NEGATIVE_REGULATION_OF_CELL_SUBSTRATE_ADHESION	-1.00	0.35	0	0	0	not significant
5661	GO_NEGATIVE_REGULATION_OF_THYMOCYTE_APOPTOTIC_PROCESS	-1.00	0.35	0	0	0	not significant
5662	GO_NEURON_PROJECTION_MAINTENANCE	-1.00	0.35	0	0	0	not significant
5663	GO_POSITIVE_REGULATION_OF_OXIDATIVE_STRESS_INDUCED_CELL_DI	-1.00	0.35	0	0	0	not significant
5664	GO_REGULATION_OF_CELL_ADHESION_MEDIATED_BY_INTEGRIN	-1.00	0.35	0	0	0	not significant
5665	GO_REGULATION_OF_NMDA_RECEPTOR_ACTIVITY	-1.00	0.35	0	0	0	not significant
5666	GO_REGULATION_OF_PERK_MEDIATED_UNFOLDED_PROTEIN_RESPONS	-1.00	0.35	0	0	0	not significant
5667	GO_REGULATION_OF_PROTEIN_DEACETYLATION	-1.00	0.35	0	0	0	not significant
5668	GO_SULFUR_COMPOUND_CATABOLIC_PROCESS	-1.00	0.35	0	0	0	not significant
5669	GROSS_HYPOXIA_VIA_ELK3_ONLY_DN	-1.00	0.35	0	0	0	not significant
5670	KAYO_AGING_MUSCLE_UP	-1.00	0.35	0	0	0	not significant
5671	NIKOLSKY_BREAST_CANCER_17Q21_Q25_AMPLICON	-1.00	0.35	0	0	0	not significant
5672	BIOCARTA_P35ALZHEIMERS_PATHWAY	-1.00	0.34	0	0	0	not significant
5673	BOCHKIS_FOXA2_TARGETS	-1.00	0.34	0	0	0	not significant
5674	BOWIE_RESPONSE_TO_EXTRACELLULAR_MATRIX	-1.00	0.34	0	0	0	not significant
5675	BROWNE_HCMV_INFECTION_16HR_UP	-1.00	0.34	0	0	0	not significant
5676	CHR6P22	-1.00	0.34	0	0	0	not significant
5677	GO_ACUTE_PHASE_RESPONSE	-1.00	0.34	0	0	0	not significant
5678	GO_AMINO_ACID_IMPORT	-1.00	0.34	0	0	0	not significant
5679	GO_CILIARY_BASAL_BODY_PLASMA_MEMBRANE_DOCKING	-1.00	0.34	0	0	0	not significant
5680	GO_CORE_MEDIATOR_COMPLEX	-1.00	0.34	0	0	0	not significant
5681	GO_EYE_PHOTORECEPTOR_CELL_DIFFERENTIATION	-1.00	0.34	0	0	0	not significant
5682	GO_HORMONE_BIOSYNTHETIC_PROCESS	-1.00	0.34	0	0	0	not significant
5683	GO_NEGATIVE_REGULATION_OF_CELL_DIVISION	-1.00	0.34	0	0	0	not significant
5684	GO_NEGATIVE_REGULATION_OF_DNA_TEMPLATED_TRANSCRIPTION_EL	-1.00	0.34	0	0	0	not significant
5685	GO_NUCLEAR_OUTER_MEMBRANE_ENDOPLASMIC_RETICULUM_MEMBRA	-1.00	0.34	0	0	0	not significant
5686	GO_NUCLEOTIDE_PHOSPHORYLATION	-1.00	0.34	0	0	0	not significant
5687	GO_ORGANIC_HYDROXY_COMPOUND_TRANSPORT	-1.00	0.34	0	0	0	not significant
5688	GO_PALLIUM_DEVELOPMENT	-1.00	0.34	0	0	0	not significant
5689	GO_POSITIVE_REGULATION_OF_RECEPTOR_RECYCLING	-1.00	0.34	0	0	0	not significant
5690	GO_REGULATION_OF_ENDOPLASMIC_RETICULUM_UNFOLDED_PROTEIN_	-1.00	0.34	0	0	0	not significant
5691	GO_SKIN_DEVELOPMENT	-1.00	0.34	0	0	0	not significant
5692	GO_SNAP_RECEPTOR_ACTIVITY	-1.00	0.34	0	0	0	not significant
5693	GO_TOR_COMPLEX	-1.00	0.34	0	0	0	not significant
5694	KEGG_SULFUR_METABOLISM	-1.00	0.34	0	0	0	not significant
5695	REACTOME_PURINE_CATABOLISM	-1.00	0.34	0	0	0	not significant
5696	STEIN_ESTROGEN_RESPONSE_NOT_VIA_ESRRA	-1.00	0.34	0	0	0	not significant
5697	CHR5Q31	-1.00	0.33	0	0	0	not significant
5698	GO_DRUG_TRANSMEMBRANE_TRANSPORTER_ACTIVITY	-1.00	0.33	0	0	0	not significant
5699	GO_LEADING_EDGE_MEMBRANE	-1.00	0.33	0	0	0	not significant
5700	GO_NEGATIVE_REGULATION_OF_EPITHELIAL_CELL_PROLIFERATION_IN\	-1.00	0.33	0	0	0	not significant
5701	GO_POSITIVE_REGULATION_OF_T_CELL_CYTOKINE_PRODUCTION	-1.00	0.33	0	0	0	not significant
5702	GO_PROTON_EXPORTING_ATPASE_ACTIVITY_PHOSPHORYLATIVE_MECH	-1.00	0.33	0	0	0	not significant
5703	GO_PROTOPORPHYRINOGEN_IX_BIOSYNTHETIC_PROCESS	-1.00	0.33	0	0	0	not significant
5704	GO_REGULATION_OF_NUCLEAR_DIVISION	-1.00	0.33	0	0	0	not significant
5705	GO_RNA_7_METHYLGUANOSINE_CAP_BINDING	-1.00	0.33	0	0	0	not significant
5706	GO_TRANS_GOLGI_NETWORK_TRANSPORT_VESICLE_MEMBRANE	-1.00	0.33	0	0	0	not significant
5707	MIKHAYLOVA_OXIDATIVE_STRESS_RESPONSE_VIA_VHL_UP	-1.00	0.33	0	0	0	not significant
5708	PID_CMYB_PATHWAY	-1.00	0.33	0	0	0	not significant
5709	REACTOME_CTLA4_INHIBITORY_SIGNALING	-1.00	0.33	0	0	0	not significant
5710	REACTOME_PHASE_I_FUNCTIONALIZATION_OF_COMPOUNDS	-1.00	0.33	0	0	0	not significant
5711	GO_AP_3_ADAPTOR_COMPLEX	-1.00	0.32	0	0	0	not significant
5712	GO_CRANIAL_SUTURE_MORPHOGENESIS	-1.00	0.32	0	0	0	not significant
5713	GO_POSITIVE_REGULATION_OF_MACROPHAGE_ACTIVATION	-1.00	0.32	0	0	0	not significant
5714	GO_PYRIMIDINE_NUCLEOSIDE_CATABOLIC_PROCESS	-1.00	0.32	0	0	0	not significant
5715	GO_REGULATION_OF_CELL_PROJECTION_ORGANIZATION	-1.00	0.32	0	0	0	not significant
5716	GO_RNA_CAP_BINDING_COMPLEX	-1.00	0.32	0	0	0	not significant
5717	MARSON_BOUND_BY_FOXP3_STIMULATED	-1.00	0.32	0	0	0	not significant
5718	PURBEY_TARGETS_OF_CTBP1_NOT_SATB1_DN	-1.00	0.32	0	0	0	not significant
5719	REACTOME_DAP12_SIGNALING	-1.00	0.32	0	0	0	not significant
5720	ZHU_SKIL_TARGETS_UP	-1.00	0.32	0	0	0	not significant
5721	GO_CALMODULIN_DEPENDENT_PROTEIN_PHOSPHATASE_ACTIVITY	-1.00	0.31	0	0	0	not significant
5722	GO_CAMERA_TYPE_EYE_DEVELOPMENT	-1.00	0.31	0	0	0	not significant
5723	GO_ANDROGEN_BIOSYNTHETIC_PROCESS	-1.00	0.30	0	0	0	not significant
5724	PID_ANGIOPOIETIN_RECEPTOR_PATHWAY	-0.99	0.37	0	0	0	not significant
5725	CHR4Q24	-0.99	0.36	0	0	0	not significant
5726	GO_POSITIVE_REGULATION_OF_STRESS_ACTIVATED_PROTEIN_KINASE	-0.99	0.36	0	0	0	not significant
5727	REACTOME_APOPTOTIC_FACTOR_MEDIATED_RESPONSE	-0.99	0.36	0	0	0	not significant
5728	THILLAINADESAN_ZNF217_TARGETS_UP	-0.99	0.36	0	0	0	not significant
5729	EPPERT_HSC_R	-0.99	0.35	0	0	0	not significant
5730	GO_POSITIVE_REGULATION_OF_CELL_MATRIX_ADHESION	-0.99	0.35	0	0	0	not significant
5731	GO_POSITIVE_REGULATION_OF_LEUKOCYTE_APOPTOTIC_PROCESS	-0.99	0.35	0	0	0	not significant
5732	GO_REGULATION_OF_T_CELL_RECEPTOR_SIGNALING_PATHWAY	-0.99	0.35	0	0	0	not significant
5733	AIYAR_COBRA1_TARGETS_DN	-0.99	0.34	0	0	0	not significant
5734	BILBAN_B_CLL_LPL_UP	-0.99	0.34	0	0	0	not significant
5735	FRASOR_RESPONSE_TO_ESTRADIOL_UP	-0.99	0.34	0	0	0	not significant
5736	GLI1_UP.V1_UP	-0.99	0.34	0	0	0	not significant
5737	GO_APOPTOTIC_CELL_CLEARANCE	-0.99	0.34	0	0	0	not significant
5738	GO_GLUCOSE_IMPORT	-0.99	0.34	0	0	0	not significant
5739	GO_MACROPHAGE_DIFFERENTIATION	-0.99	0.34	0	0	0	not significant
5740	GO_RESPONSE_TO_AXON_INJURY	-0.99	0.34	0	0	0	not significant
5741	GO_RESPONSE_TO_IRON_ION	-0.99	0.34	0	0	0	not significant
5742	GO_RNA_POLYADENYLATION	-0.99	0.34	0	0	0	not significant
5743	GO_TRANSFORMING_GROWTH_FACTOR_BETA_RECEPTOR_BINDING	-0.99	0.34	0	0	0	not significant
5744	GO_VITAMIN_D_BIOSYNTHETIC_PROCESS	-0.99	0.34	0	0	0	not significant
5745	KEGG_GNRH_SIGNALING_PATHWAY	-0.99	0.34	0	0	0	not significant
5746	KOMMAGANI_TP63_GAMMA_TARGETS	-0.99	0.34	0	0	0	not significant
5747	REACTOME_INTEGRIN_SIGNALING	-0.99	0.34	0	0	0	not significant
5748	REACTOME_RA_BIOSYNTHESIS_PATHWAY	-0.99	0.34	0	0	0	not significant
5749	TONG_INTERACT_WITH_PTTG1	-0.99	0.34	0	0	0	not significant
5750	WU_HBX_TARGETS_1_DN	-0.99	0.34	0	0	0	not significant
5751	BIOCARTA_KERATINOCYTE_PATHWAY	-0.99	0.33	0	0	0	not significant
5752	CREIGHTON_ENDOCRINE_THERAPY_RESISTANCE_3	-0.99	0.33	0	0	0	not significant
5753	GO_CELLULAR_LIPID_CATABOLIC_PROCESS	-0.99	0.33	0	0	0	not significant
5754	GO_INTERLEUKIN_1_RECEPTOR_BINDING	-0.99	0.33	0	0	0	not significant
5755	GO_LAMININ_BINDING	-0.99	0.33	0	0	0	not significant
5756	GO_NEGATIVE_REGULATION_OF_VIRAL_PROCESS	-0.99	0.33	0	0	0	not significant
5757	GO_POSITIVE_REGULATION_OF_EPIDERMIS_DEVELOPMENT	-0.99	0.33	0	0	0	not significant
5758	GO_POSITIVE_REGULATION_OF_PHAGOCYTOSIS	-0.99	0.33	0	0	0	not significant
5759	GO_POSITIVE_REGULATION_OF_PROTEIN_MATURATION	-0.99	0.33	0	0	0	not significant
5760	GO_POSITIVE_REGULATION_OF_SMAD_PROTEIN_SIGNAL_TRANSDUCTIO	-0.99	0.33	0	0	0	not significant
5761	GO_REGULATION_OF_T_HELPER_CELL_DIFFERENTIATION	-0.99	0.33	0	0	0	not significant
5762	GO_RESPONSE_TO_LEUKEMIA_INHIBITORY_FACTOR	-0.99	0.33	0	0	0	not significant
5763	GO_TRACHEA_FORMATION	-0.99	0.33	0	0	0	not significant
5764	HEIDENBLAD_AMPLIFIED_IN_PANCREATIC_CANCER	-0.99	0.33	0	0	0	not significant
5765	LEE_LIVER_CANCER_MYC_E2F1_DN	-0.99	0.33	0	0	0	not significant

5766	PID_MET_PATHWAY	-0.99	0.33	0	0	0	not significant
5767	REACTOME_DEPOLYMERISATION_OF_THE_NUCLEAR_LAMINA	-0.99	0.33	0	0	0	not significant
5768	REACTOME_SUMOYLATION_OF_IMMUNE_RESPONSE_PROTEINS	-0.99	0.33	0	0	0	not significant
5769	SCHAEFFER_PROSTATE_DEVELOPMENT_AND_CANCER_BOX4_DN	-0.99	0.33	0	0	0	not significant
5770	CHANG_IMMORTALIZED_BY_HPV31_DN	-0.99	0.32	0	0	0	not significant
5771	FONTAINE_FOLLICULAR_THYROID_ADENOMA_DN	-0.99	0.32	0	0	0	not significant
5772	GO_C21_STEROID_HORMONE_METABOLIC_PROCESS	-0.99	0.32	0	0	0	not significant
5773	GO_CELL_CELL_SIGNALING_BY_WNT	-0.99	0.32	0	0	0	not significant
5774	GO_CELL_MIGRATION_INVOLVED_IN_GASTRULATION	-0.99	0.32	0	0	0	not significant
5775	GO_CELLULAR_COPPER_ION_HOMEOSTASIS	-0.99	0.32	0	0	0	not significant
5776	GO_COAGULATION	-0.99	0.32	0	0	0	not significant
5777	GO_DNA_PACKAGING	-0.99	0.32	0	0	0	not significant
5778	GO_FOLIC_ACID_METABOLIC_PROCESS	-0.99	0.32	0	0	0	not significant
5779	GO_LIPASE_ACTIVATOR_ACTIVITY	-0.99	0.32	0	0	0	not significant
5780	GO_MLL1_2_COMPLEX	-0.99	0.32	0	0	0	not significant
5781	GO_NEGATIVE_REGULATION_OF_PROTEIN_CATABOLIC_PROCESS	-0.99	0.32	0	0	0	not significant
5782	GO_PATTERN_RECOGNITION_RECEPTOR_SIGNALING_PATHWAY	-0.99	0.32	0	0	0	not significant
5783	PID_BETA_CATENIN_NUC_PATHWAY	-0.99	0.32	0	0	0	not significant
5784	REACTOME_MRNA_DECAY_BY_5_TO_3_EXORIBONUCLEASE	-0.99	0.32	0	0	0	not significant
5785	REACTOME_PERVASIVE_DEVELOPMENTAL_DISORDERS	-0.99	0.32	0	0	0	not significant
5786	REACTOME SUMOYLATION OF DNA DAMAGE RESPONSE AND REPAIR	-0.99	0.32	0	0	0	not significant
5787	SARTIPY_BLUNTED_BY_INSULIN_RESISTANCE_DN	-0.99	0.32	0	0	0	not significant
5788	GO_INTRACELLULAR_MRNA_LOCALIZATION	-0.99	0.31	0	0	0	not significant
5789	GO_NEGATIVE_REGULATION_OF_MEMBRANE_PROTEIN_ECTODOMAIN_F	-0.99	0.31	0	0	0	not significant
5790	GO_POSITIVE_REGULATION_OF_CELL_DIFFERENTIATION	-0.99	0.31	0	0	0	not significant
5791	GO POSITIVE REGULATION OF RELEASE OF CYTOCHROME C FROM	-0.99	0.31	0	0	0	not significant
5792	GO_PROGESTERONE_RECEPTOR_SIGNALING_PATHWAY	-0.99	0.31	0	0	0	not significant
5793	GO_PROSTATE_GLAND_MORPHOGENESIS	-0.99	0.31	0	0	0	not significant
5794	GO_TELOMERE_CAPPING	-0.99	0.31	0	0	0	not significant
5795	GO_ZYMOGEN_INHIBITION	-0.99	0.31	0	0	0	not significant
5796	HILLION_HMGA1B_TARGETS	-0.99	0.31	0	0	0	not significant
5797	KANG_DOXORUBICIN_RESISTANCE_DN	-0.99	0.31	0	0	0	not significant
5798	MANTOVANI_VIRAL_GPCR_SIGNALING_DN	-0.99	0.31	0	0	0	not significant
5799	PID_RHODOPSIN_PATHWAY	-0.99	0.31	0	0	0	not significant
5800	ZHAN_MULTIPLE_MYELOMA_UP	-0.99	0.31	0	0	0	not significant
5801	GO_AXO_DENDRITIC_TRANSPORT	-0.99	0.30	0	0	0	not significant
5802	GO_CARBONATE_DEHYDRATASE_ACTIVITY	-0.99	0.30	0	0	0	not significant
5803	GO_G_PROTEIN_COUPLED_SEROTONIN_RECEPTOR_BINDING	-0.99	0.30	0	0	0	not significant
5804	GO_REGULATION_OF_TRANSFERASE_ACTIVITY	-0.99	0.30	0	0	0	not significant
5805	HUMMERICH_BENIGN_SKIN_TUMOR_DN	-0.99	0.30	0	0	0	not significant
5806	SMID_BREAST_CANCER_LUMINAL_A_UP	-0.99	0.30	0	0	0	not significant
5807	GO_NEGATIVE_REGULATION_OF_PHOSPHORUS_METABOLIC_PROCESS	-0.99	0.29	0	0	0	not significant
5808	GO_POSITIVE_REGULATION_OF_LEUKOCYTE_DEGRANULATION	-0.99	0.29	0	0	0	not significant
5809	LI_WILMS_TUMOR_ANAPLASTIC_DN	-0.99	0.29	0	0	0	not significant
5810	FRASOR_RESPONSE_TO_SERM_OR_FULVESTRANT_DN	-0.98	0.34	0	0	0	not significant
5811	GO_DNA_APURINIC_OR_APYRIMIDINIC_SITE_ENDONUCLEASE_ACTIVITY	-0.98	0.34	0	0	0	not significant
5812	GO_INTESTINAL_EPITHELIAL_CELL_DEVELOPMENT	-0.98	0.34	0	0	0	not significant
5813	GO NEGATIVE REGULATION OF CELL CYCLE G1 S PHASE TRANSITIC	-0.98	0.34	0	0	0	not significant
5814	GO_NUCLEAR_PORE_COMPLEX_ASSEMBLY	-0.98	0.34	0	0	0	not significant
5815	GO_PURINE_NUCLEOSIDE_METABOLIC_PROCESS	-0.98	0.34	0	0	0	not significant
5816	GO_REGULATION_OF_T_CELL_MIGRATION	-0.98	0.34	0	0	0	not significant
5817	GO_SODIUM_ION_TRANSMEMBRANE_TRANSPORT	-0.98	0.34	0	0	0	not significant
5818	CHR10P13	-0.98	0.33	0	0	0	not significant
5819	CHR2P25	-0.98	0.33	0	0	0	not significant
5820	GO_ADA2_GCN5_ADA3_TRANSCRIPTION_ACTIVATOR_COMPLEX	-0.98	0.33	0	0	0	not significant
5821	GO_ANTIGEN_PROCESSING_AND_PRESENTATION_OF_ENDOGENOUS_AI	-0.98	0.33	0	0	0	not significant
5822	GO_GENERAL_TRANSCRIPTION_INITIATION_FACTOR_ACTIVITY	-0.98	0.33	0	0	0	not significant
5823	GO_MITOCHONDRIAL_PROTEIN_PROCESSING	-0.98	0.33	0	0	0	not significant
5824	GO_NEURON_DEATH_IN_RESPONSE_TO_OXIDATIVE_STRESS	-0.98	0.33	0	0	0	not significant
5825	GO_POSITIVE_REGULATION_OF_FILOPODIUM_ASSEMBLY	-0.98	0.33	0	0	0	not significant
5826	GO_POSITIVE_REGULATION_OF_RNA_SPLICING	-0.98	0.33	0	0	0	not significant
5827	GO_POSTREPLICATION_REPAIR	-0.98	0.33	0	0	0	not significant
5828	GO_RESPONSE_TO_PROSTAGLANDIN_E	-0.98	0.33	0	0	0	not significant
5829	GO_RNA_STEM_LOOP_BINDING	-0.98	0.33	0	0	0	not significant
5830	LANDIS_ERBB2_BREAST_TUMORS_65_DN	-0.98	0.33	0	0	0	not significant
5831	PID_AVB3_INTEGRIN_PATHWAY	-0.98	0.33	0	0	0	not significant
5832	AZARE_NEOPLASTIC_TRANSFORMATION_BY_STAT3_UP	-0.98	0.32	0	0	0	not significant
5833	CHASSOT_SKIN_WOUND	-0.98	0.32	0	0	0	not significant
5834	FIGUEROA_AML_METHYLATION_CLUSTER_2_DN	-0.98	0.32	0	0	0	not significant
5835	GO CELLULAR RESPONSE TO LOW DENSITY LIPOPROTEIN PARTICLE	-0.98	0.32	0	0	0	not significant
5836	GO_HISTONE_H4_DEACETYLATION	-0.98	0.32	0	0	0	not significant
5837	GO_INTRINSIC_APOPTOTIC_SIGNALING_PATHWAY_IN_RESPONSE_TO_O	-0.98	0.32	0	0	0	not significant
5838	GO_MITOTIC_SISTER_CHROMATID_COHESION	-0.98	0.32	0	0	0	not significant
5839	GO_NEGATIVE_REGULATION_OF_ION_TRANSPORT	-0.98	0.32	0	0	0	not significant
5840	GO NEGATIVE REGULATION OF TRANSCRIPTION ELONGATION FROM	-0.98	0.32	0	0	0	not significant
5841	GO_NEUROTRANSMITTER_BIOSYNTHETIC_PROCESS	-0.98	0.32	0	0	0	not significant
5842	GO_OUTFLOW_TRACT_SEPTUM_MORPHOGENESIS	-0.98	0.32	0	0	0	not significant
5843	GO POSITIVE REGULATION OF EPITHELIAL TO MESENCHYMAL TRANS	-0.98	0.32	0	0	0	not significant
5844	GO_POSITIVE_REGULATION_OF_MITOCHONDRIAL_OUTER_MEMBRANE_I	-0.98	0.32	0	0	0	not significant
5845	GO PROTON TRANSPORTING TWO SECTOR ATPASE COMPLEX ASSE	-0.98	0.32	0	0	0	not significant
5846	GO_REGULATION_OF_HEART_MORPHOGENESIS	-0.98	0.32	0	0	0	not significant
5847	GO_REGULATION_OF_MEIOTIC_CELL_CYCLE	-0.98	0.32	0	0	0	not significant
5848	GO_REGULATION_OF_TRANSCRIPTION_FROM_RNA_POLYMERASE_II_PR	-0.98	0.32	0	0	0	not significant
5849	KAUFFMANN_DNA_REPLICATION_GENES	-0.98	0.32	0	0	0	not significant
5850	MANN_RESPONSE_TO_AMIFOSTINE_UP	-0.98	0.32	0	0	0	not significant
5851	PIEPOLI_LGI1_TARGETS_DN	-0.98	0.32	0	0	0	not significant
5852	REACTOME_NUCLEOTIDE_SALVAGE	-0.98	0.32	0	0	0	not significant
5853	REACTOME_SHC_MEDIATED_CASCADE:FGFR4	-0.98	0.32	0	0	0	not significant
5854	ROYLANCE_BREAST_CANCER_16Q_COPY_NUMBER_UP	-0.98	0.32	0	0	0	not significant
5855	BOYAULT_LIVER_CANCER_SUBCLASS_G6_DN	-0.98	0.31	0	0	0	not significant
5856	DACOSTA_UV_RESPONSE_VIA_ERCC3_TTD_UP	-0.98	0.31	0	0	0	not significant
5857	GO_CORONARY_VASCULATURE_DEVELOPMENT	-0.98	0.31	0	0	0	not significant
5858	GO OXIDOREDUCTASE ACTIVITY ACTING ON THE CH NH2 GROUP OI	-0.98	0.31	0	0	0	not significant
5859	GO_POSITIVE_REGULATION_OF_INTERLEUKIN_6_PRODUCTION	-0.98	0.31	0	0	0	not significant
5860	GO_POSITIVE_REGULATION_OF_PROTEIN_DEACETYLATION	-0.98	0.31	0	0	0	not significant
5861	GO_PROSTANOID_RECEPTOR_ACTIVITY	-0.98	0.31	0	0	0	not significant
5862	GO_PROTEIN_LOCALIZATION_TO_CELL_PERIPHERY	-0.98	0.31	0	0	0	not significant
5863	GO REGULATION OF DNA DAMAGE RESPONSE SIGNAL TRANSDUCTIC	-0.98	0.31	0	0	0	not significant
5864	GO_REGULATION_OF_NUCLEAR_TRANSCRIBED_MRNA_POLY_A_TAIL_SI	-0.98	0.31	0	0	0	not significant
5865	GO_REGULATION_OF_PHOSPHATASE_ACTIVITY	-0.98	0.31	0	0	0	not significant
5866	MARKEY_RB1_CHRONIC_LOF_DN	-0.98	0.31	0	0	0	not significant
5867	PID_MTOR_4PATHWAY	-0.98	0.31	0	0	0	not significant
5868	REACTOME_FBXW7_MUTANTS_AND_NOTCH1_IN_CANCER	-0.98	0.31	0	0	0	not significant
5869	REACTOME TP53 REGULATES TRANSCRIPTION OF CELL DEATH GEN	-0.98	0.31	0	0	0	not significant
5870	GENTILE_UV_RESPONSE_CLUSTER_D7	-0.98	0.30	0	0	0	not significant
5871	GO_ASPARTATE_FAMILY_AMINO_ACID_CATABOLIC_PROCESS	-0.98	0.30	0	0	0	not significant
5872	GO_CAMERA_TYPE_EYE_MORPHOGENESIS	-0.98	0.30	0	0	0	not significant
5873	GO_CELLULAR_RESPONSE_TO_RADIATION	-0.98	0.30	0	0	0	not significant
5874	GO_COPPER_CHAPERONE_ACTIVITY	-0.98	0.30	0	0	0	not significant

5875	GO_MODIFICATION_OF_POSTSYNAPTIC_ACTIN_CYTOSKELETON	-0.98	0.30	0	0	0	not significant
5876	GO_NEGATIVE_REGULATION_OF_CYSTEINE_TYPE_ENDOPEPTIDASE_AC	-0.98	0.30	0	0	0	not significant
5877	GO_NEGATIVE_REGULATION_OF_KINASE_ACTIVITY	-0.98	0.30	0	0	0	not significant
5878	GO_NEGATIVE_REGULATION_OF_PHOSPHORYLATION	-0.98	0.30	0	0	0	not significant
5879	GO_REGULATION_OF_GLUCOCORTICOID_RECEPTOR_SIGNALING_PATH	-0.98	0.30	0	0	0	not significant
5880	GO_SULFURIC_ESTER_HYDROLASE_ACTIVITY	-0.98	0.30	0	0	0	not significant
5881	GO_TORC1_COMPLEX	-0.98	0.30	0	0	0	not significant
5882	GO_TRANSCRIPTION_FACTOR_TFIIH_HOLO_COMPLEX	-0.98	0.30	0	0	0	not significant
5883	GO_TRANSPORT_VESICLE_MEMBRANE	-0.98	0.30	0	0	0	not significant
5884	HASLINGER_B_CLL_WITH_MUTATED_VH_GENES	-0.98	0.30	0	0	0	not significant
5885	ONDER_CDH1_TARGETS_2_UP	-0.98	0.30	0	0	0	not significant
5886	REACTOME_SHC_MEDIATED_CASCADE:FGFR2	-0.98	0.30	0	0	0	not significant
5887	SA_G1_AND_S_PHASES	-0.98	0.30	0	0	0	not significant
5888	SESTO_RESPONSE_TO_UV_C8	-0.98	0.30	0	0	0	not significant
5889	GO_ALDEHYDE_BIOSYNTHETIC_PROCESS	-0.98	0.29	0	0	0	not significant
5890	GO_FOREBRAIN_RADIAL_GLIAL_CELL_DIFFERENTIATION	-0.98	0.29	0	0	0	not significant
5891	GO_NEDD8_TRANSFERASE_ACTIVITY	-0.98	0.29	0	0	0	not significant
5892	GO_PHOTORECEPTOR_CELL_DEVELOPMENT	-0.98	0.29	0	0	0	not significant
5893	GO_REGULATION_OF_IRE1_MEDIATED_UNFOLDED_PROTEIN_RESPONSE	-0.98	0.29	0	0	0	not significant
5894	GO_REGULATION_OF_LEUKOCYTE_ADHESION_TO_VASCULAR_ENDOTHI	-0.98	0.29	0	0	0	not significant
5895	LOPEZ_MESOTHELIOMA_SURVIVAL_DN	-0.98	0.29	0	0	0	not significant
5896	TGFB_UP.V1_UP	-0.98	0.29	0	0	0	not significant
5897	ZHOU_INFLAMMATORY_RESPONSE_LIVE_UP	-0.98	0.29	0	0	0	not significant
5898	GO_BEHAVIOR	-0.98	0.28	0	0	0	not significant
5899	GO_CARBOHYDRATE_METABOLIC_PROCESS	-0.98	0.28	0	0	0	not significant
5900	GO_MESENCHYMAL_CELL_DIFFERENTIATION	-0.98	0.28	0	0	0	not significant
5901	GO_NEGATIVE_REGULATION_OF_CATABOLIC_PROCESS	-0.98	0.28	0	0	0	not significant
5902	GO_REGULATION_OF_NEURON_PROJECTION_DEVELOPMENT	-0.98	0.28	0	0	0	not significant
5903	GO_RIBONUCLEOSIDE_MONOPHOSPHATE_BIOSYNTHETIC_PROCESS	-0.98	0.28	0	0	0	not significant
5904	GO_TOR_SIGNALING	-0.98	0.28	0	0	0	not significant
5905	REACTOME_METABOLISM_OF_NUCLEOTIDES	-0.98	0.28	0	0	0	not significant
5906	WANG_RESPONSE_TO_ANDROGEN_UP	-0.98	0.28	0	0	0	not significant
5907	GO_REGULATION_OF_CELLULAR_COMPONENT_SIZE	-0.98	0.27	0	0	0	not significant
5908	ZHANG_RESPONSE_TO_IKK_INHIBITOR_AND_TNF_DN	-0.98	0.27	0	0	0	not significant
5909	GO_RESPONSE_TO_RADIATION	-0.98	0.26	0	0	0	not significant
5910	GO_POSITIVE_REGULATION_OF_DEVELOPMENTAL_PROCESS	-0.98	0.25	0	0	0	not significant
5911	GO_NEURON_PROJECTION	-0.98	0.24	0	0	0	not significant
5912	GO_POSITIVE_REGULATION_OF_ORGANELLE_ORGANIZATION	-0.98	0.23	0	0	0	not significant
5913	GO_POSITIVE_REGULATION_OF_PHOSPHORUS_METABOLIC_PROCESS	-0.98	0.18	0	0	0	not significant
5914	GO_INTRACELLULAR_TRANSPORT	-0.98	0.15	0	0	0	not significant
5915	GO_CYTOPLASMIC_DYNEIN_COMPLEX	-0.97	0.34	0	0	0	not significant
5916	PARK_TRETINOIN_RESPONSE_AND_PML_RARA_FUSION	-0.97	0.34	0	0	0	not significant
5917	GO_REGULATION_OF_BLOOD_PRESSURE	-0.97	0.33	0	0	0	not significant
5918	CHEOK_RESPONSE_TO_MERCAPTOPURINE_UP	-0.97	0.32	0	0	0	not significant
5919	GO_ATPASE_ACTIVITY_COUPLED_TO_MOVEMENT_OF_SUBSTANCES	-0.97	0.32	0	0	0	not significant
5920	GO_POSITIVE_REGULATION_OF_TRANSCRIPTION_ELONGATION_FROM_I	-0.97	0.32	0	0	0	not significant
5921	GO_REGULATION_OF_EPIDERMAL_CELL_DIFFERENTIATION	-0.97	0.32	0	0	0	not significant
5922	REACTOME_PRE_NOTCH_EXPRESSION_AND_PROCESSING	-0.97	0.32	0	0	0	not significant
5923	REACTOME_WNT5A_DEPENDENT_INTERNALIZATION_OF_FZD4	-0.97	0.32	0	0	0	not significant
5924	GO_ACETYL_COA_METABOLIC_PROCESS	-0.97	0.31	0	0	0	not significant
5925	GO_CHROMOCENTER	-0.97	0.31	0	0	0	not significant
5926	GO_FOREBRAIN_REGIONALIZATION	-0.97	0.31	0	0	0	not significant
5927	GO_GLIAL_CELL_PROLIFERATION	-0.97	0.31	0	0	0	not significant
5928	GO_IRON_SULFUR_CLUSTER_ASSEMBLY	-0.97	0.31	0	0	0	not significant
5929	GO_NEGATIVE_REGULATION_OF_CELLULAR_PROTEIN_CATABOLIC_PRC	-0.97	0.31	0	0	0	not significant
5930	GO_NEGATIVE_REGULATION_OF_INTRACELLULAR_PROTEIN_TRANSPOF	-0.97	0.31	0	0	0	not significant
5931	GO_NEGATIVE_REGULATION_OF_MICROTUBULE_POLYMERIZATION_OR	-0.97	0.31	0	0	0	not significant
5932	GO_POSITIVE_REGULATION_OF_LEUKOCYTE_CHEMOTAXIS	-0.97	0.31	0	0	0	not significant
5933	GO_POSITIVE_REGULATION_OF_RESPONSE_TO_OXIDATIVE_STRESS	-0.97	0.31	0	0	0	not significant
5934	GO_POSITIVE_REGULATION_OF_SUPRAMOLECULAR_FIBER_ORGANIZAT	-0.97	0.31	0	0	0	not significant
5935	GO_RENAL_SYSTEM_PROCESS	-0.97	0.31	0	0	0	not significant
5936	GO_TRANSMEMBRANE_RECEPTOR_PROTEIN_TYROSINE_KINASE_ADAPT	-0.97	0.31	0	0	0	not significant
5937	OZANNE_AP1_TARGETS_UP	-0.97	0.31	0	0	0	not significant
5938	PID_SYNDECAN_1_PATHWAY	-0.97	0.31	0	0	0	not significant
5939	REACTOME_FREE_FATTY_ACIDS_REGULATE_INSULIN_SECRETION	-0.97	0.31	0	0	0	not significant
5940	REACTOME_PTK6_REGULATES_RHO_GTPASES_RAS_GTPASE_AND_MAI	-0.97	0.31	0	0	0	not significant
5941	REACTOME_TOLL_LIKE_RECEPTOR_4_TLR4_CASCADE	-0.97	0.31	0	0	0	not significant
5942	YANG_BREAST_CANCER_ESR1_BULK_UP	-0.97	0.31	0	0	0	not significant
5943	BIOCARTA_RELA_PATHWAY	-0.97	0.30	0	0	0	not significant
5944	BIOCARTA_TEL_PATHWAY	-0.97	0.30	0	0	0	not significant
5945	CAIRO_LIVER_DEVELOPMENT_UP	-0.97	0.30	0	0	0	not significant
5946	CHR4Q27	-0.97	0.30	0	0	0	not significant
5947	GALINDO_IMMUNE_RESPONSE_TO_ENTEROTOXIN	-0.97	0.30	0	0	0	not significant
5948	GO_ASPARTATE_FAMILY_AMINO_ACID_METABOLIC_PROCESS	-0.97	0.30	0	0	0	not significant
5949	GO_BLOOD_COAGULATION_INTRINSIC_PATHWAY	-0.97	0.30	0	0	0	not significant
5950	GO_CAP_INDEPENDENT_TRANSLATIONAL_INITIATION	-0.97	0.30	0	0	0	not significant
5951	GO_ESTABLISHMENT_OF_LYMPHOCYTE_POLARITY	-0.97	0.30	0	0	0	not significant
5952	GO_INOSITOL_METABOLIC_PROCESS	-0.97	0.30	0	0	0	not significant
5953	GO_NEGATIVE_REGULATION_OF_ANTIGEN_RECEPTOR_MEDIATED_SIGN	-0.97	0.30	0	0	0	not significant
5954	GO_POSITIVE_REGULATION_OF_MYOBLAST_DIFFERENTIATION	-0.97	0.30	0	0	0	not significant
5955	GO_POSITIVE_REGULATION_OF_PROTEIN_SUMOYLATION	-0.97	0.30	0	0	0	not significant
5956	GO_REGULATION_OF_PROTEIN_AUTOPHOSPHORYLATION	-0.97	0.30	0	0	0	not significant
5957	GO_REGULATION_OF_PROTEIN_TARGETING	-0.97	0.30	0	0	0	not significant
5958	GO_RESPONSE_TO_GROWTH_HORMONE	-0.97	0.30	0	0	0	not significant
5959	GO_SITE_OF_DOUBLE_STRAND_BREAK	-0.97	0.30	0	0	0	not significant
5960	HAHTOLA_MYCOSIS_FUNGOIDES_CD4_DN	-0.97	0.30	0	0	0	not significant
5961	PID_CERAMIDE_PATHWAY	-0.97	0.30	0	0	0	not significant
5962	QI_PLASMACYTOMA_DN	-0.97	0.30	0	0	0	not significant
5963	REACTOME_RIP_MEDIATED_NFKB_ACTIVATION_VIA_ZBP1	-0.97	0.30	0	0	0	not significant
5964	TING_SILENCED_BY_DICER	-0.97	0.30	0	0	0	not significant
5965	VALK_AML_WITH_11Q23_REARRANGED	-0.97	0.30	0	0	0	not significant
5966	WU_HBX_TARGETS_2_DN	-0.97	0.30	0	0	0	not significant
5967	ALONSO_METASTASIS_DN	-0.97	0.29	0	0	0	not significant
5968	BROWNE_HCMV_INFECTION_1HR_DN	-0.97	0.29	0	0	0	not significant
5969	CHOI_ATL_STAGE_PREDICTOR	-0.97	0.29	0	0	0	not significant
5970	GO_BINDING_OF_SPERM_TO_ZONA_PELLUCIDA	-0.97	0.29	0	0	0	not significant
5971	GO_CHROMOSOME_CONDENSATION	-0.97	0.29	0	0	0	not significant
5972	GO_COLUMNAR_CUBOIDAL_EPITHELIAL_CELL_DEVELOPMENT	-0.97	0.29	0	0	0	not significant
5973	GO_CORONARY_ARTERY_MORPHOGENESIS	-0.97	0.29	0	0	0	not significant
5974	GO_ENDONUCLEOLYTIC_CLEAVAGE_INVOLVED_IN_RRNA_PROCESSING	-0.97	0.29	0	0	0	not significant
5975	GO_GLUTAMINE_TRANSPORT	-0.97	0.29	0	0	0	not significant
5976	GO_NEGATIVE_REGULATION_OF_PEPTIDYL_LYSINE_ACETYLATION	-0.97	0.29	0	0	0	not significant
5977	GO_NEURON_REMODELING	-0.97	0.29	0	0	0	not significant
5978	GO_NITRIC_OXIDE_BIOSYNTHETIC_PROCESS	-0.97	0.29	0	0	0	not significant
5979	GO_POSITIVE_REGULATION_OF_PROTEIN_LOCALIZATION_TO_MEMBRAN	-0.97	0.29	0	0	0	not significant
5980	GO_POSITIVE_REGULATION_OF_RESPONSE_TO_DRUG	-0.97	0.29	0	0	0	not significant
5981	GO_POSTSYNAPTIC_CYTOSKELETON_ORGANIZATION	-0.97	0.29	0	0	0	not significant
5982	GO_REGULATION_OF_PHOSPHOLIPID_BIOSYNTHETIC_PROCESS	-0.97	0.29	0	0	0	not significant
5983	GO_RESPONSE_TO_OXYGEN_GLUCOSE_DEPRIVATION	-0.97	0.29	0	0	0	not significant

5984	REACTOME_INTRINSIC_PATHWAY_OF_FIBRIN_CLOT_FORMATION	-0.97	0.29	0	0	0	not significant
5985	CHR20P13	-0.97	0.28	0	0	0	not significant
5986	GO_COATED_VESICLE	-0.97	0.28	0	0	0	not significant
5987	GO_POSITIVE_REGULATION_OF_CELLULAR_PROTEIN_CATABOLIC_PRO(	-0.97	0.28	0	0	0	not significant
5988	GO_PROTEIN_C_TERMINUS_BINDING	-0.97	0.28	0	0	0	not significant
5989	GO_RESPONSE_TO_BMP	-0.97	0.28	0	0	0	not significant
5990	MIDORIKAWA_AMPLIFIED_IN_LIVER_CANCER	-0.97	0.28	0	0	0	not significant
5991	MIKKELSEN_MCV6_ICP_WITH_H3K27ME3	-0.97	0.28	0	0	0	not significant
5992	EGFR_UP.V1_DN	-0.97	0.26	0	0	0	not significant
5993	ESC_V6.5_UP_LATE.V1_DN	-0.97	0.26	0	0	0	not significant
5994	GO_CELLULAR_RESPONSE_TO_INSULIN_STIMULUS	-0.97	0.26	0	0	0	not significant
5995	GO_EPIDERMIS_DEVELOPMENT	-0.97	0.26	0	0	0	not significant
5996	GO_PROTEIN_N_TERMINUS_BINDING	-0.97	0.26	0	0	0	not significant
5997	REACTOME_MEIOTIC_RECOMBINATION	-0.97	0.26	0	0	0	not significant
5998	BROWNE_HCMV_INFECTION_20HR_UP	-0.97	0.25	0	0	0	not significant
5999	GO_POSITIVE_REGULATION_OF_TRANSFERASE_ACTIVITY	-0.97	0.25	0	0	0	not significant
6000	FORTSCHEGGER_PHF8_TARGETS_DN	-0.97	0.20	0	0	0	not significant
6001	GO_CARBOHYDRATE_DERIVATIVE_METABOLIC_PROCESS	-0.97	0.17	0	0	0	not significant
6002	GO_REGULATION_OF_AMINO_ACID_TRANSPORT	-0.96	0.32	0	0	0	not significant
6003	TURJANSKI_MAPK14_TARGETS	-0.96	0.32	0	0	0	not significant
6004	AMIT_EGF_RESPONSE_60_MCF10A	-0.96	0.31	0	0	0	not significant
6005	GO_G_PROTEIN_COUPLED_ACETYLCHOLINE_RECEPTOR_SIGNALING_PA	-0.96	0.31	0	0	0	not significant
6006	GO_ISOCITRATE_DEHYDROGENASE_ACTIVITY	-0.96	0.31	0	0	0	not significant
6007	GO_LONG_CHAIN_FATTY_ACID_METABOLIC_PROCESS	-0.96	0.31	0	0	0	not significant
6008	GO_MRNA_CLEAVAGE_INVOLVED_IN_MRNA_PROCESSING	-0.96	0.31	0	0	0	not significant
6009	GO_NEGATIVE_REGULATION_OF_TUMOR_NECROSIS_FACTOR_MEDIATE	-0.96	0.31	0	0	0	not significant
6010	GO_POSITIVE_REGULATION_OF_PHOSPHATASE_ACTIVITY	-0.96	0.31	0	0	0	not significant
6011	GO_PROTEIN_TRIMERIZATION	-0.96	0.31	0	0	0	not significant
6012	GO_PURINE_NUCLEOSIDE_BIOSYNTHETIC_PROCESS	-0.96	0.31	0	0	0	not significant
6013	GO_RIBOSOMAL_PROTEIN_IMPORT_INTO_NUCLEUS	-0.96	0.31	0	0	0	not significant
6014	REACTOME_MITOCHONDRIAL_FATTY_ACID_BETA_OXIDATION_OF_SATU	-0.96	0.31	0	0	0	not significant
6015	GO_CLATHRIN_COATED_ENDOCYTIC_VESICLE_MEMBRANE	-0.96	0.30	0	0	0	not significant
6016	GO_ENDORIBONUCLEASE_ACTIVITY	-0.96	0.30	0	0	0	not significant
6017	GO_MYELOID_DENDRITIC_CELL_ACTIVATION	-0.96	0.30	0	0	0	not significant
6018	GO_MYOSIN_FILAMENT	-0.96	0.30	0	0	0	not significant
6019	GO_NEGATIVE_REGULATION_OF_TOLL_LIKE_RECEPTOR_SIGNALING_PA	-0.96	0.30	0	0	0	not significant
6020	GO_NEURAL_CREST_CELL_MIGRATION	-0.96	0.30	0	0	0	not significant
6021	GO_NEURON_CELL_CELL_ADHESION	-0.96	0.30	0	0	0	not significant
6022	GO_PHOSPHOLIPASE_ACTIVATOR_ACTIVITY	-0.96	0.30	0	0	0	not significant
6023	GO_POSITIVE_REGULATION_OF_NUCLEOTIDE_BIOSYNTHETIC_PROCESS	-0.96	0.30	0	0	0	not significant
6024	GO_POSITIVE_REGULATION_OF_SISTER_CHROMATID_COHESION	-0.96	0.30	0	0	0	not significant
6025	GO_PYRIMIDINE_NUCLEOTIDE_BIOSYNTHETIC_PROCESS	-0.96	0.30	0	0	0	not significant
6026	GO_TETRAPYRROLE_BIOSYNTHETIC_PROCESS	-0.96	0.30	0	0	0	not significant
6027	JACKSON_DNMT1_TARGETS_DN	-0.96	0.30	0	0	0	not significant
6028	SCHRAETS_MLL_TARGETS_DN	-0.96	0.30	0	0	0	not significant
6029	GARGALOVIC_RESPONSE_TO_OXIDIZED_PHOSPHOLIPIDS_BLUE_DN	-0.96	0.29	0	0	0	not significant
6030	GO_CELL_CELL_CONTACT_ZONE	-0.96	0.29	0	0	0	not significant
6031	GO_CELLULAR_HORMONE_METABOLIC_PROCESS	-0.96	0.29	0	0	0	not significant
6032	GO_NEGATIVE_REGULATION_OF_MICROTUBULE_DEPOLYMERIZATION	-0.96	0.29	0	0	0	not significant
6033	GO_POSTSYNAPTIC_ACTIN_CYTOSKELETON_ORGANIZATION	-0.96	0.29	0	0	0	not significant
6034	GO_REGULATION_OF_CELLULAR_RESPONSE_TO_INSULIN_STIMULUS	-0.96	0.29	0	0	0	not significant
6035	GO_REGULATION_OF_CHEMOKINE_C_X_C_MOTIF_LIGAND_2_PRODUCTI	-0.96	0.29	0	0	0	not significant
6036	GO_REGULATION_OF_COLLATERAL_SPROUTING	-0.96	0.29	0	0	0	not significant
6037	GO_REGULATION_OF_GASTRULATION	-0.96	0.29	0	0	0	not significant
6038	GO_RESPONSE_TO_ACETYLCHOLINE	-0.96	0.29	0	0	0	not significant
6039	GO_U6_SNRNA_BINDING	-0.96	0.29	0	0	0	not significant
6040	KRIEG_HYPOXIA_VIA_KDM3A	-0.96	0.29	0	0	0	not significant
6041	REACTOME_BASE_EXCISION_REPAIR	-0.96	0.29	0	0	0	not significant
6042	REACTOME_GASTRIN_CREB_SIGNALLING_PATHWAY_VIA_PKC_AND_MAF	-0.96	0.29	0	0	0	not significant
6043	REACTOME_SIGNALING_BY_NTRK1_TRKA	-0.96	0.29	0	0	0	not significant
6044	RIZ_ERYTHROID_DIFFERENTIATION	-0.96	0.29	0	0	0	not significant
6045	SCHAVOLT_TARGETS_OF_TP53_AND_TP63	-0.96	0.29	0	0	0	not significant
6046	VANASSE_BCL2_TARGETS_UP	-0.96	0.29	0	0	0	not significant
6047	WENG_POR_DOSAGE	-0.96	0.29	0	0	0	not significant
6048	GO_EPHRIN_RECEPTOR_ACTIVITY	-0.96	0.28	0	0	0	not significant
6049	GO_EXTRINSIC_APOPTOTIC_SIGNALING_PATHWAY	-0.96	0.28	0	0	0	not significant
6050	GO_POLYSACCHARIDE_BINDING	-0.96	0.28	0	0	0	not significant
6051	GO_POST_EMBRYONIC_CAMERA_TYPE_EYE_DEVELOPMENT	-0.96	0.28	0	0	0	not significant
6052	GO_PURINE_NUCLEOSIDE_DIPHOSPHATE_METABOLIC_PROCESS	-0.96	0.28	0	0	0	not significant
6053	GO_REGULATION_OF_OOGENESIS	-0.96	0.28	0	0	0	not significant
6054	GO_RETINA_MORPHOGENESIS_IN_CAMERA_TYPE_EYE	-0.96	0.28	0	0	0	not significant
6055	GO_SNORNA_LOCALIZATION	-0.96	0.28	0	0	0	not significant
6056	GO_SPERM_CHROMATIN_CONDENSATION	-0.96	0.28	0	0	0	not significant
6057	GO_STEROL_TRANSPORT	-0.96	0.28	0	0	0	not significant
6058	HOOI_ST7_TARGETS_UP	-0.96	0.28	0	0	0	not significant
6059	MAYBURD_RESPONSE_TO_L663536_UP	-0.96	0.28	0	0	0	not significant
6060	MORI_PLASMA_CELL_UP	-0.96	0.28	0	0	0	not significant
6061	NAM_FXYD5_TARGETS_DN	-0.96	0.28	0	0	0	not significant
6062	REACTOME_EGFR_TRANSACTIVATION_BY_GASTRIN	-0.96	0.28	0	0	0	not significant
6063	REACTOME_REPRESSION_OF_WNT_TARGET_GENES	-0.96	0.28	0	0	0	not significant
6064	SIG_INSULIN_RECEPTOR_PATHWAY_IN_CARDIAC_MYOCYTES	-0.96	0.28	0	0	0	not significant
6065	ZAMORA_NOS2_TARGETS_UP	-0.96	0.28	0	0	0	not significant
6066	BOYLAN_MULTIPLE_MYELOMA_PCA3_DN	-0.96	0.27	0	0	0	not significant
6067	GO_LIMBIC_SYSTEM_DEVELOPMENT	-0.96	0.27	0	0	0	not significant
6068	GO_NURD_COMPLEX	-0.96	0.27	0	0	0	not significant
6069	GO_REGULATION_OF_PROTEASOMAL_UBIQUITIN_DEPENDENT_PROTEIN	-0.96	0.27	0	0	0	not significant
6070	GO_REGULATION_OF_STRESS_ACTIVATED_PROTEIN_KINASE_SIGNALIN(	-0.96	0.27	0	0	0	not significant
6071	GO_RRNA_3_END_PROCESSING	-0.96	0.27	0	0	0	not significant
6072	HOLLERN_EMT_BREAST_TUMOR_UP	-0.96	0.27	0	0	0	not significant
6073	MCCLUNG_COCAINE_REWARD_5D	-0.96	0.27	0	0	0	not significant
6074	MELLMAN_TUT1_TARGETS_DN	-0.96	0.27	0	0	0	not significant
6075	NAKAYAMA_SOFT_TISSUE_TUMORS_PCA1_DN	-0.96	0.27	0	0	0	not significant
6076	PID_LKB1_PATHWAY	-0.96	0.27	0	0	0	not significant
6077	VETTER_TARGETS_OF_PRKCA_AND_ETS1_UP	-0.96	0.27	0	0	0	not significant
6078	BROWNE_HCMV_INFECTION_10HR_DN	-0.96	0.26	0	0	0	not significant
6079	CASTELLANO_NRAS_TARGETS_UP	-0.96	0.26	0	0	0	not significant
6080	GO_BIOMINERAL_TISSUE_DEVELOPMENT	-0.96	0.26	0	0	0	not significant
6081	GO_PROTEIN_TAG	-0.96	0.26	0	0	0	not significant
6082	HALLMARK_SPERMATOGENESIS	-0.96	0.26	0	0	0	not significant
6083	RAF_UP.V1_UP	-0.96	0.26	0	0	0	not significant
6084	GO_NUCLEAR_PERIPHERY	-0.96	0.25	0	0	0	not significant
6085	KATSANOU_ELAVL1_TARGETS_DN	-0.96	0.25	0	0	0	not significant
6086	SANSOM_WNT_PATHWAY_REQUIRE_MYC	-0.96	0.25	0	0	0	not significant
6087	REACTOME_CILIUM_ASSEMBLY	-0.96	0.24	0	0	0	not significant
6088	GO_NEGATIVE_REGULATION_OF_NERVOUS_SYSTEM_DEVELOPMENT	-0.96	0.22	0	0	0	not significant
6089	GO_POSITIVE_REGULATION_OF_NERVOUS_SYSTEM_DEVELOPMENT	-0.96	0.22	0	0	0	not significant
6090	GO_PROTEIN_DOMAIN_SPECIFIC_BINDING	-0.96	0.22	0	0	0	not significant
6091	PILON_KLF1_TARGETS_UP	-0.96	0.22	0	0	0	not significant
6092	GO_CATION_TRANSMEMBRANE_TRANSPORTER_ACTIVITY	-0.96	0.21	0	0	0	not significant

6093	GO_ORGANELLE_FISSION	-0.96	0.21	0	0	0	not significant
6094	GO_MICROTUBULE_CYTOSKELETON_ORGANIZATION	-0.96	0.20	0	0	0	not significant
6095	YAGI_AML_WITH_INV_16_TRANSLOCATION	-0.96	0.20	0	0	0	not significant
6096	HAN_SATB1_TARGETS_UP	-0.96	0.19	0	0	0	not significant
6097	DACOSTA_UV_RESPONSE_VIA_ERCC3_COMMON_DN	-0.96	0.17	0	0	0	not significant
6098	GO_REGULATION_OF_KINASE_ACTIVITY	-0.96	0.12	0	0	0	not significant
6099	GO_POSITIVE_REGULATION_OF_NITRIC_OXIDE_BIOSYNTHETIC_PROCES	-0.95	0.31	0	0	0	not significant
6100	GO_FATTY_ACID_DERIVATIVE_METABOLIC_PROCESS	-0.95	0.30	0	0	0	not significant
6101	GO_NEGATIVE_REGULATION_OF_CYTOKINE_SECRETION	-0.95	0.30	0	0	0	not significant
6102	GO_NUCLEOSIDE_TRIPHOSPHATE_DIPHOSPHATASE_ACTIVITY	-0.95	0.30	0	0	0	not significant
6103	GO_PROTEIN_LOCALIZATION_TO_PRESYNAPSE	-0.95	0.30	0	0	0	not significant
6104	GO_SYNAPTIC_TRANSMISSION_CHOLINERGIC	-0.95	0.30	0	0	0	not significant
6105	MCGARVEY_SILENCED_BY_METHYLATION_IN_COLON_CANCER	-0.95	0.30	0	0	0	not significant
6106	NAGY_PCAF_COMPONENTS_HUMAN	-0.95	0.30	0	0	0	not significant
6107	BILBAN_B_CLL_LPL_DN	-0.95	0.29	0	0	0	not significant
6108	CHR1P12	-0.95	0.29	0	0	0	not significant
6109	GO_ACTIVATION_OF_CYSTEINE_TYPE_ENDOPEPTIDASE_ACTIVITY_INVO	-0.95	0.29	0	0	0	not significant
6110	GO_LIPOPOLYSACCHARIDE_BINDING	-0.95	0.29	0	0	0	not significant
6111	GO_REGULATION_OF_EPIDERMIS_DEVELOPMENT	-0.95	0.29	0	0	0	not significant
6112	GO_SURFACTANT_HOMEOSTASIS	-0.95	0.29	0	0	0	not significant
6113	GO_UBIQUITINATION_LIKE_MODIFICATION_DEPENDENT_PROTEIN_BINDIN	-0.95	0.29	0	0	0	not significant
6114	GO_V_D_J_RECOMBINATION	-0.95	0.29	0	0	0	not significant
6115	KEGG_HISTIDINE_METABOLISM	-0.95	0.29	0	0	0	not significant
6116	KYNG_ENVIRONMENTAL_STRESS_RESPONSE_NOT_BY_GAMMA_IN_WS	-0.95	0.29	0	0	0	not significant
6117	WEINMANN_ADAPTATION_TO_HYPOXIA_UP	-0.95	0.29	0	0	0	not significant
6118	AMIT_EGF_RESPONSE_40_HELA	-0.95	0.28	0	0	0	not significant
6119	GO_9PLUS0_NON_MOTILE_CILIUM	-0.95	0.28	0	0	0	not significant
6120	GO_CHLORIDE_CHANNEL_REGULATOR_ACTIVITY	-0.95	0.28	0	0	0	not significant
6121	GO_EKC_KEOPS_COMPLEX	-0.95	0.28	0	0	0	not significant
6122	GO_REGULATION_OF_MRNA_BINDING	-0.95	0.28	0	0	0	not significant
6123	GO_TORC2_COMPLEX	-0.95	0.28	0	0	0	not significant
6124	GO_TRANSLESION_SYNTHESIS	-0.95	0.28	0	0	0	not significant
6125	HUPER_BREAST_BASAL_VS_LUMINAL_DN	-0.95	0.28	0	0	0	not significant
6126	KONDO_PROSTATE_CANCER_HCP_WITH_H3K27ME3	-0.95	0.28	0	0	0	not significant
6127	KRASNOSELSKAYA_ILF3_TARGETS_UP	-0.95	0.28	0	0	0	not significant
6128	BIOCARTA_AHSP_PATHWAY	-0.95	0.27	0	0	0	not significant
6129	GO_MYOSIN_FILAMENT_ORGANIZATION	-0.95	0.27	0	0	0	not significant
6130	GO_REGULATION_OF_GLIAL_CELL_MIGRATION	-0.95	0.27	0	0	0	not significant
6131	GO_SOLUTE_PROTON_SYMPORTER_ACTIVITY	-0.95	0.27	0	0	0	not significant
6132	KEGG_RIG_I_LIKE_RECEPTOR_SIGNALING_PATHWAY	-0.95	0.27	0	0	0	not significant
6133	MODY_HIPPOCAMPUS_PRENATAL	-0.95	0.27	0	0	0	not significant
6134	REACTOME_GAB1_SIGNALOSOME	-0.95	0.27	0	0	0	not significant
6135	REACTOME_MET_ACTIVATES_RAS_SIGNALING	-0.95	0.27	0	0	0	not significant
6136	ROZANOV_MMP14_CORRELATED	-0.95	0.27	0	0	0	not significant
6137	TSAI_RESPONSE_TO_RADIATION_THERAPY	-0.95	0.27	0	0	0	not significant
6138	WIERENGA_STAT5A_TARGETS_GROUP2	-0.95	0.27	0	0	0	not significant
6139	GO_DNA_MODIFICATION	-0.95	0.26	0	0	0	not significant
6140	GO_N_TERMINAL_PROTEIN_AMINO_ACID_MODIFICATION	-0.95	0.26	0	0	0	not significant
6141	GO_POSITIVE_REGULATION_OF_LYMPHOCYTE_MIGRATION	-0.95	0.26	0	0	0	not significant
6142	GO_REGULATION_OF_SPINDLE_ASSEMBLY	-0.95	0.26	0	0	0	not significant
6143	GO_VENTRICULAR_SEPTUM_DEVELOPMENT	-0.95	0.26	0	0	0	not significant
6144	NING_CHRONIC_OBSTRUCTIVE_PULMONARY_DISEASE_DN	-0.95	0.26	0	0	0	not significant
6145	XU_GH1_EXOGENOUS_TARGETS_DN	-0.95	0.26	0	0	0	not significant
6146	AUNG_GASTRIC_CANCER	-0.95	0.25	0	0	0	not significant
6147	GO_POSITIVE_REGULATION_OF_BLOOD_VESSEL_ENDOTHELIAL_CELL_I	-0.95	0.25	0	0	0	not significant
6148	GO_REGULATION_OF_TRANSMEMBRANE_RECEPTOR_PROTEIN_SERINE_	-0.95	0.25	0	0	0	not significant
6149	GO_TERTIARY_GRANULE	-0.95	0.25	0	0	0	not significant
6150	INGA_TP53_TARGETS	-0.95	0.25	0	0	0	not significant
6151	KRAS.KIDNEY_UP.V1_UP	-0.95	0.25	0	0	0	not significant
6152	PUJANA_XPRSS_INT_NETWORK	-0.95	0.25	0	0	0	not significant
6153	REACTOME_DISEASES_ASSOCIATED_WITH_GLYCOSAMINOGLYCAN_MET	-0.95	0.25	0	0	0	not significant
6154	GO_FAT_CELL_DIFFERENTIATION	-0.95	0.24	0	0	0	not significant
6155	GO_L_GLUTAMINE_TRANSMEMBRANE_TRANSPORTER_ACTIVITY	-0.95	0.24	0	0	0	not significant
6156	GO_REGULATION_OF_REGULATED_SECRETORY_PATHWAY	-0.95	0.24	0	0	0	not significant
6157	GO_CLATHRIN_COATED_VESICLE	-0.95	0.23	0	0	0	not significant
6158	GO_INSULIN_RECEPTOR_SIGNALING_PATHWAY	-0.95	0.23	0	0	0	not significant
6159	GO_GOLGI_ASSOCIATED_VESICLE_MEMBRANE	-0.95	0.22	0	0	0	not significant
6160	ACEVEDO_METHYLATED_IN_LIVER_CANCER_DN	-0.95	0.17	0	0	0	not significant
6161	GO_GTPASE_REGULATOR_ACTIVITY	-0.95	0.16	0	0	0	not significant
6162	GO_CELLULAR_COMPONENT_MORPHOGENESIS	-0.95	0.14	0	0	0	not significant
6163	GO_ENDOPLASMIC_RETICULUM_PART	-0.95	0.10	0	0	0	not significant
6164	GO_MACROMOLECULE_CATABOLIC_PROCESS	-0.95	0.10	0	0	0	not significant
6165	GO_NEURON_PART	-0.95	0.09	0	0	0	not significant
6166	GO_NEURAL_RETINA_DEVELOPMENT	-0.94	0.30	0	0	0	not significant
6167	ABDULRAHMAN_KIDNEY_CANCER_VHL_DN	-0.94	0.29	0	0	0	not significant
6168	FERRARI_RESPONSE_TO_FENRETINIDE_UP	-0.94	0.29	0	0	0	not significant
6169	GO_MODIFICATION_OF_POSTSYNAPTIC_STRUCTURE	-0.94	0.29	0	0	0	not significant
6170	GO_NEGATIVE_REGULATION_OF_POSTTRANSCRIPTIONAL_GENE_SILEN(	-0.94	0.29	0	0	0	not significant
6171	GO_T_CELL_HOMEOSTASIS	-0.94	0.29	0	0	0	not significant
6172	TSUNODA_CISPLATIN_RESISTANCE_UP	-0.94	0.29	0	0	0	not significant
6173	ZEMBUTSU_SENSITIVITY_TO_NIMUSTINE	-0.94	0.29	0	0	0	not significant
6174	GENTILE_UV_RESPONSE_CLUSTER_D9	-0.94	0.28	0	0	0	not significant
6175	GO_CLASS_I_DNA_APURINIC_OR_APYRIMIDINIC_SITE_ENDONUCLEASE_A	-0.94	0.28	0	0	0	not significant
6176	GO_REGULATION_OF_HISTONE_DEACETYLATION	-0.94	0.28	0	0	0	not significant
6177	GO_SMAD_PROTEIN_SIGNAL_TRANSDUCTION	-0.94	0.28	0	0	0	not significant
6178	HOFMANN_CELL_LYMPHOMA_UP	-0.94	0.28	0	0	0	not significant
6179	REACTOME_CROSSLINKING_OF_COLLAGEN_FIBRILS	-0.94	0.28	0	0	0	not significant
6180	REACTOME_SYNTHESIS_OF_WYBUTOSINE_AT_G37_OF_TRNA_PHE	-0.94	0.28	0	0	0	not significant
6181	ZEMBUTSU_SENSITIVITY_TO_VINCRISTINE	-0.94	0.28	0	0	0	not significant
6182	BIOCARTA_LIS1_PATHWAY	-0.94	0.27	0	0	0	not significant
6183	GERHOLD_ADIPOGENESIS_DN	-0.94	0.27	0	0	0	not significant
6184	GO_AMYLOID_BETA_CLEARANCE_BY_TRANSCYTOSIS	-0.94	0.27	0	0	0	not significant
6185	GO_CALCINEURIN_MEDIATED_SIGNALING	-0.94	0.27	0	0	0	not significant
6186	GO_CELL_MOTILITY_INVOLVED_IN_CEREBRAL_CORTEX_RADIAL_GLIA_G	-0.94	0.27	0	0	0	not significant
6187	GO_LATERAL_PLASMA_MEMBRANE	-0.94	0.27	0	0	0	not significant
6188	GO_NEGATIVE_REGULATION_OF_TELOMERE_MAINTENANCE	-0.94	0.27	0	0	0	not significant
6189	GO_NUCLEOTIDE_ACTIVATED_PROTEIN_KINASE_COMPLEX	-0.94	0.27	0	0	0	not significant
6190	GO_PHOSPHATIDYLINOSITOL_BISPHOSPHATE_PHOSPHATASE_ACTIVITY	-0.94	0.27	0	0	0	not significant
6191	GO_POSITIVE_REGULATION_OF_KIDNEY_DEVELOPMENT	-0.94	0.27	0	0	0	not significant
6192	GO_REGULATION_OF_FEEDING_BEHAVIOR	-0.94	0.27	0	0	0	not significant
6193	GO_REGULATION_OF_NECROTIC_CELL_DEATH	-0.94	0.27	0	0	0	not significant
6194	GO_REGULATION_OF_TRANSMISSION_OF_NERVE_IMPULSE	-0.94	0.27	0	0	0	not significant
6195	KEGG_ALANINE_ASPARTATE_AND_GLUTAMATE_METABOLISM	-0.94	0.27	0	0	0	not significant
6196	ROZANOV_MMP14_TARGETS_DN	-0.94	0.27	0	0	0	not significant
6197	ZHU_SKIL_TARGETS_DN	-0.94	0.27	0	0	0	not significant
6198	ABRAHAM_ALPC_VS_MULTIPLE_MYELOMA_DN	-0.94	0.26	0	0	0	not significant
6199	BROWNE_HCMV_INFECTION_30MIN_DN	-0.94	0.26	0	0	0	not significant
6200	DAWSON_METHYLATED_IN_LYMPHOMA_TCL1	-0.94	0.26	0	0	0	not significant
6201	FINETTI_BREAST_CANCERS_KINOME_BLUE	-0.94	0.26	0	0	0	not significant

6202	GO_MYD88_DEPENDENT_TOLL_LIKE_RECEPTOR_SIGNALING_PATHWAY	-0.94	0.26	0	0	0	not significant
6203	GO_NEGATIVE_REGULATION_OF_INTRACELLULAR_STEROID_HORMONE_	-0.94	0.26	0	0	0	not significant
6204	GO_NUCLEOBASE_CONTAINING_COMPOUND_KINASE_ACTIVITY	-0.94	0.26	0	0	0	not significant
6205	GO_REGULATION_OF_BONE_DEVELOPMENT	-0.94	0.26	0	0	0	not significant
6206	GO_REGULATION_OF_CELL_COMMUNICATION_BY_ELECTRICAL_COUPLI	-0.94	0.26	0	0	0	not significant
6207	GO_REGULATION_OF_LYMPHOCYTE_MIGRATION	-0.94	0.26	0	0	0	not significant
6208	KANG_FLUOROURACIL_RESISTANCE_UP	-0.94	0.26	0	0	0	not significant
6209	KIM_LIVER_CANCER_POOR_SURVIVAL_UP	-0.94	0.26	0	0	0	not significant
6210	LIU_VAV3_PROSTATE_CARCINOGENESIS_UP	-0.94	0.26	0	0	0	not significant
6211	MARSON_FOXP3_TARGETS_STIMULATED_UP	-0.94	0.26	0	0	0	not significant
6212	REACTOME_VOLTAGE_GATED_POTASSIUM_CHANNELS	-0.94	0.26	0	0	0	not significant
6213	CHR11P11	-0.94	0.25	0	0	0	not significant
6214	CHR1P13	-0.94	0.25	0	0	0	not significant
6215	GO_NEGATIVE_REGULATION_OF_SYNAPSE_ORGANIZATION	-0.94	0.25	0	0	0	not significant
6216	GO_POSITIVE_REGULATION_OF_CELL_CYCLE_G1_S_PHASE_TRANSITIO	-0.94	0.25	0	0	0	not significant
6217	GO_PROTEIN_LIPID_COMPLEX_SUBUNIT_ORGANIZATION	-0.94	0.25	0	0	0	not significant
6218	GO_REGULATION_OF_ADHERENS_JUNCTION_ORGANIZATION	-0.94	0.25	0	0	0	not significant
6219	PID_TAP63_PATHWAY	-0.94	0.25	0	0	0	not significant
6220	PUJANA_BREAST_CANCER_LIT_INT_NETWORK	-0.94	0.25	0	0	0	not significant
6221	REACTOME_PLASMA_LIPOPROTEIN_CLEARANCE	-0.94	0.25	0	0	0	not significant
6222	GO_HEART_VALVE_DEVELOPMENT	-0.94	0.24	0	0	0	not significant
6223	GO_MITOTIC_SPINDLE	-0.94	0.24	0	0	0	not significant
6224	GO_POSITIVE_REGULATION_OF_PROTEOLYSIS_INVOLVED_IN_CELLULAF	-0.94	0.24	0	0	0	not significant
6225	REACTOME_METABOLISM_OF_WATER_SOLUBLE_VITAMINS_AND_COFAC	-0.94	0.24	0	0	0	not significant
6226	SIRNA_EIF4GI_UP	-0.94	0.24	0	0	0	not significant
6227	BYSTRYKH_HEMATOPOIESIS_STEM_CELL_QTL_CIS	-0.94	0.23	0	0	0	not significant
6228	DITTMER_PTHLH_TARGETS_DN	-0.94	0.23	0	0	0	not significant
6229	GO_NEGATIVE_REGULATION_OF_DNA_BINDING_TRANSCRIPTION_FACTC	-0.94	0.23	0	0	0	not significant
6230	GO_NUCLEOSIDE_METABOLIC_PROCESS	-0.94	0.23	0	0	0	not significant
6231	HOSHIDA_LIVER_CANCER_SURVIVAL_DN	-0.94	0.23	0	0	0	not significant
6232	GO_GLUCOSE_METABOLIC_PROCESS	-0.94	0.22	0	0	0	not significant
6233	GO_POSITIVE_REGULATION_OF_JNK_CASCADE	-0.94	0.22	0	0	0	not significant
6234	GO_REGULATION_OF_MRNA_SPLICING_VIA_SPLICEOSOME	-0.94	0.22	0	0	0	not significant
6235	GO_REGULATION_OF_NEUROTRANSMITTER_LEVELS	-0.94	0.22	0	0	0	not significant
6236	GO_SPECIFIC_GRANULE	-0.94	0.22	0	0	0	not significant
6237	NIKOLSKY_BREAST_CANCER_8Q12_Q22_AMPLICON	-0.94	0.22	0	0	0	not significant
6238	RAY_TUMORIGENESIS_BY_ERBB2_CDC25A_UP	-0.94	0.22	0	0	0	not significant
6239	REACTOME_RHO_GTPASE_CYCLE	-0.94	0.22	0	0	0	not significant
6240	SMIRNOV_RESPONSE_TO_IR_6HR_UP	-0.94	0.22	0	0	0	not significant
6241	GO_CELL_PROJECTION_MEMBRANE	-0.94	0.21	0	0	0	not significant
6242	GO_MULTICELLULAR_ORGANISM_GROWTH	-0.94	0.21	0	0	0	not significant
6243	GO_NEGATIVE_REGULATION_OF_CELL_PROJECTION_ORGANIZATION	-0.94	0.21	0	0	0	not significant
6244	GO_POSITIVE_REGULATION_OF_MITOTIC_CELL_CYCLE	-0.94	0.21	0	0	0	not significant
6245	PID_TELOMERASE_PATHWAY	-0.94	0.21	0	0	0	not significant
6246	GO_TRANSPORT_VESICLE	-0.94	0.20	0	0	0	not significant
6247	GCNP_SHH_UP_EARLY.V1_UP	-0.94	0.19	0	0	0	not significant
6248	GO_STEROID_HORMONE_MEDIATED_SIGNALING_PATHWAY	-0.94	0.19	0	0	0	not significant
6249	GO_CELLULAR_RESPONSE_TO_PEPTIDE	-0.94	0.18	0	0	0	not significant
6250	GO_MAGNESIUM_ION_BINDING	-0.94	0.18	0	0	0	not significant
6251	GO_POSITIVE_REGULATION_OF_KINASE_ACTIVITY	-0.94	0.17	0	0	0	not significant
6252	LU_EZH2_TARGETS_DN	-0.94	0.16	0	0	0	not significant
6253	OSMAN_BLADDER_CANCER_UP	-0.94	0.15	0	0	0	not significant
6254	GO_CHROMOSOME_ORGANIZATION	-0.94	0.07	0	0	0	not significant
6255	GO_CALCIUM_DEPENDENT_PROTEIN_SERINE_THREONINE_PHOSPHATAS	-0.93	0.29	0	0	0	not significant
6256	GO_NEGATIVE_REGULATION_OF_MYELOID_LEUKOCYTE_MEDIATED_IMM	-0.93	0.28	0	0	0	not significant
6257	GO_POLY_PURINE_TRACT_BINDING	-0.93	0.28	0	0	0	not significant
6258	LEE_NAIVE_T_LYMPHOCYTE	-0.93	0.28	0	0	0	not significant
6259	NUNODA_RESPONSE_TO_DASATINIB_IMATINIB_DN	-0.93	0.28	0	0	0	not significant
6260	BIOCARTA_THELPER_PATHWAY	-0.93	0.27	0	0	0	not significant
6261	CHR10Q11	-0.93	0.27	0	0	0	not significant
6262	GO_DNA_METHYLTRANSFERASE_ACTIVITY	-0.93	0.27	0	0	0	not significant
6263	GO_LABYRINTHINE_LAYER_BLOOD_VESSEL_DEVELOPMENT	-0.93	0.27	0	0	0	not significant
6264	GO_NEGATIVE_REGULATION_OF_OXIDATIVE_STRESS_INDUCED_INTRINS	-0.93	0.27	0	0	0	not significant
6265	GO_STRIATUM_DEVELOPMENT	-0.93	0.27	0	0	0	not significant
6266	GO_TRANSLATIONAL_READTHROUGH	-0.93	0.27	0	0	0	not significant
6267	NIELSEN_LEIOMYOSARCOMA_CNN1_DN	-0.93	0.27	0	0	0	not significant
6268	NIKOLSKY_BREAST_CANCER_1Q21_AMPLICON	-0.93	0.27	0	0	0	not significant
6269	PYEON_CANCER_HEAD_AND_NECK_VS_CERVICAL_DN	-0.93	0.27	0	0	0	not significant
6270	REACTOME_CASPASE_ACTIVATION_VIA_DEATH_RECEPTORS_IN_THE_PI	-0.93	0.27	0	0	0	not significant
6271	BIOCARTA_P53HYPOXIA_PATHWAY	-0.93	0.26	0	0	0	not significant
6272	FIRESTEIN_CTNNB1_PATHWAY_AND_PROLIFERATION	-0.93	0.26	0	0	0	not significant
6273	GO_ARTERY_DEVELOPMENT	-0.93	0.26	0	0	0	not significant
6274	GO_CAMP_DEPENDENT_PROTEIN_KINASE_REGULATOR_ACTIVITY	-0.93	0.26	0	0	0	not significant
6275	GO_EPITHELIAL_CELL_PROLIFERATION_INVOLVED_IN_PROSTATE_GLAN	-0.93	0.26	0	0	0	not significant
6276	GO_ESTABLISHMENT_OR_MAINTENANCE_OF_BIPOLAR_CELL_POLARITY	-0.93	0.26	0	0	0	not significant
6277	GO_FOLIC_ACID_CONTAINING_COMPOUND_BIOSYNTHETIC_PROCESS	-0.93	0.26	0	0	0	not significant
6278	GO_FUCOSYLATION	-0.93	0.26	0	0	0	not significant
6279	GO_MICROTUBULE_ORGANIZING_CENTER_LOCALIZATION	-0.93	0.26	0	0	0	not significant
6280	GO_NEGATIVE_REGULATION_OF_KERATINOCYTE_PROLIFERATION	-0.93	0.26	0	0	0	not significant
6281	GO_NEGATIVE_REGULATION_OF_MONOOXYGENASE_ACTIVITY	-0.93	0.26	0	0	0	not significant
6282	GO_PHOSPHATIDYLINOSITOL_4_PHOSPHATE_BINDING	-0.93	0.26	0	0	0	not significant
6283	GO_POSITIVE_REGULATION_OF_BONE_RESORPTION	-0.93	0.26	0	0	0	not significant
6284	GO_PURINE_NUCLEOSIDE_TRIPHOSPHATE_CATABOLIC_PROCESS	-0.93	0.26	0	0	0	not significant
6285	GO_REGULATION_OF_CELLULAR_RESPONSE_TO_HYPOXIA	-0.93	0.26	0	0	0	not significant
6286	GO_REGULATION_OF_TRANSLATION_IN_RESPONSE_TO_ENDOPLASMIC_	-0.93	0.26	0	0	0	not significant
6287	GO_SINGLE_STRANDED_DNA_DEPENDENT_ATPASE_ACTIVITY	-0.93	0.26	0	0	0	not significant
6288	REACTOME_DOWNREGULATION_OF_TGF_BETA_RECEPTOR_SIGNALING	-0.93	0.26	0	0	0	not significant
6289	REACTOME_INACTIVATION_OF_CDC42_AND_RAC1	-0.93	0.26	0	0	0	not significant
6290	REACTOME_SENSING_OF_DNA_DOUBLE_STRAND_BREAKS	-0.93	0.26	0	0	0	not significant
6291	REACTOME_TRANSFERRIN_ENDOCYTOSIS_AND_RECYCLING	-0.93	0.26	0	0	0	not significant
6292	WANG_METASTASIS_OF_BREAST_CANCER_ESR1_DN	-0.93	0.26	0	0	0	not significant
6293	BOWIE_RESPONSE_TO_TAMOXIFEN	-0.93	0.25	0	0	0	not significant
6294	GO_GLOBAL_GENOME_NUCLEOTIDE_EXCISION_REPAIR	-0.93	0.25	0	0	0	not significant
6295	GO_HINDLIMB_MORPHOGENESIS	-0.93	0.25	0	0	0	not significant
6296	GO_POSITIVE_REGULATION_OF_FIBROBLAST_GROWTH_FACTOR_RECE	-0.93	0.25	0	0	0	not significant
6297	GO_REGULATION_OF_COENZYME_METABOLIC_PROCESS	-0.93	0.25	0	0	0	not significant
6298	GO_SYNAPTIC_CLEFT	-0.93	0.25	0	0	0	not significant
6299	KEGG_SNARE_INTERACTIONS_IN_VESICULAR_TRANSPORT	-0.93	0.25	0	0	0	not significant
6300	KOHOUTEK_CCNT1_TARGETS	-0.93	0.25	0	0	0	not significant
6301	MOROSETTI_FACIOSCAPULOHUMERAL_MUSCULAR_DISTROPHY_UP	-0.93	0.25	0	0	0	not significant
6302	MYLLYKANGAS_AMPLIFICATION_HOT_SPOT_23	-0.93	0.25	0	0	0	not significant
6303	REACTOME_DEFECTIVE_B4GALT7_CAUSES_EDS_PROGEROID_TYPE	-0.93	0.25	0	0	0	not significant
6304	SATO_SILENCED_BY_METHYLATION_IN_PANCREATIC_CANCER_2	-0.93	0.25	0	0	0	not significant
6305	TORCHIA_TARGETS_OF_EWSR1_FLI1_FUSION_TOP20_UP	-0.93	0.25	0	0	0	not significant
6306	BIOCARTA_PELP1_PATHWAY	-0.93	0.24	0	0	0	not significant
6307	GO_AMYLOID_BETA_BINDING	-0.93	0.24	0	0	0	not significant
6308	GO_CLEAVAGE_FURROW	-0.93	0.24	0	0	0	not significant
6309	GO_ORGANOPHOSPHATE_ESTER_TRANSMEMBRANE_TRANSPORTER_A(	-0.93	0.24	0	0	0	not significant
6310	GO_OXIDOREDUCTASE_ACTIVITY_ACTING_ON_THE_ALDEHYDE_OR_OX(	-0.93	0.24	0	0	0	not significant

6311	GO_PHOSPHATIDYLCHOLINE_BIOSYNTHETIC_PROCESS	-0.93	0.24	0	0	0	not significant
6312	GO POSITIVE REGULATION OF REACTIVE OXYGEN SPECIES BIOSYNT	-0.93	0.24	0	0	0	not significant
6313	GO_REGULATION_OF_FILOPODIUM_ASSEMBLY	-0.93	0.24	0	0	0	not significant
6314	REACTOME_SIGNALING_BY_MET	-0.93	0.24	0	0	0	not significant
6315	CHR11Q12	-0.93	0.23	0	0	0	not significant
6316	CHR5Q33	-0.93	0.23	0	0	0	not significant
6317	GO_CALCIUM_CHANNEL_COMPLEX	-0.93	0.23	0	0	0	not significant
6318	GO_EXOPEPTIDASE_ACTIVITY	-0.93	0.23	0	0	0	not significant
6319	GO_MORPHOGENESIS_OF_A_BRANCHING_STRUCTURE	-0.93	0.23	0	0	0	not significant
6320	GO_REGULATION_OF_MITOCHONDRIAL_OUTER_MEMBRANE_PERMEABIL	-0.93	0.23	0	0	0	not significant
6321	GO_VESICLE_CYTOSKELETAL_TRAFFICKING	-0.93	0.23	0	0	0	not significant
6322	MINGUEZ_LIVER_CANCER_VASCULAR_INVASION_UP	-0.93	0.23	0	0	0	not significant
6323	PASINI_SUZ12_TARGETS_UP	-0.93	0.23	0	0	0	not significant
6324	DAVICIONI_TARGETS_OF_PAX_FOXO1_FUSIONS_DN	-0.93	0.22	0	0	0	not significant
6325	GO_ATPASE_BINDING	-0.93	0.22	0	0	0	not significant
6326	GO_CELL_DIVISION_SITE	-0.93	0.22	0	0	0	not significant
6327	GO_REGULATION_OF_RESPONSE_TO_ENDOPLASMIC_RETICULUM_STRE	-0.93	0.22	0	0	0	not significant
6328	REACTOME BIOSYNTHESIS OF THE N GLYCAN PRECURSOR DOLICHC	-0.93	0.22	0	0	0	not significant
6329	REACTOME_DUAL_INCISION_IN_GG_NER	-0.93	0.22	0	0	0	not significant
6330	SASSON_RESPONSE_TO_FORSKOLIN_DN	-0.93	0.22	0	0	0	not significant
6331	GO_CONDENSED_NUCLEAR_CHROMOSOME	-0.93	0.21	0	0	0	not significant
6332	REACTOME_HATS_ACETYLATE_HISTONES	-0.93	0.21	0	0	0	not significant
6333	GO_ATPASE_COMPLEX	-0.93	0.20	0	0	0	not significant
6334	GO_MYELOID_CELL_DEVELOPMENT	-0.93	0.20	0	0	0	not significant
6335	GO_PEPTIDE_HORMONE_SECRETION	-0.93	0.20	0	0	0	not significant
6336	NRL_DN.V1_DN	-0.93	0.20	0	0	0	not significant
6337	REACTOME_RUNX1_REGULATES_GENES_INVOLVED_IN_MEGAKARYOCY	-0.93	0.20	0	0	0	not significant
6338	GO NEGATIVE REGULATION OF PROTEIN SERINE THREONINE KINASE	-0.93	0.18	0	0	0	not significant
6339	REACTOME_SLC_MEDIATED_TRANSMEMBRANE_TRANSPORT	-0.93	0.18	0	0	0	not significant
6340	GO_GLUTAMATERGIC_SYNAPSE	-0.93	0.16	0	0	0	not significant
6341	GO_UBIQUITIN_LIKE_PROTEIN_LIGASE_BINDING	-0.93	0.16	0	0	0	not significant
6342	GO_CELL_GROWTH	-0.93	0.15	0	0	0	not significant
6343	GO_POSITIVE_REGULATION_OF_CATABOLIC_PROCESS	-0.93	0.15	0	0	0	not significant
6344	OISHI_CHOLANGIOMA_STEM_CELL_LIKE_UP	-0.93	0.14	0	0	0	not significant
6345	GO_CELLULAR_LIPID_METABOLIC_PROCESS	-0.93	0.11	0	0	0	not significant
6346	GO_MICROTUBULE_CYTOSKELETON	-0.93	0.08	0	0	0	not significant
6347	GO_CELL_PROJECTION_ORGANIZATION	-0.93	0.04	0	0	0	not significant
6348	MARSON_BOUND_BY_FOXP3_UNSTIMULATED	-0.93	0.03	0	0	0	not significant
6349	GO_WHOLE_MEMBRANE	-0.93	0.02	0	0	0	not significant
6350	GO_AMINE_CATABOLIC_PROCESS	-0.92	0.28	0	0	0	not significant
6351	CHR3P26	-0.92	0.27	0	0	0	not significant
6352	GO_BASE_EXCISION_REPAIR_AP_SITE_FORMATION	-0.92	0.27	0	0	0	not significant
6353	GO_CLATHRIN_ADAPTOR_COMPLEX	-0.92	0.27	0	0	0	not significant
6354	GO_PHOSPHATASE_ACTIVATOR_ACTIVITY	-0.92	0.27	0	0	0	not significant
6355	GO_PROTEIN_ACTIVATION_CASCADE	-0.92	0.27	0	0	0	not significant
6356	PID_RHOA_REG_PATHWAY	-0.92	0.27	0	0	0	not significant
6357	BIOCARTA_EIF_PATHWAY	-0.92	0.26	0	0	0	not significant
6358	CONRAD_STEM_CELL	-0.92	0.26	0	0	0	not significant
6359	GO_CELLULAR_TRIGLYCERIDE_HOMEOSTASIS	-0.92	0.26	0	0	0	not significant
6360	GO_CHAPERONE_MEDIATED_PROTEIN_TRANSPORT	-0.92	0.26	0	0	0	not significant
6361	GO_GOLGI_RIBBON_FORMATION	-0.92	0.26	0	0	0	not significant
6362	GO_MYOSIN_V_BINDING	-0.92	0.26	0	0	0	not significant
6363	GO_RNA_SPLICING_VIA_ENDONUCLEOLYTIC_CLEAVAGE_AND_LIGATION	-0.92	0.26	0	0	0	not significant
6364	GO_SUBPALLIUM_DEVELOPMENT	-0.92	0.26	0	0	0	not significant
6365	SCHLINGEMANN_SKIN_CARCINOGENESIS_TPA_UP	-0.92	0.26	0	0	0	not significant
6366	YIH_RESPONSE_TO_ARSENITE_C4	-0.92	0.26	0	0	0	not significant
6367	ZHENG_RESPONSE_TO_ARSENITE_DN	-0.92	0.26	0	0	0	not significant
6368	BIOCARTA_REELIN_PATHWAY	-0.92	0.25	0	0	0	not significant
6369	GO_BROWN_FAT_CELL_DIFFERENTIATION	-0.92	0.25	0	0	0	not significant
6370	GO_FMN_BINDING	-0.92	0.25	0	0	0	not significant
6371	GO_INSULIN_LIKE_GROWTH_FACTOR_RECEPTOR_BINDING	-0.92	0.25	0	0	0	not significant
6372	GO NEGATIVE REGULATION OF B CELL RECEPTOR SIGNALING PATH	-0.92	0.25	0	0	0	not significant
6373	GO_NEURON_FATE_COMMITMENT	-0.92	0.25	0	0	0	not significant
6374	GO PHOSPHATIDYLINOSITOL MONOPHOSPHATE PHOSPHATASE ACTIVI	-0.92	0.25	0	0	0	not significant
6375	GO_POLAR_MICROTUBULE	-0.92	0.25	0	0	0	not significant
6376	GO_POSITIVE_REGULATION_OF_COLLATERAL_SPROUTING	-0.92	0.25	0	0	0	not significant
6377	GO POSITIVE REGULATION OF DNA DAMAGE RESPONSE SIGNAL TR/	-0.92	0.25	0	0	0	not significant
6378	GO_PROTEIN_LOCALIZATION_TO_MEMBRANE_RAFT	-0.92	0.25	0	0	0	not significant
6379	GO_REGULATION_OF_POSITIVE_CHEMOTAXIS	-0.92	0.25	0	0	0	not significant
6380	GO_RIBOSOME_DISASSEMBLY	-0.92	0.25	0	0	0	not significant
6381	IIZUKA_LIVER_CANCER_PROGRESSION_G1_G2_UP	-0.92	0.25	0	0	0	not significant
6382	KEGG_NITROGEN_METABOLISM	-0.92	0.25	0	0	0	not significant
6383	KORKOLA_EMBRYONIC_CARCINOMA_VS_SEMINOMA_UP	-0.92	0.25	0	0	0	not significant
6384	LIAN_NEUTROPHIL_GRANULE_CONSTITUENTS	-0.92	0.25	0	0	0	not significant
6385	REACTOME_SIGNALLING_TO_ERKS	-0.92	0.25	0	0	0	not significant
6386	REACTOME_SYNTHESIS_OF_PIPS_AT_THE_LATE_ENDOSOME_MEMBRAN	-0.92	0.25	0	0	0	not significant
6387	SCHRAETS_MLL_TARGETS_UP	-0.92	0.25	0	0	0	not significant
6388	BOYLAN_MULTIPLE_MYELOMA_C_UP	-0.92	0.24	0	0	0	not significant
6389	GO_COPPER_ION_BINDING	-0.92	0.24	0	0	0	not significant
6390	GO_MIRNA_BINDING	-0.92	0.24	0	0	0	not significant
6391	GO_NEGATIVE_REGULATION_OF_MACROAUTOPHAGY	-0.92	0.24	0	0	0	not significant
6392	GO_POSITIVE_REGULATION_OF_ANIMAL_ORGAN_MORPHOGENESIS	-0.92	0.24	0	0	0	not significant
6393	GO_POSITIVE_REGULATION_OF_ERAD_PATHWAY	-0.92	0.24	0	0	0	not significant
6394	GO REGULATION OF CELL COMMUNICATION BY ELECTRICAL COUPLI	-0.92	0.24	0	0	0	not significant
6395	REACTOME_EICOSANOID_LIGAND_BINDING_RECEPTORS	-0.92	0.24	0	0	0	not significant
6396	FRASOR_TAMOXIFEN_RESPONSE_UP	-0.92	0.23	0	0	0	not significant
6397	GO NEGATIVE REGULATION OF INTRINSIC APOPTOTIC SIGNALING PA	-0.92	0.23	0	0	0	not significant
6398	GO_POSITIVE_REGULATION_OF_COLLAGEN_BIOSYNTHETIC_PROCESS	-0.92	0.23	0	0	0	not significant
6399	GO_REGULATION_OF_MEIOTIC_NUCLEAR_DIVISION	-0.92	0.23	0	0	0	not significant
6400	SATO_SILENCED_BY_DEACETYLATION_IN_PANCREATIC_CANCER	-0.92	0.23	0	0	0	not significant
6401	GO_NEGATIVE_REGULATION_OF_PROTEIN_DEPHOSPHORYLATION	-0.92	0.22	0	0	0	not significant
6402	GO_RETROGRADE_VESICLE_MEDIATED_TRANSPORT_GOLGI_TO_ENDO	-0.92	0.22	0	0	0	not significant
6403	KEGG_STARCH_AND_SUCROSE_METABOLISM	-0.92	0.22	0	0	0	not significant
6404	KONDO_COLON_CANCER_HCP_WITH_H3K27ME1	-0.92	0.22	0	0	0	not significant
6405	REACTOME_POST_TRANSLATIONAL_MODIFICATION:_SYNTHESIS_OF_GF	-0.92	0.22	0	0	0	not significant
6406	RIZKI_TUMOR_INVASIVENESS_2D_UP	-0.92	0.22	0	0	0	not significant
6407	TURASHVILI_BREAST_DUCTAL_CARCINOMA_VS_LOBULAR_NORMAL_UP	-0.92	0.22	0	0	0	not significant
6408	CRX_NRL_DN.V1_UP	-0.92	0.21	0	0	0	not significant
6409	GO_SMAD_BINDING	-0.92	0.21	0	0	0	not significant
6410	REACTOME_EPH_EPHRIN_MEDIATED_REPULSION_OF_CELLS	-0.92	0.21	0	0	0	not significant
6411	BYSTRYKH_HEMATOPOIESIS_STEM_CELL_AND_BRAIN_QTL_CIS	-0.92	0.20	0	0	0	not significant
6412	GO_CELLULAR_RESPONSE_TO_HYDROGEN_PEROXIDE	-0.92	0.20	0	0	0	not significant
6413	GO_NEUROTRANSMITTER_METABOLIC_PROCESS	-0.92	0.20	0	0	0	not significant
6414	SASSON_RESPONSE_TO_GONADOTROPHINS_DN	-0.92	0.20	0	0	0	not significant
6415	CHIARADONNA_NEOPLASTIC_TRANSFORMATION_KRAS_UP	-0.92	0.19	0	0	0	not significant
6416	GO_NEGATIVE_REGULATION_OF_CYTOSKELETON_ORGANIZATION	-0.92	0.19	0	0	0	not significant
6417	SONG_TARGETS_OF_IE86_CMV_PROTEIN	-0.92	0.19	0	0	0	not significant
6418	ZHANG_TARGETS_OF_EWSR1_FLI1_FUSION	-0.92	0.19	0	0	0	not significant
6419	GO_CYTOSKELETON_DEPENDENT_INTRACELLULAR_TRANSPORT	-0.92	0.18	0	0	0	not significant

6420	GO_CULLIN_RING_UBIQUITIN_LIGASE_COMPLEX	-0.92	0.17	0	0	0	not significant
6421	GO_NUCLEAR_MATRIX	-0.92	0.17	0	0	0	not significant
6422	GO_SODIUM_ION_TRANSPORT	-0.92	0.17	0	0	0	not significant
6423	DEBIASI_APOPTOSIS_BY_REOVIRUS_INFECTION_UP	-0.92	0.13	0	0	0	not significant
6424	GO_POSITIVE_REGULATION_OF_GTPASE_ACTIVITY	-0.92	0.13	0	0	0	not significant
6425	GO_PROTEIN_HETERODIMERIZATION_ACTIVITY	-0.92	0.12	0	0	0	not significant
6426	GO_REGULATION_OF_CELLULAR_COMPONENT_BIOGENESIS	-0.92	0.07	0	0	0	not significant
6427	GO_NEURON_DEVELOPMENT	-0.92	0.06	0	0	0	not significant
6428	GO_NUCLEAR_BODY	-0.92	0.05	0	0	0	not significant
6429	GO_ENDOPLASMIC_RETICULUM	-0.92	0.03	0	0	0	not significant
6430	GO_NUCLEOSIDE_DIPHOSPHATE_KINASE_ACTIVITY	-0.91	0.27	0	0	0	not significant
6431	REACTOME_REGULATION_BY_C_FLIP	-0.91	0.27	0	0	0	not significant
6432	GO_HAIR_FOLLICLE_MATURATION	-0.91	0.26	0	0	0	not significant
6433	GO_REPLICATIVE_SENESCENCE	-0.91	0.26	0	0	0	not significant
6434	INAMURA_LUNG_CANCER_SCC_UP	-0.91	0.26	0	0	0	not significant
6435	REACTOME_A_TETRASACCHARIDE_LINKER_SEQUENCE_IS_REQUIRED_F	-0.91	0.26	0	0	0	not significant
6436	REACTOME_LOSS_OF_FUNCTION_OF_TGFBR1_IN_CANCER	-0.91	0.26	0	0	0	not significant
6437	VALK_AML_CLUSTER_6	-0.91	0.26	0	0	0	not significant
6438	CHR11P12	-0.91	0.25	0	0	0	not significant
6439	GO_CELLULAR_RESPONSE_TO_LEUCINE	-0.91	0.25	0	0	0	not significant
6440	GO_ORGANELLE_INHERITANCE	-0.91	0.25	0	0	0	not significant
6441	GO_POSTSYNAPTIC_CYTOSOL	-0.91	0.25	0	0	0	not significant
6442	GO_REGULATION_OF_PLATELET_DERIVED_GROWTH_FACTOR_RECEPT	-0.91	0.25	0	0	0	not significant
6443	HESSON_TUMOR_SUPPRESSOR_CLUSTER_3P21_3	-0.91	0.25	0	0	0	not significant
6444	WANG_BARRETTS_ESOPHAGUS_AND_ESOPHAGUS_CANCER_UP	-0.91	0.25	0	0	0	not significant
6445	WEBER_METHYLATED_IN_COLON_CANCER	-0.91	0.25	0	0	0	not significant
6446	BIOCARTA_ERK_PATHWAY	-0.91	0.24	0	0	0	not significant
6447	CARDOSO_RESPONSE_TO_GAMMA_RADIATION_AND_3AB	-0.91	0.24	0	0	0	not significant
6448	CHESLER_BRAIN_HIGHEST_GENETIC_VARIANCE	-0.91	0.24	0	0	0	not significant
6449	GO_ACTIVATION_OF_JNKK_ACTIVITY	-0.91	0.24	0	0	0	not significant
6450	GO_DYNACTIN_COMPLEX	-0.91	0.24	0	0	0	not significant
6451	GO_HISTONE_H3_K36_METHYLATION	-0.91	0.24	0	0	0	not significant
6452	GO_LONG_CHAIN_FATTY_ACID_IMPORT_INTO_CELL	-0.91	0.24	0	0	0	not significant
6453	GO_MAMMARY_GLAND_ALVEOLUS_DEVELOPMENT	-0.91	0.24	0	0	0	not significant
6454	GO_MICROFILAMENT_MOTOR_ACTIVITY	-0.91	0.24	0	0	0	not significant
6455	GO_MITOCHONDRIAL_DNA_METABOLIC_PROCESS	-0.91	0.24	0	0	0	not significant
6456	GO_NEGATIVE_REGULATION_OF_AMINE_TRANSPORT	-0.91	0.24	0	0	0	not significant
6457	GO_NEGATIVE_REGULATION_OF_PROTEIN_ACETYLATION	-0.91	0.24	0	0	0	not significant
6458	GO_NEGATIVE_REGULATION_OF_SPROUTING_ANGIOGENESIS	-0.91	0.24	0	0	0	not significant
6459	GO_POLYADENYLATION_DEPENDENT_SNORNA_3_END_PROCESSING	-0.91	0.24	0	0	0	not significant
6460	GO_POSITIVE_REGULATION_OF_B_CELL_RECEPTOR_SIGNALING_PATHW	-0.91	0.24	0	0	0	not significant
6461	GO_POSITIVE_REGULATION_OF_CARDIAC_MUSCLE_TISSUE_DEVELOPM	-0.91	0.24	0	0	0	not significant
6462	GO_POSITIVE_REGULATION_OF_EXTRINSIC_APOPTOTIC_SIGNALING_PA	-0.91	0.24	0	0	0	not significant
6463	GO_RESPIRATORY_SYSTEM_PROCESS	-0.91	0.24	0	0	0	not significant
6464	GO_SIGNAL_TRANSDUCTION_INVOLVED_IN_G2_DNA_DAMAGE_CHECKPO	-0.91	0.24	0	0	0	not significant
6465	GO_UDP_GLUCOSYLTRANSFERASE_ACTIVITY	-0.91	0.24	0	0	0	not significant
6466	PID_EPHA_FWDPATHWAY	-0.91	0.24	0	0	0	not significant
6467	AMIT_EGF_RESPONSE_20_MCF10A	-0.91	0.23	0	0	0	not significant
6468	GO_ARP2_3_COMPLEX_MEDIATED_ACTIN_NUCLEATION	-0.91	0.23	0	0	0	not significant
6469	GO_ASPARTATE_METABOLIC_PROCESS	-0.91	0.23	0	0	0	not significant
6470	GO_DIACYLGLYCEROL_BINDING	-0.91	0.23	0	0	0	not significant
6471	GO_NEGATIVE_REGULATION_OF_INCLUSION_BODY_ASSEMBLY	-0.91	0.23	0	0	0	not significant
6472	GO_NEGATIVE_REGULATION_OF_INTRACELLULAR_ESTROGEN_RECEPT	-0.91	0.23	0	0	0	not significant
6473	GO_NEGATIVE_REGULATION_OF_UBIQUITIN_DEPENDENT_PROTEIN_CAT	-0.91	0.23	0	0	0	not significant
6474	GO_PEPTIDYL_CYSTEINE_MODIFICATION	-0.91	0.23	0	0	0	not significant
6475	GO_POSITIVE_REGULATION_OF_PRODUCTION_OF_MIRNAS_INVOLVED_I	-0.91	0.23	0	0	0	not significant
6476	GO_REGULATION_OF_TRANSLATIONAL_INITIATION_IN_RESPONSE_TO_S	-0.91	0.23	0	0	0	not significant
6477	CHR6Q25	-0.91	0.22	0	0	0	not significant
6478	ELLWOOD_MYC_TARGETS_DN	-0.91	0.22	0	0	0	not significant
6479	FIGUEROA_AML_METHYLATION_CLUSTER_5_DN	-0.91	0.22	0	0	0	not significant
6480	GO_MEMBRANE_PROTEIN_PROTEOLYSIS	-0.91	0.22	0	0	0	not significant
6481	GO_NEGATIVE_REGULATION_OF_SODIUM_ION_TRANSPORT	-0.91	0.22	0	0	0	not significant
6482	GO_REGULATION_OF_ENDOPLASMIC_RETICULUM_STRESS_INDUCED_IN	-0.91	0.22	0	0	0	not significant
6483	GO_REGULATION_OF_GLUCOSE_TRANSMEMBRANE_TRANSPORT	-0.91	0.22	0	0	0	not significant
6484	GO_REGULATION_OF_MITOTIC_SPINDLE_ASSEMBLY	-0.91	0.22	0	0	0	not significant
6485	GO_SCHWANN_CELL_DEVELOPMENT	-0.91	0.22	0	0	0	not significant
6486	GO_WOUND_HEALING_SPREADING_OF_EPIDERMAL_CELLS	-0.91	0.22	0	0	0	not significant
6487	GENTILE_UV_RESPONSE_CLUSTER_D2	-0.91	0.21	0	0	0	not significant
6488	GO_MEGAKARYOCYTE_DIFFERENTIATION	-0.91	0.21	0	0	0	not significant
6489	GO_RAC_GTPASE_BINDING	-0.91	0.21	0	0	0	not significant
6490	REACTOME_ION_TRANSPORT_BY_P_TYPE_ATPASES	-0.91	0.21	0	0	0	not significant
6491	COATES_MACROPHAGE_M1_VS_M2_UP	-0.91	0.20	0	0	0	not significant
6492	GARGALOVIC_RESPONSE_TO_OXIDIZED_PHOSPHOLIPIDS_GREY_DN	-0.91	0.20	0	0	0	not significant
6493	GO_CONTRACTILE_ACTIN_FILAMENT_BUNDLE_ASSEMBLY	-0.91	0.20	0	0	0	not significant
6494	GO_CORTICAL_ACTIN_CYTOSKELETON	-0.91	0.20	0	0	0	not significant
6495	GO_G1_DNA_DAMAGE_CHECKPOINT	-0.91	0.20	0	0	0	not significant
6496	GO_PROTEIN_CONTAINING_COMPLEX_SCAFFOLD_ACTIVITY	-0.91	0.20	0	0	0	not significant
6497	GO_REGULATION_OF_ESTABLISHMENT_OF_PROTEIN_LOCALIZATION_TO	-0.91	0.20	0	0	0	not significant
6498	CHR15Q15	-0.91	0.19	0	0	0	not significant
6499	GO_EXONUCLEASE_ACTIVITY	-0.91	0.19	0	0	0	not significant
6500	GO_REACTIVE_OXYGEN_SPECIES_BIOSYNTHETIC_PROCESS	-0.91	0.19	0	0	0	not significant
6501	GO_REGULATION_OF_EPITHELIAL_TO_MESENCHYMAL_TRANSITION	-0.91	0.19	0	0	0	not significant
6502	ALCALA_APOPTOSIS	-0.91	0.18	0	0	0	not significant
6503	CHR9Q34	-0.91	0.18	0	0	0	not significant
6504	GO_EPITHELIAL_TO_MESENCHYMAL_TRANSITION	-0.91	0.18	0	0	0	not significant
6505	GO_MESENCHYME_DEVELOPMENT	-0.91	0.17	0	0	0	not significant
6506	MULLIGHAN_NPM1_MUTATED_SIGNATURE_1_DN	-0.91	0.17	0	0	0	not significant
6507	NIKOLSKY_BREAST_CANCER_11Q12_Q14_AMPLICON	-0.91	0.16	0	0	0	not significant
6508	GO_POSITIVE_REGULATION_OF_CYTOSKELETON_ORGANIZATION	-0.91	0.15	0	0	0	not significant
6509	GO_REGULATION_OF_ENDOCYTOSIS	-0.91	0.15	0	0	0	not significant
6510	GO_TRANSMEMBRANE_RECEPTOR_PROTEIN_SERINE_THREONINE_KINAS	-0.91	0.14	0	0	0	not significant
6511	GO_AXON_PART	-0.91	0.13	0	0	0	not significant
6512	GO_DENDRITE_DEVELOPMENT	-0.91	0.12	0	0	0	not significant
6513	GO_EARLY_ENDOSOME	-0.91	0.12	0	0	0	not significant
6514	GO_REGULATION_OF_ANATOMICAL_STRUCTURE_SIZE	-0.91	0.11	0	0	0	not significant
6515	AGUIRRE_PANCREATIC_CANCER_COPY_NUMBER_UP	-0.91	0.10	0	0	0	not significant
6516	GO_SIGNAL_TRANSDUCTION_BY_P53_CLASS_MEDIATOR	-0.91	0.10	0	0	0	not significant
6517	GO_NEGATIVE_REGULATION_OF_INTRACELLULAR_SIGNAL_TRANSDUCT	-0.91	0.09	0	0	0	not significant
6518	MIKKELSEN_NPC_ICP_WITH_H3K4ME3	-0.91	0.09	0	0	0	not significant
6519	GO_ORGANELLE_ASSEMBLY	-0.91	0.04	0	0	0	not significant
6520	GO_TRANSFERASE_ACTIVITY_TRANSFERRING_PHOSPHORUS_CONTAINI	-0.91	0.04	0	0	0	not significant
6521	GO_NEGATIVE_REGULATION_OF_EPITHELIAL_TO_MESENCHYMAL_TRAN	-0.90	0.25	0	0	0	not significant
6522	GO_REGULATION_OF_NEUROTRANSMITTER_UPTAKE	-0.90	0.25	0	0	0	not significant
6523	GO_VIRAL_TRANSLATION	-0.90	0.25	0	0	0	not significant
6524	REACTOME_SIGNALLING_TO_RAS	-0.90	0.25	0	0	0	not significant
6525	GO_CGMP_MEDIATED_SIGNALING	-0.90	0.24	0	0	0	not significant
6526	GO_GTP_DEPENDENT_PROTEIN_BINDING	-0.90	0.24	0	0	0	not significant
6527	GO_MAINTENANCE_OF_SYNAPSE_STRUCTURE	-0.90	0.24	0	0	0	not significant
6528	GO_NUCLEOSIDE_MONOPHOSPHATE_PHOSPHORYLATION	-0.90	0.24	0	0	0	not significant

6529	REACTOME_BETA_OXIDATION_OF_PRISTANOYL_COA	-0.90	0.24	0	0	0	not significant
6530	WOTTON_RUNX_TARGETS_DN	-0.90	0.24	0	0	0	not significant
6531	BIOCARTA_RAS_PATHWAY	-0.90	0.23	0	0	0	not significant
6532	CHR3Q24	-0.90	0.23	0	0	0	not significant
6533	GAUSSMANN_MLL_AF4_FUSION_TARGETS_C_DN	-0.90	0.23	0	0	0	not significant
6534	GO_ADIPONECTIN_ACTIVATED_SIGNALING_PATHWAY	-0.90	0.23	0	0	0	not significant
6535	GO_GLUCOSAMINE_CONTAINING_COMPOUND_METABOLIC_PROCESS	-0.90	0.23	0	0	0	not significant
6536	GO_MITOGEN_ACTIVATED_PROTEIN_KINASE_P38_BINDING	-0.90	0.23	0	0	0	not significant
6537	GO_NEGATIVE_REGULATION_OF_FIBROBLAST_MIGRATION	-0.90	0.23	0	0	0	not significant
6538	GO_OUTER_DYNEIN_ARM_ASSEMBLY	-0.90	0.23	0	0	0	not significant
6539	GO_POSITIVE_REGULATION_OF_PATHWAY_RESTRICTED_SMAD_PROTE	-0.90	0.23	0	0	0	not significant
6540	GO_PROTEIN_SERINE_THREONINE_KINASE_INHIBITOR_ACTIVITY	-0.90	0.23	0	0	0	not significant
6541	GO_REGULATION_OF_LYSOSOMAL_PROTEIN_CATABOLIC_PROCESS	-0.90	0.23	0	0	0	not significant
6542	GO_REGULATION_OF_MONOCYTE_DIFFERENTIATION	-0.90	0.23	0	0	0	not significant
6543	GO_STRUCTURAL_CONSTITUENT_OF_MUSCLE	-0.90	0.23	0	0	0	not significant
6544	MELLMAN_TUT1_TARGETS_UP	-0.90	0.23	0	0	0	not significant
6545	NUMATA_CSF3_SIGNALING_VIA_STAT3	-0.90	0.23	0	0	0	not significant
6546	PEPPER_CHRONIC_LYMPHOCYTIC_LEUKEMIA_DN	-0.90	0.23	0	0	0	not significant
6547	AIYAR_COBRA1_TARGETS_UP	-0.90	0.22	0	0	0	not significant
6548	ANDERSEN_CHOLANGIOCARCINOMA_CLASS1	-0.90	0.22	0	0	0	not significant
6549	CHR2P16	-0.90	0.22	0	0	0	not significant
6550	GO_AMYLOID_PRECURSOR_PROTEIN_CATABOLIC_PROCESS	-0.90	0.22	0	0	0	not significant
6551	GO_ANOIKIS	-0.90	0.22	0	0	0	not significant
6552	GO_DYNEIN_COMPLEX_BINDING	-0.90	0.22	0	0	0	not significant
6553	GO_HSP70_PROTEIN_BINDING	-0.90	0.22	0	0	0	not significant
6554	GO_INTERLEUKIN_8_SECRETION	-0.90	0.22	0	0	0	not significant
6555	GO_OSTEOCLAST_DEVELOPMENT	-0.90	0.22	0	0	0	not significant
6556	GO_REGULATION_OF_GLIAL_CELL_APOPTOTIC_PROCESS	-0.90	0.22	0	0	0	not significant
6557	GO_REGULATION_OF_KIDNEY_DEVELOPMENT	-0.90	0.22	0	0	0	not significant
6558	MADAN_DPPA4_TARGETS	-0.90	0.22	0	0	0	not significant
6559	PID_INTEGRIN1_PATHWAY	-0.90	0.22	0	0	0	not significant
6560	REACTOME_SYNTHESIS_OF_IP3_AND_IP4_IN_THE_CYTOSOL	-0.90	0.22	0	0	0	not significant
6561	GO_BASEMENT_MEMBRANE	-0.90	0.21	0	0	0	not significant
6562	GO_CALMODULIN_DEPENDENT_PROTEIN_KINASE_ACTIVITY	-0.90	0.21	0	0	0	not significant
6563	GO_ESTROGEN_RECEPTOR_BINDING	-0.90	0.21	0	0	0	not significant
6564	GO_NEGATIVE_REGULATION_OF_REACTIVE_OXYGEN_SPECIES_METABO	-0.90	0.21	0	0	0	not significant
6565	GO_NUCLEAR_UBIQUITIN_LIGASE_COMPLEX	-0.90	0.21	0	0	0	not significant
6566	GO_POSITIVE_REGULATION_OF_PROTEIN_TARGETING_TO_MEMBRANE	-0.90	0.21	0	0	0	not significant
6567	GO_POST_EMBRYONIC_ANIMAL_ORGAN_DEVELOPMENT	-0.90	0.21	0	0	0	not significant
6568	GO_SECRETION_BY_TISSUE	-0.90	0.21	0	0	0	not significant
6569	NUTT_GBM_VS_AO_GLIOMA_DN	-0.90	0.21	0	0	0	not significant
6570	CHR3Q29	-0.90	0.20	0	0	0	not significant
6571	GO_EXORIBONUCLEASE_ACTIVITY	-0.90	0.20	0	0	0	not significant
6572	GO_POSITIVE_REGULATION_OF_P38MAPK_CASCADE	-0.90	0.20	0	0	0	not significant
6573	GO_THYMOCYTE_APOPTOTIC_PROCESS	-0.90	0.20	0	0	0	not significant
6574	VANTVEER_BREAST_CANCER_POOR_PROGNOSIS	-0.90	0.20	0	0	0	not significant
6575	CHR22Q12	-0.90	0.19	0	0	0	not significant
6576	GO_CELLULAR_EXTRAVASATION	-0.90	0.19	0	0	0	not significant
6577	GO_RECEPTOR_INTERNALIZATION	-0.90	0.19	0	0	0	not significant
6578	GO_SITE_OF_DNA_DAMAGE	-0.90	0.19	0	0	0	not significant
6579	GO_SPECIFIC_GRANULE_MEMBRANE	-0.90	0.19	0	0	0	not significant
6580	KEGG_DILATED_CARDIOMYOPATHY	-0.90	0.19	0	0	0	not significant
6581	KYNG_DNA_DAMAGE_BY_GAMMA_RADIATION	-0.90	0.19	0	0	0	not significant
6582	PID_P53_REGULATION_PATHWAY	-0.90	0.19	0	0	0	not significant
6583	GO_POSITIVE_REGULATION_OF_LEUKOCYTE_MIGRATION	-0.90	0.18	0	0	0	not significant
6584	GO_TISSUE_REMODELING	-0.90	0.18	0	0	0	not significant
6585	GO_VASCULAR_ENDOTHELIAL_GROWTH_FACTOR_RECEPTOR_SIGNALI	-0.90	0.18	0	0	0	not significant
6586	KEGG_LONG_TERM_POTENTIATION	-0.90	0.18	0	0	0	not significant
6587	STEIN_ESR1_TARGETS	-0.90	0.18	0	0	0	not significant
6588	GO_CARDIAC_SEPTUM_DEVELOPMENT	-0.90	0.17	0	0	0	not significant
6589	GO_PERIPHERAL_NERVOUS_SYSTEM_DEVELOPMENT	-0.90	0.17	0	0	0	not significant
6590	KEGG_RENAL_CELL_CARCINOMA	-0.90	0.17	0	0	0	not significant
6591	GO_CELLULAR_RESPONSE_TO_LIGHT_STIMULUS	-0.90	0.16	0	0	0	not significant
6592	NOTCH_DN.V1_UP	-0.90	0.16	0	0	0	not significant
6593	GO_NEGATIVE_REGULATION_OF_RESPONSE_TO_EXTERNAL_STIMULUS	-0.90	0.15	0	0	0	not significant
6594	FIRESTEIN_PROLIFERATION	-0.90	0.13	0	0	0	not significant
6595	GO_REGULATION_OF_SIGNAL_TRANSDUCTION_BY_P53_CLASS_MEDIAT	-0.90	0.13	0	0	0	not significant
6596	GO_PEPTIDYL_SERINE_MODIFICATION	-0.90	0.12	0	0	0	not significant
6597	GO_POSITIVE_REGULATION_OF_GROWTH	-0.90	0.12	0	0	0	not significant
6598	GO_FOREBRAIN_DEVELOPMENT	-0.90	0.10	0	0	0	not significant
6599	GO_POSITIVE_REGULATION_OF_CELL_PROJECTION_ORGANIZATION	-0.90	0.10	0	0	0	not significant
6600	GO_RESPONSE_TO_LIGHT_STIMULUS	-0.90	0.09	0	0	0	not significant
6601	TBK1.DF_DN	-0.90	0.09	0	0	0	not significant
6602	GO_ION_TRANSMEMBRANE_TRANSPORTER_ACTIVITY	-0.90	0.06	0	0	0	not significant
6603	GO_NEGATIVE_REGULATION_OF_PROTEIN_MODIFICATION_PROCESS	-0.90	0.06	0	0	0	not significant
6604	GO_I_SMAD_BINDING	-0.89	0.24	0	0	0	not significant
6605	GO_POSITIVE_REGULATION_OF_CELL_CYCLE_CHECKPOINT	-0.89	0.24	0	0	0	not significant
6606	GO_RESPONSE_TO_LEUCINE	-0.89	0.24	0	0	0	not significant
6607	GO_TRANSLATION_REPRESSOR_ACTIVITY_MRNA_REGULATORY_ELEME	-0.89	0.24	0	0	0	not significant
6608	GO_VOLUNTARY_MUSCULOSKELETAL_MOVEMENT	-0.89	0.24	0	0	0	not significant
6609	YAMASHITA_METHYLATED_IN_PROSTATE_CANCER	-0.89	0.24	0	0	0	not significant
6610	GO_AP_1_ADAPTOR_COMPLEX	-0.89	0.23	0	0	0	not significant
6611	GO_AXONEMAL_DYNEIN_COMPLEX_ASSEMBLY	-0.89	0.23	0	0	0	not significant
6612	GO_CELLULAR_ALDEHYDE_METABOLIC_PROCESS	-0.89	0.23	0	0	0	not significant
6613	GO_FLOTILLIN_COMPLEX	-0.89	0.23	0	0	0	not significant
6614	GO_LSM2_8_COMPLEX	-0.89	0.23	0	0	0	not significant
6615	GO_REGULATION_OF_GLYCOGEN_CATABOLIC_PROCESS	-0.89	0.23	0	0	0	not significant
6616	GO_REGULATION_OF_HISTONE_DEACETYLASE_ACTIVITY	-0.89	0.23	0	0	0	not significant
6617	GO_WOUND_HEALING_INVOLVED_IN_INFLAMMATORY_RESPONSE	-0.89	0.23	0	0	0	not significant
6618	ONDER_CDH1_TARGETS_3_DN	-0.89	0.23	0	0	0	not significant
6619	REACTOME_NOTCH4_INTRACELLULAR_DOMAIN_REGULATES_TRANSCRI	-0.89	0.23	0	0	0	not significant
6620	WIERENGA_PML_INTERACTOME	-0.89	0.23	0	0	0	not significant
6621	ZHAN_MULTIPLE_MYELOMA_MS_UP	-0.89	0.23	0	0	0	not significant
6622	BIOCARTA_CCR3_PATHWAY	-0.89	0.22	0	0	0	not significant
6623	DAZARD_UV_RESPONSE_CLUSTER_G28	-0.89	0.22	0	0	0	not significant
6624	GO_ACETYLCHOLINE_BINDING	-0.89	0.22	0	0	0	not significant
6625	GO_CHONDROCYTE_DEVELOPMENT_INVOLVED_IN_ENDOCHONDRAL_BO	-0.89	0.22	0	0	0	not significant
6626	GO_POLY_PYRIMIDINE_TRACT_BINDING	-0.89	0.22	0	0	0	not significant
6627	GO_REGULATION_OF_POSTSYNAPTIC_MEMBRANE_ORGANIZATION	-0.89	0.22	0	0	0	not significant
6628	GO_REGULATION_OF_RESPIRATORY_SYSTEM_PROCESS	-0.89	0.22	0	0	0	not significant
6629	GO_RESPONSE_TO_IMMOBILIZATION_STRESS	-0.89	0.22	0	0	0	not significant
6630	GO_VENTRAL_SPINAL_CORD_DEVELOPMENT	-0.89	0.22	0	0	0	not significant
6631	IIZUKA_LIVER_CANCER_PROGRESSION_G2_G3_UP	-0.89	0.22	0	0	0	not significant
6632	MCCABE_HOXC6_TARGETS_CANCER_DN	-0.89	0.22	0	0	0	not significant
6633	NEWMAN_ERCC6_TARGETS_UP	-0.89	0.22	0	0	0	not significant
6634	REACTOME_CRMPS_IN_SEMA3A_SIGNALING	-0.89	0.22	0	0	0	not significant
6635	REACTOME_METABOLISM_OF_FOLATE_AND_PTERINES	-0.89	0.22	0	0	0	not significant
6636	SA_PTEN_PATHWAY	-0.89	0.22	0	0	0	not significant
6637	GO_CELLULAR_RESPONSE_TO_REACTIVE_NITROGEN_SPECIES	-0.89	0.21	0	0	0	not significant

6638	GO_NEGATIVE_REGULATION_OF_INFLAMMATORY_RESPONSE_TO_ANTI(	-0.89	0.21	0	0	0	not significant
6639	GO_NEGATIVE_REGULATION_OF_T_CELL_DIFFERENTIATION	-0.89	0.21	0	0	0	not significant
6640	GO_PEPTIDYL_LYSINE_DIMETHYLATION	-0.89	0.21	0	0	0	not significant
6641	GO_POSITIVE_REGULATION_OF_CHOLESTEROL_EFFLUX	-0.89	0.21	0	0	0	not significant
6642	GO_PRIMARY_MIRNA_PROCESSING	-0.89	0.21	0	0	0	not significant
6643	GO_REGULATION_OF_GRANULOCYTE_CHEMOTAXIS	-0.89	0.21	0	0	0	not significant
6644	GO_REGULATION_OF_PRODUCTION_OF_SMALL_RNA_INVOLVED_IN_GEI	-0.89	0.21	0	0	0	not significant
6645	GO_RESPONSE_TO_COCAINE	-0.89	0.21	0	0	0	not significant
6646	GO_STEROID_HORMONE_SECRETION	-0.89	0.21	0	0	0	not significant
6647	GO_TUBULIN_COMPLEX_ASSEMBLY	-0.89	0.21	0	0	0	not significant
6648	GO_VESICLE_TRANSPORT_ALONG_ACTIN_FILAMENT	-0.89	0.21	0	0	0	not significant
6649	HASLINGER_B_CLL_WITH_13Q14_DELETION	-0.89	0.21	0	0	0	not significant
6650	REACTOME_PLATELET_CALCIUM_HOMEOSTASIS	-0.89	0.21	0	0	0	not significant
6651	REACTOME_RHO_GTPASES_ACTIVATE_CIT	-0.89	0.21	0	0	0	not significant
6652	ULE_SPLICING_VIA_NOVA2	-0.89	0.21	0	0	0	not significant
6653	ZEMBUTSU_SENSITIVITY_TO_VINBLASTINE	-0.89	0.21	0	0	0	not significant
6654	GO_LIPASE_ACTIVITY	-0.89	0.20	0	0	0	not significant
6655	GO_PROTEIN_QUALITY_CONTROL_FOR_MISFOLDED_OR_INCOMPLETEL	-0.89	0.20	0	0	0	not significant
6656	GO_RESPIRATORY_GASEOUS_EXCHANGE	-0.89	0.20	0	0	0	not significant
6657	GO_U2_SNRNP	-0.89	0.20	0	0	0	not significant
6658	KUMAMOTO_RESPONSE_TO_NUTLIN_3A_UP	-0.89	0.20	0	0	0	not significant
6659	BIOCARTA_TOLL_PATHWAY	-0.89	0.19	0	0	0	not significant
6660	GO_AMINOPEPTIDASE_ACTIVITY	-0.89	0.19	0	0	0	not significant
6661	GO_CHROMATIN_REMODELING_AT_CENTROMERE	-0.89	0.19	0	0	0	not significant
6662	GO_REGULATION_OF_INTRACELLULAR_ESTROGEN_RECEPTOR_SIGNAL	-0.89	0.19	0	0	0	not significant
6663	AMIT_EGF_RESPONSE_120_MCF10A	-0.89	0.18	0	0	0	not significant
6664	GAVIN_FOXP3_TARGETS_CLUSTER_P7	-0.89	0.18	0	0	0	not significant
6665	GO_REGULATION_OF_PROTEIN_TARGETING_TO_MITOCHONDRION	-0.89	0.18	0	0	0	not significant
6666	GO_REGULATION_OF_SMOOTHENED_SIGNALING_PATHWAY	-0.89	0.18	0	0	0	not significant
6667	NELSON_RESPONSE_TO_ANDROGEN_UP	-0.89	0.18	0	0	0	not significant
6668	ZHOU_TNF_SIGNALING_4HR	-0.89	0.18	0	0	0	not significant
6669	CADWELL_ATG16L1_TARGETS_DN	-0.89	0.17	0	0	0	not significant
6670	HILLION_HMGA1_TARGETS	-0.89	0.17	0	0	0	not significant
6671	GO_REGULATION_OF_FAT_CELL_DIFFERENTIATION	-0.89	0.16	0	0	0	not significant
6672	GO_CELLULAR_RESPONSE_TO_UV	-0.89	0.15	0	0	0	not significant
6673	GO_CLATHRIN_COATED_VESICLE_MEMBRANE	-0.89	0.15	0	0	0	not significant
6674	GO_UBIQUITIN_LIKE_PROTEIN_BINDING	-0.89	0.15	0	0	0	not significant
6675	GO_NEUROTRANSMITTER_TRANSPORT	-0.89	0.14	0	0	0	not significant
6676	GO_REGULATION_OF_DENDRITE_DEVELOPMENT	-0.89	0.14	0	0	0	not significant
6677	AGUIRRE_PANCREATIC_CANCER_COPY_NUMBER_DN	-0.89	0.11	0	0	0	not significant
6678	BRUINS_UVC_RESPONSE_VIA_TP53_GROUP_B	-0.89	0.06	0	0	0	not significant
6679	GO_GTPASE_BINDING	-0.89	0.04	0	0	0	not significant
6680	GO_PROTEIN_MODIFICATION_BY_SMALL_PROTEIN_CONJUGATION	-0.89	0.02	0	0	0	not significant
6681	GO_NUCLEOPLASM_PART	-0.89	0.00	0	0	0	not significant
6682	GO_RIBONUCLEOPROTEIN_COMPLEX_DISASSEMBLY	-0.88	0.24	0	0	0	not significant
6683	BIOCARTA_LAIR_PATHWAY	-0.88	0.23	0	0	0	not significant
6684	GO_OXIDOREDUCTASE_ACTIVITY_ACTING_ON_A_SULFUR_GROUP_OF_I	-0.88	0.23	0	0	0	not significant
6685	RORIE_TARGETS_OF_EWSR1_FLI1_FUSION_UP	-0.88	0.23	0	0	0	not significant
6686	WU_HBX_TARGETS_1_UP	-0.88	0.23	0	0	0	not significant
6687	GO_AMPA_GLUTAMATE_RECEPTOR_CLUSTERING	-0.88	0.22	0	0	0	not significant
6688	GO_COENZYME_A_METABOLIC_PROCESS	-0.88	0.22	0	0	0	not significant
6689	GO_HISTONE_PRE_MRNA_3_END_PROCESSING_COMPLEX	-0.88	0.22	0	0	0	not significant
6690	GO_MITOGEN_ACTIVATED_PROTEIN_KINASE_KINASE_KINASE_BINDING	-0.88	0.22	0	0	0	not significant
6691	GO_O_ACETYLTRANSFERASE_ACTIVITY	-0.88	0.22	0	0	0	not significant
6692	GO_REGULATION_OF_SKELETAL_MUSCLE_FIBER_DEVELOPMENT	-0.88	0.22	0	0	0	not significant
6693	REACTOME_REGULATION_OF_PYRUVATE_DEHYDROGENASE_PDH_COM	-0.88	0.22	0	0	0	not significant
6694	BEGUM_TARGETS_OF_PAX3_FOXO1_FUSION_DN	-0.88	0.21	0	0	0	not significant
6695	GO_ACETYLCHOLINE_RECEPTOR_ACTIVITY	-0.88	0.21	0	0	0	not significant
6696	GO_HISTONE_H2B_UBIQUITINATION	-0.88	0.21	0	0	0	not significant
6697	GO_OXIDOREDUCTASE_ACTIVITY_ACTING_ON_THE_CH_NH2_GROUP_OI	-0.88	0.21	0	0	0	not significant
6698	GO_PERICARDIUM_DEVELOPMENT	-0.88	0.21	0	0	0	not significant
6699	GO_POSITIVE_REGULATION_OF_CELL_MIGRATION_BY_VASCULAR_END(	-0.88	0.21	0	0	0	not significant
6700	GO_REGULATION_OF_NEUTROPHIL_MIGRATION	-0.88	0.21	0	0	0	not significant
6701	GO_TRICARBOXYLIC_ACID_METABOLIC_PROCESS	-0.88	0.21	0	0	0	not significant
6702	PID_HES_HEY_PATHWAY	-0.88	0.21	0	0	0	not significant
6703	GO_ARGININE_CATABOLIC_PROCESS	-0.88	0.20	0	0	0	not significant
6704	GO_CHAPERONE_MEDIATED_AUTOPHAGY	-0.88	0.20	0	0	0	not significant
6705	GO_NUCLEOTIDE_SUGAR_METABOLIC_PROCESS	-0.88	0.20	0	0	0	not significant
6706	GO_POSITIVE_REGULATION_OF_PHOSPHOPROTEIN_PHOSPHATASE_AC	-0.88	0.20	0	0	0	not significant
6707	GO_POSITIVE_REGULATION_OF_SYNAPSE_ASSEMBLY	-0.88	0.20	0	0	0	not significant
6708	REACTOME_INTERLEUKIN_37_SIGNALING	-0.88	0.20	0	0	0	not significant
6709	REACTOME_INTERLEUKIN_RECEPTOR_SHC_SIGNALING	-0.88	0.20	0	0	0	not significant
6710	SCHWAB_TARGETS_OF_BMYB_POLYMORPHIC_VARIANTS_UP	-0.88	0.20	0	0	0	not significant
6711	STEINER_ERYTHROCYTE_MEMBRANE_GENES	-0.88	0.20	0	0	0	not significant
6712	TSENG_ADIPOGENIC_POTENTIAL_UP	-0.88	0.20	0	0	0	not significant
6713	TURASHVILI_BREAST_LOBULAR_CARCINOMA_VS_DUCTAL_NORMAL_DN	-0.88	0.20	0	0	0	not significant
6714	ZHAN_VARIABLE_EARLY_DIFFERENTIATION_GENES_UP	-0.88	0.20	0	0	0	not significant
6715	GO_POSITIVE_REGULATION_OF_PROTEIN_EXPORT_FROM_NUCLEUS	-0.88	0.19	0	0	0	not significant
6716	GO_REGULATION_OF_PHOSPHOLIPID_METABOLIC_PROCESS	-0.88	0.19	0	0	0	not significant
6717	GO_SKELETAL_MUSCLE_ADAPTATION	-0.88	0.19	0	0	0	not significant
6718	JIANG_TIP30_TARGETS_UP	-0.88	0.19	0	0	0	not significant
6719	MARTIN_NFKB_TARGETS_UP	-0.88	0.19	0	0	0	not significant
6720	BURTON_ADIPOGENESIS_7	-0.88	0.18	0	0	0	not significant
6721	GO_ENDOCRINE_PROCESS	-0.88	0.18	0	0	0	not significant
6722	GO_MONONUCLEAR_CELL_DIFFERENTIATION	-0.88	0.18	0	0	0	not significant
6723	GO_NEGATIVE_REGULATION_OF_DNA_BINDING	-0.88	0.18	0	0	0	not significant
6724	GO_POLYOL_CATABOLIC_PROCESS	-0.88	0.18	0	0	0	not significant
6725	GO_PROTEIN_INSERTION_INTO_MEMBRANE	-0.88	0.18	0	0	0	not significant
6726	GO_SAGA_TYPE_COMPLEX	-0.88	0.18	0	0	0	not significant
6727	JIANG_AGING_HYPOTHALAMUS_DN	-0.88	0.18	0	0	0	not significant
6728	KAAB_FAILED_HEART_VENTRICLE_DN	-0.88	0.18	0	0	0	not significant
6729	PID_IGF1_PATHWAY	-0.88	0.18	0	0	0	not significant
6730	REACTOME_PLASMA_LIPOPROTEIN_ASSEMBLY_REMODELING_AND_CLE	-0.88	0.18	0	0	0	not significant
6731	BIOCARTA_EIF4_PATHWAY	-0.88	0.17	0	0	0	not significant
6732	ZHAN_MULTIPLE_MYELOMA_CD1_VS_CD2_DN	-0.88	0.17	0	0	0	not significant
6733	GO_DNA_SYNTHESIS_INVOLVED_IN_DNA_REPAIR	-0.88	0.16	0	0	0	not significant
6734	GO_REGULATION_OF_PROTEIN_IMPORT	-0.88	0.15	0	0	0	not significant
6735	BOUDOUKHA_BOUND_BY_IGF2BP2	-0.88	0.14	0	0	0	not significant
6736	GO_DEVELOPMENTAL_CELL_GROWTH	-0.88	0.12	0	0	0	not significant
6737	GO_ACETYLTRANSFERASE_ACTIVITY	-0.88	0.10	0	0	0	not significant
6738	ZHOU_INFLAMMATORY_RESPONSE_LIVE_DN	-0.88	0.06	0	0	0	not significant
6739	GO_CENTROSOME	-0.88	0.03	0	0	0	not significant
6740	GO_REGULATION_OF_MIRNA_METABOLIC_PROCESS	-0.87	0.23	0	0	0	not significant
6741	GO_CYSTEINE_TYPE_ENDOPEPTIDASE_INHIBITOR_ACTIVITY_INVOLVED_	-0.87	0.22	0	0	0	not significant
6742	GO_POSITIVE_REGULATION_OF_HEART_RATE	-0.87	0.22	0	0	0	not significant
6743	GO_REGULATION_OF_RENAL_SYSTEM_PROCESS	-0.87	0.22	0	0	0	not significant
6744	GO_RESPONSE_TO_MERCURY_ION	-0.87	0.22	0	0	0	not significant
6745	GO_UBIQUITIN_LIKE_MODIFIER_ACTIVATING_ENZYME_ACTIVITY	-0.87	0.22	0	0	0	not significant
6746	OUELLET_OVARIAN_CANCER_INVASIVE_VS_LMP_DN	-0.87	0.22	0	0	0	not significant

6747	BIOCARTA_SODD_PATHWAY	-0.87	0.21	0	0	0	not significant
6748	GO_ACETYL_COA_BIOSYNTHETIC_PROCESS_FROM_PYRUVATE	-0.87	0.21	0	0	0	not significant
6749	GO_LIPOPROTEIN_LOCALIZATION	-0.87	0.21	0	0	0	not significant
6750	GO_NEGATIVE_REGULATION_OF_CARDIAC_MUSCLE_CELL_DIFFERENTIA	-0.87	0.21	0	0	0	not significant
6751	GO_POSITIVE_REGULATION_OF_INTERLEUKIN_6_SECRETION	-0.87	0.21	0	0	0	not significant
6752	GO_PROTEIN_LOCALIZATION_TO_CELL_CORTEX	-0.87	0.21	0	0	0	not significant
6753	GO_REGULATION_OF_PRESYNAPTIC_CYTOSOLIC_CALCIUM_ION_CONCE	-0.87	0.21	0	0	0	not significant
6754	GO_REGULATION_OF_T_CIRCLE_FORMATION	-0.87	0.21	0	0	0	not significant
6755	GO_SUCKLING_BEHAVIOR	-0.87	0.21	0	0	0	not significant
6756	GO_SULFUR_AMINO_ACID_METABOLIC_PROCESS	-0.87	0.21	0	0	0	not significant
6757	GO_U6_SNRNP	-0.87	0.21	0	0	0	not significant
6758	VALK_AML_CLUSTER_1	-0.87	0.21	0	0	0	not significant
6759	YANAGIHARA_ESX1_TARGETS	-0.87	0.21	0	0	0	not significant
6760	GO_DEOXYRIBOSE_PHOSPHATE_CATABOLIC_PROCESS	-0.87	0.20	0	0	0	not significant
6761	GO_DOPAMINERGIC_NEURON_DIFFERENTIATION	-0.87	0.20	0	0	0	not significant
6762	GO_HEPATOCYTE_APOPTOTIC_PROCESS	-0.87	0.20	0	0	0	not significant
6763	GO_MYOBLAST_PROLIFERATION	-0.87	0.20	0	0	0	not significant
6764	GO PLUS END DIRECTED ORGANELLE TRANSPORT ALONG MICROTU	-0.87	0.20	0	0	0	not significant
6765	GO_REGULATION_OF_ACTION_POTENTIAL	-0.87	0.20	0	0	0	not significant
6766	GO_THIOESTERASE_BINDING	-0.87	0.20	0	0	0	not significant
6767	GO_U1_SNRNA_BINDING	-0.87	0.20	0	0	0	not significant
6768	HOLLEMAN_PREDNISOLONE_RESISTANCE_ALL_UP	-0.87	0.20	0	0	0	not significant
6769	INAMURA_LUNG_CANCER_SCC_SUBTYPES_UP	-0.87	0.20	0	0	0	not significant
6770	BIOCARTA_NKCELLS_PATHWAY	-0.87	0.19	0	0	0	not significant
6771	BURTON_ADIPOGENESIS_1	-0.87	0.19	0	0	0	not significant
6772	GO_LIPOPROTEIN_CATABOLIC_PROCESS	-0.87	0.19	0	0	0	not significant
6773	GO_POSITIVE_REGULATION_OF_STRIATED_MUSCLE_CELL_DIFFERENTIA	-0.87	0.19	0	0	0	not significant
6774	GO_REGULATION_OF_ANIMAL_ORGAN_FORMATION	-0.87	0.19	0	0	0	not significant
6775	GO_REGULATION_OF_NEUROBLAST_PROLIFERATION	-0.87	0.19	0	0	0	not significant
6776	REACTOME_GLYCOGEN_STORAGE_DISEASES	-0.87	0.19	0	0	0	not significant
6777	REACTOME_ROLE_OF_PHOSPHOLIPIDS_IN_PHAGOCYTOSIS	-0.87	0.19	0	0	0	not significant
6778	WANG_RESPONSE_TO_BEXAROTENE_DN	-0.87	0.19	0	0	0	not significant
6779	WENG_POR_TARGETS_GLOBAL_UP	-0.87	0.19	0	0	0	not significant
6780	YAP1_DN	-0.87	0.19	0	0	0	not significant
6781	GO POSITIVE REGULATION OF MYELOID LEUKOCYTE MEDIATED IMMU	-0.87	0.18	0	0	0	not significant
6782	LIU_LIVER_CANCER	-0.87	0.18	0	0	0	not significant
6783	PARENT_MTOR_SIGNALING_DN	-0.87	0.18	0	0	0	not significant
6784	PARK_HSC_AND_MULTIPOTENT_PROGENITORS	-0.87	0.18	0	0	0	not significant
6785	PETROVA_PROX1_TARGETS_UP	-0.87	0.18	0	0	0	not significant
6786	REACTOME_PIWI_INTERACTING_RNA_PIRNA_BIOGENESIS	-0.87	0.18	0	0	0	not significant
6787	CHR8Q21	-0.87	0.17	0	0	0	not significant
6788	GO_CELLULAR_METABOLIC_COMPOUND_SALVAGE	-0.87	0.17	0	0	0	not significant
6789	GO_MRNA_5_UTR_BINDING	-0.87	0.17	0	0	0	not significant
6790	PID_ER_NONGENOMIC_PATHWAY	-0.87	0.17	0	0	0	not significant
6791	GUO_HEX_TARGETS_UP	-0.87	0.16	0	0	0	not significant
6792	LEE_TARGETS_OF_PTCH1_AND_SUFU_UP	-0.87	0.16	0	0	0	not significant
6793	GO_APICAL_JUNCTION_ASSEMBLY	-0.87	0.15	0	0	0	not significant
6794	GO_POSITIVE_REGULATION_OF_ENDOCYTOSIS	-0.87	0.15	0	0	0	not significant
6795	GO REGULATION OF ANTIGEN RECEPTOR MEDIATED SIGNALING PAT	-0.87	0.15	0	0	0	not significant
6796	GO_REGULATION_OF_CELL_JUNCTION_ASSEMBLY	-0.87	0.15	0	0	0	not significant
6797	REACTOME_AMYLOID_FIBER_FORMATION	-0.87	0.15	0	0	0	not significant
6798	ZHAN_MULTIPLE_MYELOMA_CD1_AND_CD2_DN	-0.87	0.15	0	0	0	not significant
6799	GO_CORTICAL_CYTOSKELETON	-0.87	0.14	0	0	0	not significant
6800	GO_RECYCLING_ENDOSOME_MEMBRANE	-0.87	0.14	0	0	0	not significant
6801	GO_SPLICEOSOMAL_COMPLEX_ASSEMBLY	-0.87	0.14	0	0	0	not significant
6802	GO_SYNAPTIC_VESICLE_EXOCYTOSIS	-0.87	0.14	0	0	0	not significant
6803	PRC1_BMI_UP.V1_DN	-0.87	0.14	0	0	0	not significant
6804	REACTOME_TOLL_LIKE_RECEPTOR_TLR1:TLR2_CASCADE	-0.87	0.14	0	0	0	not significant
6805	GO_ENSHEATHMENT_OF_NEURONS	-0.87	0.13	0	0	0	not significant
6806	GO_POSITIVE_REGULATION_OF_NEURON_DEATH	-0.87	0.13	0	0	0	not significant
6807	GO_EARLY_ENDOSOME_MEMBRANE	-0.87	0.12	0	0	0	not significant
6808	GO_CALCIUM_ION_REGULATED_EXOCYTOSIS	-0.87	0.11	0	0	0	not significant
6809	REACTOME_CLATHRIN_MEDIATED_ENDOCYTOSIS	-0.87	0.11	0	0	0	not significant
6810	GO REGULATION OF PROTEIN CATABOLIC PROCESS IN THE VACUOL	-0.86	0.22	0	0	0	not significant
6811	RAMJAUN_APOPTOSIS_BY_TGFB1_VIA_MAPK1_DN	-0.86	0.22	0	0	0	not significant
6812	DACOSTA_LOW_DOSE_UV_RESPONSE_VIA_ERCC3_XPCS_UP	-0.86	0.21	0	0	0	not significant
6813	KOINUMA_COLON_CANCER_MSI_DN	-0.86	0.21	0	0	0	not significant
6814	MCMURRAY_TP53_HRAS_COOPERATION_RESPONSE_UP	-0.86	0.21	0	0	0	not significant
6815	BIOCARTA_VIP_PATHWAY	-0.86	0.20	0	0	0	not significant
6816	CHR9P21	-0.86	0.20	0	0	0	not significant
6817	DASU_IL6_SIGNALING_SCAR_DN	-0.86	0.20	0	0	0	not significant
6818	GO_CELLULAR_RESPONSE_TO_IRON_ION	-0.86	0.20	0	0	0	not significant
6819	GO_CYTOPLASMIC_SEQUESTERING_OF_NF_KAPPAB	-0.86	0.20	0	0	0	not significant
6820	GO_POSITIVE_REGULATION_BY_HOST_OF_VIRAL_TRANSCRIPTION	-0.86	0.20	0	0	0	not significant
6821	GO_POSITIVE_REGULATION_OF_ADHERENS_JUNCTION_ORGANIZATION	-0.86	0.20	0	0	0	not significant
6822	GO_POSITIVE_REGULATION_OF_AMINE_TRANSPORT	-0.86	0.20	0	0	0	not significant
6823	GO_PURINERGIC_RECEPTOR_SIGNALING_PATHWAY	-0.86	0.20	0	0	0	not significant
6824	STEGMEIER_PREMITOTIC_CELL_CYCLE_REGULATORS	-0.86	0.20	0	0	0	not significant
6825	GO_ATPASE_ACTIVATOR_ACTIVITY	-0.86	0.19	0	0	0	not significant
6826	GO_CD95_DEATH_INDUCING_SIGNALING_COMPLEX	-0.86	0.19	0	0	0	not significant
6827	GO_ENDOPLASMIC_RETICULUM_SUBCOMPARTMENT	-0.86	0.19	0	0	0	not significant
6828	GO_FEMALE_PRONUCLEUS	-0.86	0.19	0	0	0	not significant
6829	GO_NEGATIVE_REGULATION_OF_HISTONE_ACETYLATION	-0.86	0.19	0	0	0	not significant
6830	GO_NEUROTRANSMITTER_UPTAKE	-0.86	0.19	0	0	0	not significant
6831	GO_NUCLEAR_TRANSCRIPTIONAL_REPRESSOR_COMPLEX	-0.86	0.19	0	0	0	not significant
6832	GO_PROTEIN_LOCALIZATION_TO_MICROTUBULE	-0.86	0.19	0	0	0	not significant
6833	GO_RESPONSE_TO_HYDROPEROXIDE	-0.86	0.19	0	0	0	not significant
6834	MARKS_ACETYLATED_NON_HISTONE_PROTEINS	-0.86	0.19	0	0	0	not significant
6835	ROYLANCE_BREAST_CANCER_16Q_COPY_NUMBER_DN	-0.86	0.19	0	0	0	not significant
6836	GO_EPIDERMAL_GROWTH_FACTOR_RECEPTOR_BINDING	-0.86	0.18	0	0	0	not significant
6837	GO_EPSILON_DNA_POLYMERASE_COMPLEX	-0.86	0.18	0	0	0	not significant
6838	GO_FERRIC_IRON_BINDING	-0.86	0.18	0	0	0	not significant
6839	GO_G_QUADRUPLEX_DNA_UNWINDING	-0.86	0.18	0	0	0	not significant
6840	GO_MITOTIC_SPINDLE_MIDZONE	-0.86	0.18	0	0	0	not significant
6841	GO_OLIGOSACCHARYLTRANSFERASE_COMPLEX	-0.86	0.18	0	0	0	not significant
6842	GO_PHOSPHATIDYLINOSITOL_3_KINASE_REGULATOR_ACTIVITY	-0.86	0.18	0	0	0	not significant
6843	GO_REGULATION_OF_HISTONE_H3_K36_METHYLATION	-0.86	0.18	0	0	0	not significant
6844	GO_REGULATION_OF_NEURON_MIGRATION	-0.86	0.18	0	0	0	not significant
6845	GO_REGULATION_OF_RHODOPSIN_MEDIATED_SIGNALING_PATHWAY	-0.86	0.18	0	0	0	not significant
6846	MIKKELSEN_NPC_LCP_WITH_H3K4ME3	-0.86	0.18	0	0	0	not significant
6847	SHI_SPARC_TARGETS_UP	-0.86	0.18	0	0	0	not significant
6848	CHR16Q23	-0.86	0.17	0	0	0	not significant
6849	CHR1Q31	-0.86	0.17	0	0	0	not significant
6850	CHR5Q11	-0.86	0.17	0	0	0	not significant
6851	GO_ENDOPLASMIC_RETICULUM_CALCIUM_ION_HOMEOSTASIS	-0.86	0.17	0	0	0	not significant
6852	GO_IRES_DEPENDENT_VIRAL_TRANSLATIONAL_INITIATION	-0.86	0.17	0	0	0	not significant
6853	GO_LABYRINTHINE_LAYER_DEVELOPMENT	-0.86	0.17	0	0	0	not significant
6854	GO_PRE_MRNA_BINDING	-0.86	0.17	0	0	0	not significant
6855	KEGG_REGULATION_OF_AUTOPHAGY	-0.86	0.17	0	0	0	not significant

6856	MATZUK_CENTRAL_FOR_FEMALE_FERTILITY	-0.86	0.17	0	0	0	not significant
6857	QI_HYPOXIA_TARGETS_OF_HIF1A_AND_FOXA2	-0.86	0.17	0	0	0	not significant
6858	BIOCARTA_P38MAPK_PATHWAY	-0.86	0.16	0	0	0	not significant
6859	CHR2Q24	-0.86	0.16	0	0	0	not significant
6860	GARGALOVIC_RESPONSE_TO_OXIDIZED_PHOSPHOLIPIDS_GREEN_DN	-0.86	0.16	0	0	0	not significant
6861	GO_KINETOCHORE_ORGANIZATION	-0.86	0.16	0	0	0	not significant
6862	GO_POSITIVE_REGULATION_OF_RESPONSE_TO_BIOTIC_STIMULUS	-0.86	0.16	0	0	0	not significant
6863	LEE_LIVER_CANCER_ACOX1_UP	-0.86	0.16	0	0	0	not significant
6864	GO_PROTEIN_EXIT_FROM_ENDOPLASMIC_RETICULUM	-0.86	0.15	0	0	0	not significant
6865	AMIT_SERUM_RESPONSE_120_MCF10A	-0.86	0.14	0	0	0	not significant
6866	GO_REGULATION_OF_CENTROSOME_CYCLE	-0.86	0.14	0	0	0	not significant
6867	GO_REGULATION_OF_RESPONSE_TO_DRUG	-0.86	0.14	0	0	0	not significant
6868	MILI_PSEUDOPODIA	-0.86	0.14	0	0	0	not significant
6869	PID_FAK_PATHWAY	-0.86	0.14	0	0	0	not significant
6870	REACTOME_FOXO_MEDIATED_TRANSCRIPTION	-0.86	0.14	0	0	0	not significant
6871	LIU_COMMON_CANCER_GENES	-0.86	0.13	0	0	0	not significant
6872	REACTOME_STIMULI_SENSING_CHANNELS	-0.86	0.13	0	0	0	not significant
6873	GO_POSITIVE_REGULATION_OF_ORGANELLE_ASSEMBLY	-0.86	0.12	0	0	0	not significant
6874	REACTOME_MYD88_CASCADE_INITIATED_ON_PLASMA_MEMBRANE	-0.86	0.11	0	0	0	not significant
6875	GO_TUBE_FORMATION	-0.86	0.10	0	0	0	not significant
6876	MCBRYAN_PUBERTAL_TGFB1_TARGETS_UP	-0.86	0.10	0	0	0	not significant
6877	GO_HORMONE_MEDIATED_SIGNALING_PATHWAY	-0.86	0.09	0	0	0	not significant
6878	KAYO_AGING_MUSCLE_DN	-0.86	0.09	0	0	0	not significant
6879	MCBRYAN_PUBERTAL_BREAST_6_7WK_UP	-0.86	0.09	0	0	0	not significant
6880	GO_NUCLEASE_ACTIVITY	-0.86	0.08	0	0	0	not significant
6881	GO_GLYCEROLIPID_BIOSYNTHETIC_PROCESS	-0.86	0.07	0	0	0	not significant
6882	GO_NUCLEAR_CHROMATIN	-0.86	0.04	0	0	0	not significant
6883	GO_REGULATION_OF_MAPK_CASCADE	-0.86	0.04	0	0	0	not significant
6884	GO_INTRAMOLECULAR_TRANSFERASE_ACTIVITY_PHOSPHOTRANSFERA	-0.85	0.22	0	0	0	not significant
6885	GO_IRON_ION_TRANSMEMBRANE_TRANSPORTER_ACTIVITY	-0.85	0.20	0	0	0	not significant
6886	GO_NEGATIVE_REGULATION_OF_CALCIUM_ION_TRANSPORT_INTO_CYT	-0.85	0.20	0	0	0	not significant
6887	GO_POSITIVE_REGULATION_OF_MACROPHAGE_CHEMOTAXIS	-0.85	0.20	0	0	0	not significant
6888	GO_REGULATION_OF_NEUROINFLAMMATORY_RESPONSE	-0.85	0.20	0	0	0	not significant
6889	REACTOME_NOREPINEPHRINE_NEUROTRANSMITTER_RELEASE_CYCLE	-0.85	0.20	0	0	0	not significant
6890	REACTOME_SYNTHESIS_OF_PIPS_AT_THE_GOLGI_MEMBRANE	-0.85	0.20	0	0	0	not significant
6891	REACTOME_TRISTETRAPROLIN_TTP_ZFP36_BINDS_AND_DESTABILIZES_	-0.85	0.20	0	0	0	not significant
6892	GO_3_HYDROXYACYL_COA_DEHYDROGENASE_ACTIVITY	-0.85	0.19	0	0	0	not significant
6893	GO_MALE_MATING_BEHAVIOR	-0.85	0.19	0	0	0	not significant
6894	GO_METANEPHRIC_NEPHRON_EPITHELIUM_DEVELOPMENT	-0.85	0.19	0	0	0	not significant
6895	GO_MUTLALPHA_COMPLEX_BINDING	-0.85	0.19	0	0	0	not significant
6896	GO_NEGATIVE_REGULATION_OF_ANDROGEN_RECEPTOR_SIGNALING_P	-0.85	0.19	0	0	0	not significant
6897	GO_NEGATIVE_REGULATION_OF_CYTOSOLIC_CALCIUM_ION_CONCENTF	-0.85	0.19	0	0	0	not significant
6898	GO_PROTEIN_MEMBRANE_ANCHOR	-0.85	0.19	0	0	0	not significant
6899	GO_RAGULATOR_COMPLEX	-0.85	0.19	0	0	0	not significant
6900	MURAKAMI_UV_RESPONSE_1HR_DN	-0.85	0.19	0	0	0	not significant
6901	REACTOME_HEME_BIOSYNTHESIS	-0.85	0.19	0	0	0	not significant
6902	GO_BETA_CATENIN_TCF_COMPLEX	-0.85	0.18	0	0	0	not significant
6903	GO_CATALYTIC_STEP_1_SPLICEOSOME	-0.85	0.18	0	0	0	not significant
6904	GO_DEATH_INDUCING_SIGNALING_COMPLEX	-0.85	0.18	0	0	0	not significant
6905	GO_NEGATIVE_REGULATION_OF_ANOIKIS	-0.85	0.18	0	0	0	not significant
6906	GO_NEGATIVE_REGULATION_OF_CARDIOCYTE_DIFFERENTIATION	-0.85	0.18	0	0	0	not significant
6907	GO_NEGATIVE_REGULATION_OF_T_CELL_MEDIATED_IMMUNITY	-0.85	0.18	0	0	0	not significant
6908	GO_REGULATION_OF_THYMOCYTE_APOPTOTIC_PROCESS	-0.85	0.18	0	0	0	not significant
6909	REACTOME_ACTIVATION_OF_MATRIX_METALLOPROTEINASES	-0.85	0.18	0	0	0	not significant
6910	REACTOME_DEFECTS_IN_BIOTIN_BTN_METABOLISM	-0.85	0.18	0	0	0	not significant
6911	REACTOME_HS_GAG_BIOSYNTHESIS	-0.85	0.18	0	0	0	not significant
6912	REACTOME_RAF_INDEPENDENT_MAPK1_3_ACTIVATION	-0.85	0.18	0	0	0	not significant
6913	SA_B_CELL_RECEPTOR_COMPLEXES	-0.85	0.18	0	0	0	not significant
6914	YIH_RESPONSE_TO_ARSENITE_C2	-0.85	0.18	0	0	0	not significant
6915	CUI_TCF21_TARGETS_DN	-0.85	0.17	0	0	0	not significant
6916	GO_FEAR_RESPONSE	-0.85	0.17	0	0	0	not significant
6917	GO_NEGATIVE_REGULATION_OF_JNK_CASCADE	-0.85	0.17	0	0	0	not significant
6918	GO_NEURON_PROJECTION_EXTENSION_INVOLVED_IN_NEURON_PROJEC	-0.85	0.17	0	0	0	not significant
6919	GO_OOCYTE_MATURATION	-0.85	0.17	0	0	0	not significant
6920	GO_PROTEIN_PHOSPHATASE_TYPE_1_COMPLEX	-0.85	0.17	0	0	0	not significant
6921	GO_TRANSFERRIN_TRANSPORT	-0.85	0.17	0	0	0	not significant
6922	GO_TUMOR_NECROSIS_FACTOR_RECEPTOR_BINDING	-0.85	0.17	0	0	0	not significant
6923	REACTOME_AKT_PHOSPHORYLATES_TARGETS_IN_THE_NUCLEUS	-0.85	0.17	0	0	0	not significant
6924	GO_MODIFICATION_OF_SYNAPTIC_STRUCTURE	-0.85	0.16	0	0	0	not significant
6925	GO_POSITIVE_REGULATION_OF_MITOCHONDRIAL_MEMBRANE_POTENTI	-0.85	0.16	0	0	0	not significant
6926	LIANG_HEMATOPOIESIS_STEM_CELL_NUMBER_SMALL_VS_HUGE_DN	-0.85	0.16	0	0	0	not significant
6927	PID_MYC_PATHWAY	-0.85	0.16	0	0	0	not significant
6928	REACTOME_ELEVATION_OF_CYTOSOLIC_CA2PLUS_LEVELS	-0.85	0.16	0	0	0	not significant
6929	GO_ATP_METABOLIC_PROCESS	-0.85	0.15	0	0	0	not significant
6930	GO_ZINC_ION_TRANSMEMBRANE_TRANSPORT	-0.85	0.15	0	0	0	not significant
6931	LEE_TARGETS_OF_PTCH1_AND_SUFU_DN	-0.85	0.15	0	0	0	not significant
6932	REACTOME_TRANSLESION_SYNTHESIS_BY_Y_FAMILY_DNA_POLYMERAS	-0.85	0.15	0	0	0	not significant
6933	GENTILE_UV_RESPONSE_CLUSTER_D8	-0.85	0.14	0	0	0	not significant
6934	GO_MATERNAL_PROCESS_INVOLVED_IN_FEMALE_PREGNANCY	-0.85	0.14	0	0	0	not significant
6935	GO_MONOSACCHARIDE_CATABOLIC_PROCESS	-0.85	0.14	0	0	0	not significant
6936	GO_MYOBLAST_DIFFERENTIATION	-0.85	0.14	0	0	0	not significant
6937	GO_SMOOTH_MUSCLE_CELL_MIGRATION	-0.85	0.14	0	0	0	not significant
6938	KENNY_CTNNB1_TARGETS_DN	-0.85	0.14	0	0	0	not significant
6939	MAHAJAN_RESPONSE_TO_IL1A_DN	-0.85	0.14	0	0	0	not significant
6940	GO_BLASTOCYST_FORMATION	-0.85	0.13	0	0	0	not significant
6941	GO_REGULATION_OF_TORC1_SIGNALING	-0.85	0.13	0	0	0	not significant
6942	GO_NEGATIVE_REGULATION_OF_ION_TRANSMEMBRANE_TRANSPORT	-0.85	0.12	0	0	0	not significant
6943	GO_REGULATION_OF_ACTIN_FILAMENT_BUNDLE_ASSEMBLY	-0.85	0.12	0	0	0	not significant
6944	GO_SWI_SNF_SUPERFAMILY_TYPE_COMPLEX	-0.85	0.12	0	0	0	not significant
6945	GO_NEUTRAL_LIPID_METABOLIC_PROCESS	-0.85	0.11	0	0	0	not significant
6946	GO_P53_BINDING	-0.85	0.11	0	0	0	not significant
6947	GO_CEREBRAL_CORTEX_DEVELOPMENT	-0.85	0.10	0	0	0	not significant
6948	PID_HDAC_CLASSI_PATHWAY	-0.85	0.10	0	0	0	not significant
6949	GO_REGULATION_OF_EXTRINSIC_APOPTOTIC_SIGNALING_PATHWAY	-0.85	0.08	0	0	0	not significant
6950	GO_MICROTUBULE_BASED_MOVEMENT	-0.85	0.05	0	0	0	not significant
6951	LINSLEY_MIR16_TARGETS	-0.85	0.04	0	0	0	not significant
6952	PUJANA_BRCA2_PCC_NETWORK	-0.85	0.02	0	0	0	not significant
6953	GO_REGULATION_OF_GTPASE_ACTIVITY	-0.85	0.01	0	0	0	not significant
6954	GO_CELLULAR_RESPONSE_TO_DNA_DAMAGE_STIMULUS	-0.85	0.00	0	0	0	not significant
6955	GO_CHROMATIN	-0.85	0.00	0	0	0	not significant
6956	NADERI_BREAST_CANCER_PROGNOSIS_DN	-0.84	0.20	0	0	0	not significant
6957	GO_DISTAL_TUBULE_DEVELOPMENT	-0.84	0.19	0	0	0	not significant
6958	GO_ENDORIBONUCLEASE_ACTIVITY_PRODUCING_3_PHOSPHOMONOEST	-0.84	0.19	0	0	0	not significant
6959	GO_ER_OVERLOAD_RESPONSE	-0.84	0.19	0	0	0	not significant
6960	GO_GALACTOSE_METABOLIC_PROCESS	-0.84	0.19	0	0	0	not significant
6961	GO_MACROPINOCYTOSIS	-0.84	0.19	0	0	0	not significant
6962	GO_NEGATIVE_REGULATION_OF_PRI_MIRNA_TRANSCRIPTION_BY_RNA_	-0.84	0.19	0	0	0	not significant
6963	REACTOME_METHIONINE_SALVAGE_PATHWAY	-0.84	0.19	0	0	0	not significant
6964	REACTOME_SYNTHESIS_OF_GDP_MANNOSE	-0.84	0.19	0	0	0	not significant

6965	REACTOME_SYNTHESIS_OF_PIPS_AT_THE_ER_MEMBRANE	-0.84	0.19	0	0	0	not significant
6966	BIOCARTA_PKC_PATHWAY	-0.84	0.18	0	0	0	not significant
6967	BRUNEAU_SEPTATION_VENTRICULAR	-0.84	0.18	0	0	0	not significant
6968	GO_EXTRACELLULAR_MATRIX_ASSEMBLY	-0.84	0.18	0	0	0	not significant
6969	GO_MAINTENANCE_OF_PROTEIN_LOCALIZATION_IN_ENDOPLASMIC_RET	-0.84	0.18	0	0	0	not significant
6970	GO_MICROTUBULE_SEVERING	-0.84	0.18	0	0	0	not significant
6971	GO_PHOSPHATIDYLGLYCEROL_BIOSYNTHETIC_PROCESS	-0.84	0.18	0	0	0	not significant
6972	GO_POSITIVE_REGULATION_OF_AMINO_ACID_TRANSPORT	-0.84	0.18	0	0	0	not significant
6973	GO_POSITIVE_REGULATION_OF_INTRACELLULAR_STEROID_HORMONE_	-0.84	0.18	0	0	0	not significant
6974	PID_SYNDECAN_4_PATHWAY	-0.84	0.18	0	0	0	not significant
6975	REACTOME_SUMO_IS_CONJUGATED_TO_E1_UBA2:SAE1	-0.84	0.18	0	0	0	not significant
6976	GO_ACTIVATION_OF_MAPKKK_ACTIVITY	-0.84	0.17	0	0	0	not significant
6977	GO_CILIARY_BASE	-0.84	0.17	0	0	0	not significant
6978	GO_FAT_SOLUBLE_VITAMIN_BIOSYNTHETIC_PROCESS	-0.84	0.17	0	0	0	not significant
6979	GO_MIDBODY_ABSCISSION	-0.84	0.17	0	0	0	not significant
6980	GO_N_TERMINAL_PROTEIN_AMINO_ACID_ACETYLATION	-0.84	0.17	0	0	0	not significant
6981	GO_TRANSLOCON_COMPLEX	-0.84	0.17	0	0	0	not significant
6982	HOFMANN_MYELODYSPLASTIC_SYNDROM_RISK_DN	-0.84	0.17	0	0	0	not significant
6983	KIM_MYCL1_AMPLIFICATION_TARGETS_DN	-0.84	0.17	0	0	0	not significant
6984	PID_NETRIN_PATHWAY	-0.84	0.17	0	0	0	not significant
6985	WIKMAN_ASBESTOS_LUNG_CANCER_DN	-0.84	0.17	0	0	0	not significant
6986	GO_3_UTR_MEDIATED_MRNA_STABILIZATION	-0.84	0.16	0	0	0	not significant
6987	GO_FOREBRAIN_GENERATION_OF_NEURONS	-0.84	0.16	0	0	0	not significant
6988	GO_PHOSPHATIDYLINOSITOL_3_5_BISPHOSPHATE_BINDING	-0.84	0.16	0	0	0	not significant
6989	GO_SERINE_TYPE_ENDOPEPTIDASE_INHIBITOR_ACTIVITY	-0.84	0.16	0	0	0	not significant
6990	REACTOME_SIGNALING_BY_EGFRVIII_IN_CANCER	-0.84	0.16	0	0	0	not significant
6991	GO_ATP_HYDROLYSIS_COUPLED_TRANSMEMBRANE_TRANSPORT	-0.84	0.15	0	0	0	not significant
6992	KEGG_MISMATCH_REPAIR	-0.84	0.15	0	0	0	not significant
6993	OZEN_MIR125B1_TARGETS	-0.84	0.15	0	0	0	not significant
6994	REACTOME_BBSOME_MEDIATED_CARGO_TARGETING_TO_CILIUM	-0.84	0.15	0	0	0	not significant
6995	CHR15Q24	-0.84	0.14	0	0	0	not significant
6996	GO_CYCLIN_BINDING	-0.84	0.14	0	0	0	not significant
6997	GO_NEGATIVE_REGULATION_OF_CELL_MATRIX_ADHESION	-0.84	0.14	0	0	0	not significant
6998	LIANG_HEMATOPOIESIS_STEM_CELL_NUMBER_LARGE_VS_TINY_DN	-0.84	0.14	0	0	0	not significant
6999	WIEDERSCHAIN_TARGETS_OF_BMI1_AND_PCGF2	-0.84	0.14	0	0	0	not significant
7000	BOYLAN_MULTIPLE_MYELOMA_C_CLUSTER_UP	-0.84	0.13	0	0	0	not significant
7001	GO_INTERCELLULAR_BRIDGE	-0.84	0.13	0	0	0	not significant
7002	NIKOLSKY_BREAST_CANCER_20Q11_AMPLICON	-0.84	0.13	0	0	0	not significant
7003	WOO_LIVER_CANCER_RECURRENCE_DN	-0.84	0.13	0	0	0	not significant
7004	DUTTA_APOPTOSIS_VIA_NFKB	-0.84	0.12	0	0	0	not significant
7005	GO_MANGANESE_ION_BINDING	-0.84	0.12	0	0	0	not significant
7006	GO_REGULATION_OF_BMP_SIGNALING_PATHWAY	-0.84	0.12	0	0	0	not significant
7007	NABA_ECM_REGULATORS	-0.84	0.12	0	0	0	not significant
7008	GO_GLAND_MORPHOGENESIS	-0.84	0.11	0	0	0	not significant
7009	GO_METENCEPHALON_DEVELOPMENT	-0.84	0.11	0	0	0	not significant
7010	LE_EGR2_TARGETS_DN	-0.84	0.11	0	0	0	not significant
7011	SIG_PIP3_SIGNALING_IN_CARDIAC_MYOCTES	-0.84	0.11	0	0	0	not significant
7012	GO_HISTONE_DEACETYLASE_COMPLEX	-0.84	0.10	0	0	0	not significant
7013	GO_METALLOPEPTIDASE_ACTIVITY	-0.84	0.10	0	0	0	not significant
7014	GO_OXIDOREDUCTASE_ACTIVITY_ACTING_ON_PAIRED_DONORS_WITH_	-0.84	0.10	0	0	0	not significant
7015	GO_REGULATION_OF_DNA_BINDING	-0.84	0.08	0	0	0	not significant
7016	REACTOME_G_ALPHA_Q_SIGNALLING_EVENTS	-0.84	0.08	0	0	0	not significant
7017	GO_CHROMATIN_ASSEMBLY	-0.84	0.07	0	0	0	not significant
7018	WAKABAYASHI_ADIPOGENESIS_PPARG_RXRA_BOUND_WITH_H4K20ME1_	-0.84	0.07	0	0	0	not significant
7019	REACTOME_HOMOLOGY_DIRECTED_REPAIR	-0.84	0.06	0	0	0	not significant
7020	GO_INTRACELLULAR_RECEPTOR_SIGNALING_PATHWAY	-0.84	0.03	0	0	0	not significant
7021	GO_DNA_REPAIR	-0.84	0.01	0	0	0	not significant
7022	WAKABAYASHI_ADIPOGENESIS_PPARG_BOUND_8D	-0.84	0.01	0	0	0	not significant
7023	GO_PROTEIN_MODIFICATION_BY_SMALL_PROTEIN_CONJUGATION_OR_F	-0.84	0.00	0	0	0	not significant
7024	GO_CELLULAR_RESPONSE_TO_NITROSATIVE_STRESS	-0.83	0.19	0	0	0	not significant
7025	GO_GATOR1_COMPLEX	-0.83	0.19	0	0	0	not significant
7026	GO_CELL_TRAILING_EDGE	-0.83	0.18	0	0	0	not significant
7027	GO_CELLULAR_RESPONSE_TO_ACIDIC_PH	-0.83	0.18	0	0	0	not significant
7028	GO_REGULATION_OF_AMINO_ACID_TRANSMEMBRANE_TRANSPORT	-0.83	0.18	0	0	0	not significant
7029	GO_TRAIL_ACTIVATED_APOPTOTIC_SIGNALING_PATHWAY	-0.83	0.18	0	0	0	not significant
7030	PEDERSEN_METASTASIS_BY_ERBB2_ISOFORM_5	-0.83	0.18	0	0	0	not significant
7031	REACTOME_TP53_REGULATES_TRANSCRIPTION_OF_DEATH_RECEPTOI	-0.83	0.18	0	0	0	not significant
7032	STEGMEIER_PRE-MITOTIC_CELL_CYCLE_REGULATORS	-0.83	0.18	0	0	0	not significant
7033	BIOCARTA_MAL_PATHWAY	-0.83	0.17	0	0	0	not significant
7034	DAZARD_UV_RESPONSE_CLUSTER_G5	-0.83	0.17	0	0	0	not significant
7035	GO_EGO_COMPLEX	-0.83	0.17	0	0	0	not significant
7036	GO_NEGATIVE_REGULATION_OF_EPIDERMIS_DEVELOPMENT	-0.83	0.17	0	0	0	not significant
7037	GO_PEPTIDYL_LYSINE_MONOMETHYLATION	-0.83	0.17	0	0	0	not significant
7038	GO_PHOSPHATIDYLINOSITOL_3_KINASE_COMPLEX_CLASS_I	-0.83	0.17	0	0	0	not significant
7039	GO_POSITIVE_REGULATION_OF_LIPOPOLYSACCHARIDE_MEDIATED_SIG	-0.83	0.17	0	0	0	not significant
7040	GO_POSITIVE_REGULATION_OF_MEIOTIC_CELL_CYCLE	-0.83	0.17	0	0	0	not significant
7041	GO_POSITIVE_REGULATION_OF_STEM_CELL_DIFFERENTIATION	-0.83	0.17	0	0	0	not significant
7042	GO_REGULATION_OF_DEACETYLASE_ACTIVITY	-0.83	0.17	0	0	0	not significant
7043	GO_REGULATION_OF_PROTEIN_KINASE_C_SIGNALING	-0.83	0.17	0	0	0	not significant
7044	GO_SEQUENCE_SPECIFIC_SINGLE_STRANDED_DNA_BINDING	-0.83	0.17	0	0	0	not significant
7045	PAPASPYRIDONOS_UNSTABLE_ATEROSCLEROTIC_PLAQUE_DN	-0.83	0.17	0	0	0	not significant
7046	REACTOME_ENDOSOMAL_VACUOLAR_PATHWAY	-0.83	0.17	0	0	0	not significant
7047	REACTOME_HYALURONAN_UPTAKE_AND_DEGRADATION	-0.83	0.17	0	0	0	not significant
7048	REACTOME_TP53_REGULATES_TRANSCRIPTION_OF_GENES_INVOLVED	-0.83	0.17	0	0	0	not significant
7049	CHR12Q15	-0.83	0.16	0	0	0	not significant
7050	GO_ENDOPLASMIC_RETICULUM_EXIT_SITE	-0.83	0.16	0	0	0	not significant
7051	GO_PTERIDINE_CONTAINING_COMPOUND_METABOLIC_PROCESS	-0.83	0.16	0	0	0	not significant
7052	GO_RAC_GUANYL_NUCLEOTIDE_EXCHANGE_FACTOR_ACTIVITY	-0.83	0.16	0	0	0	not significant
7053	GO_REGULATION_OF_CHOLESTEROL_EFFLUX	-0.83	0.16	0	0	0	not significant
7054	GO_RESOLUTION_OF_MEIOTIC_RECOMBINATION_INTERMEDIATES	-0.83	0.16	0	0	0	not significant
7055	GO_RRNA_CATABOLIC_PROCESS	-0.83	0.16	0	0	0	not significant
7056	GO_VERY_LOW_DENSITY_LIPOPROTEIN_PARTICLE_ASSEMBLY	-0.83	0.16	0	0	0	not significant
7057	NAGY_TFTC_COMPONENTS_HUMAN	-0.83	0.16	0	0	0	not significant
7058	REACTOME_CYTOCHROME_C_MEDIATED_APOPTOTIC_RESPONSE	-0.83	0.16	0	0	0	not significant
7059	REACTOME_PROLONGED_ERK_ACTIVATION_EVENTS	-0.83	0.16	0	0	0	not significant
7060	REACTOME_SEROTONIN_NEUROTRANSMITTER_RELEASE_CYCLE	-0.83	0.16	0	0	0	not significant
7061	FOURNIER_ACINAR_DEVELOPMENT_EARLY_UP	-0.83	0.15	0	0	0	not significant
7062	GO_POSITIVE_REGULATION_OF_PROTEIN_AUTOPHOSPHORYLATION	-0.83	0.15	0	0	0	not significant
7063	GO_POSITIVE_REGULATION_OF_SMOOTHENED_SIGNALING_PATHWAY	-0.83	0.15	0	0	0	not significant
7064	GO_PURINE_NUCLEOSIDE_MONOPHOSPHATE_METABOLIC_PROCESS	-0.83	0.15	0	0	0	not significant
7065	GO_REGULATION_OF_EXTRACELLULAR_MATRIX_DISASSEMBLY	-0.83	0.15	0	0	0	not significant
7066	GO_REGULATION_OF_MYOBLAST_DIFFERENTIATION	-0.83	0.15	0	0	0	not significant
7067	GO_REGULATION_OF_NEURONAL_SYNAPTIC_PLASTICITY	-0.83	0.15	0	0	0	not significant
7068	PLASARI_TGFB1_SIGNALING_VIA_NFIC_10HR_DN	-0.83	0.15	0	0	0	not significant
7069	SIMBULAN_UV_RESPONSE_NORMAL_DN	-0.83	0.15	0	0	0	not significant
7070	BIOCARTA_HSP27_PATHWAY	-0.83	0.14	0	0	0	not significant
7071	GO_HISTONE_H2A_ACETYLATION	-0.83	0.14	0	0	0	not significant
7072	GO_PRODUCTION_OF_SMALL_RNA_INVOLVED_IN_GENE_SILENCING_BY_	-0.83	0.14	0	0	0	not significant
7073	CHR4Q31	-0.83	0.13	0	0	0	not significant

7074	GO_MODULATION_OF_TRANSCRIPTION_IN_OTHER_ORGANISM_INVOLVE	-0.83	0.13	0	0	0	not significant
7075	GO_NEGATIVE_REGULATION_OF_RESPONSE_TO_CYTOKINE_STIMULUS	-0.83	0.13	0	0	0	not significant
7076	GO_OVULATION_CYCLE_PROCESS	-0.83	0.13	0	0	0	not significant
7077	GO_POSITIVE_REGULATION_OF_ANION_TRANSPORT	-0.83	0.13	0	0	0	not significant
7078	GO_REGULATION_OF_HISTONE_H4_ACETYLATION	-0.83	0.13	0	0	0	not significant
7079	PID_FOXO_PATHWAY	-0.83	0.13	0	0	0	not significant
7080	GO_PHOSPHATIDYLINOSITOL_4_5_BISPHOSPHATE_BINDING	-0.83	0.12	0	0	0	not significant
7081	GO_POSITIVE_REGULATION_OF_MEMBRANE_PERMEABILITY	-0.83	0.12	0	0	0	not significant
7082	GO_TETHERING_COMPLEX	-0.83	0.12	0	0	0	not significant
7083	PID_ERBB2_ERBB3_PATHWAY	-0.83	0.12	0	0	0	not significant
7084	GO_INTRACELLULAR_ESTROGEN_RECEPTOR_SIGNALING_PATHWAY	-0.83	0.11	0	0	0	not significant
7085	WENG_POR_TARGETS_LIVER_UP	-0.83	0.11	0	0	0	not significant
7086	GO_CILIARY_TRANSITION_ZONE	-0.83	0.10	0	0	0	not significant
7087	GO_EXONUCLEASE_ACTIVITY_ACTIVE_WITH_EITHER_RIBO_OR_DEOXYR	-0.83	0.10	0	0	0	not significant
7088	HALLMARK_ANDROGEN_RESPONSE	-0.83	0.10	0	0	0	not significant
7089	HOEBEKE_LYMPHOID_STEM_CELL_UP	-0.83	0.10	0	0	0	not significant
7090	DACOSTA_UV_RESPONSE_VIA_ERCC3_XPCS_DN	-0.83	0.09	0	0	0	not significant
7091	GO_MACROMOLECULE_DEACYLATION	-0.83	0.09	0	0	0	not significant
7092	GO_POSITIVE_REGULATION_OF_CELL_CYCLE_PHASE_TRANSITION	-0.83	0.09	0	0	0	not significant
7093	IKEDA_MIR30_TARGETS_UP	-0.83	0.08	0	0	0	not significant
7094	REACTOME_SIGNALING_BY_TGF_BETA_RECEPTOR_COMPLEX	-0.83	0.08	0	0	0	not significant
7095	GO_TRANSITION_METAL_ION_TRANSPORT	-0.83	0.07	0	0	0	not significant
7096	GO_UBIQUITIN_LIKE_PROTEIN_SPECIFIC_PROTEASE_ACTIVITY	-0.83	0.07	0	0	0	not significant
7097	GO_CHROMATIN_ASSEMBLY_OR_DISASSEMBLY	-0.83	0.06	0	0	0	not significant
7098	GO_REGULATION_OF_UBIQUITIN_DEPENDENT_PROTEIN_CATABOLIC_PR	-0.83	0.06	0	0	0	not significant
7099	BENPORATH_OCT4_TARGETS	-0.83	0.04	0	0	0	not significant
7100	CHR12Q24	-0.83	0.03	0	0	0	not significant
7101	GO_ALDEHYDE_CATABOLIC_PROCESS	-0.82	0.18	0	0	0	not significant
7102	GO_PARASPECKLES	-0.82	0.18	0	0	0	not significant
7103	GO_PROGRAMMED_CELL_DEATH_IN_RESPONSE_TO_REACTIVE_OXYGE	-0.82	0.18	0	0	0	not significant
7104	CHR16Q11	-0.82	0.17	0	0	0	not significant
7105	GO_MEMBRANOUS_SEPTUM_MORPHOGENESIS	-0.82	0.17	0	0	0	not significant
7106	GO_NEGATIVE_REGULATION_OF_VASCULAR_ENDOTHELIAL_GROWTH_F	-0.82	0.17	0	0	0	not significant
7107	GO_SPERM_CAPACITATION	-0.82	0.17	0	0	0	not significant
7108	HINATA_NFKB_TARGETS_KERATINOCYTE_DN	-0.82	0.17	0	0	0	not significant
7109	GO_ACTIVATION_OF_CYSTEINE_TYPE_ENDOPEPTIDASE_ACTIVITY	-0.82	0.16	0	0	0	not significant
7110	GO_HYALURONAN_CATABOLIC_PROCESS	-0.82	0.16	0	0	0	not significant
7111	GO_INNER_CELL_MASS_CELL_PROLIFERATION	-0.82	0.16	0	0	0	not significant
7112	GO_MEMBRANE_REPOLARIZATION_DURING_ACTION_POTENTIAL	-0.82	0.16	0	0	0	not significant
7113	GO_MITOTIC_CELL_CYCLE_ARREST	-0.82	0.16	0	0	0	not significant
7114	GO_NEGATIVE_REGULATION_OF_INTRINSIC_APOPTOTIC_SIGNALING_PA	-0.82	0.16	0	0	0	not significant
7115	GO_PARTURITION	-0.82	0.16	0	0	0	not significant
7116	GO_PHOSPHORYLASE_ACTIVITY	-0.82	0.16	0	0	0	not significant
7117	GO_REGULATION_OF_AMINO_ACID_IMPORT_ACROSS_PLASMA_MEMBRA	-0.82	0.16	0	0	0	not significant
7118	GO_REGULATION_OF_RETINOIC_ACID_RECEPTOR_SIGNALING_PATHWA	-0.82	0.16	0	0	0	not significant
7119	GO_RESPONSE_TO_FOLLICLE_STIMULATING_HORMONE	-0.82	0.16	0	0	0	not significant
7120	GO_TRANSCRIPTION_INITIATION_FROM_RNA_POLYMERASE_III_PROMOT	-0.82	0.16	0	0	0	not significant
7121	GO_TYPE_I_INTERFERON_SECRETION	-0.82	0.16	0	0	0	not significant
7122	GO_WALKING_BEHAVIOR	-0.82	0.16	0	0	0	not significant
7123	LI_CYTIDINE_ANALOGS_CYCTOTOXICITY	-0.82	0.16	0	0	0	not significant
7124	LUND_SILENCED_BY_METHYLATION	-0.82	0.16	0	0	0	not significant
7125	REACTOME_BETA_OXIDATION_OF_DECANOYL_COA_TO_OCTANOYL_CC	-0.82	0.16	0	0	0	not significant
7126	SA_FAS_SIGNALING	-0.82	0.16	0	0	0	not significant
7127	BIOCARTA_SPPA_PATHWAY	-0.82	0.15	0	0	0	not significant
7128	GO_ANDROGEN_METABOLIC_PROCESS	-0.82	0.15	0	0	0	not significant
7129	GO_APOLIPOPROTEIN_BINDING	-0.82	0.15	0	0	0	not significant
7130	GO_CELLULAR_RESPONSE_TO_FOLLICLE_STIMULATING_HORMONE_STI	-0.82	0.15	0	0	0	not significant
7131	GO_CELLULAR_RESPONSE_TO_LIPOPROTEIN_PARTICLE_STIMULUS	-0.82	0.15	0	0	0	not significant
7132	GO_LATERAL_VENTRICLE_DEVELOPMENT	-0.82	0.15	0	0	0	not significant
7133	GO_PHOSPHATIDYLINOSITOL_3_5_BISPHOSPHATE_3_PHOSPHATASE_AC	-0.82	0.15	0	0	0	not significant
7134	GO_POSTSYNAPTIC_ENDOCYTIC_ZONE	-0.82	0.15	0	0	0	not significant
7135	REACTOME_SYNTHESIS_OF_PA	-0.82	0.15	0	0	0	not significant
7136	RICKMAN_HEAD_AND_NECK_CANCER_B	-0.82	0.15	0	0	0	not significant
7137	BIOCARTA_HSWI_SNF_PATHWAY	-0.82	0.14	0	0	0	not significant
7138	CHARAFE_BREAST_CANCER_BASAL_VS_MESENCHYMAL_DN	-0.82	0.14	0	0	0	not significant
7139	GO_BILE_ACID_METABOLIC_PROCESS	-0.82	0.14	0	0	0	not significant
7140	GO_CORTICOSTEROID_RECEPTOR_SIGNALING_PATHWAY	-0.82	0.14	0	0	0	not significant
7141	GO_ERROR_FREE_TRANSLESION_SYNTHESIS	-0.82	0.14	0	0	0	not significant
7142	GO_HEXOSE_CATABOLIC_PROCESS	-0.82	0.14	0	0	0	not significant
7143	GO_ISOCITRATE_METABOLIC_PROCESS	-0.82	0.14	0	0	0	not significant
7144	GO_PURINE_NUCLEOTIDE_TRANSPORT	-0.82	0.14	0	0	0	not significant
7145	GO_REGULATION_OF_PROTEIN_TARGETING_TO_MEMBRANE	-0.82	0.14	0	0	0	not significant
7146	HOLLEMAN_PREDNISOLONE_RESISTANCE_B_ALL_UP	-0.82	0.14	0	0	0	not significant
7147	MURAKAMI_UV_RESPONSE_6HR_UP	-0.82	0.14	0	0	0	not significant
7148	PARK_HSC_MARKERS	-0.82	0.14	0	0	0	not significant
7149	REACTOME_GAMMA_CARBOXYLATION_HYPUSINE_FORMATION_AND_ARY	-0.82	0.14	0	0	0	not significant
7150	CHR12P11	-0.82	0.13	0	0	0	not significant
7151	GO_AXONAL_TRANSPORT	-0.82	0.13	0	0	0	not significant
7152	GO_REGULATION_OF_MYELOID_LEUKOCYTE_MEDIATED_IMMUNITY	-0.82	0.13	0	0	0	not significant
7153	HOLLERN_MICROACINAR_BREAST_TUMOR_UP	-0.82	0.13	0	0	0	not significant
7154	PID_AVB3_OPN_PATHWAY	-0.82	0.13	0	0	0	not significant
7155	GO_INTRA_GOLGI_VESICLE_MEDIATED_TRANSPORT	-0.82	0.12	0	0	0	not significant
7156	GO_REGULATION_OF_LIPID_KINASE_ACTIVITY	-0.82	0.12	0	0	0	not significant
7157	GO_RNA_PHOSPHODIESTER_BOND_HYDROLYSIS_EXONUCLEOLYTIC	-0.82	0.12	0	0	0	not significant
7158	YAO_TEMPORAL_RESPONSE_TO_PROGESTERONE_CLUSTER_9	-0.82	0.11	0	0	0	not significant
7159	CHR16P12	-0.82	0.10	0	0	0	not significant
7160	GO_H4_HISTONE_ACETYLTRANSFERASE_COMPLEX	-0.82	0.10	0	0	0	not significant
7161	GO_L_ALPHA_AMINO_ACID_TRANSMEMBRANE_TRANSPORT	-0.82	0.10	0	0	0	not significant
7162	GO_LIPID_STORAGE	-0.82	0.10	0	0	0	not significant
7163	GO_NUCLEAR_REPLICATION_FORK	-0.82	0.10	0	0	0	not significant
7164	GO_PHOSPHATASE_REGULATOR_ACTIVITY	-0.82	0.10	0	0	0	not significant
7165	SWEET_KRAS_TARGETS_UP	-0.82	0.10	0	0	0	not significant
7166	CHRXQ28	-0.82	0.09	0	0	0	not significant
7167	GO_NEGATIVE_REGULATION_OF_EXTRINSIC_APOPTOTIC_SIGNALING_PA	-0.82	0.09	0	0	0	not significant
7168	GO_NEGATIVE_REGULATION_OF_NF_KAPPAB_TRANSCRIPTION_FACTOR	-0.82	0.09	0	0	0	not significant
7169	RELA_DN.V1_UP	-0.82	0.08	0	0	0	not significant
7170	BROWNE_HCMV_INFECTION_12HR_DN	-0.82	0.07	0	0	0	not significant
7171	GO_REGULATION_OF_MUSCLE_ORGAN_DEVELOPMENT	-0.82	0.07	0	0	0	not significant
7172	PDGF_ERK_DN.V1_UP	-0.82	0.06	0	0	0	not significant
7173	RAY_TUMORIGENESIS_BY_ERBB2_CDC25A_DN	-0.82	0.03	0	0	0	not significant
7174	DAVICIONI_MOLECULAR_ARMS_VS_ERMS_UP	-0.82	0.02	0	0	0	not significant
7175	GO_TRANSFERASE_COMPLEX	-0.82	0.00	0	0	0	not significant
7176	GO_PROTEIN_PHOSPHATASE_2B_BINDING	-0.81	0.18	0	0	0	not significant
7177	GO_CELLULAR_RESPONSE_TO_ESTROGEN_STIMULUS	-0.81	0.17	0	0	0	not significant
7178	REACTOME_GALACTOSE_CATABOLISM	-0.81	0.17	0	0	0	not significant
7179	BOYAULT_LIVER_CANCER_SUBCLASS_G56_DN	-0.81	0.16	0	0	0	not significant
7180	GO_ACTIN_POLYMERIZATION_DEPENDENT_CELL_MOTILITY	-0.81	0.16	0	0	0	not significant
7181	GO_CONNECTIVE_TISSUE_REPLACEMENT	-0.81	0.16	0	0	0	not significant
7182	GO_FILOPODIUM_TIP	-0.81	0.16	0	0	0	not significant

7183	GO_VESICLE_FUSION_WITH_GOLGI_APPARATUS	-0.81	0.16	0	0	0	not significant
7184	YEMELYANOV_GR_TARGETS_DN	-0.81	0.16	0	0	0	not significant
7185	BONOME_OVARIAN_CANCER_POOR_SURVIVAL_DN	-0.81	0.15	0	0	0	not significant
7186	GHANDHI_BYSTANDER_IRRADIATION_DN	-0.81	0.15	0	0	0	not significant
7187	GO_CARBON_SULFUR_LYASE_ACTIVITY	-0.81	0.15	0	0	0	not significant
7188	GO_CELLULAR_RESPONSE_TO_NITRIC_OXIDE	-0.81	0.15	0	0	0	not significant
7189	GO_INTESTINAL_EPITHELIAL_CELL_DIFFERENTIATION	-0.81	0.15	0	0	0	not significant
7190	GO_NADP_METABOLIC_PROCESS	-0.81	0.15	0	0	0	not significant
7191	GO_PIGMENT_GRANULE_MATURATION	-0.81	0.15	0	0	0	not significant
7192	GO_PURINE_NUCLEOTIDE_TRANSMEMBRANE_TRANSPORTER_ACTIVITY	-0.81	0.15	0	0	0	not significant
7193	REACTOME_GLYCOGEN_BREAKDOWN_GLYCOGENOLYSIS	-0.81	0.15	0	0	0	not significant
7194	REACTOME_MISCELLANEOUS_SUBSTRATES	-0.81	0.15	0	0	0	not significant
7195	VERNOCHET_ADIPOGENESIS	-0.81	0.15	0	0	0	not significant
7196	BIOCARTA_EPHA4_PATHWAY	-0.81	0.14	0	0	0	not significant
7197	CHANG_POU5F1_TARGETS_UP	-0.81	0.14	0	0	0	not significant
7198	CHR5Q23	-0.81	0.14	0	0	0	not significant
7199	GO_GUANYL_NUCLEOTIDE_EXCHANGE_FACTOR_COMPLEX	-0.81	0.14	0	0	0	not significant
7200	PID_SMAD2_3PATHWAY	-0.81	0.14	0	0	0	not significant
7201	REACTOME_NEF_AND_SIGNAL_TRANSDUCTION	-0.81	0.14	0	0	0	not significant
7202	XU_AKT1_TARGETS_48HR	-0.81	0.14	0	0	0	not significant
7203	GO_AU_RICH_ELEMENT_BINDING	-0.81	0.13	0	0	0	not significant
7204	GO_CARBOXY_TERMINAL_DOMAIN_PROTEIN_KINASE_COMPLEX	-0.81	0.13	0	0	0	not significant
7205	GO_POSITIVE_REGULATION_OF_LAMELLIPODIUM_ASSEMBLY	-0.81	0.13	0	0	0	not significant
7206	GO_POSITIVE_REGULATION_OF_REGULATED_SECRETORY_PATHWAY	-0.81	0.13	0	0	0	not significant
7207	GUENTHER_GROWTH_SPHERICAL_VS_ADHERENT_DN	-0.81	0.13	0	0	0	not significant
7208	REACTOME_TRANSLESION_SYNTHESIS_BY_POLH	-0.81	0.13	0	0	0	not significant
7209	GO_EXIT_FROM_MITOSIS	-0.81	0.12	0	0	0	not significant
7210	REACTOME_LYSOSOME_VESICLE_BIOGENESIS	-0.81	0.12	0	0	0	not significant
7211	BREDEMEYER_RAG_SIGNALING_NOT_VIA_ATM_DN	-0.81	0.10	0	0	0	not significant
7212	GO_BONE_REMODELING	-0.81	0.10	0	0	0	not significant
7213	GO_CELLULAR_PIGMENTATION	-0.81	0.10	0	0	0	not significant
7214	GO_NEGATIVE_REGULATION_OF_DEVELOPMENTAL_GROWTH	-0.81	0.10	0	0	0	not significant
7215	GO_OXIDOREDUCTASE_ACTIVITY_ACTING_ON_THE_CH_CH_GROUP_OF_	-0.81	0.10	0	0	0	not significant
7216	GO_PYRIMIDINE_CONTAINING_COMPOUND_BIOSYNTHETIC_PROCESS	-0.81	0.10	0	0	0	not significant
7217	MAHAJAN_RESPONSE_TO_IL1A_UP	-0.81	0.10	0	0	0	not significant
7218	REACTOME_PKMTS_METHYLATE_HISTONE_LYSINES	-0.81	0.10	0	0	0	not significant
7219	KEGG_GLYCEROPHOSPHOLIPID_METABOLISM	-0.81	0.09	0	0	0	not significant
7220	REACTOME_TRANSPORT_OF_BILE_SALTS_AND_ORGANIC_ACIDS_META	-0.81	0.09	0	0	0	not significant
7221	GO_DEVELOPMENT_OF_PRIMARY_FEMALE_SEXUAL_CHARACTERISTICS	-0.81	0.08	0	0	0	not significant
7222	KEGG_AXON_GUIDANCE	-0.81	0.07	0	0	0	not significant
7223	REACTOME_ION_CHANNEL_TRANSPORT	-0.81	0.05	0	0	0	not significant
7224	GO_CALMODULIN_BINDING	-0.81	0.04	0	0	0	not significant
7225	GO_RESPONSE_TO_ENDOPLASMIC_RETICULUM_STRESS	-0.81	0.02	0	0	0	not significant
7226	GO_STRESS_ACTIVATED_PROTEIN_KINASE_SIGNALING_CASCADE	-0.81	0.02	0	0	0	not significant
7227	GO_NUCLEAR_MEMBRANE	-0.81	0.01	0	0	0	not significant
7228	GO_PHOSPHOLIPID_BIOSYNTHETIC_PROCESS	-0.81	0.01	0	0	0	not significant
7229	GO_ENDOMEMBRANE_SYSTEM_ORGANIZATION	-0.81	0.00	0	0	0	not significant
7230	GO_PEPTIDYL_AMINO_ACID_MODIFICATION	-0.81	0.00	0	0	0	not significant
7231	GO_GDP_METABOLIC_PROCESS	-0.80	0.17	0	0	0	not significant
7232	GO_POSITIVE_REGULATION_OF_DNA_CATABOLIC_PROCESS	-0.80	0.17	0	0	0	not significant
7233	GO_SUPERCOILED_DNA_BINDING	-0.80	0.17	0	0	0	not significant
7234	GO_HISTONE_THREONINE_PHOSPHORYLATION	-0.80	0.16	0	0	0	not significant
7235	GO_N_ACETYLGLUCOSAMINE_METABOLIC_PROCESS	-0.80	0.16	0	0	0	not significant
7236	GO_NEGATIVE_REGULATION_OF_ASTROCYTE_DIFFERENTIATION	-0.80	0.16	0	0	0	not significant
7237	GO_CELL_AGGREGATION	-0.80	0.15	0	0	0	not significant
7238	GO_CELLULAR_RESPONSE_TO_LEPTIN_STIMULUS	-0.80	0.15	0	0	0	not significant
7239	GO_ESTABLISHMENT_OF_VIRAL_LATENCY	-0.80	0.15	0	0	0	not significant
7240	GO_LONG_CHAIN_FATTY_ACID_BIOSYNTHETIC_PROCESS	-0.80	0.15	0	0	0	not significant
7241	GO_MORPHOGENESIS_OF_AN_ENDOTHELIUM	-0.80	0.15	0	0	0	not significant
7242	GO_PIGMENT_ACCUMULATION	-0.80	0.15	0	0	0	not significant
7243	GO_REGULATION_OF_LYSOSOMAL_LUMEN_PH	-0.80	0.15	0	0	0	not significant
7244	GO_TRAPP_COMPLEX	-0.80	0.15	0	0	0	not significant
7245	MUNSHI_MULTIPLE_MYELOMA_DN	-0.80	0.15	0	0	0	not significant
7246	REACTOME_E2F_ENABLED_INHIBITION_OF_PRE_REPLICATION_COMPLEX	-0.80	0.15	0	0	0	not significant
7247	REACTOME_SYNTHESIS_OF_DIPHTHAMIDE_EEF2	-0.80	0.15	0	0	0	not significant
7248	GO_CELLULAR_RESPONSE_TO_INCREASED_OXYGEN_LEVELS	-0.80	0.14	0	0	0	not significant
7249	GO_HISTONE_THREONINE_KINASE_ACTIVITY	-0.80	0.14	0	0	0	not significant
7250	GO_METALLOAMINOPEPTIDASE_ACTIVITY	-0.80	0.14	0	0	0	not significant
7251	GO_PYRUVATE_DEHYDROGENASE_COMPLEX	-0.80	0.14	0	0	0	not significant
7252	MIKKELSEN_ES_HCP_WITH_H3_UNMETHYLATED	-0.80	0.14	0	0	0	not significant
7253	REACTOME_RHO_GTPASES_ACTIVATE_PAKS	-0.80	0.14	0	0	0	not significant
7254	GO_DNA_DAMAGE_RESPONSE_SIGNAL_TRANSDUCTION_RESULTING_IN_	-0.80	0.13	0	0	0	not significant
7255	GO_POSITIVE_REGULATION_OF_VASCULAR_SMOOTH_MUSCLE_CELL_P	-0.80	0.13	0	0	0	not significant
7256	GO_SIN3_TYPE_COMPLEX	-0.80	0.13	0	0	0	not significant
7257	GO_STABILIZATION_OF_MEMBRANE_POTENTIAL	-0.80	0.13	0	0	0	not significant
7258	HOEGERKORP_CD44_TARGETS_DIRECT_DN	-0.80	0.13	0	0	0	not significant
7259	KORKOLA_CORRELATED_WITH_POU5F1	-0.80	0.13	0	0	0	not significant
7260	BIOCARTA_CARM_ER_PATHWAY	-0.80	0.12	0	0	0	not significant
7261	GO_PHOSPHATASE_INHIBITOR_ACTIVITY	-0.80	0.12	0	0	0	not significant
7262	GO_REGULATION_OF_CARDIOCYTE_DIFFERENTIATION	-0.80	0.12	0	0	0	not significant
7263	GO_REGULATION_OF_PROTEIN_SUMOYLATION	-0.80	0.12	0	0	0	not significant
7264	REACTOME_GLYCOGEN_METABOLISM	-0.80	0.12	0	0	0	not significant
7265	REACTOME_METALLOPROTEASE_DUBS	-0.80	0.12	0	0	0	not significant
7266	GO_MACROPHAGE_MIGRATION	-0.80	0.11	0	0	0	not significant
7267	GO_NEGATIVE_REGULATION_OF_NOTCH_SIGNALING_PATHWAY	-0.80	0.11	0	0	0	not significant
7268	GO_REGULATION_OF_CARDIAC_MUSCLE_CELL_DIFFERENTIATION	-0.80	0.11	0	0	0	not significant
7269	GO_CARDIAC_SEPTUM_MORPHOGENESIS	-0.80	0.10	0	0	0	not significant
7270	GO_CRANIAL_SKELETAL_SYSTEM_DEVELOPMENT	-0.80	0.10	0	0	0	not significant
7271	GO_INTRACILIARY_TRANSPORT_INVOLVED_IN_CILIUM_ASSEMBLY	-0.80	0.10	0	0	0	not significant
7272	GOLUB_ALL_VS_AML_UP	-0.80	0.10	0	0	0	not significant
7273	GO_PLASMA_MEMBRANE_RAFT	-0.80	0.09	0	0	0	not significant
7274	GO_SIGNALING_ADAPTOR_ACTIVITY	-0.80	0.09	0	0	0	not significant
7275	PID_S1P_S1P3_PATHWAY	-0.80	0.09	0	0	0	not significant
7276	REACTOME_IRON_UPTAKE_AND_TRANSPORT	-0.80	0.09	0	0	0	not significant
7277	HOSHIDA_LIVER_CANCER_LATE_RECURRENCE_DN	-0.80	0.08	0	0	0	not significant
7278	KYNG_DNA_DAMAGE_BY_4NQO_OR_UV	-0.80	0.08	0	0	0	not significant
7279	GO_INCLUSION_BODY	-0.80	0.07	0	0	0	not significant
7280	GO_NUCLEOSIDE_TRIPHOSPHATE_METABOLIC_PROCESS	-0.80	0.07	0	0	0	not significant
7281	GO_REGULATION_OF_INTRACELLULAR_STEROID_HORMONE_RECEPTOI	-0.80	0.07	0	0	0	not significant
7282	GO_TETRAPYRROLE_BINDING	-0.80	0.07	0	0	0	not significant
7283	CHEN_HOXA5_TARGETS_9HR_UP	-0.80	0.02	0	0	0	not significant
7284	GO_REGULATION_OF_MACROAUTOPHAGY	-0.80	0.02	0	0	0	not significant
7285	PASQUALUCCI_LYMPHOMA_BY_GC_STAGE_UP	-0.80	0.02	0	0	0	not significant
7286	GO_NUCLEAR_SPECK	-0.80	0.01	0	0	0	not significant
7287	GO_TRANSCRIPTION_FACTOR_COMPLEX	-0.80	0.01	0	0	0	not significant
7288	GO_ENDOSOMAL_PART	-0.80	0.00	0	0	0	not significant
7289	GO_G_PROTEIN_COUPLED_RECEPTOR_SIGNALING_PATHWAY_INVOLVE	-0.79	0.16	0	0	0	not significant
7290	GO_NEGATIVE_REGULATION_OF_HORMONE_METABOLIC_PROCESS	-0.79	0.16	0	0	0	not significant
7291	GO_AMINO_ACID_BETAINE_METABOLIC_PROCESS	-0.79	0.15	0	0	0	not significant

7292	GO_ATP_TRANSPORT	-0.79	0.15	0	0	0	not significant
7293	GO_GLYOXYLATE_METABOLIC_PROCESS	-0.79	0.15	0	0	0	not significant
7294	GO_MEIOTIC_CHROMOSOME_SEPARATION	-0.79	0.15	0	0	0	not significant
7295	REACTOME_ADVANCED_GLYCOSYLATION_ENDPRODUCT_RECEPTOR_S	-0.79	0.15	0	0	0	not significant
7296	BIOCARTA_IRES_PATHWAY	-0.79	0.14	0	0	0	not significant
7297	BIOCARTA_SUMO_PATHWAY	-0.79	0.14	0	0	0	not significant
7298	GO_CELLULAR_HYPEROSMOTIC_RESPONSE	-0.79	0.14	0	0	0	not significant
7299	GO_EMBRYONIC_NEUROCRANIUM_MORPHOGENESIS	-0.79	0.14	0	0	0	not significant
7300	GO_ENDOPLASMIC_RETICULUM_PALMITOYLTRANSFERASE_COMPLEX	-0.79	0.14	0	0	0	not significant
7301	GO_MRNA_METHYLTRANSFERASE_ACTIVITY	-0.79	0.14	0	0	0	not significant
7302	GO_MYOSIN_II_BINDING	-0.79	0.14	0	0	0	not significant
7303	GO NEGATIVE REGULATION OF CELL MIGRATION INVOLVED IN SPRO	-0.79	0.14	0	0	0	not significant
7304	GO_NEGATIVE_REGULATION_OF_ENDOTHELIAL_CELL_PROLIFERATION	-0.79	0.14	0	0	0	not significant
7305	GO_NUCLEOSIDE_DIPHOSPHATASE_ACTIVITY	-0.79	0.14	0	0	0	not significant
7306	GO_PHOSPHATIDYLCHOLINE_ACYL_CHAIN_REMODELING	-0.79	0.14	0	0	0	not significant
7307	GO_SYNAPTIC_VESICLE_MATURATION	-0.79	0.14	0	0	0	not significant
7308	NIKOLSKY_BREAST_CANCER_17P11_AMPLICON	-0.79	0.14	0	0	0	not significant
7309	BIOCARTA_IGF1R_PATHWAY	-0.79	0.13	0	0	0	not significant
7310	GO_CARBOHYDRATE_CATION_SYMPORTER_ACTIVITY	-0.79	0.13	0	0	0	not significant
7311	GO_CELLULAR_RESPONSE_TO_COLD	-0.79	0.13	0	0	0	not significant
7312	GO_HYDROLASE_ACTIVITY_ACTING_ON_ETHER_BONDS	-0.79	0.13	0	0	0	not significant
7313	GO_MRNA_3_UTR_AU_RICH_REGION_BINDING	-0.79	0.13	0	0	0	not significant
7314	GO_NEGATIVE_REGULATION_OF_OXIDATIVE_STRESS_INDUCED_NEUROI	-0.79	0.13	0	0	0	not significant
7315	GO_PEPTIDE_HORMONE_RECEPTOR_BINDING	-0.79	0.13	0	0	0	not significant
7316	GO REGULATION OF RNA POLYMERASE II REGULATORY REGION SE(	-0.79	0.13	0	0	0	not significant
7317	GO_REPLICATIVE_CELL_AGING	-0.79	0.13	0	0	0	not significant
7318	KEGG_CIRCADIAN_RHYTHM_MAMMAL	-0.79	0.13	0	0	0	not significant
7319	REACTOME_RUNX3_REGULATES_P14_ARF	-0.79	0.13	0	0	0	not significant
7320	REACTOME_UNWINDING_OF_DNA	-0.79	0.13	0	0	0	not significant
7321	WIKMAN_ASBESTOS_LUNG_CANCER_UP	-0.79	0.13	0	0	0	not significant
7322	BIOCARTA_PTEN_PATHWAY	-0.79	0.12	0	0	0	not significant
7323	GO_VESICLE_FUSION_TO_PLASMA_MEMBRANE	-0.79	0.12	0	0	0	not significant
7324	GO_MYOSIN_COMPLEX	-0.79	0.11	0	0	0	not significant
7325	GO_TRANS_GOLGI_NETWORK_TRANSPORT_VESICLE	-0.79	0.11	0	0	0	not significant
7326	KEGG_HOMOLOGOUS_RECOMBINATION	-0.79	0.11	0	0	0	not significant
7327	BROWNE_HCMV_INFECTION_30MIN_UP	-0.79	0.10	0	0	0	not significant
7328	GO_REGULATION_OF_EXTRINSIC_APOPTOTIC_SIGNALING_PATHWAY_VI/	-0.79	0.10	0	0	0	not significant
7329	GO_THYMUS_DEVELOPMENT	-0.79	0.10	0	0	0	not significant
7330	HASLINGER_B_CLL_WITH_CHROMOSOME_12_TRISOMY	-0.79	0.10	0	0	0	not significant
7331	SILIGAN_BOUND_BY_EWS_FLT1_FUSION	-0.79	0.10	0	0	0	not significant
7332	GO_3_5_EXONUCLEASE_ACTIVITY	-0.79	0.09	0	0	0	not significant
7333	GO_PHOSPHOLIPID_CATABOLIC_PROCESS	-0.79	0.09	0	0	0	not significant
7334	GO_POSITIVE_REGULATION_OF_RECEPTOR_MEDIATED_ENDOCYTOSIS	-0.79	0.09	0	0	0	not significant
7335	GO_RNA_POLYMERASE_CORE_ENZYME_BINDING	-0.79	0.09	0	0	0	not significant
7336	GO_SIGNAL_TRANSDUCTION_IN_ABSENCE_OF_LIGAND	-0.79	0.09	0	0	0	not significant
7337	STONER_ESOPHAGEAL_CARCINOGENESIS_UP	-0.79	0.09	0	0	0	not significant
7338	GO_REGULATION_OF_NEURAL_PRECURSOR_CELL_PROLIFERATION	-0.79	0.08	0	0	0	not significant
7339	GO_XENOBIOTIC_METABOLIC_PROCESS	-0.79	0.08	0	0	0	not significant
7340	GO_DETECTION_OF_ABIOTIC_STIMULUS	-0.79	0.07	0	0	0	not significant
7341	GO_REGULATION_OF_G_PROTEIN_COUPLED_RECEPTOR_SIGNALING_P/	-0.79	0.06	0	0	0	not significant
7342	GO_STEM_CELL_PROLIFERATION	-0.79	0.06	0	0	0	not significant
7343	KEGG_COLORECTAL_CANCER	-0.79	0.06	0	0	0	not significant
7344	GO_NEGATIVE_REGULATION_OF_DEPHOSPHORYLATION	-0.79	0.05	0	0	0	not significant
7345	GO_NON_MOTILE_CILIUM	-0.79	0.05	0	0	0	not significant
7346	GO REGULATION OF MICROTUBULE POLYMERIZATION OR DEPOLYME	-0.79	0.05	0	0	0	not significant
7347	GO_JNK_CASCADE	-0.79	0.02	0	0	0	not significant
7348	KAUFFMANN_DNA_REPAIR_GENES	-0.79	0.01	0	0	0	not significant
7349	GO_THO_COMPLEX	-0.78	0.15	0	0	0	not significant
7350	REACTOME_RSK_ACTIVATION	-0.78	0.15	0	0	0	not significant
7351	GO_ATP_TRANSMEMBRANE_TRANSPORTER_ACTIVITY	-0.78	0.14	0	0	0	not significant
7352	GO_DNA_REPLICATION_FACTOR_C_COMPLEX	-0.78	0.14	0	0	0	not significant
7353	GO NEGATIVE REGULATION OF EXCITATORY POSTSYNAPTIC POTENT	-0.78	0.14	0	0	0	not significant
7354	GO_PRE_MRNA_INTRONIC_BINDING	-0.78	0.14	0	0	0	not significant
7355	REACTOME_RUNX3_REGULATES_CDKN1A_TRANSCRIPTION	-0.78	0.14	0	0	0	not significant
7356	EHRLICH_ICF_SYNDROM_UP	-0.78	0.13	0	0	0	not significant
7357	GO_CELLULAR_RESPONSE_TO_INSULIN_LIKE_GROWTH_FACTOR_STIMU	-0.78	0.13	0	0	0	not significant
7358	GO_CYTOPLASMIC_UBIQUITIN_LIGASE_COMPLEX	-0.78	0.13	0	0	0	not significant
7359	GO_HOST_INTRACELLULAR_ORGANELLE	-0.78	0.13	0	0	0	not significant
7360	GO_NEGATIVE_REGULATION_OF_CHOLESTEROL_BIOSYNTHETIC_PROCI	-0.78	0.13	0	0	0	not significant
7361	GO POSITIVE REGULATION OF TYPE I INTERFERON MEDIATED SIGN/	-0.78	0.13	0	0	0	not significant
7362	GO PROTEIN LOCALIZATION TO ENDOPLASMIC RETICULUM EXIT SITE	-0.78	0.13	0	0	0	not significant
7363	GO_REGULATION_OF_BICELLULAR_TIGHT_JUNCTION_ASSEMBLY	-0.78	0.13	0	0	0	not significant
7364	GO_REGULATION_OF_MALE_GONAD_DEVELOPMENT	-0.78	0.13	0	0	0	not significant
7365	GO_SPERMATID_NUCLEUS_DIFFERENTIATION	-0.78	0.13	0	0	0	not significant
7366	BIOCARTA_RAB_PATHWAY	-0.78	0.12	0	0	0	not significant
7367	FRASOR_RESPONSE_TO_SERM_OR_FULVESTRANT_UP	-0.78	0.12	0	0	0	not significant
7368	GO_ASPARTATE_TRANSMEMBRANE_TRANSPORT	-0.78	0.12	0	0	0	not significant
7369	GO_MAMMARY_GLAND_EPITHELIAL_CELL_PROLIFERATION	-0.78	0.12	0	0	0	not significant
7370	GO_MINUS_END_DIRECTED_ORGANELLE_TRANSPORT_ALONG_MICROTU	-0.78	0.12	0	0	0	not significant
7371	GO_MITOCHONDRIAL_DEPOLARIZATION	-0.78	0.12	0	0	0	not significant
7372	GO POSITIVE REGULATION OF INSULIN SECRETION INVOLVED IN CEL	-0.78	0.12	0	0	0	not significant
7373	GO_UBIQUITIN_PROTEIN_TRANSFERASE_REGULATOR_ACTIVITY	-0.78	0.12	0	0	0	not significant
7374	KEGG_METABOLISM_OF_XENOBIOTICS_BY_CYTOCHROME_P450	-0.78	0.12	0	0	0	not significant
7375	NEBEN_AML_WITH_FLT3_OR_NRAS_DN	-0.78	0.12	0	0	0	not significant
7376	PETRETTO_LEFT_VENTRICLE_MASS_QTL_CIS_DN	-0.78	0.12	0	0	0	not significant
7377	PID_EPHA2_FWD_PATHWAY	-0.78	0.12	0	0	0	not significant
7378	RAY_TARGETS_OF_P210_BCR_ABL_FUSION_DN	-0.78	0.12	0	0	0	not significant
7379	WAGNER_APO2_SENSITIVITY	-0.78	0.12	0	0	0	not significant
7380	BIOCARTA_AKT_PATHWAY	-0.78	0.11	0	0	0	not significant
7381	BOGNI_TREATMENT_RELATED_MYELOID_LEUKEMIA_UP	-0.78	0.11	0	0	0	not significant
7382	GO_RACEMASE_AND_EPIMERASE_ACTIVITY	-0.78	0.11	0	0	0	not significant
7383	GO_VASODILATION	-0.78	0.11	0	0	0	not significant
7384	NIKOLSKY_BREAST_CANCER_6P24_P22_AMPLICON	-0.78	0.11	0	0	0	not significant
7385	PEDERSEN_METASTASIS_BY_ERBB2_ISOFORM_3	-0.78	0.11	0	0	0	not significant
7386	WELCH_GATA1_TARGETS	-0.78	0.11	0	0	0	not significant
7387	GO_NEGATIVE_REGULATION_OF_LIPID_LOCALIZATION	-0.78	0.10	0	0	0	not significant
7388	GO_SMOOTH_ENDOPLASMIC_RETICULUM	-0.78	0.10	0	0	0	not significant
7389	REACTOME ATF4 ACTIVATES GENES IN RESPONSE TO ENDOPLASMI(	-0.78	0.10	0	0	0	not significant
7390	REACTOME_CONSTITUTIVE_SIGNALING_BY_AKT1_E17K_IN_CANCER	-0.78	0.10	0	0	0	not significant
7391	GO_NEGATIVE_REGULATION_OF_ORGANELLE_ASSEMBLY	-0.78	0.09	0	0	0	not significant
7392	GO_RESPONSE_TO_EPIDERMAL_GROWTH_FACTOR	-0.78	0.09	0	0	0	not significant
7393	GO_SCHWANN_CELL_DIFFERENTIATION	-0.78	0.09	0	0	0	not significant
7394	XU_HGF_SIGNALING_NOT_VIA_AKT1_48HR_UP	-0.78	0.09	0	0	0	not significant
7395	GO_ACTIVE_ION_TRANSMEMBRANE_TRANSPORTER_ACTIVITY	-0.78	0.08	0	0	0	not significant
7396	GO_TORC1_SIGNALING	-0.78	0.08	0	0	0	not significant
7397	PID_IL2_PI3K_PATHWAY	-0.78	0.08	0	0	0	not significant
7398	PID_SHP2_PATHWAY	-0.78	0.08	0	0	0	not significant
7399	CHR18Q21	-0.78	0.07	0	0	0	not significant
7400	GO_INTRACILIARY_TRANSPORT	-0.78	0.07	0	0	0	not significant

7401	GO_NEGATIVE_REGULATION_OF_INTRACELLULAR_TRANSPORT	-0.78	0.07	0	0	0	not significant
7402	GO_SNARE_COMPLEX	-0.78	0.07	0	0	0	not significant
7403	NADLER_HYPERGLYCEMIA_AT_OBESITY	-0.78	0.07	0	0	0	not significant
7404	REACTOME_NOTCH1_INTRACELLULAR_DOMAIN_REGULATES_TRANSCRI	-0.78	0.07	0	0	0	not significant
7405	TCGA_GLIOBLASTOMA_COPY_NUMBER_UP	-0.78	0.07	0	0	0	not significant
7406	GO_IRON_ION_BINDING	-0.78	0.06	0	0	0	not significant
7407	CHRXP22	-0.78	0.05	0	0	0	not significant
7408	GO_PROTEIN_N_LINKED_GLYCOSYLATION	-0.78	0.05	0	0	0	not significant
7409	GO_NEGATIVE_REGULATION_OF_CELLULAR_AMIDE_METABOLIC_PROCE	-0.78	0.04	0	0	0	not significant
7410	REACTOME_MACROAUTOPHAGY	-0.78	0.04	0	0	0	not significant
7411	DARWICHE_PAPILLOMA_RISK_HIGH_DN	-0.78	0.02	0	0	0	not significant
7412	GO_TRANSCRIPTION_FACTOR_TFIIIC_COMPLEX	-0.77	0.15	0	0	0	not significant
7413	CHR19Q12	-0.77	0.14	0	0	0	not significant
7414	GO_NEUROTRANSMITTER_GATED_ION_CHANNEL_CLUSTERING	-0.77	0.14	0	0	0	not significant
7415	GO_RESPIRATORY_BURST_INVOLVED_IN_DEFENSE_RESPONSE	-0.77	0.14	0	0	0	not significant
7416	GO_SPINDLE_ELONGATION	-0.77	0.14	0	0	0	not significant
7417	REACTOME_SIGNAL_REGULATORY_PROTEIN_FAMILY_INTERACTIONS	-0.77	0.14	0	0	0	not significant
7418	GO_KIDNEY_MESENCHYME_DEVELOPMENT	-0.77	0.13	0	0	0	not significant
7419	GO_MUCOSAL_ASSOCIATED_LYMPHOID_TISSUE_DEVELOPMENT	-0.77	0.13	0	0	0	not significant
7420	GO_POLYAMINE_TRANSPORT	-0.77	0.13	0	0	0	not significant
7421	LU_TUMOR_ENDOTHELIAL_MARKERS_UP	-0.77	0.13	0	0	0	not significant
7422	PID_VEGF_VEGFR_PATHWAY	-0.77	0.13	0	0	0	not significant
7423	REACTOME_SIGNALING_BY_TGF_BETA_RECEPTOR_COMPLEX_IN_CANC	-0.77	0.13	0	0	0	not significant
7424	REACTOME_SUMO_IS_TRANSFERRED_FROM_E1_TO_E2_UBE2I_UBC9	-0.77	0.13	0	0	0	not significant
7425	BIOCARTA_D4GDI_PATHWAY	-0.77	0.12	0	0	0	not significant
7426	CAFFAREL_RESPONSE_TO_THC_8HR_5_DN	-0.77	0.12	0	0	0	not significant
7427	GALE_APL_WITH_FLT3_MUTATED_DN	-0.77	0.12	0	0	0	not significant
7428	GO_DNA_REPLICATION_PREINITIATION_COMPLEX	-0.77	0.12	0	0	0	not significant
7429	GO_REGULATION_OF_STEROID_HORMONE_BIOSYNTHETIC_PROCESS	-0.77	0.12	0	0	0	not significant
7430	REACTOME_GLYCEROPHOSPHOLIPID_CATABOLISM	-0.77	0.12	0	0	0	not significant
7431	WESTON_VEGFA_TARGETS_12HR	-0.77	0.12	0	0	0	not significant
7432	GO_C_ACYLTRANSFERASE_ACTIVITY	-0.77	0.11	0	0	0	not significant
7433	GO_MAMMARY_GLAND_DUCT_MORPHOGENESIS	-0.77	0.11	0	0	0	not significant
7434	GO_MISMATCHED_DNA_BINDING	-0.77	0.11	0	0	0	not significant
7435	REACTOME_RETROGRADE_NEUROTROPHIN_SIGNALLING	-0.77	0.11	0	0	0	not significant
7436	REACTOME_SODIUM_PROTON_EXCHANGERS	-0.77	0.11	0	0	0	not significant
7437	ZEMBUTSU_SENSITIVITY_TO_CYCLOPHOSPHAMIDE	-0.77	0.11	0	0	0	not significant
7438	GO_CELLULAR_BIOGENIC_AMINE_METABOLIC_PROCESS	-0.77	0.10	0	0	0	not significant
7439	GO_FERROUS_IRON_BINDING	-0.77	0.10	0	0	0	not significant
7440	GO_MEMBRANE_PROTEIN_ECTODOMAIN_PROTEOLYSIS	-0.77	0.10	0	0	0	not significant
7441	GO_MISFOLDED_PROTEIN_BINDING	-0.77	0.10	0	0	0	not significant
7442	GO_NEGATIVE_REGULATION_OF_PROTEIN_TYROSINE_KINASE_ACTIVITY	-0.77	0.10	0	0	0	not significant
7443	GO_NEGATIVE_REGULATION_OF_SIGNAL_TRANSDUCTION_BY_P53_CLA	-0.77	0.10	0	0	0	not significant
7444	GO_PEROXISOME_PROLIFERATOR_ACTIVATED_RECEPTOR_SIGNALING	-0.77	0.10	0	0	0	not significant
7445	GO_TRANSCRIPTIONALLY_ACTIVE_CHROMATIN	-0.77	0.10	0	0	0	not significant
7446	BIOCARTA_AGR_PATHWAY	-0.77	0.09	0	0	0	not significant
7447	DEMAGALHAES_AGING_UP	-0.77	0.09	0	0	0	not significant
7448	GO_DENDRITE_EXTENSION	-0.77	0.09	0	0	0	not significant
7449	GO_GLYCOSYL_COMPOUND_CATABOLIC_PROCESS	-0.77	0.09	0	0	0	not significant
7450	GO_NEGATIVE_REGULATION_OF_STRIATED_MUSCLE_CELL_DIFFERENT	-0.77	0.09	0	0	0	not significant
7451	GU_PDEF_TARGETS_UP	-0.77	0.09	0	0	0	not significant
7452	REACTOME_DISEASES_OF_CARBOHYDRATE_METABOLISM	-0.77	0.09	0	0	0	not significant
7453	BIOCARTA_INSULIN_PATHWAY	-0.77	0.08	0	0	0	not significant
7454	CHR6P25	-0.77	0.08	0	0	0	not significant
7455	GO_DETECTION_OF_VISIBLE_LIGHT	-0.77	0.08	0	0	0	not significant
7456	LUI_THYROID_CANCER_CLUSTER_1	-0.77	0.07	0	0	0	not significant
7457	PID_LYSOPHOSPHOLIPID_PATHWAY	-0.77	0.06	0	0	0	not significant
7458	WAKASUGI_HAVE_ZNF143_BINDING_SITES	-0.77	0.06	0	0	0	not significant
7459	GO_RETROGRADE_TRANSPORT_ENDOSOME_TO_GOLGI	-0.77	0.05	0	0	0	not significant
7460	GO_HISTONE_DEACETYLATION	-0.77	0.04	0	0	0	not significant
7461	GO_REGULATION_OF_PEPTIDYL_SERINE_PHOSPHORYLATION	-0.77	0.04	0	0	0	not significant
7462	GO_RNA_POLYMERASE_II_SPECIFIC_DNA_BINDING_TRANSCRIPTION_FA	-0.77	0.01	0	0	0	not significant
7463	NOUZOVA_TRETINOIN_AND_H4_ACETYLATION	-0.77	0.01	0	0	0	not significant
7464	SHEN_SMARCA2_TARGETS_UP	-0.77	0.00	0	0	0	not significant
7465	CHESLER_BRAIN_QTL_TRANS	-0.76	0.13	0	0	0	not significant
7466	GO_METANEPHRIC_MESENCHYME_DEVELOPMENT	-0.76	0.13	0	0	0	not significant
7467	GO_NEGATIVE_REGULATION_OF_PRODUCTION_OF_MIRNAS_INVOLVED	-0.76	0.13	0	0	0	not significant
7468	GO_POSITIVE_REGULATION_OF_NON_MOTILE_CILIUM_ASSEMBLY	-0.76	0.13	0	0	0	not significant
7469	GO_REGULATION_OF_GTP_BINDING	-0.76	0.13	0	0	0	not significant
7470	GO_REGULATION_OF_LIPOPROTEIN_LIPASE_ACTIVITY	-0.76	0.13	0	0	0	not significant
7471	GO_UTP_BIOSYNTHETIC_PROCESS	-0.76	0.13	0	0	0	not significant
7472	LIM_MAMMARY_LUMINAL_PROGENITOR_DN	-0.76	0.13	0	0	0	not significant
7473	REACTOME_PHOSPHATE_BOND_HYDROLYSIS_BY_NUDT_PROTEINS	-0.76	0.13	0	0	0	not significant
7474	BALLIF_DEVELOPMENTAL_DISABILITY_P16_P12_DELETION	-0.76	0.12	0	0	0	not significant
7475	GO_GABA_RECEPTOR_BINDING	-0.76	0.12	0	0	0	not significant
7476	GO_LYS48_SPECIFIC_DEUBIQUITINASE_ACTIVITY	-0.76	0.12	0	0	0	not significant
7477	GO_OLIGOPEPTIDE_TRANSPORT	-0.76	0.12	0	0	0	not significant
7478	GO_PHOSPHATIDYLINOSITOL_BISPHOSPHATE_KINASE_ACTIVITY	-0.76	0.12	0	0	0	not significant
7479	GO_POSITIVE_REGULATION_OF_PROTEIN_DEUBIQUITINATION	-0.76	0.12	0	0	0	not significant
7480	GO_REGULATION_OF_HYDROGEN_PEROXIDE_METABOLIC_PROCESS	-0.76	0.12	0	0	0	not significant
7481	GO_SINGLE_STRANDED_DNA_ENDODEOXYRIBONUCLEASE_ACTIVITY	-0.76	0.12	0	0	0	not significant
7482	GO_TRANSLATION_ACTIVATOR_ACTIVITY	-0.76	0.12	0	0	0	not significant
7483	REACTOME_VEGF_LIGAND_RECEPTOR_INTERACTIONS	-0.76	0.12	0	0	0	not significant
7484	SEMBA_FHIT_TARGETS_DN	-0.76	0.12	0	0	0	not significant
7485	ZHAN_EARLY_DIFFERENTIATION_GENES_UP	-0.76	0.12	0	0	0	not significant
7486	BIOCARTA_LEPTIN_PATHWAY	-0.76	0.11	0	0	0	not significant
7487	DORN_ADENOVIRUS_INFECTION_48HR_UP	-0.76	0.11	0	0	0	not significant
7488	GEISS_RESPONSE_TO_DSRNA_DN	-0.76	0.11	0	0	0	not significant
7489	GO_DENDRITIC_CELL_CYTOKINE_PRODUCTION	-0.76	0.11	0	0	0	not significant
7490	GO_ER_MEMBRANE_PROTEIN_COMPLEX	-0.76	0.11	0	0	0	not significant
7491	GO_LIGASE_ACTIVITY_FORMING_CARBON_CARBON_BONDS	-0.76	0.11	0	0	0	not significant
7492	GO_PALMITOYLTRANSFERASE_COMPLEX	-0.76	0.11	0	0	0	not significant
7493	GO_POSITIVE_REGULATION_OF_HEART_CONTRACTION	-0.76	0.11	0	0	0	not significant
7494	GO_POSITIVE_REGULATION_OF_HISTONE_PHOSPHORYLATION	-0.76	0.11	0	0	0	not significant
7495	GO_PURINERGIC_RECEPTOR_ACTIVITY	-0.76	0.11	0	0	0	not significant
7496	GO_REGULATION_OF_CELL_CELL_ADHESION_MEDIATED_BY_CADHERIN	-0.76	0.11	0	0	0	not significant
7497	GO_RESPONSE_TO_LEPTIN	-0.76	0.11	0	0	0	not significant
7498	ZHOU_CELL_CYCLE_GENES_IN_IR_RESPONSE_2HR	-0.76	0.11	0	0	0	not significant
7499	GO_GTP_METABOLIC_PROCESS	-0.76	0.10	0	0	0	not significant
7500	GO_NEUROPEPTIDE_SIGNALING_PATHWAY	-0.76	0.10	0	0	0	not significant
7501	GO_PLACENTA_BLOOD_VESSEL_DEVELOPMENT	-0.76	0.10	0	0	0	not significant
7502	REACTOME_MECP2_REGULATES_NEURONAL_RECEPTORS_AND_CHANN	-0.76	0.10	0	0	0	not significant
7503	GO_LEUKOCYTE_ADHESION_TO_VASCULAR_ENDOTHELIAL_CELL	-0.76	0.09	0	0	0	not significant
7504	REACTOME_PRE_NOTCH_PROCESSING_IN_GOLGI	-0.76	0.09	0	0	0	not significant
7505	WANG_BARRETTS_ESOPHAGUS_DN	-0.76	0.09	0	0	0	not significant
7506	GO_NEUROBLAST_PROLIFERATION	-0.76	0.08	0	0	0	not significant
7507	GO_POSITIVE_REGULATION_OF_GLUCOSE_TRANSMEMBRANE_TRANSP	-0.76	0.08	0	0	0	not significant
7508	GO_REGULATION_OF_ORGANIC_ACID_TRANSPORT	-0.76	0.08	0	0	0	not significant
7509	REACTOME_CARGO_CONCENTRATION_IN_THE_ER	-0.76	0.08	0	0	0	not significant

7510	REACTOME_PEROXISOMAL_LIPID_METABOLISM	-0.76	0.08	0	0	0	not significant
7511	BURTON_ADIPOGENESIS_12	-0.76	0.07	0	0	0	not significant
7512	GO_LAMELLIPODIUM_ASSEMBLY	-0.76	0.07	0	0	0	not significant
7513	GO_REGULATION_OF_VASCULAR_SMOOTH_MUSCLE_CELL_PROLIFERA	-0.76	0.07	0	0	0	not significant
7514	HAHTOLA_MYCOSIS_FUNGOIDES_CD4_UP	-0.76	0.07	0	0	0	not significant
7515	BIOCARTA_NFAT_PATHWAY	-0.76	0.06	0	0	0	not significant
7516	GO_REGULATION_OF_PROTEIN_TYROSINE_KINASE_ACTIVITY	-0.76	0.06	0	0	0	not significant
7517	CHR3Q13	-0.76	0.05	0	0	0	not significant
7518	GO_REGULATION_OF_SYNAPSE_ASSEMBLY	-0.76	0.05	0	0	0	not significant
7519	REACTOME_MEIOTIC_SYNAPSIS	-0.76	0.05	0	0	0	not significant
7520	GO_NEURAL_TUBE_FORMATION	-0.76	0.04	0	0	0	not significant
7521	GO_POSITIVE_REGULATION_OF_PROTEIN_BINDING	-0.76	0.04	0	0	0	not significant
7522	YEGNASUBRAMANIAN_PROSTATE_CANCER	-0.76	0.04	0	0	0	not significant
7523	GO_NUCLEAR_RECEPTOR_BINDING	-0.76	0.03	0	0	0	not significant
7524	GO_GOLGI_ASSOCIATED_VESICLE	-0.76	0.02	0	0	0	not significant
7525	GO_PROTEIN_KINASE_B_SIGNALING	-0.76	0.02	0	0	0	not significant
7526	GO_SYNAPTIC_VESICLE_CYCLE	-0.76	0.02	0	0	0	not significant
7527	GO_HORMONE_RECEPTOR_BINDING	-0.76	0.01	0	0	0	not significant
7528	CHR20Q12	-0.75	0.13	0	0	0	not significant
7529	GO_SUSCEPTIBILITY_TO_NATURAL_KILLER_CELL_MEDIATED_CYTOTOX	-0.75	0.12	0	0	0	not significant
7530	BAUS_TFF2_TARGETS_DN	-0.75	0.11	0	0	0	not significant
7531	GO_BIOTIN_METABOLIC_PROCESS	-0.75	0.11	0	0	0	not significant
7532	GO_DEOXYRIBOSE_PHOSPHATE_BIOSYNTHETIC_PROCESS	-0.75	0.11	0	0	0	not significant
7533	GO_LOW_DENSITY_LIPOPROTEIN_PARTICLE_RECEPTOR_ACTIVITY	-0.75	0.11	0	0	0	not significant
7534	GO_NEGATIVE_REGULATION_OF_MACROPHAGE_MIGRATION	-0.75	0.11	0	0	0	not significant
7535	GO_POSITIVE_REGULATION_OF_TRANSCRIPTION_BY_RNA_POLYMERAS	-0.75	0.11	0	0	0	not significant
7536	GO_POSTSYNAPTIC_NEUROTRANSMITTER_RECEPTOR_ACTIVITY	-0.75	0.11	0	0	0	not significant
7537	GO_REGULATION_OF_AEROBIC_RESPIRATION	-0.75	0.11	0	0	0	not significant
7538	GO_REGULATION_OF_ATP_BIOSYNTHETIC_PROCESS	-0.75	0.11	0	0	0	not significant
7539	GO_REGULATION_OF_HEART_RATE_BY_CHEMICAL_SIGNAL	-0.75	0.11	0	0	0	not significant
7540	GO_TRANSCYTOSIS	-0.75	0.11	0	0	0	not significant
7541	MIKKELSEN_IPS_ICP_WITH_H3K27ME3	-0.75	0.11	0	0	0	not significant
7542	ZHENG_FOXP3_TARGETS_IN_T_LYMPHOCYTE_UP	-0.75	0.11	0	0	0	not significant
7543	GO_COMMON_PARTNER_SMAD_PROTEIN_PHOSPHORYLATION	-0.75	0.10	0	0	0	not significant
7544	GO_GLYCOGEN_GRANULE	-0.75	0.10	0	0	0	not significant
7545	HAHTOLA_MYCOSIS_FUNGOIDES_UP	-0.75	0.10	0	0	0	not significant
7546	SPIELMAN_LYMPHOBLAST_EUROPEAN_VS_ASIAN_2FC_UP	-0.75	0.10	0	0	0	not significant
7547	BIOCARTA_HCMV_PATHWAY	-0.75	0.09	0	0	0	not significant
7548	GO_CEREBRAL_CORTEX_CELL_MIGRATION	-0.75	0.09	0	0	0	not significant
7549	REACTOME_BIOTIN_TRANSPORT_AND_METABOLISM	-0.75	0.09	0	0	0	not significant
7550	GO_EXODEOXYRIBONUCLEASE_ACTIVITY	-0.75	0.08	0	0	0	not significant
7551	GO_MITOCHONDRIAL_FUSION	-0.75	0.08	0	0	0	not significant
7552	GO_NEGATIVE_REGULATION_OF_BLOOD_PRESSURE	-0.75	0.08	0	0	0	not significant
7553	GO_ENDODERM_FORMATION	-0.75	0.07	0	0	0	not significant
7554	GO_GLYCOSYL_COMPOUND_BIOSYNTHETIC_PROCESS	-0.75	0.07	0	0	0	not significant
7555	REACTOME_CASPASE_ACTIVATION_VIA_EXTRINSIC_APOPTOTIC_SIGNAL	-0.75	0.07	0	0	0	not significant
7556	REACTOME_EGFR_DOWNREGULATION	-0.75	0.07	0	0	0	not significant
7557	CHANGOLKAR_H2AFY_TARGETS_UP	-0.75	0.06	0	0	0	not significant
7558	SUBTIL_PROGESTIN_TARGETS	-0.75	0.06	0	0	0	not significant
7559	BOYAULT_LIVER_CANCER_SUBCLASS_G12_UP	-0.75	0.05	0	0	0	not significant
7560	GO_SCF_UBIQUITIN_LIGASE_COMPLEX	-0.75	0.05	0	0	0	not significant
7561	KEGG_VEGF_SIGNALING_PATHWAY	-0.75	0.05	0	0	0	not significant
7562	PID_PI3KCI_PATHWAY	-0.75	0.05	0	0	0	not significant
7563	RIZKI_TUMOR_INVASIVENESS_2D_DN	-0.75	0.05	0	0	0	not significant
7564	GO_REGULATION_OF_EMBRYONIC_DEVELOPMENT	-0.75	0.04	0	0	0	not significant
7565	GO_NEURAL_PRECURSOR_CELL_PROLIFERATION	-0.75	0.03	0	0	0	not significant
7566	BROWNE_HCMV_INFECTION_18HR_UP	-0.75	0.01	0	0	0	not significant
7567	GO_DNA_BINDING_TRANSCRIPTION_FACTOR_BINDING	-0.75	0.00	0	0	0	not significant
7568	GO_MACROAUTOPHAGY	-0.75	0.00	0	0	0	not significant
7569	GO_REGULATION_OF_AUTOPHAGY	-0.75	0.00	0	0	0	not significant
7570	GO_COPPER_ION_TRANSMEMBRANE_TRANSPORT	-0.74	0.13	0	0	0	not significant
7571	DONATO_CELL_CYCLE_TRETINOIN	-0.74	0.12	0	0	0	not significant
7572	GO_IKAPPAB_KINASE_COMPLEX	-0.74	0.12	0	0	0	not significant
7573	GO_PERIPHERAL_NERVOUS_SYSTEM_NEURON_DIFFERENTIATION	-0.74	0.12	0	0	0	not significant
7574	GO_POSITIVE_REGULATION_OF_PROTEIN_EXIT_FROM_ENDOPLASMIC_F	-0.74	0.12	0	0	0	not significant
7575	GO_RNA_POLYMERASE_II_CTD_HEPTAPEPTIDE_REPEAT_PHOSPHATASE	-0.74	0.12	0	0	0	not significant
7576	GO_AROMATIC_AMINO_ACID_TRANSMEMBRANE_TRANSPORTER_ACTIVI	-0.74	0.11	0	0	0	not significant
7577	GO_FATTY_ACYL_COA_METABOLIC_PROCESS	-0.74	0.11	0	0	0	not significant
7578	GO_GLYCOPROTEIN_CATABOLIC_PROCESS	-0.74	0.11	0	0	0	not significant
7579	GO_GUANYLYLTRANSFERASE_ACTIVITY	-0.74	0.11	0	0	0	not significant
7580	GO_INTERLEUKIN_35_MEDIATED_SIGNALING_PATHWAY	-0.74	0.11	0	0	0	not significant
7581	GO_INTERMEDIATE_FILAMENT_BINDING	-0.74	0.11	0	0	0	not significant
7582	GO_MONOVALENT_CATION_PROTON_ANTIPORTER_ACTIVITY	-0.74	0.11	0	0	0	not significant
7583	GO_NEPHRIC_DUCT_MORPHOGENESIS	-0.74	0.11	0	0	0	not significant
7584	GO_RNA_POLYMERASE_III_TRANSCRIPTION_FACTOR_COMPLEX	-0.74	0.11	0	0	0	not significant
7585	GO_TELOMERASE_HOLOENZYME_COMPLEX_ASSEMBLY	-0.74	0.11	0	0	0	not significant
7586	REACTOME_DCC_MEDIATED_ATTRACTIVE_SIGNALING	-0.74	0.11	0	0	0	not significant
7587	REACTOME_NONCANONICAL_ACTIVATION_OF_NOTCH3	-0.74	0.11	0	0	0	not significant
7588	BIOCARTA_SPRY_PATHWAY	-0.74	0.10	0	0	0	not significant
7589	GO_ACETYL_COA_C_ACYLTRANSFERASE_ACTIVITY	-0.74	0.10	0	0	0	not significant
7590	GO_AORTIC_VALVE_DEVELOPMENT	-0.74	0.10	0	0	0	not significant
7591	GO_POSITIVE_REGULATION_OF_ATP_BIOSYNTHETIC_PROCESS	-0.74	0.10	0	0	0	not significant
7592	GO_POSITIVE_REGULATION_OF_TELOMERE_CAPPING	-0.74	0.10	0	0	0	not significant
7593	HOLLERN_MICROACINAR_BREAST_TUMOR_DN	-0.74	0.10	0	0	0	not significant
7594	NIKOLSKY_BREAST_CANCER_10Q22_AMPLICON	-0.74	0.10	0	0	0	not significant
7595	PEPPER_CHRONIC_LYMPHOCYTIC_LEUKEMIA_UP	-0.74	0.10	0	0	0	not significant
7596	REACTOME_JOSEPHIN_DOMAIN_DUBS	-0.74	0.10	0	0	0	not significant
7597	GO_UMP_BIOSYNTHETIC_PROCESS	-0.74	0.09	0	0	0	not significant
7598	MAGRANGEAS_MULTIPLE_MYELOMA_IGLL_VS_IGLK_DN	-0.74	0.09	0	0	0	not significant
7599	REACTOME_PROCESSING_AND_ACTIVATION_OF_SUMO	-0.74	0.09	0	0	0	not significant
7600	CHR17P12	-0.74	0.08	0	0	0	not significant
7601	GO_CHOLESTEROL_EFFLUX	-0.74	0.08	0	0	0	not significant
7602	GO_GLUTAMATE_METABOLIC_PROCESS	-0.74	0.08	0	0	0	not significant
7603	GO_INTRACILIARY_TRANSPORT_PARTICLE_B	-0.74	0.08	0	0	0	not significant
7604	GO_NUCLEOTIDE_EXCISION_REPAIR_DNA_DUPLEX_UNWINDING	-0.74	0.08	0	0	0	not significant
7605	GO_POSITIVE_REGULATION_OF_SMOOTH_MUSCLE_CELL_MIGRATION	-0.74	0.08	0	0	0	not significant
7606	GO_PYRIMIDINE_NUCLEOTIDE_CATABOLIC_PROCESS	-0.74	0.08	0	0	0	not significant
7607	GO_SPERM_EGG_RECOGNITION	-0.74	0.08	0	0	0	not significant
7608	REACTOME_DEFECTS_IN_VITAMIN_AND_COFACTOR_METABOLISM	-0.74	0.08	0	0	0	not significant
7609	GO_POSITIVE_REGULATION_OF_TOR_SIGNALING	-0.74	0.07	0	0	0	not significant
7610	KAAB_FAILED_HEART_ATRIUM_UP	-0.74	0.07	0	0	0	not significant
7611	PID_RAC1_REG_PATHWAY	-0.74	0.07	0	0	0	not significant
7612	ENGELMANN_CANCER_PROGENITORS_DN	-0.74	0.06	0	0	0	not significant
7613	GO_AMMONIUM_ION_BINDING	-0.74	0.06	0	0	0	not significant
7614	GO_PERICENTRIOLAR_MATERIAL	-0.74	0.06	0	0	0	not significant
7615	CHR1P31	-0.74	0.05	0	0	0	not significant
7616	CHR2P11	-0.74	0.05	0	0	0	not significant
7617	JI_RESPONSE_TO_FSH_DN	-0.74	0.05	0	0	0	not significant
7618	KEGG_DNA_REPLICATION	-0.74	0.05	0	0	0	not significant

7619	PID_INSULIN_PATHWAY	-0.74	0.05	0	0	0	not significant
7620	RASHI_RESPONSE_TO_IONIZING_RADIATION_4	-0.74	0.05	0	0	0	not significant
7621	GO_HETEROCHROMATIN	-0.74	0.03	0	0	0	not significant
7622	GO_POSITIVE_REGULATION_OF_PLASMA_MEMBRANE_BOUNDED_CELL_	-0.74	0.03	0	0	0	not significant
7623	KEGG_MAPK_SIGNALING_PATHWAY	-0.74	0.00	0	0	0	not significant
7624	GO_PONS_DEVELOPMENT	-0.73	0.12	0	0	0	not significant
7625	GO_NEGATIVE_REGULATION_OF_CALCIUM_ION_DEPENDENT_EXOCYTOS	-0.73	0.11	0	0	0	not significant
7626	GO_REGULATION_OF_RESPONSE_TO_TUMOR_CELL	-0.73	0.11	0	0	0	not significant
7627	MINGUEZ_LIVER_CANCER_VASCULAR_INVASION_DN	-0.73	0.11	0	0	0	not significant
7628	AMIT_EGF_RESPONSE_20_HELA	-0.73	0.10	0	0	0	not significant
7629	GO_AT_DNA_BINDING	-0.73	0.10	0	0	0	not significant
7630	GO_CELLULAR_RESPONSE_TO_ATP	-0.73	0.10	0	0	0	not significant
7631	GO_DIPHOSPHOTRANSFERASE_ACTIVITY	-0.73	0.10	0	0	0	not significant
7632	REACTOME_RECEPTOR_MEDIATED_MITOPHAGY	-0.73	0.10	0	0	0	not significant
7633	GO_5S_RRNA_BINDING	-0.73	0.09	0	0	0	not significant
7634	GO_LEUKOCYTE_AGGREGATION	-0.73	0.09	0	0	0	not significant
7635	GO_PHOSPHATIDYLINOSITOL_3_PHOSPHATE_BIOSYNTHETIC_PROCESS	-0.73	0.09	0	0	0	not significant
7636	GO_POSITIVE_REGULATION_OF_CARDIAC_MUSCLE_CELL_DIFFERENTIA	-0.73	0.09	0	0	0	not significant
7637	GO_PROTEIN_PROCESSING_INVOLVED_IN_PROTEIN_TARGETING_TO_MI	-0.73	0.09	0	0	0	not significant
7638	GO_PYRIMIDINE_RIBONUCLEOTIDE_BIOSYNTHETIC_PROCESS	-0.73	0.09	0	0	0	not significant
7639	BIOCARTA_RANKL_PATHWAY	-0.73	0.08	0	0	0	not significant
7640	GO_WW_DOMAIN_BINDING	-0.73	0.08	0	0	0	not significant
7641	KEGG_ONE_CARBON_POOL_BY_FOLATE	-0.73	0.08	0	0	0	not significant
7642	REACTOME_TICAM1_DEPENDENT_ACTIVATION_OF_IRF3_IRF7	-0.73	0.08	0	0	0	not significant
7643	WATANABE_COLON_CANCER_MSI_VS_MSS_DN	-0.73	0.08	0	0	0	not significant
7644	GO_BHLH_TRANSCRIPTION_FACTOR_BINDING	-0.73	0.07	0	0	0	not significant
7645	GO_SODIUM_ION_HOMEOSTASIS	-0.73	0.07	0	0	0	not significant
7646	GO_VIRAL_LATENCY	-0.73	0.07	0	0	0	not significant
7647	LEE_AGING_MUSCLE_DN	-0.73	0.07	0	0	0	not significant
7648	CHESLER_BRAIN_HIGHEST_EXPRESSION	-0.73	0.06	0	0	0	not significant
7649	GO_MICROTUBULE_PLUS_END	-0.73	0.06	0	0	0	not significant
7650	GO_REGULATION_OF_ANDROGEN_RECEPTOR_SIGNALING_PATHWAY	-0.73	0.06	0	0	0	not significant
7651	GO_LYSOSOME_LOCALIZATION	-0.73	0.04	0	0	0	not significant
7652	GO_MICROTUBULE_ORGANIZING_CENTER_ORGANIZATION	-0.73	0.02	0	0	0	not significant
7653	GO_NEURON_MIGRATION	-0.73	0.02	0	0	0	not significant
7654	GO_POST_GOLGI_VESICLE_MEDIATED_TRANSPORT	-0.73	0.02	0	0	0	not significant
7655	GO_UBIQUITIN_BINDING	-0.73	0.02	0	0	0	not significant
7656	REACTOME_SIGNALING_BY_NTRKS	-0.73	0.02	0	0	0	not significant
7657	GO_REGULATION_OF_MUSCLE_CELL_DIFFERENTIATION	-0.73	0.01	0	0	0	not significant
7658	GO_PEPTIDYL_LYSINE_MODIFICATION	-0.73	0.00	0	0	0	not significant
7659	GO_REGULATION_OF_ORGANELLE_ASSEMBLY	-0.73	0.00	0	0	0	not significant
7660	GO_PEPTIDYL_LYSINE_HYDROXYLATION	-0.72	0.11	0	0	0	not significant
7661	DING_LUNG_CANCER_MUTATED_FREQUENTLY	-0.72	0.10	0	0	0	not significant
7662	GO_DEOXYRIBONUCLEOSIDE_TRIPHOSPHATE_BIOSYNTHETIC_PROCESS	-0.72	0.10	0	0	0	not significant
7663	GO_HISTONE_METHYLTRANSFERASE_ACTIVITY_H3_K36_SPECIFIC_	-0.72	0.10	0	0	0	not significant
7664	GO_POSITIVE_REGULATION_OF_PINOCYTOSIS	-0.72	0.10	0	0	0	not significant
7665	GO_PROTON_TRANSPORTING_V_TYPE_ATPASE_V1_DOMAIN	-0.72	0.10	0	0	0	not significant
7666	GO_REGULATION_OF_NEUTROPHIL_DEGRANULATION	-0.72	0.10	0	0	0	not significant
7667	GO_REGULATION_OF_PROTEIN_K63_LINKED_UBIQUITINATION	-0.72	0.10	0	0	0	not significant
7668	MYLLYKANGAS_AMPLIFICATION_HOT_SPOT_16	-0.72	0.10	0	0	0	not significant
7669	REACTOME_INTERLEUKIN_35_SIGNALLING	-0.72	0.10	0	0	0	not significant
7670	CHR7P11	-0.72	0.09	0	0	0	not significant
7671	GO_CALYX_OF_HELD	-0.72	0.09	0	0	0	not significant
7672	GO_CILIARY_ROOTLET	-0.72	0.09	0	0	0	not significant
7673	GO_POLYPEPTIDE_N_ACETYLGALACTOSAMINYLTRANSFERASE_ACTIVIT	-0.72	0.09	0	0	0	not significant
7674	GO_PROTEIN_LIPID_COMPLEX_BINDING	-0.72	0.09	0	0	0	not significant
7675	RAFFEL_VEGFA_TARGETS_UP	-0.72	0.09	0	0	0	not significant
7676	SCHMAHL_PDGF_SIGNALING	-0.72	0.09	0	0	0	not significant
7677	GO_BLOC_1_COMPLEX	-0.72	0.08	0	0	0	not significant
7678	GO_HYDROGEN_PEROXIDE_METABOLIC_PROCESS	-0.72	0.08	0	0	0	not significant
7679	GO_REGULATION_OF_NUCLEOTIDE_BIOSYNTHETIC_PROCESS	-0.72	0.08	0	0	0	not significant
7680	GO_REGULATION_OF_PROGRAMMED_NECROTIC_CELL_DEATH	-0.72	0.08	0	0	0	not significant
7681	REACTOME_SIGNALING_BY_LIGAND_RESPONSIVE_EGFR_VARIANTS_IN_(	-0.72	0.08	0	0	0	not significant
7682	GO_DNA_POLYMERASE_COMPLEX	-0.72	0.07	0	0	0	not significant
7683	GO_DNA_REPLICATION_FACTOR_A_COMPLEX	-0.72	0.07	0	0	0	not significant
7684	GO_LATE_ENDOSOME_TO_VACUOLE_TRANSPORT	-0.72	0.07	0	0	0	not significant
7685	GO_MRNA_METHYLATION	-0.72	0.07	0	0	0	not significant
7686	GO_NEGATIVE_REGULATION_OF_TRANSLATIONAL_INITIATION	-0.72	0.07	0	0	0	not significant
7687	GO_REGULATION_OF_PROTEIN_HOMODIMERIZATION_ACTIVITY	-0.72	0.07	0	0	0	not significant
7688	REACTOME_SIGNALING_BY_EGFR_IN_CANCER	-0.72	0.07	0	0	0	not significant
7689	REACTOME_TERMINATION_OF_TRANSLESION_DNA_SYNTHESIS	-0.72	0.05	0	0	0	not significant
7690	GO_CLATHRIN_COATED_ENDOCYTIC_VESICLE	-0.72	0.04	0	0	0	not significant
7691	GO_METALLOENDOPEPTIDASE_ACTIVITY	-0.72	0.04	0	0	0	not significant
7692	GO_P38MAPK_CASCADE	-0.72	0.04	0	0	0	not significant
7693	PID_FAS_PATHWAY	-0.72	0.04	0	0	0	not significant
7694	BYSTROEM_CORRELATED_WITH_IL5_DN	-0.72	0.03	0	0	0	not significant
7695	GO_PHOSPHATIDYLINOSITOL_PHOSPHORYLATION	-0.72	0.03	0	0	0	not significant
7696	GO_ACTIVATION_OF_GTPASE_ACTIVITY	-0.72	0.02	0	0	0	not significant
7697	GO_N_ACETYLTRANSFERASE_ACTIVITY	-0.72	0.02	0	0	0	not significant
7698	GO_PROTEIN_ACETYLTRANSFERASE_COMPLEX	-0.72	0.02	0	0	0	not significant
7699	RB_P130_DN.V1_DN	-0.72	0.02	0	0	0	not significant
7700	KESHELAVA_MULTIPLE_DRUG_RESISTANCE	-0.72	0.01	0	0	0	not significant
7701	SHAFFER_IRF4_TARGETS_IN_PLASMA_CELL_VS_MATURE_B_LYMPHOCY	-0.72	0.01	0	0	0	not significant
7702	GO_NEGATIVE_REGULATION_OF_GLUCOSE_IMPORT	-0.71	0.10	0	0	0	not significant
7703	GO_PENTOSE_PHOSPHATE_SHUNT_NON_OXIDATIVE_BRANCH	-0.71	0.10	0	0	0	not significant
7704	GO_REGULATION_OF_ENDOSOME_SIZE	-0.71	0.10	0	0	0	not significant
7705	CHR13Q21	-0.71	0.09	0	0	0	not significant
7706	DORN_ADENOVIRUS_INFECTION_24HR_UP	-0.71	0.09	0	0	0	not significant
7707	GO_4_HYDROXYPROLINE_METABOLIC_PROCESS	-0.71	0.09	0	0	0	not significant
7708	GO_BONE_MARROW_DEVELOPMENT	-0.71	0.09	0	0	0	not significant
7709	GO_CELLULAR_RESPONSE_TO_PROSTAGLANDIN_E_STIMULUS	-0.71	0.09	0	0	0	not significant
7710	GO_LOW_DENSITY_LIPOPROTEIN_PARTICLE_RECEPTOR_BIOSYNTHETIC	-0.71	0.09	0	0	0	not significant
7711	GO_NEGATIVE_REGULATION_OF_MITOCHONDRIAL_FUSION	-0.71	0.09	0	0	0	not significant
7712	GO_NEGATIVE_REGULATION_OF_RESPONSE_TO_EXTRACELLULAR_STIM	-0.71	0.09	0	0	0	not significant
7713	GO_PENTOSE_CATABOLIC_PROCESS	-0.71	0.09	0	0	0	not significant
7714	GO_PICLN_SM_PROTEIN_COMPLEX	-0.71	0.09	0	0	0	not significant
7715	BIOCARTA_CTBP1_PATHWAY	-0.71	0.08	0	0	0	not significant
7716	BIOCARTA_PLCE_PATHWAY	-0.71	0.08	0	0	0	not significant
7717	GO_CELLULAR_RESPONSE_TO_PROSTAGLANDIN_STIMULUS	-0.71	0.08	0	0	0	not significant
7718	OXFORD_RALA_AND_RALB_TARGETS_UP	-0.71	0.08	0	0	0	not significant
7719	PID_BETA_CATENIN_DEG_PATHWAY	-0.71	0.08	0	0	0	not significant
7720	TAKADA_GASTRIC_CANCER_COPY_NUMBER_DN	-0.71	0.08	0	0	0	not significant
7721	CHR3Q12	-0.71	0.07	0	0	0	not significant
7722	FLOTHO_PEDIATRIC_ALL_THERAPY_RESPONSE_DN	-0.71	0.07	0	0	0	not significant
7723	GARGALOVIC_RESPONSE_TO_OXIDIZED_PHOSPHOLIPIDS_MAGENTA_DN	-0.71	0.07	0	0	0	not significant
7724	GO_C21_STEROID_HORMONE_BIOSYNTHETIC_PROCESS	-0.71	0.07	0	0	0	not significant
7725	GO_DOPAMINE_RECEPTOR_BINDING	-0.71	0.07	0	0	0	not significant
7726	GO_EXOCYST	-0.71	0.07	0	0	0	not significant
7727	GO_NEGATIVE_REGULATION_OF_NEUROLOGICAL_SYSTEM_PROCESS	-0.71	0.07	0	0	0	not significant

7728	GO_POLYSACCHARIDE_CATABOLIC_PROCESS	-0.71	0.07	0	0	0	not significant
7729	GO_POSITIVE_REGULATION_OF_CARDIOCYTE_DIFFERENTIATION	-0.71	0.07	0	0	0	not significant
7730	GO_TELOMERE_MAINTENANCE_VIA_RECOMBINATION	-0.71	0.07	0	0	0	not significant
7731	JEPSEN_SMRT_TARGETS	-0.71	0.07	0	0	0	not significant
7732	MUELLER_METHYLATED_IN_GLIOBLASTOMA	-0.71	0.07	0	0	0	not significant
7733	GO_AMYLOID_BETA_METABOLIC_PROCESS	-0.71	0.06	0	0	0	not significant
7734	GO_REGULATION_OF_PRESYNAPSE_ORGANIZATION	-0.71	0.06	0	0	0	not significant
7735	GO_ZINC_ION_TRANSPORT	-0.71	0.06	0	0	0	not significant
7736	ZHENG_IL22_SIGNALING_UP	-0.71	0.06	0	0	0	not significant
7737	BREDEMEYER_RAG_SIGNALING_VIA_ATM_NOT_VIA_NFKB_DN	-0.71	0.05	0	0	0	not significant
7738	GO_DNA_CATABOLIC_PROCESS_ENDONUCLEOLYTIC	-0.71	0.05	0	0	0	not significant
7739	GO_MITOCHONDRION_LOCALIZATION	-0.71	0.05	0	0	0	not significant
7740	GO_RETROMER_COMPLEX	-0.71	0.05	0	0	0	not significant
7741	CHR14Q22	-0.71	0.04	0	0	0	not significant
7742	BCAT_BILD_ET_AL_UP	-0.71	0.03	0	0	0	not significant
7743	HEDENFALK_BREAST_CANCER_HEREDITARY_VS_SPORADIC	-0.71	0.03	0	0	0	not significant
7744	CHR2P23	-0.71	0.02	0	0	0	not significant
7745	GO_POSITIVE_REGULATION_OF_PEPTIDYL_SERINE_PHOSPHORYLATION	-0.71	0.02	0	0	0	not significant
7746	GO_RNA_POLYMERASE_BINDING	-0.71	0.02	0	0	0	not significant
7747	GO_SYNAPTIC_VESICLE_LOCALIZATION	-0.71	0.01	0	0	0	not significant
7748	REACTOME_REGULATION_OF_TP53_ACTIVITY_THROUGH_PHOSPHORYL	-0.71	0.01	0	0	0	not significant
7749	GO_DNA_REPLICATION	-0.71	0.00	0	0	0	not significant
7750	GO_LATE_ENDOSOME	-0.71	0.00	0	0	0	not significant
7751	REACTOME_CHROMATIN_ORGANIZATION	-0.71	0.00	0	0	0	not significant
7752	GO_COPPER_ION_TRANSMEMBRANE_TRANSPORTER_ACTIVITY	-0.70	0.09	0	0	0	not significant
7753	GO_DNA_RECOMBINASE_MEDIATOR_COMPLEX	-0.70	0.09	0	0	0	not significant
7754	GO_POSITIVE_REGULATION_OF_TOLL_LIKE_RECEPTOR_2_SIGNALING_F	-0.70	0.09	0	0	0	not significant
7755	GO_STRESS_GRANULE_DISASSEMBLY	-0.70	0.09	0	0	0	not significant
7756	GO_BETA_CATENIN_DESTRUCTION_COMPLEX_ASSEMBLY	-0.70	0.08	0	0	0	not significant
7757	GO_CELLULAR_RESPONSE_TO_TESTOSTERONE_STIMULUS	-0.70	0.08	0	0	0	not significant
7758	GO_LENS_MORPHOGENESIS_IN_CAMERA_TYPE_EYE	-0.70	0.08	0	0	0	not significant
7759	GO_POSITIVE_REGULATION_OF_LYASE_ACTIVITY	-0.70	0.08	0	0	0	not significant
7760	GO_POSITIVE_REGULATION_OF_ORGANIC_ACID_TRANSPORT	-0.70	0.08	0	0	0	not significant
7761	GO_PROTEIN_LOCALIZATION_TO_NON_MOTILE_CILIUM	-0.70	0.08	0	0	0	not significant
7762	GOERING_BLOOD_HDL_CHOLESTEROL_QTL_CIS	-0.70	0.08	0	0	0	not significant
7763	REACTOME_DISEASES_OF_MISMATCH_REPAIR_MMR	-0.70	0.08	0	0	0	not significant
7764	CHEOK_RESPONSE_TO_MERCAPTOPURINE_AND_LD_MTX_UP	-0.70	0.07	0	0	0	not significant
7765	FARMER_BREAST_CANCER_CLUSTER_6	-0.70	0.07	0	0	0	not significant
7766	GO_CDP_CHOLINE_PATHWAY	-0.70	0.07	0	0	0	not significant
7767	GO_EPHRIN_RECEPTOR_BINDING	-0.70	0.07	0	0	0	not significant
7768	GO_FUCOSE_METABOLIC_PROCESS	-0.70	0.07	0	0	0	not significant
7769	GO_INVADOPODIUM	-0.70	0.07	0	0	0	not significant
7770	GO_PHARYNGEAL_ARCH_ARTERY_MORPHOGENESIS	-0.70	0.07	0	0	0	not significant
7771	GO_SODIUM_ION_IMPORT_ACROSS_PLASMA_MEMBRANE	-0.70	0.07	0	0	0	not significant
7772	GO_BLASTOCYST_GROWTH	-0.70	0.06	0	0	0	not significant
7773	GO_NEGATIVE_REGULATION_OF_HISTONE_H3_K9_METHYLATION	-0.70	0.06	0	0	0	not significant
7774	GO_NEUROTROPHIN_RECEPTOR_BINDING	-0.70	0.06	0	0	0	not significant
7775	GO_REGULATION_OF_DNA_DAMAGE_CHECKPOINT	-0.70	0.06	0	0	0	not significant
7776	PID_HNF3A_PATHWAY	-0.70	0.06	0	0	0	not significant
7777	BIOCARTA_CASPASE_PATHWAY	-0.70	0.05	0	0	0	not significant
7778	GO_REGULATION_OF_MYOTUBE_DIFFERENTIATION	-0.70	0.05	0	0	0	not significant
7779	ISSAEVA_MLL2_TARGETS	-0.70	0.05	0	0	0	not significant
7780	PID_NEPHRIN_NEPH1_PATHWAY	-0.70	0.05	0	0	0	not significant
7781	GO_EMBRYO_IMPLANTATION	-0.70	0.04	0	0	0	not significant
7782	REACTOME_TRAF6_MEDIATED_NF_KB_ACTIVATION	-0.70	0.04	0	0	0	not significant
7783	GO_MEDIATOR_COMPLEX	-0.70	0.03	0	0	0	not significant
7784	GO_NEGATIVE_REGULATION_OF_PEPTIDYL_TYROSINE_PHOSPHORYLAT	-0.70	0.03	0	0	0	not significant
7785	GO_REGULATION_OF_MITOCHONDRIAL_MEMBRANE_PERMEABILITY_INV(	-0.70	0.03	0	0	0	not significant
7786	REACTOME_ACTIVATION_OF_ATR_IN_RESPONSE_TO_REPLICATION_STF	-0.70	0.03	0	0	0	not significant
7787	SIG_BCR_SIGNALING_PATHWAY	-0.70	0.03	0	0	0	not significant
7788	GO_DENDRITIC_SPINE_DEVELOPMENT	-0.70	0.02	0	0	0	not significant
7789	GO_IRON_ION_HOMEOSTASIS	-0.70	0.02	0	0	0	not significant
7790	GO_HIGH_DENSITY_LIPOPROTEIN_PARTICLE_CLEARANCE	-0.69	0.09	0	0	0	not significant
7791	GO_NEUROTROPHIN_TRK_RECEPTOR_BINDING	-0.69	0.09	0	0	0	not significant
7792	REACTOME_TIGHT_JUNCTION_INTERACTIONS	-0.69	0.09	0	0	0	not significant
7793	GO_CDC73_PAF1_COMPLEX	-0.69	0.08	0	0	0	not significant
7794	GO_ENDONUCLEOLYTIC_CLEAVAGE_IN_ITS1_TO_SEPARATE_SSU_RRNA	-0.69	0.08	0	0	0	not significant
7795	GO_HISTONE_H3_K9_DIMETHYLATION	-0.69	0.08	0	0	0	not significant
7796	GO_REGULATION_OF_ODONTOGENESIS	-0.69	0.08	0	0	0	not significant
7797	GO_TOLL_LIKE_RECEPTOR_2_SIGNALING_PATHWAY	-0.69	0.08	0	0	0	not significant
7798	GO_XYLULOSE_5_PHOSPHATE_BIOSYNTHETIC_PROCESS	-0.69	0.08	0	0	0	not significant
7799	MIKKELSEN_PLURIPOTENT_STATE_UP	-0.69	0.08	0	0	0	not significant
7800	REACTOME_REGULATION_OF_PTEN_LOCALIZATION	-0.69	0.08	0	0	0	not significant
7801	GO_NEGATIVE_REGULATION_OF_JUN_KINASE_ACTIVITY	-0.69	0.07	0	0	0	not significant
7802	GO_OLIGOSACCHARYL_TRANSFERASE_ACTIVITY	-0.69	0.07	0	0	0	not significant
7803	GO_REGULATION_OF_SMAD_PROTEIN_SIGNAL_TRANSDUCTION	-0.69	0.07	0	0	0	not significant
7804	GO_SEQUESTERING_OF_TRIGLYCERIDE	-0.69	0.07	0	0	0	not significant
7805	GO_SOMATIC_DIVERSIFICATION_OF_T_CELL_RECEPTOR_GENES	-0.69	0.07	0	0	0	not significant
7806	WU_ALZHEIMER_DISEASE_DN	-0.69	0.07	0	0	0	not significant
7807	CHR18Q22	-0.69	0.06	0	0	0	not significant
7808	GO_PIGMENT_GRANULE_LOCALIZATION	-0.69	0.06	0	0	0	not significant
7809	GO_PLATELET_DENSE_GRANULE	-0.69	0.06	0	0	0	not significant
7810	GO_REGULATION_OF_AUTOPHAGOSOME_MATURATION	-0.69	0.06	0	0	0	not significant
7811	LU_TUMOR_VASCULATURE_UP	-0.69	0.06	0	0	0	not significant
7812	REACTOME_TGF_BETA_RECEPTOR_SIGNALING_IN_EMT_EPITHELIAL_TC	-0.69	0.06	0	0	0	not significant
7813	GO_CARBOXYPEPTIDASE_ACTIVITY	-0.69	0.05	0	0	0	not significant
7814	GO_POSITIVE_REGULATION_OF_NEUROBLAST_PROLIFERATION	-0.69	0.05	0	0	0	not significant
7815	CHR20P12	-0.69	0.04	0	0	0	not significant
7816	GO_NUCLEAR_HETEROCHROMATIN	-0.69	0.04	0	0	0	not significant
7817	GO_REGULATION_OF_FATTY_ACID_METABOLIC_PROCESS	-0.69	0.04	0	0	0	not significant
7818	GO_AP_TYPE_MEMBRANE_COAT_ADAPTOR_COMPLEX	-0.69	0.03	0	0	0	not significant
7819	GO_MITOCHONDRIAL_OUTER_MEMBRANE_PERMEABILIZATION	-0.69	0.03	0	0	0	not significant
7820	GO_POSITIVE_REGULATION_OF_NUCLEOCYTOPLASMIC_TRANSPORT	-0.69	0.02	0	0	0	not significant
7821	GO_REGULATION_OF_HISTONE_MODIFICATION	-0.69	0.01	0	0	0	not significant
7822	GO_REGULATION_OF_POSTSYNAPSE_ORGANIZATION	-0.69	0.01	0	0	0	not significant
7823	GO_INTRACELLULAR_STEROID_HORMONE_RECEPTOR_SIGNALING_PATI	-0.69	0.00	0	0	0	not significant
7824	GO_NEGATIVE_REGULATION_OF_NEURON_DEATH	-0.69	0.00	0	0	0	not significant
7825	GO_NUCLEAR_HORMONE_RECEPTOR_BINDING	-0.69	0.00	0	0	0	not significant
7826	GO_RAB_GTPASE_BINDING	-0.69	0.00	0	0	0	not significant
7827	REACTOME_REGULATION_OF_TP53_ACTIVITY	-0.69	0.00	0	0	0	not significant
7828	ITO_PTTG1_TARGETS_UP	-0.68	0.09	0	0	0	not significant
7829	REACTOME_FORMATION_OF_XYLULOSE_5_PHOSPHATE	-0.68	0.08	0	0	0	not significant
7830	CHR8P22	-0.68	0.07	0	0	0	not significant
7831	GO_ATG1_ULK1_KINASE_COMPLEX	-0.68	0.07	0	0	0	not significant
7832	GO_ER_ASSOCIATED_MISFOLDED_PROTEIN_CATABOLIC_PROCESS	-0.68	0.07	0	0	0	not significant
7833	GO_ESCRT_COMPLEX_DISASSEMBLY	-0.68	0.07	0	0	0	not significant
7834	GO_MITOCHONDRIAL_DNA_REPAIR	-0.68	0.07	0	0	0	not significant
7835	GO_NEUROTRANSMITTER_CATABOLIC_PROCESS	-0.68	0.07	0	0	0	not significant
7836	GO_NODE_OF_RANVIER	-0.68	0.07	0	0	0	not significant

7837	GO_PHAGOLYSOSOME_ASSEMBLY	-0.68	0.07	0	0	0	not significant
7838	GO_PROLINE_RICH_REGION_BINDING	-0.68	0.07	0	0	0	not significant
7839	GO_RESPONSE_TO_MACROPHAGE_COLONY_STIMULATING_FACTOR	-0.68	0.07	0	0	0	not significant
7840	GO_TRNA_TRANSCRIPTION	-0.68	0.07	0	0	0	not significant
7841	TERAMOTO_OPN_TARGETS_CLUSTER_4	-0.68	0.07	0	0	0	not significant
7842	CHR18Q23	-0.68	0.06	0	0	0	not significant
7843	GO_RNA_POLYMERASE_I_REGULATORY_REGION_DNA_BINDING	-0.68	0.06	0	0	0	not significant
7844	GO_VASCULAR_SMOOTH_MUSCLE_CELL_DIFFERENTIATION	-0.68	0.06	0	0	0	not significant
7845	HEIDENBLAD_AMPLIFIED_IN_SOFT_TISSUE_CANCER	-0.68	0.06	0	0	0	not significant
7846	BENPORATH_ES_2	-0.68	0.05	0	0	0	not significant
7847	GO_MYOTUBE_CELL_DEVELOPMENT	-0.68	0.05	0	0	0	not significant
7848	GO_PHOTOTRANSDUCTION_VISIBLE_LIGHT	-0.68	0.05	0	0	0	not significant
7849	GO_PROTEIN_NEDDYLATION	-0.68	0.05	0	0	0	not significant
7850	HOEGERKORP_CD44_TARGETS_DIRECT_UP	-0.68	0.05	0	0	0	not significant
7851	WOOD_EBV_EBNA1_TARGETS_DN	-0.68	0.05	0	0	0	not significant
7852	BIOCARTA_ALK_PATHWAY	-0.68	0.04	0	0	0	not significant
7853	BIOCARTA_IL1R_PATHWAY	-0.68	0.04	0	0	0	not significant
7854	CHANGOLKAR_H2AFY_TARGETS_DN	-0.68	0.04	0	0	0	not significant
7855	GO_NEGATIVE_REGULATION_OF_EXTRINSIC_APOPTOTIC_SIGNALING_PA	-0.68	0.04	0	0	0	not significant
7856	GO_NUCLEAR_TRANSCRIBED_MRNA_POLY_A_TAIL_SHORTENING	-0.68	0.04	0	0	0	not significant
7857	HASLINGER_B_CLL_WITH_11Q23_DELETION	-0.68	0.04	0	0	0	not significant
7858	GO_AMYLOID_PRECURSOR_PROTEIN_METABOLIC_PROCESS	-0.68	0.03	0	0	0	not significant
7859	GO_MISMATCH_REPAIR	-0.68	0.03	0	0	0	not significant
7860	GO_WATER_HOMEOSTASIS	-0.68	0.03	0	0	0	not significant
7861	KEGG_INOSITOL_PHOSPHATE_METABOLISM	-0.68	0.03	0	0	0	not significant
7862	REACTOME_DNA_DAMAGE_BYPASS	-0.68	0.02	0	0	0	not significant
7863	GO_FILOPODIUM	-0.68	0.01	0	0	0	not significant
7864	GO_PROTEIN_METHYLATION	-0.68	0.00	0	0	0	not significant
7865	GO_VESICLE_MEDIATED_TRANSPORT_IN_SYNAPSE	-0.68	0.00	0	0	0	not significant
7866	REACTOME_MEIOSIS	-0.68	0.00	0	0	0	not significant
7867	REACTOME_VITAMIN_C_ASCORBATE_METABOLISM	-0.67	0.09	0	0	0	not significant
7868	GO_REGULATION_OF_HISTONE_H2B_UBIQUITINATION	-0.67	0.08	0	0	0	not significant
7869	GO_SATELLITE_DNA_BINDING	-0.67	0.08	0	0	0	not significant
7870	FUJIWARA_PARK2_HEPATOCYTE_PROLIFERATION_DN	-0.67	0.07	0	0	0	not significant
7871	GO_DEOXYRIBONUCLEOSIDE_TRIPHOSPHATE_CATABOLIC_PROCESS	-0.67	0.07	0	0	0	not significant
7872	GO_NEGATIVE_REGULATION_OF_KIDNEY_DEVELOPMENT	-0.67	0.07	0	0	0	not significant
7873	GO_POSITIVE_REGULATION_OF_INTRACELLULAR_ESTROGEN_RECEPTC	-0.67	0.07	0	0	0	not significant
7874	GO_PROTEIN_DESUMOYLATION	-0.67	0.07	0	0	0	not significant
7875	GO_REGULATION_OF_BROWN_FAT_CELL_DIFFERENTIATION	-0.67	0.07	0	0	0	not significant
7876	GO_REGULATION_OF_GLUCONEOGENESIS_BY_REGULATION_OF_TRANS	-0.67	0.07	0	0	0	not significant
7877	GO_SNRNA_TRANSCRIPTION_BY_RNA_POLYMERASE_III	-0.67	0.07	0	0	0	not significant
7878	REACTOME_SODIUM_CALCIUM_EXCHANGERS	-0.67	0.07	0	0	0	not significant
7879	GO_POSITIVE_REGULATION_OF_ACTIN_CYTOSKELETON_REORGANIZAT	-0.67	0.06	0	0	0	not significant
7880	GO_S_ADENOSYLMETHIONINE_METABOLIC_PROCESS	-0.67	0.06	0	0	0	not significant
7881	GO_STAGA_COMPLEX	-0.67	0.06	0	0	0	not significant
7882	REACTOME_MAPK3_ERK1_ACTIVATION	-0.67	0.06	0	0	0	not significant
7883	REACTOME_WNT5A_DEPENDENT_INTERNALIZATION_OF_FZD2_FZD5_AN	-0.67	0.06	0	0	0	not significant
7884	WOTTON_RUNX_TARGETS_UP	-0.67	0.06	0	0	0	not significant
7885	FRIDMAN_IMMORTALIZATION_DN	-0.67	0.05	0	0	0	not significant
7886	GO_3_UTR_MEDIATED_MRNA_DESTABILIZATION	-0.67	0.05	0	0	0	not significant
7887	GO_LYSINE_ACETYLATED_HISTONE_BINDING	-0.67	0.05	0	0	0	not significant
7888	GO_NUCLEAR_EUCHROMATIN	-0.67	0.05	0	0	0	not significant
7889	GO_REGULATION_OF_NUCLEAR_TRANSCRIBED_MRNA_CATABOLIC_PRC	-0.67	0.05	0	0	0	not significant
7890	OUYANG_PROSTATE_CANCER_PROGRESSION_UP	-0.67	0.05	0	0	0	not significant
7891	WALLACE_JAK2_TARGETS_UP	-0.67	0.05	0	0	0	not significant
7892	CHR1Q41	-0.67	0.04	0	0	0	not significant
7893	GO_LONG_TERM_MEMORY	-0.67	0.04	0	0	0	not significant
7894	STEIN_ESRRA_TARGETS_RESPONSIVE_TO_ESTROGEN_UP	-0.67	0.04	0	0	0	not significant
7895	GO_AXON_REGENERATION	-0.67	0.03	0	0	0	not significant
7896	GO_DNA_REPLICATION_ORIGIN_BINDING	-0.67	0.03	0	0	0	not significant
7897	REACTOME_RESOLUTION_OF_AP_SITES_VIA_THE_MULTIPLE_NUCLEOTI	-0.67	0.03	0	0	0	not significant
7898	GO_MICROTUBULE_POLYMERIZATION_OR_DEPOLYMERIZATION	-0.67	0.01	0	0	0	not significant
7899	TURASHVILI_BREAST_NORMAL_DUCTAL_VS_LOBULAR_UP	-0.67	0.01	0	0	0	not significant
7900	GO_CYTOSOLIC_TRANSPORT	-0.67	0.00	0	0	0	not significant
7901	GO_METHYLTRANSFERASE_COMPLEX	-0.67	0.00	0	0	0	not significant
7902	GO_REGULATION_OF_PROTEIN_MONOUBIQUITINATION	-0.66	0.08	0	0	0	not significant
7903	GO_INDOLE_CONTAINING_COMPOUND_METABOLIC_PROCESS	-0.66	0.07	0	0	0	not significant
7904	GO_SKELETAL_MYOFIBRIL_ASSEMBLY	-0.66	0.07	0	0	0	not significant
7905	GO_MAINTENANCE_OF_DNA_METHYLATION	-0.66	0.06	0	0	0	not significant
7906	GO_PHOSPHORYLATED_CARBOHYDRATE_DEPHOSPHORYLATION	-0.66	0.06	0	0	0	not significant
7907	GO_REGULATION_OF_TRANSLATION_AT_SYNAPSE_MODULATING_SYNAI	-0.66	0.06	0	0	0	not significant
7908	GO_SECRETORY_GRANULE_LOCALIZATION	-0.66	0.06	0	0	0	not significant
7909	GO_CELLULAR_RESPONSE_TO_PH	-0.66	0.05	0	0	0	not significant
7910	GO_CHROMATIN_SILENCING_AT_TELOMERE	-0.66	0.05	0	0	0	not significant
7911	GO_GOLGI_DISASSEMBLY	-0.66	0.05	0	0	0	not significant
7912	GO_NEGATIVE_REGULATION_OF_STEROID_METABOLIC_PROCESS	-0.66	0.05	0	0	0	not significant
7913	GO_POSITIVE_REGULATION_OF_HISTONE_H3_K4_METHYLATION	-0.66	0.05	0	0	0	not significant
7914	GO_PROTEIN_KINASE_B_BINDING	-0.66	0.05	0	0	0	not significant
7915	GO_PYRIMIDINE_RIBONUCLEOSIDE_TRIPHOSPHATE_BIOSYNTHETIC_PRO	-0.66	0.05	0	0	0	not significant
7916	HUMMERICH_BENIGN_SKIN_TUMOR_UP	-0.66	0.05	0	0	0	not significant
7917	AMIT_SERUM_RESPONSE_40_MCF10A	-0.66	0.04	0	0	0	not significant
7918	GO_GALACTOSYLTRANSFERASE_ACTIVITY	-0.66	0.04	0	0	0	not significant
7919	GO_PHOSPHATIDYLINOSITOL_DEPHOSPHORYLATION	-0.66	0.04	0	0	0	not significant
7920	GO_POSITIVE_REGULATION_OF_POSTTRANSCRIPTIONAL_GENE_SILENC	-0.66	0.04	0	0	0	not significant
7921	GO_PROTEIN_HYDROXYLATION	-0.66	0.04	0	0	0	not significant
7922	GO_SMOOTH_MUSCLE_CELL_APOPTOTIC_PROCESS	-0.66	0.04	0	0	0	not significant
7923	PID_IL1_PATHWAY	-0.66	0.04	0	0	0	not significant
7924	REACTOME_PCNA_DEPENDENT_LONG_PATCH_BASE_EXCISION_REPAIR	-0.66	0.04	0	0	0	not significant
7925	REACTOME_TICAM1_RIP1_MEDIATED_IKK_COMPLEX_RECRUITMENT	-0.66	0.04	0	0	0	not significant
7926	REACTOME_TRAF6_MEDIATED_IRF7_ACTIVATION	-0.66	0.04	0	0	0	not significant
7927	KEGG_ALDOSTERONE_REGULATED_SODIUM_REABSORPTION	-0.66	0.03	0	0	0	not significant
7928	REACTOME_ENERGY_DEPENDENT_REGULATION_OF_MTOR_BY_LKB1_A	-0.66	0.03	0	0	0	not significant
7929	SIMBULAN_UV_RESPONSE_IMMORTALIZED_DN	-0.66	0.03	0	0	0	not significant
7930	ZEMBUTSU_SENSITIVITY_TO_METHOTREXATE	-0.66	0.03	0	0	0	not significant
7931	GO_TRANSCRIPTION_ELONGATION_FACTOR_COMPLEX	-0.66	0.02	0	0	0	not significant
7932	PID_ATM_PATHWAY	-0.66	0.02	0	0	0	not significant
7933	GO_REGULATION_OF_MAINTENANCE_OF_SISTER_CHROMATID_COHESIC	-0.65	0.08	0	0	0	not significant
7934	GO_INSULIN_BINDING	-0.65	0.06	0	0	0	not significant
7935	GO_METHIONINE_BIOSYNTHETIC_PROCESS	-0.65	0.06	0	0	0	not significant
7936	GO_NEGATIVE_REGULATION_OF_HISTONE_H3_K4_METHYLATION	-0.65	0.06	0	0	0	not significant
7937	GO_NEGATIVE_REGULATION_OF_MICROTUBULE_POLYMERIZATION	-0.65	0.06	0	0	0	not significant
7938	GO_REGULATION_OF_PHOSPHATIDYLINOSITOL_DEPHOSPHORYLATION	-0.65	0.06	0	0	0	not significant
7939	GO_SPHINGOMYELIN_BIOSYNTHETIC_PROCESS	-0.65	0.06	0	0	0	not significant
7940	REACTOME_FRS2_MEDIATED_ACTIVATION	-0.65	0.06	0	0	0	not significant
7941	GO_INTERLEUKIN_6_BIOSYNTHETIC_PROCESS	-0.65	0.05	0	0	0	not significant
7942	GO_LATE_ENDOSOME_TO_VACUOLE_TRANSPORT_VIA_MULTIVESICULA	-0.65	0.05	0	0	0	not significant
7943	GO_MONOUBIQUITINATED_PROTEIN_DEUBIQUITINATION	-0.65	0.05	0	0	0	not significant
7944	GO_POSITIVE_REGULATION_OF_STRIATED_MUSCLE_CONTRACTION	-0.65	0.05	0	0	0	not significant
7945	GO_CHROMATIN_DISASSEMBLY	-0.65	0.04	0	0	0	not significant

7946	GO_CLATHRIN_COAT_ASSEMBLY	-0.65	0.04	0	0	0	not significant
7947	GO_DEOXYRIBONUCLEOTIDE_BIOSYNTHETIC_PROCESS	-0.65	0.04	0	0	0	not significant
7948	GO_ESCRT_III_COMPLEX	-0.65	0.04	0	0	0	not significant
7949	GO_FILOPODIUM_MEMBRANE	-0.65	0.04	0	0	0	not significant
7950	GO_MICROTUBULE_PLUS_END_BINDING	-0.65	0.03	0	0	0	not significant
7951	GO_RESPONSE_TO_CATECHOLAMINE	-0.65	0.03	0	0	0	not significant
7952	GO_SOLUTE_PROTON_ANTIPORTER_ACTIVITY	-0.65	0.03	0	0	0	not significant
7953	NUNODA_RESPONSE_TO_DASATINIB_IMATINIB_UP	-0.65	0.03	0	0	0	not significant
7954	PID_AMB2_NEUTROPHILS_PATHWAY	-0.65	0.03	0	0	0	not significant
7955	TAVOR_CEBPA_TARGETS_DN	-0.65	0.03	0	0	0	not significant
7956	GO_MITOCHONDRIAL_FISSION	-0.65	0.02	0	0	0	not significant
7957	GO_NF_KAPPAB_BINDING	-0.65	0.02	0	0	0	not significant
7958	PID_REELIN_PATHWAY	-0.65	0.02	0	0	0	not significant
7959	GAZDA_DIAMOND_BLACKFAN_ANEMIA_PROGENITOR_DN	-0.65	0.01	0	0	0	not significant
7960	GO_ORGANELLE_TRANSPORT_ALONG_MICROTUBULE	-0.65	0.01	0	0	0	not significant
7961	GO_PIGMENTATION	-0.65	0.01	0	0	0	not significant
7962	DAZARD_RESPONSE_TO_UV_NHEK_DN	-0.65	0.00	0	0	0	not significant
7963	REACTOME_PHOSPHOLIPID_METABOLISM	-0.65	0.00	0	0	0	not significant
7964	GO_NEGATIVE_REGULATION_OF_INTERLEUKIN_12_PRODUCTION	-0.64	0.06	0	0	0	not significant
7965	GO_PHOSPHATE_ION_TRANSMEMBRANE_TRANSPORTER_ACTIVITY	-0.64	0.06	0	0	0	not significant
7966	GO_REGULATION_OF_RIBONUCLEOPROTEIN_COMPLEX_LOCALIZATION	-0.64	0.06	0	0	0	not significant
7967	GO_ENDOPLASMIC_RETICULUM_LOCALIZATION	-0.64	0.05	0	0	0	not significant
7968	GO_NEGATIVE_REGULATION_OF_SMOOTH_MUSCLE_CONTRACTION	-0.64	0.05	0	0	0	not significant
7969	GO_NEGATIVE_REGULATION_OF_TYROSINE_PHOSPHORYLATION_OF_S	-0.64	0.05	0	0	0	not significant
7970	GO POSITIVE REGULATION OF RESPONSE TO REACTIVE OXYGEN SI	-0.64	0.05	0	0	0	not significant
7971	GO_ZINC_ION_IMPORT_ACROSS_PLASMA_MEMBRANE	-0.64	0.05	0	0	0	not significant
7972	GO_ZYMOGEN_GRANULE	-0.64	0.05	0	0	0	not significant
7973	HUMMERICH_MALIGNANT_SKIN_TUMOR_DN	-0.64	0.05	0	0	0	not significant
7974	REACTOME_FORMATION_OF_FIBRIN_CLOT_CLOTTING_CASCADE	-0.64	0.05	0	0	0	not significant
7975	FIGUEROA_AML_METHYLATION_CLUSTER_6_DN	-0.64	0.04	0	0	0	not significant
7976	GO_CELLULAR_RESPONSE_TO_VITAMIN_D	-0.64	0.04	0	0	0	not significant
7977	GO_EPITHELIAL_CELL_MORPHOGENESIS	-0.64	0.04	0	0	0	not significant
7978	GO_G_RICH_STRAND_TELOMERIC_DNA_BINDING	-0.64	0.04	0	0	0	not significant
7979	GO_NEGATIVE_REGULATION_OF_CIRCADIAN_RHYTHM	-0.64	0.04	0	0	0	not significant
7980	GO_POSITIVE_REGULATION_OF_EPIDERMAL_CELL_DIFFERENTIATION	-0.64	0.04	0	0	0	not significant
7981	GO_SRP_DEPENDENT_COTRANSLATIONAL_PROTEIN_TARGETING_TO_M	-0.64	0.04	0	0	0	not significant
7982	GO_VITAMIN_D_RECEPTOR_BINDING	-0.64	0.04	0	0	0	not significant
7983	GO_ACTIN_CORTICAL_PATCH	-0.64	0.03	0	0	0	not significant
7984	GO_RESPONSE_TO_MISFOLDED_PROTEIN	-0.64	0.03	0	0	0	not significant
7985	REACTOME_AKT_PHOSPHORYLATES_TARGETS_IN_THE_CYTOSOL	-0.64	0.03	0	0	0	not significant
7986	CHR14Q23	-0.64	0.02	0	0	0	not significant
7987	GO_PHOSPHATIDIC_ACID_BIOSYNTHETIC_PROCESS	-0.64	0.02	0	0	0	not significant
7988	GARGALOVIC_RESPONSE_TO_OXIDIZED_PHOSPHOLIPIDS_BLACK_UP	-0.64	0.01	0	0	0	not significant
7989	REACTOME_TRANSCRIPTIONAL_REGULATION_BY_MECP2	-0.64	0.01	0	0	0	not significant
7990	GO_REPLICATION_FORK	-0.64	0.00	0	0	0	not significant
7991	GO_DEATH_INDUCING_SIGNALING_COMPLEX_ASSEMBLY	-0.63	0.06	0	0	0	not significant
7992	GO_NEGATIVE_REGULATION_OF_MYELINATION	-0.63	0.06	0	0	0	not significant
7993	GO_POSITIVE_REGULATION_OF_DEOXYRIBONUCLEASE_ACTIVITY	-0.63	0.06	0	0	0	not significant
7994	REACTOME_PREGNENOLONE_BIOSYNTHESIS	-0.63	0.06	0	0	0	not significant
7995	DER_IFN_GAMMA_RESPONSE_DN	-0.63	0.05	0	0	0	not significant
7996	GO_LYSOSOMAL_PROTEIN_CATABOLIC_PROCESS	-0.63	0.05	0	0	0	not significant
7997	GO_POSITIVE_REGULATION_OF_SYNAPTIC_VESICLE_RECYCLING	-0.63	0.05	0	0	0	not significant
7998	GO_REGULATION_OF_NON_MOTILE_CILIUM_ASSEMBLY	-0.63	0.05	0	0	0	not significant
7999	GO_RNA_POLYMERASE_III_TYPE_3_PROMOTER_DNA_BINDING	-0.63	0.05	0	0	0	not significant
8000	PID_P38_GAMMA_DELTA_PATHWAY	-0.63	0.05	0	0	0	not significant
8001	SCHAEFFER_PROSTATE_DEVELOPMENT_AND_CANCER_BOX1_DN	-0.63	0.05	0	0	0	not significant
8002	BIOCARTA_BARR_MAPK_PATHWAY	-0.63	0.04	0	0	0	not significant
8003	GO ENDOSOME TO LYSOSOME TRANSPORT VIA MULTIVESICULAR BO	-0.63	0.04	0	0	0	not significant
8004	GO_MEIOTIC_CHROMOSOME_CONDENSATION	-0.63	0.04	0	0	0	not significant
8005	GO_POSITIVE_REGULATION_OF_NUCLEASE_ACTIVITY	-0.63	0.04	0	0	0	not significant
8006	PUJANA_BREAST_CANCER_WITH_BRCA1_MUTATED_DN	-0.63	0.04	0	0	0	not significant
8007	REACTOME_ROLE_OF_ABL_IN_ROBO_SLIT_SIGNALING	-0.63	0.04	0	0	0	not significant
8008	ZHENG_IL22_SIGNALING_DN	-0.63	0.04	0	0	0	not significant
8009	FLECHNER_BIOPSY_KIDNEY_TRANSPLANT_OK_VS_DONOR_DN	-0.63	0.03	0	0	0	not significant
8010	GO_CELLULAR_PROTEIN_CONTAINING_COMPLEX_LOCALIZATION	-0.63	0.03	0	0	0	not significant
8011	GO_NUCLEAR_INCLUSION_BODY	-0.63	0.03	0	0	0	not significant
8012	GO_TETRAHYDROFOLATE_METABOLIC_PROCESS	-0.63	0.03	0	0	0	not significant
8013	HU_ANGIOGENESIS_UP	-0.63	0.03	0	0	0	not significant
8014	ROVERSI_GLIOMA_LOH_REGIONS	-0.63	0.03	0	0	0	not significant
8015	GO_MICROTUBULE_END	-0.63	0.02	0	0	0	not significant
8016	GO_PROTEIN_DNA_COMPLEX_DISASSEMBLY	-0.63	0.02	0	0	0	not significant
8017	GO_RAB_GUANYL_NUCLEOTIDE_EXCHANGE_FACTOR_ACTIVITY	-0.63	0.02	0	0	0	not significant
8018	REACTOME_ACTIVATION_OF_BH3_ONLY_PROTEINS	-0.63	0.02	0	0	0	not significant
8019	GO_NEGATIVE_REGULATION_OF_MUSCLE_CELL_DIFFERENTIATION	-0.63	0.01	0	0	0	not significant
8020	KEGG_GLIOMA	-0.63	0.01	0	0	0	not significant
8021	GO_NEGATIVE_REGULATION_OF_NEURON_APOPTOTIC_PROCESS	-0.63	0.00	0	0	0	not significant
8022	GO_POSITIVE_REGULATION_OF_AUTOPHAGY	-0.63	0.00	0	0	0	not significant
8023	GO_ANGIOGENESIS_INVOLVED_IN_WOUND_HEALING	-0.62	0.05	0	0	0	not significant
8024	GO_AUTOPHAGY_OF_PEROXISOME	-0.62	0.05	0	0	0	not significant
8025	GO_GLYCOPROTEIN_COMPLEX	-0.62	0.05	0	0	0	not significant
8026	GO_NUCLEOSIDE_TRIPHOSPHATE_CATABOLIC_PROCESS	-0.62	0.05	0	0	0	not significant
8027	GO_TETRAHYDROFOLATE_INTERCONVERSION	-0.62	0.05	0	0	0	not significant
8028	REACTOME_DOWNREGULATION_OF_ERBB4_SIGNALING	-0.62	0.05	0	0	0	not significant
8029	GO_CONTRACTILE_RING	-0.62	0.04	0	0	0	not significant
8030	GO_POLYOL_TRANSPORT	-0.62	0.04	0	0	0	not significant
8031	GO_REGULATION_OF_DENDRITIC_SPINE_MAINTENANCE	-0.62	0.04	0	0	0	not significant
8032	GO_SMC5_SMC6_COMPLEX	-0.62	0.04	0	0	0	not significant
8033	GO_SYNAPTIC_VESICLE_ENDOSOMAL_PROCESSING	-0.62	0.04	0	0	0	not significant
8034	BIOCARTA_ERK5_PATHWAY	-0.62	0.03	0	0	0	not significant
8035	DING_LUNG_CANCER_MUTATED_SIGNIFICANTLY	-0.62	0.03	0	0	0	not significant
8036	GO POSITIVE REGULATION OF CELL ADHESION MEDIATED BY INTEG	-0.62	0.03	0	0	0	not significant
8037	GO_RDNA_BINDING	-0.62	0.03	0	0	0	not significant
8038	GO_TRANSCRIPTION_EXPORT_COMPLEX	-0.62	0.03	0	0	0	not significant
8039	BIOCARTA_CTCF_PATHWAY	-0.62	0.02	0	0	0	not significant
8040	GO_NEGATIVE_REGULATION_OF_FIBROBLAST_PROLIFERATION	-0.62	0.02	0	0	0	not significant
8041	CHR10Q26	-0.62	0.01	0	0	0	not significant
8042	GO_MUSCLE_CELL_APOPTOTIC_PROCESS	-0.62	0.01	0	0	0	not significant
8043	GO_OVARIAN_FOLLICLE_DEVELOPMENT	-0.62	0.01	0	0	0	not significant
8044	REACTOME_APOPTOTIC_EXECUTION_PHASE	-0.62	0.01	0	0	0	not significant
8045	GO DOLICHYL PHOSPHATE MANNOSE PROTEIN MANNOSYLTRANSFER.	-0.61	0.05	0	0	0	not significant
8046	GO_L_ASCORBIC_ACID_METABOLIC_PROCESS	-0.61	0.05	0	0	0	not significant
8047	REACTOME_TRAIL_SIGNALING	-0.61	0.05	0	0	0	not significant
8048	GO_LATE_ENDOSOME_TO_LYSOSOME_TRANSPORT	-0.61	0.04	0	0	0	not significant
8049	GO NEGATIVE REGULATION OF DNA DAMAGE RESPONSE SIGNAL TR	-0.61	0.04	0	0	0	not significant
8050	GO_NUCLEAR_MEMBRANE_ORGANIZATION	-0.61	0.04	0	0	0	not significant
8051	CHRXQ23	-0.61	0.03	0	0	0	not significant
8052	GO_LATERAL_ELEMENT	-0.61	0.03	0	0	0	not significant
8053	GO_NUCLEOSOME_POSITIONING	-0.61	0.03	0	0	0	not significant
8054	KEGG_ETHER_LIPID_METABOLISM	-0.61	0.03	0	0	0	not significant

8055	REACTOME_ACTIVATION_OF_IRF3_IRF7_MEDIATED_BY_TBK1_IKK_EPSIL	-0.61	0.03	0	0	0	not significant
8056	TURJANSKI_MAPK7_TARGETS	-0.61	0.03	0	0	0	not significant
8057	WENDT_COHESIN_TARGETS_UP	-0.61	0.02	0	0	0	not significant
8058	GO_CUL4_RING_E3_UBIQUITIN_LIGASE_COMPLEX	-0.61	0.01	0	0	0	not significant
8059	GO_NEGATIVE_REGULATION_OF_DNA_REPLICATION	-0.61	0.01	0	0	0	not significant
8060	GO_THYROID_HORMONE_RECEPTOR_BINDING	-0.61	0.01	0	0	0	not significant
8061	REACTOME_TAK1_ACTIVATES_NFKB_BY_PHOSPHORYLATION_AND_ACT	-0.61	0.01	0	0	0	not significant
8062	BIOCARTA_MAPK_PATHWAY	-0.61	0.00	0	0	0	not significant
8063	GO_ACTIN_FILAMENT_SEVERING	-0.60	0.05	0	0	0	not significant
8064	GO_GROWTH_HORMONE_SECRETION	-0.60	0.04	0	0	0	not significant
8065	GO_MANGANESE_ION_TRANSPORT	-0.60	0.04	0	0	0	not significant
8066	GO_POSITIVE_REGULATION_OF_PROTEIN_LOCALIZATION_TO_EARLY_EI	-0.60	0.04	0	0	0	not significant
8067	GO_POTASSIUM_ION_ANTIPORTER_ACTIVITY	-0.60	0.04	0	0	0	not significant
8068	GO_1_PHOSPHATIDYLINOSITOL_4_PHOSPHATE_3_KINASE_ACTIVITY	-0.60	0.03	0	0	0	not significant
8069	GO_CEREBRAL_CORTEX_NEURON_DIFFERENTIATION	-0.60	0.03	0	0	0	not significant
8070	GO_CONDENSIN_COMPLEX	-0.60	0.03	0	0	0	not significant
8071	GO_INTRINSIC_COMPONENT_OF_PEROXISOMAL_MEMBRANE	-0.60	0.03	0	0	0	not significant
8072	GO_MALE_GERM_CELL_NUCLEUS	-0.60	0.03	0	0	0	not significant
8073	GO_NUCLEAR_LAMINA	-0.60	0.03	0	0	0	not significant
8074	GO_RAL_GTPASE_BINDING	-0.60	0.03	0	0	0	not significant
8075	GO_REGULATION_OF_PLASMA_MEMBRANE_ORGANIZATION	-0.60	0.03	0	0	0	not significant
8076	GO_RESPONSE_TO_CELL_CYCLE_CHECKPOINT_SIGNALING	-0.60	0.03	0	0	0	not significant
8077	REACTOME_GLYCOGEN_SYNTHESIS	-0.60	0.03	0	0	0	not significant
8078	ZEMBUTSU_SENSITIVITY_TO_FLUOROURACIL	-0.60	0.03	0	0	0	not significant
8079	GARGALOVIC_RESPONSE_TO_OXIDIZED_PHOSPHOLIPIDS_SALMON_DN	-0.60	0.02	0	0	0	not significant
8080	GO_CATECHOLAMINE_METABOLIC_PROCESS	-0.60	0.02	0	0	0	not significant
8081	GO_MAINTENANCE_OF_CELL_POLARITY	-0.60	0.02	0	0	0	not significant
8082	GO_NEURON_PROJECTION_REGENERATION	-0.60	0.02	0	0	0	not significant
8083	GO_PERICENTRIC_HETEROCHROMATIN	-0.60	0.02	0	0	0	not significant
8084	GO_VIRAL_BUDDING_VIA_HOST_ESCRT_COMPLEX	-0.60	0.02	0	0	0	not significant
8085	REACTOME_IKK_COMPLEX_RECRUITMENT_MEDIATED_BY_RIP1	-0.60	0.02	0	0	0	not significant
8086	BIOCARTA_BAD_PATHWAY	-0.60	0.01	0	0	0	not significant
8087	BIOCARTA_CREB_PATHWAY	-0.60	0.01	0	0	0	not significant
8088	GO_PHOSPHOLIPID_DEPHOSPHORYLATION	-0.60	0.01	0	0	0	not significant
8089	GO_REGULATION_OF_CENTROSOME_DUPLICATION	-0.60	0.01	0	0	0	not significant
8090	GO_CHROMATIN_REMODELING	-0.60	0.00	0	0	0	not significant
8091	REACTOME_RAB_GEFS_EXCHANGE_GTP_FOR_GDP_ON_RABS	-0.60	0.00	0	0	0	not significant
8092	GO_NEGATIVE_REGULATION_OF_DNA_ENDOREDUPLICATION	-0.59	0.05	0	0	0	not significant
8093	BIOCARTA_VITCB_PATHWAY	-0.59	0.03	0	0	0	not significant
8094	GO_MACROMOLECULE_DEPALMITOYLATION	-0.59	0.03	0	0	0	not significant
8095	GO_NEGATIVE_REGULATION_OF_VOLTAGE_GATED_CALCIUM_CHANNEL	-0.59	0.03	0	0	0	not significant
8096	JI_METASTASIS_REPRESSED_BY_STK11	-0.59	0.03	0	0	0	not significant
8097	GO_MITOTIC_RECOMBINATION	-0.59	0.02	0	0	0	not significant
8098	GO_PHOSPHOTRANSFERASE_ACTIVITY_FOR_OTHER_SUBSTITUTED_PH	-0.59	0.02	0	0	0	not significant
8099	GO_THYROID_GLAND_DEVELOPMENT	-0.59	0.02	0	0	0	not significant
8100	REACTOME_MISMATCH_REPAIR	-0.59	0.02	0	0	0	not significant
8101	GO_SELECTIVE_AUTOPHAGY	-0.59	0.01	0	0	0	not significant
8102	DAZARD_UV_RESPONSE_CLUSTER_G6	-0.59	0.00	0	0	0	not significant
8103	GO_GLYCOLIPID_TRANSPORT	-0.58	0.04	0	0	0	not significant
8104	HEIDENBLAD_AMPLIFIED_IN_BONE_CANCER	-0.58	0.04	0	0	0	not significant
8105	GO_LYSOSOMAL_LUMEN_ACIDIFICATION	-0.58	0.03	0	0	0	not significant
8106	GO_PLATELET_DENSE_GRANULE_ORGANIZATION	-0.58	0.03	0	0	0	not significant
8107	GO_POSITIVE_REGULATION_OF_NEUROTRANSMITTER_SECRETION	-0.58	0.03	0	0	0	not significant
8108	GO_POSITIVE_REGULATION_OF_PHAGOCYTOSIS_ENGULFMENT	-0.58	0.03	0	0	0	not significant
8109	GO_PRIMARY_AMINO_COMPOUND_METABOLIC_PROCESS	-0.58	0.03	0	0	0	not significant
8110	GO_PROTEIN_LOCALIZATION_TO_EARLY_ENDOSOME	-0.58	0.03	0	0	0	not significant
8111	GO_REGULATION_OF_MRNA_EXPORT_FROM_NUCLEUS	-0.58	0.03	0	0	0	not significant
8112	GO_RETINAL_GANGLION_CELL_AXON_GUIDANCE	-0.58	0.03	0	0	0	not significant
8113	GO_RNA_POLYMERASE_III_REGULATORY_REGION_DNA_BINDING	-0.58	0.03	0	0	0	not significant
8114	SINGH_KRAS_DEPENDENCY_SIGNATURE_	-0.58	0.03	0	0	0	not significant
8115	BIOCARTA_MYOSIN_PATHWAY	-0.58	0.02	0	0	0	not significant
8116	CHR13Q22	-0.58	0.02	0	0	0	not significant
8117	GO_ESTABLISHMENT_OF_ENDOTHELIAL_INTESTINAL_BARRIER	-0.58	0.02	0	0	0	not significant
8118	GO_INTERLEUKIN_12_BIOSYNTHETIC_PROCESS	-0.58	0.02	0	0	0	not significant
8119	GO_PALMITOYL_PROTEIN_HYDROLASE_ACTIVITY	-0.58	0.02	0	0	0	not significant
8120	GO_POSITIVE_REGULATION_OF_CELLULAR_RESPONSE_TO_INSULIN_ST	-0.58	0.02	0	0	0	not significant
8121	GO_POSITIVE_REGULATION_OF_EARLY_ENDOSOME_TO_LATE_ENDOSO	-0.58	0.02	0	0	0	not significant
8122	GO_POSITIVE_REGULATION_OF_NEUROTRANSMITTER_TRANSPORT	-0.58	0.02	0	0	0	not significant
8123	GO_PREFOLDIN_COMPLEX	-0.58	0.02	0	0	0	not significant
8124	GO_PRODUCTION_OF_SIRNA_INVOLVED_IN_RNA_INTERFERENCE	-0.58	0.02	0	0	0	not significant
8125	NIKOLSKY_BREAST_CANCER_12Q24_AMPLICON	-0.58	0.02	0	0	0	not significant
8126	REACTOME_SUMO_IS_PROTEOLYTICALLY_PROCESSED	-0.58	0.02	0	0	0	not significant
8127	BIOCARTA_CXCR4_PATHWAY	-0.58	0.01	0	0	0	not significant
8128	BIOCARTA_GSK3_PATHWAY	-0.58	0.01	0	0	0	not significant
8129	ZHANG_ADIPOGENESIS_BY_BMP7	-0.58	0.01	0	0	0	not significant
8130	ZHONG_RESPONSE_TO_AZACITIDINE_AND_TSA_DN	-0.58	0.00	0	0	0	not significant
8131	GO_CARDIOLIPIN_BINDING	-0.57	0.03	0	0	0	not significant
8132	GO_CUL4B_RING_E3_UBIQUITIN_LIGASE_COMPLEX	-0.57	0.03	0	0	0	not significant
8133	GO_EIF2ALPHA_PHOSPHORYLATION_IN_RESPONSE_TO_ENDOPLASMIC_	-0.57	0.03	0	0	0	not significant
8134	GO_ORGANIC_ACID_SODIUM_SYMPORTER_ACTIVITY	-0.57	0.03	0	0	0	not significant
8135	GO_S_ADENOSYLMETHIONINE_CYCLE	-0.57	0.03	0	0	0	not significant
8136	CHR8Q11	-0.57	0.02	0	0	0	not significant
8137	GO_ACTIN_FILAMENT_BASED_TRANSPORT	-0.57	0.02	0	0	0	not significant
8138	GO_C4_DICARBOXYLATE_TRANSMEMBRANE_TRANSPORTER_ACTIVITY	-0.57	0.02	0	0	0	not significant
8139	GO_DNA_STRAND_ELONGATION_INVOLVED_IN_DNA_REPLICATION	-0.57	0.02	0	0	0	not significant
8140	GO_NEGATIVE_REGULATION_OF_CYSTEINE_TYPE_ENDOPEPTIDASE_AC	-0.57	0.02	0	0	0	not significant
8141	GO_REGULATION_OF_SYNCYTIUM_FORMATION_BY_PLASMA_MEMBRANI	-0.57	0.02	0	0	0	not significant
8142	IIZUKA_LIVER_CANCER_PROGRESSION_L1_G1_DN	-0.57	0.02	0	0	0	not significant
8143	SHIRAISHI_PLZF_TARGETS_DN	-0.57	0.02	0	0	0	not significant
8144	GO_DYNEIN_COMPLEX	-0.57	0.01	0	0	0	not significant
8145	GO_ERROR_PRONE_TRANSLESION_SYNTHESIS	-0.57	0.01	0	0	0	not significant
8146	GO_PROTEIN_PHOSPHORYLATED_AMINO_ACID_BINDING	-0.57	0.01	0	0	0	not significant
8147	GO_REGULATION_OF_MRNA_POLYADENYLATION	-0.57	0.01	0	0	0	not significant
8148	HEDENFALK_BREAST_CANCER_BRACX_DN	-0.57	0.01	0	0	0	not significant
8149	KYNG_WERNER_SYNDROM_UP	-0.57	0.01	0	0	0	not significant
8150	PID_INTEGRIN_A4B1_PATHWAY	-0.57	0.01	0	0	0	not significant
8151	GO_ANDROGEN_RECEPTOR_SIGNALING_PATHWAY	-0.57	0.00	0	0	0	not significant
8152	GO_TRANSCRIPTIONAL_REPRESSOR_COMPLEX	-0.57	0.00	0	0	0	not significant
8153	GO_ESTROGEN_METABOLIC_PROCESS	-0.56	0.03	0	0	0	not significant
8154	GO_SPLICEOSOMAL_CONFORMATIONAL_CHANGES_TO_GENERATE_CAT	-0.56	0.03	0	0	0	not significant
8155	GO_TRANSCRIPTION_FACTOR_AP_1_COMPLEX	-0.56	0.03	0	0	0	not significant
8156	REACTOME_MET_ACTIVATES_RAP1_AND_RAC1	-0.56	0.03	0	0	0	not significant
8157	CHR11P14	-0.56	0.02	0	0	0	not significant
8158	CHR4Q34	-0.56	0.02	0	0	0	not significant
8159	GO_ASTRAL_MICROTUBULE_ORGANIZATION	-0.56	0.02	0	0	0	not significant
8160	GO_REGULATION_OF_HETEROTYPIC_CELL_CELL_ADHESION	-0.56	0.02	0	0	0	not significant
8161	GO_VERY_LONG_CHAIN_FATTY_ACID_METABOLIC_PROCESS	-0.56	0.02	0	0	0	not significant
8162	INAMURA_LUNG_CANCER_SCC_SUBTYPES_DN	-0.56	0.02	0	0	0	not significant
8163	LIU_CMYB_TARGETS_DN	-0.56	0.02	0	0	0	not significant

8164	REACTOME_PHASE_2_PLATEAU_PHASE	-0.56	0.02	0	0	0	not significant
8165	BIOCARTA_CERAMIDE_PATHWAY	-0.56	0.01	0	0	0	not significant
8166	GO_REGULATION_OF_MYOBLAST_FUSION	-0.56	0.01	0	0	0	not significant
8167	GO_TELOMERE_MAINTENANCE_VIA_SEMI_CONSERVATIVE_REPLICATION	-0.56	0.01	0	0	0	not significant
8168	MOREIRA_RESPONSE_TO_TSA_DN	-0.56	0.01	0	0	0	not significant
8169	REACTOME_THE_NLRP3_INFLAMMASOME	-0.56	0.01	0	0	0	not significant
8170	GO_DNA_INSERTION_OR_DELETION_BINDING	-0.55	0.03	0	0	0	not significant
8171	GO_NEGATIVE_REGULATION_OF_HEART_RATE	-0.55	0.02	0	0	0	not significant
8172	GO_NEGATIVE_REGULATION_OF_VIRAL_ENTRY_INTO_HOST_CELL	-0.55	0.02	0	0	0	not significant
8173	GO_POSITIVE_REGULATION_OF_TOLL_LIKE_RECEPTOR_3_SIGNALING_I	-0.55	0.02	0	0	0	not significant
8174	GO_REGULATION_OF_MYOSIN_LIGHT_CHAIN_PHOSPHATASE_ACTIVITY	-0.55	0.02	0	0	0	not significant
8175	GO_SEQUESTERING_OF_METAL_ION	-0.55	0.02	0	0	0	not significant
8176	GO_SKELETAL_MUSCLE_ACETYLCHOLINE_GATED_CHANNEL_CLUSTERI	-0.55	0.02	0	0	0	not significant
8177	PID_THROMBIN_PAR4_PATHWAY	-0.55	0.02	0	0	0	not significant
8178	REACTOME_DEGRADATION_OF_CYSTEINE_AND_HOMOCYSTEINE	-0.55	0.02	0	0	0	not significant
8179	REACTOME_ZINC_TRANSPORTERS	-0.55	0.02	0	0	0	not significant
8180	GO_NEGATIVE_REGULATION_OF_VIRAL_TRANSCRIPTION	-0.55	0.01	0	0	0	not significant
8181	GO_VASCULAR_SMOOTH_MUSCLE_CONTRACTION	-0.55	0.01	0	0	0	not significant
8182	REACTOME_SYNTHESIS_OF_PC	-0.55	0.01	0	0	0	not significant
8183	REACTOME_TELOMERE_C_STRAND_LAGGING_STRAND_SYNTHESIS	-0.55	0.01	0	0	0	not significant
8184	REACTOME_UPTAKE_AND_ACTIONS_OF_BACTERIAL_TOXINS	-0.55	0.01	0	0	0	not significant
8185	DACOSTA_UV_RESPONSE_VIA_ERCC3_TTD_DN	-0.55	0.00	0	0	0	not significant
8186	KEGG_ENDOMETRIAL_CANCER	-0.55	0.00	0	0	0	not significant
8187	GO_MONOCYTE_CHEMOTACTIC_PROTEIN_1_PRODUCTION	-0.54	0.03	0	0	0	not significant
8188	GO_REGULATION_OF_XENOPHAGY	-0.54	0.03	0	0	0	not significant
8189	GO_COPI_COATED_VESICLE_BUDDING	-0.54	0.02	0	0	0	not significant
8190	GO_COPI_VESICLE_COAT	-0.54	0.02	0	0	0	not significant
8191	GO_GAMMA_AMINOBUTYRIC_ACID_SECRETION	-0.54	0.02	0	0	0	not significant
8192	GO_MEIOTIC_COHESIN_COMPLEX	-0.54	0.02	0	0	0	not significant
8193	GO_OXIDOREDUCTASE_ACTIVITY_ACTING_ON_THE_ALDEHYDE_OR_OX(	-0.54	0.02	0	0	0	not significant
8194	GO_PURINE_NUCLEOSIDE_CATABOLIC_PROCESS	-0.54	0.02	0	0	0	not significant
8195	GO_REGULATION_OF_NEUROTRANSMITTER_RECEPTOR_LOCALIZATION	-0.54	0.02	0	0	0	not significant
8196	SUZUKI_AMPLIFIED_IN_ORAL_CANCER	-0.54	0.02	0	0	0	not significant
8197	GO_ENDOPLASMIC_RETICULUM_TUBULAR_NETWORK	-0.54	0.01	0	0	0	not significant
8198	GO_INTRACELLULAR_DISTRIBUTION_OF_MITOCHONDRIA	-0.54	0.01	0	0	0	not significant
8199	GO_NEGATIVE_REGULATION_OF_FATTY_ACID_BIOSYNTHETIC_PROCES	-0.54	0.01	0	0	0	not significant
8200	GO_PYRIMIDINE_NUCLEOSIDE_MONOPHOSPHATE_BIOSYNTHETIC_PROC	-0.54	0.01	0	0	0	not significant
8201	GO_REGULATION_OF_TRANSLATIONAL_INITIATION_BY_EIF2_ALPHA_PH(	-0.54	0.01	0	0	0	not significant
8202	MATZUK_SPERMATOGONIA	-0.54	0.01	0	0	0	not significant
8203	REACTOME_TRANSLESION_SYNTHESIS_BY_POLK	-0.54	0.01	0	0	0	not significant
8204	GO_BLOC_COMPLEX	-0.54	0.00	0	0	0	not significant
8205	GO_REGULATION_OF_DNA_TEMPLATED_TRANSCRIPTION_ELONGATION	-0.54	0.00	0	0	0	not significant
8206	BIOCARTA_BARRESTIN_PATHWAY	-0.53	0.02	0	0	0	not significant
8207	GO_GAMMA_AMINOBUTYRIC_ACID_TRANSPORT	-0.53	0.02	0	0	0	not significant
8208	GO_HISTONE_H3_K27_TRIMETHYLATION	-0.53	0.02	0	0	0	not significant
8209	GO_MSL_COMPLEX	-0.53	0.02	0	0	0	not significant
8210	GO_NEGATIVE_REGULATION_OF_PROTEIN_K63_LINKED_UBIQUITINATION	-0.53	0.02	0	0	0	not significant
8211	GO_SHELTERIN_COMPLEX	-0.53	0.02	0	0	0	not significant
8212	GO_SPANNING_COMPONENT_OF_MEMBRANE	-0.53	0.02	0	0	0	not significant
8213	GO_SUMO_SPECIFIC_PROTEASE_ACTIVITY	-0.53	0.02	0	0	0	not significant
8214	RUAN_RESPONSE_TO_TROGLITAZONE_DN	-0.53	0.02	0	0	0	not significant
8215	BENPORATH_ES_CORE_NINE	-0.53	0.01	0	0	0	not significant
8216	GO_DEOXYRIBONUCLEOTIDE_BINDING	-0.53	0.01	0	0	0	not significant
8217	GO_ESTABLISHMENT_OF_PIGMENT_GRANULE_LOCALIZATION	-0.53	0.01	0	0	0	not significant
8218	GO_LEWY_BODY	-0.53	0.01	0	0	0	not significant
8219	GO_POSITIVE_REGULATION_OF_AUTOPHAGOSOME_ASSEMBLY	-0.53	0.01	0	0	0	not significant
8220	GO_POSITIVE_REGULATION_OF_CENTROSOME_CYCLE	-0.53	0.01	0	0	0	not significant
8221	GO_POSITIVE_REGULATION_OF_MYELINATION	-0.53	0.01	0	0	0	not significant
8222	GO_VASCULAR_ASSOCIATED_SMOOTH_MUSCLE_CELL_MIGRATION	-0.53	0.01	0	0	0	not significant
8223	GO_CELLULAR_RESPONSE_TO_VITAMIN	-0.53	0.00	0	0	0	not significant
8224	GO_POSITIVE_REGULATION_OF_CELL_CYCLE_G2_M_PHASE_TRANSITIO	-0.53	0.00	0	0	0	not significant
8225	GO_TRANSITION_METAL_ION_TRANSMEMBRANE_TRANSPORTER_ACTIVI	-0.53	0.00	0	0	0	not significant
8226	GO_RESPONSE_TO_LIGHT_INTENSITY	-0.52	0.02	0	0	0	not significant
8227	REACTOME_MITOCHONDRIAL_FATTY_ACID_BETA_OXIDATION_OF_UNSA	-0.52	0.02	0	0	0	not significant
8228	CHR4Q35	-0.52	0.01	0	0	0	not significant
8229	GAUSSMANN_MLL_AF4_FUSION_TARGETS_G_DN	-0.52	0.01	0	0	0	not significant
8230	GO_ACTIVATION_OF_NF_KAPPAB_INDUCING_KINASE_ACTIVITY	-0.52	0.01	0	0	0	not significant
8231	GO_ZINC_ION_TRANSMEMBRANE_TRANSPORTER_ACTIVITY	-0.52	0.01	0	0	0	not significant
8232	REACTOME_REGULATION_OF_IFNG_SIGNALING	-0.52	0.01	0	0	0	not significant
8233	GO_2_OXOGLUTARATE_DEPENDENT_DIOXYGENASE_ACTIVITY	-0.52	0.00	0	0	0	not significant
8234	CHR4P13	-0.51	0.02	0	0	0	not significant
8235	GO_TRANSCRIPTION_FACTOR_TFIIIB_COMPLEX	-0.51	0.02	0	0	0	not significant
8236	BIOCARTA_BCELLSURVIVAL_PATHWAY	-0.51	0.01	0	0	0	not significant
8237	GO_CARDIAC_MYOFIBRIL_ASSEMBLY	-0.51	0.01	0	0	0	not significant
8238	GO_COMMON_MYELOID_PROGENITOR_CELL_PROLIFERATION	-0.51	0.01	0	0	0	not significant
8239	GO_NEGATIVE_REGULATION_OF_PLATELET_DERIVED_GROWTH_FACT(	-0.51	0.01	0	0	0	not significant
8240	GO_REGULATION_OF_HEXOKINASE_ACTIVITY	-0.51	0.01	0	0	0	not significant
8241	GO_RESPONSE_TO_LEAD_ION	-0.51	0.01	0	0	0	not significant
8242	GO_TYPE_B_PANCREATIC_CELL_PROLIFERATION	-0.51	0.01	0	0	0	not significant
8243	TAKEDA_TARGETS_OF_NUP98_HOXA9_FUSION_3D_DN	-0.51	0.01	0	0	0	not significant
8244	CHR5P13	-0.51	0.00	0	0	0	not significant
8245	GENTILE_RESPONSE_CLUSTER_D3	-0.51	0.00	0	0	0	not significant
8246	GO_FOLIC_ACID_BINDING	-0.51	0.00	0	0	0	not significant
8247	GO_NEGATIVE_REGULATION_OF_PROTEIN_KINASE_B_SIGNALING	-0.51	0.00	0	0	0	not significant
8248	PID_TRAIL_PATHWAY	-0.51	0.00	0	0	0	not significant
8249	REACTOME_RECOGNITION_OF_DNA_DAMAGE_BY_PCNA_CONTAINING_R	-0.51	0.00	0	0	0	not significant
8250	GO_1_ACYLGLYCEROPHOSPHOCHOLINE_O_ACYLTRANSFERASE_ACTIVI	-0.50	0.02	0	0	0	not significant
8251	BIOCARTA_CK1_PATHWAY	-0.50	0.01	0	0	0	not significant
8252	BIOCARTA_RNAPOL3_PATHWAY	-0.50	0.01	0	0	0	not significant
8253	GO_CD4_RECEPTOR_BINDING	-0.50	0.01	0	0	0	not significant
8254	GO_MRE11_COMPLEX	-0.50	0.01	0	0	0	not significant
8255	GO_NEURON_INTRINSIC_APOPTOTIC_SIGNALING_PATHWAY_IN_RESPON	-0.50	0.01	0	0	0	not significant
8256	GO_PEPTIDYL_PROLINE_4_DIOXYGENASE_ACTIVITY	-0.50	0.01	0	0	0	not significant
8257	GO_PEPTIDYL_PROLINE_HYDROXYLATION_TO_4_HYDROXY_L_PROLINE	-0.50	0.01	0	0	0	not significant
8258	GO_POSITIVE_REGULATION_OF_PROTEIN_HOMODIMERIZATION_ACTIVIT	-0.50	0.01	0	0	0	not significant
8259	GO_REGULATION_OF_NEURON_PROJECTION_REGENERATION	-0.50	0.01	0	0	0	not significant
8260	GO_SUMO_POLYMER_BINDING	-0.50	0.01	0	0	0	not significant
8261	NAGY_STAGA_COMPONENTS_HUMAN	-0.50	0.01	0	0	0	not significant
8262	WEBER_METHYLATED_HCP_IN_SPERM_UP	-0.50	0.01	0	0	0	not significant
8263	FINETTI_BREAST_CANCERS_KINOME_GRAY	-0.50	0.00	0	0	0	not significant
8264	GO_ENDODERMAL_CELL_FATE_COMMITMENT	-0.50	0.00	0	0	0	not significant
8265	GO_POLY_A_PLUS_MRNA_EXPORT_FROM_NUCLEUS	-0.50	0.00	0	0	0	not significant
8266	GO_UV_DAMAGE_EXCISION_REPAIR	-0.50	0.00	0	0	0	not significant
8267	CHR14Q31	-0.49	0.01	0	0	0	not significant
8268	GO_NEGATIVE_REGULATION_OF_CELL_SIZE	-0.49	0.01	0	0	0	not significant
8269	GO_NEUROPEPTIDE_RECEPTOR_ACTIVITY	-0.49	0.01	0	0	0	not significant
8270	GO_PANTOTHENATE_METABOLIC_PROCESS	-0.49	0.01	0	0	0	not significant
8271	SCHAEFFER_PROSTATE_DEVELOPMENT_AND_CANCER_BOX4_UP	-0.49	0.01	0	0	0	not significant
8272	GO_ENDOSOME_TRANSPORT_VIA_MULTIVESICULAR_BODY_SORTING_P.	-0.49	0.00	0	0	0	not significant

8273	GO_PHOSPHATIDYLINOSITOL_PHOSPHATE_KINASE_ACTIVITY	-0.49	0.00	0	0	0	not significant
8274	REACTOME_CLASS_I_PEROXISOMAL_MEMBRANE_PROTEIN_IMPORT	-0.49	0.00	0	0	0	not significant
8275	GO_RESPONSE_TO_DEFENSES_OF_OTHER_ORGANISM_INVOLVED_IN_S	-0.48	0.01	0	0	0	not significant
8276	BIOCARTA_PLC_PATHWAY	-0.48	0.00	0	0	0	not significant
8277	BOQUEST_STEM_CELL_CULTURED_VS_FRESH_DN	-0.48	0.00	0	0	0	not significant
8278	GARGALOVIC_RESPONSE_TO_OXIDIZED_PHOSPHOLIPIDS_RED_DN	-0.48	0.00	0	0	0	not significant
8279	GO_DNA_STRAND_ELONGATION	-0.48	0.00	0	0	0	not significant
8280	GO_PROTEIN_LOCALIZATION_TO_CILIARY_MEMBRANE	-0.48	0.00	0	0	0	not significant
8281	GO_REGULATION_OF_RESPONSE_TO_FOOD	-0.48	0.00	0	0	0	not significant
8282	GO_SEX_CHROMOSOME	-0.48	0.00	0	0	0	not significant
8283	GO_INTRACILIARY_TRANSPORT_PARTICLE	-0.47	0.01	0	0	0	not significant
8284	GO_MITOCHONDRIAL_TRICARBOXYLIC_ACID_CYCLE_ENZYME_COMPLEX	-0.47	0.01	0	0	0	not significant
8285	REACTOME_RUNX2_REGULATES_GENES_INVOLVED_IN_CELL_MIGRATIO	-0.47	0.01	0	0	0	not significant
8286	GO_GENITALIA_MORPHOGENESIS	-0.47	0.00	0	0	0	not significant
8287	GO_MULTI_ORGANISM_BEHAVIOR	-0.47	0.00	0	0	0	not significant
8288	GO_PEPTIDYL_PROLINE_DIOXYGENASE_ACTIVITY	-0.47	0.00	0	0	0	not significant
8289	GO_PHOSPHATIDYLINOSITOL_3_KINASE_ACTIVITY	-0.47	0.00	0	0	0	not significant
8290	GO_POSITIVE_REGULATION_OF_HISTONE_H4_ACETYLATION	-0.47	0.00	0	0	0	not significant
8291	REACTOME_INFLAMMASOMES	-0.47	0.00	0	0	0	not significant
8292	GO_3_5_DNA_HELICASE_ACTIVITY	-0.46	0.00	0	0	0	not significant
8293	GO_REGULATION_OF_CENTRIOLE_REPLICATION	-0.46	0.00	0	0	0	not significant
8294	GO_REGULATION_OF_MDA_5_SIGNALING_PATHWAY	-0.46	0.00	0	0	0	not significant
8295	GO_CHROMATIN_SILENCING_COMPLEX	-0.44	0.00	0	0	0	not significant
8296	GO_REGULATION_OF_B_CELL_RECEPTOR_SIGNALING_PATHWAY	-0.44	0.00	0	0	0	not significant
8297	GO_REGULATION_OF_NEURON_MATURATION	-0.44	0.00	0	0	0	not significant
8298	PID_ERB_GENOMIC_PATHWAY	-0.44	0.00	0	0	0	not significant
8299	REACTOME_TBC_RABGAPS	-0.44	0.00	0	0	0	not significant
8300	GO_POSITIVE_REGULATION_OF_KERATINOCYTE_DIFFERENTIATION	-0.43	0.00	0	0	0	not significant
8301	GO_REGULATION_OF_APPETITE	-0.43	0.00	0	0	0	not significant
8302	GO_GABA_ERGIC_SYNAPSE	-0.42	0.00	0	0	0	not significant
8303	GO_LYSOPHOSPHOLIPASE_ACTIVITY	-0.42	0.00	0	0	0	not significant
8304	GO_NEGATIVE_REGULATION_OF_PROTEIN_POLYUBIQUITINATION	-0.42	0.00	0	0	0	not significant
8305	GO_MCM_COMPLEX	-0.41	0.00	0	0	0	not significant
8306	GO_NEGATIVE_REGULATION_OF_MYOSIN_LIGHT_CHAIN_PHOSPHATASE_	-0.41	0.00	0	0	0	not significant
8307	REACTOME_ESTABLISHMENT_OF_SISTER_CHROMATID_COHESION	-0.41	0.00	0	0	0	not significant
8308	REACTOME_PROCESSIVE_SYNTHESIS_ON_THE_C_STRAND_OF_THE_TE	-0.41	0.00	0	0	0	not significant
8309	GO_PROTEIN_HEXAMERIZATION	-0.40	0.00	0	0	0	not significant
8310	GO_TUBULAR_ENDOSOME	-0.40	0.00	0	0	0	not significant
8311	GO_METHIONINE_METABOLIC_PROCESS	-0.39	0.00	0	0	0	not significant
8312	GO_NUCLEAR_OUTER_MEMBRANE	-0.39	0.00	0	0	0	not significant
8313	REACTOME_DNA_STRAND_ELONGATION	-0.36	0.00	0	0	0	not significant
8314	GO_PROTEIN_LOCALIZATION_TO_CELL_JUNCTION	-0.35	0.00	0	0	0	not significant
8315	GO_MAINTENANCE_OF_SISTER_CHROMATID_COHESION	0.34	0.00	0	0	0	not significant
8316	GO_MISMATCH_REPAIR_COMPLEX_BINDING	0.38	0.00	0	0	0	not significant
8317	GO_CARD_DOMAIN_BINDING	0.39	0.00	0	0	0	not significant
8318	GO_HISTONE_METHYLTRANSFERASE_ACTIVITY_H3_K27_SPECIFIC_	0.40	0.00	0	0	0	not significant
8319	GO_MITOTIC_CHROMOSOME_CONDENSATION	0.40	0.00	0	0	0	not significant
8320	GO_NEGATIVE_REGULATION_OF_MUSCLE_ADAPTATION	0.41	0.00	0	0	0	not significant
8321	GO_POSITIVE_REGULATION_OF_AUTOPHAGY_OF_MITOCHONDRION	0.41	0.00	0	0	0	not significant
8322	GO_REGULATION_OF_MITOCHONDRIAL_FUSION	0.41	0.00	0	0	0	not significant
8323	GO_MESSENGER_RIBONUCLEOPROTEIN_COMPLEX	0.42	0.00	0	0	0	not significant
8324	GO_MUTSALPHA_COMPLEX_BINDING	0.42	0.00	0	0	0	not significant
8325	GO_NEGATIVE_REGULATION_OF_HISTONE_H3_K9_TRIMETHYLATION	0.42	0.00	0	0	0	not significant
8326	GO_OLIGOSACCHARIDE_BIOSYNTHETIC_PROCESS	0.42	0.00	0	0	0	not significant
8327	GO_PYRIMIDINE_NUCLEOTIDE_SALVAGE	0.42	0.00	0	0	0	not significant
8328	GO_REGULATION_OF_CILIUM_BEAT_FREQUENCY_INVOLVED_IN_CILIARY	0.42	0.00	0	0	0	not significant
8329	GO_REGULATION_OF_PROTEIN_LOCALIZATION_TO_CILIUM	0.42	0.00	0	0	0	not significant
8330	GO_SULFATE_TRANSPORT	0.42	0.00	0	0	0	not significant
8331	GO_BRCA1_A_COMPLEX	0.43	0.00	0	0	0	not significant
8332	GO_PHOTORECEPTOR_DISC_MEMBRANE	0.43	0.00	0	0	0	not significant
8333	GO_REGULATION_OF_MITOTIC_RECOMBINATION	0.43	0.00	0	0	0	not significant
8334	REACTOME_APOPTOTIC_CLEAVAGE_OF_CELLULAR_PROTEINS	0.43	0.00	0	0	0	not significant
8335	GO_INTRASPECIES_INTERACTION_BETWEEN_ORGANISMS	0.44	0.00	0	0	0	not significant
8336	GO_NEGATIVE_REGULATION_OF_HISTONE_METHYLATION	0.44	0.00	0	0	0	not significant
8337	ONGUSAHA_BRCA1_TARGETS_DN	0.44	0.00	0	0	0	not significant
8338	BILD_CTNNB1_ONCOGENIC_SIGNATURE	0.45	0.00	0	0	0	not significant
8339	GO_ACYL_COA_DEHYDROGENASE_ACTIVITY	0.45	0.00	0	0	0	not significant
8340	GO_ATP_DEPENDENT_MICROTUBULE_MOTOR_ACTIVITY_MINUS_END_DI	0.45	0.00	0	0	0	not significant
8341	GO_CELL_CELL_ADHESION_INVOLVED_IN_GASTRULATION	0.45	0.00	0	0	0	not significant
8342	GO_CHOLINE_TRANSPORT	0.45	0.00	0	0	0	not significant
8343	GO_INSULIN_RESPONSIVE_COMPARTMENT	0.45	0.00	0	0	0	not significant
8344	GO_NEGATIVE_REGULATION_OF_HISTONE_MODIFICATION	0.45	0.00	0	0	0	not significant
8345	GO_REPLICATION_FORK_PROCESSING	0.45	0.00	0	0	0	not significant
8346	GO_3_PHOSPHOADENOSINE_5_PHOSPHOSULFATE_METABOLIC_PROCES	0.46	0.01	0	0	0	not significant
8347	GO_OXIDOREDUCTASE_ACTIVITY_ACTING_ON_SINGLE_DONORS_WITH_I	0.46	0.01	0	0	0	not significant
8348	GO_HETEROCHROMATIN_ORGANIZATION	0.46	0.00	0	0	0	not significant
8349	GO_REGULATION_OF_INTERLEUKIN_2_SECRETION	0.46	0.00	0	0	0	not significant
8350	GO_REGULATION_OF_WATER_LOSS_VIA_SKIN	0.46	0.00	0	0	0	not significant
8351	GO_REPLISOME	0.46	0.00	0	0	0	not significant
8352	GO_SYNTAXIN_1_BINDING	0.46	0.00	0	0	0	not significant
8353	GO_UDP_N_ACETYLGLUCOSAMINE_METABOLIC_PROCESS	0.46	0.00	0	0	0	not significant
8354	REACTOME_ATTACHMENT_OF_GPI_ANCHOR_TO_UPAR	0.46	0.00	0	0	0	not significant
8355	REACTOME_PECAM1_INTERACTIONS	0.46	0.00	0	0	0	not significant
8356	GO_AUDITORY_RECEPTOR_CELL_MORPHOGENESIS	0.47	0.01	0	0	0	not significant
8357	GO_CENTROMERIC_DNA_BINDING	0.47	0.01	0	0	0	not significant
8358	GO_CIRCADIAN_SLEEP_WAKE_CYCLE	0.47	0.01	0	0	0	not significant
8359	GO_PLATELET_ALPHA_GRANULE_MEMBRANE	0.47	0.01	0	0	0	not significant
8360	GO_POSITIVE_REGULATION_OF_GLUCOKINASE_ACTIVITY	0.47	0.00	0	0	0	not significant
8361	GO_POSITIVE_REGULATION_OF_HEXOKINASE_ACTIVITY	0.47	0.00	0	0	0	not significant
8362	GO_SULFATE_TRANSMEMBRANE_TRANSPORTER_ACTIVITY	0.47	0.00	0	0	0	not significant
8363	GO_SYMMETRIC_SYNAPSE	0.47	0.00	0	0	0	not significant
8364	ELLWOOD_MYC_TARGETS_UP	0.48	0.01	0	0	0	not significant
8365	GO_ASYMMETRIC_STEM_CELL_DIVISION	0.48	0.01	0	0	0	not significant
8366	GO_CARDIOLIPIN_METABOLIC_PROCESS	0.48	0.01	0	0	0	not significant
8367	GO_HISTONE_METHYLTRANSFERASE_ACTIVITY_H3_K4_SPECIFIC_	0.48	0.01	0	0	0	not significant
8368	GO_CELL_CELL_JUNCTION_MAINTENANCE	0.48	0.00	0	0	0	not significant
8369	GO_NEGATIVE_REGULATION_OF_VIRAL_RELEASE_FROM_HOST_CELL	0.48	0.00	0	0	0	not significant
8370	GO_REGULATION_OF_HISTONE_H3_K9_TRIMETHYLATION	0.48	0.00	0	0	0	not significant
8371	GO_REGULATION_OF_PROTEIN_POLYUBIQUITINATION	0.48	0.00	0	0	0	not significant
8372	GO_CUL4A_RING_E3_UBIQUITIN_LIGASE_COMPLEX	0.49	0.01	0	0	0	not significant
8373	GO_FUCOSYLTRANSFERASE_ACTIVITY	0.49	0.01	0	0	0	not significant
8374	GO_MIRNA_METABOLIC_PROCESS	0.49	0.01	0	0	0	not significant
8375	GO_POSITIVE_REGULATION_OF_EXTRACELLULAR_MATRIX_ORGANIZATI	0.49	0.01	0	0	0	not significant
8376	GO_PROTEIN_O_LINKED_MANNOSYLATION	0.49	0.01	0	0	0	not significant
8377	GO_FLEMMING_BODY	0.49	0.00	0	0	0	not significant
8378	GO_HEPARAN_SULFATE_PROTEOGLYCAN_METABOLIC_PROCESS	0.49	0.00	0	0	0	not significant
8379	GO_NEGATIVE_REGULATION_OF_MRNA_3_END_PROCESSING	0.49	0.00	0	0	0	not significant
8380	REACTOME_GAP_FILLING_DNA_REPAIR_SYNTHESIS_AND_LIGATION_IN_(	0.49	0.00	0	0	0	not significant
8381	REACTOME_LAGGING_STRAND_SYNTHESIS	0.49	0.00	0	0	0	not significant

8382	BIOCARTA_SAM68_PATHWAY	0.50	0.01	0	0	0	not significant
8383	GO_BETA_2_ADRENERGIC_RECEPTOR_BINDING	0.50	0.01	0	0	0	not significant
8384	GO_CAVEOLIN_MEDIATED_ENDOCYTOSIS	0.50	0.01	0	0	0	not significant
8385	GO_NEURON_FATE_SPECIFICATION	0.50	0.01	0	0	0	not significant
8386	GO_PERINUCLEAR_ENDOPLASMIC_RETICULUM	0.50	0.01	0	0	0	not significant
8387	GO_PROTEIN_LOCALIZATION_TO_ENDOSOME	0.50	0.01	0	0	0	not significant
8388	GO_TELOMERIC_LOOP_DISASSEMBLY	0.50	0.01	0	0	0	not significant
8389	GO_XENOPHAGY	0.50	0.01	0	0	0	not significant
8390	REACTOME_BETA_OXIDATION_OF_OCTANOYL_COA_TO_HEXANOYL_CO	0.50	0.01	0	0	0	not significant
8391	REACTOME_COHESIN_LOADING_ONTO_CHROMATIN	0.50	0.01	0	0	0	not significant
8392	REACTOME_VITAMIN_D_CALCIFEROL_METABOLISM	0.50	0.01	0	0	0	not significant
8393	CHR13Q34	0.50	0.00	0	0	0	not significant
8394	GO_R_SMAD_BINDING	0.50	0.00	0	0	0	not significant
8395	GO_AMINO_ACID_SALVAGE	0.51	0.02	0	0	0	not significant
8396	GO_ATP_DEPENDENT_3_5_DNA_HELICASE_ACTIVITY	0.51	0.01	0	0	0	not significant
8397	GO_AUDITORY_RECEPTOR_CELL_STEREOCILIUM_ORGANIZATION	0.51	0.01	0	0	0	not significant
8398	GO_L_METHIONINE_SALVAGE_FROM_METHYLTHIOADENOSINE	0.51	0.01	0	0	0	not significant
8399	GO_POSITIVE_REGULATION_OF_AUTOPHAGOSOME_MATURATION	0.51	0.01	0	0	0	not significant
8400	GO_PURINE_DEOXYRIBONUCLEOTIDE_CATABOLIC_PROCESS	0.51	0.01	0	0	0	not significant
8401	GO_REGULATION_OF_FERTILIZATION	0.51	0.01	0	0	0	not significant
8402	GO_REGULATION_OF_HISTONE_H3_K9_METHYLATION	0.51	0.01	0	0	0	not significant
8403	REACTOME_NEGATIVE_FEEDBACK_REGULATION_OF_MAPK_PATHWAY	0.51	0.01	0	0	0	not significant
8404	REACTOME_PROTON_COUPLED_MONOCARBOXYLATE_TRANSPORT	0.51	0.01	0	0	0	not significant
8405	GO_DNA_DAMAGE_RESPONSE_DETECTION_OF_DNA_DAMAGE	0.51	0.00	0	0	0	not significant
8406	GO_HISTONE_LYSINE_N_METHYLTRANSFERASE_ACTIVITY	0.51	0.00	0	0	0	not significant
8407	GO_PEROXISOMAL_MEMBRANE_TRANSPORT	0.51	0.00	0	0	0	not significant
8408	PID_ERBB1_RECEPTOR_PROXIMAL_PATHWAY	0.51	0.00	0	0	0	not significant
8409	GO_D1_DOPAMINE_RECEPTOR_BINDING	0.52	0.02	0	0	0	not significant
8410	GO_NEGATIVE_REGULATION_OF_EXTRACELLULAR_MATRIX_ORGANIZAT	0.52	0.02	0	0	0	not significant
8411	GO_REGULATION_OF_CELL_CELL_ADHESION_MEDIATED_BY_INTEGRIN	0.52	0.02	0	0	0	not significant
8412	CHR7Q33	0.52	0.01	0	0	0	not significant
8413	GO_PEPTIDYL_THREONINE_DEPHOSPHORYLATION	0.52	0.01	0	0	0	not significant
8414	GO_POSITIVE_REGULATION_OF_ERBB_SIGNALING_PATHWAY	0.52	0.01	0	0	0	not significant
8415	GO_POSITIVE_REGULATION_OF_MACROPHAGE_MIGRATION	0.52	0.01	0	0	0	not significant
8416	GO_RESPONSE_TO_STIMULUS_INVOLVED_IN_REGULATION_OF_MUSCLE	0.52	0.01	0	0	0	not significant
8417	REACTOME_ALPHA_OXIDATION_OF_PHYTANATE	0.52	0.01	0	0	0	not significant
8418	GO_PHOSPHATIDYLINOSITOL_METABOLIC_PROCESS	0.52	0.00	0	0	0	not significant
8419	LEE_CALORIE_RESTRICTION_NEOCORTEX_UP	0.52	0.00	0	0	0	not significant
8420	SCHEIDEREIT_IKK_TARGETS	0.52	0.00	0	0	0	not significant
8421	GO_GPI_ANCHOR_TRANSAMIDASE_ACTIVITY	0.53	0.02	0	0	0	not significant
8422	GO_RNA_DNA_HYBRID_RIBONUCLEASE_ACTIVITY	0.53	0.02	0	0	0	not significant
8423	ASGHARZADEH_NEUROBLASTOMA_POOR_SURVIVAL_DN	0.53	0.01	0	0	0	not significant
8424	GARGALOVIC_RESPONSE_TO_OXIDIZED_PHOSPHOLIPIDS_YELLOW_UP	0.53	0.01	0	0	0	not significant
8425	GO_DYNEIN_HEAVY_CHAIN_BINDING	0.53	0.01	0	0	0	not significant
8426	GO_ENDONUCLEASE_ACTIVITY_ACTIVE_WITH_EITHER_RIBO_OR_DEOXY	0.53	0.01	0	0	0	not significant
8427	GO_NEGATIVE_REGULATION_OF_TYPE_I_INTERFERON_MEDIATED_SIGN	0.53	0.01	0	0	0	not significant
8428	GO_POSITIVE_REGULATION_OF_TOLERANCE_INDUCTION	0.53	0.01	0	0	0	not significant
8429	GO_SIN3_COMPLEX	0.53	0.01	0	0	0	not significant
8430	GO_TRICARBOXYLIC_ACID_CYCLE_ENZYME_COMPLEX	0.53	0.01	0	0	0	not significant
8431	REACTOME_LEADING_STRAND_SYNTHESIS	0.53	0.01	0	0	0	not significant
8432	REACTOME_PROCESSIVE_SYNTHESIS_ON_THE_LAGGING_STRAND	0.53	0.01	0	0	0	not significant
8433	REACTOME_RETROGRADE_TRANSPORT_AT_THE_TRANS_GOLGI_NETW	0.53	0.00	0	0	0	not significant
8434	GO_PRE_MRNA_3_SPLICE_SITE_BINDING	0.54	0.03	0	0	0	not significant
8435	CHR11Q25	0.54	0.02	0	0	0	not significant
8436	GO_COLLAGEN_METABOLIC_PROCESS	0.54	0.02	0	0	0	not significant
8437	GO_EMBRYONIC_BODY_MORPHOGENESIS	0.54	0.02	0	0	0	not significant
8438	GO_GPI_ANCHOR_TRANSAMIDASE_COMPLEX	0.54	0.02	0	0	0	not significant
8439	GO_MACROPHAGE_CYTOKINE_PRODUCTION	0.54	0.02	0	0	0	not significant
8440	GO_PEPTIDYL_LYSINE_DEACETYLATION	0.54	0.02	0	0	0	not significant
8441	GO_PROTEIN_PHOSPHATASE_1_BINDING	0.54	0.02	0	0	0	not significant
8442	GO_RNA_POLYMERASE_I_PREINITIATION_COMPLEX_ASSEMBLY	0.54	0.02	0	0	0	not significant
8443	MCCABE_HOXC6_TARGETS_UP	0.54	0.02	0	0	0	not significant
8444	GO_HISTONE_H3_K9_TRIMETHYLATION	0.54	0.01	0	0	0	not significant
8445	GO_NEGATIVE_REGULATION_OF_ALCOHOL_BIOSYNTHETIC_PROCESS	0.54	0.01	0	0	0	not significant
8446	GO_REGULATION_OF_EARLY_ENDOSOME_TO_LATE_ENDOSOME_TRAN	0.54	0.01	0	0	0	not significant
8447	REACTOME_BETA_OXIDATION_OF_HEXANOYL_COA_TO_BUTANOYL_CO/	0.54	0.01	0	0	0	not significant
8448	REACTOME_SPRY_REGULATION_OF_FGF_SIGNALING	0.54	0.01	0	0	0	not significant
8449	CHR3Q26	0.54	0.00	0	0	0	not significant
8450	GO_LYSINE_N_METHYLTRANSFERASE_ACTIVITY	0.54	0.00	0	0	0	not significant
8451	NIKOLSKY_MUTATED_AND_AMPLIFIED_IN_BREAST_CANCER	0.54	0.00	0	0	0	not significant
8452	GO_ELONGATOR_HOLOENZYME_COMPLEX	0.55	0.02	0	0	0	not significant
8453	GO_EMBRYONIC_RETINA_MORPHOGENESIS_IN_CAMERA_TYPE_EYE	0.55	0.02	0	0	0	not significant
8454	GO_REGULATION_OF_METHYLATION_DEPENDENT_CHROMATIN_SILENCI	0.55	0.02	0	0	0	not significant
8455	GO_TELOMERIC_D_LOOP_DISASSEMBLY	0.55	0.02	0	0	0	not significant
8456	BIOCARTA_DEATH_PATHWAY	0.55	0.01	0	0	0	not significant
8457	GO_NEGATIVE_REGULATION_OF_TRANSCRIPTION_REGULATORY_REGIC	0.55	0.01	0	0	0	not significant
8458	GO_POSITIVE_REGULATION_OF_NEUROLOGICAL_SYSTEM_PROCESS	0.55	0.01	0	0	0	not significant
8459	GO_REGULATION_OF_HISTONE_H3_K4_METHYLATION	0.55	0.01	0	0	0	not significant
8460	KRAS.50_UP.V1_DN	0.55	0.01	0	0	0	not significant
8461	REACTOME_REGULATION_OF_MECP2_EXPRESSION_AND_ACTIVITY	0.55	0.01	0	0	0	not significant
8462	GO_MORPHOGENESIS_OF_AN_EPITHELIAL_SHEET	0.55	0.00	0	0	0	not significant
8463	GO_PEPTIDE_N_ACETYLTRANSFERASE_ACTIVITY	0.55	0.00	0	0	0	not significant
8464	REACTOME_RAB_REGULATION_OF_TRAFFICKING	0.55	0.00	0	0	0	not significant
8465	GO_CYCLOHYDROLASE_ACTIVITY	0.56	0.03	0	0	0	not significant
8466	GO_NPBAF_COMPLEX	0.56	0.03	0	0	0	not significant
8467	GO_OXIDOREDUCTASE_ACTIVITY_ACTING_ON_THE_CH_CH_GROUP_OF_	0.56	0.03	0	0	0	not significant
8468	LIU_TARGETS_OF_VMYB_VS_CMYB_UP	0.56	0.03	0	0	0	not significant
8469	REACTOME_PI5P_REGULATES_TP53_ACETYLATION	0.56	0.03	0	0	0	not significant
8470	DAVIES_MULTIPLE_MYELOMA_VS_MGUS_UP	0.56	0.02	0	0	0	not significant
8471	GO_ATTACHMENT_OF_GPI_ANCHOR_TO_PROTEIN	0.56	0.02	0	0	0	not significant
8472	GO_CATION_CATION_ANTIPORTER_ACTIVITY	0.56	0.02	0	0	0	not significant
8473	GO_GTPASE_INHIBITOR_ACTIVITY	0.56	0.02	0	0	0	not significant
8474	GO_LARGE_RIBOSOMAL_SUBUNIT_RRNA_BINDING	0.56	0.02	0	0	0	not significant
8475	GO_NEGATIVE_REGULATION_OF_OXIDOREDUCTASE_ACTIVITY	0.56	0.02	0	0	0	not significant
8476	GO_NEUROTRANSMITTER_RECEPTOR_LOCALIZATION_TO_POSTSYNAPT	0.56	0.02	0	0	0	not significant
8477	GO_PHOSPHATIDYLGLYCEROL_BINDING	0.56	0.02	0	0	0	not significant
8478	GO_PHOSPHATIDYLINOSITOL_4_5_BISPHOSPHATE_PHOSPHATASE_ACTI	0.56	0.02	0	0	0	not significant
8479	GO_PHOTORECEPTOR_OUTER_SEGMENT_MEMBRANE	0.56	0.02	0	0	0	not significant
8480	GO_REGULATION_OF_EXTRACELLULAR_MATRIX_ASSEMBLY	0.56	0.02	0	0	0	not significant
8481	REACTOME_CREB_PHOSPHORYLATION	0.56	0.02	0	0	0	not significant
8482	VISALA_AGING_LYMPHOCYTE_DN	0.56	0.02	0	0	0	not significant
8483	ZAIDI_OSTEOBLAST_TRANSCRIPTION_FACTORS	0.56	0.02	0	0	0	not significant
8484	GO_SOLUTE_CATION_ANTIPORTER_ACTIVITY	0.56	0.01	0	0	0	not significant
8485	TOMLINS_PROSTATE_CANCER_UP	0.56	0.01	0	0	0	not significant
8486	ODONNELL_METASTASIS_UP	0.56	0.00	0	0	0	not significant
8487	PID_FANCONI_PATHWAY	0.56	0.00	0	0	0	not significant
8488	GO_MYOSIN_II_COMPLEX	0.57	0.03	0	0	0	not significant
8489	GO_POSITIVE_REGULATION_OF_COAGULATION	0.57	0.03	0	0	0	not significant
8490	NIELSEN_LIPOSARCOMA_DN	0.57	0.03	0	0	0	not significant

8491	REACTOME_DNA_REPLICATION_INITIATION	0.57	0.03	0	0	0	not significant
8492	BIOCARTA_PTDINS_PATHWAY	0.57	0.02	0	0	0	not significant
8493	GO_CYCLIN_CDK_POSITIVE_TRANSCRIPTION_ELONGATION_FACTOR_CO	0.57	0.02	0	0	0	not significant
8494	GO_DECIDUALIZATION	0.57	0.02	0	0	0	not significant
8495	GO_FOUR_WAY_JUNCTION_DNA_BINDING	0.57	0.02	0	0	0	not significant
8496	GO_INTRA_S_DNA_DAMAGE_CHECKPOINT	0.57	0.02	0	0	0	not significant
8497	GO_NEGATIVE_REGULATION_BY_HOST_OF_VIRAL_TRANSCRIPTION	0.57	0.02	0	0	0	not significant
8498	GO_NEGATIVE_REGULATION_OF_BIOMINERAL_TISSUE_DEVELOPMENT	0.57	0.02	0	0	0	not significant
8499	GO_POSITIVE_REGULATION_OF_MYOTUBE_DIFFERENTIATION	0.57	0.02	0	0	0	not significant
8500	GO_PROTEIN_CATABOLIC_PROCESS_IN_THE_VACUOLE	0.57	0.02	0	0	0	not significant
8501	GO_REPLACEMENT_OSSIFICATION	0.57	0.02	0	0	0	not significant
8502	GO_SECONDARY_LYSOSOME	0.57	0.02	0	0	0	not significant
8503	WORSCHECH_TUMOR_REJECTION_UP	0.57	0.02	0	0	0	not significant
8504	CHR9Q32	0.57	0.01	0	0	0	not significant
8505	GO_COPI_COATED_VESICLE	0.57	0.01	0	0	0	not significant
8506	GO_GOLGI_TO_PLASMA_MEMBRANE_TRANSPORT	0.57	0.01	0	0	0	not significant
8507	GO_PIGMENT_GRANULE_ORGANIZATION	0.57	0.01	0	0	0	not significant
8508	GO_ENDOPLASMIC_RETICULUM_ORGANIZATION	0.57	0.00	0	0	0	not significant
8509	GO_APICAL_DENDRITE	0.58	0.03	0	0	0	not significant
8510	GO_APICAL_PROTEIN_LOCALIZATION	0.58	0.03	0	0	0	not significant
8511	GO_BRANCHING_INVOLVED_IN_BLOOD_VESSEL_MORPHOGENESIS	0.58	0.03	0	0	0	not significant
8512	GO_EPOXIDE_HYDROLASE_ACTIVITY	0.58	0.03	0	0	0	not significant
8513	GO_GLIAL_CELL_APOPTOTIC_PROCESS	0.58	0.03	0	0	0	not significant
8514	GO_ISWI_TYPE_COMPLEX	0.58	0.03	0	0	0	not significant
8515	GO_PEROXISOME_PROLIFERATOR_ACTIVATED_RECEPTOR_BINDING	0.58	0.03	0	0	0	not significant
8516	GO_RRNA_BASE_METHYLATION	0.58	0.03	0	0	0	not significant
8517	GO_VESICLE_TETHERING	0.58	0.03	0	0	0	not significant
8518	GU_PDEF_TARGETS_DN	0.58	0.03	0	0	0	not significant
8519	BIOCARTA_HBX_PATHWAY	0.58	0.02	0	0	0	not significant
8520	CHR4Q13	0.58	0.02	0	0	0	not significant
8521	GO_INOSITOL_PHOSPHATE_CATABOLIC_PROCESS	0.58	0.02	0	0	0	not significant
8522	GO_INSULIN_RECEPTOR_SUBSTRATE_BINDING	0.58	0.02	0	0	0	not significant
8523	GO_METHYLATION_DEPENDENT_CHROMATIN_SILENCING	0.58	0.02	0	0	0	not significant
8524	GO_POSITIVE_REGULATION_OF_MUSCLE_CELL_APOPTOTIC_PROCESS	0.58	0.02	0	0	0	not significant
8525	GO_PROTEIN_LIPID_COMPLEX_ASSEMBLY	0.58	0.02	0	0	0	not significant
8526	GO_REGULATION_OF_FATTY_ACID_BETA_OXIDATION	0.58	0.02	0	0	0	not significant
8527	PID_IL8_CXCR1_PATHWAY	0.58	0.02	0	0	0	not significant
8528	CHIARETTI_T_ALL_RELAPSE_PROGNOSIS	0.58	0.01	0	0	0	not significant
8529	FLECHNER_PBL_KIDNEY_TRANSPLANT_REJECTED_VS_OK_DN	0.58	0.01	0	0	0	not significant
8530	GO_CYCLIN_DEPENDENT_PROTEIN_KINASE_ACTIVITY	0.58	0.01	0	0	0	not significant
8531	CHR1Q24	0.58	0.00	0	0	0	not significant
8532	GO_NEGATIVE_REGULATION_OF_AUTOPHAGY	0.58	0.00	0	0	0	not significant
8533	KEGG_NEUROTROPHIN_SIGNALING_PATHWAY	0.58	0.00	0	0	0	not significant
8534	KEGG_PANCREATIC_CANCER	0.58	0.00	0	0	0	not significant
8535	GO_NEUROTRANSMITTER_REUPTAKE	0.59	0.04	0	0	0	not significant
8536	CHIN_BREAST_CANCER_COPY_NUMBER_DN	0.59	0.03	0	0	0	not significant
8537	CHR10P14	0.59	0.03	0	0	0	not significant
8538	GO_DNA_REPLICATION_SYNTHESIS_OF_RNA_PRIMER	0.59	0.03	0	0	0	not significant
8539	GO_ORGANIC_CATION_TRANSMEMBRANE_TRANSPORTER_ACTIVITY	0.59	0.03	0	0	0	not significant
8540	GO_POSITIVE_REGULATION_OF_CREB_TRANSCRIPTION_FACTOR_ACTIV	0.59	0.03	0	0	0	not significant
8541	GO_POSITIVE_REGULATION_OF_METHYLATION_DEPENDENT_CHROMATI	0.59	0.03	0	0	0	not significant
8542	GO_POSITIVE_REGULATION_OF_SMOOTH_MUSCLE_CELL_APOPTOTIC_I	0.59	0.03	0	0	0	not significant
8543	GO_PYRIMIDINE_NUCLEOSIDE_TRIPHOSPHATE_BIOSYNTHETIC_PROCES	0.59	0.03	0	0	0	not significant
8544	IIZUKA_LIVER_CANCER_PROGRESSION_G2_G3_DN	0.59	0.03	0	0	0	not significant
8545	GO_FORMATION_OF_EXTRACHROMOSOMAL_CIRCULAR_DNA	0.59	0.02	0	0	0	not significant
8546	GO_MISMATCH_REPAIR_COMPLEX	0.59	0.02	0	0	0	not significant
8547	GO_PHOSPHATIDYLGLYCEROL_METABOLIC_PROCESS	0.59	0.02	0	0	0	not significant
8548	GO_PIGMENT_CELL_DIFFERENTIATION	0.59	0.02	0	0	0	not significant
8549	GO_POSITIVE_REGULATION_OF_BLOOD_CIRCULATION	0.59	0.02	0	0	0	not significant
8550	GO_REGULATION_OF_MAST_CELL_ACTIVATION_INVOLVED_IN_IMMUNE_I	0.59	0.02	0	0	0	not significant
8551	GROSS_HYPOXIA_VIA_ELK3_ONLY_UP	0.59	0.02	0	0	0	not significant
8552	REACTOME_REGULATION_OF_SIGNALING_BY_CBL	0.59	0.02	0	0	0	not significant
8553	GO_CELLULAR_IRON_ION_HOMEOSTASIS	0.59	0.01	0	0	0	not significant
8554	GO_NUCLEOTIDE_EXCISION_REPAIR_DNA_GAP_FILLING	0.59	0.01	0	0	0	not significant
8555	GO_OOCYTE_DIFFERENTIATION	0.59	0.01	0	0	0	not significant
8556	GO_ENDOSOMAL_TRANSPORT	0.59	0.00	0	0	0	not significant
8557	GO_LIPID_PHOSPHORYLATION	0.59	0.00	0	0	0	not significant
8558	GO_TRANS_GOLGI_NETWORK_MEMBRANE	0.59	0.00	0	0	0	not significant
8559	KEGG_ALPHA_LINOLENIC_ACID_METABOLISM	0.60	0.05	0	0	0	not significant
8560	GO_ADAPTATION_OF_SIGNALING_PATHWAY	0.60	0.04	0	0	0	not significant
8561	GO_PHOSPHATIDYLGLYCEROL_ACYL_CHAIN_REMODELING	0.60	0.04	0	0	0	not significant
8562	GO_PROTEIN_HETEROTRIMERIZATION	0.60	0.04	0	0	0	not significant
8563	GO_REGULATION_OF_CHAPERONE_MEDIATED_AUTOPHAGY	0.60	0.04	0	0	0	not significant
8564	GO_REGULATION_OF_SEQUESTERING_OF_TRIGLYCERIDE	0.60	0.04	0	0	0	not significant
8565	REACTOME_ERYTHROPOIETIN_ACTIVATES_RAS	0.60	0.04	0	0	0	not significant
8566	REACTOME_SIGNALING_BY_FGFR4_IN_DISEASE	0.60	0.04	0	0	0	not significant
8567	DARWICHE_PAPILLOMA_RISK_HIGH_VS_LOW_UP	0.60	0.03	0	0	0	not significant
8568	GO_BASEMENT_MEMBRANE_ASSEMBLY	0.60	0.03	0	0	0	not significant
8569	GO_MRNA_3_SPLICE_SITE_RECOGNITION	0.60	0.03	0	0	0	not significant
8570	CHR13Q32	0.60	0.02	0	0	0	not significant
8571	GO_5_3_EXONUCLEASE_ACTIVITY	0.60	0.02	0	0	0	not significant
8572	GO_CILIUM_OR_FLAGELLUM_DEPENDENT_CELL_MOTILITY	0.60	0.02	0	0	0	not significant
8573	GO_PEPTIDYL_ASPARAGINE_MODIFICATION	0.60	0.02	0	0	0	not significant
8574	GO_POSITIVE_REGULATION_OF_BLOOD_VESSEL_DIAMETER	0.60	0.02	0	0	0	not significant
8575	GO_WNT_SIGNALING_PATHWAY_CALCIUM_MODULATING_PATHWAY	0.60	0.02	0	0	0	not significant
8576	HOLLEMAN_ASPARAGINASE_RESISTANCE_ALL_DN	0.60	0.02	0	0	0	not significant
8577	LIU_BREAST_CANCER	0.60	0.02	0	0	0	not significant
8578	GO_VESICLE_MEDIATED_TRANSPORT_BETWEEN_ENDOSOMAL_COMPAF	0.60	0.01	0	0	0	not significant
8579	GINESTIER_BREAST_CANCER_20Q13_AMPLIFICATION_UP	0.60	0.00	0	0	0	not significant
8580	SIRNA_EIF4GI_DN	0.60	0.00	0	0	0	not significant
8581	GO_RNA_POLYMERASE_II_C_TERMINAL_DOMAIN_BINDING	0.61	0.06	0	0	0	not significant
8582	GO_COENZYME_TRANSPORT	0.61	0.04	0	0	0	not significant
8583	GO_DORSAL_VENTRAL_AXIS_SPECIFICATION	0.61	0.04	0	0	0	not significant
8584	GO_NEGATIVE_REGULATION_OF_VIRAL_INDUCED_CYTOPLASMIC_PATTI	0.61	0.04	0	0	0	not significant
8585	GO_POSITIVE_REGULATION_OF_CYTOPLASMIC_TRANSLATION	0.61	0.04	0	0	0	not significant
8586	GO_REPLICATION_FORK_ARREST	0.61	0.04	0	0	0	not significant
8587	GO_RNA_POLYMERASE_III_GENERAL_TRANSCRIPTION_INITIATION_FACT	0.61	0.04	0	0	0	not significant
8588	GO_SYNAPTIC_VESICLE_PRIMING	0.61	0.04	0	0	0	not significant
8589	GO_COPI_COATED_VESICLE_MEMBRANE	0.61	0.03	0	0	0	not significant
8590	GO_NEGATIVE_REGULATION_OF_ANION_TRANSMEMBRANE_TRANSPORT	0.61	0.03	0	0	0	not significant
8591	GO_NEGATIVE_REGULATION_OF_HEART_CONTRACTION	0.61	0.03	0	0	0	not significant
8592	GO_POSITIVE_REGULATION_OF_VASCULAR_ASSOCIATED_SMOOTH_MU	0.61	0.03	0	0	0	not significant
8593	GO_POSITIVE_REGULATION_OF_VASCULAR_ENDOTHELIAL_GROWTH_F	0.61	0.03	0	0	0	not significant
8594	GO_RESPONSE_TO_FOLIC_ACID	0.61	0.03	0	0	0	not significant
8595	GO_SWI_SNF_COMPLEX	0.61	0.03	0	0	0	not significant
8596	REACTOME_EPHRIN_SIGNALING	0.61	0.03	0	0	0	not significant
8597	SAGIV_CD24_TARGETS_UP	0.61	0.03	0	0	0	not significant
8598	BIOCARTA_IL6_PATHWAY	0.61	0.02	0	0	0	not significant
8599	CHR8Q12	0.61	0.02	0	0	0	not significant

8600	GO_DNA_DOUBLE_STRAND_BREAK_PROCESSING	0.61	0.02	0	0	0	not significant
8601	KEGG_DORSO_VENTRAL_AXIS_FORMATION	0.61	0.02	0	0	0	not significant
8602	REACTOME_SIGNALING_BY_FGFR3_FUSIONS_IN_CANCER	0.61	0.02	0	0	0	not significant
8603	GO_CORTICAL_CYTOSKELETON_ORGANIZATION	0.61	0.01	0	0	0	not significant
8604	GO_POSITIVE_REGULATION_OF_MACROAUTOPHAGY	0.61	0.01	0	0	0	not significant
8605	GO_PROTEIN_LOCALIZATION_TO_CILIUM	0.61	0.01	0	0	0	not significant
8606	GO_VIRION_ASSEMBLY	0.61	0.01	0	0	0	not significant
8607	KEGG_BASE_EXCISION_REPAIR	0.61	0.01	0	0	0	not significant
8608	PID_IFNG_PATHWAY	0.61	0.01	0	0	0	not significant
8609	GO_AUTOPHAGOSOME_ORGANIZATION	0.61	0.00	0	0	0	not significant
8610	GO_GOLGI_ORGANIZATION	0.61	0.00	0	0	0	not significant
8611	GO_TRANS_GOLGI_NETWORK	0.61	0.00	0	0	0	not significant
8612	CLAUS_PGR_POSITIVE_MENINGIOMA_UP	0.62	0.05	0	0	0	not significant
8613	GO_REGULATION_OF_OSTEOCLAST_DEVELOPMENT	0.62	0.05	0	0	0	not significant
8614	GO_REGULATION_OF_SMAD_PROTEIN_COMPLEX_ASSEMBLY	0.62	0.05	0	0	0	not significant
8615	REACTOME_SMAC_XIAP_REGULATED_APOPTOTIC_RESPONSE	0.62	0.05	0	0	0	not significant
8616	GO_BBSOME	0.62	0.04	0	0	0	not significant
8617	GO_C4_DICARBOXYLATE_TRANSPORT	0.62	0.04	0	0	0	not significant
8618	GO_CD4_POSITIVE_ALPHA_BETA_T_CELL_PROLIFERATION	0.62	0.04	0	0	0	not significant
8619	GO_LIPOPROTEIN_PARTICLE_RECEPTOR_ACTIVITY	0.62	0.04	0	0	0	not significant
8620	GO_MUSCLE_ALPHA_ACTININ_BINDING	0.62	0.04	0	0	0	not significant
8621	GO_REGULATION_OF_SKELETAL_MUSCLE_CELL_DIFFERENTIATION	0.62	0.04	0	0	0	not significant
8622	GO_SYNAPTIC_VESICLE_DOCKING	0.62	0.04	0	0	0	not significant
8623	RAMJAUN_APOPTOSIS_BY_TGFB1_VIA_SMAD4_DN	0.62	0.04	0	0	0	not significant
8624	SYED_ESTRADIOL_RESPONSE	0.62	0.04	0	0	0	not significant
8625	CHEN_ETV5_TARGETS_TESTIS	0.62	0.03	0	0	0	not significant
8626	GO_AMINO_SUGAR_BIOSYNTHETIC_PROCESS	0.62	0.03	0	0	0	not significant
8627	GO_DIVALENT_INORGANIC_CATION_TRANSMEMBRANE_TRANSPORTER_	0.62	0.03	0	0	0	not significant
8628	GO_LYSOPHOSPHOLIPID_ACYLTRANSFERASE_ACTIVITY	0.62	0.03	0	0	0	not significant
8629	GO_MLL3_4_COMPLEX	0.62	0.03	0	0	0	not significant
8630	GO_POSITIVE_REGULATION_OF_LIPID_KINASE_ACTIVITY	0.62	0.03	0	0	0	not significant
8631	GO_RESPONSE_TO_INTERFERON_ALPHA	0.62	0.03	0	0	0	not significant
8632	GO_SUMO_TRANSFERASE_ACTIVITY	0.62	0.03	0	0	0	not significant
8633	REACTOME_NUCLEOBASE_CATABOLISM	0.62	0.03	0	0	0	not significant
8634	BIOCARTA_ATM_PATHWAY	0.62	0.02	0	0	0	not significant
8635	GAZIN_EPIGENETIC_SILENCING_BY_KRAS	0.62	0.02	0	0	0	not significant
8636	GO_DEVELOPMENTAL_PIGMENTATION	0.62	0.02	0	0	0	not significant
8637	GO_POSITIVE_REGULATION_OF_INTERLEUKIN_1_PRODUCTION	0.62	0.02	0	0	0	not significant
8638	REACTOME_BUDDING_AND_MATURATION_OF_HIV_VIRION	0.62	0.02	0	0	0	not significant
8639	REACTOME_SIGNALING_BY_FGFR3	0.62	0.02	0	0	0	not significant
8640	GO_PH_REDUCTION	0.62	0.01	0	0	0	not significant
8641	GO_REGULATION_OF_PROTEIN_ACETYLATION	0.62	0.01	0	0	0	not significant
8642	REACTOME_HDR_THROUGH_HOMOLOGOUS_RECOMBINATION_HRR	0.62	0.00	0	0	0	not significant
8643	GO_DETECTION_OF_TEMPERATURE_STIMULUS	0.63	0.05	0	0	0	not significant
8644	GO_HYALURONAN_BIOSYNTHETIC_PROCESS	0.63	0.05	0	0	0	not significant
8645	GO_LRR_DOMAIN_BINDING	0.63	0.04	0	0	0	not significant
8646	GO_POSITIVE_REGULATION_OF_CYCLASE_ACTIVITY	0.63	0.04	0	0	0	not significant
8647	GO_POSITIVE_REGULATION_OF_PROTEIN_POLYUBIQUITINATION	0.63	0.04	0	0	0	not significant
8648	GO_POSITIVE_REGULATION_OF_TRANSLATION_IN_RESPONSE_TO_STRE	0.63	0.04	0	0	0	not significant
8649	GO_PRE_MIRNA_PROCESSING	0.63	0.04	0	0	0	not significant
8650	GO_REGULATION_OF_RESPONSE_TO_INTERFERON_GAMMA	0.63	0.04	0	0	0	not significant
8651	GO_SENSORY_PERCEPTION_OF_TEMPERATURE_STIMULUS	0.63	0.04	0	0	0	not significant
8652	GO_SIGNAL_PEPTIDE_PROCESSING	0.63	0.04	0	0	0	not significant
8653	KYNG_NORMAL_AGING_UP	0.63	0.04	0	0	0	not significant
8654	BCAT.100_UP.V1_UP	0.63	0.03	0	0	0	not significant
8655	CHIBA_RESPONSE_TO_TSA_DN	0.63	0.03	0	0	0	not significant
8656	CHR9Q31	0.63	0.03	0	0	0	not significant
8657	GO_DNA_LIGATION	0.63	0.03	0	0	0	not significant
8658	GO_MAGNESIUM_DEPENDENT_PROTEIN_SERINE_THREONINE_PHOSPHAT	0.63	0.03	0	0	0	not significant
8659	GO_NUCLEOSIDE_SALVAGE	0.63	0.03	0	0	0	not significant
8660	GO_PATHWAY_RESTRICTED_SMAD_PROTEIN_PHOSPHORYLATION	0.63	0.03	0	0	0	not significant
8661	GO_CUL3_RING_UBIQUITIN_LIGASE_COMPLEX	0.63	0.02	0	0	0	not significant
8662	GO_DNA_REPAIR_COMPLEX	0.63	0.02	0	0	0	not significant
8663	GO_RNA_POLYMERASE_II_ACTIVATING_TRANSCRIPTION_FACTOR_BINDI	0.63	0.02	0	0	0	not significant
8664	REACTOME_RESOLUTION_OF_ABASIC_SITES_AP_SITES	0.63	0.02	0	0	0	not significant
8665	GO_CILIARY_TIP	0.63	0.01	0	0	0	not significant
8666	GO_DAMAGED_DNA_BINDING	0.63	0.01	0	0	0	not significant
8667	GO_REGULATION_OF_PEPTIDYL_LYSINE_ACETYLATION	0.63	0.01	0	0	0	not significant
8668	CHR13Q14	0.63	0.00	0	0	0	not significant
8669	GO_REGULATION_OF_CHROMATIN_ORGANIZATION	0.63	0.00	0	0	0	not significant
8670	RAO_BOUND_BY_SALL4_ISOFORM_A	0.63	0.00	0	0	0	not significant
8671	GO_DETECTION_OF_TEMPERATURE_STIMULUS_INVOLVED_IN_SENSORY	0.64	0.06	0	0	0	not significant
8672	GO_DOUBLE_STRAND_BREAK_REPAIR_VIA_SINGLE_STRAND_ANNEALING	0.64	0.06	0	0	0	not significant
8673	GO_FILTRATION_DIAPHRAGM	0.64	0.06	0	0	0	not significant
8674	GO_LAGGING_STRAND_ELONGATION	0.64	0.06	0	0	0	not significant
8675	GO_MELANOSOME_MEMBRANE	0.64	0.06	0	0	0	not significant
8676	BIOCARTA_CHREBP_PATHWAY	0.64	0.05	0	0	0	not significant
8677	CLASPER_LYMPHATIC_VESSELS_DURING_METASTASIS_UP	0.64	0.05	0	0	0	not significant
8678	GO_AXONAL_TRANSPORT_OF_MITOCHONDRION	0.64	0.05	0	0	0	not significant
8679	GO_CARDIOBLAST_DIFFERENTIATION	0.64	0.05	0	0	0	not significant
8680	GO_DENDRITE_TERMINUS	0.64	0.05	0	0	0	not significant
8681	GO_GOLGI_MEDIAL_CISTERNA	0.64	0.05	0	0	0	not significant
8682	GO_INTRACILIARY_RETROGRADE_TRANSPORT	0.64	0.05	0	0	0	not significant
8683	GO_MAP_KINASE_SCAFFOLD_ACTIVITY	0.64	0.05	0	0	0	not significant
8684	GO_SPHINGOMYELIN_CATABOLIC_PROCESS	0.64	0.05	0	0	0	not significant
8685	MATZUK_EARLY_ANTRAL_FOLLICLE	0.64	0.05	0	0	0	not significant
8686	NIKOLSKY_BREAST_CANCER_15Q26_AMPLICON	0.64	0.05	0	0	0	not significant
8687	REACTOME_IKBA_VARIANT_LEADS_TO_EDA_ID	0.64	0.05	0	0	0	not significant
8688	GO_CELL_CELL_ADHESION_MEDIATED_BY_INTEGRIN	0.64	0.04	0	0	0	not significant
8689	GO_OSTEOBLAST_DEVELOPMENT	0.64	0.04	0	0	0	not significant
8690	GO_PORE_COMPLEX_ASSEMBLY	0.64	0.04	0	0	0	not significant
8691	GO_REGULATION_OF_MICROTUBULE_MOTOR_ACTIVITY	0.64	0.04	0	0	0	not significant
8692	REACTOME_SIGNALING_BY_HIPPO	0.64	0.04	0	0	0	not significant
8693	REACTOME_TRP_CHANNELS	0.64	0.04	0	0	0	not significant
8694	BERNARD_PPAPDC1B_TARGETS_UP	0.64	0.03	0	0	0	not significant
8695	GO_CRANIOFACIAL_SUTURE_MORPHOGENESIS	0.64	0.03	0	0	0	not significant
8696	GO_NEGATIVE_REGULATION_OF_GENE_SILENCING	0.64	0.03	0	0	0	not significant
8697	GO_POSITIVE_REGULATION_OF_DNA_TEMPLATED_TRANSCRIPTION_ELC	0.64	0.03	0	0	0	not significant
8698	GO_PROTEIN_LOCALIZATION_TO_CHROMOSOME_CENTROMERIC_REGIC	0.64	0.03	0	0	0	not significant
8699	GO_SNARE_COMPLEX_ASSEMBLY	0.64	0.03	0	0	0	not significant
8700	REACTOME_INSERTION_OF_TAIL_ANCHORED_PROTEINS_INTO_THE_END	0.64	0.03	0	0	0	not significant
8701	BCAT_BILD_ET_AL_DN	0.64	0.02	0	0	0	not significant
8702	GAZDA_DIAMOND_BLACKFAN_ANEMIA_MYELOID_UP	0.64	0.02	0	0	0	not significant
8703	GO_CORE_PROMOTER_SEQUENCE_SPECIFIC_DNA_BINDING	0.64	0.02	0	0	0	not significant
8704	GO_DNA_SECONDARY_STRUCTURE_BINDING	0.64	0.02	0	0	0	not significant
8705	WNT_SIGNALING	0.64	0.02	0	0	0	not significant
8706	GO_NEGATIVE_REGULATION_OF_CHROMATIN_ORGANIZATION	0.64	0.01	0	0	0	not significant
8707	GO_TUBE_CLOSURE	0.64	0.01	0	0	0	not significant
8708	KEGG_NON_SMALL_CELL_LUNG_CANCER	0.64	0.01	0	0	0	not significant

8709	GO_HISTONE_METHYLATION	0.64	0.00	0	0	0	not significant
8710	GO_VACUOLE_ORGANIZATION	0.64	0.00	0	0	0	not significant
8711	REACTOME_AUTOPHAGY	0.64	0.00	0	0	0	not significant
8712	GO_APOPTOTIC_PROCESS_INVOLVED_IN_MORPHOGENESIS	0.65	0.06	0	0	0	not significant
8713	GO_AUTOLYSOSOME	0.65	0.06	0	0	0	not significant
8714	GO_NEGATIVE_REGULATION_OF_LONG_TERM_SYNAPTIC_POTENTIATIO	0.65	0.06	0	0	0	not significant
8715	GO_POSTSYNAPTIC_MEMBRANE_ASSEMBLY	0.65	0.06	0	0	0	not significant
8716	REACTOME_ACTIVATED_NTRK2_SIGNALS_THROUGH_FRS2_AND_FRS3	0.65	0.06	0	0	0	not significant
8717	REACTOME_ESTROGEN_DEPENDENT_NUCLEAR_EVENTS_DOWNSTREAM	0.65	0.06	0	0	0	not significant
8718	BIOCARTA_ERAD_PATHWAY	0.65	0.05	0	0	0	not significant
8719	DORN_ADENOVIRUS_INFECTION_32HR_UP	0.65	0.05	0	0	0	not significant
8720	FRASOR_TAMOXIFEN_RESPONSE_DN	0.65	0.05	0	0	0	not significant
8721	GO_HIGH_DENSITY_LIPOPROTEIN_PARTICLE_ASSEMBLY	0.65	0.05	0	0	0	not significant
8722	GO_NEGATIVE_REGULATION_OF_AMYLOID_PRECURSOR_PROTEIN_BIOS	0.65	0.05	0	0	0	not significant
8723	GO_NEGATIVE_REGULATION_OF_PROTEIN_HOMOOLIGOMERIZATION	0.65	0.05	0	0	0	not significant
8724	GO_POSITIVE_REGULATION_OF_CELLULAR_RESPONSE_TO_DRUG	0.65	0.05	0	0	0	not significant
8725	GO_POSITIVE_REGULATION_OF_FATTY_ACID_BETA_OXIDATION	0.65	0.05	0	0	0	not significant
8726	GO_REGULATION_OF_PROTEIN_LOCALIZATION_TO_CHROMATIN	0.65	0.05	0	0	0	not significant
8727	GO_REGULATION_OF_RESPIRATORY_BURST	0.65	0.05	0	0	0	not significant
8728	SAENZ_DETOX_PATHWAY_AND_CARCINOGENESIS_DN	0.65	0.05	0	0	0	not significant
8729	GO_CENTRIOLE_CENTRIOLE_COHESION	0.65	0.04	0	0	0	not significant
8730	GO_PHOSPHATIDIC_ACID_BINDING	0.65	0.04	0	0	0	not significant
8731	KEGG_BLADDER_CANCER	0.65	0.04	0	0	0	not significant
8732	PID_WNT_CANONICAL_PATHWAY	0.65	0.04	0	0	0	not significant
8733	CHR11P13	0.65	0.03	0	0	0	not significant
8734	DACOSTA_UV_RESPONSE_VIA_ERCC3_XPCS_UP	0.65	0.03	0	0	0	not significant
8735	GO_AUTOPHAGOSOME_MATURATION	0.65	0.03	0	0	0	not significant
8736	GO_ENDONUCLEASE_ACTIVITY_ACTIVE_WITH_EITHER_RIBO_OR_DEOXY	0.65	0.03	0	0	0	not significant
8737	GO_ENDOPLASMIC_RETICULUM_TO_CYTOSOL_TRANSPORT	0.65	0.03	0	0	0	not significant
8738	GO_TRANSFERASE_ACTIVITY_TRANSFERRING_SULFUR_CONTAINING_GI	0.65	0.03	0	0	0	not significant
8739	HERNANDEZ_MITOTIC_ARREST_BY_DOCETAXEL_1_DN	0.65	0.03	0	0	0	not significant
8740	KIM_TIAL1_TARGETS	0.65	0.03	0	0	0	not significant
8741	XU_CREBBP_TARGETS_DN	0.65	0.03	0	0	0	not significant
8742	GO_CELLULAR_RESPONSE_TO_NUTRIENT	0.65	0.02	0	0	0	not significant
8743	GO_NEGATIVE_REGULATION_OF_CELLULAR_RESPONSE_TO_TRANSFOI	0.65	0.02	0	0	0	not significant
8744	GO_REGULATION_OF_TRANSCRIPTION_REGULATORY_REGION_DNA_BIN	0.65	0.02	0	0	0	not significant
8745	GO_ROOF_OF_MOUTH_DEVELOPMENT	0.65	0.02	0	0	0	not significant
8746	REACTOME_HOMOLOGOUS_DNA_PAIRING_AND_STRAND_EXCHANGE	0.65	0.02	0	0	0	not significant
8747	GO_ANDROGEN_RECEPTOR_BINDING	0.65	0.01	0	0	0	not significant
8748	GO_PHOSPHOPROTEIN_BINDING	0.65	0.01	0	0	0	not significant
8749	TIEN_INTESTINE_PROBIOTICS_2HR_DN	0.65	0.01	0	0	0	not significant
8750	GO_CENTRIOLE_ELONGATION	0.66	0.07	0	0	0	not significant
8751	GO_VASCULAR_ASSOCIATED_SMOOTH_MUSCLE_CELL_APOPTOTIC_PR	0.66	0.07	0	0	0	not significant
8752	MYLLYKANGAS_AMPLIFICATION_HOT_SPOT_2	0.66	0.07	0	0	0	not significant
8753	BOYERINAS_ONCOFETAL_TARGETS_OF_LET7A1	0.66	0.06	0	0	0	not significant
8754	GO_COBALAMIN_BINDING	0.66	0.06	0	0	0	not significant
8755	GO_G1_TO_G0_TRANSITION	0.66	0.06	0	0	0	not significant
8756	GO_HEMIDESMOSOME_ASSEMBLY	0.66	0.06	0	0	0	not significant
8757	GO_NEGATIVE_REGULATION_OF_CHEMOKINE_PRODUCTION	0.66	0.06	0	0	0	not significant
8758	GO_NUCLEAR_TRANSCRIBED_MRNA_CATABOLIC_PROCESS_DEADENYL	0.66	0.06	0	0	0	not significant
8759	GO_POSITIVE_REGULATION_OF_FATTY_ACID_METABOLIC_PROCESS	0.66	0.06	0	0	0	not significant
8760	GO_REGULATION_OF_DEOXYRIBONUCLEASE_ACTIVITY	0.66	0.06	0	0	0	not significant
8761	PLASARI_NFIC_TARGETS_BASAL_UP	0.66	0.06	0	0	0	not significant
8762	REACTOME_TYPE_I_HEMIDESMOSOME_ASSEMBLY	0.66	0.06	0	0	0	not significant
8763	GO_GLUCOSYLTRANSFERASE_ACTIVITY	0.66	0.05	0	0	0	not significant
8764	GO_RESPONSE_TO_HEPATOCYTE_GROWTH_FACTOR	0.66	0.05	0	0	0	not significant
8765	GO_EMBRYONIC_CRANIAL_SKELETON_MORPHOGENESIS	0.66	0.04	0	0	0	not significant
8766	GO_INSULIN_RECEPTOR_BINDING	0.66	0.04	0	0	0	not significant
8767	GO_MICROTUBULE_ANCHORING	0.66	0.04	0	0	0	not significant
8768	PID_PDGFRA_PATHWAY	0.66	0.04	0	0	0	not significant
8769	SHIN_B_CELL_LYMPHOMA_CLUSTER_7	0.66	0.04	0	0	0	not significant
8770	GO_HISTONE_H3_K4_TRIMETHYLATION	0.66	0.03	0	0	0	not significant
8771	GO_PHOSPHOTYROSINE_RESIDUE_BINDING	0.66	0.03	0	0	0	not significant
8772	GO_CYTOPLASMIC_MICROTUBULE_ORGANIZATION	0.66	0.02	0	0	0	not significant
8773	GO_POSITIVE_REGULATION_OF_MRNA_CATABOLIC_PROCESS	0.66	0.02	0	0	0	not significant
8774	GRADE_COLON_VS_RECTAL_CANCER_DN	0.66	0.02	0	0	0	not significant
8775	KEGG_N_GLYCAN_BIOSYNTHESIS	0.66	0.02	0	0	0	not significant
8776	KYNG_WERNER_SYNDROM_AND_NORMAL_AGING_UP	0.66	0.02	0	0	0	not significant
8777	PID_CD8_TCR_PATHWAY	0.66	0.02	0	0	0	not significant
8778	RADMACHER_AML_PROGNOSIS	0.66	0.02	0	0	0	not significant
8779	GO_CHROMATIN_ORGANIZATION_INVOLVED_IN_REGULATION_OF_TRANS	0.66	0.01	0	0	0	not significant
8780	GO_ENERGY_RESERVE_METABOLIC_PROCESS	0.66	0.01	0	0	0	not significant
8781	GO_REGULATION_OF_DNA_REPLICATION	0.66	0.01	0	0	0	not significant
8782	GO_PROTEIN_ACETYLATION	0.66	0.00	0	0	0	not significant
8783	GO_POSITIVE_REGULATION_OF_CELL_SIZE	0.67	0.08	0	0	0	not significant
8784	GO_DENDRITE_SELF_AVOIDANCE	0.67	0.07	0	0	0	not significant
8785	GO_MYELIN_SHEATH_ABAXONAL_REGION	0.67	0.07	0	0	0	not significant
8786	GO_NEGATIVE_REGULATION_OF_PROTEIN_AUTOPHOSPHORYLATION	0.67	0.07	0	0	0	not significant
8787	GO_NUCLEOTIDE_TRANSMEMBRANE_TRANSPORT	0.67	0.07	0	0	0	not significant
8788	REACTOME_INLA_MEDIATED_ENTRY_OF_LISTERIA_MONOCYTOGENES_I	0.67	0.07	0	0	0	not significant
8789	CHR5Q15	0.67	0.06	0	0	0	not significant
8790	GO_HISTONE_MRNA_CATABOLIC_PROCESS	0.67	0.06	0	0	0	not significant
8791	GO_NEUROTRANSMITTER_SODIUM_SYMPORTER_ACTIVITY	0.67	0.06	0	0	0	not significant
8792	BIOCARTA_HDAC_PATHWAY	0.67	0.05	0	0	0	not significant
8793	GO_DYNEIN_LIGHT_CHAIN_BINDING	0.67	0.05	0	0	0	not significant
8794	GO_INSULIN_LIKE_GROWTH_FACTOR_RECEPTOR_SIGNALING_PATHWAY	0.67	0.05	0	0	0	not significant
8795	GO_NEGATIVE_REGULATION_OF_UBIQUITIN_PROTEIN_TRANSFERASE_A	0.67	0.05	0	0	0	not significant
8796	GO_POSITIVE_REGULATION_OF_ANTIGEN_RECEPTOR_MEDIATED_SIGNA	0.67	0.05	0	0	0	not significant
8797	GO_REGULATION_OF_CYTOPLASMIC_TRANSPORT	0.67	0.05	0	0	0	not significant
8798	GO_REGULATION_OF_FIBROBLAST_MIGRATION	0.67	0.05	0	0	0	not significant
8799	PID_ALK1_PATHWAY	0.67	0.05	0	0	0	not significant
8800	REACTOME_DOWNSTREAM_SIGNALING_OF_ACTIVATED_FGFR3	0.67	0.05	0	0	0	not significant
8801	CHR8P12	0.67	0.04	0	0	0	not significant
8802	GO_AMINE_BIOSYNTHETIC_PROCESS	0.67	0.04	0	0	0	not significant
8803	GO_NEGATIVE_REGULATION_OF_GTPASE_ACTIVITY	0.67	0.04	0	0	0	not significant
8804	GO_NEGATIVE_REGULATION_OF_LIPID_BIOSYNTHETIC_PROCESS	0.67	0.04	0	0	0	not significant
8805	GO_PRI_MIRNA_TRANSCRIPTION_BY_RNA_POLYMERASE_II	0.67	0.04	0	0	0	not significant
8806	GO_PEPTIDYL_LYSINE_TRIMETHYLATION	0.67	0.03	0	0	0	not significant
8807	REACTOME_REGULATION_OF_TP53_ACTIVITY_THROUGH_ACETYLATION	0.67	0.03	0	0	0	not significant
8808	SMID_BREAST_CANCER_RELAPSE_IN_BRAIN_DN	0.67	0.03	0	0	0	not significant
8809	GO_CYTOPLASMIC_MICROTUBULE	0.67	0.02	0	0	0	not significant
8810	GO_HISTONE_H4_ACETYLATION	0.67	0.02	0	0	0	not significant
8811	GO_LYTIC_VACUOLE_ORGANIZATION	0.67	0.02	0	0	0	not significant
8812	MCCLUNG_CREB1_TARGETS_DN	0.67	0.02	0	0	0	not significant
8813	REACTOME_INTRINSIC_PATHWAY_FOR_APOPTOSIS	0.67	0.02	0	0	0	not significant
8814	FIGUEROA_AML_METHYLATION_CLUSTER_7_UP	0.67	0.01	0	0	0	not significant
8815	GO_NON_RECOMBINATIONAL_REPAIR	0.67	0.01	0	0	0	not significant
8816	GO_REGULATION_OF_NEUROTRANSMITTER_TRANSPORT	0.67	0.01	0	0	0	not significant
8817	LIN_NPAS4_TARGETS_UP	0.67	0.01	0	0	0	not significant

8818	REACTOME_P75_NTR_RECEPTOR_MEDIATED_SIGNALLING	0.67	0.01	0	0	0	not significant
8819	GO_PEPTIDYL_LYSINE_ACETYLATION	0.67	0.00	0	0	0	not significant
8820	CHR8Q23	0.68	0.08	0	0	0	not significant
8821	GO_CALCIUM_ION_IMPORT_INTO_CYTOSOL	0.68	0.08	0	0	0	not significant
8822	GO_CROSSOVER_JUNCTION_ENDODEOXYRIBONUCLEASE_ACTIVITY	0.68	0.08	0	0	0	not significant
8823	GO_POSITIVE_REGULATION_OF_HETEROTYPIC_CELL_CELL_ADHESION	0.68	0.08	0	0	0	not significant
8824	GO_PRESYNAPTIC_CYTOSOL	0.68	0.08	0	0	0	not significant
8825	GO_NEUTROPHIL_DIFFERENTIATION	0.68	0.07	0	0	0	not significant
8826	GO_POST_MRNA_RELEASE_SPLICEOSOMAL_COMPLEX	0.68	0.07	0	0	0	not significant
8827	GO_RESPONSE_TO_DOPAMINE	0.68	0.07	0	0	0	not significant
8828	GO_RIPOPTOSOME	0.68	0.07	0	0	0	not significant
8829	MOOTHA_ROS	0.68	0.07	0	0	0	not significant
8830	PID_FRA_PATHWAY	0.68	0.07	0	0	0	not significant
8831	REACTOME_TRANSPORT_OF_NUCLEOTIDE_SUGARS	0.68	0.07	0	0	0	not significant
8832	REACTOME_TRIGLYCERIDE_BIOSYNTHESIS	0.68	0.07	0	0	0	not significant
8833	DORN_ADENOVIRUS_INFECTION_12HR_UP	0.68	0.06	0	0	0	not significant
8834	GO_CALCIUM_DEPENDENT_CYSTEINE_TYPE_ENDOPEPTIDASE_ACTIVITY	0.68	0.06	0	0	0	not significant
8835	GO_COLLAGEN_CATABOLIC_PROCESS	0.68	0.06	0	0	0	not significant
8836	GO_DEADENYLATION_DEPENDENT_DECAPPING_OF_NUCLEAR_TRANSCI	0.68	0.06	0	0	0	not significant
8837	GO_FORMATION_OF_TRANSLATION_PREINITIATION_COMPLEX	0.68	0.06	0	0	0	not significant
8838	GO_MICROTUBULE_ANCHORING_AT_MICROTUBULE_ORGANIZING_CENTE	0.68	0.06	0	0	0	not significant
8839	GO_NEGATIVE_REGULATION_OF_MYOTUBE_DIFFERENTIATION	0.68	0.06	0	0	0	not significant
8840	GO_POSITIVE_REGULATION_OF_GRANULOCYTE_CHEMOTAXIS	0.68	0.06	0	0	0	not significant
8841	GO_POSITIVE_REGULATION_OF_NEUTROPHIL_MIGRATION	0.68	0.06	0	0	0	not significant
8842	KAMIKUBO_MYELOID_MN1_NETWORK	0.68	0.06	0	0	0	not significant
8843	LI_CYTIDINE_ANALOG_PATHWAY	0.68	0.06	0	0	0	not significant
8844	BIOCARTA_ARF_PATHWAY	0.68	0.05	0	0	0	not significant
8845	BIOCARTA_BARRESTIN_SRC_PATHWAY	0.68	0.05	0	0	0	not significant
8846	CHR7P14	0.68	0.05	0	0	0	not significant
8847	GO_MAST_CELL_MEDIATED_IMMUNITY	0.68	0.04	0	0	0	not significant
8848	GO_SH2_DOMAIN_BINDING	0.68	0.04	0	0	0	not significant
8849	REACTOME_SIGNALING_BY_FGFR4	0.68	0.04	0	0	0	not significant
8850	SIG_PIP3_SIGNALING_IN_B_LYMPHOCYTES	0.68	0.04	0	0	0	not significant
8851	GO_MULTICELLULAR_ORGANISMAL_RESPONSE_TO_STRESS	0.68	0.03	0	0	0	not significant
8852	REACTOME_INTRA_GOLGI_TRAFFIC	0.68	0.03	0	0	0	not significant
8853	GO_PML_BODY	0.68	0.02	0	0	0	not significant
8854	GO_UBIQUITIN_DEPENDENT_ERAD_PATHWAY	0.68	0.02	0	0	0	not significant
8855	DARWICHE_PAPILLOMA_RISK_LOW_DN	0.68	0.01	0	0	0	not significant
8856	GAUSSMANN_MLL_AF4_FUSION_TARGETS_A_UP	0.68	0.01	0	0	0	not significant
8857	GO_CENTRIOLE	0.68	0.01	0	0	0	not significant
8858	GO_PROTEIN_MONOUBIQUITINATION	0.68	0.01	0	0	0	not significant
8859	GO_STEROID_HORMONE_RECEPTOR_BINDING	0.68	0.01	0	0	0	not significant
8860	HOSHIDA_LIVER_CANCER_SUBCLASS_S2	0.68	0.01	0	0	0	not significant
8861	NIKOLSKY_BREAST_CANCER_20Q12_Q13_AMPLICON	0.68	0.01	0	0	0	not significant
8862	GO_DOUBLE_STRAND_BREAK_REPAIR	0.68	0.00	0	0	0	not significant
8863	GO_MUTLALPHA_COMPLEX	0.69	0.09	0	0	0	not significant
8864	GO_THIAMINE_PYROPHOSPHATE_BINDING	0.69	0.09	0	0	0	not significant
8865	GO_ENDOSOMAL_VESICLE_FUSION	0.69	0.08	0	0	0	not significant
8866	GO_GLYCOSYLCERAMIDE_CATABOLIC_PROCESS	0.69	0.08	0	0	0	not significant
8867	GO_HYPOTONIC_RESPONSE	0.69	0.08	0	0	0	not significant
8868	GO_LYSINE_CATABOLIC_PROCESS	0.69	0.08	0	0	0	not significant
8869	GO_MITOTIC_SPINDLE_ASTRAL_MICROTUBULE	0.69	0.08	0	0	0	not significant
8870	GO_POSITIVE_REGULATION_OF_SYNAPTIC_VESICLE_TRANSPORT	0.69	0.08	0	0	0	not significant
8871	GO_RADIAL_GLIAL_CELL_DIFFERENTIATION	0.69	0.08	0	0	0	not significant
8872	SABATES_COLORECTAL_ADENOMA_SIZE_DN	0.69	0.08	0	0	0	not significant
8873	BIOCARTA_TERC_PATHWAY	0.69	0.07	0	0	0	not significant
8874	GO_GAMMA_SECRETASE_COMPLEX	0.69	0.07	0	0	0	not significant
8875	GO_HETEROCHROMATIN_ASSEMBLY	0.69	0.07	0	0	0	not significant
8876	GO_HYPEROSMOTIC_RESPONSE	0.69	0.07	0	0	0	not significant
8877	GO_MICROTUBULE_ANCHORING_AT_CENTROSOME	0.69	0.07	0	0	0	not significant
8878	GO_MYOBLAST_FUSION	0.69	0.07	0	0	0	not significant
8879	GO_NEGATIVE_REGULATION_OF_MORPHOGENESIS_OF_AN_EPITHELIUM	0.69	0.07	0	0	0	not significant
8880	GO_REGULATION_OF_AMYLOID_BETA_FORMATION	0.69	0.07	0	0	0	not significant
8881	GO_SYNAPTIC_MEMBRANE_ADHESION	0.69	0.07	0	0	0	not significant
8882	MEISSNER_BRAIN_HCP_WITH_H3_UNMETHYLATED	0.69	0.07	0	0	0	not significant
8883	GO_POSITIVE_REGULATION_OF_CHROMATIN_SILENCING	0.69	0.06	0	0	0	not significant
8884	GO_REGULATION_OF_TELOMERE_CAPPING	0.69	0.06	0	0	0	not significant
8885	CHEOK_RESPONSE_TO_HD_MTX_DN	0.69	0.05	0	0	0	not significant
8886	GO_MAST_CELL_ACTIVATION	0.69	0.05	0	0	0	not significant
8887	GRASEMANN_RETINOBLASTOMA_WITH_6P_AMPLIFICATION	0.69	0.05	0	0	0	not significant
8888	KYNG_ENVIRONMENTAL_STRESS_RESPONSE_NOT_BY_GAMMA_IN_OLD	0.69	0.05	0	0	0	not significant
8889	LUI_THYROID_CANCER_CLUSTER_2	0.69	0.05	0	0	0	not significant
8890	GO_ADENYLATE_CYCLASE_ACTIVATING_G_PROTEIN_COUPLED_RECEP	0.69	0.04	0	0	0	not significant
8891	GO_DEMETHYLATION	0.69	0.04	0	0	0	not significant
8892	KOHOUTEK_CCNT2_TARGETS	0.69	0.04	0	0	0	not significant
8893	REACTOME_NOTCH_HLH_TRANSCRIPTION_PATHWAY	0.69	0.04	0	0	0	not significant
8894	CHEN_HOXA5_TARGETS_9HR_DN	0.69	0.03	0	0	0	not significant
8895	GO_DENDRITIC_SPINE_MORPHOGENESIS	0.69	0.03	0	0	0	not significant
8896	PID_TXA2PATHWAY	0.69	0.03	0	0	0	not significant
8897	GO_BLASTOCYST_DEVELOPMENT	0.69	0.02	0	0	0	not significant
8898	GO_ENDOPLASMIC_RETICULUM_GOLGI_INTERMEDIATE_COMPARTMENT	0.69	0.01	0	0	0	not significant
8899	GO_GUANYL_NUCLEOTIDE_EXCHANGE_FACTOR_ACTIVITY	0.69	0.01	0	0	0	not significant
8900	GO_RAS_GUANYL_NUCLEOTIDE_EXCHANGE_FACTOR_ACTIVITY	0.69	0.01	0	0	0	not significant
8901	CLIMENT_BREAST_CANCER_COPY_NUMBER_DN	0.70	0.10	0	0	0	not significant
8902	BIOCARTA_PLK3_PATHWAY	0.70	0.09	0	0	0	not significant
8903	GO_CUL7_RING_UBIQUITIN_LIGASE_COMPLEX	0.70	0.09	0	0	0	not significant
8904	GO_DENDRITIC_GROWTH_CONE	0.70	0.09	0	0	0	not significant
8905	GO_PROTEIN_GERANYLGERANYLTRANSFERASE_ACTIVITY	0.70	0.09	0	0	0	not significant
8906	GO_REGULATION_OF_PLATELET_AGGREGATION	0.70	0.09	0	0	0	not significant
8907	REACTOME_LYSINE_CATABOLISM	0.70	0.09	0	0	0	not significant
8908	GO_METANEPHRIC_COLLECTING_DUCT_DEVELOPMENT	0.70	0.08	0	0	0	not significant
8909	GO_NUCLEAR_ENVELOPE_LUMEN	0.70	0.08	0	0	0	not significant
8910	GO_PROTEIN_GLYCOSYLATION_IN_GOLGI	0.70	0.08	0	0	0	not significant
8911	GO_REGULATION_OF_MICROTUBULE_BINDING	0.70	0.08	0	0	0	not significant
8912	GO_RESPONSE_TO_VITAMIN_A	0.70	0.08	0	0	0	not significant
8913	GO_RISC_LOADING_COMPLEX	0.70	0.08	0	0	0	not significant
8914	GO_SERTOLI_CELL_DEVELOPMENT	0.70	0.08	0	0	0	not significant
8915	GO_SMALL_RNA_LOADING_ONTO_RISC	0.70	0.08	0	0	0	not significant
8916	CROONQUIST_STROMAL_STIMULATION_DN	0.70	0.07	0	0	0	not significant
8917	GO_COENZYME_TRANSMEMBRANE_TRANSPORTER_ACTIVITY	0.70	0.07	0	0	0	not significant
8918	GO_EXTRINSIC_COMPONENT_OF_SYNAPTIC_MEMBRANE	0.70	0.07	0	0	0	not significant
8919	GO_NEGATIVE_REGULATION_OF_ORGANIC_ACID_TRANSPORT	0.70	0.07	0	0	0	not significant
8920	GO_NEGATIVE_T_CELL_SELECTION	0.70	0.07	0	0	0	not significant
8921	GO_UBIQUITIN_SPECIFIC_PROTEASE_BINDING	0.70	0.07	0	0	0	not significant
8922	REACTOME_FCGR_ACTIVATION	0.70	0.07	0	0	0	not significant
8923	ABBUD_LIF_SIGNALING_1_DN	0.70	0.06	0	0	0	not significant
8924	CHR3Q23	0.70	0.06	0	0	0	not significant
8925	CHR4P14	0.70	0.06	0	0	0	not significant
8926	GO_PHOSPHOLIPID_TRANSLOCATING_ATPASE_ACTIVITY	0.70	0.06	0	0	0	not significant

8927	GO_REGULATION_OF_MEMBRANE_LIPID_DISTRIBUTION	0.70	0.06	0	0	0	not significant
8928	REACTOME_BETA_CATENIN_PHOSPHORYLATION_CASCADE	0.70	0.06	0	0	0	not significant
8929	REACTOME_METAL_ION_SLC_TRANSPORTERS	0.70	0.06	0	0	0	not significant
8930	GO_MULTIVESICULAR_BODY_ORGANIZATION	0.70	0.05	0	0	0	not significant
8931	GO_NEGATIVE_REGULATION_OF_CHEMOTAXIS	0.70	0.05	0	0	0	not significant
8932	GO_RNA_DESTABILIZATION	0.70	0.05	0	0	0	not significant
8933	GO_VENTRICULAR_SYSTEM_DEVELOPMENT	0.70	0.05	0	0	0	not significant
8934	MCDOWELL_ACUTE_LUNG_INJURY_UP	0.70	0.05	0	0	0	not significant
8935	REACTOME_DEADENYLATION_OF_MRNA	0.70	0.05	0	0	0	not significant
8936	REACTOME_SIGNALING_BY_NOTCH3	0.70	0.05	0	0	0	not significant
8937	WANG_TARGETS_OF_MLL_CBP_FUSION_UP	0.70	0.05	0	0	0	not significant
8938	ZHANG_INTERFERON_RESPONSE	0.70	0.05	0	0	0	not significant
8939	GO_PROTEIN_SELF_ASSOCIATION	0.70	0.04	0	0	0	not significant
8940	PID_BARD1_PATHWAY	0.70	0.04	0	0	0	not significant
8941	GO_REGULATION_OF_TUBE_SIZE	0.70	0.03	0	0	0	not significant
8942	GO_REGULATION_OF_CELL_PROJECTION_ASSEMBLY	0.70	0.02	0	0	0	not significant
8943	GO_SMOOTHENED_SIGNALING_PATHWAY	0.70	0.02	0	0	0	not significant
8944	GO_VESICLE_MEDIATED_TRANSPORT_TO_THE_PLASMA_MEMBRANE	0.70	0.02	0	0	0	not significant
8945	CHANDRAN_METASTASIS_UP	0.70	0.00	0	0	0	not significant
8946	GO_NEGATIVE_REGULATION_OF_MEIOTIC_NUCLEAR_DIVISION	0.71	0.10	0	0	0	not significant
8947	REACTOME ACTIVATION OF CASPASES THROUGH APOPTOSOME MEI	0.71	0.10	0	0	0	not significant
8948	BIOCARTA_IFNG_PATHWAY	0.71	0.09	0	0	0	not significant
8949	GO_1_PHOSPHATIDYLINOSITOL_4_PHOSPHATE_5_KINASE_ACTIVITY	0.71	0.09	0	0	0	not significant
8950	GO_NEGATIVE_REGULATION_OF_RIG_I_SIGNALING_PATHWAY	0.71	0.09	0	0	0	not significant
8951	GO_NUCLEOBASE_METABOLIC_PROCESS	0.71	0.09	0	0	0	not significant
8952	GO POSITIVE REGULATION OF VASCULAR ASSOCIATED SMOOTH MU	0.71	0.09	0	0	0	not significant
8953	REACTOME_NAPLUS_CL_DEPENDENT_NEUROTRANSMITTER_TRANSPOR	0.71	0.09	0	0	0	not significant
8954	BIOCARTA_TGFB_PATHWAY	0.71	0.08	0	0	0	not significant
8955	GO_ACTININ_BINDING	0.71	0.08	0	0	0	not significant
8956	GO_ENTEROENDOCRINE_CELL_DIFFERENTIATION	0.71	0.08	0	0	0	not significant
8957	GO_GLUCOSIDASE_ACTIVITY	0.71	0.08	0	0	0	not significant
8958	GO_REGULATION_OF_ENDODEOXYRIBONUCLEASE_ACTIVITY	0.71	0.08	0	0	0	not significant
8959	GO_REGULATION_OF_PROTEIN_DEUBIQUITINATION	0.71	0.08	0	0	0	not significant
8960	GO_REGULATION_OF_SNARE_COMPLEX_ASSEMBLY	0.71	0.08	0	0	0	not significant
8961	GO TYPE I TRANSFORMING GROWTH FACTOR BETA RECEPTOR BIN	0.71	0.08	0	0	0	not significant
8962	LEE_LIVER_CANCER_HEPATOBLAST	0.71	0.08	0	0	0	not significant
8963	REACTOME_BUTYROPHILIN_BTN_FAMILY_INTERACTIONS	0.71	0.08	0	0	0	not significant
8964	REACTOME_DEFECTS_IN_COBALAMIN_B12_METABOLISM	0.71	0.08	0	0	0	not significant
8965	REACTOME_ROLE_OF_LAT2_NTAL_LAB_ON_CALCIUM_MOBILIZATION	0.71	0.08	0	0	0	not significant
8966	SA_PROGRAMMED_CELL_DEATH	0.71	0.08	0	0	0	not significant
8967	GO_ENDOPLASMIC_RETICULUM_QUALITY_CONTROL_COMPARTMENT	0.71	0.07	0	0	0	not significant
8968	GO_MKS_COMPLEX	0.71	0.07	0	0	0	not significant
8969	GO_RECEPTOR_INHIBITOR_ACTIVITY	0.71	0.07	0	0	0	not significant
8970	GO REGULATION OF CARBOHYDRATE METABOLIC PROCESS BY REG	0.71	0.07	0	0	0	not significant
8971	GO_RESPONSE_TO_ANTIMETABOLITE	0.71	0.07	0	0	0	not significant
8972	GO_SYNAPTIC_VESICLE_CYTOSKELETAL_TRANSPORT	0.71	0.07	0	0	0	not significant
8973	GO_VIRAL_BUDDING	0.71	0.07	0	0	0	not significant
8974	HASLINGER_B_CLL_WITH_6Q21_DELETION	0.71	0.07	0	0	0	not significant
8975	JIANG_HYPOXIA_VIA_VHL	0.71	0.07	0	0	0	not significant
8976	MATZUK_EMBRYONIC_GERM_CELL	0.71	0.07	0	0	0	not significant
8977	REACTOME_DOWNSTREAM_SIGNALING_OF_ACTIVATED_FGFR4	0.71	0.07	0	0	0	not significant
8978	REACTOME_MISCELLANEOUS_TRANSPORT_AND_BINDING_EVENTS	0.71	0.07	0	0	0	not significant
8979	REACTOME_PHASE_0_RAPID_DEPOLARISATION	0.71	0.07	0	0	0	not significant
8980	REACTOME TRANSCRIPTION OF E2F TARGETS UNDER NEGATIVE CO	0.71	0.07	0	0	0	not significant
8981	SCHAEFFER_PROSTATE_DEVELOPMENT_AND_CANCER_BOX1_UP	0.71	0.07	0	0	0	not significant
8982	BIOCARTA_ATRBRCA_PATHWAY	0.71	0.06	0	0	0	not significant
8983	GO_CERAMIDE_METABOLIC_PROCESS	0.71	0.06	0	0	0	not significant
8984	GO_MICROTUBULE_NUCLEATION	0.71	0.06	0	0	0	not significant
8985	GO_MYELOID_LEUKOCYTE_CYTOKINE_PRODUCTION	0.71	0.06	0	0	0	not significant
8986	GO_REGULATION_OF_EXIT_FROM_MITOSIS	0.71	0.06	0	0	0	not significant
8987	PETRETTO_HEART_MASS_QTL_CIS_UP	0.71	0.06	0	0	0	not significant
8988	REACTOME_NRAGE_SIGNALS_DEATH_THROUGH_JNK	0.71	0.06	0	0	0	not significant
8989	ZHAN_MULTIPLE_MYELOMA_CD1_DN	0.71	0.06	0	0	0	not significant
8990	GO_CAVEOLA	0.71	0.05	0	0	0	not significant
8991	GO_ENDODERM_DEVELOPMENT	0.71	0.05	0	0	0	not significant
8992	GO_NEGATIVE_REGULATION_OF_BLOOD_CIRCULATION	0.71	0.05	0	0	0	not significant
8993	GO_REGULATION_OF_CELL_CYCLE_CHECKPOINT	0.71	0.05	0	0	0	not significant
8994	KEGG_MELANOMA	0.71	0.05	0	0	0	not significant
8995	KYNG_RESPONSE_TO_H2O2_VIA_ERCC6_DN	0.71	0.05	0	0	0	not significant
8996	REACTOME_SIGNALING_BY_ERBB2	0.71	0.05	0	0	0	not significant
8997	GO ATP HYDROLYSIS COUPLED CATION TRANSMEMBRANE TRANSPO	0.71	0.04	0	0	0	not significant
8998	REACTOME_REPRODUCTION	0.71	0.03	0	0	0	not significant
8999	GO_LATE_ENDOSOME_MEMBRANE	0.71	0.02	0	0	0	not significant
9000	GO_NEGATIVE_REGULATION_OF_GENE_EXPRESSION_EPIGENETIC	0.71	0.02	0	0	0	not significant
9001	IVANOVSKA_MIR106B_TARGETS	0.71	0.02	0	0	0	not significant
9002	GO_NEGATIVE_REGULATION_OF_NUCLEOBASE_CONTAINING_COMPOUN	0.71	0.01	0	0	0	not significant
9003	REACTOME_CELLULAR_SENESCENCE	0.71	0.01	0	0	0	not significant
9004	GO_CHROMATIN_ORGANIZATION	0.71	0.00	0	0	0	not significant
9005	GO_MICROTUBULE_ORGANIZING_CENTER	0.71	0.00	0	0	0	not significant
9006	GO_PYRIMIDINE_DEOXYRIBONUCLEOTIDE_BIOSYNTHETIC_PROCESS	0.72	0.10	0	0	0	not significant
9007	GO_Y_FORM_DNA_BINDING	0.72	0.10	0	0	0	not significant
9008	VANDESLUIS_COMMD1_TARGETS_GROUP_4_DN	0.72	0.10	0	0	0	not significant
9009	GO_DYNACTIN_BINDING	0.72	0.09	0	0	0	not significant
9010	GO NEGATIVE REGULATION OF CYTOKINE PRODUCTION INVOLVED IN	0.72	0.09	0	0	0	not significant
9011	GO_PROTEIN_AUTOPROCESSING	0.72	0.09	0	0	0	not significant
9012	GO_REGULATION_OF_METAPHASE_PLATE_CONGRESSION	0.72	0.09	0	0	0	not significant
9013	GO REGULATION OF VIRAL INDUCED CYTOPLASMIC PATTERN RECOG	0.72	0.09	0	0	0	not significant
9014	GO_RNA_POLYMERASE_III_PREINITIATION_COMPLEX_ASSEMBLY	0.72	0.09	0	0	0	not significant
9015	GO_SMAD_PROTEIN_COMPLEX_ASSEMBLY	0.72	0.09	0	0	0	not significant
9016	KONG_E2F1_TARGETS	0.72	0.09	0	0	0	not significant
9017	MIKKELSEN_PLURIPOTENT_STATE_DN	0.72	0.09	0	0	0	not significant
9018	BIOCARTA_PITX2_PATHWAY	0.72	0.08	0	0	0	not significant
9019	GO_ATPASE_DEPENDENT_TRANSMEMBRANE_TRANSPORT_COMPLEX	0.72	0.08	0	0	0	not significant
9020	GO_LIVER_MORPHOGENESIS	0.72	0.08	0	0	0	not significant
9021	GO_MICROVILLUS_MEMBRANE	0.72	0.08	0	0	0	not significant
9022	GO_NEGATIVE_REGULATION_OF_ANION_TRANSPORT	0.72	0.08	0	0	0	not significant
9023	GO REGULATION OF EPIDERMAL GROWTH FACTOR ACTIVATED REC	0.72	0.08	0	0	0	not significant
9024	GO_REGULATION_OF_PROTEIN_LOCALIZATION_TO_CENTROSOME	0.72	0.08	0	0	0	not significant
9025	GO_SEX_DETERMINATION	0.72	0.08	0	0	0	not significant
9026	REACTOME_DOWNSTREAM_SIGNALING_OF_ACTIVATED_FGFR2	0.72	0.08	0	0	0	not significant
9027	STARK_HYPPOCAMPUS_22Q11_DELETION_DN	0.72	0.08	0	0	0	not significant
9028	BIOCARTA_CHEMICAL_PATHWAY	0.72	0.07	0	0	0	not significant
9029	BIOCARTA_NFKB_PATHWAY	0.72	0.07	0	0	0	not significant
9030	CHR18Q12	0.72	0.07	0	0	0	not significant
9031	GO_ORGANIC_CATION_TRANSPORT	0.72	0.07	0	0	0	not significant
9032	GO_PYRIMIDINE_CONTAINING_COMPOUND_METABOLIC_PROCESS	0.72	0.07	0	0	0	not significant
9033	GO_REGULATION_OF_FATTY_ACID_OXIDATION	0.72	0.07	0	0	0	not significant
9034	PID_A6B1_A6B4_INTEGRIN_PATHWAY	0.72	0.07	0	0	0	not significant
9035	REACTOME_TP53_REGULATES_TRANSCRIPTION_OF_ADDITIONAL_CELL	0.72	0.07	0	0	0	not significant

9036	CHRXQ22	0.72	0.06	0	0	0	not significant
9037	GO_S_ACYLTRANSFERASE_ACTIVITY	0.72	0.06	0	0	0	not significant
9038	REACTOME_IRAK1_RECRUITS_IKK_COMPLEX	0.72	0.06	0	0	0	not significant
9039	AMIT_EGF_RESPONSE_240_HELA	0.72	0.05	0	0	0	not significant
9040	CHEN_NEUROBLASTOMA_COPY_NUMBER_GAINS	0.72	0.05	0	0	0	not significant
9041	GO_CENTRIOLE_ASSEMBLY	0.72	0.05	0	0	0	not significant
9042	GO_ENDOPLASMIC_RETICULUM_GOLGI_INTERMEDIATE_COMPARTMENT_	0.72	0.05	0	0	0	not significant
9043	GO_NEUTROPHIL_MIGRATION	0.72	0.05	0	0	0	not significant
9044	JAZAG_TGFB1_SIGNALING_VIA_SMAD4_DN	0.72	0.05	0	0	0	not significant
9045	PID_TRKR_PATHWAY	0.72	0.05	0	0	0	not significant
9046	GO_ACTIVATING_TRANSCRIPTION_FACTOR_BINDING	0.72	0.04	0	0	0	not significant
9047	GO_REGULATION_OF_HISTONE_METHYLATION	0.72	0.04	0	0	0	not significant
9048	OUILLETTE_CLL_13Q14_DELETION_UP	0.72	0.04	0	0	0	not significant
9049	PID_P75_NTR_PATHWAY	0.72	0.04	0	0	0	not significant
9050	GO_ALTERNATIVE_MRNA_SPLICING_VIA_SPLICEOSOME	0.72	0.03	0	0	0	not significant
9051	DARWICHE_SKIN_TUMOR_PROMOTER_DN	0.72	0.02	0	0	0	not significant
9052	GO_FC_RECEPTOR_MEDIATED_STIMULATORY_SIGNALING_PATHWAY	0.72	0.02	0	0	0	not significant
9053	WEI_MIR34A_TARGETS	0.72	0.02	0	0	0	not significant
9054	GABRIELY_MIR21_TARGETS	0.72	0.00	0	0	0	not significant
9055	GO_PROCESS_UTILIZING_AUTOPHAGIC_MECHANISM	0.72	0.00	0	0	0	not significant
9056	REACTOME_IL_6_TYPE_CYTOKINE_RECEPTOR_LIGAND_INTERACTIONS	0.73	0.12	0	0	0	not significant
9057	GO_NEGATIVE_REGULATION_OF_SMOOTH_MUSCLE_CELL_DIFFERENTIA	0.73	0.11	0	0	0	not significant
9058	GO_RNA_POLYMERASE_III_TYPE_1_PROMOTER_DNA_BINDING	0.73	0.11	0	0	0	not significant
9059	MYLLYKANGAS_AMPLIFICATION_HOT_SPOT_25	0.73	0.11	0	0	0	not significant
9060	CHR3P13	0.73	0.10	0	0	0	not significant
9061	GO_MAINTENANCE_OF_APICAL_BASAL_CELL_POLARITY	0.73	0.10	0	0	0	not significant
9062	GO_MUCUS_SECRETION	0.73	0.10	0	0	0	not significant
9063	GO_NURF_COMPLEX	0.73	0.10	0	0	0	not significant
9064	GO_REGULATION_OF_PROTEIN_LOCALIZATION_TO_ENDOSOME	0.73	0.10	0	0	0	not significant
9065	GO_SIGNAL_COMPLEX_ASSEMBLY	0.73	0.10	0	0	0	not significant
9066	HOWLIN_CITED1_TARGETS_2_UP	0.73	0.10	0	0	0	not significant
9067	REACTOME_ACYL_CHAIN_REMODELLING_OF_PG	0.73	0.10	0	0	0	not significant
9068	GENTILE_UV_HIGH_DOSE_UP	0.73	0.09	0	0	0	not significant
9069	GO_ATTACHMENT_OF_MITOTIC_SPINDLE_MICROTUBULES_TO_KINETOC	0.73	0.09	0	0	0	not significant
9070	GO_KERATINOCYTE_PROLIFERATION	0.73	0.09	0	0	0	not significant
9071	GO_MITOCHONDRION_DISTRIBUTION	0.73	0.09	0	0	0	not significant
9072	GO_NEGATIVE_REGULATION_OF_BONE_MINERALIZATION	0.73	0.09	0	0	0	not significant
9073	GO_OXIDOREDUCTASE_ACTIVITY_ACTING_ON_A_SULFUR_GROUP_OF_I	0.73	0.09	0	0	0	not significant
9074	GO_OXOGLUTARATE_DEHYDROGENASE_COMPLEX	0.73	0.09	0	0	0	not significant
9075	GO_PROTEIN_LOCALIZATION_TO_KINETOCHORE	0.73	0.09	0	0	0	not significant
9076	GO_UBIQUITIN_UBIQUITIN_LIGASE_ACTIVITY	0.73	0.09	0	0	0	not significant
9077	GROSS_HYPOXIA_VIA_HIF1A_ONLY	0.73	0.09	0	0	0	not significant
9078	NIELSEN_SYNOVIAL_SARCOMA_UP	0.73	0.09	0	0	0	not significant
9079	REACTOME_MYOCLONIC_EPILEPSY_OF_LAFORA	0.73	0.09	0	0	0	not significant
9080	TORCHIA_TARGETS_OF_EWSR1_FLI1_FUSION_TOP20_DN	0.73	0.09	0	0	0	not significant
9081	GO_G_PROTEIN_COUPLED_RECEPTOR_INTERNALIZATION	0.73	0.08	0	0	0	not significant
9082	GO_MEMBRANE_PROTEIN_INTRACELLULAR_DOMAIN_PROTEOLYSIS	0.73	0.08	0	0	0	not significant
9083	GO_NBAF_COMPLEX	0.73	0.08	0	0	0	not significant
9084	GO_PRIMITIVE_HEMOPOIESIS	0.73	0.08	0	0	0	not significant
9085	GO_REGULATION_OF_EXECUTION_PHASE_OF_APOPTOSIS	0.73	0.08	0	0	0	not significant
9086	GO_REGULATION_OF_INTEGRIN_ACTIVATION	0.73	0.08	0	0	0	not significant
9087	SCHLINGEMANN_SKIN_CARCINOGENESIS_TPA_DN	0.73	0.08	0	0	0	not significant
9088	GO_HISTONE_H3_K9_METHYLATION	0.73	0.06	0	0	0	not significant
9089	GO_INTERSTRAND_CROSS_LINK_REPAIR	0.73	0.06	0	0	0	not significant
9090	KEGG_NOD_LIKE_RECEPTOR_SIGNALING_PATHWAY	0.73	0.06	0	0	0	not significant
9091	GO_DNA_DEPENDENT_DNA_REPLICATION_MAINTENANCE_OF_FIDELITY	0.73	0.05	0	0	0	not significant
9092	REACTOME_HDR_THROUGH_SINGLE_STRAND_ANNEALING_SSA	0.73	0.05	0	0	0	not significant
9093	BROWNE_HCMV_INFECTION_8HR_UP	0.73	0.04	0	0	0	not significant
9094	GO_MYOTUBE_DIFFERENTIATION	0.73	0.04	0	0	0	not significant
9095	GO_REGULATION_OF_MEMBRANE_PERMEABILITY	0.73	0.04	0	0	0	not significant
9096	CHR1Q32	0.73	0.03	0	0	0	not significant
9097	GO_POSITIVE_REGULATION_OF_CHROMATIN_ORGANIZATION	0.73	0.03	0	0	0	not significant
9098	DAZARD_RESPONSE_TO_UV_SCC_DN	0.73	0.02	0	0	0	not significant
9099	GO_PRESYNAPSE	0.73	0.01	0	0	0	not significant
9100	GO_COVALENT_CHROMATIN_MODIFICATION	0.73	0.00	0	0	0	not significant
9101	LASTOWSKA_NEUROBLASTOMA_COPY_NUMBER_DN	0.73	0.00	0	0	0	not significant
9102	MILI_PSEUDOPODIA_HAPTOTAXIS_DN	0.73	0.00	0	0	0	not significant
9103	GO_CDP_ALCOHOL_PHOSPHATIDYLTRANSFERASE_ACTIVITY	0.74	0.13	0	0	0	not significant
9104	GO_CUL2_RING_UBIQUITIN_LIGASE_COMPLEX	0.74	0.12	0	0	0	not significant
9105	GO_INTERLEUKIN_4_MEDIATED_SIGNALING_PATHWAY	0.74	0.11	0	0	0	not significant
9106	GO_REGULATION_OF_DENDRITIC_CELL_DIFFERENTIATION	0.74	0.11	0	0	0	not significant
9107	GO_T_CELL_TOLERANCE_INDUCTION	0.74	0.11	0	0	0	not significant
9108	CHANG_POU5F1_TARGETS_DN	0.74	0.10	0	0	0	not significant
9109	GO_DNA_N_GLYCOSYLASE_ACTIVITY	0.74	0.10	0	0	0	not significant
9110	GO_LEPTIN_MEDIATED_SIGNALING_PATHWAY	0.74	0.10	0	0	0	not significant
9111	GO_MESENCHYMAL_TO_EPITHELIAL_TRANSITION_INVOLVED_IN_METANE	0.74	0.10	0	0	0	not significant
9112	GO_NEGATIVE_REGULATION_OF_UBIQUITIN_PROTEIN_LIGASE_ACTIVITY	0.74	0.10	0	0	0	not significant
9113	GO_PHOSPHATIDYLINOSITOL_KINASE_ACTIVITY	0.74	0.10	0	0	0	not significant
9114	GO_POSITIVE_REGULATION_OF_RECEPTOR_INTERNALIZATION	0.74	0.10	0	0	0	not significant
9115	PID_ALK2_PATHWAY	0.74	0.10	0	0	0	not significant
9116	REACTOME_KSRP_KHSRP_BINDS_AND_DESTABILIZES_MRNA	0.74	0.10	0	0	0	not significant
9117	REACTOME_NOTCH4_ACTIVATION_AND_TRANSMISSION_OF_SIGNAL_TO_	0.74	0.10	0	0	0	not significant
9118	REACTOME_RUNX3_REGULATES_WNT_SIGNALING	0.74	0.10	0	0	0	not significant
9119	REACTOME_SOS_MEDIATED_SIGNALLING	0.74	0.10	0	0	0	not significant
9120	GO_DIOL_BIOSYNTHETIC_PROCESS	0.74	0.09	0	0	0	not significant
9121	KYNG_ENVIRONMENTAL_STRESS_RESPONSE_NOT_BY_UV_IN_OLD	0.74	0.09	0	0	0	not significant
9122	PIONTEK_PKD1_TARGETS_UP	0.74	0.09	0	0	0	not significant
9123	REACTOME_FRS_MEDIATED_FGFR3_SIGNALING	0.74	0.09	0	0	0	not significant
9124	WEST_ADRENOCORTICAL_CARCINOMA_VS_ADENOMA_DN	0.74	0.09	0	0	0	not significant
9125	CHR3P24	0.74	0.08	0	0	0	not significant
9126	GO_AMYLOID_BETA_FORMATION	0.74	0.08	0	0	0	not significant
9127	GO_PHENOL_CONTAINING_COMPOUND_METABOLIC_PROCESS	0.74	0.08	0	0	0	not significant
9128	GO_REGULATION_OF_EXTRACELLULAR_MATRIX_ORGANIZATION	0.74	0.08	0	0	0	not significant
9129	GO_REGULATION_OF_PROTEIN_EXIT_FROM_ENDOPLASMIC_RETICULUM	0.74	0.08	0	0	0	not significant
9130	WONG_IFNA2_RESISTANCE_UP	0.74	0.08	0	0	0	not significant
9131	XU_CREBBP_TARGETS_UP	0.74	0.08	0	0	0	not significant
9132	GO_O_ACYLTRANSFERASE_ACTIVITY	0.74	0.07	0	0	0	not significant
9133	GO_PROTEIN_INSERTION_INTO_MITOCHONDRIAL_MEMBRANE_INVOLVED	0.74	0.07	0	0	0	not significant
9134	HU_GENOTOXIN_ACTION_DIRECT_VS_INDIRECT_4HR	0.74	0.07	0	0	0	not significant
9135	MIZUSHIMA_AUTOPHAGOSOME_FORMATION	0.74	0.07	0	0	0	not significant
9136	REACTOME_NEGATIVE_REGULATION_OF_FGFR3_SIGNALING	0.74	0.07	0	0	0	not significant
9137	SCHUETZ_BREAST_CANCER_DUCTAL_INVASIVE_DN	0.74	0.07	0	0	0	not significant
9138	SHIPP_DLBCL_CURED_VS_FATAL_DN	0.74	0.07	0	0	0	not significant
9139	GO_DNA_CATABOLIC_PROCESS	0.74	0.06	0	0	0	not significant
9140	GO_HISTONE_UBIQUITINATION	0.74	0.06	0	0	0	not significant
9141	GO_NEGATIVE_REGULATION_OF_MUSCLE_TISSUE_DEVELOPMENT	0.74	0.06	0	0	0	not significant
9142	REACTOME_FANCONI_ANEMIA_PATHWAY	0.74	0.06	0	0	0	not significant
9143	GO_DNA_METHYLATION	0.74	0.05	0	0	0	not significant
9144	REACTOME_CELL_DEATH_SIGNALLING_VIA_NRAGE_NRIF_AND_NADE	0.74	0.05	0	0	0	not significant

9145	ROPERO_HDAC2_TARGETS	0.74	0.05	0	0	0	not significant
9146	GO_CARBOXYLIC_ESTER_HYDROLASE_ACTIVITY	0.74	0.04	0	0	0	not significant
9147	GO_MORPHOGENESIS_OF_EMBRYONIC_EPITHELIUM	0.74	0.04	0	0	0	not significant
9148	GO_SH3_DOMAIN_BINDING	0.74	0.04	0	0	0	not significant
9149	JNK_DN.V1_UP	0.74	0.04	0	0	0	not significant
9150	VERHAAK_GLIOBLASTOMA_NEURAL	0.74	0.04	0	0	0	not significant
9151	GO_NEGATIVE_REGULATION_OF_CELL_GROWTH	0.74	0.03	0	0	0	not significant
9152	THUM_SYSTOLIC_HEART_FAILURE_DN	0.74	0.02	0	0	0	not significant
9153	GO_UBIQUITIN_LIGASE_COMPLEX	0.74	0.00	0	0	0	not significant
9154	GO_MUCOPOLYSACCHARIDE_METABOLIC_PROCESS	0.75	0.13	0	0	0	not significant
9155	GO_MITOCHONDRIAL_ALPHA_KETOGLUTARATE_DEHYDROGENASE_COM	0.75	0.12	0	0	0	not significant
9156	GO_MUSCLE_MYOSIN_COMPLEX	0.75	0.12	0	0	0	not significant
9157	GO_NEGATIVE_REGULATION_OF_EXECUTION_PHASE_OF_APOPTOSIS	0.75	0.12	0	0	0	not significant
9158	GO_PHOSPHATIDYLINOSITOL_TRANSPORTER_ACTIVITY	0.75	0.12	0	0	0	not significant
9159	GO_ARF_GUANYL_NUCLEOTIDE_EXCHANGE_FACTOR_ACTIVITY	0.75	0.11	0	0	0	not significant
9160	GO_GLYCEROPHOSPHOLIPID_CATABOLIC_PROCESS	0.75	0.11	0	0	0	not significant
9161	GO_IRON_ION_TRANSMEMBRANE_TRANSPORT	0.75	0.11	0	0	0	not significant
9162	GO_MDA_5_SIGNALING_PATHWAY	0.75	0.11	0	0	0	not significant
9163	GO_POSITIVE_REGULATION_OF_STRIATED_MUSCLE_CELL_APOPTOTIC	0.75	0.11	0	0	0	not significant
9164	GO_REGULATION_OF_CYTOPLASMIC_TRANSLATIONAL_INITIATION	0.75	0.11	0	0	0	not significant
9165	GO_REGULATION_OF_DENDRITE_EXTENSION	0.75	0.11	0	0	0	not significant
9166	ABDELMOHSEN_ELAVL4_TARGETS	0.75	0.10	0	0	0	not significant
9167	CHR6Q27	0.75	0.10	0	0	0	not significant
9168	GO_CELL_DIFFERENTIATION_INVOLVED_IN_METANEPHROS_DEVELOPME	0.75	0.10	0	0	0	not significant
9169	GO_ENDODEOXYRIBONUCLEASE_ACTIVITY_PRODUCING_3_PHOSPHOMC	0.75	0.10	0	0	0	not significant
9170	GO_FIBROBLAST_ACTIVATION	0.75	0.10	0	0	0	not significant
9171	GO_NEURON_RECOGNITION	0.75	0.10	0	0	0	not significant
9172	GO_POSITIVE_REGULATION_OF_MITOCHONDRIAL_FISSION	0.75	0.10	0	0	0	not significant
9173	GO_POSITIVE_REGULATION_OF_MITOTIC_SISTER_CHROMATID_SEGREG	0.75	0.10	0	0	0	not significant
9174	GO_POSITIVE_REGULATION_OF_T_CELL_RECEPTOR_SIGNALING_PATHW	0.75	0.10	0	0	0	not significant
9175	GO_RAP_PROTEIN_SIGNAL_TRANSDUCTION	0.75	0.10	0	0	0	not significant
9176	GO_REGULATION_OF_FC_RECEPTOR_MEDIATED_STIMULATORY_SIGNA	0.75	0.10	0	0	0	not significant
9177	GO_REGULATION_OF_TUBULIN_DEACETYLATION	0.75	0.10	0	0	0	not significant
9178	GO_RESPONSE_TO_SALT	0.75	0.10	0	0	0	not significant
9179	GO_SODIUM_ION_BINDING	0.75	0.10	0	0	0	not significant
9180	MIKKELSEN_IPS_LCP_WITH_H3K27ME3	0.75	0.10	0	0	0	not significant
9181	REACTOME_P38MAPK_EVENTS	0.75	0.10	0	0	0	not significant
9182	GO_AMYLOID_BETA_CLEARANCE	0.75	0.09	0	0	0	not significant
9183	GO_EATING_BEHAVIOR	0.75	0.09	0	0	0	not significant
9184	GO_MULTIVESICULAR_BODY	0.75	0.09	0	0	0	not significant
9185	LIU_SMARCA4_TARGETS	0.75	0.09	0	0	0	not significant
9186	GO_DNA_POLYMERASE_ACTIVITY	0.75	0.08	0	0	0	not significant
9187	GO_MYD88_INDEPENDENT_TOLL_LIKE_RECEPTOR_SIGNALING_PATHWA	0.75	0.08	0	0	0	not significant
9188	GO_REGULATION_OF_MAST_CELL_ACTIVATION	0.75	0.08	0	0	0	not significant
9189	GO_VENTRICULAR_SEPTUM_MORPHOGENESIS	0.75	0.08	0	0	0	not significant
9190	SCHAEFFER_SOX9_TARGETS_IN_PROSTATE_DEVELOPMENT_DN	0.75	0.08	0	0	0	not significant
9191	WAKABAYASHI_ADIPOGENESIS_PPARG_BOUND_36HR	0.75	0.08	0	0	0	not significant
9192	GO_NEGATIVE_REGULATION_OF_AXONOGENESIS	0.75	0.07	0	0	0	not significant
9193	GO_POSITIVE_REGULATION_OF_MUSCLE_CELL_DIFFERENTIATION	0.75	0.07	0	0	0	not significant
9194	REACTOME_OVARIAN_TUMOR_DOMAIN_PROTEASES	0.75	0.07	0	0	0	not significant
9195	ROESSLER_LIVER_CANCER_METASTASIS_DN	0.75	0.07	0	0	0	not significant
9196	BROWNE_HCMV_INFECTION_10HR_UP	0.75	0.05	0	0	0	not significant
9197	CAMPS_COLON_CANCER_COPY_NUMBER_UP	0.75	0.05	0	0	0	not significant
9198	GO_FEMALE_GAMETE_GENERATION	0.75	0.05	0	0	0	not significant
9199	GO_NEGATIVE_REGULATION_OF_PROTEOLYSIS_INVOLVED_IN_CELLULA	0.75	0.05	0	0	0	not significant
9200	GO_POSITIVE_REGULATION_OF_UBIQUITIN_DEPENDENT_PROTEIN_CATA	0.75	0.05	0	0	0	not significant
9201	GO_PROTEIN_LOCALIZATION_TO_CYTOSKELETON	0.75	0.05	0	0	0	not significant
9202	GO_REGULATION_OF_CALCIUM_ION_DEPENDENT_EXOCYTOSIS	0.75	0.05	0	0	0	not significant
9203	GO_CYSTEINE_TYPE_ENDOPEPTIDASE_ACTIVITY	0.75	0.04	0	0	0	not significant
9204	GO_ERAD_PATHWAY	0.75	0.04	0	0	0	not significant
9205	GO_NEGATIVE_REGULATION_OF_INFLAMMATORY_RESPONSE	0.75	0.04	0	0	0	not significant
9206	GO_CELLULAR_RESPONSE_TO_TOPOLOGICALLY_INCORRECT_PROTEIN	0.75	0.03	0	0	0	not significant
9207	CHR1P36	0.75	0.01	0	0	0	not significant
9208	GO_SMALL_GTPASE_BINDING	0.75	0.01	0	0	0	not significant
9209	GO_BLOOD_VESSEL_REMODELING	0.76	0.13	0	0	0	not significant
9210	GO_NEGATIVE_REGULATION_OF_ACTIN_NUCLEATION	0.76	0.13	0	0	0	not significant
9211	REACTOME_TRIF_MEDIATED_PROGRAMMED_CELL_DEATH	0.76	0.13	0	0	0	not significant
9212	GO_NEGATIVE_REGULATION_OF_HOMOTYPIC_CELL_CELL_ADHESION	0.76	0.12	0	0	0	not significant
9213	GO_PROTEIN_PRENYLTRANSFERASE_ACTIVITY	0.76	0.12	0	0	0	not significant
9214	GO_RNA_POLYMERASE_I_CORE_BINDING	0.76	0.12	0	0	0	not significant
9215	REACTOME_ACTIVATION_OF_RAC1	0.76	0.12	0	0	0	not significant
9216	BIOCARTA_EICOSANOID_PATHWAY	0.76	0.11	0	0	0	not significant
9217	GO_ANTIBIOTIC_BIOSYNTHETIC_PROCESS	0.76	0.11	0	0	0	not significant
9218	GO_BASAL_PLASMA_MEMBRANE	0.76	0.11	0	0	0	not significant
9219	GO_COLLECTING_DUCT_DEVELOPMENT	0.76	0.11	0	0	0	not significant
9220	GO_DIACYLGLYCEROL_KINASE_ACTIVITY	0.76	0.11	0	0	0	not significant
9221	GO_EXCITATORY_EXTRACELLULAR_LIGAND_GATED_ION_CHANNEL_ACT	0.76	0.11	0	0	0	not significant
9222	GO_KERATINOCYTE_DEVELOPMENT	0.76	0.11	0	0	0	not significant
9223	GO_POSITIVE_REGULATION_OF_FC_RECEPTOR_MEDIATED_STIMULATO	0.76	0.11	0	0	0	not significant
9224	GO_POSTSYNAPTIC_NEUROTRANSMITTER_RECEPTOR_INTERNALIZATIO	0.76	0.11	0	0	0	not significant
9225	GO_PROTEIN_IMPORT_INTO_PEROXISOME_MEMBRANE	0.76	0.11	0	0	0	not significant
9226	GO_REGULATION_OF_AXON_GUIDANCE	0.76	0.11	0	0	0	not significant
9227	GO_REGULATION_OF_EXOSOMAL_SECRETION	0.76	0.11	0	0	0	not significant
9228	GO_TRIGLYCERIDE_RICH_PLASMA_LIPOPROTEIN_PARTICLE	0.76	0.11	0	0	0	not significant
9229	GO_UDP_GALACTOSYLTRANSFERASE_ACTIVITY	0.76	0.11	0	0	0	not significant
9230	LI_CISPLATIN_RESISTANCE_UP	0.76	0.11	0	0	0	not significant
9231	REACTOME_ACTIVATION_OF_SMO	0.76	0.11	0	0	0	not significant
9232	REACTOME_JNK_C_JUN_KINASES_PHOSPHORYLATION_AND_ACTIVATION	0.76	0.11	0	0	0	not significant
9233	SMID_BREAST_CANCER_RELAPSE_IN_LUNG_DN	0.76	0.11	0	0	0	not significant
9234	BIOCARTA_41BB_PATHWAY	0.76	0.10	0	0	0	not significant
9235	BIOCARTA_RACCYCD_PATHWAY	0.76	0.10	0	0	0	not significant
9236	BONOME_OVARIAN_CANCER_POOR_SURVIVAL_UP	0.76	0.10	0	0	0	not significant
9237	CHR21Q21	0.76	0.10	0	0	0	not significant
9238	CHR2Q23	0.76	0.10	0	0	0	not significant
9239	FIGUEROA_AML_METHYLATION_CLUSTER_3_DN	0.76	0.10	0	0	0	not significant
9240	GO_ACETYLGALACTOSAMINYLTRANSFERASE_ACTIVITY	0.76	0.10	0	0	0	not significant
9241	GO_AORTA_DEVELOPMENT	0.76	0.10	0	0	0	not significant
9242	GO_CATALYTIC_ACTIVITY_ACTING_ON_A_GLYCOPROTEIN	0.76	0.10	0	0	0	not significant
9243	GO_MESENCHYMAL_TO_EPITHELIAL_TRANSITION	0.76	0.10	0	0	0	not significant
9244	GO_NEUROTRANSMITTER_RECEPTOR_INTERNALIZATION	0.76	0.10	0	0	0	not significant
9245	GO_POSITIVE_THYMIC_T_CELL_SELECTION	0.76	0.10	0	0	0	not significant
9246	GO_REGULATION_OF_PLATELET_DERIVED_GROWTH_FACTOR_RECEPT	0.76	0.10	0	0	0	not significant
9247	GO_TETRAPYRROLE_METABOLIC_PROCESS	0.76	0.10	0	0	0	not significant
9248	REACTOME_BUTYRATE_RESPONSE_FACTOR_1_BRF1_BINDS_AND_DES	0.76	0.10	0	0	0	not significant
9249	CASORELLI_APL_SECONDARY_VS_DE_NOVO_UP	0.76	0.09	0	0	0	not significant
9250	CERIBELLI_GENES_INACTIVE_AND_BOUND_BY_NFY	0.76	0.09	0	0	0	not significant
9251	CHANDRAN_METASTASIS_TOP50_UP	0.76	0.09	0	0	0	not significant
9252	GO_AGGRESOME	0.76	0.09	0	0	0	not significant
9253	GO_AXONAL_GROWTH_CONE	0.76	0.09	0	0	0	not significant

9254	GO_HEME_BIOSYNTHETIC_PROCESS	0.76	0.09	0	0	0	not significant
9255	GO_REGULATION_OF_TRANSCRIPTION_BY_RNA_POLYMERASE_III	0.76	0.09	0	0	0	not significant
9256	REACTOME_INSULIN_PROCESSING	0.76	0.09	0	0	0	not significant
9257	SHAFFER_IRF4_MULTIPLE_MYELOMA_PROGRAM	0.76	0.09	0	0	0	not significant
9258	WU_HBX_TARGETS_2_UP	0.76	0.09	0	0	0	not significant
9259	GO_ENDOCYTIC_RECYCLING	0.76	0.08	0	0	0	not significant
9260	GO_HISTONE_MONOUBIQUITINATION	0.76	0.08	0	0	0	not significant
9261	GO_NEGATIVE_REGULATION_OF_RESPONSE_TO_ENDOPLASMIC_RETICU	0.76	0.08	0	0	0	not significant
9262	PID_PI3K_PLC_TRK_PATHWAY	0.76	0.08	0	0	0	not significant
9263	GO_NUCLEOSIDE_MONOPHOSPHATE_BIOSYNTHETIC_PROCESS	0.76	0.07	0	0	0	not significant
9264	GO_OOGENESIS	0.76	0.07	0	0	0	not significant
9265	JOHNSTONE_PARVB_TARGETS_1_DN	0.76	0.07	0	0	0	not significant
9266	LUI_TARGETS_OF_PAX8_PPARG_FUSION	0.76	0.07	0	0	0	not significant
9267	PID_AR_PATHWAY	0.76	0.07	0	0	0	not significant
9268	ZHAN_MULTIPLE_MYELOMA_MF_DN	0.76	0.07	0	0	0	not significant
9269	GO_CELL_RECOGNITION	0.76	0.06	0	0	0	not significant
9270	GO_GLUCAN_METABOLIC_PROCESS	0.76	0.06	0	0	0	not significant
9271	GO_REGULATION_OF_DENDRITIC_SPINE_DEVELOPMENT	0.76	0.06	0	0	0	not significant
9272	KEGG_TGF_BETA_SIGNALING_PATHWAY	0.76	0.06	0	0	0	not significant
9273	KEGG_TOLL_LIKE_RECEPTOR_SIGNALING_PATHWAY	0.76	0.06	0	0	0	not significant
9274	PID_TCR_PATHWAY	0.76	0.06	0	0	0	not significant
9275	PKCA_DN.V1_UP	0.76	0.06	0	0	0	not significant
9276	REACTOME_G_ALPHA_12_13_SIGNALLING_EVENTS	0.76	0.06	0	0	0	not significant
9277	GO_ORGANELLE_DISASSEMBLY	0.76	0.04	0	0	0	not significant
9278	REACTOME_SIGNALING_BY_TGF_BETA_FAMILY_MEMBERS	0.76	0.04	0	0	0	not significant
9279	DARWICHE_SQUAMOUS_CELL_CARCINOMA_DN	0.76	0.03	0	0	0	not significant
9280	GO_REGULATION_OF_INTRINSIC_APOPTOTIC_SIGNALING_PATHWAY	0.76	0.03	0	0	0	not significant
9281	GO_RECYCLING_ENDOSOME	0.76	0.02	0	0	0	not significant
9282	STARK_PREFRONTAL_CORTEX_22Q11_DELETION_UP	0.76	0.02	0	0	0	not significant
9283	WANG_CISPLATIN_RESPONSE_AND_XPC_DN	0.76	0.02	0	0	0	not significant
9284	GO_PEROXISOME_FISSION	0.77	0.14	0	0	0	not significant
9285	GO_HEMATOPOIETIC_STEM_CELL_PROLIFERATION	0.77	0.13	0	0	0	not significant
9286	KYNG_ENVIRONMENTAL_STRESS_RESPONSE_NOT_BY_4NQO_IN_OLD	0.77	0.13	0	0	0	not significant
9287	TONKS_TARGETS_OF_RUNX1_RUNX1T1_FUSION_ERYTHROCYTE_DN	0.77	0.13	0	0	0	not significant
9288	BIOCARTA_PAR1_PATHWAY	0.77	0.12	0	0	0	not significant
9289	GO_ACTIVATION_OF_PROTEIN_KINASE_B_ACTIVITY	0.77	0.12	0	0	0	not significant
9290	GO_DNA_BINDING_BENDING	0.77	0.12	0	0	0	not significant
9291	GO_INOSITOL_TRISPHOSPHATE_PHOSPHATASE_ACTIVITY	0.77	0.12	0	0	0	not significant
9292	GO_MODULATION_OF_EXCITATORY_POSTSYNAPTIC_POTENTIAL	0.77	0.12	0	0	0	not significant
9293	GO_POSITIVE_REGULATION_OF_MICROTUBULE_BINDING	0.77	0.12	0	0	0	not significant
9294	GO_POSITIVE_REGULATION_OF_OSTEOCLAST_DIFFERENTIATION	0.77	0.12	0	0	0	not significant
9295	WILLIAMS_ESR2_TARGETS_DN	0.77	0.12	0	0	0	not significant
9296	GHANDHI_DIRECT_IRRADIATION_DN	0.77	0.11	0	0	0	not significant
9297	GO_CYTOPLASMIC_MRNA_PROCESSING_BODY_ASSEMBLY	0.77	0.11	0	0	0	not significant
9298	GO_MITOGEN_ACTIVATED_PROTEIN_KINASE_KINASE_BINDING	0.77	0.11	0	0	0	not significant
9299	GO_REGULATION_OF_ACUTE_INFLAMMATORY_RESPONSE_TO_ANTIGEN	0.77	0.11	0	0	0	not significant
9300	GO_REGULATION_OF_MITOCHONDRIAL_FISSION	0.77	0.11	0	0	0	not significant
9301	GO_RESPONSE_TO_FUNGICIDE	0.77	0.11	0	0	0	not significant
9302	GO_RNAI_EFFECTOR_COMPLEX	0.77	0.11	0	0	0	not significant
9303	KEGG_DRUG_METABOLISM_CYTOCHROME_P450	0.77	0.11	0	0	0	not significant
9304	OLSSON_E2F3_TARGETS_UP	0.77	0.11	0	0	0	not significant
9305	REACTOME_DOWNREGULATION_OF_ERBB2_SIGNALING	0.77	0.11	0	0	0	not significant
9306	EBAUER_TARGETS_OF_PAX3_FOXO1_FUSION_DN	0.77	0.10	0	0	0	not significant
9307	GO_CENTRIOLAR_SUBDISTAL_APPENDAGE	0.77	0.10	0	0	0	not significant
9308	GO_CYTOPLASMIC_TRANSLATIONAL_INITIATION	0.77	0.10	0	0	0	not significant
9309	GO_MULTIVESICULAR_BODY_SORTING_PATHWAY	0.77	0.10	0	0	0	not significant
9310	GO_NEGATIVE_REGULATION_OF_MYELOID_LEUKOCYTE_DIFFERENTIATI	0.77	0.10	0	0	0	not significant
9311	GO_TOLL_LIKE_RECEPTOR_3_SIGNALING_PATHWAY	0.77	0.10	0	0	0	not significant
9312	REACTOME_CYTOCHROME_P450_ARRANGED_BY_SUBSTRATE_TYPE	0.77	0.10	0	0	0	not significant
9313	BEGUM_TARGETS_OF_PAX3_FOXO1_FUSION_UP	0.77	0.09	0	0	0	not significant
9314	CHR8P11	0.77	0.09	0	0	0	not significant
9315	GO_FIBROBLAST_MIGRATION	0.77	0.09	0	0	0	not significant
9316	GO_ION_ANTIPORTER_ACTIVITY	0.77	0.09	0	0	0	not significant
9317	GO_NUCLEOSIDE_MONOPHOSPHATE_METABOLIC_PROCESS	0.77	0.09	0	0	0	not significant
9318	GO_REGULATION_OF_ERAD_PATHWAY	0.77	0.09	0	0	0	not significant
9319	KEGG_NOTCH_SIGNALING_PATHWAY	0.77	0.09	0	0	0	not significant
9320	CHANDRAN_METASTASIS_TOP50_DN	0.77	0.08	0	0	0	not significant
9321	CHEBOTAEV_GR_TARGETS_UP	0.77	0.08	0	0	0	not significant
9322	GO_ACTIVATION_OF_MAPKK_ACTIVITY	0.77	0.08	0	0	0	not significant
9323	GO_IRON_ION_TRANSPORT	0.77	0.08	0	0	0	not significant
9324	GO_NEUROMUSCULAR_JUNCTION	0.77	0.08	0	0	0	not significant
9325	GO_PROMOTER_SPECIFIC_CHROMATIN_BINDING	0.77	0.08	0	0	0	not significant
9326	LUI_THYROID_CANCER_PAX8_PPARG_DN	0.77	0.08	0	0	0	not significant
9327	PID_ERA_GENOMIC_PATHWAY	0.77	0.08	0	0	0	not significant
9328	REACTOME_REGULATION_OF_TP53_EXPRESSION_AND_DEGRADATION	0.77	0.08	0	0	0	not significant
9329	REACTOME_SIGNALING_BY_NOTCH1_IN_CANCER	0.77	0.08	0	0	0	not significant
9330	CHR1Q23	0.77	0.07	0	0	0	not significant
9331	GO_INTEGRIN_MEDIATED_SIGNALING_PATHWAY	0.77	0.07	0	0	0	not significant
9332	GO_NEGATIVE_REGULATION_OF_MYELOID_CELL_DIFFERENTIATION	0.77	0.07	0	0	0	not significant
9333	KEGG_VIBRIO_CHOLERAE_INFECTION	0.77	0.07	0	0	0	not significant
9334	AMIT_EGF_RESPONSE_120_HELA	0.77	0.06	0	0	0	not significant
9335	CROMER_METASTASIS_UP	0.77	0.06	0	0	0	not significant
9336	ZHAN_MULTIPLE_MYELOMA_CD1_AND_CD2_UP	0.77	0.06	0	0	0	not significant
9337	GO_POSITIVE_REGULATION_OF_CELL_GROWTH	0.77	0.05	0	0	0	not significant
9338	REACTOME_DEATH_RECEPTOR_SIGNALLING	0.77	0.04	0	0	0	not significant
9339	GO_CELL_CYCLE_CHECKPOINT	0.77	0.03	0	0	0	not significant
9340	GO_CYSTEINE_TYPE_PEPTIDASE_ACTIVITY	0.77	0.03	0	0	0	not significant
9341	GO_CILIUM	0.77	0.01	0	0	0	not significant
9342	GO_COMMISSURAL_NEURON_AXON_GUIDANCE	0.78	0.14	0	0	0	not significant
9343	GO_NEGATIVE_REGULATION_OF_SMOOTHENED_SIGNALING_PATHWAY_	0.78	0.14	0	0	0	not significant
9344	GO_PHOSPHATIDYLINOSITOL_3_KINASE_COMPLEX_CLASS_III	0.78	0.14	0	0	0	not significant
9345	EHLERS_ANEUPLOIDY_DN	0.78	0.13	0	0	0	not significant
9346	GO_NEGATIVE_REGULATION_OF_LIPID_TRANSPORT	0.78	0.13	0	0	0	not significant
9347	GO_PEPTIDYL_PROLINE_HYDROXYLATION	0.78	0.13	0	0	0	not significant
9348	GO_VIRAL_TRANSLATIONAL_TERMINATION_REINITIATION	0.78	0.13	0	0	0	not significant
9349	REACTOME_SIGNALING_BY_NTRK2_TRKB	0.78	0.13	0	0	0	not significant
9350	GO_DENDRITE_MEMBRANE	0.78	0.12	0	0	0	not significant
9351	GO_DIHYDROLIPOYL_DEHYDROGENASE_COMPLEX	0.78	0.12	0	0	0	not significant
9352	GO_INSULIN_LIKE_GROWTH_FACTOR_BINDING	0.78	0.12	0	0	0	not significant
9353	GO_POSITIVE_REGULATION_OF_TORC1_SIGNALING	0.78	0.12	0	0	0	not significant
9354	GO_REGULATION_OF_INTRINSIC_APOPTOTIC_SIGNALING_PATHWAY_IN	0.78	0.12	0	0	0	not significant
9355	GO_RETINAL_METABOLIC_PROCESS	0.78	0.12	0	0	0	not significant
9356	REACTOME_PLATELET_ADHESION_TO_EXPOSED_COLLAGEN	0.78	0.12	0	0	0	not significant
9357	REACTOME_TP53_REGULATES_TRANSCRIPTION_OF_SEVERAL_ADDITIO	0.78	0.12	0	0	0	not significant
9358	REACTOME_TRAF3_DEPENDENT_IRF_ACTIVATION_PATHWAY	0.78	0.12	0	0	0	not significant
9359	SMID_BREAST_CANCER_RELAPSE_IN_BRAIN_UP	0.78	0.12	0	0	0	not significant
9360	SPIRA_SMOKERS_LUNG_CANCER_DN	0.78	0.12	0	0	0	not significant
9361	GO_HISTONE_H4_K16_ACETYLATION	0.78	0.11	0	0	0	not significant
9362	GO_NEGATIVE_REGULATION_OF_INSULIN_SECRETION_INVOLVED_IN_CE	0.78	0.11	0	0	0	not significant

9363	GO_PHOTORECEPTOR_CELL_DIFFERENTIATION	0.78	0.11	0	0	0	not significant
9364	GO_REGULATION_OF_CARDIAC_MUSCLE_TISSUE_DEVELOPMENT	0.78	0.11	0	0	0	not significant
9365	PID_GLYPICAN_1PATHWAY	0.78	0.11	0	0	0	not significant
9366	BROWNE_HCMV_INFECTION_8HR_DN	0.78	0.10	0	0	0	not significant
9367	BURTON_ADIPOGENESIS_PEAK_AT_0HR	0.78	0.10	0	0	0	not significant
9368	GO_CELLULAR_CARBOHYDRATE_CATABOLIC_PROCESS	0.78	0.10	0	0	0	not significant
9369	GO_DETECTION_OF_STIMULUS_INVOLVED_IN_SENSORY_PERCEPTION	0.78	0.10	0	0	0	not significant
9370	GO_HISTONE_ACETYLTRANSFERASE_BINDING	0.78	0.10	0	0	0	not significant
9371	GO_PALMITOYLTRANSFERASE_ACTIVITY	0.78	0.10	0	0	0	not significant
9372	GO_POSITIVE_REGULATION_OF_PROTEIN_IMPORT	0.78	0.10	0	0	0	not significant
9373	GO_PROTEIN_DEACETYLASE_ACTIVITY	0.78	0.10	0	0	0	not significant
9374	GO_REGULATION_OF_TOLL_LIKE_RECEPTOR_SIGNALING_PATHWAY	0.78	0.10	0	0	0	not significant
9375	REACTOME_ENDOSOMAL_SORTING_COMPLEX_REQUIRED_FOR_TRANSI	0.78	0.10	0	0	0	not significant
9376	GO_RECEPTOR_CLUSTERING	0.78	0.09	0	0	0	not significant
9377	GO_SCAFFOLD_PROTEIN_BINDING	0.78	0.09	0	0	0	not significant
9378	PID_NOTCH_PATHWAY	0.78	0.09	0	0	0	not significant
9379	LEIN_CHOROID_PLEXUS_MARKERS	0.78	0.08	0	0	0	not significant
9380	POMEROY_MEDULLOBLASTOMA_PROGNOSIS_DN	0.78	0.08	0	0	0	not significant
9381	REACTOME_SYNTHESIS_OF_SUBSTRATES_IN_N_GLYCAN_BIOSYTHESIS	0.78	0.08	0	0	0	not significant
9382	DCA_UP.V1_UP	0.78	0.06	0	0	0	not significant
9383	GO_HYDROLASE_ACTIVITY_ACTING_ON_CARBON_NITROGEN_BUT_NOT	0.78	0.06	0	0	0	not significant
9384	GO_LIPID_TRANSPORTER_ACTIVITY	0.78	0.06	0	0	0	not significant
9385	FIGUEROA_AML_METHYLATION_CLUSTER_3_UP	0.78	0.05	0	0	0	not significant
9386	GO_PLATELET_ACTIVATION	0.78	0.05	0	0	0	not significant
9387	REACTOME_OXIDATIVE_STRESS_INDUCED_SENESCENCE	0.78	0.05	0	0	0	not significant
9388	BAKKER_FOXO3_TARGETS_DN	0.78	0.04	0	0	0	not significant
9389	GO_PLASMA_MEMBRANE_BOUNDED_CELL_PROJECTION_CYTOPLASM	0.78	0.04	0	0	0	not significant
9390	GO_REGULATION_OF_RNA_SPLICING	0.78	0.04	0	0	0	not significant
9391	GO_MICROTUBULE_BINDING	0.78	0.03	0	0	0	not significant
9392	GO_MICROTUBULE_ORGANIZING_CENTER_PART	0.78	0.03	0	0	0	not significant
9393	GO_REGULATION_OF_MITOCHONDRION_ORGANIZATION	0.78	0.03	0	0	0	not significant
9394	HALLMARK_HEME_METABOLISM	0.78	0.03	0	0	0	not significant
9395	DURCHDEWALD_SKIN_CARCINOGENESIS_DN	0.78	0.02	0	0	0	not significant
9396	GO_INTRINSIC_COMPONENT_OF_ORGANELLE_MEMBRANE	0.78	0.02	0	0	0	not significant
9397	GO_TRANSITION_METAL_ION_BINDING	0.78	0.00	0	0	0	not significant
9398	CHRXQ11	0.79	0.15	0	0	0	not significant
9399	GO_AP_1_ADAPTOR_COMPLEX_BINDING	0.79	0.15	0	0	0	not significant
9400	GO_NEGATIVE_REGULATION_OF_T_CELL_MEDIATED_CYTOTOXICITY	0.79	0.15	0	0	0	not significant
9401	GO_NUCLEAR_PERICENTRIC_HETEROCHROMATIN	0.79	0.15	0	0	0	not significant
9402	REACTOME_PI3K_AKT_ACTIVATION	0.79	0.15	0	0	0	not significant
9403	BANDRES_RESPONSE_TO_CARMUSTIN_WITHOUT_MGMT_24HR_DN	0.79	0.14	0	0	0	not significant
9404	GO_ENDOPLASMIC_RETICULUM_TUBULAR_NETWORK_ORGANIZATION	0.79	0.14	0	0	0	not significant
9405	GO_EUKARYOTIC_TRANSLATION_INITIATION_FACTOR_4F_COMPLEX	0.79	0.14	0	0	0	not significant
9406	GO_FATTY_ACID_ALPHA_OXIDATION	0.79	0.14	0	0	0	not significant
9407	GO_NEGATIVE_REGULATION_OF_BEHAVIOR	0.79	0.14	0	0	0	not significant
9408	GO_POSITIVE_REGULATION_OF_CHROMOSOME_SEPARATION	0.79	0.14	0	0	0	not significant
9409	GO_REGULATION_OF_MESONEPHROS_DEVELOPMENT	0.79	0.14	0	0	0	not significant
9410	GO_REGULATION_OF_SYNAPTIC_VESICLE_FUSION_TO_PRESYNAPTIC_A	0.79	0.14	0	0	0	not significant
9411	HINATA_NFKB_IMMU_INF	0.79	0.14	0	0	0	not significant
9412	ZHENG_FOXP3_TARGETS_IN_THYMUS_DN	0.79	0.14	0	0	0	not significant
9413	CHRXQ24	0.79	0.13	0	0	0	not significant
9414	GO_CELL_FATE_DETERMINATION	0.79	0.13	0	0	0	not significant
9415	GO_LEUKOCYTE_TETHERING_OR_ROLLING	0.79	0.13	0	0	0	not significant
9416	GO_LYMPH_NODE_DEVELOPMENT	0.79	0.13	0	0	0	not significant
9417	GO_MANNOSYL_OLIGOSACCHARIDE_1_2_ALPHA_MANNOSIDASE_ACTIVIT	0.79	0.13	0	0	0	not significant
9418	GO_POSITIVE_REGULATION_OF_EMBRYONIC_DEVELOPMENT	0.79	0.13	0	0	0	not significant
9419	GO_POSITIVE_REGULATION_OF_ISOTYPE_SWITCHING	0.79	0.13	0	0	0	not significant
9420	GO_PROTEIN_MATURATION_BY_IRON_SULFUR_CLUSTER_TRANSFER	0.79	0.13	0	0	0	not significant
9421	GO_REGULATION_OF_KERATINOCYTE_PROLIFERATION	0.79	0.13	0	0	0	not significant
9422	MANTOVANI_NFKB_TARGETS_UP	0.79	0.13	0	0	0	not significant
9423	REACTOME_CD28_DEPENDENT_VAV1_PATHWAY	0.79	0.13	0	0	0	not significant
9424	REACTOME_MAP3K8_TPL2_DEPENDENT_MAPK1_3_ACTIVATION	0.79	0.13	0	0	0	not significant
9425	GO_DOSAGE_COMPENSATION	0.79	0.12	0	0	0	not significant
9426	GO_HYDROLASE_ACTIVITY_HYDROLYZING_N_GLYCOSYL_COMPOUNDS	0.79	0.12	0	0	0	not significant
9427	GO_MAGNESIUM_ION_TRANSPORT	0.79	0.12	0	0	0	not significant
9428	GO_NEGATIVE_REGULATION_OF_OSTEOBLAST_DIFFERENTIATION	0.79	0.12	0	0	0	not significant
9429	GO_POSTSYNAPTIC_MEMBRANE_ORGANIZATION	0.79	0.12	0	0	0	not significant
9430	GO_REPRODUCTIVE_BEHAVIOR	0.79	0.12	0	0	0	not significant
9431	GO_STRESS_GRANULE_ASSEMBLY	0.79	0.12	0	0	0	not significant
9432	LEE_AGING_MUSCLE_UP	0.79	0.12	0	0	0	not significant
9433	REACTOME_E2F_MEDIATED_REGULATION_OF_DNA_REPLICATION	0.79	0.12	0	0	0	not significant
9434	REACTOME_MAPK1_ERK2_ACTIVATION	0.79	0.12	0	0	0	not significant
9435	REACTOME_SIGNALING_BY_NOTCH2	0.79	0.12	0	0	0	not significant
9436	VALK_AML_CLUSTER_7	0.79	0.12	0	0	0	not significant
9437	GO_PLATELET_DERIVED_GROWTH_FACTOR_RECEPTOR_SIGNALING_PA	0.79	0.11	0	0	0	not significant
9438	REACTOME_SIGNALING_BY_FGFR1	0.79	0.11	0	0	0	not significant
9439	DELASERNA_MYOD_TARGETS_DN	0.79	0.10	0	0	0	not significant
9440	FIGUEROA_AML_METHYLATION_CLUSTER_1_DN	0.79	0.10	0	0	0	not significant
9441	GO_CERAMIDE_BIOSYNTHETIC_PROCESS	0.79	0.10	0	0	0	not significant
9442	GO_EMBRYONIC_PLACENTA_DEVELOPMENT	0.79	0.10	0	0	0	not significant
9443	GO_REGULATION_OF_SYSTEMIC_ARTERIAL_BLOOD_PRESSURE	0.79	0.10	0	0	0	not significant
9444	NIKOLSKY_BREAST_CANCER_8P12_P11_AMPLICON	0.79	0.10	0	0	0	not significant
9445	STAMBOLSKY_TARGETS_OF_MUTATED_TP53_DN	0.79	0.10	0	0	0	not significant
9446	CHRXQ26	0.79	0.09	0	0	0	not significant
9447	GO_NEGATIVE_REGULATION_OF_SMALL_GTPASE_MEDIATED_SIGNAL_T	0.79	0.09	0	0	0	not significant
9448	KEGG_LONG_TERM_DEPRESSION	0.79	0.09	0	0	0	not significant
9449	LIN_MELANOMA_COPY_NUMBER_UP	0.79	0.09	0	0	0	not significant
9450	CHEN_LIVER_METABOLISM_QTL_CIS	0.79	0.08	0	0	0	not significant
9451	GO_CELLULAR_MONOVALENT_INORGANIC_CATION_HOMEOSTASIS	0.79	0.08	0	0	0	not significant
9452	GO_NEURON_PROJECTION_ORGANIZATION	0.79	0.08	0	0	0	not significant
9453	GOLDRATH_IMMUNE_MEMORY	0.79	0.08	0	0	0	not significant
9454	REACTOME_TRANSPORT_OF_INORGANIC_CATIONS_ANIONS_AND_AMINO	0.79	0.08	0	0	0	not significant
9455	PID_CXCR4_PATHWAY	0.79	0.07	0	0	0	not significant
9456	GO_N_ACYLTRANSFERASE_ACTIVITY	0.79	0.06	0	0	0	not significant
9457	GO_SEX_DIFFERENTIATION	0.79	0.05	0	0	0	not significant
9458	GO_TRANSFERASE_ACTIVITY_TRANSFERRING_ACYL_GROUPS	0.79	0.04	0	0	0	not significant
9459	IVANOVA_HEMATOPOIESIS_MATURE_CELL	0.79	0.03	0	0	0	not significant
9460	DOUGLAS_BMI1_TARGETS_DN	0.79	0.02	0	0	0	not significant
9461	GO_ENDOSOME	0.79	0.00	0	0	0	not significant
9462	GO_ACTOMYOSIN_CONTRACTILE_RING_ORGANIZATION	0.80	0.16	0	0	0	not significant
9463	GO_POSITIVE_REGULATION_OF_ARP2_3_COMPLEX_MEDIATED_ACTIN_N	0.80	0.16	0	0	0	not significant
9464	GO_REGULATION_OF_CHOLESTEROL_METABOLIC_PROCESS	0.80	0.15	0	0	0	not significant
9465	GO_REGULATION_OF_VASCULAR_SMOOTH_MUSCLE_CELL_DIFFERENTI	0.80	0.15	0	0	0	not significant
9466	BIOCARTA_PDZS_PATHWAY	0.80	0.14	0	0	0	not significant
9467	BIOCARTA_PGC1A_PATHWAY	0.80	0.14	0	0	0	not significant
9468	CHR3Q25	0.80	0.14	0	0	0	not significant
9469	FONTAINE_THYROID_TUMOR_UNCERTAIN_MALIGNANCY_DN	0.80	0.14	0	0	0	not significant
9470	GO_ACTIVIN_BINDING	0.80	0.14	0	0	0	not significant
9471	GO_COBALAMIN_METABOLIC_PROCESS	0.80	0.14	0	0	0	not significant

9472	GO_ENDOTHELIAL_CELL_CHEMOTAXIS	0.80	0.14	0	0	0	not significant
9473	GO_EPIBOLY	0.80	0.14	0	0	0	not significant
9474	GO_EUKARYOTIC_TRANSLATION_INITIATION_FACTOR_3_COMPLEX_EIF3	0.80	0.14	0	0	0	not significant
9475	GO_HIPPOCAMPAL_MOSSY_FIBER_TO_CA3_SYNAPSE	0.80	0.14	0	0	0	not significant
9476	GO_LYS63_SPECIFIC_DEUBIQUITINASE_ACTIVITY	0.80	0.14	0	0	0	not significant
9477	GO_NEGATIVE_REGULATION_OF_STEM_CELL_DIFFERENTIATION	0.80	0.14	0	0	0	not significant
9478	GO_POSITIVE_REGULATION_OF_ACUTE_INFLAMMATORY_RESPONSE_T(	0.80	0.14	0	0	0	not significant
9479	GO_RESPONSE_TO_DIETARY_EXCESS	0.80	0.14	0	0	0	not significant
9480	GO_STORE_OPERATED_CALCIUM_CHANNEL_ACTIVITY	0.80	0.14	0	0	0	not significant
9481	GOUYER_TATI_TARGETS_DN	0.80	0.14	0	0	0	not significant
9482	REACTOME_DOPAMINE_NEUROTRANSMITTER_RELEASE_CYCLE	0.80	0.14	0	0	0	not significant
9483	REACTOME_TRANSCRIPTION_OF_E2F_TARGETS_UNDER_NEGATIVE_CO	0.80	0.14	0	0	0	not significant
9484	SASSON_FSH_RESPONSE	0.80	0.14	0	0	0	not significant
9485	SCIAN_INVERSED_TARGETS_OF_TP53_AND_TP73_UP	0.80	0.14	0	0	0	not significant
9486	AMUNDSON_DNA_DAMAGE_RESPONSE_TP53	0.80	0.13	0	0	0	not significant
9487	GO_HISTONE_H3_DEACETYLATION	0.80	0.13	0	0	0	not significant
9488	GO_PHENOL_CONTAINING_COMPOUND_BIOSYNTHETIC_PROCESS	0.80	0.13	0	0	0	not significant
9489	GO_PHOSPHATIDYLINOSITOL_3_4_5_TRISPHOSPHATE_BINDING	0.80	0.13	0	0	0	not significant
9490	HOFFMANN_PRE_BI_TO_LARGE_PRE_BII_LYMPHOCYTE_UP	0.80	0.13	0	0	0	not significant
9491	REACTOME_ACYL_CHAIN_REMODELLING_OF_PC	0.80	0.13	0	0	0	not significant
9492	BAUS_TFF2_TARGETS_UP	0.80	0.12	0	0	0	not significant
9493	CHR14Q21	0.80	0.12	0	0	0	not significant
9494	GO_PROTON_EXPORTING_ATPASE_ACTIVITY	0.80	0.12	0	0	0	not significant
9495	GO_RIBONUCLEOTIDE_CATABOLIC_PROCESS	0.80	0.12	0	0	0	not significant
9496	SIG_IL4RECEPTOR_IN_B_LYPHOCYTES	0.80	0.12	0	0	0	not significant
9497	PHONG_TNF_TARGETS_UP	0.80	0.11	0	0	0	not significant
9498	REACTOME_MICROAUTOPHAGY	0.80	0.11	0	0	0	not significant
9499	CHIANG_LIVER_CANCER_SUBCLASS_UNANNOTATED_UP	0.80	0.10	0	0	0	not significant
9500	GO_REGULATION_OF_AUTOPHAGY_OF_MITOCHONDRION	0.80	0.10	0	0	0	not significant
9501	KEGG_EPITHELIAL_CELL_SIGNALING_IN_HELICOBACTER_PYLORI_INFECT	0.80	0.10	0	0	0	not significant
9502	MARIADASON_REGULATED_BY_HISTONE_ACETYLATION_DN	0.80	0.10	0	0	0	not significant
9503	MARIADASON_REGULATED_BY_HISTONE_ACETYLATION_UP	0.80	0.10	0	0	0	not significant
9504	GO_REGULATION_OF_NEUROTRANSMITTER_SECRETION	0.80	0.09	0	0	0	not significant
9505	OXFORD_RALA_OR_RALB_TARGETS_UP	0.80	0.09	0	0	0	not significant
9506	GO_REGULATION_OF_MEGAKARYOCYTE_DIFFERENTIATION	0.80	0.08	0	0	0	not significant
9507	GO_NEGATIVE_REGULATION_OF_PROTEIN_COMPLEX_ASSEMBLY	0.80	0.07	0	0	0	not significant
9508	KRAS.DF.V1_DN	0.80	0.07	0	0	0	not significant
9509	LINDGREN_BLADDER_CANCER_CLUSTER_1_UP	0.80	0.06	0	0	0	not significant
9510	GO_CARBOHYDRATE_BIOSYNTHETIC_PROCESS	0.80	0.05	0	0	0	not significant
9511	GO_PROTEIN_ACYLATION	0.80	0.05	0	0	0	not significant
9512	RIZKI_TUMOR_INVASIVENESS_3D_UP	0.80	0.05	0	0	0	not significant
9513	GO_PROTEIN_SERINE_THREONINE_KINASE_ACTIVITY	0.80	0.02	0	0	0	not significant
9514	RAO_BOUND_BY_SALL4_ISOFORM_B	0.80	0.02	0	0	0	not significant
9515	GO_ECTODERMAL_PLACODE_DEVELOPMENT	0.81	0.17	0	0	0	not significant
9516	BARRIER_CANCER_RELAPSE_TUMOR_SAMPLE_DN	0.81	0.16	0	0	0	not significant
9517	GO_REGULATION_OF_STEM_CELL_DIVISION	0.81	0.16	0	0	0	not significant
9518	GO_ZYMOGEN_GRANULE_MEMBRANE	0.81	0.16	0	0	0	not significant
9519	MYLLYKANGAS_AMPLIFICATION_HOT_SPOT_22	0.81	0.16	0	0	0	not significant
9520	NIKOLSKY_BREAST_CANCER_14Q22_AMPLICON	0.81	0.16	0	0	0	not significant
9521	REACTOME_FRS_MEDIATED_FGFR2_SIGNALING	0.81	0.16	0	0	0	not significant
9522	REACTOME_TLR3_MEDIATED_TICAM1_DEPENDENT_PROGRAMMED_CEL	0.81	0.16	0	0	0	not significant
9523	GO_ALDEHYDE_DEHYDROGENASE_NAD_ACTIVITY	0.81	0.15	0	0	0	not significant
9524	GO_AORTA_MORPHOGENESIS	0.81	0.15	0	0	0	not significant
9525	GO_ARRESTIN_FAMILY_PROTEIN_BINDING	0.81	0.15	0	0	0	not significant
9526	GO_HISTONE_DEMETHYLASE_ACTIVITY_H3_K4_SPECIFIC_	0.81	0.15	0	0	0	not significant
9527	GO_MAGNESIUM_ION_TRANSMEMBRANE_TRANSPORTER_ACTIVITY	0.81	0.15	0	0	0	not significant
9528	GO_NEGATIVE_REGULATION_OF_MAST_CELL_ACTIVATION	0.81	0.15	0	0	0	not significant
9529	GO_NEGATIVE_REGULATION_OF_MAST_CELL_ACTIVATION_INVOLVED_I	0.81	0.15	0	0	0	not significant
9530	GO_NEGATIVE_REGULATION_OF_VASCULAR_SMOOTH_MUSCLE_CELL_F	0.81	0.15	0	0	0	not significant
9531	GO_POSITIVE_REGULATION_OF_RELEASE_OF_SEQUESTERED_CALCIUM	0.81	0.15	0	0	0	not significant
9532	GO_PYRIMIDINE_NUCLEOSIDE_BIOSYNTHETIC_PROCESS	0.81	0.15	0	0	0	not significant
9533	GO_REGULATION_OF_SMOOTHENED_SIGNALING_PATHWAY_INVOLVED_	0.81	0.15	0	0	0	not significant
9534	REACTOME_PHASE_4_RESTING_MEMBRANE_POTENTIAL	0.81	0.15	0	0	0	not significant
9535	WANG_RESPONSE_TO_FORSKOLIN_DN	0.81	0.15	0	0	0	not significant
9536	CHR4Q12	0.81	0.14	0	0	0	not significant
9537	CHR6P23	0.81	0.14	0	0	0	not significant
9538	FINAK_BREAST_CANCER_SDPP_SIGNATURE	0.81	0.14	0	0	0	not significant
9539	GO_1_PHOSPHATIDYLINOSITOL_4_KINASE_ACTIVITY	0.81	0.14	0	0	0	not significant
9540	GO_CELL_BODY_MEMBRANE	0.81	0.14	0	0	0	not significant
9541	GO_INTERLEUKIN_21_MEDIATED_SIGNALING_PATHWAY	0.81	0.14	0	0	0	not significant
9542	GO_NUCLEOTIDE_SUGAR_TRANSMEMBRANE_TRANSPORT	0.81	0.14	0	0	0	not significant
9543	GO_REGULATION_OF_BILE_ACID_METABOLIC_PROCESS	0.81	0.14	0	0	0	not significant
9544	GO_REGULATION_OF_ENDOTHELIAL_CELL_CHEMOTAXIS	0.81	0.14	0	0	0	not significant
9545	GO_SMALL_RIBOSOMAL_SUBUNIT_RRNA_BINDING	0.81	0.14	0	0	0	not significant
9546	PATTERSON_DOCETAXEL_RESISTANCE	0.81	0.14	0	0	0	not significant
9547	REACTOME_FRS_MEDIATED_FGFR4_SIGNALING	0.81	0.14	0	0	0	not significant
9548	REACTOME_NEGATIVE_REGULATION_OF_FGFR2_SIGNALING	0.81	0.14	0	0	0	not significant
9549	STAMBOLSKY_RESPONSE_TO_VITAMIN_D3_DN	0.81	0.14	0	0	0	not significant
9550	BRIDEAU_IMPRINTED_GENES	0.81	0.13	0	0	0	not significant
9551	GO_DORSAL_VENTRAL_NEURAL_TUBE_PATTERNING	0.81	0.13	0	0	0	not significant
9552	GO_ESTABLISHMENT_OF_ENDOTHELIAL_BARRIER	0.81	0.13	0	0	0	not significant
9553	GO_HEART_TRABECULA_MORPHOGENESIS	0.81	0.13	0	0	0	not significant
9554	GO_POLY_A_SPECIFIC_RIBONUCLEASE_ACTIVITY	0.81	0.13	0	0	0	not significant
9555	GO_POSITIVE_REGULATION_OF_FOCAL_ADHESION_ASSEMBLY	0.81	0.13	0	0	0	not significant
9556	GO_POSITIVE_REGULATION_OF_MICROTUBULE_POLYMERIZATION_OR_[	0.81	0.13	0	0	0	not significant
9557	PID_CIRCADIAN_PATHWAY	0.81	0.13	0	0	0	not significant
9558	REACTOME_MYOGENESIS	0.81	0.13	0	0	0	not significant
9559	CORRE_MULTIPLE_MYELOMA_DN	0.81	0.12	0	0	0	not significant
9560	ENGELMANN_CANCER_PROGENITORS_UP	0.81	0.12	0	0	0	not significant
9561	GO_COFACTOR_TRANSPORT	0.81	0.12	0	0	0	not significant
9562	GO_ENDOTHELIAL_CELL_DEVELOPMENT	0.81	0.12	0	0	0	not significant
9563	GO_G0_TO_G1_TRANSITION	0.81	0.12	0	0	0	not significant
9564	GO_NEGATIVE_REGULATION_OF_LIPID_METABOLIC_PROCESS	0.81	0.12	0	0	0	not significant
9565	GO_NEGATIVE_REGULATION_OF_OSSIFICATION	0.81	0.12	0	0	0	not significant
9566	GO_PROTON_TRANSPORTING_TWO_SECTOR_ATPASE_COMPLEX	0.81	0.12	0	0	0	not significant
9567	GO_RAC_PROTEIN_SIGNAL_TRANSDUCTION	0.81	0.12	0	0	0	not significant
9568	GO_TRANSFERASE_ACTIVITY_TRANSFERRING_PENTOSYL_GROUPS	0.81	0.12	0	0	0	not significant
9569	GO_UNSATURATED_FATTY_ACID_METABOLIC_PROCESS	0.81	0.12	0	0	0	not significant
9570	LASTOWSKA_COAMPLIFIED_WITH_MYCN	0.81	0.12	0	0	0	not significant
9571	WEIGEL_OXIDATIVE_STRESS_BY_HNE_AND_TBH	0.81	0.12	0	0	0	not significant
9572	GO_CELLULAR_RESPONSE_TO_VIRUS	0.81	0.11	0	0	0	not significant
9573	GO_ESCRT_COMPLEX	0.81	0.11	0	0	0	not significant
9574	GO_HISTONE_H3_K4_METHYLATION	0.81	0.11	0	0	0	not significant
9575	GO_ODONTOGENESIS	0.81	0.11	0	0	0	not significant
9576	GO_REGULATION_OF_LIPID_CATABOLIC_PROCESS	0.81	0.11	0	0	0	not significant
9577	KRAS.LUNG.BREAST_UP.V1_UP	0.81	0.10	0	0	0	not significant
9578	GO_EPIDERMAL_GROWTH_FACTOR_RECEPTOR_SIGNALING_PATHWAY	0.81	0.09	0	0	0	not significant
9579	GO_NEGATIVE_REGULATION_OF_RESPONSE_TO_DNA_DAMAGE_STIMUL	0.81	0.09	0	0	0	not significant
9580	GO_SPERM_PART	0.81	0.09	0	0	0	not significant

9581	KOKKINAKIS_METHIONINE_DEPRIVATION_96HR_UP	0.81	0.08	0	0	0	not significant
9582	GO_RECOMBINATIONAL_REPAIR	0.81	0.07	0	0	0	not significant
9583	FOSTER_KDM1A_TARGETS_DN	0.81	0.06	0	0	0	not significant
9584	GO_DEVELOPMENTAL_PROCESS_INVOLVED_IN_REPRODUCTION	0.81	0.03	0	0	0	not significant
9585	BRUINS_UVC_RESPONSE_VIA_TP53_GROUP_A	0.81	0.01	0	0	0	not significant
9586	GO_NUCLEOTIDE_SUGAR_TRANSMEMBRANE_TRANSPORTER_ACTIVITY	0.82	0.18	0	0	0	not significant
9587	CHR4P12	0.82	0.17	0	0	0	not significant
9588	GO_BLOOD_VESSEL_ENDOTHELIAL_CELL_PROLIFERATION_INVOLVED_I	0.82	0.17	0	0	0	not significant
9589	GO_CELLULAR_RESPONSE_TO_INTERFERON_ALPHA	0.82	0.17	0	0	0	not significant
9590	GO_ELL_EAF_COMPLEX	0.82	0.17	0	0	0	not significant
9591	GO_INTRAMOLECULAR_OXIDOREDUCTASE_ACTIVITY_INTERCONVERTIN(	0.82	0.17	0	0	0	not significant
9592	GO_MHC_CLASS_II_BIOSYNTHETIC_PROCESS	0.82	0.17	0	0	0	not significant
9593	GO_MOTOR_BEHAVIOR	0.82	0.17	0	0	0	not significant
9594	GO_SPECIFICATION_OF_ANIMAL_ORGAN_IDENTITY	0.82	0.17	0	0	0	not significant
9595	KEGG_PENTOSE_AND_GLUCURONATE_INTERCONVERSIONS	0.82	0.17	0	0	0	not significant
9596	PID_TOLL_ENDOGENOUS_PATHWAY	0.82	0.17	0	0	0	not significant
9597	REACTOME_EICOSANOIDS	0.82	0.17	0	0	0	not significant
9598	REACTOME_POLB_DEPENDENT_LONG_PATCH_BASE_EXCISION_REPAIR	0.82	0.17	0	0	0	not significant
9599	REACTOME_SYNTHESIS_OF_PG	0.82	0.17	0	0	0	not significant
9600	CHR13Q13	0.82	0.16	0	0	0	not significant
9601	GO_1_PHOSPHATIDYLINOSITOL_BINDING	0.82	0.16	0	0	0	not significant
9602	GO_DOPAMINERGIC_SYNAPSE	0.82	0.16	0	0	0	not significant
9603	GO_INNERVATION	0.82	0.16	0	0	0	not significant
9604	GO_L_ASCORBIC_ACID_BINDING	0.82	0.16	0	0	0	not significant
9605	GO_POSITIVE_REGULATION_OF_MAST_CELL_ACTIVATION_INVOLVED_IN	0.82	0.16	0	0	0	not significant
9606	GO_VERY_LONG_CHAIN_FATTY_ACID_COA_LIGASE_ACTIVITY	0.82	0.16	0	0	0	not significant
9607	GO_VOCALIZATION_BEHAVIOR	0.82	0.16	0	0	0	not significant
9608	REACTOME_TRAFFICKING_OF_GLUR2_CONTAINING_AMPA_RECEPTORS	0.82	0.16	0	0	0	not significant
9609	GO_CORNIFICATION	0.82	0.15	0	0	0	not significant
9610	GO_ENERGY_COUPLED_PROTON_TRANSMEMBRANE_TRANSPORT_AGA	0.82	0.15	0	0	0	not significant
9611	GO_GLIAL_CELL_PROJECTION	0.82	0.15	0	0	0	not significant
9612	GO_MICROTUBULE_MINUS_END_BINDING	0.82	0.15	0	0	0	not significant
9613	GO_NEGATIVE_REGULATION_OF_PLASMA_MEMBRANE_BOUNDED_CELL_	0.82	0.15	0	0	0	not significant
9614	GO_NEUROPILIN_BINDING	0.82	0.15	0	0	0	not significant
9615	GO_PEPTIDYL_L_CYSTEINE_S_PALMITOYLATION	0.82	0.15	0	0	0	not significant
9616	GO_POSITIVE_REGULATION_OF_INTERLEUKIN_6_BIOSYNTHETIC_PROCE	0.82	0.15	0	0	0	not significant
9617	GO_SUBSTRATE_DEPENDENT_CELL_MIGRATION	0.82	0.15	0	0	0	not significant
9618	GO_THYMIC_T_CELL_SELECTION	0.82	0.15	0	0	0	not significant
9619	MYLLYKANGAS_AMPLIFICATION_HOT_SPOT_12	0.82	0.15	0	0	0	not significant
9620	REACTOME_NEGATIVE_REGULATION_OF_FGFR4_SIGNALING	0.82	0.15	0	0	0	not significant
9621	GO_CELLULAR_RESPONSE_TO_ARSENIC_CONTAINING_SUBSTANCE	0.82	0.14	0	0	0	not significant
9622	GO_NEGATIVE_REGULATION_OF_ENDOCYTOSIS	0.82	0.14	0	0	0	not significant
9623	GO_POLY_A_BINDING	0.82	0.14	0	0	0	not significant
9624	GO_POSITIVE_REGULATION_OF_CYTOPLASMIC_TRANSPORT	0.82	0.14	0	0	0	not significant
9625	GO_PRESYNAPSE_ORGANIZATION	0.82	0.14	0	0	0	not significant
9626	GO_REGULATION_OF_COAGULATION	0.82	0.14	0	0	0	not significant
9627	GO_REGULATION_OF_CYSTEINE_TYPE_ENDOPEPTIDASE_ACTIVITY_INV(	0.82	0.14	0	0	0	not significant
9628	REACTOME_ROS_AND_RNS_PRODUCTION_IN_PHAGOCYTES	0.82	0.14	0	0	0	not significant
9629	REACTOME_SPHINGOLIPID_DE_NOVO_BIOSYNTHESIS	0.82	0.14	0	0	0	not significant
9630	REACTOME_VXPX_CARGO_TARGETING_TO_CILIUM	0.82	0.14	0	0	0	not significant
9631	WEIGEL_OXIDATIVE_STRESS_BY_HNE_AND_H2O2	0.82	0.14	0	0	0	not significant
9632	ZHAN_V1_LATE_DIFFERENTIATION_GENES_UP	0.82	0.14	0	0	0	not significant
9633	GO_LIGASE_ACTIVITY_FORMING_CARBON_NITROGEN_BONDS	0.82	0.13	0	0	0	not significant
9634	GO_NEGATIVE_REGULATION_OF_ERBB_SIGNALING_PATHWAY	0.82	0.13	0	0	0	not significant
9635	GO_PRODUCTION_OF_MOLECULAR_MEDIATOR_INVOLVED_IN_INFLAMMA	0.82	0.13	0	0	0	not significant
9636	MOSERLE_IFNA_RESPONSE	0.82	0.13	0	0	0	not significant
9637	ALK_DN.V1_DN	0.82	0.12	0	0	0	not significant
9638	CHR17Q12	0.82	0.12	0	0	0	not significant
9639	GO_CIS_GOLGI_NETWORK	0.82	0.12	0	0	0	not significant
9640	GO_INNER_EAR_MORPHOGENESIS	0.82	0.12	0	0	0	not significant
9641	MORI_IMMATURE_B_LYMPHOCYTE_UP	0.82	0.12	0	0	0	not significant
9642	REACTOME_G1_PHASE	0.82	0.12	0	0	0	not significant
9643	DIRMEIER_LMP1_RESPONSE_LATE_DN	0.82	0.11	0	0	0	not significant
9644	GO_PROTEASE_BINDING	0.82	0.11	0	0	0	not significant
9645	GO_SH3_SH2_ADAPTOR_ACTIVITY	0.82	0.11	0	0	0	not significant
9646	KYNG_DNA_DAMAGE_BY_GAMMA_AND_UV_RADIATION	0.82	0.11	0	0	0	not significant
9647	MORI_SMALL_PRE_BII_LYMPHOCYTE_UP	0.82	0.11	0	0	0	not significant
9648	GO_CELL_AGING	0.82	0.10	0	0	0	not significant
9649	GO_POSITIVE_REGULATION_OF_RESPONSE_TO_DNA_DAMAGE_STIMULU	0.82	0.10	0	0	0	not significant
9650	CHR1Q21	0.82	0.09	0	0	0	not significant
9651	GO_ACTIN_CYTOSKELETON_REORGANIZATION	0.82	0.09	0	0	0	not significant
9652	GO_EXOCYTIC_VESICLE	0.82	0.09	0	0	0	not significant
9653	VERHAAK_GLIOBLASTOMA_CLASSICAL	0.82	0.09	0	0	0	not significant
9654	PUIFFE_INVASION_INHIBITED_BY_ASCITES_DN	0.82	0.08	0	0	0	not significant
9655	GO_DNA_DEPENDENT_DNA_REPLICATION	0.82	0.07	0	0	0	not significant
9656	GO_ENDOCYTIC_VESICLE_MEMBRANE	0.82	0.07	0	0	0	not significant
9657	GO_MOLECULAR_ADAPTOR_ACTIVITY	0.82	0.07	0	0	0	not significant
9658	BLALOCK_ALZHEIMERS_DISEASE_INCIPIENT_UP	0.82	0.04	0	0	0	not significant
9659	GO_CILIARY_PART	0.82	0.04	0	0	0	not significant
9660	GO_TRANSCRIPTION_COACTIVATOR_ACTIVITY	0.82	0.04	0	0	0	not significant
9661	GO_REGULATION_OF_NEURON_DIFFERENTIATION	0.82	0.03	0	0	0	not significant
9662	GO_GOLGI_APPARATUS_PART	0.82	0.01	0	0	0	not significant
9663	GO_POSITIVE_REGULATION_OF_RNA_BIOSYNTHETIC_PROCESS	0.82	0.01	0	0	0	not significant
9664	FLORIO_HUMAN_NEOCORTEX	0.83	0.18	0	0	0	not significant
9665	GO_MACROPHAGE_ACTIVATION_INVOLVED_IN_IMMUNE_RESPONSE	0.83	0.18	0	0	0	not significant
9666	GO_PLATELET_DERIVED_GROWTH_FACTOR_RECEPTOR_BINDING	0.83	0.18	0	0	0	not significant
9667	GO_POSITIVE_REGULATION_OF_MITOTIC_SISTER_CHROMATID_SEPARA	0.83	0.18	0	0	0	not significant
9668	GO_POSTSYNAPTIC_MODULATION_OF_CHEMICAL_SYNAPTIC_TRANSMIS	0.83	0.18	0	0	0	not significant
9669	GO_REGULATION_OF_ENDOTHELIAL_CELL_DEVELOPMENT	0.83	0.18	0	0	0	not significant
9670	NIELSEN_MALIGNAT_FIBROUS_HISTIOCYTOMA_DN	0.83	0.18	0	0	0	not significant
9671	REACTOME_SMALL_INTERFERING_RNA_SIRNA_BIOGENESIS	0.83	0.18	0	0	0	not significant
9672	DACOSTA_ERCC3_ALLELE_XPCS_VS_TTD_DN	0.83	0.17	0	0	0	not significant
9673	GO_PARKIN_MEDIATED_STIMULATION_OF_MITOPHAGY_IN_RESPONSE_T	0.83	0.17	0	0	0	not significant
9674	GO_POSITIVE_REGULATION_OF_CELL_AGING	0.83	0.17	0	0	0	not significant
9675	GO_POSITIVE_REGULATION_OF_FC_GAMMA_RECEPTOR_SIGNALING_PA	0.83	0.17	0	0	0	not significant
9676	GO_POST_EMBRYONIC_EYE_MORPHOGENESIS	0.83	0.17	0	0	0	not significant
9677	KYNG_DNA_DAMAGE_BY_4NQO	0.83	0.17	0	0	0	not significant
9678	REACTOME_NUCLEOTIDE_LIKE_PURINERGIC_RECEPTORS	0.83	0.17	0	0	0	not significant
9679	ABE_INNER_EAR	0.83	0.16	0	0	0	not significant
9680	BIOCARTA_RECK_PATHWAY	0.83	0.16	0	0	0	not significant
9681	KEGG_GLYCOSAMINOGLYCAN_BIOSYNTHESIS_HEPARAN_SULFATE	0.83	0.16	0	0	0	not significant
9682	REACTOME_BLOOD_GROUP_SYSTEMS_BIOSYNTHESIS	0.83	0.16	0	0	0	not significant
9683	REACTOME_NF_KB_ACTIVATION_THROUGH_FADD_RIP_1_PATHWAY_ME	0.83	0.16	0	0	0	not significant
9684	SHIPP_DLBCL_CURED_VS_FATAL_UP	0.83	0.16	0	0	0	not significant
9685	VALK_AML_CLUSTER_8	0.83	0.16	0	0	0	not significant
9686	GO_B_CELL_APOPTOTIC_PROCESS	0.83	0.15	0	0	0	not significant
9687	GO_CARDIAC_MUSCLE_CELL_ACTION_POTENTIAL	0.83	0.15	0	0	0	not significant
9688	GO_EMBRYONIC_SKELETAL_JOINT_DEVELOPMENT	0.83	0.15	0	0	0	not significant
9689	GO_GLYCEROLIPID_CATABOLIC_PROCESS	0.83	0.15	0	0	0	not significant

9690	GO_HOLLIDAY_JUNCTION_RESOLVASE_COMPLEX	0.83	0.15	0	0	0	not significant
9691	GO_KERATINIZATION	0.83	0.15	0	0	0	not significant
9692	GO_MOLTING_CYCLE_PROCESS	0.83	0.15	0	0	0	not significant
9693	GO_NEGATIVE_REGULATION_OF_SYNAPTIC_VESICLE_TRANSPORT	0.83	0.15	0	0	0	not significant
9694	GO_NUCLEOTIDE_SUGAR_BIOSYNTHETIC_PROCESS	0.83	0.15	0	0	0	not significant
9695	GO_OXIDOREDUCTASE_ACTIVITY_ACTING_ON_PAIRED_DONORS_WITH_	0.83	0.15	0	0	0	not significant
9696	GO_POSITIVE_REGULATION_OF_INTERLEUKIN_2_BIOSYNTHETIC_PROCE	0.83	0.15	0	0	0	not significant
9697	GO_PROTEIN_HORMONE_RECEPTOR_ACTIVITY	0.83	0.15	0	0	0	not significant
9698	GO_REGULATION_OF_INTRINSIC_APOPTOTIC_SIGNALING_PATHWAY_BY_	0.83	0.15	0	0	0	not significant
9699	GO_REGULATION_OF_MICROTUBULE_POLYMERIZATION	0.83	0.15	0	0	0	not significant
9700	GO_TRANSFERASE_ACTIVITY_TRANSFERRING_AMINO_ACYL_GROUPS	0.83	0.15	0	0	0	not significant
9701	LEE_LIVER_CANCER_ACOX1_DN	0.83	0.15	0	0	0	not significant
9702	BIOCARTA_CBL_PATHWAY	0.83	0.14	0	0	0	not significant
9703	GO_BONE_MINERALIZATION	0.83	0.14	0	0	0	not significant
9704	GO_ERYTHROCYTE_DEVELOPMENT	0.83	0.14	0	0	0	not significant
9705	GO_EXTRINSIC_COMPONENT_OF_CYTOPLASMIC_SIDE_OF_PLASMA_MEI	0.83	0.14	0	0	0	not significant
9706	GO_GOLGI_TO_PLASMA_MEMBRANE_PROTEIN_TRANSPORT	0.83	0.14	0	0	0	not significant
9707	PID_EPHRINB_REV_PATHWAY	0.83	0.14	0	0	0	not significant
9708	GINESTIER_BREAST_CANCER_ZNF217_AMPLIFIED_UP	0.83	0.13	0	0	0	not significant
9709	GO_EMBRYONIC_SKELETAL_SYSTEM_MORPHOGENESIS	0.83	0.12	0	0	0	not significant
9710	GO_NEGATIVE_REGULATION_OF_PROTEASOMAL_PROTEIN_CATABOLIC	0.83	0.12	0	0	0	not significant
9711	GO_PRESYNAPTIC_MEMBRANE	0.83	0.12	0	0	0	not significant
9712	MCLACHLAN_DENTAL_CARIES_DN	0.83	0.12	0	0	0	not significant
9713	WINTER_HYPOXIA_DN	0.83	0.12	0	0	0	not significant
9714	AMBROSINI_FLAVOPIRIDOL_TREATMENT_TP53	0.83	0.10	0	0	0	not significant
9715	CHR20Q11	0.83	0.10	0	0	0	not significant
9716	GO_ENDONUCLEASE_ACTIVITY	0.83	0.09	0	0	0	not significant
9717	GO_LOCALIZATION_WITHIN_MEMBRANE	0.83	0.09	0	0	0	not significant
9718	GO_PHOSPHATASE_BINDING	0.83	0.09	0	0	0	not significant
9719	GO_PHOSPHATIDYLINOSITOL_BIOSYNTHETIC_PROCESS	0.83	0.09	0	0	0	not significant
9720	GO_RESPONSE_TO_TRANSFORMING_GROWTH_FACTOR_BETA	0.83	0.09	0	0	0	not significant
9721	GO_SIGNAL_RELEASE_FROM_SYNAPSE	0.83	0.09	0	0	0	not significant
9722	NING_CHRONIC_OBSTRUCTIVE_PULMONARY_DISEASE_UP	0.83	0.09	0	0	0	not significant
9723	GO_POSITIVE_REGULATION_OF_I_KAPPAB_KINASE_NF_KAPPAB_SIGNAL	0.83	0.07	0	0	0	not significant
9724	GO_DNA_RECOMBINATION	0.83	0.06	0	0	0	not significant
9725	GO_POSITIVE_REGULATION_OF_CELL_CYCLE_PROCESS	0.83	0.06	0	0	0	not significant
9726	DEURIG_T_CELL_PROLYMPHOCYTIC_LEUKEMIA_DN	0.83	0.05	0	0	0	not significant
9727	GO_POSITIVE_REGULATION_OF_MAPK_CASCADE	0.83	0.05	0	0	0	not significant
9728	GO_CELL_PROJECTION_ASSEMBLY	0.83	0.04	0	0	0	not significant
9729	GO_CELL_MORPHOGENESIS_INVOLVED_IN_DIFFERENTIATION	0.83	0.03	0	0	0	not significant
9730	GO_KINASE_BINDING	0.83	0.02	0	0	0	not significant
9731	GO_POSITIVE_REGULATION_OF_TRANSCRIPTION_BY_RNA_POLYMERAS	0.83	0.02	0	0	0	not significant
9732	GO_TRANSCRIPTION_FACTOR_BINDING	0.83	0.02	0	0	0	not significant
9733	GO_CELL_PROJECTION_PART	0.83	0.01	0	0	0	not significant
9734	GO_INTRACELLULAR_PROTEIN_TRANSPORT	0.83	0.01	0	0	0	not significant
9735	GO_RACEMASE_AND_EPIMERASE_ACTIVITY_ACTING_ON_CARBOHYDRAT	0.84	0.19	0	0	0	not significant
9736	GO_SOMATIC_DIVERSIFICATION_OF_IMMUNE_RECEPTORS_VIA_SOMATI(	0.84	0.19	0	0	0	not significant
9737	GO_LUTEINIZATION	0.84	0.18	0	0	0	not significant
9738	GO_LYMPH_VESSEL_MORPHOGENESIS	0.84	0.18	0	0	0	not significant
9739	GO_MHC_CLASS_I_PROTEIN_BINDING	0.84	0.18	0	0	0	not significant
9740	GO_NEGATIVE_REGULATION_OF_NEUROINFLAMMATORY_RESPONSE	0.84	0.18	0	0	0	not significant
9741	HE_PTEN_TARGETS_DN	0.84	0.18	0	0	0	not significant
9742	WANG_IMMORTALIZED_BY_HOXA9_AND_MEIS1_UP	0.84	0.18	0	0	0	not significant
9743	WENG_POR_TARGETS_LIVER_DN	0.84	0.18	0	0	0	not significant
9744	DING_LUNG_CANCER_MUTATED_RECURRENTLY	0.84	0.17	0	0	0	not significant
9745	GO_CYTOPLASMIC_PATTERN_RECOGNITION_RECEPTOR_SIGNALING_PA	0.84	0.17	0	0	0	not significant
9746	GO_EMBRYONIC_HEMOPOIESIS	0.84	0.17	0	0	0	not significant
9747	GO_HINDBRAIN_MORPHOGENESIS	0.84	0.17	0	0	0	not significant
9748	GO_HOPS_COMPLEX	0.84	0.17	0	0	0	not significant
9749	GO_POSITIVE_REGULATION_OF_CELL_JUNCTION_ASSEMBLY	0.84	0.17	0	0	0	not significant
9750	GO_POSITIVE_REGULATION_OF_DOUBLE_STRAND_BREAK_REPAIR_VIA_	0.84	0.17	0	0	0	not significant
9751	GO_POSITIVE_REGULATION_OF_GRANULOCYTE_DIFFERENTIATION	0.84	0.17	0	0	0	not significant
9752	GO_RIBOSOMAL_SMALL_SUBUNIT_ASSEMBLY	0.84	0.17	0	0	0	not significant
9753	GO_TITIN_BINDING	0.84	0.17	0	0	0	not significant
9754	GO_WNT_SIGNALOSOME	0.84	0.17	0	0	0	not significant
9755	KANG_CISPLATIN_RESISTANCE_DN	0.84	0.17	0	0	0	not significant
9756	MIKKELSEN_MCV6_ICP_WITH_H3K4ME3_AND_H3K27ME3	0.84	0.17	0	0	0	not significant
9757	NABA_BASEMENT_MEMBRANES	0.84	0.17	0	0	0	not significant
9758	REACTOME_O_LINKED_GLYCOSYLATION_OF_MUCINS	0.84	0.17	0	0	0	not significant
9759	REACTOME_TP53_REGULATES_TRANSCRIPTION_OF_CASPASE_ACTIVAT	0.84	0.17	0	0	0	not significant
9760	ZHANG_RESPONSE_TO_CANTHARIDIN_UP	0.84	0.17	0	0	0	not significant
9761	CHR6Q14	0.84	0.16	0	0	0	not significant
9762	GO_DNA_DIRECTED_DNA_POLYMERASE_ACTIVITY	0.84	0.16	0	0	0	not significant
9763	GO_GUANOSINE_CONTAINING_COMPOUND_BIOSYNTHETIC_PROCESS	0.84	0.16	0	0	0	not significant
9764	GO_MYELOID_DENDRITIC_CELL_DIFFERENTIATION	0.84	0.16	0	0	0	not significant
9765	GO_POSITIVE_REGULATION_OF_CELLULAR_RESPONSE_TO_TRANSFOR	0.84	0.16	0	0	0	not significant
9766	GO_POSITIVE_REGULATION_OF_HISTONE_METHYLATION	0.84	0.16	0	0	0	not significant
9767	GO_RESPONSE_TO_SODIUM_ARSENITE	0.84	0.16	0	0	0	not significant
9768	MEISSNER_BRAIN_ICP_WITH_H3K4ME3	0.84	0.16	0	0	0	not significant
9769	MYLLYKANGAS_AMPLIFICATION_HOT_SPOT_5	0.84	0.16	0	0	0	not significant
9770	PID_TCR_RAS_PATHWAY	0.84	0.16	0	0	0	not significant
9771	POS_HISTAMINE_RESPONSE_NETWORK	0.84	0.16	0	0	0	not significant
9772	REACTOME_EFFECTS_OF_PIP2_HYDROLYSIS	0.84	0.16	0	0	0	not significant
9773	BIOCARTA_IL2RB_PATHWAY	0.84	0.15	0	0	0	not significant
9774	CHR17Q23	0.84	0.15	0	0	0	not significant
9775	DARWICHE_PAPILLOMA_PROGRESSION_RISK	0.84	0.15	0	0	0	not significant
9776	GO_LAMELLIPODIUM_MEMBRANE	0.84	0.15	0	0	0	not significant
9777	GO_POLYOL_BIOSYNTHETIC_PROCESS	0.84	0.15	0	0	0	not significant
9778	GO_POLYUBIQUITIN_MODIFICATION_DEPENDENT_PROTEIN_BINDING	0.84	0.15	0	0	0	not significant
9779	DAVICIONI_PAX_FOXO1_SIGNATURE_IN_ARMS_UP	0.84	0.14	0	0	0	not significant
9780	GO_EMBRYONIC_HEART_TUBE_DEVELOPMENT	0.84	0.14	0	0	0	not significant
9781	GO_PROTEIN_K48_LINKED_UBIQUITINATION	0.84	0.14	0	0	0	not significant
9782	GO_REGULATION_OF_T_CELL_MEDIATED_CYTOTOXICITY	0.84	0.14	0	0	0	not significant
9783	HOLLERN_SQUAMOUS_BREAST_TUMOR	0.84	0.14	0	0	0	not significant
9784	PID_NFKAPPAB_CANONICAL_PATHWAY	0.84	0.14	0	0	0	not significant
9785	PRAMOONJAGO_SOX4_TARGETS_UP	0.84	0.14	0	0	0	not significant
9786	GO_REGULATION_OF_SYNAPTIC_PLASTICITY	0.84	0.13	0	0	0	not significant
9787	GO_RESPONSE_TO_ETHANOL	0.84	0.13	0	0	0	not significant
9788	KEGG_ADHERENS_JUNCTION	0.84	0.13	0	0	0	not significant
9789	PLASARI_TGFB1_SIGNALING_VIA_NFIC_1HR_DN	0.84	0.13	0	0	0	not significant
9790	FALVELLA_SMOKERS_WITH_LUNG_CANCER	0.84	0.12	0	0	0	not significant
9791	GO_MOTILE_CILIUM	0.84	0.12	0	0	0	not significant
9792	GO_NUCLEAR_DNA_REPLICATION	0.84	0.12	0	0	0	not significant
9793	GO_NEGATIVE_REGULATION_OF_LEUKOCYTE_CELL_CELL_ADHESION	0.84	0.11	0	0	0	not significant
9794	GO_SECONDARY_ACTIVE_TRANSMEMBRANE_TRANSPORTER_ACTIVITY	0.84	0.11	0	0	0	not significant
9795	REACTOME_CARGO_RECOGNITION_FOR_CLATHRIN_MEDIATED_ENDOCY	0.84	0.11	0	0	0	not significant
9796	GO_ERBB_SIGNALING_PATHWAY	0.84	0.10	0	0	0	not significant
9797	RB_DN.V1_UP	0.84	0.10	0	0	0	not significant
9798	CAIRO_HEPATOBLASTOMA_UP	0.84	0.08	0	0	0	not significant

9799	GO_ANION_TRANSMEMBRANE_TRANSPORT	0.84	0.08	0	0	0	not significant
9800	GO_CILIUM_ORGANIZATION	0.84	0.07	0	0	0	not significant
9801	GO_POSITIVE_REGULATION_OF_LOCOMOTION	0.84	0.07	0	0	0	not significant
9802	DACOSTA_UV_RESPONSE_VIA_ERCC3_UP	0.84	0.06	0	0	0	not significant
9803	GO_ORGANELLE_SUBCOMPARTMENT	0.84	0.06	0	0	0	not significant
9804	GO_REGULATION_OF_CELL_MORPHOGENESIS	0.84	0.06	0	0	0	not significant
9805	GO_SMALL_GTPASE_MEDIATED_SIGNAL_TRANSDUCTION	0.84	0.04	0	0	0	not significant
9806	GO_SYNAPSE	0.84	0.02	0	0	0	not significant
9807	NUYTTEN_NIPP1_TARGETS_DN	0.84	0.02	0	0	0	not significant
9808	GO_NON_MEMBRANE_SPANNING_PROTEIN_TYROSINE_PHOSPHATASE_A	0.85	0.20	0	0	0	not significant
9809	ZEMBUTSU_SENSITIVITY_TO_MITOMYCIN	0.85	0.20	0	0	0	not significant
9810	GO_CELLULAR_POTASSIUM_ION_HOMEOSTASIS	0.85	0.19	0	0	0	not significant
9811	GO_EXTRACELLULAR_VESICLE_BIOGENESIS	0.85	0.19	0	0	0	not significant
9812	GO_LOW_DENSITY_LIPOPROTEIN_PARTICLE	0.85	0.19	0	0	0	not significant
9813	GO_NEGATIVE_REGULATION_OF_CYTOPLASMIC_TRANSLATION	0.85	0.19	0	0	0	not significant
9814	GO_NEGATIVE_REGULATION_OF_ENDOPLASMIC_RETICULUM_UNFOLDEI	0.85	0.19	0	0	0	not significant
9815	GO_POSITIVE_REGULATION_OF_NEURAL_PRECURSOR_CELL_PROLIFER	0.85	0.19	0	0	0	not significant
9816	GO_TELOMERE_MAINTENANCE_IN_RESPONSE_TO_DNA_DAMAGE	0.85	0.19	0	0	0	not significant
9817	GO_TRANSMEMBRANE_RECEPTOR_PROTEIN_PHOSPHATASE_ACTIVITY	0.85	0.19	0	0	0	not significant
9818	GO_VENOUS_BLOOD_VESSEL_MORPHOGENESIS	0.85	0.19	0	0	0	not significant
9819	REACTOME_APEX1_INDEPENDENT_RESOLUTION_OF_AP_SITES_VIA_THE	0.85	0.19	0	0	0	not significant
9820	VANLOO_SP3_TARGETS_UP	0.85	0.19	0	0	0	not significant
9821	BCAT.100_UP.V1_DN	0.85	0.18	0	0	0	not significant
9822	GO_ACTIN_CORTICAL_PATCH_LOCALIZATION	0.85	0.18	0	0	0	not significant
9823	GO_DEVELOPMENT_OF_SECONDARY_SEXUAL_CHARACTERISTICS	0.85	0.18	0	0	0	not significant
9824	GO_PHOSPHORUS_OXYGEN_LYASE_ACTIVITY	0.85	0.18	0	0	0	not significant
9825	GO_POSITIVE_REGULATION_OF_HEAT_GENERATION	0.85	0.18	0	0	0	not significant
9826	GO_PROTEIN_PHOSPHATASE_2A_BINDING	0.85	0.18	0	0	0	not significant
9827	GO_PYRIMIDINE_NUCLEOSIDE_METABOLIC_PROCESS	0.85	0.18	0	0	0	not significant
9828	GO_REGULATION_OF_T_CELL_TOLERANCE_INDUCTION	0.85	0.18	0	0	0	not significant
9829	GO_UDP_N_ACETYLGLUCOSAMINE_BIOSYNTHETIC_PROCESS	0.85	0.18	0	0	0	not significant
9830	HOEGERKORP_CD44_TARGETS_TEMPORAL_UP	0.85	0.18	0	0	0	not significant
9831	REACTOME_O_GLYCOSYLATION_OF_TSR_DOMAIN_CONTAINING_PROTE	0.85	0.18	0	0	0	not significant
9832	BIOCARTA_GRANULOCYTES_PATHWAY	0.85	0.17	0	0	0	not significant
9833	DELACROIX_RAR_TARGETS_UP	0.85	0.17	0	0	0	not significant
9834	GO_CALCIUM_ACTIVATED_CATION_CHANNEL_ACTIVITY	0.85	0.17	0	0	0	not significant
9835	GO_CELLULAR_RESPONSE_TO_SODIUM_ARSENITE	0.85	0.17	0	0	0	not significant
9836	GO_METALLOENDOPEPTIDASE_INHIBITOR_ACTIVITY	0.85	0.17	0	0	0	not significant
9837	GO_POSITIVE_REGULATION_OF_AXON_EXTENSION	0.85	0.17	0	0	0	not significant
9838	GO_POSITIVE_REGULATION_OF_ENDOTHELIAL_CELL_CHEMOTAXIS	0.85	0.17	0	0	0	not significant
9839	GO_POSITIVE_REGULATION_OF_LONG_TERM_SYNAPTIC_POTENTIATION	0.85	0.17	0	0	0	not significant
9840	GO_POSITIVE_REGULATION_OF_PEPTIDYL_LYSINE_ACETYLATION	0.85	0.17	0	0	0	not significant
9841	GO_REGULATION_OF_BILE_ACID_BIOSYNTHETIC_PROCESS	0.85	0.17	0	0	0	not significant
9842	GO_RNA_SECONDARY_STRUCTURE_UNWINDING	0.85	0.17	0	0	0	not significant
9843	KEGG_O_GLYCAN_BIOSYNTHESIS	0.85	0.17	0	0	0	not significant
9844	OKAMOTO_LIVER_CANCER_MULTICENTRIC_OCCURRENCE_UP	0.85	0.17	0	0	0	not significant
9845	REACTOME_ACTIVATION_OF_THE_PRE_REPLICATIVE_COMPLEX	0.85	0.17	0	0	0	not significant
9846	REACTOME_ENDOGENOUS_STEROLS	0.85	0.17	0	0	0	not significant
9847	REACTOME_HYALURONAN_METABOLISM	0.85	0.17	0	0	0	not significant
9848	REACTOME_NEUROTRANSMITTER_RELEASE_CYCLE	0.85	0.17	0	0	0	not significant
9849	CHIARADONNA_NEOPLASTIC_TRANSFORMATION_KRAS_CDC25_UP	0.85	0.16	0	0	0	not significant
9850	GO_REGULATION_OF_DENDRITIC_SPINE_MORPHOGENESIS	0.85	0.16	0	0	0	not significant
9851	MMS_MOUSE_LYMPH_HIGH_4HRS_UP	0.85	0.16	0	0	0	not significant
9852	SEKI_INFLAMMATORY_RESPONSE_LPS_UP	0.85	0.16	0	0	0	not significant
9853	CHR16Q21	0.85	0.15	0	0	0	not significant
9854	DUAN_PRDM5_TARGETS	0.85	0.15	0	0	0	not significant
9855	GO_NEGATIVE_REGULATION_OF_G_PROTEIN_COUPLED_RECEPTOR_SI	0.85	0.15	0	0	0	not significant
9856	GO_PROTEIN_SERINE_THREONINE_TYROSINE_KINASE_ACTIVITY	0.85	0.15	0	0	0	not significant
9857	GO_SMOOTH_MUSCLE_CELL_DIFFERENTIATION	0.85	0.15	0	0	0	not significant
9858	GO_SOMITOGENESIS	0.85	0.15	0	0	0	not significant
9859	PID_HDAC_CLASSII_PATHWAY	0.85	0.15	0	0	0	not significant
9860	GO_INTRINSIC_COMPONENT_OF_POSTSYNAPTIC_MEMBRANE	0.85	0.14	0	0	0	not significant
9861	GO_MICROTUBULE_POLYMERIZATION	0.85	0.14	0	0	0	not significant
9862	GO_POSITIVE_REGULATION_OF_DNA_REPAIR	0.85	0.14	0	0	0	not significant
9863	BERTUCCI_MEDULLARY_VS_DUCTAL_BREAST_CANCER_DN	0.85	0.13	0	0	0	not significant
9864	GO_CILIARY_BASAL_BODY	0.85	0.13	0	0	0	not significant
9865	KIM_GASTRIC_CANCER_CHEMOSENSITIVITY	0.85	0.13	0	0	0	not significant
9866	HALLMARK_UV_RESPONSE_DN	0.85	0.12	0	0	0	not significant
9867	DCA_UP.V1_DN	0.85	0.11	0	0	0	not significant
9868	GO_ENDOPLASMIC_RETICULUM_UNFOLDED_PROTEIN_RESPONSE	0.85	0.11	0	0	0	not significant
9869	MULLIGHAN_NPM1_SIGNATURE_3_DN	0.85	0.10	0	0	0	not significant
9870	CHR17P13	0.85	0.09	0	0	0	not significant
9871	GO_ACTIVATION_OF_PROTEIN_KINASE_ACTIVITY	0.85	0.09	0	0	0	not significant
9872	GO_PROTEIN_AUTOPHOSPHORYLATION	0.85	0.09	0	0	0	not significant
9873	GO_POSITIVE_REGULATION_OF_NEURON_DIFFERENTIATION	0.85	0.08	0	0	0	not significant
9874	GO_TUBULIN_BINDING	0.85	0.08	0	0	0	not significant
9875	GO_KINASE_ACTIVITY	0.85	0.04	0	0	0	not significant
9876	GO_PHARYNGEAL_SYSTEM_DEVELOPMENT	0.86	0.20	0	0	0	not significant
9877	GO_PROTEIN_KINASE_C_ACTIVITY	0.86	0.20	0	0	0	not significant
9878	GO_QUATERNARY_AMMONIUM_GROUP_BINDING	0.86	0.20	0	0	0	not significant
9879	GO_REGULATION_OF_CILIUM_BEAT_FREQUENCY	0.86	0.20	0	0	0	not significant
9880	GO_REGULATION_OF_CILIUM_MOVEMENT	0.86	0.20	0	0	0	not significant
9881	GO_RESPONSE_TO_HYDROXYUREA	0.86	0.20	0	0	0	not significant
9882	GO_SEMINIFEROUS_TUBULE_DEVELOPMENT	0.86	0.20	0	0	0	not significant
9883	NEWMAN_ERCC6_TARGETS_DN	0.86	0.20	0	0	0	not significant
9884	RADAEVA_RESPONSE_TO_IFNA1_DN	0.86	0.20	0	0	0	not significant
9885	REACTOME_TNFR1_MEDIATED_CERAMIDE_PRODUCTION	0.86	0.20	0	0	0	not significant
9886	GO_ALPHA_DNA_POLYMERASE_PRIMASE_COMPLEX	0.86	0.19	0	0	0	not significant
9887	GO_ARF_PROTEIN_SIGNAL_TRANSDUCTION	0.86	0.19	0	0	0	not significant
9888	GO_CELL_PROLIFERATION_IN_HINDBRAIN	0.86	0.19	0	0	0	not significant
9889	GO_JUN_KINASE_BINDING	0.86	0.19	0	0	0	not significant
9890	GO_MULTIVESICULAR_BODY_MEMBRANE	0.86	0.19	0	0	0	not significant
9891	GO_NEGATIVE_REGULATION_OF_ALPHA_BETA_T_CELL_ACTIVATION	0.86	0.19	0	0	0	not significant
9892	GO_NEGATIVE_REGULATION_OF_RECEPTOR_BINDING	0.86	0.19	0	0	0	not significant
9893	GO_REGULATION_OF_ANION_TRANSMEMBRANE_TRANSPORT	0.86	0.19	0	0	0	not significant
9894	GO_REGULATION_OF_CELLULAR_RESPONSE_TO_VASCULAR_ENDOTHE	0.86	0.19	0	0	0	not significant
9895	GO_REGULATION_OF_DNA_DEMETHYLATION	0.86	0.19	0	0	0	not significant
9896	GO_REGULATION_OF_HIPPO_SIGNALING	0.86	0.19	0	0	0	not significant
9897	GO_REGULATION_OF_HISTONE_PHOSPHORYLATION	0.86	0.19	0	0	0	not significant
9898	GO_T_CELL_ACTIVATION_VIA_T_CELL_RECEPTOR_CONTACT_WITH_AN	0.86	0.19	0	0	0	not significant
9899	REACTOME_COLLAGEN_DEGRADATION	0.86	0.19	0	0	0	not significant
9900	REACTOME_FCERI_MEDIATED_CAPLUS2_MOBILIZATION	0.86	0.19	0	0	0	not significant
9901	VETTER_TARGETS_OF_PRKCA_AND_ETS1_DN	0.86	0.19	0	0	0	not significant
9902	BALDWIN_PRKCI_TARGETS_UP	0.86	0.18	0	0	0	not significant
9903	BIOCARTA_MET_PATHWAY	0.86	0.18	0	0	0	not significant
9904	CHR3Q22	0.86	0.18	0	0	0	not significant
9905	GO_BRAIN_MORPHOGENESIS	0.86	0.18	0	0	0	not significant
9906	GO_CYCLOOXYGENASE_PATHWAY	0.86	0.18	0	0	0	not significant
9907	GO_ERBB2_SIGNALING_PATHWAY	0.86	0.18	0	0	0	not significant

9908	GO_GOLGI_TRANS_CISTERNA	0.86	0.18	0	0	0	not significant
9909	GO_HEXOSAMINIDASE_ACTIVITY	0.86	0.18	0	0	0	not significant
9910	GO_MRNA_TRANSCRIPTION	0.86	0.18	0	0	0	not significant
9911	PID_AJDISS_2PATHWAY	0.86	0.18	0	0	0	not significant
9912	REACTOME_REGULATED_PROTEOLYSIS_OF_P75NTR	0.86	0.18	0	0	0	not significant
9913	REACTOME_TRANSPORT_OF_VITAMINS_NUCLEOSIDES_AND_RELATED_	0.86	0.18	0	0	0	not significant
9914	GO_ANTIPORTER_ACTIVITY	0.86	0.17	0	0	0	not significant
9915	GO_AXIS_SPECIFICATION	0.86	0.17	0	0	0	not significant
9916	GO_NUCLEOSIDE_KINASE_ACTIVITY	0.86	0.17	0	0	0	not significant
9917	GO_POSITIVE_REGULATION_OF_PROTEIN_ACETYLATION	0.86	0.17	0	0	0	not significant
9918	GO_REGULATION_OF_CHROMATIN_BINDING	0.86	0.17	0	0	0	not significant
9919	REACTOME_ACTIVATED_TAK1_MEDIATES_P38_MAPK_ACTIVATION	0.86	0.17	0	0	0	not significant
9920	REACTOME_REGULATION_OF_GENE_EXPRESSION_IN_BETA_CELLS	0.86	0.17	0	0	0	not significant
9921	TAKEDA_TARGETS_OF_NUP98_HOXA9_FUSION_6HR_DN	0.86	0.17	0	0	0	not significant
9922	CHR2Q14	0.86	0.16	0	0	0	not significant
9923	GO_LIPOPOLYSACCHARIDE_MEDIATED_SIGNALING_PATHWAY	0.86	0.16	0	0	0	not significant
9924	GO_REGULATION_OF_PHAGOCYTOSIS	0.86	0.16	0	0	0	not significant
9925	KRAS.AMP.LUNG_UP.V1_UP	0.86	0.16	0	0	0	not significant
9926	SHEDDEN_LUNG_CANCER_GOOD_SURVIVAL_A5	0.86	0.16	0	0	0	not significant
9927	CHIBA_RESPONSE_TO_TSA_UP	0.86	0.15	0	0	0	not significant
9928	GAL_LEUKEMIC_STEM_CELL_UP	0.86	0.15	0	0	0	not significant
9929	GO_PROTEIN_AUTOUBIQUITINATION	0.86	0.15	0	0	0	not significant
9930	NIELSEN_GIST	0.86	0.15	0	0	0	not significant
9931	RAGHAVACHARI_PLATELET_SPECIFIC_GENES	0.86	0.15	0	0	0	not significant
9932	CHR14Q32	0.86	0.14	0	0	0	not significant
9933	COULOUARN_TEMPORAL_TGFB1_SIGNATURE_DN	0.86	0.14	0	0	0	not significant
9934	GO_REGULATION_OF_REACTIVE_OXYGEN_SPECIES_BIOSYNTHETIC_PR(	0.86	0.14	0	0	0	not significant
9935	GO_SERINE_THREONINE_PROTEIN_KINASE_COMPLEX	0.86	0.14	0	0	0	not significant
9936	NATSUME_RESPONSE_TO_INTERFERON_BETA_UP	0.86	0.14	0	0	0	not significant
9937	GO_CENTRAL_NERVOUS_SYSTEM_NEURON_DIFFERENTIATION	0.86	0.13	0	0	0	not significant
9938	GO_REGULATION_OF_PEPTIDE_HORMONE_SECRETION	0.86	0.13	0	0	0	not significant
9939	RODWELL_AGING_KIDNEY_DN	0.86	0.13	0	0	0	not significant
9940	GO_INTRINSIC_COMPONENT_OF_ENDOPLASMIC_RETICULUM_MEMBRANI	0.86	0.12	0	0	0	not significant
9941	GO_POSITIVE_REGULATION_OF_PROTEIN_KINASE_B_SIGNALING	0.86	0.12	0	0	0	not significant
9942	PRC2_EED_UP.V1_DN	0.86	0.12	0	0	0	not significant
9943	VERHAAK_AML_WITH_NPM1_MUTATED_UP	0.86	0.12	0	0	0	not significant
9944	BRUINS_UVC_RESPONSE_VIA_TP53_GROUP_D	0.86	0.11	0	0	0	not significant
9945	GO_DEVELOPMENT_OF_PRIMARY_SEXUAL_CHARACTERISTICS	0.86	0.11	0	0	0	not significant
9946	GO_REGULATION_OF_CELLULAR_RESPONSE_TO_GROWTH_FACTOR_S	0.86	0.11	0	0	0	not significant
9947	KASLER_HDAC7_TARGETS_1_UP	0.86	0.10	0	0	0	not significant
9948	GO_REGULATION_OF_INTRACELLULAR_PROTEIN_TRANSPORT	0.86	0.09	0	0	0	not significant
9949	PEDERSEN_METASTASIS_BY_ERBB2_ISOFORM_7	0.86	0.07	0	0	0	not significant
9950	MIKKELSEN_ES_ICP_WITH_H3K4ME3	0.86	0.06	0	0	0	not significant
9951	GO_SOMATODENDRITIC_COMPARTMENT	0.86	0.05	0	0	0	not significant
9952	GO_C_TERMINAL_PROTEIN_LIPIDATION	0.87	0.22	0	0	0	not significant
9953	GO_CYTOKINE_SECRETION_INVOLVED_IN_IMMUNE_RESPONSE	0.87	0.22	0	0	0	not significant
9954	FERRANDO_TAL1_NEIGHBORS	0.87	0.21	0	0	0	not significant
9955	GO_CHRONIC_INFLAMMATORY_RESPONSE	0.87	0.21	0	0	0	not significant
9956	GO_HISTONE_H4_K20_METHYLATION	0.87	0.21	0	0	0	not significant
9957	GO_INHIBITORY_POSTSYNAPTIC_POTENTIAL	0.87	0.21	0	0	0	not significant
9958	GO_MATURE_B_CELL_DIFFERENTIATION	0.87	0.21	0	0	0	not significant
9959	GO_MATURE_B_CELL_DIFFERENTIATION_INVOLVED_IN_IMMUNE_RESPOI	0.87	0.21	0	0	0	not significant
9960	GO_MICROTUBULE_SEVERING_ATPASE_ACTIVITY	0.87	0.21	0	0	0	not significant
9961	GO_NEGATIVE_REGULATION_OF_POTASSIUM_ION_TRANSMEMBRANE_TI	0.87	0.21	0	0	0	not significant
9962	PARK_TRETINOIN_RESPONSE	0.87	0.21	0	0	0	not significant
9963	GENTLES_LEUKEMIC_STEM_CELL_UP	0.87	0.20	0	0	0	not significant
9964	GO_CYCLOSPORIN_A_BINDING	0.87	0.20	0	0	0	not significant
9965	GO_FLUID_TRANSPORT	0.87	0.20	0	0	0	not significant
9966	GO_LYMPHOID_PROGENITOR_CELL_DIFFERENTIATION	0.87	0.20	0	0	0	not significant
9967	GO_NEURAL_TUBE_PATTERNING	0.87	0.20	0	0	0	not significant
9968	GO_PHOSPHATIDYLINOSITOL_PHOSPHATE_5_PHOSPHATASE_ACTIVITY	0.87	0.20	0	0	0	not significant
9969	GO_POSITIVE_REGULATION_OF_GLIAL_CELL_PROLIFERATION	0.87	0.20	0	0	0	not significant
9970	GO_PRIMARY_MIRNA_BINDING	0.87	0.20	0	0	0	not significant
9971	GO_SCHWANN_CELL_PROLIFERATION	0.87	0.20	0	0	0	not significant
9972	GO_SMOOTHENED_SIGNALING_PATHWAY_INVOLVED_IN_DORSAL_VENTI	0.87	0.20	0	0	0	not significant
9973	REACTOME_BIOSYNTHESIS_OF_SPECIALIZED_PRORESOLVING_MEDIATO	0.87	0.20	0	0	0	not significant
9974	REACTOME_CLASS_B_2_SECRETIN_FAMILY_RECEPTORS	0.87	0.20	0	0	0	not significant
9975	REACTOME_FORMATION_OF_THE_CORNIFIED_ENVELOPE	0.87	0.20	0	0	0	not significant
9976	REACTOME_SYNTHESIS_OF_PROSTAGLANDINS_PG_AND_THROMBOXAN	0.87	0.20	0	0	0	not significant
9977	RIZ_ERYTHROID_DIFFERENTIATION_6HR	0.87	0.20	0	0	0	not significant
9978	YAMANAKA_GLIOBLASTOMA_SURVIVAL_DN	0.87	0.20	0	0	0	not significant
9979	BANDRES_RESPONSE_TO_CARMUSTIN_MGMT_48HR_UP	0.87	0.19	0	0	0	not significant
9980	BIOCARTA_PRC2_PATHWAY	0.87	0.19	0	0	0	not significant
9981	CHR16Q12	0.87	0.19	0	0	0	not significant
9982	FOURNIER_ACINAR_DEVELOPMENT_LATE_UP	0.87	0.19	0	0	0	not significant
9983	GALLUZZI_PREVENT_MITOCHONDIAL_PERMEABILIZATION	0.87	0.19	0	0	0	not significant
9984	GO_DNA_POLYMERASE_BINDING	0.87	0.19	0	0	0	not significant
9985	GO_ENDOTHELIAL_CELL_MORPHOGENESIS	0.87	0.19	0	0	0	not significant
9986	GO_ISG15_PROTEIN_CONJUGATION	0.87	0.19	0	0	0	not significant
9987	GO_NUCLEOBASE_TRANSPORT	0.87	0.19	0	0	0	not significant
9988	GO_POSITIVE_REGULATION_OF_MESONEPHROS_DEVELOPMENT	0.87	0.19	0	0	0	not significant
9989	GO_POSITIVE_REGULATION_OF_STEM_CELL_PROLIFERATION	0.87	0.19	0	0	0	not significant
9990	GO_PRP19_COMPLEX	0.87	0.19	0	0	0	not significant
9991	GO_REGULATION_OF_ACTIVIN_RECEPTOR_SIGNALING_PATHWAY	0.87	0.19	0	0	0	not significant
9992	GO_REGULATION_OF_INTRINSIC_APOPTOTIC_SIGNALING_PATHWAY_IN_	0.87	0.19	0	0	0	not significant
9993	REACTOME_INHIBITION_OF_REPLICATION_INITIATION_OF_DAMAGED_DN/	0.87	0.19	0	0	0	not significant
9994	REACTOME_KERATINIZATION	0.87	0.19	0	0	0	not significant
9995	WANG_RESPONSE_TO_BEXAROTENE_UP	0.87	0.19	0	0	0	not significant
9996	FIGUEROA_AML_METHYLATION_CLUSTER_2_UP	0.87	0.18	0	0	0	not significant
9997	FRIDMAN_SENESCENCE_UP	0.87	0.18	0	0	0	not significant
9998	GO_PHAGOSOME_ACIDIFICATION	0.87	0.18	0	0	0	not significant
9999	GO_POSTSYNAPTIC_SPECIALIZATION_INTRACELLULAR_COMPONENT	0.87	0.18	0	0	0	not significant
10000	GO_REGULATION_OF_RAC_PROTEIN_SIGNAL_TRANSDUCTION	0.87	0.18	0	0	0	not significant
10001	HEIDENBLAD_AMPLICON_8Q24_DN	0.87	0.18	0	0	0	not significant
10002	NAGASHIMA_NRG1_SIGNALING_DN	0.87	0.18	0	0	0	not significant
10003	ONO_FOXP3_TARGETS_DN	0.87	0.18	0	0	0	not significant
10004	PID_P38_MKK3_6PATHWAY	0.87	0.18	0	0	0	not significant
10005	REACTOME_CARGO_TRAFFICKING_TO_THE_PERICILIARY_MEMBRANE	0.87	0.18	0	0	0	not significant
10006	REACTOME_HDMS_DEMETHYLATE_HISTONES	0.87	0.18	0	0	0	not significant
10007	GO_CENTRIOLAR_SATELLITE	0.87	0.17	0	0	0	not significant
10008	GO_NATURAL_KILLER_CELL_MEDIATED_IMMUNITY	0.87	0.17	0	0	0	not significant
10009	GO_POSITIVE_REGULATION_OF_PROTEIN_TYROSINE_KINASE_ACTIVITY	0.87	0.17	0	0	0	not significant
10010	GO_CENTROSOME_DUPLICATION	0.87	0.16	0	0	0	not significant
10011	GO_REGULATION_OF_SYNAPTIC_VESICLE_CYCLE	0.87	0.16	0	0	0	not significant
10012	GO_RNA_POLYMERASE_II_CORE_PROMOTER_SEQUENCE_SPECIFIC_DN	0.87	0.16	0	0	0	not significant
10013	LIU_PROSTATE_CANCER_UP	0.87	0.16	0	0	0	not significant
10014	DELASERNA_MYOD_TARGETS_UP	0.87	0.15	0	0	0	not significant
10015	GO_ANTERIOR_POSTERIOR_PATTERN_SPECIFICATION	0.87	0.15	0	0	0	not significant
10016	GO_NEGATIVE_REGULATION_OF_TRANSMEMBRANE_RECEPTOR_PROTE	0.87	0.15	0	0	0	not significant

10017	GO_OSTEOCLAST_DIFFERENTIATION	0.87	0.15	0	0	0	not significant
10018	REACTOME_CELL_SURFACE_INTERACTIONS_AT_THE_VASCULAR_WALL	0.87	0.15	0	0	0	not significant
10019	GO_NEGATIVE_REGULATION_OF_CELLULAR_RESPONSE_TO_GROWTH_	0.87	0.14	0	0	0	not significant
10020	PID_PDGFRB_PATHWAY	0.87	0.14	0	0	0	not significant
10021	RAPA_EARLY_UP.V1_DN	0.87	0.14	0	0	0	not significant
10022	REACTOME_SIGNALING_BY_NOTCH1	0.87	0.14	0	0	0	not significant
10023	GO_EPITHELIAL_CELL_DEVELOPMENT	0.87	0.13	0	0	0	not significant
10024	GO_EXTRINSIC_COMPONENT_OF_MEMBRANE	0.87	0.12	0	0	0	not significant
10025	GO_PHOSPHATIDYLINOSITOL_BINDING	0.87	0.12	0	0	0	not significant
10026	PDGF_ERK_DN.V1_DN	0.87	0.12	0	0	0	not significant
10027	BOYLAN_MULTIPLE_MYELOMA_C_D_DN	0.87	0.11	0	0	0	not significant
10028	CHEMNITZ_RESPONSE_TO_PROSTAGLANDIN_E2_DN	0.87	0.11	0	0	0	not significant
10029	GO_HORMONE_TRANSPORT	0.87	0.11	0	0	0	not significant
10030	GO_IN_UTERO_EMBRYONIC_DEVELOPMENT	0.87	0.10	0	0	0	not significant
10031	GO_POSITIVE_REGULATION_OF_PROTEIN_SERINE_THREONINE_KINASE_	0.87	0.10	0	0	0	not significant
10032	GO_UBIQUITIN_LIKE_PROTEIN_LIGASE_ACTIVITY	0.87	0.10	0	0	0	not significant
10033	RUTELLA_RESPONSE_TO_HGF_VS_CSF2RB_AND_IL4_DN	0.87	0.10	0	0	0	not significant
10034	GO_POSITIVE_REGULATION_OF_CELLULAR_PROTEIN_LOCALIZATION	0.87	0.09	0	0	0	not significant
10035	GO_UBIQUITIN_LIKE_PROTEIN_TRANSFERASE_ACTIVITY	0.87	0.08	0	0	0	not significant
10036	GO_CHROMATIN_BINDING	0.87	0.07	0	0	0	not significant
10037	GO_ENDOTHELIAL_CELL_ACTIVATION	0.88	0.22	0	0	0	not significant
10038	GO_LABYRINTHINE_LAYER_MORPHOGENESIS	0.88	0.22	0	0	0	not significant
10039	GO_REGULATION_OF_LIGASE_ACTIVITY	0.88	0.22	0	0	0	not significant
10040	GO_UDP_XYLOSYLTRANSFERASE_ACTIVITY	0.88	0.22	0	0	0	not significant
10041	BIOCARTA_TERT_PATHWAY	0.88	0.21	0	0	0	not significant
10042	BREUHAHN_GROWTH_FACTOR_SIGNALING_IN_LIVER_CANCER	0.88	0.21	0	0	0	not significant
10043	GO_7SK_SNRNA_BINDING	0.88	0.21	0	0	0	not significant
10044	GO_ATPASE_ACTIVITY_COUPLED_TO_TRANSMEMBRANE_MOVEMENT_O	0.88	0.21	0	0	0	not significant
10045	GO_AXON_ENSHEATHMENT_IN_CENTRAL_NERVOUS_SYSTEM	0.88	0.21	0	0	0	not significant
10046	GO_EMBRYONIC_FORELIMB_MORPHOGENESIS	0.88	0.21	0	0	0	not significant
10047	GO_EXCITATORY_SYNAPSE_ASSEMBLY	0.88	0.21	0	0	0	not significant
10048	GO_NEGATIVE_REGULATION_OF_B_CELL_APOPTOTIC_PROCESS	0.88	0.21	0	0	0	not significant
10049	GO_NEGATIVE_REGULATION_OF_CELL_ADHESION_MEDIATED_BY_INTEG	0.88	0.21	0	0	0	not significant
10050	GO_NEGATIVE_REGULATION_OF_CHROMATIN_SILENCING	0.88	0.21	0	0	0	not significant
10051	GO_POSITIVE_REGULATION_OF_PROTEIN_LOCALIZATION_TO_CENTROS	0.88	0.21	0	0	0	not significant
10052	GO_PRE_B_CELL_DIFFERENTIATION	0.88	0.21	0	0	0	not significant
10053	GO_REGULATION_OF_CHROMATIN_ASSEMBLY_OR_DISASSEMBLY	0.88	0.21	0	0	0	not significant
10054	GO_REGULATION_OF_SPINDLE_CHECKPOINT	0.88	0.21	0	0	0	not significant
10055	GO_SUMO_LIGASE_ACTIVITY	0.88	0.21	0	0	0	not significant
10056	GRAHAM_CML_QUIESCENT_VS_CML_DIVIDING_UP	0.88	0.21	0	0	0	not significant
10057	GUTIERREZ_CHRONIC_LYMPHOCYTIC_LEUKEMIA_UP	0.88	0.21	0	0	0	not significant
10058	KEGG_NICOTINATE_AND_NICOTINAMIDE_METABOLISM	0.88	0.21	0	0	0	not significant
10059	MARKS_HDAC_TARGETS_UP	0.88	0.21	0	0	0	not significant
10060	REACTOME_BH3_ONLY_PROTEINS_ASSOCIATE_WITH_AND_INACTIVATE_	0.88	0.21	0	0	0	not significant
10061	CHR6Q23	0.88	0.20	0	0	0	not significant
10062	GO_DEVELOPMENT_OF_SECONDARY_FEMALE_SEXUAL_CHARACTERIST	0.88	0.20	0	0	0	not significant
10063	GO_MONOOXYGENASE_ACTIVITY	0.88	0.20	0	0	0	not significant
10064	GO_POSITIVE_REGULATION_OF_DOUBLE_STRAND_BREAK_REPAIR	0.88	0.20	0	0	0	not significant
10065	GO_POSITIVE_REGULATION_OF_T_CELL_MEDIATED_CYTOTOXICITY	0.88	0.20	0	0	0	not significant
10066	GO_PROTECTION_FROM_NON_HOMOLOGOUS_END_JOINING_AT_TELOM	0.88	0.20	0	0	0	not significant
10067	GO_PROTON_TRANSPORTING_ATPASE_ACTIVITY_ROTATIONAL_MECHAI	0.88	0.20	0	0	0	not significant
10068	GO_REGULATION_OF_LIPOPOLYSACCHARIDE_MEDIATED_SIGNALING_PA	0.88	0.20	0	0	0	not significant
10069	GO_REGULATION_OF_RESPIRATORY_GASEOUS_EXCHANGE	0.88	0.20	0	0	0	not significant
10070	REACTOME_ASSOCIATION_OF_TRIC_CCT_WITH_TARGET_PROTEINS_DU	0.88	0.20	0	0	0	not significant
10071	BIOCARTA_TNFR2_PATHWAY	0.88	0.19	0	0	0	not significant
10072	GO_CLATHRIN_DEPENDENT_ENDOCYTOSIS	0.88	0.19	0	0	0	not significant
10073	GO_DIAPEDESIS	0.88	0.19	0	0	0	not significant
10074	GO_GAMMA_CATENIN_BINDING	0.88	0.19	0	0	0	not significant
10075	GO_PHOSPHATIDYLCHOLINE_TRANSPORTER_ACTIVITY	0.88	0.19	0	0	0	not significant
10076	GO_POSITIVE_REGULATION_OF_T_CELL_DIFFERENTIATION_IN_THYMUS	0.88	0.19	0	0	0	not significant
10077	GO_PROTEIN_POLYUFMYLATION	0.88	0.19	0	0	0	not significant
10078	GO_REGULATION_OF_CILIUM_ASSEMBLY	0.88	0.19	0	0	0	not significant
10079	GO_REGULATION_OF_RECEPTOR_INTERNALIZATION	0.88	0.19	0	0	0	not significant
10080	GO_SMOOTH_MUSCLE_CONTRACTION	0.88	0.19	0	0	0	not significant
10081	GO_XENOBIOTIC_TRANSPORT	0.88	0.19	0	0	0	not significant
10082	KAMMINGA_SENESCENCE	0.88	0.19	0	0	0	not significant
10083	PID_TNF_PATHWAY	0.88	0.19	0	0	0	not significant
10084	PIONTEK_PKD1_TARGETS_DN	0.88	0.19	0	0	0	not significant
10085	REACTOME_DEACTIVATION_OF_THE_BETA_CATENIN_TRANSACTIVATING	0.88	0.19	0	0	0	not significant
10086	REACTOME_O_LINKED_GLYCOSYLATION	0.88	0.19	0	0	0	not significant
10087	ROVERSI_GLIOMA_COPY_NUMBER_UP	0.88	0.19	0	0	0	not significant
10088	BACOLOD_RESISTANCE_TO_ALKYLATING_AGENTS_DN	0.88	0.18	0	0	0	not significant
10089	GEORGANTAS_HSC_MARKERS	0.88	0.18	0	0	0	not significant
10090	GO_LUMENAL_SIDE_OF_MEMBRANE	0.88	0.18	0	0	0	not significant
10091	GO_POSITIVE_REGULATION_OF_NUCLEAR_DIVISION	0.88	0.18	0	0	0	not significant
10092	GO_PROTEIN_K48_LINKED_DEUBIQUITINATION	0.88	0.18	0	0	0	not significant
10093	GO_REGULATION_OF_LEUKOCYTE_CHEMOTAXIS	0.88	0.18	0	0	0	not significant
10094	DIRMEIER_LMP1_RESPONSE_EARLY	0.88	0.17	0	0	0	not significant
10095	GO_METALLOEXOPEPTIDASE_ACTIVITY	0.88	0.17	0	0	0	not significant
10096	KATSANOU_ELAVL1_TARGETS_UP	0.88	0.17	0	0	0	not significant
10097	WAMUNYOKOLI_OVARIAN_CANCER_GRADES_1_2_DN	0.88	0.17	0	0	0	not significant
10098	EIF4E_DN	0.88	0.16	0	0	0	not significant
10099	GO_ACTION_POTENTIAL	0.88	0.16	0	0	0	not significant
10100	GO_ANCHORED_COMPONENT_OF_MEMBRANE	0.88	0.16	0	0	0	not significant
10101	GO_ENDOSOME_ORGANIZATION	0.88	0.16	0	0	0	not significant
10102	LIU_VMYB_TARGETS_UP	0.88	0.16	0	0	0	not significant
10103	MCBRYAN_PUBERTAL_BREAST_5_6WK_UP	0.88	0.16	0	0	0	not significant
10104	GO_POSITIVE_REGULATION_OF_CELL_MORPHOGENESIS_INVOLVED_IN_	0.88	0.15	0	0	0	not significant
10105	GO_SENSORY_PERCEPTION_OF_LIGHT_STIMULUS	0.88	0.15	0	0	0	not significant
10106	GO_TRANSFORMING_GROWTH_FACTOR_BETA_RECEPTOR_SIGNALING_	0.88	0.15	0	0	0	not significant
10107	MCBRYAN_PUBERTAL_BREAST_5_6WK_DN	0.88	0.15	0	0	0	not significant
10108	GO_HINDBRAIN_DEVELOPMENT	0.88	0.14	0	0	0	not significant
10109	GO_MUSCLE_CELL_PROLIFERATION	0.88	0.13	0	0	0	not significant
10110	GO_REGULATION_OF_RESPONSE_TO_DNA_DAMAGE_STIMULUS	0.88	0.13	0	0	0	not significant
10111	GO_DNA_BINDING_TRANSCRIPTION_ACTIVATOR_ACTIVITY	0.88	0.12	0	0	0	not significant
10112	GO_AXON	0.88	0.10	0	0	0	not significant
10113	GO_CELL_PART_MORPHOGENESIS	0.88	0.09	0	0	0	not significant
10114	GO_VESICLE_MEMBRANE	0.88	0.09	0	0	0	not significant
10115	GO_NERVOUS_SYSTEM_PROCESS	0.88	0.08	0	0	0	not significant
10116	MILI_PSEUDOPODIA_CHEMOTAXIS_DN	0.88	0.08	0	0	0	not significant
10117	BENPORATH_SOX2_TARGETS	0.88	0.07	0	0	0	not significant
10118	GO_ZINC_ION_BINDING	0.88	0.07	0	0	0	not significant
10119	KRIEG_HYPOXIA_NOT_VIA_KDM3A	0.88	0.07	0	0	0	not significant
10120	ODONNELL_METASTASIS_DN	0.89	0.24	0	0	0	not significant
10121	GO_CELLULAR_ANION_HOMEOSTASIS	0.89	0.23	0	0	0	not significant
10122	GO_NEGATIVE_REGULATION_OF_LYMPHOCYTE_MIGRATION	0.89	0.23	0	0	0	not significant
10123	GO_REGULATION_OF_RECEPTOR_BINDING	0.89	0.23	0	0	0	not significant
10124	GO_SLEEP	0.89	0.23	0	0	0	not significant
10125	KEGG_GLYCOSAMINOGLYCAN_BIOSYNTHESIS_KERATAN_SULFATE	0.89	0.23	0	0	0	not significant

10126	REACTOME_TRANSCRIPTIONAL_REGULATION_OF_PLURIPOTENT_STEM_	0.89	0.23	0	0	0	not significant
10127	DISTECHE_ESCAPED_FROM_X_INACTIVATION	0.89	0.22	0	0	0	not significant
10128	GO_DNA_CATABOLIC_PROCESS_EXONUCLEOLYTIC	0.89	0.22	0	0	0	not significant
10129	GO_HETEROPHILIC_CELL_CELL_ADHESION_VIA_PLASMA_MEMBRANE_CI	0.89	0.22	0	0	0	not significant
10130	GO_INTERLEUKIN_2_BIOSYNTHETIC_PROCESS	0.89	0.22	0	0	0	not significant
10131	GO_ORIGIN_RECOGNITION_COMPLEX	0.89	0.22	0	0	0	not significant
10132	GO_PHOSPHATIDYLINOSITOL_4_5_BISPHOSPHATE_5_PHOSPHATASE_AC	0.89	0.22	0	0	0	not significant
10133	GO_POSITIVE_REGULATION_OF_CHROMATIN_BINDING	0.89	0.22	0	0	0	not significant
10134	GO_POSITIVE_REGULATION_OF_HISTONE_H3_K9_ACETYLATION	0.89	0.22	0	0	0	not significant
10135	GO_REGULATION_OF_ARP2_3_COMPLEX_MEDIATED_ACTIN_NUCLEATIO	0.89	0.22	0	0	0	not significant
10136	GO_REGULATION_OF_AUTOPHAGY_OF_MITOCHONDRION_IN_RESPONSE	0.89	0.22	0	0	0	not significant
10137	SAKAI_TUMOR_INFILTRATING_MONOCYTES_UP	0.89	0.22	0	0	0	not significant
10138	BURTON_ADIPOGENESIS_9	0.89	0.21	0	0	0	not significant
10139	CHR8Q22	0.89	0.21	0	0	0	not significant
10140	GO_GLYCOSYLPHOSPHATIDYLINOSITOL_N_ACETYLGLUCOSAMINYLTRAN	0.89	0.21	0	0	0	not significant
10141	GO_RENAL_SYSTEM_PROCESS_INVOLVED_IN_REGULATION_OF_SYSTEI	0.89	0.21	0	0	0	not significant
10142	GO_SEGMENT_SPECIFICATION	0.89	0.21	0	0	0	not significant
10143	GO_UNCONVENTIONAL_MYOSIN_COMPLEX	0.89	0.21	0	0	0	not significant
10144	KEGG_HEDGEHOG_SIGNALING_PATHWAY	0.89	0.21	0	0	0	not significant
10145	PAL_PRMT5_TARGETS_DN	0.89	0.21	0	0	0	not significant
10146	PARK_TRETINOIN_RESPONSE_AND_RARA_PLZF_FUSION	0.89	0.21	0	0	0	not significant
10147	REACTOME_PLASMA_LIPOPROTEIN_REMODELING	0.89	0.21	0	0	0	not significant
10148	SHIN_B_CELL_LYMPHOMA_CLUSTER_8	0.89	0.21	0	0	0	not significant
10149	ZHAN_MULTIPLE_MYELOMA_MS_DN	0.89	0.21	0	0	0	not significant
10150	BIOCARTA_DC_PATHWAY	0.89	0.20	0	0	0	not significant
10151	FIRESTEIN_CTNNB1_PATHWAY	0.89	0.20	0	0	0	not significant
10152	GO_CARBOHYDRATE_DERIVATIVE_TRANSMEMBRANE_TRANSPORTER_A	0.89	0.20	0	0	0	not significant
10153	GO_GALACTOSIDASE_ACTIVITY	0.89	0.20	0	0	0	not significant
10154	GO_NEGATIVE_REGULATION_OF_COLD_INDUCED_THERMOGENESIS	0.89	0.20	0	0	0	not significant
10155	GO_POLYSACCHARIDE_METABOLIC_PROCESS	0.89	0.20	0	0	0	not significant
10156	GO_POSITIVE_REGULATION_OF_CIRCADIAN_RHYTHM	0.89	0.20	0	0	0	not significant
10157	GO_PRESYNAPTIC_ACTIVE_ZONE	0.89	0.20	0	0	0	not significant
10158	GO_REGULATION_OF_MEMBRANE_TUBULATION	0.89	0.20	0	0	0	not significant
10159	GO_RETINAL_ROD_CELL_DEVELOPMENT	0.89	0.20	0	0	0	not significant
10160	REACTOME_NOTCH3_ACTIVATION_AND_TRANSMISSION_OF_SIGNAL_TO	0.89	0.20	0	0	0	not significant
10161	REACTOME_SUMOYLATION_OF_TRANSCRIPTION_COFACTORS	0.89	0.20	0	0	0	not significant
10162	VANHARANTA_UTERINE_FIBROID_WITH_7Q_DELETION_DN	0.89	0.20	0	0	0	not significant
10163	ZEMBUTSU_SENSITIVITY_TO_CISPLATIN	0.89	0.20	0	0	0	not significant
10164	BILD_SRC_ONCOGENIC_SIGNATURE	0.89	0.19	0	0	0	not significant
10165	CHUNG_BLISTER_CYTOTOXICITY_DN	0.89	0.19	0	0	0	not significant
10166	GO_CILIARY_MEMBRANE	0.89	0.19	0	0	0	not significant
10167	GO_KINASE_INHIBITOR_ACTIVITY	0.89	0.19	0	0	0	not significant
10168	GO_NUCLEOBASE_CONTAINING_COMPOUND_TRANSMEMBRANE_TRANSI	0.89	0.19	0	0	0	not significant
10169	GO_O_GLYCAN_PROCESSING	0.89	0.19	0	0	0	not significant
10170	GO_PLACENTA_DEVELOPMENT	0.89	0.19	0	0	0	not significant
10171	HALLMARK_TGF_BETA_SIGNALING	0.89	0.19	0	0	0	not significant
10172	KEGG_HYPERTROPHIC_CARDIOMYOPATHY_HCM	0.89	0.19	0	0	0	not significant
10173	RIGGINS_TAMOXIFEN_RESISTANCE_UP	0.89	0.19	0	0	0	not significant
10174	GO_AUTOPHAGY_OF_MITOCHONDRION	0.89	0.18	0	0	0	not significant
10175	GO_REGULATION_OF_CELLULAR_RESPONSE_TO_TRANSFORMING_GRC	0.89	0.18	0	0	0	not significant
10176	POOLA_INVASIVE_BREAST_CANCER_DN	0.89	0.18	0	0	0	not significant
10177	GO_ACTIVATION_OF_MAPK_ACTIVITY	0.89	0.17	0	0	0	not significant
10178	GO_ERYTHROCYTE_HOMEOSTASIS	0.89	0.17	0	0	0	not significant
10179	GO_NEGATIVE_REGULATION_OF_CELL_CELL_ADHESION	0.89	0.17	0	0	0	not significant
10180	GO_NEURON_PROJECTION_EXTENSION	0.89	0.17	0	0	0	not significant
10181	GO_NOTCH_SIGNALING_PATHWAY	0.89	0.17	0	0	0	not significant
10182	GO_REGULATION_OF_LIPASE_ACTIVITY	0.89	0.17	0	0	0	not significant
10183	GREGORY_SYNTHETIC_LETHAL_WITH_IMATINIB	0.89	0.16	0	0	0	not significant
10184	YAGI_AML_WITH_T_9_11_TRANSLOCATION	0.89	0.16	0	0	0	not significant
10185	GO_MITOTIC_CELL_CYCLE_CHECKPOINT	0.89	0.14	0	0	0	not significant
10186	MONNIER_POSTRADIATION_TUMOR_ESCAPE_DN	0.89	0.10	0	0	0	not significant
10187	GO_REGULATION_OF_NERVOUS_SYSTEM_DEVELOPMENT	0.89	0.08	0	0	0	not significant
10188	MARKS_HDAC_TARGETS_DN	0.90	0.25	0	0	0	not significant
10189	GO_FOREBRAIN_MORPHOGENESIS	0.90	0.24	0	0	0	not significant
10190	GO_THROMBIN_ACTIVATED_RECEPTOR_SIGNALING_PATHWAY	0.90	0.24	0	0	0	not significant
10191	NOJIMA_SFRP2_TARGETS_DN	0.90	0.24	0	0	0	not significant
10192	REACTOME_NF_KB_IS_ACTIVATED_AND_SIGNALS_SURVIVAL	0.90	0.24	0	0	0	not significant
10193	GO_INOSITOL_TRISPHOSPHATE_BIOSYNTHETIC_PROCESS	0.90	0.23	0	0	0	not significant
10194	GO_MICROTUBULE_MINUS_END	0.90	0.23	0	0	0	not significant
10195	GO_PEPTIDYL_SERINE_DEPHOSPHORYLATION	0.90	0.23	0	0	0	not significant
10196	GO_PHAGOPHORE_ASSEMBLY_SITE_MEMBRANE	0.90	0.23	0	0	0	not significant
10197	GO_POSITIVE_REGULATION_OF_GLYCOPROTEIN_BIOSYNTHETIC_PROCI	0.90	0.23	0	0	0	not significant
10198	GO_REGULATION_OF_ACTIN_FILAMENT_BASED_MOVEMENT	0.90	0.23	0	0	0	not significant
10199	GO_TORC2_SIGNALING	0.90	0.23	0	0	0	not significant
10200	GO_TRANSEPITHELIAL_TRANSPORT	0.90	0.23	0	0	0	not significant
10201	GO_VOLTAGE_GATED_ANION_CHANNEL_ACTIVITY	0.90	0.23	0	0	0	not significant
10202	HASINA_NOL7_TARGETS_UP	0.90	0.23	0	0	0	not significant
10203	MCGOWAN_RSP6_TARGETS_UP	0.90	0.23	0	0	0	not significant
10204	WANG_TNF_TARGETS	0.90	0.23	0	0	0	not significant
10205	BIOCARTA_GABA_PATHWAY	0.90	0.22	0	0	0	not significant
10206	GO_ATP_DEPENDENT_PROTEIN_BINDING	0.90	0.22	0	0	0	not significant
10207	GO_BONE_RESORPTION	0.90	0.22	0	0	0	not significant
10208	GO_CILIUM_MOVEMENT_INVOLVED_IN_CELL_MOTILITY	0.90	0.22	0	0	0	not significant
10209	GO_FRIZZLED_BINDING	0.90	0.22	0	0	0	not significant
10210	GO_MUSCLE_ATROPHY	0.90	0.22	0	0	0	not significant
10211	GO_NOTCH_RECEPTOR_PROCESSING_LIGAND_DEPENDENT	0.90	0.22	0	0	0	not significant
10212	GO_NUCLEOTIDE_EXCISION_REPAIR_COMPLEX	0.90	0.22	0	0	0	not significant
10213	GO_RELAXATION_OF_CARDIAC_MUSCLE	0.90	0.22	0	0	0	not significant
10214	GO_RESPONSE_TO_COBALT_ION	0.90	0.22	0	0	0	not significant
10215	GO_STEM_CELL_DIVISION	0.90	0.22	0	0	0	not significant
10216	GO_SYNAPTIC_GROWTH_AT_NEUROMUSCULAR_JUNCTION	0.90	0.22	0	0	0	not significant
10217	HALLMARK_HEDGEHOG_SIGNALING	0.90	0.22	0	0	0	not significant
10218	HUI_MAPK14_TARGETS_UP	0.90	0.22	0	0	0	not significant
10219	WANG_METASTASIS_OF_BREAST_CANCER	0.90	0.22	0	0	0	not significant
10220	BERTUCCI_INVASIVE_CARCINOMA_DUCTAL_VS_LOBULAR_UP	0.90	0.21	0	0	0	not significant
10221	GO_EXCRETION	0.90	0.21	0	0	0	not significant
10222	GO_FORMATION_OF_PRIMARY_GERM_LAYER	0.90	0.21	0	0	0	not significant
10223	GO_IMMUNOLOGICAL_SYNAPSE	0.90	0.21	0	0	0	not significant
10224	GO_NEGATIVE_REGULATION_OF_INTERLEUKIN_1_BETA_PRODUCTION	0.90	0.21	0	0	0	not significant
10225	GO_POSITIVE_REGULATION_OF_CYSTEINE_TYPE_ENDOPEPTIDASE_ACT	0.90	0.21	0	0	0	not significant
10226	GO_PURINE_NUCLEOSIDE_MONOPHOSPHATE_BIOSYNTHETIC_PROCESS	0.90	0.21	0	0	0	not significant
10227	GO_REGULATION_OF_LYMPHOID_PROGENITOR_CELL_DIFFERENTIATIOI	0.90	0.21	0	0	0	not significant
10228	GO_RESPONSE_TO_INTERLEUKIN_4	0.90	0.21	0	0	0	not significant
10229	LUI_THYROID_CANCER_PAX8_PPARG_UP	0.90	0.21	0	0	0	not significant
10230	REACTOME_ACTIVATION_OF_THE_TFAP2_AP_2_FAMILY_OF_TRANSCRII	0.90	0.21	0	0	0	not significant
10231	REACTOME_CELL_CELL_COMMUNICATION	0.90	0.21	0	0	0	not significant
10232	REACTOME_SHC_MEDIATED_CASCADE:FGFR1	0.90	0.21	0	0	0	not significant
10233	REACTOME_TNFR1_INDUCED_NFKAPPAB_SIGNALING_PATHWAY	0.90	0.21	0	0	0	not significant
10234	SCIBETTA_KDM5B_TARGETS_UP	0.90	0.21	0	0	0	not significant

10235	WESTON_VEGFA_TARGETS	0.90	0.21	0	0	0	not significant
10236	GO_BETA_TUBULIN_BINDING	0.90	0.20	0	0	0	not significant
10237	GO_MEIOTIC_CHROMOSOME_SEGREGATION	0.90	0.20	0	0	0	not significant
10238	GO_REGULATION_OF_MYELOID_LEUKOCYTE_DIFFERENTIATION	0.90	0.20	0	0	0	not significant
10239	GO_RRNA_BINDING	0.90	0.20	0	0	0	not significant
10240	GO_SYNAPTONEMAL_STRUCTURE	0.90	0.20	0	0	0	not significant
10241	HENDRICKS_SMARCA4_TARGETS_UP	0.90	0.20	0	0	0	not significant
10242	REACTOME_REGULATION_OF_PTEN_GENE_TRANSCRIPTION	0.90	0.20	0	0	0	not significant
10243	GO_RESPONSE_TO_ACTIVITY	0.90	0.19	0	0	0	not significant
10244	KEGG_SMALL_CELL_LUNG_CANCER	0.90	0.19	0	0	0	not significant
10245	KEGG_WNT_SIGNALING_PATHWAY	0.90	0.19	0	0	0	not significant
10246	XU_HGF_TARGETS_REPRESSED_BY_AKT1_DN	0.90	0.19	0	0	0	not significant
10247	GO_PEPTIDYL_LYSINE_METHYLATION	0.90	0.18	0	0	0	not significant
10248	GO_REGULATION_OF_CELL_SIZE	0.90	0.18	0	0	0	not significant
10249	GO_REGULATION_OF_PH	0.90	0.18	0	0	0	not significant
10250	SANSOM_APC_TARGETS_UP	0.90	0.18	0	0	0	not significant
10251	GO_MAMMARY_GLAND_DEVELOPMENT	0.90	0.17	0	0	0	not significant
10252	GO_REGULATION_OF_MYELOID_CELL_DIFFERENTIATION	0.90	0.17	0	0	0	not significant
10253	KYNG_DNA_DAMAGE_UP	0.90	0.17	0	0	0	not significant
10254	PECE_MAMMARY_STEM_CELL_DN	0.90	0.17	0	0	0	not significant
10255	BROWNE_HCMV_INFECTION_4HR_DN	0.90	0.16	0	0	0	not significant
10256	GO_DNA_INTEGRITY_CHECKPOINT	0.90	0.16	0	0	0	not significant
10257	GO_POSITIVE_REGULATION_OF_INTRACELLULAR_TRANSPORT	0.90	0.16	0	0	0	not significant
10258	GO_RESPONSE_TO_UV	0.90	0.16	0	0	0	not significant
10259	GO_POSITIVE_REGULATION_OF_NEURON_PROJECTION_DEVELOPMENT	0.90	0.15	0	0	0	not significant
10260	SCHAEFFER_PROSTATE_DEVELOPMENT_48HR_UP	0.90	0.15	0	0	0	not significant
10261	GO_REGULATION_OF_PROTEIN_COMPLEX_ASSEMBLY	0.90	0.14	0	0	0	not significant
10262	GO_SENSORY_ORGAN_DEVELOPMENT	0.90	0.14	0	0	0	not significant
10263	RATTENBACHER_BOUND_BY_CELF1	0.90	0.13	0	0	0	not significant
10264	GO_DENDRITIC_TREE	0.90	0.12	0	0	0	not significant
10265	GO_SIGNAL_RELEASE	0.90	0.12	0	0	0	not significant
10266	GO_VACUOLAR_PART	0.90	0.12	0	0	0	not significant
10267	PARENT_MTOR_SIGNALING_UP	0.90	0.11	0	0	0	not significant
10268	GO_CARBOHYDRATE_DERIVATIVE_BIOSYNTHETIC_PROCESS	0.90	0.10	0	0	0	not significant
10269	GO_PLASMA_MEMBRANE_REGION	0.90	0.10	0	0	0	not significant
10270	GO_EMBRYO_DEVELOPMENT	0.90	0.09	0	0	0	not significant
10271	GO_POSITIVE_REGULATION_OF_SIGNALING	0.90	0.07	0	0	0	not significant
10272	REACTOME_GENE_EXPRESSION_TRANSCRIPTION	0.90	0.07	0	0	0	not significant
10273	GO_ADRENERGIC_RECEPTOR_BINDING	0.91	0.25	0	0	0	not significant
10274	GO_INTERLEUKIN_17_PRODUCTION	0.91	0.25	0	0	0	not significant
10275	GO_ARMADILLO_REPEAT_DOMAIN_BINDING	0.91	0.24	0	0	0	not significant
10276	GO_ESCRT_I_COMPLEX	0.91	0.24	0	0	0	not significant
10277	GO_EXPLORATION_BEHAVIOR	0.91	0.24	0	0	0	not significant
10278	GO_GTP_BIOSYNTHETIC_PROCESS	0.91	0.24	0	0	0	not significant
10279	GO_HEPARIN_BIOSYNTHETIC_PROCESS	0.91	0.24	0	0	0	not significant
10280	REACTOME_COBALAMIN_CBL_VITAMIN_B12_TRANSPORT_AND_METABO	0.91	0.24	0	0	0	not significant
10281	REACTOME_HS_GAG_DEGRADATION	0.91	0.24	0	0	0	not significant
10282	REACTOME_P75NTR_SIGNALS_VIA_NF_KB	0.91	0.24	0	0	0	not significant
10283	SUZUKI_CTCFL_TARGETS_UP	0.91	0.24	0	0	0	not significant
10284	ZERBINI_RESPONSE_TO_SULINDAC_UP	0.91	0.24	0	0	0	not significant
10285	BIOCARTA_BTG2_PATHWAY	0.91	0.23	0	0	0	not significant
10286	CHOW_RASSF1_TARGETS_DN	0.91	0.23	0	0	0	not significant
10287	GO_14_3_3_PROTEIN_BINDING	0.91	0.23	0	0	0	not significant
10288	GO_CORE_PROMOTER_BINDING	0.91	0.23	0	0	0	not significant
10289	GO_NADPLUS_BINDING	0.91	0.23	0	0	0	not significant
10290	GO_NEGATIVE_REGULATION_OF_DNA_DEPENDENT_DNA_REPLICATION	0.91	0.23	0	0	0	not significant
10291	GO_NEGATIVE_REGULATION_OF_SYSTEMIC_ARTERIAL_BLOOD_PRESSU	0.91	0.23	0	0	0	not significant
10292	GO_POSITIVE_REGULATION_OF_ADENYLATE_CYCLASE_ACTIVITY	0.91	0.23	0	0	0	not significant
10293	GO_PRO_B_CELL_DIFFERENTIATION	0.91	0.23	0	0	0	not significant
10294	GO_PROTEIN_TRANSPORT_WITHIN_LIPID_BILAYER	0.91	0.23	0	0	0	not significant
10295	GO_RESPONSE_TO_INTERLEUKIN_6	0.91	0.23	0	0	0	not significant
10296	GO_TROPHOBLAST_GIANT_CELL_DIFFERENTIATION	0.91	0.23	0	0	0	not significant
10297	HERNANDEZ_ABERRANT_MITOSIS_BY_DOCETACEL_2NM_UP	0.91	0.23	0	0	0	not significant
10298	IYENGAR_RESPONSE_TO_ADIPOCYTE_FACTORS	0.91	0.23	0	0	0	not significant
10299	KRAS.BREAST_UP.V1_UP	0.91	0.23	0	0	0	not significant
10300	LIN_TUMOR_ESCAPE_FROM_IMMUNE_ATTACK	0.91	0.23	0	0	0	not significant
10301	MISHRA_CARCINOMA_ASSOCIATED_FIBROBLAST_DN	0.91	0.23	0	0	0	not significant
10302	REACTOME_G0_AND_EARLY_G1	0.91	0.23	0	0	0	not significant
10303	SANCHEZ_MDM2_TARGETS	0.91	0.23	0	0	0	not significant
10304	ZHAN_LATE_DIFFERENTIATION_GENES_DN	0.91	0.23	0	0	0	not significant
10305	BCAT_GDS748_UP	0.91	0.22	0	0	0	not significant
10306	GO_ADHERENS_JUNCTION_ASSEMBLY	0.91	0.22	0	0	0	not significant
10307	GO_B_CELL_RECEPTOR_SIGNALING_PATHWAY	0.91	0.22	0	0	0	not significant
10308	GO_CELL_DIFFERENTIATION_INVOLVED_IN_EMBRYONIC_PLACENTA_DEV	0.91	0.22	0	0	0	not significant
10309	GO_HEART_VALVE_FORMATION	0.91	0.22	0	0	0	not significant
10310	GO_N_TERMINAL_PROTEIN_LIPIDATION	0.91	0.22	0	0	0	not significant
10311	GO_PROTEIN_GERANYLGERANYLATION	0.91	0.22	0	0	0	not significant
10312	GO_RESPONSE_TO_AMYLOID_BETA	0.91	0.22	0	0	0	not significant
10313	GO_SCAR_COMPLEX	0.91	0.22	0	0	0	not significant
10314	GRANDVAUX_IRF3_TARGETS_DN	0.91	0.22	0	0	0	not significant
10315	GUILLAUMOND_KLF10_TARGETS_UP	0.91	0.22	0	0	0	not significant
10316	REACTOME_FATTY_ACIDS_BOUND_TO_GPR40_FFAR1_REGULATE_INSU	0.91	0.22	0	0	0	not significant
10317	GO_CELLULAR_RESPONSE_TO_ANTIBIOTIC	0.91	0.21	0	0	0	not significant
10318	GO_GROWTH_FACTOR_BINDING	0.91	0.21	0	0	0	not significant
10319	GO_NAD_DEPENDENT_PROTEIN_DEACETYLASE_ACTIVITY	0.91	0.21	0	0	0	not significant
10320	GO_PHOSPHATIDYLINOSITOL_BISPHOSPHATE_BINDING	0.91	0.21	0	0	0	not significant
10321	PID_RB_1PATHWAY	0.91	0.21	0	0	0	not significant
10322	REACTOME_L1CAM_INTERACTIONS	0.91	0.21	0	0	0	not significant
10323	BONCI_TARGETS_OF_MIR15A_AND_MIR16_1	0.91	0.20	0	0	0	not significant
10324	GO_MEIOTIC_CELL_CYCLE_PROCESS	0.91	0.20	0	0	0	not significant
10325	PANGAS_TUMOR_SUPPRESSION_BY_SMAD1_AND_SMAD5_UP	0.91	0.20	0	0	0	not significant
10326	PID_FCER1_PATHWAY	0.91	0.20	0	0	0	not significant
10327	TONKS_TARGETS_OF_RUNX1_RUNX1T1_FUSION_GRANULOCYTE_UP	0.91	0.20	0	0	0	not significant
10328	GO_CELL_CYCLE_DNA_REPLICATION	0.91	0.19	0	0	0	not significant
10329	GO_CYTOPLASMIC_SIDE_OF_MEMBRANE	0.91	0.19	0	0	0	not significant
10330	GO_RNA_POLYMERASE_II_DISTAL_ENHANCER_SEQUENCE_SPECIFIC_DN	0.91	0.19	0	0	0	not significant
10331	SLEBOS_HEAD_AND_NECK_CANCER_WITH_HPV_UP	0.91	0.19	0	0	0	not significant
10332	GO_ACTIVE_TRANSMEMBRANE_TRANSPORTER_ACTIVITY	0.91	0.17	0	0	0	not significant
10333	GO_GERM_CELL_DEVELOPMENT	0.91	0.17	0	0	0	not significant
10334	KARLSSON_TGFB1_TARGETS_DN	0.91	0.17	0	0	0	not significant
10335	FULCHER_INFLAMMATORY_RESPONSE_LECTIN_VS_LPS_DN	0.91	0.15	0	0	0	not significant
10336	GO_EMBRYONIC_MORPHOGENESIS	0.91	0.15	0	0	0	not significant
10337	KUMAR_TARGETS_OF_MLL_AF9_FUSION	0.91	0.15	0	0	0	not significant
10338	RODRIGUES_THYROID_CARCINOMA_ANAPLASTIC_DN	0.91	0.14	0	0	0	not significant
10339	GO_VACUOLE	0.91	0.13	0	0	0	not significant
10340	GO_POSITIVE_REGULATION_OF_CELL_DEATH	0.91	0.12	0	0	0	not significant
10341	GO_SYNAPSE_PART	0.91	0.11	0	0	0	not significant
10342	GO_PROTEIN_LOCALIZATION_TO_ORGANELLE	0.91	0.10	0	0	0	not significant
10343	GO_REGULATION_OF_STORE_OPERATED_CALCIUM_CHANNEL_ACTIVITY	0.92	0.27	0	0	0	not significant

10344	VANDESLUIS_COMMD1_TARGETS_GROUP_3_DN	0.92	0.27	0	0	0	not significant
10345	CHR7P13	0.92	0.26	0	0	0	not significant
10346	GO_ANION_ANION_ANTIPORTER_ACTIVITY	0.92	0.26	0	0	0	not significant
10347	GO_NEGATIVE_REGULATION_OF_ORGAN_GROWTH	0.92	0.26	0	0	0	not significant
10348	GO_NEGATIVE_REGULATION_OF_STRESS_ACTIVATED_PROTEIN_KINASE	0.92	0.26	0	0	0	not significant
10349	GO_SINGLE_STRAND_BREAK_REPAIR	0.92	0.26	0	0	0	not significant
10350	TSENG_ADIPOGENIC_POTENTIAL_DN	0.92	0.26	0	0	0	not significant
10351	ABDULRAHMAN_KIDNEY_CANCER_VHL_UP	0.92	0.25	0	0	0	not significant
10352	GO_FOREBRAIN_CELL_MIGRATION	0.92	0.25	0	0	0	not significant
10353	GO_INTRINSIC_APOPTOTIC_SIGNALING_PATHWAY_IN_RESPONSE_TO_DI	0.92	0.25	0	0	0	not significant
10354	GO_PHOSPHATIDYLINOSITOL_5_PHOSPHATE_BINDING	0.92	0.25	0	0	0	not significant
10355	GO_POSITIVE_REGULATION_OF_SIGNAL_TRANSDUCTION_BY_P53_CLAS	0.92	0.25	0	0	0	not significant
10356	GO_PROSTANOID_METABOLIC_PROCESS	0.92	0.25	0	0	0	not significant
10357	GO_REGULATION_OF_SMOOTH_MUSCLE_CELL_DIFFERENTIATION	0.92	0.25	0	0	0	not significant
10358	GO_REGULATION_OF_STEM_CELL_PROLIFERATION	0.92	0.25	0	0	0	not significant
10359	GO_REPLICATION_FORK_PROTECTION	0.92	0.25	0	0	0	not significant
10360	GO_SYNAPTONEMAL_COMPLEX_ORGANIZATION	0.92	0.25	0	0	0	not significant
10361	HERNANDEZ_MITOTIC_ARREST_BY_DOCETAXEL_2_UP	0.92	0.25	0	0	0	not significant
10362	HOLLERN_PAPILLARY_BREAST_TUMOR	0.92	0.25	0	0	0	not significant
10363	MARIADASON_RESPONSE_TO_CURCUMIN_SULINDAC_7	0.92	0.25	0	0	0	not significant
10364	PLASARI_TGFB1_SIGNALING_VIA_NFIC_10HR_UP	0.92	0.25	0	0	0	not significant
10365	REACTOME_PTK6_REGULATES_RTKS_AND_THEIR_EFFECTORS_AKT1_A	0.92	0.25	0	0	0	not significant
10366	REACTOME_REGULATION_OF_KIT_SIGNALING	0.92	0.25	0	0	0	not significant
10367	REACTOME_TP53_REGULATES_TRANSCRIPTION_OF_GENES_INVOLVED_	0.92	0.25	0	0	0	not significant
10368	VERRECCHIA_RESPONSE_TO_TGFB1_C1	0.92	0.25	0	0	0	not significant
10369	VILIMAS_NOTCH1_TARGETS_UP	0.92	0.25	0	0	0	not significant
10370	GARGALOVIC_RESPONSE_TO_OXIDIZED_PHOSPHOLIPIDS_MAGENTA_UP	0.92	0.24	0	0	0	not significant
10371	GO_CELL_SUBSTRATE_JUNCTION_ASSEMBLY	0.92	0.24	0	0	0	not significant
10372	GO_FIBRONECTIN_BINDING	0.92	0.24	0	0	0	not significant
10373	GO_MICROVILLUS_ASSEMBLY	0.92	0.24	0	0	0	not significant
10374	GO_NOTCH_RECEPTOR_PROCESSING	0.92	0.24	0	0	0	not significant
10375	GO_PROTEIN_KINASE_A_SIGNALING	0.92	0.24	0	0	0	not significant
10376	GO_REGULATION_OF_FEMALE_GONAD_DEVELOPMENT	0.92	0.24	0	0	0	not significant
10377	GO_REGULATION_OF_TRANSCRIPTION_INVOLVED_IN_CELL_FATE_COMI	0.92	0.24	0	0	0	not significant
10378	PID_INTEGRIN_CS_PATHWAY	0.92	0.24	0	0	0	not significant
10379	REACTOME_CHAPERONE_MEDIATED_AUTOPHAGY	0.92	0.24	0	0	0	not significant
10380	REACTOME_RUNX2_REGULATES_OSTEOBLAST_DIFFERENTIATION	0.92	0.24	0	0	0	not significant
10381	CHR7P22	0.92	0.23	0	0	0	not significant
10382	GO_ANGIOTENSIN_RECEPTOR_BINDING	0.92	0.23	0	0	0	not significant
10383	GO_BASEMENT_MEMBRANE_ORGANIZATION	0.92	0.23	0	0	0	not significant
10384	GO_BLEB	0.92	0.23	0	0	0	not significant
10385	GO_CELLULAR_RESPONSE_TO_X_RAY	0.92	0.23	0	0	0	not significant
10386	GO_NEGATIVE_REGULATION_OF_HIPPO_SIGNALING	0.92	0.23	0	0	0	not significant
10387	GO_POSITIVE_REGULATION_OF_AXONOGENESIS	0.92	0.23	0	0	0	not significant
10388	GO_PROTEIN_LOCALIZATION_TO_VACUOLE	0.92	0.23	0	0	0	not significant
10389	LUI_THYROID_CANCER_CLUSTER_4	0.92	0.23	0	0	0	not significant
10390	PID_P38_ALPHA_BETA_DOWNSTREAM_PATHWAY	0.92	0.23	0	0	0	not significant
10391	REACTOME_BICARBONATE_TRANSPORTERS	0.92	0.23	0	0	0	not significant
10392	GO_INTRINSIC_COMPONENT_OF_GOLGI_MEMBRANE	0.92	0.22	0	0	0	not significant
10393	GO_OMEGA_PEPTIDASE_ACTIVITY	0.92	0.22	0	0	0	not significant
10394	GO_POSITIVE_REGULATION_OF_MORPHOGENESIS_OF_AN_EPITHELIUM	0.92	0.22	0	0	0	not significant
10395	GO_REGULATION_OF_EXOCYTOSIS	0.92	0.22	0	0	0	not significant
10396	GO_RESPONSE_TO_FIBROBLAST_GROWTH_FACTOR	0.92	0.22	0	0	0	not significant
10397	PID_ARF6_TRAFFICKING_PATHWAY	0.92	0.22	0	0	0	not significant
10398	YU_MYC_TARGETS_DN	0.92	0.22	0	0	0	not significant
10399	ZHAN_MULTIPLE_MYELOMA_LB_DN	0.92	0.22	0	0	0	not significant
10400	GO_DENDRITE_MORPHOGENESIS	0.92	0.21	0	0	0	not significant
10401	GO_DIOXYGENASE_ACTIVITY	0.92	0.21	0	0	0	not significant
10402	GO_REGULATION_OF_RECEPTOR_MEDIATED_ENDOCYTOSIS	0.92	0.21	0	0	0	not significant
10403	REACTOME_GLUTAMATE_BINDING_ACTIVATION_OF_AMPA_RECEPTORS_	0.92	0.21	0	0	0	not significant
10404	STEARMAN_LUNG_CANCER_EARLY_VS_LATE_UP	0.92	0.21	0	0	0	not significant
10405	GO_APICAL_PLASMA_MEMBRANE	0.92	0.20	0	0	0	not significant
10406	GO_CELL_JUNCTION_ORGANIZATION	0.92	0.20	0	0	0	not significant
10407	GO_POSITIVE_REGULATION_OF_MITOCHONDRION_ORGANIZATION	0.92	0.20	0	0	0	not significant
10408	REACTOME_DDX58_IFIH1_MEDIATED_INDUCTION_OF_INTERFERON_ALPI	0.92	0.20	0	0	0	not significant
10409	WAMUNYOKOLI_OVARIAN_CANCER_LMP_DN	0.92	0.20	0	0	0	not significant
10410	ZHANG_BREAST_CANCER_PROGENITORS_DN	0.92	0.20	0	0	0	not significant
10411	GO_REGULATION_OF_ACTIN_FILAMENT_ORGANIZATION	0.92	0.19	0	0	0	not significant
10412	KEGG_PATHWAYS_IN_CANCER	0.92	0.18	0	0	0	not significant
10413	GO_NEGATIVE_REGULATION_OF_CELL_DIFFERENTIATION	0.92	0.17	0	0	0	not significant
10414	GO_RAS_PROTEIN_SIGNAL_TRANSDUCTION	0.92	0.17	0	0	0	not significant
10415	GO_REGULATION_OF_CELL_MORPHOGENESIS_INVOLVED_IN_DIFFEREN	0.92	0.17	0	0	0	not significant
10416	TORCHIA_TARGETS_OF_EWSR1_FLI1_FUSION_DN	0.92	0.17	0	0	0	not significant
10417	GO_CELL_SURFACE_RECEPTOR_SIGNALING_PATHWAY_INVOLVED_IN_C	0.92	0.16	0	0	0	not significant
10418	ZWANG_CLASS_1_TRANSIENTLY_INDUCED_BY_EGF	0.92	0.16	0	0	0	not significant
10419	GO_REGULATION_OF_SECRETION	0.92	0.14	0	0	0	not significant
10420	GO_CELL_CELL_SIGNALING	0.92	0.12	0	0	0	not significant
10421	GO_REPRODUCTION	0.92	0.11	0	0	0	not significant
10422	GO_REGULATION_OF_CELL_DIFFERENTIATION	0.92	0.10	0	0	0	not significant
10423	FERRANDO_HOX11_NEIGHBORS	0.93	0.27	0	0	0	not significant
10424	GO_AMYLOID_PRECURSOR_PROTEIN_BIOSYNTHETIC_PROCESS	0.93	0.27	0	0	0	not significant
10425	GO_CHOLESTEROL_TRANSPORTER_ACTIVITY	0.93	0.27	0	0	0	not significant
10426	GO_POSITIVE_REGULATION_OF_CHEMOTAXIS	0.93	0.27	0	0	0	not significant
10427	GO_SPINAL_CORD_PATTERNING	0.93	0.27	0	0	0	not significant
10428	KHETCHOUMIAN_TRIM24_TARGETS_UP	0.93	0.27	0	0	0	not significant
10429	MULLIGAN_NTF3_SIGNALING_VIA_INSR_AND_IGF1R_DN	0.93	0.27	0	0	0	not significant
10430	WHITE_NEUROBLASTOMA_WITH_1P36.3_DELETION	0.93	0.27	0	0	0	not significant
10431	BAELDE_DIABETIC_NEPHROPATHY_UP	0.93	0.26	0	0	0	not significant
10432	GO_CORPUS_CALLOSUM_DEVELOPMENT	0.93	0.26	0	0	0	not significant
10433	GO_HAUS_COMPLEX	0.93	0.26	0	0	0	not significant
10434	GO_PHOSPHODIESTERASE_I_ACTIVITY	0.93	0.26	0	0	0	not significant
10435	GO_POSITIVE_REGULATION_OF_PRI_MIRNA_TRANSCRIPTION_BY_RNA_F	0.93	0.26	0	0	0	not significant
10436	GO_PROTEIN_KINASE_C_ACTIVATING_G_PROTEIN_COUPLED_RECEPTO	0.93	0.26	0	0	0	not significant
10437	GO_PYRIDINE_CONTAINING_COMPOUND_BIOSYNTHETIC_PROCESS	0.93	0.26	0	0	0	not significant
10438	GO_SALIVARY_GLAND_DEVELOPMENT	0.93	0.26	0	0	0	not significant
10439	PID_ATF2_PATHWAY	0.93	0.26	0	0	0	not significant
10440	REACTOME_NEGATIVE_REGULATION_OF_MET_ACTIVITY	0.93	0.26	0	0	0	not significant
10441	RODRIGUES_THYROID_CARCINOMA_UP	0.93	0.26	0	0	0	not significant
10442	DAIRKEE_TERT_TARGETS_DN	0.93	0.25	0	0	0	not significant
10443	GO_ACUTE_INFLAMMATORY_RESPONSE	0.93	0.25	0	0	0	not significant
10444	GO_CYTOKINE_PRODUCTION_INVOLVED_IN_INFLAMMATORY_RESPONSE	0.93	0.25	0	0	0	not significant
10445	GO_FLAP_ENDONUCLEASE_ACTIVITY	0.93	0.25	0	0	0	not significant
10446	GO_HEPARAN_SULFATE_PROTEOGLYCAN_BIOSYNTHETIC_PROCESS_PO	0.93	0.25	0	0	0	not significant
10447	GO_HOMOCYSTEINE_METABOLIC_PROCESS	0.93	0.25	0	0	0	not significant
10448	GO_NEGATIVE_REGULATION_OF_PERK_MEDIATED_UNFOLDED_PROTEI	0.93	0.25	0	0	0	not significant
10449	GO_NEUROMUSCULAR_JUNCTION_DEVELOPMENT	0.93	0.25	0	0	0	not significant
10450	GO_PEPTIDE_HORMONE_BINDING	0.93	0.25	0	0	0	not significant
10451	GO_POSITIVE_REGULATION_OF_LIGASE_ACTIVITY	0.93	0.25	0	0	0	not significant
10452	GO_POSITIVE_REGULATION_OF_SPHINGOLIPID_BIOSYNTHETIC_PROCES	0.93	0.25	0	0	0	not significant

10453	GO_RIG_I_SIGNALING_PATHWAY	0.93	0.25	0	0	0	not significant
10454	GO_TUBULIN_DEACETYLATION	0.93	0.25	0	0	0	not significant
10455	KORKOLA_YOLK_SAC_TUMOR	0.93	0.25	0	0	0	not significant
10456	ONO_AML1_TARGETS_UP	0.93	0.25	0	0	0	not significant
10457	STAMBOLSKY_RESPONSE_TO_VITAMIN_D3_UP	0.93	0.25	0	0	0	not significant
10458	YAO_TEMPORAL_RESPONSE_TO_PROGESTERONE_CLUSTER_2	0.93	0.25	0	0	0	not significant
10459	BIOCARTA_CELLCYCLE_PATHWAY	0.93	0.24	0	0	0	not significant
10460	GO_MOLTING_CYCLE	0.93	0.24	0	0	0	not significant
10461	GO_POSITIVE_REGULATION_OF_DENDRITE_DEVELOPMENT	0.93	0.24	0	0	0	not significant
10462	GO_RNA_POLYMERASE_II_TRANSCRIPTION_FACTOR_BINDING	0.93	0.24	0	0	0	not significant
10463	GO_VASCULAR_ENDOTHELIAL_GROWTH_FACTOR_SIGNALING_PATHWA	0.93	0.24	0	0	0	not significant
10464	CHR10Q24	0.93	0.23	0	0	0	not significant
10465	GO_MIDBRAIN_DOPAMINERGIC_NEURON_DIFFERENTIATION	0.93	0.23	0	0	0	not significant
10466	GO_NEGATIVE_REGULATION_OF_CELL_ACTIVATION	0.93	0.23	0	0	0	not significant
10467	GO_ORGANOPHOSPHATE_ESTER_TRANSPORT	0.93	0.23	0	0	0	not significant
10468	GO_PROTEIN_LOCALIZATION_TO_SYNAPSE	0.93	0.23	0	0	0	not significant
10469	GO_REGULATION_OF_ANION_TRANSPORT	0.93	0.23	0	0	0	not significant
10470	GO FIBROBLAST GROWTH FACTOR RECEPTOR SIGNALING PATHWA	0.93	0.22	0	0	0	not significant
10471	GO_GLIOGENESIS	0.93	0.22	0	0	0	not significant
10472	GO_MODIFICATION_DEPENDENT_PROTEIN_BINDING	0.93	0.22	0	0	0	not significant
10473	GO_POSITIVE_REGULATION_OF_T_CELL_MEDIATED_IMMUNITY	0.93	0.22	0	0	0	not significant
10474	HALLMARK_APOPTOSIS	0.93	0.22	0	0	0	not significant
10475	JNK_DN.V1_DN	0.93	0.22	0	0	0	not significant
10476	KEGG_MELANOGENESIS	0.93	0.22	0	0	0	not significant
10477	KUMAR_AUTOPHAGY_NETWORK	0.93	0.22	0	0	0	not significant
10478	LTE2_UP.V1_UP	0.93	0.22	0	0	0	not significant
10479	DOANE_RESPONSE_TO_ANDROGEN_DN	0.93	0.21	0	0	0	not significant
10480	GO_AXON_DEVELOPMENT	0.93	0.19	0	0	0	not significant
10481	GINESTIER_BREAST_CANCER_ZNF217_AMPLIFIED_DN	0.93	0.18	0	0	0	not significant
10482	GO_METAL_ION_TRANSMEMBRANE_TRANSPORTER_ACTIVITY	0.93	0.18	0	0	0	not significant
10483	GO REGULATION OF DNA BINDING TRANSCRIPTION FACTOR ACTIVIT	0.93	0.18	0	0	0	not significant
10484	CHICAS_RB1_TARGETS_SENESCENT	0.93	0.17	0	0	0	not significant
10485	GO_REGULATION_OF_PROTEIN_SERINE_THREONINE_KINASE_ACTIVITY	0.93	0.17	0	0	0	not significant
10486	GO_TRANSMEMBRANE_RECEPTOR_PROTEIN_TYROSINE_KINASE_SIGNA	0.93	0.17	0	0	0	not significant
10487	GO_GOLGI_APPARATUS	0.93	0.13	0	0	0	not significant
10488	GO_NEUROGENESIS	0.93	0.13	0	0	0	not significant
10489	GO_NEURON_DIFFERENTIATION	0.93	0.12	0	0	0	not significant
10490	GO_GAMMA_AMINOBUTYRIC_ACID_SIGNALING_PATHWAY	0.94	0.29	0	0	0	not significant
10491	LI_PROSTATE_CANCER_EPIGENETIC	0.94	0.29	0	0	0	not significant
10492	ABE_VEGFA_TARGETS_30MIN	0.94	0.28	0	0	0	not significant
10493	GO_GAMMA_TUBULIN_COMPLEX	0.94	0.28	0	0	0	not significant
10494	GO_GLUCOCORTICOID_RECEPTOR_BINDING	0.94	0.28	0	0	0	not significant
10495	GO_MESONEPHRIC_TUBULE_MORPHOGENESIS	0.94	0.28	0	0	0	not significant
10496	GO_PROTEIN_LOCALIZATION_TO_POSTSYNAPTIC_MEMBRANE	0.94	0.28	0	0	0	not significant
10497	BENNETT_SYSTEMIC_LUPUS_ERYTHEMATOSUS	0.94	0.27	0	0	0	not significant
10498	BOSCO_INTERFERON_INDUCED_ANTIVIRAL_MODULE	0.94	0.27	0	0	0	not significant
10499	GO_ADRENERGIC_RECEPTOR_SIGNALING_PATHWAY	0.94	0.27	0	0	0	not significant
10500	GO_BICARBONATE_TRANSPORT	0.94	0.27	0	0	0	not significant
10501	GO ESTABLISHMENT OF PROTEIN LOCALIZATION TO PLASMA MEMBR	0.94	0.27	0	0	0	not significant
10502	GO_GLYCOLIPID_CATABOLIC_PROCESS	0.94	0.27	0	0	0	not significant
10503	GO_NEGATIVE_REGULATION_OF_INTERLEUKIN_1_PRODUCTION	0.94	0.27	0	0	0	not significant
10504	GO_NEGATIVE_REGULATION_OF_SIGNALING_RECEPTOR_ACTIVITY	0.94	0.27	0	0	0	not significant
10505	GO_REGULATION_OF_CHROMATIN_SILENCING	0.94	0.27	0	0	0	not significant
10506	REACTOME RESOLUTION OF D LOOP STRUCTURES THROUGH SYNT	0.94	0.27	0	0	0	not significant
10507	ATM_DN.V1_UP	0.94	0.26	0	0	0	not significant
10508	FUJII_YBX1_TARGETS_UP	0.94	0.26	0	0	0	not significant
10509	GARGALOVIC_RESPONSE_TO_OXIDIZED_PHOSPHOLIPIDS_YELLOW_DN	0.94	0.26	0	0	0	not significant
10510	GO CELL SURFACE RECEPTOR SIGNALING PATHWAY INVOLVED IN H	0.94	0.26	0	0	0	not significant
10511	GO_CHEMOSENSORY_BEHAVIOR	0.94	0.26	0	0	0	not significant
10512	GO_GROWTH_FACTOR_RECEPTOR_BINDING	0.94	0.26	0	0	0	not significant
10513	GO_HIGH_DENSITY_LIPOPROTEIN_PARTICLE	0.94	0.26	0	0	0	not significant
10514	GO_INTRACILIARY_TRANSPORT_PARTICLE_A	0.94	0.26	0	0	0	not significant
10515	GO_NEGATIVE_REGULATION_OF_FIBROBLAST_GROWTH_FACTOR_REC	0.94	0.26	0	0	0	not significant
10516	GO_NEGATIVE_REGULATION_OF_MEMBRANE_PERMEABILITY	0.94	0.26	0	0	0	not significant
10517	GO POSITIVE REGULATION OF CELLULAR AMIDE METABOLIC PROCE	0.94	0.26	0	0	0	not significant
10518	GO_POSITIVE_REGULATION_OF_FIBROBLAST_MIGRATION	0.94	0.26	0	0	0	not significant
10519	GO_POSITIVE_REGULATION_OF_LIPID_CATABOLIC_PROCESS	0.94	0.26	0	0	0	not significant
10520	GO_POSITIVE_REGULATION_OF_MITOPHAGY	0.94	0.26	0	0	0	not significant
10521	GO_PROTEIN_RETENTION_IN_ER_LUMEN	0.94	0.26	0	0	0	not significant
10522	GO_REGULATION_OF_HAIR_FOLLICLE_DEVELOPMENT	0.94	0.26	0	0	0	not significant
10523	GO REGULATION OF PEROXISOME PROLIFERATOR ACTIVATED RECE	0.94	0.26	0	0	0	not significant
10524	GO_TRANSLATION_INITIATION_FACTOR_BINDING	0.94	0.26	0	0	0	not significant
10525	GO_UBIQUITIN_DEPENDENT_PROTEIN_CATABOLIC_PROCESS_VIA_THE_I	0.94	0.26	0	0	0	not significant
10526	INGRAM_SHH_TARGETS_UP	0.94	0.26	0	0	0	not significant
10527	MATTIOLI_MULTIPLE_MYELOMA_WITH_14Q32_TRANSLOCATIONS	0.94	0.26	0	0	0	not significant
10528	PID_PS1_PATHWAY	0.94	0.26	0	0	0	not significant
10529	REACTOME_FERTILIZATION	0.94	0.26	0	0	0	not significant
10530	GO_CEREBELLAR_CORTEX_FORMATION	0.94	0.25	0	0	0	not significant
10531	GO_COCHLEA_DEVELOPMENT	0.94	0.25	0	0	0	not significant
10532	GO_NEGATIVE_CHEMOTAXIS	0.94	0.25	0	0	0	not significant
10533	GO_POSITIVE_REGULATION_OF_LYMPHOCYTE_DIFFERENTIATION	0.94	0.25	0	0	0	not significant
10534	GO_REGULATION_OF_CYTOKINE_PRODUCTION_INVOLVED_IN_IMMUNE_I	0.94	0.25	0	0	0	not significant
10535	GO_RETINOL_METABOLIC_PROCESS	0.94	0.25	0	0	0	not significant
10536	GO_SPHINGOSINE_N_ACYLTRANSFERASE_ACTIVITY	0.94	0.25	0	0	0	not significant
10537	IZADPANAH_STEM_CELL_ADIPOSE_VS_BONE_UP	0.94	0.25	0	0	0	not significant
10538	LENAOUR_DENDRITIC_CELL_MATURATION_UP	0.94	0.25	0	0	0	not significant
10539	MIKKELSEN_MEF_HCP_WITH_H3_UNMETHYLATED	0.94	0.25	0	0	0	not significant
10540	REACTOME_NRIF_SIGNALS_CELL_DEATH_FROM_THE_NUCLEUS	0.94	0.25	0	0	0	not significant
10541	TIEN_INTESTINE_PROBIOTICS_2HR_UP	0.94	0.25	0	0	0	not significant
10542	GO_FIBROBLAST_GROWTH_FACTOR_PRODUCTION	0.94	0.24	0	0	0	not significant
10543	GO_REGULATION_OF_B_CELL_ACTIVATION	0.94	0.24	0	0	0	not significant
10544	GO_RIBOSOME	0.94	0.24	0	0	0	not significant
10545	GOTTWEIN_TARGETS_OF_KSHV_MIR_K12_11	0.94	0.24	0	0	0	not significant
10546	PID_P53_DOWNSTREAM_PATHWAY	0.94	0.24	0	0	0	not significant
10547	GO_CELLULAR_SENESCENCE	0.94	0.23	0	0	0	not significant
10548	JUBAN_TARGETS_OF_SPI1_AND_FLI1_UP	0.94	0.23	0	0	0	not significant
10549	KYNG_RESPONSE_TO_H2O2	0.94	0.23	0	0	0	not significant
10550	SCHAEFFER_PROSTATE_DEVELOPMENT_48HR_DN	0.94	0.23	0	0	0	not significant
10551	BROWNE_HCMV_INFECTION_6HR_DN	0.94	0.22	0	0	0	not significant
10552	GO_ANION_TRANSMEMBRANE_TRANSPORTER_ACTIVITY	0.94	0.22	0	0	0	not significant
10553	GO_POSITIVE_REGULATION_OF_RESPONSE_TO_EXTERNAL_STIMULUS	0.94	0.22	0	0	0	not significant
10554	GO_REGULATION_OF_NUCLEOTIDE_BINDING_OLIGOMERIZATION_DOMAI	0.94	0.22	0	0	0	not significant
10555	SATO_SILENCED_BY_METHYLATION_IN_PANCREATIC_CANCER_1	0.94	0.22	0	0	0	not significant
10556	GO_PROTEIN_KINASE_ACTIVITY	0.94	0.19	0	0	0	not significant
10557	CREIGHTON_ENDOCRINE_THERAPY_RESISTANCE_5	0.94	0.18	0	0	0	not significant
10558	GO_REGULATION_OF_ESTABLISHMENT_OF_PROTEIN_LOCALIZATION	0.94	0.18	0	0	0	not significant
10559	GO_GOLGI_MEMBRANE	0.94	0.17	0	0	0	not significant
10560	GO_REGULATION_OF_INTRACELLULAR_SIGNAL_TRANSDUCTION	0.94	0.13	0	0	0	not significant
10561	GO_SPECIFIC_GRANULE_LUMEN	0.95	0.30	0	0	0	not significant

10562	BIOCARTA_ETS_PATHWAY	0.95	0.29	0	0	0	not significant
10563	BIOCARTA_NTHI_PATHWAY	0.95	0.29	0	0	0	not significant
10564	CHR10Q25	0.95	0.29	0	0	0	not significant
10565	DANG_MYC_TARGETS_DN	0.95	0.29	0	0	0	not significant
10566	GO_PROTEIN_COMPLEX_INVOLVED_IN_CELL_ADHESION	0.95	0.29	0	0	0	not significant
10567	GO_PROTEIN_KINASE_C_SIGNALING	0.95	0.29	0	0	0	not significant
10568	PID_TCR_JNK_PATHWAY	0.95	0.29	0	0	0	not significant
10569	CHR7Q11	0.95	0.28	0	0	0	not significant
10570	CHRXQ13	0.95	0.28	0	0	0	not significant
10571	GO_CARDIAC_MUSCLE_CELL_ACTION_POTENTIAL_INVOLVED_IN_CONTR	0.95	0.28	0	0	0	not significant
10572	GO_EUKARYOTIC_48S_PREINITIATION_COMPLEX	0.95	0.28	0	0	0	not significant
10573	GO_H4_HISTONE_ACETYLTRANSFERASE_ACTIVITY	0.95	0.28	0	0	0	not significant
10574	GO_LIGAND_GATED_CATION_CHANNEL_ACTIVITY	0.95	0.28	0	0	0	not significant
10575	GO_POSITIVE_REGULATION_OF_PEROXISOME_PROLIFERATOR_ACTIVAT	0.95	0.28	0	0	0	not significant
10576	GO_REGULATION_OF_SYNAPTIC_TRANSMISSION_GLUTAMATERGIC	0.95	0.28	0	0	0	not significant
10577	GO_SPHINGOLIPID_BIOSYNTHETIC_PROCESS	0.95	0.28	0	0	0	not significant
10578	HUANG_FOXA2_TARGETS_UP	0.95	0.28	0	0	0	not significant
10579	KANNAN_TP53_TARGETS_DN	0.95	0.28	0	0	0	not significant
10580	LA_MEN1_TARGETS	0.95	0.28	0	0	0	not significant
10581	LAU_APOPTOSIS_CDKN2A_UP	0.95	0.28	0	0	0	not significant
10582	MCCABE_HOXC6_TARGETS_DN	0.95	0.28	0	0	0	not significant
10583	BROWNE_HCMV_INFECTION_2HR_UP	0.95	0.27	0	0	0	not significant
10584	GO_COLLATERAL_SPROUTING	0.95	0.27	0	0	0	not significant
10585	GO_INTERLEUKIN_8_BIOSYNTHETIC_PROCESS	0.95	0.27	0	0	0	not significant
10586	GO_NEGATIVE_REGULATION_OF_SUPRAMOLECULAR_FIBER_ORGANIZAT	0.95	0.27	0	0	0	not significant
10587	GO_POSITIVE_REGULATION_OF_GLUCONEOGENESIS	0.95	0.27	0	0	0	not significant
10588	GO_POSITIVE_REGULATION_OF_MITOTIC_NUCLEAR_DIVISION	0.95	0.27	0	0	0	not significant
10589	GO_REGULATION_OF_HEPATOCYTE_PROLIFERATION	0.95	0.27	0	0	0	not significant
10590	GO_URIDYLYLTRANSFERASE_ACTIVITY	0.95	0.27	0	0	0	not significant
10591	GO_VOLTAGE_GATED_CALCIUM_CHANNEL_ACTIVITY	0.95	0.27	0	0	0	not significant
10592	JEON_SMAD6_TARGETS_UP	0.95	0.27	0	0	0	not significant
10593	KOKKINAKIS_METHIONINE_DEPRIVATION_48HR_DN	0.95	0.27	0	0	0	not significant
10594	PID_TCR_CALCIUM_PATHWAY	0.95	0.27	0	0	0	not significant
10595	TCGA_GLIOBLASTOMA_COPY_NUMBER_DN	0.95	0.27	0	0	0	not significant
10596	WALLACE_PROSTATE_CANCER_RACE_DN	0.95	0.27	0	0	0	not significant
10597	ZWANG_CLASS_2_TRANSIENTLY_INDUCED_BY_EGF	0.95	0.27	0	0	0	not significant
10598	CHR11Q22	0.95	0.26	0	0	0	not significant
10599	CHR3Q11	0.95	0.26	0	0	0	not significant
10600	GO_DEOXYRIBONUCLEOSIDE_MONOPHOSPHATE_BIOSYNTHETIC_PROCE	0.95	0.26	0	0	0	not significant
10601	GO_POSITIVE_REGULATION_OF_INTERLEUKIN_5_PRODUCTION	0.95	0.26	0	0	0	not significant
10602	GO_POSITIVE_REGULATION_OF_SYNAPTIC_TRANSMISSION_GLUTAMATE	0.95	0.26	0	0	0	not significant
10603	GO_PRESYNAPTIC_ENDOCYTOSIS	0.95	0.26	0	0	0	not significant
10604	GO_PROTEIN_PHOSPHATASE_BINDING	0.95	0.26	0	0	0	not significant
10605	GO_REGULATION_OF_ALPHA_BETA_T_CELL_DIFFERENTIATION	0.95	0.26	0	0	0	not significant
10606	GO_RESPONSE_TO_HERBICIDE	0.95	0.26	0	0	0	not significant
10607	GO_SYNAPTIC_VESICLE_RECYCLING	0.95	0.26	0	0	0	not significant
10608	GO_UBIQUITIN_PROTEIN_TRANSFERASE_INHIBITOR_ACTIVITY	0.95	0.26	0	0	0	not significant
10609	GO_VASCULAR_PROCESS_IN_CIRCULATORY_SYSTEM	0.95	0.26	0	0	0	not significant
10610	JACKSON_DNMT1_TARGETS_UP	0.95	0.26	0	0	0	not significant
10611	LIU_NASOPHARYNGEAL_CARCINOMA	0.95	0.26	0	0	0	not significant
10612	REACTOME_ACTIVATION_OF_NOXA_AND_TRANSLOCATION_TO_MITOCH	0.95	0.26	0	0	0	not significant
10613	WANG_IMMORTALIZED_BY_HOXA9_AND_MEIS1_DN	0.95	0.26	0	0	0	not significant
10614	GO_DEVELOPMENTAL_GROWTH_INVOLVED_IN_MORPHOGENESIS	0.95	0.25	0	0	0	not significant
10615	GO_NEPHRIC_DUCT_DEVELOPMENT	0.95	0.25	0	0	0	not significant
10616	GO_PHOSPHATIDYLINOSITOL_PHOSPHATE_BINDING	0.95	0.25	0	0	0	not significant
10617	GO_POSITIVE_REGULATION_OF_DNA_REPLICATION	0.95	0.25	0	0	0	not significant
10618	GO_PYROPTOSIS	0.95	0.25	0	0	0	not significant
10619	GO_SENSORY_ORGAN_MORPHOGENESIS	0.95	0.25	0	0	0	not significant
10620	REACTOME_DISEASES_OF_IMMUNE_SYSTEM	0.95	0.25	0	0	0	not significant
10621	REACTOME_SYNTHESIS_OF_5_EICOSATETRAENOIC_ACIDS	0.95	0.25	0	0	0	not significant
10622	ZHOU_INFLAMMATORY_RESPONSE_LPS_UP	0.95	0.25	0	0	0	not significant
10623	GO_CLATHRIN_LIGHT_CHAIN_BINDING	0.95	0.24	0	0	0	not significant
10624	GO_NEGATIVE_REGULATION_OF_GROWTH	0.95	0.24	0	0	0	not significant
10625	GO_SYNAPSE_ORGANIZATION	0.95	0.24	0	0	0	not significant
10626	HOXA9_DN.V1_UP	0.95	0.24	0	0	0	not significant
10627	GO_TRANSCRIPTION_COREGULATOR_ACTIVITY	0.95	0.23	0	0	0	not significant
10628	TBK1.DF_UP	0.95	0.23	0	0	0	not significant
10629	BONOME_OVARIAN_CANCER_SURVIVAL_SUBOPTIMAL_DEBULKING	0.95	0.21	0	0	0	not significant
10630	GO_CENTRAL_NERVOUS_SYSTEM_DEVELOPMENT	0.95	0.21	0	0	0	not significant
10631	GO_LACTATION	0.96	0.32	0	0	0	not significant
10632	BRACHAT_RESPONSE_TO_CAMPTOTHECIN_UP	0.96	0.31	0	0	0	not significant
10633	FONTAINE_FOLLICULAR_THYROID_ADENOMA_UP	0.96	0.31	0	0	0	not significant
10634	GO_POSITIVE_REGULATION_OF_ESTABLISHMENT_OF_PROTEIN_LOCALI	0.96	0.31	0	0	0	not significant
10635	GO_ASPARTIC_TYPE_PEPTIDASE_ACTIVITY	0.96	0.30	0	0	0	not significant
10636	GO_CALCIUM_ION_IMPORT	0.96	0.30	0	0	0	not significant
10637	GO_EARLY_PHAGOSOME	0.96	0.30	0	0	0	not significant
10638	GO_HEPARIN_BINDING	0.96	0.30	0	0	0	not significant
10639	GO_RESPONSE_TO_RETINOIC_ACID	0.96	0.30	0	0	0	not significant
10640	PID_PRL_SIGNALING_EVENTS_PATHWAY	0.96	0.30	0	0	0	not significant
10641	REACTOME_GLUCURONIDATION	0.96	0.30	0	0	0	not significant
10642	REACTOME_SYNTHESIS_OF_GLYCOSYLPHOSPHATIDYLINOSITOL_GPI	0.96	0.30	0	0	0	not significant
10643	BIOCARTA_TALL1_PATHWAY	0.96	0.29	0	0	0	not significant
10644	CHR2Q37	0.96	0.29	0	0	0	not significant
10645	GO_ACYLGLYCEROL_O_ACYLTRANSFERASE_ACTIVITY	0.96	0.29	0	0	0	not significant
10646	GO_HISTONE_DEACETYLASE_ACTIVITY_H3_K14_SPECIFIC_	0.96	0.29	0	0	0	not significant
10647	GO_NEGATIVE_REGULATION_OF_LEUKOCYTE_MIGRATION	0.96	0.29	0	0	0	not significant
10648	GO_NEGATIVE_REGULATION_OF_PROTEIN_OLIGOMERIZATION	0.96	0.29	0	0	0	not significant
10649	GO_PHOSPHATIDYLINOSITOL_3_PHOSPHATE_BINDING	0.96	0.29	0	0	0	not significant
10650	GO_PHOSPHOLIPASE_BINDING	0.96	0.29	0	0	0	not significant
10651	GO_POSITIVE_REGULATION_OF_ENDOPLASMIC_RETICULUM_STRESS_IN	0.96	0.29	0	0	0	not significant
10652	GO_POSITIVE_REGULATION_OF_EXECUTION_PHASE_OF_APOPTOSIS	0.96	0.29	0	0	0	not significant
10653	GO_POSITIVE_REGULATION_OF_EXOCYTOSIS	0.96	0.29	0	0	0	not significant
10654	GO_POSTSYNAPTIC_DENSITY_INTRACELLULAR_COMPONENT	0.96	0.29	0	0	0	not significant
10655	GO_PRENYLATION	0.96	0.29	0	0	0	not significant
10656	GRADE_COLON_AND_RECTAL_CANCER_DN	0.96	0.29	0	0	0	not significant
10657	HAN_JNK_SINGALING_UP	0.96	0.29	0	0	0	not significant
10658	TARTE_PLASMA_CELL_VS_B_LYMPHOCYTE_DN	0.96	0.29	0	0	0	not significant
10659	CHEMELLO_SOLEUS_VS_EDL_MYOFIBERS_UP	0.96	0.28	0	0	0	not significant
10660	CHR8Q13	0.96	0.28	0	0	0	not significant
10661	GO_CARDIAC_MUSCLE_CELL_PROLIFERATION	0.96	0.28	0	0	0	not significant
10662	GO_EMBRYONIC_AXIS_SPECIFICATION	0.96	0.28	0	0	0	not significant
10663	GO_HISTONE_H3_K9_MODIFICATION	0.96	0.28	0	0	0	not significant
10664	GO_INORGANIC_ANION_EXCHANGER_ACTIVITY	0.96	0.28	0	0	0	not significant
10665	GO_NAD_BIOSYNTHESIS_VIA_NICOTINAMIDE_RIBOSIDE_SALVAGE_PATHW	0.96	0.28	0	0	0	not significant
10666	GO_NEGATIVE_REGULATION_OF_PROTEIN_SUMOYLATION	0.96	0.28	0	0	0	not significant
10667	GO_NEGATIVE_REGULATION_OF_SODIUM_ION_TRANSMEMBRANE_TRAN	0.96	0.28	0	0	0	not significant
10668	GO_POSITIVE_REGULATION_OF_HEMOPOIESIS	0.96	0.28	0	0	0	not significant
10669	GO_PYRIMIDINE_DEOXYRIBONUCLEOSIDE_MONOPHOSPHATE_BIOSYNTH	0.96	0.28	0	0	0	not significant
10670	GO_REGULATION_OF_DNA_REPAIR	0.96	0.28	0	0	0	not significant

10671	GO_REGULATION_OF_INFLAMMATORY_RESPONSE_TO_ANTIGENIC_STIM	0.96	0.28	0	0	0	not significant
10672	GO_SEGMENTATION	0.96	0.28	0	0	0	not significant
10673	GO_SOMATIC_DIVERSIFICATION_OF_IMMUNE_RECEPTORS	0.96	0.28	0	0	0	not significant
10674	GO_SPERM_MOTILITY	0.96	0.28	0	0	0	not significant
10675	GO_STEROL_METABOLIC_PROCESS	0.96	0.28	0	0	0	not significant
10676	GO_WNT_SIGNALING_PATHWAY_INVOLVED_IN_MIDBRAIN_DOPAMINERGIC	0.96	0.28	0	0	0	not significant
10677	KIM_WT1_TARGETS_UP	0.96	0.28	0	0	0	not significant
10678	REACTOME_ACETYLCHOLINE_REGULATES_INSULIN_SECRETION	0.96	0.28	0	0	0	not significant
10679	REACTOME_AFLATOXIN_ACTIVATION_AND_DETOXIFICATION	0.96	0.28	0	0	0	not significant
10680	REACTOME_COLLAGEN_CHAIN_TRIMERIZATION	0.96	0.28	0	0	0	not significant
10681	REACTOME_MUCOPOLYSACCHARIDOSES	0.96	0.28	0	0	0	not significant
10682	REACTOME_N_GLYCAN_TRIMMING_IN_THE_ER_AND_CALNEXIN_CALRETI	0.96	0.28	0	0	0	not significant
10683	WATANABE_ULCERATIVE_COLITIS_WITH_CANCER_UP	0.96	0.28	0	0	0	not significant
10684	CAMP_UP.V1_DN	0.96	0.27	0	0	0	not significant
10685	CHR20Q13	0.96	0.27	0	0	0	not significant
10686	GO_DNA_DAMAGE_RESPONSE_SIGNAL_TRANSDUCTION_BY_P53_CLASS	0.96	0.27	0	0	0	not significant
10687	GO_ENDOCRINE_SYSTEM_DEVELOPMENT	0.96	0.27	0	0	0	not significant
10688	GO_GLUCURONOSYLTRANSFERASE_ACTIVITY	0.96	0.27	0	0	0	not significant
10689	GO_LEUCINE_ZIPPER_DOMAIN_BINDING	0.96	0.27	0	0	0	not significant
10690	GO_NEGATIVE_REGULATION_OF_NEURON_PROJECTION_REGENERATIO	0.96	0.27	0	0	0	not significant
10691	GO_REGULATION_OF_ICOSANOID_SECRETION	0.96	0.27	0	0	0	not significant
10692	GO_REGULATION_OF_JUN_KINASE_ACTIVITY	0.96	0.27	0	0	0	not significant
10693	GO_REGULATION_OF_TOLL_LIKE_RECEPTOR_2_SIGNALING_PATHWAY	0.96	0.27	0	0	0	not significant
10694	GO_SYMPATHETIC_NERVOUS_SYSTEM_DEVELOPMENT	0.96	0.27	0	0	0	not significant
10695	HALLMARK_INTERFERON_ALPHA_RESPONSE	0.96	0.27	0	0	0	not significant
10696	HECKER_IFNB1_TARGETS	0.96	0.27	0	0	0	not significant
10697	KEGG_ARRHYTHMOGENIC_RIGHT_VENTRICULAR_CARDIOMYOPATHY_AF	0.96	0.27	0	0	0	not significant
10698	REACTOME_DEX_H_BOX_HELICASES_ACTIVATE_TYPE_I_IFN_AND_INFLA	0.96	0.27	0	0	0	not significant
10699	REACTOME_EXTRACELLULAR_MATRIX_ORGANIZATION	0.96	0.27	0	0	0	not significant
10700	BIOCARTA_PS1_PATHWAY	0.96	0.26	0	0	0	not significant
10701	GO_NEGATIVE_REGULATION_OF_GLYCOGEN_METABOLIC_PROCESS	0.96	0.26	0	0	0	not significant
10702	SCHEIDEREIT_IKK_INTERACTING_PROTEINS	0.96	0.26	0	0	0	not significant
10703	GO_RECEPTOR_ANTAGONIST_ACTIVITY	0.96	0.25	0	0	0	not significant
10704	JOHNSTONE_PARVB_TARGETS_3_UP	0.96	0.25	0	0	0	not significant
10705	MULLIGHAN_MLL_SIGNATURE_2_DN	0.96	0.25	0	0	0	not significant
10706	GO_CELL_CYCLE_ARREST	0.96	0.23	0	0	0	not significant
10707	ENK_UV_RESPONSE_EPIDERMIS_DN	0.96	0.22	0	0	0	not significant
10708	GO_EMBRYO_DEVELOPMENT_ENDING_IN_BIRTH_OR_EGG_HATCHING	0.96	0.22	0	0	0	not significant
10709	GO_CELL_JUNCTION	0.96	0.20	0	0	0	not significant
10710	GO_LOCOMOTION	0.96	0.20	0	0	0	not significant
10711	GO_MRNA_TRANSCRIPTION_BY_RNA_POLYMERASE_II	0.97	0.33	0	0	0	not significant
10712	BIOCARTA_EGF_PATHWAY	0.97	0.32	0	0	0	not significant
10713	GO_3_5_RNA_HELICASE_ACTIVITY	0.97	0.32	0	0	0	not significant
10714	GO_POSITIVE_REGULATION_OF_B_CELL_MEDIATED_IMMUNITY	0.97	0.32	0	0	0	not significant
10715	RAMALHO_STEMNESS_DN	0.97	0.32	0	0	0	not significant
10716	REACTOME_REGULATION_OF_IFNA_SIGNALING	0.97	0.32	0	0	0	not significant
10717	GO_KERATAN_SULFATE_BIOSYNTHETIC_PROCESS	0.97	0.31	0	0	0	not significant
10718	GO_POSITIVE_REGULATION_OF_DEFENSE_RESPONSE_TO_VIRUS_BY_H	0.97	0.31	0	0	0	not significant
10719	GO_REGULATION_OF_PROTEIN_HOMOOLIGOMERIZATION	0.97	0.31	0	0	0	not significant
10720	KEGG_GLYCEROLIPID_METABOLISM	0.97	0.31	0	0	0	not significant
10721	MARIADASON_RESPONSE_TO_BUTYRATE_SULINDAC_6	0.97	0.31	0	0	0	not significant
10722	PRC2_SUZ12_UP.V1_DN	0.97	0.31	0	0	0	not significant
10723	YOSHIOKA_LIVER_CANCER_EARLY_RECURRENCE_UP	0.97	0.31	0	0	0	not significant
10724	BIOCARTA_WNT_PATHWAY	0.97	0.30	0	0	0	not significant
10725	GO_ASTER	0.97	0.30	0	0	0	not significant
10726	GO_CARDIAC_MUSCLE_CELL_CONTRACTION	0.97	0.30	0	0	0	not significant
10727	GO_GLIAL_CELL_DIFFERENTIATION	0.97	0.30	0	0	0	not significant
10728	GO_GROWTH_CONE_PART	0.97	0.30	0	0	0	not significant
10729	GO_NEGATIVE_REGULATION_OF_CILIUM_ASSEMBLY	0.97	0.30	0	0	0	not significant
10730	GO_NEGATIVE_REGULATION_OF_TISSUE_REMODELING	0.97	0.30	0	0	0	not significant
10731	GO_NEUROEPITHELIAL_CELL_DIFFERENTIATION	0.97	0.30	0	0	0	not significant
10732	GO_PROTEIN_IMPORT_INTO_PEROXISOME_MATRIX	0.97	0.30	0	0	0	not significant
10733	GO_REGULATION_OF_IMMUNOGLOBULIN_PRODUCTION	0.97	0.30	0	0	0	not significant
10734	GO_TRANSLATION_PREINITIATION_COMPLEX	0.97	0.30	0	0	0	not significant
10735	GO_TRIGLYCERIDE_CATABOLIC_PROCESS	0.97	0.30	0	0	0	not significant
10736	KASLER_HDAC7_TARGETS_1_DN	0.97	0.30	0	0	0	not significant
10737	MATZUK_SPERMATOCYTE	0.97	0.30	0	0	0	not significant
10738	NABA_COLLAGENS	0.97	0.30	0	0	0	not significant
10739	PID_LPA4_PATHWAY	0.97	0.30	0	0	0	not significant
10740	CHR22Q11	0.97	0.29	0	0	0	not significant
10741	FAELT_B_CLL_WITH_VH3_21_DN	0.97	0.29	0	0	0	not significant
10742	GO_3_5_EXODEOXYRIBONUCLEASE_ACTIVITY	0.97	0.29	0	0	0	not significant
10743	GO_ACTIN_NUCLEATION	0.97	0.29	0	0	0	not significant
10744	GO_AROMATIC_AMINO_ACID_TRANSPORT	0.97	0.29	0	0	0	not significant
10745	GO_CARBOHYDRATE_DERIVATIVE_CATABOLIC_PROCESS	0.97	0.29	0	0	0	not significant
10746	GO_DERLIN_1_RETROTRANSLOCATION_COMPLEX	0.97	0.29	0	0	0	not significant
10747	GO_EQUATORIAL_MICROTUBULE_ORGANIZING_CENTER	0.97	0.29	0	0	0	not significant
10748	GO_GTPASE_ACTIVATING_PROTEIN_BINDING	0.97	0.29	0	0	0	not significant
10749	GO_HISTONE_H3_K9_ACETYLATION	0.97	0.29	0	0	0	not significant
10750	GO_POSITIVE_REGULATION_OF_LEUKOCYTE_MEDIATED_IMMUNITY	0.97	0.29	0	0	0	not significant
10751	GO_REGULATION_OF_B_CELL_PROLIFERATION	0.97	0.29	0	0	0	not significant
10752	GO_REGULATION_OF_OSTEOCLAST_DIFFERENTIATION	0.97	0.29	0	0	0	not significant
10753	GO_SINGLE_STRANDED_DNA_EXODEOXYRIBONUCLEASE_ACTIVITY	0.97	0.29	0	0	0	not significant
10754	LINDSTEDT_DENDRITIC_CELL_MATURATION_C	0.97	0.29	0	0	0	not significant
10755	MIKKELSEN_IPS_LCP_WITH_H3K4ME3	0.97	0.29	0	0	0	not significant
10756	NRL_DN.V1_UP	0.97	0.29	0	0	0	not significant
10757	REACTOME_HDR_THROUGH_MMEJ_ALT_NHEJ	0.97	0.29	0	0	0	not significant
10758	SHETH_LIVER_CANCER_VS_TXNIP_LOSS_PAM5	0.97	0.29	0	0	0	not significant
10759	FRASOR_RESPONSE_TO_ESTRADIOL_DN	0.97	0.28	0	0	0	not significant
10760	GO_CARDIAC_EPITHELIAL_TO_MESENCHYMAL_TRANSITION	0.97	0.28	0	0	0	not significant
10761	GO_NEGATIVE_REGULATION_OF_AUTOPHAGY_OF_MITOCHONDRION	0.97	0.28	0	0	0	not significant
10762	GO_REGULATION_OF_CELLULAR_CARBOHYDRATE_CATABOLIC_PROCE	0.97	0.28	0	0	0	not significant
10763	HAN_SATB1_TARGETS_DN	0.97	0.28	0	0	0	not significant
10764	IWANAGA_CARCINOGENESIS_BY_KRAS_PTEN_DN	0.97	0.28	0	0	0	not significant
10765	KEGG_TASTE_TRANSDUCTION	0.97	0.28	0	0	0	not significant
10766	REACTOME_GLYCEROPHOSPHOLIPID_BIOSYNTHESIS	0.97	0.28	0	0	0	not significant
10767	SMIRNOV_RESPONSE_TO_IR_2HR_DN	0.97	0.28	0	0	0	not significant
10768	VANTVEER_BREAST_CANCER_BRCA1_DN	0.97	0.28	0	0	0	not significant
10769	GO_BETA_2_MICROGLOBULIN_BINDING	0.97	0.27	0	0	0	not significant
10770	GO_TAXIS	0.97	0.27	0	0	0	not significant
10771	ZHENG_FOXP3_TARGETS_IN_THYMUS_UP	0.97	0.27	0	0	0	not significant
10772	GRESHOCK_CANCER_COPY_NUMBER_UP	0.97	0.26	0	0	0	not significant
10773	GO_CELL_MORPHOGENESIS_INVOLVED_IN_NEURON_DIFFERENTIATION	0.97	0.24	0	0	0	not significant
10774	GO_ION_HOMEOSTASIS	0.97	0.24	0	0	0	not significant
10775	GO_MULTI_ORGANISM_REPRODUCTIVE_PROCESS	0.97	0.24	0	0	0	not significant
10776	HALLMARK_WNT_BETA_CATENIN_SIGNALING	0.98	0.34	0	0	0	not significant
10777	DING_LUNG_CANCER_BY_MUTATION_RATE	0.98	0.33	0	0	0	not significant
10778	GO_LIGAND_GATED_ION_CHANNEL_ACTIVITY	0.98	0.33	0	0	0	not significant
10779	PID_GMCSF_PATHWAY	0.98	0.33	0	0	0	not significant

10780	BIOCARTA_IL4_PATHWAY	0.98	0.32	0	0	0	not significant
10781	GO_FIBROBLAST_APOPTOTIC_PROCESS	0.98	0.32	0	0	0	not significant
10782	GO_INTERLEUKIN_5_PRODUCTION	0.98	0.32	0	0	0	not significant
10783	GO_PHOSPHORIC_DIESTER_HYDROLASE_ACTIVITY	0.98	0.32	0	0	0	not significant
10784	GO_POSITIVE_REGULATION_OF_B_CELL_PROLIFERATION	0.98	0.32	0	0	0	not significant
10785	GO_POSITIVE_REGULATION_OF_DNA_RECOMBINATION	0.98	0.32	0	0	0	not significant
10786	GO_POST_EMBRYONIC_ANIMAL_MORPHOGENESIS	0.98	0.32	0	0	0	not significant
10787	GO_REGULATION_OF_GONAD_DEVELOPMENT	0.98	0.32	0	0	0	not significant
10788	GO_XY_BODY	0.98	0.32	0	0	0	not significant
10789	LEE_LIVER_CANCER_CIPROFIBRATE_UP	0.98	0.32	0	0	0	not significant
10790	BASSO_CD40_SIGNALING_UP	0.98	0.31	0	0	0	not significant
10791	DURAND_STROMA_NS_UP	0.98	0.31	0	0	0	not significant
10792	GO_DETECTION_OF_MECHANICAL_STIMULUS	0.98	0.31	0	0	0	not significant
10793	GO_NEGATIVE_REGULATION_OF_SMOOTH_MUSCLE_CELL_PROLIFERAT	0.98	0.31	0	0	0	not significant
10794	GO_PHOSPHOLIPID_METABOLIC_PROCESS	0.98	0.31	0	0	0	not significant
10795	GO_POSITIVE_REGULATION_OF_DENDRITIC_SPINE_DEVELOPMENT	0.98	0.31	0	0	0	not significant
10796	GO_POSITIVE_REGULATION_OF_NATURAL_KILLER_CELL_MEDIATED_IMM	0.98	0.31	0	0	0	not significant
10797	GO_PRESYNAPTIC_MEMBRANE_ORGANIZATION	0.98	0.31	0	0	0	not significant
10798	GO_PYRIMIDINE_DIMER_REPAIR	0.98	0.31	0	0	0	not significant
10799	GO_SYNCYTIUM_FORMATION	0.98	0.31	0	0	0	not significant
10800	GO_TERPENOID_BIOSYNTHETIC_PROCESS	0.98	0.31	0	0	0	not significant
10801	GO_VENTRICULAR_TRABECULA_MYOCARDIUM_MORPHOGENESIS	0.98	0.31	0	0	0	not significant
10802	KONDO_EZH2_TARGETS	0.98	0.31	0	0	0	not significant
10803	LOPEZ_TRANSLATION_VIA_FN1_SIGNALING	0.98	0.31	0	0	0	not significant
10804	PRC1_BMI_UP.V1_UP	0.98	0.31	0	0	0	not significant
10805	REACTOME_NETRIN_1_SIGNALING	0.98	0.31	0	0	0	not significant
10806	VART_KSHV_INFECTION_ANGIOGENIC_MARKERS_DN	0.98	0.31	0	0	0	not significant
10807	WATTEL_AUTONOMOUS_THYROID_ADENOMA_DN	0.98	0.31	0	0	0	not significant
10808	BIOCARTA_CFTR_PATHWAY	0.98	0.30	0	0	0	not significant
10809	FAELT_B_CLL_WITH_VH_REARRANGEMENTS_UP	0.98	0.30	0	0	0	not significant
10810	GO_ACTIVATION_OF_JUN_KINASE_ACTIVITY	0.98	0.30	0	0	0	not significant
10811	GO_CALCIUM_ACTIVATED_POTASSIUM_CHANNEL_ACTIVITY	0.98	0.30	0	0	0	not significant
10812	GO_CALCIUM_ION_TRANSMEMBRANE_TRANSPORTER_ACTIVITY	0.98	0.30	0	0	0	not significant
10813	GO_DETECTION_OF_STIMULUS	0.98	0.30	0	0	0	not significant
10814	GO_EXECUTION_PHASE_OF_APOPTOSIS	0.98	0.30	0	0	0	not significant
10815	GO_EXTRACELLULAR_MATRIX_STRUCTURAL_CONSTITUENT_CONFERRI	0.98	0.30	0	0	0	not significant
10816	GO_NEGATIVE_REGULATION_OF_POTASSIUM_ION_TRANSPORT	0.98	0.30	0	0	0	not significant
10817	GO_NEGATIVE_REGULATION_OF_PROTEIN_POLYMERIZATION	0.98	0.30	0	0	0	not significant
10818	GO_NEGATIVE_REGULATION_OF_VASCULAR_SMOOTH_MUSCLE_CELL_I	0.98	0.30	0	0	0	not significant
10819	GO_NODAL_SIGNALING_PATHWAY	0.98	0.30	0	0	0	not significant
10820	GO_POSITIVE_REGULATION_OF_NEURON_APOPTOTIC_PROCESS	0.98	0.30	0	0	0	not significant
10821	GO_REGULATION_OF_DNA_DEPENDENT_DNA_REPLICATION_INITIATION	0.98	0.30	0	0	0	not significant
10822	GO_REGULATION_OF_SKELETAL_MUSCLE_CONTRACTION	0.98	0.30	0	0	0	not significant
10823	GO_RETINA_LAYER_FORMATION	0.98	0.30	0	0	0	not significant
10824	GO_SODIUM_BICARBONATE_SYMPORTER_ACTIVITY	0.98	0.30	0	0	0	not significant
10825	GO_VITAMIN_CATABOLIC_PROCESS	0.98	0.30	0	0	0	not significant
10826	HALLMARK_APICAL_SURFACE	0.98	0.30	0	0	0	not significant
10827	LEIN_ASTROCYTE_MARKERS	0.98	0.30	0	0	0	not significant
10828	LINDGREN_BLADDER_CANCER_CLUSTER_2B	0.98	0.30	0	0	0	not significant
10829	MARTIN_VIRAL_GPCR_SIGNALING_DN	0.98	0.30	0	0	0	not significant
10830	PID_REG_GR_PATHWAY	0.98	0.30	0	0	0	not significant
10831	RAPA_EARLY_UP.V1_UP	0.98	0.30	0	0	0	not significant
10832	REACTOME_SYNTHESIS_OF_LEUKOTRIENES_LT_AND_EOXINS_EX	0.98	0.30	0	0	0	not significant
10833	REACTOME_TICAM1_TRAF6_DEPENDENT_INDUCTION_OF_TAK1_COMPL	0.98	0.30	0	0	0	not significant
10834	ZHOU_INFLAMMATORY_RESPONSE_FIMA_DN	0.98	0.30	0	0	0	not significant
10835	GO_CHROMAFFIN_GRANULE_MEMBRANE	0.98	0.29	0	0	0	not significant
10836	GO_CORECEPTOR_ACTIVITY_INVOLVED_IN_WNT_SIGNALING_PATHWAY	0.98	0.29	0	0	0	not significant
10837	GO_G_PROTEIN_COUPLED_RECEPTOR_SIGNALING_PATHWAY	0.98	0.29	0	0	0	not significant
10838	GO_NEGATIVE_REGULATION_OF_MITOPHAGY	0.98	0.29	0	0	0	not significant
10839	KREPPEL_CD99_TARGETS_DN	0.98	0.29	0	0	0	not significant
10840	NAKAMURA_ADIPOGENESIS_LATE_DN	0.98	0.29	0	0	0	not significant
10841	SANA_TNF_SIGNALING_DN	0.98	0.29	0	0	0	not significant
10842	SU_THYMUS	0.98	0.29	0	0	0	not significant
10843	CAFFAREL_RESPONSE_TO_THC_24HR_3_UP	0.98	0.28	0	0	0	not significant
10844	GO_HISTONE_BINDING	0.98	0.28	0	0	0	not significant
10845	GO_IMMUNE_SYSTEM_DEVELOPMENT	0.98	0.28	0	0	0	not significant
10846	GO_LIPID_GLYCOSYLATION	0.98	0.28	0	0	0	not significant
10847	GO_METAL_ION_HOMEOSTASIS	0.98	0.28	0	0	0	not significant
10848	GO_MRNA_SPLICE_SITE_SELECTION	0.98	0.28	0	0	0	not significant
10849	FEVR_CTNNB1_TARGETS_UP	0.98	0.26	0	0	0	not significant
10850	CHR12Q12	0.99	0.35	0	0	0	not significant
10851	GO_POSTSYNAPSE_ORGANIZATION	0.99	0.35	0	0	0	not significant
10852	GO_CELL_MATRIX_ADHESION	0.99	0.34	0	0	0	not significant
10853	GO_NEGATIVE_REGULATION_OF_GLYCOPROTEIN_BIOSYNTHETIC_PROC	0.99	0.34	0	0	0	not significant
10854	GO_PHOSPHOLIPID_TRANSPORTER_ACTIVITY	0.99	0.34	0	0	0	not significant
10855	GO_REGULATION_OF_CD4_POSITIVE_ALPHA_BETA_T_CELL_ACTIVATIOI	0.99	0.34	0	0	0	not significant
10856	GO_SPROUTING_ANGIOGENESIS	0.99	0.34	0	0	0	not significant
10857	CHEBOTAEV_GR_TARGETS_DN	0.99	0.33	0	0	0	not significant
10858	GO_DEOXYRIBONUCLEASE_ACTIVITY	0.99	0.33	0	0	0	not significant
10859	GO_MAP_KINASE_KINASE_KINASE_ACTIVITY	0.99	0.33	0	0	0	not significant
10860	GO_NON_MOTILE_CILIUM_ASSEMBLY	0.99	0.33	0	0	0	not significant
10861	GO_POSITIVE_REGULATION_OF_NF_KAPPAB_TRANSCRIPTION_FACTOR_	0.99	0.33	0	0	0	not significant
10862	GO_STEROL_BINDING	0.99	0.33	0	0	0	not significant
10863	KANG_AR_TARGETS_DN	0.99	0.33	0	0	0	not significant
10864	REACTOME_RUNX2_REGULATES_BONE_DEVELOPMENT	0.99	0.33	0	0	0	not significant
10865	SENGUPTA_EBNA1_ANTICORRELATED	0.99	0.33	0	0	0	not significant
10866	ATF2_S_UP.V1_DN	0.99	0.32	0	0	0	not significant
10867	BIOCARTA_IFNA_PATHWAY	0.99	0.32	0	0	0	not significant
10868	BIOCARTA_SKP2E2F_PATHWAY	0.99	0.32	0	0	0	not significant
10869	CHR16Q22	0.99	0.32	0	0	0	not significant
10870	CHR9P13	0.99	0.32	0	0	0	not significant
10871	GO_ACTIN_POLYMERIZATION_OR_DEPOLYMERIZATION	0.99	0.32	0	0	0	not significant
10872	GO_NEGATIVE_REGULATION_OF_PRODUCTION_OF_MOLECULAR_MEDIA	0.99	0.32	0	0	0	not significant
10873	GO_POSITIVE_REGULATION_OF_FATTY_ACID_TRANSPORT	0.99	0.32	0	0	0	not significant
10874	GO_POSTSYNAPTIC_SPECIALIZATION_ASSEMBLY	0.99	0.32	0	0	0	not significant
10875	GO_RETINA_DEVELOPMENT_IN_CAMERA_TYPE_EYE	0.99	0.32	0	0	0	not significant
10876	GO_VACUOLAR_MEMBRANE	0.99	0.32	0	0	0	not significant
10877	HOWLIN_CITED1_TARGETS_1_UP	0.99	0.32	0	0	0	not significant
10878	HUMMERICH_MALIGNANT_SKIN_TUMOR_UP	0.99	0.32	0	0	0	not significant
10879	KEGG_FC_EPSILON_RI_SIGNALING_PATHWAY	0.99	0.32	0	0	0	not significant
10880	PARK_APL_PATHOGENESIS_DN	0.99	0.32	0	0	0	not significant
10881	REACTOME_ER_QUALITY_CONTROL_COMPARTMENT_ERQC	0.99	0.32	0	0	0	not significant
10882	REACTOME_KERATAN_SULFATE_BIOSYNTHESIS	0.99	0.32	0	0	0	not significant
10883	TAKEDA_TARGETS_OF_NUP98_HOXA9_FUSION_16D_UP	0.99	0.32	0	0	0	not significant
10884	WHITESIDE_CISPLATIN_RESISTANCE_DN	0.99	0.32	0	0	0	not significant
10885	WONG_IFNA2_RESISTANCE_DN	0.99	0.32	0	0	0	not significant
10886	ZHANG_TLX_TARGETS_36HR_UP	0.99	0.32	0	0	0	not significant
10887	BIOCARTA_ACH_PATHWAY	0.99	0.31	0	0	0	not significant
10888	GO_ESTABLISHMENT_OF_SISTER_CHROMATID_COHESION	0.99	0.31	0	0	0	not significant

10889	GO_LEUKOTRIENE_METABOLIC_PROCESS	0.99	0.31	0	0	0	not significant
10890	GO_MICROTUBULE_NUCLEATION_BY_INTERPHASE_MICROTUBULE_ORG/	0.99	0.31	0	0	0	not significant
10891	GO_NEGATIVE_REGULATION_OF_METALLOPEPTIDASE_ACTIVITY	0.99	0.31	0	0	0	not significant
10892	GO_POSITIVE_REGULATION_OF_ACTIVATED_T_CELL_PROLIFERATION	0.99	0.31	0	0	0	not significant
10893	GO_POSITIVE_REGULATION_OF_VASOCONSTRICTION	0.99	0.31	0	0	0	not significant
10894	GO_PROTEIN_KINASE_COMPLEX	0.99	0.31	0	0	0	not significant
10895	GO_REGULATION_OF_DEVELOPMENTAL_PIGMENTATION	0.99	0.31	0	0	0	not significant
10896	GO_REGULATION_OF_RESPONSE_TO_EXTERNAL_STIMULUS	0.99	0.31	0	0	0	not significant
10897	GO_REGULATION_OF_SPHINGOLIPID_BIOSYNTHETIC_PROCESS	0.99	0.31	0	0	0	not significant
10898	GO_REGULATION_OF_TOLL_LIKE_RECEPTOR_3_SIGNALING_PATHWAY	0.99	0.31	0	0	0	not significant
10899	REACTOME_CALNEXIN_CALRETICULIN_CYCLE	0.99	0.31	0	0	0	not significant
10900	REACTOME_SEMA4D_MEDIATED_INHIBITION_OF_CELL_ATTACHMENT_AN	0.99	0.31	0	0	0	not significant
10901	GO_BASIC_AMINO_ACID_TRANSPORT	0.99	0.30	0	0	0	not significant
10902	GO_POSITIVE_REGULATION_OF_CENTRIOLE_REPLICATION	0.99	0.30	0	0	0	not significant
10903	GO_REGULATION_OF_CELL_POPULATION_PROLIFERATION	0.99	0.30	0	0	0	not significant
10904	GO_REGULATION_OF_HISTONE_H3_K9_ACETYLATION	0.99	0.30	0	0	0	not significant
10905	MARIADASON_RESPONSE_TO_BUTYRATE_CURCUMIN_SULINDAC_TSA_2	0.99	0.30	0	0	0	not significant
10906	MCDOWELL_ACUTE_LUNG_INJURY_DN	0.99	0.30	0	0	0	not significant
10907	REACTOME_AXON_GUIDANCE	0.99	0.30	0	0	0	not significant
10908	REACTOME_NEUROTOXICITY_OF_CLOSTRIDIUM_TOXINS	0.99	0.30	0	0	0	not significant
10909	STARK_BRAIN_22Q11_DELETION	0.99	0.30	0	0	0	not significant
10910	VEGF_A_UP.V1_UP	0.99	0.30	0	0	0	not significant
10911	GO_PROTEIN_O_LINKED_GLYCOSYLATION	0.99	0.29	0	0	0	not significant
10912	REACTOME_ARYL_HYDROCARBON_RECEPTOR_SIGNALLING	0.99	0.29	0	0	0	not significant
10913	REACTOME_CASPASE_ACTIVATION_VIA_DEPENDENCE_RECEPTORS_IN_	0.99	0.28	0	0	0	not significant
10914	CRX_DN.V1_DN	1.00	0.36	0	0	0	not significant
10915	GO_CELLULAR_MODIFIED_AMINO_ACID_METABOLIC_PROCESS	1.00	0.36	0	0	0	not significant
10916	GO_REGULATION_OF_AXONOGENESIS	1.00	0.36	0	0	0	not significant
10917	MIKKELSEN_NPC_HCP_WITH_H3K4ME3_AND_H3K27ME3	1.00	0.36	0	0	0	not significant
10918	BIOCARTA_P27_PATHWAY	1.00	0.35	0	0	0	not significant
10919	DOANE_BREAST_CANCER_CLASSES_DN	1.00	0.35	0	0	0	not significant
10920	GO_ANTIGEN_BINDING	1.00	0.35	0	0	0	not significant
10921	GO_C_TERMINAL_PROTEIN_AMINO_ACID_MODIFICATION	1.00	0.35	0	0	0	not significant
10922	GO_MEMBRANE_DEPOLARIZATION	1.00	0.35	0	0	0	not significant
10923	GO_NEGATIVE_REGULATION_OF_CARBOHYDRATE_METABOLIC_PROCES	1.00	0.35	0	0	0	not significant
10924	GO_SPERMATID_DIFFERENTIATION	1.00	0.35	0	0	0	not significant
10925	GO_TRANSCRIPTION_COACTIVATOR_BINDING	1.00	0.35	0	0	0	not significant
10926	HOLLEMAN_ASPARAGINASE_RESISTANCE_B_ALL_DN	1.00	0.35	0	0	0	not significant
10927	OKAWA_NEUROBLASTOMA_1P36_31_DELETION	1.00	0.35	0	0	0	not significant
10928	VANTVEER_BREAST_CANCER_ESR1_UP	1.00	0.35	0	0	0	not significant
10929	YAO_TEMPORAL_RESPONSE_TO_PROGESTERONE_CLUSTER_1	1.00	0.35	0	0	0	not significant
10930	ZWANG_EGF_PERSISTENTLY_DN	1.00	0.35	0	0	0	not significant
10931	CHIANG_LIVER_CANCER_SUBCLASS_INTERFERON_DN	1.00	0.34	0	0	0	not significant
10932	CHR1Q22	1.00	0.34	0	0	0	not significant
10933	CHR5Q14	1.00	0.34	0	0	0	not significant
10934	GO_CELL_CELL_ADHESION_MEDIATED_BY_CADHERIN	1.00	0.34	0	0	0	not significant
10935	GO_CELLULAR_ION_HOMEOSTASIS	1.00	0.34	0	0	0	not significant
10936	GO_DENDRITIC_SHAFT	1.00	0.34	0	0	0	not significant
10937	GO_EMBRYONIC_SKELETAL_SYSTEM_DEVELOPMENT	1.00	0.34	0	0	0	not significant
10938	GO_POSITIVE_REGULATION_OF_MUSCLE_CONTRACTION	1.00	0.34	0	0	0	not significant
10939	GO_PREASSEMBLY_OF_GPI_ANCHOR_IN_ER_MEMBRANE	1.00	0.34	0	0	0	not significant
10940	GO_REGULATION_OF_MAP_KINASE_ACTIVITY	1.00	0.34	0	0	0	not significant
10941	GO_REGULATION_OF_PROTEIN_COMPLEX_STABILITY	1.00	0.34	0	0	0	not significant
10942	GO_REGULATION_OF_TRANSCRIPTION_FROM_RNA_POLYMERASE_II_PR	1.00	0.34	0	0	0	not significant
10943	GO_RESPONSE_TO_OSMOTIC_STRESS	1.00	0.34	0	0	0	not significant
10944	GO_SIGNAL_RECOGNITION_PARTICLE	1.00	0.34	0	0	0	not significant
10945	GO_TRANSITION_METAL_ION_HOMEOSTASIS	1.00	0.34	0	0	0	not significant
10946	GO_VACUOLAR_ACIDIFICATION	1.00	0.34	0	0	0	not significant
10947	HELLER_HDAC_TARGETS_SILENCED_BY_METHYLATION_DN	1.00	0.34	0	0	0	not significant
10948	VANDESLUIS_NORMAL_EMBRYOS_DN	1.00	0.34	0	0	0	not significant
10949	WAKABAYASHI_ADIPOGENESIS_PPARG_RXRA_BOUND_36HR	1.00	0.34	0	0	0	not significant
10950	YOSHIOKA_LIVER_CANCER_EARLY_RECURRENCE_DN	1.00	0.34	0	0	0	not significant
10951	AMUNDSON_RESPONSE_TO_ARSENITE	1.00	0.33	0	0	0	not significant
10952	CADWELL_ATG16L1_TARGETS_UP	1.00	0.33	0	0	0	not significant
10953	DAZARD_UV_RESPONSE_CLUSTER_G3	1.00	0.33	0	0	0	not significant
10954	GO_HORMONE_CATABOLIC_PROCESS	1.00	0.33	0	0	0	not significant
10955	GO_NEGATIVE_REGULATION_OF_CELL_AGING	1.00	0.33	0	0	0	not significant
10956	GO_OTIC_VESICLE_DEVELOPMENT	1.00	0.33	0	0	0	not significant
10957	GO_PHOSPHOLIPID_BINDING	1.00	0.33	0	0	0	not significant
10958	GO_REGULATION_OF_BONE_REMODELING	1.00	0.33	0	0	0	not significant
10959	GO_REGULATION_OF_FC_GAMMA_RECEPTOR_SIGNALING_PATHWAY_IN	1.00	0.33	0	0	0	not significant
10960	GO_RHO_PROTEIN_SIGNAL_TRANSDUCTION	1.00	0.33	0	0	0	not significant
10961	GO_VENTRICULAR_CARDIAC_MUSCLE_CELL_ACTION_POTENTIAL	1.00	0.33	0	0	0	not significant
10962	LIU_CMYB_TARGETS_UP	1.00	0.33	0	0	0	not significant
10963	PENG_GLUTAMINE_DEPRIVATION_UP	1.00	0.33	0	0	0	not significant
10964	REACTOME_SIGNALING_BY_RECEPTOR_TYROSINE_KINASES	1.00	0.33	0	0	0	not significant
10965	VALK_AML_CLUSTER_3	1.00	0.33	0	0	0	not significant
10966	CAFFAREL_RESPONSE_TO_THC_8HR_5_UP	1.00	0.32	0	0	0	not significant
10967	GEORGES_TARGETS_OF_MIR192_AND_MIR215	1.00	0.32	0	0	0	not significant
10968	GO_AXON_EXTENSION	1.00	0.32	0	0	0	not significant
10969	GO_BH_DOMAIN_BINDING	1.00	0.32	0	0	0	not significant
10970	GO_ESC_E_Z_COMPLEX	1.00	0.32	0	0	0	not significant
10971	GO_NEGATIVE_REGULATION_OF_FATTY_ACID_OXIDATION	1.00	0.32	0	0	0	not significant
10972	GO_POSITIVE_REGULATION_OF_EXCITATORY_POSTSYNAPTIC_POTENTI	1.00	0.32	0	0	0	not significant
10973	GO_POSITIVE_REGULATION_OF_PROTEIN_COMPLEX_ASSEMBLY	1.00	0.32	0	0	0	not significant
10974	GO_REGULATION_OF_ISOTYPE_SWITCHING	1.00	0.32	0	0	0	not significant
10975	JI_CARCINOGENESIS_BY_KRAS_AND_STK11_DN	1.00	0.32	0	0	0	not significant
10976	PEDRIOLI_MIR31_TARGETS_DN	1.00	0.32	0	0	0	not significant
10977	REACTOME_DISPLACEMENT_OF_DNA_GLYCOSYLASE_BY_APEX1	1.00	0.32	0	0	0	not significant
10978	GO_INSULIN_LIKE_GROWTH_FACTOR_I_BINDING	1.00	0.31	0	0	0	not significant
10979	GO_LIPOXYGENASE_PATHWAY	1.00	0.31	0	0	0	not significant
10980	GO_POSITIVE_REGULATION_OF_MAST_CELL_ACTIVATION	1.00	0.31	0	0	0	not significant
10981	GO_REGULATION_OF_ANTIGEN_PROCESSING_AND_PRESENTATION	1.00	0.31	0	0	0	not significant
10982	GO_3_PHOSPHOADENOSINE_5_PHOSPHOSULFATE_BINDING	1.00	0.30	0	0	0	not significant
10983	GO_CHROMAFFIN_GRANULE	1.00	0.30	0	0	0	not significant
10984	KORKOLA_EMBRYONAL_CARCINOMA	1.00	0.28	0	0	0	not significant
10985	GO_ESTABLISHMENT_OF_PROTEIN_LOCALIZATION_TO_ORGANELLE	1.01	0.38	0	0	0	not significant
10986	GO_MITOCHONDRIA_ASSOCIATED_ENDOPLASMIC_RETICULUM_MEMBRA	1.01	0.38	0	0	0	not significant
10987	BROWNE_HCMV_INFECTION_24HR_DN	1.01	0.37	0	0	0	not significant
10988	GO_RAB_PROTEIN_SIGNAL_TRANSDUCTION	1.01	0.37	0	0	0	not significant
10989	GO_VACUOLAR_TRANSPORT	1.01	0.37	0	0	0	not significant
10990	GRAHAM_CML_QUIESCENT_VS_NORMAL_DIVIDING_UP	1.01	0.37	0	0	0	not significant
10991	HATADA_METHYLATED_IN_LUNG_CANCER_UP	1.01	0.37	0	0	0	not significant
10992	PID_IL23_PATHWAY	1.01	0.37	0	0	0	not significant
10993	REACTOME_SMAD2_SMAD3:SMAD4_HETEROTRIMER_REGULATES_TRAN	1.01	0.37	0	0	0	not significant
10994	RUTELLA_RESPONSE_TO_HGF_DN	1.01	0.37	0	0	0	not significant
10995	VALK_AML_CLUSTER_12	1.01	0.37	0	0	0	not significant
10996	BIOCARTA_FBW7_PATHWAY	1.01	0.36	0	0	0	not significant
10997	GO_NEGATIVE_REGULATION_OF_PROTEIN_MODIFICATION_BY_SMALL_F	1.01	0.36	0	0	0	not significant

10998	GO_POSITIVE_REGULATION_OF_JUN_KINASE_ACTIVITY	1.01	0.36	0	0	0	not significant
10999	GO_REGULATION_OF_CELLULAR_COMPONENT_MOVEMENT	1.01	0.36	0	0	0	not significant
11000	RODRIGUES_DCC_TARGETS_DN	1.01	0.36	0	0	0	not significant
11001	SNF5_DN.V1_DN	1.01	0.36	0	0	0	not significant
11002	URS_ADIPOCYTE_DIFFERENTIATION_DN	1.01	0.36	0	0	0	not significant
11003	ACEVEDO_LIVER_CANCER_WITH_H3K9ME3_UP	1.01	0.35	0	0	0	not significant
11004	ESC_V6.5_UP_EARLY.V1_DN	1.01	0.35	0	0	0	not significant
11005	GO_CELL_CELL_ADHESION_VIA_PLASMA_MEMBRANE_ADHESION_MOLE(	1.01	0.35	0	0	0	not significant
11006	GO_HISTONE_H3_ACETYLATION	1.01	0.35	0	0	0	not significant
11007	GO_HISTONE_METHYLTRANSFERASE_ACTIVITY_H3_K9_SPECIFIC_	1.01	0.35	0	0	0	not significant
11008	GO_HISTONE_METHYLTRANSFERASE_COMPLEX	1.01	0.35	0	0	0	not significant
11009	GO_INTERLEUKIN_2_PRODUCTION	1.01	0.35	0	0	0	not significant
11010	GO_LIGAND_GATED_CALCIUM_CHANNEL_ACTIVITY	1.01	0.35	0	0	0	not significant
11011	GO_LYMPHANGIOGENESIS	1.01	0.35	0	0	0	not significant
11012	GO_NEGATIVE_REGULATION_OF_LYMPHOCYTE_DIFFERENTIATION	1.01	0.35	0	0	0	not significant
11013	GO_POSITIVE_REGULATION_OF_TRANSCRIPTION_REGULATORY_REGIO	1.01	0.35	0	0	0	not significant
11014	GO_REGULATION_OF_RETROGRADE_PROTEIN_TRANSPORT_ER_TO_CY	1.01	0.35	0	0	0	not significant
11015	GO_REPRESSING_TRANSCRIPTION_FACTOR_BINDING	1.01	0.35	0	0	0	not significant
11016	GO_SYMPORTER_ACTIVITY	1.01	0.35	0	0	0	not significant
11017	GO_TYPE_B_PANCREATIC_CELL_DEVELOPMENT	1.01	0.35	0	0	0	not significant
11018	REACTOME_FOXO_MEDIATED_TRANSCRIPTION_OF_CELL_DEATH_GENE	1.01	0.35	0	0	0	not significant
11019	REACTOME_SUMOYLATION_OF_DNA_METHYLATION_PROTEINS	1.01	0.35	0	0	0	not significant
11020	REACTOME_VEGFR2_MEDIATED_VASCULAR_PERMEABILITY	1.01	0.35	0	0	0	not significant
11021	BIOCARTA_PCAF_PATHWAY	1.01	0.34	0	0	0	not significant
11022	GO_ACUTE_INFLAMMATORY_RESPONSE_TO_ANTIGENIC_STIMULUS	1.01	0.34	0	0	0	not significant
11023	GO_ADULT_LOCOMOTORY_BEHAVIOR	1.01	0.34	0	0	0	not significant
11024	GO_CEREBELLAR_CORTEX_MORPHOGENESIS	1.01	0.34	0	0	0	not significant
11025	GO_COHESIN_LOADING	1.01	0.34	0	0	0	not significant
11026	GO_DIPEPTIDYL_PEPTIDASE_ACTIVITY	1.01	0.34	0	0	0	not significant
11027	GO_DNA_DAMAGE_INDUCED_PROTEIN_PHOSPHORYLATION	1.01	0.34	0	0	0	not significant
11028	GO_GLYCEROPHOSPHOLIPID_METABOLIC_PROCESS	1.01	0.34	0	0	0	not significant
11029	GO_INSULIN_SECRETION_INVOLVED_IN_CELLULAR_RESPONSE_TO_GLU	1.01	0.34	0	0	0	not significant
11030	GO_METANEPHRIC_NEPHRON_DEVELOPMENT	1.01	0.34	0	0	0	not significant
11031	GO_NEGATIVE_REGULATION_OF_BLOOD_VESSEL_DIAMETER	1.01	0.34	0	0	0	not significant
11032	GO_POSITIVE_REGULATION_OF_DNA_BINDING_TRANSCRIPTION_FACTOI	1.01	0.34	0	0	0	not significant
11033	GO_RESPONSE_TO_WATER	1.01	0.34	0	0	0	not significant
11034	GO_SYNAPTIC_VESICLE_MEMBRANE	1.01	0.34	0	0	0	not significant
11035	GO_T_HELPER_2_CELL_DIFFERENTIATION	1.01	0.34	0	0	0	not significant
11036	GO_TYPE_2_IMMUNE_RESPONSE	1.01	0.34	0	0	0	not significant
11037	MEISSNER_NPC_ICP_WITH_H3K4ME3	1.01	0.34	0	0	0	not significant
11038	PID_IL6_7_PATHWAY	1.01	0.34	0	0	0	not significant
11039	REACTOME_NCAM1_INTERACTIONS	1.01	0.34	0	0	0	not significant
11040	REACTOME_SIGNALING_BY_TYPE_1_INSULIN_LIKE_GROWTH_FACTOR_1	1.01	0.34	0	0	0	not significant
11041	ZHENG_GLIOBLASTOMA_PLASTICITY_DN	1.01	0.34	0	0	0	not significant
11042	BRACHAT_RESPONSE_TO_METHOTREXATE_UP	1.01	0.33	0	0	0	not significant
11043	GO_CYCLIC_NUCLEOTIDE_BIOSYNTHETIC_PROCESS	1.01	0.33	0	0	0	not significant
11044	GO_REGULATION_OF_MAMMARY_GLAND_EPITHELIAL_CELL_PROLIFERA	1.01	0.33	0	0	0	not significant
11045	GO_WNT_ACTIVATED_RECEPTOR_ACTIVITY	1.01	0.33	0	0	0	not significant
11046	LIAN_LIPA_TARGETS_6M	1.01	0.33	0	0	0	not significant
11047	MARTIN_NFKB_TARGETS_DN	1.01	0.33	0	0	0	not significant
11048	NIKOLSKY_BREAST_CANCER_21Q22_AMPLICON	1.01	0.33	0	0	0	not significant
11049	PID_INTEGRIN2_PATHWAY	1.01	0.33	0	0	0	not significant
11050	GO_CERAMIDE_1_PHOSPHATE_BINDING	1.01	0.32	0	0	0	not significant
11051	GO_PHOSPHATIDYLINOSITOL_N_ACETYLGLUCOSAMINYLTRANSFERASE	1.01	0.32	0	0	0	not significant
11052	GO_REGULATION_OF_INORGANIC_ANION_TRANSMEMBRANE_TRANSPOF	1.01	0.32	0	0	0	not significant
11053	GO_NEGATIVE_REGULATION_OF_FATTY_ACID_METABOLIC_PROCESS	1.01	0.31	0	0	0	not significant
11054	WANG_RESPONSE_TO_PACLITAXEL_VIA_MAPK8_UP	1.01	0.30	0	0	0	not significant
11055	GO_GLUCOCORTICOID_METABOLIC_PROCESS	1.01	0.29	0	0	0	not significant
11056	WONG_ADULT_TISSUE_STEM_MODULE	1.02	0.41	0	0	0	not significant
11057	ESC_V6.5_UP_EARLY.V1_UP	1.02	0.39	0	0	0	not significant
11058	GO_CELL_JUNCTION_ASSEMBLY	1.02	0.39	0	0	0	not significant
11059	GO_MEIOTIC_CELL_CYCLE	1.02	0.39	0	0	0	not significant
11060	GO_PEPTIDE_SECRETION	1.02	0.39	0	0	0	not significant
11061	GO_POSITIVE_REGULATION_OF_SECRETION	1.02	0.39	0	0	0	not significant
11062	GO_T_CELL_PROLIFERATION	1.02	0.39	0	0	0	not significant
11063	CAIRO_HEPATOBLASTOMA_CLASSES_DN	1.02	0.38	0	0	0	not significant
11064	CHYLA_CBFA2T3_TARGETS_DN	1.02	0.38	0	0	0	not significant
11065	FOSTER_TOLERANT_MACROPHAGE_DN	1.02	0.38	0	0	0	not significant
11066	GO_LEUKOCYTE_PROLIFERATION	1.02	0.38	0	0	0	not significant
11067	GO_PHOSPHOLIPID_TRANSPORT	1.02	0.38	0	0	0	not significant
11068	GO_RESPONSE_TO_INTERLEUKIN_15	1.02	0.38	0	0	0	not significant
11069	MITSIADES_RESPONSE_TO_APLIDIN_UP	1.02	0.38	0	0	0	not significant
11070	SMID_BREAST_CANCER_RELAPSE_IN_BONE_UP	1.02	0.38	0	0	0	not significant
11071	CHIANG_LIVER_CANCER_SUBCLASS_POLYSOMY7_UP	1.02	0.37	0	0	0	not significant
11072	CHR11Q24	1.02	0.37	0	0	0	not significant
11073	CHRXP11	1.02	0.37	0	0	0	not significant
11074	GO_MITOTIC_DNA_INTEGRITY_CHECKPOINT	1.02	0.37	0	0	0	not significant
11075	GO_MOTOR_NEURON_AXON_GUIDANCE	1.02	0.37	0	0	0	not significant
11076	GO_ODONTOGENESIS_OF_DENTIN_CONTAINING_TOOTH	1.02	0.37	0	0	0	not significant
11077	GO_POSITIVE_REGULATION_OF_INSULIN_SECRETION	1.02	0.37	0	0	0	not significant
11078	GO_POSITIVE_REGULATION_OF_T_CELL_APOPTOTIC_PROCESS	1.02	0.37	0	0	0	not significant
11079	GO_PRESYNAPTIC_MEMBRANE_ASSEMBLY	1.02	0.37	0	0	0	not significant
11080	GO_RECEPTOR_TYROSINE_KINASE_BINDING	1.02	0.37	0	0	0	not significant
11081	GO_REGULATION_OF_IMMUNE_EFFECTOR_PROCESS	1.02	0.37	0	0	0	not significant
11082	GO_REGULATION_OF_POSTSYNAPTIC_MEMBRANE_NEUROTRANSMITTEI	1.02	0.37	0	0	0	not significant
11083	PETROVA_ENDOTHELIUM_LYMPHATIC_VS_BLOOD_DN	1.02	0.37	0	0	0	not significant
11084	REACTOME_HEPARAN_SULFATE_HEPARIN_HS_GAG_METABOLISM	1.02	0.37	0	0	0	not significant
11085	RODRIGUES_THYROID_CARCINOMA_DN	1.02	0.37	0	0	0	not significant
11086	BARIS_THYROID_CANCER_DN	1.02	0.36	0	0	0	not significant
11087	GAUSSMANN_MLL_AF4_FUSION_TARGETS_B_UP	1.02	0.36	0	0	0	not significant
11088	GO_BLOOD_VESSEL_ENDOTHELIAL_CELL_MIGRATION	1.02	0.36	0	0	0	not significant
11089	GO_GLUTAMATE_RECEPTOR_BINDING	1.02	0.36	0	0	0	not significant
11090	GO_NEGATIVE_REGULATION_OF_DOUBLE_STRAND_BREAK_REPAIR_VIA	1.02	0.36	0	0	0	not significant
11091	GO_REGULATION_OF_CHEMOTAXIS	1.02	0.36	0	0	0	not significant
11092	KEGG_P53_SIGNALING_PATHWAY	1.02	0.36	0	0	0	not significant
11093	MARTIN_INTERACT_WITH_HDAC	1.02	0.36	0	0	0	not significant
11094	NIKOLSKY_BREAST_CANCER_8Q23_Q24_AMPLICON	1.02	0.36	0	0	0	not significant
11095	REACTOME_SURFACTANT_METABOLISM	1.02	0.36	0	0	0	not significant
11096	BIOCARTA_BIOPEPTIDES_PATHWAY	1.02	0.35	0	0	0	not significant
11097	GO_MIRNA_MEDIATED_INHIBITION_OF_TRANSLATION	1.02	0.35	0	0	0	not significant
11098	GO_NEGATIVE_REGULATION_OF_EPIDERMAL_GROWTH_FACTOR_ACTIV	1.02	0.35	0	0	0	not significant
11099	GO_NEUREXIN_FAMILY_PROTEIN_BINDING	1.02	0.35	0	0	0	not significant
11100	GO_PROTEIN_LOCALIZATION_TO_LYSOSOME	1.02	0.35	0	0	0	not significant
11101	GO_SET1C_COMPASS_COMPLEX	1.02	0.35	0	0	0	not significant
11102	PID_S1P_S1P1_PATHWAY	1.02	0.35	0	0	0	not significant
11103	REACTOME_SYNTHESIS_OF_UDP_N_ACETYL_GLUCOSAMINE	1.02	0.35	0	0	0	not significant
11104	GO_CELLULAR_RESPONSE_TO_OSMOTIC_STRESS	1.02	0.34	0	0	0	not significant
11105	GO_HISTONE_H3_K9_DEMETHYLATION	1.02	0.34	0	0	0	not significant
11106	GO_LAMELLAR_BODY	1.02	0.34	0	0	0	not significant

11107	GO_NATURAL_KILLER_CELL_LECTIN_LIKE_RECEPTOR_BINDING	1.02	0.34	0	0	0	not significant
11108	GO_POSITIVE_REGULATION_OF_ENDOTHELIAL_CELL_DIFFERENTIATION	1.02	0.34	0	0	0	not significant
11109	GO_PROTEIN_K11_LINKED_DEUBIQUITINATION	1.02	0.34	0	0	0	not significant
11110	NAKAMURA_ADIPOGENESIS_EARLY_DN	1.02	0.34	0	0	0	not significant
11111	PETRETTO_BLOOD_PRESSURE_DN	1.02	0.34	0	0	0	not significant
11112	REACTOME_TERMINATION_OF_O_GLYCAN_BIOSYNTHESIS	1.02	0.34	0	0	0	not significant
11113	GO_DNA_LIGATION_INVOLVED_IN_DNA_REPAIR	1.02	0.33	0	0	0	not significant
11114	GO_G_QUADRUPLEX_RNA_BINDING	1.02	0.33	0	0	0	not significant
11115	GO_INTERLEUKIN_12_SECRETION	1.02	0.33	0	0	0	not significant
11116	GO_OLIGOSACCHARIDE_BINDING	1.02	0.33	0	0	0	not significant
11117	GO_REGULATION_OF_METALLOENDOPEPTIDASE_ACTIVITY	1.02	0.33	0	0	0	not significant
11118	GO_RESPONSE_TO_MUSCLE_ACTIVITY	1.02	0.33	0	0	0	not significant
11119	PID_P38_ALPHA_BETA_PATHWAY	1.02	0.33	0	0	0	not significant
11120	GO_CD4_POSITIVE_ALPHA_BETA_T_CELL_CYTOKINE_PRODUCTION	1.02	0.32	0	0	0	not significant
11121	GO_NEGATIVE_REGULATION_OF_ARP2_3_COMPLEX_MEDIATED_ACTIN	1.02	0.32	0	0	0	not significant
11122	GO_REGULATION_OF_SMALL_GTPASE_MEDIATED_SIGNAL_TRANSDUCT	1.03	0.43	0	0	0	not significant
11123	GOZGIT_ESR1_TARGETS_DN	1.03	0.43	0	0	0	not significant
11124	GO_LIPID_BINDING	1.03	0.42	0	0	0	not significant
11125	BERENJENO_TRANSFORMED_BY_RHOA_DN	1.03	0.41	0	0	0	not significant
11126	CHR4P16	1.03	0.41	0	0	0	not significant
11127	GO_ANATOMICAL_STRUCTURE_FORMATION_INVOLVED_IN_MORPHOGEN	1.03	0.41	0	0	0	not significant
11128	GO_CELLULAR_CARBOHYDRATE_METABOLIC_PROCESS	1.03	0.41	0	0	0	not significant
11129	GO_I_KAPPAB_KINASE_NF_KAPPAB_SIGNALING	1.03	0.41	0	0	0	not significant
11130	GO_MAINTENANCE_OF_LOCATION_IN_CELL	1.03	0.41	0	0	0	not significant
11131	GO_NEGATIVE_REGULATION_OF_DNA_REPAIR	1.03	0.41	0	0	0	not significant
11132	GO_NEGATIVE_REGULATION_OF_LYMPHOCYTE_ACTIVATION	1.03	0.41	0	0	0	not significant
11133	KEGG_AMYOTROPHIC_LATERAL_SCLEROSIS_ALS	1.03	0.41	0	0	0	not significant
11134	AKT_UP_MTOR_DN.V1_DN	1.03	0.40	0	0	0	not significant
11135	BIOCARTA_PPARA_PATHWAY	1.03	0.40	0	0	0	not significant
11136	GO_NEGATIVE_REGULATION_OF_LEUKOCYTE_DIFFERENTIATION	1.03	0.40	0	0	0	not significant
11137	GO_NEGATIVE_REGULATION_OF_TRANSCRIPTION_BY_RNA_POLYMERAS	1.03	0.40	0	0	0	not significant
11138	GO_REGULATION_OF_RESPONSE_TO_BIOTIC_STIMULUS	1.03	0.40	0	0	0	not significant
11139	GO_RHO_GUANYL_NUCLEOTIDE_EXCHANGE_FACTOR_ACTIVITY	1.03	0.40	0	0	0	not significant
11140	GO_RNA_POLYMERASE_II_REPRESSING_TRANSCRIPTION_FACTOR_BIND	1.03	0.40	0	0	0	not significant
11141	MULLIGHAN_MLL_SIGNATURE_1_DN	1.03	0.40	0	0	0	not significant
11142	PHONG_TNF_RESPONSE_VIA_P38_PARTIAL	1.03	0.40	0	0	0	not significant
11143	PID_WNT_NONCANONICAL_PATHWAY	1.03	0.40	0	0	0	not significant
11144	SANA_RESPONSE_TO_IFNG_UP	1.03	0.40	0	0	0	not significant
11145	SHARMA_PILOCYTIC_ASTROCYTOMA_LOCATION_UP	1.03	0.40	0	0	0	not significant
11146	VANTVEER_BREAST_CANCER_METASTASIS_UP	1.03	0.40	0	0	0	not significant
11147	CHIARADONNA_NEOPLASTIC_TRANSFORMATION_CDC25_UP	1.03	0.39	0	0	0	not significant
11148	CHR6Q15	1.03	0.39	0	0	0	not significant
11149	CHR7Q22	1.03	0.39	0	0	0	not significant
11150	GO_POSITIVE_REGULATION_OF_B_CELL_ACTIVATION	1.03	0.39	0	0	0	not significant
11151	GO_POSITIVE_REGULATION_OF_MYELOID_CELL_DIFFERENTIATION	1.03	0.39	0	0	0	not significant
11152	GO_PROTEIN_POLYMERIZATION	1.03	0.39	0	0	0	not significant
11153	GO_PROTON_TRANSPORTING_V_TYPE_ATPASE_COMPLEX	1.03	0.39	0	0	0	not significant
11154	GO_REGULATION_OF_RESPONSE_TO_CYTOKINE_STIMULUS	1.03	0.39	0	0	0	not significant
11155	GO_SYNAPTIC_SIGNALING	1.03	0.39	0	0	0	not significant
11156	REACTOME_DISASSEMBLY_OF_THE_DESTRUCTION_COMPLEX_AND_RE	1.03	0.39	0	0	0	not significant
11157	GO_HIPPO_SIGNALING	1.03	0.38	0	0	0	not significant
11158	GO_NUCLEOTIDE_BINDING_DOMAIN_LEUCINE_RICH_REPEAT_CONTAININ	1.03	0.38	0	0	0	not significant
11159	GO_REGULATION_OF_MITOPHAGY	1.03	0.38	0	0	0	not significant
11160	REACTOME_CELL_JUNCTION_ORGANIZATION	1.03	0.38	0	0	0	not significant
11161	CHR14Q11	1.03	0.37	0	0	0	not significant
11162	CYCLIN_D1_UP.V1_DN	1.03	0.37	0	0	0	not significant
11163	GO_EUKARYOTIC_TRANSLATION_INITIATION_FACTOR_3_COMPLEX	1.03	0.37	0	0	0	not significant
11164	GO_KERATAN_SULFATE_CATABOLIC_PROCESS	1.03	0.37	0	0	0	not significant
11165	GO_PEPTIDYL_GLUTAMIC_ACID_MODIFICATION	1.03	0.37	0	0	0	not significant
11166	GO_RESPONSE_TO_SALT_STRESS	1.03	0.37	0	0	0	not significant
11167	GO_SOMITE_DEVELOPMENT	1.03	0.37	0	0	0	not significant
11168	GO_UDP_GLYCOSYLTRANSFERASE_ACTIVITY	1.03	0.37	0	0	0	not significant
11169	REACTOME_ASSEMBLY_OF_THE_HIV_VIRION	1.03	0.37	0	0	0	not significant
11170	REACTOME_ASSEMBLY_OF_THE_ORC_COMPLEX_AT_THE_ORIGIN_OF	1.03	0.37	0	0	0	not significant
11171	REACTOME_TRANSCRIPTIONAL_ACTIVITY_OF_SMAD2_SMAD3:SMAD4_HI	1.03	0.37	0	0	0	not significant
11172	WNT_UP.V1_DN	1.03	0.37	0	0	0	not significant
11173	GO_BASIC_AMINO_ACID_TRANSMEMBRANE_TRANSPORTER_ACTIVITY	1.03	0.36	0	0	0	not significant
11174	GO_NEGATIVE_REGULATION_OF_CARDIAC_MUSCLE_TISSUE_DEVELOPM	1.03	0.36	0	0	0	not significant
11175	GO_NEGATIVE_REGULATION_OF_GLYCOLYTIC_PROCESS	1.03	0.36	0	0	0	not significant
11176	GO_OLIGOSACCHARIDE_METABOLIC_PROCESS	1.03	0.36	0	0	0	not significant
11177	GO_PYRIMIDINE_CONTAINING_COMPOUND_SALVAGE	1.03	0.36	0	0	0	not significant
11178	GOERING_BLOOD_HDL_CHOLESTEROL_QTL_TRANS	1.03	0.36	0	0	0	not significant
11179	LANDEMAINE_LUNG_METASTASIS	1.03	0.36	0	0	0	not significant
11180	NOJIMA_SFRP2_TARGETS_UP	1.03	0.36	0	0	0	not significant
11181	REACTOME_CASPASE_MEDIATED_CLEAVAGE_OF_CYTOSKELETAL_PRC	1.03	0.36	0	0	0	not significant
11182	ABE_VEGFA_TARGETS	1.03	0.35	0	0	0	not significant
11183	GO_POSITIVE_REGULATION_OF_DOUBLE_STRAND_BREAK_REPAIR_VIA	1.03	0.35	0	0	0	not significant
11184	GO_RESPONSE_TO_STEROL_DEPLETION	1.03	0.35	0	0	0	not significant
11185	IIZUKA_LIVER_CANCER_PROGRESSION_L0_L1_UP	1.03	0.35	0	0	0	not significant
11186	GO_CERAMIDE_1_PHOSPHATE_TRANSPORT	1.03	0.34	0	0	0	not significant
11187	GO_CHEMOREPELLENT_ACTIVITY	1.03	0.34	0	0	0	not significant
11188	GO_REGULATION_OF_ACYL_COA_BIOSYNTHETIC_PROCESS	1.03	0.34	0	0	0	not significant
11189	GO_SULFATION	1.03	0.34	0	0	0	not significant
11190	GO_UBIQUITIN_DEPENDENT_ENDOCYTOSIS	1.03	0.34	0	0	0	not significant
11191	MANTOVANI_NFKB_TARGETS_DN	1.03	0.34	0	0	0	not significant
11192	BIOCARTA_INFLAM_PATHWAY	1.03	0.33	0	0	0	not significant
11193	GO_CONVERGENT_EXTENSION_INVOLVED_IN_GASTRULATION	1.03	0.33	0	0	0	not significant
11194	REACTOME_IONOTROPIC_ACTIVITY_OF_KAINATE_RECEPTORS	1.03	0.32	0	0	0	not significant
11195	GO_MULTICELLULAR_ORGANISM_REPRODUCTION	1.04	0.48	0	0	0	not significant
11196	GO_DIVALENT_INORGANIC_CATION_HOMEOSTASIS	1.04	0.44	0	0	0	not significant
11197	GO_POSITIVE_REGULATION_OF_INTRACELLULAR_SIGNAL_TRANSDUCTI	1.04	0.44	0	0	0	not significant
11198	GO_REGULATION_OF_CARBOHYDRATE_METABOLIC_PROCESS	1.04	0.44	0	0	0	not significant
11199	GO_SITE_OF_POLARIZED_GROWTH	1.04	0.44	0	0	0	not significant
11200	HALLMARK_COMPLEMENT	1.04	0.44	0	0	0	not significant
11201	CHR12P13	1.04	0.42	0	0	0	not significant
11202	CHR17Q25	1.04	0.42	0	0	0	not significant
11203	FIGUEROA_AML_METHYLATION_CLUSTER_6_UP	1.04	0.42	0	0	0	not significant
11204	GHANDHI_DIRECT_IRRADIATION_UP	1.04	0.42	0	0	0	not significant
11205	GO_POSITIVE_REGULATION_OF_INTRACELLULAR_PROTEIN_TRANSPORT	1.04	0.42	0	0	0	not significant
11206	GO_REGULATION_OF_DEFENSE_RESPONSE_TO_VIRUS	1.04	0.42	0	0	0	not significant
11207	MIKKELSEN_MEF_HCP_WITH_H3K27ME3	1.04	0.42	0	0	0	not significant
11208	RIGGI_EWING_SARCOMA_PROGENITOR_DN	1.04	0.42	0	0	0	not significant
11209	BIDUS_METASTASIS_DN	1.04	0.41	0	0	0	not significant
11210	CSR_LATE_UP.V1_DN	1.04	0.41	0	0	0	not significant
11211	GO_POSITIVE_REGULATION_OF_SIGNALING_RECEPTOR_ACTIVITY	1.04	0.41	0	0	0	not significant
11212	GO_PROTEIN_K63_LINKED_DEUBIQUITINATION	1.04	0.41	0	0	0	not significant
11213	GO_REGULATION_OF_CYCLIN_DEPENDENT_PROTEIN_KINASE_ACTIVITY	1.04	0.41	0	0	0	not significant
11214	GO_SENSORY_PERCEPTION_OF_PAIN	1.04	0.41	0	0	0	not significant
11215	MAGRANGEAS_MULTIPLE_MYELOMA_IGLL_VS_IGLK_UP	1.04	0.41	0	0	0	not significant

11216	RASHI_RESPONSE_TO_IONIZING_RADIATION_2	1.04	0.41	0	0	0	not significant
11217	REACTOME_ASSEMBLY_OF_COLLAGEN_FIBRILS_AND_OTHER_MULTIME	1.04	0.41	0	0	0	not significant
11218	REACTOME_INSULIN_RECEPTOR_RECYCLING	1.04	0.41	0	0	0	not significant
11219	REACTOME_SIGNALING_BY_ERBB4	1.04	0.41	0	0	0	not significant
11220	FERRANDO_LYL1_NEIGHBORS	1.04	0.40	0	0	0	not significant
11221	IKEDA_MIR30_TARGETS_DN	1.04	0.40	0	0	0	not significant
11222	KRAS.PROSTATE_UP.V1_UP	1.04	0.40	0	0	0	not significant
11223	MYC_UP.V1_DN	1.04	0.40	0	0	0	not significant
11224	PID_E2F_PATHWAY	1.04	0.40	0	0	0	not significant
11225	PID_KIT_PATHWAY	1.04	0.40	0	0	0	not significant
11226	REACTOME_NUCLEOBASE_BIOSYNTHESIS	1.04	0.40	0	0	0	not significant
11227	YOKOE_CANCER_TESTIS_ANTIGENS	1.04	0.40	0	0	0	not significant
11228	BIOCARTA_IL2_PATHWAY	1.04	0.39	0	0	0	not significant
11229	GO_CELLULAR_CARBOHYDRATE_BIOSYNTHETIC_PROCESS	1.04	0.39	0	0	0	not significant
11230	GO_NEGATIVE_REGULATION_OF_REACTIVE_OXYGEN_SPECIES_BIOSYN	1.04	0.39	0	0	0	not significant
11231	GO_STEROL_TRANSPORTER_ACTIVITY	1.04	0.39	0	0	0	not significant
11232	OUELLET_CULTURED_OVARIAN_CANCER_INVASIVE_VS_LMP_DN	1.04	0.39	0	0	0	not significant
11233	REACTOME_COLLAGEN_FORMATION	1.04	0.39	0	0	0	not significant
11234	VERRECCHIA_EARLY_RESPONSE_TO_TGFB1	1.04	0.39	0	0	0	not significant
11235	CHR15Q23	1.04	0.38	0	0	0	not significant
11236	GO_NEGATIVE_REGULATION_OF_PROTEIN_EXIT_FROM_ENDOPLASMIC_	1.04	0.38	0	0	0	not significant
11237	GO_POSITIVE_REGULATION_OF_ACUTE_INFLAMMATORY_RESPONSE	1.04	0.38	0	0	0	not significant
11238	GO_REGULATION_OF_LONG_TERM_SYNAPTIC_POTENTIATION	1.04	0.38	0	0	0	not significant
11239	GO_REGULATION_OF_PROTEIN_OLIGOMERIZATION	1.04	0.38	0	0	0	not significant
11240	HALLMARK_ANGIOGENESIS	1.04	0.38	0	0	0	not significant
11241	PID_PTP1B_PATHWAY	1.04	0.38	0	0	0	not significant
11242	REACTOME_PI_3K_CASCADE:FGFR3	1.04	0.38	0	0	0	not significant
11243	GO_BLEB_ASSEMBLY	1.04	0.37	0	0	0	not significant
11244	GO_CALCIUM_ION_TRANSMEMBRANE_TRANSPORT_VIA_HIGH_VOLTAGE	1.04	0.37	0	0	0	not significant
11245	GO_CYCLIC_NUCLEOTIDE_CATABOLIC_PROCESS	1.04	0.37	0	0	0	not significant
11246	GO_HISTONE_DEMETHYLASE_ACTIVITY_H3_K36_SPECIFIC_	1.04	0.37	0	0	0	not significant
11247	GO_PHOSPHATIDYLETHANOLAMINE_BINDING	1.04	0.37	0	0	0	not significant
11248	GO_POSITIVE_REGULATION_OF_TISSUE_REMODELING	1.04	0.37	0	0	0	not significant
11249	GO_POSTSYNAPSE_ASSEMBLY	1.04	0.37	0	0	0	not significant
11250	GO_POSTSYNAPTIC_DENSITY_ASSEMBLY	1.04	0.37	0	0	0	not significant
11251	GO_SERINE_PHOSPHORYLATION_OF_STAT_PROTEIN	1.04	0.37	0	0	0	not significant
11252	GO_SPECTRIN	1.04	0.37	0	0	0	not significant
11253	BIOCARTA_CTLA4_PATHWAY	1.04	0.36	0	0	0	not significant
11254	GO_G_PROTEIN_COUPLED_GLUTAMATE_RECEPTOR_BINDING	1.04	0.36	0	0	0	not significant
11255	GO_RESPONSE_TO_PROTOZOAN	1.04	0.36	0	0	0	not significant
11256	GO_VCP_NPL4_UFD1_AAA_ATPASE_COMPLEX	1.04	0.36	0	0	0	not significant
11257	GO_VENOUS_BLOOD_VESSEL_DEVELOPMENT	1.04	0.36	0	0	0	not significant
11258	GO_CYCLASE_ACTIVITY	1.04	0.35	0	0	0	not significant
11259	GO_HISTONE_H3_K4_DEMETHYLATION	1.04	0.35	0	0	0	not significant
11260	GO_NUCLEOTIDASE_ACTIVITY	1.04	0.35	0	0	0	not significant
11261	MARSON_FOXP3_TARGETS_STIMULATED_DN	1.04	0.35	0	0	0	not significant
11262	REACTOME_KERATAN_SULFATE_DEGRADATION	1.04	0.35	0	0	0	not significant
11263	REACTOME_SERINE_BIOSYNTHESIS	1.04	0.35	0	0	0	not significant
11264	REACTOME_SIGNALING_BY_BMP	1.04	0.35	0	0	0	not significant
11265	GO_LIPID_KINASE_ACTIVITY	1.04	0.34	0	0	0	not significant
11266	GO_NLRP3_INFLAMMASOME_COMPLEX	1.04	0.34	0	0	0	not significant
11267	GO_REGULATION_OF_POSTSYNAPTIC_SPECIALIZATION_ASSEMBLY	1.04	0.34	0	0	0	not significant
11268	MARTENS_TRETINOIN_RESPONSE_DN	1.05	0.53	0	0	0	not significant
11269	GO_NEGATIVE_REGULATION_OF_MULTICELLULAR_ORGANISMAL_PROCI	1.05	0.50	0	0	0	not significant
11270	GO_TRANSCRIPTION_COREPRESSOR_ACTIVITY	1.05	0.49	0	0	0	not significant
11271	STK33_NOMO_DN	1.05	0.49	0	0	0	not significant
11272	ZWANG_CLASS_3_TRANSIENTLY_INDUCED_BY_EGF	1.05	0.49	0	0	0	not significant
11273	TIEN_INTESTINE_PROBIOTICS_24HR_DN	1.05	0.48	0	0	0	not significant
11274	GO_NEGATIVE_REGULATION_OF_PEPTIDASE_ACTIVITY	1.05	0.47	0	0	0	not significant
11275	GO_PLASMA_MEMBRANE_PROTEIN_COMPLEX	1.05	0.47	0	0	0	not significant
11276	GO_REGULATION_OF_LYMPHOCYTE_ACTIVATION	1.05	0.46	0	0	0	not significant
11277	GO_RIBOSOMAL_SUBUNIT	1.05	0.46	0	0	0	not significant
11278	ASTON_MAJOR_DEPRESSIVE_DISORDER_DN	1.05	0.45	0	0	0	not significant
11279	GO_EPITHELIAL_CELL_PROLIFERATION	1.05	0.45	0	0	0	not significant
11280	GO_LOCOMOTORY_BEHAVIOR	1.05	0.45	0	0	0	not significant
11281	BENPORATH_NOS_TARGETS	1.05	0.44	0	0	0	not significant
11282	GO_REGULATION_OF_ALPHA_BETA_T_CELL_ACTIVATION	1.05	0.44	0	0	0	not significant
11283	LI_WILMS_TUMOR_VS_FETAL_KIDNEY_2_UP	1.05	0.44	0	0	0	not significant
11284	TAKEDA_TARGETS_OF_NUP98_HOXA9_FUSION_8D_UP	1.05	0.44	0	0	0	not significant
11285	GO_CYTOPLASMIC_TRANSLATION	1.05	0.43	0	0	0	not significant
11286	GO_EXTRINSIC_COMPONENT_OF_PLASMA_MEMBRANE	1.05	0.43	0	0	0	not significant
11287	YAGI_AML_SURVIVAL	1.05	0.43	0	0	0	not significant
11288	CHR3Q21	1.05	0.42	0	0	0	not significant
11289	GARGALOVIC_RESPONSE_TO_OXIDIZED_PHOSPHOLIPIDS_CYAN_UP	1.05	0.42	0	0	0	not significant
11290	GO_AMMONIUM_ION_METABOLIC_PROCESS	1.05	0.42	0	0	0	not significant
11291	GO_POSITIVE_REGULATION_OF_PEPTIDE_HORMONE_SECRETION	1.05	0.42	0	0	0	not significant
11292	GO_REGULATION_OF_MACROPHAGE_MIGRATION	1.05	0.42	0	0	0	not significant
11293	SHIN_B_CELL_LYMPHOMA_CLUSTER_2	1.05	0.42	0	0	0	not significant
11294	STEIN_ESRRA_TARGETS_DN	1.05	0.42	0	0	0	not significant
11295	BIOCARTA_HER2_PATHWAY	1.05	0.41	0	0	0	not significant
11296	GO_INTERLEUKIN_6_PRODUCTION	1.05	0.41	0	0	0	not significant
11297	GO_NEGATIVE_REGULATION_OF_ERYTHROCYTE_DIFFERENTIATION	1.05	0.41	0	0	0	not significant
11298	GO_POSITIVE_REGULATION_OF_CENTROSOME_DUPLICATION	1.05	0.41	0	0	0	not significant
11299	GO_POSITIVE_REGULATION_OF_HORMONE_SECRETION	1.05	0.41	0	0	0	not significant
11300	GO_POSITIVE_REGULATION_OF_LYMPHOCYTE_MEDIATED_IMMUNITY	1.05	0.41	0	0	0	not significant
11301	GO_REGULATION_OF_B_CELL_APOPTOTIC_PROCESS	1.05	0.41	0	0	0	not significant
11302	REACTOME_G_ALPHA_S_SIGNALLING_EVENTS	1.05	0.41	0	0	0	not significant
11303	URS_ADIPOCYTE_DIFFERENTIATION_UP	1.05	0.41	0	0	0	not significant
11304	BCAT_GDS748_DN	1.05	0.40	0	0	0	not significant
11305	BIOCARTA_TFF_PATHWAY	1.05	0.40	0	0	0	not significant
11306	GO_BRUSH_BORDER_MEMBRANE	1.05	0.40	0	0	0	not significant
11307	GO_NEGATIVE_REGULATION_OF_ACTIN_FILAMENT_POLYMERIZATION	1.05	0.40	0	0	0	not significant
11308	GO_PANCREAS_DEVELOPMENT	1.05	0.40	0	0	0	not significant
11309	GO_REGULATION_OF_GENE_EXPRESSION_BY_GENETIC_IMPRINTING	1.05	0.40	0	0	0	not significant
11310	GO_TRANSLATION_ELONGATION_FACTOR_ACTIVITY	1.05	0.40	0	0	0	not significant
11311	LOPEZ_MESOTHELIOMA_SURVIVAL_UP	1.05	0.40	0	0	0	not significant
11312	SWEET_KRAS_ONCOGENIC_SIGNATURE	1.05	0.40	0	0	0	not significant
11313	WINZEN_DEGRADED_VIA_KHSRP	1.05	0.40	0	0	0	not significant
11314	CHIARETTI_ACUTE_LYMPHOBLASTIC_LEUKEMIA_ZAP70	1.05	0.39	0	0	0	not significant
11315	GO_CERAMIDE_TRANSPORT	1.05	0.39	0	0	0	not significant
11316	GO_MITOTIC_DNA_REPLICATION_CHECKPOINT	1.05	0.39	0	0	0	not significant
11317	GO_NADPLUS_KINASE_ACTIVITY	1.05	0.39	0	0	0	not significant
11318	GO_NEUTRAL_LIPID_CATABOLIC_PROCESS	1.05	0.39	0	0	0	not significant
11319	GO_POSITIVE_REGULATION_OF_PROTEIN_GLYCOSYLATION	1.05	0.39	0	0	0	not significant
11320	GO_POST_EMBRYONIC_ANIMAL_ORGAN_MORPHOGENESIS	1.05	0.39	0	0	0	not significant
11321	GO_REGULATION_OF_CARDIAC_MUSCLE_CELL_ACTION_POTENTIAL	1.05	0.39	0	0	0	not significant
11322	MARIADASON_RESPONSE_TO_BUTYRATE_CURCUMIN_SULINDAC_TSA_8	1.05	0.39	0	0	0	not significant
11323	MEISSNER_BRAIN_HCP_WITH_H3K4ME2	1.05	0.39	0	0	0	not significant
11324	REACTOME_INTEGRIN_CELL_SURFACE_INTERACTIONS	1.05	0.39	0	0	0	not significant

11325	REACTOME_RESOLUTION_OF_D_LOOP_STRUCTURES	1.05	0.39	0	0	0	not significant
11326	SA_MMP_CYTOKINE_CONNECTION	1.05	0.39	0	0	0	not significant
11327	CLAUS_PGR_POSITIVE_MENINGIOMA_DN	1.05	0.38	0	0	0	not significant
11328	GO_CELL_JUNCTION_MAINTENANCE	1.05	0.38	0	0	0	not significant
11329	GO_CYTOPLASM_ORGANIZATION	1.05	0.38	0	0	0	not significant
11330	GO_HISTONE_H3_K36_DEMETHYLATION	1.05	0.38	0	0	0	not significant
11331	GO_CTF18_RFC_LIKE_COMPLEX	1.05	0.37	0	0	0	not significant
11332	GO_MEMBRANE_HYPERPOLARIZATION	1.05	0.37	0	0	0	not significant
11333	GO_NATURAL_KILLER_CELL_CYTOKINE_PRODUCTION	1.05	0.37	0	0	0	not significant
11334	HAHTOLA_MYCOSIS_FUNGOIDES_DN	1.05	0.37	0	0	0	not significant
11335	SCHAEFFER_PROSTATE_DEVELOPMENT_AND_CANCER_BOX5_UP	1.05	0.37	0	0	0	not significant
11336	TERAMOTO_OPN_TARGETS_CLUSTER_5	1.05	0.37	0	0	0	not significant
11337	YANAGISAWA_LUNG_CANCER_RECURRENCE	1.05	0.37	0	0	0	not significant
11338	GO_HYPERSENSITIVITY	1.05	0.36	0	0	0	not significant
11339	GO_REGULATION_OF_ADENYLATE_CYCLASE_ACTIVATING_G_PROTEIN_	1.05	0.36	0	0	0	not significant
11340	GO_AMINO_SUGAR_CATABOLIC_PROCESS	1.05	0.35	0	0	0	not significant
11341	GRAESSMANN_APOPTOSIS_BY_DOXORUBICIN_UP	1.06	0.59	0	0	0	not significant
11342	ZHANG_TLX_TARGETS_60HR_UP	1.06	0.51	0	0	0	not significant
11343	GO_REGULATION_OF_PEPTIDE_SECRETION	1.06	0.50	0	0	0	not significant
11344	LIU_PROSTATE_CANCER_DN	1.06	0.50	0	0	0	not significant
11345	UDAYAKUMAR_MED1_TARGETS_DN	1.06	0.50	0	0	0	not significant
11346	CHR17Q11	1.06	0.49	0	0	0	not significant
11347	YAGI_AML_WITH_T_8_21_TRANSLOCATION	1.06	0.49	0	0	0	not significant
11348	GO_SKELETAL_SYSTEM_DEVELOPMENT	1.06	0.48	0	0	0	not significant
11349	PEREZ_TP63_TARGETS	1.06	0.48	0	0	0	not significant
11350	WANG_HCP_PROSTATE_CANCER	1.06	0.48	0	0	0	not significant
11351	XU_GH1_AUTOCRINE_TARGETS_DN	1.06	0.48	0	0	0	not significant
11352	CTIP_DN.V1_DN	1.06	0.46	0	0	0	not significant
11353	GO_CATION_CHANNEL_ACTIVITY	1.06	0.46	0	0	0	not significant
11354	GO_HYDROLASE_ACTIVITY_ACTING_ON_CARBON_NITROGEN_BUT_NOT_	1.06	0.46	0	0	0	not significant
11355	GO_OLIGODENDROCYTE_DIFFERENTIATION	1.06	0.46	0	0	0	not significant
11356	CHIANG_LIVER_CANCER_SUBCLASS_CTNNB1_UP	1.06	0.45	0	0	0	not significant
11357	MARTIN_VIRAL_GPCR_SIGNALING_UP	1.06	0.45	0	0	0	not significant
11358	PGF_UP.V1_DN	1.06	0.45	0	0	0	not significant
11359	VERNELL_RETINOBLASTOMA_PATHWAY_UP	1.06	0.45	0	0	0	not significant
11360	PRC2_SUZ12_UP.V1_UP	1.06	0.44	0	0	0	not significant
11361	GO_K63_LINKED_POLYUBIQUITIN_MODIFICATION_DEPENDENT_PROTEIN_	1.06	0.43	0	0	0	not significant
11362	GO_KIDNEY_MORPHOGENESIS	1.06	0.43	0	0	0	not significant
11363	GO_SECONDARY_ALCOHOL_METABOLIC_PROCESS	1.06	0.43	0	0	0	not significant
11364	GO_SOMATIC_DIVERSIFICATION_OF_IMMUNE_RECEPTORS_VIA_GERMLI	1.06	0.43	0	0	0	not significant
11365	GO_TIGHT_JUNCTION	1.06	0.43	0	0	0	not significant
11366	KIM_GERMINAL_CENTER_T_HELPER_DN	1.06	0.43	0	0	0	not significant
11367	REACTOME_IMMUNOREGULATORY_INTERACTIONS_BETWEEN_A_LYMPH	1.06	0.43	0	0	0	not significant
11368	REACTOME_SIGNALING_BY_FGFR_IN_DISEASE	1.06	0.43	0	0	0	not significant
11369	CAFFAREL_RESPONSE_TO_THC_UP	1.06	0.42	0	0	0	not significant
11370	CHIARADONNA_NEOPLASTIC_TRANSFORMATION_KRAS_CDC25_DN	1.06	0.42	0	0	0	not significant
11371	CHR15Q14	1.06	0.42	0	0	0	not significant
11372	GO_REGULATION_OF_ESTABLISHMENT_OR_MAINTENANCE_OF_CELL_P(	1.06	0.42	0	0	0	not significant
11373	KANG_CISPLATIN_RESISTANCE_UP	1.06	0.42	0	0	0	not significant
11374	REACTOME_DOWNSTREAM_SIGNALING_OF_ACTIVATED_FGFR1	1.06	0.42	0	0	0	not significant
11375	CHR15Q13	1.06	0.41	0	0	0	not significant
11376	CHR4Q21	1.06	0.41	0	0	0	not significant
11377	GO_ATRIOVENTRICULAR_VALVE_DEVELOPMENT	1.06	0.41	0	0	0	not significant
11378	GO_CELL_FATE_COMMITMENT_INVOLVED_IN_FORMATION_OF_PRIMARY	1.06	0.41	0	0	0	not significant
11379	GO_CELL_PROLIFERATION_IN_FOREBRAIN	1.06	0.41	0	0	0	not significant
11380	GO_CELLULAR_RESPONSE_TO_ETHANOL	1.06	0.41	0	0	0	not significant
11381	GO_COHESIN_COMPLEX	1.06	0.41	0	0	0	not significant
11382	GO_INTERMEDIATE_FILAMENT_BASED_PROCESS	1.06	0.41	0	0	0	not significant
11383	GO_LYMPH_VESSEL_DEVELOPMENT	1.06	0.41	0	0	0	not significant
11384	GO_NEGATIVE_REGULATION_OF_DEFENSE_RESPONSE_TO_VIRUS	1.06	0.41	0	0	0	not significant
11385	GO_PROTEIN_DEMANNOSYLATION	1.06	0.41	0	0	0	not significant
11386	GO_REGULATION_OF_SMOOTH_MUSCLE_CONTRACTION	1.06	0.41	0	0	0	not significant
11387	PID_INTEGRIN3_PATHWAY	1.06	0.41	0	0	0	not significant
11388	GO_NEGATIVE_REGULATION_OF_OSTEOCLAST_DIFFERENTIATION	1.06	0.40	0	0	0	not significant
11389	GO_SENSORY_PERCEPTION_OF_TASTE	1.06	0.40	0	0	0	not significant
11390	GO_SPINAL_CORD_MOTOR_NEURON_DIFFERENTIATION	1.06	0.40	0	0	0	not significant
11391	HANSON_HRAS_SIGNALING_VIA_NFKB	1.06	0.40	0	0	0	not significant
11392	KEGG_GLYOXYLATE_AND_DICARBOXYLATE_METABOLISM	1.06	0.40	0	0	0	not significant
11393	SU_PLACENTA	1.06	0.40	0	0	0	not significant
11394	GO_ARYL_HYDROCARBON_RECEPTOR_BINDING	1.06	0.39	0	0	0	not significant
11395	GO_MEMBRANE_DEPOLARIZATION_DURING_ACTION_POTENTIAL	1.06	0.39	0	0	0	not significant
11396	GO_POSTSYNAPTIC_RECYCLING_ENDOSOME	1.06	0.39	0	0	0	not significant
11397	GO_RESPONSE_TO_POTASSIUM_ION	1.06	0.39	0	0	0	not significant
11398	GO_KETONE_CATABOLIC_PROCESS	1.06	0.38	0	0	0	not significant
11399	GO_MEMBRANE_RAFT_DISTRIBUTION	1.06	0.38	0	0	0	not significant
11400	HOLLEMAN_PREDNISOLONE_RESISTANCE_B_ALL_DN	1.06	0.38	0	0	0	not significant
11401	GO_CELL_MIGRATION_INVOLVED_IN_HEART_DEVELOPMENT	1.06	0.37	0	0	0	not significant
11402	GO_FAT_CELL_PROLIFERATION	1.06	0.37	0	0	0	not significant
11403	GO_HYALURONIC_ACID_BINDING	1.06	0.37	0	0	0	not significant
11404	GO_LOCOMOTION_INVOLVED_IN_LOCOMOTORY_BEHAVIOR	1.06	0.37	0	0	0	not significant
11405	GO_NEGATIVE_REGULATION_OF_B_CELL_PROLIFERATION	1.06	0.37	0	0	0	not significant
11406	GO_NEGATIVE_REGULATION_OF_INTERLEUKIN_1_SECRETION	1.06	0.37	0	0	0	not significant
11407	GO_POSITIVE_REGULATION_OF_CELL_POPULATION_PROLIFERATION	1.07	0.55	0	0	0	not significant
11408	BROWNE_HCMV_INFECTION_48HR_DN	1.07	0.54	0	0	0	not significant
11409	GO_REGULATION_OF_CELL_ACTIVATION	1.07	0.53	0	0	0	not significant
11410	TGFB_UP.V1_DN	1.07	0.51	0	0	0	not significant
11411	GO_PASSIVE_TRANSMEMBRANE_TRANSPORTER_ACTIVITY	1.07	0.50	0	0	0	not significant
11412	GO_TRANSFERASE_ACTIVITY_TRANSFERRING_HEXOSYL_GROUPS	1.07	0.50	0	0	0	not significant
11413	GO_POSITIVE_REGULATION_OF_LEUKOCYTE_CELL_CELL_ADHESION	1.07	0.49	0	0	0	not significant
11414	GO_REGULATION_OF_OSSIFICATION	1.07	0.49	0	0	0	not significant
11415	GO_REGULATION_OF_PROTEIN_POLYMERIZATION	1.07	0.49	0	0	0	not significant
11416	ESC_V6.5_UP_LATE.V1_UP	1.07	0.48	0	0	0	not significant
11417	GO_DEACETYLASE_ACTIVITY	1.07	0.47	0	0	0	not significant
11418	REACTOME_TRAF6_MEDIATED_INDUCTION_OF_TAK1_COMPLEX_WITHIN	1.07	0.46	0	0	0	not significant
11419	GO_HISTONE_H2A_MONOUBIQUITINATION	1.07	0.45	0	0	0	not significant
11420	GO_INTRINSIC_APOPTOTIC_SIGNALING_PATHWAY_BY_P53_CLASS_MEDI	1.07	0.45	0	0	0	not significant
11421	TAYLOR_METHYLATED_IN_ACUTE_LYMPHOBLASTIC_LEUKEMIA	1.07	0.45	0	0	0	not significant
11422	BHAT_ESR1_TARGETS_NOT_VIA_AKT1_DN	1.07	0.44	0	0	0	not significant
11423	GO_ESTABLISHMENT_OF_PROTEIN_LOCALIZATION_TO_VACUOLE	1.07	0.44	0	0	0	not significant
11424	GO_MAINTENANCE_OF_PROTEIN_LOCALIZATION_IN_ORGANELLE	1.07	0.44	0	0	0	not significant
11425	GO_SEMAPHORIN_RECEPTOR_BINDING	1.07	0.44	0	0	0	not significant
11426	MIKKELSEN_IPS_HCP_WITH_H3_UNMETHYLATED	1.07	0.44	0	0	0	not significant
11427	REACTOME_DEGRADATION_OF_THE_EXTRACELLULAR_MATRIX	1.07	0.44	0	0	0	not significant
11428	REACTOME_IRS_MEDIATED_SIGNALLING	1.07	0.44	0	0	0	not significant
11429	CHR10P12	1.07	0.43	0	0	0	not significant
11430	GO_POSITIVE_REGULATION_OF_CYTOKINE_PRODUCTION_INVOLVED_IN	1.07	0.43	0	0	0	not significant
11431	GO_POSITIVE_REGULATION_OF_PROTEIN_POLYMERIZATION	1.07	0.43	0	0	0	not significant
11432	GO_PROTEIN_TARGETING_TO_VACUOLE	1.07	0.43	0	0	0	not significant
11433	GO_REGULATION_OF_GLYCOPROTEIN_BIOSYNTHETIC_PROCESS	1.07	0.43	0	0	0	not significant

11434	MYLLYKANGAS_AMPLIFICATION_HOT_SPOT_8	1.07	0.43	0	0	0	not significant
11435	POMEROY_MEDULLOBLASTOMA_PROGNOSIS_UP	1.07	0.43	0	0	0	not significant
11436	REACTOME_TRANSCRIPTIONAL_REGULATION_BY_THE_AP_2_TFAP2_FA	1.07	0.43	0	0	0	not significant
11437	GO_CYTOPLASMIC_SEQUESTERING_OF_PROTEIN	1.07	0.42	0	0	0	not significant
11438	GO_FORMATION_OF_CYTOPLASMIC_TRANSLATION_INITIATION_COMPLE	1.07	0.42	0	0	0	not significant
11439	GO_GENETIC_IMPRINTING	1.07	0.42	0	0	0	not significant
11440	GO_HISTONE_H2A_UBIQUITINATION	1.07	0.42	0	0	0	not significant
11441	GO_INTRINSIC_COMPONENT_OF_POSTSYNAPTIC_SPECIALIZATION_MEMI	1.07	0.42	0	0	0	not significant
11442	GO_NEGATIVE_REGULATION_OF_INTERLEUKIN_6_PRODUCTION	1.07	0.42	0	0	0	not significant
11443	GO_OVULATION	1.07	0.42	0	0	0	not significant
11444	GO_REGULATION_OF_ENDOCYTIC_RECYCLING	1.07	0.42	0	0	0	not significant
11445	LEE_METASTASIS_AND_ALTERNATIVE_SPLICING_UP	1.07	0.42	0	0	0	not significant
11446	MAEKAWA_ATF2_TARGETS	1.07	0.42	0	0	0	not significant
11447	XU_GH1_EXOGENOUS_TARGETS_UP	1.07	0.42	0	0	0	not significant
11448	GO_CONVERGENT_EXTENSION	1.07	0.41	0	0	0	not significant
11449	GO_NEURONAL_ION_CHANNEL_CLUSTERING	1.07	0.41	0	0	0	not significant
11450	GO_POSITIVE_REGULATION_OF_KILLING_OF_CELLS_OF_OTHER_ORGAI	1.07	0.41	0	0	0	not significant
11451	MATZUK_MEIOTIC_AND_DNA_REPAIR	1.07	0.41	0	0	0	not significant
11452	YAO_TEMPORAL_RESPONSE_TO_PROGESTERONE_CLUSTER_3	1.07	0.41	0	0	0	not significant
11453	GO_CEREBELLAR_PURKINJE_CELL_LAYER_MORPHOGENESIS	1.07	0.40	0	0	0	not significant
11454	GO_INTERMEDIATE_FILAMENT_ORGANIZATION	1.07	0.40	0	0	0	not significant
11455	GO_NEGATIVE_REGULATION_OF_PATHWAY_RESTRICTED_SMAD_PROTI	1.07	0.40	0	0	0	not significant
11456	GO_STEROID_DEHYDROGENASE_ACTIVITY	1.07	0.40	0	0	0	not significant
11457	GO_STRAND_INVASION	1.07	0.40	0	0	0	not significant
11458	GYORFFY_DOXORUBICIN_RESISTANCE	1.07	0.40	0	0	0	not significant
11459	BAKER_HEMATOPOESIS_STAT1_TARGETS	1.07	0.39	0	0	0	not significant
11460	GO_NEGATIVE_REGULATION_OF_T_CELL_DIFFERENTIATION_IN_THYMUS	1.07	0.39	0	0	0	not significant
11461	GO_POSITIVE_REGULATION_OF_MAMMARY_GLAND_EPITHELIAL_CELL_F	1.07	0.39	0	0	0	not significant
11462	LIU_TOPBP1_TARGETS	1.07	0.39	0	0	0	not significant
11463	GO_ALPHA_BETA_T_CELL_RECEPTOR_COMPLEX	1.07	0.38	0	0	0	not significant
11464	REACTOME_MET_RECEPTOR_RECYCLING	1.07	0.38	0	0	0	not significant
11465	GO_QUATERNARY_AMMONIUM_GROUP_TRANSPORT	1.07	0.37	0	0	0	not significant
11466	KRIGE_RESPONSE_TO_TOSEDOSTAT_6HR_UP	1.08	0.66	0	0	0	not significant
11467	GO_POSITIVE_REGULATION_OF_ESTABLISHMENT_OF_PROTEIN_LOCALI	1.08	0.57	0	0	0	not significant
11468	GO_POSITIVE_REGULATION_OF_MAP_KINASE_ACTIVITY	1.08	0.55	0	0	0	not significant
11469	MEISSNER_NPC_HCP_WITH_H3_UNMETHYLATED	1.08	0.54	0	0	0	not significant
11470	GO_SENSORY_PERCEPTION	1.08	0.52	0	0	0	not significant
11471	GO_RECEPTOR_REGULATOR_ACTIVITY	1.08	0.51	0	0	0	not significant
11472	REACTOME_COSTIMULATION_BY_THE_CD28_FAMILY	1.08	0.50	0	0	0	not significant
11473	REACTOME_NEGATIVE_REGULATORS_OF_DDX58_IFIH1_SIGNALING	1.08	0.49	0	0	0	not significant
11474	BILANGES_RAPAMYCIN_SENSITIVE_GENES	1.08	0.48	0	0	0	not significant
11475	BROCKE_APOPTOSIS_REVERSED_BY_IL6	1.08	0.48	0	0	0	not significant
11476	GO_CYTOKINE_PRODUCTION_INVOLVED_IN_IMMUNE_RESPONSE	1.08	0.48	0	0	0	not significant
11477	GO_CYTOPLASMIC_PATTERN_RECOGNITION_RECEPTOR_SIGNALING_PA	1.08	0.48	0	0	0	not significant
11478	GO_EAR_MORPHOGENESIS	1.08	0.48	0	0	0	not significant
11479	GO_REGULATION_OF_DENDRITE_MORPHOGENESIS	1.08	0.48	0	0	0	not significant
11480	GO_REGULATION_OF_EXTENT_OF_CELL_GROWTH	1.08	0.48	0	0	0	not significant
11481	CHR12Q21	1.08	0.47	0	0	0	not significant
11482	GO_LIPOPROTEIN_METABOLIC_PROCESS	1.08	0.47	0	0	0	not significant
11483	GO_NEGATIVE_REGULATION_OF_I_KAPPAB_KINASE_NF_KAPPAB_SIGNAI	1.08	0.47	0	0	0	not significant
11484	GO_NEGATIVE_REGULATION_OF_EPITHELIAL_CELL_DIFFERENTIATION	1.08	0.46	0	0	0	not significant
11485	GO_REGULATION_OF_GLUCONEOGENESIS	1.08	0.46	0	0	0	not significant
11486	GO_ACETYLGLUCOSAMINYLTRANSFERASE_ACTIVITY	1.08	0.45	0	0	0	not significant
11487	GO_AMINE_TRANSPORT	1.08	0.45	0	0	0	not significant
11488	GO_CYTOPLASMIC_SEQUESTERING_OF_TRANSCRIPTION_FACTOR	1.08	0.45	0	0	0	not significant
11489	GO_G_PROTEIN_ALPHA_SUBUNIT_BINDING	1.08	0.45	0	0	0	not significant
11490	GO_PROTEIN_ADP_RIBOSYLATION	1.08	0.45	0	0	0	not significant
11491	GO_REGULATION_OF_OLIGODENDROCYTE_DIFFERENTIATION	1.08	0.45	0	0	0	not significant
11492	KEGG_GLYCOSYLPHOSPHATIDYLINOSITOL_GPI_ANCHOR_BIOSYNTHESIS	1.08	0.45	0	0	0	not significant
11493	KEGG_PRIMARY_IMMUNODEFICIENCY	1.08	0.45	0	0	0	not significant
11494	GO_ENDOCARDIAL_CUSHION_DEVELOPMENT	1.08	0.44	0	0	0	not significant
11495	GO_POSITIVE_REGULATION_OF_CALCIUM_ION_DEPENDENT_EXOCYTOS	1.08	0.44	0	0	0	not significant
11496	GO_STEROID_DEHYDROGENASE_ACTIVITY_ACTING_ON_THE_CH_OH_GI	1.08	0.44	0	0	0	not significant
11497	GO_STRIATED_MUSCLE_CELL_PROLIFERATION	1.08	0.44	0	0	0	not significant
11498	GO_TRANSFORMING_GROWTH_FACTOR_BETA_BINDING	1.08	0.44	0	0	0	not significant
11499	REACTOME_KERATAN_SULFATE_KERATIN_METABOLISM	1.08	0.44	0	0	0	not significant
11500	REACTOME_SEMA4D_IN_SEMAPHORIN_SIGNALING	1.08	0.44	0	0	0	not significant
11501	GO_NEGATIVE_REGULATION_OF_IMMUNOGLOBULIN_PRODUCTION	1.08	0.43	0	0	0	not significant
11502	GO_NEGATIVE_REGULATION_OF_INTERLEUKIN_1_BETA_SECRETION	1.08	0.43	0	0	0	not significant
11503	CHEOK_RESPONSE_TO_MERCAPTOPURINE_AND_LD_MTX_DN	1.08	0.42	0	0	0	not significant
11504	GO_FAT_SOLUBLE_VITAMIN_METABOLIC_PROCESS	1.08	0.42	0	0	0	not significant
11505	GO_NEGATIVE_REGULATION_OF_MEIOTIC_CELL_CYCLE	1.08	0.42	0	0	0	not significant
11506	GO_SREBP_SIGNALING_PATHWAY	1.08	0.42	0	0	0	not significant
11507	REACTOME_COLLAGEN_BIOSYNTHESIS_AND_MODIFYING_ENZYMES	1.08	0.42	0	0	0	not significant
11508	GO_CHROMATIN_MEDIATED_MAINTENANCE_OF_TRANSCRIPTION	1.08	0.41	0	0	0	not significant
11509	GO_DOUBLE_STRAND_BREAK_REPAIR_VIA_BREAK_INDUCED_REPLICATI	1.08	0.41	0	0	0	not significant
11510	GO_NEGATIVE_REGULATION_OF_NITRIC_OXIDE_BIOSYNTHETIC_PROCES	1.08	0.41	0	0	0	not significant
11511	GO_NEGATIVE_REGULATION_OF_RECEPTOR_INTERNALIZATION	1.08	0.41	0	0	0	not significant
11512	GO_POSITIVE_REGULATION_OF_CYTOKINE_SECRETION_INVOLVED_IN_II	1.08	0.41	0	0	0	not significant
11513	GO_STRIATED_MUSCLE_ATROPHY	1.08	0.41	0	0	0	not significant
11514	REACTOME_ACTIVATION_OF_PUMA_AND_TRANSLOCATION_TO_MITOCH	1.08	0.41	0	0	0	not significant
11515	GO_AUTOPHAGY_OF_NUCLEUS	1.08	0.40	0	0	0	not significant
11516	GO_MELANOSOME_ASSEMBLY	1.08	0.40	0	0	0	not significant
11517	GO_VITAMIN_D_METABOLIC_PROCESS	1.08	0.40	0	0	0	not significant
11518	GO_LOCOMOTORY_EXPLORATION_BEHAVIOR	1.08	0.39	0	0	0	not significant
11519	GO_PLATELET_DENSE_GRANULE_LUMEN	1.08	0.39	0	0	0	not significant
11520	GO_REELIN_MEDIATED_SIGNALING_PATHWAY	1.08	0.39	0	0	0	not significant
11521	GO_X_CHROMOSOME	1.08	0.39	0	0	0	not significant
11522	GO_INTERLEUKIN_13_SECRETION	1.08	0.38	0	0	0	not significant
11523	GO_REGULATION_OF_TRIGLYCERIDE_METABOLIC_PROCESS	1.08	0.38	0	0	0	not significant
11524	GO_NEGATIVE_REGULATION_OF_BIOSYNTHETIC_PROCESS	1.09	0.74	0	0	0	not significant
11525	LIAO_METASTASIS	1.09	0.62	0	0	0	not significant
11526	BENPORATH_EED_TARGETS	1.09	0.60	0	0	0	not significant
11527	GO_CELLULAR_PROCESS_INVOLVED_IN_REPRODUCTION_IN_MULTICELL	1.09	0.59	0	0	0	not significant
11528	HIRSCH_CELLULAR_TRANSFORMATION_SIGNATURE_UP	1.09	0.57	0	0	0	not significant
11529	GO_GLYCOSYLATION	1.09	0.56	0	0	0	not significant
11530	GO_MONOVALENT_INORGANIC_CATION_HOMEOSTASIS	1.09	0.54	0	0	0	not significant
11531	GO_NEGATIVE_REGULATION_OF_DNA_METABOLIC_PROCESS	1.09	0.54	0	0	0	not significant
11532	SENESE_HDAC1_AND_HDAC2_TARGETS_DN	1.09	0.54	0	0	0	not significant
11533	GO_NEGATIVE_REGULATION_OF_IMMUNE_RESPONSE	1.09	0.53	0	0	0	not significant
11534	GO_NEURON_PROJECTION_GUIDANCE	1.09	0.53	0	0	0	not significant
11535	GO_RESPONSE_TO_CALCIUM_ION	1.09	0.53	0	0	0	not significant
11536	CHR2Q11	1.09	0.51	0	0	0	not significant
11537	GO_CELL_CELL_JUNCTION_ORGANIZATION	1.09	0.51	0	0	0	not significant
11538	GO_POSITIVE_REGULATION_OF_SYNAPTIC_TRANSMISSION	1.09	0.51	0	0	0	not significant
11539	MISSIAGLIA_REGULATED_BY_METHYLATION_UP	1.09	0.51	0	0	0	not significant
11540	SERVITJA_LIVER_HNF1A_TARGETS_DN	1.09	0.51	0	0	0	not significant
11541	BOQUEST_STEM_CELL_UP	1.09	0.49	0	0	0	not significant
11542	GO_IMMUNOGLOBULIN_PRODUCTION_INVOLVED_IN_IMMUNOGLOBULIN_I	1.09	0.49	0	0	0	not significant

11543	GO_MAMMARY_GLAND_EPITHELIUM_DEVELOPMENT	1.09	0.49	0	0	0	not significant
11544	GO_PDZ_DOMAIN_BINDING	1.09	0.49	0	0	0	not significant
11545	GO_POSITIVE_REGULATION_OF_OSSIFICATION	1.09	0.49	0	0	0	not significant
11546	GO_POSITIVE_REGULATION_OF_OSTEOBLAST_DIFFERENTIATION	1.09	0.49	0	0	0	not significant
11547	GO_SNARE_BINDING	1.09	0.49	0	0	0	not significant
11548	GO_STEROID_BINDING	1.09	0.49	0	0	0	not significant
11549	GO_T_CELL_DIFFERENTIATION_IN_THYMUS	1.09	0.49	0	0	0	not significant
11550	INGRAM_SHH_TARGETS_DN	1.09	0.49	0	0	0	not significant
11551	CLASPER_LYMPHATIC_VESSELS_DURING_METASTASIS_DN	1.09	0.48	0	0	0	not significant
11552	GO_INOSITOL_PHOSPHATE_BIOSYNTHETIC_PROCESS	1.09	0.48	0	0	0	not significant
11553	GO_POSITIVE_REGULATION_OF_INTRINSIC_APOPTOTIC_SIGNALING_PAT	1.09	0.48	0	0	0	not significant
11554	GO_TRANSCRIPTION_COFACTOR_BINDING	1.09	0.48	0	0	0	not significant
11555	MANTOVANI_VIRAL_GPCR_SIGNALING_UP	1.09	0.48	0	0	0	not significant
11556	OLSSON_E2F3_TARGETS_DN	1.09	0.48	0	0	0	not significant
11557	GO_COLLAGEN_TRIMER	1.09	0.47	0	0	0	not significant
11558	GO_ENDOSOME_TO_LYSOSOME_TRANSPORT	1.09	0.47	0	0	0	not significant
11559	GO_ENERGY_HOMEOSTASIS	1.09	0.47	0	0	0	not significant
11560	WILENSKY_RESPONSE_TO_DARAPLADIB	1.09	0.47	0	0	0	not significant
11561	CHR4P15	1.09	0.46	0	0	0	not significant
11562	GO_RESPONSE_TO_ATP	1.09	0.46	0	0	0	not significant
11563	CHR5Q21	1.09	0.45	0	0	0	not significant
11564	GO_CERAMIDE_CATABOLIC_PROCESS	1.09	0.45	0	0	0	not significant
11565	GO_NEGATIVE_REGULATION_OF_CARDIAC_MUSCLE_TISSUE_GROWTH	1.09	0.45	0	0	0	not significant
11566	GO_POSITIVE_REGULATION_OF_DENDRITIC_SPINE_MORPHOGENESIS	1.09	0.45	0	0	0	not significant
11567	GO_TAU_PROTEIN_KINASE_ACTIVITY	1.09	0.45	0	0	0	not significant
11568	WANG_METHYLATED_IN_BREAST_CANCER	1.09	0.45	0	0	0	not significant
11569	BRACHAT_RESPONSE_TO_CISPLATIN	1.09	0.44	0	0	0	not significant
11570	GO_REGULATION_OF_PROTEIN_GLYCOSYLATION	1.09	0.44	0	0	0	not significant
11571	SIMBULAN_PARP1_TARGETS_UP	1.09	0.44	0	0	0	not significant
11572	BIOCARTA_AKAPCENTROSOME_PATHWAY	1.09	0.43	0	0	0	not significant
11573	GO_CELLULAR_RESPONSE_TO_MURAMYL_DIPEPTIDE	1.09	0.43	0	0	0	not significant
11574	GO_PLATELET_DERIVED_GROWTH_FACTOR_RECEPTOR_BETA_SIGNAL	1.09	0.43	0	0	0	not significant
11575	GO_REGULATION_OF_CALCIUM_ION_IMPORT	1.09	0.43	0	0	0	not significant
11576	BIOCARTA_IL10_PATHWAY	1.09	0.42	0	0	0	not significant
11577	GO_ACTIN_FILAMENT_REORGANIZATION	1.09	0.42	0	0	0	not significant
11578	GO_CERAMIDE_TRANSPORTER_ACTIVITY	1.09	0.42	0	0	0	not significant
11579	GO_EUCHROMATIN	1.09	0.42	0	0	0	not significant
11580	GO_RNA_POLYMERASE_II_INTRONIC_TRANSCRIPTION_REGULATORY_RE	1.09	0.42	0	0	0	not significant
11581	WEBER_METHYLATED_HCP_IN_FIBROBLAST_DN	1.09	0.42	0	0	0	not significant
11582	GO_NEUROBLAST_DIVISION	1.09	0.41	0	0	0	not significant
11583	GO_MITRAL_VALVE_DEVELOPMENT	1.09	0.40	0	0	0	not significant
11584	GO_THALAMUS_DEVELOPMENT	1.09	0.40	0	0	0	not significant
11585	BIOCARTA_NOTCH_PATHWAY	1.09	0.39	0	0	0	not significant
11586	BLALOCK_ALZHEIMERS_DISEASE_UP	1.10	0.93	0	0	0	not significant
11587	REACTOME_GENERIC_TRANSCRIPTION_PATHWAY	1.10	0.81	0	0	0	not significant
11588	GRAESSMANN_RESPONSE_TO_MC_AND_DOXORUBICIN_UP	1.10	0.68	0	0	0	not significant
11589	LIM_MAMMARY_STEM_CELL_DN	1.10	0.64	0	0	0	not significant
11590	GAZDA_DIAMOND_BLACKFAN_ANEMIA_ERYTHROID_DN	1.10	0.63	0	0	0	not significant
11591	GO_NEGATIVE_REGULATION_OF_IMMUNE_SYSTEM_PROCESS	1.10	0.62	0	0	0	not significant
11592	CREIGHTON_ENDOCRINE_THERAPY_RESISTANCE_2	1.10	0.60	0	0	0	not significant
11593	HADDAD_B_LYMPHOCYTE_PROGENITOR	1.10	0.60	0	0	0	not significant
11594	SMID_BREAST_CANCER_BASAL_DN	1.10	0.60	0	0	0	not significant
11595	MCCABE_BOUND_BY_HOXC6	1.10	0.58	0	0	0	not significant
11596	CHR17Q21	1.10	0.57	0	0	0	not significant
11597	PLASARI_TGFB1_TARGETS_10HR_DN	1.10	0.57	0	0	0	not significant
11598	SHEDDEN_LUNG_CANCER_GOOD_SURVIVAL_A12	1.10	0.57	0	0	0	not significant
11599	GO_ENDOPLASMIC_RETICULUM_LUMEN	1.10	0.56	0	0	0	not significant
11600	HUANG_GATA2_TARGETS_UP	1.10	0.55	0	0	0	not significant
11601	GO_G_PROTEIN_COUPLED_RECEPTOR_BINDING	1.10	0.54	0	0	0	not significant
11602	GO_MALE_SEX_DIFFERENTIATION	1.10	0.53	0	0	0	not significant
11603	ZHANG_TLX_TARGETS_UP	1.10	0.53	0	0	0	not significant
11604	GO_HOMOPHILIC_CELL_ADHESION_VIA_PLASMA_MEMBRANE_ADHESION	1.10	0.52	0	0	0	not significant
11605	GO_SINGLE_FERTILIZATION	1.10	0.52	0	0	0	not significant
11606	GENTILE_UV_RESPONSE_CLUSTER_D6	1.10	0.51	0	0	0	not significant
11607	GO_GLYCOGEN_BIOSYNTHETIC_PROCESS	1.10	0.51	0	0	0	not significant
11608	GO_RECEPTOR_LOCALIZATION_TO_SYNAPSE	1.10	0.51	0	0	0	not significant
11609	GO_REGULATION_OF_OSTEOBLAST_DIFFERENTIATION	1.10	0.51	0	0	0	not significant
11610	GO_REGULATION_OF_PHOSPHOLIPASE_ACTIVITY	1.10	0.51	0	0	0	not significant
11611	CHR18P11	1.10	0.50	0	0	0	not significant
11612	KRAS.LUNG.BREAST_UP.V1_DN	1.10	0.50	0	0	0	not significant
11613	CHR20P11	1.10	0.49	0	0	0	not significant
11614	GO_POLYSACCHARIDE_BIOSYNTHETIC_PROCESS	1.10	0.49	0	0	0	not significant
11615	REACTOME_SIGNAL_TRANSDUCTION_BY_L1	1.10	0.49	0	0	0	not significant
11616	CHR1Q44	1.10	0.48	0	0	0	not significant
11617	GO_UBIQUITIN_CONJUGATING_ENZYME_BINDING	1.10	0.48	0	0	0	not significant
11618	KEGG_BASAL_CELL_CARCINOMA	1.10	0.48	0	0	0	not significant
11619	REACTOME_ASSEMBLY_AND_CELL_SURFACE_PRESENTATION_OF_NMD	1.10	0.48	0	0	0	not significant
11620	CHR9P24	1.10	0.47	0	0	0	not significant
11621	GO_ENDOPLASMIC_RETICULUM_MANNOSE_TRIMMING	1.10	0.47	0	0	0	not significant
11622	GO_INTERLEUKIN_6_SECRETION	1.10	0.47	0	0	0	not significant
11623	GO_MACROPHAGE_CHEMOTAXIS	1.10	0.47	0	0	0	not significant
11624	GO_SYNAPTIC_TRANSMISSION_GLUTAMATERGIC	1.10	0.47	0	0	0	not significant
11625	GO_TUMOR_NECROSIS_FACTOR_BIOSYNTHETIC_PROCESS	1.10	0.47	0	0	0	not significant
11626	KYNG_RESPONSE_TO_H2O2_VIA_ERCC6	1.10	0.47	0	0	0	not significant
11627	LIN_SILENCED_BY_TUMOR_MICROENVIRONMENT	1.10	0.47	0	0	0	not significant
11628	REACTOME_LISTERIA_MONOCYTOGENES_ENTRY_INTO_HOST_CELLS	1.10	0.47	0	0	0	not significant
11629	GO_HEPATOCYTE_GROWTH_FACTOR_RECEPTOR_SIGNALING_PATHWA	1.10	0.46	0	0	0	not significant
11630	GO_NEGATIVE_REGULATION_OF_B_CELL_ACTIVATION	1.10	0.46	0	0	0	not significant
11631	GO_POSITIVE_REGULATION_OF_PROTEIN_OLIGOMERIZATION	1.10	0.46	0	0	0	not significant
11632	GO_REGULATION_OF_CD4_POSITIVE_ALPHA_BETA_T_CELL_DIFFERENT	1.10	0.46	0	0	0	not significant
11633	GO_STEROID_CATABOLIC_PROCESS	1.10	0.46	0	0	0	not significant
11634	REACTOME_LAMININ_INTERACTIONS	1.10	0.46	0	0	0	not significant
11635	GO_CHORION_DEVELOPMENT	1.10	0.45	0	0	0	not significant
11636	GO_LONG_TERM_SYNAPTIC_DEPRESSION	1.10	0.45	0	0	0	not significant
11637	GO_NATURAL_KILLER_CELL_ACTIVATION_INVOLVED_IN_IMMUNE_RESPO	1.10	0.45	0	0	0	not significant
11638	GO_PHOSPHATE_ION_TRANSMEMBRANE_TRANSPORT	1.10	0.45	0	0	0	not significant
11639	REACTOME_DOWNREGULATION_OF_ERBB2:ERBB3_SIGNALING	1.10	0.45	0	0	0	not significant
11640	REACTOME_SHC1_EVENTS_IN_ERBB2_SIGNALING	1.10	0.45	0	0	0	not significant
11641	GO_MANNOSE_BINDING	1.10	0.44	0	0	0	not significant
11642	GO_NEGATIVE_REGULATION_OF_CALCINEURIN_MEDIATED_SIGNALING	1.10	0.44	0	0	0	not significant
11643	GO_OXIDIZED_BASE_LESION_DNA_N_GLYCOSYLASE_ACTIVITY	1.10	0.44	0	0	0	not significant
11644	GO_POSITIVE_REGULATION_OF_INFLAMMATORY_RESPONSE_TO_ANTIG	1.10	0.44	0	0	0	not significant
11645	RUNNE_GENDER_EFFECT_UP	1.10	0.44	0	0	0	not significant
11646	GO_CALCIUM_CATION_ANTIPORTER_ACTIVITY	1.10	0.43	0	0	0	not significant
11647	GO_EXTRAEMBRYONIC_MEMBRANE_DEVELOPMENT	1.10	0.43	0	0	0	not significant
11648	GO_MARGINAL_ZONE_B_CELL_DIFFERENTIATION	1.10	0.43	0	0	0	not significant
11649	GO_REGULATION_OF_KILLING_OF_CELLS_OF_OTHER_ORGANISM	1.10	0.43	0	0	0	not significant
11650	GO_REGULATION_OF_POSTSYNAPTIC_NEUROTRANSMITTER_RECEPTOI	1.10	0.42	0	0	0	not significant
11651	GO_ADP_RIBOSYLATION_FACTOR_BINDING	1.10	0.41	0	0	0	not significant

11652	GO_CYTOSKELETAL_ANCHORING_AT_PLASMA_MEMBRANE	1.10	0.41	0	0	0	not significant
11653	GO_HISTONE_H3_K27_DEMETHYLATION	1.10	0.40	0	0	0	not significant
11654	GO_REGULATION_OF_BLOOD_VESSEL_REMODELING	1.10	0.40	0	0	0	not significant
11655	GO_PROTEIN_LOCALIZATION_TO_MEMBRANE	1.11	0.74	0	0	0	not significant
11656	GO_LYMPHOCYTE_ACTIVATION	1.11	0.72	0	0	0	not significant
11657	CHR22Q13	1.11	0.63	0	0	0	not significant
11658	GO_REGULATION_OF_PEPTIDYL_TYROSINE_PHOSPHORYLATION	1.11	0.61	0	0	0	not significant
11659	KEGG_CALCIUM_SIGNALING_PATHWAY	1.11	0.60	0	0	0	not significant
11660	CHR8Q24	1.11	0.59	0	0	0	not significant
11661	CHR11Q13	1.11	0.57	0	0	0	not significant
11662	GO_NEGATIVE_REGULATION_OF_DEFENSE_RESPONSE	1.11	0.56	0	0	0	not significant
11663	RB_P107_DN.V1_UP	1.11	0.56	0	0	0	not significant
11664	GO_REGULATION_OF_LEUKOCYTE_MEDIATED_IMMUNITY	1.11	0.55	0	0	0	not significant
11665	GO_POSITIVE_REGULATION_OF_CYTOKINE_SECRETION	1.11	0.54	0	0	0	not significant
11666	REACTOME_NEGATIVE_REGULATION_OF_THE_PI3K_AKT_NETWORK	1.11	0.54	0	0	0	not significant
11667	BOHN_PRIMARY_IMMUNODEFICIENCY_SYNDROM_UP	1.11	0.53	0	0	0	not significant
11668	PID_AP1_PATHWAY	1.11	0.53	0	0	0	not significant
11669	PID_ECADHERIN_NASCENT_AJ_PATHWAY	1.11	0.53	0	0	0	not significant
11670	REACTOME_TRANSCRIPTIONAL_REGULATION_BY_E2F6	1.11	0.53	0	0	0	not significant
11671	GO_BASE_EXCISION_REPAIR	1.11	0.52	0	0	0	not significant
11672	GO_MITOGEN_ACTIVATED_PROTEIN_KINASE_BINDING	1.11	0.52	0	0	0	not significant
11673	GO_REGULATION_OF_POLYSACCHARIDE_METABOLIC_PROCESS	1.11	0.52	0	0	0	not significant
11674	KONDO_PROSTATE_CANCER_WITH_H3K27ME3	1.11	0.52	0	0	0	not significant
11675	WANG_BARRETTS_ESOPHAGUS_UP	1.11	0.52	0	0	0	not significant
11676	GO_DETOXIFICATION	1.11	0.51	0	0	0	not significant
11677	GO_NEGATIVE_REGULATION_OF_GLUCONEOGENESIS	1.11	0.51	0	0	0	not significant
11678	GO_NEGATIVE_REGULATION_OF_SMALL_MOLECULE_METABOLIC_PROC	1.11	0.51	0	0	0	not significant
11679	GO_POSITIVE_REGULATION_OF_CILIUM_ASSEMBLY	1.11	0.51	0	0	0	not significant
11680	VANHARANTA_UTERINE_FIBROID_DN	1.11	0.51	0	0	0	not significant
11681	CHR11Q21	1.11	0.50	0	0	0	not significant
11682	GO_DNA_REPLICATION_INITIATION	1.11	0.50	0	0	0	not significant
11683	GO_PROTEIN_PALMITOYLATION	1.11	0.50	0	0	0	not significant
11684	PID_BMP_PATHWAY	1.11	0.50	0	0	0	not significant
11685	GO_GTP_RHO_BINDING	1.11	0.49	0	0	0	not significant
11686	REACTOME INLB MEDIATED ENTRY OF LISTERIA MONOCYTOGENES I	1.11	0.49	0	0	0	not significant
11687	GO_APOPTOTIC_PROCESS_INVOLVED_IN_DEVELOPMENT	1.11	0.48	0	0	0	not significant
11688	GO_POSITIVE_REGULATION_OF_ALPHA_BETA_T_CELL_PROLIFERATION	1.11	0.48	0	0	0	not significant
11689	GO_PROTEIN_POLY_ADP_RIBOSYLATION	1.11	0.48	0	0	0	not significant
11690	GO_METALLOCARBOXYPEPTIDASE_ACTIVITY	1.11	0.47	0	0	0	not significant
11691	GO_PHOSPHATIDYLCHOLINE_METABOLIC_PROCESS	1.11	0.47	0	0	0	not significant
11692	GUENTHER_GROWTH_SPHERICAL_VS_ADHERENT_UP	1.11	0.47	0	0	0	not significant
11693	REACTOME_SIGNAL_ATTENUATION	1.11	0.47	0	0	0	not significant
11694	CHR13Q33	1.11	0.46	0	0	0	not significant
11695	GO_EMBRYONIC_PLACENTA_MORPHOGENESIS	1.11	0.46	0	0	0	not significant
11696	GO_EXPORT_ACROSS_PLASMA_MEMBRANE	1.11	0.46	0	0	0	not significant
11697	GO_NEGATIVE_REGULATION_OF_PLATELET_ACTIVATION	1.11	0.46	0	0	0	not significant
11698	GO_PROTEIN_LOCALIZATION_TO_BASOLATERAL_PLASMA_MEMBRANE	1.11	0.46	0	0	0	not significant
11699	GO_CEREBELLAR_PURKINJE_CELL_LAYER_FORMATION	1.11	0.44	0	0	0	not significant
11700	GO_RNA_STRAND_ANNEALING_ACTIVITY	1.11	0.44	0	0	0	not significant
11701	GO_T_CELL_RECEPTOR_BINDING	1.11	0.44	0	0	0	not significant
11702	GO_TRANSFORMING_GROWTH_FACTOR_BETA_ACTIVATION	1.11	0.44	0	0	0	not significant
11703	ONGUSAHA_BRCA1_TARGETS_UP	1.11	0.44	0	0	0	not significant
11704	GO_DENSE_CORE_GRANULE	1.11	0.43	0	0	0	not significant
11705	GO_HISTONE_DEMETHYLASE_ACTIVITY_H3_K27_SPECIFIC_	1.11	0.43	0	0	0	not significant
11706	GO NEGATIVE REGULATION OF ACTIVIN RECEPTOR SIGNALING PATH	1.11	0.43	0	0	0	not significant
11707	REACTOME_CDC6_ASSOCIATION_WITH_THE_ORC:ORIGIN_COMPLEX	1.11	0.43	0	0	0	not significant
11708	GO_CELLULAR_RESPONSE_TO_POTASSIUM_ION	1.11	0.39	0	0	0	not significant
11709	GO_REGULATION_OF_TRANS_SYNAPTIC_SIGNALING	1.12	0.72	0	0	0	not significant
11710	GO_PROXIMAL_PROMOTER_SEQUENCE_SPECIFIC_DNA_BINDING	1.12	0.69	0	0	0	not significant
11711	GO_REGULATION_OF_LEUKOCYTE_DIFFERENTIATION	1.12	0.69	0	0	0	not significant
11712	CHARAFE_BREAST_CANCER_LUMINAL_VS_BASAL_UP	1.12	0.68	0	0	0	not significant
11713	STK33_NOMO_UP	1.12	0.68	0	0	0	not significant
11714	GO_REGULATION_OF_T_CELL_ACTIVATION	1.12	0.66	0	0	0	not significant
11715	LEE_DIFFERENTIATING_T_LYMPHOCYTE	1.12	0.63	0	0	0	not significant
11716	PODAR_RESPONSE_TO_ADAPHOSTIN_UP	1.12	0.60	0	0	0	not significant
11717	GO_B_CELL_ACTIVATION_INVOLVED_IN_IMMUNE_RESPONSE	1.12	0.58	0	0	0	not significant
11718	GO_G_PROTEIN_COUPLED_RECEPTOR_ACTIVITY	1.12	0.58	0	0	0	not significant
11719	GO_POSITIVE_REGULATION_OF_ACTIN_FILAMENT_POLYMERIZATION	1.12	0.58	0	0	0	not significant
11720	HALLMARK_KRAS_SIGNALING_DN	1.12	0.58	0	0	0	not significant
11721	GO_REGULATION_OF_DNA_DEPENDENT_DNA_REPLICATION	1.12	0.57	0	0	0	not significant
11722	GO_CELLULAR_RESPONSE_TO_ALCOHOL	1.12	0.56	0	0	0	not significant
11723	RADAEVA_RESPONSE_TO_IFNA1_UP	1.12	0.56	0	0	0	not significant
11724	GO_LYMPHOCYTE_HOMEOSTASIS	1.12	0.55	0	0	0	not significant
11725	GO_POSITIVE_REGULATION_OF_CELL_KILLING	1.12	0.55	0	0	0	not significant
11726	GO_REGULATION_OF_POSTSYNAPTIC_MEMBRANE_POTENTIAL	1.12	0.55	0	0	0	not significant
11727	GO_MEMBRANE_INVAGINATION	1.12	0.54	0	0	0	not significant
11728	GO_NEGATIVE_REGULATION_OF_T_CELL_PROLIFERATION	1.12	0.54	0	0	0	not significant
11729	RELA_DN.V1_DN	1.12	0.54	0	0	0	not significant
11730	GO_REGULATION_OF_CELL_AGING	1.12	0.53	0	0	0	not significant
11731	REACTOME RUNX1 INTERACTS WITH CO FACTORS WHOSE PRECISE	1.12	0.53	0	0	0	not significant
11732	GO_CELLULAR_RESPONSE_TO_RETINOIC_ACID	1.12	0.52	0	0	0	not significant
11733	GO_GOLGI_TO_ENDOSOME_TRANSPORT	1.12	0.52	0	0	0	not significant
11734	GO_SOMATIC_RECOMBINATION_OF_IMMUNOGLOBULIN_GENE_SEGMENT	1.12	0.52	0	0	0	not significant
11735	KIM_ALL_DISORDERS_OLIGODENDROCYTE_NUMBER_CORR_DN	1.12	0.52	0	0	0	not significant
11736	REACTOME_REGULATION_OF_INSULIN_LIKE_GROWTH_FACTOR_IGF_TR	1.12	0.52	0	0	0	not significant
11737	GO_NADPLUS_ADP_RIBOSYLTRANSFERASE_ACTIVITY	1.12	0.51	0	0	0	not significant
11738	GO POSITIVE REGULATION OF PROTEIN TARGETING TO MITOCHOND	1.12	0.51	0	0	0	not significant
11739	GO_AXONAL_FASCICULATION	1.12	0.50	0	0	0	not significant
11740	GO_ORGANELLE_MEMBRANE_CONTACT_SITE	1.12	0.50	0	0	0	not significant
11741	GO_POSITIVE_REGULATION_OF_EPITHELIAL_CELL_DIFFERENTIATION	1.12	0.50	0	0	0	not significant
11742	GO_PROTEIN_MANNOSYLATION	1.12	0.50	0	0	0	not significant
11743	GO_PROTEIN_TRANSPORT_WITHIN_PLASMA_MEMBRANE	1.12	0.50	0	0	0	not significant
11744	GO_REGULATION_OF_T_CELL_DIFFERENTIATION_IN_THYMUS	1.12	0.50	0	0	0	not significant
11745	MEISSNER_ES_ICP_WITH_H3K4ME3	1.12	0.50	0	0	0	not significant
11746	BIOCARTA_P53_PATHWAY	1.12	0.49	0	0	0	not significant
11747	GO_CYCLIC_NUCLEOTIDE_BINDING	1.12	0.49	0	0	0	not significant
11748	KEGG_GLYCOSAMINOGLYCAN_DEGRADATION	1.12	0.49	0	0	0	not significant
11749	GAUSSMANN_MLL_AF4_FUSION_TARGETS_D_UP	1.12	0.48	0	0	0	not significant
11750	GO_DICARBOXYLIC_ACID_CATABOLIC_PROCESS	1.12	0.48	0	0	0	not significant
11751	GO_METANEPHROS_MORPHOGENESIS	1.12	0.48	0	0	0	not significant
11752	GO_TRANSPOSITION	1.12	0.48	0	0	0	not significant
11753	GO_WHITE_FAT_CELL_DIFFERENTIATION	1.12	0.48	0	0	0	not significant
11754	MATHEW_FANCONI_ANEMIA_GENES	1.12	0.48	0	0	0	not significant
11755	REACTOME_SUMOYLATION_OF_TRANSCRIPTION_FACTORS	1.12	0.48	0	0	0	not significant
11756	TONKS_TARGETS_OF_RUNX1_RUNX1T1_FUSION_GRANULOCYTE_DN	1.12	0.48	0	0	0	not significant
11757	YANG_BREAST_CANCER_ESR1_DN	1.12	0.48	0	0	0	not significant
11758	GO_PROTEIN_POLYGLUTAMYLATION	1.12	0.47	0	0	0	not significant
11759	GO_REGULATION_OF_DNA_LIGATION	1.12	0.47	0	0	0	not significant
11760	GO_AMMONIUM_TRANSMEMBRANE_TRANSPORTER_ACTIVITY	1.12	0.46	0	0	0	not significant

11761	GO_ANTIGEN_PROCESSING_AND_PRESENTATION_OF_LIPID_ANTIGEN_V	1.12	0.46	0	0	0	not significant
11762	GO_MEMBRANE_RAFT_LOCALIZATION	1.12	0.46	0	0	0	not significant
11763	GO_MICROFIBRIL	1.12	0.46	0	0	0	not significant
11764	GO_SPONGIOTROPHOBLAST_LAYER_DEVELOPMENT	1.12	0.46	0	0	0	not significant
11765	GO_NEGATIVE_REGULATION_OF_ACTIVATED_T_CELL_PROLIFERATION	1.12	0.45	0	0	0	not significant
11766	MYLLYKANGAS_AMPLIFICATION_HOT_SPOT_9	1.12	0.45	0	0	0	not significant
11767	REACTOME_CLEC7A_INFLAMMASOME_PATHWAY	1.12	0.44	0	0	0	not significant
11768	CUI_TCF21_TARGETS_2_DN	1.13	0.86	0	0	0	not significant
11769	GO_SEXUAL_REPRODUCTION	1.13	0.85	0	0	0	not significant
11770	GO_GAMETE_GENERATION	1.13	0.79	0	0	0	not significant
11771	ZHENG_BOUND_BY_FOXP3	1.13	0.78	0	0	0	not significant
11772	HELLER_HDAC_TARGETS_UP	1.13	0.76	0	0	0	not significant
11773	STK33_UP	1.13	0.70	0	0	0	not significant
11774	GO_PLASMA_MEMBRANE_RECEPTOR_COMPLEX	1.13	0.65	0	0	0	not significant
11775	GO_DEVELOPMENTAL_MATURATION	1.13	0.64	0	0	0	not significant
11776	LIU_SOX4_TARGETS_UP	1.13	0.64	0	0	0	not significant
11777	GO_ACTIN_FILAMENT_POLYMERIZATION	1.13	0.63	0	0	0	not significant
11778	GO_NEGATIVE_REGULATION_OF_HEMOPOIESIS	1.13	0.62	0	0	0	not significant
11779	CHEN_LVAD_SUPPORT_OF_FAILING_HEART_UP	1.13	0.59	0	0	0	not significant
11780	GO_CELL_MATURATION	1.13	0.59	0	0	0	not significant
11781	FIGUEROA_AML_METHYLATION_CLUSTER_4_UP	1.13	0.58	0	0	0	not significant
11782	REACTOME_GLYCOSAMINOGLYCAN_METABOLISM	1.13	0.58	0	0	0	not significant
11783	GO_PROTEIN_LOCALIZATION_TO_MICROTUBULE_ORGANIZING_CENTER	1.13	0.56	0	0	0	not significant
11784	KEGG_ACUTE_MYELOID_LEUKEMIA	1.13	0.56	0	0	0	not significant
11785	BIOCARTA_PDGF_PATHWAY	1.13	0.55	0	0	0	not significant
11786	GO_MESONEPHROS_DEVELOPMENT	1.13	0.55	0	0	0	not significant
11787	GO_NEURON_MATURATION	1.13	0.54	0	0	0	not significant
11788	RAMASWAMY_METASTASIS_DN	1.13	0.54	0	0	0	not significant
11789	REACTOME_SIGNALING_BY_NON_RECEPTOR_TYROSINE_KINASES	1.13	0.54	0	0	0	not significant
11790	DARWICHE_PAPILLOMA_RISK_HIGH_VS_LOW_DN	1.13	0.53	0	0	0	not significant
11791	GO_COLLAGEN_BINDING	1.13	0.53	0	0	0	not significant
11792	GO_RESPONSE_TO_ELECTRICAL_STIMULUS	1.13	0.53	0	0	0	not significant
11793	GO_RETROGRADE_TRANSPORT_ENDOSOME_TO_PLASMA_MEMBRANE	1.13	0.53	0	0	0	not significant
11794	KYNG_NORMAL_AGING_DN	1.13	0.53	0	0	0	not significant
11795	REACTOME_INSULIN_RECEPTOR_SIGNALLING_CASCADE	1.13	0.53	0	0	0	not significant
11796	GO_COFACTOR_TRANSMEMBRANE_TRANSPORTER_ACTIVITY	1.13	0.52	0	0	0	not significant
11797	GO_ERYTHROCYTE_MATURATION	1.13	0.52	0	0	0	not significant
11798	GO_HISTONE_DEUBIQUITINATION	1.13	0.52	0	0	0	not significant
11799	GO_TRABECULA_MORPHOGENESIS	1.13	0.52	0	0	0	not significant
11800	UZONYI_RESPONSE_TO_LEUKOTRIENE_AND_THROMBIN	1.13	0.52	0	0	0	not significant
11801	BOYLAN_MULTIPLE_MYELOMA_D_CLUSTER_UP	1.13	0.51	0	0	0	not significant
11802	CHR6Q24	1.13	0.51	0	0	0	not significant
11803	GO_NEGATIVE_REGULATION_OF_NEUROTRANSMITTER_TRANSPORT	1.13	0.51	0	0	0	not significant
11804	GO_PROTEIN_LIPID_COMPLEX	1.13	0.51	0	0	0	not significant
11805	GO_ACROSOME_REACTION	1.13	0.50	0	0	0	not significant
11806	GO_CD40_RECEPTOR_COMPLEX	1.13	0.50	0	0	0	not significant
11807	GO_DYNEIN_LIGHT_INTERMEDIATE_CHAIN_BINDING	1.13	0.50	0	0	0	not significant
11808	GO_MONOVALENT_INORGANIC_ANION_HOMEOSTASIS	1.13	0.50	0	0	0	not significant
11809	PID_LYMPH_ANGIOGENESIS_PATHWAY	1.13	0.50	0	0	0	not significant
11810	REACTOME_NEGATIVE_REGULATION_OF_FGFR1_SIGNALING	1.13	0.50	0	0	0	not significant
11811	BIOCARTA_STAT3_PATHWAY	1.13	0.49	0	0	0	not significant
11812	COURTOIS_SENESCENCE_TRIGGERS	1.13	0.49	0	0	0	not significant
11813	NIELSEN_GIST_AND_SYNOVIAL_SARCOMA_UP	1.13	0.49	0	0	0	not significant
11814	GO_NEGATIVE_REGULATION_OF_TELOMERE_CAPPING	1.13	0.48	0	0	0	not significant
11815	GO_SKELETAL_MUSCLE_CELL_PROLIFERATION	1.13	0.48	0	0	0	not significant
11816	GO_REGULATION_OF_PROTEIN_ADP_RIBOSYLATION	1.13	0.47	0	0	0	not significant
11817	BIOCARTA_ASBCELL_PATHWAY	1.13	0.46	0	0	0	not significant
11818	GO_DETECTION_OF_BIOTIC_STIMULUS	1.13	0.46	0	0	0	not significant
11819	GO_NEGATIVE_REGULATION_OF_BONE_REMODELING	1.13	0.46	0	0	0	not significant
11820	REACTOME_GRB2_EVENTS_IN_ERBB2_SIGNALING	1.13	0.46	0	0	0	not significant
11821	GO_MRNA_EDITING_COMPLEX	1.13	0.45	0	0	0	not significant
11822	PALOMERO_GSI_SENSITIVITY_UP	1.13	0.45	0	0	0	not significant
11823	SUZUKI_RESPONSE_TO_TSA_AND_DECITABINE_1A	1.13	0.45	0	0	0	not significant
11824	BUYTAERT_PHOTODYNAMIC_THERAPY_STRESS_UP	1.14	1.01	0	0	0	not significant
11825	GINESTIER_BREAST_CANCER_20Q13_AMPLIFICATION_DN	1.14	0.72	0	0	0	not significant
11826	MIYAGAWA_TARGETS_OF_EWSR1_ETS_FUSIONS_UP	1.14	0.69	0	0	0	not significant
11827	PICCALUGA_ANGIOIMMUNOBLASTIC_LYMPHOMA_DN	1.14	0.67	0	0	0	not significant
11828	CHR16P11	1.14	0.66	0	0	0	not significant
11829	LAIHO_COLORECTAL_CANCER_SERRATED_DN	1.14	0.65	0	0	0	not significant
11830	GO_NEGATIVE_REGULATION_OF_IMMUNE_EFFECTOR_PROCESS	1.14	0.64	0	0	0	not significant
11831	RAO_BOUND_BY_SALL4	1.14	0.64	0	0	0	not significant
11832	HALLMARK_IL6_JAK_STAT3_SIGNALING	1.14	0.63	0	0	0	not significant
11833	FIGUEROA_AML_METHYLATION_CLUSTER_1_UP	1.14	0.62	0	0	0	not significant
11834	HOLLERN_EMT_BREAST_TUMOR_DN	1.14	0.61	0	0	0	not significant
11835	GO_CHEMICAL_SYNAPTIC_TRANSMISSION_POSTSYNAPTIC	1.14	0.60	0	0	0	not significant
11836	REACTOME_TNF_SIGNALING	1.14	0.60	0	0	0	not significant
11837	BREDEMEYER_RAG_SIGNALING_VIA_ATM_NOT_VIA_NFKB_UP	1.14	0.59	0	0	0	not significant
11838	GO_NEGATIVE_REGULATION_OF_LEUKOCYTE_PROLIFERATION	1.14	0.59	0	0	0	not significant
11839	GO_REGULATION_OF_ACTIN_NUCLEATION	1.14	0.58	0	0	0	not significant
11840	REACTOME_CD28_CO_STIMULATION	1.14	0.58	0	0	0	not significant
11841	GO_CLATHRIN_BINDING	1.14	0.57	0	0	0	not significant
11842	GO_DEMETHYLASE_ACTIVITY	1.14	0.57	0	0	0	not significant
11843	GO_REGULATION_OF_ERYTHROCYTE_DIFFERENTIATION	1.14	0.57	0	0	0	not significant
11844	SHETH_LIVER_CANCER_VS_TXNIP_LOSS_PAM6	1.14	0.57	0	0	0	not significant
11845	GO_G2_DNA_DAMAGE_CHECKPOINT	1.14	0.56	0	0	0	not significant
11846	GO_GAMMA_TUBULIN_BINDING	1.14	0.56	0	0	0	not significant
11847	GO_B_CELL_HOMEOSTASIS	1.14	0.55	0	0	0	not significant
11848	GO_POSITIVE_REGULATION_OF_DENDRITE_MORPHOGENESIS	1.14	0.55	0	0	0	not significant
11849	GO_REGULATION_OF_BIOMINERAL_TISSUE_DEVELOPMENT	1.14	0.55	0	0	0	not significant
11850	GO_UBIQUITIN_LIKE_PROTEIN_CONJUGATING_ENZYME_BINDING	1.14	0.55	0	0	0	not significant
11851	GO_FILAMENTOUS_ACTIN	1.14	0.53	0	0	0	not significant
11852	GO_METANEPHRIC_NEPHRON_MORPHOGENESIS	1.14	0.53	0	0	0	not significant
11853	GO_POSITIVE_REGULATION_OF_SMOOTH_MUSCLE_CELL_DIFFERENTIA	1.14	0.53	0	0	0	not significant
11854	GO_REGULATION_OF_TRANSPOSITION	1.14	0.53	0	0	0	not significant
11855	RIZ_ERYTHROID_DIFFERENTIATION_12HR	1.14	0.53	0	0	0	not significant
11856	GO_NEGATIVE_REGULATION_OF_RHO_PROTEIN_SIGNAL_TRANSDUCTIO	1.14	0.52	0	0	0	not significant
11857	GO_AUTOPHAGOSOME_MEMBRANE	1.14	0.51	0	0	0	not significant
11858	GO_CATECHOLAMINE_BIOSYNTHETIC_PROCESS	1.14	0.51	0	0	0	not significant
11859	GO_INFLAMMASOME_COMPLEX	1.14	0.51	0	0	0	not significant
11860	GO_MELANOCYTE_DIFFERENTIATION	1.14	0.51	0	0	0	not significant
11861	GO_POSITIVE_REGULATION_OF_LYMPHOCYTE_APOPTOTIC_PROCESS	1.14	0.51	0	0	0	not significant
11862	MARIADASON_RESPONSE_TO_BUTYRATE_SULINDAC_4	1.14	0.51	0	0	0	not significant
11863	REACTOME_INTERLEUKIN_6_FAMILY_SIGNALING	1.14	0.51	0	0	0	not significant
11864	REACTOME_PURINE_RIBONUCLEOSIDE_MONOPHOSPHATE_BIOSYNTHES	1.14	0.51	0	0	0	not significant
11865	BACOLOD_RESISTANCE_TO_ALKYLATING_AGENTS_UP	1.14	0.50	0	0	0	not significant
11866	GO_PARAXIAL_MESODERM_MORPHOGENESIS	1.14	0.50	0	0	0	not significant
11867	GO_POSITIVE_REGULATION_OF_INTERLEUKIN_1_SECRETION	1.14	0.50	0	0	0	not significant
11868	GO_REGULATION_OF_AMYLOID_BETA_CLEARANCE	1.14	0.50	0	0	0	not significant
11869	GO_REGULATION_OF_TOLERANCE_INDUCTION	1.14	0.50	0	0	0	not significant

11870	REACTOME_NITRIC_OXIDE_STIMULATES_GUANYLATE_CYCLASE	1.14	0.50	0	0	0	not significant
11871	UROSEVIC_RESPONSE_TO_IMIQUIMOD	1.14	0.50	0	0	0	not significant
11872	GO_CELLULAR_RESPONSE_TO_SALT_STRESS	1.14	0.49	0	0	0	not significant
11873	GO_CORPUS_CALLOSUM_MORPHOGENESIS	1.14	0.49	0	0	0	not significant
11874	GO_DNA_CLAMP_LOADER_ACTIVITY	1.14	0.49	0	0	0	not significant
11875	REACTOME_IRAK2_MEDIATED_ACTIVATION_OF_TAK1_COMPLEX	1.14	0.49	0	0	0	not significant
11876	GO_BETA_GALACTOSIDASE_ACTIVITY	1.14	0.48	0	0	0	not significant
11877	GO_MANNOSE_METABOLIC_PROCESS	1.14	0.48	0	0	0	not significant
11878	GO_POLE_PLASM	1.14	0.48	0	0	0	not significant
11879	GO_DNA_ENDOREDUPLICATION	1.14	0.47	0	0	0	not significant
11880	REACTOME_PI_3K_CASCADE:FGFR2	1.14	0.46	0	0	0	not significant
11881	GO_SEQUENCE_SPECIFIC_DNA_BINDING	1.15	0.99	0	0	0	not significant
11882	GO_INTRINSIC_COMPONENT_OF_PLASMA_MEMBRANE	1.15	0.97	0	0	0	not significant
11883	RICKMAN_METASTASIS_UP	1.15	0.87	0	0	0	not significant
11884	GO_T_CELL_ACTIVATION	1.15	0.80	0	0	0	not significant
11885	GO_RECEPTOR_COMPLEX	1.15	0.77	0	0	0	not significant
11886	ACEVEDO_LIVER_TUMOR_VS_NORMAL_ADJACENT_TISSUE_DN	1.15	0.76	0	0	0	not significant
11887	STK33_SKM_UP	1.15	0.74	0	0	0	not significant
11888	COLDREN_GEFITINIB_RESISTANCE_DN	1.15	0.71	0	0	0	not significant
11889	IVANOVA_HEMATOPOIESIS_STEM_CELL_LONG_TERM	1.15	0.71	0	0	0	not significant
11890	BROWNE_HCMV_INFECTION_18HR_DN	1.15	0.69	0	0	0	not significant
11891	GO_MEMBRANE_LIPID_BIOSYNTHETIC_PROCESS	1.15	0.69	0	0	0	not significant
11892	AKT_UP.V1_DN	1.15	0.66	0	0	0	not significant
11893	GO_CELLULAR_TRANSITION_METAL_ION_HOMEOSTASIS	1.15	0.65	0	0	0	not significant
11894	GO_REGULATION_OF_NIK_NF_KAPPAB_SIGNALING	1.15	0.64	0	0	0	not significant
11895	BRUINS_UVC_RESPONSE_MIDDLE	1.15	0.63	0	0	0	not significant
11896	GO_LYSOSOMAL_TRANSPORT	1.15	0.63	0	0	0	not significant
11897	REACTOME_RAB_GERANYLGERANYLATION	1.15	0.61	0	0	0	not significant
11898	CHR13Q12	1.15	0.60	0	0	0	not significant
11899	GO_SOLUTE_CATION_SYMPORTER_ACTIVITY	1.15	0.60	0	0	0	not significant
11900	GO_SYNTAXIN_BINDING	1.15	0.60	0	0	0	not significant
11901	GO_REGULATION_OF_GLUCOSE_METABOLIC_PROCESS	1.15	0.58	0	0	0	not significant
11902	SENESE_HDAC2_TARGETS_DN	1.15	0.58	0	0	0	not significant
11903	CHR2P21	1.15	0.57	0	0	0	not significant
11904	GO_MANNOSYLATION	1.15	0.57	0	0	0	not significant
11905	GO_CYCLIN_DEPENDENT_PROTEIN_SERINE_THREONINE_KINASE_REGUL	1.15	0.56	0	0	0	not significant
11906	GO_PROTEIN_ADP_RIBOSYLASE_ACTIVITY	1.15	0.56	0	0	0	not significant
11907	LIAN_LIPA_TARGETS_3M	1.15	0.56	0	0	0	not significant
11908	BIOCARTA_EPO_PATHWAY	1.15	0.55	0	0	0	not significant
11909	GO_REGULATION_OF_MORPHOGENESIS_OF_A_BRANCHING_STRUCTUR	1.15	0.55	0	0	0	not significant
11910	GO_SOLUTE_SODIUM_SYMPORTER_ACTIVITY	1.15	0.55	0	0	0	not significant
11911	LOPES_METHYLATED_IN_COLON_CANCER_DN	1.15	0.55	0	0	0	not significant
11912	REACTOME_DOWNREGULATION_OF_SMAD2_3:SMAD4_TRANSCRIPTIONA	1.15	0.55	0	0	0	not significant
11913	GO_INTERLEUKIN_6_MEDIATED_SIGNALING_PATHWAY	1.15	0.54	0	0	0	not significant
11914	GO_NEGATIVE_REGULATION_OF_ERAD_PATHWAY	1.15	0.54	0	0	0	not significant
11915	GO_REGULATION_OF_MACROPHAGE_CHEMOTAXIS	1.15	0.54	0	0	0	not significant
11916	GARGALOVIC_RESPONSE_TO_OXIDIZED_PHOSPHOLIPIDS_PINK_DN	1.15	0.53	0	0	0	not significant
11917	GO_GAMMA_TUBULIN_LARGE_COMPLEX	1.15	0.53	0	0	0	not significant
11918	GO_INOSITOL_1_4_5_TRISPHOSPHATE_BINDING	1.15	0.53	0	0	0	not significant
11919	GO_NEGATIVE_REGULATION_OF_BLOOD_VESSEL_ENDOTHELIAL_CELL_	1.15	0.53	0	0	0	not significant
11920	GO_OPSONIN_BINDING	1.15	0.53	0	0	0	not significant
11921	GO_NEGATIVE_REGULATION_OF_CYTOKINE_PRODUCTION_INVOLVED_IN	1.15	0.51	0	0	0	not significant
11922	GO_REGULATION_OF_ISOTYPE_SWITCHING_TO_IGE_ISOTYPES	1.15	0.51	0	0	0	not significant
11923	GO_RETINA_VASCULATURE_DEVELOPMENT_IN_CAMERA_TYPE_EYE	1.15	0.51	0	0	0	not significant
11924	GO_GLYCOSIDE_CATABOLIC_PROCESS	1.15	0.50	0	0	0	not significant
11925	REACTOME_LIPOPHAGY	1.15	0.50	0	0	0	not significant
11926	REACTOME_PI_3K_CASCADE:FGFR4	1.15	0.50	0	0	0	not significant
11927	GO_DEAD_H_BOX_RNA_HELICASE_BINDING	1.15	0.49	0	0	0	not significant
11928	GO_REGULATION_OF_GASTRIC_ACID_SECRETION	1.15	0.49	0	0	0	not significant
11929	GO_UBIQUITIN_LIGASE_INHIBITOR_ACTIVITY	1.15	0.49	0	0	0	not significant
11930	TCGA_GLIOBLASTOMA_MUTATED	1.15	0.49	0	0	0	not significant
11931	GO_DOUBLE_STRANDED_DNA_BINDING	1.16	1.05	0	0	0	not significant
11932	BASAKI_YBX1_TARGETS_DN	1.16	0.84	0	0	0	not significant
11933	GO_SECRETORY_GRANULE_MEMBRANE	1.16	0.77	0	0	0	not significant
11934	GO_STRUCTURAL_CONSTITUENT_OF_RIBOSOME	1.16	0.75	0	0	0	not significant
11935	GO_POSITIVE_REGULATION_OF_PEPTIDE_SECRETION	1.16	0.74	0	0	0	not significant
11936	GO_REGIONALIZATION	1.16	0.74	0	0	0	not significant
11937	HALLMARK_TNFA_SIGNALING_VIA_NFKB	1.16	0.71	0	0	0	not significant
11938	MATZUK_SPERMATOZOA	1.16	0.70	0	0	0	not significant
11939	GO_INTERMEDIATE_FILAMENT_CYTOSKELETON	1.16	0.67	0	0	0	not significant
11940	MCBRYAN_PUBERTAL_BREAST_3_4WK_UP	1.16	0.67	0	0	0	not significant
11941	REACTOME_NUCLEOTIDE_BINDING_DOMAIN_LEUCINE_RICH_REPEAT_CC	1.16	0.66	0	0	0	not significant
11942	CHR17P11	1.16	0.65	0	0	0	not significant
11943	WNT_UP.V1_UP	1.16	0.65	0	0	0	not significant
11944	GARGALOVIC_RESPONSE_TO_OXIDIZED_PHOSPHOLIPIDS_TURQUOISE_U	1.16	0.64	0	0	0	not significant
11945	GO_ADHERENS_JUNCTION_ORGANIZATION	1.16	0.64	0	0	0	not significant
11946	CHR3P22	1.16	0.63	0	0	0	not significant
11947	GO_EMBRYONIC_PATTERN_SPECIFICATION	1.16	0.61	0	0	0	not significant
11948	GO_MESODERM_DEVELOPMENT	1.16	0.61	0	0	0	not significant
11949	GO_NEGATIVE_REGULATION_OF_RESPONSE_TO_BIOTIC_STIMULUS	1.16	0.61	0	0	0	not significant
11950	GO_PHOSPHATIDYLINOSITOL_3_4_BISPHOSPHATE_BINDING	1.16	0.61	0	0	0	not significant
11951	SAMOLS_TARGETS_OF_KHSV_MIRNAS_DN	1.16	0.61	0	0	0	not significant
11952	GO_ACROSOMAL_VESICLE	1.16	0.60	0	0	0	not significant
11953	GO_INTRINSIC_COMPONENT_OF_SYNAPTIC_MEMBRANE	1.16	0.60	0	0	0	not significant
11954	CHR1P21	1.16	0.59	0	0	0	not significant
11955	GO_FICOLIN_1_RICH_GRANULE_MEMBRANE	1.16	0.59	0	0	0	not significant
11956	GO_REGULATION_OF_DOUBLE_STRAND_BREAK_REPAIR_VIA_NONHOMO	1.16	0.59	0	0	0	not significant
11957	GO_NEGATIVE_REGULATION_OF_AXON_EXTENSION	1.16	0.58	0	0	0	not significant
11958	GO_POSITIVE_REGULATION_OF_BIOMINERAL_TISSUE_DEVELOPMENT	1.16	0.58	0	0	0	not significant
11959	GO_REGULATION_OF_NATURAL_KILLER_CELL_MEDIATED_IMMUNITY	1.16	0.58	0	0	0	not significant
11960	GO_RESPONSE_TO_INTERLEUKIN_7	1.16	0.58	0	0	0	not significant
11961	NIKOLSKY_BREAST_CANCER_7P22_AMPLICON	1.16	0.57	0	0	0	not significant
11962	GO_POSITIVE_REGULATION_OF_GLUCOSE_METABOLIC_PROCESS	1.16	0.56	0	0	0	not significant
11963	GO_RESPONSE_TO_MITOCHONDRIAL_DEPOLARISATION	1.16	0.56	0	0	0	not significant
11964	REACTOME_CYTOSOLIC_IRON_SULFUR_CLUSTER_ASSEMBLY	1.16	0.56	0	0	0	not significant
11965	GO_ENTRY_OF_BACTERIUM_INTO_HOST_CELL	1.16	0.53	0	0	0	not significant
11966	GO_PEPTIDYL_SERINE_AUTOPHOSPHORYLATION	1.16	0.53	0	0	0	not significant
11967	GO_REGULATION_OF_EXCITATORY_SYNAPSE_ASSEMBLY	1.16	0.53	0	0	0	not significant
11968	KEGG_GLYCOSPHINGOLIPID_BIOSYNTHESIS_LACTO_AND_NEOLACTO_S	1.16	0.53	0	0	0	not significant
11969	GARGALOVIC_RESPONSE_TO_OXIDIZED_PHOSPHOLIPIDS_LIGHTGREEN	1.16	0.52	0	0	0	not significant
11970	GO_SENSORY_PERCEPTION_OF_BITTER_TASTE	1.16	0.52	0	0	0	not significant
11971	GO_VOLTAGE_GATED_SODIUM_CHANNEL_ACTIVITY	1.16	0.52	0	0	0	not significant
11972	IRITANI_MAD1_TARGETS_UP	1.16	0.52	0	0	0	not significant
11973	KEGG_NON_HOMOLOGOUS_END_JOINING	1.16	0.52	0	0	0	not significant
11974	REACTOME_PYRIMIDINE_CATABOLISM	1.16	0.52	0	0	0	not significant
11975	GO_MRNA_3_END_PROCESSING_BY_STEM_LOOP_BINDING_AND_CLEAV/	1.16	0.51	0	0	0	not significant
11976	GO_REGULATION_OF_PROTEIN_KINASE_A_SIGNALING	1.16	0.51	0	0	0	not significant
11977	GO_REGULATION_OF_TYPE_B_PANCREATIC_CELL_DEVELOPMENT	1.16	0.51	0	0	0	not significant
11978	HOLLEMAN_DAUNORUBICIN_ALL_DN	1.16	0.51	0	0	0	not significant

11979	GO_LIGASE_ACTIVITY_FORMING_PHOSPHORIC_ESTER_BONDS	1.16	0.50	0	0	0	not significant
11980	COULOUARN_TEMPORAL_TGFB1_SIGNATURE_UP	1.17	0.73	0	0	0	not significant
11981	KEGG_JAK_STAT_SIGNALING_PATHWAY	1.17	0.73	0	0	0	not significant
11982	GO_CELLULAR_RESPONSE_TO_INORGANIC_SUBSTANCE	1.17	0.71	0	0	0	not significant
11983	GO_GASTRULATION	1.17	0.71	0	0	0	not significant
11984	LI_INDUCED_T_TO_NATURAL_KILLER_DN	1.17	0.71	0	0	0	not significant
11985	GO_ADULT_BEHAVIOR	1.17	0.69	0	0	0	not significant
11986	GO_REGULATION_OF_DOUBLE_STRAND_BREAK_REPAIR	1.17	0.67	0	0	0	not significant
11987	GO_AMINOGLYCAN_CATABOLIC_PROCESS	1.17	0.64	0	0	0	not significant
11988	GO_NON_MEMBRANE_SPANNING_PROTEIN_TYROSINE_KINASE_ACTIVITY	1.17	0.64	0	0	0	not significant
11989	GO_POST_EMBRYONIC_DEVELOPMENT	1.17	0.63	0	0	0	not significant
11990	YAO_TEMPORAL_RESPONSE_TO_PROGESTERONE_CLUSTER_6	1.17	0.63	0	0	0	not significant
11991	GO_POSITIVE_REGULATION_OF_RECEPTOR_SIGNALING_PATHWAY_VIA	1.17	0.62	0	0	0	not significant
11992	MATZUK_SPERMATID_DIFFERENTIATION	1.17	0.61	0	0	0	not significant
11993	ABBUD_LIF_SIGNALING_1_UP	1.17	0.59	0	0	0	not significant
11994	CHR6P12	1.17	0.59	0	0	0	not significant
11995	GO_ADIPOSE_TISSUE_DEVELOPMENT	1.17	0.59	0	0	0	not significant
11996	GYORFFY_MITOXANTRONE_RESISTANCE	1.17	0.59	0	0	0	not significant
11997	VANHARANTA_UTERINE_FIBROID_UP	1.17	0.59	0	0	0	not significant
11998	CHR4Q22	1.17	0.58	0	0	0	not significant
11999	GO_REGULATION_OF_FATTY_ACID_TRANSPORT	1.17	0.58	0	0	0	not significant
12000	RICKMAN_HEAD_AND_NECK_CANCER_F	1.17	0.58	0	0	0	not significant
12001	GO_CELLULAR_RESPONSE_TO_STEROL	1.17	0.56	0	0	0	not significant
12002	GO_NEGATIVE_REGULATION_OF_CYCLIN_DEPENDENT_PROTEIN_KINASE	1.17	0.56	0	0	0	not significant
12003	GO_PROTEIN_AUTO_ADP_RIBOSYLATION	1.17	0.56	0	0	0	not significant
12004	STEARMAN_TUMOR_FIELD_EFFECT_UP	1.17	0.56	0	0	0	not significant
12005	GO_INOSITOL_TRISPHOSPHATE_KINASE_ACTIVITY	1.17	0.55	0	0	0	not significant
12006	GO_NEGATIVE_REGULATION_OF_AMYLOID_PRECURSOR_PROTEIN_CAT	1.17	0.55	0	0	0	not significant
12007	SCHURINGA_STAT5A_TARGETS_DN	1.17	0.55	0	0	0	not significant
12008	BANDRES_RESPONSE_TO_CARMUSTIN_MGMT_24HR_UP	1.17	0.54	0	0	0	not significant
12009	GO_POSITIVE_REGULATION_OF_HISTONE_H3_K27_METHYLATION	1.17	0.54	0	0	0	not significant
12010	KYNG_WERNER_SYNDROM_DN	1.17	0.54	0	0	0	not significant
12011	MAHADEVAN_IMATINIB_RESISTANCE_DN	1.17	0.54	0	0	0	not significant
12012	NIKOLSKY_BREAST_CANCER_22Q13_AMPLICON	1.17	0.54	0	0	0	not significant
12013	GO_NK_T_CELL_ACTIVATION	1.17	0.53	0	0	0	not significant
12014	GO_PROTEIN_TYROSINE_KINASE_ACTIVATOR_ACTIVITY	1.17	0.53	0	0	0	not significant
12015	BIOCARTA_NEUTROPHIL_PATHWAY	1.17	0.52	0	0	0	not significant
12016	GO_MAINTENANCE_OF_POSTSYNAPTIC_SPECIALIZATION_STRUCTURE	1.17	0.52	0	0	0	not significant
12017	GO_REGULATION_OF_ANION_CHANNEL_ACTIVITY	1.17	0.51	0	0	0	not significant
12018	GO_HMG_BOX_DOMAIN_BINDING	1.17	0.50	0	0	0	not significant
12019	GO_REGULATION_OF_EPITHELIAL_CELL_DIFFERENTIATION_INVOLVED_I	1.17	0.50	0	0	0	not significant
12020	REACTOME_ACYL_CHAIN_REMODELING_OF_CL	1.17	0.50	0	0	0	not significant
12021	HEDVAT_ELF4_TARGETS_UP	1.17	0.48	0	0	0	not significant
12022	GO_NEGATIVE_REGULATION_OF_RNA_BIOSYNTHETIC_PROCESS	1.18	1.40	0	0	0	not significant
12023	RODRIGUES_THYROID_CARCINOMA_POORLY_DIFFERENTIATED_DN	1.18	1.19	0	0	0	not significant
12024	GO_MOLECULAR_TRANSDUCER_ACTIVITY	1.18	1.03	0	0	0	not significant
12025	GO_GLYCOPROTEIN_BIOSYNTHETIC_PROCESS	1.18	0.94	0	0	0	not significant
12026	LINDGREN_BLADDER_CANCER_CLUSTER_3_DN	1.18	0.83	0	0	0	not significant
12027	ZHANG_RESPONSE_TO_IKK_INHIBITOR_AND_TNF_UP	1.18	0.82	0	0	0	not significant
12028	WANG_SMARCE1_TARGETS_UP	1.18	0.76	0	0	0	not significant
12029	PRC2_EED_UP.V1_UP	1.18	0.74	0	0	0	not significant
12030	GO_REGULATION_OF_LYMPHOCYTE_MEDIATED_IMMUNITY	1.18	0.72	0	0	0	not significant
12031	GO_NEURON_SPINE	1.18	0.71	0	0	0	not significant
12032	NABA_CORE_MATRISOME	1.18	0.70	0	0	0	not significant
12033	GO_SODIUM_ION_TRANSMEMBRANE_TRANSPORTER_ACTIVITY	1.18	0.69	0	0	0	not significant
12034	KEGG_T_CELL_RECEPTOR_SIGNALING_PATHWAY	1.18	0.69	0	0	0	not significant
12035	BORCZUK_MALIGNANT_MESOTHELIOMA_DN	1.18	0.68	0	0	0	not significant
12036	GO_NEURON_PROJECTION_TERMINUS	1.18	0.68	0	0	0	not significant
12037	CHR2Q33	1.18	0.64	0	0	0	not significant
12038	GO_PRESYNAPTIC_ACTIVE_ZONE_MEMBRANE	1.18	0.63	0	0	0	not significant
12039	GO_ADENYLATE_CYCLASE_INHIBITING_G_PROTEIN_COUPLED_RECEPTC	1.18	0.62	0	0	0	not significant
12040	REACTOME_CD209_DC_SIGN_SIGNALING	1.18	0.61	0	0	0	not significant
12041	TAKEDA_TARGETS_OF_NUP98_HOXA9_FUSION_6HR_UP	1.18	0.61	0	0	0	not significant
12042	GO_REGULATION_OF_ENDOCRINE_PROCESS	1.18	0.60	0	0	0	not significant
12043	GO_REGULATION_OF_NON_CANONICAL_WNT_SIGNALING_PATHWAY	1.18	0.59	0	0	0	not significant
12044	LEIN_LOCALIZED_TO_DISTAL_AND_PROXIMAL_DENDRITES	1.18	0.59	0	0	0	not significant
12045	GO_BICARBONATE_TRANSMEMBRANE_TRANSPORTER_ACTIVITY	1.18	0.58	0	0	0	not significant
12046	GO_INTERLEUKIN_27_MEDIATED_SIGNALING_PATHWAY	1.18	0.58	0	0	0	not significant
12047	GO_NEGATIVE_REGULATION_OF_RECEPTOR_SIGNALING_PATHWAY_VIA	1.18	0.58	0	0	0	not significant
12048	REACTOME_P75NTR_RECRUITS_SIGNALLING_COMPLEXES	1.18	0.58	0	0	0	not significant
12049	GO_AMP_BIOSYNTHETIC_PROCESS	1.18	0.57	0	0	0	not significant
12050	GO_MAP_KINASE_ACTIVITY	1.18	0.57	0	0	0	not significant
12051	MALIK_REPRESSED_BY_ESTROGEN	1.18	0.57	0	0	0	not significant
12052	CAIRO_PML_TARGETS_BOUND_BY_MYC_DN	1.18	0.56	0	0	0	not significant
12053	GO_EPITHELIAL_CELL_CELL_ADHESION	1.18	0.56	0	0	0	not significant
12054	GO_REGULATION_OF_MESODERM_DEVELOPMENT	1.18	0.56	0	0	0	not significant
12055	GO_REGULATION_OF_STEM_CELL_POPULATION_MAINTENANCE	1.18	0.56	0	0	0	not significant
12056	GO_STRUCTURAL_CONSTITUENT_OF_SYNAPSE	1.18	0.56	0	0	0	not significant
12057	REACTOME_TNFR1_INDUCED_PROAPOPTOTIC_SIGNALING	1.18	0.56	0	0	0	not significant
12058	GO_RESPONSE_TO_CAMPTOTHECIN	1.18	0.54	0	0	0	not significant
12059	REACTOME_REACTIONS_SPECIFIC_TO_THE_COMPLEX_N_GLYCAN_SYN	1.18	0.54	0	0	0	not significant
12060	GO_MIRNA_LOADING_ONTO_RISC_INVOLVED_IN_GENE_SILENCING_BY_M	1.18	0.53	0	0	0	not significant
12061	SHIN_B_CELL_LYMPHOMA_CLUSTER_1	1.18	0.53	0	0	0	not significant
12062	GO_SEQUENCE_SPECIFIC_DOUBLE_STRANDED_DNA_BINDING	1.19	1.20	0	0	0	not significant
12063	BENPORATH_PRC2_TARGETS	1.19	0.91	0	0	0	not significant
12064	GO_SYNAPTIC_MEMBRANE	1.19	0.91	0	0	0	not significant
12065	KOYAMA_SEMA3B_TARGETS_UP	1.19	0.87	0	0	0	not significant
12066	LEE_RECENT_THYMIC_EMIGRANT	1.19	0.85	0	0	0	not significant
12067	ROME_INSULIN_TARGETS_IN_MUSCLE_DN	1.19	0.83	0	0	0	not significant
12068	GO_REGULATION_OF_LYMPHOCYTE_DIFFERENTIATION	1.19	0.82	0	0	0	not significant
12069	GO_REGULATION_OF_RAS_PROTEIN_SIGNAL_TRANSDUCTION	1.19	0.82	0	0	0	not significant
12070	PEDRIOLI_MIR31_TARGETS_UP	1.19	0.78	0	0	0	not significant
12071	GO_CYCLIC_NUCLEOTIDE_MEDIATED_SIGNALING	1.19	0.76	0	0	0	not significant
12072	KIM_MYCN_AMPLIFICATION_TARGETS_DN	1.19	0.76	0	0	0	not significant
12073	GO_MAINTENANCE_OF_PROTEIN_LOCATION	1.19	0.75	0	0	0	not significant
12074	BRCA1_DN.V1_DN	1.19	0.73	0	0	0	not significant
12075	GO_EPITHELIAL_CELL_APOPTOTIC_PROCESS	1.19	0.71	0	0	0	not significant
12076	GO_HOMOLOGOUS_RECOMBINATION	1.19	0.70	0	0	0	not significant
12077	KEGG_ERBB_SIGNALING_PATHWAY	1.19	0.70	0	0	0	not significant
12078	REACTOME_SIGNALING_BY_INSULIN_RECEPTOR	1.19	0.70	0	0	0	not significant
12079	BIOCARTA_ARAP_PATHWAY	1.19	0.67	0	0	0	not significant
12080	CHR10P11	1.19	0.67	0	0	0	not significant
12081	GO_MESODERM_MORPHOGENESIS	1.19	0.67	0	0	0	not significant
12082	GO_ROUGH_ENDOPLASMIC_RETICULUM_MEMBRANE	1.19	0.66	0	0	0	not significant
12083	REACTOME_REGULATION_OF_TNFR1_SIGNALING	1.19	0.66	0	0	0	not significant
12084	MORI_PLASMA_CELL_DN	1.19	0.64	0	0	0	not significant
12085	BIOCARTA_TOB1_PATHWAY	1.19	0.62	0	0	0	not significant
12086	GO_POSITIVE_REGULATION_OF_ACTIN_NUCLEATION	1.19	0.61	0	0	0	not significant
12087	GO_REGULATION_OF_MEMBRANE_INVAGINATION	1.19	0.60	0	0	0	not significant

12088	GO_REGULATION_OF_RECEPTOR_LOCALIZATION_TO_SYNAPSE	1.19	0.60	0	0	0	not significant
12089	HANN_RESISTANCE_TO_BCL2_INHIBITOR_UP	1.19	0.60	0	0	0	not significant
12090	GO_DENTATE_GYRUS_DEVELOPMENT	1.19	0.59	0	0	0	not significant
12091	GO_REGULATION_OF_MODIFICATION_OF_SYNAPTIC_STRUCTURE	1.19	0.59	0	0	0	not significant
12092	KEGG_ALLOGRAFT_REJECTION	1.19	0.59	0	0	0	not significant
12093	REACTOME_SIGNALING_BY_ACTIVIN	1.19	0.59	0	0	0	not significant
12094	GO_NEGATIVE_REGULATION_OF_INTERFERON_BETA_PRODUCTION	1.19	0.58	0	0	0	not significant
12095	GO POSITIVE REGULATION OF CYTOPLASMIC MRNA PROCESSING BC	1.19	0.57	0	0	0	not significant
12096	GO POSITIVE REGULATION OF TUMOR NECROSIS FACTOR MEDIATEI	1.19	0.57	0	0	0	not significant
12097	GO_WATER_TRANSPORT	1.19	0.57	0	0	0	not significant
12098	ABBUD_LIF_SIGNALING_2_UP	1.19	0.56	0	0	0	not significant
12099	GO_NEGATIVE_REGULATION_OF_LEUKOCYTE_CHEMOTAXIS	1.19	0.56	0	0	0	not significant
12100	ZHENG_FOXP3_TARGETS_DN	1.19	0.56	0	0	0	not significant
12101	GO_DETECTION_OF_VIRUS	1.19	0.55	0	0	0	not significant
12102	GO POSITIVE REGULATION OF ACTIVIN RECEPTOR SIGNALING PATHW	1.19	0.55	0	0	0	not significant
12103	GO POSITIVE REGULATION OF NUCLEAR CELL CYCLE DNA REPLICAT	1.19	0.55	0	0	0	not significant
12104	GO_REGULATION_OF_CYTOPLASMIC_MRNA_PROCESSING_BODY_ASSEI	1.19	0.55	0	0	0	not significant
12105	BIOCARTA_DICER_PATHWAY	1.19	0.54	0	0	0	not significant
12106	GO_NEGATIVE_REGULATION_OF_CYTOKINESIS	1.19	0.54	0	0	0	not significant
12107	REACTOME_ERYTHROPOIETIN_ACTIVATES_PHOSPHOLIPASE_C_GAMMA	1.19	0.54	0	0	0	not significant
12108	GO_POSITIVE_REGULATION_OF_PROTEIN_HOMOOLIGOMERIZATION	1.19	0.53	0	0	0	not significant
12109	GO_THYROID_HORMONE_METABOLIC_PROCESS	1.19	0.53	0	0	0	not significant
12110	GO_CYTOKINE_PRODUCTION	1.20	1.20	0	0	0	not significant
12111	GO_PROTEIN_TARGETING	1.20	1.16	0	0	0	not significant
12112	GO_POSTSYNAPSE	1.20	1.04	0	0	0	not significant
12113	CHANDRAN_METASTASIS_DN	1.20	0.92	0	0	0	not significant
12114	GO_GLYCEROLIPID_METABOLIC_PROCESS	1.20	0.91	0	0	0	not significant
12115	PASQUALUCCI_LYMPHOMA_BY_GC_STAGE_DN	1.20	0.87	0	0	0	not significant
12116	GO_RHO_GTPASE_BINDING	1.20	0.86	0	0	0	not significant
12117	GO_GATED_CHANNEL_ACTIVITY	1.20	0.85	0	0	0	not significant
12118	PEREZ_TP53_AND_TP63_TARGETS	1.20	0.82	0	0	0	not significant
12119	GO_REGULATION_OF_ADAPTIVE_IMMUNE_RESPONSE	1.20	0.81	0	0	0	not significant
12120	GO_DEFENSE_RESPONSE_TO_BACTERIUM	1.20	0.80	0	0	0	not significant
12121	MIKKELSEN_MCV6_HCP_WITH_H3K27ME3	1.20	0.80	0	0	0	not significant
12122	BENPORATH_ES_CORE_NINE_CORRELATED	1.20	0.79	0	0	0	not significant
12123	CHR16Q24	1.20	0.79	0	0	0	not significant
12124	GO_INTRINSIC_APOPTOTIC_SIGNALING_PATHWAY_IN_RESPONSE_TO_DI	1.20	0.79	0	0	0	not significant
12125	IVANOVA_HEMATOPOIESIS_STEM_CELL	1.20	0.77	0	0	0	not significant
12126	REACTOME_PI3K_AKT_SIGNALING_IN_CANCER	1.20	0.76	0	0	0	not significant
12127	GO_GLYCOSAMINOGLYCAN_BINDING	1.20	0.75	0	0	0	not significant
12128	GO_MAINTENANCE_OF_PROTEIN_LOCATION_IN_CELL	1.20	0.72	0	0	0	not significant
12129	GO_PROTEIN_KINASE_C_BINDING	1.20	0.72	0	0	0	not significant
12130	YAGI_AML_RELAPSE_PROGNOSIS	1.20	0.69	0	0	0	not significant
12131	ZHAN_MULTIPLE_MYELOMA_PR_DN	1.20	0.66	0	0	0	not significant
12132	GO_ACTIVATED_T_CELL_PROLIFERATION	1.20	0.64	0	0	0	not significant
12133	GO_GRANULOCYTE_DIFFERENTIATION	1.20	0.64	0	0	0	not significant
12134	GO_REGULATION_OF_ACUTE_INFLAMMATORY_RESPONSE	1.20	0.64	0	0	0	not significant
12135	GO_POSITIVE_REGULATION_OF_ERYTHROCYTE_DIFFERENTIATION	1.20	0.63	0	0	0	not significant
12136	KEGG_AUTOIMMUNE_THYROID_DISEASE	1.20	0.63	0	0	0	not significant
12137	PID_SYNDECAN_3_PATHWAY	1.20	0.61	0	0	0	not significant
12138	XU_RESPONSE_TO_TRETINOIN_UP	1.20	0.61	0	0	0	not significant
12139	GO REGULATION OF MYD88 INDEPENDENT TOLL LIKE RECEPTOR SI	1.20	0.60	0	0	0	not significant
12140	GO_TRIGLYCERIDE_METABOLIC_PROCESS	1.20	0.60	0	0	0	not significant
12141	HOLLERN_SOLID_NODULAR_BREAST_TUMOR_DN	1.20	0.60	0	0	0	not significant
12142	COWLING_MYCN_TARGETS	1.20	0.59	0	0	0	not significant
12143	GO_PYRIMIDINE_NUCLEOBASE_METABOLIC_PROCESS	1.20	0.59	0	0	0	not significant
12144	GO_REGULATION_OF_POSTSYNAPTIC_DENSITY_ORGANIZATION	1.20	0.59	0	0	0	not significant
12145	REACTOME_WNT_MEDIATED_ACTIVATION_OF_DVL	1.20	0.59	0	0	0	not significant
12146	RORIE_TARGETS_OF_EWSR1_FLI1_FUSION_DN	1.20	0.59	0	0	0	not significant
12147	BOYAULT_LIVER_CANCER_SUBCLASS_G56_UP	1.20	0.58	0	0	0	not significant
12148	GO_DNA_INTEGRATION	1.20	0.58	0	0	0	not significant
12149	GO_HISTONE_DEMETHYLASE_ACTIVITY_H3_K9_SPECIFIC_	1.20	0.58	0	0	0	not significant
12150	GO_MALE_SEX_DETERMINATION	1.20	0.58	0	0	0	not significant
12151	GO_POSITIVE_REGULATION_OF_DNA_LIGATION	1.20	0.58	0	0	0	not significant
12152	GO CHANGES TO DNA METHYLATION INVOLVED IN EMBRYO DEVELO	1.20	0.57	0	0	0	not significant
12153	GO_SPHINGOLIPID_TRANSPORTER_ACTIVITY	1.20	0.57	0	0	0	not significant
12154	GO_HIGH_VOLTAGE_GATED_CALCIUM_CHANNEL_ACTIVITY	1.20	0.56	0	0	0	not significant
12155	GO_VOLTAGE_GATED_SODIUM_CHANNEL_COMPLEX	1.20	0.56	0	0	0	not significant
12156	GO_BONE_TRABECULA_MORPHOGENESIS	1.20	0.53	0	0	0	not significant
12157	DUTERTRE_ESTRADIOL_RESPONSE_24HR_DN	1.21	1.26	0	0	0	not significant
12158	WANG_LMO4_TARGETS_UP	1.21	1.08	0	0	0	not significant
12159	GO_MALE_GAMETE_GENERATION	1.21	1.06	0	0	0	not significant
12160	REACTOME_SIGNALING_BY_ROBO_RECEPTORS	1.21	1.01	0	0	0	not significant
12161	GO_NEURAL_TUBE_DEVELOPMENT	1.21	0.90	0	0	0	not significant
12162	MCBRYAN_PUBERTAL_BREAST_4_5WK_UP	1.21	0.90	0	0	0	not significant
12163	DELYS_THYROID_CANCER_DN	1.21	0.88	0	0	0	not significant
12164	SHEDDEN_LUNG_CANCER_GOOD_SURVIVAL_A4	1.21	0.87	0	0	0	not significant
12165	KRAS.600_UP.V1_UP	1.21	0.82	0	0	0	not significant
12166	GO_APICAL_JUNCTION_COMPLEX	1.21	0.81	0	0	0	not significant
12167	GO TUMOR NECROSIS FACTOR SUPERFAMILY CYTOKINE PRODUCTIC	1.21	0.79	0	0	0	not significant
12168	NIKOLSKY_BREAST_CANCER_16Q24_AMPLICON	1.21	0.76	0	0	0	not significant
12169	YAUCH_HEDGEHOG_SIGNALING_PARACRINE_UP	1.21	0.76	0	0	0	not significant
12170	GO_REGULATION_OF_TUMOR_NECROSIS_FACTOR_MEDIATED_SIGNALII	1.21	0.75	0	0	0	not significant
12171	GO_PROTEOGLYCAN_METABOLIC_PROCESS	1.21	0.73	0	0	0	not significant
12172	KEGG_CELL_ADHESION_MOLECULES_CAMS	1.21	0.73	0	0	0	not significant
12173	GO_CELLULAR_RESPONSE_TO_CALCIUM_ION	1.21	0.72	0	0	0	not significant
12174	MARSON_FOXP3_TARGETS_DN	1.21	0.72	0	0	0	not significant
12175	BOYLAN_MULTIPLE_MYELOMA_D_CLUSTER_DN	1.21	0.70	0	0	0	not significant
12176	GO_ANTIMICROBIAL_HUMORAL_RESPONSE	1.21	0.70	0	0	0	not significant
12177	FONTAINE_PAPILLARY_THYROID_CARCINOMA_DN	1.21	0.69	0	0	0	not significant
12178	GO_GPI_ANCHOR_BIOSYNTHETIC_PROCESS	1.21	0.68	0	0	0	not significant
12179	GO_IONOTROPIC_GLUTAMATE_RECEPTOR_BINDING	1.21	0.68	0	0	0	not significant
12180	GO_STORE_OPERATED_CALCIUM_ENTRY	1.21	0.68	0	0	0	not significant
12181	DAIRKEE_CANCER_PRONE_RESPONSE_E2	1.21	0.67	0	0	0	not significant
12182	STREICHER_LSM1_TARGETS_UP	1.21	0.66	0	0	0	not significant
12183	GO_T_CELL_SELECTION	1.21	0.65	0	0	0	not significant
12184	GARGALOVIC RESPONSE TO OXIDIZED PHOSPHOLIPIDS LIGHTYELLOW	1.21	0.64	0	0	0	not significant
12185	GO_REGULATION_OF_DEFENSE_RESPONSE_TO_BACTERIUM	1.21	0.64	0	0	0	not significant
12186	LE_NEURONAL_DIFFERENTIATION_UP	1.21	0.64	0	0	0	not significant
12187	VALK_AML_CLUSTER_10	1.21	0.64	0	0	0	not significant
12188	GO POSITIVE REGULATION OF TUMOR NECROSIS FACTOR SECRETIC	1.21	0.63	0	0	0	not significant
12189	GO_REGULATION_OF_SYNAPTIC_VESICLE_ENDOCYTOSIS	1.21	0.63	0	0	0	not significant
12190	MARSHALL_VIRAL_INFECTION_RESPONSE_DN	1.21	0.63	0	0	0	not significant
12191	REACTOME NOTCH2 ACTIVATION AND TRANSMISSION OF SIGNAL TO	1.21	0.63	0	0	0	not significant
12192	GO_FANCONI_ANAEMIA_NUCLEAR_COMPLEX	1.21	0.62	0	0	0	not significant
12193	GO_IMMATURE_B_CELL_DIFFERENTIATION	1.21	0.62	0	0	0	not significant
12194	GO_NUCLEOTIDE_RECEPTOR_ACTIVITY	1.21	0.62	0	0	0	not significant
12195	GO_NEGATIVE_REGULATION_OF_ANIMAL_ORGAN_MORPHOGENESIS	1.21	0.61	0	0	0	not significant
12196	GO_VOLTAGE_GATED_CHLORIDE_CHANNEL_ACTIVITY	1.21	0.61	0	0	0	not significant

12197	PID_HEDGEHOG_2PATHWAY	1.21	0.61	0	0	0	not significant
12198	REACTOME_PYRIMIDINE_SALVAGE	1.21	0.61	0	0	0	not significant
12199	SEKI_INFLAMMATORY_RESPONSE_LPS_DN	1.21	0.60	0	0	0	not significant
12200	MATZUK_POST-IMPLANTATION_AND_POST-PARTUM	1.21	0.59	0	0	0	not significant
12201	PID_WNT_SIGNALING_PATHWAY	1.21	0.59	0	0	0	not significant
12202	GO_CALCIUM_ION_REGULATED_EXOCYTOSIS_OF_NEUROTRANSMITTER	1.21	0.58	0	0	0	not significant
12203	GO_INTERLEUKIN_10_SECRETION	1.21	0.58	0	0	0	not significant
12204	GO_ORGAN_INDUCTION	1.21	0.58	0	0	0	not significant
12205	WANG_NEOPLASTIC_TRANSFORMATION_BY_CCND1_MYC	1.21	0.58	0	0	0	not significant
12206	REACTOME_INTERLEUKIN_27_SIGNALING	1.21	0.57	0	0	0	not significant
12207	SHI_SPARC_TARGETS_DN	1.21	0.57	0	0	0	not significant
12208	GO_SPECTRIN_ASSOCIATED_CYTOSKELETON	1.21	0.56	0	0	0	not significant
12209	MARSHALL_VIRAL_INFECTION_RESPONSE_UP	1.21	0.56	0	0	0	not significant
12210	MYLLYKANGAS_AMPLIFICATION_HOT_SPOT_7	1.21	0.56	0	0	0	not significant
12211	GO_SEQUESTERING_OF_EXTRACELLULAR_LIGAND_FROM_RECEPTOR	1.21	0.54	0	0	0	not significant
12212	GO_REGULATORY_REGION_NUCLEIC_ACID_BINDING	1.22	1.38	0	0	0	not significant
12213	GO_TRANSMEMBRANE_SIGNALING_RECEPTOR_ACTIVITY	1.22	1.21	0	0	0	not significant
12214	CHARAFE_BREAST_CANCER_LUMINAL_VS_MESENCHYMAL_UP	1.22	1.12	0	0	0	not significant
12215	WANG_CLIM2_TARGETS_UP	1.22	1.07	0	0	0	not significant
12216	LINDGREN_BLADDER_CANCER_CLUSTER_2A_DN	1.22	0.98	0	0	0	not significant
12217	GO_DISTAL_AXON	1.22	0.97	0	0	0	not significant
12218	PID_SMAD2_3NUCLEAR_PATHWAY	1.22	0.88	0	0	0	not significant
12219	BOYAULT_LIVER_CANCER_SUBCLASS_G1_UP	1.22	0.86	0	0	0	not significant
12220	GO_REGULATION_OF_DNA_RECOMBINATION	1.22	0.86	0	0	0	not significant
12221	GO_CYTOKINE_ACTIVITY	1.22	0.81	0	0	0	not significant
12222	CRX_NRL_DN.V1_DN	1.22	0.80	0	0	0	not significant
12223	GO_PHAGOSOME_MATURATION	1.22	0.77	0	0	0	not significant
12224	GO_CELLULAR_COMPONENT_MAINTENANCE	1.22	0.73	0	0	0	not significant
12225	GO_NATURAL_KILLER_CELL_ACTIVATION	1.22	0.73	0	0	0	not significant
12226	LINDSTEDT_DENDRITIC_CELL_MATURATION_B	1.22	0.72	0	0	0	not significant
12227	BIOCARTA_GLEEVEC_PATHWAY	1.22	0.71	0	0	0	not significant
12228	GO_INTERLEUKIN_8_PRODUCTION	1.22	0.70	0	0	0	not significant
12229	REACTOME_NICOTINATE_METABOLISM	1.22	0.70	0	0	0	not significant
12230	MURATA_VIRULENCE_OF_H_PILORI	1.22	0.68	0	0	0	not significant
12231	GO_ENDOCRINE_HORMONE_SECRETION	1.22	0.67	0	0	0	not significant
12232	BANDRES_RESPONSE_TO_CARMUSTIN_WITHOUT_MGMT_24HR_UP	1.22	0.65	0	0	0	not significant
12233	CHR7P12	1.22	0.65	0	0	0	not significant
12234	GO_EPITHELIAL_CILIUM_MOVEMENT	1.22	0.65	0	0	0	not significant
12235	GO_CLATHRIN_SCULPTED_GAMMA_AMINOBUTYRIC_ACID_TRANSPORT_	1.22	0.64	0	0	0	not significant
12236	GO_MANNOSYL_OLIGOSACCHARIDE_MANNOSIDASE_ACTIVITY	1.22	0.64	0	0	0	not significant
12237	GO_GLYCOSPHINGOLIPID_BIOSYNTHETIC_PROCESS	1.22	0.63	0	0	0	not significant
12238	GO_NEGATIVE_REGULATION_OF_GLIAL_CELL_DIFFERENTIATION	1.22	0.63	0	0	0	not significant
12239	GO_NUCLEAR_MEMBRANE_PART	1.22	0.63	0	0	0	not significant
12240	GO_DETECTION_OF_EXTERNAL_BIOTIC_STIMULUS	1.22	0.62	0	0	0	not significant
12241	GO_GASTRIC_ACID_SECRETION	1.22	0.62	0	0	0	not significant
12242	GO_IRON_COORDINATION_ENTITY_TRANSPORT	1.22	0.62	0	0	0	not significant
12243	GO_NEGATIVE_REGULATION_OF_OLIGODENDROCYTE_DIFFERENTIATIO	1.22	0.62	0	0	0	not significant
12244	BIOCARTA_CIRCADIAN_PATHWAY	1.22	0.61	0	0	0	not significant
12245	GO_INTERMEMBRANE_LIPID_TRANSFER	1.22	0.61	0	0	0	not significant
12246	GO_MAMMARY_GLAND_EPITHELIAL_CELL_DIFFERENTIATION	1.22	0.61	0	0	0	not significant
12247	GO_NEURAL_PLATE_REGIONALIZATION	1.22	0.61	0	0	0	not significant
12248	GRANDVAUX_IFN_RESPONSE_NOT_VIA_IRF3	1.22	0.61	0	0	0	not significant
12249	MATZUK_PREOVULATORY_FOLLICLE	1.22	0.61	0	0	0	not significant
12250	TURJANSKI_MAPK1_AND_MAPK2_TARGETS	1.22	0.61	0	0	0	not significant
12251	GO_BERGMANN_GLIAL_CELL_DIFFERENTIATION	1.22	0.60	0	0	0	not significant
12252	GO_PROTEIN_UFMYLATION	1.22	0.60	0	0	0	not significant
12253	REACTOME_THE_ACTIVATION_OF_ARYLSULFATASES	1.22	0.59	0	0	0	not significant
12254	ZIRN_TRETINOIN_RESPONSE_WT1_DN	1.22	0.59	0	0	0	not significant
12255	GO_PHOTORECEPTOR_CELL_OUTER_SEGMENT_ORGANIZATION	1.22	0.56	0	0	0	not significant
12256	ZWANG_TRANSIENTLY_UP_BY_1ST_EGF_PULSE_ONLY	1.23	2.01	0	0	0	not significant
12257	GO_TRANSFERASE_ACTIVITY_TRANSFERRING_GLYCOSYL_GROUPS	1.23	1.04	0	0	0	not significant
12258	GO_REGULATION_OF_SIGNALING_RECEPTOR_ACTIVITY	1.23	1.01	0	0	0	not significant
12259	GO_APPENDAGE_DEVELOPMENT	1.23	0.89	0	0	0	not significant
12260	GO_ENDOTHELIAL_CELL_PROLIFERATION	1.23	0.84	0	0	0	not significant
12261	PEDERSEN_TARGETS_OF_611CTF_ISOFORM_OF_ERBB2	1.23	0.81	0	0	0	not significant
12262	GO_AMINOGLYCAN_BIOSYNTHETIC_PROCESS	1.23	0.80	0	0	0	not significant
12263	KEGG_CHRONIC_MYELOID_LEUKEMIA	1.23	0.80	0	0	0	not significant
12264	GO_POSITIVE_REGULATION_OF_INTERFERON_GAMMA_PRODUCTION	1.23	0.78	0	0	0	not significant
12265	GO_VOLTAGE_GATED_CATION_CHANNEL_ACTIVITY	1.23	0.75	0	0	0	not significant
12266	WINNEPENNINCKX_MELANOMA_METASTASIS_DN	1.23	0.75	0	0	0	not significant
12267	GO_POSITIVE_REGULATION_OF_BONE_MINERALIZATION	1.23	0.72	0	0	0	not significant
12268	REACTOME_SIALIC_ACID_METABOLISM	1.23	0.72	0	0	0	not significant
12269	BIOCARTA_TPO_PATHWAY	1.23	0.70	0	0	0	not significant
12270	GO_LEFT_RIGHT_PATTERN_FORMATION	1.23	0.70	0	0	0	not significant
12271	LIEN_BREAST_CARCINOMA_METAPLASTIC_VS_DUCTAL_DN	1.23	0.70	0	0	0	not significant
12272	PID_IL2_STAT5_PATHWAY	1.23	0.70	0	0	0	not significant
12273	BERTUCCI_INVASIVE_CARCINOMA_DUCTAL_VS_LOBULAR_DN	1.23	0.69	0	0	0	not significant
12274	GO_CAMP_RESPONSE_ELEMENT_BINDING	1.23	0.69	0	0	0	not significant
12275	HOLLEMAN_VINCRISTINE_RESISTANCE_ALL_UP	1.23	0.69	0	0	0	not significant
12276	GO_TRANSCRIPTION_COREPRESSOR_BINDING	1.23	0.68	0	0	0	not significant
12277	HATADA_METHYLATED_IN_LUNG_CANCER_DN	1.23	0.68	0	0	0	not significant
12278	REACTOME_RHO_GTPASES_ACTIVATE_RHOTEKIN_AND_RHOPHILINS	1.23	0.67	0	0	0	not significant
12279	WESTON_VEGFA_TARGETS_6HR	1.23	0.67	0	0	0	not significant
12280	GO_REGULATION_OF_GLUTAMATE_RECEPTOR_SIGNALING_PATHWAY	1.23	0.66	0	0	0	not significant
12281	GO_SMAD_PROTEIN_COMPLEX	1.23	0.66	0	0	0	not significant
12282	GO_ACROSOMAL_MEMBRANE	1.23	0.65	0	0	0	not significant
12283	BIOCARTA_SHH_PATHWAY	1.23	0.64	0	0	0	not significant
12284	GO_ARP2_3_COMPLEX_BINDING	1.23	0.64	0	0	0	not significant
12285	GO_MALE_MEIOSIS_I	1.23	0.64	0	0	0	not significant
12286	GO_CYCLIC_NUCLEOTIDE_PHOSPHODIESTERASE_ACTIVITY	1.23	0.63	0	0	0	not significant
12287	GO_CELLULAR_RESPONSE_TO_CHOLESTEROL	1.23	0.62	0	0	0	not significant
12288	GO_FOUR_WAY_JUNCTION_HELICASE_ACTIVITY	1.23	0.61	0	0	0	not significant
12289	REACTOME_FRS_MEDIATED_FGFR1_SIGNALING	1.23	0.61	0	0	0	not significant
12290	REACTOME_TFAP2_AP_2_FAMILY_REGULATES_TRANSCRIPTION_OF_GF	1.23	0.61	0	0	0	not significant
12291	GO_ESTABLISHMENT_OF_PROTEIN_LOCALIZATION_TO_POSTSYNAPTIC_	1.23	0.59	0	0	0	not significant
12292	GARY_CD5_TARGETS_UP	1.24	1.49	0	0	0	not significant
12293	GO_LEUKOCYTE_DIFFERENTIATION	1.24	1.33	0	0	0	not significant
12294	PENG_RAPAMYCIN_RESPONSE_UP	1.24	1.00	0	0	0	not significant
12295	GAVIN_FOXP3_TARGETS_CLUSTER_P4	1.24	0.84	0	0	0	not significant
12296	GO_REGULATION_OF_BONE_MINERALIZATION	1.24	0.79	0	0	0	not significant
12297	HOLLEMAN_VINCRISTINE_RESISTANCE_B_ALL_UP	1.24	0.79	0	0	0	not significant
12298	GO_DETERMINATION_OF_HEART_LEFT_RIGHT_ASYMMETRY	1.24	0.78	0	0	0	not significant
12299	VECCHI_GASTRIC_CANCER_ADVANCED_VS_EARLY_UP	1.24	0.78	0	0	0	not significant
12300	GO_MEMBRANE_LIPID_CATABOLIC_PROCESS	1.24	0.75	0	0	0	not significant
12301	BIOCARTA_G1_PATHWAY	1.24	0.74	0	0	0	not significant
12302	GO_INFLAMMATORY_RESPONSE_TO_ANTIGENIC_STIMULUS	1.24	0.74	0	0	0	not significant
12303	YANG_BREAST_CANCER_ESR1_LASER_UP	1.24	0.73	0	0	0	not significant
12304	GO_MAINTENANCE_OF_PROTEIN_LOCATION_IN_NUCLEUS	1.24	0.72	0	0	0	not significant
12305	GO_NAD_BIOSYNTHETIC_PROCESS	1.24	0.71	0	0	0	not significant

12306	MIKKELSEN_ES_ICP_WITH_H3K4ME3_AND_H3K27ME3	1.24	0.70	0	0	0	not significant
12307	GO_HEPARAN_SULFATE_SULFOTRANSFERASE_ACTIVITY	1.24	0.69	0	0	0	not significant
12308	REACTOME_ERYTHROPOIETIN_ACTIVATES_PHOSPHOINOSITIDE_3_KINAS	1.24	0.69	0	0	0	not significant
12309	GO_MITOTIC_DNA_REPLICATION	1.24	0.67	0	0	0	not significant
12310	GO_PHOSPHORYLASE_KINASE_COMPLEX	1.24	0.67	0	0	0	not significant
12311	GO_REGULATION_OF_DNA_DIRECTED_DNA_POLYMERASE_ACTIVITY	1.24	0.67	0	0	0	not significant
12312	VALK_AML_CLUSTER_2	1.24	0.67	0	0	0	not significant
12313	CHR4Q26	1.24	0.66	0	0	0	not significant
12314	BAKER_HEMATOPOESIS_STAT5_TARGETS	1.24	0.65	0	0	0	not significant
12315	GO_INTRINSIC_COMPONENT_OF_POSTSYNAPTIC_DENSITY_MEMBRANE	1.24	0.65	0	0	0	not significant
12316	GO_MESODERMAL_CELL_DIFFERENTIATION	1.24	0.65	0	0	0	not significant
12317	GO_TRANSMEMBRANE_RECEPTOR_PROTEIN_SERINE_THREONINE_KINAS	1.24	0.65	0	0	0	not significant
12318	GO_RESPONSE_TO_CISPLATIN	1.24	0.64	0	0	0	not significant
12319	GO_ATP_BINDING_CASSETTE_ABC_TRANSPORTER_COMPLEX	1.24	0.63	0	0	0	not significant
12320	GO_VESICLE_UNCOATING	1.24	0.63	0	0	0	not significant
12321	GO_REGULATION_OF_CARDIAC_EPITHELIAL_TO_MESENCHYMAL_TRANS	1.24	0.62	0	0	0	not significant
12322	OSWALD_HEMATOPOIETIC_STEM_CELL_IN_COLLAGEN_GEL_DN	1.25	1.17	0	0	0	not significant
12323	GO_LYMPHOCYTE_MEDIATED_IMMUNITY	1.25	1.10	0	0	0	not significant
12324	SENESE_HDAC1_TARGETS_DN	1.25	1.08	0	0	0	not significant
12325	REACTOME_REGULATION_OF_EXPRESSION_OF_SLITS_AND_ROBOS	1.25	1.00	0	0	0	not significant
12326	GO_FERTILIZATION	1.25	0.89	0	0	0	not significant
12327	GO_AUTOPHAGOSOME	1.25	0.87	0	0	0	not significant
12328	GO_CYTOKINE_METABOLIC_PROCESS	1.25	0.86	0	0	0	not significant
12329	KRAS.PROSTATE_UP.V1_DN	1.25	0.86	0	0	0	not significant
12330	GO_INTERFERON_GAMMA_PRODUCTION	1.25	0.84	0	0	0	not significant
12331	JI_RESPONSE_TO_FSH_UP	1.25	0.83	0	0	0	not significant
12332	REACTOME_INTERLEUKIN_3_INTERLEUKIN_5_AND_GM_CSF_SIGNALING	1.25	0.81	0	0	0	not significant
12333	KRAS.300_UP.V1_DN	1.25	0.80	0	0	0	not significant
12334	BOGNI_TREATMENT_RELATED_MYELOID_LEUKEMIA_DN	1.25	0.76	0	0	0	not significant
12335	GO_PROTEIN_TARGETING_TO_LYSOSOME	1.25	0.76	0	0	0	not significant
12336	GO_HEME_TRANSPORTER_ACTIVITY	1.25	0.74	0	0	0	not significant
12337	GO_INTEGRAL_COMPONENT_OF_SYNAPTIC_VESICLE_MEMBRANE	1.25	0.74	0	0	0	not significant
12338	GO_NEGATIVE_REGULATION_OF_BMP_SIGNALING_PATHWAY	1.25	0.74	0	0	0	not significant
12339	GO_RESPONSE_TO_ZINC_ION	1.25	0.74	0	0	0	not significant
12340	RIZ_ERYTHROID_DIFFERENTIATION_HBZ	1.25	0.74	0	0	0	not significant
12341	REACTOME_DSCAM_INTERACTIONS	1.25	0.72	0	0	0	not significant
12342	REACTOME_REGULATION_OF_RUNX1_EXPRESSION_AND_ACTIVITY	1.25	0.71	0	0	0	not significant
12343	BARRIER_COLON_CANCER_RECURRENCE_DN	1.25	0.70	0	0	0	not significant
12344	GO_SULFOTRANSFERASE_ACTIVITY	1.25	0.70	0	0	0	not significant
12345	GO_BETA_CATENIN_DESTRUCTION_COMPLEX_DISASSEMBLY	1.25	0.69	0	0	0	not significant
12346	REACTOME_ACTIVATION_OF_THE_AP_1_FAMILY_OF_TRANSCRIPTION_F	1.25	0.69	0	0	0	not significant
12347	GO_CYCLIN_DEPENDENT_PROTEIN_SERINE_THREONINE_KINASE_INHIBIT	1.25	0.68	0	0	0	not significant
12348	GO_CYSTEINE_TYPE_ENDOPEPTIDASE_ACTIVITY_INVOLVED_IN_APOPTO	1.25	0.68	0	0	0	not significant
12349	GO_NEGATIVE_REGULATION_OF_PHAGOCYTOSIS	1.25	0.68	0	0	0	not significant
12350	GO_POSITIVE_REGULATION_OF_GRANULOCYTE_MACROPHAGE_COLON	1.25	0.68	0	0	0	not significant
12351	GO_POSITIVE_REGULATION_OF_HISTONE_H3_K9_METHYLATION	1.25	0.68	0	0	0	not significant
12352	GO_POSITIVE_REGULATION_OF_SMOOTH_MUSCLE_CONTRACTION	1.25	0.68	0	0	0	not significant
12353	GO_REGULATION_OF_CD8_POSITIVE_ALPHA_BETA_T_CELL_DIFFERENT	1.25	0.68	0	0	0	not significant
12354	GO_CERAMIDE_BINDING	1.25	0.67	0	0	0	not significant
12355	GO_NEGATIVE_REGULATION_OF_RECEPTOR_MEDIATED_ENDOCYTOSIS	1.25	0.67	0	0	0	not significant
12356	REACTOME_FOXO_MEDIATED_TRANSCRIPTION_OF_CELL_CYCLE_GENE	1.25	0.67	0	0	0	not significant
12357	GO_NEGATIVE_REGULATION_OF_ALPHA_BETA_T_CELL_PROLIFERATIOI	1.25	0.66	0	0	0	not significant
12358	GO_RESPONSE_TO_MURAMYL_DIPEPTIDE	1.25	0.66	0	0	0	not significant
12359	MATZUK_POSTIMPLANTATION_AND_POSTPARTUM	1.25	0.66	0	0	0	not significant
12360	SCHOEN_NFKB_SIGNALING	1.25	0.66	0	0	0	not significant
12361	GO_REGULATION_OF_HAIR_CYCLE	1.25	0.65	0	0	0	not significant
12362	GO_PLASMA_MEMBRANE_PHOSPHOLIPID_SCRAMBLING	1.25	0.64	0	0	0	not significant
12363	LIEN_BREAST_CARCINOMA_METAPLASTIC	1.25	0.64	0	0	0	not significant
12364	REACTOME_INTERLEUKIN_6_SIGNALING	1.25	0.64	0	0	0	not significant
12365	GO_POSITIVE_REGULATION_OF_INTEGRIN_ACTIVATION	1.25	0.63	0	0	0	not significant
12366	GO_POLYNUCLEOTIDE_DEPHOSPHORYLATION	1.25	0.62	0	0	0	not significant
12367	GO_PARENTAL_BEHAVIOR	1.25	0.60	0	0	0	not significant
12368	GO_PEPTIDYL_TYROSINE_MODIFICATION	1.26	1.15	0	0	0	not significant
12369	GO_B_CELL_ACTIVATION	1.26	1.09	0	0	0	not significant
12370	SCHLOSSER_SERUM_RESPONSE_UP	1.26	1.01	0	0	0	not significant
12371	GAUSSMANN_MLL_AF4_FUSION_TARGETS_C_UP	1.26	0.99	0	0	0	not significant
12372	GO_LIPOPROTEIN_BIOSYNTHETIC_PROCESS	1.26	0.95	0	0	0	not significant
12373	RUIZ_TNC_TARGETS_UP	1.26	0.95	0	0	0	not significant
12374	KEGG_PROSTATE_CANCER	1.26	0.93	0	0	0	not significant
12375	BOYLAN_MULTIPLE_MYELOMA_C_DN	1.26	0.90	0	0	0	not significant
12376	VART_KSHV_INFECTION_ANGIOGENIC_MARKERS_UP	1.26	0.89	0	0	0	not significant
12377	GO_VOLTAGE_GATED_ION_CHANNEL_ACTIVITY	1.26	0.87	0	0	0	not significant
12378	GO_B_CELL_PROLIFERATION	1.26	0.86	0	0	0	not significant
12379	BILANGES_RAPAMYCIN_SENSITIVE_VIA_TSC1_AND_TSC2	1.26	0.85	0	0	0	not significant
12380	DELPUECH_FOXO3_TARGETS_UP	1.26	0.85	0	0	0	not significant
12381	FURUKAWA_DUSP6_TARGETS_PCI35_UP	1.26	0.83	0	0	0	not significant
12382	TURASHVILI_BREAST_LOBULAR_CARCINOMA_VS_LOBULAR_NORMAL_DN	1.26	0.81	0	0	0	not significant
12383	LIM_MAMMARY_LUMINAL_PROGENITOR_UP	1.26	0.77	0	0	0	not significant
12384	BECKER_TAMOXIFEN_RESISTANCE_DN	1.26	0.76	0	0	0	not significant
12385	GO_PITUITARY_GLAND_DEVELOPMENT	1.26	0.76	0	0	0	not significant
12386	GO_REGULATION_OF_PROTEIN_LOCALIZATION_TO_SYNAPSE	1.26	0.75	0	0	0	not significant
12387	GO_REGULATION_OF_CLATHRIN_DEPENDENT_ENDOCYTOSIS	1.26	0.74	0	0	0	not significant
12388	APPEL_IMATINIB_RESPONSE	1.26	0.73	0	0	0	not significant
12389	KEGG_ARACHIDONIC_ACID_METABOLISM	1.26	0.73	0	0	0	not significant
12390	BIOCARTA_GH_PATHWAY	1.26	0.72	0	0	0	not significant
12391	GO_FIBROBLAST_GROWTH_FACTOR_BINDING	1.26	0.72	0	0	0	not significant
12392	GO_REGULATION_OF_GLYCOGEN_METABOLIC_PROCESS	1.26	0.72	0	0	0	not significant
12393	GARGALOVIC_RESPONSE_TO_OXIDIZED_PHOSPHOLIPIDS_TAN_DN	1.26	0.71	0	0	0	not significant
12394	GO_CYTOLYSIS	1.26	0.71	0	0	0	not significant
12395	SA_REG_CASCADE_OF_CYCLIN_EXPR	1.26	0.71	0	0	0	not significant
12396	TOMLINS_METASTASIS_DN	1.26	0.71	0	0	0	not significant
12397	GO_NEGATIVE_REGULATION_OF_LIPOPOLYSACCHARIDE_MEDIATED_SIG	1.26	0.70	0	0	0	not significant
12398	WU_ALZHEIMER_DISEASE_UP	1.26	0.70	0	0	0	not significant
12399	AMUNDSON_GAMMA_RADIATION_RESISTANCE	1.26	0.69	0	0	0	not significant
12400	GO_CD8_POSITIVE_ALPHA_BETA_T_CELL_DIFFERENTIATION	1.26	0.69	0	0	0	not significant
12401	GO_REGULATION_OF_STORE_OPERATED_CALCIUM_ENTRY	1.26	0.69	0	0	0	not significant
12402	GO_SINGLE_STRANDED_DNA_3_5_EXODEOXYRIBONUCLEASE_ACTIVITY	1.26	0.69	0	0	0	not significant
12403	GO_NEGATIVE_REGULATION_OF_OSTEOBLAST_PROLIFERATION	1.26	0.68	0	0	0	not significant
12404	REACTOME_INTERLEUKIN_23_SIGNALING	1.26	0.68	0	0	0	not significant
12405	CHR4P11	1.26	0.67	0	0	0	not significant
12406	GO_EPITHELIAL_TO_MESENCHYMAL_TRANSITION_INVOLVED_IN_ENDOCA	1.26	0.67	0	0	0	not significant
12407	GO_PROTEIN_CARBOXYL_O_METHYLTRANSFERASE_ACTIVITY	1.26	0.67	0	0	0	not significant
12408	GO_BUBBLE_DNA_BINDING	1.26	0.66	0	0	0	not significant
12409	GO_NUCLEAR_MEMBRANE_PROTEIN_COMPLEX	1.26	0.66	0	0	0	not significant
12410	GO_ACYLGLYCEROL_LIPASE_ACTIVITY	1.26	0.65	0	0	0	not significant
12411	ZHONG_RESPONSE_TO_AZACITIDINE_AND_TSA_UP	1.27	1.10	0	0	0	not significant
12412	GO_REGULATION_OF_T_CELL_DIFFERENTIATION	1.27	1.00	0	0	0	not significant
12413	PTEN_DN.V2_DN	1.27	1.00	0	0	0	not significant
12414	GO_CELL_CELL_JUNCTION_ASSEMBLY	1.27	0.93	0	0	0	not significant

12415	GO_GLYCOLIPID_BIOSYNTHETIC_PROCESS	1.27	0.91	0	0	0	not significant
12416	GO_T_CELL_ACTIVATION_INVOLVED_IN_IMMUNE_RESPONSE	1.27	0.90	0	0	0	not significant
12417	GO_REGULATION_OF_TISSUE_REMODELING	1.27	0.89	0	0	0	not significant
12418	CHR15Q26	1.27	0.88	0	0	0	not significant
12419	DORSAM_HOXA9_TARGETS_DN	1.27	0.86	0	0	0	not significant
12420	EHLERS_ANEUPLOIDY_UP	1.27	0.85	0	0	0	not significant
12421	GO_HOMOLOGOUS_CHROMOSOME_SEGREGATION	1.27	0.85	0	0	0	not significant
12422	KEGG_LEISHMANIA_INFECTION	1.27	0.83	0	0	0	not significant
12423	NICK_RESPONSE_TO_PROC_TREATMENT_DN	1.27	0.83	0	0	0	not significant
12424	PID_NFAT_TFPATHWAY	1.27	0.83	0	0	0	not significant
12425	GO_MANNOSYLTRANSFERASE_ACTIVITY	1.27	0.82	0	0	0	not significant
12426	GO_NEGATIVE_REGULATION_OF_DNA_RECOMBINATION	1.27	0.82	0	0	0	not significant
12427	DAUER_STAT3_TARGETS_UP	1.27	0.81	0	0	0	not significant
12428	LUI_THYROID_CANCER_CLUSTER_3	1.27	0.79	0	0	0	not significant
12429	ZHAN_MULTIPLE_MYELOMA_CD2_UP	1.27	0.79	0	0	0	not significant
12430	GO_BCL_2_FAMILY_PROTEIN_COMPLEX	1.27	0.78	0	0	0	not significant
12431	GO_PHOSPHORYLASE_KINASE_ACTIVITY	1.27	0.78	0	0	0	not significant
12432	SNIJDERS_AMPLIFIED_IN_HEAD_AND_NECK_TUMORS	1.27	0.78	0	0	0	not significant
12433	BOYLAN_MULTIPLE_MYELOMA_C_CLUSTER_DN	1.27	0.77	0	0	0	not significant
12434	BIOCARTA_CYTOKINE_PATHWAY	1.27	0.76	0	0	0	not significant
12435	GILMORE_CORE_NFKB_PATHWAY	1.27	0.75	0	0	0	not significant
12436	GO_PROTEIN_DEGLYCOSYLATION	1.27	0.75	0	0	0	not significant
12437	PID_IL3_PATHWAY	1.27	0.75	0	0	0	not significant
12438	BIOCARTA_MONOCYTE_PATHWAY	1.27	0.74	0	0	0	not significant
12439	GO_POSITIVE_REGULATION_OF_REGULATORY_T_CELL_DIFFERENTIATI(	1.27	0.73	0	0	0	not significant
12440	GO_REGULATION_OF_OVARIAN_FOLLICLE_DEVELOPMENT	1.27	0.73	0	0	0	not significant
12441	REACTOME_INTERLEUKIN_2_SIGNALING	1.27	0.73	0	0	0	not significant
12442	SEMBA_FHIT_TARGETS_UP	1.27	0.73	0	0	0	not significant
12443	VERRECCHIA_RESPONSE_TO_TGFB1_C2	1.27	0.73	0	0	0	not significant
12444	BIOCARTA_LYM_PATHWAY	1.27	0.72	0	0	0	not significant
12445	REACTOME_PRESYNAPTIC_DEPOLARIZATION_AND_CALCIUM_CHANNEL_	1.27	0.72	0	0	0	not significant
12446	SASAKI_TARGETS_OF_TP73_AND_TP63	1.27	0.72	0	0	0	not significant
12447	GO_ASPARTIC_ENDOPEPTIDASE_ACTIVITY_INTRAMEMBRANE_CLEAVING	1.27	0.71	0	0	0	not significant
12448	GO_TRIGLYCERIDE_LIPASE_ACTIVITY	1.27	0.71	0	0	0	not significant
12449	GO_INTERLEUKIN_23_MEDIATED_SIGNALING_PATHWAY	1.27	0.70	0	0	0	not significant
12450	GO_INTRINSIC_COMPONENT_OF_VACUOLAR_MEMBRANE	1.27	0.69	0	0	0	not significant
12451	GO_NEGATIVE_REGULATION_OF_NEUROTRANSMITTER_SECRETION	1.27	0.67	0	0	0	not significant
12452	GO_ADAPTIVE_IMMUNE_RESPONSE	1.28	1.33	0	0	0	not significant
12453	GAUSSMANN_MLL_AF4_FUSION_TARGETS_F_UP	1.28	1.07	0	0	0	not significant
12454	GO_POSITIVE_REGULATION_OF_PEPTIDYL_TYROSINE_PHOSPHORYLATI	1.28	0.92	0	0	0	not significant
12455	BILANGES_SERUM_SENSITIVE_GENES	1.28	0.90	0	0	0	not significant
12456	GO_POSITIVE_REGULATION_OF_SMALL_GTPASE_MEDIATED_SIGNAL_TF	1.28	0.89	0	0	0	not significant
12457	GO_REGULATION_OF_CELL_KILLING	1.28	0.89	0	0	0	not significant
12458	GO_PHAGOPHORE_ASSEMBLY_SITE	1.28	0.88	0	0	0	not significant
12459	REACTOME_NCAM_SIGNALING_FOR_NEURITE_OUT_GROWTH	1.28	0.88	0	0	0	not significant
12460	GO_REGULATION_OF_DEFENSE_RESPONSE_TO_VIRUS_BY_HOST	1.28	0.80	0	0	0	not significant
12461	GO_PROTON_TRANSPORTING_V_TYPE_ATPASE_V0_DOMAIN	1.28	0.78	0	0	0	not significant
12462	HOLLEMAN_DAUNORUBICIN_B_ALL_DN	1.28	0.78	0	0	0	not significant
12463	GO_SODIUM_CHANNEL_COMPLEX	1.28	0.77	0	0	0	not significant
12464	GO_HISTONE_H3_K27_METHYLATION	1.28	0.76	0	0	0	not significant
12465	TIAN_TNF_SIGNALING_VIA_NFKB	1.28	0.76	0	0	0	not significant
12466	GO_HYPOTHALAMUS_DEVELOPMENT	1.28	0.75	0	0	0	not significant
12467	GO_NEGATIVE_REGULATION_OF_CELL_KILLING	1.28	0.75	0	0	0	not significant
12468	GO_REGULATION_OF_INOSITOL_PHOSPHATE_BIOSYNTHETIC_PROCESS	1.28	0.75	0	0	0	not significant
12469	CHR2Q34	1.28	0.74	0	0	0	not significant
12470	GO_PHOSPHATE_ION_BINDING	1.28	0.74	0	0	0	not significant
12471	GO_SPHINGOID_BIOSYNTHETIC_PROCESS	1.28	0.74	0	0	0	not significant
12472	GO_GRANULOCYTE_MACROPHAGE_COLONY_STIMULATING_FACTOR_PI	1.28	0.73	0	0	0	not significant
12473	GO_NEGATIVE_REGULATION_OF_LIPID_CATABOLIC_PROCESS	1.28	0.73	0	0	0	not significant
12474	GO_NEGATIVE_REGULATION_OF_NATURAL_KILLER_CELL_MEDIATED_IM	1.28	0.73	0	0	0	not significant
12475	GO_SODIUM_CHANNEL_ACTIVITY	1.28	0.73	0	0	0	not significant
12476	GO_SENSORY_PERCEPTION_OF_SMELL	1.28	0.72	0	0	0	not significant
12477	REACTOME_ASSEMBLY_OF_ACTIVE_LPL_AND_LIPC_LIPASE_COMPLEXE	1.28	0.72	0	0	0	not significant
12478	CONRAD_GERMLINE_STEM_CELL	1.28	0.71	0	0	0	not significant
12479	YAO_HOXA10_TARGETS_VIA_PROGESTERONE_DN	1.28	0.71	0	0	0	not significant
12480	CASTELLANO_HRAS_AND_NRAS_TARGETS_DN	1.28	0.69	0	0	0	not significant
12481	GO_POTASSIUM_ION_LEAK_CHANNEL_ACTIVITY	1.28	0.69	0	0	0	not significant
12482	THILLAINADESAN_ZNF217_TARGETS_DN	1.28	0.68	0	0	0	not significant
12483	GO_CELL_FATE_COMMITMENT	1.29	1.11	0	0	0	not significant
12484	KRAS.600.LUNG.BREAST_UP.V1_UP	1.29	1.07	0	0	0	not significant
12485	RICKMAN_TUMOR_DIFFERENTIATED_WELL_VS_MODERATELY_UP	1.29	1.07	0	0	0	not significant
12486	KRAS.600.LUNG.BREAST_UP.V1_DN	1.29	1.04	0	0	0	not significant
12487	GO_ENDOTHELIUM_DEVELOPMENT	1.29	1.01	0	0	0	not significant
12488	KIM_MYC_AMPLIFICATION_TARGETS_DN	1.29	0.96	0	0	0	not significant
12489	CHR7Q21	1.29	0.95	0	0	0	not significant
12490	GO_AMINOGLYCAN_METABOLIC_PROCESS	1.29	0.95	0	0	0	not significant
12491	AMIT_SERUM_RESPONSE_60_MCF10A	1.29	0.94	0	0	0	not significant
12492	GO_POLYSOME	1.29	0.94	0	0	0	not significant
12493	JECHLINGER_EPITHELIAL_TO_MESENCHYMAL_TRANSITION_UP	1.29	0.91	0	0	0	not significant
12494	GO_REGULATION_OF_RECEPTOR_SIGNALING_PATHWAY_VIA_STAT	1.29	0.90	0	0	0	not significant
12495	REACTOME_CD28_DEPENDENT_PI3K_AKT_SIGNALING	1.29	0.88	0	0	0	not significant
12496	CAHOY_NEURONAL	1.29	0.85	0	0	0	not significant
12497	GO_MESENCHYMAL_CELL_PROLIFERATION	1.29	0.85	0	0	0	not significant
12498	GO_POSITIVE_REGULATION_OF_INTERLEUKIN_8_PRODUCTION	1.29	0.85	0	0	0	not significant
12499	HOFFMANN_IMMATURE_TO_MATURE_B_LYMPHOCYTE_UP	1.29	0.84	0	0	0	not significant
12500	GO_MITOPHAGY	1.29	0.83	0	0	0	not significant
12501	CHR9P22	1.29	0.81	0	0	0	not significant
12502	GO_LENS_FIBER_CELL_DIFFERENTIATION	1.29	0.81	0	0	0	not significant
12503	GO_MICROTUBULE_NUCLEATION_BY_MICROTUBULE_ORGANIZING_CENT	1.29	0.81	0	0	0	not significant
12504	ISHIKAWA_STING_SIGNALING	1.29	0.81	0	0	0	not significant
12505	GO_NEGATIVE_REGULATION_OF_NIK_NF_KAPPAB_SIGNALING	1.29	0.80	0	0	0	not significant
12506	GO_RECEPTOR_SIGNALING_COMPLEX_SCAFFOLD_ACTIVITY	1.29	0.80	0	0	0	not significant
12507	HOLLEMAN_ASPARAGINASE_RESISTANCE_ALL_UP	1.29	0.80	0	0	0	not significant
12508	RASHI_NFKB1_TARGETS	1.29	0.79	0	0	0	not significant
12509	GO_NATURAL_KILLER_CELL_DIFFERENTIATION	1.29	0.78	0	0	0	not significant
12510	GO_NUCLEOTIDE_BINDING_OLIGOMERIZATION_DOMAIN_CONTAINING_2_	1.29	0.78	0	0	0	not significant
12511	GO_SOMATIC_STEM_CELL_DIVISION	1.29	0.78	0	0	0	not significant
12512	GO_WNT_PROTEIN_BINDING	1.29	0.78	0	0	0	not significant
12513	GO_PROTEIN_BINDING_BRIDGING_INVOLVED_IN_SUBSTRATE_RECOGNIT	1.29	0.77	0	0	0	not significant
12514	GO_VOLTAGE_GATED_CALCIUM_CHANNEL_COMPLEX	1.29	0.77	0	0	0	not significant
12515	ZIRN_TRETINOIN_RESPONSE_WT1_UP	1.29	0.77	0	0	0	not significant
12516	GARGALOVIC_RESPONSE_TO_OXIDIZED_PHOSPHOLIPIDS_LIGHTYELLOW	1.29	0.76	0	0	0	not significant
12517	GO_BASIC_AMINO_ACID_TRANSMEMBRANE_TRANSPORT	1.29	0.76	0	0	0	not significant
12518	GO_INHIBITORY_SYNAPSE	1.29	0.76	0	0	0	not significant
12519	GO_NEGATIVE_REGULATION_OF_ACTIN_FILAMENT_BUNDLE_ASSEMBLY	1.29	0.75	0	0	0	not significant
12520	BIOCARTA_TCYTOTOXIC_PATHWAY	1.29	0.74	0	0	0	not significant
12521	GO_ALPHA_CATENIN_BINDING	1.29	0.74	0	0	0	not significant
12522	GO_DIACYLGLYCEROL_METABOLIC_PROCESS	1.29	0.74	0	0	0	not significant
12523	GO_HISTONE_H3_K14_ACETYLATION	1.29	0.74	0	0	0	not significant

12524	GO_REGULATION_OF_HEAT_GENERATION	1.29	0.74	0	0	0	not significant
12525	REACTOME_INTERACTION_BETWEEN_L1_AND_ANKYRINS	1.29	0.74	0	0	0	not significant
12526	GO_GAP_JUNCTION	1.29	0.73	0	0	0	not significant
12527	CHR5P12	1.29	0.72	0	0	0	not significant
12528	KIM_ALL_DISORDERS_DURATION_CORR_UP	1.29	0.72	0	0	0	not significant
12529	REACTOME_NEGATIVE_REGULATION_OF_ACTIVITY_OF_TFAP2_AP_2_FA	1.29	0.72	0	0	0	not significant
12530	LEI_HOXC8_TARGETS_UP	1.29	0.70	0	0	0	not significant
12531	BENPORATH_ES_WITH_H3K27ME3	1.30	1.53	0	0	0	not significant
12532	GO_ADAPTIVE_IMMUNE_RESPONSE_BASED_ON_SOMATIC_RECOMBINAT	1.30	1.23	0	0	0	not significant
12533	KEGG_LYSOSOME	1.30	1.15	0	0	0	not significant
12534	MTOR_UP.V1_DN	1.30	1.15	0	0	0	not significant
12535	NABA_SECRETED_FACTORS	1.30	1.09	0	0	0	not significant
12536	GO_LYMPHOCYTE_ACTIVATION_INVOLVED_IN_IMMUNE_RESPONSE	1.30	1.08	0	0	0	not significant
12537	GO_B_CELL_DIFFERENTIATION	1.30	1.04	0	0	0	not significant
12538	SARRIO_EPITHELIAL_MESENCHYMAL_TRANSITION_DN	1.30	1.03	0	0	0	not significant
12539	DACOSTA_UV_RESPONSE_VIA_ERCC3_COMMON_UP	1.30	1.01	0	0	0	not significant
12540	KIM_GERMINAL_CENTER_T_HELPER_UP	1.30	0.98	0	0	0	not significant
12541	GO_LENS_DEVELOPMENT_IN_CAMERA_TYPE_EYE	1.30	0.94	0	0	0	not significant
12542	ZHAN_MULTIPLE_MYELOMA_HP_UP	1.30	0.93	0	0	0	not significant
12543	GO_ENDODEOXYRIBONUCLEASE_ACTIVITY	1.30	0.90	0	0	0	not significant
12544	SUNG_METASTASIS_STROMA_DN	1.30	0.90	0	0	0	not significant
12545	GO_TRANSMEMBRANE_RECEPTOR_PROTEIN_KINASE_ACTIVITY	1.30	0.88	0	0	0	not significant
12546	CHR7Q34	1.30	0.86	0	0	0	not significant
12547	CHR10Q21	1.30	0.85	0	0	0	not significant
12548	CHR6Q22	1.30	0.85	0	0	0	not significant
12549	GO_INTRINSIC_COMPONENT_OF_PRESYNAPTIC_MEMBRANE	1.30	0.83	0	0	0	not significant
12550	HEIDENBLAD_AMPLICON_12P11_12_DN	1.30	0.83	0	0	0	not significant
12551	PID_DNA_PK_PATHWAY	1.30	0.83	0	0	0	not significant
12552	GO_GLUTAMINE_METABOLIC_PROCESS	1.30	0.82	0	0	0	not significant
12553	GO_NEGATIVE_REGULATION_OF_INTERFERON_GAMMA_PRODUCTION	1.30	0.81	0	0	0	not significant
12554	REACTOME_TRANSPORT_OF_NUCLEOSIDES_AND_FREE_PURINE_AND_F	1.30	0.81	0	0	0	not significant
12555	GO_ENDOCARDIAL_CUSHION_MORPHOGENESIS	1.30	0.80	0	0	0	not significant
12556	GO_INTERMEMBRANE_LIPID_TRANSFER_ACTIVITY	1.30	0.79	0	0	0	not significant
12557	GO_SECONDARY_PALATE_DEVELOPMENT	1.30	0.78	0	0	0	not significant
12558	GO_CARDIAC_RIGHT_VENTRICLE_MORPHOGENESIS	1.30	0.76	0	0	0	not significant
12559	GO_N_ACETYLNEURAMINATE_CATABOLIC_PROCESS	1.30	0.76	0	0	0	not significant
12560	GO_TELOMERE_LOCALIZATION	1.30	0.76	0	0	0	not significant
12561	GO_POSITIVE_REGULATION_OF_AMYLOID_BETA_CLEARANCE	1.30	0.75	0	0	0	not significant
12562	KOBAYASHI_EGFR_SIGNALING_6HR_UP	1.30	0.75	0	0	0	not significant
12563	REACTOME_SIGNALING_BY_NODAL	1.30	0.75	0	0	0	not significant
12564	CHRXQ25	1.30	0.74	0	0	0	not significant
12565	GO_RESPONSE_TO_INTERLEUKIN_2	1.30	0.70	0	0	0	not significant
12566	ONKEN_UVEAL_MELANOMA_DN	1.31	1.96	0	0	0	not significant
12567	GO_T_CELL_DIFFERENTIATION	1.31	1.43	0	0	0	not significant
12568	CASORELLI_ACUTE_PROMYELOCYTIC_LEUKEMIA_UP	1.31	1.20	0	0	0	not significant
12569	GO_MEIOSIS_I_CELL_CYCLE_PROCESS	1.31	1.12	0	0	0	not significant
12570	GO_HUMORAL_IMMUNE_RESPONSE	1.31	1.11	0	0	0	not significant
12571	GO_ROUGH_ENDOPLASMIC_RETICULUM	1.31	1.04	0	0	0	not significant
12572	CROONQUIST_STROMAL_STIMULATION_UP	1.31	0.97	0	0	0	not significant
12573	GO_POSITIVE_REGULATION_OF_CARBOHYDRATE_METABOLIC_PROCES	1.31	0.95	0	0	0	not significant
12574	KRAS.LUNG_UP.V1_UP	1.31	0.95	0	0	0	not significant
12575	LIM_MAMMARY_LUMINAL_MATURE_DN	1.31	0.95	0	0	0	not significant
12576	GO_MODIFIED_AMINO_ACID_BINDING	1.31	0.93	0	0	0	not significant
12577	CHR15Q11	1.31	0.91	0	0	0	not significant
12578	REACTOME_PROLACTIN_RECEPTOR_SIGNALING	1.31	0.91	0	0	0	not significant
12579	GO_TRANSMEMBRANE_RECEPTOR_PROTEIN_TYROSINE_KINASE_ACTIVI	1.31	0.90	0	0	0	not significant
12580	PID_TCPTP_PATHWAY	1.31	0.90	0	0	0	not significant
12581	GO_POSITIVE_REGULATION_OF_LIPASE_ACTIVITY	1.31	0.88	0	0	0	not significant
12582	GO_PROTEIN_DEMETHYLATION	1.31	0.88	0	0	0	not significant
12583	MCBRYAN_PUBERTAL_TGFB1_TARGETS_DN	1.31	0.88	0	0	0	not significant
12584	REACTOME_ARACHIDONIC_ACID_METABOLISM	1.31	0.87	0	0	0	not significant
12585	ZHENG_FOXP3_TARGETS_UP	1.31	0.87	0	0	0	not significant
12586	GO_ALPHA_BETA_T_CELL_PROLIFERATION	1.31	0.86	0	0	0	not significant
12587	GO_ARACHIDONIC_ACID_METABOLIC_PROCESS	1.31	0.86	0	0	0	not significant
12588	GO_DETERMINATION_OF_DORSAL_VENTRAL_ASYMMETRY	1.31	0.86	0	0	0	not significant
12589	HOQUE_METHYLATED_IN_CANCER	1.31	0.86	0	0	0	not significant
12590	REACTOME_REGULATION_OF_TP53_ACTIVITY_THROUGH_METHYLATION	1.31	0.86	0	0	0	not significant
12591	BIOCARTA_LYMPHOCYTE_PATHWAY	1.31	0.85	0	0	0	not significant
12592	GO_CELL_FATE_SPECIFICATION	1.31	0.85	0	0	0	not significant
12593	GAUSSMANN_MLL_AF4_FUSION_TARGETS_D_DN	1.31	0.82	0	0	0	not significant
12594	GO_NEGATIVE_REGULATION_OF_AXON_GUIDANCE	1.31	0.82	0	0	0	not significant
12595	GO_REGULATION_OF_HISTONE_H3_K27_METHYLATION	1.31	0.82	0	0	0	not significant
12596	GO_ARGININE_TRANSPORT	1.31	0.80	0	0	0	not significant
12597	GO_POSITIVE_REGULATION_OF_FIBROBLAST_APOPTOTIC_PROCESS	1.31	0.80	0	0	0	not significant
12598	GO_ANTIBIOTIC_TRANSPORT	1.31	0.78	0	0	0	not significant
12599	GO_ENDOSOME_TO_PLASMA_MEMBRANE_PROTEIN_TRANSPORT	1.31	0.78	0	0	0	not significant
12600	GO_NARROW_PORE_CHANNEL_ACTIVITY	1.31	0.77	0	0	0	not significant
12601	GO_PROTON_TRANSPORTING_TWO_SECTOR_ATPASE_COMPLEX_PROT	1.31	0.77	0	0	0	not significant
12602	GO_ANCHORED_COMPONENT_OF_SYNAPTIC_VESICLE_MEMBRANE	1.31	0.76	0	0	0	not significant
12603	GO_INTRONIC_TRANSCRIPTION_REGULATORY_REGION_DNA_BINDING	1.31	0.76	0	0	0	not significant
12604	GO_SEQUESTERING_OF_ACTIN_MONOMERS	1.31	0.76	0	0	0	not significant
12605	GO_POST_ANAL_TAIL_MORPHOGENESIS	1.31	0.75	0	0	0	not significant
12606	REACTOME_GABA_SYNTHESIS_RELEASE_REUPTAKE_AND_DEGRADATIC	1.31	0.73	0	0	0	not significant
12607	GO_DIPEPTIDASE_ACTIVITY	1.31	0.72	0	0	0	not significant
12608	CHR19P13	1.32	1.94	0	0	0	not significant
12609	DURAND_STROMA_S_UP	1.32	1.36	0	0	0	not significant
12610	GO_POSTSYNAPTIC_MEMBRANE	1.32	1.27	0	0	0	not significant
12611	ZWANG_EGF_INTERVAL_DN	1.32	1.27	0	0	0	not significant
12612	TAKEDA_TARGETS_OF_NUP98_HOXA9_FUSION_3D_UP	1.32	1.18	0	0	0	not significant
12613	AFFAR_YY1_TARGETS_UP	1.32	1.14	0	0	0	not significant
12614	GO_CILIARY_PLASM	1.32	1.11	0	0	0	not significant
12615	GO_SYNAPSE_ASSEMBLY	1.32	1.06	0	0	0	not significant
12616	GO_APPENDAGE_MORPHOGENESIS	1.32	1.05	0	0	0	not significant
12617	GO_GLYCOPROTEIN_METABOLIC_PROCESS	1.32	1.04	0	0	0	not significant
12618	GO_DORSAL_VENTRAL_PATTERN_FORMATION	1.32	1.02	0	0	0	not significant
12619	REACTOME_NOD1_2_SIGNALING_PATHWAY	1.32	1.00	0	0	0	not significant
12620	GO_AMMONIUM_TRANSPORT	1.32	0.97	0	0	0	not significant
12621	LIU_TARGETS_OF_VMYB_VS_CMYB_DN	1.32	0.96	0	0	0	not significant
12622	DORSAM_HOXA9_TARGETS_UP	1.32	0.93	0	0	0	not significant
12623	GO_RESPONSE_TO_SORBITOL	1.32	0.93	0	0	0	not significant
12624	KUUSELO_PANCREATIC_CANCER_19Q13_AMPLIFICATION	1.32	0.92	0	0	0	not significant
12625	GO_HISTONE_DEMETHYLASE_ACTIVITY	1.32	0.89	0	0	0	not significant
12626	GO_REGULATION_OF_SYNAPTIC_VESICLE_RECYCLING	1.32	0.89	0	0	0	not significant
12627	GO_CARDIAC_ATRIUM_DEVELOPMENT	1.32	0.87	0	0	0	not significant
12628	OHM_METHYLATED_IN_ADULT_CANCERS	1.32	0.86	0	0	0	not significant
12629	PID_IL5_PATHWAY	1.32	0.86	0	0	0	not significant
12630	GO_CELL_VOLUME_HOMEOSTASIS	1.32	0.85	0	0	0	not significant
12631	MUELLER_COMMON_TARGETS_OF_AML_FUSIONS_UP	1.32	0.85	0	0	0	not significant
12632	TUOMISTO_TUMOR_SUPPRESSION_BY_COL13A1_UP	1.32	0.85	0	0	0	not significant

12633	GO_COMPLEMENT_ACTIVATION_ALTERNATIVE_PATHWAY	1.32	0.83	0	0	0	not significant
12634	GO_NEGATIVE_REGULATION_OF_CELLULAR_SENESCENCE	1.32	0.83	0	0	0	not significant
12635	GO_POSITIVE_REGULATION_OF_NATURAL_KILLER_CELL_DIFFERENTIAT	1.32	0.82	0	0	0	not significant
12636	REACTOME_SEMA4D_INDUCED_CELL_MIGRATION_AND_GROWTH_CONE	1.32	0.82	0	0	0	not significant
12637	GO_INTERLEUKIN_13_PRODUCTION	1.32	0.81	0	0	0	not significant
12638	GO_MESODERMAL_CELL_FATE_COMMITMENT	1.32	0.81	0	0	0	not significant
12639	MATZUK_MALE_REPRODUCTION_SERTOLI	1.32	0.81	0	0	0	not significant
12640	GO_POSITIVE_REGULATION_OF_CD8_POSITIVE_ALPHA_BETA_T_CELL_A	1.32	0.79	0	0	0	not significant
12641	GO_LYMPHOCYTE_DIFFERENTIATION	1.33	1.56	0	0	0	not significant
12642	ODONNELL_TFRC_TARGETS_UP	1.33	1.49	0	0	0	not significant
12643	GO_PATTERN_SPECIFICATION_PROCESS	1.33	1.44	0	0	0	not significant
12644	GO_ENHANCER_BINDING	1.33	1.30	0	0	0	not significant
12645	GO_REGULATION_OF_RHO_PROTEIN_SIGNAL_TRANSDUCTION	1.33	1.13	0	0	0	not significant
12646	GO_CD4_POSITIVE_ALPHA_BETA_T_CELL_ACTIVATION	1.33	1.11	0	0	0	not significant
12647	KRAS.AMP.LUNG_UP.V1_DN	1.33	1.05	0	0	0	not significant
12648	WATTEL_AUTONOMOUS_THYROID_ADENOMA_UP	1.33	1.03	0	0	0	not significant
12649	REACTOME_SIGNALING_BY_PDGF	1.33	1.01	0	0	0	not significant
12650	GO_TYROSINE_PHOSPHORYLATION_OF_STAT_PROTEIN	1.33	1.00	0	0	0	not significant
12651	BEIER_GLIOMA_STEM_CELL_DN	1.33	0.99	0	0	0	not significant
12652	BURTON_ADIPOGENESIS_11	1.33	0.95	0	0	0	not significant
12653	GO_MEIOTIC_TELOMERE_CLUSTERING	1.33	0.94	0	0	0	not significant
12654	GO_REGULATION_OF_HUMORAL_IMMUNE_RESPONSE	1.33	0.94	0	0	0	not significant
12655	SAKAI_CHRONIC_HEPATITIS_VS_LIVER_CANCER_DN	1.33	0.90	0	0	0	not significant
12656	GO_NEGATIVE_REGULATION_OF_NATURAL_KILLER_CELL_ACTIVATION	1.33	0.88	0	0	0	not significant
12657	GOBERT_CORE_OLIGODENDROCYTE_DIFFERENTIATION	1.33	0.88	0	0	0	not significant
12658	GO_LEFT_RIGHT_AXIS_SPECIFICATION	1.33	0.87	0	0	0	not significant
12659	BUCKANOVICH_T_LYMPHOCYTE_HOMING_ON_TUMOR_UP	1.33	0.85	0	0	0	not significant
12660	GO_NEURAL_PLATE_MORPHOGENESIS	1.33	0.84	0	0	0	not significant
12661	CLIMENT_BREAST_CANCER_COPY_NUMBER_UP	1.33	0.82	0	0	0	not significant
12662	GO_BRANCHING_INVOLVED_IN_SALIVARY_GLAND_MORPHOGENESIS	1.33	0.82	0	0	0	not significant
12663	IGLESIAS_E2F_TARGETS_DN	1.33	0.79	0	0	0	not significant
12664	REACTOME_ACYL_CHAIN_REMODELLING_OF_PS	1.33	0.79	0	0	0	not significant
12665	GO_NEURON_TO_NEURON_SYNAPSE	1.34	1.56	0	0	0	not significant
12666	TORCHIA_TARGETS_OF_EWSR1_FLI1_FUSION_UP	1.34	1.45	0	0	0	not significant
12667	GO_LYSOSOMAL_LUMEN	1.34	1.22	0	0	0	not significant
12668	GO_SMALL_RIBOSOMAL_SUBUNIT	1.34	1.14	0	0	0	not significant
12669	KANG_IMMORTALIZED_BY_TERT_DN	1.34	1.14	0	0	0	not significant
12670	LEF1_UP.V1_DN	1.34	1.13	0	0	0	not significant
12671	GO_MICROTUBULE_BUNDLE_FORMATION	1.34	1.06	0	0	0	not significant
12672	HOFFMANN_LARGE_TO_SMALL_PRE_BII_LYMPHOCYTE_DN	1.34	1.06	0	0	0	not significant
12673	GO_CHLORIDE_TRANSPORT	1.34	1.04	0	0	0	not significant
12674	GO_MALE_MEIOTIC_NUCLEAR_DIVISION	1.34	0.97	0	0	0	not significant
12675	NIKOLSKY_BREAST_CANCER_7Q21_Q22_AMPLICON	1.34	0.97	0	0	0	not significant
12676	GO_BASAL_PART_OF_CELL	1.34	0.96	0	0	0	not significant
12677	GO_NEGATIVE_REGULATION_OF_FAT_CELL_DIFFERENTIATION	1.34	0.95	0	0	0	not significant
12678	RODRIGUES_NTN1_AND_DCC_TARGETS	1.34	0.94	0	0	0	not significant
12679	GO_SYNAPTIC_TRANSMISSION_GABAERGIC	1.34	0.93	0	0	0	not significant
12680	ZEILSTRA_CD44_TARGETS_UP	1.34	0.92	0	0	0	not significant
12681	HEDENFALK_BREAST_CANCER_BRACX_UP	1.34	0.91	0	0	0	not significant
12682	GO_DEATH_RECEPTOR_ACTIVITY	1.34	0.90	0	0	0	not significant
12683	GO_IMMUNOLOGICAL_MEMORY_FORMATION_PROCESS	1.34	0.90	0	0	0	not significant
12684	GO_CELLULAR_RESPONSE_TO_GROWTH_HORMONE_STIMULUS	1.34	0.89	0	0	0	not significant
12685	GO_ARGININE_TRANSMEMBRANE_TRANSPORT	1.34	0.88	0	0	0	not significant
12686	BUSA_SAM68_TARGETS_UP	1.34	0.86	0	0	0	not significant
12687	GO_PHOSPHATIDYLSERINE_ACYL_CHAIN_REMODELING	1.34	0.86	0	0	0	not significant
12688	STAEGE_EWING_FAMILY_TUMOR	1.34	0.86	0	0	0	not significant
12689	VERRECCHIA_DELAYED_RESPONSE_TO_TGFB1	1.34	0.86	0	0	0	not significant
12690	GO_PARAXIAL_MESODERM_DEVELOPMENT	1.34	0.85	0	0	0	not significant
12691	DAZARD_UV_RESPONSE_CLUSTER_G24	1.34	0.84	0	0	0	not significant
12692	GO_PURINERGIC_NUCLEOTIDE_RECEPTOR_SIGNALING_PATHWAY	1.34	0.84	0	0	0	not significant
12693	GO_NEGATIVE_REGULATION_OF_CELL_CYCLE_CHECKPOINT	1.34	0.83	0	0	0	not significant
12694	GO_POSITIVE_REGULATION_OF_INTERLEUKIN_13_PRODUCTION	1.34	0.83	0	0	0	not significant
12695	PID_INTEGRIN5_PATHWAY	1.34	0.83	0	0	0	not significant
12696	VERHAAK_AML_WITH_NPM1_MUTATED_DN	1.35	1.54	0	0	0	not significant
12697	GO_CYTOSOLIC_PART	1.35	1.52	0	0	0	not significant
12698	ZWANG_DOWN_BY_2ND_EGF_PULSE	1.35	1.46	0	0	0	not significant
12699	GO_ALPHA_BETA_T_CELL_ACTIVATION	1.35	1.35	0	0	0	not significant
12700	CHICAS_RB1_TARGETS_LOW_SERUM	1.35	1.29	0	0	0	not significant
12701	PID_IL2_1PATHWAY	1.35	1.13	0	0	0	not significant
12702	TONKS_TARGETS_OF_RUNX1_RUNX1T1_FUSION_SUSTAINDED_IN_ERYTH	1.35	1.07	0	0	0	not significant
12703	GO_NEGATIVE_REGULATION_OF_SYNAPTIC_TRANSMISSION	1.35	1.03	0	0	0	not significant
12704	GO_CELL_DIFFERENTIATION_IN_SPINAL_CORD	1.35	0.99	0	0	0	not significant
12705	GO_POSITIVE_T_CELL_SELECTION	1.35	0.99	0	0	0	not significant
12706	RICKMAN_HEAD_AND_NECK_CANCER_A	1.35	0.98	0	0	0	not significant
12707	GO_SENSORY_PERCEPTION_OF_CHEMICAL_STIMULUS	1.35	0.95	0	0	0	not significant
12708	KAUFFMANN_MELANOMA_RELAPSE_DN	1.35	0.92	0	0	0	not significant
12709	GO_FOREBRAIN_NEUROBLAST_DIVISION	1.35	0.90	0	0	0	not significant
12710	GO_HEME_TRANSPORT	1.35	0.89	0	0	0	not significant
12711	REACTOME_P2Y_RECEPTORS	1.35	0.88	0	0	0	not significant
12712	GO_COMPLEMENT_BINDING	1.35	0.87	0	0	0	not significant
12713	BIOCARTA_IL3_PATHWAY	1.35	0.86	0	0	0	not significant
12714	GO_HEAT_GENERATION	1.35	0.86	0	0	0	not significant
12715	GO_POSITIVE_REGULATION_OF_RIG_I_SIGNALING_PATHWAY	1.35	0.86	0	0	0	not significant
12716	GO_LENS_FIBER_CELL_DEVELOPMENT	1.35	0.85	0	0	0	not significant
12717	GO_PROFILIN_BINDING	1.35	0.85	0	0	0	not significant
12718	REACTOME_CATION_COUPLED_CHLORIDE_COTRANSPORTERS	1.35	0.85	0	0	0	not significant
12719	GO_CYSTEINE_TYPE_ENDOPEPTIDASE_ACTIVITY_INVOLVED_IN_APOPTO	1.35	0.84	0	0	0	not significant
12720	GO_HISTONE_H4_K12_ACETYLATION	1.35	0.84	0	0	0	not significant
12721	GO_POSITIVE_REGULATION_OF_PROTEIN_LOCALIZATION_TO_SYNAPSE	1.35	0.84	0	0	0	not significant
12722	REACTOME_OLFACTORY_SIGNALING_PATHWAY	1.35	0.84	0	0	0	not significant
12723	GO_ARGININE_TRANSMEMBRANE_TRANSPORTER_ACTIVITY	1.35	0.82	0	0	0	not significant
12724	WEST_ADRENOCORTICAL_TUMOR_DN	1.36	1.99	0	0	0	not significant
12725	GO_MEMBRANE_LIPID_METABOLIC_PROCESS	1.36	1.48	0	0	0	not significant
12726	VECCHI_GASTRIC_CANCER_EARLY_DN	1.36	1.43	0	0	0	not significant
12727	REACTOME_SPHINGOLIPID_METABOLISM	1.36	1.38	0	0	0	not significant
12728	GO_GOLGI_STACK	1.36	1.34	0	0	0	not significant
12729	RODWELL_AGING_KIDNEY_NO_BLOOD_UP	1.36	1.30	0	0	0	not significant
12730	ALTEMEIER_RESPONSE_TO_LPS_WITH_MECHANICAL_VENTILATION	1.36	1.20	0	0	0	not significant
12731	ATM_DN.V1_DN	1.36	1.13	0	0	0	not significant
12732	GO_NEGATIVE_REGULATION_OF_INNATE_IMMUNE_RESPONSE	1.36	1.10	0	0	0	not significant
12733	GO_POSTSYNAPTIC_SPECIALIZATION_MEMBRANE	1.36	1.04	0	0	0	not significant
12734	GO_REGULATION_OF_VASOCONSTRICTION	1.36	0.99	0	0	0	not significant
12735	REACTOME_G1_S_SPECIFIC_TRANSCRIPTION	1.36	0.97	0	0	0	not significant
12736	IWANAGA_E2F1_TARGETS_INDUCED_BY_SERUM	1.36	0.96	0	0	0	not significant
12737	GO_CELLULAR_RESPONSE_TO_OXIDISED_LOW_DENSITY_LIPOPROTEIN_	1.36	0.95	0	0	0	not significant
12738	TOMLINS_PROSTATE_CANCER_DN	1.36	0.94	0	0	0	not significant
12739	GO_SPERM_AXONEME_ASSEMBLY	1.36	0.93	0	0	0	not significant
12740	GALI_TP53_TARGETS_APOPTOTIC_UP	1.36	0.92	0	0	0	not significant
12741	GO_CALCIUM_DEPENDENT_CELL_CELL_ADHESION_VIA_PLASMA_MEMBF	1.36	0.92	0	0	0	not significant

12742	GO_FOREGUT_MORPHOGENESIS	1.36	0.91	0	0	0	not significant
12743	GO_URETER_DEVELOPMENT	1.36	0.91	0	0	0	not significant
12744	GO_SIGNAL_RECOGNITION_PARTICLE_RECEPTOR_COMPLEX	1.36	0.90	0	0	0	not significant
12745	REACTOME_RUNX3_REGULATES_BCL2L11_BIM_TRANSCRIPTION	1.36	0.89	0	0	0	not significant
12746	GO_PROXIMAL_DISTAL_PATTERN_FORMATION	1.36	0.88	0	0	0	not significant
12747	REACTOME_WNT_LIGAND_BIOGENESIS_AND_TRAFFICKING	1.36	0.88	0	0	0	not significant
12748	BIOCARTA_GATA3_PATHWAY	1.36	0.86	0	0	0	not significant
12749	BIOCARTA_BARD1_PATHWAY	1.36	0.85	0	0	0	not significant
12750	GO_NEGATIVE_REGULATION_OF_P38MAPK_CASCADE	1.36	0.84	0	0	0	not significant
12751	GO_PRESYNAPTIC_MODULATION_OF_CHEMICAL_SYNAPTIC_TRANSMISS	1.36	0.83	0	0	0	not significant
12752	GO_AUTOPHAGIC_CELL_DEATH	1.36	0.82	0	0	0	not significant
12753	JAATINEN_HEMATOPOIETIC_STEM_CELL_UP	1.37	1.65	0	0	0	not significant
12754	PYEON_CANCER_HEAD_AND_NECK_VS_CERVICAL_UP	1.37	1.65	0	0	0	not significant
12755	NAKAMURA_ADIPOGENESIS_EARLY_UP	1.37	1.16	0	0	0	not significant
12756	TONKS_TARGETS_OF_RUNX1_RUNX1T1_FUSION_MONOCYTE_DN	1.37	1.15	0	0	0	not significant
12757	CTIP_DN.V1_UP	1.37	1.10	0	0	0	not significant
12758	GO_POSITIVE_REGULATION_OF_CELLULAR_CARBOHYDRATE_METABOL	1.37	1.06	0	0	0	not significant
12759	GO NEGATIVE REGULATION OF EPITHELIAL CELL APOPTOTIC PROCE	1.37	1.03	0	0	0	not significant
12760	REACTOME ACTIVATION OF PPARGC1A PGC 1ALPHA BY PHOSPHORY	1.37	1.03	0	0	0	not significant
12761	GO_PHOSPHATE_ION_TRANSPORT	1.37	1.02	0	0	0	not significant
12762	GO_EXTRACELLULAR_REGULATION_OF_SIGNAL_TRANSDUCTION	1.37	1.00	0	0	0	not significant
12763	GO_LAMIN_BINDING	1.37	0.99	0	0	0	not significant
12764	GO_POTASSIUM_ION_HOMEOSTASIS	1.37	0.97	0	0	0	not significant
12765	GO_REGULATION_OF_SYNAPTIC_VESICLE_CLUSTERING	1.37	0.96	0	0	0	not significant
12766	GO ENDODEOXYRIBONUCLEASE ACTIVITY PRODUCING 5 PHOSPHOMC	1.37	0.95	0	0	0	not significant
12767	GO_FORELIMB_MORPHOGENESIS	1.37	0.95	0	0	0	not significant
12768	GO_CELLULAR_RESPONSE_TO_ELECTRICAL_STIMULUS	1.37	0.94	0	0	0	not significant
12769	GO TRANSFORMING GROWTH FACTOR BETA RECEPTOR CYTOPLASI	1.37	0.93	0	0	0	not significant
12770	KIM_GLIS2_TARGETS_DN	1.37	0.93	0	0	0	not significant
12771	GO_ALPHA_BETA_T_CELL_LINEAGE_COMMITMENT	1.37	0.91	0	0	0	not significant
12772	MEISSNER_NPC_ICP_WITH_H3_UNMETHYLATED	1.37	0.91	0	0	0	not significant
12773	SPIELMAN_LYMPHOBLAST_EUROPEAN_VS_ASIAN_2FC_DN	1.37	0.89	0	0	0	not significant
12774	SHIN_B_CELL_LYMPHOMA_CLUSTER_5	1.37	0.88	0	0	0	not significant
12775	GOBERT_OLIGODENDROCYTE_DIFFERENTIATION_DN	1.38	2.91	0	0	0	not significant
12776	BENPORATH_SUZ12_TARGETS	1.38	2.04	0	0	0	not significant
12777	CHR5Q35	1.38	1.34	0	0	0	not significant
12778	GO_REGULATION_OF_CARBOHYDRATE_BIOSYNTHETIC_PROCESS	1.38	1.29	0	0	0	not significant
12779	BOYLAN_MULTIPLE_MYELOMA_PCA3_UP	1.38	1.28	0	0	0	not significant
12780	LEIN_CEREBELLUM_MARKERS	1.38	1.15	0	0	0	not significant
12781	REACTOME_CONSTITUTIVE_SIGNALING_BY_ABERRANT_PI3K_IN_CANCEF	1.38	1.13	0	0	0	not significant
12782	GO_POTASSIUM_CHLORIDE_SYMPORTER_ACTIVITY	1.38	1.11	0	0	0	not significant
12783	GO_PROTEOGLYCAN_BIOSYNTHETIC_PROCESS	1.38	1.06	0	0	0	not significant
12784	GO_CELL_DIFFERENTIATION_IN_HINDBRAIN	1.38	1.00	0	0	0	not significant
12785	GO POSITIVE REGULATION OF T HELPER 17 CELL DIFFERENTIATION	1.38	0.99	0	0	0	not significant
12786	GO_GLYCOLIPID_BINDING	1.38	0.98	0	0	0	not significant
12787	GO_RENAL_VESICLE_DEVELOPMENT	1.38	0.98	0	0	0	not significant
12788	CHR2P24	1.38	0.97	0	0	0	not significant
12789	GO_DNA_REPLICATION_CHECKPOINT	1.38	0.97	0	0	0	not significant
12790	GO_OLIGOSACCHARIDE_CATABOLIC_PROCESS	1.38	0.96	0	0	0	not significant
12791	GO_POSITIVE_REGULATION_OF_EPITHELIAL_CELL_APOPTOTIC_PROCE:	1.38	0.96	0	0	0	not significant
12792	FOURNIER_ACINAR_DEVELOPMENT_EARLY_DN	1.38	0.95	0	0	0	not significant
12793	GO_NEUROTRANSMITTER_RECEPTOR_ACTIVITY	1.38	0.94	0	0	0	not significant
12794	REACTOME_SIGNALING_BY_ERYTHROPOIETIN	1.38	0.94	0	0	0	not significant
12795	GO_NEURAL_PLATE_PATTERN_SPECIFICATION	1.38	0.93	0	0	0	not significant
12796	GO_PHOSPHATIDYLETHANOLAMINE_ACYL_CHAIN_REMODELING	1.38	0.92	0	0	0	not significant
12797	REACTOME_ACYL_CHAIN_REMODELLING_OF_PE	1.38	0.92	0	0	0	not significant
12798	HOFMANN_MYELODYSPLASTIC_SYNDROM_HIGH_RISK_UP	1.38	0.91	0	0	0	not significant
12799	KEGG_GLYCOSPHINGOLIPID_BIOSYNTHESIS_GANGLIO_SERIES	1.38	0.83	0	0	0	not significant
12800	IVANOVA_HEMATOPOIESIS_STEM_CELL_AND_PROGENITOR	1.39	2.30	0	0	0	not significant
12801	GO_DNA_BINDING_TRANSCRIPTION_REPRESSOR_ACTIVITY_RNA_POLYM	1.39	1.73	0	0	0	not significant
12802	YAUCH_HEDGEHOG_SIGNALING_PARACRINE_DN	1.39	1.41	0	0	0	not significant
12803	DIAZ_CHRONIC_MEYLOGENOUS_LEUKEMIA_DN	1.39	1.33	0	0	0	not significant
12804	MORI_PRE_BI_LYMPHOCYTE_DN	1.39	1.33	0	0	0	not significant
12805	GO_METHYLATED_HISTONE_BINDING	1.39	1.22	0	0	0	not significant
12806	IL21_UP.V1_DN	1.39	1.22	0	0	0	not significant
12807	GO_REGULATION_OF_NEURON_PROJECTION_ARBORIZATION	1.39	1.20	0	0	0	not significant
12808	GO CHROMOSOME ORGANIZATION INVOLVED IN MEIOTIC CELL CYCL	1.39	1.18	0	0	0	not significant
12809	GO_HETEROTYPIC_CELL_CELL_ADHESION	1.39	1.18	0	0	0	not significant
12810	REACTOME_SIGNALING_BY_SCF_KIT	1.39	1.12	0	0	0	not significant
12811	GO_NUCLEAR_MIGRATION	1.39	1.08	0	0	0	not significant
12812	GO_GOLGI_CIS_CISTERNA	1.39	1.07	0	0	0	not significant
12813	GO_METANEPHRIC_RENAL_VESICLE_MORPHOGENESIS	1.39	1.06	0	0	0	not significant
12814	GO_CYTOPLASMIC_SIDE_OF_ENDOPLASMIC_RETICULUM_MEMBRANE	1.39	1.05	0	0	0	not significant
12815	GO_TRANSMITTER_GATED_CHANNEL_ACTIVITY	1.39	1.05	0	0	0	not significant
12816	BIOCARTA_NOS1_PATHWAY	1.39	1.04	0	0	0	not significant
12817	GO_NEGATIVE_REGULATION_OF_ADAPTIVE_IMMUNE_RESPONSE	1.39	1.04	0	0	0	not significant
12818	CHO_NR4A1_TARGETS	1.39	1.03	0	0	0	not significant
12819	GO_T_HELPER_17_CELL_LINEAGE_COMMITMENT	1.39	1.01	0	0	0	not significant
12820	GO_LIPID_ANTIGEN_BINDING	1.39	1.00	0	0	0	not significant
12821	REACTOME_PI_3K_CASCADE:FGFR1	1.39	0.99	0	0	0	not significant
12822	VALK_AML_WITH_EVI1	1.39	0.99	0	0	0	not significant
12823	BIOCARTA_CD40_PATHWAY	1.39	0.98	0	0	0	not significant
12824	GO_NEURON_PROJECTION_ARBORIZATION	1.39	0.97	0	0	0	not significant
12825	REACTOME_INTERLEUKIN_20_FAMILY_SIGNALING	1.39	0.97	0	0	0	not significant
12826	MEISSNER_NPC_HCP_WITH_H3K27ME3	1.39	0.96	0	0	0	not significant
12827	PID_IL27_PATHWAY	1.39	0.96	0	0	0	not significant
12828	VERRECCHIA_RESPONSE_TO_TGFB1_C5	1.39	0.96	0	0	0	not significant
12829	GO ANTIGEN PROCESSING AND PRESENTATION ENDOGENOUS LIPID	1.39	0.95	0	0	0	not significant
12830	GARGALOVIC_RESPONSE_TO_OXIDIZED_PHOSPHOLIPIDS_GREY_UP	1.39	0.93	0	0	0	not significant
12831	GO_HISTONE_H2A_K119_MONOUBIQUITINATION	1.39	0.92	0	0	0	not significant
12832	GO_PROTEIN_TYROSINE_KINASE_ACTIVITY	1.40	1.68	0	0	0	not significant
12833	GO_ALPHA_BETA_T_CELL_DIFFERENTIATION	1.40	1.39	0	0	0	not significant
12834	GO_RECEPTOR_SIGNALING_PATHWAY_VIA_STAT	1.40	1.39	0	0	0	not significant
12835	BARRIER_CANCER_RELAPSE_NORMAL_SAMPLE_DN	1.40	1.20	0	0	0	not significant
12836	KEGG_NEUROACTIVE_LIGAND_RECEPTOR_INTERACTION	1.40	1.20	0	0	0	not significant
12837	GO_NEGATIVE_REGULATION_OF_LEUKOCYTE_MEDIATED_IMMUNITY	1.40	1.18	0	0	0	not significant
12838	GO_ENDOCARDIAL_CUSHION_FORMATION	1.40	1.14	0	0	0	not significant
12839	GO REGULATION OF DOUBLE STRAND BREAK REPAIR VIA HOMOLOC	1.40	1.14	0	0	0	not significant
12840	POMEROY_MEDULLOBLASTOMA_DESMOPLASIC_VS_CLASSIC_DN	1.40	1.14	0	0	0	not significant
12841	GO_PROTEIN_RETENTION_IN_GOLGI_APPARATUS	1.40	1.12	0	0	0	not significant
12842	GO_CLATHRIN_SCULPTED_VESICLE	1.40	1.10	0	0	0	not significant
12843	GO_SYNAPTIC_VESICLE_CLUSTERING	1.40	1.10	0	0	0	not significant
12844	VISALA_RESPONSE_TO_HEAT_SHOCK_AND_AGING_UP	1.40	1.09	0	0	0	not significant
12845	GO_DIENCEPHALON_DEVELOPMENT	1.40	1.08	0	0	0	not significant
12846	MEDINA_SMARCA4_TARGETS	1.40	1.08	0	0	0	not significant
12847	GO_REGULATION_OF_CD40_SIGNALING_PATHWAY	1.40	1.07	0	0	0	not significant
12848	GO_T_CELL_LINEAGE_COMMITMENT	1.40	1.07	0	0	0	not significant
12849	REACTOME_CELL_CELL_JUNCTION_ORGANIZATION	1.40	1.07	0	0	0	not significant
12850	GO_ALPHA_TUBULIN_BINDING	1.40	1.06	0	0	0	not significant

12851	GO_TOOTH_MINERALIZATION	1.40	1.04	0	0	0	not significant
12852	REACTOME_TNF_RECEPTOR_SUPERFAMILY_TNFSF_MEMBERS_MEDIAT	1.40	1.04	0	0	0	not significant
12853	GO_NEPHRON_TUBULE_FORMATION	1.40	1.03	0	0	0	not significant
12854	GO_CO_SMAD_BINDING	1.40	1.02	0	0	0	not significant
12855	MARIADASON_RESPONSE_TO_BUTYRATE_CURCUMIN_SULINDAC_TSA_1	1.40	1.02	0	0	0	not significant
12856	GO_EPIDERMIS_MORPHOGENESIS	1.40	1.01	0	0	0	not significant
12857	GO_PROTEIN_LOCALIZATION_TO_PHAGOPHORE_ASSEMBLY_SITE	1.40	1.01	0	0	0	not significant
12858	GO_INTEGRAL_COMPONENT_OF_CYTOPLASMIC_SIDE_OF_ENDOPLASMI	1.40	1.00	0	0	0	not significant
12859	GARGALOVIC_RESPONSE_TO_OXIDIZED_PHOSPHOLIPIDS_PURPLE_DN	1.40	0.99	0	0	0	not significant
12860	CASORELLI_APL_SECONDARY_VS_DE_NOVO_DN	1.40	0.98	0	0	0	not significant
12861	CHR1P33	1.40	0.96	0	0	0	not significant
12862	GO_PHOSPHOLIPASE_A2_ACTIVITY_CONSUMING_1_2_DIPALMITOYLPHOS	1.40	0.95	0	0	0	not significant
12863	GO_DNA_STRAND_RENATURATION	1.40	0.94	0	0	0	not significant
12864	GO_PYRIMIDINE_NUCLEOTIDE_METABOLIC_PROCESS	1.40	0.93	0	0	0	not significant
12865	GO_AUDITORY_RECEPTOR_CELL_DEVELOPMENT	1.40	0.89	0	0	0	not significant
12866	PEREZ_TP53_TARGETS	1.41	2.90	0	0	0	not significant
12867	GO_VASCULOGENESIS	1.41	1.43	0	0	0	not significant
12868	IWANAGA_CARCINOGENESIS_BY_KRAS_DN	1.41	1.40	0	0	0	not significant
12869	GO_NUCLEAR_INNER_MEMBRANE	1.41	1.25	0	0	0	not significant
12870	CHR15Q21	1.41	1.20	0	0	0	not significant
12871	BANDRES_RESPONSE_TO_CARMUSTIN_WITHOUT_MGMT_48HR_UP	1.41	1.17	0	0	0	not significant
12872	GO_ICOSANOID_METABOLIC_PROCESS	1.41	1.17	0	0	0	not significant
12873	GO_REGULATION_OF_EPITHELIAL_CELL_APOPTOTIC_PROCESS	1.41	1.14	0	0	0	not significant
12874	REACTOME_ADHERENS_JUNCTIONS_INTERACTIONS	1.41	1.12	0	0	0	not significant
12875	REACTOME_INTERLEUKIN_2_FAMILY_SIGNALING	1.41	1.10	0	0	0	not significant
12876	REACTOME_ONCOGENE_INDUCED_SENESCENCE	1.41	1.10	0	0	0	not significant
12877	GO_HISTONE_ACETYLTRANSFERASE_ACTIVITY_H4_K5_SPECIFIC_	1.41	1.09	0	0	0	not significant
12878	GO_TRANSMISSION_OF_NERVE_IMPULSE	1.41	1.09	0	0	0	not significant
12879	ABRAHAM_ALPC_VS_MULTIPLE_MYELOMA_UP	1.41	1.08	0	0	0	not significant
12880	GO_EMBRYONIC_BRAIN_DEVELOPMENT	1.41	1.05	0	0	0	not significant
12881	REACTOME_SIGNALING_BY_NOTCH1_HD_DOMAIN_MUTANTS_IN_CANCEF	1.41	1.05	0	0	0	not significant
12882	GO_POSITIVE_REGULATION_OF_INTRINSIC_APOPTOTIC_SIGNALING_PAT	1.41	1.04	0	0	0	not significant
12883	GO_REGULATION_OF_SYNAPTIC_TRANSMISSION_GABAERGIC	1.41	1.04	0	0	0	not significant
12884	GO_ENGULFMENT_OF_APOPTOTIC_CELL	1.41	1.03	0	0	0	not significant
12885	GO_REGULATION_OF_METALLOPEPTIDASE_ACTIVITY	1.41	1.02	0	0	0	not significant
12886	BERENJENO_TRANSFORMED_BY_RHOA_FOREVER_UP	1.41	1.01	0	0	0	not significant
12887	GO_AMELOGENESIS	1.41	1.01	0	0	0	not significant
12888	BIOCARTA_RACC_PATHWAY	1.41	1.00	0	0	0	not significant
12889	GO_ACID_AMINO_ACID_LIGASE_ACTIVITY	1.41	0.99	0	0	0	not significant
12890	GO_ATRIAL_SEPTUM_MORPHOGENESIS	1.41	0.97	0	0	0	not significant
12891	GO_REGULATION_OF_CD8_POSITIVE_ALPHA_BETA_T_CELL_ACTIVATIOI	1.41	0.96	0	0	0	not significant
12892	BIOCARTA_AKAP13_PATHWAY	1.41	0.94	0	0	0	not significant
12893	DODD_NASOPHARYNGEAL_CARCINOMA_UP	1.42	4.00	0	0	0	not significant
12894	TARTE_PLASMA_CELL_VS_PLASMABLAST_UP	1.42	2.08	0	0	0	not significant
12895	GO_ANATOMICAL_STRUCTURE_MATURATION	1.42	1.59	0	0	0	not significant
12896	KUNINGER_IGF1_VS_PDGFB_TARGETS_UP	1.42	1.36	0	0	0	not significant
12897	GO_LIPOSACCHARIDE_METABOLIC_PROCESS	1.42	1.33	0	0	0	not significant
12898	GO_AZUROPHIL_GRANULE_MEMBRANE	1.42	1.30	0	0	0	not significant
12899	GO_PEPTIDYL_TYROSINE_AUTOPHOSPHORYLATION	1.42	1.24	0	0	0	not significant
12900	MOREAUX_B_LYMPHOCYTE_MATURATION_BY_TACI_UP	1.42	1.24	0	0	0	not significant
12901	GO_REGULATION_OF_TRANSCRIPTION_INVOLVED_IN_G1_S_TRANSITION	1.42	1.21	0	0	0	not significant
12902	GO_ACYLGLYCEROL_METABOLIC_PROCESS	1.42	1.17	0	0	0	not significant
12903	GO_SCHAFFER_COLLATERAL__CA1_SYNAPSE	1.42	1.17	0	0	0	not significant
12904	GO_ORGANISM_EMERGENCE_FROM_PROTECTIVE_STRUCTURE	1.42	1.14	0	0	0	not significant
12905	GO_MONOCYTE_ACTIVATION	1.42	1.10	0	0	0	not significant
12906	GO_REGULATION_OF_CYTOLYSIS	1.42	1.09	0	0	0	not significant
12907	KEGG_GLYCOSAMINOGLYCAN_BIOSYNTHESIS_CHONDROITIN_SULFATE	1.42	1.09	0	0	0	not significant
12908	GO_GPI_ANCHOR_BINDING	1.42	1.08	0	0	0	not significant
12909	GO_NEGATIVE_REGULATION_OF_ENDOTHELIAL_CELL_APOPTOTIC_PRO	1.42	1.05	0	0	0	not significant
12910	GO_MODULATION_OF_PROGRAMMED_CELL_DEATH_IN_OTHER_ORGANI	1.42	1.00	0	0	0	not significant
12911	GO_RESPONSE_TO_PAIN	1.42	0.96	0	0	0	not significant
12912	NAKAMURA_TUMOR_ZONE_PERIPHERAL_VS_CENTRAL_DN	1.43	2.88	0	0	0	not significant
12913	RICKMAN_TUMOR_DIFFERENTIATED_WELL_VS_POORLY_UP	1.43	2.10	0	0	0	not significant
12914	VERHAAK_GLIOBLASTOMA_PRONEURAL	1.43	1.66	0	0	0	not significant
12915	GO_CATION_CHANNEL_COMPLEX	1.43	1.55	0	0	0	not significant
12916	BOYLAN_MULTIPLE_MYELOMA_D_DN	1.43	1.42	0	0	0	not significant
12917	KOBAYASHI_EGFR_SIGNALING_24HR_UP	1.43	1.39	0	0	0	not significant
12918	SABATES_COLORECTAL_ADENOMA_DN	1.43	1.37	0	0	0	not significant
12919	GO_REGULATION_OF_NEUROLOGICAL_SYSTEM_PROCESS	1.43	1.33	0	0	0	not significant
12920	GO_T_CELL_DIFFERENTIATION_INVOLVED_IN_IMMUNE_RESPONSE	1.43	1.33	0	0	0	not significant
12921	GO_CD8_POSITIVE_ALPHA_BETA_T_CELL_ACTIVATION	1.43	1.20	0	0	0	not significant
12922	REACTOME_INTERLEUKIN_15_SIGNALING	1.43	1.20	0	0	0	not significant
12923	GO_MESENCHYME_MORPHOGENESIS	1.43	1.17	0	0	0	not significant
12924	GO_REGULATION_OF_NUCLEAR_CELL_CYCLE_DNA_REPLICATION	1.43	1.17	0	0	0	not significant
12925	GO_DELAYED_RECTIFIER_POTASSIUM_CHANNEL_ACTIVITY	1.43	1.15	0	0	0	not significant
12926	GO_POSTSYNAPTIC_SPECIALIZATION_ORGANIZATION	1.43	1.11	0	0	0	not significant
12927	GO_NITRIC_OXIDE_MEDIATED_SIGNAL_TRANSDUCTION	1.43	1.10	0	0	0	not significant
12928	GO_VACUOLAR_PROTON_TRANSPORTING_V_TYPE_ATPASE_COMPLEX	1.43	1.10	0	0	0	not significant
12929	REACTOME_ACTIVATED_NOTCH1_TRANSMITS_SIGNAL_TO_THE_NUCLEU	1.43	1.10	0	0	0	not significant
12930	GO_CD4_POSITIVE_ALPHA_BETA_T_CELL_LINEAGE_COMMITMENT	1.43	1.09	0	0	0	not significant
12931	GO_VOLUME_SENSITIVE_ANION_CHANNEL_ACTIVITY	1.43	1.09	0	0	0	not significant
12932	GO_CD40_SIGNALING_PATHWAY	1.43	1.08	0	0	0	not significant
12933	GO_REGULATION_OF_SENSORY_PERCEPTION	1.43	1.08	0	0	0	not significant
12934	GO_LIPOPEPTIDE_BINDING	1.43	1.07	0	0	0	not significant
12935	DAVICIONI_PAX_FOXO1_SIGNATURE_IN_ARMS_DN	1.43	1.04	0	0	0	not significant
12936	GO_PHOSPHATIDYLSERINE_BINDING	1.43	1.04	0	0	0	not significant
12937	GO_ISOTYPE_SWITCHING_TO_IGG_ISOTYPES	1.43	1.02	0	0	0	not significant
12938	THEODOROU_MAMMARY_TUMORIGENESIS	1.43	1.00	0	0	0	not significant
12939	GO_TRANSPORTER_COMPLEX	1.44	1.84	0	0	0	not significant
12940	MIKKELSEN_NPC_HCP_WITH_H3K27ME3	1.44	1.52	0	0	0	not significant
12941	SIG_CD40PATHWAYMAP	1.44	1.39	0	0	0	not significant
12942	MEISSNER_NPC_HCP_WITH_H3K4ME2_AND_H3K27ME3	1.44	1.38	0	0	0	not significant
12943	GO_POSITIVE_REGULATION_OF_RESPONSE_TO_CYTOKINE_STIMULUS	1.44	1.31	0	0	0	not significant
12944	REACTOME_PROTEIN_PROTEIN_INTERACTIONS_AT_SYNAPSES	1.44	1.31	0	0	0	not significant
12945	GO_REGULATION_OF_ENDOTHELIAL_CELL_DIFFERENTIATION	1.44	1.30	0	0	0	not significant
12946	PID_EPO_PATHWAY	1.44	1.29	0	0	0	not significant
12947	GHANDHI_BYSTANDER_IRRADIATION_UP	1.44	1.24	0	0	0	not significant
12948	REACTOME_POST_TRANSCRIPTIONAL_SILENCING_BY_SMALL_RNAS	1.44	1.17	0	0	0	not significant
12949	GO_CATECHOLAMINE_SECRETION	1.44	1.16	0	0	0	not significant
12950	GO_DERMATAN_SULFATE_PROTEOGLYCAN_BIOSYNTHETIC_PROCESS	1.44	1.16	0	0	0	not significant
12951	GO_GLYCOSPHINGOLIPID_BINDING	1.44	1.15	0	0	0	not significant
12952	GO_EXOCRINE_SYSTEM_DEVELOPMENT	1.44	1.14	0	0	0	not significant
12953	GO_POSITIVE_REGULATION_OF_INTERLEUKIN_4_PRODUCTION	1.44	1.14	0	0	0	not significant
12954	GO_CYTOPLASMIC_SIDE_OF_ROUGH_ENDOPLASMIC_RETICULUM_MEME	1.44	1.13	0	0	0	not significant
12955	MOTAMED_RESPONSE_TO_ANDROGEN_DN	1.44	1.13	0	0	0	not significant
12956	BIOCARTA_TH1TH2_PATHWAY	1.44	1.12	0	0	0	not significant
12957	SCHWAB_TARGETS_OF_BMYB_POLYMORPHIC_VARIANTS_DN	1.44	1.10	0	0	0	not significant
12958	GO_ATP_GATED_ION_CHANNEL_ACTIVITY	1.44	1.09	0	0	0	not significant
12959	GO_NEURONAL_ACTION_POTENTIAL	1.44	1.09	0	0	0	not significant

12960	REACTOME_N_GLYCAN_ANTENNAE_ELONGATION	1.44	1.09	0	0	0	not significant
12961	WORSCHECH_TUMOR_EVASION_AND_TOLEROGENICITY_UP	1.44	1.09	0	0	0	not significant
12962	TONKS_TARGETS_OF_RUNX1_RUNX1T1_FUSION_SUSTAINED_IN_MONOC	1.44	1.08	0	0	0	not significant
12963	HORIUCHI_WTAP_TARGETS_UP	1.45	1.97	0	0	0	not significant
12964	ESC_J1_UP_EARLY.V1_UP	1.45	1.75	0	0	0	not significant
12965	CHR7Q36	1.45	1.59	0	0	0	not significant
12966	GO_REGULATION_OF_EPITHELIAL_CELL_DIFFERENTIATION	1.45	1.48	0	0	0	not significant
12967	TIEN_INTESTINE_PROBIOTICS_6HR_UP	1.45	1.38	0	0	0	not significant
12968	GO_ECTODERM_DEVELOPMENT	1.45	1.29	0	0	0	not significant
12969	REACTOME_N_GLYCAN_ANTENNAE_ELONGATION_IN_THE_MEDIAL_TRAN	1.45	1.27	0	0	0	not significant
12970	JAZAERI_BREAST_CANCER_BRCA1_VS_BRCA2_DN	1.45	1.26	0	0	0	not significant
12971	GO_INTRINSIC_COMPONENT_OF_SYNAPTIC_VESICLE_MEMBRANE	1.45	1.24	0	0	0	not significant
12972	REACTOME_CHONDROITIN_SULFATE_DERMATAN_SULFATE_METABOLIS	1.45	1.22	0	0	0	not significant
12973	DELACROIX_RAR_TARGETS_DN	1.45	1.17	0	0	0	not significant
12974	GO_INHIBITORY_SYNAPSE_ASSEMBLY	1.45	1.16	0	0	0	not significant
12975	REACTOME_PI3K_EVENTS_IN_ERBB2_SIGNALING	1.45	1.14	0	0	0	not significant
12976	GO_CYTOSKELETAL_ANCHORING_AT_NUCLEAR_MEMBRANE	1.45	1.13	0	0	0	not significant
12977	GO_REGULATION_OF_VASCULAR_PERMEABILITY	1.45	1.13	0	0	0	not significant
12978	PASTURAL_RIZ1_TARGETS_UP	1.45	1.12	0	0	0	not significant
12979	GO_RESPONSE_TO_UV_C	1.45	1.11	0	0	0	not significant
12980	OSADA_ASCL1_TARGETS_DN	1.45	1.11	0	0	0	not significant
12981	GO_REGULATION_OF_AMPA_RECEPTOR_ACTIVITY	1.45	1.10	0	0	0	not significant
12982	TIAN_BHLHA15_TARGETS	1.45	1.10	0	0	0	not significant
12983	GO_PIRNA_METABOLIC_PROCESS	1.45	1.08	0	0	0	not significant
12984	KOBAYASHI_RESPONSE_TO_ROMIDEPSIN	1.45	1.06	0	0	0	not significant
12985	GO_OXIDIZED_DNA_BINDING	1.45	1.00	0	0	0	not significant
12986	KRIGE_RESPONSE_TO_TOSEDOSTAT_24HR_UP	1.46	4.00	0	0	0	not significant
12987	GO_HYDROLASE_ACTIVITY_ACTING_ON_GLYCOSYL_BONDS	1.46	1.61	0	0	0	not significant
12988	GO_NEGATIVE_REGULATION_OF_TYPE_I_INTERFERON_PRODUCTION	1.46	1.46	0	0	0	not significant
12989	GO_RESPONSE_TO_CADMIUM_ION	1.46	1.43	0	0	0	not significant
12990	KLEIN_TARGETS_OF_BCR_ABL1_FUSION	1.46	1.38	0	0	0	not significant
12991	GO_ANNEALING_HELICASE_ACTIVITY	1.46	1.37	0	0	0	not significant
12992	GO_PCG_PROTEIN_COMPLEX	1.46	1.30	0	0	0	not significant
12993	PID_ERBB4_PATHWAY	1.46	1.28	0	0	0	not significant
12994	GO_INTERLEUKIN_4_PRODUCTION	1.46	1.27	0	0	0	not significant
12995	GO_NEGATIVE_REGULATION_OF_CYTOKINE_BIOSYNTHETIC_PROCESS	1.46	1.26	0	0	0	not significant
12996	SAGIV_CD24_TARGETS_DN	1.46	1.25	0	0	0	not significant
12997	GO_POSITIVE_REGULATION_OF_T_HELPER_17_TYPE_IMMUNE_RESPONS	1.46	1.23	0	0	0	not significant
12998	GAVIN_PDE3B_TARGETS	1.46	1.22	0	0	0	not significant
12999	GO_INTERLEUKIN_7_MEDIATED_SIGNALING_PATHWAY	1.46	1.22	0	0	0	not significant
13000	GO_POSITIVE_REGULATION_OF_GLYCOGEN_METABOLIC_PROCESS	1.46	1.22	0	0	0	not significant
13001	MAHADEVAN_RESPONSE_TO_MP470_DN	1.46	1.21	0	0	0	not significant
13002	GO_CD4_POSITIVE_OR_CD8_POSITIVE_ALPHA_BETA_T_CELL_LINEAGE_	1.46	1.18	0	0	0	not significant
13003	GO_CELLULAR_RESPONSE_TO_SALT	1.46	1.17	0	0	0	not significant
13004	KEGG_GLYCOSPHINGOLIPID_BIOSYNTHESIS_GLOBO_SERIES	1.46	1.17	0	0	0	not significant
13005	GO_REGULATION_OF_FIBROBLAST_GROWTH_FACTOR_RECEPTOR_SIG	1.46	1.16	0	0	0	not significant
13006	TRAYNOR_RETT_SYNDROM_UP	1.46	1.16	0	0	0	not significant
13007	REACTOME_NICOTINAMIDE_SALVAGING	1.46	1.15	0	0	0	not significant
13008	GO_COCHLEA_MORPHOGENESIS	1.46	1.13	0	0	0	not significant
13009	NABA_PROTEOGLYCANS	1.46	1.13	0	0	0	not significant
13010	GO_DEVELOPMENTAL_INDUCTION	1.46	1.12	0	0	0	not significant
13011	GO_NUCLEAR_TRANSCRIBED_MRNA_CATABOLIC_PROCESS	1.47	2.25	0	0	0	not significant
13012	REACTOME_INFLUENZA_INFECTION	1.47	1.99	0	0	0	not significant
13013	GO_REGULATION_OF_CELLULAR_CARBOHYDRATE_METABOLIC_PROCE	1.47	1.71	0	0	0	not significant
13014	BHAT_ESR1_TARGETS_VIA_AKT1_DN	1.47	1.58	0	0	0	not significant
13015	FLOTHO_PEDIATRIC_ALL_THERAPY_RESPONSE_UP	1.47	1.41	0	0	0	not significant
13016	GO_ENAMEL_MINERALIZATION	1.47	1.39	0	0	0	not significant
13017	BRCA1_DN.V1_UP	1.47	1.34	0	0	0	not significant
13018	GO_CHLORIDE_CHANNEL_COMPLEX	1.47	1.33	0	0	0	not significant
13019	PID_FGF_PATHWAY	1.47	1.31	0	0	0	not significant
13020	GO_REGULATION_OF_NATURAL_KILLER_CELL_DIFFERENTIATION	1.47	1.30	0	0	0	not significant
13021	PID_S1P_META_PATHWAY	1.47	1.29	0	0	0	not significant
13022	GO_REGULATION_OF_HEART_RATE_BY_CARDIAC_CONDUCTION	1.47	1.25	0	0	0	not significant
13023	POS_RESPONSE_TO_HISTAMINE_DN	1.47	1.25	0	0	0	not significant
13024	REACTOME_DOWNSTREAM_SIGNAL_TRANSDUCTION	1.47	1.25	0	0	0	not significant
13025	GO_POSITIVE_REGULATION_OF_METALLOPEPTIDASE_ACTIVITY	1.47	1.24	0	0	0	not significant
13026	GO_POSITIVE_REGULATION_OF_TRANSFORMING_GROWTH_FACTOR_BI	1.47	1.22	0	0	0	not significant
13027	WONG_ENDMETRIUM_CANCER_DN	1.47	1.20	0	0	0	not significant
13028	GO_H3_HISTONE_ACETYLTRANSFERASE_COMPLEX	1.47	1.18	0	0	0	not significant
13029	GO_SUBSTRATE_DEPENDENT_CELL_MIGRATION_CELL_EXTENSION	1.47	1.16	0	0	0	not significant
13030	AIGNER_ZEB1_TARGETS	1.47	1.15	0	0	0	not significant
13031	GO_POSITIVE_REGULATION_OF_DNA_DEPENDENT_DNA_REPLICATION	1.47	1.09	0	0	0	not significant
13032	GO_INOSITOL_LIPID_MEDIATED_SIGNALING	1.48	1.87	0	0	0	not significant
13033	KEGG_HEMATOPOIETIC_CELL_LINEAGE	1.48	1.48	0	0	0	not significant
13034	GO_REGULATION_OF_B_CELL_MEDIATED_IMMUNITY	1.48	1.41	0	0	0	not significant
13035	NOUZOVA_METHYLATED_IN_APL	1.48	1.39	0	0	0	not significant
13036	GO_NSL_COMPLEX	1.48	1.35	0	0	0	not significant
13037	GO_REGULATION_OF_HUMORAL_IMMUNE_RESPONSE_MEDIATED_BY_CI	1.48	1.32	0	0	0	not significant
13038	MA_PITUITARY_FETAL_VS_ADULT_UP	1.48	1.29	0	0	0	not significant
13039	VALK_AML_CLUSTER_4	1.48	1.27	0	0	0	not significant
13040	GO_REGULATION_OF_MICROTUBULE_BASED_MOVEMENT	1.48	1.25	0	0	0	not significant
13041	KEGG_OTHER_GLYCAN_DEGRADATION	1.48	1.23	0	0	0	not significant
13042	CHR6Q13	1.48	1.22	0	0	0	not significant
13043	REACTOME_INTERLEUKIN_21_SIGNALING	1.48	1.21	0	0	0	not significant
13044	GO_CAMP_BINDING	1.48	1.14	0	0	0	not significant
13045	CHANG_CORE_SERUM_RESPONSE_DN	1.49	2.15	0	0	0	not significant
13046	GO_POSITIVE_REGULATION_OF_EPITHELIAL_CELL_PROLIFERATION	1.49	1.86	0	0	0	not significant
13047	GO_PHOSPHATIDYLINOSITOL_3_KINASE_SIGNALING	1.49	1.64	0	0	0	not significant
13048	TAKEDA_TARGETS_OF_NUP98_HOXA9_FUSION_16D_DN	1.49	1.58	0	0	0	not significant
13049	PID_IL4_2PATHWAY	1.49	1.49	0	0	0	not significant
13050	CHR5Q22	1.49	1.45	0	0	0	not significant
13051	GO_LONG_TERM_SYNAPTIC_POTENTIATION	1.49	1.43	0	0	0	not significant
13052	SOTIRIOU_BREAST_CANCER_GRADE_1_VS_3_DN	1.49	1.42	0	0	0	not significant
13053	GO_REGULATION_OF_INTERLEUKIN_4_PRODUCTION	1.49	1.39	0	0	0	not significant
13054	REACTOME_GROWTH_HORMONE_RECEPTOR_SIGNALING	1.49	1.36	0	0	0	not significant
13055	REACTOME_SIGNALING_BY_CYTOSOLIC_FGFR1_FUSION_MUTANTS	1.49	1.28	0	0	0	not significant
13056	GO_POSITIVE_REGULATION_OF_RHO_PROTEIN_SIGNAL_TRANSDUCTION	1.49	1.27	0	0	0	not significant
13057	GO_REGULATION_OF_MESENCHYMAL_CELL_PROLIFERATION	1.49	1.26	0	0	0	not significant
13058	IIZUKA_LIVER_CANCER_EARLY_RECURRENCE	1.49	1.25	0	0	0	not significant
13059	KEGG_SPHINGOLIPID_METABOLISM	1.49	1.25	0	0	0	not significant
13060	GO_POSITIVE_REGULATION_OF_NATURAL_KILLER_CELL_ACTIVATION	1.49	1.23	0	0	0	not significant
13061	LU_TUMOR_ANGIOGENESIS_UP	1.49	1.22	0	0	0	not significant
13062	GO_COBALAMIN_TRANSPORT	1.49	1.21	0	0	0	not significant
13063	GO_ANION_CATION_SYMPORTER_ACTIVITY	1.49	1.20	0	0	0	not significant
13064	GO_DENDRITIC_SPINE_MAINTENANCE	1.49	1.20	0	0	0	not significant
13065	GO_ESTABLISHMENT_OF_PROTEIN_LOCALIZATION_TO_MEMBRANE	1.50	2.85	0	0	0	Enhanced
13066	KRAS.600_UP.V1_DN	1.50	2.04	0	0	0	Enhanced
13067	REACTOME_INTERLEUKIN_7_SIGNALING	1.50	1.39	0	0	0	Enhanced
13068	REACTOME_CREB3_FACTORS_ACTIVATE_GENES	1.50	1.36	0	0	0	Enhanced

13069	GO_TOLERANCE_INDUCTION	1.50	1.34	0	0	0	Enhanced
13070	SCHRAMM_INHBA_TARGETS_DN	1.50	1.28	0	0	0	Enhanced
13071	HOLLEMAN_VINCRISTINE_RESISTANCE_B_ALL_DN	1.50	1.21	0	0	0	Enhanced
13072	REACTOME_NUCLEAR_SIGNALING_BY_ERBB4	1.50	1.20	0	0	0	Enhanced
13073	GO_GOLGI_CISTERNA	1.51	1.84	0	0	0	Enhanced
13074	P53_DN.V2_UP	1.51	1.80	0	0	0	Enhanced
13075	GO_POSITIVE_REGULATION_OF_ENDOTHELIAL_CELL_PROLIFERATION	1.51	1.77	0	0	0	Enhanced
13076	GO_SPINAL_CORD_DEVELOPMENT	1.51	1.68	0	0	0	Enhanced
13077	GO_LYMPHOCYTE_COSTIMULATION	1.51	1.60	0	0	0	Enhanced
13078	GO_REGULATION_OF_PLATELET_ACTIVATION	1.51	1.50	0	0	0	Enhanced
13079	GO_PROTEIN_C_LINKED_GLYCOSYLATION	1.51	1.48	0	0	0	Enhanced
13080	GO_DOPAMINE_SECRETION	1.51	1.38	0	0	0	Enhanced
13081	GO_POSTSYNAPTIC_ENDOSOME	1.51	1.36	0	0	0	Enhanced
13082	YAGUE_PRETUMOR_DRUG_RESISTANCE_DN	1.51	1.36	0	0	0	Enhanced
13083	GO_LATERAL_MESODERM_DEVELOPMENT	1.51	1.30	0	0	0	Enhanced
13084	GO_ESTROGEN_BIOSYNTHETIC_PROCESS	1.51	1.27	0	0	0	Enhanced
13085	GO_CATION_CHLORIDE_SYMPORTER_ACTIVITY	1.51	1.26	0	0	0	Enhanced
13086	GO_NEGATIVE_REGULATION_OF_INTERLEUKIN_10_PRODUCTION	1.51	1.21	0	0	0	Enhanced
13087	GO_NEGATIVE_REGULATION_OF_CYTOKINE_PRODUCTION	1.52	2.55	0	0	0	Enhanced
13088	CHR16P13	1.52	2.38	0	0	0	Enhanced
13089	MARTORIATI_MDM4_TARGETS_NEUROEPITHELIUM_DN	1.52	2.10	0	0	0	Enhanced
13090	PDGF_UP.V1_DN	1.52	1.83	0	0	0	Enhanced
13091	GO_REGULATION_OF_POSTSYNAPTIC_NEUROTRANSMITTER_RECEPTOI	1.52	1.47	0	0	0	Enhanced
13092	LOPES_METHYLATED_IN_COLON_CANCER_UP	1.52	1.44	0	0	0	Enhanced
13093	MEISSNER_BRAIN_HCP_WITH_H3K4ME2_AND_H3K27ME3	1.52	1.42	0	0	0	Enhanced
13094	BIOCARTA_BLYMPHOCYTE_PATHWAY	1.52	1.40	0	0	0	Enhanced
13095	GO_REGULATION_OF_NEUROTRANSMITTER_RECEPTOR_ACTIVITY	1.52	1.39	0	0	0	Enhanced
13096	GO_MITOTIC_G2_DNA_DAMAGE_CHECKPOINT	1.52	1.33	0	0	0	Enhanced
13097	GO_CHLORIDE_ION_HOMEOSTASIS	1.52	1.28	0	0	0	Enhanced
13098	REACTOME_REGULATION_OF_PTEN_MRNA_TRANSLATION	1.52	1.28	0	0	0	Enhanced
13099	REACTOME_CREB1_PHOSPHORYLATION_THROUGH_NMDA_RECEPTOR	1.52	1.27	0	0	0	Enhanced
13100	CHRYQ11	1.52	1.26	0	0	0	Enhanced
13101	GERHOLD_RESPONSE_TO_TZD_DN	1.52	1.26	0	0	0	Enhanced
13102	NIELSEN_LEIOMYOSARCOMA_DN	1.52	1.26	0	0	0	Enhanced
13103	FERRANDO_T_ALL_WITH_MLL_ENL_FUSION_UP	1.53	2.11	0	0	0	Enhanced
13104	FERREIRA_EWINGS_SARCOMA_UNSTABLE_VS_STABLE_DN	1.53	1.95	0	0	0	Enhanced
13105	PECE_MAMMARY_STEM_CELL_UP	1.53	1.89	0	0	0	Enhanced
13106	BAKKER_FOXO3_TARGETS_UP	1.53	1.86	0	0	0	Enhanced
13107	CHR3Q27	1.53	1.64	0	0	0	Enhanced
13108	GO_ACTIVATION_OF_JANUS_KINASE_ACTIVITY	1.53	1.61	0	0	0	Enhanced
13109	GO_NEGATIVE_REGULATION_OF_INTERLEUKIN_6_SECRETION	1.53	1.60	0	0	0	Enhanced
13110	LE_SKI_TARGETS_UP	1.53	1.55	0	0	0	Enhanced
13111	ANDERSEN_LIVER_CANCER_KRT19_UP	1.53	1.54	0	0	0	Enhanced
13112	PHESSE_TARGETS_OF_APC_AND_MBD2_UP	1.53	1.53	0	0	0	Enhanced
13113	GO_MITOTIC_G2_M_TRANSITION_CHECKPOINT	1.53	1.52	0	0	0	Enhanced
13114	GO_IMMUNOLOGICAL_MEMORY_PROCESS	1.53	1.51	0	0	0	Enhanced
13115	DORSEY_GAB2_TARGETS	1.53	1.44	0	0	0	Enhanced
13116	GO_NUCLEUS_LOCALIZATION	1.53	1.41	0	0	0	Enhanced
13117	GO_REGULATION_OF_T_HELPER_17_TYPE_IMMUNE_RESPONSE	1.53	1.40	0	0	0	Enhanced
13118	REACTOME_GLYCOSPHINGOLIPID_METABOLISM	1.53	1.40	0	0	0	Enhanced
13119	GO_POSITIVE_REGULATION_OF_INTERLEUKIN_8_SECRETION	1.53	1.36	0	0	0	Enhanced
13120	LEE_DOUBLE_POLAR_THYMOCYTE	1.53	1.36	0	0	0	Enhanced
13121	GO_ESTABLISHMENT_OF_PLANAR_POLARITY_INVOLVED_IN_NEURAL_TU	1.53	1.34	0	0	0	Enhanced
13122	BIOCARTA_MCM_PATHWAY	1.53	1.32	0	0	0	Enhanced
13123	FISCHER_G1_S_CELL_CYCLE	1.54	2.83	0	0	0	Enhanced
13124	GO_REGULATION_OF_PHOSPHATIDYLINOSITOL_3_KINASE_SIGNALING	1.54	1.96	0	0	0	Enhanced
13125	GO_ANION_CHANNEL_ACTIVITY	1.54	1.82	0	0	0	Enhanced
13126	GO_CAMP_CATABOLIC_PROCESS	1.54	1.73	0	0	0	Enhanced
13127	GO_ENDOTHELIAL_CELL_APOPTOTIC_PROCESS	1.54	1.57	0	0	0	Enhanced
13128	GO_ESTRADIOL_17_BETA_DEHYDROGENASE_ACTIVITY	1.54	1.49	0	0	0	Enhanced
13129	GO_CHONDROITIN_SULFATE_PROTEOGLYCAN_BIOSYNTHETIC_PROCES	1.54	1.48	0	0	0	Enhanced
13130	GO_POSITIVE_REGULATION_OF_INTERLEUKIN_17_PRODUCTION	1.54	1.42	0	0	0	Enhanced
13131	HUMMEL_BURKITTS_LYMPHOMA_DN	1.54	1.41	0	0	0	Enhanced
13132	REACTOME_RAS_ACTIVATION_UPON_CA2PLUS_INFLUX_THROUGH_NMD/	1.54	1.41	0	0	0	Enhanced
13133	GO_MIDDLE_EAR_MORPHOGENESIS	1.54	1.40	0	0	0	Enhanced
13134	REACTOME_COMPETING_ENDOGENOUS_RNAS_CERNAS_REGULATE_PT	1.54	1.39	0	0	0	Enhanced
13135	GO_DEFINITIVE_HEMOPOIESIS	1.54	1.38	0	0	0	Enhanced
13136	GO_INTERFERON_GAMMA_SECRETION	1.54	1.32	0	0	0	Enhanced
13137	GO_VIRAL_GENE_EXPRESSION	1.55	4.00	0	0	0	Enhanced
13138	WHITFIELD_CELL_CYCLE_G1_S	1.55	2.52	0	0	0	Enhanced
13139	GO_INORGANIC_ANION_TRANSPORT	1.55	1.96	0	0	0	Enhanced
13140	BAE_BRCA1_TARGETS_DN	1.55	1.64	0	0	0	Enhanced
13141	GO_EXTRACELLULAR_LIGAND_GATED_ION_CHANNEL_ACTIVITY	1.55	1.63	0	0	0	Enhanced
13142	GO_SIGNAL_TRANSDUCTION_INVOLVED_IN_REGULATION_OF_GENE_EXF	1.55	1.61	0	0	0	Enhanced
13143	GO_PHOSPHOLIPASE_C_ACTIVATING_G_PROTEIN_COUPLED_RECEPTOI	1.55	1.58	0	0	0	Enhanced
13144	GO_SIALYLTRANSFERASE_ACTIVITY	1.55	1.55	0	0	0	Enhanced
13145	PID_ECADHERIN_KERATINOCYTE_PATHWAY	1.55	1.55	0	0	0	Enhanced
13146	GO_NATURAL_KILLER_CELL_PROLIFERATION	1.55	1.53	0	0	0	Enhanced
13147	GO_PHOSPHOLIPASE_A2_ACTIVITY	1.55	1.52	0	0	0	Enhanced
13148	LEE_LIVER_CANCER_CIPROFIBRATE_DN	1.55	1.50	0	0	0	Enhanced
13149	GO_CHONDROITIN_SULFATE_PROTEOGLYCAN_METABOLIC_PROCESS	1.55	1.49	0	0	0	Enhanced
13150	GO_DNA_METHYLATION_INVOLVED_IN_GAMETE_GENERATION	1.55	1.44	0	0	0	Enhanced
13151	GO_MANNOSIDASE_ACTIVITY	1.55	1.39	0	0	0	Enhanced
13152	GO_OSTEOBLAST_PROLIFERATION	1.55	1.35	0	0	0	Enhanced
13153	LEE_BMP2_TARGETS_UP	1.56	4.00	0	0	0	Enhanced
13154	ZWANG_TRANSIENTLY_UP_BY_2ND_EGF_PULSE_ONLY	1.56	4.00	0	0	0	Enhanced
13155	WOO_LIVER_CANCER_RECURRENCE_UP	1.56	2.02	0	0	0	Enhanced
13156	GO_SPHINGOLIPID_METABOLIC_PROCESS	1.56	1.84	0	0	0	Enhanced
13157	GO_POSITIVE_REGULATION_OF_NATURAL_KILLER_CELL_PROLIFERATIC	1.56	1.69	0	0	0	Enhanced
13158	CHR7Q31	1.56	1.65	0	0	0	Enhanced
13159	GO_GOLGI_CISTERNA_MEMBRANE	1.56	1.63	0	0	0	Enhanced
13160	REACTOME_UNBLOCKING_OF_NMDA_RECEPTORS_GLUTAMATE_BINDIN(	1.56	1.63	0	0	0	Enhanced
13161	GO_GAMMA_DELTA_T_CELL_DIFFERENTIATION	1.56	1.57	0	0	0	Enhanced
13162	GO_NEGATIVE_REGULATION_OF_INTERLEUKIN_2_PRODUCTION	1.56	1.54	0	0	0	Enhanced
13163	GO_INTERLEUKIN_10_PRODUCTION	1.56	1.53	0	0	0	Enhanced
13164	GO_POSITIVE_REGULATION_OF_TYROSINE_PHOSPHORYLATION_OF_ST	1.56	1.51	0	0	0	Enhanced
13165	GO_T_HELPER_17_TYPE_IMMUNE_RESPONSE	1.56	1.51	0	0	0	Enhanced
13166	GO_CHONDROITIN_SULFATE_BIOSYNTHETIC_PROCESS	1.56	1.50	0	0	0	Enhanced
13167	GO_TUMOR_NECROSIS_FACTOR_SECRETION	1.56	1.50	0	0	0	Enhanced
13168	GO_POSITIVE_REGULATION_OF_ISOTYPE_SWITCHING_TO_IGG_ISOTYPE	1.56	1.49	0	0	0	Enhanced
13169	CROONQUIST_IL6_DEPRIVATION_UP	1.56	1.45	0	0	0	Enhanced
13170	REACTOME_METABOLISM_OF_STEROID_HORMONES	1.56	1.45	0	0	0	Enhanced
13171	BIOCARTA_AMAN_PATHWAY	1.56	1.44	0	0	0	Enhanced
13172	GO_ATRIAL_SEPTUM_DEVELOPMENT	1.56	1.41	0	0	0	Enhanced
13173	GO_REGULATION_OF_EPITHELIAL_TUBE_FORMATION	1.56	1.40	0	0	0	Enhanced
13174	GO_TRANSFORMING_GROWTH_FACTOR_BETA_PRODUCTION	1.56	1.39	0	0	0	Enhanced
13175	REACTOME_SIGNALING_BY_FGFR1_IN_DISEASE	1.56	1.38	0	0	0	Enhanced
13176	GO_DESMOSOME	1.56	1.35	0	0	0	Enhanced
13177	CHR19Q13	1.57	4.00	0	0	0	Enhanced

13178	TAKEDA_TARGETS_OF_NUP98_HOXA9_FUSION_10D_UP	1.57	2.14	0	0	0	Enhanced
13179	MOLENAAR_TARGETS_OF_CCND1_AND_CDK4_UP	1.57	1.89	0	0	0	Enhanced
13180	GO_INORGANIC_ANION_TRANSMEMBRANE_TRANSPORT	1.57	1.87	0	0	0	Enhanced
13181	KANG_AR_TARGETS_UP	1.57	1.72	0	0	0	Enhanced
13182	GO_REGULATION_OF_CAMP_MEDIATED_SIGNALING	1.57	1.66	0	0	0	Enhanced
13183	GO_HISTONE_H4_K5_ACETYLATION	1.57	1.59	0	0	0	Enhanced
13184	GO_NEURAL_PLATE_DEVELOPMENT	1.57	1.50	0	0	0	Enhanced
13185	GO_TERMINAL_BOUTON	1.57	1.47	0	0	0	Enhanced
13186	IIZUKA_LIVER_CANCER_PROGRESSION_L1_G1_UP	1.57	1.47	0	0	0	Enhanced
13187	REACTOME_STAT5_ACTIVATION	1.57	1.44	0	0	0	Enhanced
13188	ZHENG_RESPONSE_TO_ARSENITE_UP	1.57	1.44	0	0	0	Enhanced
13189	GO_BONE_MATURATION	1.57	1.43	0	0	0	Enhanced
13190	GO_TRANSLATIONAL_INITIATION	1.58	2.83	0	0	0	Enhanced
13191	WU_CELL_MIGRATION	1.58	2.78	0	0	0	Enhanced
13192	GO_POSITIVE_REGULATION_OF_INTERFERON_GAMMA_SECRETION	1.58	1.95	0	0	0	Enhanced
13193	PYEON_HPV_POSITIVE_TUMORS_UP	1.58	1.82	0	0	0	Enhanced
13194	GO_ESTABLISHMENT_OF_PLANAR_POLARITY_OF_EMBRYONIC_EPITHEL	1.58	1.64	0	0	0	Enhanced
13195	GO_EXCITATORY_SYNAPSE	1.58	1.59	0	0	0	Enhanced
13196	GO_EXTRACELLULAR_MATRIX_STRUCTURAL_CONSTITUENT_CONFERRI	1.58	1.58	0	0	0	Enhanced
13197	KEGG_COMPLEMENT_AND_COAGULATION_CASCADES	1.58	1.58	0	0	0	Enhanced
13198	PID_NFKAPPAB_ATYPICAL_PATHWAY	1.58	1.58	0	0	0	Enhanced
13199	GO_NEGATIVE_REGULATION_OF_CARDIAC_MUSCLE_CELL_PROLIFERAT	1.58	1.56	0	0	0	Enhanced
13200	BIOCARTA_IL7_PATHWAY	1.58	1.53	0	0	0	Enhanced
13201	GO_SIALYLATION	1.58	1.51	0	0	0	Enhanced
13202	RIZ_ERYTHROID_DIFFERENTIATION_HEMGN	1.58	1.46	0	0	0	Enhanced
13203	GO_POSITIVE_REGULATION_OF_ENDOTHELIAL_CELL_APOPTOTIC_PRO	1.58	1.39	0	0	0	Enhanced
13204	REACTOME_ERYTHROPOIETIN_ACTIVATES_STAT5	1.59	2.12	0	0	0	Enhanced
13205	JAK2_DN.V1_DN	1.59	2.06	0	0	0	Enhanced
13206	TERAMOTO_OPN_TARGETS_CLUSTER_3	1.59	1.95	0	0	0	Enhanced
13207	REACTOME_NEGATIVE_REGULATION_OF_NMDA_RECEPTOR_MEDIATED_	1.59	1.67	0	0	0	Enhanced
13208	MCCABE_HOXC6_TARGETS_CANCER_UP	1.59	1.64	0	0	0	Enhanced
13209	BIOCARTA_EDG1_PATHWAY	1.59	1.62	0	0	0	Enhanced
13210	SABATES_COLORECTAL_ADENOMA_SIZE_UP	1.59	1.61	0	0	0	Enhanced
13211	NIKOLSKY_BREAST_CANCER_19Q13.1_AMPLICON	1.59	1.58	0	0	0	Enhanced
13212	GO_INTRINSIC_COMPONENT_OF_PRESYNAPTIC_ACTIVE_ZONE_MEMBRA	1.59	1.56	0	0	0	Enhanced
13213	GAUSSMANN_MLL_AF4_FUSION_TARGETS_E_DN	1.59	1.52	0	0	0	Enhanced
13214	JISON_SICKLE_CELL_DISEASE_DN	1.60	4.00	0	0	0	Enhanced
13215	ODONNELL_TARGETS_OF_MYC_AND_TFRC_UP	1.60	2.10	0	0	0	Enhanced
13216	KRAS.BREAST_UP.V1_DN	1.60	1.96	0	0	0	Enhanced
13217	GO_POTASSIUM_ION_EXPORT_ACROSS_PLASMA_MEMBRANE	1.60	1.88	0	0	0	Enhanced
13218	PID_NCADHERIN_PATHWAY	1.60	1.70	0	0	0	Enhanced
13219	GO_GLUTAMATE_RECEPTOR_SIGNALING_PATHWAY	1.60	1.69	0	0	0	Enhanced
13220	GO_GASTRULATION_WITH_MOUTH_FORMING_SECOND	1.60	1.64	0	0	0	Enhanced
13221	GO_REGULATORY_T_CELL_DIFFERENTIATION	1.60	1.64	0	0	0	Enhanced
13222	RAMJAUN_APOPTOSIS_BY_TGFB1_VIA_SMAD4_UP	1.60	1.55	0	0	0	Enhanced
13223	BHATI_G2M_ARREST_BY_2METHOXYESTRADIOL_DN	1.61	2.33	0	0	0	Enhanced
13224	GARCIA_TARGETS_OF_FLI1_AND_DAX1_UP	1.61	2.07	0	0	0	Enhanced
13225	MIKKELSEN_IPS_ICP_WITH_H3K4ME3_AND_H327ME3	1.61	1.87	0	0	0	Enhanced
13226	GO_POSITIVE_REGULATION_OF_INTERLEUKIN_10_PRODUCTION	1.61	1.86	0	0	0	Enhanced
13227	REACTOME_FGFR1_MUTANT_RECEPTOR_ACTIVATION	1.61	1.79	0	0	0	Enhanced
13228	GO_INTEGRAL_COMPONENT_OF_PRESYNAPTIC_ACTIVE_ZONE_MEMBRA	1.61	1.78	0	0	0	Enhanced
13229	WEBER_METHYLATED_HCP_IN_SPERM_DN	1.61	1.75	0	0	0	Enhanced
13230	REACTOME_CHONDROITIN_SULFATE_BIOSYNTHESIS	1.61	1.66	0	0	0	Enhanced
13231	GO_DOPAMINE_TRANSPORT	1.61	1.59	0	0	0	Enhanced
13232	MIKKELSEN_MEF_ICP_WITH_H3K27ME3	1.62	2.30	0	0	0	Enhanced
13233	GO_INORGANIC_ANION_TRANSMEMBRANE_TRANSPORTER_ACTIVITY	1.62	2.13	0	0	0	Enhanced
13234	GO_INTRACELLULAR_CALCIUM_ACTIVATED_CHLORIDE_CHANNEL_ACTIV	1.62	1.95	0	0	0	Enhanced
13235	KEGG_ASTHMA	1.62	1.85	0	0	0	Enhanced
13236	GO_REGULATION_OF_NATURAL_KILLER_CELL_ACTIVATION	1.62	1.80	0	0	0	Enhanced
13237	GO_NEGATIVE_REGULATION_OF_LYMPHOCYTE_MEDIATED_IMMUNITY	1.62	1.72	0	0	0	Enhanced
13238	REACTOME_LONG_TERM_POTENTIATION	1.62	1.49	0	0	0	Enhanced
13239	GO_ACTIVATION_OF_PHOSPHOLIPASE_C_ACTIVITY	1.63	1.98	0	0	0	Enhanced
13240	MONTERO_THYROID_CANCER_POOR_SURVIVAL_DN	1.63	1.88	0	0	0	Enhanced
13241	REACTOME_SIGNALING_BY_LEPTIN	1.63	1.74	0	0	0	Enhanced
13242	GO_3_5_CYCLIC_AMP_PHOSPHODIESTERASE_ACTIVITY	1.63	1.72	0	0	0	Enhanced
13243	RIZ_ERYTHROID_DIFFERENTIATION_APOBEC2	1.63	1.71	0	0	0	Enhanced
13244	GO_POSITIVE_REGULATION_OF_MESENCHYMAL_CELL_PROLIFERATION	1.63	1.70	0	0	0	Enhanced
13245	GO_AMPA_GLUTAMATE_RECEPTOR_COMPLEX	1.63	1.47	0	0	0	Enhanced
13246	YAO_TEMPORAL_RESPONSE_TO_PROGESTERONE_CLUSTER_0	1.64	2.31	0	0	0	Enhanced
13247	CHIARETTI_T_ALL_REFRACTORY_TO_THERAPY	1.64	1.99	0	0	0	Enhanced
13248	HALMOS_CEBPA_TARGETS_UP	1.64	1.99	0	0	0	Enhanced
13249	GO_SPHINGOLIPID_BINDING	1.64	1.75	0	0	0	Enhanced
13250	NIELSEN_GIST_VS_SYNOVIAL_SARCOMA_UP	1.64	1.75	0	0	0	Enhanced
13251	GO_IONOTROPIC_GLUTAMATE_RECEPTOR_SIGNALING_PATHWAY	1.64	1.69	0	0	0	Enhanced
13252	GO_COMPLEMENT_ACTIVATION	1.64	1.68	0	0	0	Enhanced
13253	CHR16Q13	1.64	1.65	0	0	0	Enhanced
13254	GO_N_GLYCAN_PROCESSING	1.64	1.60	0	0	0	Enhanced
13255	GO_HYDROLASE_ACTIVITY_HYDROLYZING_O_GLYCOSYL_COMPOUNDS	1.65	2.49	0	0	0	Enhanced
13256	IL15_UP.V1_DN	1.65	2.49	0	0	0	Enhanced
13257	GO_POSITIVE_REGULATION_OF_PHOSPHATIDYLINOSITOL_3_KINASE_SIG	1.65	2.48	0	0	0	Enhanced
13258	GO_REGULATION_OF_INTERFERON_GAMMA_SECRETION	1.65	2.27	0	0	0	Enhanced
13259	WILLIAMS_ESR1_TARGETS_DN	1.65	2.11	0	0	0	Enhanced
13260	GO_CHLORIDE_CHANNEL_ACTIVITY	1.65	1.97	0	0	0	Enhanced
13261	REACTOME_CS_DS_DEGRADATION	1.65	1.96	0	0	0	Enhanced
13262	GO_CHLORIDE_TRANSMEMBRANE_TRANSPORT	1.65	1.82	0	0	0	Enhanced
13263	GO_MONOAMINE_TRANSPORT	1.65	1.81	0	0	0	Enhanced
13264	BIOCARTA_IL22BP_PATHWAY	1.65	1.75	0	0	0	Enhanced
13265	GO_POSTSYNAPTIC_DENSITY_MEMBRANE	1.65	1.73	0	0	0	Enhanced
13266	GO_POSITIVE_REGULATION_OF_PHOSPHOLIPASE_ACTIVITY	1.66	1.90	0	0	0	Enhanced
13267	GO_REGULATION_OF_CELL_FATE_COMMITMENT	1.66	1.65	0	0	0	Enhanced
13268	TONKS_TARGETS_OF_RUNX1_RUNX1T1_FUSION_HSC_UP	1.67	2.81	0	0	0	Enhanced
13269	GO_APICOLATERAL_PLASMA_MEMBRANE	1.67	2.42	0	0	0	Enhanced
13270	GO_RESPONSE_TO_COPPER_ION	1.67	2.17	0	0	0	Enhanced
13271	GAUSSMANN_MLL_AF4_FUSION_TARGETS_G_UP	1.68	4.00	0	0	0	Enhanced
13272	HOELZEL_NF1_TARGETS_DN	1.68	2.48	0	0	0	Enhanced
13273	GO_EMBRYONIC_DIGIT_MORPHOGENESIS	1.68	2.17	0	0	0	Enhanced
13274	GO_REGULATION_OF_GERMINAL_CENTER_FORMATION	1.68	2.13	0	0	0	Enhanced
13275	STEGER_ADIPOGENESIS_DN	1.68	2.12	0	0	0	Enhanced
13276	GO_FAT_PAD_DEVELOPMENT	1.68	1.95	0	0	0	Enhanced
13277	MATTHEWS_AP1_TARGETS	1.68	1.89	0	0	0	Enhanced
13278	WANG_RESPONSE_TO_GSK3_INHIBITOR_SB216763_UP	1.69	4.00	0	0	0	Enhanced
13279	GO_CHONDROITIN_SULFATE_CATABOLIC_PROCESS	1.69	2.24	0	0	0	Enhanced
13280	GO_SPLEEN_DEVELOPMENT	1.69	2.17	0	0	0	Enhanced
13281	GO_RESPONSE_TO_INTERLEUKIN_9	1.69	2.13	0	0	0	Enhanced
13282	GO_STRESS_RESPONSE_TO_METAL_ION	1.69	2.13	0	0	0	Enhanced
13283	GO_GAMMA_DELTA_T_CELL_ACTIVATION	1.69	1.96	0	0	0	Enhanced
13284	GO_CILIUM_MOVEMENT	1.69	1.92	0	0	0	Enhanced
13285	REACTOME_COMPLEMENT_CASCADE	1.69	1.90	0	0	0	Enhanced
13286	REACTOME_INTERLEUKIN_9_SIGNALING	1.70	2.43	0	0	0	Enhanced

13287	GO_DORSAL_SPINAL_CORD_DEVELOPMENT	1.70	2.42	0	0	0	Enhanced
13288	GO_CELLULAR_RESPONSE_TO_CADMIUM_ION	1.70	2.29	0	0	0	Enhanced
13289	GO_POSITIVE_REGULATION_OF_VASCULAR_ENDOTHELIAL_CELL_PROLI	1.70	2.25	0	0	0	Enhanced
13290	GO_CATENIN_COMPLEX	1.70	2.13	0	0	0	Enhanced
13291	GO_VASCULAR_ENDOTHELIAL_CELL_PROLIFERATION	1.70	2.04	0	0	0	Enhanced
13292	WHITFIELD_CELL_CYCLE_S	1.71	4.00	0	0	0	Enhanced
13293	NIKOLSKY_BREAST_CANCER_16P13_AMPLICON	1.71	2.79	0	0	0	Enhanced
13294	GO_STARTLE_RESPONSE	1.71	2.26	0	0	0	Enhanced
13295	GO_NEGATIVE_REGULATION_OF_B_CELL_MEDIATED_IMMUNITY	1.71	2.14	0	0	0	Enhanced
13296	LEIN_OLIGODENDROCYTE_MARKERS	1.71	2.08	0	0	0	Enhanced
13297	REACTOME_SYNAPTIC_ADHESION_LIKE_MOLECULES	1.71	1.97	0	0	0	Enhanced
13298	GO_GROWTH_HORMONE_RECEPTOR_SIGNALING_PATHWAY_VIA_JAK_S	1.72	2.73	0	0	0	Enhanced
13299	GO_POLYSOMAL_RIBOSOME	1.72	2.28	0	0	0	Enhanced
13300	CHR18Q11	1.72	2.16	0	0	0	Enhanced
13301	BILD_E2F3_ONCOGENIC_SIGNATURE	1.73	4.00	0	0	0	Enhanced
13302	OSMAN_BLADDER_CANCER_DN	1.73	4.00	0	0	0	Enhanced
13303	GO_NEGATIVE_REGULATION_OF_CAMP_MEDIATED_SIGNALING	1.73	2.26	0	0	0	Enhanced
13304	TONKS_TARGETS_OF_RUNX1_RUNX1T1_FUSION_SUSTAINED_IN_GRANUI	1.73	2.13	0	0	0	Enhanced
13305	ZHENG_FOXP3_TARGETS_IN_T_LYMPHOCYTE_DN	1.74	4.00	0	0	0	Enhanced
13306	GO_POSITIVE_REGULATION_OF_CYTOSOLIC_CALCIUM_ION_CONCENTRA	1.74	2.74	0	0	0	Enhanced
13307	SHIPP_DLBCL_VS_FOLLICULAR_LYMPHOMA_DN	1.74	2.74	0	0	0	Enhanced
13308	GO_ZINC_ION_HOMEOSTASIS	1.74	2.44	0	0	0	Enhanced
13309	REACTOME_METALLOTHIONEINS_BIND_METALS	1.74	2.25	0	0	0	Enhanced
13310	GO_AXONEME_PART	1.74	1.96	0	0	0	Enhanced
13311	SENGUPTA_NASOPHARYNGEAL_CARCINOMA_DN	1.76	4.00	0	0	0	Enhanced
13312	YAMASHITA_LIVER_CANCER_WITH_EPCAM_UP	1.76	2.76	0	0	0	Enhanced
13313	PID_CD40_PATHWAY	1.76	2.75	0	0	0	Enhanced
13314	WATANABE_COLON_CANCER_MSI_VS_MSS_UP	1.76	2.27	0	0	0	Enhanced
13315	GO_DNA_BINDING_TRANSCRIPTION_FACTOR_ACTIVITY	1.77	4.00	0	0	0	Enhanced
13316	IL2_UP.V1_DN	1.77	4.00	0	0	0	Enhanced
13317	REACTOME_SELENOAMINO_ACID_METABOLISM	1.77	4.00	0	0	0	Enhanced
13318	GO_PRC1_COMPLEX	1.77	2.27	0	0	0	Enhanced
13319	GO_REGULATION_OF_PHOSPHOLIPASE_C_ACTIVITY	1.77	2.00	0	0	0	Enhanced
13320	GO_CYTOSOLIC_LARGE_RIBOSOMAL_SUBUNIT	1.78	4.00	0	0	0	Enhanced
13321	KINSEY_TARGETS_OF_EWSR1_FLII_FUSION_DN	1.78	4.00	0	0	0	Enhanced
13322	GO_SPHINGOLIPID_MEDIATED_SIGNALING_PATHWAY	1.79	4.00	0	0	0	Enhanced
13323	IVANOVA_HEMATOPOIESIS_STEM_CELL_SHORT_TERM	1.79	4.00	0	0	0	Enhanced
13324	REACTOME_RESPONSE_TO_METAL_IONS	1.79	4.00	0	0	0	Enhanced
13325	TENEDINI_MEGAKARYOCYTE_MARKERS	1.79	4.00	0	0	0	Enhanced
13326	GO_B_CELL_MEDIATED_IMMUNITY	1.79	2.78	0	0	0	Enhanced
13327	GO_SPHINGOSINE_1_PHOSPHATE_RECEPTOR_SIGNALING_PATHWAY	1.79	2.73	0	0	0	Enhanced
13328	HOLLEMAN_VINCRISTINE_RESISTANCE_ALL_DN	1.79	2.43	0	0	0	Enhanced
13329	GO_PHOSPHATIDYLSERINE_METABOLIC_PROCESS	1.79	2.42	0	0	0	Enhanced
13330	GO_CHLORIDE_TRANSMEMBRANE_TRANSPORTER_ACTIVITY	1.80	4.00	0	0	0	Enhanced
13331	REACTOME_ACTIVATION_OF_THE_MRNA_UPON_BINDING_OF_THE_CAP_	1.80	4.00	0	0	0	Enhanced
13332	YAN_ESCAPE_FROM_ANOIKIS	1.80	2.73	0	0	0	Enhanced
13333	GO_SYNAPSIS	1.80	2.17	0	0	0	Enhanced
13334	GO_ANIMAL_ORGAN_MATURATION	1.81	4.00	0	0	0	Enhanced
13335	GO_SPHINGOSINE_1_PHOSPHATE_RECEPTOR_ACTIVITY	1.82	4.00	0	0	0	Enhanced
13336	GO_SPECIFICATION_OF_SYMMETRY	1.82	2.79	0	0	0	Enhanced
13337	REACTOME_LYSOSPHINGOLIPID_AND_LPA_RECEPTORS	1.83	4.00	0	0	0	Enhanced
13338	GO_GERMINAL_CENTER_FORMATION	1.83	2.71	0	0	0	Enhanced
13339	DUTERTRE_ESTRADIOL_RESPONSE_6HR_DN	1.84	4.00	0	0	0	Enhanced
13340	E2F3_UP.V1_UP	1.85	4.00	0	0	0	Enhanced
13341	KEGG_INTESTINAL_IMMUNE_NETWORK_FOR_IGA_PRODUCTION	1.85	2.75	0	0	0	Enhanced
13342	REACTOME_NEUREXINS_AND_NEUROLIGINS	1.86	4.00	0	0	0	Enhanced
13343	GO_CELLULAR_RESPONSE_TO_COPPER_ION	1.86	2.73	0	0	0	Enhanced
13344	GO_PROTEIN_TARGETING_TO_MEMBRANE	1.87	4.00	0	0	0	Enhanced
13345	REACTOME_NONSENSE_MEDIATED_DECAY_NMD	1.87	4.00	0	0	0	Enhanced
13346	CSR_EARLY_UP.V1_DN	1.88	4.00	0	0	0	Enhanced
13347	MARZEC_IL2_SIGNALING_DN	1.88	2.73	0	0	0	Enhanced
13348	GO_NUCLEAR_TRANSCRIBED_MRNA_CATABOLIC_PROCESS_NONSENSE	1.89	4.00	0	0	0	Enhanced
13349	MEISSNER_BRAIN_HCP_WITH_H3K27ME3	1.89	4.00	0	0	0	Enhanced
13350	NAKAJIMA_MAST_CELL	1.89	2.75	0	0	0	Enhanced
13351	SARTIPY_NORMAL_AT_INSULIN_RESISTANCE_DN	1.89	2.44	0	0	0	Enhanced
13352	GO_CELLULAR_RESPONSE_TO_ZINC_ION	1.92	4.00	0	0	0	Enhanced
13353	HOLLEMAN_ASPARAGINASE_RESISTANCE_B_ALL_UP	1.92	4.00	0	0	0	Enhanced
13354	REACTOME_EUKARYOTIC_TRANSLATION_INITIATION	1.92	4.00	0	0	0	Enhanced
13355	BILANGES_SERUM_RESPONSE_TRANSLATION	1.95	4.00	0	0	0	Enhanced
13356	GO_AXONEME_ASSEMBLY	1.95	4.00	0	0	0	Enhanced
13357	CHNG_MULTIPLE_MYELOMA_HYPERPLOID_UP	1.96	4.00	0	0	0	Enhanced
13358	GO_PROTEIN_LOCALIZATION_TO_ENDOPLASMIC_RETICULUM	1.97	4.00	0	0	0	Enhanced
13359	GO_HUMORAL_IMMUNE_RESPONSE_MEDIATED_BY_CIRCULATING_IMMUN	1.99	4.00	0	0	0	Enhanced
13360	GO_NEGATIVE_REGULATION_OF_TUMOR_NECROSIS_FACTOR_SUPERFA	1.99	4.00	0	0	0	Enhanced
13361	GO_NEUROTRANSMITTER_RECEPTOR_COMPLEX	1.99	4.00	0	0	0	Enhanced
13362	GO_CYTOSOLIC_SMALL_RIBOSOMAL_SUBUNIT	2.04	4.00	0	0	0	Enhanced
13363	GO_ESTABLISHMENT_OF_PROTEIN_LOCALIZATION_TO_ENDOPLASMIC_F	2.04	4.00	0	0	0	Enhanced
13364	GO_BIOACTIVE_LIPID_RECEPTOR_ACTIVITY	2.05	4.00	0	0	0	Enhanced
13365	GO_CYTOSOLIC_RIBOSOME	2.06	4.00	0	0	0	Enhanced
13366	NAKAYAMA_FRA2_TARGETS	2.06	4.00	0	0	0	Enhanced
13367	CHR19P12	2.14	4.00	0	0	0	Enhanced
13368	REACTOME_SRP_DEPENDENT_COTRANSLATIONAL_PROTEIN_TARGETIN	2.14	4.00	0	0	0	Enhanced
13369	REACTOME_NONSENSE_MEDIATED_DECAY_NMD_INDEPENDENT_OF_THI	2.15	4.00	0	0	0	Enhanced
13370	KEGG_RIBOSOME	2.27	4.00	0	0	0	Enhanced
13371	MTOR_UP.N4.V1_DN	2.30	4.00	0	0	0	Enhanced
13372	BILANGES_SERUM_AND_RAPAMYCIN_SENSITIVE_GENES	2.31	4.00	0	0	0	Enhanced
13373	GO_COTRANSLATIONAL_PROTEIN_TARGETING_TO_MEMBRANE	2.34	4.00	0	0	0	Enhanced

Supplementary Table 7B. Gene set enrichment for the transcriptional changes induced by RZ-2994 at Day 1 on KOPTK1 cells. MSigDB v7.0 collection, significance -log10(P-value+0.0001)>1. Effect of RZ-2994 on pathway based on Normalized Enrichment Score (NES): NES < -1.5 Repressed, NES > 1.5 Enhanced.

#	Gene set	Normalized Enrichment Score (NES) at Day 1	-log10 (P-value+0.0001) at Day 1	Amino Acid gene set signatures (1=yes, 0=no)	MYC gene set signatures (1=yes, 0=no)	Cell Cycle gene set signatures (1=yes, 0=no)	RZ-2994 vs. DMSO transcriptional effect on pathway at Day 1
1	GO_PRERIBOSOME	-2.59	4	0	0	0	Repressed
2	FISCHER_G2_M_CELL_CYCLE	-2.57	4	0	0	1	Repressed
3	GARY_CD5_TARGETS_DN	-2.50	4	0	0	0	Repressed
4	REACTOME_RRNA_MODIFICATION_IN_THE_NUCLEUS_AND_CYTOSOL	-2.47	4	0	0	0	Repressed
5	NAKAYAMA_SOFT_TISSUE_TUMORS_PCA2_UP	-2.46	4	0	0	0	Repressed
6	GO_RIBOSOME_BIOGENESIS	-2.45	4	0	0	0	Repressed
7	WHITFIELD_CELL_CYCLE_G2_M	-2.45	4	0	0	0	Repressed
8	PENG_GLUTAMINE_DEPRIVATION_DN	-2.44	4	1	0	0	Repressed
9	TANG_SENESCENCE_TP53_TARGETS_DN	-2.43	4	0	0	0	Repressed
10	YAO_TEMPORAL_RESPONSE_TO_PROGESTERONE_CLUSTER_11	-2.43	4	0	0	0	Repressed
11	PENG_LEUCINE_DEPRIVATION_DN	-2.41	4	1	0	0	Repressed
12	AMUNDSON_GAMMA_RADIATION_RESPONSE	-2.40	4	0	0	0	Repressed
13	REACTOME_APC_C:CDH1_MEDIATED_DEGRADATION_OF_CDC20_AND_OTHER_AP(	-2.40	4	0	0	0	Repressed
14	REACTOME_REGULATION_OF_MITOTIC_CELL_CYCLE	-2.40	4	0	0	0	Repressed
15	ELVIDGE_HYPOXIA_DN	-2.38	4	0	0	0	Repressed
16	GO_RRNA_METABOLIC_PROCESS	-2.38	4	0	0	0	Repressed
17	HALLMARK_MYC_TARGETS_V2	-2.38	4	0	1	0	Repressed
18	GO_ANAPHASE_PROMOTING_COMPLEX_DEPENDENT_CATABOLIC_PROCESS	-2.37	4	0	0	0	Repressed
19	HALLMARK_MYC_TARGETS_V1	-2.37	4	0	1	0	Repressed
20	REACTOME ACTIVATION OF APC C AND APC C:CDC20 MEDIATED DEGRADATIC	-2.37	4	0	0	0	Repressed
21	SCHLOSSER_MYC_TARGETS_AND_SERUM_RESPONSE_DN	-2.35	4	0	0	0	Repressed
22	GO_NCRNA_METABOLIC_PROCESS	-2.34	4	0	0	0	Repressed
23	KARLSSON_TGFB1_TARGETS_UP	-2.34	4	0	0	0	Repressed
24	REACTOME_MITOTIC_METAPHASE_AND_ANAPHASE	-2.33	4	0	0	0	Repressed
25	GO_NCRNA_PROCESSING	-2.31	4	0	0	0	Repressed
26	GO_SMALL_SUBUNIT_PROCESSOME	-2.31	4	0	0	0	Repressed
27	ODONNELL_TFRC_TARGETS_DN	-2.31	4	0	0	0	Repressed
28	MENSSEN_MYC_TARGETS	-2.30	4	0	1	0	Repressed
29	MALONEY_RESPONSE_TO_17AAG_DN	-2.29	4	0	0	0	Repressed
30	REACTOME THE ROLE OF GTSE1 IN G2 M PROGRESSION AFTER G2 CHECKP	-2.29	4	0	0	0	Repressed
31	SOTIRIOU_BREAST_CANCER_GRADE_1_VS_3_UP	-2.29	4	0	0	0	Repressed
32	GO_RIBONUCLEOPROTEIN_COMPLEX_BIOGENESIS	-2.27	4	0	0	0	Repressed
33	GO_RNA_LOCALIZATION	-2.26	4	0	0	0	Repressed
34	REICHERT_MITOSIS_LIN9_TARGETS	-2.26	4	0	0	0	Repressed
35	RHODES_UNDIFFERENTIATED_CANCER	-2.26	4	0	0	0	Repressed
36	KRIGE_RESPONSE_TO_TOSEDOSTAT_24HR_DN	-2.24	4	0	0	0	Repressed
37	CSR_EARLY_UP.V1_UP	-2.23	4	0	0	0	Repressed
38	REACTOME_RESOLUTION_OF_SISTER_CHROMATID_COHESION	-2.23	4	0	0	0	Repressed
39	FLORIO_NEOCORTEX_BASAL_RADIAL_GLIA_DN	-2.22	4	0	0	0	Repressed
40	REACTOME_MITOTIC_SPINDLE_CHECKPOINT	-2.22	4	0	0	0	Repressed
41	KRIGE_RESPONSE_TO_TOSEDOSTAT_6HR_DN	-2.21	4	0	0	0	Repressed
42	PENG_RAPAMYCIN_RESPONSE_DN	-2.21	4	0	0	0	Repressed
43	GO_MATURATION_OF_SSU_RRNA	-2.19	4	0	0	0	Repressed
44	LEE_BMP2_TARGETS_DN	-2.19	4	0	0	0	Repressed
45	PID_AURORA_B_PATHWAY	-2.19	4	0	0	0	Repressed
46	REACTOME_METABOLISM_OF_RNA	-2.19	4	0	0	0	Repressed
47	GO_PROTEASOME_ACCESSORY_COMPLEX	-2.18	4	0	0	0	Repressed
48	ROSTY_CERVICAL_CANCER_PROLIFERATION_CLUSTER	-2.18	4	0	0	0	Repressed
49	GO_90S_PRERIBOSOME	-2.17	4	0	0	0	Repressed
50	MANALO_HYPOXIA_DN	-2.17	4	0	0	0	Repressed
51	REACTOME FBXL7 DOWN REGULATES AURKA DURING MITOTIC ENTRY AND IN	-2.17	4	0	0	0	Repressed
52	FARMER_BREAST_CANCER_CLUSTER_2	-2.16	4	0	0	0	Repressed
53	GO_PRERIBOSOME_LARGE_SUBUNIT_PRECURSOR	-2.16	4	0	0	0	Repressed
54	GO_MITOTIC_SPINDLE_ORGANIZATION	-2.15	4	0	0	0	Repressed
55	GO_RIBOSOMAL_SMALL_SUBUNIT_BIOGENESIS	-2.15	4	0	0	0	Repressed
56	GO_SPINDLE_MIDZONE	-2.15	4	0	0	0	Repressed
57	REACTOME_DEGRADATION_OF_GLI1_BY_THE_PROTEASOME	-2.14	4	0	0	0	Repressed
58	SCHLOSSER_MYC_TARGETS_REPRESSED_BY_SERUM	-2.14	4	0	0	0	Repressed
59	WEI_MYCN_TARGETS_WITH_E_BOX	-2.14	4	0	0	0	Repressed
60	BOYAULT_LIVER_CANCER_SUBCLASS_G3_UP	-2.13	4	0	0	0	Repressed
61	CAIRO_HEPATOBLASTOMA_CLASSES_UP	-2.13	4	0	0	0	Repressed
62	ELVIDGE_HIF1A_TARGETS_UP	-2.13	4	0	0	0	Repressed
63	GO MATURATION OF SSU RRNA FROM TRICISTRONIC RRNA TRANSCRIPT SSU	-2.13	4	0	0	0	Repressed
64	ODONNELL_TARGETS_OF_MYC_AND_TFRC_DN	-2.13	4	0	0	0	Repressed
65	WHITFIELD_CELL_CYCLE_M_G1	-2.13	4	0	0	0	Repressed
66	GO_RIBOSOMAL_LARGE_SUBUNIT_BIOGENESIS	-2.12	4	0	0	0	Repressed
67	BIDUS_METASTASIS_UP	-2.11	4	0	0	0	Repressed
68	REACTOME_DEGRADATION_OF_BETA_CATENIN_BY_THE_DESTRUCTION_COMPLEX	-2.11	4	0	0	0	Repressed
69	BURTON_ADIPOGENESIS_PEAK_AT_24HR	-2.10	4	0	0	0	Repressed
70	GO_SPINDLE_MICROTUBULE	-2.10	4	0	0	0	Repressed
71	PUJANA_CHEK2_PCC_NETWORK	-2.10	4	0	0	0	Repressed
72	REACTOME_REGULATION_OF_RUNX2_EXPRESSION_AND_ACTIVITY	-2.10	4	0	0	0	Repressed
73	REACTOME_RRNA_PROCESSING	-2.10	4	0	0	0	Repressed
74	GO_ESTABLISHMENT_OF_RNA_LOCALIZATION	-2.09	4	0	0	0	Repressed
75	GO_MICROTUBULE_CYTOSKELETON_ORGANIZATION_INVOLVED_IN_MITOSIS	-2.09	4	0	0	0	Repressed
76	REACTOME_VIF_MEDIATED_DEGRADATION_OF_APOBEC3G	-2.09	4	0	0	0	Repressed
77	CHANG_CYCLING_GENES	-2.08	4	0	0	0	Repressed
78	MOHANKUMAR_HOXA1_TARGETS_UP	-2.08	4	0	0	0	Repressed
79	REACTOME_CELLULAR_RESPONSE_TO_HYPOXIA	-2.08	4	0	0	0	Repressed
80	REACTOME_RRNA_PROCESSING_IN_THE_NUCLEUS_AND_CYTOSOL	-2.08	4	0	0	0	Repressed
81	WONG_EMBRYONIC_STEM_CELL_CORE	-2.08	4	0	0	0	Repressed
82	GO_RNA_BINDING	-2.07	4	0	0	0	Repressed
83	RHODES_CANCER_META_SIGNATURE	-2.07	4	0	0	0	Repressed
84	GO_ENDOPEPTIDASE_COMPLEX	-2.06	4	0	0	0	Repressed
85	GO_RNA_METABOLIC_PROCESS	-2.06	4	0	0	0	Repressed
86	SCIAN_CELL_CYCLE_TARGETS_OF_TP53_AND_TP73_DN	-2.06	4	0	0	0	Repressed
87	SHEDDEN_LUNG_CANCER_POOR_SURVIVAL_A6	-2.06	4	0	0	0	Repressed
88	VEGF_A_UP.V1_DN	-2.06	4	0	0	0	Repressed
89	WONG_PROTEASOME_GENE_MODULE	-2.06	4	0	0	0	Repressed
90	CROONQUIST_IL6_DEPRIVATION_DN	-2.05	4	0	0	0	Repressed
91	CROONQUIST_NRAS_SIGNALING_DN	-2.05	4	0	0	0	Repressed
92	KANG_DOXORUBICIN_RESISTANCE_UP	-2.05	4	0	0	0	Repressed
93	KEGG_OOCYTE_MEIOSIS	-2.05	4	0	0	0	Repressed
94	REACTOME_CROSS_PRESENTATION_OF_SOLUBLE_EXOGENOUS_ANTIGENS_ENDC	-2.05	4	0	0	0	Repressed
95	REACTOME_NEGATIVE_REGULATION_OF_NOTCH4_SIGNALING	-2.05	4	0	0	0	Repressed
96	ZHOU_CELL_CYCLE_GENES_IN_IR_RESPONSE_24HR	-2.05	4	0	0	0	Repressed
97	GO_MATURATION_OF_LSU_RRNA	-2.04	4	0	0	0	Repressed

98	HALLMARK_G2M_CHECKPOINT	-2.04	4	0	0	0	Repressed
99	HOFFMANN_LARGE_TO_SMALL_PRE_BII_LYMPHOCYTE_UP	-2.04	4	0	0	0	Repressed
100	LY_AGING_MIDDLE_DN	-2.04	4	0	0	0	Repressed
101	LY_AGING_OLD_DN	-2.04	4	0	0	0	Repressed
102	REACTOME_PROCESSING_OF_CAPPED_INTRON_CONTAINING_PRE_MRNA	-2.04	4	0	0	0	Repressed
103	REACTOME_REGULATION_OF_APOPTOSIS	-2.04	4	0	0	0	Repressed
104	YU_MYC_TARGETS_UP	-2.04	4	0	0	0	Repressed
105	BASAKI_YBX1_TARGETS_UP	-2.03	4	0	0	0	Repressed
106	GO_MOLECULAR_CARRIER_ACTIVITY	-2.03	4	0	0	0	Repressed
107	GO_SMALL_NUCLEOLAR_RIBONUCLEOPROTEIN_COMPLEX	-2.03	4	0	0	0	Repressed
108	MOHANKUMAR_TLX1_TARGETS_UP	-2.03	4	0	0	0	Repressed
109	PID_MYC_ACTIV_PATHWAY	-2.03	4	0	1	0	Repressed
110	GO_CATALYTIC_ACTIVITY_ACTING_ON_A_TRNA	-2.02	4	0	0	0	Repressed
111	GO_NUCLEAR_EXPORT	-2.02	4	0	0	0	Repressed
112	GO_NUCLEAR_PORE	-2.02	4	0	0	0	Repressed
113	MONNIER_POSTRADIATION_TUMOR_ESCAPE_UP	-2.02	4	0	0	0	Repressed
114	NAKAMURA_TUMOR_ZONE_PERIPHERAL_VS_CENTRAL_UP	-2.02	4	0	0	0	Repressed
115	VECCHI_GASTRIC_CANCER_EARLY_UP	-2.02	4	0	0	0	Repressed
116	GO_NLS_BEARING_PROTEIN_IMPORT_INTO_NUCLEUS	-2.01	4	0	0	0	Repressed
117	GO_PROTEASOME_REGULATORY_PARTICLE_BASE_SUBCOMPLEX	-2.01	4	0	0	0	Repressed
118	GO_TRNA_METABOLIC_PROCESS	-2.01	4	1	0	0	Repressed
119	GRAHAM_CML_DIVIDING_VS_NORMAL_QUIESCENT_UP	-2.01	4	0	0	0	Repressed
120	MORI_LARGE_PRE_BII_LYMPHOCYTE_UP	-2.01	4	0	0	0	Repressed
121	PID_AURORA_A_PATHWAY	-2.01	4	0	0	0	Repressed
122	REACTOME_HSP90_CHAPERONE_CYCLE_FOR_STEROID_HORMONE_RECEPTORS	-2.01	4	0	0	0	Repressed
123	GO_POSITIVE_REGULATION_OF_TELOMERASE_RNA_LOCALIZATION_TO_CAJAL_BC	-2.00	4	0	0	0	Repressed
124	GO_RNA_EXPORT_FROM_NUCLEUS	-2.00	4	0	0	0	Repressed
125	REACTOME_AUF1_HNRNP_D0_BINDS_AND_DESTABILIZES_MRNA	-2.00	4	0	0	0	Repressed
126	SU_TESTIS	-2.00	4	0	0	0	Repressed
127	ZHAN_MULTIPLE_MYELOMA_PR_UP	-2.00	4	0	0	0	Repressed
128	GO_NUCLEAR_TRANSPORT	-1.99	4	0	0	0	Repressed
129	GO_NUCLEOCYTOPLASMIC_CARRIER_ACTIVITY	-1.99	4	0	0	0	Repressed
130	GO_PEPTIDASE_COMPLEX	-1.99	4	0	0	0	Repressed
131	KEGG_PROTEASOME	-1.98	4	0	0	0	Repressed
132	KIM_MYC_AMPLIFICATION_TARGETS_UP	-1.98	4	0	0	0	Repressed
133	REACTOME_DOWNSTREAM_SIGNALING_EVENTS_OF_B_CELL_RECEPTOR_BCR	-1.98	4	0	0	0	Repressed
134	REACTOME_GLYCOLYSIS	-1.98	4	0	0	0	Repressed
135	REACTOME_RHO_GTPASES_ACTIVATE_FORMINS	-1.98	4	0	0	0	Repressed
136	REACTOME_SIGNALING_BY_THE_B_CELL_RECEPTOR_BCR	-1.98	4	0	0	0	Repressed
137	REACTOME_TRNA_AMINOACYLATION	-1.98	4	0	0	0	Repressed
138	RODRIGUES_THYROID_CARCINOMA_ANAPLASTIC_UP	-1.98	4	0	0	0	Repressed
139	SCHLOSSER_MYC_TARGETS_AND_SERUM_RESPONSE_UP	-1.98	4	0	0	0	Repressed
140	GO_AMINO_ACID_ACTIVATION	-1.97	4	0	0	0	Repressed
141	GO_NUCLEOLAR_PART	-1.97	4	0	0	0	Repressed
142	GO_TELOMERASE_RNA_LOCALIZATION	-1.97	4	0	0	0	Repressed
143	HORIUCHI_WTAP_TARGETS_DN	-1.97	4	0	0	0	Repressed
144	REACTOME_ACTIVATION_OF_NF_KAPPAB_IN_B_CELLS	-1.97	4	0	0	0	Repressed
145	REACTOME_M_PHASE	-1.97	4	0	0	0	Repressed
146	REACTOME_REGULATION_OF_RAS_BY_GAPS	-1.97	4	0	0	0	Repressed
147	SCHUHMACHER_MYC_TARGETS_UP	-1.97	4	0	0	0	Repressed
148	SHEPARD_CRUSH_AND_BURN_MUTANT_DN	-1.97	4	0	0	0	Repressed
149	ANDERSEN_CHOLANGIOCARCINOMA_CLASS2	-1.96	4	0	0	0	Repressed
150	BURTON_ADIPOGENESIS_4	-1.96	4	0	0	0	Repressed
151	FOURNIER_ACINAR_DEVELOPMENT_LATE_2	-1.96	4	0	0	0	Repressed
152	REACTOME_APC_CDC20_MEDIATED_DEGRADATION_OF_NEK2A	-1.96	4	0	0	0	Repressed
153	REACTOME_DEGRADATION_OF_AXIN	-1.96	4	0	0	0	Repressed
154	REACTOME_HEDGEHOG_ON_STATE	-1.96	4	0	0	0	Repressed
155	REACTOME_MRNA_SPLICING	-1.96	4	0	0	0	Repressed
156	DODD_NASOPHARYNGEAL_CARCINOMA_DN	-1.95	4	0	0	0	Repressed
157	ELVIDGE_HIF1A_AND_HIF2A_TARGETS_UP	-1.95	4	0	0	0	Repressed
158	GO_LIGASE_ACTIVITY_FORMING_CARBON_OXYGEN_BONDS	-1.95	4	0	0	0	Repressed
159	GO_MATURATION_OF_LSU_RRNA_FROM_TRICISTRONIC_RRNA_TRANSCRIPT_SSU	-1.95	4	0	0	0	Repressed
160	KONG_E2F3_TARGETS	-1.95	4	0	0	0	Repressed
161	REACTOME_DEGRADATION_OF_DVL	-1.95	4	0	0	0	Repressed
162	REACTOME_HIV_INFECTION	-1.95	4	0	0	0	Repressed
163	REACTOME_HOST_INTERACTIONS_OF_HIV_FACTORS	-1.95	4	0	0	0	Repressed
164	REACTOME_REGULATION_OF_PTEN_STABILITY_AND_ACTIVITY	-1.95	4	0	0	0	Repressed
165	BIOCARTA_RANMS_PATHWAY	-1.95	2.68	0	0	0	Repressed
166	GO_REGULATION_OF_PROTEIN_LOCALIZATION_TO_CHROMOSOME_TELOMERIC_R	-1.95	2.34	0	0	0	Repressed
167	GO_CATALYTIC_ACTIVITY_ACTING_ON_RNA	-1.94	4	0	0	0	Repressed
168	GO_SPINDLE_ORGANIZATION	-1.94	4	0	0	0	Repressed
169	WILLIAMS_ESR1_TARGETS_UP	-1.94	4	0	0	0	Repressed
170	WINNEPENNINCKX_MELANOMA_METASTASIS_UP	-1.94	4	0	0	0	Repressed
171	GAVIN_FOXP3_TARGETS_CLUSTER_P6	-1.93	4	0	0	0	Repressed
172	GO_OTHER_ORGANISM	-1.93	4	0	0	0	Repressed
173	GO_PROTEIN_IMPORT_INTO_MITOCHONDRIAL_MATRIX	-1.93	4	0	0	0	Repressed
174	GO_SPINDLE	-1.93	4	0	0	0	Repressed
175	GO_SPINDLE_POLE	-1.93	4	0	0	0	Repressed
176	REACTOME_HEDGEHOG_OFF_STATE	-1.93	4	0	0	0	Repressed
177	REACTOME_METABOLISM_OF_NON_CODING_RNA	-1.93	4	0	0	0	Repressed
178	REACTOME_REGULATION_OF_RUNX3_EXPRESSION_AND_ACTIVITY	-1.93	4	0	0	0	Repressed
179	REACTOME_PHOSPHORYLATION_OF_THE_APC_C	-1.93	2.62	0	0	0	Repressed
180	CHIANG_LIVER_CANCER_SUBCLASS_PROLIFERATION_UP	-1.92	4	0	0	0	Repressed
181	GO_MRNA_TRANSPORT	-1.92	4	0	0	0	Repressed
182	GO_RAN_GTPASE_BINDING	-1.92	4	0	0	0	Repressed
183	GO_REGULATION_OF_CHROMOSOME_SEGREGATION	-1.92	4	0	0	0	Repressed
184	GO_RNA_SPLICING_VIA_TRANSESTERIFICATION_REACTIONS	-1.92	4	0	0	0	Repressed
185	PUIFFE_INVASION_INHIBITED_BY_ASCITES_UP	-1.92	4	0	0	0	Repressed
186	REACTOME_NUCLEAR_PORE_COMPLEX_NPC_DISASSEMBLY	-1.92	4	0	0	0	Repressed
187	REACTOME_UCH_PROTEINASES	-1.92	4	0	0	0	Repressed
188	REACTOME_VIRAL_MESSENGER_RNA_SYNTHESIS	-1.92	4	0	0	0	Repressed
189	GO_PRERIBOSOME_SMALL_SUBUNIT_PRECURSOR	-1.92	2.64	0	0	0	Repressed
190	REACTOME_APC_C:CDC20_MEDIATED_DEGRADATION_OF_CYCLIN_B	-1.92	2.59	0	0	0	Repressed
191	REACTOME_INTERACTIONS_OF_REV_WITH_HOST_CELLULAR_PROTEINS	-1.92	2.56	0	0	0	Repressed
192	BERENJENO_TRANSFORMED_BY_RHOA_UP	-1.91	4	0	0	0	Repressed
193	FERREIRA_EWINGS_SARCOMA_UNSTABLE_VS_STABLE_UP	-1.91	4	0	0	0	Repressed
194	FURUKAWA_DUSP6_TARGETS_PCI35_DN	-1.91	4	0	0	0	Repressed
195	GO_DOUBLE_STRANDED_RNA_BINDING	-1.91	4	0	0	0	Repressed
196	GO_REGULATION_OF_CHROMOSOME_SEPARATION	-1.91	4	0	0	0	Repressed
197	GO_REGULATION_OF_TRANSLATIONAL_ELONGATION	-1.91	4	0	0	0	Repressed
198	REACTOME_MAPK6_MAPK4_SIGNALING	-1.91	4	0	0	0	Repressed
199	REACTOME_STABILIZATION_OF_P53	-1.91	4	0	0	0	Repressed
200	REACTOME_SUMOYLATION_OF_DNA_REPLICATION_PROTEINS	-1.91	4	0	0	0	Repressed
201	GO_RIBONUCLEOPROTEIN_COMPLEX_BINDING	-1.90	4	0	0	0	Repressed
202	GO_SCF_DEPENDENT_PROTEASOMAL_UBIQUITIN_DEPENDENT_PROTEIN_CATABOI	-1.90	4	0	0	0	Repressed
203	LEE_EARLY_T_LYMPHOCYTE_UP	-1.90	4	0	0	0	Repressed
204	TARTE_PLASMA_CELL_VS_PLASMABLAST_DN	-1.90	4	0	0	0	Repressed
205	TIEN_INTESTINE_PROBIOTICS_24HR_UP	-1.90	4	0	0	0	Repressed
206	REACTOME_MITOTIC_TELOPHASE_CYTOKINESIS	-1.90	2.64	0	0	0	Repressed

207	BHAT_ESR1_TARGETS_NOT_VIA_AKT1_UP	-1.89	4	0	0	0	Repressed
208	CHAUHAN_RESPONSE_TO_METHOXYESTRADIOL_UP	-1.89	4	0	0	0	Repressed
209	GO_CHROMOSOME_CENTROMERIC_REGION	-1.89	4	0	0	0	Repressed
210	KINSEY_TARGETS_OF_EWSR1_FLII_FUSION_UP	-1.89	4	0	0	0	Repressed
211	REACTOME_RNA_POLYMERASE_II_TRANSCRIPTION_PRE_INITIATION_AND_PROMOT	-1.89	4	0	0	0	Repressed
212	REACTOME_TRNA_PROCESSING	-1.89	4	0	0	0	Repressed
213	GO_TRNA_TRANSPORT	-1.89	2.56	0	0	0	Repressed
214	GO_HEMATOPOIETIC_STEM_CELL_DIFFERENTIATION	-1.88	4	0	0	0	Repressed
215	GO_MITOTIC_SPINDLE_ASSEMBLY	-1.88	4	0	0	0	Repressed
216	GO_NCRNA_TRANSCRIPTION	-1.88	4	0	0	0	Repressed
217	MARTINEZ_RESPONSE_TO_TRABECTEDIN_DN	-1.88	4	0	0	0	Repressed
218	REACTOME_PCP_CE_PATHWAY	-1.88	4	0	0	0	Repressed
219	GO_PROTEIN_K11_LINKED_UBIQUITINATION	-1.88	2.59	0	0	0	Repressed
220	GO_TRNA_MODIFICATION	-1.88	2.51	0	0	0	Repressed
221	GENTILE_UV_RESPONSE_CLUSTER_D4	-1.87	4	0	0	0	Repressed
222	GENTILE_UV_RESPONSE_CLUSTER_D5	-1.87	4	0	0	0	Repressed
223	GO_CONDENSED_CHROMOSOME_OUTER_KINETOCHORE	-1.87	4	0	0	0	Repressed
224	GO_NUCLEOBASE_CONTAINING_COMPOUND_TRANSPORT	-1.87	4	0	0	0	Repressed
225	GO_REGULATION_OF_SISTER_CHROMATID_SEGREGATION	-1.87	4	0	0	0	Repressed
226	GREENBAUM_E2A_TARGETS_UP	-1.87	4	0	0	0	Repressed
227	REACTOME_CDK_MEDIATED_PHOSPHORYLATION_AND_REMOVAL_OF_CDC6	-1.87	4	0	0	0	Repressed
228	REACTOME_FCERI_MEDIATED_NF_KB_ACTIVATION	-1.87	4	0	0	0	Repressed
229	REACTOME_RNA_POLYMERASE_II_PRE_TRANSCRIPTION_EVENTS	-1.87	4	0	0	0	Repressed
230	REACTOME_CYTOSOLIC_TRNA_AMINOACYLATION	-1.87	2.6	0	0	0	Repressed
231	GO_MITOCHONDRIAL_RNA_METABOLIC_PROCESS	-1.86	4	0	0	0	Repressed
232	GO_MITOTIC_NUCLEAR_ENVELOPE_DISASSEMBLY	-1.86	4	0	0	0	Repressed
233	GO_MITOTIC_SISTER_CHROMATID_SEGREGATION	-1.86	4	0	0	0	Repressed
234	GO_MITOTIC_SPINDLE	-1.86	4	0	0	0	Repressed
235	GRADE_METASTASIS_DN	-1.86	4	0	0	0	Repressed
236	KOBAYASHI_EGFR_SIGNALING_24HR_DN	-1.86	4	0	0	0	Repressed
237	REACTOME_RNA_POLYMERASE_II_TRANSCRIBES_SNRNA_GENES	-1.86	4	0	0	0	Repressed
238	SHEPARD_BMYB_TARGETS	-1.86	4	0	0	0	Repressed
239	CROMER_METASTASIS_DN	-1.86	2.54	0	0	0	Repressed
240	DASU_IL6_SIGNALING_SCAR_UP	-1.85	4	0	0	0	Repressed
241	GO_PROTEIN_IMPORT	-1.85	4	0	0	0	Repressed
242	GO_SISTER_CHROMATID_SEGREGATION	-1.85	4	0	0	0	Repressed
243	KEGG_SPLICEOSOME	-1.85	4	0	0	0	Repressed
244	MUELLER_PLURINET	-1.85	4	0	0	0	Repressed
245	REACTOME_DOWNSTREAM_TCR_SIGNALING	-1.85	4	0	0	0	Repressed
246	REACTOME_NUCLEAR_IMPORT_OF_REV_PROTEIN	-1.85	4	0	0	0	Repressed
247	REACTOME_TRANSCRIPTIONAL_REGULATION_BY_RUNX2	-1.85	4	0	0	0	Repressed
248	RICKMAN_TUMOR_DIFFERENTIATED_WELL_VS_MODERATELY_DN	-1.85	4	0	0	0	Repressed
249	GO_CHAPERONE_COMPLEX	-1.85	2.63	0	0	0	Repressed
250	BURTON_ADIPOGENESIS_3	-1.85	2.5	0	0	0	Repressed
251	DAIRKEE_CANCER_PRONE_RESPONSE_BPA	-1.84	4	0	0	0	Repressed
252	GO_REGULATION_OF_CELLULAR_RESPONSE_TO_HEAT	-1.84	4	0	0	0	Repressed
253	GO_SPLICEOSOMAL_SNRNP_ASSEMBLY	-1.84	4	0	0	0	Repressed
254	GO_TRANSLATIONAL_TERMINATION	-1.84	4	0	0	0	Repressed
255	PID_PLK1_PATHWAY	-1.84	4	0	0	0	Repressed
256	REACTOME_ASYMMETRIC_LOCALIZATION_OF_PCP_PROTEINS	-1.84	4	0	0	0	Repressed
257	REACTOME_CONVERSION_FROM_APC_C:CDC20_TO_APC_C:CDH1_IN_LATE_ANAPI	-1.84	4	0	0	0	Repressed
258	REACTOME_MRNA_SPLICING_MINOR_PATHWAY	-1.84	4	0	0	0	Repressed
259	LEE_INTRATHYMIC_T_PROGENITOR	-1.84	2.65	0	0	0	Repressed
260	NIKOLSKY_BREAST_CANCER_14Q22_AMPLICON	-1.84	2.65	0	0	0	Repressed
261	BOSCO_EPITHELIAL_DIFFERENTIATION_MODULE	-1.84	2.64	0	0	0	Repressed
262	GO_REGULATION_OF_RIBOSOME_BIOGENESIS	-1.84	2.62	0	0	0	Repressed
263	GO_TRANSLATION_INITIATION_FACTOR_ACTIVITY	-1.84	2.56	0	0	0	Repressed
264	PID_ILK_PATHWAY	-1.84	2.56	0	0	0	Repressed
265	ELVIDGE_HYPOXIA_BY_DMOG_DN	-1.83	4	0	0	0	Repressed
266	GO_METAPHASE_ANAPHASE_TRANSITION_OF_CELL_CYCLE	-1.83	4	0	0	1	Repressed
267	JUBAN_TARGETS_OF_SPI1_AND_FLI1_DN	-1.83	4	0	0	0	Repressed
268	KEGG_AMINOACYL_TRNA_BIOSYNTHESIS	-1.83	4	1	0	0	Repressed
269	KUMAMOTO_RESPONSE_TO_NUTLIN_3A_DN	-1.83	4	0	0	0	Repressed
270	PUJANA_BRCA1_PCC_NETWORK	-1.83	4	0	0	0	Repressed
271	REACTOME_CELL_CYCLE_CHECKPOINTS	-1.83	4	0	0	0	Repressed
272	REACTOME_COOPERATION_OF_PDCL_PHLP1_AND_TRIC_CCT_IN_G_PROTEIN_BET	-1.83	4	0	0	0	Repressed
273	REACTOME_SIGNALING_BY_HEDGEHOG	-1.83	4	0	0	0	Repressed
274	REACTOME_TRANSCRIPTION_OF_THE_HIV_GENOME	-1.83	4	0	0	0	Repressed
275	REACTOME_TRANSPORT_OF_THE_SLBP_DEPENDANT_MATURE_MRNA	-1.83	4	0	0	0	Repressed
276	SESTO_RESPONSE_TO_UV_C0	-1.83	4	0	0	0	Repressed
277	GO_PROTEIN_LOCALIZATION_TO_NUCLEOPLASM	-1.83	2.66	0	0	0	Repressed
278	LOPEZ_MESOTELIOMA_SURVIVAL_TIME_UP	-1.83	2.66	0	0	0	Repressed
279	GO_POSITIVE_REGULATION_OF_PEPTIDYL_THREONINE_PHOSPHORYLATION	-1.83	2.62	0	0	0	Repressed
280	REACTOME_TRANSCRIPTIONAL_ACTIVATION_OF_MITOCHONDRIAL_BIOGENESIS	-1.83	2.52	0	0	0	Repressed
281	GO_NUCLEOLUS	-1.82	4	0	0	0	Repressed
282	GO_RIBONUCLEOPROTEIN_COMPLEX	-1.82	4	0	0	0	Repressed
283	REACTOME_HOST_INTERACTIONS_WITH_INFLUENZA_FACTORS	-1.82	4	0	0	0	Repressed
284	REACTOME_RUNX1_REGULATES_TRANSCRIPTION_OF_GENES_INVOLVED_IN_DIFFE	-1.82	4	0	0	0	Repressed
285	WHITEFORD_PEDIATRIC_CANCER_MARKERS	-1.82	4	0	0	0	Repressed
286	WHITFIELD_CELL_CYCLE_LITERATURE	-1.82	4	0	0	0	Repressed
287	FINETTI_BREAST_CANCER_KINOME_RED	-1.82	2.63	0	0	0	Repressed
288	GO_RNA_CAP_BINDING	-1.82	2.6	0	0	0	Repressed
289	KEGG_RNA_POLYMERASE	-1.82	2.6	0	0	0	Repressed
290	REACTOME_TRANSPORT_OF_MATURE_MRNAS_DERIVED_FROM_INTRONLESS_TR/	-1.82	2.58	0	0	0	Repressed
291	GO_NCRNA_EXPORT_FROM_NUCLEUS	-1.82	2.56	0	0	0	Repressed
292	GO_TBP_CLASS_PROTEIN_BINDING	-1.82	2.32	0	0	0	Repressed
293	BENPORATH_PROLIFERATION	-1.81	4	0	0	0	Repressed
294	BIOCARTA_EGFR_SMRTE_PATHWAY	-1.81	4	0	0	0	Repressed
295	BLALOCK_ALZHEIMERS_DISEASE_INCIPIENT_DN	-1.81	4	0	0	0	Repressed
296	DANG_MYC_TARGETS_UP	-1.81	4	0	1	0	Repressed
297	GO_MITOCHONDRIAL_RNA_PROCESSING	-1.81	4	0	0	0	Repressed
298	GO_NUCLEOTIDYLTRANSFERASE_ACTIVITY	-1.81	4	0	0	0	Repressed
299	GO_REGULATION_OF_MRNA_CATABOLIC_PROCESS	-1.81	4	0	0	0	Repressed
300	GO_RNA_POLYMERASE_ACTIVITY	-1.81	4	0	0	0	Repressed
301	GO_SIGNAL_SEQUENCE_BINDING	-1.81	4	0	0	0	Repressed
302	GO_SISTER_CHROMATID_COHESION	-1.81	4	0	0	0	Repressed
303	LE_EGR2_TARGETS_UP	-1.81	4	0	0	0	Repressed
304	MILI_PSEUDOPODIA_HAPTOTAXIS_UP	-1.81	4	0	0	0	Repressed
305	ONDER_CDH1_TARGETS_1_DN	-1.81	4	0	0	0	Repressed
306	REACTOME_DECTIN_1_MEDIATED_NONCANONICAL_NF_KB_SIGNALING	-1.81	4	0	0	0	Repressed
307	REACTOME_MHC_CLASS_II_ANTIGEN_PRESENTATION	-1.81	4	0	0	0	Repressed
308	VISALA_RESPONSE_TO_HEAT_SHOCK_AND_AGING_DN	-1.81	4	0	0	0	Repressed
309	ZHANG_TLX_TARGETS_36HR_DN	-1.81	4	0	0	0	Repressed
310	GO_SNORNA_BINDING	-1.81	2.61	0	0	0	Repressed
311	GO_CATALYTIC_STEP_2_SPLICEOSOME	-1.80	4	0	0	0	Repressed
312	GO_CHROMOSOME_SEPARATION	-1.80	4	0	0	0	Repressed
313	GO_CYTOSOLIC_PROTEASOME_COMPLEX	-1.80	4	0	0	0	Repressed
314	GO_REGULATION_OF_CELLULAR_AMINO_ACID_METABOLIC_PROCESS	-1.80	4	0	0	0	Repressed
315	GO_REGULATION_OF_MEIOTIC_NUCLEAR_DIVISION	-1.80	4	0	0	0	Repressed

316	GO_TRNA_PROCESSING	-1.80	4	0	0	0	Repressed
317	HALLMARK_MITOTIC_SPINDLE	-1.80	4	0	0	0	Repressed
318	REACTOME INHIBITION OF THE PROTEOLYTIC ACTIVITY OF APC C REQUIRED	-1.80	4	0	0	0	Repressed
319	GO_REGULATION_OF_ATTACHMENT_OF_SPINDLE_MICROTUBULES_TO_KINETOCH	-1.80	2.63	0	0	0	Repressed
320	KARAKAS_TGFB1_SIGNALING	-1.80	2.63	0	0	0	Repressed
321	GO_REGULATION_OF_DNA_METHYLATION	-1.80	1.8	0	0	0	Repressed
322	GO_DNA_TEMPLATED_TRANSCRIPTION_TERMINATION	-1.79	4	0	0	0	Repressed
323	GO_KINETOCHORE	-1.79	4	0	0	0	Repressed
324	GO_POSITIVE_REGULATION_OF_UBIQUITIN_PROTEIN_LIGASE_ACTIVITY	-1.79	4	0	0	0	Repressed
325	GO_PRE_SNORNP_COMPLEX	-1.79	4	0	0	0	Repressed
326	GO_REGULATION_OF_CYTOKINESIS	-1.79	4	0	0	0	Repressed
327	GO_RIBONUCLEOPROTEIN_COMPLEX_SUBUNIT_ORGANIZATION	-1.79	4	0	0	0	Repressed
328	GO_RIBOSOME_ASSEMBLY	-1.79	4	0	0	0	Repressed
329	GO_RNA_MODIFICATION	-1.79	4	0	0	0	Repressed
330	GO_SUBSTANTIA_NIGRA_DEVELOPMENT	-1.79	4	0	0	0	Repressed
331	GO_TRANSCRIPTION_BY_RNA_POLYMERASE_I	-1.79	4	0	0	0	Repressed
332	GO_NUCLEAR_LOCALIZATION_SEQUENCE_BINDING	-1.79	2.62	0	0	0	Repressed
333	GO_TELOMERASE_HOLOENZYME_COMPLEX	-1.79	2.62	0	0	0	Repressed
334	GO_POSITIVE_REGULATION_OF_CYTOKINESIS	-1.79	2.61	0	0	0	Repressed
335	GO_MEMBRANE_DISASSEMBLY	-1.79	2.31	0	0	0	Repressed
336	CHEMNITZ_RESPONSE_TO_PROSTAGLANDIN_E2_UP	-1.78	4	0	0	0	Repressed
337	GO_IMPORT_INTO_NUCLEUS	-1.78	4	0	0	0	Repressed
338	GO_MORPHOGENESIS_OF_A_POLARIZED_EPITHELIUM	-1.78	4	0	0	0	Repressed
339	GO_MRNA_METABOLIC_PROCESS	-1.78	4	0	0	0	Repressed
340	GO_MULTI_ORGANISM_LOCALIZATION	-1.78	4	0	0	0	Repressed
341	GRAHAM_CML_QUIESCENT_VS_NORMAL_QUIESCENT_UP	-1.78	4	0	0	0	Repressed
342	LEE_METASTASIS_AND_ALTERNATIVE_SPLICING_DN	-1.78	4	0	0	0	Repressed
343	REACTOME_BMAL1:CLOCK_NPAS2_ACTIVATES_CIRCADIAN_GENE_EXPRESSION	-1.78	4	0	0	0	Repressed
344	REACTOME_CONDENSATION_OF_PROPHASE_CHROMOSOMES	-1.78	4	0	0	0	Repressed
345	REACTOME_NUCLEAR_ENVELOPE_BREAKDOWN	-1.78	4	0	0	0	Repressed
346	GO_RNA_5_END_PROCESSING	-1.78	2.62	0	0	0	Repressed
347	REACTOME_METABOLISM_OF_POLYAMINES	-1.78	2.54	0	0	0	Repressed
348	REACTOME_HEDGEHOG_LIGAND_BIOGENESIS	-1.78	2.23	0	0	0	Repressed
349	GO_CELL_DIVISION	-1.77	4	0	0	0	Repressed
350	GO POSITIVE REGULATION OF ESTABLISHMENT OF PROTEIN LOCALIZATION TC	-1.77	4	0	0	0	Repressed
351	GO_REGULATION_OF_CELLULAR_AMINE_METABOLIC_PROCESS	-1.77	4	0	0	0	Repressed
352	GO_RNA_POLYMERASE_COMPLEX	-1.77	4	0	0	0	Repressed
353	REACTOME_ACTIVATION_OF_NIMA_KINASES_NEK9_NEK6_NEK7	-1.77	4	0	0	0	Repressed
354	REACTOME_CIRCADIAN_CLOCK	-1.77	4	0	0	0	Repressed
355	REACTOME_MITOTIC_PROMETAPHASE	-1.77	4	0	0	0	Repressed
356	GO_CENTROSOME_SEPARATION	-1.77	2.64	0	0	0	Repressed
357	FOURNIER_ACINAR_DEVELOPMENT_LATE_DN	-1.77	2.62	0	0	0	Repressed
358	DEN_INTERACT_WITH_LCA5	-1.77	2.61	0	0	0	Repressed
359	EGUCHI_CELL_CYCLE_RB1_TARGETS	-1.77	2.59	0	0	1	Repressed
360	GO_TRNA_METHYLTRANSFERASE_ACTIVITY	-1.77	2.59	0	0	0	Repressed
361	KEGG_BASAL_TRANSCRIPTION_FACTORS	-1.77	2.57	0	0	0	Repressed
362	SHIPP_DLBCL_VS_FOLLICULAR_LYMPHOMA_UP	-1.77	2.57	0	0	0	Repressed
363	REACTOME_KINESINS	-1.77	2.56	0	0	0	Repressed
364	GO_SIRNA_BINDING	-1.77	2.34	0	0	0	Repressed
365	SIMBULAN_PARP1_TARGETS_DN	-1.77	2.34	0	0	0	Repressed
366	REACTOME_FORMATION_OF_TUBULIN_FOLDING_INTERMEDIATES_BY_CCT_TRIC	-1.77	2.33	0	0	0	Repressed
367	REACTOME SYNTHESIS OF ACTIVE UBIQUITIN: ROLES OF E1 AND E2 ENZYMES	-1.77	2.16	0	0	0	Repressed
368	BHAT_ESR1_TARGETS_VIA_AKT1_UP	-1.76	4	0	0	0	Repressed
369	GO INNATE IMMUNE RESPONSE ACTIVATING CELL SURFACE RECEPTOR SIGNAI	-1.76	4	0	0	0	Repressed
370	GO_REGULATION_OF_RNA_METABOLIC_PROCESS	-1.76	4	0	0	0	Repressed
371	GO_REGULATION_OF_STEM_CELL_DIFFERENTIATION	-1.76	4	0	0	0	Repressed
372	GO_SNRNA_TRANSCRIPTION	-1.76	4	0	0	0	Repressed
373	REACTOME_HIV_LIFE_CYCLE	-1.76	4	0	0	0	Repressed
374	REACTOME_PHOSPHORYLATION_OF_EMI1	-1.76	2.67	0	0	0	Repressed
375	COLLIS_PRKDC_REGULATORS	-1.76	2.6	0	0	0	Repressed
376	GO_GENERAL_TRANSCRIPTION_INITIATION_FACTOR_BINDING	-1.76	2.59	0	0	0	Repressed
377	REACTOME_EXPORT_OF_VIRAL_RIBONUCLEOPROTEINS_FROM_NUCLEUS	-1.76	2.57	0	0	0	Repressed
378	SPIRA_SMOKERS_LUNG_CANCER_DN	-1.76	2.34	0	0	0	Repressed
379	DACOSTA_UV_RESPONSE_VIA_ERCC3_COMMON_DN	-1.75	4	0	0	0	Repressed
380	DACOSTA_UV_RESPONSE_VIA_ERCC3_DN	-1.75	4	0	0	0	Repressed
381	GO_CONDENSED_CHROMOSOME_CENTROMERIC_REGION	-1.75	4	0	0	0	Repressed
382	GO_DNA_TEMPLATED_TRANSCRIPTION_INITIATION	-1.75	4	0	0	0	Repressed
383	GO_MRNA_EXPORT_FROM_NUCLEUS	-1.75	4	0	0	0	Repressed
384	GO_SPINDLE_ASSEMBLY	-1.75	4	0	0	0	Repressed
385	HALLMARK_E2F_TARGETS	-1.75	4	0	0	0	Repressed
386	MARCHINI_TRABECTEDIN_RESISTANCE_DN	-1.75	4	0	0	0	Repressed
387	REACTOME_GOLGI_CISTERNAE_PERICENTRIOLAR_STACK_REORGANIZATION	-1.75	4	0	0	0	Repressed
388	REACTOME_REGULATION_OF_MRNA_STABILITY_BY_PROTEINS_THAT_BIND_AU_RI	-1.75	4	0	0	0	Repressed
389	REACTOME_TRNA_PROCESSING_IN_THE_NUCLEUS	-1.75	4	0	0	0	Repressed
390	VANHARANTA_UTERINE_FIBROID_WITH_7Q_DELETION_UP	-1.75	4	0	0	0	Repressed
391	FUNG_IL2_SIGNALING_1	-1.75	2.64	0	0	0	Repressed
392	GO_NEGATIVE_REGULATION_OF_MRNA_SPLICING_VIA_SPLICEOSOME	-1.75	2.63	0	0	0	Repressed
393	GO_CONDENSED_NUCLEAR_CHROMOSOME_CENTROMERIC_REGION	-1.75	2.62	0	0	0	Repressed
394	ZEMBUTSU_SENSITIVITY_TO_NIMUSTINE	-1.75	2.62	0	0	0	Repressed
395	REACTOME_TRANSCRIPTIONAL_REGULATION_BY_SMALL_RNAS	-1.75	2.51	0	0	0	Repressed
396	GO TIM23 MITOCHONDRIAL IMPORT INNER MEMBRANE TRANSLOCASE COMPLE	-1.75	2.34	0	0	0	Repressed
397	FU_INTERACT_WITH_ALKBH8	-1.75	2.18	0	0	0	Repressed
398	XU_HGF_SIGNALING_NOT_VIA_AKT1_48HR_DN	-1.75	2.04	0	0	0	Repressed
399	GO_BOX_H_ACA_RNP_COMPLEX	-1.74	4	0	0	0	Repressed
400	GO_PROTEIN_PHOSPHORYLATED_AMINO_ACID_BINDING	-1.74	4	0	0	0	Repressed
401	GO_REGULATION_OF_PEPTIDYL_THREONINE_PHOSPHORYLATION	-1.74	4	0	0	0	Repressed
402	GO REGULATION OF TRANSCRIPTION FROM RNA POLYMERASE II PROMOTER	-1.74	4	0	0	0	Repressed
403	GO_STRUCTURAL_CONSTITUENT_OF_NUCLEAR_PORE	-1.74	4	0	0	0	Repressed
404	REACTOME_SUMOYLATION_OF_UBIQUITINYLATION_PROTEINS	-1.74	4	0	0	0	Repressed
405	REACTOME_TRANSCRIPTIONAL_REGULATION_BY_RUNX3	-1.74	4	0	0	0	Repressed
406	REACTOME PHOSPHORYLATION SITE MUTANTS OF CTNNB1 ARE NOT TARGETI	-1.74	2.63	0	0	0	Repressed
407	REACTOME_DEFECTIVE_TPR_MAY_CONFER_SUSCEPTIBILITY_TOWARDS_THYROID	-1.74	2.57	0	0	0	Repressed
408	GO_REGULATION_OF_HEMATOPOIETIC_PROGENITOR_CELL_DIFFERENTIATION	-1.74	2.54	0	0	0	Repressed
409	GO_REGULATION_OF_ESTABLISHMENT_OF_PLANAR_POLARITY	-1.74	2.47	0	0	0	Repressed
410	GO_CHAPERONIN_CONTAINING_T_COMPLEX	-1.74	2.2	0	0	0	Repressed
411	GO_TRNA_METHYLATION	-1.74	2.1	0	0	0	Repressed
412	EPPERT_PROGENITOR	-1.73	4	0	0	0	Repressed
413	GO_ALTERNATIVE_MRNA_SPLICING_VIA_SPLICEOSOME	-1.73	4	0	0	0	Repressed
414	GO_RESPONSE_TO_HEAT	-1.73	4	0	0	0	Repressed
415	GO_TRANSPORT_OF_VIRUS	-1.73	4	0	0	0	Repressed
416	GO_UNFOLDED_PROTEIN_BINDING	-1.73	4	0	0	0	Repressed
417	REACTOME_MITOTIC_G2_G2_M_PHASES	-1.73	4	0	0	0	Repressed
418	RODRIGUES_THYROID_CARCINOMA_POORLY_DIFFERENTIATED_UP	-1.73	4	0	0	0	Repressed
419	GO_POSITIVE_REGULATION_OF_RIBOSOME_BIOGENESIS	-1.73	2.66	0	0	0	Repressed
420	MONTERO_THYROID_CANCER_POOR_SURVIVAL_UP	-1.73	2.66	0	0	0	Repressed
421	GO_MICROTUBULE_PLUS_END	-1.73	2.6	0	0	0	Repressed
422	GO_MICROTUBULE_MOTOR_ACTIVITY	-1.73	2.51	0	0	0	Repressed
423	GO_PROTEIN_LOCALIZATION_TO_CYTOPLASMIC_STRESS_GRANULE	-1.73	2.35	0	0	0	Repressed
424	GO_RNA_CAPPING	-1.73	2.31	0	0	0	Repressed

425	GO_KINESIN_COMPLEX	-1.73	2.25	0	0	0	Repressed
426	GO_SPINDLE_LOCALIZATION	-1.73	2.25	0	0	0	Repressed
427	GO_ATP_DEPENDENT_MICROTUBULE_MOTOR_ACTIVITY	-1.73	2.12	0	0	0	Repressed
428	GO_NEURAL_NUCLEUS_DEVELOPMENT	-1.73	1.98	0	0	0	Repressed
429	REACTOME_HSF1_DEPENDENT_TRANSACTIVATION	-1.73	1.96	0	0	0	Repressed
430	BILD_MYC_ONCOGENIC_SIGNATURE	-1.72	4	0	1	0	Repressed
431	BOYAULT_LIVER_CANCER_SUBCLASS_G123_UP	-1.72	4	0	0	0	Repressed
432	CONCANNON_APOPTOSIS_BY_EPOXOMICIN_DN	-1.72	4	0	0	0	Repressed
433	FISCHER_DREAM_TARGETS	-1.72	4	0	0	0	Repressed
434	GO_BOX_C_D_SNORNP_ASSEMBLY	-1.72	4	0	0	0	Repressed
435	GO_BOX_H_ACA_SNORNA_BINDING	-1.72	4	0	0	0	Repressed
436	GO_NUCLEAR_PERIPHERY	-1.72	4	0	0	0	Repressed
437	GO_REGULATION_OF_PROTEIN_DEPHOSPHORYLATION	-1.72	4	0	0	0	Repressed
438	GO_REGULATION_OF_UBIQUITIN_PROTEIN_LIGASE_ACTIVITY	-1.72	4	0	0	0	Repressed
439	INAMURA_LUNG_CANCER_SCC_UP	-1.72	4	0	0	0	Repressed
440	PETROVA_ENDOTHELIUM_LYMPHATIC_VS_BLOOD_UP	-1.72	4	0	0	0	Repressed
441	REACTOME_ANTIVIRAL_MECHANISM_BY_IFN_STIMULATED_GENES	-1.72	4	0	0	0	Repressed
442	REACTOME_FOLDING_OF_ACTIN_BY_CCT_TRIC	-1.72	4	0	0	0	Repressed
443	REACTOME_REGULATION_OF_HSF1_MEDIATED_HEAT_SHOCK_RESPONSE	-1.72	4	0	0	0	Repressed
444	WINTER_HYPOXIA_UP	-1.72	4	0	0	0	Repressed
445	GO_SMALL_NUCLEOLAR_RIBONUCLEOPROTEIN_COMPLEX_ASSEMBLY	-1.72	2.66	0	0	0	Repressed
446	FUNG_IL2_TARGETS_WITH_STAT5_BINDING_SITES	-1.72	2.65	0	0	0	Repressed
447	GO_NEURON_PROJECTION_CYTOPLASM	-1.72	2.53	0	0	0	Repressed
448	HU_GENOTOXIC_DAMAGE_4HR	-1.72	2.3	0	0	0	Repressed
449	GO_REGULATION_OF_ESTABLISHMENT_OF_PROTEIN_LOCALIZATION_TO_CHROMO	-1.72	2.02	0	0	0	Repressed
450	REACTOME_ATTENUATION_PHASE	-1.72	2	0	0	0	Repressed
451	LIANG_SILENCED_BY_METHYLATION_UP	-1.72	1.87	0	0	0	Repressed
452	GO_CELLULAR_RESPONSE_TO_HEAT	-1.71	4	0	0	0	Repressed
453	GO_MITOTIC_NUCLEAR_DIVISION	-1.71	4	0	0	0	Repressed
454	GO_REGULATION_OF_MRNA_PROCESSING	-1.71	4	0	0	0	Repressed
455	GO_REGULATION_OF_TRANSLATIONAL_TERMINATION	-1.71	4	0	0	0	Repressed
456	REACTOME_BETA_CATENIN_INDEPENDENT_WNT_SIGNALING	-1.71	4	0	0	0	Repressed
457	REACTOME_CELLULAR_RESPONSE_TO_HEAT_STRESS	-1.71	4	0	0	0	Repressed
458	TOYOTA_TARGETS_OF_MIR34B_AND_MIR34C	-1.71	4	0	0	0	Repressed
459	WHITFIELD_CELL_CYCLE_G2	-1.71	4	0	0	0	Repressed
460	XU_RESPONSE_TO_TRETINOIN_AND_NSC682994_DN	-1.71	4	0	0	0	Repressed
461	GO_HISTONE_SERINE_PHOSPHORYLATION	-1.71	2.67	0	0	0	Repressed
462	GO_RIBOSOMAL_PROTEIN_IMPORT_INTO_NUCLEUS	-1.71	2.65	0	0	0	Repressed
463	CHR17Q22	-1.71	2.59	0	0	0	Repressed
464	KENNY_CTNNB1_TARGETS_UP	-1.71	2.57	0	0	0	Repressed
465	KEGG_PROGESTERONE_MEDIATED_OOCYTE_MATURATION	-1.71	2.55	0	0	0	Repressed
466	REACTOME_DEFECTIVE_CFTR_CAUSES_CYSTIC_FIBROSIS	-1.71	2.53	0	0	0	Repressed
467	REACTOME_FGFR2_ALTERNATIVE_SPLICING	-1.71	2.34	0	0	0	Repressed
468	REACTOME_RORA_ACTIVATES_GENE_EXPRESSION	-1.71	2.34	0	0	0	Repressed
469	GO_RNA_HELICASE_ACTIVITY	-1.71	2.26	0	0	0	Repressed
470	REACTOME_MRNA_CAPPING	-1.71	2.26	0	0	0	Repressed
471	REACTOME_COOPERATION_OF_PREFOLDIN_AND_TRIC_CCT_IN_ACTIN_AND_TUBU	-1.71	1.94	0	0	0	Repressed
472	BHATTACHARYA_EMBRYONIC_STEM_CELL	-1.70	4	0	0	0	Repressed
473	CHR1P34	-1.70	4	0	0	0	Repressed
474	GO_POSITIVE_REGULATION_OF_CANONICAL_WNT_SIGNALING_PATHWAY	-1.70	4	0	0	0	Repressed
475	GO_RNA_SPLICING	-1.70	4	0	0	0	Repressed
476	GRADE_COLON_AND_RECTAL_CANCER_UP	-1.70	4	0	0	0	Repressed
477	LI_WILMS_TUMOR_VS_FETAL_KIDNEY_1_DN	-1.70	4	0	0	0	Repressed
478	BIOCARTA_RARRXR_PATHWAY	-1.70	2.65	0	0	0	Repressed
479	CHENG_RESPONSE_TO_NICKEL_ACETATE	-1.70	2.56	0	0	0	Repressed
480	REACTOME_MITOCHONDRIAL_PROTEIN_IMPORT	-1.70	2.53	0	0	0	Repressed
481	GO_POSITIVE_REGULATION_OF_RNA_METABOLIC_PROCESS	-1.70	2.5	0	0	0	Repressed
482	GO_VENTRICULAR_CARDIAC_MUSCLE_CELL_DIFFERENTIATION	-1.70	2.33	0	0	0	Repressed
483	REACTOME_PREFOLDIN_MEDIATED_TRANSFER_OF_SUBSTRATE_TO_CCT_TRIC	-1.70	2.3	0	0	0	Repressed
484	GO_NUCLEAR_EXPORT_SIGNAL_RECEPTOR_ACTIVITY	-1.70	2.2	0	0	0	Repressed
485	REACTOME_TRANSPORT_OF_MATURE_TRANSCRIPT_TO_CYTOPLASM	-1.70	2.2	0	0	0	Repressed
486	LE_NEURONAL_DIFFERENTIATION_DN	-1.70	2.14	0	0	0	Repressed
487	WAESCH_ANAPHASE_PROMOTING_COMPLEX	-1.70	2.06	0	0	0	Repressed
488	GO_PROTEIN_PHOSPHATASE_TYPE_2A_COMPLEX	-1.70	2.05	0	0	0	Repressed
489	CHR5Q32	-1.70	2.04	0	0	0	Repressed
490	BIOCARTA_EIF2_PATHWAY	-1.69	4	0	0	0	Repressed
491	GO_BOX_H_ACA_SNORNP_COMPLEX	-1.69	4	0	0	0	Repressed
492	GO_CHROMOSOME_LOCALIZATION	-1.69	4	0	0	0	Repressed
493	GO_CHROMOSOME_PASSENGER_COMPLEX	-1.69	4	0	0	0	Repressed
494	GO_NEGATIVE_REGULATION_OF_CELL_CYCLE_G2_M_PHASE_TRANSITION	-1.69	4	0	0	0	Repressed
495	GO_NEGATIVE_REGULATION_OF_NUCLEAR_DIVISION	-1.69	4	0	0	0	Repressed
496	GO_PROTEIN_LOCALIZATION_TO_CHROMOSOME	-1.69	4	0	0	0	Repressed
497	GO_REGULATION_OF_MORPHOGENESIS_OF_AN_EPITHELIUM	-1.69	4	0	0	0	Repressed
498	MITSIADES_RESPONSE_TO_APLIDIN_DN	-1.69	4	0	0	0	Repressed
499	REACTOME_TRNA_MODIFICATION_IN_THE_NUCLEUS_AND_CYTOSOL	-1.69	4	0	0	0	Repressed
500	SESTO_RESPONSE_TO_UV_C5	-1.69	4	0	0	0	Repressed
501	GO_SAGA_COMPLEX	-1.69	2.63	0	0	0	Repressed
502	REACTOME_CYCLIN_A_B1_B2_ASSOCIATED_EVENTS_DURING_G2_M_TRANSITION	-1.69	2.6	0	0	0	Repressed
503	GO_TOXIN_TRANSPORT	-1.69	2.58	0	0	0	Repressed
504	JIANG_HYPOXIA_CANCER	-1.69	2.57	0	0	0	Repressed
505	REACTOME_RNA_POLYMERASE_II_TRANSCRIPTION_ELONGATION	-1.69	2.53	0	0	0	Repressed
506	GO_CHROMOSOME_MOVEMENT_TOWARDS_SPINDLE_POLE	-1.69	2.37	0	0	0	Repressed
507	GO_REGULATION_OF_WOUND_HEALING_SPREADING_OF_EPIDERMAL_CELLS	-1.69	2.37	0	0	0	Repressed
508	GO_ATP_DEPENDENT_MICROTUBULE_MOTOR_ACTIVITY_PLUS_END_DIRECTED	-1.69	2.32	0	0	0	Repressed
509	GO_UBIQUITIN_LIKE_PROTEIN_CONJUGATING_ENZYME_ACTIVITY	-1.69	2.28	0	0	0	Repressed
510	GO_NUCLEAR_IMPORT_SIGNAL_RECEPTOR_ACTIVITY	-1.69	2.19	0	0	0	Repressed
511	GO_DE_NOVO_PROTEIN_FOLDING	-1.69	2.13	0	0	0	Repressed
512	GO_EXON_EXON_JUNCTION_COMPLEX	-1.69	2.04	0	0	0	Repressed
513	GO_RRNA_CONTAINING_RIBONUCLEOPROTEIN_COMPLEX_EXPORT_FROM_NUCLEU	-1.69	1.92	0	0	0	Repressed
514	GO_AXON_HILLOCK	-1.68	4	0	0	0	Repressed
515	GO_CHROMOSOME_SEGREGATION	-1.68	4	0	0	0	Repressed
516	GO_ENDONUCLEOLYTIC_CLEAVAGE_IN_5_ETS_OF_TRICISTRONIC_RRNA_TRANSCI	-1.68	4	0	0	0	Repressed
517	GO_KINETOCHORE_BINDING	-1.68	4	0	0	0	Repressed
518	GO_NUCLEAR_ENVELOPE_ORGANIZATION	-1.68	4	0	0	0	Repressed
519	GO_NUCLEOLUS_ORGANIZATION	-1.68	4	0	0	0	Repressed
520	GO_PROTEIN_DEPHOSPHORYLATION	-1.68	4	0	0	0	Repressed
521	KIM_WT1_TARGETS_8HR_UP	-1.68	4	0	0	0	Repressed
522	REACTOME_GENE_SILENCING_BY_RNA	-1.68	4	0	0	0	Repressed
523	REACTOME_ORGANIC_CATION_TRANSPORT	-1.68	4	0	0	0	Repressed
524	REACTOME_RHO_GTPASE_EFFECTORS	-1.68	4	0	0	0	Repressed
525	REACTOME_TCF_DEPENDENT_SIGNALING_IN_RESPONSE_TO_WNT	-1.68	4	0	0	0	Repressed
526	GO_AXO_DENDRITIC_TRANSPORT	-1.68	2.56	0	0	0	Repressed
527	GO_TRANSCRIPTION_INITIATION_FROM_RNA_POLYMERASE_II_PROMOTER	-1.68	2.4	0	0	0	Repressed
528	GO_UBIQUITIN_RECYCLING	-1.68	2.37	0	0	0	Repressed
529	GO_REGULATION_OF_CARDIAC_CONDUCTION	-1.68	2.29	0	0	0	Repressed
530	GO_ENDONUCLEOLYTIC_CLEAVAGE_INVOLVED_IN_RRNA_PROCESSING	-1.68	2.18	0	0	0	Repressed
531	GO_NEGATIVE_REGULATION_OF_MRNA_PROCESSING	-1.68	2.12	0	0	0	Repressed
532	REACTOME_REGULATION_OF_GLYCOLYSIS_BY_FRUCTOSE_2_6_BISPHOSPHATE_I	-1.68	1.87	0	0	0	Repressed
533	BORCZUK_MALIGNANT_MESOTHELIOMA_UP	-1.67	4	0	0	0	Repressed

534	DAZARD_RESPONSE_TO_UV_NHEK_DN	-1.67	4	0	0	0	Repressed
535	GO_NUCLEAR_CHROMOSOME_SEGREGATION	-1.67	4	0	0	0	Repressed
536	GO_RNA_METHYLTRANSFERASE_ACTIVITY	-1.67	4	0	0	0	Repressed
537	GROSS_HYPOXIA_VIA_ELK3_UP	-1.67	4	0	0	0	Repressed
538	TONKS_TARGETS_OF_RUNX1_RUNX1T1_FUSION_MONOCYTE_UP	-1.67	4	0	0	0	Repressed
539	REACTOME_SIGNALING_BY_FGFR	-1.67	2.54	0	0	0	Repressed
540	MOLENAAR_TARGETS_OF_CCND1_AND_CDK4_DN	-1.67	2.25	0	0	0	Repressed
541	GO_RIBOSOMAL_SMALL_SUBUNIT_BINDING	-1.67	2.05	0	0	0	Repressed
542	GO_3_5_RNA_HELICASE_ACTIVITY	-1.67	2.01	0	0	0	Repressed
543	GO_TRNA_THREONYLCARBAMOYLADENOSINE_METABOLIC_PROCESS	-1.67	1.94	1	0	0	Repressed
544	GO_FEMALE_MEIOTIC_NUCLEAR_DIVISION	-1.67	1.93	0	0	0	Repressed
545	BIOCARTA_VDR_PATHWAY	-1.67	1.85	0	0	0	Repressed
546	KORKOLA_TERATOMA	-1.67	1.84	0	0	0	Repressed
547	JAEGER_METASTASIS_UP	-1.67	1.8	0	0	0	Repressed
548	REACTOME_IMPORT_OF_PALMITOYL_COA_INTO_THE_MITOCHONDRIAL_MATRIX	-1.67	1.7	0	0	0	Repressed
549	BOYAULT_LIVER_CANCER_SUBCLASS_G23_UP	-1.66	4	0	0	0	Repressed
550	BROWNE_HCMV_INFECTION_20HR_UP	-1.66	4	0	0	0	Repressed
551	GO_MICROTUBULE_ASSOCIATED_COMPLEX	-1.66	4	0	0	0	Repressed
552	GO_MITOCHONDRIAL_GENE_EXPRESSION	-1.66	4	0	0	0	Repressed
553	GO_MRNA_BINDING	-1.66	4	0	0	0	Repressed
554	GO_PROTEIN_TRANSPORTER_ACTIVITY	-1.66	4	0	0	0	Repressed
555	GO_REGULATION_OF_POSTTRANSCRIPTIONAL_GENE_SILENCING	-1.66	4	0	0	0	Repressed
556	GO_RNA_3_END_PROCESSING	-1.66	4	0	0	0	Repressed
557	REACTOME_FORMATION_OF_TC_NER_PRE_INCISION_COMPLEX	-1.66	4	0	0	0	Repressed
558	REACTOME_NEDDYLATION	-1.66	4	0	0	0	Repressed
559	GO_DNA_TOPOISOMERASE_ACTIVITY	-1.66	2.67	0	0	0	Repressed
560	LIU_CDX2_TARGETS_UP	-1.66	2.63	0	0	0	Repressed
561	GO_EPITHELIAL_STRUCTURE_MAINTENANCE	-1.66	2.6	0	0	0	Repressed
562	YANG_BREAST_CANCER_ESR1_LASER_DN	-1.66	2.56	0	0	0	Repressed
563	GO_DENDRITIC_TRANSPORT	-1.66	2.39	0	0	0	Repressed
564	WU_APOPTOSIS_BY_CDKN1A_VIA_TP53	-1.66	2.25	0	0	0	Repressed
565	GO_REGULATION_OF_NUCLEOCYTOPLASMIC_TRANSPORT	-1.66	2.24	0	0	0	Repressed
566	GO_POSITIVE_REGULATION_OF_TYPE_I_INTERFERON_PRODUCTION	-1.66	2.23	0	0	0	Repressed
567	GO_NEGATIVE_REGULATION_OF_CARTILAGE_DEVELOPMENT	-1.66	2.19	0	0	0	Repressed
568	GO_CHROMOCENTER	-1.66	2.17	0	0	0	Repressed
569	BILANGES_SERUM_SENSITIVE_VIA_TSC2	-1.66	2.15	0	0	0	Repressed
570	GO_ATTACHMENT_OF_SPINDLE_MICROTUBULES_TO_KINETOCHORE	-1.66	2.11	0	0	0	Repressed
571	REACTOME_SIGNALING_BY_FGFR2	-1.66	2.09	0	0	0	Repressed
572	REACTOME_CONDENSATION_OF_PROMETAPHASE_CHROMOSOMES	-1.66	2.05	0	0	0	Repressed
573	REACTOME_SIGNALING_BY_FGFR2_IIIA_TM	-1.66	1.94	0	0	0	Repressed
574	GO_WOUND_HEALING_SPREADING_OF_EPIDERMAL_CELLS	-1.66	1.93	0	0	0	Repressed
575	CHANDRAN_METASTASIS_TOP50_UP	-1.66	1.9	0	0	0	Repressed
576	REACTOME_PLATELET_SENSITIZATION_BY_LDL	-1.66	1.86	0	0	0	Repressed
577	GO_DYNEIN_INTERMEDIATE_CHAIN_BINDING	-1.66	1.85	0	0	0	Repressed
578	REACTOME_FGFR2_MUTANT_RECEPTOR_ACTIVATION	-1.66	1.85	0	0	0	Repressed
579	SMID_BREAST_CANCER_LUMINAL_A_DN	-1.66	1.66	0	0	0	Repressed
580	BASSO_B_LYMPHOCYTE_NETWORK	-1.65	4	0	0	0	Repressed
581	DING_LUNG_CANCER_EXPRESSION_BY_COPY_NUMBER	-1.65	4	0	0	0	Repressed
582	GO_AXON_CYTOPLASM	-1.65	4	0	0	0	Repressed
583	GOBERT_OLIGODENDROCYTE_DIFFERENTIATION_UP	-1.65	4	0	0	0	Repressed
584	JOHNSTONE_PARVB_TARGETS_3_DN	-1.65	4	0	0	0	Repressed
585	MARTORIATI_MDM4_TARGETS_FETAL_LIVER_DN	-1.65	4	0	0	0	Repressed
586	YORDY_RECIPROCAL_REGULATION_BY_ETS1_AND_SP100_DN	-1.65	2.57	0	0	0	Repressed
587	GO_ANAPHASE_PROMOTING_COMPLEX_BINDING	-1.65	2.38	0	0	0	Repressed
588	GO_PROTEASOME_ACTIVATING_ATPASE_ACTIVITY	-1.65	2.22	0	0	0	Repressed
589	GO_MAINTENANCE_OF_GASTROINTESTINAL_EPITHELIUM	-1.65	2.07	0	0	0	Repressed
590	GO_CONDENSED_NUCLEAR_CHROMOSOME_KINETOCHORE	-1.65	1.97	0	0	0	Repressed
591	GO_CELLULAR_RESPONSE_TO_MANGANESE_ION	-1.65	1.92	0	0	0	Repressed
592	GO_NEURON_FATE_COMMITMENT	-1.65	1.78	0	0	0	Repressed
593	GO_CELL_CYCLE_G2_M_PHASE_TRANSITION	-1.64	4	0	0	1	Repressed
594	GO_DNA_TEMPLATED_TRANSCRIPTION_ELONGATION	-1.64	4	0	0	0	Repressed
595	GO_PHOSPHOPROTEIN_BINDING	-1.64	4	0	0	0	Repressed
596	GO_REGULATION_OF_CELL_DIVISION	-1.64	4	0	0	0	Repressed
597	LASTOWSKA_NEUROBLASTOMA_COPY_NUMBER_UP	-1.64	4	0	0	0	Repressed
598	REACTOME_CELL_CYCLE_MITOTIC	-1.64	4	0	0	0	Repressed
599	REACTOME_MITOTIC_PROPHASE	-1.64	4	0	0	0	Repressed
600	REACTOME_ORGANIC_CATION_ANION_ZWITTERION_TRANSPORT	-1.64	4	0	0	0	Repressed
601	REACTOME_GOLGI_TO_ER_RETROGRADE_TRANSPORT	-1.64	2.44	0	0	0	Repressed
602	GO_BOX_H_ACA_TELOMERASE_RNP_COMPLEX	-1.64	2.36	0	0	0	Repressed
603	GO_RRNA_TRANSCRIPTION	-1.64	2.31	0	0	0	Repressed
604	GO_MITOCHONDRIAL_TRNA_PROCESSING	-1.64	2.16	0	0	0	Repressed
605	GO_MRNA_PSEUDOURIDINE_SYNTHESIS	-1.64	2.08	0	0	0	Repressed
606	GO_NEGATIVE_REGULATION_OF_MEMBRANE_PROTEIN_ECTODOMAIN_PROTEOLYS	-1.64	2.05	0	0	0	Repressed
607	HO_LIVER_CANCER_VASCULAR_INVASION	-1.64	1.99	0	0	0	Repressed
608	GO_SPLICEOSOMAL_TRI_SNRNP_COMPLEX_ASSEMBLY	-1.64	1.96	0	0	0	Repressed
609	WANG_METASTASIS_OF_BREAST_CANCER_ESR1_UP	-1.64	1.85	0	0	0	Repressed
610	GO_MRNA_5_UTR_BINDING	-1.64	1.83	0	0	0	Repressed
611	GO_PROTEIN_LOCALIZATION_TO_CHROMOSOME_TELOMERIC_REGION	-1.64	1.83	0	0	0	Repressed
612	GO_ANAPHASE_PROMOTING_COMPLEX	-1.64	1.8	0	0	0	Repressed
613	GO_U6_SNRNA_BINDING	-1.64	1.78	0	0	0	Repressed
614	GO_POSITIVE_REGULATION_OF_DNA_TEMPLATED_TRANSCRIPTION_INITIATION	-1.64	1.73	0	0	0	Repressed
615	ABRAMSON_INTERACT_WITH_AIRE	-1.63	4	0	0	0	Repressed
616	GO_NUCLEUS_ORGANIZATION	-1.63	4	0	0	0	Repressed
617	GO_POSITIVE_REGULATION_OF_WNT_SIGNALING_PATHWAY	-1.63	4	0	0	0	Repressed
618	GO_PROTEIN_CONTAINING_COMPLEX_LOCALIZATION	-1.63	4	0	0	0	Repressed
619	REACTOME_MITOCHONDRIAL_BIOGENESIS	-1.63	4	0	0	0	Repressed
620	REACTOME_SIGNALING_BY_NOTCH4	-1.63	4	0	0	0	Repressed
621	GO_ENDOCYTIC_VESICLE_LUMEN	-1.63	2.67	0	0	0	Repressed
622	GO_REGULATION_OF_PROTEIN_EXPORT_FROM_NUCLEUS	-1.63	2.6	0	0	0	Repressed
623	DAIRKEE_CANCER_PRONE_RESPONSE_BPA_E2	-1.63	2.46	0	0	0	Repressed
624	GO_POSITIVE_REGULATION_OF_TRANSCRIPTION_BY_RNA_POLYMERASE_I	-1.63	2.32	0	0	0	Repressed
625	GO_NEGATIVE_REGULATION_OF_CHROMOSOME_SEGREGATION	-1.63	2.25	0	0	0	Repressed
626	GO_REGULATION_OF_MRNA_SPLICING_VIA_SPLICEOSOME	-1.63	2.22	0	0	0	Repressed
627	REACTOME_ABC_TRANSPORTER_DISORDERS	-1.63	2.22	0	0	0	Repressed
628	ZHAN_VARIABLE_EARLY_DIFFERENTIATION_GENES_DN	-1.63	2.16	0	0	0	Repressed
629	REACTOME_SIGNALING_BY_FGFR2_IN_DISEASE	-1.63	2.13	0	0	0	Repressed
630	NAKAMURA_CANCER_MICROENVIRONMENT_DN	-1.63	2.07	0	0	0	Repressed
631	GO_ADENYLATE_CYCLASE_BINDING	-1.63	1.96	0	0	0	Repressed
632	GO_AMINOACYL_TRNA_SYNTHETASE_MULTIENZYME_COMPLEX	-1.63	1.93	0	0	0	Repressed
633	OHASHI_AURKA_TARGETS	-1.63	1.9	0	0	0	Repressed
634	HOFFMANN_SMALL_PRE_BII_TO_IMMATURE_B_LYMPHOCYTE_DN	-1.63	1.84	0	0	0	Repressed
635	REACTOME_ABORTIVE_ELONGATION_OF_HIV_1_TRANSCRIPT_IN_THE_ABSENCE_C	-1.63	1.84	0	0	0	Repressed
636	REACTOME_PIWI_INTERACTING_RNA_PIRNA_BIOGENESIS	-1.63	1.84	0	0	0	Repressed
637	GO_RNA_7_METHYLGUANOSINE_CAP_BINDING	-1.63	1.82	0	0	0	Repressed
638	GO_DICARBOXYLIC_ACID_BIOSYNTHETIC_PROCESS	-1.63	1.72	0	0	0	Repressed
639	GO_MATURATION_OF_5_8S_RRNA_FROM_TRICISTRONIC_RRNA_TRANSCRIPT_SSU	-1.63	1.71	0	0	0	Repressed
640	GO_EMBRYO_IMPLANTATION	-1.63	1.69	0	0	0	Repressed
641	NAGY_TFTC_COMPONENTS_HUMAN	-1.63	1.63	0	0	0	Repressed
642	GO_MITOTIC_SPINDLE_MIDZONE	-1.63	1.59	0	0	0	Repressed

643	BLUM_RESPONSE_TO_SALIRASIB_DN	-1.62	4	0	0	0	Repressed
644	CASORELLI_ACUTE_PROMYELOCYTIC_LEUKEMIA_DN	-1.62	4	0	0	0	Repressed
645	LI_DCP2_BOUND_MRNA	-1.62	4	0	0	0	Repressed
646	REACTOME_ACTIVATION_OF_HOX_GENES_DURING_DIFFERENTIATION	-1.62	4	0	0	0	Repressed
647	REACTOME_COPI_DEPENDENT_GOLGI_TO_ER_RETROGRADE_TRAFFIC	-1.62	4	0	0	0	Repressed
648	REACTOME_INFECTIOUS_DISEASE	-1.62	4	0	0	0	Repressed
649	REACTOME_TCR_SIGNALING	-1.62	4	0	0	0	Repressed
650	REACTOME_FORMATION_OF_THE_EARLY_ELONGATION_COMPLEX	-1.62	2.6	0	0	0	Repressed
651	HU_GENOTOXIN_ACTION_DIRECT_VS_INDIRECT_24HR	-1.62	2.59	0	0	0	Repressed
652	LIU_COMMON_CANCER_GENES	-1.62	2.55	0	0	0	Repressed
653	REACTOME_SUMOYLATION_OF_RNA_BINDING_PROTEINS	-1.62	2.24	0	0	0	Repressed
654	GO_ESTABLISHMENT_OF_MITOTIC_SPINDLE_LOCALIZATION	-1.62	2.01	0	0	0	Repressed
655	GO_MITOCHONDRIAL_RNA_MODIFICATION	-1.62	1.89	0	0	0	Repressed
656	REACTOME_SUMOYLATION_OF_SUMOYLATION_PROTEINS	-1.62	1.87	0	0	0	Repressed
657	GO_RNA_POLYMERASE_III_COMPLEX	-1.62	1.85	0	0	0	Repressed
658	YUAN_ZNF143_PARTNERS	-1.62	1.83	0	0	0	Repressed
659	GO_REGULATION_OF_MYELINATION	-1.62	1.81	0	0	0	Repressed
660	MULLIGAN_NTF3_SIGNALING_VIA_INSR_AND_IGF1R_UP	-1.62	1.8	0	0	0	Repressed
661	GO_REGULATION_OF_CELL_MATURATION	-1.62	1.59	0	0	0	Repressed
662	BENPORATH_CYCLING_GENES	-1.61	4	0	0	0	Repressed
663	CHANG_CORE_SERUM_RESPONSE_UP	-1.61	4	0	0	0	Repressed
664	GO_CONDENSED_CHROMOSOME	-1.61	4	0	0	0	Repressed
665	GO_FIBRILLAR_CENTER	-1.61	4	0	0	0	Repressed
666	GO_MICROTUBULE	-1.61	4	0	0	0	Repressed
667	GO_PLASMA_MEMBRANE_BOUNDED_CELL_PROJECTION_CYTOPLASM	-1.61	4	0	0	0	Repressed
668	GO_U4_SNRNA_BINDING	-1.61	4	0	0	0	Repressed
669	KIM_WT1_TARGETS_12HR_DN	-1.61	4	0	0	0	Repressed
670	LU_AGING_BRAIN_DN	-1.61	4	0	0	0	Repressed
671	MYC_UP.V1_UP	-1.61	4	0	1	0	Repressed
672	REACTOME_GLUCOSE_METABOLISM	-1.61	4	0	0	0	Repressed
673	YAO_TEMPORAL_RESPONSE_TO_PROGESTERONE_CLUSTER_7	-1.61	2.55	0	0	0	Repressed
674	SERVITJA_LIVER_HNF1A_TARGETS_UP	-1.61	2.51	0	0	0	Repressed
675	GO_NEGATIVE_REGULATION_OF_MEIOTIC_NUCLEAR_DIVISION	-1.61	2.37	0	0	0	Repressed
676	BHATI_G2M_ARREST_BY_2METHOXYESTRADIOL_UP	-1.61	2.21	0	0	0	Repressed
677	GAJATE_RESPONSE_TO_TRABECTEDIN_DN	-1.61	2.13	0	0	0	Repressed
678	REACTOME_NOTCH3_INTRACELLULAR_DOMAIN_REGULATES_TRANSCRIPTION	-1.61	2.04	0	0	0	Repressed
679	REACTOME_DISEASES_ASSOCIATED_WITH_O_GLYCOSYLATION_OF_PROTEINS	-1.61	2	0	0	0	Repressed
680	GO_TFIID_CLASS_TRANSCRIPTION_FACTOR_COMPLEX_BINDING	-1.61	1.98	0	0	0	Repressed
681	KOMMAGANI_TP63_GAMMA_TARGETS	-1.61	1.9	0	0	0	Repressed
682	GO_PROTEIN_LOCALIZATION_TO_NUCLEOLUS	-1.61	1.88	0	0	0	Repressed
683	GO_RIBOSOMAL_LARGE_SUBUNIT_ASSEMBLY	-1.61	1.83	0	0	0	Repressed
684	OHASHI_AURKB_TARGETS	-1.61	1.82	0	0	0	Repressed
685	GO_REGULATION_OF_NUCLEOBASE_CONTAINING_COMPOUND_TRANSPORT	-1.61	1.75	0	0	0	Repressed
686	CHR1Q43	-1.61	1.71	0	0	0	Repressed
687	GO_POSITIVE_REGULATION_OF_BEHAVIOR	-1.61	1.7	0	0	0	Repressed
688	BIOCARTA_NPC_PATHWAY	-1.61	1.68	0	0	0	Repressed
689	GO_CLATHRIN_COAT_ASSEMBLY	-1.61	1.67	0	0	0	Repressed
690	GO_SPINDLE_ELONGATION	-1.61	1.62	0	0	0	Repressed
691	BENPORATH_ES_1	-1.60	4	0	0	0	Repressed
692	GO_CARDIAC_MUSCLE_CELL_DIFFERENTIATION	-1.60	4	0	0	0	Repressed
693	GO_POSTTRANSCRIPTIONAL_REGULATION_OF_GENE_EXPRESSION	-1.60	4	0	0	0	Repressed
694	GO_REGULATION_OF_REPRODUCTIVE_PROCESS	-1.60	4	0	0	0	Repressed
695	PAL_PRMT5_TARGETS_UP	-1.60	4	0	0	0	Repressed
696	REACTOME_ANTIGEN_PROCESSING:_UBIQUITINATION_PROTEASOME_DEGRADATIC	-1.60	4	0	0	0	Repressed
697	REACTOME_SIGNALING_BY_WNT	-1.60	4	0	0	0	Repressed
698	GO_BOX_C_D_SNORNP_COMPLEX	-1.60	2.67	0	0	0	Repressed
699	ITO_PTTG1_TARGETS_DN	-1.60	2.36	0	0	0	Repressed
700	REACTOME_MAPK_FAMILY_SIGNALING_CASCADES	-1.60	2.32	0	0	0	Repressed
701	CROSBY_E2F4_TARGETS	-1.60	2.19	0	0	0	Repressed
702	BYSTROEM_CORRELATED_WITH_IL5_DN	-1.60	2.08	0	0	0	Repressed
703	GO_EXTRINSIC_COMPONENT_OF_POSTSYNAPTIC_MEMBRANE	-1.60	2.06	0	0	0	Repressed
704	GO_MEIOTIC_CELL_CYCLE_CHECKPOINT	-1.60	2.06	0	0	1	Repressed
705	BIOCARTA_AKAP95_PATHWAY	-1.60	1.96	0	0	0	Repressed
706	GO_RNA_POLYMERASE_I_ACTIVITY	-1.60	1.89	0	0	0	Repressed
707	GO_MATURATION_OF_5_8S_RRNA	-1.60	1.82	0	0	0	Repressed
708	PID_RANBP2_PATHWAY	-1.60	1.78	0	0	0	Repressed
709	GO_PROTEIN_SERINE_THREONINE_KINASE_ACTIVATOR_ACTIVITY	-1.60	1.77	0	0	0	Repressed
710	BERENJENO_TRANSFORMED_BY_RHOA_REVERSIBLY_DN	-1.60	1.71	0	0	0	Repressed
711	GO_ANTERIOR_POSTERIOR_AXIS_SPECIFICATION	-1.60	1.7	0	0	0	Repressed
712	PID_RETINOIC_ACID_PATHWAY	-1.60	1.66	0	0	0	Repressed
713	GO_CHROMOSOMAL_REGION	-1.59	4	0	0	0	Repressed
817	GO_MITOTIC_CELL_CYCLE	-1.55	4	0	0	1	Repressed
818	GO_REGULATION_OF_CELL_CYCLE_G2_M_PHASE_TRANSITION	-1.55	4	0	0	1	Repressed
716	RAMALHO_STEMNESS_UP	-1.59	4	0	0	0	Repressed
717	SENESE_HDAC1_TARGETS_UP	-1.59	4	0	0	0	Repressed
718	GO_TELOMERE_MAINTENANCE_VIA_TELOMERE_LENGTHENING	-1.59	2.49	0	0	0	Repressed
719	BERNARD_PPAPDC1B_TARGETS_DN	-1.59	2.06	0	0	0	Repressed
720	GO_T_CELL_EXTRAVASATION	-1.59	2.06	0	0	0	Repressed
721	MOREAUX_B_LYMPHOCYTE_MATURATION_BY_TACI_DN	-1.59	2.03	0	0	0	Repressed
722	GO_POSITIVE_REGULATION_OF_CELL_DIVISION	-1.59	1.96	0	0	0	Repressed
723	GO_REGULATION_OF_PHOSPHOPROTEIN_PHOSPHATASE_ACTIVITY	-1.59	1.91	0	0	0	Repressed
724	GO_MICROTUBULE_PLUS_END_BINDING	-1.59	1.86	0	0	0	Repressed
725	REACTOME_REGULATION_OF_SIGNALING_BY_CBL	-1.59	1.79	0	0	0	Repressed
726	GO_MITOTIC_SISTER_CHROMATID_COHESION	-1.59	1.74	0	0	0	Repressed
727	GO_REGULATION_OF_MRNA_3_END_PROCESSING	-1.59	1.7	0	0	0	Repressed
728	GO_POSITIVE_REGULATION_OF_UBIQUITIN_PROTEIN_TRANSFERASE_ACTIVITY	-1.59	1.68	0	0	0	Repressed
729	GO_CDP_DIACYLGLYCEROL_BIOSYNTHETIC_PROCESS	-1.59	1.66	0	0	0	Repressed
730	GO_RIBONUCLEOSIDE_MONOPHOSPHATE_BIOSYNTHETIC_PROCESS	-1.59	1.64	0	0	0	Repressed
731	PID_TCR_JNK_PATHWAY	-1.59	1.6	0	0	0	Repressed
732	GO_POSITIVE_REGULATION_OF_MRNA_3_END_PROCESSING	-1.59	1.59	0	0	0	Repressed
733	GO_PEPTIDYL_SERINE_MODIFICATION	-1.58	4	0	0	0	Repressed
734	LEE_LIVER_CANCER_SURVIVAL_DN	-1.58	4	0	0	0	Repressed
735	REACTOME_CELLULAR_RESPONSES_TO_STRESS	-1.58	4	0	0	0	Repressed
736	WANG_TUMOR_INVASIVENESS_UP	-1.58	4	0	0	0	Repressed
737	REACTOME_DISORDERS_OF_TRANSMEMBRANE_TRANSPORTERS	-1.58	2.46	0	0	0	Repressed
738	THUM_SYSTOLIC_HEART_FAILURE_DN	-1.58	2.39	0	0	0	Repressed
741	ACOSTA_PROLIFERATION_INDEPENDENT_MYC_TARGETS_UP	-1.58	2.21	0	1	0	Repressed
740	GO_POSITIVE_REGULATION_OF_NUCLEOCYTOPLASMIC_TRANSPORT	-1.58	2.27	0	0	0	Repressed
739	ALFANO_MYC_TARGETS	-1.48	2.35	0	0	0	Repressed
742	KEGG_LONG_TERM_POTENTIATION	-1.58	2.09	0	0	0	Repressed
743	GO_PROTEIN_SERINE_THREONINE_PHOSPHATASE_COMPLEX	-1.58	2	0	0	0	Repressed
744	GENTILE_UV_LOW_DOSE_DN	-1.58	1.97	0	0	0	Repressed
745	GO_NEGATIVE_REGULATION_OF_RNA_METABOLIC_PROCESS	-1.58	1.97	0	0	0	Repressed
746	REACTOME_RAF_ACTIVATION	-1.58	1.91	0	0	0	Repressed
747	GO_REGULATION_OF_TRANSCRIPTION_BY_RNA_POLYMERASE_I	-1.58	1.89	0	0	0	Repressed
748	GO_POSITIVE_REGULATION_OF_MRNA_PROCESSING	-1.58	1.87	0	0	0	Repressed
749	GO_NEGATIVE_REGULATION_OF_PATHWAY_RESTRICTED_SMAD_PROTEIN_PHOSP	-1.58	1.85	0	0	0	Repressed
750	GO_NEGATIVE_REGULATION_OF_CALCIUM_MEDIATED_SIGNALING	-1.58	1.7	0	0	0	Repressed
751	GO_SPLICEOSOMAL_TRI_SNRNP_COMPLEX	-1.58	1.7	0	0	0	Repressed

996	GO_CELL_CYCLE_PHASE_TRANSITION	-1.50	4	0	0	1	Repressed
753	GO_RIBOSOME_BINDING	-1.58	1.69	0	0	0	Repressed
754	GO_EMBRYONIC_CLEAVAGE	-1.58	1.63	0	0	0	Repressed
755	GO_POSITIVE_REGULATION_OF_TELOMERASE_ACTIVITY	-1.58	1.55	0	0	0	Repressed
756	GO POSITIVE REGULATION OF TRANSCRIPTION OF NOTCH RECEPTOR TARGET	-1.58	1.53	0	0	0	Repressed
757	GO_SYNAPSE_MATURATION	-1.58	1.53	0	0	0	Repressed
758	BIOCARTA_LIS1_PATHWAY	-1.58	1.47	0	0	0	Repressed
759	GO_ALPHA_ACTININ_BINDING	-1.58	1.47	0	0	0	Repressed
760	GO_CELLULAR_RESPONSE_TO_ARSENIC_CONTAINING_SUBSTANCE	-1.58	1.45	0	0	0	Repressed
761	CSR_LATE_UP.V1_UP	-1.57	4	0	0	0	Repressed
762	GO_PURINE_NTP_DEPENDENT_HELICASE_ACTIVITY	-1.57	4	0	0	0	Repressed
763	GO_REGULATION_OF_CHROMOSOME_ORGANIZATION	-1.57	4	0	0	0	Repressed
764	GO ANTIGEN PROCESSING AND PRESENTATION OF PEPTIDE OR POLYSACCHA	-1.57	2.24	0	0	0	Repressed
765	GO_RNA_DEPENDENT_DNA_BIOSYNTHETIC_PROCESS	-1.57	2.24	0	0	0	Repressed
766	GO_REGULATION_OF_MICROTUBULE_MOTOR_ACTIVITY	-1.57	2.08	0	0	0	Repressed
767	GO_METAPHASE_PLATE_CONGRESSION	-1.57	2.05	0	0	0	Repressed
768	BIOCARTA_PPARG_PATHWAY	-1.57	1.9	0	0	0	Repressed
769	REACTOME_ACTIVATION_OF_RAC1_DOWNSTREAM_OF_NMDARS	-1.57	1.89	0	0	0	Repressed
770	JIANG_AGING_CEREBRAL_CORTEX_DN	-1.57	1.81	0	0	0	Repressed
771	GO_NONHOMOLOGOUS_END_JOINING_COMPLEX	-1.57	1.75	0	0	0	Repressed
772	WANG_TARGETS_OF_MLL_CBP_FUSION_DN	-1.57	1.75	0	0	0	Repressed
773	REACTOME_POLO_LIKE_KINASE_MEDIATED_EVENTS	-1.57	1.68	0	0	0	Repressed
774	GO_RNA_CAP_BINDING_COMPLEX	-1.57	1.62	0	0	0	Repressed
775	GO_DNA_HELICASE_COMPLEX	-1.57	1.61	0	0	0	Repressed
776	REACTOME_RECYCLING_PATHWAY_OF_L1	-1.57	1.58	0	0	0	Repressed
777	GO_RESPONSE_TO_MORPHINE	-1.57	1.51	0	0	0	Repressed
778	GO_TRNA_5_END_PROCESSING	-1.57	1.45	0	0	0	Repressed
779	GO_IMP_BIOSYNTHETIC_PROCESS	-1.57	1.44	0	0	0	Repressed
780	MANN_RESPONSE_TO_AMIFOSTINE_DN	-1.57	1.35	0	0	0	Repressed
714	GO_REGULATION_OF_CELL_CYCLE_PHASE_TRANSITION	-1.49	4	0	0	0	not significant
782	DUTERTRE_ESTRADIOL_RESPONSE_6HR_UP	-1.56	4	0	0	0	Repressed
783	GO_CADHERIN_BINDING	-1.56	4	0	0	0	Repressed
715	GO_REGULATION_OF_CELL_CYCLE_PROCESS	-1.49	4	0	0	0	not significant
785	GO_ORGANELLE_FISSION	-1.56	4	0	0	0	Repressed
786	GO_STEM_CELL_DIFFERENTIATION	-1.56	4	0	0	0	Repressed
787	MARKEY_RB1_ACUTE_LOF_UP	-1.56	4	0	0	0	Repressed
788	MOREAUX_MULTIPLE_MYELOMA_BY_TACI_DN	-1.56	4	0	0	0	Repressed
789	REACTOME_CELL_CYCLE	-1.56	4	0	0	0	Repressed
790	ZHANG_BREAST_CANCER_PROGENITORS_UP	-1.56	4	0	0	0	Repressed
791	GO_PEPTIDYL_THREONINE_MODIFICATION	-1.56	2.5	0	0	0	Repressed
792	GO_TRANSLATION_REGULATOR_ACTIVITY_NUCLEIC_ACID_BINDING	-1.56	2.48	0	0	0	Repressed
793	GO_TRANSCRIPTION_ELONGATION_FROM_RNA_POLYMERASE_II_PROMOTER	-1.56	2.46	0	0	0	Repressed
794	MCBRYAN_PUBERTAL_BREAST_6_7WK_DN	-1.56	2.06	0	0	0	Repressed
795	MORI_IMMATURE_B_LYMPHOCYTE_DN	-1.56	2.01	0	0	0	Repressed
796	NADERI_BREAST_CANCER_PROGNOSIS_UP	-1.56	1.99	0	0	0	Repressed
797	GO_AXONAL_TRANSPORT	-1.56	1.87	0	0	0	Repressed
798	GO_MAINTENANCE_OF_CENTROSOME_LOCATION	-1.56	1.84	0	0	0	Repressed
799	GO_REGULATION_OF_SPINDLE_ORGANIZATION	-1.56	1.81	0	0	0	Repressed
800	GO_POSITIVE_REGULATION_OF_INTERFERON_BETA_PRODUCTION	-1.56	1.75	0	0	0	Repressed
801	GO_PHOSPHOTYROSINE_RESIDUE_BINDING	-1.56	1.74	0	0	0	Repressed
802	GO_PROTEIN_LOCALIZATION_TO_NUCLEAR_ENVELOPE	-1.56	1.72	0	0	0	Repressed
803	REACTOME_ROLE_OF_ABL_IN_ROBO_SLIT_SIGNALING	-1.56	1.72	0	0	0	Repressed
804	GO_AP_2_ADAPTOR_COMPLEX_BINDING	-1.56	1.71	0	0	0	Repressed
805	GO_EXTRINSIC_COMPONENT_OF_SYNAPTIC_MEMBRANE	-1.56	1.67	0	0	0	Repressed
806	GO NEGATIVE REGULATION OF PRI MIRNA TRANSCRIPTION BY RNA POLYMER	-1.56	1.66	0	0	0	Repressed
807	GO_POSITIVE_REGULATION_OF_CHEMOKINE_C_X_C_MOTIF_LIGAND_2_PRODUCTI	-1.56	1.66	0	0	0	Repressed
808	SUH_COEXPRESSED_WITH_ID1_AND_ID2_UP	-1.56	1.62	0	0	0	Repressed
809	GO_CYTOPLASMIC_DYNEIN_COMPLEX	-1.56	1.6	0	0	0	Repressed
810	REACTOME_RECYCLING_OF_EIF2:GDP	-1.56	1.58	0	0	0	Repressed
811	GO_PSEUDOURIDINE_SYNTHESIS	-1.56	1.54	0	0	0	Repressed
812	CAFFAREL_RESPONSE_TO_THC_24HR_3_DN	-1.56	1.39	0	0	0	Repressed
813	CHR2Q36	-1.56	1.38	0	0	0	Repressed
814	GO_SERTOLI_CELL_DIFFERENTIATION	-1.56	1.27	0	0	0	Repressed
993	COLLER_MYC_TARGETS_UP	-1.41	1.14	0	0	0	Repressed
816	GO_MICROTUBULE_CYTOSKELETON_ORGANIZATION	-1.55	4	0	0	0	Repressed
781	GO_REGULATION_OF_MEIOTIC_CELL_CYCLE	-1.37	1.05	0	0	0	not significant
784	GO_CELL_CYCLE_PROCESS	-1.36	4	0	0	0	not significant
819	GO_REGULATION_OF_NUCLEAR_DIVISION	-1.55	4	0	0	0	Repressed
820	OUELLET_OVARIAN_CANCER_INVASIVE_VS_LMP_UP	-1.55	4	0	0	0	Repressed
821	REACTOME_MITOCHONDRIAL_TRANSLATION	-1.55	4	0	0	0	Repressed
822	REACTOME_TRANSCRIPTIONAL_REGULATION_BY_RUNX1	-1.55	4	0	0	0	Repressed
823	RICKMAN_TUMOR_DIFFERENTIATED_WELL_VS_POORLY_DN	-1.55	4	0	0	0	Repressed
824	REACTOME_SWITCHING_OF_ORIGINS_TO_A_POST_REPLICATIVE_STATE	-1.55	2.21	0	0	0	Repressed
825	VANTVEER_BREAST_CANCER_METASTASIS_DN	-1.55	2.19	0	0	0	Repressed
826	GO_NUCLEAR_RECEPTOR_TRANSCRIPTION_COACTIVATOR_ACTIVITY	-1.55	1.86	0	0	0	Repressed
827	GO_REGULATION_OF_DNA_DUPLEX_UNWINDING	-1.55	1.8	0	0	0	Repressed
828	GO_MEIOTIC_CELL_CYCLE_PROCESS	-1.35	1.76	0	0	0	not significant
829	GO_MITOTIC_SPINDLE_MIDZONE_ASSEMBLY	-1.55	1.63	0	0	0	Repressed
830	GARGALOVIC_RESPONSE_TO_OXIDIZED_PHOSPHOLIPIDS_TURQUOISE_DN	-1.55	1.6	0	0	0	Repressed
831	GO_POSITIVE_REGULATION_OF_ANOIKIS	-1.55	1.6	0	0	0	Repressed
832	GO_IRES_DEPENDENT_VIRAL_TRANSLATIONAL_INITIATION	-1.55	1.58	0	0	0	Repressed
833	BIOCARTA_EFP_PATHWAY	-1.55	1.57	0	0	0	Repressed
834	GO_NEGATIVE_REGULATION_OF_RNA_SPLICING	-1.55	1.54	0	0	0	Repressed
835	GO_NITRIC_OXIDE_SYNTHASE_BIOSYNTHETIC_PROCESS	-1.55	1.51	0	0	0	Repressed
836	SCHLOSSER_MYC_AND_SERUM_RESPONSE_SYNERGY	-1.55	1.5	0	0	0	Repressed
837	KEGG_DORSO_VENTRAL_AXIS_FORMATION	-1.55	1.49	0	0	0	Repressed
838	GO_PSEUDOPODIUM	-1.55	1.45	0	0	0	Repressed
839	GO_RESPONSE_TO_MANGANESE_ION	-1.55	1.43	0	0	0	Repressed
840	CHEOK_RESPONSE_TO_MERCAPTOPURINE_UP	-1.55	1.4	0	0	0	Repressed
841	GO_CENTROMERIC_SISTER_CHROMATID_COHESION	-1.55	1.36	0	0	0	Repressed
842	GO_NEGATIVE_REGULATION_OF_CHROMOSOME_ORGANIZATION	-1.54	4	0	0	0	Repressed
843	GO_REGULATION_OF_CELL_CYCLE	-1.34	4	0	0	0	not significant
844	REACTOME_INTERLEUKIN_1_SIGNALING	-1.54	4	0	0	0	Repressed
845	REACTOME NEUROTRANSMITTER RECEPTORS AND POSTSYNAPTIC SIGNAL TR	-1.54	4	0	0	0	Repressed
846	KEGG_PURINE_METABOLISM	-1.50	2.45	1	0	0	Repressed
847	GO_BASAL_TRANSCRIPTION_MACHINERY_BINDING	-1.54	2.24	0	0	0	Repressed
848	GO_SNORNA_LOCALIZATION	-1.54	2.2	0	0	0	Repressed
849	GO_REGULATION_OF_TELOMERE_MAINTENANCE	-1.54	1.94	0	0	0	Repressed
850	GO_TRNA_BINDING	-1.54	1.94	0	0	0	Repressed
851	GO_TERMINATION_OF_RNA_POLYMERASE_II_TRANSCRIPTION	-1.54	1.88	0	0	0	Repressed
852	GO_SNRNA_MODIFICATION	-1.54	1.85	0	0	0	Repressed
853	GO_ENDORIBONUCLEASE_ACTIVITY_PRODUCING_5_PHOSPHOMONOESTERS	-1.54	1.83	0	0	0	Repressed
854	REACTOME_MOLYBDENUM_COFACTOR_BIOSYNTHESIS	-1.54	1.83	0	0	0	Repressed
855	GO_ADENYLATE_CYCLASE_ACTIVATOR_ACTIVITY	-1.54	1.82	0	0	0	Repressed
856	PID_RAS_PATHWAY	-1.54	1.77	0	0	0	Repressed
857	REACTOME_RNA_POLYMERASE_III_TRANSCRIPTION	-1.54	1.77	0	0	0	Repressed
858	GO REGULATION OF MITOCHONDRIAL ATP SYNTHESIS COUPLED ELECTRON T	-1.54	1.71	0	0	0	Repressed
859	GO_CORNIFIED_ENVELOPE	-1.54	1.66	0	0	0	Repressed
860	GO_TERMINATION_OF_RNA_POLYMERASE_I_TRANSCRIPTION	-1.54	1.62	0	0	0	Repressed

861	CHR9Q33	-1.54	1.61	0	0	0	Repressed
862	REACTOME_GLUTAMATE_AND_GLUTAMINE_METABOLISM	-1.54	1.61	0	0	0	Repressed
863	REACTOME_TRIGLYCERIDE_CATABOLISM	-1.54	1.6	0	0	0	Repressed
864	GO_RNA_POLYMERASE_III_ACTIVITY	-1.54	1.59	0	0	0	Repressed
865	GO_PATHWAY_RESTRICTED_SMAD_PROTEIN_PHOSPHORYLATION	-1.54	1.58	0	0	0	Repressed
866	GO_NUCLEOSIDE_DIPHOSPHATE_KINASE_ACTIVITY	-1.54	1.53	0	0	0	Repressed
867	GO_RNA_STEM_LOOP_BINDING	-1.54	1.52	0	0	0	Repressed
868	GO_CELL_CORTEX_REGION	-1.54	1.49	0	0	0	Repressed
869	GO_REGULATION_OF_OOCYTE_MATURATION	-1.54	1.48	0	0	0	Repressed
870	GO_REGULATION_OF_SYNAPSE_MATURATION	-1.54	1.48	0	0	0	Repressed
871	GO_RIBONUCLEASE_P_COMPLEX	-1.54	1.48	0	0	0	Repressed
872	MOOTHA_GLYCOLYSIS	-1.54	1.44	0	0	0	Repressed
873	NIKOLSKY_BREAST_CANCER_10Q22_AMPLICON	-1.54	1.44	0	0	0	Repressed
874	TOMIDA_METASTASIS_UP	-1.54	1.42	0	0	0	Repressed
875	VALK_AML_CLUSTER_16	-1.54	1.39	0	0	0	Repressed
876	NAKAMURA_CANCER_MICROENVIRONMENT_UP	-1.54	1.38	0	0	0	Repressed
877	LIANG_SILENCED_BY_METHYLATION_DN	-1.54	1.37	0	0	0	Repressed
878	NIELSEN_MALIGNAT_FIBROUS_HISTIOCYTOMA_UP	-1.54	1.37	0	0	0	Repressed
879	GLINSKY_CANCER_DEATH_UP	-1.54	1.35	0	0	0	Repressed
880	GO_POSITIVE_REGULATION_OF_NUCLEOBASE_CONTAINING_COMPOUND_TRANSP(	-1.54	1.35	0	0	0	Repressed
881	GEORGES_CELL_CYCLE_MIR192_TARGETS	-1.34	1.12	0	0	0	not significant
882	GO_HEMATOPOIETIC_PROGENITOR_CELL_DIFFERENTIATION	-1.53	4	0	0	0	Repressed
883	GO_SPLICEOSOMAL_COMPLEX	-1.53	4	0	0	0	Repressed
884	REACTOME_SIGNALING_BY_RHO_GTPASES	-1.53	4	0	0	0	Repressed
885	RPS14_DN.V1_DN	-1.53	4	0	0	0	Repressed
886	DAZARD_UV_RESPONSE_CLUSTER_G6	-1.53	2.43	0	0	0	Repressed
887	GO_TRANSLATION_REGULATOR_ACTIVITY	-1.53	2.43	0	0	0	Repressed
888	GO_REGULATION_OF_DEPHOSPHORYLATION	-1.53	2.41	0	0	0	Repressed
889	SCIBETTA_KDM5B_TARGETS_DN	-1.53	2.22	0	0	0	Repressed
890	GO_TRANSLATION_FACTOR_ACTIVITY_RNA_BINDING	-1.53	2.19	0	0	0	Repressed
891	GO_PHOSPHATASE_REGULATOR_ACTIVITY	-1.53	2.05	0	0	0	Repressed
892	GO_RNA_POLYMERASE_II_HOLOENZYME	-1.53	2.03	0	0	0	Repressed
893	GO_PRECATALYTIC_SPLICEOSOME	-1.53	1.96	0	0	0	Repressed
894	REACTOME_PLATELET_HOMEOSTASIS	-1.53	1.88	0	0	0	Repressed
895	GO_REGULATION_OF_TELOMERASE_ACTIVITY	-1.53	1.87	0	0	0	Repressed
896	REACTOME_ORC1_REMOVAL_FROM_CHROMATIN	-1.53	1.84	0	0	0	Repressed
897	REACTOME_RECYCLING_OF_BILE_ACIDS_AND_SALTS	-1.53	1.77	0	0	0	Repressed
898	REACTOME_SCF_SKP2_MEDIATED_DEGRADATION_OF_P27_P21	-1.53	1.76	0	0	0	Repressed
899	GO_MIDBRAIN_DEVELOPMENT	-1.53	1.72	0	0	0	Repressed
900	MEINHOLD_OVARIAN_CANCER_LOW_GRADE_DN	-1.53	1.58	0	0	0	Repressed
901	GO_U2_TYPE_CATALYTIC_STEP_2_SPLICEOSOME	-1.53	1.54	0	0	0	Repressed
902	SARTIPY_BLUNTED_BY_INSULIN_RESISTANCE_UP	-1.53	1.52	0	0	0	Repressed
903	SCHAEFFER_PROSTATE_DEVELOPMENT_12HR_DN	-1.53	1.5	0	0	0	Repressed
904	GO_HISTONE_PHOSPHORYLATION	-1.53	1.48	0	0	0	Repressed
905	GO_MICROTUBULE_END	-1.53	1.48	0	0	0	Repressed
906	GO_PRONUCLEUS	-1.53	1.48	0	0	0	Repressed
907	JEON_SMAD6_TARGETS_DN	-1.53	1.47	0	0	0	Repressed
908	SARTIPY_NORMAL_AT_INSULIN_RESISTANCE_UP	-1.53	1.44	0	0	0	Repressed
909	GO_ANATOMICAL_STRUCTURE_ARRANGEMENT	-1.53	1.42	0	0	0	Repressed
910	GO_SPINDLE_POLE_CENTROSOME	-1.53	1.4	0	0	0	Repressed
911	GO_BILE_ACID_AND_BILE_SALT_TRANSPORT	-1.53	1.36	0	0	0	Repressed
912	GO_CLATHRIN_COAT_OF_COATED_PIT	-1.53	1.34	0	0	0	Repressed
913	GO_MRNA_MODIFICATION	-1.53	1.31	0	0	0	Repressed
914	HAMAI_APOPTOSIS_VIA_TRAIL_UP	-1.52	4	0	0	0	Repressed
915	MARSON_BOUND_BY_E2F4_UNSTIMULATED	-1.52	4	0	0	0	Repressed
916	ROME_INSULIN_TARGETS_IN_MUSCLE_UP	-1.52	4	0	0	0	Repressed
917	REACTOME_RNA_POLYMERASE_II_TRANSCRIPTION_TERMINATION	-1.52	2.53	0	0	0	Repressed
918	REACTOME_NEGATIVE_EPIGENETIC_REGULATION_OF_RRNA_EXPRESSION	-1.52	2.49	0	0	0	Repressed
919	RUIZ_TNC_TARGETS_DN	-1.52	2.48	0	0	0	Repressed
920	GRABARCZYK_BCL11B_TARGETS_UP	-1.52	2.23	0	0	0	Repressed
921	GO_MITOCHONDRIAL_TRANSLATIONAL_TERMINATION	-1.52	2.2	0	0	0	Repressed
922	GO_RNA_METHYLATION	-1.52	2.18	0	0	0	Repressed
923	REACTOME_SUMOYLATION_OF_CHROMATIN_ORGANIZATION_PROTEINS	-1.52	1.81	0	0	0	Repressed
924	GO_ZONA_PELLUCIDA_RECEPTOR_COMPLEX	-1.52	1.79	0	0	0	Repressed
925	REACTOME_CA_DEPENDENT_EVENTS	-1.52	1.69	0	0	0	Repressed
926	GO_PROTEIN_LOCALIZATION_TO_CHROMATIN	-1.52	1.67	0	0	0	Repressed
927	GO_CONDENSED_NUCLEAR_CHROMOSOME	-1.52	1.65	0	0	0	Repressed
928	AMIT_SERUM_RESPONSE_240_MCF10A	-1.52	1.6	0	0	0	Repressed
929	GO_REGULATION_OF_CHEMOKINE_C_X_C_MOTIF_LIGAND_2_PRODUCTION	-1.52	1.6	0	0	0	Repressed
930	GO_PTW_PP1_PHOSPHATASE_COMPLEX	-1.52	1.55	0	0	0	Repressed
931	GO_NCRNA_3_END_PROCESSING	-1.52	1.54	0	0	0	Repressed
932	GO_POSITIVE_REGULATION_OF_OXIDATIVE_STRESS_INDUCED_INTRINSIC_APOPTC	-1.52	1.53	0	0	0	Repressed
933	GO_POTASSIUM_ION_BINDING	-1.52	1.51	0	0	0	Repressed
934	GO_PWP2P_CONTAINING_SUBCOMPLEX_OF_90S_PRERIBOSOME	-1.52	1.47	0	0	0	Repressed
935	REACTOME_CHK1_CHK2_CDS1_MEDIATED_INACTIVATION_OF_CYCLIN_B:CDK1_CO	-1.52	1.46	0	0	0	Repressed
936	REACTOME_RNA_POLYMERASE_III_TRANSCRIPTION_INITIATION_FROM_TYPE_1_PR	-1.52	1.45	0	0	0	Repressed
937	GO_CLEAVAGE_INVOLVED_IN_RRNA_PROCESSING	-1.52	1.44	0	0	0	Repressed
938	GO_AGGREPHAGY	-1.52	1.4	0	0	0	Repressed
939	GO_CHAPERONE_COFACTOR_DEPENDENT_PROTEIN_REFOLDING	-1.52	1.4	0	0	0	Repressed
940	BIOCARTA_PRION_PATHWAY	-1.52	1.39	0	0	0	Repressed
941	GO_ACTININ_BINDING	-1.52	1.38	0	0	0	Repressed
951	GO_CELL_CYCLE	-1.31	4	0	0	0	not significant
943	REACTOME_HUR_ELAVL1_BINDS_AND_STABILIZES_MRNA	-1.52	1.37	0	0	0	Repressed
944	GO_TRACHEA_DEVELOPMENT	-1.52	1.32	0	0	0	Repressed
945	REACTOME_PROLONGED_ERK_ACTIVATION_EVENTS	-1.52	1.32	0	0	0	Repressed
946	WANG_ADIPOGENIC_GENES_REPRESSED_BY_SIRT1	-1.52	1.29	0	0	0	Repressed
947	REACTOME_RNA_POLYMERASE_III_CHAIN_ELONGATION	-1.52	1.27	0	0	0	Repressed
948	GO_RETROGRADE_AXONAL_TRANSPORT	-1.52	1.25	0	0	0	Repressed
815	DANG_REGULATED_BY_MYC_UP	-1.36	1.24	0	0	0	Repressed
950	DIAZ_CHRONIC_MEYLOGENOUS_LEUKEMIA_UP	-1.51	4	0	0	0	Repressed
752	GO_POSITIVE_REGULATION_OF_CELL_CYCLE	-1.28	1.69	0	0	0	not significant
952	GO_MITOCHONDRIAL_PROTEIN_COMPLEX	-1.51	4	0	0	0	Repressed
953	GO_ORGANELLAR_RIBOSOME	-1.51	4	0	0	0	Repressed
954	GO_PROTEASOMAL_PROTEIN_CATABOLIC_PROCESS	-1.51	4	0	0	0	Repressed
955	ZHANG_TLX_TARGETS_60HR_DN	-1.51	4	0	0	0	Repressed
956	GROSS_HYPOXIA_VIA_ELK3_AND_HIF1A_DN	-1.51	2.5	0	0	0	Repressed
957	GO_ATP_DEPENDENT_CHROMATIN_REMODELING	-1.51	2.23	0	0	0	Repressed
958	GO_REGULATION_OF_CIRCADIAN_RHYTHM	-1.51	2.22	0	0	0	Repressed
959	BROWNE_HCMV_INFECTION_30MIN_DN	-1.51	2.16	0	0	0	Repressed
960	PID_P73PATHWAY	-1.51	2.03	0	0	0	Repressed
961	GO_MOTOR_ACTIVITY	-1.51	2.02	0	0	0	Repressed
962	BURTON_ADIPOGENESIS_2	-1.51	1.92	0	0	0	Repressed
963	PID_CDC42_PATHWAY	-1.51	1.92	0	0	0	Repressed
964	GO_NEGATIVE_REGULATION_OF_METAPHASE_ANAPHASE_TRANSITION_OF_CELL_(	-1.51	1.85	0	0	0	Repressed
965	GO_POSITIVE_REGULATION_OF_MICROTUBULE_BINDING	-1.51	1.74	0	0	0	Repressed
966	GO_ENDOPEPTIDASE_ACTIVATOR_ACTIVITY	-1.51	1.73	0	0	0	Repressed
967	GO_REGULATION_OF_TELOMERE_MAINTENANCE_VIA_TELOMERE_LENGTHENING	-1.51	1.73	0	0	0	Repressed
968	ISHIDA_E2F_TARGETS	-1.51	1.67	0	0	0	Repressed
969	GO_RRNA_BINDING	-1.51	1.65	0	0	0	Repressed

970	REACTOME_ACTIVATION_OF_NMDA_RECEPTORS_AND_POSTSYNAPTIC_EVENTS	-1.51	1.63	0	0	0	Repressed
971	MCCLUNG_COCAINE_REWARD_5D	-1.51	1.6	0	0	0	Repressed
972	GO_SNRNA_BINDING	-1.51	1.59	0	0	0	Repressed
973	GO_MEIOTIC_SPINDLE	-1.51	1.57	0	0	0	Repressed
974	REACTOME_RNA_POLYMERASE_III_TRANSCRIPTION_INITIATION_FROM_TYPE_3_PR	-1.51	1.57	0	0	0	Repressed
975	REACTOME_FORMATION_OF_HIV_ELONGATION_COMPLEX_IN_THE_ABSENCE_OF_I	-1.51	1.55	0	0	0	Repressed
976	GO_ATP_DEPENDENT_5_3_DNA_HELICASE_ACTIVITY	-1.51	1.52	0	0	0	Repressed
977	GO_IMPORTIN_ALPHA_FAMILY_PROTEIN_BINDING	-1.51	1.49	0	0	0	Repressed
978	GO_ISOPRENOID_BINDING	-1.51	1.48	0	0	0	Repressed
979	GO_DE_NOVO_IMP_BIOSYNTHETIC_PROCESS	-1.51	1.47	0	0	0	Repressed
980	GO_MODULATION_BY_SYMBIONT_OF_HOST_CELLULAR_PROCESS	-1.51	1.47	0	0	0	Repressed
981	GO_REGULATION_OF_TRANSCRIPTION_BY_RNA_POLYMERASE_III	-1.51	1.42	0	0	0	Repressed
982	REACTOME_PROCESSING_OF_INTRONLESS_PRE_MRNAS	-1.51	1.41	0	0	0	Repressed
983	KEGG_CYSTEINE_AND_METHIONINE_METABOLISM	-1.48	1.39	1	0	0	Repressed
984	GO_CRD_MEDIATED_MRNA_STABILITY_COMPLEX	-1.51	1.38	0	0	0	Repressed
985	WIEMANN_TELOMERE_SHORTENING_AND_CHRONIC_LIVER_DAMAGE_UP	-1.51	1.33	0	0	0	Repressed
986	GO_REGULATION_OF_MICROTUBULE_BINDING	-1.51	1.32	0	0	0	Repressed
987	LI_WILMS_TUMOR_ANAPLASTIC_UP	-1.51	1.31	0	0	0	Repressed
988	BIOCARTA_CARM1_PATHWAY	-1.51	1.3	0	0	0	Repressed
989	HOUSTIS_ROS	-1.51	1.3	0	0	0	Repressed
990	CAFFAREL_RESPONSE_TO_THC_8HR_3_DN	-1.51	1.29	0	0	0	Repressed
991	GO_KERATINOCYTE_MIGRATION	-1.51	1.29	0	0	0	Repressed
992	GO_VIRAL_TRANSLATION	-1.51	1.25	0	0	0	Repressed
949	BENPORATH_MYC_MAX_TARGETS	-1.31	4	0	0	0	Repressed
994	GO_TRNA_CYTOSINE_METHYLTRANSFERASE_ACTIVITY	-1.41	1.06	1	0	0	Repressed
995	GO_ANTIGEN_PROCESSING_AND_PRESENTATION_OF_PEPTIDE_ANTIGEN	-1.50	4	0	0	0	Repressed
942	GO_POSITIVE_REGULATION_OF_CELL_CYCLE_PROCESS	-1.22	1.38	0	0	0	not significant
997	GO_CELLULAR_RESPONSE_TO_OXYGEN_LEVELS	-1.50	4	0	0	0	Repressed
998	GO_MICROTUBULE_BINDING	-1.50	4	0	0	0	Repressed
999	GO_REGULATION_OF_NUCLEOBASE_CONTAINING_COMPOUND_METABOLIC_PROCI	-1.50	4	0	0	0	Repressed
1000	GO_TUBULIN_BINDING	-1.50	4	0	0	0	Repressed
1001	REACTOME_INTRA_GOLGI_AND_RETROGRADE_GOLGI_TO_ER_TRAFFIC	-1.50	2.36	0	0	0	Repressed
1002	GO_ORGANELLAR_LARGE_RIBOSOMAL_SUBUNIT	-1.50	1.94	0	0	0	Repressed
1003	SANA_RESPONSE_TO_IFNG_DN	-1.50	1.9	0	0	0	Repressed
1004	GO_HIPPOCAMPUS_DEVELOPMENT	-1.50	1.77	0	0	0	Repressed
1005	GO_U2_TYPE_SPLICEOSOMAL_COMPLEX	-1.50	1.7	0	0	0	Repressed
1006	GO_POSITIVE_REGULATION_OF_DEPHOSPHORYLATION	-1.50	1.69	0	0	0	Repressed
1007	GO_REGULATION_OF_OOGENESIS	-1.50	1.65	0	0	0	Repressed
1008	GO_CAJAL_BODY	-1.50	1.63	0	0	0	Repressed
1009	GO_NUCLEAR_UBIQUITIN_LIGASE_COMPLEX	-1.50	1.62	0	0	0	Repressed
1010	GO_SCARNA_LOCALIZATION_TO_CAJAL_BODY	-1.50	1.61	0	0	0	Repressed
1011	GO_VESICLE_CYTOSKELETAL_TRAFFICKING	-1.50	1.61	0	0	0	Repressed
1012	REACTOME_PRE_NOTCH_PROCESSING_IN_THE_ENDOPLASMIC_RETICULUM	-1.50	1.59	0	0	0	Repressed
1013	GO_MITOTIC_METAPHASE_PLATE_CONGRESSION	-1.50	1.57	0	0	0	Repressed
1014	CHIN_BREAST_CANCER_COPY_NUMBER_UP	-1.50	1.53	0	0	0	Repressed
1015	REACTOME_TELOMERE_EXTENSION_BY_TELOMERASE	-1.50	1.52	0	0	0	Repressed
1016	GO_CELL_CELL_RECOGNITION	-1.50	1.51	0	0	0	Repressed
1017	BIOCARTA_G2_PATHWAY	-1.50	1.47	0	0	0	Repressed
1018	GO_REGULATION_OF_PHOSPHATIDYLINOSITOL_DEPHOSPHORYLATION	-1.50	1.44	0	0	0	Repressed
1019	REACTOME_INTERACTIONS_OF_VPR_WITH_HOST_CELLULAR_PROTEINS	-1.50	1.44	0	0	0	Repressed
1020	BIOCARTA_EIF_PATHWAY	-1.50	1.42	0	0	0	Repressed
1021	GO_MODIFICATION_BY_SYMBIONT_OF_HOST_MORPHOLOGY_OR_PHYSIOLOGY	-1.50	1.42	0	0	0	Repressed
1022	GO_PEBOW_COMPLEX	-1.50	1.37	0	0	0	Repressed
1023	GO_NITRIC_OXIDE_SYNTHASE_REGULATOR_ACTIVITY	-1.50	1.34	0	0	0	Repressed
1024	GO_NEURAL_CREST_FORMATION	-1.50	1.3	0	0	0	Repressed
1025	GO_C2H2_ZINC_FINGER_DOMAIN_BINDING	-1.50	1.29	0	0	0	Repressed
1026	GO_METHYLOSOME	-1.50	1.29	0	0	0	Repressed
1027	NAM_FXYD5_TARGETS_DN	-1.50	1.26	0	0	0	Repressed
1028	MA_PITUITARY_FETAL_VS_ADULT_DN	-1.50	1.25	0	0	0	Repressed
1029	GO_TRNA_CYTOSINE_5__METHYLTRANSFERASE_ACTIVITY	-1.39	1.06	1	0	0	Repressed
1030	KEGG_VALINE_LEUCINE_AND_ISOLEUCINE_BIOSYNTHESIS	-1.40	1.02	1	0	0	Repressed
1031	GO_MICROTUBULE_BASED_PROCESS	-1.49	4	0	0	0	not significant
1032	GO_POSITIVE_REGULATION_OF_PROTEIN_MODIFICATION_BY_SMALL_PROTEIN_CC	-1.49	4	0	0	0	not significant
1033	GO_REGULATION_OF_MICROTUBULE_BASED_PROCESS	-1.49	4	0	0	0	not significant
1034	GO_REGULATION_OF_PROTEASOMAL_PROTEIN_CATABOLIC_PROCESS	-1.49	4	0	0	0	not significant
1035	REACTOME_CELLULAR_RESPONSES_TO_EXTERNAL_STIMULI	-1.49	4	0	0	0	not significant
1036	SCHLOSSER_SERUM_RESPONSE_AUGMENTED_BY_MYC	-1.49	2.48	0	0	0	not significant
1037	GO_MRNA_3_END_PROCESSING	-1.49	2.18	0	0	0	not significant
1038	REACTOME_REGULATION_OF_PLK1_ACTIVITY_AT_G2_M_TRANSITION	-1.49	1.88	0	0	0	not significant
1039	REACTOME_PROTEIN_FOLDING	-1.49	1.82	0	0	0	not significant
1040	KAMMINGA_EZH2_TARGETS	-1.49	1.74	0	0	0	not significant
1041	REACTOME_OPIOID_SIGNALLING	-1.49	1.69	0	0	0	not significant
1042	GO_PROTEIN_SERINE_THREONINE_TYROSINE_KINASE_ACTIVITY	-1.49	1.66	0	0	0	not significant
1043	GO_RESPONSE_TO_ARSENIC_CONTAINING_SUBSTANCE	-1.49	1.56	0	0	0	not significant
1044	BIOCARTA_SARS_PATHWAY	-1.49	1.54	0	0	0	not significant
1045	GO_TRANSCRIPTION_INITIATION_FROM_RNA_POLYMERASE_I_PROMOTER	-1.49	1.51	0	0	0	not significant
1046	GO_PROTEIN_KINASE_A_BINDING	-1.49	1.5	0	0	0	not significant
1047	GO_OXIDATIVE_RNA_DEMETHYLATION	-1.49	1.44	0	0	0	not significant
1048	GO_EUKARYOTIC_TRANSLATION_INITIATION_FACTOR_2B_COMPLEX	-1.49	1.42	0	0	0	not significant
1049	GO_REGULATION_OF_TRANSLATION_AT_SYNAPSE_MODULATING_SYNAPTIC_TRAN	-1.49	1.4	0	0	0	not significant
1050	BURTON_ADIPOGENESIS_12	-1.49	1.38	0	0	0	not significant
1051	GO_VENTRICULAR_CARDIAC_MUSCLE_TISSUE_DEVELOPMENT	-1.49	1.35	0	0	0	not significant
1052	GO_OUTER_MITOCHONDRIAL_MEMBRANE_ORGANIZATION	-1.49	1.33	0	0	0	not significant
1053	REACTOME_BETA_CATENIN_PHOSPHORYLATION_CASCADE	-1.49	1.33	0	0	0	not significant
1054	TURASHVILI_BREAST_DUCTAL_CARCINOMA_VS_DUCTAL_NORMAL_UP	-1.49	1.33	0	0	0	not significant
1055	REACTOME_CRISTAE_FORMATION	-1.49	1.32	0	0	0	not significant
1056	GO_REGULATION_OF_SISTER_CHROMATID_COHESION	-1.49	1.3	0	0	0	not significant
1057	GO_FEMALE_MEIOSIS_I	-1.49	1.28	0	0	0	not significant
1058	YIH_RESPONSE_TO_ARSENITE_C4	-1.49	1.27	0	0	0	not significant
1059	GO_HISTONE_KINASE_ACTIVITY	-1.49	1.25	0	0	0	not significant
1060	GO_OXIDATIVE_RNA_DEMETHYLASE_ACTIVITY	-1.49	1.25	0	0	0	not significant
1061	GO_NEGATIVE_REGULATION_OF_RELEASE_OF_CYTOCHROME_C_FROM_MITOCHO	-1.49	1.24	0	0	0	not significant
1062	GO_POSITIVE_REGULATION_OF_OXIDATIVE_PHOSPHORYLATION	-1.49	1.24	0	0	0	not significant
1063	GO_TRABECULA_FORMATION	-1.49	1.22	0	0	0	not significant
1064	NAGY_STAGA_COMPONENTS_HUMAN	-1.49	1.22	0	0	0	not significant
1065	REACTOME_BINDING_AND_UPTAKE_OF_LIGANDS_BY_SCAVENGER_RECEPTORS	-1.49	1.22	0	0	0	not significant
1066	REACTOME_FRS_MEDIATED_FGFR1_SIGNALING	-1.49	1.21	0	0	0	not significant
1067	REACTOME_NUCLEOBASE_BIOSYNTHESIS	-1.49	1.17	0	0	0	not significant
1068	GO_MRNA_CLEAVAGE_FACTOR_COMPLEX	-1.49	1.15	0	0	0	not significant
1069	GENTILE_UV_HIGH_DOSE_DN	-1.48	4	0	0	0	not significant
1070	GO_CELLULAR_PROTEIN_COMPLEX_DISASSEMBLY	-1.48	4	0	0	0	not significant
1071	GO_CELLULAR_PROTEIN_CONTAINING_COMPLEX_ASSEMBLY	-1.48	4	0	0	0	not significant
1072	GO_REGULATION_OF_MITOTIC_CELL_CYCLE	-1.48	4	0	0	0	not significant
1073	KEGG_CELL_CYCLE	-1.48	2.47	0	0	0	not significant
1074	BROWNE_HCMV_INFECTION_14HR_UP	-1.48	2.42	0	0	0	not significant
1075	GO_CYTOKINESIS	-1.48	2.12	0	0	0	not significant
1076	SASAKI_ADULT_T_CELL_LEUKEMIA	-1.48	2.12	0	0	0	not significant
1077	DACOSTA_UV_RESPONSE_VIA_ERCC3_XPCS_DN	-1.48	2.04	0	0	0	not significant
1078	GO_REGULATION_OF_PROTEASOMAL_UBIQUITIN_DEPENDENT_PROTEIN_CATABOL	-1.48	1.97	0	0	0	not significant

1079	REACTOME_POSITIVE_EPIGENETIC_REGULATION_OF_RRNA_EXPRESSION	-1.48	1.89	0	0	0	not significant
1080	REACTOME_G1_S_DNA_DAMAGE_CHECKPOINTS	-1.48	1.86	0	0	0	not significant
1081	YAO_TEMPORAL_RESPONSE_TO_PROGESTERONE_CLUSTER_10	-1.48	1.81	0	0	0	not significant
1082	GO_PERICHROMATIN_FIBRILS	-1.48	1.68	0	0	0	not significant
1083	GO_MATURE_RIBOSOME_ASSEMBLY	-1.48	1.64	0	0	0	not significant
1084	GO_TRANSCRIPTION_BY_RNA_POLYMERASE_III	-1.48	1.63	0	0	0	not significant
1085	REACTOME_MITOCHONDRIAL_TRNA_AMINOACYLATION	-1.48	1.49	0	0	0	not significant
1086	REACTOME_ION_HOMEOSTASIS	-1.48	1.48	0	0	0	not significant
1087	GO_REGULATION_OF_TRANSLATIONAL_FIDELITY	-1.48	1.46	0	0	0	not significant
1088	GO_REGULATORY_RNA_BINDING	-1.48	1.43	0	0	0	not significant
1089	GO_PURINE_NUCLEOSIDE_MONOPHOSPHATE_BIOSYNTHETIC_PROCESS	-1.48	1.41	0	0	0	not significant
1090	GO_ESTABLISHMENT_OF_PROTEIN_LOCALIZATION_TO_CHROMOSOME	-1.48	1.39	0	0	0	not significant
1091	GO_MLL1_2_COMPLEX	-1.48	1.38	0	0	0	not significant
1092	GO_MITOTIC_SPINDLE_POLE	-1.48	1.37	0	0	0	not significant
1093	GO_TELOMERASE_RNA_BINDING	-1.48	1.36	0	0	0	not significant
1094	MIKKELSEN_PLURIPOTENT_STATE_UP	-1.48	1.36	0	0	0	not significant
1095	GO_ESTABLISHMENT_OF_PROTEIN_LOCALIZATION_TO_TELOMERE	-1.48	1.33	0	0	0	not significant
1096	GO_PORIN_ACTIVITY	-1.48	1.32	0	0	0	not significant
1097	GO_PROTEIN_DESUMOYLATION	-1.48	1.3	0	0	0	not significant
1098	REACTOME_RHO_GTPASES_ACTIVATE_PAKS	-1.48	1.28	0	0	0	not significant
1099	GO_TRANSCRIPTION_ELONGATION_FROM_RNA_POLYMERASE_I_PROMOTER	-1.48	1.27	0	0	0	not significant
1100	GO_NEGATIVE_REGULATION_OF_SIGNAL_TRANSDUCTION_BY_P53_CLASS_MEDIAT	-1.48	1.26	0	0	0	not significant
1101	GO_PROTEIN_LOCALIZATION_TO_CELL_CORTEX	-1.48	1.26	0	0	0	not significant
1102	GO_U5_SNRNP	-1.48	1.26	0	0	0	not significant
1103	LOPEZ_MESOTHELIOMA_SURVIVAL_OVERALL_DN	-1.48	1.24	0	0	0	not significant
1104	GO_CELLULAR_RESPONSE_TO_ESTRADIOL_STIMULUS	-1.48	1.23	0	0	0	not significant
1105	GO_SNRNA_TRANSCRIPTION_BY_RNA_POLYMERASE_III	-1.48	1.2	0	0	0	not significant
1106	CAIRO_HEPATOBLASTOMA_POOR_SURVIVAL	-1.48	1.16	0	0	0	not significant
1107	GO_UBIQUITIN_PROTEIN_TRANSFERASE_ACTIVATOR_ACTIVITY	-1.48	1.16	0	0	0	not significant
1108	GO_CRISTAE_FORMATION	-1.48	1.14	0	0	0	not significant
1109	REACTOME_SUMOYLATION	-1.47	2.35	0	0	0	not significant
1110	GO_SINGLE_STRANDED_RNA_BINDING	-1.47	2.21	0	0	0	not significant
1111	GO_RETROGRADE_VESICLE_MEDIATED_TRANSPORT_GOLGI_TO_ENDOPLASMIC_F	-1.47	2.19	0	0	0	not significant
1112	REACTOME_PROGRAMMED_CELL_DEATH	-1.47	2.11	0	0	0	not significant
1113	NIKOLSKY_BREAST_CANCER_17Q11_Q21_AMPLICON	-1.47	1.73	0	0	0	not significant
1114	GO_SM_LIKE_PROTEIN_FAMILY_COMPLEX	-1.47	1.6	0	0	0	not significant
1115	GO_REGULATION_OF_UBIQUITIN_PROTEIN_TRANSFERASE_ACTIVITY	-1.47	1.53	0	0	0	not significant
1116	GO_PROTEIN_TRANSMEMBRANE_IMPORT_INTO_INTRACELLULAR_ORGANELLE	-1.47	1.51	0	0	0	not significant
1117	CHR15Q22	-1.47	1.49	0	0	0	not significant
1118	LEE_AGING_NEOCORTEX_DN	-1.47	1.44	0	0	0	not significant
1119	REACTOME_PROCESSING_OF_CAPPED_INTRONLESS_PRE_MRNA	-1.47	1.44	0	0	0	not significant
1120	GO_RESPONSE_TO_ETHER	-1.47	1.42	0	0	0	not significant
1121	FARDIN_HYPOXIA_11	-1.47	1.41	0	0	0	not significant
1122	GO_POSITIVE_REGULATION_OF_CELL_MATURATION	-1.47	1.38	0	0	0	not significant
1123	GO_ATP_METABOLIC_PROCESS	-1.47	1.37	0	0	0	not significant
1124	GO_OXIDATIVE_DEMETHYLATION	-1.47	1.36	0	0	0	not significant
1125	REACTOME_SCAVENGING_BY_CLASS_F_RECEPTORS	-1.47	1.35	0	0	0	not significant
1126	CHR3Q22	-1.47	1.34	0	0	0	not significant
1127	GO_ESTABLISHMENT_OF_SPINDLE_ORIENTATION	-1.47	1.34	0	0	0	not significant
1128	NAGY_PCAF_COMPONENTS_HUMAN	-1.47	1.33	0	0	0	not significant
1129	GO_PROTEIN_DNA_COMPLEX_DISASSEMBLY	-1.47	1.32	0	0	0	not significant
1130	GO_WD40_REPEAT_DOMAIN_BINDING	-1.47	1.32	0	0	0	not significant
1131	GO_EPITHELIAL_CELL_DIFFERENTIATION_INVOLVED_IN_PROSTATE_GLAND_DEVEL	-1.47	1.3	0	0	0	not significant
1132	GO_HISTONE_SERINE_KINASE_ACTIVITY	-1.47	1.29	0	0	0	not significant
1133	GO_POSITIVE_REGULATION_OF_TRANSCRIPTION_ELONGATION_FROM_RNA_POLY	-1.47	1.29	0	0	0	not significant
1134	REACTOME_SYNTHESIS_OF_BILE_ACIDS_AND_BILE_SALTS_VIA_7ALPHA_HYDROXY	-1.47	1.28	0	0	0	not significant
1135	GO_RNA_POLYMERASE_II_PREINITIATION_COMPLEX_ASSEMBLY	-1.47	1.27	0	0	0	not significant
1136	GO_TRNA_5_LEADER_REMOVAL	-1.47	1.27	0	0	0	not significant
1137	GO_HISTONE_ARGININE_N_METHYLTRANSFERASE_ACTIVITY	-1.47	1.26	0	0	0	not significant
1138	GO_MHC_CLASS_II_PROTEIN_COMPLEX_BINDING	-1.47	1.26	0	0	0	not significant
1139	REACTOME_LOSS_OF_MECP2_BINDING_ABILITY_TO_THE_NCOR_SMRT_COMPLEX	-1.47	1.23	0	0	0	not significant
1140	REACTOME_RETROGRADE_NEUROTROPHIN_SIGNALLING	-1.47	1.23	0	0	0	not significant
1141	GO_EYELID_DEVELOPMENT_IN_CAMERA_TYPE_EYE	-1.47	1.21	0	0	0	not significant
1142	GO_GLUTAMATE_CATABOLIC_PROCESS	-1.47	1.2	0	0	0	not significant
1143	REACTOME_APOPTOSIS_INDUCED_DNA_FRAGMENTATION	-1.47	1.2	0	0	0	not significant
1144	GO_OLFACTORY_LOBE_DEVELOPMENT	-1.47	1.18	0	0	0	not significant
1145	GO_PROTEASOME_ASSEMBLY	-1.47	1.17	0	0	0	not significant
1146	GO_REGULATION_OF_RNA_SPLICING	-1.46	2.42	0	0	0	not significant
1147	GO_NUCLEAR_MATRIX	-1.46	1.86	0	0	0	not significant
1148	GO_MITOCHONDRIAL_TRANSLATION	-1.46	1.84	0	0	0	not significant
1149	GRAHAM_NORMAL_QUIESCENT_VS_NORMAL_DIVIDING_DN	-1.46	1.78	0	0	0	not significant
1150	GO_CHAPERONE_MEDIATED_PROTEIN_FOLDING	-1.46	1.69	0	0	0	not significant
1151	KEGG_RENAL_CELL_CARCINOMA	-1.46	1.62	0	0	0	not significant
1152	REACTOME_COPI_INDEPENDENT_GOLGI_TO_ER_RETROGRADE_TRAFFIC	-1.46	1.53	0	0	0	not significant
1153	REACTOME_SYNTHESIS_OF_BILE_ACIDS_AND_BILE_SALTS_VIA_27_HYDROXYCHOL	-1.46	1.5	0	0	0	not significant
1154	REACTOME_RNA_POLYMERASE_III_TRANSCRIPTION_TERMINATION	-1.46	1.44	0	0	0	not significant
1155	GO_ENDONUCLEASE_COMPLEX	-1.46	1.41	0	0	0	not significant
1156	RAHMAN_TP53_TARGETS_PHOSPHORYLATED	-1.46	1.34	0	0	0	not significant
1157	GO_NEGATIVE_REGULATION_OF_MYELOID_LEUKOCYTE_DIFFERENTIATION	-1.46	1.33	0	0	0	not significant
1158	REACTOME_SIGNALING_BY_WNT_IN_CANCER	-1.46	1.33	0	0	0	not significant
1159	WEST_ADRENOCORTICAL_CARCINOMA_VS_ADENOMA_UP	-1.46	1.33	0	0	0	not significant
1160	GO_SMN_SM_PROTEIN_COMPLEX	-1.46	1.32	0	0	0	not significant
1161	GO_CELL_DIFFERENTIATION_INVOLVED_IN_KIDNEY_DEVELOPMENT	-1.46	1.3	0	0	0	not significant
1162	GO_SNORNA_3_END_PROCESSING	-1.46	1.3	0	0	0	not significant
1163	KEGG_THYROID_CANCER	-1.46	1.3	0	0	0	not significant
1164	REACTOME_MICRORNA_MIRNA_BIOGENESIS	-1.46	1.28	0	0	0	not significant
1165	GO_REGULATION_OF_DNA_TEMPLATED_TRANSCRIPTION_INITIATION	-1.46	1.27	0	0	0	not significant
1166	REACTOME_SIGNALING_BY_RETINOIC_ACID	-1.46	1.27	0	0	0	not significant
1167	GO_MODULATION_BY_HOST_OF_VIRAL_PROCESS	-1.46	1.26	0	0	0	not significant
1168	GO_POSITIVE_REGULATION_OF_CAMP_MEDIATED_SIGNALING	-1.46	1.26	0	0	0	not significant
1169	GO_INTRAMOLECULAR_TRANSFERASE_ACTIVITY	-1.46	1.25	0	0	0	not significant
1170	GO_POSITIVE_REGULATION_OF_NITRIC_OXIDE_SYNTHASE_BIOSYNTHETIC_PROCE	-1.46	1.25	0	0	0	not significant
1171	BIOCARTA_NDKDYNAMIN_PATHWAY	-1.46	1.24	0	0	0	not significant
1172	GO_RRNA_PSEUDOURIDINE_SYNTHESIS	-1.46	1.24	0	0	0	not significant
1173	GO_ADENYLATE_KINASE_ACTIVITY	-1.46	1.23	0	0	0	not significant
1174	GO_RAL_GTPASE_BINDING	-1.46	1.22	0	0	0	not significant
1175	GO_POSITIVE_REGULATION_OF_ASTROCYTE_DIFFERENTIATION	-1.46	1.18	0	0	0	not significant
1176	GO_DNA_TOPOLOGICAL_CHANGE	-1.46	1.16	0	0	0	not significant
1177	REACTOME_TIE2_SIGNALING	-1.46	1.16	0	0	0	not significant
1178	GO_REGULATION_OF_INCLUSION_BODY_ASSEMBLY	-1.46	1.15	0	0	0	not significant
1179	GO_POSITIVE_REGULATION_OF_TRANSCRIPTION_INITIATION_FROM_RNA_POLYMEI	-1.46	1.13	0	0	0	not significant
1180	GO_QUINONE_BIOSYNTHETIC_PROCESS	-1.46	1.13	0	0	0	not significant
1181	GO_ENDONUCLEOLYTIC_CLEAVAGE_IN_ITS1_TO_SEPARATE_SSU_RRNA_FROM_5	-1.46	1.11	0	0	0	not significant
1182	GO_TRANSCRIPTION_FACTOR_TFTC_COMPLEX	-1.46	1.06	0	0	0	not significant
1183	FEVR_CTNNB1_TARGETS_DN	-1.45	4	0	0	0	not significant
1184	GO_RNA_CATABOLIC_PROCESS	-1.45	4	0	0	0	not significant
1185	KEGG_UBIQUITIN_MEDIATED_PROTEOLYSIS	-1.45	4	0	0	0	not significant
1186	REACTOME_CLASS_I_MHC_MEDIATED_ANTIGEN_PROCESSING_PRESENTATION	-1.45	4	0	0	0	not significant
1187	REACTOME_UB_SPECIFIC_PROCESSING_PROTEASES	-1.45	4	0	0	0	not significant

1188	SANSOM_APC_TARGETS_REQUIRE_MYC	-1.45	4	0	0	0	not significant
1189	WANG_LMO4_TARGETS_DN	-1.45	4	0	0	0	not significant
1190	WELCSH_BRCA1_TARGETS_DN	-1.45	2.46	0	0	0	not significant
1191	REACTOME_ABC_FAMILY_PROTEINS_MEDIATED_TRANSPORT	-1.45	2	0	0	0	not significant
1192	GO_HELICASE_ACTIVITY	-1.45	1.84	0	0	0	not significant
1193	SESTO_RESPONSE_TO_UV_C1	-1.45	1.54	0	0	0	not significant
1194	GO_NEGATIVE_REGULATION_OF_GRANULOCYTE_DIFFERENTIATION	-1.45	1.43	0	0	0	not significant
1195	GO_KINASE_ACTIVATOR_ACTIVITY	-1.45	1.42	0	0	0	not significant
1196	GO_RESPONSE_TO_MAGNESIUM_ION	-1.45	1.4	0	0	0	not significant
1197	GO_NUCLEOSIDE_TRIPHOSPHATE_BIOSYNTHETIC_PROCESS	-1.45	1.33	0	0	0	not significant
1198	GO_ADENYLYLTRANSFERASE_ACTIVITY	-1.45	1.32	0	0	0	not significant
1199	GO_OXIDATIVE_DNA_DEMETHYLATION	-1.45	1.31	0	0	0	not significant
1200	GO_POSITIVE_REGULATION_OF_CELLULAR_RESPIRATION	-1.45	1.31	0	0	0	not significant
1201	GLI1_UP.V1_UP	-1.45	1.24	0	0	0	not significant
1202	GO_BHLH_TRANSCRIPTION_FACTOR_BINDING	-1.45	1.24	0	0	0	not significant
1203	GO_MOLYBDOPTERIN_COFACTOR_BIOSYNTHETIC_PROCESS	-1.45	1.24	0	0	0	not significant
1204	ZHAN_MULTIPLE_MYELOMA_LB_UP	-1.45	1.24	0	0	0	not significant
1205	GO_SAGA_TYPE_COMPLEX	-1.45	1.21	0	0	0	not significant
1206	MYLLYKANGAS_AMPLIFICATION_HOT_SPOT_8	-1.45	1.2	0	0	0	not significant
1207	GO_GLYCOLYTIC_PROCESS_THROUGH_FRUCTOSE_6_PHOSPHATE	-1.45	1.19	0	0	0	not significant
1208	GO_NEGATIVE_REGULATION_OF_METANEPHROS_DEVELOPMENT	-1.45	1.19	0	0	0	not significant
1209	GO_METHYL_CPG_BINDING	-1.45	1.18	0	0	0	not significant
1210	GO_SNORNA_PROCESSING	-1.45	1.18	0	0	0	not significant
1211	GO_POSITIVE_REGULATION_OF_PROTEIN_EXPORT_FROM_NUCLEUS	-1.45	1.14	0	0	0	not significant
1212	CHEN_LUNG_CANCER_SURVIVAL	-1.45	1.13	0	0	0	not significant
1213	LU_TUMOR_ENDOTHELIAL_MARKERS_UP	-1.45	1.13	0	0	0	not significant
1214	GO_REGULATION_OF_CELL_COMMUNICATION_BY_ELECTRICAL_COUPLING_INVOL	-1.45	1.12	0	0	0	not significant
1215	GO_MULTIMERIC_RIBONUCLEASE_P_COMPLEX	-1.45	1.11	0	0	0	not significant
1216	LU_TUMOR_VASCULATURE_UP	-1.45	1.09	0	0	0	not significant
1217	CHUANG_OXIDATIVE_STRESS_RESPONSE_DN	-1.45	1.08	0	0	0	not significant
1218	GO_REGULATION_OF_KERATINOCYTE_MIGRATION	-1.45	1.07	0	0	0	not significant
1219	GO_NEGATIVE_REGULATION_OF_CELL_CYCLE_PROCESS	-1.44	4	0	0	0	not significant
1220	GO_PROTEIN_MODIFICATION_BY_SMALL_PROTEIN_REMOVAL	-1.44	4	0	0	0	not significant
1221	OISHI_CHOLANGIOMA_STEM_CELL_LIKE_UP	-1.44	4	0	0	0	not significant
1222	GO_PALLIUM_DEVELOPMENT	-1.44	2.43	0	0	0	not significant
1223	GO_NON_CANONICAL_WNT_SIGNALING_PATHWAY	-1.44	2.02	0	0	0	not significant
1224	RASHI_RESPONSE_TO_IONIZING_RADIATION_5	-1.44	2	0	0	0	not significant
1225	REACTOME_TNFR2_NON_CANONICAL_NF_KB_PATHWAY	-1.44	2	0	0	0	not significant
1226	MARZEC_IL2_SIGNALING_UP	-1.44	1.89	0	0	0	not significant
1227	REACTOME_CLEC7A_DECTIN_1_SIGNALING	-1.44	1.86	0	0	0	not significant
1228	REACTOME_HDACS_DEACETYLATE_HISTONES	-1.44	1.82	0	0	0	not significant
1229	GROSS_HYPOXIA_VIA_HIF1A_UP	-1.44	1.67	0	0	0	not significant
1230	REACTOME_CYCLIN_A:CDK2_ASSOCIATED_EVENTS_AT_S_PHASE_ENTRY	-1.44	1.55	0	0	0	not significant
1231	GO_TAU_PROTEIN_BINDING	-1.44	1.44	0	0	0	not significant
1232	LEE_CALORIE_RESTRICTION_NEOCORTEX_DN	-1.44	1.39	0	0	0	not significant
1233	GO_PROTEIN_O_LINKED_GLYCOSYLATION_VIA_SERINE	-1.44	1.33	0	0	0	not significant
1234	GO_REGULATION_OF_RNA_POLYMERASE_II_TRANSCRIPTIONAL_PREINITIATION_C	-1.44	1.27	0	0	0	not significant
1235	GO_PSEUDOURIDINE_SYNTHASE_ACTIVITY	-1.44	1.23	0	0	0	not significant
1236	SUZUKI_RESPONSE_TO_TSA_AND_DECITABINE_1B	-1.44	1.22	0	0	0	not significant
1237	GO_MHC_PROTEIN_COMPLEX_BINDING	-1.44	1.2	0	0	0	not significant
1238	GO_MODULATION_BY_HOST_OF_VIRAL_GENOME_REPLICATION	-1.44	1.2	0	0	0	not significant
1239	GO_NEURON_NEURON_SYNAPTIC_TRANSMISSION	-1.44	1.2	0	0	0	not significant
1240	GO_PROTEIN_INSERTION_INTO_MITOCHONDRIAL_OUTER_MEMBRANE	-1.44	1.17	0	0	0	not significant
1241	GARGALOVIC_RESPONSE_TO_OXIDIZED_PHOSPHOLIPIDS_GREEN_DN	-1.44	1.16	0	0	0	not significant
1242	GO_MYELIN_SHEATH_ADAXONAL_REGION	-1.44	1.12	0	0	0	not significant
1243	STONER_ESOPHAGEAL_CARCINOGENESIS_DN	-1.44	1.12	0	0	0	not significant
1244	GO_VENTRICULAR_CARDIAC_MUSCLE_CELL_DEVELOPMENT	-1.44	1.11	0	0	0	not significant
1245	REACTOME_FRS_MEDIATED_FGFR2_SIGNALING	-1.44	1.11	0	0	0	not significant
1246	GO_5_3_DNA_HELICASE_ACTIVITY	-1.44	1.1	0	0	0	not significant
1247	GO_MACROMOLECULE_TRANSMEMBRANE_TRANSPORTER_ACTIVITY	-1.44	1.1	0	0	0	not significant
1248	GO_SEQUENCE_SPECIFIC_MRNA_BINDING	-1.44	1.1	0	0	0	not significant
1249	GO_REGULATION_OF_CELL_COMMUNICATION_BY_ELECTRICAL_COUPLING	-1.44	1.09	0	0	0	not significant
1250	REACTOME_FRS_MEDIATED_FGFR4_SIGNALING	-1.44	1.09	0	0	0	not significant
1251	RAMPON_ENRICHED_LEARNING_ENVIRONMENT_EARLY_UP	-1.44	1.08	0	0	0	not significant
1252	MATZUK_IMPLANTATION_AND_UTERINE	-1.44	1.04	0	0	0	not significant
1253	BLALOCK_ALZHEIMERS_DISEASE_DN	-1.43	4	0	0	0	not significant
1254	GO_PROTEIN_FOLDING	-1.43	4	0	0	0	not significant
1255	REACTOME_C_TYPE_LECTIN_RECEPTORS_CLRS	-1.43	2.15	0	0	0	not significant
1256	GO_INTERLEUKIN_1_MEDIATED_SIGNALING_PATHWAY	-1.43	2.01	0	0	0	not significant
1257	GO_ESTABLISHMENT_OR_MAINTENANCE_OF_CELL_POLARITY	-1.43	1.92	0	0	0	not significant
1258	GO_INNER_MITOCHONDRIAL_MEMBRANE_PROTEIN_COMPLEX	-1.43	1.87	0	0	0	not significant
1259	GO_AMINE_METABOLIC_PROCESS	-1.43	1.79	0	0	0	not significant
1260	GO_LIMBIC_SYSTEM_DEVELOPMENT	-1.43	1.7	0	0	0	not significant
1261	MORI_PRE_BI_LYMPHOCYTE_UP	-1.43	1.68	0	0	0	not significant
1262	SARRIO_EPITHELIAL_MESENCHYMAL_TRANSITION_UP	-1.43	1.67	0	0	0	not significant
1263	SMITH_LIVER_CANCER	-1.43	1.38	0	0	0	not significant
1264	GO_CENTROMERE_COMPLEX_ASSEMBLY	-1.43	1.36	0	0	0	not significant
1265	REACTOME_RNA_POLYMERASE_I_TRANSCRIPTION_INITIATION	-1.43	1.36	0	0	0	not significant
1266	REACTOME_INTRAFLAGELLAR_TRANSPORT	-1.43	1.33	0	0	0	not significant
1267	PID_HEDGEHOG_GLI_PATHWAY	-1.43	1.28	0	0	0	not significant
1268	GROSS_HYPOXIA_VIA_ELK3_ONLY_DN	-1.43	1.25	0	0	0	not significant
1269	GO_MEIOTIC_SPINDLE_ORGANIZATION	-1.43	1.2	0	0	0	not significant
1270	REACTOME_ACTIVATION_OF_AMPK_DOWNSTREAM_OF_NMDARS	-1.43	1.18	0	0	0	not significant
1271	GO_LATERAL_VENTRICLE_DEVELOPMENT	-1.43	1.15	0	0	0	not significant
1272	GO_REGULATION_OF_HOMOTYPIC_CELL_CELL_ADHESION	-1.43	1.15	0	0	0	not significant
1273	GO_CATALYTIC_STEP_1_SPLICEOSOME	-1.43	1.14	0	0	0	not significant
1274	GO_NEGATIVE_REGULATION_OF_ESTABLISHMENT_OF_PROTEIN_LOCALIZATION_T	-1.43	1.14	0	0	0	not significant
1275	GO_PLASMINOGEN_ACTIVATION	-1.43	1.14	0	0	0	not significant
1276	GO_CYCLIN_DEPENDENT_PROTEIN_SERINE_THREONINE_KINASE_ACTIVATOR_ACT	-1.43	1.13	0	0	0	not significant
1277	PID_IGF1_PATHWAY	-1.43	1.13	0	0	0	not significant
1278	DORN_ADENOVIRUS_INFECTION_24HR_UP	-1.43	1.12	0	0	0	not significant
1279	FIRESTEIN_CTNNB1_PATHWAY	-1.43	1.12	0	0	0	not significant
1280	GO_ARP2_3_PROTEIN_COMPLEX	-1.43	1.1	0	0	0	not significant
1281	GO_POSITIVE_REGULATION_OF_RYANODINE_SENSITIVE_CALCIUM_RELEASE_CHAN	-1.43	1.09	0	0	0	not significant
1282	VANDESLUIS_COMMD1_TARGETS_GROUP_2_UP	-1.43	1.09	0	0	0	not significant
1283	GO_HISTONE_PRE_MRNA_3_END_PROCESSING_COMPLEX	-1.43	1.07	0	0	0	not significant
1284	GO_CDC42_PROTEIN_SIGNAL_TRANSDUCTION	-1.43	1.06	0	0	0	not significant
1285	GO_PROTEASOME_REGULATORY_PARTICLE_LID_SUBCOMPLEX	-1.43	1.06	0	0	0	not significant
1286	GO_RNA_POLYMERASE_II_GENERAL_TRANSCRIPTION_INITIATION_FACTOR_BINDIN	-1.43	1.06	0	0	0	not significant
1287	BIOCARTA_TEL_PATHWAY	-1.43	1.04	0	0	0	not significant
1288	GO_CULLIN_FAMILY_PROTEIN_BINDING	-1.43	1.04	0	0	0	not significant
1289	REACTOME_FRS_MEDIATED_FGFR3_SIGNALING	-1.43	1.03	0	0	0	not significant
1290	CHANDRAN_METASTASIS_UP	-1.42	4	0	0	0	not significant
1291	GO_CATALYTIC_COMPLEX	-1.42	4	0	0	0	not significant
1292	GO_NEGATIVE_REGULATION_OF_MITOTIC_CELL_CYCLE	-1.42	4	0	0	0	not significant
1293	GO_POLYMERIC_CYTOSKELETAL_FIBER	-1.42	4	0	0	0	not significant
1294	REACTOME_GENE_EXPRESSION_TRANSCRIPTION	-1.42	4	0	0	0	not significant
1295	VILLANUEVA_LIVER_CANCER_KRT19_UP	-1.42	2.37	0	0	0	not significant
1296	GO_GENE_SILENCING_BY_RNA	-1.42	2.17	0	0	0	not significant

1297	GO_PROTEIN_LOCALIZATION_TO_NUCLEUS	-1.42	2.03	0	0	0	not significant
1298	REACTOME_COPI_MEDIATED_ANTEROGRADE_TRANSPORT	-1.42	1.87	0	0	0	not significant
1299	GO_GLYCOLYTIC_PROCESS	-1.42	1.68	0	0	0	not significant
1300	KEGG_GAP_JUNCTION	-1.42	1.53	0	0	0	not significant
1301	GO_INTERACTION_WITH_SYMBIONT	-1.42	1.49	0	0	0	not significant
1302	GO_ANTIGEN_PROCESSING_AND_PRESENTATION_OF_EXOGENOUS_PEPTIDE_ANTI	-1.42	1.47	0	0	0	not significant
1303	GO_RESPONSE_TO_ANESTHETIC	-1.42	1.42	0	0	0	not significant
1304	REACTOME_REGULATION_OF_ACTIN_DYNAMICS_FOR_PHAGOCYTIC_CUP_FORMAT	-1.42	1.4	0	0	0	not significant
1305	BROWNE_HCMV_INFECTION_10HR_DN	-1.42	1.33	0	0	0	not significant
1306	KORKOLA_SEMINOMA_UP	-1.42	1.33	0	0	0	not significant
1307	GO_BLASTOCYST_FORMATION	-1.42	1.23	0	0	0	not significant
1308	GO_DISORDERED_DOMAIN_SPECIFIC_BINDING	-1.42	1.22	0	0	0	not significant
1309	GO_U12_TYPE_SPLICEOSOMAL_COMPLEX	-1.42	1.2	0	0	0	not significant
1310	SHIN_B_CELL_LYMPHOMA_CLUSTER_7	-1.42	1.2	0	0	0	not significant
1311	CHEOK_RESPONSE_TO_MERCAPTOPURINE_AND_HD_MTX_UP	-1.42	1.18	0	0	0	not significant
1312	YIH_RESPONSE_TO_ARSENITE_C1	-1.42	1.18	0	0	0	not significant
1313	GO_MICOS_COMPLEX	-1.42	1.16	0	0	0	not significant
1314	GO_REGULATION_OF_SMAD_PROTEIN_COMPLEX_ASSEMBLY	-1.42	1.14	0	0	0	not significant
1315	GO_RIBOSOMAL_SMALL_SUBUNIT_EXPORT_FROM_NUCLEUS	-1.42	1.14	0	0	0	not significant
1316	CHR17P12	-1.42	1.13	0	0	0	not significant
1317	GO_2_IRON_2_SULFUR_CLUSTER_BINDING	-1.42	1.13	0	0	0	not significant
1318	GO_HISTONE_H4_R3_METHYLATION	-1.42	1.13	0	0	0	not significant
1319	GO_POSITIVE_REGULATION_OF_TRANSCRIPTION_BY_RNA_POLYMERASE_III	-1.42	1.12	0	0	0	not significant
1320	GO_MALE_MATING_BEHAVIOR	-1.42	1.11	0	0	0	not significant
1321	GO_MITOCHONDRION_DISTRIBUTION	-1.42	1.11	0	0	0	not significant
1322	GO_SPINDLE_MIDZONE_ASSEMBLY	-1.42	1.11	0	0	0	not significant
1323	GO_ENTRAINMENT_OF_CIRCADIAN_CLOCK_BY_PHOTOPERIOD	-1.42	1.1	0	0	0	not significant
1324	BIOCARTA_PTC1_PATHWAY	-1.42	1.09	0	0	0	not significant
1325	GO_NEGATIVE_REGULATION_OF_EPITHELIAL_TO_MESENCHYMAL_TRANSITION	-1.42	1.09	0	0	0	not significant
1326	GO_ENDOCYTIC_ADAPTOR_ACTIVITY	-1.42	1.08	0	0	0	not significant
1327	GO_RESPONSE_TO_VITAMIN_A	-1.42	1.07	0	0	0	not significant
1328	FARMER_BREAST_CANCER_CLUSTER_5	-1.42	1.06	0	0	0	not significant
1329	REACTOME_ACTIVATED_NTRK2_SIGNALS_THROUGH_FRS2_AND_FRS3	-1.42	1.06	0	0	0	not significant
1330	REACTOME_REGULATION_OF_PYRUVATE_DEHYDROGENASE_PDH_COMPLEX	-1.42	1.06	0	0	0	not significant
1331	CERIBELLI_PROMOTERS_INACTIVE_AND_BOUND_BY_NFY	-1.42	1.05	0	0	0	not significant
1332	GO_OUTFLOW_TRACT_SEPTUM_MORPHOGENESIS	-1.42	1.04	0	0	0	not significant
1333	GO_SARCOPLASMIC_RETICULUM_CALCIUM_ION_TRANSPORT	-1.42	1.03	0	0	0	not significant
1334	REACTOME_IL_6_TYPE_CYTOKINE_RECEPTOR_LIGAND_INTERACTIONS	-1.42	1.03	0	0	0	not significant
1335	REACTOME_PURINE_RIBONUCLEOSIDE_MONOPHOSPHATE_BIOSYNTHESIS	-1.42	1.03	0	0	0	not significant
1336	GO_RIBOSOMAL_LARGE_SUBUNIT_EXPORT_FROM_NUCLEUS	-1.42	1.01	0	0	0	not significant
1337	REACTOME_MASTL_FACILITATES_MITOTIC_PROGRESSION	-1.42	0.99	0	0	0	not significant
1338	GO_ADENYL_NUCLEOTIDE_BINDING	-1.41	4	0	0	0	not significant
1339	GO_DRUG_BINDING	-1.41	4	0	0	0	not significant
1340	GO_NEGATIVE_REGULATION_OF_CELL_CYCLE_PHASE_TRANSITION	-1.41	4	0	0	0	not significant
1341	GO_REGULATION_OF_PROTEIN_STABILITY	-1.41	4	0	0	0	not significant
1342	GO_SUPRAMOLECULAR_COMPLEX	-1.41	4	0	0	0	not significant
1343	JOHNSTONE_PARVB_TARGETS_2_DN	-1.41	4	0	0	0	not significant
1344	GO_TRANSFERASE_COMPLEX_TRANSFERRING_PHOSPHORUS_CONTAINING_GROU	-1.41	2.04	0	0	0	not significant
1345	WATANABE_RECTAL_CANCER_RADIOTHERAPY_RESPONSIVE_DN	-1.41	1.93	0	0	0	not significant
1346	DITTMER_PTHLH_TARGETS_UP	-1.41	1.85	0	0	0	not significant
1347	EIF4E_UP	-1.41	1.75	0	0	0	not significant
1348	BROWNE_HCMV_INFECTION_12HR_UP	-1.41	1.72	0	0	0	not significant
1349	REACTOME_ANTIGEN_PROCESSING_CROSS_PRESENTATION	-1.41	1.67	0	0	0	not significant
1350	GCNP_SHH_UP_EARLY.V1_UP	-1.41	1.61	0	0	0	not significant
1351	REACTOME_INTEGRATION_OF_ENERGY_METABOLISM	-1.41	1.55	0	0	0	not significant
1352	REACTOME_RNA_POLYMERASE_I_PROMOTER_ESCAPE	-1.41	1.55	0	0	0	not significant
1353	GO_POSITIVE_REGULATION_OF_PROTEIN_LOCALIZATION_TO_NUCLEUS	-1.41	1.47	0	0	0	not significant
1354	CHNG_MULTIPLE_MYELOMA_HYPERPLOID_DN	-1.41	1.3	0	0	0	not significant
1355	CHR5Q33	-1.41	1.28	0	0	0	not significant
1356	GO_ENTRAINMENT_OF_CIRCADIAN_CLOCK	-1.41	1.27	0	0	0	not significant
1357	GO_CARTILAGE_DEVELOPMENT_INVOLVED_IN_ENDOCHONDRAL_BONE_MORPHOG	-1.41	1.21	0	0	0	not significant
1358	BIOCARTA_SALMONELLA_PATHWAY	-1.41	1.2	0	0	0	not significant
1359	REACTOME_DAG_AND_IP3_SIGNALING	-1.41	1.2	0	0	0	not significant
1360	XU_HGF_TARGETS_INDUCED_BY_AKT1_48HR_DN	-1.41	1.2	0	0	0	not significant
1361	HWANG_PROSTATE_CANCER_MARKERS	-1.41	1.15	0	0	0	not significant
1362	BIOCARTA_SRCRPTP_PATHWAY	-1.41	1.14	0	0	0	not significant
1363	GO_ESTABLISHMENT_OF_PROTEIN_LOCALIZATION_TO_MITOCHONDRIAL_MEMBRAI	-1.41	1.14	0	0	0	not significant
1364	GO_RESPONSE_TO_COPPER_ION	-1.41	1.13	0	0	0	not significant
1365	REACTOME_ERKS_ARE_INACTIVATED	-1.41	1.12	0	0	0	not significant
1366	GO_IRON_SULFUR_CLUSTER_ASSEMBLY	-1.41	1.11	0	0	0	not significant
1367	GO_PROTEASOME_BINDING	-1.41	1.11	0	0	0	not significant
1368	GO_SMN_COMPLEX	-1.41	1.09	0	0	0	not significant
1369	YORDY_RECIPROCAL_REGULATION_BY_ETS1_AND_SP100_UP	-1.41	1.08	0	0	0	not significant
1370	GO_BINDING_OF_SPERM_TO_ZONA_PELLUCIDA	-1.41	1.07	0	0	0	not significant
1371	BIOCARTA_RAN_PATHWAY	-1.41	1.06	0	0	0	not significant
1372	GO_HEART_TRABECULA_FORMATION	-1.41	1.06	0	0	0	not significant
1373	GO_SUCCINATE_METABOLIC_PROCESS	-1.41	1.05	0	0	0	not significant
1374	GO_ADENYL_NUCLEOTIDE_EXCHANGE_FACTOR_ACTIVITY	-1.41	1.02	0	0	0	not significant
1375	GO_POSITIVE_REGULATION_OF_AMYLOID_PRECURSOR_PROTEIN_CATABOLIC_PR	-1.41	1.01	0	0	0	not significant
1376	GAVIN_IL2_RESPONSIVE_FOXP3_TARGETS_UP	-1.41	0.96	0	0	0	not significant
1377	PUJANA_ATM_PCC_NETWORK	-1.40	4	0	0	0	not significant
1378	REACTOME_ADAPTIVE_IMMUNE_SYSTEM	-1.40	4	0	0	0	not significant
1379	HALLMARK_MTORC1_SIGNALING	-1.40	2.36	0	0	0	not significant
1380	GO_NUCLEOSOME_ORGANIZATION	-1.40	1.93	0	0	0	not significant
1381	GO_MICROTUBULE_BASED_MOVEMENT	-1.40	1.92	0	0	0	not significant
1382	MISSIAGLIA_REGULATED_BY_METHYLATION_DN	-1.40	1.87	0	0	0	not significant
1383	CROONQUIST_NRAS_VS_STROMAL_STIMULATION_DN	-1.40	1.83	0	0	0	not significant
1384	SMIRNOV_RESPONSE_TO_IR_6HR_DN	-1.40	1.61	0	0	0	not significant
1385	GO_REGULATION_OF_VIRAL_TRANSCRIPTION	-1.40	1.51	0	0	0	not significant
1386	SAKAI_CHRONIC_HEPATITIS_VS_LIVER_CANCER_UP	-1.40	1.46	0	0	0	not significant
1387	TURASHVILI_BREAST_DUCTAL_CARCINOMA_VS_LOBULAR_NORMAL_UP	-1.40	1.41	0	0	0	not significant
1388	GO_POSITIVE_REGULATION_OF_PROTEIN_LOCALIZATION_TO_CELL_PERIPHERY	-1.40	1.4	0	0	0	not significant
1389	GO_CHROMOSOME_CONDENSATION	-1.40	1.3	0	0	0	not significant
1390	REACTOME_SIGNALING_BY_NOTCH3	-1.40	1.23	0	0	0	not significant
1391	ZHU_CMV_8_HR_DN	-1.40	1.21	0	0	0	not significant
1392	REACTOME_GOLGI_ASSOCIATED_VESICLE_BIOGENESIS	-1.40	1.2	0	0	0	not significant
1393	GO_SPERM_EGG_RECOGNITION	-1.40	1.17	0	0	0	not significant
1394	GO_PROSTATE_GLAND_DEVELOPMENT	-1.40	1.16	0	0	0	not significant
1395	GO_REGULATION_OF_EPITHELIAL_TO_MESENCHYMAL_TRANSITION	-1.40	1.15	0	0	0	not significant
1396	GO_REGULATION_OF_RYANODINE_SENSITIVE_CALCIUM_RELEASE_CHANNEL_ACTI	-1.40	1.11	0	0	0	not significant
1397	GO_POLYNUCLEOTIDE_ADENYLYLTRANSFERASE_ACTIVITY	-1.40	1.09	0	0	0	not significant
1398	BIOCARTA_AT1R_PATHWAY	-1.40	1.07	0	0	0	not significant
1399	GO_NEGATIVE_REGULATION_OF_NEURON_PROJECTION_REGENERATION	-1.40	1.07	0	0	0	not significant
1400	REACTOME_G_PROTEIN_MEDIATED_EVENTS	-1.40	1.07	0	0	0	not significant
1401	GO_DENSE_CORE_GRANULE_TRANSPORT	-1.40	1.06	0	0	0	not significant
1402	GO_RNA_POLYMERASE_II_CTD_HEPTAPEPTIDE_REPEAT_KINASE_ACTIVITY	-1.40	1.06	0	0	0	not significant
1403	GO_COMPACT_MYELIN	-1.40	1.04	0	0	0	not significant
1404	GO_KIDNEY_MESENCHYME_DEVELOPMENT	-1.40	1.04	0	0	0	not significant
1405	GO_MITOCHONDRIAL_RNA_3_END_PROCESSING	-1.40	1.04	0	0	0	not significant

1406	MINGUEZ_LIVER_CANCER_VASCULAR_INVASION_UP	-1.40	1.01	0	0	0	not significant
1407	GO_PRE_MIRNA_BINDING	-1.40	1	0	0	0	not significant
1408	GO_GLUCOSE_BINDING	-1.40	0.99	0	0	0	not significant
1409	GO_HEART_TRABECULA_MORPHOGENESIS	-1.40	0.99	0	0	0	not significant
1410	GO_NEGATIVE_REGULATION_OF_ANDROGEN_RECEPTOR_SIGNALING_PATHWAY	-1.40	0.99	0	0	0	not significant
1411	GO_CYCLOSPORIN_A_BINDING	-1.40	0.97	0	0	0	not significant
1412	GO_NEGATIVE_REGULATION_OF_ORGANELLE_ORGANIZATION	-1.39	4	0	0	0	not significant
1413	GO_REGULATION_OF_SIGNAL_TRANSDUCTION_BY_P53_CLASS_MEDIATOR	-1.39	2.42	0	0	0	not significant
1414	REACTOME_TRANSLATION	-1.39	2.31	0	0	0	not significant
1415	GO_REGULATION_OF_ANIMAL_ORGAN_MORPHOGENESIS	-1.39	2.08	0	0	0	not significant
1416	GCNP_SHH_UP_LATE.V1_UP	-1.39	1.91	0	0	0	not significant
1417	GO_REGULATION_OF_TRANSLATIONAL_INITIATION	-1.39	1.75	0	0	0	not significant
1418	REACTOME_RNA_POLYMERASE_I_TRANSCRIPTION	-1.39	1.71	0	0	0	not significant
1419	GO_REGULATION_OF_GENE_SILENCING	-1.39	1.69	0	0	0	not significant
1420	HALLMARK_PI3K_AKT_MTOR_SIGNALING	-1.39	1.64	0	0	0	not significant
1421	GO_REGULATION_OF_PROTEIN_LOCALIZATION_TO_NUCLEUS	-1.39	1.63	0	0	0	not significant
1422	REACTOME_ER_TO_GOLGI_ANTEROGRADE_TRANSPORT	-1.39	1.62	0	0	0	not significant
1423	GO_POSITIVE_REGULATION_OF_VIRAL_PROCESS	-1.39	1.59	0	0	0	not significant
1424	IKEDA_MIR30_TARGETS_UP	-1.39	1.56	0	0	0	not significant
1425	GO_MODIFICATION_OF_MORPHOLOGY_OR_PHYSIOLOGY_OF_OTHER_ORGANISM_	-1.39	1.3	0	0	0	not significant
1426	BRUINS_UVC_RESPONSE_VIA_TP53_GROUP_C	-1.39	1.27	0	0	0	not significant
1427	CHR14Q22	-1.39	1.25	0	0	0	not significant
1428	SWEET_KRAS_TARGETS_DN	-1.39	1.23	0	0	0	not significant
1429	GO_REGULATION_OF_PROTEIN_LOCALIZATION_TO_CELL_CORTEX	-1.39	1.19	0	0	0	not significant
1430	VANTVEER_BREAST_CANCER_BRCA1_UP	-1.39	1.18	0	0	0	not significant
1431	BIOCARTA_PYK2_PATHWAY	-1.39	1.16	0	0	0	not significant
1432	GUTIERREZ_MULTIPLE_MYELOMA_UP	-1.39	1.16	0	0	0	not significant
1433	REACTOME_HIV_ELONGATION_ARREST_AND_RECOVERY	-1.39	1.16	0	0	0	not significant
1434	REACTOME_METABOLIC_DISORDERS_OF_BIOLOGICAL_OXIDATION_ENZYMES	-1.39	1.16	0	0	0	not significant
1435	REACTOME_VEGFR2_MEDIATED_VASCULAR_PERMEABILITY	-1.39	1.15	0	0	0	not significant
1436	GO_GLANDULAR_EPITHELIAL_CELL_DIFFERENTIATION	-1.39	1.14	0	0	0	not significant
1437	CHR2P22	-1.39	1.13	0	0	0	not significant
1438	KEGG_FRUCTOSE_AND_MANNOSE_METABOLISM	-1.39	1.11	0	0	0	not significant
1439	KEGG_TYPE_II_DIABETES_MELLITUS	-1.39	1.11	0	0	0	not significant
1440	REACTOME_HSF1_ACTIVATION	-1.39	1.1	0	0	0	not significant
1441	GO_AZOLE_TRANSPORT	-1.39	1.08	0	0	0	not significant
1442	GO_NEGATIVE_REGULATION_OF_INTRINSIC_APOPTOTIC_SIGNALING_PATHWAY_BY	-1.39	1.06	0	0	0	not significant
1443	GO_POSITIVE_REGULATION_OF_AMYLOID_BETA_FORMATION	-1.39	1.06	0	0	0	not significant
1444	GO_ELL_EAF_COMPLEX	-1.39	1.04	0	0	0	not significant
1445	REACTOME_CTLA4_INHIBITORY_SIGNALING	-1.39	1.04	0	0	0	not significant
1446	GO_REGULATION_OF_CARDIAC_MUSCLE_CONTRACTION_BY_CALCIUM_ION_SIGNA	-1.39	1.03	0	0	0	not significant
1447	BIOCARTA_IGF1MTOR_PATHWAY	-1.39	1.02	0	0	0	not significant
1448	FARDIN_HYPOXIA_9	-1.39	1.02	0	0	0	not significant
1449	GO_MAINTENANCE_OF_APICAL_BASAL_CELL_POLARITY	-1.39	1.02	0	0	0	not significant
1450	REACTOME_MET_ACTIVATES_RAP1_AND_RAC1	-1.39	1.01	0	0	0	not significant
1451	GO_LRR_DOMAIN_BINDING	-1.39	1	0	0	0	not significant
1452	KANG_CISPLATIN_RESISTANCE_DN	-1.39	1	0	0	0	not significant
1453	REACTOME_THROMBOXANE_SIGNALLING_THROUGH_TP_RECEPTOR	-1.39	1	0	0	0	not significant
1454	GO_PHOTOPERIODISM	-1.39	0.99	0	0	0	not significant
1455	GO_TRNA_3_END_PROCESSING	-1.39	0.99	0	0	0	not significant
1456	KEGG_PHENYLALANINE_METABOLISM	-1.39	0.99	0	0	0	not significant
1457	GO_REGULATION_OF_MEMBRANE_PROTEIN_ECTODOMAIN_PROTEOLYSIS	-1.39	0.98	0	0	0	not significant
1458	GO_UBIQUITIN_PROTEIN_TRANSFERASE_REGULATOR_ACTIVITY	-1.39	0.98	0	0	0	not significant
1459	REACTOME_ARMS_MEDIATED_ACTIVATION	-1.39	0.98	0	0	0	not significant
1460	GO_DE_NOVO_PYRIMIDINE_NUCLEOBASE_BIOSYNTHETIC_PROCESS	-1.39	0.97	0	0	0	not significant
1461	OLSSON_E2F3_TARGETS_UP	-1.39	0.97	0	0	0	not significant
1462	GO_RNA_POLYMERASE_I_COMPLEX	-1.39	0.93	0	0	0	not significant
1463	GO_DNA_PACKAGING	-1.38	4	0	0	0	not significant
1464	GO_NUCLEOPLASM_PART	-1.38	4	0	0	0	not significant
1465	GO_RIBONUCLEOTIDE_BINDING	-1.38	4	0	0	0	not significant
1466	LINDGREN_BLADDER_CANCER_CLUSTER_3_UP	-1.38	4	0	0	0	not significant
1467	WANG_SMARCE1_TARGETS_DN	-1.38	2.25	0	0	0	not significant
1468	REACTOME_FC_EPSILON_RECEPTOR_FCERI_SIGNALING	-1.38	2.15	0	0	0	not significant
1469	REACTOME_DEUBIQUITINATION	-1.38	2.01	0	0	0	not significant
1470	GO_ESTABLISHMENT_OF_CELL_POLARITY	-1.38	1.61	0	0	0	not significant
1471	TAKAO_RESPONSE_TO_UVB_RADIATION_DN	-1.38	1.6	0	0	0	not significant
1472	GO_DNA_REPLICATION_INDEPENDENT_NUCLEOSOME_ORGANIZATION	-1.38	1.35	0	0	0	not significant
1473	PID_FOXO_PATHWAY	-1.38	1.31	0	0	0	not significant
1474	GO_CELLULAR_SENESCENCE	-1.38	1.3	0	0	0	not significant
1475	REACTOME_TRANSLOCATION_OF_SLC2A4_GLUT4_TO_THE_PLASMA_MEMBRANE	-1.38	1.3	0	0	0	not significant
1476	KYNG_RESPONSE_TO_H2O2_VIA_ERCC6_UP	-1.38	1.12	0	0	0	not significant
1477	GO_REGULATION_OF_ACTION_POTENTIAL	-1.38	1.09	0	0	0	not significant
1478	CHR2Q32	-1.38	1.08	0	0	0	not significant
1479	GO_ALDITOL_CATABOLIC_PROCESS	-1.38	1.08	0	0	0	not significant
1480	REACTOME_GLUCAGON_LIKE_PEPTIDE_1_GLP1_REGULATES_INSULIN_SECRETION	-1.38	1.07	0	0	0	not significant
1481	GO_SNRNA_PROCESSING	-1.38	1.05	0	0	0	not significant
1482	REACTOME_PINK_PARKIN_MEDIATED_MITOPHAGY	-1.38	1.05	0	0	0	not significant
1483	FUKUSHIMA_TNFSF11_TARGETS	-1.38	1.04	0	0	0	not significant
1484	GO_POSITIVE_REGULATION_OF_PATHWAY_RESTRICTED_SMAD_PROTEIN_PHOSPH	-1.38	1.03	0	0	0	not significant
1485	REACTOME_SHC_RELATED_EVENTS_TRIGGERED_BY_IGF1R	-1.38	1.03	0	0	0	not significant
1486	BIOCARTA_NUCLEARRS_PATHWAY	-1.38	1.02	0	0	0	not significant
1487	GO_DNA_TOPOISOMERASE_BINDING	-1.38	1.02	0	0	0	not significant
1488	PHESSE_TARGETS_OF_APC_AND_MBD2_DN	-1.38	1	0	0	0	not significant
1489	GO_CALCIUM_ION_EXPORT	-1.38	0.99	0	0	0	not significant
1490	STEIN_ESTROGEN_RESPONSE_NOT_VIA_ESRRA	-1.38	0.99	0	0	0	not significant
1491	GO_ASYMMETRIC_CELL_DIVISION	-1.38	0.98	0	0	0	not significant
1492	GO_MAINTENANCE_OF_ORGANELLE_LOCATION	-1.38	0.97	0	0	0	not significant
1493	LOPEZ_MESOTHELIOMA_SURVIVAL_DN	-1.38	0.97	0	0	0	not significant
1494	REACTOME_FRS2_MEDIATED_ACTIVATION	-1.38	0.97	0	0	0	not significant
1495	TERAO_AOX4_TARGETS_SKIN_DN	-1.38	0.97	0	0	0	not significant
1496	GO_RESPONSE_TO_SALT	-1.38	0.96	0	0	0	not significant
1497	REACTOME_G_BETA:GAMMA_SIGNALLING_THROUGH_CDC42	-1.38	0.96	0	0	0	not significant
1498	GALLUZZI_PREVENT_MITOCHONDIAL_PERMEABILIZATION	-1.38	0.95	0	0	0	not significant
1499	REACTOME_RECEPTOR_TYPE_TYROSINE_PROTEIN_PHOSPHATASES	-1.38	0.94	0	0	0	not significant
1500	REACTOME_TRNA_MODIFICATION_IN_THE_MITOCHONDRION	-1.38	0.93	0	0	0	not significant
1501	GO_EPITHELIAL_CELL_FATE_COMMITMENT	-1.38	0.91	0	0	0	not significant
1502	GO_DEPYRIMIDINATION	-1.38	0.9	0	0	0	not significant
1503	GO_PROTEIN_POLYUBIQUITINATION	-1.37	4	0	0	0	not significant
1504	SPIELMAN_LYMPHOBLAST_EUROPEAN_VS_ASIAN_DN	-1.37	4	0	0	0	not significant
1505	BROWNE_HCMV_INFECTION_16HR_UP	-1.37	2.4	0	0	0	not significant
1506	REACTOME_SIGNALING_BY_NUCLEAR_RECEPTORS	-1.37	2.39	0	0	0	not significant
1507	WEST_ADRENOCORTICAL_TUMOR_UP	-1.37	2.24	0	0	0	not significant
1508	REACTOME_EPIGENETIC_REGULATION_OF_GENE_EXPRESSION	-1.37	2.15	0	0	0	not significant
1509	GO_PHOSPHOPROTEIN_PHOSPHATASE_ACTIVITY	-1.37	1.59	0	0	0	not significant
1510	PUJANA_BRCA_CENTERED_NETWORK	-1.37	1.43	0	0	0	not significant
1511	RAMASWAMY_METASTASIS_UP	-1.37	1.3	0	0	0	not significant
1512	GO_TRANSCRIPTION_ELONGATION_FACTOR_COMPLEX	-1.37	1.25	0	0	0	not significant
1513	REACTOME_INTERCONVERSION_OF_NUCLEOTIDE_DI_AND_TRIPHOSPHATES	-1.37	1.24	0	0	0	not significant
1514	GO_HSP90_PROTEIN_BINDING	-1.37	1.21	0	0	0	not significant

1515	GO_POSITIVE_REGULATION_OF_TELOMERE_MAINTENANCE	-1.37	1.21	0	0	0	not significant
1516	REACTOME_G_ALPHA_Z_SIGNALLING_EVENTS	-1.37	1.13	0	0	0	not significant
1517	REACTOME_NEGATIVE_REGULATION_OF_MAPK_PATHWAY	-1.37	1.1	0	0	0	not significant
1518	GO_CLATHRIN_COAT_OF_ENDOCYTIC_VESICLE	-1.37	1.03	0	0	0	not significant
1519	GO_DENDRITE_CYTOPLASM	-1.37	1.03	0	0	0	not significant
1520	GO_NUCLEAR_NUCLEOSOME	-1.37	1.03	0	0	0	not significant
1521	GO_NUCLEAR_STRESS_GRANULE	-1.37	1.03	0	0	0	not significant
1522	NIKOLSKY_BREAST_CANCER_19Q13.1_AMPLICON	-1.37	1.01	0	0	0	not significant
1523	REACTOME_POST_CHAPERONIN_TUBULIN_FOLDING_PATHWAY	-1.37	1	0	0	0	not significant
1524	GO_VITAMIN_D_RECEPTOR_SIGNALING_PATHWAY	-1.37	0.99	0	0	0	not significant
1525	LIU_IL13_MEMORY_MODEL_UP	-1.37	0.98	0	0	0	not significant
1526	ONGUSAHA_BRCA1_TARGETS_DN	-1.37	0.98	0	0	0	not significant
1527	CAFFAREL_RESPONSE_TO_THC_8HR_5_DN	-1.37	0.97	0	0	0	not significant
1528	GO_BONE_GROWTH	-1.37	0.97	0	0	0	not significant
1529	GO_INTEGRATOR_COMPLEX	-1.37	0.97	0	0	0	not significant
1530	GO_MAP_KINASE_KINASE_ACTIVITY	-1.37	0.97	0	0	0	not significant
1531	GO_RESPONSE_TO_NITRIC_OXIDE	-1.37	0.97	0	0	0	not significant
1532	TAKEDA_TARGETS_OF_NUP98_HOXA9_FUSION_3D_DN	-1.37	0.97	0	0	0	not significant
1533	GO_7S_RNA_BINDING	-1.37	0.96	0	0	0	not significant
1534	GO_RNA_DIRECTED_DNA_POLYMERASE_ACTIVITY	-1.37	0.96	0	0	0	not significant
1535	CONRAD_STEM_CELL	-1.37	0.95	0	0	0	not significant
1536	GO_NEGATIVE_REGULATION_OF_INCLUSION_BODY_ASSEMBLY	-1.37	0.95	0	0	0	not significant
1537	GO_RNA_POLYMERASE_II_ACTIVITY	-1.37	0.94	0	0	0	not significant
1538	NIELSEN_LEIOMYOSARCOMA_CNN1_UP	-1.37	0.94	0	0	0	not significant
1539	SEIDEN_MET_SIGNALING	-1.37	0.94	0	0	0	not significant
1540	CHR3P13	-1.37	0.93	0	0	0	not significant
1541	GO_POSITIVE_REGULATION_OF_ATTACHMENT_OF_SPINDLE_MICROTUBULES_TO_	-1.37	0.92	0	0	0	not significant
1542	GO_REGULATION_OF_T_HELPER_2_CELL_DIFFERENTIATION	-1.37	0.91	0	0	0	not significant
1543	GO_POLY_G_BINDING	-1.37	0.87	0	0	0	not significant
1544	GO_KINETOCHORE_ASSEMBLY	-1.37	0.86	0	0	0	not significant
1545	ENK_UV_RESPONSE_KERATINOCYTE_DN	-1.36	4	0	0	0	not significant
1546	GO_CHROMOSOME	-1.36	4	0	0	0	not significant
1547	GO_DEPHOSPHORYLATION	-1.36	4	0	0	0	not significant
1548	GO_ESTABLISHMENT_OF_ORGANELLE_LOCALIZATION	-1.36	4	0	0	0	not significant
1549	GO_GENERATION_OF_PRECURSOR_METABOLITES_AND_ENERGY	-1.36	4	0	0	0	not significant
1550	GO_MODIFICATION_DEPENDENT_MACROMOLECULE_CATABOLIC_PROCESS	-1.36	4	0	0	0	not significant
1551	GO_NUCLEAR_BODY	-1.36	4	0	0	0	not significant
1552	REACTOME_ORGANELLE_BIOGENESIS_AND_MAINTENANCE	-1.36	4	0	0	0	not significant
1553	REACTOME_SIGNALING_BY_NOTCH	-1.36	2.37	0	0	0	not significant
1554	REACTOME_OTHER_INTERLEUKIN_SIGNALING	-1.36	2.32	0	0	0	not significant
1555	GO_PROTEIN_CONTAINING_COMPLEX_DISASSEMBLY	-1.36	1.93	0	0	0	not significant
1556	REACTOME_G2_M_CHECKPOINTS	-1.36	1.74	0	0	0	not significant
1557	PURBEY_TARGETS_OF_CTBP1_AND_SATB1_DN	-1.36	1.65	0	0	0	not significant
1558	GO_N_ACETYLTRANSFERASE_ACTIVITY	-1.36	1.51	0	0	0	not significant
1559	REACTOME_AURKA_ACTIVATION_BY_TPX2	-1.36	1.48	0	0	0	not significant
1560	SENESE_HDAC2_TARGETS_UP	-1.36	1.47	0	0	0	not significant
1561	GO_LAMELLIPODIUM_ORGANIZATION	-1.36	1.42	0	0	0	not significant
1562	GO_MITOCHONDRIAL_TRANSMEMBRANE_TRANSPORT	-1.36	1.38	0	0	0	not significant
1563	GO_P53_BINDING	-1.36	1.31	0	0	0	not significant
1564	GO_NEGATIVE_REGULATION_OF_RNA_CATABOLIC_PROCESS	-1.36	1.3	0	0	0	not significant
1565	ZHAN_MULTIPLE_MYELOMA_CD2_DN	-1.36	1.27	0	0	0	not significant
1566	REACTOME_TRANSCRIPTIONAL_REGULATION_BY_MECP2	-1.36	1.26	0	0	0	not significant
1567	REACTOME_RNA_POLYMERASE_I_TRANSCRIPTION_TERMINATION	-1.36	1.15	0	0	0	not significant
1568	PID_PI3KCI_AKT_PATHWAY	-1.36	1.13	0	0	0	not significant
1569	GO_POSITIVE_REGULATION_OF_PROTEIN_COMPLEX_DISASSEMBLY	-1.36	1.03	0	0	0	not significant
1570	GO_HETEROTRIMERIC_G_PROTEIN_COMPLEX	-1.36	1.02	0	0	0	not significant
1571	GO_CENTRAL_NERVOUS_SYSTEM_NEURON_AXONOGENESIS	-1.36	1.01	0	0	0	not significant
1572	REACTOME_ACTIVATION_OF_RAS_IN_B_CELLS	-1.36	1	0	0	0	not significant
1573	GO_EUKARYOTIC_TRANSLATION_INITIATION_FACTOR_3_COMPLEX_EIF3M	-1.36	0.99	0	0	0	not significant
1574	GO_TROPHECTODERMAL_CELL_DIFFERENTIATION	-1.36	0.99	0	0	0	not significant
1575	CAIRO_PML_TARGETS_BOUND_BY_MYC_UP	-1.36	0.98	0	0	0	not significant
1576	GO_SCHMIDT_LANTERMAN_INCISURE	-1.36	0.98	0	0	0	not significant
1577	REACTOME_EGFR_TRANSACTIVATION_BY_GASTRIN	-1.36	0.96	0	0	0	not significant
1578	GO_RNA_POLYMERASE_II_CORE_COMPLEX	-1.36	0.95	0	0	0	not significant
1579	LEE_METASTASIS_AND_RNA_PROCESSING_UP	-1.36	0.94	0	0	0	not significant
1580	REACTOME_PP2A_MEDIATED_DEPHOSPHORYLATION_OF_KEY_METABOLIC_FACT(	-1.36	0.94	0	0	0	not significant
1581	ZERBINI_RESPONSE_TO_SULINDAC_DN	-1.36	0.94	0	0	0	not significant
1582	HONRADO_BREAST_CANCER_BRCA1_VS_BRCA2	-1.36	0.93	0	0	0	not significant
1583	GO_MINUS_END_DIRECTED_ORGANELLE_TRANSPORT_ALONG_MICROTUBULE	-1.36	0.92	0	0	0	not significant
1584	GO_PYRIMIDINE_NUCLEOBASE_BIOSYNTHETIC_PROCESS	-1.36	0.92	0	0	0	not significant
1585	PYEON_CANCER_HEAD_AND_NECK_VS_CERVICAL_DN	-1.36	0.92	0	0	0	not significant
1586	GO_BETA_CATENIN_DESTRUCTION_COMPLEX_ASSEMBLY	-1.36	0.91	0	0	0	not significant
1587	GO_RESPONSE_TO_INSECTICIDE	-1.36	0.9	0	0	0	not significant
1588	REACTOME_JOSEPHIN_DOMAIN_DUBS	-1.36	0.9	0	0	0	not significant
1589	GO_REGULATION_OF_HIPPO_SIGNALING	-1.36	0.87	0	0	0	not significant
1590	GO_POSITIVE_REGULATION_OF_RNA_POLYMERASE_II_TRANSCRIPTIONAL_PREINIT	-1.36	0.84	0	0	0	not significant
1591	REACTOME_METHYLATION	-1.36	0.81	0	0	0	not significant
1592	GO_PROTEIN_CONTAINING_COMPLEX_ASSEMBLY	-1.35	4	0	0	0	not significant
1593	GO_ANTIGEN_PROCESSING_AND_PRESENTATION	-1.35	1.88	0	0	0	not significant
1594	GO_POSITIVE_REGULATION_OF_NUCLEOBASE_CONTAINING_COMPOUND_METABO	-1.35	1.75	0	0	0	not significant
1595	GO_REGULATION_OF_UBIQUITIN_DEPENDENT_PROTEIN_CATABOLIC_PROCESS	-1.35	1.68	0	0	0	not significant
1596	GO_PROTEIN_STABILIZATION	-1.35	1.66	0	0	0	not significant
1597	YANG_BCL3_TARGETS_UP	-1.35	1.65	0	0	0	not significant
1598	ZHENG_GLIOBLASTOMA_PLASTICITY_UP	-1.35	1.61	0	0	0	not significant
1599	GO_RESPIRATORY_ELECTRON_TRANSPORT_CHAIN	-1.35	1.57	0	0	0	not significant
1600	GO_ANTERIOR_POSTERIOR_PATTERN_SPECIFICATION	-1.35	1.46	0	0	0	not significant
1601	CHR22Q12	-1.35	1.41	0	0	0	not significant
1602	GO_FC_EPSILON_RECEPTOR_SIGNALING_PATHWAY	-1.35	1.4	0	0	0	not significant
1603	MORI_MATURE_B_LYMPHOCYTE_DN	-1.35	1.33	0	0	0	not significant
1604	REACTOME_ASSEMBLY_OF_THE_PRE_REPLICATIVE_COMPLEX	-1.35	1.29	0	0	0	not significant
1605	GAZDA_DIAMOND_BLACKFAN_ANEMIA_MYELOID_DN	-1.35	1.28	0	0	0	not significant
1606	GO_PEPTIDE_N_ACETYLTRANSFERASE_ACTIVITY	-1.35	1.24	0	0	0	not significant
1607	GO_REGULATION_OF_CARBOHYDRATE_CATABOLIC_PROCESS	-1.35	1.24	0	0	0	not significant
1608	GO_TRANSCRIPTION_FACTOR_TFIID_COMPLEX	-1.35	1.13	0	0	0	not significant
1609	THILLAINADESAN_ZNF217_TARGETS_UP	-1.35	1.08	0	0	0	not significant
1610	OLSSON_E2F3_TARGETS_DN	-1.35	1.07	0	0	0	not significant
1611	GO_POSITIVE_REGULATION_OF_CHROMOSOME_SEGREGATION	-1.35	1.06	0	0	0	not significant
1612	REACTOME_PYRUVATE_METABOLISM	-1.35	1.06	0	0	0	not significant
1613	CHR10Q21	-1.35	1.05	0	0	0	not significant
1614	GO_NUCLEAR_TRANSCRIPTIONAL_REPRESSOR_COMPLEX	-1.35	1.05	0	0	0	not significant
1615	GRADE_COLON_VS_RECTAL_CANCER_DN	-1.35	1.02	0	0	0	not significant
1616	GO_POSITIVE_REGULATION_OF_DNA_TEMPLATED_TRANSCRIPTION_ELONGATION	-1.35	1	0	0	0	not significant
1617	GO_ANNULATE_LAMELLAE	-1.35	0.97	0	0	0	not significant
1618	GO_REGULATION_OF_ANOIKIS	-1.35	0.97	0	0	0	not significant
1619	GO_POSITIVE_REGULATION_OF_GLIAL_CELL_DIFFERENTIATION	-1.35	0.96	0	0	0	not significant
1620	PETRETTO_HEART_MASS_QTL_CIS_UP	-1.35	0.96	0	0	0	not significant
1621	GO_PROTEIN_FOLDING_IN_ENDOPLASMIC_RETICULUM	-1.35	0.95	0	0	0	not significant
1622	BIOCARTA_MPR_PATHWAY	-1.35	0.93	0	0	0	not significant
1623	GO_GEMINI_OF_COILED_BODIES	-1.35	0.93	0	0	0	not significant

1624	GO_MICROTUBULE_ORGANIZING_CENTER_LOCALIZATION	-1.35	0.93	0	0	0	not significant
1625	GO_NUCLEOBASE_BIOSYNTHETIC_PROCESS	-1.35	0.93	0	0	0	not significant
1626	MARSON_FOXP3_TARGETS_STIMULATED_DN	-1.35	0.91	0	0	0	not significant
1627	REACTOME_REGULATION_OF_FOXO_TRANSCRIPTIONAL_ACTIVITY_BY_ACETYLATI	-1.35	0.91	0	0	0	not significant
1628	GO_PROTEIN_ARGININE_N_METHYLTRANSFERASE_ACTIVITY	-1.35	0.89	0	0	0	not significant
1629	GO_SYNAPTIC_VESICLE_BUDDING	-1.35	0.89	0	0	0	not significant
1630	GO_TRNA_WOBBLE_URIDINE_MODIFICATION	-1.35	0.89	0	0	0	not significant
1631	VALK_AML_CLUSTER_6	-1.35	0.89	0	0	0	not significant
1632	GO_CALCIUM_DEPENDENT_PROTEIN_KINASE_ACTIVITY	-1.35	0.87	0	0	0	not significant
1633	GO_DEPHOSPHORYLATION_OF_RNA_POLYMERASE_II_C_TERMINAL_DOMAIN	-1.35	0.87	0	0	0	not significant
1634	GARGALOVIC_RESPONSE_TO_OXIDIZED_PHOSPHOLIPIDS_SALMON_DN	-1.35	0.85	0	0	0	not significant
1635	REACTOME_TRANSPORT_OF_CONNEXONS_TO_THE_PLASMA_MEMBRANE	-1.35	0.84	0	0	0	not significant
1636	GO_NLRP3_INFLAMMASOME_COMPLEX_ASSEMBLY	-1.35	0.83	0	0	0	not significant
1637	GO_MORPHOGENESIS_OF_AN_EPITHELIUM	-1.34	4	0	0	0	not significant
1638	GO_DNA_CONFORMATION_CHANGE	-1.34	2.33	0	0	0	not significant
1639	GOLDRATH_ANTIGEN_RESPONSE	-1.34	2.26	0	0	0	not significant
1640	GO_ATPASE_ACTIVITY	-1.34	2.24	0	0	0	not significant
1641	REACTOME_PTEN_REGULATION	-1.34	1.67	0	0	0	not significant
1642	GO_REGULATION_OF_PHOSPHATASE_ACTIVITY	-1.34	1.6	0	0	0	not significant
1643	IWANAGA_CARCINOGENESIS_BY_KRAS_PTEN_UP	-1.34	1.41	0	0	0	not significant
1644	GO_ANTIGEN_PROCESSING_AND_PRESENTATION_OF_PEPTIDE_ANTIGEN_VIA_MHC	-1.34	1.33	0	0	0	not significant
1645	GO_HISTONE_EXCHANGE	-1.34	1.23	0	0	0	not significant
1646	GO_CARDIOCYTE_DIFFERENTIATION	-1.34	1.22	0	0	0	not significant
1647	MIDORIKAWA_AMPLIFIED_IN_LIVER_CANCER	-1.34	1.15	0	0	0	not significant
1648	HALLMARK_SPERMATOGENESIS	-1.34	1.12	0	0	0	not significant
1649	APRELIKOVA_BRCA1_TARGETS	-1.34	1.1	0	0	0	not significant
1650	GO_TORC2_SIGNALING	-1.34	1.06	0	0	0	not significant
1651	TONG_INTERACT_WITH_PTTG1	-1.34	1.02	0	0	0	not significant
1652	CHR5P15	-1.34	1	0	0	0	not significant
1653	GO_MOVEMENT_IN_ENVIRONMENT_OF_OTHER_ORGANISM_INVOLVED_IN_SYMBIOT	-1.34	1	0	0	0	not significant
1654	GO_TRABECULA_MORPHOGENESIS	-1.34	0.97	0	0	0	not significant
1655	GO_GLOMERULUS_DEVELOPMENT	-1.34	0.96	0	0	0	not significant
1656	GO_NEGATIVE_REGULATION_OF_CELL_AGING	-1.34	0.95	0	0	0	not significant
1657	GO_REGULATION_OF_MALE_GONAD_DEVELOPMENT	-1.34	0.95	0	0	0	not significant
1658	REACTOME_SIGNALING_BY_EGFR_IN_CANCER	-1.34	0.95	0	0	0	not significant
1659	GO_REGULATION_OF_SPINDLE_ASSEMBLY	-1.34	0.94	0	0	0	not significant
1660	GO_PORE_COMPLEX	-1.34	0.93	0	0	0	not significant
1661	GO_POSITIVE_REGULATION_OF_BONE_RESORPTION	-1.34	0.93	0	0	0	not significant
1662	GO_SUMO_BINDING	-1.34	0.93	0	0	0	not significant
1663	GO_LEUCINE_CATABOLIC_PROCESS	-1.34	0.92	0	0	0	not significant
1664	REACTOME_ADP_SIGNALLING_THROUGH_P2Y_PURINOCEPTOR_12	-1.34	0.91	0	0	0	not significant
1665	GO_VITAMIN_TRANSPORT	-1.34	0.9	0	0	0	not significant
1666	GRAHAM_CML_QUIESCENT_VS_CML_DIVIDING_UP	-1.34	0.87	0	0	0	not significant
1667	GO_AMP_METABOLIC_PROCESS	-1.34	0.86	0	0	0	not significant
1668	GO_RESPONSE_TO_OXYGEN_GLUCOSE_DEPRIVATION	-1.34	0.86	0	0	0	not significant
1669	BIOCARTA_AGPCR_PATHWAY	-1.34	0.85	0	0	0	not significant
1670	GO_NEGATIVE_REGULATION_OF_HOMOTYPIC_CELL_CELL_ADHESION	-1.34	0.85	0	0	0	not significant
1671	GO_DENDRITE_TERMINUS	-1.34	0.84	0	0	0	not significant
1672	GO_STAGA_COMPLEX	-1.34	0.84	0	0	0	not significant
1673	GO_TRANSLATION_TERMINATION_FACTOR_ACTIVITY	-1.34	0.82	0	0	0	not significant
1674	GO_REGULATION_OF_INTRACELLULAR_LIPID_TRANSPORT	-1.34	0.81	0	0	0	not significant
1675	GO_CELLULAR_COMPONENT_DISASSEMBLY	-1.33	4	0	0	0	not significant
1676	GO_PROTEIN_SERINE_THREONINE_KINASE_ACTIVITY	-1.33	4	0	0	0	not significant
1677	GO_POSITIVE_REGULATION_OF_MULTI_ORGANISM_PROCESS	-1.33	1.75	0	0	0	not significant
1678	ERBB2_UP.V1_DN	-1.33	1.71	0	0	0	not significant
1679	AFFAR_YY1_TARGETS_DN	-1.33	1.61	0	0	0	not significant
1680	GO_NEGATIVE_REGULATION_OF_NEURON_DIFFERENTIATION	-1.33	1.58	0	0	0	not significant
1681	REACTOME_INTERLEUKIN_1_FAMILY_SIGNALING	-1.33	1.38	0	0	0	not significant
1682	GO_FEMALE_GAMETE_GENERATION	-1.33	1.36	0	0	0	not significant
1683	DE_YY1_TARGETS_DN	-1.33	1.34	0	0	0	not significant
1684	KEGG_GLYCOLYSIS_GLUCONEOGENESIS	-1.33	1.11	0	0	0	not significant
1685	GO_RNA_STABILIZATION	-1.33	1.06	0	0	0	not significant
1686	GO_RNA_POLYMERASE_CORE_ENZYME_BINDING	-1.33	1.04	0	0	0	not significant
1687	BOHN_PRIMARY_IMMUNODEFICIENCY_SYNDROM_UP	-1.33	1.01	0	0	0	not significant
1688	GO_ACTIVATION_OF_MAPKK_ACTIVITY	-1.33	1.01	0	0	0	not significant
1689	REACTOME_SIGNALING_BY_MODERATE_KINASE_ACTIVITY_BRAF_MUTANTS	-1.33	1	0	0	0	not significant
1690	IIZUKA_LIVER_CANCER_PROGRESSION_G1_G2_DN	-1.33	0.97	0	0	0	not significant
1691	VARELA_ZMPSTE24_TARGETS_UP	-1.33	0.97	0	0	0	not significant
1692	GO_TELOMERASE_ACTIVITY	-1.33	0.96	0	0	0	not significant
1693	BARIS_THYROID_CANCER_UP	-1.33	0.95	0	0	0	not significant
1694	GO_REGULATION_OF_RESPIRATORY_GASEOUS_EXCHANGE_BY_NEUROLOGICAL_S	-1.33	0.95	0	0	0	not significant
1695	GO_MODULATION_OF_TRANSCRIPTION_IN_OTHER_ORGANISM_INVOLVED_IN_SYME	-1.33	0.94	0	0	0	not significant
1696	GO_POTASSIUM_CHANNEL_REGULATOR_ACTIVITY	-1.33	0.94	0	0	0	not significant
1697	GO_TRNA_WOBBLE_BASE_MODIFICATION	-1.33	0.94	0	0	0	not significant
1698	GO_NUCLEOSIDE_DIPHOSPHATE_METABOLIC_PROCESS	-1.33	0.93	0	0	0	not significant
1699	GO_POSITIVE_REGULATION_OF_HEART_GROWTH	-1.33	0.93	0	0	0	not significant
1700	KIM_MYCL1_AMPLIFICATION_TARGETS_DN	-1.33	0.91	0	0	0	not significant
1701	GO_HOST_CELL_PART	-1.33	0.88	0	0	0	not significant
1702	GO_PEPTIDYL_ARGININE_N_METHYLATION	-1.33	0.87	0	0	0	not significant
1703	GO_TRANSCRIPTIONALLY_ACTIVE_CHROMATIN	-1.33	0.87	0	0	0	not significant
1704	GO_CHROMATIN_SILENCING_COMPLEX	-1.33	0.86	0	0	0	not significant
1705	GO_LARGE_RIBOSOMAL_SUBUNIT_RRNA_BINDING	-1.33	0.86	0	0	0	not significant
1706	GO_NEUROBLAST_DIVISION	-1.33	0.86	0	0	0	not significant
1707	GO_REGULATION_OF_PLASMINOGEN_ACTIVATION	-1.33	0.86	0	0	0	not significant
1708	GO_RNA_INTERFERENCE	-1.33	0.86	0	0	0	not significant
1709	REACTOME_MAP3K8_TPL2_DEPENDENT_MAPK1_3_ACTIVATION	-1.33	0.86	0	0	0	not significant
1710	REACTOME_RUNX1_REGULATES_ESTROGEN_RECEPTOR_MEDIATED_TRANSCRIPT	-1.33	0.86	0	0	0	not significant
1711	GO_ESTABLISHMENT_OR_MAINTENANCE_OF_CYTOSKELETON_POLARITY	-1.33	0.85	0	0	0	not significant
1712	GO_CEREBELLAR_PURKINJE_CELL_LAYER_DEVELOPMENT	-1.33	0.84	0	0	0	not significant
1713	GO_NEGATIVE_REGULATION_OF_EXCITATORY_POSTSYNAPTIC_POTENTIAL	-1.33	0.84	0	0	0	not significant
1714	GO_SIGNAL_RECOGNITION_PARTICLE_ENDOPLASMIC_RETICULUM_TARGETING	-1.33	0.84	0	0	0	not significant
1715	REACTOME_SIGNALING_BY_LIGAND_RESPONSIVE_EGFR_VARIANTS_IN_CANCER	-1.33	0.84	0	0	0	not significant
1716	GO_GERM_CELL_NUCLEUS	-1.33	0.83	0	0	0	not significant
1717	GO_POSITIVE_REGULATION_OF_CIRCADIAN_RHYTHM	-1.33	0.83	0	0	0	not significant
1718	REACTOME_GAP_JUNCTION_ASSEMBLY	-1.33	0.82	0	0	0	not significant
1719	GO_DOUBLE_STRANDED_TELOMERIC_DNA_BINDING	-1.33	0.81	0	0	0	not significant
1720	GO_NERVE_GROWTH_FACTOR_SIGNALING_PATHWAY	-1.33	0.8	0	0	0	not significant
1721	REACTOME_SHC_MEDIATED_CASCADE:FGFR1	-1.33	0.79	0	0	0	not significant
1722	CHR13Q22	-1.33	0.78	0	0	0	not significant
1723	REACTOME_REGULATION_OF_CYTOSKELETAL_REMODELING_AND_CELL_SPREAD	-1.33	0.77	0	0	0	not significant
1724	GO_MORPHOGENESIS_OF_AN_EPITHELIAL_FOLD	-1.33	0.75	0	0	0	not significant
1725	REACTOME_MET_ACTIVATES_RAS_SIGNALING	-1.33	0.75	0	0	0	not significant
1726	GO_CELL_ADHESION_MOLECULE_BINDING	-1.32	4	0	0	0	not significant
1727	GO_MICROTUBULE_CYTOSKELETON	-1.32	4	0	0	0	not significant
1728	GO_ORGANIC_CYCLIC_COMPOUND_CATABOLIC_PROCESS	-1.32	4	0	0	0	not significant
1729	LINSLEY_MIR16_TARGETS	-1.32	2.09	0	0	0	not significant
1730	REACTOME_SIGNALING_BY_ROBO_RECEPTORS	-1.32	1.98	0	0	0	not significant
1731	GO_GOLGI_VESICLE_TRANSPORT	-1.32	1.92	0	0	0	not significant
1732	GO_CELLULAR_AMINO_ACID_METABOLIC_PROCESS	-1.32	1.89	0	0	0	not significant

1733	GO_PROTEIN_DNA_COMPLEX_SUBUNIT_ORGANIZATION	-1.32	1.85	0	0	0	not significant
1734	MARTORIATI_MDM4_TARGETS_NEUROEPITHELIUM_UP	-1.32	1.74	0	0	0	not significant
1735	PUJANA_BREAST_CANCER_LIT_INT_NETWORK	-1.32	1.4	0	0	0	not significant
1736	GO_NEGATIVE_REGULATION_OF_WNT_SIGNALING_PATHWAY	-1.32	1.36	0	0	0	not significant
1737	GO_REGULATION_OF_PROTEIN_LOCALIZATION_TO_CELL_PERIPHERY	-1.32	1.36	0	0	0	not significant
1738	DARWICHE_PAPILLOMA_RISK_LOW_UP	-1.32	1.3	0	0	0	not significant
1739	GO_CEREBRAL_CORTEX_DEVELOPMENT	-1.32	1.22	0	0	0	not significant
1740	GO_PEPTIDYL_TYROSINE_DEPHOSPHORYLATION	-1.32	1.21	0	0	0	not significant
1741	BILD_CTNNB1_ONCOGENIC_SIGNATURE	-1.32	1.2	0	0	0	not significant
1742	GO_TRANSCRIPTIONAL_REPRESSOR_COMPLEX	-1.32	1.18	0	0	0	not significant
1743	PEART_HDAC_PROLIFERATION_CLUSTER_DN	-1.32	1.16	0	0	0	not significant
1744	DAIRKEE_TERT_TARGETS_DN	-1.32	1.14	0	0	0	not significant
1745	AMIT_SERUM_RESPONSE_120_MCF10A	-1.32	1.12	0	0	0	not significant
1746	CHR5Q13	-1.32	1.07	0	0	0	not significant
1747	GO_MULTI_ORGANISM_BEHAVIOR	-1.32	1.02	0	0	0	not significant
1748	REACTOME_NEGATIVE_REGULATION_OF_FGFR4_SIGNALING	-1.32	0.94	0	0	0	not significant
1749	CHR16Q21	-1.32	0.93	0	0	0	not significant
1750	REACTOME_NEGATIVE_REGULATION_OF_FGFR1_SIGNALING	-1.32	0.93	0	0	0	not significant
1751	CHR6P24	-1.32	0.92	0	0	0	not significant
1752	GO_RIBONUCLEOPROTEIN_COMPLEX_DISASSEMBLY	-1.32	0.91	0	0	0	not significant
1753	REACTOME_GAP_JUNCTION_TRAFFICKING_AND_REGULATION	-1.32	0.91	0	0	0	not significant
1754	GO_POSITIVE_REGULATION_OF_RNA_SPLICING	-1.32	0.9	0	0	0	not significant
1755	GO_GROWTH_PLATE_CARTILAGE_DEVELOPMENT	-1.32	0.89	0	0	0	not significant
1756	GO_MRNA_TRANSCRIPTION	-1.32	0.88	0	0	0	not significant
1757	REACTOME_RHO_GTPASES_ACTIVATE_IQGAPS	-1.32	0.88	0	0	0	not significant
1758	GO_ESTABLISHMENT_OF_PROTEIN_LOCALIZATION_TO_CHROMATIN	-1.32	0.87	0	0	0	not significant
1759	GO_MODULATION_OF_EXCITATORY_POSTSYNAPTIC_POTENTIAL	-1.32	0.87	0	0	0	not significant
1760	GO_POSITIVE_REGULATION_OF_DENDRITIC_CELL_CYTOKINE_PRODUCTION	-1.32	0.86	0	0	0	not significant
1761	GO_CATALYTIC_ACTIVITY_ACTING_ON_A_RRNA	-1.32	0.85	0	0	0	not significant
1762	GO_POSITIVE_REGULATION_OF_MYELOID_LEUKOCYTE_CYTOKINE_PRODUCTION_I	-1.32	0.85	0	0	0	not significant
1763	GO_PROTEIN_K6_LINKED_UBIQUITINATION	-1.32	0.85	0	0	0	not significant
1764	REACTOME_NEGATIVE_REGULATION_OF_FGFR2_SIGNALING	-1.32	0.85	0	0	0	not significant
1765	GO_AT_DNA_BINDING	-1.32	0.84	0	0	0	not significant
1766	SEMENZA_HIF1_TARGETS	-1.32	0.84	0	0	0	not significant
1767	GO_MITOCHONDRIAL_TRNA_METHYLATION	-1.32	0.82	0	0	0	not significant
1768	GO_G_PROTEIN_COUPLED_GLUTAMATE_RECEPTOR_BINDING	-1.32	0.81	0	0	0	not significant
1769	SEMBA_FHIT_TARGETS_DN	-1.32	0.81	0	0	0	not significant
1770	GO_REGULATION_OF_MITOTIC_CENTROSOME_SEPARATION	-1.32	0.8	0	0	0	not significant
1771	GO_EUKARYOTIC_TRANSLATION_INITIATION_FACTOR_4F_COMPLEX	-1.32	0.78	0	0	0	not significant
1772	REACTOME_SHC1_EVENTS_IN_ERBB4_SIGNALING	-1.32	0.78	0	0	0	not significant
1773	GO_MUSCLE_ATROPHY	-1.32	0.76	0	0	0	not significant
1774	GO_NUCLEOLAR_RIBONUCLEASE_P_COMPLEX	-1.32	0.76	0	0	0	not significant
1775	GO_POSITIVE_REGULATION_OF_B_CELL_RECEPTOR_SIGNALING_PATHWAY	-1.32	0.74	0	0	0	not significant
1776	GO_PROTEIN_CONTAINING_COMPLEX_BINDING	-1.31	4	0	0	0	not significant
1777	GO_PROTEIN_DOMAIN_SPECIFIC_BINDING	-1.31	4	0	0	0	not significant
1778	GO_TRANSFERASE_ACTIVITY_TRANSFERRING_PHOSPHORUS_CONTAINING_GROUI	-1.31	4	0	0	0	not significant
1779	GO_PROTEIN_AUTOPHOSPHORYLATION	-1.31	1.68	0	0	0	not significant
1780	GO_REGULATION_OF_DNA_TEMPLATED_TRANSCRIPTION_IN_RESPONSE_TO_STRE	-1.31	1.59	0	0	0	not significant
1781	GO_CYTOSKELETON_DEPENDENT_CYTOKINESIS	-1.31	1.3	0	0	0	not significant
1782	ACEVEDO_LIVER_CANCER_WITH_H3K9ME3_DN	-1.31	1.26	0	0	0	not significant
1783	GO_ION_CHANNEL_BINDING	-1.31	1.2	0	0	0	not significant
1784	GO_PROTEIN_SUMOYLATION	-1.31	1.17	0	0	0	not significant
1785	REACTOME_RMTS_METHYLATE_HISTONE_ARGININES	-1.31	1.1	0	0	0	not significant
1786	GO_TRANSCRIPTION_PREINITIATION_COMPLEX_ASSEMBLY	-1.31	1.02	0	0	0	not significant
1787	GUTIERREZ_CHRONIC_LYMPHOCYTIC_LEUKEMIA_DN	-1.31	1	0	0	0	not significant
1788	PID_FOXM1_PATHWAY	-1.31	0.99	0	0	0	not significant
1789	GO_TELOMERIC_DNA_BINDING	-1.31	0.98	0	0	0	not significant
1790	REACTOME_MITOPHAGY	-1.31	0.98	0	0	0	not significant
1791	REACTOME_SIGNALING_BY_FGFR4	-1.31	0.98	0	0	0	not significant
1792	GO_2_OXOGLUTARATE_DEPENDENT_DIOXYGENASE_ACTIVITY	-1.31	0.97	0	0	0	not significant
1793	GO_AMIDE_TRANSMEMBRANE_TRANSPORTER_ACTIVITY	-1.31	0.96	0	0	0	not significant
1794	KEGG_OLFACTORY_TRANSDUCTION	-1.31	0.91	0	0	0	not significant
1795	GO_PROTEIN_REFOLDING	-1.31	0.9	0	0	0	not significant
1796	REACTOME_INTERLEUKIN_6_FAMILY_SIGNALING	-1.31	0.9	0	0	0	not significant
1797	GO_RIBONUCLEASE_P_ACTIVITY	-1.31	0.85	0	0	0	not significant
1798	BIOCARTA_RNA_PATHWAY	-1.31	0.81	0	0	0	not significant
1799	GO_HISTONE_METHYLTRANSFERASE_BINDING	-1.31	0.81	0	0	0	not significant
1800	BIOCARTA_NOS1_PATHWAY	-1.31	0.8	0	0	0	not significant
1801	GO_ATP_DEPENDENT_PEPTIDASE_ACTIVITY	-1.31	0.8	0	0	0	not significant
1802	GO_REGULATION_OF_MRNA_POLYADENYLATION	-1.31	0.8	0	0	0	not significant
1803	GO_TYPE_I_INTERFERON_SECRETION	-1.31	0.8	0	0	0	not significant
1804	GO_ENDOPLASMIC_RETICULUM_EXIT_SITE	-1.31	0.79	0	0	0	not significant
1805	GO_RIBOSOMAL_LARGE_SUBUNIT_BINDING	-1.31	0.79	0	0	0	not significant
1806	WANG_IMMORTALIZED_BY_HOXA9_AND_MEIS1_UP	-1.31	0.78	0	0	0	not significant
1807	GO_FEMALE_PRONUCLEUS	-1.31	0.77	0	0	0	not significant
1808	GO_ALKALI_METAL_ION_BINDING	-1.31	0.75	0	0	0	not significant
1809	GO_RIBONUCLEASE_MRP_COMPLEX	-1.31	0.75	0	0	0	not significant
1810	GO_STAT_FAMILY_PROTEIN_BINDING	-1.31	0.73	0	0	0	not significant
1811	GO_CHROMOSOME_ORGANIZATION	-1.30	4	0	0	0	not significant
1812	GO_HYDROLASE_ACTIVITY_ACTING_ON_ACID_ANHYDRIDES	-1.30	4	0	0	0	not significant
1813	GO_NEGATIVE_REGULATION_OF_CELLULAR_COMPONENT_ORGANIZATION	-1.30	4	0	0	0	not significant
1814	GO_REGULATION_OF_CELLULAR_PROTEIN_CATABOLIC_PROCESS	-1.30	1.56	0	0	0	not significant
1815	TGFB_UP.V1_UP	-1.30	1.3	0	0	0	not significant
1816	GO_HEAT_SHOCK_PROTEIN_BINDING	-1.30	1.28	0	0	0	not significant
1817	GO_PROTEIN_TARGETING_TO_MITOCHONDRION	-1.30	1.2	0	0	0	not significant
1818	CHR14Q32	-1.30	1.16	0	0	0	not significant
1819	FAELT_B_CLL_WITH_VH3_21_UP	-1.30	1.11	0	0	0	not significant
1820	GO_INTRACELLULAR_PROTEIN_TRANSMEMBRANE_TRANSPORT	-1.30	1.02	0	0	0	not significant
1821	GO_RNA_POLYADENYLATION	-1.30	1	0	0	0	not significant
1822	KORKOLA_EMBRYONAL_CARCINOMA_UP	-1.30	0.98	0	0	0	not significant
1823	REACTOME_SIGNALING_BY_FGFR3	-1.30	0.94	0	0	0	not significant
1824	REACTOME_SIGNALING_BY_FGFR1	-1.30	0.93	0	0	0	not significant
1825	STARK_HYPPOCAMPUS_22Q11_DELETION_UP	-1.30	0.93	0	0	0	not significant
1826	REACTOME_GENE_AND_PROTEIN_EXPRESSION_BY_JAK_STAT_SIGNALING_AFTER	-1.30	0.91	0	0	0	not significant
1827	KEGG_HEDGEHOG_SIGNALING_PATHWAY	-1.30	0.9	0	0	0	not significant
1828	AMIT_EGF_RESPONSE_120_MCF10A	-1.30	0.88	0	0	0	not significant
1829	GO_DYNEIN_COMPLEX_BINDING	-1.30	0.87	0	0	0	not significant
1830	CHR1Q41	-1.30	0.85	0	0	0	not significant
1831	KIM_HYPOXIA	-1.30	0.85	0	0	0	not significant
1832	GO_NEGATIVE_REGULATION_OF_CALCINEURIN_MEDIATED_SIGNALING	-1.30	0.83	0	0	0	not significant
1833	GO_ENUCLEATE_ERYTHROCYTE_DIFFERENTIATION	-1.30	0.82	0	0	0	not significant
1834	GO_METANEPHRIC_EPITHELIUM_DEVELOPMENT	-1.30	0.82	0	0	0	not significant
1835	GO_SODIUM_CHANNEL_REGULATOR_ACTIVITY	-1.30	0.82	0	0	0	not significant
1836	GO_FEMALE_MEIOSIS_CHROMOSOME_SEGREGATION	-1.30	0.8	0	0	0	not significant
1837	GO_RESPONSE_TO_IRON_III_ION	-1.30	0.8	0	0	0	not significant
1838	GO_CARDIOLIPIN_METABOLIC_PROCESS	-1.30	0.79	0	0	0	not significant
1839	REACTOME_ASSEMBLY_AND_CELL_SURFACE_PRESENTATION_OF_NMDA_RECEPT	-1.30	0.79	0	0	0	not significant
1840	SUZUKI_RESPONSE_TO_TSA	-1.30	0.79	0	0	0	not significant
1841	GO_DYNACTIN_BINDING	-1.30	0.78	0	0	0	not significant

1842	GO_NUCLEAR_PORE_CENTRAL_TRANSPORT_CHANNEL	-1.30	0.78	0	0	0	not significant
1843	YAMANAKA_GLIOBLASTOMA_SURVIVAL_UP	-1.30	0.78	0	0	0	not significant
1844	GO_CHEMOKINE_C_X_C_MOTIF_LIGAND_2_PRODUCTION	-1.30	0.77	0	0	0	not significant
1845	GO_PERICARDIUM_DEVELOPMENT	-1.30	0.77	0	0	0	not significant
1846	GO_NADH_METABOLIC_PROCESS	-1.30	0.76	0	0	0	not significant
1847	REACTOME_PROSTACYCLIN_SIGNALLING_THROUGH_PROSTACYCLIN_RECEPTOR	-1.30	0.75	0	0	0	not significant
1848	GO_DENDRITIC_GROWTH_CONE	-1.30	0.74	0	0	0	not significant
1849	GO_HOST_INTRACELLULAR_PART	-1.30	0.73	0	0	0	not significant
1850	GO_MIRNA_CATABOLIC_PROCESS	-1.30	0.72	0	0	0	not significant
1851	REACTOME_BINDING_OF_TCF_LEF:CTNNB1_TO_TARGET_GENE_PROMOTERS	-1.30	0.72	0	0	0	not significant
1852	FARMER_BREAST_CANCER_BASAL_VS_LULMINAL	-1.29	1.83	0	0	0	not significant
1853	REACTOME_REGULATION_OF_EXPRESSION_OF_SLITS_AND_ROBOS	-1.29	1.74	0	0	0	not significant
1854	REACTOME_ESR_MEDIATED_SIGNALING	-1.29	1.61	0	0	0	not significant
1855	ALCALAY_AML_BY_NPM1_LOCALIZATION_DN	-1.29	1.53	0	0	0	not significant
1856	GO_MITOCHONDRIAL_MEMBRANE_PART	-1.29	1.45	0	0	0	not significant
1857	SMITH_TERT_TARGETS_UP	-1.29	1.32	0	0	0	not significant
1858	LI_AMPLIFIED_IN_LUNG_CANCER	-1.29	1.26	0	0	0	not significant
1859	CHIARADONNA_NEOPLASTIC_TRANSFORMATION_CDC25_UP	-1.29	1.17	0	0	0	not significant
1860	FERRANDO_T_ALL_WITH_MLL_ENL_FUSION_DN	-1.29	1.17	0	0	0	not significant
1861	NIKOLSKY_MUTATED_AND_AMPLIFIED_IN_BREAST_CANCER	-1.29	1.02	0	0	0	not significant
1862	PUJANA_BREAST_CANCER_WITH_BRCA1_MUTATED_UP	-1.29	1.02	0	0	0	not significant
1863	CHR3Q29	-1.29	0.99	0	0	0	not significant
1864	GAZDA_DIAMOND_BLACKFAN_ANEMIA_ERYTHROID_UP	-1.29	0.95	0	0	0	not significant
1865	CHR1P31	-1.29	0.94	0	0	0	not significant
1866	JIANG_AGING_HYPOTHALAMUS_DN	-1.29	0.94	0	0	0	not significant
1867	GO_REGULATION_OF_LAMELLIPODIUM_ORGANIZATION	-1.29	0.9	0	0	0	not significant
1868	GO_POSITIVE_REGULATION_OF_PROTEIN_DEPHOSPHORYLATION	-1.29	0.87	0	0	0	not significant
1869	REACTOME_PERVASIVE_DEVELOPMENTAL_DISORDERS	-1.29	0.86	0	0	0	not significant
1870	GO_GLUTAMINE_FAMILY_AMINO_ACID_CATABOLIC_PROCESS	-1.29	0.84	0	0	0	not significant
1871	GO_NEGATIVE_REGULATION_OF_CELLULAR_RESPONSE_TO_INSULIN_STIMULUS	-1.29	0.84	0	0	0	not significant
1872	GO_RRNA_MODIFICATION	-1.29	0.83	0	0	0	not significant
1873	GO_POSITIVE_REGULATION_OF_PROTEIN_LOCALIZATION_TO_CENTROSOME	-1.29	0.82	0	0	0	not significant
1874	GO_REGULATION_OF_TYPE_I_INTERFERON_MEDIATED_SIGNALING_PATHWAY	-1.29	0.81	0	0	0	not significant
1875	GO_NEGATIVE_REGULATION_OF_TYROSINE_PHOSPHORYLATION_OF_STAT_PROT	-1.29	0.8	0	0	0	not significant
1876	GO_PROTEIN_KINASE_A_REGULATORY_SUBUNIT_BINDING	-1.29	0.8	0	0	0	not significant
1877	REACTOME_CREB1_PHOSPHORYLATION_THROUGH_THE_ACTIVATION_OF_ADENYL	-1.29	0.78	0	0	0	not significant
1878	GO_DNA_APURINIC_OR_APYRIMIDINIC_SITE_ENDONUCLEASE_ACTIVITY	-1.29	0.77	0	0	0	not significant
1879	IVANOV_MUTATED_IN_COLON_CANCER	-1.29	0.77	0	0	0	not significant
1880	GO_CLASS_I_DNA_APURINIC_OR_APYRIMIDINIC_SITE_ENDONUCLEASE_ACTIVITY	-1.29	0.75	0	0	0	not significant
1881	GO_NEGATIVE_REGULATION_OF_NITRIC_OXIDE_BIOSYNTHETIC_PROCESS	-1.29	0.74	0	0	0	not significant
1882	GO_NEGATIVE_REGULATION_OF_CELL_ADHESION_MEDIATED_BY_INTEGRIN	-1.29	0.72	0	0	0	not significant
1883	REACTOME_WAX_AND_PLASMALOGEN_BIOSYNTHESIS	-1.29	0.72	0	0	0	not significant
1884	SCHWAB_TARGETS_OF_BMYB_POLYMORPHIC_VARIANTS_UP	-1.29	0.72	0	0	0	not significant
1885	GO_RESPONSE_TO_INACTIVITY	-1.29	0.71	0	0	0	not significant
1886	SUZUKI_CTCFL_TARGETS_UP	-1.29	0.71	0	0	0	not significant
1887	GO_REGULATION_OF_VITAMIN_D_RECEPTOR_SIGNALING_PATHWAY	-1.29	0.68	0	0	0	not significant
1888	GO_CELLULAR_MACROMOLECULE_CATABOLIC_PROCESS	-1.28	4	0	0	0	not significant
1889	IVANOVA_HEMATOPOIESIS_EARLY_PROGENITOR	-1.28	1.85	0	0	0	not significant
1890	HALLMARK_GLYCOLYSIS	-1.28	1.67	0	0	0	not significant
1891	GO_CANONICAL_WNT_SIGNALING_PATHWAY	-1.28	1.57	0	0	0	not significant
1892	PUJANA_XPRSS_INT_NETWORK	-1.28	1.4	0	0	0	not significant
1893	BOYLAN_MULTIPLE_MYELOMA_C_D_UP	-1.28	1.35	0	0	0	not significant
1894	REACTOME_REGULATION_OF_TP53_ACTIVITY_THROUGH_PHOSPHORYLATION	-1.28	1.2	0	0	0	not significant
1895	GO_REGULATION_OF_SYNAPTIC_PLASTICITY	-1.28	1.18	0	0	0	not significant
1896	GO_PROTEIN_TRANSMEMBRANE_TRANSPORT	-1.28	1.08	0	0	0	not significant
1897	GO_MUSCLE_CELL_APOPTOTIC_PROCESS	-1.28	0.99	0	0	0	not significant
1898	GO_ADP_BINDING	-1.28	0.98	0	0	0	not significant
1899	GO_ANIMAL_ORGAN_REGENERATION	-1.28	0.95	0	0	0	not significant
1900	DORN_ADENOVIRUS_INFECTION_12HR_DN	-1.28	0.93	0	0	0	not significant
1901	GO_POSITIVE_REGULATION_OF_VIRAL_TRANSCRIPTION	-1.28	0.92	0	0	0	not significant
1902	GO_NEGATIVE_REGULATION_OF_REPRODUCTIVE_PROCESS	-1.28	0.89	0	0	0	not significant
1903	DELPUECH_FOXO3_TARGETS_DN	-1.28	0.88	0	0	0	not significant
1904	YOKOE_CANCER_TESTIS_ANTIGENS	-1.28	0.87	0	0	0	not significant
1905	PID_EPHB_FWD_PATHWAY	-1.28	0.85	0	0	0	not significant
1906	REACTOME_NEGATIVE_REGULATION_OF_FGFR3_SIGNALING	-1.28	0.85	0	0	0	not significant
1907	GO_MRNA_CLEAVAGE	-1.28	0.84	0	0	0	not significant
1908	GO_PROTEIN_PEPTIDYL_PROLYL_ISOMERIZATION	-1.28	0.84	0	0	0	not significant
1909	GO_ATP_BIOSYNTHETIC_PROCESS	-1.28	0.83	0	0	0	not significant
1910	WEST_ADRENOCORTICAL_TUMOR_MARKERS_UP	-1.28	0.83	0	0	0	not significant
1911	YAO_TEMPORAL_RESPONSE_TO_PROGESTERONE_CLUSTER_8	-1.28	0.83	0	0	0	not significant
1912	GO_A_BAND	-1.28	0.82	0	0	0	not significant
1913	REACTOME_ANTIGEN_ACTIVATES_B_CELL_RECEPTOR_BCR_LEADING_TO_GENER	-1.28	0.82	0	0	0	not significant
1914	GO_CELL_COMMUNICATION_BY_ELECTRICAL_COUPLING_INVOLVED_IN_CARDIAC_(	-1.28	0.81	0	0	0	not significant
1915	GO_EUCHROMATIN	-1.28	0.81	0	0	0	not significant
1916	GO_REGULATION_OF_LAMELLIPODIUM_ASSEMBLY	-1.28	0.81	0	0	0	not significant
1917	BIOCARTA_WNT_PATHWAY	-1.28	0.8	0	0	0	not significant
1918	GO_NEGATIVE_REGULATION_OF_NEUROLOGICAL_SYSTEM_PROCESS	-1.28	0.79	0	0	0	not significant
1919	REACTOME_SIGNALING_BY_FGFR3_FUSIONS_IN_CANCER	-1.28	0.78	0	0	0	not significant
1920	COLIN_PILOCYTIC_ASTROCYTOMA_VS_GLIOBLASTOMA_DN	-1.28	0.77	0	0	0	not significant
1921	GO_COPPER_ION_HOMEOSTASIS	-1.28	0.76	0	0	0	not significant
1922	GO_E_BOX_BINDING	-1.28	0.76	0	0	0	not significant
1923	GO_PHOSPHORYLATION_OF_RNA_POLYMERASE_II_C_TERMINAL_DOMAIN	-1.28	0.76	0	0	0	not significant
1924	GO_BEHAVIORAL_RESPONSE_TO_COCAINE	-1.28	0.75	0	0	0	not significant
1925	GO_POSITIVE_REGULATION_OF_SMOOTH_MUSCLE_CELL_APOPTOTIC_PROCESS	-1.28	0.75	0	0	0	not significant
1926	REACTOME_ATF6_ATF6_ALPHA_ACTIVATES_CHAPERONES	-1.28	0.75	0	0	0	not significant
1927	REACTOME_NOTCH3_ACTIVATION_AND_TRANSMISSION_OF_SIGNAL_TO_THE_NUCI	-1.28	0.75	0	0	0	not significant
1928	REACTOME_TRAF3_DEPENDENT_IRF_ACTIVATION_PATHWAY	-1.28	0.75	0	0	0	not significant
1929	GO_POSITIVE_REGULATION_OF_T_HELPER_2_CELL_DIFFERENTIATION	-1.28	0.74	0	0	0	not significant
1930	GO_ARRESTIN_FAMILY_PROTEIN_BINDING	-1.28	0.73	0	0	0	not significant
1931	GO_CARDIOLIPIN_ACYL_CHAIN_REMODELING	-1.28	0.73	0	0	0	not significant
1932	GO_PROTEIN_COFACTOR_LINKAGE	-1.28	0.73	0	0	0	not significant
1933	REACTOME_G2_M_DNA_REPLICATION_CHECKPOINT	-1.28	0.73	0	0	0	not significant
1934	REACTOME_SHC_MEDIATED_CASCADE:FGFR4	-1.28	0.73	0	0	0	not significant
1935	GO_MULTI_ORGANISM_CELLULAR_LOCALIZATION	-1.28	0.72	0	0	0	not significant
1936	GO_NEGATIVE_REGULATION_OF_HIPPO_SIGNALING	-1.28	0.7	0	0	0	not significant
1937	GO_INSULIN_RECEPTOR_SUBSTRATE_BINDING	-1.28	0.69	0	0	0	not significant
1938	GO_NEGATIVE_REGULATION_OF_NUCLEASE_ACTIVITY	-1.28	0.69	0	0	0	not significant
1939	GO_RESPONSE_TO_MUSCLE_INACTIVITY	-1.28	0.69	0	0	0	not significant
1940	GO_REGULATION_OF_DNA_TOPOISOMERASE_ATP_HYDROLYZING_ACTIVITY	-1.28	0.68	0	0	0	not significant
1941	GO_HISTONE_METHYLTRANSFERASE_ACTIVITY_H3_K36_SPECIFIC_	-1.28	0.67	0	0	0	not significant
1942	GO_N_TERMINAL_PROTEIN_ACETYLTRANSFERASE_COMPLEX	-1.28	0.67	0	0	0	not significant
1943	GO_REGULATION_OF_PLATELET_AGGREGATION	-1.28	0.67	0	0	0	not significant
1944	BIOCARTA_CACAM_PATHWAY	-1.28	0.65	0	0	0	not significant
1945	GO_ESTABLISHMENT_OF_PROTEIN_LOCALIZATION_TO_ORGANELLE	-1.27	4	0	0	0	not significant
1946	GO_TISSUE_MORPHOGENESIS	-1.27	4	0	0	0	not significant
1947	KIM_WT1_TARGETS_DN	-1.27	2.16	0	0	0	not significant
1948	FLECHNER_BIOPSY_KIDNEY_TRANSPLANT_OK_VS_DONOR_UP	-1.27	2.13	0	0	0	not significant
1949	GO_PEPTIDE_BIOSYNTHETIC_PROCESS	-1.27	1.94	0	0	0	not significant
1950	DUTERTRE_ESTRADIOL_RESPONSE_24HR_UP	-1.27	1.87	0	0	0	not significant

1951	GO_ORGANELLE_LOCALIZATION	-1.27	1.7	0	0	0	not significant
1952	GO_NEGATIVE_REGULATION_OF_CELLULAR_CATABOLIC_PROCESS	-1.27	1.57	0	0	0	not significant
1953	GO_ELECTRON_TRANSPORT_CHAIN	-1.27	1.21	0	0	0	not significant
1954	DAZARD_RESPONSE_TO_UV_SCC_DN	-1.27	1.11	0	0	0	not significant
1955	BROWN_MYELOID_CELL_DEVELOPMENT_DN	-1.27	1.07	0	0	0	not significant
1956	GO_PROTEIN_TYROSINE_PHOSPHATASE_ACTIVITY	-1.27	1.06	0	0	0	not significant
1957	GO_CHROMATIN_DNA_BINDING	-1.27	1.05	0	0	0	not significant
1958	GO_REGULATION_OF_DNA_BIOSYNTHETIC_PROCESS	-1.27	1.05	0	0	0	not significant
1959	REACTOME_DNA_DOUBLE_STRAND_BREAK_RESPONSE	-1.27	1.04	0	0	0	not significant
1960	REACTOME_PROTEIN_UBIQUITINATION	-1.27	1.02	0	0	0	not significant
1961	DACOSTA_UV_RESPONSE_VIA_ERCC3_TTD_DN	-1.27	1.01	0	0	0	not significant
1962	GO_CARBON_OXYGEN_LYASE_ACTIVITY	-1.27	0.95	0	0	0	not significant
1963	DAVICIONI_TARGETS_OF_PAX_FOXO1_FUSIONS_DN	-1.27	0.94	0	0	0	not significant
1964	FRASOR_RESPONSE_TO_SERM_OR_FULVESTRANT_DN	-1.27	0.94	0	0	0	not significant
1965	GO_POSITIVE_REGULATION_OF_VIRAL_GENOME_REPLICATION	-1.27	0.93	0	0	0	not significant
1966	GO_POSITIVE_REGULATION_OF_ATPASE_ACTIVITY	-1.27	0.92	0	0	0	not significant
1967	REACTOME_REGULATION_OF_INSULIN_SECRETION	-1.27	0.87	0	0	0	not significant
1968	BIOCARTA_ERK_PATHWAY	-1.27	0.85	0	0	0	not significant
1969	BIOCARTA_IGF1R_PATHWAY	-1.27	0.85	0	0	0	not significant
1970	GO_RESPONSE_TO_CATECHOLAMINE	-1.27	0.84	0	0	0	not significant
1971	GO_NEGATIVE_REGULATION_OF_TELOMERE_MAINTENANCE_VIA_TELOMERE_LENG	-1.27	0.81	0	0	0	not significant
1972	MACLACHLAN_BRCA1_TARGETS_UP	-1.27	0.81	0	0	0	not significant
1973	CHR11P13	-1.27	0.79	0	0	0	not significant
1974	REACTOME_TRIGLYCERIDE_METABOLISM	-1.27	0.79	0	0	0	not significant
1975	STEIN_ESRRA_TARGETS_RESPONSIVE_TO_ESTROGEN_UP	-1.27	0.79	0	0	0	not significant
1976	SILIGAN_BOUND_BY_EWS_FLT1_FUSION	-1.27	0.78	0	0	0	not significant
1977	GO_AXONAL_GROWTH_CONE	-1.27	0.77	0	0	0	not significant
1978	GO_TRANSCRIPTION_INITIATION_FROM_RNA_POLYMERASE_III_PROMOTER	-1.27	0.75	0	0	0	not significant
1979	HERNANDEZ_ABERRANT_MITOSIS_BY_DOCETACEL_4NM_DN	-1.27	0.75	0	0	0	not significant
1980	GO_1_PHOSPHATIDYLINOSITOL_3_KINASE_REGULATOR_ACTIVITY	-1.27	0.74	0	0	0	not significant
1981	GO_POSITIVE_REGULATION_OF_PROTEIN_MONOUBIQUITINATION	-1.27	0.74	0	0	0	not significant
1982	FINETTI_BREAST_CANCERS_KINOME_GRAY	-1.27	0.73	0	0	0	not significant
1983	GO_EMBRYONIC_HINDLIMB_MORPHOGENESIS	-1.27	0.73	0	0	0	not significant
1984	GO_RESPONSE_TO_IRON_ION	-1.27	0.73	0	0	0	not significant
1985	GO_RESPONSE_TO_PROSTAGLANDIN_E	-1.27	0.73	0	0	0	not significant
1986	MACLACHLAN_BRCA1_TARGETS_DN	-1.27	0.73	0	0	0	not significant
1987	GO_SMOOTH_MUSCLE_CELL_APOPTOTIC_PROCESS	-1.27	0.72	0	0	0	not significant
1988	GO_MITOCHONDRIAL_OUTER_MEMBRANE_TRANSLOCASE_COMPLEX	-1.27	0.71	0	0	0	not significant
1989	GO_REGULATION_OF_GLIAL_CELL_APOPTOTIC_PROCESS	-1.27	0.7	0	0	0	not significant
1990	YAO_TEMPORAL_RESPONSE_TO_PROGESTERONE_CLUSTER_4	-1.27	0.7	0	0	0	not significant
1991	GO_CELLULAR_RESPONSE_TO_INSULIN_LIKE_GROWTH_FACTOR_STIMULUS	-1.27	0.69	0	0	0	not significant
1992	GO_STRESS_RESPONSE_TO_METAL_ION	-1.27	0.69	0	0	0	not significant
1993	REACTOME_ACTIVATED_NTRK3_SIGNALS_THROUGH_RAS	-1.27	0.69	0	0	0	not significant
1994	REACTOME_SHC1_EVENTS_IN_EGFR_SIGNALING	-1.27	0.68	0	0	0	not significant
1995	GO_REGULATION_OF_CYTOSKELETON_ORGANIZATION	-1.26	2.18	0	0	0	not significant
1996	GO_REGULATION_OF_DNA_METABOLIC_PROCESS	-1.26	1.73	0	0	0	not significant
1997	GO_CYTOPLASMIC_REGION	-1.26	1.57	0	0	0	not significant
1998	GO_TRANSLATIONAL_INITIATION	-1.26	1.33	0	0	0	not significant
1999	GO_TRANSLATIONAL_ELONGATION	-1.26	1.26	0	0	0	not significant
2000	REACTOME_FACTORS_INVOLVED_IN_MEGAKARYOCYTE_DEVELOPMENT_AND_PLA	-1.26	1.23	0	0	0	not significant
2001	GO_HISTONE_DEACETYLASE_BINDING	-1.26	1.21	0	0	0	not significant
2002	YAMAZAKI_TCEB3_TARGETS_DN	-1.26	1.21	0	0	0	not significant
2003	GO_MESENCHYMAL_CELL_DIFFERENTIATION	-1.26	1.17	0	0	0	not significant
2004	MILI_PSEUDOPODIA_CHEMOTAXIS_UP	-1.26	1.15	0	0	0	not significant
2005	CAIRO_LIVER_DEVELOPMENT_UP	-1.26	1.14	0	0	0	not significant
2006	LABBE_WNT3A_TARGETS_UP	-1.26	1.11	0	0	0	not significant
2007	CHR1Q42	-1.26	1.08	0	0	0	not significant
2008	GO_CARDIAC_MUSCLE_TISSUE_DEVELOPMENT	-1.26	1.07	0	0	0	not significant
2009	REACTOME_SIGNALING_BY_TGF_BETA_RECEPTOR_COMPLEX	-1.26	1	0	0	0	not significant
2010	REACTOME_TRANSCRIPTIONAL_ACTIVITY_OF_SMAD2_SMAD3:SMAD4_HETEROTRIM	-1.26	0.99	0	0	0	not significant
2011	PID_P53_REGULATION_PATHWAY	-1.26	0.96	0	0	0	not significant
2012	GO_INTRINSIC_COMPONENT_OF_MITOCHONDRIAL_MEMBRANE	-1.26	0.95	0	0	0	not significant
2013	GO_LAMELLIPODIUM_ASSEMBLY	-1.26	0.91	0	0	0	not significant
2014	DUAN_PRDM5_TARGETS	-1.26	0.9	0	0	0	not significant
2015	GO_MEDIATOR_COMPLEX	-1.26	0.89	0	0	0	not significant
2016	GO_ATPASE_REGULATOR_ACTIVITY	-1.26	0.87	0	0	0	not significant
2017	PID_TRKR_PATHWAY	-1.26	0.86	0	0	0	not significant
2018	GO_POSITIVE_REGULATION_OF_REPRODUCTIVE_PROCESS	-1.26	0.84	0	0	0	not significant
2019	GO_RIBONUCLEOSIDE_TRIPHOSPHATE_BIOSYNTHETIC_PROCESS	-1.26	0.84	0	0	0	not significant
2020	GO_NEGATIVE_REGULATION_OF_MITOCHONDRION_ORGANIZATION	-1.26	0.82	0	0	0	not significant
2021	CORDENONSI_YAP_CONSERVED_SIGNATURE	-1.26	0.81	0	0	0	not significant
2022	REACTOME_DARPP_32_EVENTS	-1.26	0.81	0	0	0	not significant
2023	GO_PROTEIN_DESTABILIZATION	-1.26	0.8	0	0	0	not significant
2024	REACTOME_AQUAPORIN_MEDIATED_TRANSPORT	-1.26	0.8	0	0	0	not significant
2025	REACTOME_TP53_REGULATES_TRANSCRIPTION_OF_GENES_INVOLVED_IN_G2_CE	-1.26	0.8	0	0	0	not significant
2026	SCHLINGEMANN_SKIN_CARCINOGENESIS_TPA_UP	-1.26	0.79	0	0	0	not significant
2027	GO_NUCLEOBASE_CONTAINING_SMALL_MOLECULE_INTERCONVERSION	-1.26	0.78	0	0	0	not significant
2028	GO_CELLULAR_RESPONSE_TO_COPPER_ION	-1.26	0.77	0	0	0	not significant
2029	GO_CHAPERONE_MEDIATED_PROTEIN_COMPLEX_ASSEMBLY	-1.26	0.75	0	0	0	not significant
2030	GO_COPII_VESICLE_COAT	-1.26	0.75	0	0	0	not significant
2031	LI_CYTIDINE_ANALOGS_CYCTOTOXICITY	-1.26	0.75	0	0	0	not significant
2032	STAMBOLSKY_RESPONSE_TO_VITAMIN_D3_DN	-1.26	0.75	0	0	0	not significant
2033	BIOCARTA_DNAFRAGMENT_PATHWAY	-1.26	0.73	0	0	0	not significant
2034	REACTOME_NEURODEGENERATIVE_DISEASES	-1.26	0.73	0	0	0	not significant
2035	GO_ER_OVERLOAD_RESPONSE	-1.26	0.72	0	0	0	not significant
2036	GO_SIN3_TYPE_COMPLEX	-1.26	0.72	0	0	0	not significant
2037	REACTOME_INWARDLY_RECTIFYING_KPLUS_CHANNELS	-1.26	0.72	0	0	0	not significant
2038	GO_PEPTIDYL_ARGININE_MODIFICATION	-1.26	0.71	0	0	0	not significant
2039	GO_LEWY_BODY	-1.26	0.7	0	0	0	not significant
2040	GO_NUCLEAR_PORE_OUTER_RING	-1.26	0.7	0	0	0	not significant
2041	GO_GLUCOSE_6_PHOSPHATE_METABOLIC_PROCESS	-1.26	0.69	0	0	0	not significant
2042	GO_REGULATION_OF_GOLGI_TO_PLASMA_MEMBRANE_PROTEIN_TRANSPORT	-1.26	0.69	0	0	0	not significant
2043	REACTOME_ATF6_ATF6_ALPHA_ACTIVATES_CHAPERONE_GENES	-1.26	0.69	0	0	0	not significant
2044	GO_ACROSOME_ASSEMBLY	-1.26	0.68	0	0	0	not significant
2045	GO_ACTIVATION_INDUCED_CELL_DEATH_OF_T_CELLS	-1.26	0.68	0	0	0	not significant
2046	GO_CELLULAR_RESPONSE_TO_IRON_ION	-1.26	0.67	0	0	0	not significant
2047	GO_MRNA_TRANSCRIPTION_BY_RNA_POLYMERASE_II	-1.26	0.67	0	0	0	not significant
2048	GO_REGULATION_OF_TRIGLYCERIDE_METABOLIC_PROCESS	-1.26	0.67	0	0	0	not significant
2049	GO_TROPHOBLAST_CELL_MIGRATION	-1.26	0.67	0	0	0	not significant
2050	REACTOME_PI_3K_CASCADE:FGFR1	-1.26	0.66	0	0	0	not significant
2051	GO_POLYADENYLATION_DEPENDENT_SNORNA_3_END_PROCESSING	-1.26	0.64	0	0	0	not significant
2052	GO_CELLULAR_PROTEIN_CATABOLIC_PROCESS	-1.25	4	0	0	0	not significant
2053	GO_ENZYME_REGULATOR_ACTIVITY	-1.25	4	0	0	0	not significant
2054	GO_REGULATION_OF_ORGANELLE_ORGANIZATION	-1.25	4	0	0	0	not significant
2055	LINDGREN_BLADDER_CANCER_CLUSTER_1_DN	-1.25	2.24	0	0	0	not significant
2056	KIM_ALL_DISORDERS_CALB1_CORR_UP	-1.25	2.06	0	0	0	not significant
2057	CREIGHTON_ENDOCRINE_THERAPY_RESISTANCE_1	-1.25	1.7	0	0	0	not significant
2058	GO_MITOCHONDRIAL_MATRIX	-1.25	1.64	0	0	0	not significant
2059	GO_REGULATION_OF_CELLULAR_AMIDE_METABOLIC_PROCESS	-1.25	1.56	0	0	0	not significant

2060	SHEPARD_CRUSH_AND_BURN_MUTANT_UP	-1.25	1.46	0	0	0	not significant
2061	GO_GTPASE_ACTIVITY	-1.25	1.45	0	0	0	not significant
2062	GO_REGULATION_OF_PROTEIN_MODIFICATION_BY_SMALL_PROTEIN_CONJUGATIO	-1.25	1.39	0	0	0	not significant
2063	BROWNE_HCMV_INFECTION_18HR_UP	-1.25	1.35	0	0	0	not significant
2064	GO_PROTEIN_LOCALIZATION_TO_MITOCHONDRION	-1.25	1.27	0	0	0	not significant
2065	REACTOME_TRANSPORT_TO_THE_GOLGI_AND_SUBSEQUENT_MODIFICATION	-1.25	1.21	0	0	0	not significant
2066	YAO_TEMPORAL_RESPONSE_TO_PROGESTERONE_CLUSTER_14	-1.25	1.2	0	0	0	not significant
2067	GO_REGULATION_OF_GENERATION_OF_PRECURSOR_METABOLITES_AND_ENERG	-1.25	1.18	0	0	0	not significant
2068	GO_OXIDATIVE_PHOSPHORYLATION	-1.25	1.17	0	0	0	not significant
2069	COLDREN_GEFITINIB_RESISTANCE_UP	-1.25	1.02	0	0	0	not significant
2070	GO_MODIFICATION_OF_MORPHOLOGY_OR_PHYSIOLOGY_OF_OTHER_ORGANISM	-1.25	1	0	0	0	not significant
2071	JI_RESPONSE_TO_FSH_DN	-1.25	0.92	0	0	0	not significant
2072	KEGG_GLIOMA	-1.25	0.92	0	0	0	not significant
2073	KEGG_TIGHT_JUNCTION	-1.25	0.92	0	0	0	not significant
2074	CERVERA_SDHB_TARGETS_2	-1.25	0.91	0	0	0	not significant
2075	GO_MUSCLE_ORGAN_MORPHOGENESIS	-1.25	0.89	0	0	0	not significant
2076	AMIT_EGF_RESPONSE_480_MCF10A	-1.25	0.88	0	0	0	not significant
2077	AUNG_GASTRIC_CANCER	-1.25	0.88	0	0	0	not significant
2078	REACTOME_ASSOCIATION_OF_TRIC_CCT_WITH_TARGET_PROTEINS_DURING_BIOS	-1.25	0.85	0	0	0	not significant
2079	CAMPS_COLON_CANCER_COPY_NUMBER_DN	-1.25	0.84	0	0	0	not significant
2080	GO_TRANSLATION_INITIATION_FACTOR_BINDING	-1.25	0.84	0	0	0	not significant
2081	GO_CENTRAL_NERVOUS_SYSTEM_NEURON_DEVELOPMENT	-1.25	0.83	0	0	0	not significant
2082	GO_FACE_DEVELOPMENT	-1.25	0.81	0	0	0	not significant
2083	GROSS_HIF1A_TARGETS_DN	-1.25	0.81	0	0	0	not significant
2084	WIERENGA_PML_INTERACTOME	-1.25	0.81	0	0	0	not significant
2085	GO_REGULATION_OF_CELL_AGING	-1.25	0.8	0	0	0	not significant
2086	CHR2Q21	-1.25	0.78	0	0	0	not significant
2087	PID_NEPHRIN_NEPH1_PATHWAY	-1.25	0.78	0	0	0	not significant
2088	GO_ORGANELLAR_SMALL_RIBOSOMAL_SUBUNIT	-1.25	0.77	0	0	0	not significant
2089	PID_THROMBIN_PAR1_PATHWAY	-1.25	0.77	0	0	0	not significant
2090	KAPOSI_LIVER_CANCER_MET_UP	-1.25	0.76	0	0	0	not significant
2091	GO_NUCLEAR_EUCHROMATIN	-1.25	0.74	0	0	0	not significant
2092	GO_POSITIVE_REGULATION_OF_ORGAN_GROWTH	-1.25	0.74	0	0	0	not significant
2093	PID_AJDISS_2PATHWAY	-1.25	0.74	0	0	0	not significant
2094	GO_GLUTATHIONE_DISULFIDE_OXIDOREDUCTASE_ACTIVITY	-1.25	0.72	0	0	0	not significant
2095	GO_NEGATIVE_REGULATION_OF_TELOMERE_MAINTENANCE_VIA_TELOMERASE	-1.25	0.72	0	0	0	not significant
2096	REACTOME_FORMATION_OF_SENESCENCE_ASSOCIATED_HETEROCHROMATIN_FC	-1.25	0.72	0	0	0	not significant
2097	GO_POSITIVE_REGULATION_OF_EPIDERMAL_CELL_DIFFERENTIATION	-1.25	0.7	0	0	0	not significant
2098	GO_POSITIVE_REGULATION_OF_MRNA_SPLICING_VIA_SPLICEOSOME	-1.25	0.7	0	0	0	not significant
2099	GO_RESPONSE_TO_COCAINE	-1.25	0.7	0	0	0	not significant
2100	GO_U4_SNRNP	-1.25	0.7	0	0	0	not significant
2101	GO_REGULATION_OF_ISOMERASE_ACTIVITY	-1.25	0.69	0	0	0	not significant
2102	REACTOME_TRAFFICKING_OF_GLUR2_CONTAINING_AMPA_RECEPTORS	-1.25	0.69	0	0	0	not significant
2103	LOPEZ_MESOTHELIOMA_SURVIVAL_WORST_VS_BEST_UP	-1.25	0.68	0	0	0	not significant
2104	REACTOME_G_PROTEIN_GATED_POTASSIUM_CHANNELS	-1.25	0.68	0	0	0	not significant
2105	REACTOME_SYNTHESIS_OF_PIPS_AT_THE_LATE_ENDOSOME_MEMBRANE	-1.25	0.68	0	0	0	not significant
2106	CHR2Q13	-1.25	0.67	0	0	0	not significant
2107	GO_U7_SNRNP	-1.25	0.67	0	0	0	not significant
2108	REACTOME_DNA_DAMAGE_REVERSAL	-1.25	0.67	0	0	0	not significant
2109	GO_5S_RRNA_BINDING	-1.25	0.66	0	0	0	not significant
2110	GO_FOREBRAIN_NEUROBLAST_DIVISION	-1.25	0.66	0	0	0	not significant
2111	GO_INTERLEUKIN_18_PRODUCTION	-1.25	0.66	0	0	0	not significant
2112	IVANOVA_HEMATOPOIESIS_STEM_CELL_SHORT_TERM	-1.25	0.66	0	0	0	not significant
2113	GO_OXIDOREDUCTASE_ACTIVITY_ACTING_ON_A_SULFUR_GROUP_OF_DONORS_N	-1.25	0.65	0	0	0	not significant
2114	REACTOME_PI_3K_CASCADE:FGFR4	-1.25	0.65	0	0	0	not significant
2115	REACTOME_SYNTHESIS_OF_PYROPHOSPHATES_IN_THE_CYTOSOL	-1.25	0.64	0	0	0	not significant
2116	GO_CHEMOKINE_C_C_MOTIF_LIGAND_2_SECRETION	-1.25	0.63	0	0	0	not significant
2117	GO_GLYCEROL_ETHER_BIOSYNTHETIC_PROCESS	-1.25	0.62	0	0	0	not significant
2118	GO_P_P_BOND_HYDROLYSIS_DRIVEN_PROTEIN_TRANSMEMBRANE_TRANSPORTEF	-1.25	0.61	0	0	0	not significant
2119	GO_PROTEIN_MODIFICATION_BY_SMALL_PROTEIN_CONJUGATION	-1.24	4	0	0	0	not significant
2120	GO_PROTEIN_MODIFICATION_BY_SMALL_PROTEIN_CONJUGATION_OR_REMOVAL	-1.24	4	0	0	0	not significant
2121	GO_NUCLEAR_SPECK	-1.24	2.22	0	0	0	not significant
2122	GO_REGULATION_OF_CELLULAR_CATABOLIC_PROCESS	-1.24	2	0	0	0	not significant
2123	GO_POSITIVE_REGULATION_OF_TRANSFERASE_ACTIVITY	-1.24	1.8	0	0	0	not significant
2124	GO_REGULATION_OF_PROTEIN_CATABOLIC_PROCESS	-1.24	1.57	0	0	0	not significant
2125	GAL_LEUKEMIC_STEM_CELL_DN	-1.24	1.34	0	0	0	not significant
2126	GO_TELENCEPHALON_DEVELOPMENT	-1.24	1.25	0	0	0	not significant
2127	REACTOME_INFLUENZA_INFECTION	-1.24	1.24	0	0	0	not significant
2128	GO_CULLIN_RING_UBIQUITIN_LIGASE_COMPLEX	-1.24	1.14	0	0	0	not significant
2129	KEGG_INSULIN_SIGNALING_PATHWAY	-1.24	1.12	0	0	0	not significant
2130	HIRSCH_CELLULAR_TRANSFORMATION_SIGNATURE_DN	-1.24	1.01	0	0	0	not significant
2131	LINDGREN_BLADDER_CANCER_WITH_LOH_IN_CHR9Q	-1.24	1	0	0	0	not significant
2132	KEGG_VASCULAR_SMOOTH_MUSCLE_CONTRACTION	-1.24	0.98	0	0	0	not significant
2133	KOKKINAKIS_METHIONINE_DEPRIVATION_96HR_DN	-1.24	0.95	0	0	0	not significant
2134	GO_PLATELET_DERIVED_GROWTH_FACTOR_RECEPTOR_SIGNALING_PATHWAY	-1.24	0.88	0	0	0	not significant
2135	GO_MITOTIC_CYTOKINESIS	-1.24	0.87	0	0	0	not significant
2136	GO_AXIS_SPECIFICATION	-1.24	0.86	0	0	0	not significant
2137	ZHANG_RESPONSE_TO_CANTHARIDIN_DN	-1.24	0.85	0	0	0	not significant
2138	GO_ENDONUCLEASE_ACTIVITY_ACTIVE_WITH_EITHER_RIBO_OR_DEOXYRIBONUCLI	-1.24	0.84	0	0	0	not significant
2139	REACTOME_CYTOSOLIC_SENSORS_OF_PATHOGEN_ASSOCIATED_DNA	-1.24	0.84	0	0	0	not significant
2140	GO_POSITIVE_REGULATION_OF_TELOMERE_MAINTENANCE_VIA_TELOMERE_LENG	-1.24	0.82	0	0	0	not significant
2141	GO_PYRIMIDINE_NUCLEOTIDE_BIOSYNTHETIC_PROCESS	-1.24	0.8	0	0	0	not significant
2142	TOMLINS_PROSTATE_CANCER_DN	-1.24	0.8	0	0	0	not significant
2143	GO_NEGATIVE_REGULATION_OF_PROTEIN_LOCALIZATION_TO_CELL_PERIPHERY	-1.24	0.78	0	0	0	not significant
2144	AMIT_SERUM_RESPONSE_480_MCF10A	-1.24	0.76	0	0	0	not significant
2145	REACTOME_VASOPRESSIN_REGULATES_RENAL_WATER_HOMEOSTASIS_VIA_AQUA	-1.24	0.76	0	0	0	not significant
2146	GO_RIBOSOMAL_SMALL_SUBUNIT_ASSEMBLY	-1.24	0.75	0	0	0	not significant
2147	JAZAG_TGFB1_SIGNALING_DN	-1.24	0.75	0	0	0	not significant
2148	REACTOME_SUMOYLATION_OF_INTRACELLULAR_RECEPTORS	-1.24	0.73	0	0	0	not significant
2149	GO_POLARIZED_EPITHELIAL_CELL_DIFFERENTIATION	-1.24	0.72	0	0	0	not significant
2150	HOFMANN_MYELODYSPLASTIC_SYNDROM_LOW_RISK_DN	-1.24	0.72	0	0	0	not significant
2151	GO_HINDLIMB_MORPHOGENESIS	-1.24	0.71	0	0	0	not significant
2152	GO_METANEPHROS_MORPHOGENESIS	-1.24	0.71	0	0	0	not significant
2153	AMIT_SERUM_RESPONSE_20_MCF10A	-1.24	0.69	0	0	0	not significant
2154	GO_MITOCHONDRION_MORPHOGENESIS	-1.24	0.69	0	0	0	not significant
2155	GO_REGULATION_OF_PSEUDOPODIUM_ASSEMBLY	-1.24	0.69	0	0	0	not significant
2156	GO_REPRODUCTIVE_BEHAVIOR	-1.24	0.69	0	0	0	not significant
2157	GO_RESPONSE_TO_PROSTAGLANDIN	-1.24	0.69	0	0	0	not significant
2158	KYNG_ENVIRONMENTAL_STRESS_RESPONSE_DN	-1.24	0.69	0	0	0	not significant
2159	REACTOME_ESTROGEN_STIMULATED_SIGNALING_THROUGH_PRKCZ	-1.24	0.69	0	0	0	not significant
2160	REACTOME_G_PROTEIN_ACTIVATION	-1.24	0.69	0	0	0	not significant
2161	GO_PROTEIN_KINASE_B_BINDING	-1.24	0.68	0	0	0	not significant
2162	GO_AMINOACYL_TRNA_EDITING_ACTIVITY	-1.24	0.67	0	0	0	not significant
2163	GO_PEPTIDE_HORMONE_RECEPTOR_BINDING	-1.24	0.67	0	0	0	not significant
2164	GO_MITOCHONDRIAL_ELECTRON_TRANSPORT_UBIQUINOL_TO_CYTOCHROME_C	-1.24	0.66	0	0	0	not significant
2165	GO_REGULATION_OF_PLASMA_MEMBRANE_ORGANIZATION	-1.24	0.66	0	0	0	not significant
2166	GO_FASCIA_ADHERENS	-1.24	0.65	0	0	0	not significant
2167	GO_LOW_DENSITY_LIPOPROTEIN_PARTICLE_RECEPTOR_BINDING	-1.24	0.65	0	0	0	not significant
2168	GO_MITOCHONDRIAL_RNA_CATABOLIC_PROCESS	-1.24	0.65	0	0	0	not significant

2169	GO_NEGATIVE_REGULATION_OF_DENDRITIC_SPINE_DEVELOPMENT	-1.24	0.65	0	0	0	not significant
2170	REACTOME_LRR_FLII_INTERACTING_PROTEIN_1_LRRFIP1_ACTIVATES_TYPE_I_IFN	-1.24	0.65	0	0	0	not significant
2171	REACTOME_RHO_GTPASES_ACTIVATE_KTN1	-1.24	0.65	0	0	0	not significant
2172	REACTOME_SHC_MEDIATED_CASCADE:FGFR2	-1.24	0.65	0	0	0	not significant
2173	REACTOME_SLBP_DEPENDENT_PROCESSING_OF_REPLICATION_DEPENDENT_HIST	-1.24	0.65	0	0	0	not significant
2174	GO_BENZENE_CONTAINING_COMPOUND_METABOLIC_PROCESS	-1.24	0.64	0	0	0	not significant
2175	GO_SERINE_PHOSPHORYLATION_OF_STAT_PROTEIN	-1.24	0.64	0	0	0	not significant
2176	GO_NEGATIVE_REGULATION_OF_PLATELET_ACTIVATION	-1.24	0.63	0	0	0	not significant
2177	GO_NUCLEAR_PORE_NUCLEAR_BASKET	-1.24	0.63	0	0	0	not significant
2178	GO_POSITIVE_REGULATION_OF_MITOCHONDRIAL_MEMBRANE_POTENTIAL	-1.24	0.63	0	0	0	not significant
2179	REACTOME_SHC_MEDIATED_CASCADE:FGFR3	-1.24	0.62	0	0	0	not significant
2180	SCHMAHL_PDGF_SIGNALING	-1.24	0.62	0	0	0	not significant
2181	GO_CELLULAR_RESPONSE_TO_NICOTINE	-1.24	0.6	0	0	0	not significant
2182	GO_POSITIVE_REGULATION_OF_FIBROBLAST_GROWTH_FACTOR_RECEPTOR_SIG	-1.24	0.6	0	0	0	not significant
2183	GO_CLATHRIN_LIGHT_CHAIN_BINDING	-1.24	0.58	0	0	0	not significant
2184	GO_CYTOSKELETAL_PROTEIN_BINDING	-1.23	4	0	0	0	not significant
2185	GRAESSMANN_RESPONSE_TO_MC_AND_DOXORUBICIN_DN	-1.23	1.86	0	0	0	not significant
2186	GO_REGULATION_OF_CATABOLIC_PROCESS	-1.23	1.8	0	0	0	not significant
2187	GO_REGULATION_OF_WNT_SIGNALING_PATHWAY	-1.23	1.52	0	0	0	not significant
2188	GO_CELLULAR_KETONE_METABOLIC_PROCESS	-1.23	1.39	0	0	0	not significant
2189	SHETH_LIVER_CANCER_VS_TXNIP_LOSS_PAM1	-1.23	1.31	0	0	0	not significant
2190	MCBRYAN_PUBERTAL_BREAST_6_7WK_UP	-1.23	1.19	0	0	0	not significant
2191	GO_MESENCHYME_DEVELOPMENT	-1.23	1.14	0	0	0	not significant
2192	GO_OSTEOBLAST_DIFFERENTIATION	-1.23	1.08	0	0	0	not significant
2193	GO_MICROTUBULE_POLYMERIZATION_OR_DEPOLYMERIZATION	-1.23	0.99	0	0	0	not significant
2194	GO_EPITHELIAL_TO_MESENCHYMAL_TRANSITION	-1.23	0.94	0	0	0	not significant
2195	GO_NEGATIVE_REGULATION_OF_LEUKOCYTE_DIFFERENTIATION	-1.23	0.9	0	0	0	not significant
2196	BROWNE_HCMV_INFECTION_4HR_UP	-1.23	0.88	0	0	0	not significant
2197	GO_CARDIAC_CONDUCTION	-1.23	0.88	0	0	0	not significant
2198	GO_POSITIVE_REGULATION_OF_SMOOTH_MUSCLE_CELL_PROLIFERATION	-1.23	0.85	0	0	0	not significant
2199	NAKAMURA_METASTASIS	-1.23	0.84	0	0	0	not significant
2200	HU_ANGIOGENESIS_DN	-1.23	0.81	0	0	0	not significant
2201	SIG_INSULIN_RECEPTOR_PATHWAY_IN_CARDIAC_MYOCYTES	-1.23	0.81	0	0	0	not significant
2202	GO_ARTERY_MORPHOGENESIS	-1.23	0.79	0	0	0	not significant
2203	ZHAN_MULTIPLE_MYELOMA_SUBGROUPS	-1.23	0.78	0	0	0	not significant
2204	GO_INTRINSIC_COMPONENT_OF_MITOCHONDRIAL_INNER_MEMBRANE	-1.23	0.77	0	0	0	not significant
2205	GO_CILIARY_BASE	-1.23	0.74	0	0	0	not significant
2206	GO_COP9_SIGNALOSOME	-1.23	0.74	0	0	0	not significant
2207	REACTOME_MITOCHONDRIAL_CALCIUM_ION_TRANSPORT	-1.23	0.73	0	0	0	not significant
2208	GO_MONOCARBOXYLIC_ACID_BINDING	-1.23	0.72	0	0	0	not significant
2209	BIOCARTA_GCR_PATHWAY	-1.23	0.7	0	0	0	not significant
2210	GO_INTRINSIC_COMPONENT_OF_MITOCHONDRIAL_OUTER_MEMBRANE	-1.23	0.7	0	0	0	not significant
2211	GO_MAINTENANCE_OF_CELL_POLARITY	-1.23	0.7	0	0	0	not significant
2212	GO_MEMBRANE_FISSION	-1.23	0.69	0	0	0	not significant
2213	GO_REGULATION_OF_OLIGODENDROCYTE_DIFFERENTIATION	-1.23	0.69	0	0	0	not significant
2214	ROVERSI_GLIOMA_LOH_REGIONS	-1.23	0.69	0	0	0	not significant
2215	HALLMARK_NOTCH_SIGNALING	-1.23	0.68	0	0	0	not significant
2216	GO_AMINOACYL_TRNA_METABOLISM_INVOLVED_IN_TRANSLATIONAL_FIDELITY	-1.23	0.67	0	0	0	not significant
2217	GO_METANEPHRIC_NEPHRON_MORPHOGENESIS	-1.23	0.67	0	0	0	not significant
2218	GO_PLUS_END_DIRECTED_ORGANELLE_TRANSPORT_ALONG_MICROTUBULE	-1.23	0.67	0	0	0	not significant
2219	GO_RETINOIC_ACID_RECEPTOR_BINDING	-1.23	0.67	0	0	0	not significant
2220	GO_NEGATIVE_REGULATION_OF_PEPTIDYL_THREONINE_PHOSPHORYLATION	-1.23	0.66	0	0	0	not significant
2221	GO_MITOCHONDRIAL_RIBOSOME_ASSEMBLY	-1.23	0.65	0	0	0	not significant
2222	GO_SULFUR_AMINO_ACID_METABOLIC_PROCESS	-1.23	0.65	0	0	0	not significant
2223	GO_TRNA_SPECIFIC_RIBONUCLEASE_ACTIVITY	-1.23	0.65	0	0	0	not significant
2224	MASRI_RESISTANCE_TO_TAMOXIFEN_AND_AROMATASE_INHIBITORS_UP	-1.23	0.65	0	0	0	not significant
2225	REACTOME_GLYCOGEN_BREAKDOWN_GLYCOGENOLYSIS	-1.23	0.65	0	0	0	not significant
2226	BRUNEAU_SEPTATION_VENTRICULAR	-1.23	0.64	0	0	0	not significant
2227	EHRLICH_ICF_SYNDROM_UP	-1.23	0.64	0	0	0	not significant
2228	GO_ASTER	-1.23	0.64	0	0	0	not significant
2229	GO_CYTOPLASM_ORGANIZATION	-1.23	0.64	0	0	0	not significant
2230	GO_POSITIVE_REGULATION_OF_MYELINATION	-1.23	0.64	0	0	0	not significant
2231	GO_5_3_EXODEOXYRIBONUCLEASE_ACTIVITY	-1.23	0.63	0	0	0	not significant
2232	GO_ENOYL_COA_HYDRATASE_ACTIVITY	-1.23	0.63	0	0	0	not significant
2233	GO_TPR_DOMAIN_BINDING	-1.23	0.63	0	0	0	not significant
2234	LUND_SILENCED_BY_METHYLATION	-1.23	0.63	0	0	0	not significant
2235	REACTOME_CREB1_PHOSPHORYLATION_THROUGH_THE_ACTIVATION_OF_CAMKII	-1.23	0.63	0	0	0	not significant
2236	GO_ACTOMYOSIN_CONTRACTILE_RING_ORGANIZATION	-1.23	0.62	0	0	0	not significant
2237	GO_POSITIVE_REGULATION_OF_HISTONE_UBIQUITINATION	-1.23	0.62	0	0	0	not significant
2238	REACTOME_ACTIVATED_NTRK2_SIGNALS_THROUGH_RAS	-1.23	0.61	0	0	0	not significant
2239	REACTOME_CAMK_IV_MEDIATED_PHOSPHORYLATION_OF_CREB	-1.23	0.61	0	0	0	not significant
2240	GALI_TP53_TARGETS_APOPTOTIC_DN	-1.23	0.6	0	0	0	not significant
2241	GO_RNA_POLYMERASE_III_TYPE_3_PROMOTER_DNA_BINDING	-1.23	0.6	0	0	0	not significant
2242	KEGG_LIMONENE_AND_PINENE_DEGRADATION	-1.23	0.6	0	0	0	not significant
2243	REACTOME_INACTIVATION_OF_CDC42_AND_RAC1	-1.23	0.59	0	0	0	not significant
2244	GO_PHOSPHATIDYLINOSITOL_3_KINASE_REGULATORY_SUBUNIT_BINDING	-1.23	0.58	0	0	0	not significant
2245	GO_PROTEIN_LOCALIZATION_TO_ORGANELLE	-1.22	4	0	0	0	not significant
2246	GO_CENTROSOME	-1.22	1.82	0	0	0	not significant
2247	SCHAEFFER_PROSTATE_DEVELOPMENT_6HR_DN	-1.22	1.66	0	0	0	not significant
2248	MOOTHA_PGC	-1.22	1.57	0	0	0	not significant
2249	SENGUPTA_NASOPHARYNGEAL_CARCINOMA_WITH_LMP1_UP	-1.22	1.39	0	0	0	not significant
2250	KEGG_HUNTINGTONS_DISEASE	-1.22	1.16	0	0	0	not significant
2251	GO_S_ADENOSYLMETHIONINE_DEPENDENT_METHYLTRANSFERASE_ACTIVITY	-1.22	1.1	0	0	0	not significant
2252	GO_FC_RECEPTOR_SIGNALING_PATHWAY	-1.22	1.03	0	0	0	not significant
2253	GO_MAGNESIUM_ION_BINDING	-1.22	1.02	0	0	0	not significant
2254	GO_ORGAN_GROWTH	-1.22	0.96	0	0	0	not significant
2255	GO_REGENERATION	-1.22	0.95	0	0	0	not significant
2256	GO_RNA_PHOSPHODIESTER_BOND_HYDROLYSIS_ENDONUCLEOLYTIC	-1.22	0.91	0	0	0	not significant
2257	DURCHDEWALD_SKIN_CARCINOGENESIS_UP	-1.22	0.89	0	0	0	not significant
2258	JAIN_NFKB_SIGNALING	-1.22	0.84	0	0	0	not significant
2259	REACTOME_COSTIMULATION_BY_THE_CD28_FAMILY	-1.22	0.84	0	0	0	not significant
2260	REACTOME_SIGNALING_BY_RAS_MUTANTS	-1.22	0.84	0	0	0	not significant
2261	WANG_PROSTATE_CANCER_ANDROGEN_INDEPENDENT	-1.22	0.82	0	0	0	not significant
2262	MODY_HIPPOCAMPUS_PRENATAL	-1.22	0.81	0	0	0	not significant
2263	REACTOME_PYRUVATE_METABOLISM_AND_CITRIC_ACID_TCA_CYCLE	-1.22	0.79	0	0	0	not significant
2264	GO_NEGATIVE_REGULATION_OF_UBIQUITIN_DEPENDENT_PROTEIN_CATABOLIC_PF	-1.22	0.78	0	0	0	not significant
2265	CHR5Q23	-1.22	0.76	0	0	0	not significant
2266	APPIERTO_RESPONSE_TO_FENRETINIDE_UP	-1.22	0.75	0	0	0	not significant
2267	GO_RESPIRATORY_CHAIN_COMPLEX_IV_ASSEMBLY	-1.22	0.74	0	0	0	not significant
2268	REACTOME_SPRY_REGULATION_OF_FGF_SIGNALING	-1.22	0.72	0	0	0	not significant
2269	GO_POLY_PURINE_TRACT_BINDING	-1.22	0.71	0	0	0	not significant
2270	HOQUE_METHYLATED_IN_CANCER	-1.22	0.7	0	0	0	not significant
2271	STAMBOLSKY_TARGETS_OF_MUTATED_TP53_UP	-1.22	0.69	0	0	0	not significant
2272	BIOCARTA_LONGEVITY_PATHWAY	-1.22	0.68	0	0	0	not significant
2273	GO_LIPOPROTEIN_PARTICLE_RECEPTOR_BINDING	-1.22	0.68	0	0	0	not significant
2274	GO_REGULATION_OF_OXIDATIVE_PHOSPHORYLATION	-1.22	0.67	0	0	0	not significant
2275	ABRAHAM_ALPC_VS_MULTIPLE_MYELOMA_DN	-1.22	0.66	0	0	0	not significant
2276	GO_CHANNEL_INHIBITOR_ACTIVITY	-1.22	0.66	0	0	0	not significant
2277	GO_CYTOSKELETAL_ADAPTOR_ACTIVITY	-1.22	0.66	0	0	0	not significant

2278	GO_PROTEIN_DENEDDYLATION	-1.22	0.66	0	0	0	not significant
2279	GO_VENTRICULAR_TRABECULA_MYOCARDIUM_MORPHOGENESIS	-1.22	0.66	0	0	0	not significant
2280	REACTOME_DEFECTS_IN_BIOTIN_BTN_METABOLISM	-1.22	0.66	0	0	0	not significant
2281	GO_CELLULAR_RESPONSE_TO_NITRIC_OXIDE	-1.22	0.65	0	0	0	not significant
2282	WU_HBX_TARGETS_1_DN	-1.22	0.65	0	0	0	not significant
2283	REACTOME_GLUCAGON_SIGNALING_IN_METABOLIC_REGULATION	-1.22	0.64	0	0	0	not significant
2284	GO_GERM_CELL_PROLIFERATION	-1.22	0.63	0	0	0	not significant
2285	GO_LIGASE_ACTIVITY_FORMING_CARBON_CARBON_BONDS	-1.22	0.63	0	0	0	not significant
2286	GO_NUCLEOLAR_LARGE_RRNA_TRANSCRIPTION_BY_RNA_POLYMERASE_I	-1.22	0.63	0	0	0	not significant
2287	GO_POSITIVE_REGULATION_OF_SMAD_PROTEIN_SIGNAL_TRANSDUCTION	-1.22	0.63	0	0	0	not significant
2288	GO_CADHERIN_BINDING_INVOLVED_IN_CELL_CELL_ADHESION	-1.22	0.62	0	0	0	not significant
2289	GO_KINETOCHORE_MICROTUBULE	-1.22	0.62	0	0	0	not significant
2290	GO_MITOCHONDRIAL_DNA_REPLICATION	-1.22	0.62	0	0	0	not significant
2291	GO_PEPTIDYL_ARGININE_METHYLATION	-1.22	0.62	0	0	0	not significant
2292	GO_POSTSYNAPTIC_SIGNAL_TRANSDUCTION	-1.22	0.62	0	0	0	not significant
2293	REACTOME_RESPONSE_TO_METAL_IONS	-1.22	0.62	0	0	0	not significant
2294	GO_INOSITOL_TRISPHOSPHATE_KINASE_ACTIVITY	-1.22	0.61	0	0	0	not significant
2295	WAGNER_APO2_SENSITIVITY	-1.22	0.61	0	0	0	not significant
2296	GO_PRIMARY_AMINO_COMPOUND_METABOLIC_PROCESS	-1.22	0.6	0	0	0	not significant
2297	GO_MECHANOSENSORY_BEHAVIOR	-1.22	0.59	0	0	0	not significant
2298	GO_NEGATIVE_REGULATION_OF_EXECUTION_PHASE_OF_APOPTOSIS	-1.22	0.59	0	0	0	not significant
2299	GO_CORE_MEDIATOR_COMPLEX	-1.22	0.58	0	0	0	not significant
2300	VICENT_METASTASIS_UP	-1.22	0.58	0	0	0	not significant
2301	GO_PHOSPHATIDYLINOSITOL_3_5_BISPHOSPHATE_3_PHOSPHATASE_ACTIVITY	-1.22	0.57	0	0	0	not significant
2302	GO_LIPID_KINASE_ACTIVITY	-1.22	0.55	0	0	0	not significant
2303	GO_MITOCHONDRIAL_PART	-1.21	4	0	0	0	not significant
2304	GO_REGULATION_OF_CELLULAR_COMPONENT_BIOGENESIS	-1.21	4	0	0	0	not significant
2305	PILON_KLF1_TARGETS_DN	-1.21	4	0	0	0	not significant
2306	REACTOME_VESICLE_MEDIATED_TRANSPORT	-1.21	2.04	0	0	0	not significant
2307	GO_RESPONSE_TO_OXYGEN_LEVELS	-1.21	1.53	0	0	0	not significant
2308	MOOTHA_HUMAN_MITODB_6_2002	-1.21	1.53	0	0	0	not significant
2309	GO_RESPONSE_TO_TEMPERATURE_STIMULUS	-1.21	1.38	0	0	0	not significant
2310	CREIGHTON_ENDOCRINE_THERAPY_RESISTANCE_4	-1.21	1.26	0	0	0	not significant
2311	GO_CELLULAR_RESPIRATION	-1.21	1.22	0	0	0	not significant
2312	PGF_UP.V1_UP	-1.21	1.18	0	0	0	not significant
2313	GO_CHROMATIN_ASSEMBLY_OR_DISASSEMBLY	-1.21	1.17	0	0	0	not significant
2314	GO_ENDOPLASMIC_RETICULUM_TO_GOLGI_VESICLE_MEDIATED_TRANSPORT	-1.21	1.06	0	0	0	not significant
2315	GO_RNA_PHOSPHODIESTER_BOND_HYDROLYSIS	-1.21	1.04	0	0	0	not significant
2316	XU_GH1_AUTOCRINE_TARGETS_DN	-1.21	0.96	0	0	0	not significant
2317	RIGGI_EWING_SARCOMA_PROGENITOR_DN	-1.21	0.95	0	0	0	not significant
2318	DOANE_RESPONSE_TO_ANDROGEN_UP	-1.21	0.94	0	0	0	not significant
2319	GO_REGULATION_OF_CELLULAR_KETONE_METABOLIC_PROCESS	-1.21	0.92	0	0	0	not significant
2320	KEGG_WNT_SIGNALING_PATHWAY	-1.21	0.91	0	0	0	not significant
2321	REACTOME_SIGNALING_BY_NTRK1_TRKA	-1.21	0.9	0	0	0	not significant
2322	GO_NUCLEOSOMAL_DNA_BINDING	-1.21	0.89	0	0	0	not significant
2323	GO_POLYSOME	-1.21	0.89	0	0	0	not significant
2324	GO_CIRCADIAN_REGULATION_OF_GENE_EXPRESSION	-1.21	0.82	0	0	0	not significant
2325	GO_ORGANELLE_TRANSPORT_ALONG_MICROTUBULE	-1.21	0.82	0	0	0	not significant
2326	GO_RESPONSE_TO_NERVE_GROWTH_FACTOR	-1.21	0.82	0	0	0	not significant
2327	GALE_APL_WITH_FLT3_MUTATED_UP	-1.21	0.81	0	0	0	not significant
2328	GO_REGULATION_OF_GLIAL_CELL_DIFFERENTIATION	-1.21	0.79	0	0	0	not significant
2329	GO_REGULATION_OF_HEART_RATE	-1.21	0.76	0	0	0	not significant
2330	GO_NUCLEOID	-1.21	0.75	0	0	0	not significant
2331	GO_ARTERY_DEVELOPMENT	-1.21	0.74	0	0	0	not significant
2332	GO_CARDIAC_MUSCLE_TISSUE_MORPHOGENESIS	-1.21	0.74	0	0	0	not significant
2333	CAFFAREL_RESPONSE_TO_THC_24HR_5_UP	-1.21	0.73	0	0	0	not significant
2334	REACTOME_DOWNREGULATION_OF_SMAD2_3:SMAD4_TRANSCRIPTIONAL_ACTIVIT	-1.21	0.73	0	0	0	not significant
2335	AMIT_EGF_RESPONSE_60_HELA	-1.21	0.72	0	0	0	not significant
2336	CHR9Q22	-1.21	0.72	0	0	0	not significant
2337	GO_MRNA_CIS_SPLICING_VIA_SPLICEOSOME	-1.21	0.72	0	0	0	not significant
2338	CHUANG_OXIDATIVE_STRESS_RESPONSE_UP	-1.21	0.69	0	0	0	not significant
2339	GO_MATING	-1.21	0.68	0	0	0	not significant
2340	GO_DOPAMINE_RECEPTOR_SIGNALING_PATHWAY	-1.21	0.66	0	0	0	not significant
2341	GO_POSITIVE_REGULATION_OF_VIRAL_RELEASE_FROM_HOST_CELL	-1.21	0.64	0	0	0	not significant
2342	GO_POSITIVE_REGULATION_OF_LAMELLIPODIUM_ORGANIZATION	-1.21	0.63	0	0	0	not significant
2343	REACTOME_TRAF6_MEDIATED_IRF7_ACTIVATION	-1.21	0.63	0	0	0	not significant
2344	GO_MITOCHONDRIAL_TRANSCRIPTION	-1.21	0.62	0	0	0	not significant
2345	GO_PEROXISOME_PROLIFERATOR_ACTIVATED_RECEPTOR_SIGNALING_PATHWAY	-1.21	0.62	0	0	0	not significant
2346	GO_POSITIVE_REGULATION_BY_HOST_OF_VIRAL_PROCESS	-1.21	0.62	0	0	0	not significant
2347	GO_RNA_STRAND_ANNEALING_ACTIVITY	-1.21	0.62	0	0	0	not significant
2348	GO_SKELETAL_MUSCLE_ADAPTATION	-1.21	0.61	0	0	0	not significant
2349	GO_N6_METHYLADENOSINE_CONTAINING_RNA_BINDING	-1.21	0.6	0	0	0	not significant
2350	REACTOME_RUNX1_REGULATES_TRANSCRIPTION_OF_GENES_INVOLVED_IN_DIFFE	-1.21	0.58	0	0	0	not significant
2351	GO_S_SHAPED_BODY_MORPHOGENESIS	-1.21	0.57	0	0	0	not significant
2352	GO_SENSORY_PERCEPTION_OF_TEMPERATURE_STIMULUS	-1.21	0.56	0	0	0	not significant
2353	GO_CYTOSKELETAL_PART	-1.20	4	0	0	0	not significant
2354	GO_CYTOSKELETON_ORGANIZATION	-1.20	4	0	0	0	not significant
2355	GO_POSITIVE_REGULATION_OF_CELLULAR_COMPONENT_ORGANIZATION	-1.20	4	0	0	0	not significant
2356	LASTOWSKA_NEUROBLASTOMA_COPY_NUMBER_DN	-1.20	4	0	0	0	not significant
2357	REACTOME_POST_TRANSLATIONAL_PROTEIN_MODIFICATION	-1.20	4	0	0	0	not significant
2358	GO_PROTEIN_KINASE_ACTIVITY	-1.20	1.6	0	0	0	not significant
2359	MOOTHA_MITOCHONDRIA	-1.20	1.55	0	0	0	not significant
2360	COLINA_TARGETS_OF_4EBP1_AND_4EBP2	-1.20	1.39	0	0	0	not significant
2361	GO_ATPASE_ACTIVITY_COUPLED	-1.20	1.27	0	0	0	not significant
2362	REACTOME_INTRACELLULAR_SIGNALING_BY_SECOND_MESSENGERS	-1.20	1.26	0	0	0	not significant
2363	GO_RIBOSOME	-1.20	1.16	0	0	0	not significant
2364	GO_NEGATIVE_REGULATION_OF_CATABOLIC_PROCESS	-1.20	1.15	0	0	0	not significant
2365	REACTOME_REGULATION_OF_TP53_ACTIVITY	-1.20	1.1	0	0	0	not significant
2366	KYNG_WERNER_SYNDROM_AND_NORMAL_AGING_DN	-1.20	1.06	0	0	0	not significant
2367	PRC2_EZH2_UP.V1_DN	-1.20	1	0	0	0	not significant
2368	GO_POSITIVE_REGULATION_OF_PROTEOLYSIS_INVOLVED_IN_CELLULAR_PROTEIN	-1.20	0.93	0	0	0	not significant
2369	ZHONG_SECRETOME_OF_LUNG_CANCER_AND_FIBROBLAST	-1.20	0.83	0	0	0	not significant
2370	GO_I_BAND	-1.20	0.82	0	0	0	not significant
2371	GO_PYRIMIDINE_CONTAINING_COMPOUND_BIOSYNTHETIC_PROCESS	-1.20	0.81	0	0	0	not significant
2372	REACTOME_FCGAMMA_RECEPTOR_FCGR_DEPENDENT_PHAGOCYTOSIS	-1.20	0.8	0	0	0	not significant
2373	REACTOME_SLC_TRANSPORTER_DISORDERS	-1.20	0.8	0	0	0	not significant
2374	GO_POSITIVE_REGULATION_OF_PEPTIDYL_SERINE_PHOSPHORYLATION	-1.20	0.75	0	0	0	not significant
2375	GO_SARCOPLASM	-1.20	0.75	0	0	0	not significant
2376	GO_MULTICELLULAR_ORGANISMAL_MOVEMENT	-1.20	0.71	0	0	0	not significant
2377	KIM_LIVER_CANCER_POOR_SURVIVAL_UP	-1.20	0.7	0	0	0	not significant
2378	PID_HIF1A_PATHWAY	-1.20	0.7	0	0	0	not significant
2379	WILCOX_RESPONSE_TO_PROGESTERONE_DN	-1.20	0.7	0	0	0	not significant
2380	CHR11Q14	-1.20	0.69	0	0	0	not significant
2381	GO_PHOSPHATASE_INHIBITOR_ACTIVITY	-1.20	0.69	0	0	0	not significant
2382	REACTOME_GPVI_MEDIATED_ACTIVATION_CASCADE	-1.20	0.69	0	0	0	not significant
2383	GO_CHROMATIN_DISASSEMBLY	-1.20	0.68	0	0	0	not significant
2384	PID_NCADHERIN_PATHWAY	-1.20	0.68	0	0	0	not significant
2385	GO_CELL_COMMUNICATION_INVOLVED_IN_CARDIAC_CONDUCTION	-1.20	0.67	0	0	0	not significant
2386	GO_INCLUSION_BODY_ASSEMBLY	-1.20	0.67	0	0	0	not significant

2387	GO_NUCLEOTIDE_PHOSPHORYLATION	-1.20	0.66	0	0	0	not significant
2388	GO_REGULATION_OF_SUBSTRATE_ADHESION_DEPENDENT_CELL_SPREADING	-1.20	0.66	0	0	0	not significant
2389	PARENT_MTOR_SIGNALING_DN	-1.20	0.66	0	0	0	not significant
2390	ANASTASSIOU_MULTICANCER_INVASIVENESS_SIGNATURE	-1.20	0.65	0	0	0	not significant
2391	GO_REGULATION_OF_CARDIAC_MUSCLE_CONTRACTION_BY_REGULATION_OF_TH	-1.20	0.65	0	0	0	not significant
2392	GO_PROTEIN_LOCALIZATION_TO_CHROMOSOME_CENTROMERIC_REGION	-1.20	0.64	0	0	0	not significant
2393	GO_REGULATION_OF_CELLULAR_RESPIRATION	-1.20	0.64	0	0	0	not significant
2394	REACTOME_DOWNSTREAM_SIGNALING_OF_ACTIVATED_FGFR4	-1.20	0.64	0	0	0	not significant
2395	GO_POSITIVE_REGULATION_OF_TRANSCRIPTION_OF_NUCLEOLAR_LARGE_RRNA	-1.20	0.63	0	0	0	not significant
2396	REACTOME_DOWNSTREAM_SIGNALING_OF_ACTIVATED_FGFR3	-1.20	0.63	0	0	0	not significant
2397	ZHAN_V1_LATE_DIFFERENTIATION_GENES_DN	-1.20	0.63	0	0	0	not significant
2398	GO_MATERNAL_PLACENTA_DEVELOPMENT	-1.20	0.62	0	0	0	not significant
2399	GO_POSITIVE_REGULATION_BY_HOST_OF_VIRAL_TRANSCRIPTION	-1.20	0.62	0	0	0	not significant
2400	GO_SUCKLING_BEHAVIOR	-1.20	0.61	0	0	0	not significant
2401	PETRETTO_HEART_MASS_QTL_CIS_DN	-1.20	0.61	0	0	0	not significant
2402	GO_AXO_DENDRITIC_PROTEIN_TRANSPORT	-1.20	0.6	0	0	0	not significant
2403	HOEGERKORP_CD44_TARGETS_DIRECT_UP	-1.20	0.6	0	0	0	not significant
2404	REACTOME_DOWNSTREAM_SIGNALING_OF_ACTIVATED_FGFR1	-1.20	0.6	0	0	0	not significant
2405	REACTOME_DOWNSTREAM_SIGNALING_OF_ACTIVATED_FGFR2	-1.20	0.6	0	0	0	not significant
2406	GO_ACTIN_DEPENDENT_ATPASE_ACTIVITY	-1.20	0.59	0	0	0	not significant
2407	GO_HISTONE_H3_K36_METHYLATION	-1.20	0.59	0	0	0	not significant
2408	GO_NEGATIVE_REGULATION_OF_DNA_DAMAGE_RESPONSE_SIGNAL_TRANSDUCTI	-1.20	0.59	0	0	0	not significant
2409	GO_SUMO_SPECIFIC_PROTEASE_ACTIVITY	-1.20	0.59	0	0	0	not significant
2410	REACTOME_PI_3K_CASCADE:FGFR3	-1.20	0.59	0	0	0	not significant
2411	GO_MEIOSIS_II_CELL_CYCLE_PROCESS	-1.20	0.58	0	0	0	not significant
2412	GO_MYOSIN_II_COMPLEX	-1.20	0.58	0	0	0	not significant
2413	GO_PURINE_NUCLEOBASE_BIOSYNTHETIC_PROCESS	-1.20	0.58	0	0	0	not significant
2414	REACTOME_GLUCAGON_TYPE_LIGAND_RECEPTORS	-1.20	0.58	0	0	0	not significant
2415	REACTOME_SURFACTANT_METABOLISM	-1.20	0.58	0	0	0	not significant
2416	GO_AXONAL_TRANSPORT_OF_MITOCHONDRION	-1.20	0.57	0	0	0	not significant
2417	GO_POSITIVE_REGULATION_OF_NEURON_MIGRATION	-1.20	0.57	0	0	0	not significant
2418	GO_RESPONSE_TO_TUMOR_CELL	-1.20	0.57	0	0	0	not significant
2419	WU_ALZHEIMER_DISEASE_UP	-1.20	0.57	0	0	0	not significant
2420	REACTOME_UBIQUINOL_BIOSYNTHESIS	-1.20	0.56	0	0	0	not significant
2421	GO_7SK_SNRNA_BINDING	-1.20	0.55	0	0	0	not significant
2422	GO_MALE_GENITALIA_DEVELOPMENT	-1.20	0.55	0	0	0	not significant
2423	GO_MOTOR_NEURON_APOPTOTIC_PROCESS	-1.20	0.54	0	0	0	not significant
2424	REACTOME_PI_3K_CASCADE:FGFR2	-1.20	0.54	0	0	0	not significant
2425	GO_HOST_INTRACELLULAR_ORGANELLE	-1.20	0.53	0	0	0	not significant
2426	GO_INTRACELLULAR_PROTEIN_TRANSPORT	-1.19	4	0	0	0	not significant
2427	GO_MACROMOLECULE_CATABOLIC_PROCESS	-1.19	4	0	0	0	not significant
2428	GO_ORGANELLE_ASSEMBLY	-1.19	4	0	0	0	not significant
2429	GO_TRANSFERASE_COMPLEX	-1.19	1.89	0	0	0	not significant
2430	GO_MITOCHONDRIAL_ENVELOPE	-1.19	1.54	0	0	0	not significant
2431	GO_NEGATIVE_REGULATION_OF_CELL_CYCLE	-1.19	1.48	0	0	0	not significant
2432	GO_POSITIVE_REGULATION_OF_PROTEOLYSIS	-1.19	1.32	0	0	0	not significant
2433	YOSHIMURA_MAPK8_TARGETS_DN	-1.19	1.17	0	0	0	not significant
2434	GO_CYTOSKELETON_DEPENDENT_INTRACELLULAR_TRANSPORT	-1.19	1.12	0	0	0	not significant
2435	WANG_TUMOR_INVASIVENESS_DN	-1.19	1.11	0	0	0	not significant
2436	WONG_MITOCHONDRIA_GENE_MODULE	-1.19	1.04	0	0	0	not significant
2437	REACTOME_PLATELET_ACTIVATION_SIGNALING_AND_AGGREGATION	-1.19	0.96	0	0	0	not significant
2438	REACTOME_PROTEIN_LOCALIZATION	-1.19	0.95	0	0	0	not significant
2439	BURTON_ADIPOGENESIS_5	-1.19	0.92	0	0	0	not significant
2440	REACTOME_SIGNALING_BY_NTRKS	-1.19	0.89	0	0	0	not significant
2441	GO_HETEROCHROMATIN	-1.19	0.86	0	0	0	not significant
2442	GO_CYTOPLASMIC_STRESS_GRANULE	-1.19	0.85	0	0	0	not significant
2443	GO_DNA_GEOMETRIC_CHANGE	-1.19	0.79	0	0	0	not significant
2444	FIGUEROA_AML_METHYLATION_CLUSTER_1_UP	-1.19	0.78	0	0	0	not significant
2445	RODRIGUES_THYROID_CARCINOMA_DN	-1.19	0.78	0	0	0	not significant
2446	GO_POSITIVE_REGULATION_OF_GENE_EXPRESSION_EPIGENETIC	-1.19	0.77	0	0	0	not significant
2447	GO_CYTOCHROME_COMPLEX_ASSEMBLY	-1.19	0.73	0	0	0	not significant
2448	REACTOME_RHO_GTPASES_ACTIVATE_WASPS_AND_WAVES	-1.19	0.73	0	0	0	not significant
2449	PID_LKB1_PATHWAY	-1.19	0.71	0	0	0	not significant
2450	GO_GROWTH_FACTOR_ACTIVITY	-1.19	0.7	0	0	0	not significant
2451	GO_NEURAL_CREST_CELL_DIFFERENTIATION	-1.19	0.7	0	0	0	not significant
2452	HASLINGER_B_CLL_WITH_17P13_DELETION	-1.19	0.69	0	0	0	not significant
2453	MOREIRA_RESPONSE_TO_TSA_UP	-1.19	0.69	0	0	0	not significant
2454	GO_THYMUS_DEVELOPMENT	-1.19	0.66	0	0	0	not significant
2455	GO_REGULATION_OF_CALCINEURIN_MEDIATED_SIGNALING	-1.19	0.65	0	0	0	not significant
2456	GO_T_TUBULE	-1.19	0.65	0	0	0	not significant
2457	REACTOME_SMAD2_SMAD3:SMAD4_HETEROTRIMER_REGULATES_TRANSCRIPTION	-1.19	0.65	0	0	0	not significant
2458	TIEN_INTESTINE_PROBIOTICS_2HR_UP	-1.19	0.65	0	0	0	not significant
2459	GO_OOCYTE_DIFFERENTIATION	-1.19	0.63	0	0	0	not significant
2460	REACTOME_METABOLISM_OF_COFACTORS	-1.19	0.63	0	0	0	not significant
2461	GO_REGULATION_OF_ASTROCYTE_DIFFERENTIATION	-1.19	0.61	0	0	0	not significant
2462	GO_RELEASE_OF_SEQUESTERED_CALCIUM_ION_INTO_CYTOSOL_BY_ENDOPLASM	-1.19	0.61	0	0	0	not significant
2463	GO_CELLULAR_RESPONSE_TO_VITAMIN	-1.19	0.6	0	0	0	not significant
2464	GO_NEGATIVE_REGULATION_OF_CENTROSOME_CYCLE	-1.19	0.6	0	0	0	not significant
2465	GO_POSITIVE_REGULATION_OF_KERATINOCYTE_DIFFERENTIATION	-1.19	0.6	0	0	0	not significant
2466	REACTOME_GLUTAMATE_BINDING_ACTIVATION_OF_AMPA_RECEPTORS_AND_SYN	-1.19	0.6	0	0	0	not significant
2467	DISTECHE_ESCAPED_FROM_X_INACTIVATION	-1.19	0.59	0	0	0	not significant
2468	GO_FRUCTOSE_METABOLIC_PROCESS	-1.19	0.58	0	0	0	not significant
2469	GO_GENITALIA_DEVELOPMENT	-1.19	0.58	0	0	0	not significant
2470	GO_PSEUDOPODIUM_ORGANIZATION	-1.19	0.58	0	0	0	not significant
2471	MYLLYKANGAS_AMPLIFICATION_HOT_SPOT_17	-1.19	0.58	0	0	0	not significant
2472	RICKMAN_TUMOR_DIFFERENTIATED_MODERATELY_VS_POORLY_DN	-1.19	0.58	0	0	0	not significant
2473	GO_EXPLORATION_BEHAVIOR	-1.19	0.57	0	0	0	not significant
2474	GO_MYOSIN_FILAMENT	-1.19	0.57	0	0	0	not significant
2475	GO_NEGATIVE_REGULATION_OF_GLUCOSE_TRANSMEMBRANE_TRANSPORT	-1.19	0.57	0	0	0	not significant
2476	GO_PEPTIDYL_ARGININE_METHYLATION_TO_ASYMMETRICAL_DIMETHYL_ARGININE	-1.19	0.57	0	0	0	not significant
2477	GO_POSITIVE_REGULATION_OF_LONG_TERM_SYNAPTIC_POTENTIATION	-1.19	0.57	0	0	0	not significant
2478	GO_REGULATION_OF_LONG_TERM_NEURONAL_SYNAPTIC_PLASTICITY	-1.19	0.57	0	0	0	not significant
2479	GO_TRNA_GUANINE_METHYLTRANSFERASE_ACTIVITY	-1.19	0.57	0	0	0	not significant
2480	REACTOME_TRNA_PROCESSING_IN_THE_MITOCHONDRION	-1.19	0.56	0	0	0	not significant
2481	GO_ALDEHYDE_DEHYDROGENASE_NAD_ACTIVITY	-1.19	0.55	0	0	0	not significant
2482	GO_TRANSCRIPTION_FACTOR_TFIIIB_COMPLEX	-1.19	0.55	0	0	0	not significant
2483	GO_WASH_COMPLEX	-1.19	0.55	0	0	0	not significant
2484	REACTOME_REGULATION_OF_PTEN_LOCALIZATION	-1.19	0.55	0	0	0	not significant
2485	GO_CALCIUM_CHANNEL_INHIBITOR_ACTIVITY	-1.19	0.54	0	0	0	not significant
2486	REACTOME_SUMO_IS_PROTEOLYTICALLY_PROCESSED	-1.19	0.54	0	0	0	not significant
2487	GO_GROWTH_HORMONE_SECRETION	-1.19	0.53	0	0	0	not significant
2488	GO_NEGATIVE_REGULATION_OF_CHONDROCYTE_DIFFERENTIATION	-1.19	0.53	0	0	0	not significant
2489	GO_OPSONIN_BINDING	-1.19	0.53	0	0	0	not significant
2490	GO_STRESS_GRANULE_DISASSEMBLY	-1.19	0.52	0	0	0	not significant
2491	GO_HYALURONIC_ACID_BINDING	-1.19	0.51	0	0	0	not significant
2492	GO_RESPONSE_TO_ISOLATION_STRESS	-1.19	0.51	0	0	0	not significant
2493	GO_PROTEIN_PHOSPHORYLATION	-1.18	4	0	0	0	not significant
2494	REACTOME_GENERIC_TRANSCRIPTION_PATHWAY	-1.18	4	0	0	0	not significant
2495	REACTOME_DISEASES_OF_SIGNAL_TRANSDUCTION	-1.18	1.65	0	0	0	not significant

2496	GO_REGULATION_OF_NERVOUS_SYSTEM_DEVELOPMENT	-1.18	1.45	0	0	0	not significant
2497	STEIN_ESRRA_TARGETS_UP	-1.18	1.42	0	0	0	not significant
2498	GO_CELL_CYCLE_CHECKPOINT	-1.18	1.05	0	0	0	not significant
2499	GO_FICOLIN_1_RICH_GRANULE	-1.18	0.97	0	0	0	not significant
2500	GO_MIDBODY	-1.18	0.94	0	0	0	not significant
2501	GARCIA_TARGETS_OF_FLI1_AND_DAX1_DN	-1.18	0.9	0	0	0	not significant
2502	GO_MITOTIC_CELL_CYCLE_CHECKPOINT	-1.18	0.89	0	0	0	not significant
2503	REACTOME_TRANSMISSION_ACROSS_CHEMICAL_SYNAPSES	-1.18	0.89	0	0	0	not significant
2504	GOLDRATH_HOMEOSTATIC_PROLIFERATION	-1.18	0.88	0	0	0	not significant
2505	FIRESTEIN_PROLIFERATION	-1.18	0.84	0	0	0	not significant
2506	GO_ACETYLTRANSFERASE_ACTIVITY	-1.18	0.84	0	0	0	not significant
2507	P53_DN.V2_DN	-1.18	0.81	0	0	0	not significant
2508	GO_REGULATION_OF_PEPTIDYL_SERINE_PHOSPHORYLATION	-1.18	0.79	0	0	0	not significant
2509	TSENG_IRS1_TARGETS_UP	-1.18	0.78	0	0	0	not significant
2510	BERTUCCI_MEDULLARY_VS_DUCTAL_BREAST_CANCER_DN	-1.18	0.77	0	0	0	not significant
2511	GO_REGULATION_OF_PROTEIN_COMPLEX_DISASSEMBLY	-1.18	0.77	0	0	0	not significant
2512	GO_ENDOPEPTIDASE_REGULATOR_ACTIVITY	-1.18	0.76	0	0	0	not significant
2513	SESTO_RESPONSE_TO_UV_C2	-1.18	0.74	0	0	0	not significant
2514	KEGG_PATHOGENIC_ESCHERICHIA_COLI_INFECTION	-1.18	0.72	0	0	0	not significant
2515	GO_ATP_DEPENDENT_DNA_HELICASE_ACTIVITY	-1.18	0.7	0	0	0	not significant
2516	BROWNE_HCMV_INFECTION_6HR_UP	-1.18	0.69	0	0	0	not significant
2517	CHOI_ATL_STAGE_PREDICTOR	-1.18	0.68	0	0	0	not significant
2518	PID_ECADHERIN_NASCENT_AJ_PATHWAY	-1.18	0.67	0	0	0	not significant
2519	BROWNE_HCMV_INFECTION_8HR_DN	-1.18	0.66	0	0	0	not significant
2520	GO_NEGATIVE_REGULATION_OF_INTRINSIC_APOPTOTIC_SIGNALING_PATHWAY_IN	-1.18	0.66	0	0	0	not significant
2521	GENTILE_UV_RESPONSE_CLUSTER_D6	-1.18	0.65	0	0	0	not significant
2522	WIEDERSCHAIN_TARGETS_OF_BMI1_AND_PCGF2	-1.18	0.65	0	0	0	not significant
2523	CHANGOLKAR_H2AFY_TARGETS_DN	-1.18	0.64	0	0	0	not significant
2524	ANASTASSIOU_CANCER_MESENCHYMAL_TRANSITION_SIGNATURE	-1.18	0.62	0	0	0	not significant
2525	GO_INTRASPECIES_INTERACTION_BETWEEN_ORGANISMS	-1.18	0.62	0	0	0	not significant
2526	GO_TRANSFORMING_GROWTH_FACTOR_BETA_RECEPTOR_BINDING	-1.18	0.62	0	0	0	not significant
2527	GO_NEGATIVE_REGULATION_OF_CATION_CHANNEL_ACTIVITY	-1.18	0.61	0	0	0	not significant
2528	GO_RETINOIC_ACID_RECEPTOR_SIGNALING_PATHWAY	-1.18	0.61	0	0	0	not significant
2529	REACTOME_SIGNALING_BY_EGFRVIII_IN_CANCER	-1.18	0.6	0	0	0	not significant
2530	GO_OXIDOREDUCTASE_ACTIVITY_ACTING_ON_THE_CH_NH2_GROUP_OF_DONORS	-1.18	0.59	0	0	0	not significant
2531	GO_REGULATION_OF_CARDIAC_MUSCLE_CELL_ACTION_POTENTIAL	-1.18	0.59	0	0	0	not significant
2532	GO_RESPIRATORY_CHAIN_COMPLEX_III	-1.18	0.59	0	0	0	not significant
2533	GO_GOLGI_TRANSPORT_COMPLEX	-1.18	0.58	0	0	0	not significant
2534	MATTIOLI_MULTIPLE_MYELOMA_SUBGROUPS	-1.18	0.58	0	0	0	not significant
2535	SA_G2_AND_M_PHASES	-1.18	0.57	0	0	0	not significant
2536	GO_CELLULAR_RESPONSE_TO_EPINEPHRINE_STIMULUS	-1.18	0.56	0	0	0	not significant
2537	GO_N_TERMINAL_PEPTIDYL_METHIONINE_ACETYLATION	-1.18	0.56	0	0	0	not significant
2538	GO_NITRIC_OXIDE_SYNTHASE_BINDING	-1.18	0.56	0	0	0	not significant
2539	GO_POSITIVE_REGULATION_OF_MEMBRANE_POTENTIAL	-1.18	0.56	0	0	0	not significant
2540	GO_NEGATIVE_REGULATION_OF_SKELETAL_MUSCLE_TISSUE_DEVELOPMENT	-1.18	0.55	0	0	0	not significant
2541	GO_NUCLEOSIDE_DIPHOSPHATE_BIOSYNTHETIC_PROCESS	-1.18	0.55	0	0	0	not significant
2542	GO_POSITIVE_REGULATION_OF_SODIUM_ION_TRANSMEMBRANE_TRANSPORTER_/	-1.18	0.55	0	0	0	not significant
2543	GO_SPONGIOTROPHOBLAST_LAYER_DEVELOPMENT	-1.18	0.55	0	0	0	not significant
2544	MOROSETTI_FACIOSCAPULOHUMERAL_MUSCULAR_DISTROPHY_UP	-1.18	0.55	0	0	0	not significant
2545	GO_NEGATIVE_REGULATION_OF_SMOOTHENED_SIGNALING_PATHWAY_INVOLVED_	-1.18	0.54	0	0	0	not significant
2546	GO_POSITIVE_REGULATION_OF_HISTONE_PHOSPHORYLATION	-1.18	0.54	0	0	0	not significant
2547	GO_REGULATION_BY_VIRUS_OF_VIRAL_PROTEIN_LEVELS_IN_HOST_CELL	-1.18	0.54	0	0	0	not significant
2548	REACTOME_PTK6_REGULATES_RHO_GTPASES_RAS_GTPASE_AND_MAP_KINASES	-1.18	0.54	0	0	0	not significant
2549	SUZUKI_AMPLIFIED_IN_ORAL_CANCER	-1.18	0.54	0	0	0	not significant
2550	GO_SCHWANN_CELL_PROLIFERATION	-1.18	0.53	0	0	0	not significant
2551	BIOCARTA_P35ALZHEIMERS_PATHWAY	-1.18	0.52	0	0	0	not significant
2552	GO_AMIDE_BIOSYNTHETIC_PROCESS	-1.17	4	0	0	0	not significant
2553	GO_INTRACELLULAR_TRANSPORT	-1.17	4	0	0	0	not significant
2554	SENESE_HDAC3_TARGETS_UP	-1.17	1.52	0	0	0	not significant
2555	GO_NUCLEAR_CHROMOSOME	-1.17	1.49	0	0	0	not significant
2556	GO_CHROMATIN	-1.17	1.4	0	0	0	not significant
2557	SMID_BREAST_CANCER_BASAL_UP	-1.17	1.28	0	0	0	not significant
2558	DEBIASI_APOPTOSIS_BY_REOVIRUS_INFECTION_UP	-1.17	1.18	0	0	0	not significant
2559	PURBEY_TARGETS_OF_CTBP1_NOT_SATB1_UP	-1.17	1.12	0	0	0	not significant
2560	GO_DNA_BIOSYNTHETIC_PROCESS	-1.17	1.04	0	0	0	not significant
2561	FUJII_YBX1_TARGETS_DN	-1.17	1.01	0	0	0	not significant
2562	SANSOM_APC_MYC_TARGETS	-1.17	1	0	0	0	not significant
2563	SENESE_HDAC1_AND_HDAC2_TARGETS_UP	-1.17	0.95	0	0	0	not significant
2564	GO_CIRCADIAN_RHYTHM	-1.17	0.91	0	0	0	not significant
2565	KAAB_FAILED_HEART_ATRIUM_DN	-1.17	0.91	0	0	0	not significant
2566	GO_MICROTUBULE_ORGANIZING_CENTER_ORGANIZATION	-1.17	0.9	0	0	0	not significant
2567	GO_POSITIVE_REGULATION_OF_CHROMOSOME_ORGANIZATION	-1.17	0.89	0	0	0	not significant
2568	GO_TELOMERE_ORGANIZATION	-1.17	0.89	0	0	0	not significant
2569	PENG_GLUCOSE_DEPRIVATION_DN	-1.17	0.89	0	0	0	not significant
2570	FIGUEROA_AML_METHYLATION_CLUSTER_3_UP	-1.17	0.84	0	0	0	not significant
2571	GO_RNA_POLYMERASE_II_DISTAL_ENHANCER_SEQUENCE_SPECIFIC_DNA_BINDING	-1.17	0.84	0	0	0	not significant
2572	SHEN_SMARCA2_TARGETS_DN	-1.17	0.83	0	0	0	not significant
2573	KAYO_AGING_MUSCLE_DN	-1.17	0.76	0	0	0	not significant
2574	GO_REGULATION_OF_MUSCLE_CONTRACTION	-1.17	0.75	0	0	0	not significant
2575	REACTOME_NONHOMOLOGOUS_END_JOINING_NHEJ	-1.17	0.73	0	0	0	not significant
2576	GO_CELLULAR_RESPONSE_TO_UV	-1.17	0.72	0	0	0	not significant
2577	PID_TAP63_PATHWAY	-1.17	0.71	0	0	0	not significant
2578	ZAMORA_NOS2_TARGETS_DN	-1.17	0.71	0	0	0	not significant
2579	REN_BOUND_BY_E2F	-1.17	0.7	0	0	0	not significant
2580	JAZAERI_BREAST_CANCER_BRCA1_VS_BRCA2_UP	-1.17	0.68	0	0	0	not significant
2581	GO_POSITIVE_REGULATION_OF_FIBROBLAST_PROLIFERATION	-1.17	0.67	0	0	0	not significant
2582	JAERVINEN_AMPLIFIED_IN_LARYNGEAL_CANCER	-1.17	0.66	0	0	0	not significant
2583	PID_ECADHERIN_STABILIZATION_PATHWAY	-1.17	0.66	0	0	0	not significant
2584	GO_NEUROTROPHIN_SIGNALING_PATHWAY	-1.17	0.65	0	0	0	not significant
2585	GO_REGULATION_OF_INTRINSIC_APOPTOTIC_SIGNALING_PATHWAY_BY_P53_CLAS	-1.17	0.65	0	0	0	not significant
2586	GO_REGULATION_OF_STRIATED_MUSCLE_CONTRACTION	-1.17	0.65	0	0	0	not significant
2587	GO_VESICLE_COAT	-1.17	0.65	0	0	0	not significant
2588	PID_WNT_CANONICAL_PATHWAY	-1.17	0.65	0	0	0	not significant
2589	GO_INNER_MITOCHONDRIAL_MEMBRANE_ORGANIZATION	-1.17	0.64	0	0	0	not significant
2590	GO_POSITIVE_REGULATION_OF_POSTTRANSCRIPTIONAL_GENE_SILENCING	-1.17	0.63	0	0	0	not significant
2591	GO_PROTEASOMAL_UBIQUITIN_INDEPENDENT_PROTEIN_CATABOLIC_PROCESS	-1.17	0.63	0	0	0	not significant
2592	CHESLER_BRAIN_HIGHEST_GENETIC_VARIANCE	-1.17	0.62	0	0	0	not significant
2593	GO_POSITIVE_REGULATION_OF_MUSCLE_CELL_APOPTOTIC_PROCESS	-1.17	0.62	0	0	0	not significant
2594	SIMBULAN_UV_RESPONSE_NORMAL_DN	-1.17	0.61	0	0	0	not significant
2595	GO_GLIAL_CELL_APOPTOTIC_PROCESS	-1.17	0.59	0	0	0	not significant
2596	GO_REGULATION_OF_GRANULOCYTE_CHEMOTAXIS	-1.17	0.59	0	0	0	not significant
2597	GRATIAS_RETINOBLASTOMA_16Q24	-1.17	0.59	0	0	0	not significant
2598	BIOCARTA_PML_PATHWAY	-1.17	0.58	0	0	0	not significant
2599	GO_EMBRYONIC_CAMERA_TYPE_EYE_DEVELOPMENT	-1.17	0.58	0	0	0	not significant
2600	GO_REGULATION_OF_TRANSCRIPTION_INITIATION_FROM_RNA_POLYMERASE_II_PI	-1.17	0.58	0	0	0	not significant
2601	WU_HBX_TARGETS_2_DN	-1.17	0.58	0	0	0	not significant
2602	GO_CELLULAR_RESPONSE_TO_REACTIVE_NITROGEN_SPECIES	-1.17	0.57	0	0	0	not significant
2603	GO_PLATELET_MORPHOGENESIS	-1.17	0.57	0	0	0	not significant
2604	GO_REGULATION_OF_SMOOTHENED_SIGNALING_PATHWAY_INVOLVED_IN_DORSAI	-1.17	0.57	0	0	0	not significant

2605	GO_SIGNAL_TRANSDUCTION_INVOLVED_IN_G2_DNA_DAMAGE_CHECKPOINT	-1.17	0.57	0	0	0	not significant
2606	GO_VITAMIN_D_RECEPTOR_BINDING	-1.17	0.57	0	0	0	not significant
2607	LY_AGING_PREMATURE_DN	-1.17	0.57	0	0	0	not significant
2608	GO_LACTATE_METABOLIC_PROCESS	-1.17	0.55	0	0	0	not significant
2609	GO_NEGATIVE_REGULATION_OF_CIRCADIAN_RHYTHM	-1.17	0.55	0	0	0	not significant
2610	GO_RESPONSE_TO_DOPAMINE	-1.17	0.55	0	0	0	not significant
2611	REACTOME_ACTIVATION_OF_BAD_AND_TRANSLOCATION_TO_MITOCHONDRIA	-1.17	0.55	0	0	0	not significant
2612	GO_INSULIN_LIKE_GROWTH_FACTOR_RECEPTOR_BINDING	-1.17	0.54	0	0	0	not significant
2613	GO_OXIDOREDUCTASE_ACTIVITY_ACTING_ON_THE_CH_CH_GROUP_OF_DONORS	-1.17	0.54	0	0	0	not significant
2614	KEGG_ASCORBATE_AND_ALDARATE_METABOLISM	-1.17	0.54	0	0	0	not significant
2615	GO_NEGATIVE_REGULATION_OF_RYANODINE_SENSITIVE_CALCIUM_RELEASE_CHA	-1.17	0.53	0	0	0	not significant
2616	GO_RNA_POLYMERASE_III_TRANSCRIPTION_FACTOR_COMPLEX	-1.17	0.53	0	0	0	not significant
2617	GO_POSTTRANSLATIONAL_PROTEIN_TARGETING_TO_ENDOPLASMIC_RETICULUM_	-1.17	0.52	0	0	0	not significant
2618	BIOCARTA_BARRESTIN_PATHWAY	-1.17	0.51	0	0	0	not significant
2619	GO_AMP_BIOSYNTHETIC_PROCESS	-1.17	0.51	0	0	0	not significant
2620	GO_TORC1_COMPLEX	-1.17	0.51	0	0	0	not significant
2621	GO_MODULATION_OF_SIGNAL_TRANSDUCTION_IN_OTHER_ORGANISM	-1.17	0.5	0	0	0	not significant
2622	PALOMERO_GSI_SENSITIVITY_UP	-1.17	0.49	0	0	0	not significant
2623	GO_PROTEIN_CATABOLIC_PROCESS	-1.16	1.82	0	0	0	not significant
2624	DANG_BOUND_BY_MYC	-1.16	1.72	0	0	0	not significant
2625	GO_MITOCHONDRION_ORGANIZATION	-1.16	1.58	0	0	0	not significant
2626	FLECHNER_BIOPSY_KIDNEY_TRANSPLANT_REJECTED_VS_OK_DN	-1.16	1.44	0	0	0	not significant
2627	SHEN_SMARCA2_TARGETS_UP	-1.16	1.43	0	0	0	not significant
2628	GO_ACTIVATION_OF_INNATE_IMMUNE_RESPONSE	-1.16	1.03	0	0	0	not significant
2629	SENGUPTA_NASOPHARYNGEAL_CARCINOMA_UP	-1.16	1.01	0	0	0	not significant
2630	GO_INTERACTION_WITH_HOST	-1.16	0.93	0	0	0	not significant
2631	GO_MAINTENANCE_OF_CELL_NUMBER	-1.16	0.91	0	0	0	not significant
2632	REACTOME_ESTROGEN_DEPENDENT_GENE_EXPRESSION	-1.16	0.88	0	0	0	not significant
2633	GO_NEGATIVE_REGULATION_OF_NERVOUS_SYSTEM_DEVELOPMENT	-1.16	0.85	0	0	0	not significant
2634	GO_VESICLE_TARGETING_TO_FROM_OR_WITHIN_GOLGI	-1.16	0.79	0	0	0	not significant
2635	GO_POSITIVE_REGULATION_OF_MITOTIC_CELL_CYCLE	-1.16	0.77	0	0	0	not significant
2636	KEGG_NEUROTROPHIN_SIGNALING_PATHWAY	-1.16	0.76	0	0	0	not significant
2637	GO_REGULATION_OF_CENTROSOME_CYCLE	-1.16	0.72	0	0	0	not significant
2638	RB_P130_DN.V1_UP	-1.16	0.72	0	0	0	not significant
2639	GO_NUCLEOSIDE_TRIPHOSPHATE_METABOLIC_PROCESS	-1.16	0.69	0	0	0	not significant
2640	GO_MEIOTIC_CHROMOSOME_SEGREGATION	-1.16	0.68	0	0	0	not significant
2641	GO_MATERNAL_PROCESS_INVOLVED_IN_FEMALE_PREGNANCY	-1.16	0.67	0	0	0	not significant
2642	BIOCARTA_NFAT_PATHWAY	-1.16	0.64	0	0	0	not significant
2643	GO_POSITIVE_REGULATION_OF_TYROSINE_PHOSPHORYLATION_OF_STAT_PROTE	-1.16	0.64	0	0	0	not significant
2644	REACTOME_DNA_DAMAGE_RECOGNITION_IN_GG_NER	-1.16	0.64	0	0	0	not significant
2645	BIOCARTA_MTOR_PATHWAY	-1.16	0.63	0	0	0	not significant
2646	GO_ESTROGEN_RECEPTOR_BINDING	-1.16	0.63	0	0	0	not significant
2647	GO_CIS_TRANS_ISOMERASE_ACTIVITY	-1.16	0.62	0	0	0	not significant
2648	SCHAEFFER_PROSTATE_DEVELOPMENT_AND_CANCER_BOX4_DN	-1.16	0.62	0	0	0	not significant
2649	REACTOME_GASTRIN_CREB_SIGNALLING_PATHWAY_VIA_PKC_AND_MAPK	-1.16	0.6	0	0	0	not significant
2650	BIOCARTA_STATHMIN_PATHWAY	-1.16	0.59	0	0	0	not significant
2651	GO_NEGATIVE_REGULATION_OF_CELL_MATRIX_ADHESION	-1.16	0.59	0	0	0	not significant
2652	GO_NUCLEAR_PORE_ORGANIZATION	-1.16	0.58	0	0	0	not significant
2653	GO_POSITIVE_REGULATION_OF_TRANSLATIONAL_INITIATION	-1.16	0.58	0	0	0	not significant
2654	GO_CALMODULIN_DEPENDENT_PROTEIN_KINASE_ACTIVITY	-1.16	0.57	0	0	0	not significant
2655	GO_DENDRITE_EXTENSION	-1.16	0.57	0	0	0	not significant
2656	GO_PURINE_NUCLEOSIDE_MONOPHOSPHATE_METABOLIC_PROCESS	-1.16	0.57	0	0	0	not significant
2657	PETROVA_PROX1_TARGETS_UP	-1.16	0.57	0	0	0	not significant
2658	GO_TRANSLATION_PREINITIATION_COMPLEX	-1.16	0.56	0	0	0	not significant
2659	GO_ENDOLYSOSOME_MEMBRANE	-1.16	0.55	0	0	0	not significant
2660	GO_MAMMARY_GLAND_DUCT_MORPHOGENESIS	-1.16	0.55	0	0	0	not significant
2661	REACTOME_ESTABLISHMENT_OF_SISTER_CHROMATID_COHESION	-1.16	0.55	0	0	0	not significant
2662	GO_DETECTION_OF_TEMPERATURE_STIMULUS	-1.16	0.54	0	0	0	not significant
2663	GO_REGULATION_OF_ATP_BIOSYNTHETIC_PROCESS	-1.16	0.54	0	0	0	not significant
2664	BACOLOD_RESISTANCE_TO_ALKYLATING_AGENTS_UP	-1.16	0.53	0	0	0	not significant
2665	GO_HISTONE_H2B_UBIQUITINATION	-1.16	0.53	0	0	0	not significant
2666	GO_VITAMIN_TRANSMEMBRANE_TRANSPORT	-1.16	0.53	0	0	0	not significant
2667	REACTOME_COHESIN_LOADING_ONTO_CHROMATIN	-1.16	0.53	0	0	0	not significant
2668	ZEMBUTSU_SENSITIVITY_TO_DOXORUBICIN	-1.16	0.53	0	0	0	not significant
2669	BIOCARTA_RNAPOL3_PATHWAY	-1.16	0.52	0	0	0	not significant
2670	GO_MATING_BEHAVIOR	-1.16	0.52	0	0	0	not significant
2671	BIOCARTA_CCR3_PATHWAY	-1.16	0.51	0	0	0	not significant
2672	GO_F_ACTIN_CAPPING_PROTEIN_COMPLEX	-1.16	0.51	0	0	0	not significant
2673	GO_MITOCHONDRIAL_RESPIRATORY_CHAIN_COMPLEX_III_ASSEMBLY	-1.16	0.5	0	0	0	not significant
2674	GO_NEGATIVE_REGULATION_OF_AUTOPHAGOSOME_ASSEMBLY	-1.16	0.5	0	0	0	not significant
2675	GO_NEGATIVE_REGULATION_OF_MYELINATION	-1.16	0.5	0	0	0	not significant
2676	GO_NOSE_DEVELOPMENT	-1.16	0.5	0	0	0	not significant
2677	GO_POSITIVE_REGULATION_OF_MACROPHAGE_CHEMOTAXIS	-1.16	0.5	0	0	0	not significant
2678	GO_INTRACELLULAR_DISTRIBUTION_OF_MITOCHONDRIA	-1.16	0.49	0	0	0	not significant
2679	GO_STEREOCILIUM_TIP	-1.16	0.49	0	0	0	not significant
2680	GO_POSTTRANSLATIONAL_PROTEIN_TARGETING_TO_MEMBRANE_TRANSLOCATIC	-1.16	0.48	0	0	0	not significant
2681	BENPORATH_NANOG_TARGETS	-1.15	4	0	0	0	not significant
2682	GO_POSITIVE_REGULATION_OF_GENE_EXPRESSION	-1.15	4	0	0	0	not significant
2683	GO_POSITIVE_REGULATION_OF_PROTEIN_METABOLIC_PROCESS	-1.15	4	0	0	0	not significant
2684	GRYDER_PAX3FOXO1_ENHANCERS_IN_TADS	-1.15	1.83	0	0	0	not significant
2685	GO_NEGATIVE_REGULATION_OF_TRANSCRIPTION_BY_RNA_POLYMERASE_II	-1.15	1.67	0	0	0	not significant
2686	GO_KINASE_ACTIVITY	-1.15	1.49	0	0	0	not significant
2687	GO_GUANYL_NUCLEOTIDE_BINDING	-1.15	1.12	0	0	0	not significant
2688	GO_UBIQUITIN_LIKE_PROTEIN_LIGASE_BINDING	-1.15	1.06	0	0	0	not significant
2689	HALLMARK_OXIDATIVE_PHOSPHORYLATION	-1.15	1.04	0	0	0	not significant
2690	GO_POSITIVE_REGULATION_OF_NERVOUS_SYSTEM_DEVELOPMENT	-1.15	1.03	0	0	0	not significant
2691	GO_FOREBRAIN_DEVELOPMENT	-1.15	0.96	0	0	0	not significant
2692	TBK1.DF_DN	-1.15	0.95	0	0	0	not significant
2693	GO_RHYTHMIC_PROCESS	-1.15	0.91	0	0	0	not significant
2694	KUMAR_PATHOGEN_LOAD_BY_MACROPHAGES	-1.15	0.91	0	0	0	not significant
2695	GO_PHOSPHATASE_ACTIVITY	-1.15	0.89	0	0	0	not significant
2696	GO_TRANSFERASE_ACTIVITY_TRANSFERRING_ONE_CARBON_GROUPS	-1.15	0.89	0	0	0	not significant
2697	GO_PROTEIN_TETRAMERIZATION	-1.15	0.86	0	0	0	not significant
2698	GO_POSITIVE_REGULATION_OF_CELLULAR_PROTEIN_CATABOLIC_PROCESS	-1.15	0.82	0	0	0	not significant
2699	GO_ATP_SYNTHESIS_COUPLED_ELECTRON_TRANSPORT	-1.15	0.74	0	0	0	not significant
2700	GO_CELL_AGING	-1.15	0.73	0	0	0	not significant
2701	GO_METAL_CLUSTER_BINDING	-1.15	0.73	0	0	0	not significant
2702	QUINTENS_EMBRYONIC_BRAIN_RESPONSE_TO_IR	-1.15	0.71	0	0	0	not significant
2703	GO_HEART_GROWTH	-1.15	0.7	0	0	0	not significant
2704	SAKAI_TUMOR_INFILTRATING_MONOCYTES_DN	-1.15	0.7	0	0	0	not significant
2705	GO_SIGNAL_TRANSDUCTION_INVOLVED_IN_CELL_CYCLE_CHECKPOINT	-1.15	0.69	0	0	0	not significant
2706	CHR16P12	-1.15	0.67	0	0	0	not significant
2707	REACTOME_TRANSCRIPTIONAL_REGULATION_OF_WHITE_ADIPOCYTE_DIFFERENTI	-1.15	0.67	0	0	0	not significant
2708	REACTOME_REGULATION_OF_MECP2_EXPRESSION_AND_ACTIVITY	-1.15	0.63	0	0	0	not significant
2709	GO_NEGATIVE_REGULATION_OF_CELL_SUBSTRATE_ADHESION	-1.15	0.62	0	0	0	not significant
2710	REACTOME_GLYCOGEN_METABOLISM	-1.15	0.62	0	0	0	not significant
2711	GO_PHOSPHATIDYLINOSITOL_3_KINASE_COMPLEX	-1.15	0.6	0	0	0	not significant
2712	GO_INTRA_GOLGI_VESICLE_MEDIATED_TRANSPORT	-1.15	0.59	0	0	0	not significant
2713	BIOCARTA_INTEGRIN_PATHWAY	-1.15	0.58	0	0	0	not significant

2714	GO_SNRNA_3_END_PROCESSING	-1.15	0.58	0	0	0	not significant
2715	REACTOME_DISASSEMBLY_OF_THE_DESTRUCTION_COMPLEX_AND_RECRUITMENT	-1.15	0.58	0	0	0	not significant
2716	GO_POSITIVE_REGULATION_OF_LAMELLIPODIUM_ASSEMBLY	-1.15	0.57	0	0	0	not significant
2717	GO_ACTIVATION_OF_ADENYLATE_CYCLASE_ACTIVITY	-1.15	0.55	0	0	0	not significant
2718	GO_POSITIVE_REGULATION_OF_CYTOKINE_PRODUCTION_INVOLVED_IN_INFLAMMA	-1.15	0.54	0	0	0	not significant
2719	GU_PDEF_TARGETS_DN	-1.15	0.54	0	0	0	not significant
2720	MORI_EMU_MYC_LYMPHOMA_BY_ONSET_TIME_DN	-1.15	0.54	0	0	0	not significant
2721	REACTOME_MITOCHONDRIAL_IRON_SULFUR_CLUSTER_BIOGENESIS	-1.15	0.54	0	0	0	not significant
2722	GO_CLATHRIN_COATED_ENDOCYTIC_VESICLE_MEMBRANE	-1.15	0.53	0	0	0	not significant
2723	GO_HAIR_FOLLICLE_MATURATION	-1.15	0.53	0	0	0	not significant
2724	GO_RENAL_SYSTEM_VASCULATURE_DEVELOPMENT	-1.15	0.53	0	0	0	not significant
2725	REACTOME_E2F_ENABLED_INHIBITION_OF_PRE_REPLICATION_COMPLEX_FORMAT	-1.15	0.53	0	0	0	not significant
2726	GO_DETECTION_OF_TEMPERATURE_STIMULUS_INVOLVED_IN_SENSORY_PERCEPT	-1.15	0.52	0	0	0	not significant
2727	GO_LEUCINE_ZIPPER_DOMAIN_BINDING	-1.15	0.52	0	0	0	not significant
2728	GO_NEGATIVE_REGULATION_OF_MRNA_3_END_PROCESSING	-1.15	0.52	0	0	0	not significant
2729	GO_RNA_POLYMERASE_I_CORE_BINDING	-1.15	0.52	0	0	0	not significant
2730	GO_EMBRYONIC_BODY_MORPHOGENESIS	-1.15	0.51	0	0	0	not significant
2731	GO_PEPTIDE_ALPHA_N_ACETYLTRANSFERASE_ACTIVITY	-1.15	0.51	0	0	0	not significant
2732	SCHAEFFER_PROSTATE_DEVELOPMENT_AND_CANCER_BOX4_UP	-1.15	0.51	0	0	0	not significant
2733	GO_MONOUBIQUITINATED_PROTEIN_DEUBIQUITINATION	-1.15	0.5	0	0	0	not significant
2734	GO_OUTER_MITOCHONDRIAL_MEMBRANE_PROTEIN_COMPLEX	-1.15	0.5	0	0	0	not significant
2735	BIOCARTA_RAB_PATHWAY	-1.15	0.49	0	0	0	not significant
2736	GO_MRNA_5_SPLICE_SITE_RECOGNITION	-1.15	0.49	0	0	0	not significant
2737	GO_POSITIVE_REGULATION_OF_CARDIAC_MUSCLE_CELL_PROLIFERATION	-1.15	0.49	0	0	0	not significant
2738	GO_PROSTATE_GLAND_MORPHOGENESIS	-1.15	0.49	0	0	0	not significant
2739	GO_REGULATION_OF_MIRNA_METABOLIC_PROCESS	-1.15	0.49	0	0	0	not significant
2740	GO_TRNA_SPLICING_LIGASE_COMPLEX	-1.15	0.49	0	0	0	not significant
2741	LANDEMAINE_LUNG_METASTASIS	-1.15	0.49	0	0	0	not significant
2742	GO_CARBOHYDRATE_PHOSPHATASE_ACTIVITY	-1.15	0.48	0	0	0	not significant
2743	GO_CELLULAR_RESPONSE_TO_BRAIN_DERIVED_NEUROTROPHIC_FACTOR_STIMUI	-1.15	0.48	0	0	0	not significant
2744	GO_LIPASE_ACTIVATOR_ACTIVITY	-1.15	0.48	0	0	0	not significant
2745	GO_PHOSPHOLIPASE_ACTIVATOR_ACTIVITY	-1.15	0.48	0	0	0	not significant
2746	GO_REGULATION_OF_CALCIUM_TRANSPORTING_ATPASE_ACTIVITY	-1.15	0.48	0	0	0	not significant
2747	REACTOME_CASPASE_ACTIVATION_VIA_DEPENDENCE_RECEPTORS_IN_THE_ABSE	-1.15	0.48	0	0	0	not significant
2748	GO_BASEMENT_MEMBRANE_ASSEMBLY	-1.15	0.45	0	0	0	not significant
2749	GO_MITOCHONDRION	-1.14	4	0	0	0	not significant
2750	REACTOME_DISEASE	-1.14	1.76	0	0	0	not significant
2751	GO_POSITIVE_REGULATION_OF_PHOSPHORUS_METABOLIC_PROCESS	-1.14	1.73	0	0	0	not significant
2752	GO_POSITIVE_REGULATION_OF_CATALYTIC_ACTIVITY	-1.14	1.7	0	0	0	not significant
2753	GO_REGULATION_OF_PROTEOLYSIS	-1.14	1.26	0	0	0	not significant
2754	GO_REGULATION_OF_CELL_DEVELOPMENT	-1.14	1.18	0	0	0	not significant
2755	CHARAFE_BREAST_CANCER_LUMINAL_VS_MESENCHYMAL_DN	-1.14	1.05	0	0	0	not significant
2756	GO_POST_TRANSLATIONAL_PROTEIN_MODIFICATION	-1.14	0.91	0	0	0	not significant
2757	REACTOME_MITOTIC_G1_G1_S_PHASES	-1.14	0.83	0	0	0	not significant
2758	GO_NUCLEAR_TRANSCRIPTION_FACTOR_COMPLEX	-1.14	0.82	0	0	0	not significant
2759	GO_NEGATIVE_REGULATION_OF_NUCLEOBASE_CONTAINING_COMPOUND_METABO	-1.14	0.79	0	0	0	not significant
2760	REACTOME_DNA_REPLICATION_PRE_INITIATION	-1.14	0.75	0	0	0	not significant
2761	GO_MEIOTIC_CELL_CYCLE	-1.14	0.74	0	0	0	not significant
2762	CHR1P35	-1.14	0.68	0	0	0	not significant
2763	GO_POSITIVE_REGULATION_OF_UBIQUITIN_DEPENDENT_PROTEIN_CATABOLIC_PR	-1.14	0.68	0	0	0	not significant
2764	GO_REGULATION_OF_PROTEIN_LOCALIZATION_TO_PLASMA_MEMBRANE	-1.14	0.68	0	0	0	not significant
2765	GO_RESPONSE_TO_ALKALOID	-1.14	0.68	0	0	0	not significant
2766	REACTOME_PI_METABOLISM	-1.14	0.68	0	0	0	not significant
2767	SESTO_RESPONSE_TO_UV_C7	-1.14	0.68	0	0	0	not significant
2768	ZHAN_V2_LATE_DIFFERENTIATION_GENES	-1.14	0.67	0	0	0	not significant
2769	FONTAINE_FOLLICULAR_THYROID_ADENOMA_DN	-1.14	0.64	0	0	0	not significant
2770	GO_PROTEIN_TRANSPORT_ALONG_MICROTUBULE	-1.14	0.64	0	0	0	not significant
2771	GO_RESPONSE_TO_AMMONIUM_ION	-1.14	0.63	0	0	0	not significant
2772	BIOCARTA_TNFR1_PATHWAY	-1.14	0.6	0	0	0	not significant
2773	GO_NUCLEOSIDE_MONOPHOSPHATE_BIOSYNTHETIC_PROCESS	-1.14	0.6	0	0	0	not significant
2774	GO_POSITIVE_REGULATION_OF_VIRAL_LIFE_CYCLE	-1.14	0.6	0	0	0	not significant
2775	GO_ASTROCYTE_DIFFERENTIATION	-1.14	0.59	0	0	0	not significant
2776	LIN_MELANOMA_COPY_NUMBER_DN	-1.14	0.59	0	0	0	not significant
2777	GO_CEREBELLAR_CORTEX_DEVELOPMENT	-1.14	0.58	0	0	0	not significant
2778	GO_DYNEIN_COMPLEX	-1.14	0.58	0	0	0	not significant
2779	GO_ENDOCHONDRAL_BONE_MORPHOGENESIS	-1.14	0.58	0	0	0	not significant
2780	SNIJDERS_AMPLIFIED_IN_HEAD_AND_NECK_TUMORS	-1.14	0.57	0	0	0	not significant
2781	GO_EPITHELIAL_CELL_DIFFERENTIATION_INVOLVED_IN_KIDNEY_DEVELOPMENT	-1.14	0.56	0	0	0	not significant
2782	WHITEHURST_PACLITAXEL_SENSITIVITY	-1.14	0.56	0	0	0	not significant
2783	YIH_RESPONSE_TO_ARSENITE_C3	-1.14	0.56	0	0	0	not significant
2784	GO_MITOCHONDRIAL_CALCIUM_ION_TRANSMEMBRANE_TRANSPORT	-1.14	0.55	0	0	0	not significant
2785	GO_NURD_COMPLEX	-1.14	0.55	0	0	0	not significant
2786	GO_PHOSPHATIDYLINOSITOL_3_KINASE_REGULATOR_ACTIVITY	-1.14	0.55	0	0	0	not significant
2787	GO_CELLULAR_RESPONSE_TO_PROSTAGLANDIN_STIMULUS	-1.14	0.54	0	0	0	not significant
2788	PID_SYNDECAN_4_PATHWAY	-1.14	0.54	0	0	0	not significant
2789	REACTOME_VEGFR2_MEDIATED_CELL_PROLIFERATION	-1.14	0.54	0	0	0	not significant
2790	GO_REGULATION_OF_PLATELET_ACTIVATION	-1.14	0.53	0	0	0	not significant
2791	KEGG_BETA_ALANINE_METABOLISM	-1.14	0.53	0	0	0	not significant
2792	CHR14Q31	-1.14	0.52	0	0	0	not significant
2793	GO_GROWTH_CONE_PART	-1.14	0.52	0	0	0	not significant
2794	GO_NUCLEAR_CYCLIN_DEPENDENT_PROTEIN_KINASE_HOLOENZYME_COMPLEX	-1.14	0.52	0	0	0	not significant
2795	CHANG_POU5F1_TARGETS_DN	-1.14	0.51	0	0	0	not significant
2796	GO_CLATHRIN_COMPLEX	-1.14	0.51	0	0	0	not significant
2797	GO_EPITHELIAL_CELL_CELL_ADHESION	-1.14	0.51	0	0	0	not significant
2798	GO_NEUROTRANSMITTER_CATABOLIC_PROCESS	-1.14	0.51	0	0	0	not significant
2799	PARK_HSC_VS_MULTIPOTENT_PROGENITORS_DN	-1.14	0.51	0	0	0	not significant
2800	RAMPON_ENRICHED_LEARNING_ENVIRONMENT_EARLY_DN	-1.14	0.51	0	0	0	not significant
2801	REACTOME_SEMA3A_PAK_DEPENDENT_AXON_REPULSION	-1.14	0.51	0	0	0	not significant
2802	BIOCARTA_HIF_PATHWAY	-1.14	0.5	0	0	0	not significant
2803	REACTOME_MECP2_REGULATES_NEURONAL_RECEPTORS_AND_CHANNELS	-1.14	0.5	0	0	0	not significant
2804	SCHURINGA_STAT5A_TARGETS_UP	-1.14	0.5	0	0	0	not significant
2805	GO_FOREBRAIN_NEURON_DEVELOPMENT	-1.14	0.49	0	0	0	not significant
2806	GO_LAMELLIPODIUM_MORPHOGENESIS	-1.14	0.49	0	0	0	not significant
2807	GO_POSITIVE_REGULATION_OF_ATP_BIOSYNTHETIC_PROCESS	-1.14	0.49	0	0	0	not significant
2808	GO_RETINOID_X_RECEPTOR_BINDING	-1.14	0.49	0	0	0	not significant
2809	GO_CYTIDYLYLTRANSFERASE_ACTIVITY	-1.14	0.48	0	0	0	not significant
2810	GO_ECTODERMAL_PLACODE_DEVELOPMENT	-1.14	0.48	0	0	0	not significant
2811	GO_CENTRIOLAR_SUBDISTAL_APPENDAGE	-1.14	0.47	0	0	0	not significant
2812	GO_POSTSYNAPTIC_MODULATION_OF_CHEMICAL_SYNAPTIC_TRANSMISSION	-1.14	0.47	0	0	0	not significant
2813	GO_BLASTODERM_SEGMENTATION	-1.14	0.45	0	0	0	not significant
2814	GO_SATELLITE_DNA_BINDING	-1.14	0.44	0	0	0	not significant
2815	GO_REGULATION_OF_PHOSPHORUS_METABOLIC_PROCESS	-1.13	1.55	0	0	0	not significant
2816	DOUGLAS_BMI1_TARGETS_UP	-1.13	1.12	0	0	0	not significant
2817	GABRIELY_MIR21_TARGETS	-1.13	1.11	0	0	0	not significant
2818	GO_REGULATION_OF_GENE_EXPRESSION_EPIGENETIC	-1.13	1.05	0	0	0	not significant
2819	GO_REGULATION_OF_NEURON_DIFFERENTIATION	-1.13	1.03	0	0	0	not significant
2820	VANTVEER_BREAST_CANCER_ESR1_DN	-1.13	0.86	0	0	0	not significant
2821	REACTOME_S_PHASE	-1.13	0.81	0	0	0	not significant
2822	SHEPARD_BMYB_MORPHOLINO_DN	-1.13	0.77	0	0	0	not significant

2823	DARWICHE_SQUAMOUS_CELL_CARCINOMA_UP	-1.13	0.76	0	0	0	not significant
2824	HALLMARK_UNFOLDED_PROTEIN_RESPONSE	-1.13	0.76	0	0	0	not significant
2825	HOSHIDA_LIVER_CANCER_SUBCLASS_S2	-1.13	0.72	0	0	0	not significant
2826	GO_ISOMERASE_ACTIVITY	-1.13	0.71	0	0	0	not significant
2827	GO_REGULATION_OF_RECEPTOR_MEDIATED_ENDOCYTOSIS	-1.13	0.67	0	0	0	not significant
2828	BYSTRYKH_HEMATOPOIESIS_STEM_CELL_AND_BRAIN_QTL_TRANS	-1.13	0.66	0	0	0	not significant
2829	GO_COPII_COATED_VESICLE_BUDDING	-1.13	0.66	0	0	0	not significant
2830	NABA_SECRETED_FACTORS	-1.13	0.66	0	0	0	not significant
2831	WANG_ESOPHAGUS_CANCER_VS_NORMAL_DN	-1.13	0.65	0	0	0	not significant
2832	PID_ATR_PATHWAY	-1.13	0.64	0	0	0	not significant
2833	WNT_SIGNALING	-1.13	0.64	0	0	0	not significant
2834	PID_ERBB1_INTERNALIZATION_PATHWAY	-1.13	0.63	0	0	0	not significant
2835	SHEDDEN_LUNG_CANCER_GOOD_SURVIVAL_A5	-1.13	0.63	0	0	0	not significant
2836	GO_MESODERM_DEVELOPMENT	-1.13	0.62	0	0	0	not significant
2837	GO_REGULATION_OF_PROTEIN_IMPORT	-1.13	0.62	0	0	0	not significant
2838	GO_RECEPTOR_INTERNALIZATION	-1.13	0.61	0	0	0	not significant
2839	GO_RNA_POLYMERASE_BINDING	-1.13	0.61	0	0	0	not significant
2840	GO_MYELIN_SHEATH	-1.13	0.6	0	0	0	not significant
2841	PID_SHP2_PATHWAY	-1.13	0.59	0	0	0	not significant
2842	GO_MIRNA_BINDING	-1.13	0.58	0	0	0	not significant
2843	BIOCARTA_MEF2D_PATHWAY	-1.13	0.55	0	0	0	not significant
2844	GO OXIDOREDUCTASE ACTIVITY ACTING ON THE ALDEHYDE OR OXO GROUP_	-1.13	0.55	0	0	0	not significant
2845	HOFMANN_MYELODYSPLASTIC_SYNDROM_LOW_RISK_UP	-1.13	0.54	0	0	0	not significant
2846	GO_NEGATIVE_REGULATION_OF_SMOOTHENED_SIGNALING_PATHWAY	-1.13	0.53	0	0	0	not significant
2847	GO_PRE_MRNA_BINDING	-1.13	0.53	0	0	0	not significant
2848	PID_CDC42_REG_PATHWAY	-1.13	0.53	0	0	0	not significant
2849	GO_POSITIVE_REGULATION_OF_PHOSPHATASE_ACTIVITY	-1.13	0.52	0	0	0	not significant
2850	MARCINIAK_ER_STRESS_RESPONSE_VIA_CHOP	-1.13	0.52	0	0	0	not significant
2851	GO_NEURONAL_STEM_CELL_POPULATION_MAINTENANCE	-1.13	0.51	0	0	0	not significant
2852	GO_REGULATION_OF_DIGESTIVE_SYSTEM_PROCESS	-1.13	0.51	0	0	0	not significant
2853	GO_REGULATION_OF_RIBONUCLEASE_ACTIVITY	-1.13	0.51	0	0	0	not significant
2854	GO_GOLGI_LOCALIZATION	-1.13	0.49	0	0	0	not significant
2855	MAINA_VHL_TARGETS_DN	-1.13	0.49	0	0	0	not significant
2856	KEGG_NON_HOMOLOGOUS_END_JOINING	-1.13	0.48	0	0	0	not significant
2857	CHRXQ11	-1.13	0.47	0	0	0	not significant
2858	GO_AXONEMAL_DYNEIN_COMPLEX_ASSEMBLY	-1.13	0.47	0	0	0	not significant
2859	GO_PURINE_NUCLEOSIDE_DIPHOSPHATE_BIOSYNTHETIC_PROCESS	-1.13	0.47	0	0	0	not significant
2860	GO_REGULATION_OF_HISTONE_UBIQUITINATION	-1.13	0.47	0	0	0	not significant
2861	HAHTOLA_MYCOSIS_FUNGOIDES_UP	-1.13	0.47	0	0	0	not significant
2862	GO_ALDEHYDE_BIOSYNTHETIC_PROCESS	-1.13	0.46	0	0	0	not significant
2863	GO_INTRAMOLECULAR_TRANSFERASE_ACTIVITY_PHOSPHOTRANSFERASES	-1.13	0.46	0	0	0	not significant
2864	GO_POSITIVE_REGULATION_OF_GONAD_DEVELOPMENT	-1.13	0.46	0	0	0	not significant
2865	GO_REGULATION_OF_LOW_DENSITY_LIPOPROTEIN_PARTICLE_CLEARANCE	-1.13	0.46	0	0	0	not significant
2866	REACTOME_MET_RECEPTOR_RECYCLING	-1.13	0.46	0	0	0	not significant
2867	GO_REGULATION_OF_HIGH_VOLTAGE_GATED_CALCIUM_CHANNEL_ACTIVITY	-1.13	0.45	0	0	0	not significant
2868	GO_RIBOSOMAL_PROTEIN_S6_KINASE_ACTIVITY	-1.13	0.45	0	0	0	not significant
2869	GO_TRANSLATION_ACTIVATOR_ACTIVITY	-1.13	0.45	0	0	0	not significant
2870	GO_TRNA_PSEUDOURIDINE_SYNTHESIS	-1.13	0.45	0	0	0	not significant
2871	GO_REGULATION_OF_PROTEIN_LOCALIZATION_TO_CHROMATIN	-1.13	0.44	0	0	0	not significant
2872	MATZUK_EMBRYONIC_GERM_CELL	-1.13	0.44	0	0	0	not significant
2873	GO_REGULATION_OF_T_CIRCLE_FORMATION	-1.13	0.43	0	0	0	not significant
2874	REACTOME RUNX1 REGULATES TRANSCRIPTION OF GENES INVOLVED IN DIFFE	-1.13	0.43	0	0	0	not significant
2875	GO_POSITIVE_REGULATION_OF_PROTEIN_MODIFICATION_PROCESS	-1.12	1.85	0	0	0	not significant
2876	GO_MOLECULAR_FUNCTION_REGULATOR	-1.12	1.73	0	0	0	not significant
2877	GO_REGULATION_OF_TRANSFERASE_ACTIVITY	-1.12	1.44	0	0	0	not significant
2878	REACTOME_SIGNALING_BY_INTERLEUKINS	-1.12	1.03	0	0	0	not significant
2879	GO_NEGATIVE_REGULATION_OF_PHOSPHORYLATION	-1.12	0.98	0	0	0	not significant
2880	GO_ENZYME_ACTIVATOR_ACTIVITY	-1.12	0.95	0	0	0	not significant
2881	GO_POSITIVE_REGULATION_OF_CELLULAR_COMPONENT_BIOGENESIS	-1.12	0.9	0	0	0	not significant
2882	GO_CYTOSOLIC_PART	-1.12	0.89	0	0	0	not significant
2883	FARMER_BREAST_CANCER_APOCRINE_VS_BASAL	-1.12	0.78	0	0	0	not significant
2884	GO_POSITIVE_REGULATION_OF_GROWTH	-1.12	0.75	0	0	0	not significant
2885	GO_T_CELL_RECEPTOR_SIGNALING_PATHWAY	-1.12	0.74	0	0	0	not significant
2886	ESC_J1_UP_LATE.V1_DN	-1.12	0.71	0	0	0	not significant
2887	GARGALOVIC_RESPONSE_TO_OXIDIZED_PHOSPHOLIPIDS_BLUE_UP	-1.12	0.69	0	0	0	not significant
2888	GO_NUCLEOSOME_BINDING	-1.12	0.68	0	0	0	not significant
2889	GO_MUSCLE_CELL_PROLIFERATION	-1.12	0.67	0	0	0	not significant
2890	GO_UBIQUITIN_LIKE_PROTEIN_SPECIFIC_PROTEASE_ACTIVITY	-1.12	0.67	0	0	0	not significant
2891	GO_STRIATED_MUSCLE_CONTRACTION	-1.12	0.64	0	0	0	not significant
2892	GO_CELLULAR_RESPONSE_TO_LIGHT_STIMULUS	-1.12	0.61	0	0	0	not significant
2893	GO_REGULATION_OF_MICROTUBULE_POLYMERIZATION_OR_DEPOLYMERIZATION	-1.12	0.6	0	0	0	not significant
2894	PID_AR_PATHWAY	-1.12	0.6	0	0	0	not significant
2895	GO_METENCEPHALON_DEVELOPMENT	-1.12	0.59	0	0	0	not significant
2896	GO_OOGENESIS	-1.12	0.59	0	0	0	not significant
2897	GO_REGULATION_OF_ADHERENS_JUNCTION_ORGANIZATION	-1.12	0.59	0	0	0	not significant
2898	GO_REGULATION_OF_CELLULAR_RESPONSE_TO_TRANSFORMING_GROWTH_FAC	-1.12	0.59	0	0	0	not significant
2899	GO_ORGANIC_HYDROXY_COMPOUND_CATABOLIC_PROCESS	-1.12	0.58	0	0	0	not significant
2900	POMEROY_MEDULLOBLASTOMA_DESMOPLASIC_VS_CLASSIC_UP	-1.12	0.57	0	0	0	not significant
2901	GO_ADULT_LOCOMOTORY_BEHAVIOR	-1.12	0.56	0	0	0	not significant
2902	GO_MICROTUBULE_BUNDLE_FORMATION	-1.12	0.56	0	0	0	not significant
2903	GO_RESPONSE_TO_ESTROGEN	-1.12	0.56	0	0	0	not significant
2904	CHR19P12	-1.12	0.55	0	0	0	not significant
2905	GO_KINESIN_BINDING	-1.12	0.55	0	0	0	not significant
2906	GO POSITIVE REGULATION OF SUBSTRATE ADHESION DEPENDENT CELL SPRE	-1.12	0.55	0	0	0	not significant
2907	BEIER_GLIOMA_STEM_CELL_UP	-1.12	0.54	0	0	0	not significant
2908	GO_ALCOHOL_CATABOLIC_PROCESS	-1.12	0.53	0	0	0	not significant
2909	GO_LIVER_REGENERATION	-1.12	0.53	0	0	0	not significant
2910	GO_POSITIVE_REGULATION_OF_RECEPTOR_MEDIATED_ENDOCYTOSIS	-1.12	0.53	0	0	0	not significant
2911	GO_STEM_CELL_DIVISION	-1.12	0.53	0	0	0	not significant
2912	WU_HBX_TARGETS_3_UP	-1.12	0.53	0	0	0	not significant
2913	BIOCARTA_CREB_PATHWAY	-1.12	0.52	0	0	0	not significant
2914	CHR2P15	-1.12	0.52	0	0	0	not significant
2915	GO_CYTOKINETIC_PROCESS	-1.12	0.52	0	0	0	not significant
2916	GO_DNA_BINDING_BENDING	-1.12	0.51	0	0	0	not significant
2917	GO_SYNAPTONEMAL_COMPLEX_ORGANIZATION	-1.12	0.51	0	0	0	not significant
2918	GO_PYRIMIDINE_RIBONUCLEOTIDE_BIOSYNTHETIC_PROCESS	-1.12	0.5	0	0	0	not significant
2919	GO_TAU_PROTEIN_KINASE_ACTIVITY	-1.12	0.5	0	0	0	not significant
2920	WILLERT_WNT_SIGNALING	-1.12	0.5	0	0	0	not significant
2921	WHITE_NEUROBLASTOMA_WITH_1P36.3_DELETION	-1.12	0.49	0	0	0	not significant
2922	BIOCARTA_GSK3_PATHWAY	-1.12	0.48	0	0	0	not significant
2923	BOYERINAS_ONCOFETAL_TARGETS_OF_LET7A1	-1.12	0.48	0	0	0	not significant
2924	GO_TRNA_TRANSCRIPTION	-1.12	0.48	0	0	0	not significant
2925	BIOCARTA_IRES_PATHWAY	-1.12	0.47	0	0	0	not significant
2926	CHR5Q34	-1.12	0.47	0	0	0	not significant
2927	GO_CAMP_DEPENDENT_PROTEIN_KINASE_COMPLEX	-1.12	0.47	0	0	0	not significant
2928	GO_CELLULAR_RESPONSE_TO_PROSTAGLANDIN_E_STIMULUS	-1.12	0.47	0	0	0	not significant
2929	GO_EXOCYST	-1.12	0.47	0	0	0	not significant
2930	GO_NEGATIVE_REGULATION_BY_HOST_OF_VIRAL_TRANSCRIPTION	-1.12	0.47	0	0	0	not significant
2931	GO_NEGATIVE_REGULATION_OF_CALCIUM_ION_DEPENDENT_EXOCYTOSIS	-1.12	0.46	0	0	0	not significant

2932	GO_S100_PROTEIN_BINDING	-1.12	0.46	0	0	0	not significant
2933	REACTOME_NONCANONICAL_ACTIVATION_OF_NOTCH3	-1.12	0.46	0	0	0	not significant
2934	GO_EMBRYONIC_VISCEROCRANIUM_MORPHOGENESIS	-1.12	0.45	0	0	0	not significant
2935	GO_LONG_CHAIN_FATTY_ACID_TRANSPORTER_ACTIVITY	-1.12	0.45	0	0	0	not significant
2936	GO_MRE11_COMPLEX	-1.12	0.45	0	0	0	not significant
2937	GO_NEGATIVE_REGULATION_OF_MEGAKARYOCYTE_DIFFERENTIATION	-1.12	0.45	0	0	0	not significant
2938	WU_APOPTOSIS_BY_CDKN1A_NOT_VIA_TP53	-1.12	0.45	0	0	0	not significant
2939	GO_CDP_CHOLINE_PATHWAY	-1.12	0.44	0	0	0	not significant
2940	GO_GAS_HOMEOSTASIS	-1.12	0.44	0	0	0	not significant
2941	GO_REGULATION_OF_HISTONE_H3_K36_METHYLATION	-1.12	0.44	0	0	0	not significant
2942	REACTOME_OAS_ANTIVIRAL_RESPONSE	-1.12	0.44	0	0	0	not significant
2943	GO_TETRAHYDROFOLATE_BIOSYNTHETIC_PROCESS	-1.12	0.43	0	0	0	not significant
2944	REACTOME_RUNX3_REGULATES_P14_ARF	-1.12	0.43	0	0	0	not significant
2945	GO_POSITIVE_REGULATION_OF_MOLECULAR_FUNCTION	-1.11	4	0	0	0	not significant
2946	NUYTTEN_EZH2_TARGETS_DN	-1.11	1.73	0	0	0	not significant
2947	GO_PEPTIDYL_AMINO_ACID_MODIFICATION	-1.11	1.57	0	0	0	not significant
2948	GO_ENVELOPE	-1.11	1.32	0	0	0	not significant
2949	PUJANA_BRCA2_PCC_NETWORK	-1.11	1.19	0	0	0	not significant
2950	STARK_PREFRONTAL_CORTEX_22Q11_DELETION_DN	-1.11	1.01	0	0	0	not significant
2951	GO_POSITIVE_REGULATION_OF_KINASE_ACTIVITY	-1.11	0.85	0	0	0	not significant
2952	REACTOME_SIGNALING_BY_RECEPTOR_TYROSINE_KINASES	-1.11	0.84	0	0	0	not significant
2953	KAAB_HEART_ATRIUM_VS_VENTRICLE_DN	-1.11	0.82	0	0	0	not significant
2954	GO_ENERGY_DERIVATION_BY_OXIDATION_OF_ORGANIC_COMPOUNDS	-1.11	0.79	0	0	0	not significant
2955	GO_PROTEIN_POLYMERIZATION	-1.11	0.79	0	0	0	not significant
2956	LEI_MYB_TARGETS	-1.11	0.78	0	0	0	not significant
2957	GO_PROTEIN_LOCALIZATION_TO_CELL_PERIPHERY	-1.11	0.76	0	0	0	not significant
2958	GO_VIRAL_GENE_EXPRESSION	-1.11	0.76	0	0	0	not significant
2959	GO_RNA_POLYMERASE_II_TRANSCRIPTION_FACTOR_COMPLEX	-1.11	0.73	0	0	0	not significant
2960	CYCLIN_D1_KE_.V1_UP	-1.11	0.68	0	0	0	not significant
2961	GO_VESICLE_TARGETING	-1.11	0.67	0	0	0	not significant
2962	GO_UROGENITAL_SYSTEM_DEVELOPMENT	-1.11	0.66	0	0	0	not significant
2963	MCBRYAN_PUBERTAL_BREAST_4_5WK_DN	-1.11	0.65	0	0	0	not significant
2964	DARWICHE_PAPILLOMA_RISK_HIGH_UP	-1.11	0.64	0	0	0	not significant
2965	GO_VESICLE_BUDDING_FROM_MEMBRANE	-1.11	0.63	0	0	0	not significant
2966	GO_ENSHEATHMENT_OF_NEURONS	-1.11	0.6	0	0	0	not significant
2967	GO_HISTONE_METHYLTRANSFERASE_ACTIVITY	-1.11	0.59	0	0	0	not significant
2968	GO_PROTEIN_LOCALIZATION_TO_CYTOSKELETON	-1.11	0.59	0	0	0	not significant
2969	REACTOME_TGF_BETA_RECEPTOR_SIGNALING_ACTIVATES_SMADS	-1.11	0.59	0	0	0	not significant
2970	GO_GDP_BINDING	-1.11	0.57	0	0	0	not significant
2971	GO_MICROTUBULE_POLYMERIZATION	-1.11	0.57	0	0	0	not significant
2972	GO_POSITIVE_REGULATION_OF_TRANSMEMBRANE_RECEPTOR_PROTEIN_SERINE_	-1.11	0.57	0	0	0	not significant
2973	TCGA_GLIOBLASTOMA_COPY_NUMBER_UP	-1.11	0.57	0	0	0	not significant
2974	LIAO_HAVE_SOX4_BINDING_SITES	-1.11	0.56	0	0	0	not significant
2975	PARK_HSC_AND_MULTIPOTENT_PROGENITORS	-1.11	0.56	0	0	0	not significant
2976	REACTOME_COPII_MEDIATED_VESICLE_TRANSPORT	-1.11	0.55	0	0	0	not significant
2977	CHIARETTI_T_ALL_REFRACTORY_TO_THERAPY	-1.11	0.54	0	0	0	not significant
2978	GO_MYD88_DEPENDENT_TOLL_LIKE_RECEPTOR_SIGNALING_PATHWAY	-1.11	0.53	0	0	0	not significant
2979	GO_POSITIVE_REGULATION_OF_PROTEIN_IMPORT	-1.11	0.53	0	0	0	not significant
2980	PID_ALPHA_SYNUCLEIN_PATHWAY	-1.11	0.53	0	0	0	not significant
2981	GO_CYSTEINE_TYPE_ENDOPEPTIDASE_INHIBITOR_ACTIVITY	-1.11	0.52	0	0	0	not significant
2982	GO_NEGATIVE_REGULATION_OF_NOTCH_SIGNALING_PATHWAY	-1.11	0.52	0	0	0	not significant
2983	GO_PEPTIDE_TRANSMEMBRANE_TRANSPORTER_ACTIVITY	-1.11	0.52	0	0	0	not significant
2984	REACTOME_DOWNREGULATION_OF_TGF_BETA_RECEPTOR_SIGNALING	-1.11	0.52	0	0	0	not significant
2985	BIOCARTA_VEGF_PATHWAY	-1.11	0.51	0	0	0	not significant
2986	GO_POSITIVE_REGULATION_OF_INTERFERON_ALPHA_PRODUCTION	-1.11	0.51	0	0	0	not significant
2987	GO_POSITIVE_REGULATION_OF_TISSUE_REMODELING	-1.11	0.51	0	0	0	not significant
2988	PID_DELTA_NP63_PATHWAY	-1.11	0.51	0	0	0	not significant
2989	GO_MRNA_METHYLATION	-1.11	0.5	0	0	0	not significant
2990	GO_POSITIVE_REGULATION_OF_EPIDERMIS_DEVELOPMENT	-1.11	0.5	0	0	0	not significant
2991	GO_OOCYTE_MATURATION	-1.11	0.49	0	0	0	not significant
2992	GO_REGULATION_OF_INTRINSIC_APOPTOTIC_SIGNALING_PATHWAY_IN_RESPONSE	-1.11	0.49	0	0	0	not significant
2993	GO_ANTIBIOTIC_CATABOLIC_PROCESS	-1.11	0.48	0	0	0	not significant
2994	GO_NEGATIVE_REGULATION_OF_COAGULATION	-1.11	0.48	0	0	0	not significant
2995	GO_NEGATIVE_REGULATION_OF_PEPTIDYL_SERINE_PHOSPHORYLATION	-1.11	0.48	0	0	0	not significant
2996	GO_BRANCHING_INVOLVED_IN_MAMMARY_GLAND_DUCT_MORPHOGENESIS	-1.11	0.47	0	0	0	not significant
2997	GO_DNA_INTEGRATION	-1.11	0.47	0	0	0	not significant
2998	GO_GLUCAN_CATABOLIC_PROCESS	-1.11	0.47	0	0	0	not significant
2999	GO_POSITIVE_REGULATION_OF_NUCLEOTIDE_BIOSYNTHETIC_PROCESS	-1.11	0.47	0	0	0	not significant
3000	REACTOME_CHREBP_ACTIVATES_METABOLIC_GENE_EXPRESSION	-1.11	0.46	0	0	0	not significant
3001	GO_LONG_CHAIN_FATTY_ACID_COA_LIGASE_ACTIVITY	-1.11	0.45	0	0	0	not significant
3002	IRITANI_MAD1_TARGETS_UP	-1.11	0.45	0	0	0	not significant
3003	REACTOME_WNT5A_DEPENDENT_INTERNALIZATION_OF_FZD2_FZD5_AND_ROR2	-1.11	0.45	0	0	0	not significant
3004	GO_MOTILE_CILIUM_ASSEMBLY	-1.11	0.44	0	0	0	not significant
3005	GO_POSITIVE_REGULATION_OF_TYPE_I_INTERFERON_MEDIATED_SIGNALING_PATI	-1.11	0.44	0	0	0	not significant
3006	GO_RESPONSE_TO_EPINEPHRINE	-1.11	0.44	0	0	0	not significant
3007	KEGG_CIRCADIAN_RHYTHM_MAMMAL	-1.11	0.44	0	0	0	not significant
3008	ZEILSTRA_CD44_TARGETS_DN	-1.11	0.44	0	0	0	not significant
3009	GO_NSL_COMPLEX	-1.11	0.43	0	0	0	not significant
3010	GO_OXIDIZED_BASE_LESION_DNA_N_GLYCOSYLASE_ACTIVITY	-1.11	0.41	0	0	0	not significant
3011	GO_CELLULAR_MACROMOLECULE_LOCALIZATION	-1.10	4	0	0	0	not significant
3012	GO_MICROTUBULE_ORGANIZING_CENTER	-1.10	1.11	0	0	0	not significant
3013	REACTOME_TRANSCRIPTIONAL_REGULATION_BY_TP53	-1.10	0.92	0	0	0	not significant
3014	GO_PROTEIN_HETERODIMERIZATION_ACTIVITY	-1.10	0.83	0	0	0	not significant
3015	REACTOME_CHROMATIN_ORGANIZATION	-1.10	0.75	0	0	0	not significant
3016	GO_CENTRIOLE	-1.10	0.65	0	0	0	not significant
3017	GO_NEGATIVE_REGULATION_OF_INTRINSIC_APOPTOTIC_SIGNALING_PATHWAY	-1.10	0.64	0	0	0	not significant
3018	GO_LARGE_RIBOSOMAL_SUBUNIT	-1.10	0.62	0	0	0	not significant
3019	GO_NEGATIVE_REGULATION_OF_PROTEIN_BINDING	-1.10	0.62	0	0	0	not significant
3020	MCBRYAN_PUBERTAL_BREAST_5_6WK_DN	-1.10	0.62	0	0	0	not significant
3021	GO_CELLULAR_COMPONENT_ASSEMBLY_INVOLVED_IN_MORPHOGENESIS	-1.10	0.61	0	0	0	not significant
3022	GO_MUSCLE_CELL_DEVELOPMENT	-1.10	0.61	0	0	0	not significant
3023	GO_NEGATIVE_REGULATION_OF_MYELOID_CELL_DIFFERENTIATION	-1.10	0.59	0	0	0	not significant
3024	BRUECKNER_TARGETS_OF_MIRLET7A3_DN	-1.10	0.58	0	0	0	not significant
3025	GO_REGULATION_OF_DNA_TEMPLATED_TRANSCRIPTION_ELONGATION	-1.10	0.58	0	0	0	not significant
3026	IVANOVA_HEMATOPOIESIS_INTERMEDIATE_PROGENITOR	-1.10	0.57	0	0	0	not significant
3027	GO_NEPHRON_DEVELOPMENT	-1.10	0.56	0	0	0	not significant
3028	MULLIGHAN_NPM1_MUTATED_SIGNATURE_2_DN	-1.10	0.56	0	0	0	not significant
3029	GO_MEIOSIS_I_CELL_CYCLE_PROCESS	-1.10	0.53	0	0	0	not significant
3030	GO_POSITIVE_REGULATION_OF_DNA_BIOSYNTHETIC_PROCESS	-1.10	0.53	0	0	0	not significant
3031	GOTZMANN_EPITHELIAL_TO_MESENCHYMAL_TRANSITION_UP	-1.10	0.52	0	0	0	not significant
3032	MARIADASON_REGULATED_BY_HISTONE_ACETYLATION_DN	-1.10	0.52	0	0	0	not significant
3033	MILI_PSEUDOPODIA	-1.10	0.52	0	0	0	not significant
3034	REACTOME_SIGNALING_BY_TYPE_1_INSULIN_LIKE_GROWTH_FACTOR_1_RECEPTC	-1.10	0.52	0	0	0	not significant
3035	GUO_HEX_TARGETS_DN	-1.10	0.51	0	0	0	not significant
3036	HOELZEL_NF1_TARGETS_UP	-1.10	0.51	0	0	0	not significant
3037	SIMBULAN_UV_RESPONSE_IMMORTALIZED_DN	-1.10	0.51	0	0	0	not significant
3038	GO_INO80_TYPE_COMPLEX	-1.10	0.5	0	0	0	not significant
3039	KEGG_MELANOMA	-1.10	0.5	0	0	0	not significant
3040	REACTOME_CD28_DEPENDENT_PI3K_AKT_SIGNALING	-1.10	0.5	0	0	0	not significant

3041	AZARE_STAT3_TARGETS	-1.10	0.48	0	0	0	not significant
3042	GO_INOSITOL_PHOSPHATE_MEDIATED_SIGNALING	-1.10	0.48	0	0	0	not significant
3043	YAMASHITA_LIVER_CANCER_STEM_CELL_UP	-1.10	0.48	0	0	0	not significant
3044	GO_PHOSPHATIDYLINOSITOL_MONOPHOSPHATE_PHOSPHATASE_ACTIVITY	-1.10	0.47	0	0	0	not significant
3045	GO_POSITIVE_REGULATION_OF_MITOCHONDRIAL_TRANSLATION	-1.10	0.47	0	0	0	not significant
3046	PID_SMAD2_3PATHWAY	-1.10	0.47	0	0	0	not significant
3047	REACTOME_DEPOLYMERISATION_OF_THE_NUCLEAR_LAMINA	-1.10	0.47	0	0	0	not significant
3048	KORKOLA_YOLK_SAC_TUMOR_UP	-1.10	0.46	0	0	0	not significant
3049	GO_ACTIVIN_BINDING	-1.10	0.45	0	0	0	not significant
3050	GO_CYTOSKELETAL_ANCHORING_AT_PLASMA_MEMBRANE	-1.10	0.45	0	0	0	not significant
3051	GO_STARTLE_RESPONSE	-1.10	0.44	0	0	0	not significant
3052	PEPPER_CHRONIC_LYMPHOCYTIC_LEUKEMIA_DN	-1.10	0.44	0	0	0	not significant
3053	REACTOME_PROTEIN_METHYLATION	-1.10	0.44	0	0	0	not significant
3054	GO_CELLULAR_HYPEROSMOTIC_RESPONSE	-1.10	0.43	0	0	0	not significant
3055	GO_INTERLEUKIN_5_PRODUCTION	-1.10	0.43	0	0	0	not significant
3056	GO_PHOSPHOSERINE_RESIDUE_BINDING	-1.10	0.43	0	0	0	not significant
3057	GO_SECRETORY_GRANULE_LOCALIZATION	-1.10	0.43	0	0	0	not significant
3058	GO_UMP_BIOSYNTHETIC_PROCESS	-1.10	0.43	0	0	0	not significant
3059	GO_XYLULOSE_5_PHOSPHATE_BIOSYNTHETIC_PROCESS	-1.10	0.43	0	0	0	not significant
3060	GO_ACTIN_CROSSLINK_FORMATION	-1.10	0.42	0	0	0	not significant
3061	GO_POSITIVE_REGULATION_OF_PROTEIN_HOMODIMERIZATION_ACTIVITY	-1.10	0.42	0	0	0	not significant
3062	GO_RESPONSE_TO_STIMULUS_INVOLVED_IN_REGULATION_OF_MUSCLE_ADAPTAT	-1.10	0.42	0	0	0	not significant
3063	GO_SPERM_CHROMATIN_CONDENSATION	-1.10	0.42	0	0	0	not significant
3064	GO_TRANSCRIPTION_FACTOR_TFIIH_CORE_COMPLEX	-1.10	0.42	0	0	0	not significant
3065	GO_TRANSCRIPTIONAL_PREINITIATION_COMPLEX	-1.10	0.42	0	0	0	not significant
3066	REACTOME_FORMATION_OF_XYLULOSE_5_PHOSPHATE	-1.10	0.42	0	0	0	not significant
3067	THUM_MIR21_TARGETS_HEART_DISEASE_DN	-1.10	0.42	0	0	0	not significant
3068	GO_REGULATION_OF_GLYCOGEN_CATABOLIC_PROCESS	-1.10	0.41	0	0	0	not significant
3069	GO_REGULATION_OF_LIPOPROTEIN_PARTICLE_CLEARANCE	-1.10	0.41	0	0	0	not significant
3070	GO_REGULATION_OF_MESODERM_DEVELOPMENT	-1.10	0.41	0	0	0	not significant
3071	GO_UBIQUINONE_BINDING	-1.10	0.41	0	0	0	not significant
3072	PETRETTO_BLOOD_PRESSURE_UP	-1.10	0.41	0	0	0	not significant
3073	GO_REGULATION_OF_CARBOHYDRATE_METABOLIC_PROCESS_BY_REGULATION_(	-1.10	0.4	0	0	0	not significant
3074	TONKS_TARGETS_OF_RUNX1_RUNX1T1_FUSION_SUSTAINED_IN_MONOCYTE_DN	-1.10	0.4	0	0	0	not significant
3075	GO_CERAMIDE_1_PHOSPHATE_BINDING	-1.10	0.39	0	0	0	not significant
3076	GO_COVALENT_CHROMATIN_MODIFICATION	-1.09	1.01	0	0	0	not significant
3077	OSMAN_BLADDER_CANCER_UP	-1.09	0.89	0	0	0	not significant
3078	ZWANG_CLASS_1_TRANSIENTLY_INDUCED_BY_EGF	-1.09	0.79	0	0	0	not significant
3079	GO_UBIQUITIN_LIGASE_COMPLEX	-1.09	0.75	0	0	0	not significant
3080	RAY_TUMORIGENESIS_BY_ERBB2_CDC25A_DN	-1.09	0.71	0	0	0	not significant
3081	REACTOME_NEURONAL_SYSTEM	-1.09	0.69	0	0	0	not significant
3082	GO_REGULATION_OF_PROTEIN_BINDING	-1.09	0.66	0	0	0	not significant
3083	GO_RIBONUCLEOPROTEIN_GRANULE	-1.09	0.66	0	0	0	not significant
3084	GO_CHROMATIN_ASSEMBLY	-1.09	0.63	0	0	0	not significant
3085	GO_PROTEIN_C_TERMINUS_BINDING	-1.09	0.63	0	0	0	not significant
3086	GO_STRIATED_MUSCLE_CELL_DIFFERENTIATION	-1.09	0.63	0	0	0	not significant
3087	CHR11P15	-1.09	0.62	0	0	0	not significant
3088	GO_PEPTIDE_BINDING	-1.09	0.59	0	0	0	not significant
3089	HALLMARK_PROTEIN_SECRETION	-1.09	0.59	0	0	0	not significant
3090	REACTOME_RECRUITMENT_OF_NUMA_TO_MITOTIC_CENTROSOMES	-1.09	0.59	0	0	0	not significant
3091	GO_RIBOSE_PHOSPHATE_BIOSYNTHETIC_PROCESS	-1.09	0.58	0	0	0	not significant
3092	GO_CHANNEL_REGULATOR_ACTIVITY	-1.09	0.56	0	0	0	not significant
3093	GO_CHROMATIN_REMODELING_AT_CENTROMERE	-1.09	0.55	0	0	0	not significant
3094	GO_FIBROBLAST_GROWTH_FACTOR_RECEPTOR_SIGNALING_PATHWAY	-1.09	0.55	0	0	0	not significant
3095	HALLMARK_REACTIVE_OXYGEN_SPECIES_PATHWAY	-1.09	0.55	0	0	0	not significant
3096	REACTOME_SIGNALING_BY_TGF_BETA_FAMILY_MEMBERS	-1.09	0.55	0	0	0	not significant
3097	GO_CYCLIN_DEPENDENT_PROTEIN_KINASE_HOLOENZYME_COMPLEX	-1.09	0.53	0	0	0	not significant
3098	YAGI_AML_WITH_T_9_11_TRANSLOCATION	-1.09	0.53	0	0	0	not significant
3099	GO_STEM_CELL_PROLIFERATION	-1.09	0.52	0	0	0	not significant
3100	GO_TISSUE_REGENERATION	-1.09	0.52	0	0	0	not significant
3101	GO_TIGHT_JUNCTION_ORGANIZATION	-1.09	0.51	0	0	0	not significant
3102	NADLER_HYPERGLYCEMIA_AT_OBESITY	-1.09	0.51	0	0	0	not significant
3103	PID_RET_PATHWAY	-1.09	0.51	0	0	0	not significant
3104	GO_POSITIVE_REGULATION_OF_CYCLIN_DEPENDENT_PROTEIN_KINASE_ACTIVITY	-1.09	0.5	0	0	0	not significant
3105	GO_REGULATION_OF_GASTRULATION	-1.09	0.5	0	0	0	not significant
3106	GO_REGULATION_OF_TRANSCRIPTION_ELONGATION_FROM_RNA_POLYMERASE_II	-1.09	0.5	0	0	0	not significant
3107	GO_NEUROBLAST_PROLIFERATION	-1.09	0.49	0	0	0	not significant
3108	HALMOS_CEBPA_TARGETS_DN	-1.09	0.49	0	0	0	not significant
3109	SIG_REGULATION_OF_THE_ACTIN_CYTOSKELETON_BY_RHO_GTPASES	-1.09	0.49	0	0	0	not significant
3110	KIM_TIAL1_TARGETS	-1.09	0.48	0	0	0	not significant
3111	GARGALOVIC_RESPONSE_TO_OXIDIZED_PHOSPHOLIPIDS_YELLOW_DN	-1.09	0.47	0	0	0	not significant
3112	KEGG_DRUG_METABOLISM_OTHER_ENZYMES	-1.09	0.47	0	0	0	not significant
3113	BIOCARTA_CK1_PATHWAY	-1.09	0.46	0	0	0	not significant
3114	BIOCARTA_PITX2_PATHWAY	-1.09	0.46	0	0	0	not significant
3115	CHR12P12	-1.09	0.46	0	0	0	not significant
3116	GO_CELLULAR_RESPONSE_TO_VITAMIN_D	-1.09	0.46	0	0	0	not significant
3117	GO_NEGATIVE_REGULATION_OF_VIRAL_TRANSCRIPTION	-1.09	0.46	0	0	0	not significant
3118	GO_REGULATION_OF_MITOTIC_SPINDLE_ASSEMBLY	-1.09	0.46	0	0	0	not significant
3119	GO_TRANSLATION_REPRESSOR_ACTIVITY_MRNA_REGULATORY_ELEMENT_BINDIN	-1.09	0.46	0	0	0	not significant
3120	LIU_BREAST_CANCER	-1.09	0.46	0	0	0	not significant
3121	REACTOME_BIOTIN_TRANSPORT_AND_METABOLISM	-1.09	0.45	0	0	0	not significant
3122	GO_METANEPHRIC_MESENCHYME_DEVELOPMENT	-1.09	0.44	0	0	0	not significant
3123	REACTOME_SYNTHESIS_OF_PE	-1.09	0.44	0	0	0	not significant
3124	GO_DECIDUALIZATION	-1.09	0.43	0	0	0	not significant
3125	GO_MESENCHYMAL_TO_EPITHELIAL_TRANSITION	-1.09	0.43	0	0	0	not significant
3126	GO_POSITIVE_REGULATION_OF_G_PROTEIN_COUPLED_RECEPTOR_SIGNALING_PA	-1.09	0.43	0	0	0	not significant
3127	GO_POSITIVE_REGULATION_OF_INTERLEUKIN_5_PRODUCTION	-1.09	0.43	0	0	0	not significant
3128	REACTOME_PKA_MEDIATED_PHOSPHORYLATION_OF_CREB	-1.09	0.43	0	0	0	not significant
3129	GO_WIDE_PORE_CHANNEL_ACTIVITY	-1.09	0.42	0	0	0	not significant
3130	REACTOME_RUNX3_REGULATES_WNT_SIGNALING	-1.09	0.42	0	0	0	not significant
3131	BIOCARTA_TERC_PATHWAY	-1.09	0.41	0	0	0	not significant
3132	KRISHNAN_FURIN_TARGETS_DN	-1.09	0.41	0	0	0	not significant
3133	RAFFEL_VEGFA_TARGETS_UP	-1.09	0.41	0	0	0	not significant
3134	GO_METANEPHRIC_RENAL_VESICLE_MORPHOGENESIS	-1.09	0.4	0	0	0	not significant
3135	GO_NEURON_FATE_SPECIFICATION	-1.09	0.4	0	0	0	not significant
3136	GO_PARENTAL_BEHAVIOR	-1.09	0.4	0	0	0	not significant
3137	MUNSHI_MULTIPLE_MYELOMA_DN	-1.09	0.4	0	0	0	not significant
3138	GO_CARDIOBLAST_DIFFERENTIATION	-1.09	0.37	0	0	0	not significant
3139	GO_PROTEOLYSIS	-1.08	1.23	0	0	0	not significant
3140	GEORGES_TARGETS_OF_MIR192_AND_MIR215	-1.08	1	0	0	0	not significant
3141	GO_REGULATION_OF_PROTEIN_MODIFICATION_PROCESS	-1.08	0.92	0	0	0	not significant
3142	GO_ANATOMICAL_STRUCTURE_HOMEOSTASIS	-1.08	0.74	0	0	0	not significant
3143	GO_POSITIVE_REGULATION_OF_ORGANELLE_ORGANIZATION	-1.08	0.74	0	0	0	not significant
3144	GO_CELL_CELL_SIGNALING_BY_WNT	-1.08	0.73	0	0	0	not significant
3145	GO_POSITIVE_REGULATION_OF_CELL_DEVELOPMENT	-1.08	0.73	0	0	0	not significant
3146	GO_REGULATION_OF_INNATE_IMMUNE_RESPONSE	-1.08	0.7	0	0	0	not significant
3147	REACTOME_CILIUM_ASSEMBLY	-1.08	0.63	0	0	0	not significant
3148	CHIANG_LIVER_CANCER_SUBCLASS_UNANNOTATED_DN	-1.08	0.6	0	0	0	not significant
3149	REACTOME_RESPIRATORY_ELECTRON_TRANSPORT	-1.08	0.59	0	0	0	not significant

3150	GO_PROTEIN_ACETYLTRANSFERASE_COMPLEX	-1.08	0.58	0	0	0	not significant
3151	WAMUNYOKOLI_OVARIAN_CANCER_LMP_DN	-1.08	0.58	0	0	0	not significant
3152	REACTOME_DNA_REPLICATION	-1.08	0.55	0	0	0	not significant
3153	REACTOME_SIGNALING_BY_ERBB2	-1.08	0.55	0	0	0	not significant
3154	LIU_NASOPHARYNGEAL_CARCINOMA	-1.08	0.53	0	0	0	not significant
3155	MOOTHA_VOXPHOS	-1.08	0.53	0	0	0	not significant
3156	RB_P107_DN.V1_DN	-1.08	0.53	0	0	0	not significant
3157	GO_POSITIVE_REGULATION_OF_CARDIAC_MUSCLE_TISSUE_DEVELOPMENT	-1.08	0.52	0	0	0	not significant
3158	GENTILE_RESPONSE_CLUSTER_D3	-1.08	0.51	0	0	0	not significant
3159	GO_NEURON_MIGRATION	-1.08	0.51	0	0	0	not significant
3160	GO_REGULATION_OF_NEUROLOGICAL_SYSTEM_PROCESS	-1.08	0.51	0	0	0	not significant
3161	GO_SIGNAL_TRANSDUCTION_IN_ABSENCE_OF_LIGAND	-1.08	0.51	0	0	0	not significant
3162	REACTOME_NUCLEOSOME_ASSEMBLY	-1.08	0.51	0	0	0	not significant
3163	CHR2Q14	-1.08	0.5	0	0	0	not significant
3164	CHR8P21	-1.08	0.5	0	0	0	not significant
3165	GO_9PLUS2_MOTILE_CILIUM	-1.08	0.5	0	0	0	not significant
3166	GO_BLASTOCYST_DEVELOPMENT	-1.08	0.5	0	0	0	not significant
3167	GO_POSITIVE_REGULATION_OF_CATION_CHANNEL_ACTIVITY	-1.08	0.5	0	0	0	not significant
3168	GO_PROTEIN_K63_LINKED_UBIQUITINATION	-1.08	0.5	0	0	0	not significant
3169	GO_UBIQUITIN_LIKE_PROTEIN_BINDING	-1.08	0.5	0	0	0	not significant
3170	GO_ENDOPLASMIC_RETICULUM_ORGANIZATION	-1.08	0.49	0	0	0	not significant
3171	GO_STRUCTURAL_CONSTITUENT_OF_CYTOSKELETON	-1.08	0.49	0	0	0	not significant
3172	TANAKA_METHYLATED_IN_ESOPHAGEAL_CARCINOMA	-1.08	0.49	0	0	0	not significant
3173	GO_MAIN_AXON	-1.08	0.48	0	0	0	not significant
3174	GO_NEGATIVE_REGULATION_OF_PROTEASOMAL_UBIQUITIN_DEPENDENT_PROTEI	-1.08	0.48	0	0	0	not significant
3175	GO_REGULATION_OF_MICROTUBULE_POLYMERIZATION	-1.08	0.48	0	0	0	not significant
3176	MARTIN_INTERACT_WITH_HDAC	-1.08	0.47	0	0	0	not significant
3177	REACTOME_CARGO_CONCENTRATION_IN_THE_ER	-1.08	0.47	0	0	0	not significant
3178	GO_LUNG_MORPHOGENESIS	-1.08	0.46	0	0	0	not significant
3179	TURASHVILI_BREAST_LOBULAR_CARCINOMA_VS_DUCTAL_NORMAL_DN	-1.08	0.46	0	0	0	not significant
3180	GO_CUL4_RING_E3_UBIQUITIN_LIGASE_COMPLEX	-1.08	0.45	0	0	0	not significant
3181	GO_GLANDULAR_EPITHELIAL_CELL_DEVELOPMENT	-1.08	0.45	0	0	0	not significant
3182	GO_SYNAPTONEMAL_STRUCTURE	-1.08	0.45	0	0	0	not significant
3183	KEGG_STARCH_AND_SUCROSE_METABOLISM	-1.08	0.45	0	0	0	not significant
3184	GO_DOPAMINE_RECEPTOR_BINDING	-1.08	0.44	0	0	0	not significant
3185	GO_NEGATIVE_REGULATION_OF_MEIOTIC_CELL_CYCLE	-1.08	0.44	0	0	0	not significant
3186	GO_PHYSIOLOGICAL_CARDIAC_MUSCLE_HYPERTROPHY	-1.08	0.44	0	0	0	not significant
3187	GO_POLYOL_CATABOLIC_PROCESS	-1.08	0.44	0	0	0	not significant
3188	REACTOME_GLYCOGEN_SYNTHESIS	-1.08	0.44	0	0	0	not significant
3189	BIOCARTA_EIF4_PATHWAY	-1.08	0.43	0	0	0	not significant
3190	GO_POSITIVE_REGULATION_OF_MACROPHAGE_DIFFERENTIATION	-1.08	0.43	0	0	0	not significant
3191	REACTOME_REDUCTION_OF_CYTOSOLIC_CAPLUSPLUS_LEVELS	-1.08	0.43	0	0	0	not significant
3192	REACTOME_RRNA_PROCESSING_IN_THE_MITOCHONDRION	-1.08	0.43	0	0	0	not significant
3193	GO_ESTABLISHMENT_OF_EPITHELIAL_CELL_APICAL_BASAL_POLARITY	-1.08	0.42	0	0	0	not significant
3194	GO_POSITIVE_REGULATION_OF_HISTONE_H4_ACETYLATION	-1.08	0.42	0	0	0	not significant
3195	GO_CERAMIDE_1_PHOSPHATE_TRANSPORT	-1.08	0.41	0	0	0	not significant
3196	GO_EARLY_ENDOSOME_TO_GOLGI_TRANSPORT	-1.08	0.41	0	0	0	not significant
3197	GO_ENDOTHELIAL_CELL_ACTIVATION	-1.08	0.41	0	0	0	not significant
3198	GO_MYOBLAST_PROLIFERATION	-1.08	0.41	0	0	0	not significant
3199	GO_PHOSPHATIDYLETHANOLAMINE_BIOSYNTHETIC_PROCESS	-1.08	0.41	0	0	0	not significant
3200	GO_PRESYNAPTIC_CYTOSOL	-1.08	0.41	0	0	0	not significant
3201	GO_REGULATION_OF_TRANSLATIONAL_INITIATION_BY_EIF2_ALPHA_PHOSPHORYL	-1.08	0.41	0	0	0	not significant
3202	REACTOME_GAP_JUNCTION_DEGRADATION	-1.08	0.41	0	0	0	not significant
3203	GO_REGULATION_OF_PROTEIN_LOCALIZATION_TO_NUCLEOLUS	-1.08	0.4	0	0	0	not significant
3204	GO_RESPONSE_TO_SODIUM_ARSENITE	-1.08	0.4	0	0	0	not significant
3205	GO_RIBOSOME_DISASSEMBLY	-1.08	0.4	0	0	0	not significant
3206	GO_ANDROGEN_BIOSYNTHETIC_PROCESS	-1.08	0.39	0	0	0	not significant
3207	GO_PURINE_NUCLEOTIDE_SALVAGE	-1.08	0.39	0	0	0	not significant
3208	GO_REGULATION_OF_SKELETAL_MUSCLE_CONTRACTION	-1.08	0.39	0	0	0	not significant
3209	REACTOME_SYNTHESIS_OF_WYBUTOSINE_AT_G37_OF_TRNA_PHE	-1.08	0.39	0	0	0	not significant
3210	GO_REGULATION_OF_RECEPTOR_CATABOLIC_PROCESS	-1.08	0.38	0	0	0	not significant
3211	GO_NEGATIVE_REGULATION_OF_MEMBRANE_PERMEABILITY	-1.08	0.37	0	0	0	not significant
3212	GO_PRESYNAPTIC_ENDOCYTIC_ZONE	-1.08	0.35	0	0	0	not significant
3213	GO_EMBRYO_DEVELOPMENT	-1.07	0.96	0	0	0	not significant
3214	GO_SIGNAL_TRANSDUCTION_BY_PROTEIN_PHOSPHORYLATION	-1.07	0.8	0	0	0	not significant
3215	GO_REGULATION_OF_CELLULAR_PROTEIN_LOCALIZATION	-1.07	0.77	0	0	0	not significant
3216	GO_NEGATIVE_REGULATION_OF_PHOSPHORUS_METABOLIC_PROCESS	-1.07	0.74	0	0	0	not significant
3217	GO_POSITIVE_REGULATION_OF_CELLULAR_PROTEIN_LOCALIZATION	-1.07	0.66	0	0	0	not significant
3218	GO_NUCLEIC_ACID_PHOSPHODIESTER_BOND_HYDROLYSIS	-1.07	0.62	0	0	0	not significant
3219	GO_AXON_PART	-1.07	0.6	0	0	0	not significant
3220	GO_NEGATIVE_REGULATION_OF_CELL_DEVELOPMENT	-1.07	0.6	0	0	0	not significant
3221	GO_CELLULAR_PROCESS_INVOLVED_IN_REPRODUCTION_IN_MULTICELLULAR_OR	-1.07	0.59	0	0	0	not significant
3222	GO_REGULATION_OF_TRANSMEMBRANE_RECEPTOR_PROTEIN_SERINE_THREONIN	-1.07	0.56	0	0	0	not significant
3223	GO_MITOCHONDRIAL_MEMBRANE_ORGANIZATION	-1.07	0.55	0	0	0	not significant
3224	KAYO_AGING_MUSCLE_UP	-1.07	0.54	0	0	0	not significant
3225	MASSARWEH_TAMOXIFEN_RESISTANCE_DN	-1.07	0.54	0	0	0	not significant
3226	GO_CONTRACTILE_FIBER	-1.07	0.53	0	0	0	not significant
3227	GO_EPIDERMAL_CELL_DIFFERENTIATION	-1.07	0.53	0	0	0	not significant
3228	GO_SMOOTH_MUSCLE_CELL_PROLIFERATION	-1.07	0.53	0	0	0	not significant
3229	GO_PRODUCTION_OF_SMALL_RNA_INVOLVED_IN_GENE_SILENCING_BY_RNA	-1.07	0.52	0	0	0	not significant
3230	GO_PROTEIN_SERINE_THREONINE_PHOSPHATASE_ACTIVITY	-1.07	0.52	0	0	0	not significant
3231	GO_SYNAPSE_ASSEMBLY	-1.07	0.52	0	0	0	not significant
3232	GO_REGULATION_OF_REACTIVE_OXYGEN_SPECIES_METABOLIC_PROCESS	-1.07	0.51	0	0	0	not significant
3233	FIGUEROA_AML_METHYLATION_CLUSTER_6_UP	-1.07	0.5	0	0	0	not significant
3234	GO_TYROSINE_PHOSPHORYLATION_OF_STAT_PROTEIN	-1.07	0.5	0	0	0	not significant
3235	ESC_J1_UP_LATE.V1_UP	-1.07	0.49	0	0	0	not significant
3236	GO_MAP_KINASE_KINASE_KINASE_ACTIVITY	-1.07	0.49	0	0	0	not significant
3237	BONCI_TARGETS_OF_MIR15A_AND_MIR16_1	-1.07	0.48	0	0	0	not significant
3238	GO_HORMONE_BINDING	-1.07	0.48	0	0	0	not significant
3239	GO_REGULATION_OF_PHOSPHOLIPID_METABOLIC_PROCESS	-1.07	0.46	0	0	0	not significant
3240	HONMA_DOCETAXEL_RESISTANCE	-1.07	0.46	0	0	0	not significant
3241	REACTOME_CD28_CO_STIMULATION	-1.07	0.46	0	0	0	not significant
3242	GO_KINETOCHORE_ORGANIZATION	-1.07	0.45	0	0	0	not significant
3243	REACTOME_SIGNAL_AMPLIFICATION	-1.07	0.45	0	0	0	not significant
3244	TIMOFEEVA_GROWTH_STRESS_VIA_STAT1_DN	-1.07	0.45	0	0	0	not significant
3245	GO_POLY_A_BINDING	-1.07	0.44	0	0	0	not significant
3246	GO_RESPONSE_TO_PLATELET_DERIVED_GROWTH_FACTOR	-1.07	0.44	0	0	0	not significant
3247	ALONSO_METASTASIS_EMT_UP	-1.07	0.43	0	0	0	not significant
3248	BIOCARTA_NO1_PATHWAY	-1.07	0.43	0	0	0	not significant
3249	GO_MRNA_CLEAVAGE_INVOLVED_IN_MRNA_PROCESSING	-1.07	0.43	0	0	0	not significant
3250	GO_SKELETAL_MUSCLE_CONTRACTION	-1.07	0.43	0	0	0	not significant
3251	GO_MUSCLE_CELL_CELLULAR_HOMEOSTASIS	-1.07	0.42	0	0	0	not significant
3252	GO_POST_MRNA_RELEASE_SPLICEOSOMAL_COMPLEX	-1.07	0.42	0	0	0	not significant
3253	REACTOME_TGF_BETA_RECEPTOR_SIGNALING_IN_EMT_EPITHELIAL_TO_MESENC	-1.07	0.42	0	0	0	not significant
3254	GO_COSTAMERE	-1.07	0.41	0	0	0	not significant
3255	GO_NEGATIVE_REGULATION_OF_CELL_SIZE	-1.07	0.41	0	0	0	not significant
3256	GO_NEGATIVE_REGULATION_OF_VIRAL_RELEASE_FROM_HOST_CELL	-1.07	0.41	0	0	0	not significant
3257	MOOTHA_GLUCONEOGENESIS	-1.07	0.41	0	0	0	not significant
3258	REACTOME_ACTIVATION_OF_SMO	-1.07	0.41	0	0	0	not significant

3259	BIOCARTA_BARR_MAPK_PATHWAY	-1.07	0.4	0	0	0	not significant
3260	GO_CHOLESTEROL_CATABOLIC_PROCESS	-1.07	0.4	0	0	0	not significant
3261	GO_DEOXYRIBOSE_PHOSPHATE_CATABOLIC_PROCESS	-1.07	0.4	0	0	0	not significant
3262	GO_EUKARYOTIC_48S_PREINITIATION_COMPLEX	-1.07	0.4	0	0	0	not significant
3263	GO_GTP_BIOSYNTHETIC_PROCESS	-1.07	0.4	0	0	0	not significant
3264	GO_NEGATIVE_REGULATION_OF_TRANSCRIPTION_FROM_RNA_POLYMERASE_II_PI	-1.07	0.4	0	0	0	not significant
3265	GO_PROTEIN_HEXAMERIZATION	-1.07	0.4	0	0	0	not significant
3266	HINATA_NFKB_TARGETS_KERATINOCYTE_DN	-1.07	0.4	0	0	0	not significant
3267	GO_INTRINSIC_COMPONENT_OF_EXTERNAL_SIDE_OF_PLASMA_MEMBRANE	-1.07	0.39	0	0	0	not significant
3268	GO_POSITIVE_REGULATION_OF_SISTER_CHROMATID_COHESION	-1.07	0.39	0	0	0	not significant
3269	GO_REGULATION_OF_PROTEIN_HETERODIMERIZATION_ACTIVITY	-1.07	0.39	0	0	0	not significant
3270	GO_RENAL_VESICLE_DEVELOPMENT	-1.07	0.39	0	0	0	not significant
3271	GO_SEX_DETERMINATION	-1.07	0.39	0	0	0	not significant
3272	MATZUK_MATERNAL_EFFECT	-1.07	0.39	0	0	0	not significant
3273	VALK_AML_WITH_11Q23_REARRANGED	-1.07	0.39	0	0	0	not significant
3274	GO_OXALOACETATE_METABOLIC_PROCESS	-1.07	0.38	0	0	0	not significant
3275	GO_TRANSCRIPTION_FACTOR_TFIIIC_COMPLEX	-1.07	0.38	0	0	0	not significant
3276	GO_BONE_TRABECULA_MORPHOGENESIS	-1.07	0.37	0	0	0	not significant
3277	GO_COMMITMENT_COMPLEX	-1.07	0.37	0	0	0	not significant
3278	GO_MSL_COMPLEX	-1.07	0.37	0	0	0	not significant
3279	GO_NEGATIVE_REGULATION_OF_PROTEIN_SUMOYLATION	-1.07	0.37	0	0	0	not significant
3280	INAMURA_LUNG_CANCER_SCC_SUBTYPES_DN	-1.07	0.37	0	0	0	not significant
3281	GO_REGULATION_OF_HYDROLASE_ACTIVITY	-1.06	0.99	0	0	0	not significant
3282	GO_CELLULAR_AMIDE_METABOLIC_PROCESS	-1.06	0.9	0	0	0	not significant
3283	GO_REGULATION_OF_INTRACELLULAR_TRANSPORT	-1.06	0.74	0	0	0	not significant
3284	RHEIN_ALL_GLUCOCORTICOID_THERAPY_DN	-1.06	0.64	0	0	0	not significant
3285	STEIN_ESRRA_TARGETS	-1.06	0.64	0	0	0	not significant
3286	GO_MACROMOLECULE_METHYLATION	-1.06	0.58	0	0	0	not significant
3287	GO_ANTIGEN_RECEPTOR_MEDIATED_SIGNALING_PATHWAY	-1.06	0.55	0	0	0	not significant
3288	GO_NEGATIVE_REGULATION_OF_RESPONSE_TO_DNA_DAMAGE_STIMULUS	-1.06	0.55	0	0	0	not significant
3289	GO_NUCLEOSIDE_PHOSPHATE_BIOSYNTHETIC_PROCESS	-1.06	0.55	0	0	0	not significant
3290	AMIT_EGF_RESPONSE_480_HELA	-1.06	0.54	0	0	0	not significant
3291	E2F1_UP.V1_UP	-1.06	0.53	0	0	0	not significant
3292	GO_PROTEIN_N_TERMINUS_BINDING	-1.06	0.52	0	0	0	not significant
3293	ZHONG_RESPONSE_TO_AZACITIDINE_AND_TSA_UP	-1.06	0.52	0	0	0	not significant
3294	GO_ELECTRON_TRANSFER_ACTIVITY	-1.06	0.51	0	0	0	not significant
3295	GO_POSITIVE_REGULATION_OF_TRANSPORTER_ACTIVITY	-1.06	0.51	0	0	0	not significant
3296	HAHTOLA_MYCOSIS_FUNGOIDES_CD4_DN	-1.06	0.5	0	0	0	not significant
3297	REACTOME_REGULATION_OF_LIPID_METABOLISM_BY_PEROXISOME_PROLIFERAT(	-1.06	0.5	0	0	0	not significant
3298	E2F3_UP.V1_DN	-1.06	0.49	0	0	0	not significant
3299	GO_PHOSPHATIDYLINOSITOL_METABOLIC_PROCESS	-1.06	0.49	0	0	0	not significant
3300	CHR20Q11	-1.06	0.48	0	0	0	not significant
3301	GO_MRNA_3_UTR_BINDING	-1.06	0.47	0	0	0	not significant
3302	GO_N_ACYLTRANSFERASE_ACTIVITY	-1.06	0.47	0	0	0	not significant
3303	GO_SCAFFOLD_PROTEIN_BINDING	-1.06	0.47	0	0	0	not significant
3304	GO_SOMITOGENESIS	-1.06	0.47	0	0	0	not significant
3305	KEGG_PYRIMIDINE_METABOLISM	-1.06	0.47	0	0	0	not significant
3306	SCHAEFFER_PROSTATE_DEVELOPMENT_12HR_UP	-1.06	0.47	0	0	0	not significant
3307	GO_EMBRYONIC_CRANIAL_SKELETON_MORPHOGENESIS	-1.06	0.46	0	0	0	not significant
3308	JOHANSSON_GLIOMAGENESIS_BY_PDGFB_UP	-1.06	0.46	0	0	0	not significant
3309	PRAMOONJAGO_SOX4_TARGETS_DN	-1.06	0.46	0	0	0	not significant
3310	REACTOME_METABOLISM_OF_NUCLEOTIDES	-1.06	0.46	0	0	0	not significant
3311	TURASHVILI_BREAST_LOBULAR_CARCINOMA_VS_DUCTAL_NORMAL_UP	-1.06	0.46	0	0	0	not significant
3312	TERAO_AOX4_TARGETS_HG_UP	-1.06	0.45	0	0	0	not significant
3313	GO_HISTONE_MRNA_METABOLIC_PROCESS	-1.06	0.44	0	0	0	not significant
3314	MCCLUNG_DELTA_FOSB_TARGETS_8WK	-1.06	0.44	0	0	0	not significant
3315	REACTOME_MAP2K_AND_MAPK_ACTIVATION	-1.06	0.44	0	0	0	not significant
3316	GO_MIRNA_METABOLIC_PROCESS	-1.06	0.43	0	0	0	not significant
3317	LI_CISPLATIN_RESISTANCE_UP	-1.06	0.43	0	0	0	not significant
3318	MIKKELSEN_ES_ICP_WITH_H3K4ME3_AND_H3K27ME3	-1.06	0.43	0	0	0	not significant
3319	CHEOK_RESPONSE_TO_HD_MTX_DN	-1.06	0.42	0	0	0	not significant
3320	GO_ATPASE_ACTIVATOR_ACTIVITY	-1.06	0.42	0	0	0	not significant
3321	GO_REGULATION_OF_RNA_POLYMERASE_II_REGULATORY_REGION_SEQUENCE_S	-1.06	0.42	0	0	0	not significant
3322	GO_ACYLGLYCEROL_O_ACYLTRANSFERASE_ACTIVITY	-1.06	0.41	0	0	0	not significant
3323	GO_RNA_POLYMERASE_II_GENERAL_TRANSCRIPTION_INITIATION_FACTOR_ACTIVIT	-1.06	0.41	0	0	0	not significant
3324	GO_SKELETAL_MUSCLE_CELL_PROLIFERATION	-1.06	0.41	0	0	0	not significant
3325	REACTOME_APOPTOTIC_FACTOR_MEDIATED_RESPONSE	-1.06	0.41	0	0	0	not significant
3326	REACTOME_INTERLEUKIN_12_SIGNALING	-1.06	0.41	0	0	0	not significant
3327	STAMBOLSKY_BOUND_BY_MUTATED_TP53	-1.06	0.41	0	0	0	not significant
3328	GO_RESPONSE_TO_ACETYLCHOLINE	-1.06	0.4	0	0	0	not significant
3329	REACTOME_SIGNALING_BY_FGFR4_IN_DISEASE	-1.06	0.4	0	0	0	not significant
3330	GO_REGULATION_OF_MACROPHAGE_DIFFERENTIATION	-1.06	0.39	0	0	0	not significant
3331	GO_REGULATION_OF_MAINTENANCE_OF_SISTER_CHROMATID_COHESION	-1.06	0.39	0	0	0	not significant
3332	GO_UDP_GLUCOSYLTRANSFERASE_ACTIVITY	-1.06	0.39	0	0	0	not significant
3333	GO_INTRINSIC_APOPTOTIC_SIGNALING_PATHWAY_IN_RESPONSE_TO_OSMOTIC_ST	-1.06	0.37	0	0	0	not significant
3334	MYLLYKANGAS_AMPLIFICATION_HOT_SPOT_7	-1.06	0.37	0	0	0	not significant
3335	GO_CHROMATOID_BODY	-1.06	0.36	0	0	0	not significant
3336	GO_EMBRYONIC_RETINA_MORPHOGENESIS_IN_CAMERA_TYPE_EYE	-1.06	0.35	0	0	0	not significant
3337	GO_REGULATION_OF_SKELETAL_MUSCLE_ADAPTATION	-1.06	0.35	0	0	0	not significant
3338	GO_ORGANONITROGEN_COMPOUND_CATABOLIC_PROCESS	-1.05	0.75	0	0	0	not significant
3339	GO_INTERSPECIES_INTERACTION_BETWEEN_ORGANISMS	-1.05	0.74	0	0	0	not significant
3340	ACEVEDO_LIVER_TUMOR_VS_NORMAL_ADJACENT_TISSUE_UP	-1.05	0.72	0	0	0	not significant
3341	GO_CHROMATIN_ORGANIZATION	-1.05	0.69	0	0	0	not significant
3342	GO_HEAD_DEVELOPMENT	-1.05	0.64	0	0	0	not significant
3343	GO_REGULATION_OF_PROTEIN_COMPLEX_ASSEMBLY	-1.05	0.58	0	0	0	not significant
3344	MARTINEZ_RB1_TARGETS_UP	-1.05	0.58	0	0	0	not significant
3345	GO_CELL_SURFACE_RECEPTOR_SIGNALING_PATHWAY_INVOLVED_IN_CELL_CELL	-1.05	0.57	0	0	0	not significant
3346	PILON_KLF1_TARGETS_UP	-1.05	0.56	0	0	0	not significant
3347	GO_ENZYME_INHIBITOR_ACTIVITY	-1.05	0.55	0	0	0	not significant
3348	GO_STRUCTURAL_MOLECULE_ACTIVITY	-1.05	0.52	0	0	0	not significant
3349	GO_POSITIVE_REGULATION_OF_PROTEIN_CATABOLIC_PROCESS	-1.05	0.51	0	0	0	not significant
3350	WANG_CLIM2_TARGETS_DN	-1.05	0.49	0	0	0	not significant
3351	FIGUEROA_AML_METHYLATION_CLUSTER_7_UP	-1.05	0.48	0	0	0	not significant
3352	REACTOME_SIGNALING_BY_VEGF	-1.05	0.48	0	0	0	not significant
3353	GO_DNA_HELICASE_ACTIVITY	-1.05	0.47	0	0	0	not significant
3354	PELLICCIOTTA_HDAC_IN_ANTIGEN_PRESENTATION_UP	-1.05	0.46	0	0	0	not significant
3355	PID_NFAT_3PATHWAY	-1.05	0.46	0	0	0	not significant
3356	GO_DNA_PACKAGING_COMPLEX	-1.05	0.45	0	0	0	not significant
3357	GO_NEGATIVE_REGULATION_OF_PROTEOLYSIS_INVOLVED_IN_CELLULAR_PROTEI	-1.05	0.45	0	0	0	not significant
3358	GO_REGULATION_OF_ORGAN_GROWTH	-1.05	0.45	0	0	0	not significant
3359	SHAFFER_IRF4_TARGETS_IN_ACTIVATED_B_LYMPHOCYTE	-1.05	0.45	0	0	0	not significant
3360	GO_POSITIVE_REGULATION_OF_GLIOGENESIS	-1.05	0.44	0	0	0	not significant
3361	LOPEZ_MBD_TARGETS_IMPRINTED_AND_X_LINKED	-1.05	0.43	0	0	0	not significant
3362	CHR3P14	-1.05	0.42	0	0	0	not significant
3363	GO_BONE_CELL_DEVELOPMENT	-1.05	0.42	0	0	0	not significant
3364	GO_MYELOID_LEUKOCYTE_CYTOKINE_PRODUCTION	-1.05	0.42	0	0	0	not significant
3365	GO_NEGATIVE_REGULATION_OF_TELOMERE_MAINTENANCE	-1.05	0.42	0	0	0	not significant
3366	GO_REGULATION_OF_PROTEIN_AUTOPHOSPHORYLATION	-1.05	0.42	0	0	0	not significant
3367	IKEDA_MIR133_TARGETS_UP	-1.05	0.42	0	0	0	not significant

3368	PID_LIS1_PATHWAY	-1.05	0.42	0	0	0	not significant
3369	CHR10P15	-1.05	0.41	0	0	0	not significant
3370	GO_CELLULAR_COPPER_ION_HOMEOSTASIS	-1.05	0.41	0	0	0	not significant
3371	GO_LYMPH_NODE_DEVELOPMENT	-1.05	0.41	0	0	0	not significant
3372	BIOCARTA_ACTINY_PATHWAY	-1.05	0.4	0	0	0	not significant
3373	CHR8P23	-1.05	0.4	0	0	0	not significant
3374	GO_HEMATOPOIETIC_STEM_CELL_PROLIFERATION	-1.05	0.4	0	0	0	not significant
3375	PID_NECTIN_PATHWAY	-1.05	0.4	0	0	0	not significant
3376	REACTOME CREB1 PHOSPHORYLATION THROUGH NMDA RECEPTOR MEDIATED	-1.05	0.4	0	0	0	not significant
3377	REACTOME_SYNTHESIS_OF_IP2_IP_AND_INS_IN_THE_CYTOSOL	-1.05	0.4	0	0	0	not significant
3378	GO_MEGAKARYOCYTE_DEVELOPMENT	-1.05	0.39	0	0	0	not significant
3379	GO_NEGATIVE_REGULATION_OF_JNK_CASCADE	-1.05	0.39	0	0	0	not significant
3380	GO_NUCLEOSIDE_TRIPHOSPHATE_DIPHOSPHATASE_ACTIVITY	-1.05	0.39	0	0	0	not significant
3381	GO_POSITIVE_REGULATION_OF_SODIUM_ION_TRANSMEMBRANE_TRANSPORT	-1.05	0.39	0	0	0	not significant
3382	GO_SKELETAL_MUSCLE_ACETYLCHOLINE_GATED_CHANNEL_CLUSTERING	-1.05	0.39	0	0	0	not significant
3383	BIOCARTA_CDC42RAC_PATHWAY	-1.05	0.38	0	0	0	not significant
3384	HAHTOLA_CTCL_PATHOGENESIS	-1.05	0.38	0	0	0	not significant
3385	GO_ASTRAL_MICROTUBULE_ORGANIZATION	-1.05	0.37	0	0	0	not significant
3386	GO_NEGATIVE_REGULATION_OF_PROTEIN_EXPORT_FROM_NUCLEUS	-1.05	0.37	0	0	0	not significant
3387	GO_TRANSLOCATION_OF_MOLECULES_INTO_HOST	-1.05	0.37	0	0	0	not significant
3388	GO_POSITIVE_REGULATION_OF_OLIGODENDROCYTE_DIFFERENTIATION	-1.05	0.36	0	0	0	not significant
3389	GO_SNRNP_BINDING	-1.05	0.36	0	0	0	not significant
3390	REACTOME_SODIUM_CALCIUM_EXCHANGERS	-1.05	0.36	0	0	0	not significant
3391	SHIN_B_CELL_LYMPHOMA_CLUSTER_9	-1.05	0.36	0	0	0	not significant
3392	GO_CELL_DIFFERENTIATION_INVOLVED_IN_METANEPHROS_DEVELOPMENT	-1.05	0.35	0	0	0	not significant
3393	GO_POSITIVE_REGULATION_OF_SPINDLE_CHECKPOINT	-1.05	0.35	0	0	0	not significant
3394	GO_CELLULAR_RESPONSE_TO_SODIUM_ARSENITE	-1.05	0.34	0	0	0	not significant
3395	GO_PYRUVATE_DEHYDROGENASE_ACTIVITY	-1.05	0.34	0	0	0	not significant
3396	GO_REGULATION_OF_ALKALINE_PHOSPHATASE_ACTIVITY	-1.05	0.34	0	0	0	not significant
3397	GO_TRANSFERRIN_RECEPTOR_BINDING	-1.05	0.34	0	0	0	not significant
3398	REACTOME_FBXW7_MUTANTS_AND_NOTCH1_IN_CANCER	-1.05	0.34	0	0	0	not significant
3399	GO_DNA_METABOLIC_PROCESS	-1.04	0.84	0	0	0	not significant
3400	GO_POSITIVE_REGULATION_OF_HYDROLASE_ACTIVITY	-1.04	0.71	0	0	0	not significant
3401	BYSTRYKH_HEMATOPOIESIS_STEM_CELL_QTL_TRANS	-1.04	0.61	0	0	0	not significant
3402	GO_CENTRAL_NERVOUS_SYSTEM_DEVELOPMENT	-1.04	0.57	0	0	0	not significant
3403	GO_TRANSCRIPTION_COREGULATOR_ACTIVITY	-1.04	0.57	0	0	0	not significant
3404	GO_GTPASE_BINDING	-1.04	0.52	0	0	0	not significant
3405	GO_ACTIVATION_OF_PROTEIN_KINASE_ACTIVITY	-1.04	0.49	0	0	0	not significant
3406	GO_NEGATIVE_REGULATION_OF_BINDING	-1.04	0.48	0	0	0	not significant
3407	GO_CYTOPLASMIC_TRANSLATION	-1.04	0.47	0	0	0	not significant
3408	GO_CHROMATIN_REMODELING	-1.04	0.46	0	0	0	not significant
3409	GO_REGIONALIZATION	-1.04	0.46	0	0	0	not significant
3410	GO_REGULATION_OF_PROTEIN_POLYMERIZATION	-1.04	0.46	0	0	0	not significant
3411	KEGG_GNRH_SIGNALING_PATHWAY	-1.04	0.46	0	0	0	not significant
3412	REACTOME_L1CAM_INTERACTIONS	-1.04	0.46	0	0	0	not significant
3413	GO_NEGATIVE_REGULATION_OF_HEMOPOIESIS	-1.04	0.45	0	0	0	not significant
3414	GO_PURINE_CONTAINING_COMPOUND_BIOSYNTHETIC_PROCESS	-1.04	0.45	0	0	0	not significant
3415	GO_RIBOSOMAL_SUBUNIT	-1.04	0.45	0	0	0	not significant
3416	GO_SMOOTH_MUSCLE_CELL_MIGRATION	-1.04	0.45	0	0	0	not significant
3417	FLECHNER_PBL_KIDNEY_TRANSPLANT_REJECTED_VS_OK_UP	-1.04	0.44	0	0	0	not significant
3418	LAIHO_COLORECTAL_CANCER_SERRATED_UP	-1.04	0.44	0	0	0	not significant
3419	BROWNE_HCMV_INFECTION_10HR_UP	-1.04	0.43	0	0	0	not significant
3420	REACTOME_REGULATION_OF_TP53_EXPRESSION_AND_DEGRADATION	-1.04	0.43	0	0	0	not significant
3421	REACTOME RUNX1 INTERACTS WITH CO FACTORS WHOSE PRECISE EFFECT (	-1.04	0.43	0	0	0	not significant
3422	TIEN_INTESTINE_PROBIOTICS_6HR_DN	-1.04	0.43	0	0	0	not significant
3423	GO_PROTEIN_HETEROOLIGOMERIZATION	-1.04	0.42	0	0	0	not significant
3424	GO_REGULATION_OF_CARDIAC_MUSCLE_CONTRACTION	-1.04	0.42	0	0	0	not significant
3425	REACTOME_RESPONSE_TO_ELEVATED_PLATELET_CYTOSOLIC_CA2PLUS	-1.04	0.42	0	0	0	not significant
3426	BARRIER_CANCER_RELAPSE_NORMAL_SAMPLE_UP	-1.04	0.41	0	0	0	not significant
3427	GO_POSITIVE_REGULATION_OF_NITRIC_OXIDE_SYNTHASE_ACTIVITY	-1.04	0.41	0	0	0	not significant
3428	GO_PROTEIN_TYROSINE_SERINE_THREONINE_PHOSPHATASE_ACTIVITY	-1.04	0.41	0	0	0	not significant
3429	GO_REGULATION_OF_VOLTAGE_GATED_CALCIUM_CHANNEL_ACTIVITY	-1.04	0.41	0	0	0	not significant
3430	REACTOME_SIGNALING_BY_FGFR_IN_DISEASE	-1.04	0.41	0	0	0	not significant
3431	GO_LYSOPHOSPHOLIPID_ACYLTRANSFERASE_ACTIVITY	-1.04	0.4	0	0	0	not significant
3432	GO_SARCOPLASMIC_RETICULUM_MEMBRANE	-1.04	0.4	0	0	0	not significant
3433	GO_SYNAPTIC_TRANSMISSION_GABAERGIC	-1.04	0.4	0	0	0	not significant
3434	NUNODA_RESPONSE_TO_DASATINIB_IMATINIB_UP	-1.04	0.4	0	0	0	not significant
3435	BIOCARTA_P53HYPOXIA_PATHWAY	-1.04	0.39	0	0	0	not significant
3436	REACTOME_TP53_REGULATES_TRANSCRIPTION_OF_SEVERAL_ADDITIONAL_CELL_	-1.04	0.39	0	0	0	not significant
3437	ZUCCHI_METASTASIS_UP	-1.04	0.39	0	0	0	not significant
3438	GO_NUCLEOTIDE_EXCISION_REPAIR_DNA_DAMAGE_RECOGNITION	-1.04	0.38	0	0	0	not significant
3439	GO_REGULATION_OF_SYNAPTIC_TRANSMISSION_GABAERGIC	-1.04	0.38	0	0	0	not significant
3440	GO_U2_SNRNP	-1.04	0.38	0	0	0	not significant
3441	PID_CXCR3_PATHWAY	-1.04	0.38	0	0	0	not significant
3442	WANG_RECURRENT_LIVER_CANCER_DN	-1.04	0.38	0	0	0	not significant
3443	GO_ADA2_GCN5_ADA3_TRANSCRIPTION_ACTIVATOR_COMPLEX	-1.04	0.37	0	0	0	not significant
3444	GO_COENZYME_CATABOLIC_PROCESS	-1.04	0.37	0	0	0	not significant
3445	GO_NEGATIVE_REGULATION_OF_MICROTUBULE_POLYMERIZATION	-1.04	0.37	0	0	0	not significant
3446	GO_PROTEASOME_CORE_COMPLEX	-1.04	0.37	0	0	0	not significant
3447	REACTOME_CALCINEURIN_ACTIVATES_NFAT	-1.04	0.37	0	0	0	not significant
3448	GO_CELLULAR_RESPONSE_TO_SALT	-1.04	0.36	0	0	0	not significant
3449	GO_EUKARYOTIC_TRANSLATION_INITIATION_FACTOR_3_COMPLEX	-1.04	0.36	0	0	0	not significant
3450	GO_NUCLEAR_BODY_ORGANIZATION	-1.04	0.36	0	0	0	not significant
3451	GO SMOOTHENED SIGNALING PATHWAY INVOLVED IN DORSAL VENTRAL NEUR/	-1.04	0.36	0	0	0	not significant
3452	GO_HIGH_DENSITY_LIPOPROTEIN_PARTICLE	-1.04	0.35	0	0	0	not significant
3453	GO_OXIDOREDUCTASE_ACTIVITY_ACTING_ON_THE_CH_NH2_GROUP_OF_DONORS	-1.04	0.35	0	0	0	not significant
3454	GO_TRNA_THIO_MODIFICATION	-1.04	0.35	0	0	0	not significant
3455	MIKKELSEN_MCV6_ICP_WITH_H3K4ME3_AND_H3K27ME3	-1.04	0.35	0	0	0	not significant
3456	GO_MESENCHYMAL_TO_EPITHELIAL_TRANSITION_INVOLVED_IN_METANEPHROS_M	-1.04	0.34	0	0	0	not significant
3457	REACTOME_CGMP_EFFECTS	-1.04	0.34	0	0	0	not significant
3458	REACTOME NEGATIVE REGULATION OF ACTIVITY OF TFAP2 AP 2 FAMILY TRA	-1.04	0.34	0	0	0	not significant
3459	GO_POSITIVE_REGULATION_OF_CELL_CYCLE_CHECKPOINT	-1.04	0.33	0	0	0	not significant
3460	REACTOME_SENSING_OF_DNA_DOUBLE_STRAND_BREAKS	-1.04	0.32	0	0	0	not significant
3461	FIGUEROA_AML_METHYLATION_CLUSTER_2_DN	-1.04	0.31	0	0	0	not significant
3462	REACTOME_DEVELOPMENTAL_BIOLOGY	-1.03	0.58	0	0	0	not significant
3463	ACEVEDO_METHYLATED_IN_LIVER_CANCER_DN	-1.03	0.56	0	0	0	not significant
3464	GO_PERINUCLEAR_REGION_OF_CYTOPLASM	-1.03	0.54	0	0	0	not significant
3465	GO_EMBRYO_DEVELOPMENT_ENDING_IN_BIRTH_OR_EGG_HATCHING	-1.03	0.5	0	0	0	not significant
3466	LU_EZH2_TARGETS_DN	-1.03	0.5	0	0	0	not significant
3467	GRADE_COLON_CANCER_UP	-1.03	0.47	0	0	0	not significant
3468	GO_CATALYTIC_ACTIVITY_ACTING_ON_DNA	-1.03	0.46	0	0	0	not significant
3469	GO_NUCLEAR_CHROMOSOME_TELOMERIC_REGION	-1.03	0.45	0	0	0	not significant
3470	GO_SINGLE_STRANDED_DNA_BINDING	-1.03	0.45	0	0	0	not significant
3471	MULLIGHAN_NPM1_SIGNATURE_3_UP	-1.03	0.45	0	0	0	not significant
3472	GO_REGULATION_OF_SYMBIOSIS_ENCOMPASSING_MUTUALISM_THROUGH_PARASI	-1.03	0.44	0	0	0	not significant
3473	GO_TYPE_I_INTERFERON_PRODUCTION	-1.03	0.44	0	0	0	not significant
3474	GO_HINDBRAIN_MORPHOGENESIS	-1.03	0.43	0	0	0	not significant
3475	GO_REGULATION_OF_CELL_CYCLE_G1_S_PHASE_TRANSITION	-1.03	0.43	0	0	0	not significant
3476	MCMURRAY_TP53_HRAS_COOPERATION_RESPONSE_DN	-1.03	0.43	0	0	0	not significant

3477	REACTOME_ONCOGENIC_MAPK_SIGNALING	-1.03	0.43	0	0	0	not significant
3478	GO_CELL_CORTEX_PART	-1.03	0.42	0	0	0	not significant
3479	GO_POSITIVE_REGULATION_OF_DEVELOPMENTAL_GROWTH	-1.03	0.42	0	0	0	not significant
3480	HESS_TARGETS_OF_HOXA9_AND_MEIS1_UP	-1.03	0.42	0	0	0	not significant
3481	REACTOME_ANCHORING_OF_THE_BASAL_BODY_TO_THE_PLASMA_MEMBRANE	-1.03	0.42	0	0	0	not significant
3482	ATF2_S_UP.V1_UP	-1.03	0.41	0	0	0	not significant
3483	GO_POSITIVE_REGULATION_OF_PEPTIDASE_ACTIVITY	-1.03	0.41	0	0	0	not significant
3484	GO_POSITIVE_REGULATION_OF_PROTEIN_POLYMERIZATION	-1.03	0.41	0	0	0	not significant
3485	KEGG_VASOPRESSIN_REGULATED_WATER_REABSORPTION	-1.03	0.41	0	0	0	not significant
3486	ZHANG_TLX_TARGETS_DN	-1.03	0.41	0	0	0	not significant
3487	GO_PEPTIDYL_PROLINE_MODIFICATION	-1.03	0.4	0	0	0	not significant
3488	GO_SOMATIC_STEM_CELL_POPULATION_MAINTENANCE	-1.03	0.4	0	0	0	not significant
3489	GO_SPLICEOSOMAL_COMPLEX_ASSEMBLY	-1.03	0.4	0	0	0	not significant
3490	GRADE_COLON_VS_RECTAL_CANCER_UP	-1.03	0.4	0	0	0	not significant
3491	PARK_HSC_MARKERS	-1.03	0.4	0	0	0	not significant
3492	PID_HIF1_TFPATHWAY	-1.03	0.4	0	0	0	not significant
3493	TERAO_AOX4_TARGETS_SKIN_UP	-1.03	0.4	0	0	0	not significant
3494	ZHU_CMV_ALL_DN	-1.03	0.4	0	0	0	not significant
3495	CHESLER_BRAIN_QTL_CIS	-1.03	0.39	0	0	0	not significant
3496	GARGALOVIC_RESPONSE_TO_OXIDIZED_PHOSPHOLIPIDS_MAGENTA_DN	-1.03	0.39	0	0	0	not significant
3497	GO_NEGATIVE_REGULATION_OF_CELLULAR_RESPONSE_TO_TRANSFORMING_GR(	-1.03	0.39	0	0	0	not significant
3498	GO_POSITIVE_REGULATION_OF_CELLULAR_RESPONSE_TO_TRANSFORMING_GRC	-1.03	0.39	0	0	0	not significant
3499	HEIDENBLAD_AMPLICON_12P11_12_UP	-1.03	0.39	0	0	0	not significant
3500	KATSANOU_ELAVL1_TARGETS_DN	-1.03	0.39	0	0	0	not significant
3501	MCBRYAN_PUBERTAL_TGFB1_TARGETS_UP	-1.03	0.39	0	0	0	not significant
3502	REACTOME_SYNTHESIS_OF_PIPS_AT_THE_PLASMA_MEMBRANE	-1.03	0.39	0	0	0	not significant
3503	ABDELMOHSEN_ELAVL4_TARGETS	-1.03	0.38	0	0	0	not significant
3504	GO_HISTONE_H4_DEACETYLATION	-1.03	0.38	0	0	0	not significant
3505	GO_MEIOTIC_CHROMOSOME_SEPARATION	-1.03	0.38	0	0	0	not significant
3506	GO_MRNA_METHYLTRANSFERASE_ACTIVITY	-1.03	0.38	0	0	0	not significant
3507	GO_PHOSPHATIDYLINOSITOL_DEPHOSPHORYLATION	-1.03	0.38	0	0	0	not significant
3508	KEGG_TGF_BETA_SIGNALING_PATHWAY	-1.03	0.38	0	0	0	not significant
3509	MYLLYKANGAS_AMPLIFICATION_HOT_SPOT_27	-1.03	0.38	0	0	0	not significant
3510	PID_IFNG_PATHWAY	-1.03	0.38	0	0	0	not significant
3511	WEIGEL_OXIDATIVE_STRESS_BY_HNE_AND_H2O2	-1.03	0.38	0	0	0	not significant
3512	GO_HISTONE_H4_K16_ACETYLATION	-1.03	0.37	0	0	0	not significant
3513	GO_NEGATIVE_REGULATION_OF_MYOBLAST_DIFFERENTIATION	-1.03	0.37	0	0	0	not significant
3514	GO_PROTEIN_PHOSPHATASE_ACTIVATOR_ACTIVITY	-1.03	0.37	0	0	0	not significant
3515	GO_REGULATION_OF_NUCLEOTIDE_BIOSYNTHETIC_PROCESS	-1.03	0.37	0	0	0	not significant
3516	PID_FRA_PATHWAY	-1.03	0.37	0	0	0	not significant
3517	REACTOME_FOXO_MEDIATED_TRANSCRIPTION_OF_CELL_DEATH_GENES	-1.03	0.37	0	0	0	not significant
3518	GO_APOPTOTIC_DNA_FRAGMENTATION	-1.03	0.36	0	0	0	not significant
3519	GO_ENDOPLASMIC_RETICULUM_SUBCOMPARTMENT	-1.03	0.36	0	0	0	not significant
3520	GO_GUANOSINE_CONTAINING_COMPOUND_BIOSYNTHETIC_PROCESS	-1.03	0.36	0	0	0	not significant
3521	GO_MYELIN_ASSEMBLY	-1.03	0.36	0	0	0	not significant
3522	GO_POSITIVE_REGULATION_OF_HELICASE_ACTIVITY	-1.03	0.36	0	0	0	not significant
3523	REACTOME_INTEGRATION_OF_PROVIRUS	-1.03	0.36	0	0	0	not significant
3524	BERENJENO_TRANSFORMED_BY_RHOA_FOREVER_DN	-1.03	0.35	0	0	0	not significant
3525	GO_CERAMIDE_TRANSPORTER_ACTIVITY	-1.03	0.35	0	0	0	not significant
3526	GO_NUCLEOSIDE_MONOPHOSPHATE_KINASE_ACTIVITY	-1.03	0.35	0	0	0	not significant
3527	GO_POLYADENYLATION_DEPENDENT_RNA_CATABOLIC_PROCESS	-1.03	0.35	0	0	0	not significant
3528	GO_REGULATION_OF_HEXOKINASE_ACTIVITY	-1.03	0.35	0	0	0	not significant
3529	GO_REGULATION_OF_RESPIRATORY_SYSTEM_PROCESS	-1.03	0.35	0	0	0	not significant
3530	GO_DEADENYLATION_DEPENDENT_DECAPPING_OF_NUCLEAR_TRANSCRIBED_MRN	-1.03	0.34	0	0	0	not significant
3531	GO_SPERM_PRINCIPAL_PIECE	-1.03	0.34	0	0	0	not significant
3532	REACTOME_ACTIVATION_OF_CASPASES_THROUGH_APOPTOSOME_MEDIATED_CL	-1.03	0.34	0	0	0	not significant
3533	WEBER_METHYLATED_IN_COLON_CANCER	-1.03	0.34	0	0	0	not significant
3534	GO_NEGATIVE_REGULATION_OF_RNA_POLYMERASE_II_REGULATORY_REGION_SE	-1.03	0.33	0	0	0	not significant
3535	GO_NEGATIVE_REGULATION_OF_SYNAPTIC_VESICLE_TRANSPORT	-1.03	0.33	0	0	0	not significant
3536	REACTOME_EICOSANOIDS	-1.03	0.33	0	0	0	not significant
3537	REACTOME_SCAVENGING_BY_CLASS_A_RECEPTORS	-1.03	0.33	0	0	0	not significant
3538	GO_MEMBRANE_ORGANIZATION	-1.02	0.61	0	0	0	not significant
3539	REACTOME_AXON_GUIDANCE	-1.02	0.48	0	0	0	not significant
3540	FLECHNER_PBL_KIDNEY_TRANSPLANT_OK_VS_DONOR_UP	-1.02	0.44	0	0	0	not significant
3541	PATIL_LIVER_CANCER	-1.02	0.44	0	0	0	not significant
3542	GO_ORGANELLE_INNER_MEMBRANE	-1.02	0.43	0	0	0	not significant
3543	GO_OSSIFICATION	-1.02	0.43	0	0	0	not significant
3544	GO_TRANSCRIPTION_COACTIVATOR_ACTIVITY	-1.02	0.43	0	0	0	not significant
3545	KIM_GASTRIC_CANCER_CHEMOSENSITIVITY	-1.02	0.43	0	0	0	not significant
3546	REACTOME_HEMOSTASIS	-1.02	0.43	0	0	0	not significant
3547	GO_KINASE_REGULATOR_ACTIVITY	-1.02	0.42	0	0	0	not significant
3548	GO_CARBOHYDRATE_CATABOLIC_PROCESS	-1.02	0.41	0	0	0	not significant
3549	REACTOME_AUTOPHAGY	-1.02	0.41	0	0	0	not significant
3550	BIOCARTA_P38MAPK_PATHWAY	-1.02	0.4	0	0	0	not significant
3551	GO_CYTOPLASMIC_MICROTUBULE	-1.02	0.4	0	0	0	not significant
3552	QI_PLASMACYTOMA_DN	-1.02	0.4	0	0	0	not significant
3553	ACEVEDO_NORMAL_TISSUE_ADJACENT_TO_LIVER_TUMOR_UP	-1.02	0.39	0	0	0	not significant
3554	GO_METHYLATION	-1.02	0.39	0	0	0	not significant
3555	GO_MITOCHONDRIAL_TRANSPORT	-1.02	0.39	0	0	0	not significant
3556	GO_NEURON_MATURATION	-1.02	0.39	0	0	0	not significant
3557	GO_POSITIVE_REGULATION_OF_MITOTIC_NUCLEAR_DIVISION	-1.02	0.39	0	0	0	not significant
3558	REACTOME_SIGNALING_BY_MET	-1.02	0.39	0	0	0	not significant
3559	BROWNE_HCMV_INFECTION_24HR_UP	-1.02	0.38	0	0	0	not significant
3560	DARWICHE_SKIN_TUMOR_PROMOTER_DN	-1.02	0.38	0	0	0	not significant
3561	GO_CLATHRIN_VESICLE_COAT	-1.02	0.38	0	0	0	not significant
3562	GO_CYTOKINE_ACTIVITY	-1.02	0.38	0	0	0	not significant
3563	GO_PHOTORECEPTOR_OUTER_SEGMENT	-1.02	0.38	0	0	0	not significant
3564	GO_PROTEIN_HOMOTETRAMERIZATION	-1.02	0.38	0	0	0	not significant
3565	GO_REGULATION_OF_SYNAPSE_ASSEMBLY	-1.02	0.38	0	0	0	not significant
3566	GO_THYROID_HORMONE_RECEPTOR_BINDING	-1.02	0.38	0	0	0	not significant
3567	HOLLEMAN_PREDNISOLONE_RESISTANCE_B_ALL_UP	-1.02	0.38	0	0	0	not significant
3568	REACTOME_PLATELET_AGGREGATION_PLUG_FORMATION	-1.02	0.38	0	0	0	not significant
3569	BOYAULT_LIVER_CANCER_SUBCLASS_G12_UP	-1.02	0.37	0	0	0	not significant
3570	GO_NEGATIVE_REGULATION_OF_ADHERENS_JUNCTION_ORGANIZATION	-1.02	0.37	0	0	0	not significant
3571	GO_PERICENTRIC_HETEROCHROMATIN	-1.02	0.37	0	0	0	not significant
3572	GO_POSITIVE_REGULATION_OF_ADHERENS_JUNCTION_ORGANIZATION	-1.02	0.37	0	0	0	not significant
3573	GO_POSITIVE_REGULATION_OF_NUCLEAR_DIVISION	-1.02	0.37	0	0	0	not significant
3574	GO_PROTEIN_HETEROTETRAMERIZATION	-1.02	0.37	0	0	0	not significant
3575	GO_PROTEIN_LOCALIZATION_TO_KINETOCHORE	-1.02	0.37	0	0	0	not significant
3576	GO_REGULATION_OF_HEART_GROWTH	-1.02	0.37	0	0	0	not significant
3577	VART_KSHV_INFECTION_ANGIOGENIC_MARKERS_DN	-1.02	0.37	0	0	0	not significant
3578	ABRAHAM_ALPC_VS_MULTIPLE_MYELOMA_UP	-1.02	0.36	0	0	0	not significant
3579	GO_CARDIAC_CHAMBER_MORPHOGENESIS	-1.02	0.36	0	0	0	not significant
3580	GO_CELLULAR_ALDEHYDE_METABOLIC_PROCESS	-1.02	0.36	0	0	0	not significant
3581	GO_M_BAND	-1.02	0.36	0	0	0	not significant
3582	GO_NEGATIVE_REGULATION_OF_PROTEIN_LOCALIZATION_TO_MEMBRANE	-1.02	0.36	0	0	0	not significant
3583	GO_NUCLEOTIDE_SALVAGE	-1.02	0.36	0	0	0	not significant
3584	GO_POSITIVE_REGULATION_OF_SEQUESTERING_OF_CALCIUM_ION	-1.02	0.36	0	0	0	not significant
3585	GO_PRESYNAPSE_ORGANIZATION	-1.02	0.36	0	0	0	not significant

3586	ALONSO_METASTASIS_DN	-1.02	0.35	0	0	0	not significant
3587	GO_CAMP_RESPONSE_ELEMENT_BINDING_PROTEIN_BINDING	-1.02	0.35	0	0	0	not significant
3588	GO_CARBOXY_TERMINAL_DOMAIN_PROTEIN_KINASE_COMPLEX	-1.02	0.35	0	0	0	not significant
3589	GO_CORTICOSTEROID_RECEPTOR_SIGNALING_PATHWAY	-1.02	0.35	0	0	0	not significant
3590	GO_ESTABLISHMENT_OF_MITOCHONDRION_LOCALIZATION	-1.02	0.35	0	0	0	not significant
3591	GO_INTRONIC_TRANSCRIPTION_REGULATORY_REGION_DNA_BINDING	-1.02	0.35	0	0	0	not significant
3592	GO_LYS48_SPECIFIC_DEUBIQUITINASE_ACTIVITY	-1.02	0.35	0	0	0	not significant
3593	GO_PROTEIN_LOCALIZATION_TO_CELL_JUNCTION	-1.02	0.35	0	0	0	not significant
3594	GO_SKELETAL_MUSCLE_TISSUE_REGENERATION	-1.02	0.35	0	0	0	not significant
3595	MURATA_VIRULENCE_OF_H_PILORI	-1.02	0.35	0	0	0	not significant
3596	CHR10P14	-1.02	0.34	0	0	0	not significant
3597	GO_POSITIVE_REGULATION_OF_NUCLEAR_CELL_CYCLE_DNA_REPLICATION	-1.02	0.34	0	0	0	not significant
3598	GO_REGULATION_OF_RNA_BINDING	-1.02	0.34	0	0	0	not significant
3599	GO_RNA_POLYMERASE_II_INTRONIC_TRANSCRIPTION_REGULATORY_REGION_SEC	-1.02	0.34	0	0	0	not significant
3600	MARIADASON_RESPONSE_TO_BUTYRATE_CURCUMIN_SULINDAC_TSA_8	-1.02	0.34	0	0	0	not significant
3601	REACTOME_TETRAHYDROBIOPTERIN_BH4_SYNTHESIS_RECYCLING_SALVAGE_ANE	-1.02	0.34	0	0	0	not significant
3602	SAGIV_CD24_TARGETS_UP	-1.02	0.34	0	0	0	not significant
3603	GO_CAMP_DEPENDENT_PROTEIN_KINASE_ACTIVITY	-1.02	0.33	0	0	0	not significant
3604	GO_MITOTIC_SPINDLE_ASTRAL_MICROTUBULE	-1.02	0.33	0	0	0	not significant
3605	GO_NEGATIVE_REGULATION_OF_GLYCOLYTIC_PROCESS	-1.02	0.33	0	0	0	not significant
3606	REACTOME_WNT5A_DEPENDENT_INTERNALIZATION_OF_FZD4	-1.02	0.33	0	0	0	not significant
3607	XU_HGF_TARGETS_REPRESSED_BY_AKT1_UP	-1.02	0.33	0	0	0	not significant
3608	GO_CARBONYL_REDUCTASE_NADPH_ACTIVITY	-1.02	0.32	0	0	0	not significant
3609	GO_EGO_COMPLEX	-1.02	0.32	0	0	0	not significant
3610	GO_POSTSYNAPTIC_ENDOCYTIC_ZONE	-1.02	0.32	0	0	0	not significant
3611	GO_PYRAMIDAL_NEURON_DIFFERENTIATION	-1.02	0.32	0	0	0	not significant
3612	GO_REGULATION_OF_METAPHASE_PLATE_CONGRESSION	-1.02	0.32	0	0	0	not significant
3613	GO_SUBSTRATE_DEPENDENT_CELL_MIGRATION_CELL_EXTENSION	-1.02	0.32	0	0	0	not significant
3614	GO_RRNA_TRANSPORT	-1.02	0.3	0	0	0	not significant
3615	LIU_SOX4_TARGETS_DN	-1.01	0.43	0	0	0	not significant
3616	CHR17P13	-1.01	0.41	0	0	0	not significant
3617	CHR10Q22	-1.01	0.4	0	0	0	not significant
3618	GO_ACTIN_FILAMENT_BUNDLE_ORGANIZATION	-1.01	0.4	0	0	0	not significant
3619	MORI_EMU_MYC_LYMPHOMA_BY_ONSET_TIME_UP	-1.01	0.4	0	0	0	not significant
3620	CHR3P25	-1.01	0.39	0	0	0	not significant
3621	GO_MULTICELLULAR_ORGANISMAL_SIGNALING	-1.01	0.39	0	0	0	not significant
3622	REACTOME_SYNTHESIS_OF_PA	-1.01	0.39	0	0	0	not significant
3623	REACTOME_TP53_REGULATES_METABOLIC_GENES	-1.01	0.39	0	0	0	not significant
3624	BROWNE_HCMV_INFECTION_1HR_DN	-1.01	0.38	0	0	0	not significant
3625	GO_REGULATION_OF_HISTONE_PHOSPHORYLATION	-1.01	0.38	0	0	0	not significant
3626	GO_RESPIRATORY_SYSTEM_PROCESS	-1.01	0.38	0	0	0	not significant
3627	GO_RESPONSE_TO_OSMOTIC_STRESS	-1.01	0.38	0	0	0	not significant
3628	IVANOVSKA_MIR106B_TARGETS	-1.01	0.38	0	0	0	not significant
3629	MCCLUNG_CREB1_TARGETS_DN	-1.01	0.38	0	0	0	not significant
3630	SHETH_LIVER_CANCER_VS_TXNIP_LOSS_PAM2	-1.01	0.38	0	0	0	not significant
3631	BROWNE_HCMV_INFECTION_6HR_DN	-1.01	0.37	0	0	0	not significant
3632	GO_INCLUSION_BODY	-1.01	0.37	0	0	0	not significant
3633	GO_LONG_TERM_MEMORY	-1.01	0.37	0	0	0	not significant
3634	GO_MUSCLE_CELL_MIGRATION	-1.01	0.37	0	0	0	not significant
3635	GO_NEGATIVE_REGULATION_OF_CELLULAR_SENESCENCE	-1.01	0.37	0	0	0	not significant
3636	GO_NEURAL_PRECURSOR_CELL_PROLIFERATION	-1.01	0.37	0	0	0	not significant
3637	GO_POSITIVE_REGULATION_OF_MRNA_CATABOLIC_PROCESS	-1.01	0.37	0	0	0	not significant
3638	GO_REGULATION_OF_CELLULAR_RESPONSE_TO_INSULIN_STIMULUS	-1.01	0.37	0	0	0	not significant
3639	MELLMAN_TUT1_TARGETS_UP	-1.01	0.37	0	0	0	not significant
3640	PID_CERAMIDE_PATHWAY	-1.01	0.37	0	0	0	not significant
3641	REACTOME_TRAF6_MEDIATED_IRF7_ACTIVATION_IN_TLR7_8_OR_9_SIGNALING	-1.01	0.37	0	0	0	not significant
3642	BIOCARTA_FAS_PATHWAY	-1.01	0.36	0	0	0	not significant
3643	GO_EMBRYONIC_AXIS_SPECIFICATION	-1.01	0.36	0	0	0	not significant
3644	GO_INTRACILIARY_RETROGRADE_TRANSPORT	-1.01	0.36	0	0	0	not significant
3645	GO_NON_RECOMBINATIONAL_REPAIR	-1.01	0.36	0	0	0	not significant
3646	GO_REGULATION_OF_HISTONE_H4_ACETYLATION	-1.01	0.36	0	0	0	not significant
3647	GO_RENAL_WATER_HOMEOSTASIS	-1.01	0.36	0	0	0	not significant
3648	GO_STRIATED_MUSCLE_ADAPTATION	-1.01	0.36	0	0	0	not significant
3649	GO_TRANSCRIPTION_COREPRESSOR_ACTIVITY	-1.01	0.36	0	0	0	not significant
3650	LOPEZ_TRANSLATION_VIA_FN1_SIGNALING	-1.01	0.36	0	0	0	not significant
3651	PID_P38_MK2_PATHWAY	-1.01	0.36	0	0	0	not significant
3652	REACTOME_INTEGRIN_SIGNALING	-1.01	0.36	0	0	0	not significant
3653	SWEET_KRAS_TARGETS_UP	-1.01	0.36	0	0	0	not significant
3654	ZHOU_CELL_CYCLE_GENES_IN_IR_RESPONSE_6HR	-1.01	0.36	0	0	0	not significant
3655	BERNARD_PPAPDC1B_TARGETS_UP	-1.01	0.35	0	0	0	not significant
3656	GO_AP_3_ADAPTOR_COMPLEX	-1.01	0.35	0	0	0	not significant
3657	GO_ENDORIBONUCLEASE_ACTIVITY	-1.01	0.35	0	0	0	not significant
3658	GO_MICROTUBULE_DEPOLYMERIZATION	-1.01	0.35	0	0	0	not significant
3659	GO_RESPONSE_TO_HYPEROXIA	-1.01	0.35	0	0	0	not significant
3660	GO_SEGMENTATION	-1.01	0.35	0	0	0	not significant
3661	KEGG_RNA_DEGRADATION	-1.01	0.35	0	0	0	not significant
3662	MASSARWEH_RESPONSE_TO_ESTRADIOL	-1.01	0.35	0	0	0	not significant
3663	BIOCARTA_MYOSIN_PATHWAY	-1.01	0.34	0	0	0	not significant
3664	BIOCARTA_SET_PATHWAY	-1.01	0.34	0	0	0	not significant
3665	GO_CERAMIDE_CATABOLIC_PROCESS	-1.01	0.34	0	0	0	not significant
3666	GO_DETECTION_OF_CALCIUM_ION	-1.01	0.34	0	0	0	not significant
3667	GO_FOLIC_ACID_CONTAINING_COMPOUND_BIOSYNTHETIC_PROCESS	-1.01	0.34	0	0	0	not significant
3668	GO_NUCLEAR_LAMINA	-1.01	0.34	0	0	0	not significant
3669	GO_POST_EMBRYONIC_ANIMAL_ORGAN_DEVELOPMENT	-1.01	0.34	0	0	0	not significant
3670	KANG_DOXORUBICIN_RESISTANCE_DN	-1.01	0.34	0	0	0	not significant
3671	ZHENG_RESPONSE_TO_ARSENITE_UP	-1.01	0.34	0	0	0	not significant
3672	FIGUEROA_AML_METHYLATION_CLUSTER_4_DN	-1.01	0.33	0	0	0	not significant
3673	NGO_MALIGNANT_GLIOMA_1P_LOH	-1.01	0.33	0	0	0	not significant
3674	REACTOME_ADRENALINE_NORADRENALINE_INHIBITS_INSULIN_SECRETION	-1.01	0.33	0	0	0	not significant
3675	WU_HBX_TARGETS_3_DN	-1.01	0.33	0	0	0	not significant
3676	GO_CYCLIC_NUCLEOTIDE_DEPENDENT_PROTEIN_KINASE_ACTIVITY	-1.01	0.32	0	0	0	not significant
3677	GO_MICRO_RIBONUCLEOPROTEIN_COMPLEX	-1.01	0.32	0	0	0	not significant
3678	GO_MUSCLE_MYOSIN_COMPLEX	-1.01	0.32	0	0	0	not significant
3679	GO_NADH_PYROPHOSPHATASE_ACTIVITY	-1.01	0.32	0	0	0	not significant
3680	GO_NEDD8_TRANSFERASE_ACTIVITY	-1.01	0.32	0	0	0	not significant
3681	GO_POSITIVE_REGULATION_OF_VASCULAR_ASSOCIATED_SMOOTH_MUSCLE_CELI	-1.01	0.32	0	0	0	not significant
3682	GO_FLOTILLIN_COMPLEX	-1.01	0.31	0	0	0	not significant
3683	GO_PLATELET_DENSE_TUBULAR_NETWORK_MEMBRANE	-1.01	0.31	0	0	0	not significant
3684	GO_POLY_A_PLUS_MRNA_EXPORT_FROM_NUCLEUS	-1.01	0.31	0	0	0	not significant
3685	GO_SPHINGOMYELIN_CATABOLIC_PROCESS	-1.01	0.31	0	0	0	not significant
3686	GO_SUMO_POLYMER_BINDING	-1.01	0.3	0	0	0	not significant
3687	REACTOME_UPTAKE_AND_FUNCTION_OF_DIPHTHERIA_TOXIN	-1.01	0.29	0	0	0	not significant
3688	HEDENFALK_BREAST_CANCER_BRCA1_VS_BRCA2	-1.00	0.4	0	0	0	not significant
3689	MACAEVA_PBMC_RESPONSE_TO_IR	-1.00	0.39	0	0	0	not significant
3690	CHR10Q23	-1.00	0.38	0	0	0	not significant
3691	GO_NEGATIVE_REGULATION_OF_APOPTOTIC_SIGNALING_PATHWAY	-1.00	0.38	0	0	0	not significant
3692	GO_SWI_SNF_SUPERFAMILY_TYPE_COMPLEX	-1.00	0.38	0	0	0	not significant
3693	GO_INTERFERON_BETA_PRODUCTION	-1.00	0.37	0	0	0	not significant
3694	GO_INTRINSIC_APOPTOTIC_SIGNALING_PATHWAY_IN_RESPONSE_TO_OXIDATIVE_S	-1.00	0.37	0	0	0	not significant

3695	GO_POSITIVE_REGULATION_OF_DNA_METABOLIC_PROCESS	-1.00	0.37	0	0	0	not significant
3696	GO_REGULATION_OF_CELL_JUNCTION_ASSEMBLY	-1.00	0.37	0	0	0	not significant
3697	GO_REGULATION_OF_NITRIC_OXIDE_SYNTHASE_ACTIVITY	-1.00	0.37	0	0	0	not significant
3698	GO_RESPONSE_TO_INTERLEUKIN_12	-1.00	0.37	0	0	0	not significant
3699	YAUCH_HEDGEHOG_SIGNALING_PARACRINE_DN	-1.00	0.37	0	0	0	not significant
3700	DARWICHE_SKIN_TUMOR_PROMOTER_UP	-1.00	0.36	0	0	0	not significant
3701	GO_ACTIN_FILAMENT_BUNDLE	-1.00	0.36	0	0	0	not significant
3702	GO_ANDROGEN_RECEPTOR_SIGNALING_PATHWAY	-1.00	0.36	0	0	0	not significant
3703	GO_METHYLTRANSFERASE_COMPLEX	-1.00	0.36	0	0	0	not significant
3704	GO_MICROTUBULE_ORGANIZING_CENTER_PART	-1.00	0.36	0	0	0	not significant
3705	GO_POSITIVE_REGULATION_OF_MICROTUBULE_POLYMERIZATION_OR_DEPOLYME	-1.00	0.36	0	0	0	not significant
3706	GO_POSTSYNAPTIC_SPECIALIZATION_MEMBRANE	-1.00	0.36	0	0	0	not significant
3707	GO_REGULATION_OF_ATPASE_ACTIVITY	-1.00	0.36	0	0	0	not significant
3708	GO_REGULATION_OF_CELL_SUBSTRATE_ADHESION	-1.00	0.36	0	0	0	not significant
3709	REACTOME_ACTIVATION_OF_BH3_ONLY_PROTEINS	-1.00	0.36	0	0	0	not significant
3710	GO_CELL_FATE_COMMITMENT	-1.00	0.35	0	0	0	not significant
3711	GO_CHAPERONE_BINDING	-1.00	0.35	0	0	0	not significant
3712	GO_GLYCOSYL_COMPOUND_BIOSYNTHETIC_PROCESS	-1.00	0.35	0	0	0	not significant
3713	GO_GOLGI_TO_PLASMA_MEMBRANE_TRANSPORT	-1.00	0.35	0	0	0	not significant
3714	GO_POSITIVE_REGULATION_OF_NEUROLOGICAL_SYSTEM_PROCESS	-1.00	0.35	0	0	0	not significant
3715	PID_FAK_PATHWAY	-1.00	0.35	0	0	0	not significant
3716	REACTOME_CYTOCHROME_C_MEDIATED_APOPTOTIC_RESPONSE	-1.00	0.35	0	0	0	not significant
3717	REACTOME_THE_CITRIC_ACID_TCA_CYCLE_AND_RESPIRATORY_ELECTRON_TRAN	-1.00	0.35	0	0	0	not significant
3718	DEMAGALHAES_AGING_DN	-1.00	0.34	0	0	0	not significant
3719	GO_ANOIKIS	-1.00	0.34	0	0	0	not significant
3720	GO_CYTOPLASMIC_MRNA_PROCESSING_BODY_ASSEMBLY	-1.00	0.34	0	0	0	not significant
3721	GO_FIBROBLAST_APOPTOTIC_PROCESS	-1.00	0.34	0	0	0	not significant
3722	GO_GLYCOLIPID_CATABOLIC_PROCESS	-1.00	0.34	0	0	0	not significant
3723	GO_NEGATIVE_REGULATION_OF_DNA_BIOSYNTHETIC_PROCESS	-1.00	0.34	0	0	0	not significant
3724	GO_PHOSPHORIC_ESTER_HYDROLASE_ACTIVITY	-1.00	0.34	0	0	0	not significant
3725	GO_REGULATION_OF_EMBRYONIC_DEVELOPMENT	-1.00	0.34	0	0	0	not significant
3726	OZEN_MIR125B1_TARGETS	-1.00	0.34	0	0	0	not significant
3727	RAY_TARGETS_OF_P210_BCR_ABL_FUSION_UP	-1.00	0.34	0	0	0	not significant
3728	UDAYAKUMAR_MED1_TARGETS_UP	-1.00	0.34	0	0	0	not significant
3729	BCAT.100_UP.V1_UP	-1.00	0.33	0	0	0	not significant
3730	CHR12Q15	-1.00	0.33	0	0	0	not significant
3731	CHR17Q24	-1.00	0.33	0	0	0	not significant
3732	GO_CARNITINE_SHUTTLE	-1.00	0.33	0	0	0	not significant
3733	GO_CYCLIN_CDK_POSITIVE_TRANSCRIPTION_ELONGATION_FACTOR_COMPLEX	-1.00	0.33	0	0	0	not significant
3734	GO_NEGATIVE_REGULATION_OF_INTRACELLULAR_STEROID_HORMONE_RECEPTO	-1.00	0.33	0	0	0	not significant
3735	GO_NUCLEAR_ENVELOPE_REASSEMBLY	-1.00	0.33	0	0	0	not significant
3736	GO_ORGANELLE_ENVELOPE_LUMEN	-1.00	0.33	0	0	0	not significant
3737	GO_PEPTIDYL_METHIONINE_MODIFICATION	-1.00	0.33	0	0	0	not significant
3738	GO_PHOSPHATASE_ACTIVATOR_ACTIVITY	-1.00	0.33	0	0	0	not significant
3739	GO_REGULATION_OF_SMAD_PROTEIN_SIGNAL_TRANSDUCTION	-1.00	0.33	0	0	0	not significant
3740	GO_SYNAPTIC_VESICLE_CYTOSKELETAL_TRANSPORT	-1.00	0.33	0	0	0	not significant
3741	GO_T_CELL_HOMEOSTASIS	-1.00	0.33	0	0	0	not significant
3742	HOFMANN_CELL_LYMPHOMA_DN	-1.00	0.33	0	0	0	not significant
3743	PID_DNA_PK_PATHWAY	-1.00	0.33	0	0	0	not significant
3744	REACTOME_CENTROSOME_MATURATION	-1.00	0.33	0	0	0	not significant
3745	REACTOME_SYNTHESIS_OF_PROSTAGLANDINS_PG_AND_THROMBOXANES_TX	-1.00	0.33	0	0	0	not significant
3746	BIOCARTA_CPSF_PATHWAY	-1.00	0.32	0	0	0	not significant
3747	CHR20Q12	-1.00	0.32	0	0	0	not significant
3748	GO_EPIDERMAL_GROWTH_FACTOR_RECEPTOR_BINDING	-1.00	0.32	0	0	0	not significant
3749	GO_FMN_BINDING	-1.00	0.32	0	0	0	not significant
3750	GO_FORMATION_OF_TRANSLATION_PREINITIATION_COMPLEX	-1.00	0.32	0	0	0	not significant
3751	GO_MITOCHONDRIAL_RESPIRATORY_CHAIN_COMPLEX_ASSEMBLY	-1.00	0.32	0	0	0	not significant
3752	GO_REGULATION_OF_AUTOPHAGOSOME_MATURATION	-1.00	0.32	0	0	0	not significant
3753	MARIADASON_RESPONSE_TO_CURCUMIN_SULINDAC_5	-1.00	0.32	0	0	0	not significant
3754	REACTOME_CLATHRIN_MEDIATED_ENDOCYTOSIS	-1.00	0.32	0	0	0	not significant
3755	BIOCARTA_PELP1_PATHWAY	-1.00	0.31	0	0	0	not significant
3756	GO_ESTABLISHMENT_OF_LYMPHOCYTE_POLARITY	-1.00	0.31	0	0	0	not significant
3757	GO_MUSCLE_CELL_DIFFERENTIATION	-1.00	0.31	0	0	0	not significant
3758	GO_THYROID_GLAND_DEVELOPMENT	-1.00	0.31	0	0	0	not significant
3759	GO_SHELTERIN_COMPLEX	-1.00	0.3	0	0	0	not significant
3760	GO_TRNA_AMINOACYLATION_FOR_MITOCHONDRIAL_PROTEIN_TRANSLATION	-1.00	0.3	0	0	0	not significant
3761	GO_NEGATIVE_REGULATION_OF_SMOOTH_MUSCLE_CONTRACTION	-1.00	0.29	0	0	0	not significant
3762	GO_PLATELET_DENSE_GRANULE_MEMBRANE	-1.00	0.29	0	0	0	not significant
3763	BIOCARTA_AKT_PATHWAY	-0.99	0.36	0	0	0	not significant
3764	GO_NEGATIVE_REGULATION_OF_CELL_CYCLE_ARREST	-0.99	0.35	0	0	0	not significant
3765	BURTON_ADIPOGENESIS_PEAK_AT_8HR	-0.99	0.34	0	0	0	not significant
3766	BYSTRYKH_HEMATOPOIESIS_STEM_CELL_AND_BRAIN_QTL_CIS	-0.99	0.34	0	0	0	not significant
3767	GO_MITOCHONDRIAL_RESPIRATORY_CHAIN_COMPLEX_IV_ASSEMBLY	-0.99	0.34	0	0	0	not significant
3768	GO_PEPTIDASE_REGULATOR_ACTIVITY	-0.99	0.34	0	0	0	not significant
3769	GO_PROTEIN_LOCALIZATION_TO_PLASMA_MEMBRANE	-0.99	0.34	0	0	0	not significant
3770	GO_SOMATIC_STEM_CELL_DIVISION	-0.99	0.34	0	0	0	not significant
3771	PID_MTOR_4PATHWAY	-0.99	0.34	0	0	0	not significant
3772	PID_TGFBR_PATHWAY	-0.99	0.34	0	0	0	not significant
3773	REACTOME_FORMATION_OF_THE_BETA_CATENIN:TCF_TRANSACTIVATING_COMPL	-0.99	0.34	0	0	0	not significant
3774	CHR10P13	-0.99	0.33	0	0	0	not significant
3775	CYCLIN_D1_UP.V1_UP	-0.99	0.33	0	0	0	not significant
3776	GO_CARDIAC_CHAMBER_DEVELOPMENT	-0.99	0.33	0	0	0	not significant
3777	GO_CILIARY_BASAL_BODY_PLASMA_MEMBRANE_DOCKING	-0.99	0.33	0	0	0	not significant
3778	GO_DEVELOPMENTAL_MATURATION	-0.99	0.33	0	0	0	not significant
3779	GO_FERROUS_IRON_BINDING	-0.99	0.33	0	0	0	not significant
3780	GO_P_BODY	-0.99	0.33	0	0	0	not significant
3781	GO_PROTEIN_KINASE_C_ACTIVITY	-0.99	0.33	0	0	0	not significant
3782	GO_REGULATION_OF_BROWN_FAT_CELL_DIFFERENTIATION	-0.99	0.33	0	0	0	not significant
3783	GO_REGULATION_OF_CALCIUM_MEDIATED_SIGNALING	-0.99	0.33	0	0	0	not significant
3784	GO_TRANS_GOLGI_NETWORK_MEMBRANE	-0.99	0.33	0	0	0	not significant
3785	HOEGERKORP_CD44_TARGETS_DIRECT_DN	-0.99	0.33	0	0	0	not significant
3786	MAGRANGEAS_MULTIPLE_MYELOMA_IGG_VS_IGA_DN	-0.99	0.33	0	0	0	not significant
3787	REACTOME_IRS_MEDIATED_SIGNALLING	-0.99	0.33	0	0	0	not significant
3788	GO_GOLGI_VESICLE_BUDDING	-0.99	0.32	0	0	0	not significant
3789	GO_HEART_PROCESS	-0.99	0.32	0	0	0	not significant
3790	GO_POSTSYNAPTIC_NEUROTRANSMITTER_RECEPTOR_INTERNALIZATION	-0.99	0.32	0	0	0	not significant
3791	GO_SUPEROXIDE_ANION_GENERATION	-0.99	0.32	0	0	0	not significant
3792	GO_UBIQUITIN_LIKE_PROTEIN_CONJUGATING_ENZYME_BINDING	-0.99	0.32	0	0	0	not significant
3793	HANN_RESISTANCE_TO_BCL2_INHIBITOR_UP	-0.99	0.32	0	0	0	not significant
3794	REACTOME_ASPARAGINE_N_LINKED_GLYCOSYLATION	-0.99	0.32	0	0	0	not significant
3795	REACTOME_DOWNREGULATION_OF_ERBB4_SIGNALING	-0.99	0.32	0	0	0	not significant
3796	SU_LIVER	-0.99	0.32	0	0	0	not significant
3797	DACOSTA_UV_RESPONSE_VIA_ERCC3_TTD_UP	-0.99	0.31	0	0	0	not significant
3798	GO_CARDIAC_MYOFIBRIL_ASSEMBLY	-0.99	0.31	0	0	0	not significant
3799	GO_REGULATION_OF_PEPTIDASE_ACTIVITY	-0.99	0.31	0	0	0	not significant
3800	GO_REGULATION_OF_PROTEIN_HOMODIMERIZATION_ACTIVITY	-0.99	0.31	0	0	0	not significant
3801	GO_RETINA_LAYER_FORMATION	-0.99	0.31	0	0	0	not significant
3802	GO_SMALL_GTPASE_BINDING	-0.99	0.31	0	0	0	not significant
3803	REACTOME_INOSITOL_PHOSPHATE_METABOLISM	-0.99	0.31	0	0	0	not significant

3804	GO_IRON_ION_TRANSMEMBRANE_TRANSPORTER_ACTIVITY	-0.99	0.3	0	0	0	not significant
3805	GO_POSITIVE_REGULATION_OF_MONOOXYGENASE_ACTIVITY	-0.99	0.3	0	0	0	not significant
3806	GO_REGULATION_OF_KERATINOCYTE_DIFFERENTIATION	-0.99	0.3	0	0	0	not significant
3807	GO_REGULATION_OF_PROTEIN_LOCALIZATION_TO_CENTROSOME	-0.99	0.3	0	0	0	not significant
3808	GO_REGULATION_OF_VASCULOGENESIS	-0.99	0.3	0	0	0	not significant
3809	GO_INTRAMOLECULAR_OXIDOREDUCTASE_ACTIVITY_INTERCONVERTING_ALDOSE	-0.99	0.29	0	0	0	not significant
3810	GO_NEGATIVE_REGULATION_OF_PHOSPHATIDYLINOSITOL_3_KINASE_SIGNALING	-0.99	0.29	0	0	0	not significant
3811	GO_PEPTIDYL_LYSINE_MODIFICATION	-0.99	0.29	0	0	0	not significant
3812	GO_PHOTORECEPTOR_DISC_MEMBRANE	-0.99	0.29	0	0	0	not significant
3813	GO_UTP_BIOSYNTHETIC_PROCESS	-0.99	0.29	0	0	0	not significant
3814	GO_CYCLIC_NUCLEOTIDE_BIOSYNTHETIC_PROCESS	-0.99	0.28	0	0	0	not significant
3815	GO_HISTONE_H2A_K63_LINKED_UBIQUITINATION	-0.99	0.28	0	0	0	not significant
3816	GO_REGULATION_OF_CYCLIC_NUCLEOTIDE_PHOSPHODIESTERASE_ACTIVITY	-0.99	0.28	0	0	0	not significant
3817	GO_LSM1_7_PAT1_COMPLEX	-0.99	0.27	0	0	0	not significant
3818	GO_GLOBAL_GENOME_NUCLEOTIDE_EXCISION_REPAIR	-0.98	0.34	0	0	0	not significant
3819	GO_REGULATION_OF_MUSCLE_SYSTEM_PROCESS	-0.98	0.34	0	0	0	not significant
3820	CHR16Q22	-0.98	0.33	0	0	0	not significant
3821	GO_CERAMIDE_BINDING	-0.98	0.33	0	0	0	not significant
3822	GO_N_METHYLTRANSFERASE_ACTIVITY	-0.98	0.33	0	0	0	not significant
3823	GO_POSITIVE_REGULATION_OF_SMOOTH_MUSCLE_CELL_MIGRATION	-0.98	0.33	0	0	0	not significant
3824	GO_REGULATION_OF_NUCLEASE_ACTIVITY	-0.98	0.33	0	0	0	not significant
3825	RIZKI_TUMOR_INVASIVENESS_3D_UP	-0.98	0.33	0	0	0	not significant
3826	SMIRNOV_RESPONSE_TO_IR_6HR_UP	-0.98	0.33	0	0	0	not significant
3827	BIOCARTA_AGR_PATHWAY	-0.98	0.32	0	0	0	not significant
3828	BIOCARTA_CALCINEURIN_PATHWAY	-0.98	0.32	0	0	0	not significant
3829	CHOI_ATL_CHRONIC_VS_ACUTE_DN	-0.98	0.32	0	0	0	not significant
3830	CHR9P21	-0.98	0.32	0	0	0	not significant
3831	FIGUEROA_AML_METHYLATION_CLUSTER_2_UP	-0.98	0.32	0	0	0	not significant
3832	GO_BASE_EXCISION_REPAIR_AP_SITE_FORMATION	-0.98	0.32	0	0	0	not significant
3833	GO_CARDIAC_CELL_DEVELOPMENT	-0.98	0.32	0	0	0	not significant
3834	GO_COATED_MEMBRANE	-0.98	0.32	0	0	0	not significant
3835	GO_PHOSPHATIDYLINOSITOL_3_5_BISPHOSPHATE_PHOSPHATASE_ACTIVITY	-0.98	0.32	0	0	0	not significant
3836	GO_PHOSPHATIDYLINOSITOL_3_KINASE_BINDING	-0.98	0.32	0	0	0	not significant
3837	GO_POSITIVE_REGULATION_OF_CALCIUM_ION_TRANSMEMBRANE_TRANSPORTER_	-0.98	0.32	0	0	0	not significant
3838	GO_PRE_MIRNA_PROCESSING	-0.98	0.32	0	0	0	not significant
3839	GO_REGULATION_OF_CELL_MATRIX_ADHESION	-0.98	0.32	0	0	0	not significant
3840	GO_REGULATION_OF_ER_TO_GOLGI_VESICLE_MEDIATED_TRANSPORT	-0.98	0.32	0	0	0	not significant
3841	GO_REGULATION_OF_MEMBRANE_DEPOLARIZATION	-0.98	0.32	0	0	0	not significant
3842	MULLIGHAN_NPM1_MUTATED_SIGNATURE_2_UP	-0.98	0.32	0	0	0	not significant
3843	REACTOME_SHC1_EVENTS_IN_ERBB2_SIGNALING	-0.98	0.32	0	0	0	not significant
3844	WALLACE_PROSTATE_CANCER_UP	-0.98	0.32	0	0	0	not significant
3845	GO_PIGMENT_GRANULE_MATURATION	-0.98	0.31	0	0	0	not significant
3846	GO_POSITIVE_REGULATION_OF_VASCULOGENESIS	-0.98	0.31	0	0	0	not significant
3847	GO_REGULATION_OF_CELL_PROJECTION_ASSEMBLY	-0.98	0.31	0	0	0	not significant
3848	OUYANG_PROSTATE_CANCER_PROGRESSION_DN	-0.98	0.31	0	0	0	not significant
3849	REACTOME_SIGNALLING_TO_ERKS	-0.98	0.31	0	0	0	not significant
3850	GO_CORONARY_ARTERY_MORPHOGENESIS	-0.98	0.3	0	0	0	not significant
3851	GO_DEAMINASE_ACTIVITY	-0.98	0.3	0	0	0	not significant
3852	GO_IMMUNOLOGICAL_SYNAPSE_FORMATION	-0.98	0.3	0	0	0	not significant
3853	GO_LAYER_FORMATION_IN_CEREBRAL_CORTEX	-0.98	0.3	0	0	0	not significant
3854	GO_NEGATIVE_REGULATION_OF_PHAGOCYTOSIS	-0.98	0.3	0	0	0	not significant
3855	GO_PHOSPHATIDYLGLYCEROL_METABOLIC_PROCESS	-0.98	0.3	0	0	0	not significant
3856	GO_PYRIMIDINE_RIBONUCLEOSIDE_TRIPHOSPHATE_BIOSYNTHETIC_PROCESS	-0.98	0.3	0	0	0	not significant
3857	GO_REGULATION_OF_MONOCYTE_DIFFERENTIATION	-0.98	0.3	0	0	0	not significant
3858	GO_REGULATION_OF_RUFFLE_ASSEMBLY	-0.98	0.3	0	0	0	not significant
3859	GO_RESPONSE_TO_NITROSATIVE_STRESS	-0.98	0.3	0	0	0	not significant
3860	MOOTHA_TCA	-0.98	0.3	0	0	0	not significant
3861	REACTOME_PLASMA_LIPOPROTEIN_ASSEMBLY	-0.98	0.3	0	0	0	not significant
3862	REACTOME_REPRESSION_OF_WNT_TARGET_GENES	-0.98	0.3	0	0	0	not significant
3863	REACTOME_SMAC_XIAP_REGULATED_APOPTOTIC_RESPONSE	-0.98	0.3	0	0	0	not significant
3864	CHR5Q31	-0.98	0.29	0	0	0	not significant
3865	GO_B_CELL_LINEAGE_COMMITMENT	-0.98	0.29	0	0	0	not significant
3866	GO_COHESIN_LOADING	-0.98	0.29	0	0	0	not significant
3867	GO_MODIFICATION_OF_POSTSYNAPTIC_ACTIN_CYTOSKELETON	-0.98	0.29	0	0	0	not significant
3868	GO_NK_T_CELL_ACTIVATION	-0.98	0.29	0	0	0	not significant
3869	GO_PROTEIN_SERINE_THREONINE_PHOSPHATASE_INHIBITOR_ACTIVITY	-0.98	0.29	0	0	0	not significant
3870	REACTOME_SIGNALING_BY_NTRK2_TRKB	-0.98	0.29	0	0	0	not significant
3871	CHR2P13	-0.98	0.28	0	0	0	not significant
3872	GO_CELLULAR_RESPONSE_TO_ESTROGEN_STIMULUS	-0.98	0.28	0	0	0	not significant
3873	GO_PEROXISOME_PROLIFERATOR_ACTIVATED_RECEPTOR_BINDING	-0.98	0.28	0	0	0	not significant
3874	GO_POSITIVE_REGULATION_BY_HOST_OF_VIRAL_GENOME_REPLICATION	-0.98	0.28	0	0	0	not significant
3875	GO_POSITIVE_REGULATION_OF_INTRACELLULAR_TRANSPORT	-0.98	0.28	0	0	0	not significant
3876	GO_PROTEIN_LIPOYLATION	-0.98	0.28	0	0	0	not significant
3877	GO_PYRUVATE_DEHYDROGENASE_COMPLEX	-0.98	0.28	0	0	0	not significant
3878	GO_REACTIVE_OXYGEN_SPECIES_METABOLIC_PROCESS	-0.98	0.28	0	0	0	not significant
3879	GO_REGULATION_OF_PROTEIN_LOCALIZATION_TO_CILIUM	-0.98	0.28	0	0	0	not significant
3880	GO_PARANODE_REGION_OF_AXON	-0.98	0.27	0	0	0	not significant
3881	BENPORATH_ES_2	-0.97	0.33	0	0	0	not significant
3882	GO_BLOOD_VESSEL_REMODELING	-0.97	0.33	0	0	0	not significant
3883	GO_ATPASE_BINDING	-0.97	0.32	0	0	0	not significant
3884	GO_MITOTIC_CYTOKINETIC_PROCESS	-0.97	0.32	0	0	0	not significant
3885	GO_NEGATIVE_REGULATION_OF_RESPONSE_TO_DRUG	-0.97	0.32	0	0	0	not significant
3886	GO_STRIATED_MUSCLE_CELL_PROLIFERATION	-0.97	0.32	0	0	0	not significant
3887	REACTOME_BASE_EXCISION_REPAIR_AP_SITE_FORMATION	-0.97	0.32	0	0	0	not significant
3888	AIGNER_ZEB1_TARGETS	-0.97	0.31	0	0	0	not significant
3889	GO_POSITIVE_REGULATION_OF_EPITHELIAL_TO_MESENCHYMAL_TRANSITION	-0.97	0.31	0	0	0	not significant
3890	GO_REGION_OF_CYTOSOL	-0.97	0.31	0	0	0	not significant
3891	GO_SPERMATID_NUCLEUS_DIFFERENTIATION	-0.97	0.31	0	0	0	not significant
3892	JIANG_AGING_HYPOTHALAMUS_UP	-0.97	0.31	0	0	0	not significant
3893	KEGG_RIBOFLAVIN_METABOLISM	-0.97	0.31	0	0	0	not significant
3894	BAKER_HEMATOPOIESIS_STAT3_TARGETS	-0.97	0.3	0	0	0	not significant
3895	GO_CYTOPLASMIC_TRANSLATIONAL_INITIATION	-0.97	0.3	0	0	0	not significant
3896	GO_POSTSYNAPTIC_CYTOSOL	-0.97	0.3	0	0	0	not significant
3897	GO_PROTEIN_FOLDING_CHAPERONE	-0.97	0.3	0	0	0	not significant
3898	GO_PURINE_NUCLEOSIDE_BIOSYNTHETIC_PROCESS	-0.97	0.3	0	0	0	not significant
3899	GO_REGULATION_OF_TRANSLATIONAL_INITIATION_IN_RESPONSE_TO_STRESS	-0.97	0.3	0	0	0	not significant
3900	GO_RESPONSE_TO_VITAMIN_D	-0.97	0.3	0	0	0	not significant
3901	KYNG_WERNER_SYNDROM_DN	-0.97	0.3	0	0	0	not significant
3902	PEDERSEN_METASTASIS_BY_ERBB2_ISOFORM_6	-0.97	0.3	0	0	0	not significant
3903	REACTOME_BILE_ACID_AND_BILE_SALT_METABOLISM	-0.97	0.3	0	0	0	not significant
3904	REACTOME_CHAPERONE_MEDIATED_AUTOPHAGY	-0.97	0.3	0	0	0	not significant
3905	REACTOME_DUAL_INCISION_IN_TC_NER	-0.97	0.3	0	0	0	not significant
3906	REACTOME_MET_PROMOTES_CELL_MOTILITY	-0.97	0.3	0	0	0	not significant
3907	REACTOME_SYNTHESIS_OF_BILE_ACIDS_AND_BILE_SALTS	-0.97	0.3	0	0	0	not significant
3908	REACTOME_UPTAKE_AND_FUNCTION_OF_ANTHRAX_TOXINS	-0.97	0.3	0	0	0	not significant
3909	SCHAEFFER_PROSTATE_DEVELOPMENT_AND_CANCER_BOX1_UP	-0.97	0.3	0	0	0	not significant
3910	ALONSO_METASTASIS_UP	-0.97	0.29	0	0	0	not significant
3911	BIOCARTA_HIVNEF_PATHWAY	-0.97	0.29	0	0	0	not significant
3912	GO_EXIT_FROM_MITOSIS	-0.97	0.29	0	0	0	not significant

3913	GO_G_QUADRUPLEX_DNA_BINDING	-0.97	0.29	0	0	0	not significant
3914	GO_METHYLATION_DEPENDENT_CHROMATIN_SILENCING	-0.97	0.29	0	0	0	not significant
3915	GO_NEGATIVE_REGULATION_OF_TRANSPORTER_ACTIVITY	-0.97	0.29	0	0	0	not significant
3916	GO_NEUROTROPHIN_TRK_RECEPTOR_SIGNALING_PATHWAY	-0.97	0.29	0	0	0	not significant
3917	GO_REGULATION_OF_CALCIUM_ION_DEPENDENT_EXOCYTOSIS	-0.97	0.29	0	0	0	not significant
3918	GO_REGULATION_OF_CALCIUM_ION_TRANSMEMBRANE_TRANSPORTER_ACTIVITY	-0.97	0.29	0	0	0	not significant
3919	KEGG_CYTOSOLIC_DNA_SENSING_PATHWAY	-0.97	0.29	0	0	0	not significant
3920	KOINUMA_COLON_CANCER_MSI_DN	-0.97	0.29	0	0	0	not significant
3921	MYLLYKANGAS_AMPLIFICATION_HOT_SPOT_24	-0.97	0.29	0	0	0	not significant
3922	GO_COLUMNAR_CUBOIDAL_EPITHELIAL_CELL_DIFFERENTIATION	-0.97	0.28	0	0	0	not significant
3923	GO_REGULATION_OF_RIBONUCLEOPROTEIN_COMPLEX_LOCALIZATION	-0.97	0.28	0	0	0	not significant
3924	GO_VASCULAR_ASSOCIATED_SMOOTH_MUSCLE_CELL_APOPTOTIC_PROCESS	-0.97	0.28	0	0	0	not significant
3925	GO_NEUROTRANSMITTER_RECEPTOR_INTERNALIZATION	-0.97	0.27	0	0	0	not significant
3926	GO_SCF_UBIQUITIN_LIGASE_COMPLEX	-0.97	0.27	0	0	0	not significant
3927	GO_VIRAL_TRANSLATIONAL_TERMINATION_REINITIATION	-0.97	0.27	0	0	0	not significant
3928	REACTOME_METHIONINE_SALVAGE_PATHWAY	-0.97	0.27	0	0	0	not significant
3929	SCHAEFFER_PROSTATE_DEVELOPMENT_6HR_UP	-0.97	0.27	0	0	0	not significant
3930	SCHUHMACHER_MYC_TARGETS_DN	-0.97	0.27	0	0	0	not significant
3931	GO_CELL_CORTEX	-0.97	0.26	0	0	0	not significant
3932	GO_REGULATION_OF_GLUCOSE_TRANSMEMBRANE_TRANSPORT	-0.97	0.26	0	0	0	not significant
3933	GO_TUMOR_NECROSIS_FACTOR_MEDIATED_SIGNALING_PATHWAY	-0.97	0.26	0	0	0	not significant
3934	GO_VESICLE_TRANSPORT_ALONG_ACTIN_FILAMENT	-0.97	0.26	0	0	0	not significant
3935	GO_REGULATION_OF_ORGANELLE_ASSEMBLY	-0.97	0.25	0	0	0	not significant
3936	RICKMAN_METASTASIS_DN	-0.97	0.24	0	0	0	not significant
3937	VANOEVELEN_MYOGENESIS_SIN3A_TARGETS	-0.97	0.23	0	0	0	not significant
3938	GO_CELL_GROWTH	-0.97	0.2	0	0	0	not significant
3939	ASGHARZADEH_NEUROBLASTOMA_POOR_SURVIVAL_DN	-0.96	0.32	0	0	0	not significant
3940	GO_NEGATIVE_REGULATION_OF_EXOCYTOSIS	-0.96	0.31	0	0	0	not significant
3941	KYNG_RESPONSE_TO_H2O2_VIA_ERCC6	-0.96	0.31	0	0	0	not significant
3942	CHR2P23	-0.96	0.3	0	0	0	not significant
3943	GO_CORTICAL_CYTOSKELETON_ORGANIZATION	-0.96	0.3	0	0	0	not significant
3944	GO_GTP_METABOLIC_PROCESS	-0.96	0.3	0	0	0	not significant
3945	GO_LABYRINTHINE_LAYER_MORPHOGENESIS	-0.96	0.3	0	0	0	not significant
3946	GO_REGULATION_OF_PRESYNAPSE_ORGANIZATION	-0.96	0.3	0	0	0	not significant
3947	GO_S_ADENOSYL_L_METHIONINE_BINDING	-0.96	0.3	0	0	0	not significant
3948	HEIDENBLAD_AMPLIFIED_IN_PANCREATIC_CANCER	-0.96	0.3	0	0	0	not significant
3949	REACTOME_PRE_NOTCH_PROCESSING_IN_GOLGI	-0.96	0.3	0	0	0	not significant
3950	BIOCARTA_BAD_PATHWAY	-0.96	0.29	0	0	0	not significant
3951	GO_POSITIVE_REGULATION_OF_CELL_GROWTH	-0.96	0.29	0	0	0	not significant
3952	GO_POSITIVE_REGULATION_OF_MITOTIC_SISTER_CHROMATID_SEGREGATION	-0.96	0.29	0	0	0	not significant
3953	GO_POSITIVE_REGULATION_OF_OXIDOREDUCTASE_ACTIVITY	-0.96	0.29	0	0	0	not significant
3954	GO_RETROMER_COMPLEX	-0.96	0.29	0	0	0	not significant
3955	GO_SOMITE_DEVELOPMENT	-0.96	0.29	0	0	0	not significant
3956	KERLEY_RESPONSE_TO_CISPLATIN_UP	-0.96	0.29	0	0	0	not significant
3957	PID_NOTCH_PATHWAY	-0.96	0.29	0	0	0	not significant
3958	REACTOME_NEF_MEDIATED_CD4_DOWN_REGULATION	-0.96	0.29	0	0	0	not significant
3959	REACTOME_RAP1_SIGNALLING	-0.96	0.29	0	0	0	not significant
3960	ZHENG_IL22_SIGNALING_DN	-0.96	0.29	0	0	0	not significant
3961	ZHONG_SECRETOME_OF_LUNG_CANCER_AND_ENDOTHELIUM	-0.96	0.29	0	0	0	not significant
3962	BIOCARTA_AKAP13_PATHWAY	-0.96	0.28	0	0	0	not significant
3963	CHR15Q13	-0.96	0.28	0	0	0	not significant
3964	CHR7Q33	-0.96	0.28	0	0	0	not significant
3965	GO_CELLULAR_RESPONSE_TO_NUTRIENT	-0.96	0.28	0	0	0	not significant
3966	GO_REGULATION_OF_GRANULOCYTE_DIFFERENTIATION	-0.96	0.28	0	0	0	not significant
3967	GO_REGULATION_OF_SUPEROXIDE_ANION_GENERATION	-0.96	0.28	0	0	0	not significant
3968	GO_RESPONSE_TO_HEPATOCYTE_GROWTH_FACTOR	-0.96	0.28	0	0	0	not significant
3969	PID_RAC1_PATHWAY	-0.96	0.28	0	0	0	not significant
3970	YE_METASTATIC_LIVER_CANCER	-0.96	0.28	0	0	0	not significant
3971	BIOCARTA_CBL_PATHWAY	-0.96	0.27	0	0	0	not significant
3972	DAZARD_UV_RESPONSE_CLUSTER_G1	-0.96	0.27	0	0	0	not significant
3973	FIRESTEIN_CTNNB1_PATHWAY_AND_PROLIFERATION	-0.96	0.27	0	0	0	not significant
3974	GO_ELONGATOR_HOLOENZYME_COMPLEX	-0.96	0.27	0	0	0	not significant
3975	GO_NEGATIVE_REGULATION_OF_PEPTIDASE_ACTIVITY	-0.96	0.27	0	0	0	not significant
3976	GO_NEGATIVE_REGULATION_OF_PROTEIN_COMPLEX_ASSEMBLY	-0.96	0.27	0	0	0	not significant
3977	GO_PANCREATIC_JUICE_SECRETION	-0.96	0.27	0	0	0	not significant
3978	GO_PIGMENT_ACCUMULATION	-0.96	0.27	0	0	0	not significant
3979	GO_UBIQUITIN_CONJUGATING_ENZYME_BINDING	-0.96	0.27	0	0	0	not significant
3980	GO_CYCLOOXYGENASE_PATHWAY	-0.96	0.26	0	0	0	not significant
3981	GO_H4_HISTONE_ACETYLTRANSFERASE_COMPLEX	-0.96	0.26	0	0	0	not significant
3982	GO_INTRINSIC_APOPTOTIC_SIGNALING_PATHWAY_IN_RESPONSE_TO_HYPOXIA	-0.96	0.26	0	0	0	not significant
3983	GO_NEGATIVE_REGULATION_OF_CELL_DIVISION	-0.96	0.26	0	0	0	not significant
3984	MATTIOLI_MGUS_VS_PCL	-0.96	0.26	0	0	0	not significant
3985	MCBRYAN_TERMINAL_END_BUD_UP	-0.96	0.26	0	0	0	not significant
3986	GO_NEGATIVE_REGULATION_OF_MAPK_CASCADE	-0.96	0.24	0	0	0	not significant
3987	BENPORATH_OCT4_TARGETS	-0.96	0.23	0	0	0	not significant
3988	GO_POSITIVE_REGULATION_OF_PROTEIN_COMPLEX_ASSEMBLY	-0.96	0.23	0	0	0	not significant
3989	GRYDER_PAX3FOXO1_TOP_ENHANCERS	-0.96	0.18	0	0	0	not significant
3990	GO_CILIUM	-0.96	0.17	0	0	0	not significant
3991	GO_NUCLEAR_CHROMATIN	-0.96	0.17	0	0	0	not significant
3992	GO_ZINC_ION_BINDING	-0.96	0.14	0	0	0	not significant
3993	BIOCARTA_CTCF_PATHWAY	-0.95	0.31	0	0	0	not significant
3994	GO_BIOTIN_METABOLIC_PROCESS	-0.95	0.3	0	0	0	not significant
3995	GO_CATION_CATION_ANTIPORTER_ACTIVITY	-0.95	0.3	0	0	0	not significant
3996	GO_CALCINEURIN_MEDIATED_SIGNALING	-0.95	0.29	0	0	0	not significant
3997	GO_NEGATIVE_REGULATION_OF_PROTEASOMAL_PROTEIN_CATABOLIC_PROCESS	-0.95	0.29	0	0	0	not significant
3998	GO_POSITIVE_REGULATION_OF_OXIDATIVE_STRESS_INDUCED_CELL_DEATH	-0.95	0.29	0	0	0	not significant
3999	CHR7Q32	-0.95	0.28	0	0	0	not significant
4000	GO_3_UTR_MEDIATED_MRNA_STABILIZATION	-0.95	0.28	0	0	0	not significant
4001	GO_MONONUCLEAR_CELL_DIFFERENTIATION	-0.95	0.28	0	0	0	not significant
4002	GO_MRNA_3_SPLICE_SITE_RECOGNITION	-0.95	0.28	0	0	0	not significant
4003	GO_NEGATIVE_REGULATION_OF_KIDNEY_DEVELOPMENT	-0.95	0.28	0	0	0	not significant
4004	GO_NUCLEOSOME_POSITIONING	-0.95	0.28	0	0	0	not significant
4005	GO_POSITIVE_REGULATION_OF_RUFFLE_ASSEMBLY	-0.95	0.28	0	0	0	not significant
4006	GO_REGULATION_OF_INTRINSIC_APOPTOTIC_SIGNALING_PATHWAY_IN_RESPONSE	-0.95	0.28	0	0	0	not significant
4007	GO_REGULATION_OF_MYOBLAST_DIFFERENTIATION	-0.95	0.28	0	0	0	not significant
4008	GO_SMALL_RNA_LOADING_ONTO_RISC	-0.95	0.28	0	0	0	not significant
4009	LI_WILMS_TUMOR_VS_FETAL_KIDNEY_2_UP	-0.95	0.28	0	0	0	not significant
4010	PID_ERBB1_RECEPTOR_PROXIMAL_PATHWAY	-0.95	0.28	0	0	0	not significant
4011	REACTOME_CITRIC_ACID_CYCLE_TCA_CYCLE	-0.95	0.28	0	0	0	not significant
4012	THEODOROU_MAMMARY_TUMORIGENESIS	-0.95	0.28	0	0	0	not significant
4013	CHR8Q21	-0.95	0.27	0	0	0	not significant
4014	CLIMENT_BREAST_CANCER_COPY_NUMBER_DN	-0.95	0.27	0	0	0	not significant
4015	GENTILE_UV_RESPONSE_CLUSTER_D2	-0.95	0.27	0	0	0	not significant
4016	GO_BRONCHUS_DEVELOPMENT	-0.95	0.27	0	0	0	not significant
4017	GO_ER_MEMBRANE_PROTEIN_COMPLEX	-0.95	0.27	0	0	0	not significant
4018	GO_FUCOSYLTRANSFERASE_ACTIVITY	-0.95	0.27	0	0	0	not significant
4019	GO_GLUTAMATE_RECEPTOR_BINDING	-0.95	0.27	0	0	0	not significant
4020	GO_NEURON_REMODELING	-0.95	0.27	0	0	0	not significant
4021	GO_PEPTIDYL_DIPHTHAMIDE_BIOSYNTHETIC_PROCESS_FROM_PEPTIDYL_HISTIDIN	-0.95	0.27	0	0	0	not significant

4022	GO_PEROXIREDOXIN_ACTIVITY	-0.95	0.27	0	0	0	not significant
4023	GO_POSITIVE_REGULATION_OF_RESPONSE_TO_OXIDATIVE_STRESS	-0.95	0.27	0	0	0	not significant
4024	GO_REGULATION_OF_METANEPHROS_DEVELOPMENT	-0.95	0.27	0	0	0	not significant
4025	GO_RESPONSE_TO_BRONCHODILATOR	-0.95	0.27	0	0	0	not significant
4026	GO_SIN3_COMPLEX	-0.95	0.27	0	0	0	not significant
4027	GO_TELOMERASE_HOLOENZYME_COMPLEX_ASSEMBLY	-0.95	0.27	0	0	0	not significant
4028	CHR2Q35	-0.95	0.26	0	0	0	not significant
4029	GO_MACROLIDE_BINDING	-0.95	0.26	0	0	0	not significant
4030	GO_NEGATIVE_REGULATION_OF_SUBSTRATE_ADHESION_DEPENDENT_CELL_SPRI	-0.95	0.26	0	0	0	not significant
4031	GO_PHOSPHOLIPID_DEPHOSPHORYLATION	-0.95	0.26	0	0	0	not significant
4032	GO_POSITIVE_REGULATION_OF_SMOOTH_MUSCLE_CELL_DIFFERENTIATION	-0.95	0.26	0	0	0	not significant
4033	GO_REGULATION_OF_NUCLEAR_TRANSCRIBED_MRNA_POLY_A_TAIL_SHORTENING	-0.95	0.26	0	0	0	not significant
4034	GO_RESPONSE_TO_THYROID_HORMONE	-0.95	0.26	0	0	0	not significant
4035	NIELSEN_SYNOVIAL_SARCOMA_UP	-0.95	0.26	0	0	0	not significant
4036	SIG_CHEMOTAXIS	-0.95	0.26	0	0	0	not significant
4037	TBK1.DN.48HRS_UP	-0.95	0.26	0	0	0	not significant
4038	CHR7Q34	-0.95	0.25	0	0	0	not significant
4039	GO_CDC73_PAF1_COMPLEX	-0.95	0.25	0	0	0	not significant
4040	GO_CYSTEINE_TYPE_PEPTIDASE_ACTIVITY	-0.95	0.25	0	0	0	not significant
4041	GO_D1_DOPAMINE_RECEPTOR_BINDING	-0.95	0.25	0	0	0	not significant
4042	GO_MONOVALENT_CATION_PROTON_ANTIPORTER_ACTIVITY	-0.95	0.25	0	0	0	not significant
4043	GO_PARASPECKLES	-0.95	0.25	0	0	0	not significant
4044	GO_STEROID_HORMONE_SECRETION	-0.95	0.25	0	0	0	not significant
4045	PRC2_EZH2_UP.V1_UP	-0.95	0.25	0	0	0	not significant
4046	GO_CAMERA_TYPE_EYE_PHOTORECEPTOR_CELL_DIFFERENTIATION	-0.95	0.24	0	0	0	not significant
4047	GO_NEGATIVE_REGULATION_OF_AUTOPHAGY	-0.95	0.24	0	0	0	not significant
4048	GO_POSITIVE_REGULATION_OF_BINDING	-0.95	0.24	0	0	0	not significant
4049	GO_POSITIVE_REGULATION_OF_LAMELLIPODIUM_MORPHOGENESIS	-0.95	0.24	0	0	0	not significant
4050	GO_REGULATION_OF_DENDRITE_DEVELOPMENT	-0.95	0.24	0	0	0	not significant
4051	GO_AMIDE_BINDING	-0.95	0.23	0	0	0	not significant
4052	GO_DEVELOPMENTAL_CELL_GROWTH	-0.95	0.22	0	0	0	not significant
4053	GO_INTRACELLULAR_STEROID_HORMONE_RECEPTOR_SIGNALING_PATHWAY	-0.95	0.22	0	0	0	not significant
4054	GO_PATTERN_SPECIFICATION_PROCESS	-0.95	0.21	0	0	0	not significant
4055	KOINUMA_TARGETS_OF_SMAD2_OR_SMAD3	-0.95	0.18	0	0	0	not significant
4056	GO_REGULATION_OF_SYSTEM_PROCESS	-0.95	0.17	0	0	0	not significant
4057	GO_EPITHELIUM_DEVELOPMENT	-0.95	0.12	0	0	0	not significant
4058	GO_TRANSITION_METAL_ION_BINDING	-0.95	0.12	0	0	0	not significant
4059	GO_CELL_CELL_SIGNALING	-0.95	0.1	0	0	0	not significant
4060	GO_POSITIVE_REGULATION_OF_EXTRINSIC_APOPTOTIC_SIGNALING_PATHWAY_VIA	-0.94	0.29	0	0	0	not significant
4061	TONKS_TARGETS_OF_RUNX1_RUNX1T1_FUSION_GRANULOCYTE_UP	-0.94	0.29	0	0	0	not significant
4062	BIOCARTA_BARRESTIN_SRC_PATHWAY	-0.94	0.28	0	0	0	not significant
4063	CHANG_IMMORTALIZED_BY_HPV31_UP	-0.94	0.28	0	0	0	not significant
4064	GO_CELLULAR_COMPONENT_DISASSEMBLY_INVOLVED_IN_EXECUTION_PHASE_OF	-0.94	0.28	0	0	0	not significant
4065	GO_COMPLEMENT_ACTIVATION	-0.94	0.28	0	0	0	not significant
4066	GO_CYTOCHROME_COMPLEX	-0.94	0.28	0	0	0	not significant
4067	GO_DETECTION_OF_STIMULUS_INVOLVED_IN_SENSORY_PERCEPTION	-0.94	0.28	0	0	0	not significant
4068	GO_GOLGI_TO_VACUOLE_TRANSPORT	-0.94	0.28	0	0	0	not significant
4069	GO_INHIBITORY_SYNAPSE	-0.94	0.28	0	0	0	not significant
4070	GO_MAMMARY_GLAND_MORPHOGENESIS	-0.94	0.28	0	0	0	not significant
4071	GO_MITOTIC_CHROMOSOME_CONDENSATION	-0.94	0.28	0	0	0	not significant
4072	JAZAG_TGFB1_SIGNALING_VIA_SMAD4_DN	-0.94	0.28	0	0	0	not significant
4073	GO_9PLUS0_NON_MOTILE_CILIUM	-0.94	0.27	0	0	0	not significant
4074	GO_AXONEME_PART	-0.94	0.27	0	0	0	not significant
4075	GO_CELLULAR_RESPONSE_TO_OXYGEN_RADICAL	-0.94	0.27	0	0	0	not significant
4076	GO_NEGATIVE_REGULATION_OF_CARBOHYDRATE_METABOLIC_PROCESS	-0.94	0.27	0	0	0	not significant
4077	GO_REGULATION_OF_FILOPODIUM_ASSEMBLY	-0.94	0.27	0	0	0	not significant
4078	GO_TRACHEA_MORPHOGENESIS	-0.94	0.27	0	0	0	not significant
4079	GRASEMANN_RETINOBLASTOMA_WITH_6P_AMPLIFICATION	-0.94	0.27	0	0	0	not significant
4080	KEGG_SELENOAMINO_ACID_METABOLISM	-0.94	0.27	0	0	0	not significant
4081	NADERI_BREAST_CANCER_PROGNOSIS_DN	-0.94	0.27	0	0	0	not significant
4082	REACTOME_MRNA_DECAY_BY_3_TO_5_EXORIBONUCLEASE	-0.94	0.27	0	0	0	not significant
4083	BIOCARTA_ARF_PATHWAY	-0.94	0.26	0	0	0	not significant
4084	CHR4Q12	-0.94	0.26	0	0	0	not significant
4085	GENTILE_UV_RESPONSE_CLUSTER_D8	-0.94	0.26	0	0	0	not significant
4086	GO_BETA_CATENIN_DESTRUCTION_COMPLEX	-0.94	0.26	0	0	0	not significant
4087	GO_CARDIAC_CONDUCTION_SYSTEM_DEVELOPMENT	-0.94	0.26	0	0	0	not significant
4088	GO_CORONARY_VASCULATURE_MORPHOGENESIS	-0.94	0.26	0	0	0	not significant
4089	GO_PEPTIDYL_HISTIDINE_MODIFICATION	-0.94	0.26	0	0	0	not significant
4090	GO_POSITIVE_REGULATION_OF_AMINO_ACID_TRANSPORT	-0.94	0.26	0	0	0	not significant
4091	GO_POSITIVE_REGULATION_OF_CELLULAR_RESPONSE_TO_INSULIN_STIMULUS	-0.94	0.26	0	0	0	not significant
4092	GO_REGULATION_OF_CHONDROCYTE_DIFFERENTIATION	-0.94	0.26	0	0	0	not significant
4093	KEGG_AMINO_SUGAR_AND_NUCLEOTIDE_SUGAR_METABOLISM	-0.94	0.26	0	0	0	not significant
4094	KEGG_PYRUVATE_METABOLISM	-0.94	0.26	0	0	0	not significant
4095	KOKKINAKIS_METHIONINE_DEPRIVATION_48HR_DN	-0.94	0.26	0	0	0	not significant
4096	LABBE_TGFB1_TARGETS_DN	-0.94	0.26	0	0	0	not significant
4097	PID_IL2_PI3K_PATHWAY	-0.94	0.26	0	0	0	not significant
4098	REACTOME_RAS_ACTIVATION_UPON_CA2PLUS_INFLUX_THROUGH_NMDA_RECEPTO	-0.94	0.26	0	0	0	not significant
4099	XU_CREBBP_TARGETS_UP	-0.94	0.26	0	0	0	not significant
4100	FLOTHO_PEDIATRIC_ALL_THERAPY_RESPONSE_DN	-0.94	0.25	0	0	0	not significant
4101	GO_DNA_DEAMINATION	-0.94	0.25	0	0	0	not significant
4102	GO_MEMBRANE_DEPOLARIZATION_DURING_CARDIAC_MUSCLE_CELL_ACTION_POT	-0.94	0.25	0	0	0	not significant
4103	GO_OBSERVATIONAL_LEARNING	-0.94	0.25	0	0	0	not significant
4104	GO_POSTSYNAPTIC_DENSITY_ASSEMBLY	-0.94	0.25	0	0	0	not significant
4105	GO_SINGLE_STRANDED_VIRAL_RNA_REPLICATION_VIA_DOUBLE_STRANDED_DNA	-0.94	0.25	0	0	0	not significant
4106	GO_SUBSTRATE_ADHESION_DEPENDENT_CELL_SPREADING	-0.94	0.25	0	0	0	not significant
4107	GO_SUCCINYL_COA_METABOLIC_PROCESS	-0.94	0.25	0	0	0	not significant
4108	LUI_THYROID_CANCER_CLUSTER_4	-0.94	0.25	0	0	0	not significant
4109	REACTOME_RSK_ACTIVATION	-0.94	0.25	0	0	0	not significant
4110	ROYLANCE_BREAST_CANCER_16Q_COPY_NUMBER_UP	-0.94	0.25	0	0	0	not significant
4111	TURASHVILI_BREAST_NORMAL_DUCTAL_VS_LOBULAR_DN	-0.94	0.25	0	0	0	not significant
4112	GO_INSULIN_BINDING	-0.94	0.24	0	0	0	not significant
4113	GO_MUSCLE_HYPERTROPHY	-0.94	0.24	0	0	0	not significant
4114	GO_PROTEIN_LOCALIZATION_TO_CILIUM	-0.94	0.24	0	0	0	not significant
4115	GO_REGULATION_OF_NOTCH_SIGNALING_PATHWAY	-0.94	0.24	0	0	0	not significant
4116	GO_RESPONSE_TO_LEUKEMIA_INHIBITORY_FACTOR	-0.94	0.24	0	0	0	not significant
4117	PID_HIF2PATHWAY	-0.94	0.24	0	0	0	not significant
4118	REACTOME_SIGNALING_BY_BRAF_AND_RAF_FUSIONS	-0.94	0.24	0	0	0	not significant
4119	SEKI_INFLAMMATORY_RESPONSE_LPS_DN	-0.94	0.24	0	0	0	not significant
4120	GO_CONTRACTILE_ACTIN_FILAMENT_BUNDLE_ASSEMBLY	-0.94	0.23	0	0	0	not significant
4121	GO_POSITIVE_REGULATION_OF_PLASMA_MEMBRANE_BOUNDED_CELL_PROJECTIO	-0.94	0.23	0	0	0	not significant
4122	GO_ENHANCER_BINDING	-0.94	0.22	0	0	0	not significant
4123	GO_EPIDERMIS_DEVELOPMENT	-0.94	0.22	0	0	0	not significant
4124	GO_TRANSCRIPTION_COUPLED_NUCLEOTIDE_EXCISION_REPAIR	-0.94	0.22	0	0	0	not significant
4125	YAO_TEMPORAL_RESPONSE_TO_PROGESTERONE_CLUSTER_17	-0.94	0.22	0	0	0	not significant
4126	GO_RETROGRADE_TRANSPORT_ENDOSOME_TO_GOLGI	-0.94	0.21	0	0	0	not significant
4127	GO_ANIMAL_ORGAN_MORPHOGENESIS	-0.94	0.13	0	0	0	not significant
4128	GO_ACETYL_COA_BIOSYNTHETIC_PROCESS_FROM_PYRUVATE	-0.93	0.28	0	0	0	not significant
4129	GO_PEPTIDYL_LYSINE_DIMETHYLATION	-0.93	0.28	0	0	0	not significant
4130	GO_BODY_MORPHOGENESIS	-0.93	0.27	0	0	0	not significant

4131	ONGUSAHA_TP53_TARGETS	-0.93	0.27	0	0	0	not significant
4132	REACTOME_RECEPTOR_MEDIATED_MITOPHAGY	-0.93	0.27	0	0	0	not significant
4133	ROZANOV_MMP14_TARGETS_DN	-0.93	0.27	0	0	0	not significant
4134	BIOCARTA_VIP_PATHWAY	-0.93	0.26	0	0	0	not significant
4135	GO_CORONARY_VASCULATURE_DEVELOPMENT	-0.93	0.26	0	0	0	not significant
4136	GO_CYTOPLASMIC_TRANSLATIONAL_ELONGATION	-0.93	0.26	0	0	0	not significant
4137	GO_EXONUCLEOLYTIC_TRIMMING_INVOLVED_IN_RRNA_PROCESSING	-0.93	0.26	0	0	0	not significant
4138	GO_NEGATIVE_REGULATION_OF_CALCIUM_ION_TRANSPORT_INTO_CYTOSOL	-0.93	0.26	0	0	0	not significant
4139	GO_NEGATIVE_REGULATION_OF_DENDRITE_DEVELOPMENT	-0.93	0.26	0	0	0	not significant
4140	GO_NEGATIVE_REGULATION_OF_OLIGODENDROCYTE_DIFFERENTIATION	-0.93	0.26	0	0	0	not significant
4141	GO_POSITIVE_REGULATION_OF_RESPONSE_TO_CYTOKINE_STIMULUS	-0.93	0.26	0	0	0	not significant
4142	GO_REGULATION_OF_PROTON_TRANSPORT	-0.93	0.26	0	0	0	not significant
4143	GO_STRUCTURAL_CONSTITUENT_OF_MUSCLE	-0.93	0.26	0	0	0	not significant
4144	PID_IL1_PATHWAY	-0.93	0.26	0	0	0	not significant
4145	REACTOME_CYTOCHROME_P450_ARRANGED_BY_SUBSTRATE_TYPE	-0.93	0.26	0	0	0	not significant
4146	SETLUR_PROSTATE_CANCER_TMPRSS2_ERG_FUSION_UP	-0.93	0.26	0	0	0	not significant
4147	CHR13Q12	-0.93	0.25	0	0	0	not significant
4148	CHR1P22	-0.93	0.25	0	0	0	not significant
4149	GO_DENDRITIC_SPINE_MAINTENANCE	-0.93	0.25	0	0	0	not significant
4150	GO_NEGATIVE_REGULATION_OF_TELOMERASE_ACTIVITY	-0.93	0.25	0	0	0	not significant
4151	GO_NUCLEOTIDE_SUGAR_BIOSYNTHETIC_PROCESS	-0.93	0.25	0	0	0	not significant
4152	GO_PEPTIDASE_ACTIVATOR_ACTIVITY	-0.93	0.25	0	0	0	not significant
4153	GO_POSITIVE_REGULATION_OF_GRANULOCYTE_CHEMOTAXIS	-0.93	0.25	0	0	0	not significant
4154	GO_POSITIVE_REGULATION_OF_HEXOKINASE_ACTIVITY	-0.93	0.25	0	0	0	not significant
4155	GO_PRESYNAPTIC_ACTIVE_ZONE	-0.93	0.25	0	0	0	not significant
4156	GO_REGULATION_OF_HISTONE_H4_K16_ACETYLATION	-0.93	0.25	0	0	0	not significant
4157	GO_REGULATION_OF_MITOCHONDRIAL_TRANSLATION	-0.93	0.25	0	0	0	not significant
4158	HADDAD_T_LYMPHOCYTE_AND_NK_PROGENITOR_DN	-0.93	0.25	0	0	0	not significant
4159	JAZAERI_BREAST_CANCER_BRCA1_VS_BRCA2_DN	-0.93	0.25	0	0	0	not significant
4160	LEE_LIVER_CANCER_DENA_UP	-0.93	0.25	0	0	0	not significant
4161	REACTOME_NUCLEAR_EVENTS_KINASE_AND_TRANSCRIPTION_FACTOR_ACTIVATIC	-0.93	0.25	0	0	0	not significant
4162	BARRIER_COLON_CANCER_RECURRENCE_UP	-0.93	0.24	0	0	0	not significant
4163	CHR2Q33	-0.93	0.24	0	0	0	not significant
4164	DAVIES_MULTIPLE_MYELOMA_VS_MGUS_UP	-0.93	0.24	0	0	0	not significant
4165	DORN_ADENOVIRUS_INFECTION_32HR_UP	-0.93	0.24	0	0	0	not significant
4166	GO_ADHERENS_JUNCTION_ASSEMBLY	-0.93	0.24	0	0	0	not significant
4167	GO_ANDROGEN_METABOLIC_PROCESS	-0.93	0.24	0	0	0	not significant
4168	GO_BETA_CATENIN_TCF_COMPLEX_ASSEMBLY	-0.93	0.24	0	0	0	not significant
4169	GO_CELLULAR_COMPONENT_MAINTENANCE	-0.93	0.24	0	0	0	not significant
4170	GO_CLATHRIN_DEPENDENT_ENDOCYTOSIS	-0.93	0.24	0	0	0	not significant
4171	GO_DNA_DEMETHYLATION	-0.93	0.24	0	0	0	not significant
4172	GO_NUCLEAR_EXOSOME_RNASE_COMPLEX_	-0.93	0.24	0	0	0	not significant
4173	GO_PRESYNAPTIC_MEMBRANE_ORGANIZATION	-0.93	0.24	0	0	0	not significant
4174	REACTOME_GABA_B_RECEPTOR_ACTIVATION	-0.93	0.24	0	0	0	not significant
4175	TURJANSKI_MAPK8_AND_MAPK9_TARGETS	-0.93	0.24	0	0	0	not significant
4176	HAHTOLA_SEZARY_SYNDROM_DN	-0.93	0.23	0	0	0	not significant
4177	REACTOME_INTRINSIC_PATHWAY_FOR_APOPTOSIS	-0.93	0.23	0	0	0	not significant
4178	REACTOME_LOSS_OF_FUNCTION_OF_TGFBR1_IN_CANCER	-0.93	0.23	0	0	0	not significant
4179	CHR10Q24	-0.93	0.22	0	0	0	not significant
4180	GNATENKO_PLATELET_SIGNATURE	-0.93	0.22	0	0	0	not significant
4181	GO_G_PROTEIN_COUPLED_SEROTONIN_RECEPTOR_BINDING	-0.93	0.22	0	0	0	not significant
4182	GO_NEGATIVE_REGULATION_OF_TRANSMEMBRANE_TRANSPORT	-0.93	0.22	0	0	0	not significant
4183	GO_POSITIVE_REGULATION_OF_CELL_CYCLE_PHASE_TRANSITION	-0.93	0.22	0	0	0	not significant
4184	GO_POSITIVE_REGULATION_OF_IRE1_MEDIATED_UNFOLDED_PROTEIN_RESPONSE	-0.93	0.22	0	0	0	not significant
4185	KEGG_MTOR_SIGNALING_PATHWAY	-0.93	0.22	0	0	0	not significant
4186	PECE_MAMMARY_STEM_CELL_DN	-0.93	0.22	0	0	0	not significant
4187	GO_NEGATIVE_REGULATION_OF_PROTEIN_DEPHOSPHORYLATION	-0.93	0.21	0	0	0	not significant
4188	GO_OXIDOREDUCTASE_ACTIVITY_ACTING_ON_NAD_P_H_QUINONE_OR_SIMILAR_C	-0.93	0.21	0	0	0	not significant
4189	GO_U1_SNRNP_BINDING	-0.93	0.21	0	0	0	not significant
4190	REACTOME_NONSENSE_MEDIATED_DECAY_NMD	-0.93	0.21	0	0	0	not significant
4191	GO_POSITIVE_REGULATION_OF_CYTOSKELETON_ORGANIZATION	-0.93	0.2	0	0	0	not significant
4192	KEGG_CHEMOKINE_SIGNALING_PATHWAY	-0.93	0.2	0	0	0	not significant
4193	REACTOME_NUCLEOTIDE_EXCISION_REPAIR	-0.93	0.19	0	0	0	not significant
4194	GO_CAMERA_TYPE_EYE_DEVELOPMENT	-0.93	0.18	0	0	0	not significant
4195	THEILGAARD_NEUTROPHIL_AT_SKIN_WOUND_DN	-0.93	0.18	0	0	0	not significant
4196	GO_NEGATIVE_REGULATION_OF_T_CELL_APOPTOTIC_PROCESS	-0.92	0.26	0	0	0	not significant
4197	GO_REGULATION_OF_NON_MOTILE_CILIUM_ASSEMBLY	-0.92	0.26	0	0	0	not significant
4198	GO_TRANSCRIPTION_FACTOR_TFIIH_HOLO_COMPLEX	-0.92	0.26	0	0	0	not significant
4199	REACTOME_ADP_SIGNALLING_THROUGH_P2Y_PURINOCEPTOR_1	-0.92	0.26	0	0	0	not significant
4200	WANG_TNF_TARGETS	-0.92	0.26	0	0	0	not significant
4201	BIOCARTA_MAL_PATHWAY	-0.92	0.25	0	0	0	not significant
4202	CHR18Q21	-0.92	0.25	0	0	0	not significant
4203	GO_ATTACHMENT_OF_MITOTIC_SPINDLE_MICROTUBULES_TO_KINETOCHORE	-0.92	0.25	0	0	0	not significant
4204	GO_CENTRAL_NERVOUS_SYSTEM_PROJECTION_NEURON_AXONOGENESIS	-0.92	0.25	0	0	0	not significant
4205	GO_PROTEIN_LOCALIZATION_TO_MICROTUBULE	-0.92	0.25	0	0	0	not significant
4206	MATZUK_SPERMATID_DIFFERENTIATION	-0.92	0.25	0	0	0	not significant
4207	REACTOME_IRAK2_MEDIATED_ACTIVATION_OF_TAK1_COMPLEX	-0.92	0.25	0	0	0	not significant
4208	CHR4Q27	-0.92	0.24	0	0	0	not significant
4209	GO_ATF6_MEDIATED_UNFOLDED_PROTEIN_RESPONSE	-0.92	0.24	0	0	0	not significant
4210	GO_CORNIFICATION	-0.92	0.24	0	0	0	not significant
4211	GO_ENDODERMAL_CELL_FATE_COMMITMENT	-0.92	0.24	0	0	0	not significant
4212	GO_HOMEOSTASIS_OF_NUMBER_OF_CELLS_WITHIN_A_TISSUE	-0.92	0.24	0	0	0	not significant
4213	GO_HYPOTONIC_RESPONSE	-0.92	0.24	0	0	0	not significant
4214	GO_PICLN_SM_PROTEIN_COMPLEX	-0.92	0.24	0	0	0	not significant
4215	GO_PRIMARY_MIRNA_PROCESSING	-0.92	0.24	0	0	0	not significant
4216	GO_SERTOLI_CELL_DEVELOPMENT	-0.92	0.24	0	0	0	not significant
4217	GO_SNAP_RECEPTOR_ACTIVITY	-0.92	0.24	0	0	0	not significant
4218	KUROKAWA_LIVER_CANCER_CHEMOTHERAPY_DN	-0.92	0.24	0	0	0	not significant
4219	LI_LUNG_CANCER	-0.92	0.24	0	0	0	not significant
4220	PID_TRAIL_PATHWAY	-0.92	0.24	0	0	0	not significant
4221	PID_VEGFR1_PATHWAY	-0.92	0.24	0	0	0	not significant
4222	REACTOME_SYNTHESIS_OF_PIPS_AT_THE_EARLY_ENDOSOME_MEMBRANE	-0.92	0.24	0	0	0	not significant
4223	SHIN_B_CELL_LYMPHOMA_CLUSTER_8	-0.92	0.24	0	0	0	not significant
4224	STONER_ESOPHAGEAL_CARCINOGENESIS_UP	-0.92	0.24	0	0	0	not significant
4225	SYED_ESTRADIOL_RESPONSE	-0.92	0.24	0	0	0	not significant
4226	TURASHVILI_BREAST_CARCINOMA_DUCTAL_VS_LOBULAR_UP	-0.92	0.24	0	0	0	not significant
4227	BIOCARTA_CELL2CELL_PATHWAY	-0.92	0.23	0	0	0	not significant
4228	GO_CELL_COMMUNICATION_BY_ELECTRICAL_COUPLING	-0.92	0.23	0	0	0	not significant
4229	GO_GENERAL_TRANSCRIPTION_INITIATION_FACTOR_ACTIVITY	-0.92	0.23	0	0	0	not significant
4230	GO_HISTONE_H3_K9_DIMETHYLATION	-0.92	0.23	0	0	0	not significant
4231	GO_NEGATIVE_REGULATION_OF_CELLULAR_PROTEIN_CATABOLIC_PROCESS	-0.92	0.23	0	0	0	not significant
4232	GO_NEGATIVE_REGULATION_OF_CYTOSOLIC_CALCIUM_ION_CONCENTRATION	-0.92	0.23	0	0	0	not significant
4233	GO_NEGATIVE_REGULATION_OF_PLATELET_DERIVED_GROWTH_FACTOR_RECEPT	-0.92	0.23	0	0	0	not significant
4234	GO_PML_BODY_ORGANIZATION	-0.92	0.23	0	0	0	not significant
4235	GO_POSITIVE_REGULATION_OF_VASCULAR_SMOOTH_MUSCLE_CELL_PROLIFERAT	-0.92	0.23	0	0	0	not significant
4236	GO_PROTEASOME_CORE_COMPLEX_ALPHA_SUBUNIT_COMPLEX	-0.92	0.23	0	0	0	not significant
4237	GO_REGULATION_OF_ACTIN_FILAMENT_BASED_MOVEMENT	-0.92	0.23	0	0	0	not significant
4238	GO_REGULATION_OF_TRANSCRIPTION_FROM_RNA_POLYMERASE_II_PROMOTER_	-0.92	0.23	0	0	0	not significant
4239	GO_TRANSCRIPTION_DEPENDENT_TETHERING_OF_RNA_POLYMERASE_II_GENE_D	-0.92	0.23	0	0	0	not significant

4240	KYNG_ENVIRONMENTAL_STRESS_RESPONSE_NOT_BY_4NQO_IN_WS	-0.92	0.23	0	0	0	not significant
4241	MATZUK_SPERMATOCYTE	-0.92	0.23	0	0	0	not significant
4242	REACTOME_KERATINIZATION	-0.92	0.23	0	0	0	not significant
4243	WANG_ESOPHAGUS_CANCER_PROGRESSION_UP	-0.92	0.23	0	0	0	not significant
4244	WOTTON_RUNX_TARGETS_DN	-0.92	0.23	0	0	0	not significant
4245	YANG_BREAST_CANCER_ESR1_BULK_DN	-0.92	0.23	0	0	0	not significant
4246	AKL_HTLV1_INFECTION_UP	-0.92	0.22	0	0	0	not significant
4247	GO_DNA_POLYMERASE_ACTIVITY	-0.92	0.22	0	0	0	not significant
4248	GO_SMALL_PROTEIN_ACTIVATING_ENZYME_BINDING	-0.92	0.22	0	0	0	not significant
4249	KEGG_LONG_TERM_DEPRESSION	-0.92	0.22	0	0	0	not significant
4250	LUI_THYROID_CANCER_CLUSTER_5	-0.92	0.22	0	0	0	not significant
4251	REACTOME_PERK_REGULATES_GENE_EXPRESSION	-0.92	0.22	0	0	0	not significant
4252	REACTOME_TRANSCRIPTION_COUPLED_NUCLEOTIDE_EXCISION_REPAIR_TC_NER	-0.92	0.2	0	0	0	not significant
4253	GO_NUCLEAR_TRANSCRIBED_MRNA_CATABOLIC_PROCESS_DEADENYLATION_DEI	-0.92	0.19	0	0	0	not significant
4254	GO_NUCLEAR_TRANSCRIBED_MRNA_CATABOLIC_PROCESS_NONSENSE_MEDIATEI	-0.92	0.19	0	0	0	not significant
4255	REACTOME_HATS_ACETYLATE_HISTONES	-0.92	0.19	0	0	0	not significant
4256	TAYLOR_METHYLATED_IN_ACUTE_LYMPHOBLASTIC_LEUKEMIA	-0.92	0.19	0	0	0	not significant
4257	GO_CYTOSOLIC_TRANSPORT	-0.92	0.18	0	0	0	not significant
4258	REACTOME_PRE_NOTCH_EXPRESSION_AND_PROCESSING	-0.92	0.18	0	0	0	not significant
4259	KEGG_REGULATION_OF_ACTIN_CYTOSKELETON	-0.92	0.17	0	0	0	not significant
4260	GO_STRUCTURAL_CONSTITUENT_OF_RIBOSOME	-0.92	0.15	0	0	0	not significant
4261	GO_GLUTAMATERGIC_SYNAPSE	-0.92	0.13	0	0	0	not significant
4262	GO_MEMBRANE_DOCKING	-0.92	0.11	0	0	0	not significant
4263	BRUINS_UVC_RESPONSE_LATE	-0.92	0.04	0	0	0	not significant
4264	GO_POSITIVE_REGULATION_OF_PROTEIN_SUMOYLATION	-0.91	0.25	0	0	0	not significant
4265	GO_RESPIRATORY_GASEOUS_EXCHANGE	-0.91	0.25	0	0	0	not significant
4266	REACTOME_ERYTHROPOIETIN_ACTIVATES_RAS	-0.91	0.25	0	0	0	not significant
4267	REACTOME_FORMATION_OF_THE_CORNIFIED_ENVELOPE	-0.91	0.25	0	0	0	not significant
4268	GO_ACTIVATION_OF_NF_KAPPAB_INDUCING_KINASE_ACTIVITY	-0.91	0.24	0	0	0	not significant
4269	GO_AMYLOID_FIBRIL_FORMATION	-0.91	0.24	0	0	0	not significant
4270	GO_INTERLEUKIN_12_BIOSYNTHETIC_PROCESS	-0.91	0.24	0	0	0	not significant
4271	GO_NEDD8_SPECIFIC_PROTEASE_ACTIVITY	-0.91	0.24	0	0	0	not significant
4272	GO_POSITIVE_REGULATION_OF_NEUTROPHIL_MIGRATION	-0.91	0.24	0	0	0	not significant
4273	GO_PRESYNAPTIC_MEMBRANE_ASSEMBLY	-0.91	0.24	0	0	0	not significant
4274	PID_MYC_PATHWAY	-0.91	0.24	0	0	0	not significant
4275	URS_ADIPOCYTE_DIFFERENTIATION_DN	-0.91	0.24	0	0	0	not significant
4276	GO_JUN_KINASE_KINASE_KINASE_ACTIVITY	-0.91	0.23	0	0	0	not significant
4277	GO_MYOBLAST_DIFFERENTIATION	-0.91	0.23	0	0	0	not significant
4278	GO_REGULATION_OF_ENDOSOME_SIZE	-0.91	0.23	0	0	0	not significant
4279	GO_REGULATION_OF_PANCREATIC_JUICE_SECRETION	-0.91	0.23	0	0	0	not significant
4280	GO_V_D_J_RECOMBINATION	-0.91	0.23	0	0	0	not significant
4281	IKEDA_MIR1_TARGETS_UP	-0.91	0.23	0	0	0	not significant
4282	NIELSEN_LEIOMYOSARCOMA_UP	-0.91	0.23	0	0	0	not significant
4283	REACTOME_EGFR_DOWNREGULATION	-0.91	0.23	0	0	0	not significant
4284	REACTOME_GLUCONEOGENESIS	-0.91	0.23	0	0	0	not significant
4285	WEST_ADRENOCORTICAL_CARCINOMA_VS_ADENOMA_DN	-0.91	0.23	0	0	0	not significant
4286	GAUTSCHI_SRC_SIGNALING	-0.91	0.22	0	0	0	not significant
4287	GO_FORMATION_OF_CYTOPLASMIC_TRANSLATION_INITIATION_COMPLEX	-0.91	0.22	0	0	0	not significant
4288	GO_INTERLEUKIN_23_PRODUCTION	-0.91	0.22	0	0	0	not significant
4289	GO_LACTATION	-0.91	0.22	0	0	0	not significant
4290	GO_MACROPHAGE_DIFFERENTIATION	-0.91	0.22	0	0	0	not significant
4291	GO_PHOSPHATIDYLINOSITOL_3_KINASE_COMPLEX_CLASS_III	-0.91	0.22	0	0	0	not significant
4292	GO_POSITIVE_REGULATION_OF_RECEPTOR_SIGNALING_PATHWAY_VIA_STAT	-0.91	0.22	0	0	0	not significant
4293	GO_PYRIMIDINE_NUCLEOSIDE_TRIPHOSPHATE_BIOSYNTHETIC_PROCESS	-0.91	0.22	0	0	0	not significant
4294	GO_RRNA_ADENINE_METHYLTRANSFERASE_ACTIVITY	-0.91	0.22	0	0	0	not significant
4295	PID_EPHRINB_REV_PATHWAY	-0.91	0.22	0	0	0	not significant
4296	REACTOME_METALLOTHIONEINS_BIND_METALS	-0.91	0.22	0	0	0	not significant
4297	ROVERSI_GLIOMA_COPY_NUMBER_DN	-0.91	0.22	0	0	0	not significant
4298	WENDT_COHESIN_TARGETS_UP	-0.91	0.22	0	0	0	not significant
4299	GO_AP_TYPE_MEMBRANE_COAT_ADAPTOR_COMPLEX	-0.91	0.21	0	0	0	not significant
4300	TIEN_INTESTINE_PROBIOTICS_6HR_UP	-0.91	0.21	0	0	0	not significant
4301	CHR2Q37	-0.91	0.2	0	0	0	not significant
4302	FALVELLA_SMOKERS_WITH_LUNG_CANCER	-0.91	0.2	0	0	0	not significant
4303	GO_POSITIVE_REGULATION_OF_GLUCOKINASE_ACTIVITY	-0.91	0.2	0	0	0	not significant
4304	GO_RUFFLE_ORGANIZATION	-0.91	0.2	0	0	0	not significant
4305	BOYLAN_MULTIPLE_MYELOMA_D_UP	-0.91	0.19	0	0	0	not significant
4306	REACTOME_TRANS_GOLGI_NETWORK_VESICLE_BUDDING	-0.91	0.19	0	0	0	not significant
4307	CHR1Q32	-0.91	0.18	0	0	0	not significant
4308	GO_RESPONSE_TO_VITAMIN	-0.91	0.18	0	0	0	not significant
4309	BROWNE_HCMV_INFECTION_12HR_DN	-0.91	0.17	0	0	0	not significant
4310	GO_DNA_DEPENDENT_ATPASE_ACTIVITY	-0.91	0.17	0	0	0	not significant
4311	KIM_WT1_TARGETS_8HR_DN	-0.91	0.17	0	0	0	not significant
4312	LIN_NPAS4_TARGETS_UP	-0.91	0.17	0	0	0	not significant
4313	REACTOME_SUMOYLATION_OF_DNA_DAMAGE_RESPONSE_AND_REPAIR_PROTEIN	-0.91	0.17	0	0	0	not significant
4314	GO_CELLULAR_TRANSITION_METAL_ION_HOMEOSTASIS	-0.91	0.16	0	0	0	not significant
4315	GO_NUCLEAR_TRANSCRIBED_MRNA_CATABOLIC_PROCESS	-0.91	0.12	0	0	0	not significant
4316	RATTENBACHER_BOUND_BY_CELF1	-0.91	0.1	0	0	0	not significant
4317	GO_ACTIN_CYTOSKELETON	-0.91	0.09	0	0	0	not significant
4318	BOYAULT_LIVER_CANCER_SUBCLASS_G56_DN	-0.90	0.24	0	0	0	not significant
4319	GO_MAINTENANCE_OF_SISTER_CHROMATID_COHESION	-0.90	0.24	0	0	0	not significant
4320	GO_GUANYL_NUCLEOTIDE_EXCHANGE_FACTOR_COMPLEX	-0.90	0.23	0	0	0	not significant
4321	GO_NEGATIVE_REGULATION_OF_CELL_GROWTH_INVOLVED_IN_CARDIAC_MUSCLE	-0.90	0.23	0	0	0	not significant
4322	GO_POSITIVE_REGULATION_OF_SODIUM_ION_TRANSPORT	-0.90	0.23	0	0	0	not significant
4323	HEDENFALK_BREAST_CANCER_BRACX_DN	-0.90	0.23	0	0	0	not significant
4324	PID_INSULIN_GLUCOSE_PATHWAY	-0.90	0.23	0	0	0	not significant
4325	REACTOME_CELL_EXTRACELLULAR_MATRIX_INTERACTIONS	-0.90	0.23	0	0	0	not significant
4326	REACTOME_SIGNALING_BY_TGF_BETA_RECEPTOR_COMPLEX_IN_CANCER	-0.90	0.23	0	0	0	not significant
4327	ZHAN_LATE_DIFFERENTIATION_GENES_DN	-0.90	0.23	0	0	0	not significant
4328	GO_EMBRYONIC_PLACENTA_MORPHOGENESIS	-0.90	0.22	0	0	0	not significant
4329	GO_METANEPHRIC_NEPHRON_EPITHELIUM_DEVELOPMENT	-0.90	0.22	0	0	0	not significant
4330	GO_MITOCHONDRION_LOCALIZATION	-0.90	0.22	0	0	0	not significant
4331	GO_NEGATIVE_REGULATION_OF_PRODUCTION_OF_MIRNAS_INVOLVED_IN_GENE_	-0.90	0.22	0	0	0	not significant
4332	GO_NEGATIVE_REGULATION_OF_PROTEIN_KINASE_B_SIGNALING	-0.90	0.22	0	0	0	not significant
4333	GO_NEGATIVE_REGULATION_OF_STRIATED_MUSCLE_CELL_APOPTOTIC_PROCES	-0.90	0.22	0	0	0	not significant
4334	GO_POSITIVE_REGULATION_OF_EPITHELIAL_CELL_DIFFERENTIATION	-0.90	0.22	0	0	0	not significant
4335	GO_RDNA_BINDING	-0.90	0.22	0	0	0	not significant
4336	GO_REGULATION_OF_MRNA_BINDING	-0.90	0.22	0	0	0	not significant
4337	GO_REGULATION_OF_THE_FORCE_OF_HEART_CONTRACTION	-0.90	0.22	0	0	0	not significant
4338	GO_SUPERCOILED_DNA_BINDING	-0.90	0.22	0	0	0	not significant
4339	KEGG_TASTE_TRANSDUCTION	-0.90	0.22	0	0	0	not significant
4340	NIKOLSKY_BREAST_CANCER_12Q13_Q21_AMPLICON	-0.90	0.22	0	0	0	not significant
4341	NIKOLSKY_BREAST_CANCER_15Q26_AMPLICON	-0.90	0.22	0	0	0	not significant
4342	POMEROY_MEDULLOBLASTOMA_PROGNOSIS_DN	-0.90	0.22	0	0	0	not significant
4343	CHR16Q23	-0.90	0.21	0	0	0	not significant
4344	GENTLES_LEUKEMIC_STEM_CELL_DN	-0.90	0.21	0	0	0	not significant
4345	GO_NEGATIVE_REGULATION_OF_HORMONE_SECRETION	-0.90	0.21	0	0	0	not significant
4346	GO_POSITIVE_REGULATION_OF_MITOTIC_SISTER_CHROMATID_SEPARATION	-0.90	0.21	0	0	0	not significant
4347	GO_POSITIVE_REGULATION_OF_RIG_I_SIGNALING_PATHWAY	-0.90	0.21	0	0	0	not significant
4348	GO_PROTEIN_K48_LINKED_UBIQUITINATION	-0.90	0.21	0	0	0	not significant

4349	HOFMANN_MYELODYSPLASTIC_SYNDROM_HIGH_RISK_UP	-0.90	0.21	0	0	0	not significant
4350	HOLLERN_SQUAMOUS_BREAST_TUMOR	-0.90	0.21	0	0	0	not significant
4351	KONDO_PROSTATE_CANCER_WITH_H3K27ME3	-0.90	0.21	0	0	0	not significant
4352	MYLLYKANGAS_AMPLIFICATION_HOT_SPOT_2	-0.90	0.21	0	0	0	not significant
4353	REACTOME_INTERLEUKIN_12_FAMILY_SIGNALING	-0.90	0.21	0	0	0	not significant
4354	ZHANG_ANTIVIRAL_RESPONSE_TO_RIBAVIRIN_DN	-0.90	0.21	0	0	0	not significant
4355	BIOCARTA_FMLP_PATHWAY	-0.90	0.2	0	0	0	not significant
4356	GO_ASYMMETRIC_STEM_CELL_DIVISION	-0.90	0.2	0	0	0	not significant
4357	GO_PANTOTHENATE_METABOLIC_PROCESS	-0.90	0.2	0	0	0	not significant
4358	GO_POSITIVE_REGULATION_OF_DENDRITIC_SPINE_DEVELOPMENT	-0.90	0.2	0	0	0	not significant
4359	GO_TELOMERE_CAPPING	-0.90	0.2	0	0	0	not significant
4360	KANNAN_TP53_TARGETS_UP	-0.90	0.2	0	0	0	not significant
4361	KEGG_ACUTE_MYELOID_LEUKEMIA	-0.90	0.2	0	0	0	not significant
4362	OUILLETTE_CLL_13Q14_DELETION_DN	-0.90	0.2	0	0	0	not significant
4363	CHR12Q23	-0.90	0.19	0	0	0	not significant
4364	CHR17Q12	-0.90	0.19	0	0	0	not significant
4365	GO_CLATHRIN_COATED_PIT	-0.90	0.19	0	0	0	not significant
4366	GO_REGULATION_OF_SYNAPTIC_VESICLE_TRANSPORT	-0.90	0.19	0	0	0	not significant
4367	KEGG_ADHERENS_JUNCTION	-0.90	0.19	0	0	0	not significant
4368	REACTOME_BBSOME_MEDIATED_CARGO_TARGETING_TO_CILIUM	-0.90	0.19	0	0	0	not significant
4369	CHR7Q11	-0.90	0.18	0	0	0	not significant
4370	GO_NEGATIVE_REGULATION_OF_PROTEIN_POLYMERIZATION	-0.90	0.18	0	0	0	not significant
4371	GO_PROTEIN_INSERTION_INTO_MITOCHONDRIAL_MEMBRANE	-0.90	0.18	0	0	0	not significant
4372	GO_HINDBRAIN_DEVELOPMENT	-0.90	0.17	0	0	0	not significant
4373	GO_G_PROTEIN_COUPLED_RECEPTOR_BINDING	-0.90	0.16	0	0	0	not significant
4374	GO_PROTEIN_METHYLTRANSFERASE_ACTIVITY	-0.90	0.16	0	0	0	not significant
4375	PDGF_ERK_DN.V1_UP	-0.90	0.16	0	0	0	not significant
4376	LINDGREN_BLADDER_CANCER_CLUSTER_1_UP	-0.90	0.15	0	0	0	not significant
4377	KEGG_ALZHEIMERS_DISEASE	-0.90	0.13	0	0	0	not significant
4378	GO_VESICLE_LOCALIZATION	-0.90	0.11	0	0	0	not significant
4379	GO_CELL_SUBSTRATE_JUNCTION	-0.90	0.06	0	0	0	not significant
4380	GO_REGULATION_OF_MULTI_ORGANISM_PROCESS	-0.90	0.06	0	0	0	not significant
4381	GO_COPPER_ION_TRANSPORT	-0.89	0.23	0	0	0	not significant
4382	GO_ESTABLISHMENT_OF_SISTER_CHROMATID_COHESION	-0.89	0.23	0	0	0	not significant
4383	GO_OVULATION_CYCLE_PROCESS	-0.89	0.23	0	0	0	not significant
4384	DORMOY_ELAVL1_TARGETS	-0.89	0.22	0	0	0	not significant
4385	GO_EMBRYONIC_CAMERA_TYPE_EYE_FORMATION	-0.89	0.22	0	0	0	not significant
4386	GO_POSITIVE_REGULATION_OF_TELOMERE_CAPPING	-0.89	0.22	0	0	0	not significant
4387	GO_RECEPTOR_BIOSYNTHETIC_PROCESS	-0.89	0.22	0	0	0	not significant
4388	GO_VOLUNTARY_MUSCULOSKELETAL_MOVEMENT	-0.89	0.22	0	0	0	not significant
4389	REACTOME_CLEC7A_DECTIN_1_INDUCES_NFAT_ACTIVATION	-0.89	0.22	0	0	0	not significant
4390	WILLIAMS_ESR2_TARGETS_UP	-0.89	0.22	0	0	0	not significant
4391	GO_CELLULAR_RESPONSE_TO_ELECTRICAL_STIMULUS	-0.89	0.21	0	0	0	not significant
4392	GO_CENTRIOLAR_SATELLITE	-0.89	0.21	0	0	0	not significant
4393	GO_CHEMOKINE_C_C_MOTIF_LIGAND_5_PRODUCTION	-0.89	0.21	0	0	0	not significant
4394	GO_CILIARY_ROOTLET	-0.89	0.21	0	0	0	not significant
4395	GO_ER_MEMBRANE_INSERTION_COMPLEX	-0.89	0.21	0	0	0	not significant
4396	GO_MITOGEN_ACTIVATED_PROTEIN_KINASE_KINASE_KINASE_BINDING	-0.89	0.21	0	0	0	not significant
4397	GO_N_TERMINAL_PROTEIN_AMINO_ACID_ACETYLATION	-0.89	0.21	0	0	0	not significant
4398	GO_POSITIVE_REGULATION_OF_ACTIN_CYTOSKELETON_REORGANIZATION	-0.89	0.21	0	0	0	not significant
4399	GO_POSITIVE_REGULATION_OF_SUPEROXIDE_ANION_GENERATION	-0.89	0.21	0	0	0	not significant
4400	GO_PREFOLDIN_COMPLEX	-0.89	0.21	0	0	0	not significant
4401	GO_REGULATION_OF_CYTOPLASMIC_TRANSLATION	-0.89	0.21	0	0	0	not significant
4402	GO_REGULATION_OF_RECEPTOR_BIOSYNTHETIC_PROCESS	-0.89	0.21	0	0	0	not significant
4403	HOFMANN_MYELODYSPLASTIC_SYNDROM_RISK_DN	-0.89	0.21	0	0	0	not significant
4404	KORKOLA_EMBRYONIC_CARCINOMA_VS_SEMINOMA_UP	-0.89	0.21	0	0	0	not significant
4405	NIELSEN_SCHWANNOMA_UP	-0.89	0.21	0	0	0	not significant
4406	PID_ARF6_DOWNSTREAM_PATHWAY	-0.89	0.21	0	0	0	not significant
4407	PID_SYNDECAN_2_PATHWAY	-0.89	0.21	0	0	0	not significant
4408	REACTOME_PROCESSING_AND_ACTIVATION_OF_SUMO	-0.89	0.21	0	0	0	not significant
4409	GO_NUCLEOSIDE_MONOPHOSPHATE_METABOLIC_PROCESS	-0.89	0.2	0	0	0	not significant
4410	GO_NUCLEOSIDE_MONOPHOSPHATE_PHOSPHORYLATION	-0.89	0.2	0	0	0	not significant
4411	GO_PIGMENT_CELL_DIFFERENTIATION	-0.89	0.2	0	0	0	not significant
4412	GO_REGULATION_OF_AMINO_ACID_IMPORT_ACROSS_PLASMA_MEMBRANE	-0.89	0.2	0	0	0	not significant
4413	GO_REGULATION_OF_CD8_POSITIVE_ALPHA_BETA_T_CELL_DIFFERENTIATION	-0.89	0.2	0	0	0	not significant
4414	REACTOME_ROLE_OF_LAT2_NTAL_LAB_ON_CALCIUM_MOBILIZATION	-0.89	0.2	0	0	0	not significant
4415	TAVAZOIE_METASTASIS	-0.89	0.2	0	0	0	not significant
4416	TURASHVILI_BREAST_NORMAL_DUCTAL_VS_LOBULAR_UP	-0.89	0.2	0	0	0	not significant
4417	YOSHIOKA_LIVER_CANCER_EARLY_RECURRENCE_UP	-0.89	0.2	0	0	0	not significant
4418	GO_CELLULAR_RESPONSE_TO_OSMOTIC_STRESS	-0.89	0.19	0	0	0	not significant
4419	GO_INTRACELLULAR_ESTROGEN_RECEPTOR_SIGNALING_PATHWAY	-0.89	0.19	0	0	0	not significant
4420	GO_NEGATIVE_REGULATION_OF_MACROAUTOPHAGY	-0.89	0.19	0	0	0	not significant
4421	GO_PHOTORECEPTOR_INNER_SEGMENT	-0.89	0.19	0	0	0	not significant
4422	MARTINEZ_RESPONSE_TO_TRABECTEDIN	-0.89	0.19	0	0	0	not significant
4423	REACTOME_REGULATION_OF_TP53_ACTIVITY_THROUGH_ACETYLATION	-0.89	0.19	0	0	0	not significant
4424	TAVOR_CEBPA_TARGETS_UP	-0.89	0.19	0	0	0	not significant
4425	GO_INOSITOL_PHOSPHATE_METABOLIC_PROCESS	-0.89	0.18	0	0	0	not significant
4426	GO_POSITIVE_REGULATION_OF_CELL_CYCLE_G2_M_PHASE_TRANSITION	-0.89	0.18	0	0	0	not significant
4427	KUMAR_AUTOPHAGY_NETWORK	-0.89	0.18	0	0	0	not significant
4428	PELLICCIOTTA_HDAC_IN_ANTIGEN_PRESENTATION_DN	-0.89	0.18	0	0	0	not significant
4429	WALLACE_PROSTATE_CANCER_RACE_DN	-0.89	0.18	0	0	0	not significant
4430	CHR20P13	-0.89	0.17	0	0	0	not significant
4431	GO_CARDIAC_MUSCLE_CONTRACTION	-0.89	0.17	0	0	0	not significant
4432	GO_CILIARY_PLASM	-0.89	0.17	0	0	0	not significant
4433	GO_POSITIVE_REGULATION_OF_ORGANELLE_ASSEMBLY	-0.89	0.17	0	0	0	not significant
4434	GO_REGULATION_OF_LIPID_KINASE_ACTIVITY	-0.89	0.17	0	0	0	not significant
4435	GO_CARDIAC_VENTRICLE_DEVELOPMENT	-0.89	0.16	0	0	0	not significant
4436	GO_RELEASE_OF_CYTOCHROME_C_FROM_MITOCHONDRIA	-0.89	0.15	0	0	0	not significant
4437	GO_RESPIRATORY_CHAIN_COMPLEX	-0.89	0.15	0	0	0	not significant
4438	GO_TUBE_CLOSURE	-0.89	0.15	0	0	0	not significant
4439	KEGG_FC_GAMMA_R_MEDIATED_PHAGOCYTOSIS	-0.89	0.15	0	0	0	not significant
4440	REACTOME_CARGO_RECOGNITION_FOR_CLATHRIN_MEDIATED_ENDOCYTOSIS	-0.89	0.15	0	0	0	not significant
4441	GO_POST_GOLGI_VESICLE_MEDIATED_TRANSPORT	-0.89	0.13	0	0	0	not significant
4442	GO_PEPTIDYL_LYSINE_ACETYLATION	-0.89	0.09	0	0	0	not significant
4443	MYLLYKANGAS_AMPLIFICATION_HOT_SPOT_22	-0.88	0.22	0	0	0	not significant
4444	GO_ESCRT_I_COMPLEX	-0.88	0.21	0	0	0	not significant
4445	GO_ESTABLISHMENT_OR_MAINTENANCE_OF_MONOPOLAR_CELL_POLARITY	-0.88	0.21	0	0	0	not significant
4446	GO_FILAMENTOUS_ACTIN	-0.88	0.21	0	0	0	not significant
4447	GO_INTERLEUKIN_2_SECRETION	-0.88	0.21	0	0	0	not significant
4448	GO_NUCLEAR_PERICENTRIC_HETEROCHROMATIN	-0.88	0.21	0	0	0	not significant
4449	GO_POSITIVE_REGULATION_OF_AMINE_TRANSPORT	-0.88	0.21	0	0	0	not significant
4450	GO_RESPONSE_TO_LEAD_ION	-0.88	0.21	0	0	0	not significant
4451	GO_ZINC_ION_IMPORT_ACROSS_PLASMA_MEMBRANE	-0.88	0.21	0	0	0	not significant
4452	REACTOME_ENDOGENOUS_STEROLS	-0.88	0.21	0	0	0	not significant
4453	CHESLER_BRAIN_QTL_TRANS	-0.88	0.2	0	0	0	not significant
4454	CHR4Q24	-0.88	0.2	0	0	0	not significant
4455	GO_INSULIN_RECEPTOR_BINDING	-0.88	0.2	0	0	0	not significant
4456	GO_MITOGEN_ACTIVATED_PROTEIN_KINASE_KINASE_BINDING	-0.88	0.2	0	0	0	not significant
4457	GO_NUCLEAR_TRANSCRIBED_MRNA_CATABOLIC_PROCESS_DEADENYLATION_INDI	-0.88	0.2	0	0	0	not significant

4458	GO_PHOSPHATIDYLINOSITOL_PHOSPHORYLATION	-0.88	0.2	0	0	0	not significant
4459	GO_REGULATION_OF_SYNAPTIC_VESICLE_CLUSTERING	-0.88	0.2	0	0	0	not significant
4460	GO_SINGLE_STRANDED_DNA_DEPENDENT_ATPASE_ACTIVITY	-0.88	0.2	0	0	0	not significant
4461	GO_TELOMERE_CAP_COMPLEX	-0.88	0.2	0	0	0	not significant
4462	GO_TRANSCRIPTION_EXPORT_COMPLEX	-0.88	0.2	0	0	0	not significant
4463	GO_VESICLE_CARGO_LOADING	-0.88	0.2	0	0	0	not significant
4464	MUELLER_COMMON_TARGETS_OF_AML_FUSIONS_UP	-0.88	0.2	0	0	0	not significant
4465	REACTOME_INLB_MEDIATED_ENTRY_OF_LISTERIA_MONOCYTOGENES_INTO_HOST	-0.88	0.2	0	0	0	not significant
4466	VANDESLUIS_COMMD1_TARGETS_GROUP_4_UP	-0.88	0.2	0	0	0	not significant
4467	GO_ENDOPLASMIC_RETICULUM_TUBULAR_NETWORK	-0.88	0.19	0	0	0	not significant
4468	GO_NEURON_PROJECTION_MEMBRANE	-0.88	0.19	0	0	0	not significant
4469	GO_REGULATION_OF_CARTILAGE_DEVELOPMENT	-0.88	0.19	0	0	0	not significant
4470	GO_REGULATION_OF_INTEGRIN_MEDIATED_SIGNALING_PATHWAY	-0.88	0.19	0	0	0	not significant
4471	GO_RRNA_METHYLATION	-0.88	0.19	0	0	0	not significant
4472	GO_WATER_HOMEOSTASIS	-0.88	0.19	0	0	0	not significant
4473	SCHLESINGER_METHYLATED_DE_NOVO_IN_CANCER	-0.88	0.19	0	0	0	not significant
4474	VANTVEER_BREAST_CANCER_BRCA1_DN	-0.88	0.19	0	0	0	not significant
4475	GO_CHROMATIN_SILENCING_AT_RDNA	-0.88	0.18	0	0	0	not significant
4476	GO_INSULIN_RESPONSIVE_COMPARTMENT	-0.88	0.18	0	0	0	not significant
4477	GO_NON_MEMBRANE_SPANNING_PROTEIN_TYROSINE_KINASE_ACTIVITY	-0.88	0.18	0	0	0	not significant
4478	GO_NUCLEAR_TRANSCRIBED_MRNA_CATABOLIC_PROCESS_EXONUCLEOLYTIC	-0.88	0.18	0	0	0	not significant
4479	GO_SCHWANN_CELL_DIFFERENTIATION	-0.88	0.18	0	0	0	not significant
4480	XU_GH1_EXOGENOUS_TARGETS_DN	-0.88	0.18	0	0	0	not significant
4481	CHR2P11	-0.88	0.17	0	0	0	not significant
4482	CHR6Q21	-0.88	0.17	0	0	0	not significant
4483	GO_ACTIVATION_OF_PROTEIN_KINASE_B_ACTIVITY	-0.88	0.17	0	0	0	not significant
4484	GO_PROTEIN_INSERTION_INTO_MEMBRANE	-0.88	0.17	0	0	0	not significant
4485	GO_SUPEROXIDE_METABOLIC_PROCESS	-0.88	0.17	0	0	0	not significant
4486	REACTOME_PHASE_I_FUNCTIONALIZATION_OF_COMPOUNDS	-0.88	0.17	0	0	0	not significant
4487	STAMBOLSKY_RESPONSE_TO_VITAMIN_D3_UP	-0.88	0.17	0	0	0	not significant
4488	GO_DNA_REPAIR_COMPLEX	-0.88	0.16	0	0	0	not significant
4489	GO_NADH_DEHYDROGENASE_ACTIVITY	-0.88	0.16	0	0	0	not significant
4490	GO_FIBROBLAST_PROLIFERATION	-0.88	0.15	0	0	0	not significant
4491	GO_NON_MOTILE_CILIUM	-0.88	0.15	0	0	0	not significant
4492	GO_APPENDAGE_MORPHOGENESIS	-0.88	0.13	0	0	0	not significant
4493	RAO_BOUND_BY_SALL4_ISOFORM_A	-0.88	0.12	0	0	0	not significant
4494	WEI_MIR34A_TARGETS	-0.88	0.12	0	0	0	not significant
4495	LTE2_UP.V1_DN	-0.88	0.1	0	0	0	not significant
4496	GO_POSITIVE_REGULATION_OF_SUPRAMOLECULAR_FIBER_ORGANIZATION	-0.88	0.09	0	0	0	not significant
4497	GO_DENDRITE_DEVELOPMENT	-0.88	0.07	0	0	0	not significant
4498	GO_CELL_SUBSTRATE_ADHESION	-0.88	0.05	0	0	0	not significant
4499	RICKMAN_METASTASIS_UP	-0.88	0.04	0	0	0	not significant
4500	REACTOME_HYALURONAN_METABOLISM	-0.87	0.22	0	0	0	not significant
4501	GO_POSITIVE_REGULATION_OF_GLUCONEOGENESIS	-0.87	0.21	0	0	0	not significant
4502	GO_POSITIVE_REGULATION_OF_NON_MOTILE_CILIUM_ASSEMBLY	-0.87	0.21	0	0	0	not significant
4503	BIOCARTA_LEPTIN_PATHWAY	-0.87	0.2	0	0	0	not significant
4504	BIOCARTA_TALL1_PATHWAY	-0.87	0.2	0	0	0	not significant
4505	GO_CELL_PROLIFERATION_IN_FOREBRAIN	-0.87	0.2	0	0	0	not significant
4506	GO_CENTROMERIC_DNA_BINDING	-0.87	0.2	0	0	0	not significant
4507	GO_NAD_BIOSYNTHESIS_VIA_NICOTINAMIDE_RIBOSIDE_SALVAGE_PATHWAY	-0.87	0.2	0	0	0	not significant
4508	GO_POSITIVE_REGULATION_OF_CYTOPLASMIC_TRANSLATION	-0.87	0.2	0	0	0	not significant
4509	GO_POSITIVE_REGULATION_OF_MUSCLE_CONTRACTION	-0.87	0.2	0	0	0	not significant
4510	GO_POSITIVE_REGULATION_OF_RECEPTOR_INTERNALIZATION	-0.87	0.2	0	0	0	not significant
4511	GO_RESOLUTION_OF_MEIOTIC_RECOMBINATION_INTERMEDIATES	-0.87	0.2	0	0	0	not significant
4512	GO_VIRAL_LATENCY	-0.87	0.2	0	0	0	not significant
4513	REACTOME_SYNTHESIS_OF_GDP_MANNOSE	-0.87	0.2	0	0	0	not significant
4514	AMUNDSON_GAMMA_RADIATION_RESISTANCE	-0.87	0.19	0	0	0	not significant
4515	GO_ASPARTATE_FAMILY_AMINO_ACID_CATABOLIC_PROCESS	-0.87	0.19	0	0	0	not significant
4516	GO_CEREBELLAR_PURKINJE_CELL_LAYER_MORPHOGENESIS	-0.87	0.19	0	0	0	not significant
4517	GO_HIGH_DENSITY_LIPOPROTEIN_PARTICLE_CLEARANCE	-0.87	0.19	0	0	0	not significant
4518	GO_NUCLEAR_INCLUSION_BODY	-0.87	0.19	0	0	0	not significant
4519	GO_PHOSPHATIDYLINOSITOL_TRANSPORTER_ACTIVITY	-0.87	0.19	0	0	0	not significant
4520	GO_POSITIVE_REGULATION_OF_PHOSPHOLIPID_TRANSPORT	-0.87	0.19	0	0	0	not significant
4521	GO_TUBULAR_ENDOSOME	-0.87	0.19	0	0	0	not significant
4522	NEBEN_AML_WITH_FLT3_OR_NRAS_UP	-0.87	0.19	0	0	0	not significant
4523	REACTOME_CREB_PHOSPHORYLATION	-0.87	0.19	0	0	0	not significant
4524	REACTOME_P75NTR_RECRUITS_SIGNALLING_COMPLEXES	-0.87	0.19	0	0	0	not significant
4525	REACTOME_REGULATION_OF_TP53_ACTIVITY_THROUGH_METHYLATION	-0.87	0.19	0	0	0	not significant
4526	REACTOME_RUNX3_REGULATES_NOTCH_SIGNALING	-0.87	0.19	0	0	0	not significant
4527	BIOCARTA_RAC1_PATHWAY	-0.87	0.18	0	0	0	not significant
4528	CHEN_ETV5_TARGETS_SERTOLI	-0.87	0.18	0	0	0	not significant
4529	GO_CALCIUM_CHANNEL_COMPLEX	-0.87	0.18	0	0	0	not significant
4530	GO_COLUMNAR_CUBOIDAL_EPITHELIAL_CELL_DEVELOPMENT	-0.87	0.18	0	0	0	not significant
4531	GO_DENTATE_GYRUS_DEVELOPMENT	-0.87	0.18	0	0	0	not significant
4532	GO_INTERFERON_ALPHA_PRODUCTION	-0.87	0.18	0	0	0	not significant
4533	GO_POSITIVE_REGULATION_OF_CHROMOSOME_SEPARATION	-0.87	0.18	0	0	0	not significant
4534	GO_ROSTROCAUDAL_NEURAL_TUBE_PATTERNING	-0.87	0.18	0	0	0	not significant
4535	GO_TRNA_SPECIFIC_ADENOSINE_DEAMINASE_ACTIVITY	-0.87	0.18	0	0	0	not significant
4536	PID_ERB_GENOMIC_PATHWAY	-0.87	0.18	0	0	0	not significant
4537	REACTOME_THROMBIN_SIGNALLING_THROUGH_PROTEINASE_ACTIVATED_RECEPT	-0.87	0.18	0	0	0	not significant
4538	YAGI_AML_RELAPSE_PROGNOSIS	-0.87	0.18	0	0	0	not significant
4539	CERVERA_SDHB_TARGETS_1_UP	-0.87	0.17	0	0	0	not significant
4540	GO_PROTEIN_K63_LINKED_DEUBIQUITINATION	-0.87	0.17	0	0	0	not significant
4541	GO_STRIATED_MUSCLE_CELL_APOPTOTIC_PROCESS	-0.87	0.17	0	0	0	not significant
4542	MA_MYELOID_DIFFERENTIATION_UP	-0.87	0.17	0	0	0	not significant
4543	PID_ATM_PATHWAY	-0.87	0.17	0	0	0	not significant
4544	REACTOME_OVARIAN_TUMOR_DOMAIN_PROTEASES	-0.87	0.17	0	0	0	not significant
4545	ZHAN_MULTIPLE_MYELOMA_CD1_AND_CD2_DN	-0.87	0.17	0	0	0	not significant
4546	CHR17Q23	-0.87	0.16	0	0	0	not significant
4547	CHR2P25	-0.87	0.16	0	0	0	not significant
4548	GO_CLATHRIN_BINDING	-0.87	0.16	0	0	0	not significant
4549	REACTOME_APOPTOTIC_EXECUTION_PHASE	-0.87	0.16	0	0	0	not significant
4550	REACTOME_INTRA_GOLGI_TRAFFIC	-0.87	0.16	0	0	0	not significant
4551	KEGG_ENDOMETRIAL_CANCER	-0.87	0.15	0	0	0	not significant
4552	LANDIS_ERBB2_BREAST_PRENEOPLASTIC_DN	-0.87	0.15	0	0	0	not significant
4553	REACTOME_CARDIAC_CONDUCTION	-0.87	0.15	0	0	0	not significant
4554	REN_ALVEOLAR_RHABDOMYOSARCOMA_UP	-0.87	0.15	0	0	0	not significant
4555	OUILLETTE_CLL_13Q14_DELETION_UP	-0.87	0.14	0	0	0	not significant
4556	RIZ_ERYTHROID_DIFFERENTIATION	-0.87	0.14	0	0	0	not significant
4557	GO_EPHRIN_RECEPTOR_SIGNALING_PATHWAY	-0.87	0.13	0	0	0	not significant
4558	GO_PROTEIN_TYROSINE_KINASE_BINDING	-0.87	0.13	0	0	0	not significant
4559	GO_RIBONUCLEASE_ACTIVITY	-0.87	0.13	0	0	0	not significant
4560	GO_UBIQUITIN_BINDING	-0.87	0.13	0	0	0	not significant
4561	RAGHAVACHARI_PLATELET_SPECIFIC_GENES	-0.87	0.13	0	0	0	not significant
4562	REACTOME_MACROAUTOPHAGY	-0.87	0.13	0	0	0	not significant
4563	GO_NUCLEOBASE_CONTAINING_SMALL_MOLECULE_BIOSYNTHETIC_PROCESS	-0.87	0.12	0	0	0	not significant
4564	REACTOME_NEGATIVE_REGULATION_OF_THE_PI3K_AKT_NETWORK	-0.87	0.12	0	0	0	not significant
4565	ROME_INSULIN_TARGETS_IN_MUSCLE_DN	-0.87	0.11	0	0	0	not significant
4566	CHR12Q24	-0.87	0.07	0	0	0	not significant

4567	GO_REGULATION_OF_ACTIN_FILAMENT_BASED_PROCESS	-0.87	0.05	0	0	0	not significant
4568	BRUINS_UVC_RESPONSE_EARLY_LATE	-0.87	0.04	0	0	0	not significant
4569	GO_REGULATION_OF_SUPRAMOLECULAR_FIBER_ORGANIZATION	-0.87	0.03	0	0	0	not significant
4570	GO_CARDIOBLAST_PROLIFERATION	-0.86	0.21	0	0	0	not significant
4571	GO_RESPONSE_TO_MUSCLE_ACTIVITY	-0.86	0.21	0	0	0	not significant
4572	GO_ALDITOL_METABOLIC_PROCESS	-0.86	0.2	0	0	0	not significant
4573	GO_NEUROTROPHIN_TRK_RECEPTOR_BINDING	-0.86	0.2	0	0	0	not significant
4574	GO_NUCLEAR_MEMBRANE_ORGANIZATION	-0.86	0.2	0	0	0	not significant
4575	GO_PEPTIDE_HORMONE_BINDING	-0.86	0.2	0	0	0	not significant
4576	GO_POSITIVE_REGULATION_OF_METANEPHROS_DEVELOPMENT	-0.86	0.2	0	0	0	not significant
4577	GO_PRE_MRNA_INTRONIC_BINDING	-0.86	0.2	0	0	0	not significant
4578	GO_REGULATION_OF_ENDODEOXYRIBONUCLEASE_ACTIVITY	-0.86	0.2	0	0	0	not significant
4579	GO_UBIQUITIN_SPECIFIC_PROTEASE_BINDING	-0.86	0.2	0	0	0	not significant
4580	ROZANOV_MMP14_CORRELATED	-0.86	0.2	0	0	0	not significant
4581	GENTILE_UV_LOW_DOSE_UP	-0.86	0.19	0	0	0	not significant
4582	GO_EMBRYONIC_NEUROCRANIUM_MORPHOGENESIS	-0.86	0.19	0	0	0	not significant
4583	GO_INOSITOL_PHOSPHATE_PHOSPHATASE_ACTIVITY	-0.86	0.19	0	0	0	not significant
4584	GO_INTEGRAL_COMPONENT_OF_PRESYNAPTIC_ACTIVE_ZONE_MEMBRANE	-0.86	0.19	0	0	0	not significant
4585	GO_MODIFICATION_OF_POSTSYNAPTIC_STRUCTURE	-0.86	0.19	0	0	0	not significant
4586	GO_POSITIVE_REGULATION_OF_CELL_AGING	-0.86	0.19	0	0	0	not significant
4587	GO_PRIMARY_MIRNA_BINDING	-0.86	0.19	0	0	0	not significant
4588	GO_REGULATION_OF_FEEDING_BEHAVIOR	-0.86	0.19	0	0	0	not significant
4589	GO_REGULATION_OF_NEUROTRANSMITTER_UPTAKE	-0.86	0.19	0	0	0	not significant
4590	GO_RISC_LOADING_COMPLEX	-0.86	0.19	0	0	0	not significant
4591	GO_VASCULAR_ASSOCIATED_SMOOTH_MUSCLE_CELL_MIGRATION	-0.86	0.19	0	0	0	not significant
4592	REACTOME_PKA_ACTIVATION_IN_GLUCAGON_SIGNALLING	-0.86	0.19	0	0	0	not significant
4593	REACTOME_ZINC_INFLUX_INTO_CELLS_BY_THE_SLC39_GENE_FAMILY	-0.86	0.19	0	0	0	not significant
4594	CHR3P26	-0.86	0.18	0	0	0	not significant
4595	GENTILE_UV_RESPONSE_CLUSTER_D9	-0.86	0.18	0	0	0	not significant
4596	GO_ACTIN_MONOMER_BINDING	-0.86	0.18	0	0	0	not significant
4597	GO_ACTIVATION_OF_CYSTEINE_TYPE_ENDOPEPTIDASE_ACTIVITY_INVOLVED_IN_A	-0.86	0.18	0	0	0	not significant
4598	GO_DOUBLE_STRAND_BREAK_REPAIR_VIA_SINGLE_STRAND_ANNEALING	-0.86	0.18	0	0	0	not significant
4599	GO_MAINTENANCE_OF_DNA_REPEAT_ELEMENTS	-0.86	0.18	0	0	0	not significant
4600	GO_NEGATIVE_REGULATION_OF_DEFENSE_RESPONSE_TO_VIRUS	-0.86	0.18	0	0	0	not significant
4601	GO_PEPTIDYL_LYSINE_MONOMETHYLATION	-0.86	0.18	0	0	0	not significant
4602	GO_POSITIVE_REGULATION_OF_GLYCOPROTEIN_METABOLIC_PROCESS	-0.86	0.18	0	0	0	not significant
4603	GO_REGULATION_OF_SUPEROXIDE_METABOLIC_PROCESS	-0.86	0.18	0	0	0	not significant
4604	REACTOME_MAPK1_ERK2_ACTIVATION	-0.86	0.18	0	0	0	not significant
4605	REACTOME_REGULATION_OF_LOCALIZATION_OF_FOXO_TRANSCRIPTION_FACTOF	-0.86	0.18	0	0	0	not significant
4606	BIOCARTA_SM_PATHWAY	-0.86	0.17	0	0	0	not significant
4607	GO_NEGATIVE_REGULATION_OF_CALCIUM_ION_TRANSMEMBRANE_TRANSPORT	-0.86	0.17	0	0	0	not significant
4608	KORKOLA_EMBRYONAL_CARCINOMA	-0.86	0.17	0	0	0	not significant
4609	OKAWA_NEUROBLASTOMA_1P36_31_DELETION	-0.86	0.17	0	0	0	not significant
4610	TSENG_ADIPOGENIC_POTENTIAL_UP	-0.86	0.17	0	0	0	not significant
4611	GO_OLIGODENDROCYTE_DIFFERENTIATION	-0.86	0.16	0	0	0	not significant
4612	TOMLINS_PROSTATE_CANCER_UP	-0.86	0.16	0	0	0	not significant
4613	GO_NAD_BINDING	-0.86	0.15	0	0	0	not significant
4614	GO_PROTEIN_MONOUBIQUITINATION	-0.86	0.15	0	0	0	not significant
4615	GO_HISTONE_UBIQUITINATION	-0.86	0.14	0	0	0	not significant
4616	GO_REGULATION_OF_GLIOGENESIS	-0.86	0.14	0	0	0	not significant
4617	GO_TETHERING_COMPLEX	-0.86	0.14	0	0	0	not significant
4618	PID_RAC1_REG_PATHWAY	-0.86	0.14	0	0	0	not significant
4619	GO_NEGATIVE_REGULATION_OF_EPITHELIAL_CELL_PROLIFERATION	-0.86	0.12	0	0	0	not significant
4620	GO_NEGATIVE_REGULATION_OF_TRANSMEMBRANE_RECEPTOR_PROTEIN_SERINE	-0.86	0.12	0	0	0	not significant
4621	REACTOME_RHO_GTPASES_ACTIVATE_PKNS	-0.86	0.12	0	0	0	not significant
4622	GO_MONOCARBOXYLIC_ACID_TRANSPORT	-0.86	0.11	0	0	0	not significant
4623	REACTOME_EUKARYOTIC_TRANSLATION_INITIATION	-0.86	0.1	0	0	0	not significant
4624	GO_SPERM_PART	-0.86	0.09	0	0	0	not significant
4625	PROVENZANI_METASTASIS_DN	-0.86	0.08	0	0	0	not significant
4626	GO_PYRIMIDINE_NUCLEOTIDE_CATABOLIC_PROCESS	-0.85	0.19	0	0	0	not significant
4627	MATZUK_SPERMATOGONIA	-0.85	0.19	0	0	0	not significant
4628	REACTOME_TYSND1_CLEAVES_PEROXISOMAL_PROTEINS	-0.85	0.19	0	0	0	not significant
4629	FINETTI_BREAST_CANCERS_KINOME_BLUE	-0.85	0.18	0	0	0	not significant
4630	GO_COPII_COATED_VESICLE_CARGO_LOADING	-0.85	0.18	0	0	0	not significant
4631	GO_DYNEIN_LIGHT_INTERMEDIATE_CHAIN_BINDING	-0.85	0.18	0	0	0	not significant
4632	GO_HISTONE_H4_K20_METHYLATION	-0.85	0.18	0	0	0	not significant
4633	GO_NEGATIVE_REGULATION_OF_NATURAL_KILLER_CELL_ACTIVATION	-0.85	0.18	0	0	0	not significant
4634	GO_NUCLEAR_PORE_COMPLEX_ASSEMBLY	-0.85	0.18	0	0	0	not significant
4635	GO_REGULATION_OF_INTEGRIN_ACTIVATION	-0.85	0.18	0	0	0	not significant
4636	GO_REGULATION_OF_RHODOPSIN_MEDIATED_SIGNALING_PATHWAY	-0.85	0.18	0	0	0	not significant
4637	GO_SKELETAL_MYOFIBRIL_ASSEMBLY	-0.85	0.18	0	0	0	not significant
4638	HEIDENBLAD_AMPLIFIED_IN_SOFT_TISSUE_CANCER	-0.85	0.18	0	0	0	not significant
4639	REACTOME_METABOLISM_OF_NITRIC_OXIDE:_ENOS_ACTIVATION_AND_REGULATIC	-0.85	0.18	0	0	0	not significant
4640	BIOCARTA_PLCE_PATHWAY	-0.85	0.17	0	0	0	not significant
4641	GO_CEREBELLAR_CORTEX_MORPHOGENESIS	-0.85	0.17	0	0	0	not significant
4642	GO_G_PROTEIN_BETA_SUBUNIT_BINDING	-0.85	0.17	0	0	0	not significant
4643	GO_IONOTROPIC_GLUTAMATE_RECEPTOR_BINDING	-0.85	0.17	0	0	0	not significant
4644	GO_NEGATIVE_REGULATION_OF_CELL_JUNCTION_ASSEMBLY	-0.85	0.17	0	0	0	not significant
4645	GO_NEGATIVE_REGULATION_OF_GENE_SILENCING	-0.85	0.17	0	0	0	not significant
4646	GO_NEGATIVE_REGULATION_OF_MICROTUBULE_POLYMERIZATION_OR_DEPOLYMI	-0.85	0.17	0	0	0	not significant
4647	GO_NEGATIVE_REGULATION_OF_OSTEOCLAST_DIFFERENTIATION	-0.85	0.17	0	0	0	not significant
4648	GO_NEGATIVE_REGULATION_OF_POTASSIUM_ION_TRANSPORT	-0.85	0.17	0	0	0	not significant
4649	GO_REGULATION_OF_GLUCOCORTICOID_RECEPTOR_SIGNALING_PATHWAY	-0.85	0.17	0	0	0	not significant
4650	NUMATA_CSF3_SIGNALING_VIA_STAT3	-0.85	0.17	0	0	0	not significant
4651	REACTOME_INTERLEUKIN_37_SIGNALING	-0.85	0.17	0	0	0	not significant
4652	REACTOME_TRISTETRAPROLIN_TTP_ZFP36_BINDS_AND_DESTABILIZES_MRNA	-0.85	0.17	0	0	0	not significant
4653	SESTO_RESPONSE_TO_UV_C3	-0.85	0.17	0	0	0	not significant
4654	WANG_RECURRENT_LIVER_CANCER_UP	-0.85	0.17	0	0	0	not significant
4655	ZHANG_ADIPOGENESIS_BY_BMP7	-0.85	0.17	0	0	0	not significant
4656	CREIGHTON_AKT1_SIGNALING_VIA_MTOR_UP	-0.85	0.16	0	0	0	not significant
4657	GAZDA_DIAMOND_BLACKFAN_ANEMIA_PROGENITOR_UP	-0.85	0.16	0	0	0	not significant
4658	REACTOME_NEF_AND_SIGNAL_TRANSDUCTION	-0.85	0.16	0	0	0	not significant
4659	GARGALOVIC_RESPONSE_TO_OXIDIZED_PHOSPHOLIPIDS_YELLOW_UP	-0.85	0.15	0	0	0	not significant
4660	GO_DNA_DEALKYLATION	-0.85	0.15	0	0	0	not significant
4661	JOHANSSON_BRAIN_CANCER_EARLY_VS_LATE_DN	-0.85	0.15	0	0	0	not significant
4662	GO_RESPONSE_TO_AXON_INJURY	-0.85	0.14	0	0	0	not significant
4663	HENDRICKS_SMARCA4_TARGETS_UP	-0.85	0.14	0	0	0	not significant
4664	REACTOME_INSULIN_RECEPTOR_SIGNALLING_CASCADE	-0.85	0.14	0	0	0	not significant
4665	GO_INTRACILIARY_TRANSPORT_INVOLVED_IN_CILIUM_ASSEMBLY	-0.85	0.13	0	0	0	not significant
4666	KYNG_WERNER_SYNDROM_AND_NORMAL_AGING_UP	-0.85	0.11	0	0	0	not significant
4667	GO_GLUCOSE_METABOLIC_PROCESS	-0.85	0.1	0	0	0	not significant
4668	BYSTRYKH_HEMATOPOIESIS_STEM_CELL_QTL_CIS	-0.85	0.08	0	0	0	not significant
4669	GO_INSULIN_RECEPTOR_SIGNALING_PATHWAY	-0.85	0.07	0	0	0	not significant
4670	GRYDER_PAX3FOXO1_ENHANCERS_KO_DOWN	-0.85	0.02	0	0	0	not significant
4671	GO_REGULATION_OF_GLYCOPROTEIN_METABOLIC_PROCESS	-0.84	0.19	0	0	0	not significant
4672	GO_POSITIVE_REGULATION_OF_CALCINEURIN_MEDIATED_SIGNALING	-0.84	0.18	0	0	0	not significant
4673	GO_RESPONSE_TO_METHYLMERCURY	-0.84	0.18	0	0	0	not significant
4674	GO_RETINAL_METABOLIC_PROCESS	-0.84	0.18	0	0	0	not significant
4675	MARIADASON_RESPONSE_TO_CURCUMIN_SULINDAC_7	-0.84	0.18	0	0	0	not significant

4676	REACTOME_PROCESSING_OF_SMDT1	-0.84	0.18	0	0	0	not significant
4677	AMIT_DELAYED_EARLY_GENES	-0.84	0.17	0	0	0	not significant
4678	BIOCARTA_CHREBP_PATHWAY	-0.84	0.17	0	0	0	not significant
4679	GO_ADRENAL_GLAND_DEVELOPMENT	-0.84	0.17	0	0	0	not significant
4680	GO_CONTRACTILE_RING	-0.84	0.17	0	0	0	not significant
4681	GO_EXCITATORY_SYNAPSE_ASSEMBLY	-0.84	0.17	0	0	0	not significant
4682	GO_NUCLEOTIDE_EXCISION_REPAIR_DNA_DUPLEX_UNWINDING	-0.84	0.17	0	0	0	not significant
4683	GO_PROTEIN_NEDDYLATION	-0.84	0.17	0	0	0	not significant
4684	GO_REGULATION_OF_GTP_BINDING	-0.84	0.17	0	0	0	not significant
4685	GO_TRANSLOCON_COMPLEX	-0.84	0.17	0	0	0	not significant
4686	REACTOME_NEGATIVE_FEEDBACK_REGULATION_OF_MAPK_PATHWAY	-0.84	0.17	0	0	0	not significant
4687	REACTOME_REGULATION_OF_IFNG_SIGNALING	-0.84	0.17	0	0	0	not significant
4688	WORSCHECH_TUMOR_EVASION_AND_TOLEROGENICITY_DN	-0.84	0.17	0	0	0	not significant
4689	XU_RESPONSE_TO_TRETINOIN_DN	-0.84	0.17	0	0	0	not significant
4690	BIOCARTA_AKAPCENTROSOME_PATHWAY	-0.84	0.16	0	0	0	not significant
4691	BIOCARTA_ETC_PATHWAY	-0.84	0.16	0	0	0	not significant
4692	GARGALOVIC_RESPONSE_TO_OXIDIZED_PHOSPHOLIPIDS_GREEN_UP	-0.84	0.16	0	0	0	not significant
4693	GO_CELLULAR_RESPONSE_TO_GONADOTROPIN_STIMULUS	-0.84	0.16	0	0	0	not significant
4694	GO_FACE_MORPHOGENESIS	-0.84	0.16	0	0	0	not significant
4695	GO_MITOCHONDRIAL_ACETYL_COA_BIOSYNTHETIC_PROCESS_FROM_PYRUVATE	-0.84	0.16	0	0	0	not significant
4696	GO_MYOSIN_V_BINDING	-0.84	0.16	0	0	0	not significant
4697	GO_NEGATIVE_REGULATION_OF_RETINOIC_ACID_RECEPTOR_SIGNALING_PATHWA	-0.84	0.16	0	0	0	not significant
4698	GO_REGULATION_OF_FEMALE_GONAD_DEVELOPMENT	-0.84	0.16	0	0	0	not significant
4699	GO_SMAD_PROTEIN_COMPLEX_ASSEMBLY	-0.84	0.16	0	0	0	not significant
4700	GO_VENTRICULAR_SYSTEM_DEVELOPMENT	-0.84	0.16	0	0	0	not significant
4701	KEGG_PROPANOATE_METABOLISM	-0.84	0.16	0	0	0	not significant
4702	LANDIS_ERBB2_BREAST_TUMORS_65_UP	-0.84	0.16	0	0	0	not significant
4703	PID_THROMBIN_PAR4_PATHWAY	-0.84	0.16	0	0	0	not significant
4704	REACTOME_FOXO_MEDIATED_TRANSCRIPTION_OF_OXIDATIVE_STRESS_METABOL	-0.84	0.16	0	0	0	not significant
4705	ZEMBUTSU_SENSITIVITY_TO_VINBLASTINE	-0.84	0.16	0	0	0	not significant
4706	BIOCARTA_CXCR4_PATHWAY	-0.84	0.15	0	0	0	not significant
4707	GO_HISTONE_MONOUBIQUITINATION	-0.84	0.15	0	0	0	not significant
4708	GO_REGULATION_OF_IRE1_MEDIATED_UNFOLDED_PROTEIN_RESPONSE	-0.84	0.15	0	0	0	not significant
4709	GO_REGULATION_OF_TELOMERE_CAPPING	-0.84	0.15	0	0	0	not significant
4710	GO_RESPONSE_TO_AUDITORY_STIMULUS	-0.84	0.15	0	0	0	not significant
4711	GO_SYNAPTIC_VESICLE_CLUSTERING	-0.84	0.15	0	0	0	not significant
4712	PID_ALK1_PATHWAY	-0.84	0.15	0	0	0	not significant
4713	PID_IL8_CXCR2_PATHWAY	-0.84	0.15	0	0	0	not significant
4714	REACTOME_INTERLEUKIN_RECEPTOR_SHC_SIGNALING	-0.84	0.15	0	0	0	not significant
4715	WEIGEL_OXIDATIVE_STRESS_BY_TBH_AND_H2O2	-0.84	0.15	0	0	0	not significant
4716	GO_DNA_DEALKYLATION_INVOLVED_IN_DNA_REPAIR	-0.84	0.14	0	0	0	not significant
4717	GO_FATTY_ACID_BINDING	-0.84	0.14	0	0	0	not significant
4718	GO_NEGATIVE_REGULATION_OF_RESPONSE_TO_WOUNDING	-0.84	0.14	0	0	0	not significant
4719	GO_REGULATION_OF_STEM_CELL_PROLIFERATION	-0.84	0.14	0	0	0	not significant
4720	REACTOME_UPTAKE_AND_ACTIONS_OF_BACTERIAL_TOXINS	-0.84	0.14	0	0	0	not significant
4721	GO_CARDIAC_SEPTUM_MORPHOGENESIS	-0.84	0.13	0	0	0	not significant
4722	GO_RNA_DESTABILIZATION	-0.84	0.13	0	0	0	not significant
4723	LEE_CALORIE_RESTRICTION_MUSCLE_DN	-0.84	0.13	0	0	0	not significant
4724	REACTOME_NRAGE_SIGNALS_DEATH_THROUGH_JNK	-0.84	0.12	0	0	0	not significant
4725	VANTVEER_BREAST_CANCER_POOR_PROGNOSIS	-0.84	0.12	0	0	0	not significant
4726	CHR1Q25	-0.84	0.11	0	0	0	not significant
4727	KEGG_MELANOGENESIS	-0.84	0.11	0	0	0	not significant
4728	PID_BETA_CATENIN_NUC_PATHWAY	-0.84	0.11	0	0	0	not significant
4729	REACTOME_DEADENYLATION_DEPENDENT_MRNA_DECAY	-0.84	0.11	0	0	0	not significant
4730	REACTOME_DEPURINATION	-0.84	0.11	0	0	0	not significant
4731	OUELLET_CULTURED_OVARIAN_CANCER_INVASIVE_VS_LMP_UP	-0.84	0.09	0	0	0	not significant
4732	ZHONG_RESPONSE_TO_AZACITIDINE_AND_TSA_DN	-0.84	0.09	0	0	0	not significant
4733	GO_REGULATION_OF_BLOOD_CIRCULATION	-0.84	0.06	0	0	0	not significant
4734	GO_NEURON_APOPTOTIC_PROCESS	-0.84	0.04	0	0	0	not significant
4735	GO_PROTEIN_ACETYLATION	-0.84	0.04	0	0	0	not significant
4736	GO_SENSORY_SYSTEM_DEVELOPMENT	-0.84	0.03	0	0	0	not significant
4737	KEGG_PENTOSE_AND_GLUCURONATE_INTERCONVERSIONS	-0.83	0.18	0	0	0	not significant
4738	CHRXQ21	-0.83	0.17	0	0	0	not significant
4739	GO_BERGMANN_GLIAL_CELL_DIFFERENTIATION	-0.83	0.17	0	0	0	not significant
4740	GO_DYNEIN_HEAVY_CHAIN_BINDING	-0.83	0.17	0	0	0	not significant
4741	GO_GLYCOSYLCERAMIDE_CATABOLIC_PROCESS	-0.83	0.17	0	0	0	not significant
4742	GO_LOW_DENSITY_LIPOPROTEIN_PARTICLE_RECEPTOR_BIOSYNTHETIC_PROCES	-0.83	0.17	0	0	0	not significant
4743	GO_NEGATIVE_REGULATION_OF_POSTTRANSCRIPTIONAL_GENE_SILENCING	-0.83	0.17	0	0	0	not significant
4744	GO_POSITIVE_REGULATION_OF_MACROPHAGE_MIGRATION	-0.83	0.17	0	0	0	not significant
4745	GO_REGULATION_OF_AEROBIC_RESPIRATION	-0.83	0.17	0	0	0	not significant
4746	GO_REGULATION_OF_WATER_LOSS_VIA_SKIN	-0.83	0.17	0	0	0	not significant
4747	GO_VESICLE_FUSION_WITH_GOLGI_APPARATUS	-0.83	0.17	0	0	0	not significant
4748	HEIDENBLAD_AMPLIFIED_IN_BONE_CANCER	-0.83	0.17	0	0	0	not significant
4749	BIOCARTA_TID_PATHWAY	-0.83	0.16	0	0	0	not significant
4750	GO_ERYTHROCYTE_DEVELOPMENT	-0.83	0.16	0	0	0	not significant
4751	GO_NEGATIVE_REGULATION_OF_CARDIAC_MUSCLE_CELL_DIFFERENTIATION	-0.83	0.16	0	0	0	not significant
4752	GO_NEGATIVE_REGULATION_OF_SYSTEMIC_ARTERIAL_BLOOD_PRESSURE	-0.83	0.16	0	0	0	not significant
4753	GO_PRODUCTION_OF_SIRNA_INVOLVED_IN_RNA_INTERFERENCE	-0.83	0.16	0	0	0	not significant
4754	GO_REGULATION_OF_GLUCONEOGENESIS_BY_REGULATION_OF_TRANSCRIPTION_	-0.83	0.16	0	0	0	not significant
4755	GO_UBIQUITIN_CONJUGATING_ENZYME_COMPLEX	-0.83	0.16	0	0	0	not significant
4756	REACTOME_INSULIN_PROCESSING	-0.83	0.16	0	0	0	not significant
4757	VERNELL_RETINOBLASTOMA_PATHWAY_DN	-0.83	0.16	0	0	0	not significant
4758	GO_CORPUS_CALLOSUM_DEVELOPMENT	-0.83	0.15	0	0	0	not significant
4759	GO_GUANOSINE_CONTAINING_COMPOUND_METABOLIC_PROCESS	-0.83	0.15	0	0	0	not significant
4760	GO_INTRINSIC_COMPONENT_OF_PRESYNAPTIC_ACTIVE_ZONE_MEMBRANE	-0.83	0.15	0	0	0	not significant
4761	GO_POSITIVE_REGULATION_OF_MYOBLAST_DIFFERENTIATION	-0.83	0.15	0	0	0	not significant
4762	GO_REGULATION_OF_OXIDATIVE_STRESS_INDUCED_INTRINSIC_APOPTOTIC_SIGNA	-0.83	0.15	0	0	0	not significant
4763	KEGG_TYROSINE_METABOLISM	-0.83	0.15	0	0	0	not significant
4764	REACTOME_ENOS_ACTIVATION	-0.83	0.15	0	0	0	not significant
4765	REACTOME_EPHB_MEDIATED_FORWARD_SIGNALING	-0.83	0.15	0	0	0	not significant
4766	SMID_BREAST_CANCER_RELAPSE_IN_BRAIN_UP	-0.83	0.15	0	0	0	not significant
4767	BIOCARTA_RAS_PATHWAY	-0.83	0.14	0	0	0	not significant
4768	GO_B_CELL_APOPTOTIC_PROCESS	-0.83	0.14	0	0	0	not significant
4769	GO_NEGATIVE_REGULATION_OF_CYCLIN_DEPENDENT_PROTEIN_KINASE_ACTIVITY	-0.83	0.14	0	0	0	not significant
4770	GO_REGULATION_OF_NEURONAL_SYNAPTIC_PLASTICITY	-0.83	0.14	0	0	0	not significant
4771	REACTOME_ACTIVATION_OF_IRF3_IRF7_MEDIATED_BY_TBK1_IKK_EPSILON	-0.83	0.14	0	0	0	not significant
4772	CHR9Q31	-0.83	0.13	0	0	0	not significant
4773	GO_ACTIN_NUCLEATION	-0.83	0.13	0	0	0	not significant
4774	GO_HEAD_MORPHOGENESIS	-0.83	0.13	0	0	0	not significant
4775	REACTOME_BUDDING_AND_MATURATION_OF_HIV_VIRION	-0.83	0.13	0	0	0	not significant
4776	CHR3Q25	-0.83	0.12	0	0	0	not significant
4777	GO_RESPONSE_TO_TESTOSTERONE	-0.83	0.12	0	0	0	not significant
4778	GO_ION_CHANNEL_REGULATOR_ACTIVITY	-0.83	0.11	0	0	0	not significant
4779	GO_MANGANESE_ION_BINDING	-0.83	0.11	0	0	0	not significant
4780	ZHAN_MULTIPLE_MYELOMA_UP	-0.83	0.11	0	0	0	not significant
4781	GO_CHEMICAL_SYNAPTIC_TRANSMISSION_POSTSYNAPTIC	-0.83	0.1	0	0	0	not significant
4782	GO_MEGAKARYOCYTE_DIFFERENTIATION	-0.83	0.1	0	0	0	not significant
4783	RIZKI_TUMOR_INVASIVENESS_2D_DN	-0.83	0.1	0	0	0	not significant
4784	DITTMER_PTHLH_TARGETS_DN	-0.83	0.08	0	0	0	not significant

4785	GO_NEURAL_TUBE_FORMATION	-0.83	0.08	0	0	0	not significant
4786	GO_POSITIVE_REGULATION_OF_ACTIN_FILAMENT_POLYMERIZATION	-0.83	0.08	0	0	0	not significant
4787	SIG_PIP3_SIGNALING_IN_CARDIAC_MYOCTES	-0.83	0.08	0	0	0	not significant
4788	GO_NEGATIVE_REGULATION_OF_NEURON_APOPTOTIC_PROCESS	-0.83	0.07	0	0	0	not significant
4789	GO_REGULATION_OF_ENDOCYTOSIS	-0.83	0.05	0	0	0	not significant
4790	GO_NEGATIVE_REGULATION_OF_CARDIOCYTE_DIFFERENTIATION	-0.82	0.16	0	0	0	not significant
4791	GO_NEGATIVE_REGULATION_OF_INFLAMMATORY_RESPONSE_TO_ANTIGENIC_STIM	-0.82	0.16	0	0	0	not significant
4792	GO_NEGATIVE_REGULATION_OF_KERATINOCYTE_PROLIFERATION	-0.82	0.16	0	0	0	not significant
4793	GO_POSITIVE_REGULATION_OF_MORPHOGENESIS_OF_AN_EPITHELIUM	-0.82	0.16	0	0	0	not significant
4794	GO_PROTON_TRANSPORTING_V_TYPE_ATPASE_V1_DOMAIN	-0.82	0.16	0	0	0	not significant
4795	IIZUKA_LIVER_CANCER_PROGRESSION_L1_G1_DN	-0.82	0.16	0	0	0	not significant
4796	ZHU_SKIL_TARGETS_DN	-0.82	0.16	0	0	0	not significant
4797	GO_ALDEHYDE_CATABOLIC_PROCESS	-0.82	0.15	0	0	0	not significant
4798	GO_MHC_CLASS_I_PEPTIDE_LOADING_COMPLEX	-0.82	0.15	0	0	0	not significant
4799	GO_NEGATIVE_REGULATION_OF_B_CELL_PROLIFERATION	-0.82	0.15	0	0	0	not significant
4800	GO_OXIDOREDUCTASE_ACTIVITY_OXIDIZING_METAL_IONS	-0.82	0.15	0	0	0	not significant
4801	GO_PHOSPHORYLATED_CARBOHYDRATE_DEPHOSPHORYLATION	-0.82	0.15	0	0	0	not significant
4802	GO_REGULATION_OF_HISTONE_H2B_UBIQUITINATION	-0.82	0.15	0	0	0	not significant
4803	GO_RETINA_MORPHOGENESIS_IN_CAMERA_TYPE_EYE	-0.82	0.15	0	0	0	not significant
4804	GO_SCHWANN_CELL_DEVELOPMENT	-0.82	0.15	0	0	0	not significant
4805	GO_SULFURTRANSFERASE_ACTIVITY	-0.82	0.15	0	0	0	not significant
4806	GO_SYMMETRIC_SYNAPSE	-0.82	0.15	0	0	0	not significant
4807	GO_VITAMIN_TRANSMEMBRANE_TRANSPORTER_ACTIVITY	-0.82	0.15	0	0	0	not significant
4808	REACTOME_PHOSPHATE_BOND_HYDROLYSIS_BY_NUDT_PROTEINS	-0.82	0.15	0	0	0	not significant
4809	REACTOME_PLATELET_CALCIUM_HOMEOSTASIS	-0.82	0.15	0	0	0	not significant
4810	BUCKANOVICH_T_LYMPHOCYTE_HOMING_ON_TUMOR_DN	-0.82	0.14	0	0	0	not significant
4811	CHR6Q14	-0.82	0.14	0	0	0	not significant
4812	GO_ACTIVATION_OF_PROTEIN_KINASE_A_ACTIVITY	-0.82	0.14	0	0	0	not significant
4813	GO_EMBRYONIC_PATTERN_SPECIFICATION	-0.82	0.14	0	0	0	not significant
4814	GO_ENDOLYSOSOME	-0.82	0.14	0	0	0	not significant
4815	GO_MICROFIBRIL	-0.82	0.14	0	0	0	not significant
4816	REACTOME_ASSEMBLY_OF_THE_HIV_VIRION	-0.82	0.14	0	0	0	not significant
4817	REACTOME_HYALURONAN_UPTAKE_AND_DEGRADATION	-0.82	0.14	0	0	0	not significant
4818	REACTOME_JNK_C_JUN_KINASES_PHOSPHORYLATION_AND_ACTIVATION_MEDIATE	-0.82	0.14	0	0	0	not significant
4819	RICKMAN_HEAD_AND_NECK_CANCER_A	-0.82	0.13	0	0	0	not significant
4820	GO_POLYSOMAL_RIBOSOME	-0.82	0.12	0	0	0	not significant
4821	REACTOME_DEADENYLATION_OF_MRNA	-0.82	0.12	0	0	0	not significant
4822	REACTOME_METALLOPROTEASE_DUBS	-0.82	0.12	0	0	0	not significant
4823	CHEN_NEUROBLASTOMA_COPY_NUMBER_GAINS	-0.82	0.11	0	0	0	not significant
4824	CHR1Q44	-0.82	0.11	0	0	0	not significant
4825	GO_CYCLIN_DEPENDENT_PROTEIN_KINASE_ACTIVITY	-0.82	0.11	0	0	0	not significant
4826	GO_NF_KAPPAB_BINDING	-0.82	0.11	0	0	0	not significant
4827	REACTOME_SIGNALING_BY_EGFR	-0.82	0.11	0	0	0	not significant
4828	GO_MORPHOGENESIS_OF_AN_EPITHELIAL_SHEET	-0.82	0.1	0	0	0	not significant
4829	PID_KIT_PATHWAY	-0.82	0.1	0	0	0	not significant
4830	GO_POSITIVE_REGULATION_OF_PROTEIN_LOCALIZATION_TO_MEMBRANE	-0.82	0.07	0	0	0	not significant
4831	CHR1P13	-0.82	0.06	0	0	0	not significant
4832	GO_NUCLEOTIDE_EXCISION_REPAIR	-0.82	0.06	0	0	0	not significant
4833	GO_NEGATIVE_REGULATION_OF_NEURON_DEATH	-0.82	0.04	0	0	0	not significant
4834	GO_ENDOSOMAL_TRANSPORT	-0.82	0.01	0	0	0	not significant
4835	GO_CELL_CELL_ADHESION_INVOLVED_IN_GASTRULATION	-0.81	0.17	0	0	0	not significant
4836	CHR4P12	-0.81	0.16	0	0	0	not significant
4837	GO_DORSAL_SPINAL_CORD_DEVELOPMENT	-0.81	0.16	0	0	0	not significant
4838	GO_POSITIVE_REGULATION_OF_NUCLEASE_ACTIVITY	-0.81	0.16	0	0	0	not significant
4839	GO_SPINAL_CORD_PATTERNING	-0.81	0.16	0	0	0	not significant
4840	MIKHAYLOVA_OXIDATIVE_STRESS_RESPONSE_VIA_VHL_UP	-0.81	0.16	0	0	0	not significant
4841	GO_AMINO_ACID_SALVAGE	-0.81	0.15	0	0	0	not significant
4842	GO_CERAMIDE_TRANSPORT	-0.81	0.15	0	0	0	not significant
4843	GO_EXTRINSIC_COMPONENT_OF_MITOCHONDRIAL_INNER_MEMBRANE	-0.81	0.15	0	0	0	not significant
4844	GO_GATOR1_COMPLEX	-0.81	0.15	0	0	0	not significant
4845	GO_INTERMEDIATE_FILAMENT_BINDING	-0.81	0.15	0	0	0	not significant
4846	GO_POSITIVE_REGULATION_OF_PROTEIN_DEUBIQUITINATION	-0.81	0.15	0	0	0	not significant
4847	GO_PROTEIN_LOCALIZATION_TO_CILIARY_MEMBRANE	-0.81	0.15	0	0	0	not significant
4848	GO_PROTEIN_LOCALIZATION_TO_EARLY_ENDOSOME	-0.81	0.15	0	0	0	not significant
4849	GO_REGULATION_OF_CELLULAR_RESPONSE_TO_HYPOXIA	-0.81	0.15	0	0	0	not significant
4850	GO_REGULATION_OF_HELICASE_ACTIVITY	-0.81	0.15	0	0	0	not significant
4851	HOFMANN_MYELODYSPLASTIC_SYNDROM_RISK_UP	-0.81	0.15	0	0	0	not significant
4852	KIM_RESPONSE_TO_TSA_AND_DECITABINE_DN	-0.81	0.15	0	0	0	not significant
4853	BAKER_HEMATOPOESIS_STAT1_TARGETS	-0.81	0.14	0	0	0	not significant
4854	GO_ALPHA_TUBULIN_BINDING	-0.81	0.14	0	0	0	not significant
4855	GO_CELLULAR_RESPONSE_TO_NITROSATIVE_STRESS	-0.81	0.14	0	0	0	not significant
4856	GO_DENDRITIC_CELL_ANTIGEN_PROCESSING_AND_PRESENTATION	-0.81	0.14	0	0	0	not significant
4857	GO_GUANYLYLTRANSFERASE_ACTIVITY	-0.81	0.14	0	0	0	not significant
4858	GO_HYPEROSMOTIC_RESPONSE	-0.81	0.14	0	0	0	not significant
4859	GO_REGULATION_OF_DENDRITIC_SPINE_MAINTENANCE	-0.81	0.14	0	0	0	not significant
4860	GO_RESPONSE_TO_INTERLEUKIN_6	-0.81	0.14	0	0	0	not significant
4861	GO_SPLICEOSOMAL_CONFORMATIONAL_CHANGES_TO_GENERATE_CATALYTIC_C	-0.81	0.14	0	0	0	not significant
4862	GO_STRESS_GRANULE_ASSEMBLY	-0.81	0.14	0	0	0	not significant
4863	GO_SUMO_TRANSFERASE_ACTIVITY	-0.81	0.14	0	0	0	not significant
4864	REACTOME_2_LTR_CIRCLE_FORMATION	-0.81	0.14	0	0	0	not significant
4865	REACTOME_NF_KB_ACTIVATION_THROUGH_FADD_RIP_1_PATHWAY_MEDIATED_BY	-0.81	0.14	0	0	0	not significant
4866	CHR2Q12	-0.81	0.13	0	0	0	not significant
4867	GO_POSITIVE_REGULATION_OF_MUSCLE_HYPERTROPHY	-0.81	0.13	0	0	0	not significant
4868	REACTOME_MRNA_DECAY_BY_5_TO_3_EXORIBONUCLEASE	-0.81	0.13	0	0	0	not significant
4869	GO_NEUROPEPTIDE_SIGNALING_PATHWAY	-0.81	0.12	0	0	0	not significant
4870	GO_POSITIVE_REGULATION_OF_REACTIVE_OXYGEN_SPECIES_METABOLIC_PROCE	-0.81	0.12	0	0	0	not significant
4871	REACTOME_FCERI_MEDIATED_CAPLUS2_MOBILIZATION	-0.81	0.12	0	0	0	not significant
4872	GO_CLATHRIN_ADAPTOR_COMPLEX	-0.81	0.11	0	0	0	not significant
4873	GO_CLATHRIN_COAT	-0.81	0.11	0	0	0	not significant
4874	CHR6Q25	-0.81	0.1	0	0	0	not significant
4875	CHRXP22	-0.81	0.1	0	0	0	not significant
4876	CROONQUIST_NRAS_VS_STROMAL_STIMULATION_UP	-0.81	0.1	0	0	0	not significant
4877	GO_CELLULAR_CARBOHYDRATE_CATABOLIC_PROCESS	-0.81	0.1	0	0	0	not significant
4878	GO_O_ACYLTRANSFERASE_ACTIVITY	-0.81	0.1	0	0	0	not significant
4879	GO_EMBRYONIC_SKELETAL_SYSTEM_MORPHOGENESIS	-0.81	0.09	0	0	0	not significant
4880	GO_REGULATION_OF_MONOOXYGENASE_ACTIVITY	-0.81	0.09	0	0	0	not significant
4881	GO_REGULATION_OF_SODIUM_ION_TRANSPORT	-0.81	0.09	0	0	0	not significant
4882	JOSEPH_RESPONSE_TO_SODIUM_BUTYRATE_DN	-0.81	0.09	0	0	0	not significant
4883	GO_REGULATION_OF_ACTIN_FILAMENT_BUNDLE_ASSEMBLY	-0.81	0.08	0	0	0	not significant
4884	GO_INTRACILIARY_TRANSPORT	-0.81	0.07	0	0	0	not significant
4885	ICHIBA_GRAFT_VERSUS_HOST_DISEASE_35D_UP	-0.81	0.06	0	0	0	not significant
4886	GO_TRANSITION_METAL_ION_HOMEOSTASIS	-0.81	0.04	0	0	0	not significant
4887	GO_CHROMOSOME_TELOMERIC_REGION	-0.81	0.03	0	0	0	not significant
4888	GO_COENZYME_BIOSYNTHETIC_PROCESS	-0.81	0.03	0	0	0	not significant
4889	GO_REGULATION_OF_AMINO_ACID_TRANSMEMBRANE_TRANSPORT	-0.80	0.16	0	0	0	not significant
4890	GO_GLUCOSAMINE_CONTAINING_COMPOUND_METABOLIC_PROCESS	-0.80	0.15	0	0	0	not significant
4891	GO_NEGATIVE_REGULATION_OF_POTASSIUM_ION_TRANSMEMBRANE_TRANSPORT	-0.80	0.15	0	0	0	not significant
4892	GO_NEPHRON_TUBULE_EPITHELIAL_CELL_DIFFERENTIATION	-0.80	0.15	0	0	0	not significant
4893	GO_OXIDOREDUCTASE_ACTIVITY_ACTING_ON_DIPHENOLS_AND_RELATED_SUBST/	-0.80	0.15	0	0	0	not significant

4894	GO_PROTEIN_NITROSYLATION	-0.80	0.15	0	0	0	not significant
4895	REACTOME_IRAK4_DEFICIENCY_TLR2_4	-0.80	0.15	0	0	0	not significant
4896	GO_CELLULAR_RESPONSE_TO_SALT_STRESS	-0.80	0.14	0	0	0	not significant
4897	GO_CEREBELLAR_CORTEX_FORMATION	-0.80	0.14	0	0	0	not significant
4898	GO_CILIARY_TRANSITION_FIBER	-0.80	0.14	0	0	0	not significant
4899	GO_FOREBRAIN_MORPHOGENESIS	-0.80	0.14	0	0	0	not significant
4900	GO_NEGATIVE_REGULATION_OF_DNA_TEMPLATED_TRANSCRIPTION_ELONGATION	-0.80	0.14	0	0	0	not significant
4901	GO_NEUROTROPHIN_RECEPTOR_BINDING	-0.80	0.14	0	0	0	not significant
4902	GO_POSITIVE_REGULATION_OF_FIBROBLAST_MIGRATION	-0.80	0.14	0	0	0	not significant
4903	GO_PROTEIN_DISULFIDE_OXIDOREDUCTASE_ACTIVITY	-0.80	0.14	0	0	0	not significant
4904	GO_REGULATION_OF_PROTEIN_MONOUBIQUITINATION	-0.80	0.14	0	0	0	not significant
4905	GO_RNA_SECONDARY_STRUCTURE_UNWINDING	-0.80	0.14	0	0	0	not significant
4906	GO_SOLUTE_PROTON_ANTIPORTER_ACTIVITY	-0.80	0.14	0	0	0	not significant
4907	GO_UDP_XYLOSYLTRANSFERASE_ACTIVITY	-0.80	0.14	0	0	0	not significant
4908	NIELSEN_LEIOMYOSARCOMA_CNN1_DN	-0.80	0.14	0	0	0	not significant
4909	REACTOME_PRESYNAPTIC_FUNCTION_OF_KAINATE_RECEPTORS	-0.80	0.14	0	0	0	not significant
4910	REACTOME_REGULATION_OF_FZD_BY_UBIQUITINATION	-0.80	0.14	0	0	0	not significant
4911	VERRECCHIA_RESPONSE_TO_TGFB1_C1	-0.80	0.14	0	0	0	not significant
4912	WORSCHECH_TUMOR_EVASION_AND_TOLEROGENICITY_UP	-0.80	0.14	0	0	0	not significant
4913	CREIGHTON_AKT1_SIGNALING_VIA_MTOR_DN	-0.80	0.13	0	0	0	not significant
4914	GO_CELLULAR_RESPONSE_TO_EXOGENOUS_DSRNA	-0.80	0.13	0	0	0	not significant
4915	GO_HYALURONAN_CATABOLIC_PROCESS	-0.80	0.13	0	0	0	not significant
4916	GO_PROTON_TRANSPORTING_TWO_SECTOR_ATPASE_COMPLEX_CATALYTIC_DO	-0.80	0.13	0	0	0	not significant
4917	GO_REGULATION_OF_NEURON_MIGRATION	-0.80	0.13	0	0	0	not significant
4918	GO_REGULATION_OF_OSTEOCLAST_DEVELOPMENT	-0.80	0.13	0	0	0	not significant
4919	GO_UBIQUITIN_LIGASE_INHIBITOR_ACTIVITY	-0.80	0.13	0	0	0	not significant
4920	LEE_LIVER_CANCER	-0.80	0.13	0	0	0	not significant
4921	NIELSEN_LEIOMYOSARCOMA_DN	-0.80	0.13	0	0	0	not significant
4922	GO_DETECTION_OF_VISIBLE_LIGHT	-0.80	0.12	0	0	0	not significant
4923	GO_POSITIVE_REGULATION_OF_SYNAPSE_ASSEMBLY	-0.80	0.12	0	0	0	not significant
4924	GO_REGULATION_OF_RESPONSE_TO_EXTRACELLULAR_STIMULUS	-0.80	0.12	0	0	0	not significant
4925	PID_CIRCADIAN_PATHWAY	-0.80	0.12	0	0	0	not significant
4926	BIOCARTA_CHEMICAL_PATHWAY	-0.80	0.11	0	0	0	not significant
4927	GARGALOVIC_RESPONSE_TO_OXIDIZED_PHOSPHOLIPIDS_MAGENTA_UP	-0.80	0.11	0	0	0	not significant
4928	GARGALOVIC_RESPONSE_TO_OXIDIZED_PHOSPHOLIPIDS_RED_DN	-0.80	0.11	0	0	0	not significant
4929	GO_DEVELOPMENTAL_PIGMENTATION	-0.80	0.11	0	0	0	not significant
4930	GO_HISTONE_METHYLTRANSFERASE_ACTIVITY_H3_K4_SPECIFIC_	-0.80	0.11	0	0	0	not significant
4931	GO_REGULATION_OF_ANDROGEN_RECEPTOR_SIGNALING_PATHWAY	-0.80	0.11	0	0	0	not significant
4932	GO_CELLULAR_IRON_ION_HOMEOSTASIS	-0.80	0.1	0	0	0	not significant
4933	REACTOME_IRON_UPTAKE_AND_TRANSPORT	-0.80	0.1	0	0	0	not significant
4934	ROY_WOUND_BLOOD_VESSEL_UP	-0.80	0.1	0	0	0	not significant
4935	BOYLAN_MULTIPLE_MYELOMA_C_CLUSTER_UP	-0.80	0.09	0	0	0	not significant
4936	GO_POSITIVE_REGULATION_OF_MUSCLE_TISSUE_DEVELOPMENT	-0.80	0.09	0	0	0	not significant
4937	REACTOME_SUMOYLATION_OF_TRANSCRIPTION_COFACTORS	-0.80	0.09	0	0	0	not significant
4938	GO_CILIARY_TIP	-0.80	0.08	0	0	0	not significant
4939	GO_CRANIAL_SKELETAL_SYSTEM_DEVELOPMENT	-0.80	0.08	0	0	0	not significant
4940	KEGG_NON_SMALL_CELL_LUNG_CANCER	-0.80	0.08	0	0	0	not significant
4941	REACTOME_RET_SIGNALING	-0.80	0.08	0	0	0	not significant
4942	GO_REGULATION_OF_POSTSYNAPTIC_MEMBRANE_POTENTIAL	-0.80	0.07	0	0	0	not significant
4943	KANG_IMMORTALIZED_BY_TERT_UP	-0.80	0.07	0	0	0	not significant
4944	REACTOME_RETROGRADE_TRANSPORT_AT_THE_TRANS_GOLGI_NETWORK	-0.80	0.07	0	0	0	not significant
4945	PID_TCR_PATHWAY	-0.80	0.06	0	0	0	not significant
4946	REACTOME_COMPLEX_I_BIOGENESIS	-0.80	0.06	0	0	0	not significant
4947	GO_NADH_DEHYDROGENASE_COMPLEX_ASSEMBLY	-0.80	0.05	0	0	0	not significant
4948	GO_REGULATION_OF_ACTIN_FILAMENT_ORGANIZATION	-0.80	0.01	0	0	0	not significant
4949	GO_CILIARY_PART	-0.80	0	0	0	0	not significant
4950	GO_NEGATIVE_REGULATION_OF_CENTRIOLE_REPLICATION	-0.79	0.15	0	0	0	not significant
4951	GO_NEGATIVE_REGULATION_OF_MULTICELLULAR_ORGANISM_GROWTH	-0.79	0.15	0	0	0	not significant
4952	GO_REGULATION_OF_NEURON_MATURATION	-0.79	0.15	0	0	0	not significant
4953	REACTOME_GP1B_IX_V_ACTIVATION_SIGNALLING	-0.79	0.15	0	0	0	not significant
4954	ABDULRAHMAN_KIDNEY_CANCER_VHL_DN	-0.79	0.14	0	0	0	not significant
4955	CORRADETTI_MTOR_PATHWAY_REGULATORS_DN	-0.79	0.14	0	0	0	not significant
4956	GO_HEART_FIELD_SPECIFICATION	-0.79	0.14	0	0	0	not significant
4957	GO_NEGATIVE_REGULATION_OF_INTRACELLULAR_ESTROGEN_RECEPTOR_SIGNAI	-0.79	0.14	0	0	0	not significant
4958	KEGG_PRIMARY_BILE_ACID_BIOSYNTHESIS	-0.79	0.14	0	0	0	not significant
4959	PETRETTO_LEFT_VENTRICLE_MASS_QTL_CIS_DN	-0.79	0.14	0	0	0	not significant
4960	BARRIER_CANCER_RELAPSE_TUMOR_SAMPLE_DN	-0.79	0.13	0	0	0	not significant
4961	CHR13Q21	-0.79	0.13	0	0	0	not significant
4962	DING_LUNG_CANCER_MUTATED_FREQUENTLY	-0.79	0.13	0	0	0	not significant
4963	GO_PHOSPHATIDYLGLYCEROL_BIOSYNTHETIC_PROCESS	-0.79	0.13	0	0	0	not significant
4964	GO_PHOSPHATIDYLINOSITOL_5_PHOSPHATE_BINDING	-0.79	0.13	0	0	0	not significant
4965	GO_POSITIVE_REGULATION_OF_MACROPHAGE_ACTIVATION	-0.79	0.13	0	0	0	not significant
4966	GO_POSITIVE_REGULATION_OF_PROTEIN_DEPOLYMERIZATION	-0.79	0.13	0	0	0	not significant
4967	GO_PROTEIN_LOCALIZATION_TO_MEMBRANE_RAFT	-0.79	0.13	0	0	0	not significant
4968	GO_U4_SNRNA_3_END_PROCESSING	-0.79	0.13	0	0	0	not significant
4969	LI_WILMS_TUMOR_ANAPLASTIC_DN	-0.79	0.13	0	0	0	not significant
4970	MARKS_ACETYLATED_NON_HISTONE_PROTEINS	-0.79	0.13	0	0	0	not significant
4971	MURAKAMI_UV_RESPONSE_1HR_UP	-0.79	0.13	0	0	0	not significant
4972	REACTOME_ACTIVATION_OF_THE_TFAP2_AP_2_FAMILY_OF_TRANSCRIPTION_FAC	-0.79	0.13	0	0	0	not significant
4973	REACTOME_GAB1_SIGNALOSOME	-0.79	0.13	0	0	0	not significant
4974	REACTOME_PD_1_SIGNALING	-0.79	0.13	0	0	0	not significant
4975	DAWSON_METHYLATED_IN_LYMPHOMA_TCL1	-0.79	0.12	0	0	0	not significant
4976	GO_BRANCHING_INVOLVED_IN_BLOOD_VESSEL_MORPHOGENESIS	-0.79	0.12	0	0	0	not significant
4977	GO_CLATHRIN_COATED_ENDOCYTIC_VESICLE	-0.79	0.12	0	0	0	not significant
4978	GO_GRANULOCYTE_DIFFERENTIATION	-0.79	0.12	0	0	0	not significant
4979	GO_NATURAL_KILLER_CELL_DEGRANULATION	-0.79	0.12	0	0	0	not significant
4980	MOOTHA_GLYCOGEN_METABOLISM	-0.79	0.12	0	0	0	not significant
4981	PID_NETRIN_PATHWAY	-0.79	0.12	0	0	0	not significant
4982	REACTOME_INLA_MEDIATED_ENTRY_OF_LISTERIA_MONOCYTOGENES_INTO_HOST	-0.79	0.12	0	0	0	not significant
4983	REACTOME_LISTERIA_MONOCYTOGENES_ENTRY_INTO_HOST_CELLS	-0.79	0.12	0	0	0	not significant
4984	GO_REGULATION_OF_DNA_DAMAGE_RESPONSE_SIGNAL_TRANSDUCTION_BY_P53	-0.79	0.11	0	0	0	not significant
4985	GO_REGULATION_OF_NEUTROPHIL_MIGRATION	-0.79	0.11	0	0	0	not significant
4986	GO_REGULATION_OF_TRIGLYCERIDE_BIOSYNTHETIC_PROCESS	-0.79	0.11	0	0	0	not significant
4987	GO_SH2_DOMAIN_BINDING	-0.79	0.11	0	0	0	not significant
4988	PID_IL8_CXCR1_PATHWAY	-0.79	0.11	0	0	0	not significant
4989	GAZDA_DIAMOND_BLACKFAN_ANEMIA_MYELOID_UP	-0.79	0.1	0	0	0	not significant
4990	GO_N_TERMINAL_PROTEIN_AMINO_ACID_MODIFICATION	-0.79	0.09	0	0	0	not significant
4991	GO_POSITIVE_REGULATION_OF_INTRINSIC_APOPTOTIC_SIGNALING_PATHWAY	-0.79	0.09	0	0	0	not significant
4992	GO_AMINOPEPTIDASE_ACTIVITY	-0.79	0.08	0	0	0	not significant
4993	ZHU_CMV_24_HR_DN	-0.79	0.08	0	0	0	not significant
4994	GO_CAMERA_TYPE_EYE_MORPHOGENESIS	-0.79	0.07	0	0	0	not significant
4995	GO_RESPONSE_TO_ACTIVITY	-0.79	0.07	0	0	0	not significant
4996	KONDO_PROSTATE_CANCER_HCP_WITH_H3K27ME3	-0.79	0.07	0	0	0	not significant
4997	PID_VEGFR1_2_PATHWAY	-0.79	0.07	0	0	0	not significant
4998	REACTOME_CA2PLUS_PATHWAY	-0.79	0.07	0	0	0	not significant
4999	REACTOME_CARGO_TRAFFICKING_TO_THE_PERICILIARY_MEMBRANE	-0.79	0.07	0	0	0	not significant
5000	REACTOME_ERCC6_CSB_AND_EHMT2_G9A_POSITIVELY_REGULATE_RRNA_EXPRE	-0.79	0.07	0	0	0	not significant
5001	GO_BETA_CATENIN_BINDING	-0.79	0.06	0	0	0	not significant
5002	GO_POLYOL_METABOLIC_PROCESS	-0.79	0.06	0	0	0	not significant

5003	GO_REGULATION_OF_SMOOTHENED_SIGNALING_PATHWAY	-0.79	0.06	0	0	0	not significant
5004	GO_TIGHT_JUNCTION	-0.79	0.05	0	0	0	not significant
5005	GO_VESICLE_MEDIATED_TRANSPORT_TO_THE_PLASMA_MEMBRANE	-0.79	0.04	0	0	0	not significant
5006	GO_SKELETAL_SYSTEM_MORPHOGENESIS	-0.79	0.03	0	0	0	not significant
5007	GO_RESPONSE_TO_DEFENSES_OF_OTHER_ORGANISM_INVOLVED_IN_SYMBIOTIC	-0.78	0.15	0	0	0	not significant
5008	GO_NEGATIVE_REGULATION_OF_DENDRITE_MORPHOGENESIS	-0.78	0.14	0	0	0	not significant
5009	BERENJENO_TRANSFORMED_BY_RHOA_REVERSIBLY_UP	-0.78	0.13	0	0	0	not significant
5010	BIOCARTA_ECM_PATHWAY	-0.78	0.13	0	0	0	not significant
5011	BIOCARTA_UCALPAIN_PATHWAY	-0.78	0.13	0	0	0	not significant
5012	DING_LUNG_CANCER_MUTATED_RECURRENTLY	-0.78	0.13	0	0	0	not significant
5013	GO_DENDRITIC_CELL_CYTOKINE_PRODUCTION	-0.78	0.13	0	0	0	not significant
5014	GO_G_PROTEIN_COUPLED_RECEPTOR_INTERNALIZATION	-0.78	0.13	0	0	0	not significant
5015	GO_HYALURONAN_BIOSYNTHETIC_PROCESS	-0.78	0.13	0	0	0	not significant
5016	GO_L_METHIONINE_SALVAGE_FROM_METHYLTHIOADENOSINE	-0.78	0.13	0	0	0	not significant
5017	GO_MALE_GERM_CELL_NUCLEUS	-0.78	0.13	0	0	0	not significant
5018	GO_NATURAL_KILLER_CELL_ACTIVATION_INVOLVED_IN_IMMUNE_RESPONSE	-0.78	0.13	0	0	0	not significant
5019	GO_NEGATIVE_REGULATION_OF_SODIUM_ION_TRANSPORT	-0.78	0.13	0	0	0	not significant
5020	GO_PEPTIDYL_PROLINE_DIOXYGENASE_ACTIVITY	-0.78	0.13	0	0	0	not significant
5021	GO_REGULATION_OF_EPITHELIAL_CELL_DIFFERENTIATION_INVOLVED_IN_KIDNEY_	-0.78	0.13	0	0	0	not significant
5022	GO_RNAI_EFFECTOR_COMPLEX	-0.78	0.13	0	0	0	not significant
5023	GO_SECONDARY_HEART_FIELD_SPECIFICATION	-0.78	0.13	0	0	0	not significant
5024	KONDO_HYPOXIA	-0.78	0.13	0	0	0	not significant
5025	REACTOME_ALPHA_OXIDATION_OF_PHYTANATE	-0.78	0.13	0	0	0	not significant
5026	REACTOME_GLYCOGEN_STORAGE_DISEASES	-0.78	0.13	0	0	0	not significant
5027	REACTOME_IONOTROPIC_ACTIVITY_OF_KAINATE_RECEPTORS	-0.78	0.13	0	0	0	not significant
5028	GO_CYTIDINE_TO_URIDINE_EDITING	-0.78	0.12	0	0	0	not significant
5029	GO_EIF2ALPHA_PHOSPHORYLATION_IN_RESPONSE_TO_ENDOPLASMIC_RETICULUM	-0.78	0.12	0	0	0	not significant
5030	GO_MITOCHONDRIAL_FRAGMENTATION_INVOLVED_IN_APOPTOTIC_PROCESS	-0.78	0.12	0	0	0	not significant
5031	GO_NEGATIVE_REGULATION_OF_SMOOTH_MUSCLE_CELL_MIGRATION	-0.78	0.12	0	0	0	not significant
5032	GO_NUCLEAR_TRANSCRIBED_MRNA_CATABOLIC_PROCESS_EXONUCLEOLYTIC_3_	-0.78	0.12	0	0	0	not significant
5033	GO_REGULATION_OF_PROTEIN_DEUBIQUITINATION	-0.78	0.12	0	0	0	not significant
5034	GO_SEQUESTERING_OF_METAL_ION	-0.78	0.12	0	0	0	not significant
5035	GO_SMOOTH_MUSCLE_TISSUE_DEVELOPMENT	-0.78	0.12	0	0	0	not significant
5036	GO_UBIQUITINATION_LIKE_MODIFICATION_DEPENDENT_PROTEIN_BINDING	-0.78	0.12	0	0	0	not significant
5037	REACTOME_SUMOYLATION_OF_DNA_METHYLATION_PROTEINS	-0.78	0.12	0	0	0	not significant
5038	REACTOME_TRAF6_MEDIATED_INDUCTION_OF_TAK1_COMPLEX_WITHIN_TLR4_CO	-0.78	0.12	0	0	0	not significant
5039	GO_THREONINE_TYPE_PEPTIDASE_ACTIVITY	-0.78	0.11	0	0	0	not significant
5040	WINNEPENNINCKX_MELANOMA_METASTASIS_DN	-0.78	0.11	0	0	0	not significant
5041	GO_BLASTOCYST_GROWTH	-0.78	0.1	0	0	0	not significant
5042	PID_HDAC_CLASSIII_PATHWAY	-0.78	0.1	0	0	0	not significant
5043	SATO_SILENCED_BY_DEACETYLATION_IN_PANCREATIC_CANCER	-0.78	0.1	0	0	0	not significant
5044	GO_ANIMAL_ORGAN_FORMATION	-0.78	0.09	0	0	0	not significant
5045	GO_AORTA_DEVELOPMENT	-0.78	0.09	0	0	0	not significant
5046	GO_BETA_TUBULIN_BINDING	-0.78	0.09	0	0	0	not significant
5047	REACTOME_MTORC1_MEDIATED_SIGNALLING	-0.78	0.08	0	0	0	not significant
5048	GO_PDZ_DOMAIN_BINDING	-0.78	0.07	0	0	0	not significant
5049	GO_NEURON_SPINE	-0.78	0.05	0	0	0	not significant
5050	GO_REGULATION_OF_ACTOMYOSIN_STRUCTURE_ORGANIZATION	-0.78	0.05	0	0	0	not significant
5051	GO_REGULATION_OF_G_PROTEIN_COUPLED_RECEPTOR_SIGNALING_PATHWAY	-0.78	0.05	0	0	0	not significant
5052	BIOCARTA_GABA_PATHWAY	-0.77	0.13	0	0	0	not significant
5053	BIOCARTA_TRKA_PATHWAY	-0.77	0.12	0	0	0	not significant
5054	GO_CHEMICAL_HOMEOSTASIS_WITHIN_A_TISSUE	-0.77	0.12	0	0	0	not significant
5055	GO_MEMBRANE_DEPOLARIZATION_DURING_ACTION_POTENTIAL	-0.77	0.12	0	0	0	not significant
5056	GO_NEGATIVE_REGULATION_OF_NUCLEOCYTOPLASMIC_TRANSPORT	-0.77	0.12	0	0	0	not significant
5057	GO_OSTEOCLAST_DEVELOPMENT	-0.77	0.12	0	0	0	not significant
5058	GO_POSITIVE_REGULATION_OF_CELL_PROLIFERATION_INVOLVED_IN_HEART_MOF	-0.77	0.12	0	0	0	not significant
5059	GO_REGULATION_OF_CELLULAR_CARBOHYDRATE_CATABOLIC_PROCESS	-0.77	0.12	0	0	0	not significant
5060	GO_REGULATION_OF_LAMELLIPODIUM_MORPHOGENESIS	-0.77	0.12	0	0	0	not significant
5061	GO_REGULATION_OF_MEMBRANE_TUBULATION	-0.77	0.12	0	0	0	not significant
5062	GO_UBIQUITIN_PROTEIN_TRANSFERASE_INHIBITOR_ACTIVITY	-0.77	0.12	0	0	0	not significant
5063	REACTOME_ACTIVATION_OF_KAINATE_RECEPTORS_UPON_GLUTAMATE_BINDING	-0.77	0.12	0	0	0	not significant
5064	REACTOME_GLUCURONIDATION	-0.77	0.12	0	0	0	not significant
5065	REACTOME_P130CAS_LINKAGE_TO_MAPK_SIGNALING_FOR_INTEGRINS	-0.77	0.12	0	0	0	not significant
5066	REACTOME_REGULATION_OF_GENE_EXPRESSION_BY_HYPOXIA_INDUCIBLE_FACTO	-0.77	0.12	0	0	0	not significant
5067	BIOCARTA_RELA_PATHWAY	-0.77	0.11	0	0	0	not significant
5068	BIOCARTA_TERT_PATHWAY	-0.77	0.11	0	0	0	not significant
5069	GO_CATECHOLAMINE_METABOLIC_PROCESS	-0.77	0.11	0	0	0	not significant
5070	GO_CYTOPLASMIC_UBIQUITIN_LIGASE_COMPLEX	-0.77	0.11	0	0	0	not significant
5071	GO_DNA_LIGATION	-0.77	0.11	0	0	0	not significant
5072	GO_ERROR_FREE_TRANSLESION_SYNTHESIS	-0.77	0.11	0	0	0	not significant
5073	GO_ISWI_TYPE_COMPLEX	-0.77	0.11	0	0	0	not significant
5074	GO_NEGATIVE_REGULATION_OF_B_CELL_RECEPTOR_SIGNALING_PATHWAY	-0.77	0.11	0	0	0	not significant
5075	GO_PERIPHERAL_NERVOUS_SYSTEM_NEURON_DIFFERENTIATION	-0.77	0.11	0	0	0	not significant
5076	GO_POSITIVE_REGULATION_OF_VASCULAR_ASSOCIATED_SMOOTH_MUSCLE_CELI	-0.77	0.11	0	0	0	not significant
5077	GO_REGULATION_OF_GONAD_DEVELOPMENT	-0.77	0.11	0	0	0	not significant
5078	GO_STRUCTURAL_MOLECULE_ACTIVITY_CONFERRING_ELASTICITY	-0.77	0.11	0	0	0	not significant
5079	MIKKELSEN_PLURIPOTENT_STATE_DN	-0.77	0.11	0	0	0	not significant
5080	PID_ANTHRAX_PATHWAY	-0.77	0.11	0	0	0	not significant
5081	GO_NEGATIVE_REGULATION_OF_MUSCLE_CELL_APOPTOTIC_PROCESS	-0.77	0.1	0	0	0	not significant
5082	GO_NEGATIVE_REGULATION_OF_REACTIVE_OXYGEN_SPECIES_METABOLIC_PROC	-0.77	0.1	0	0	0	not significant
5083	GO_SOLUTE_CATION_ANTIPORTER_ACTIVITY	-0.77	0.1	0	0	0	not significant
5084	REACTOME_TRANSLESION_SYNTHESIS_BY_POLH	-0.77	0.1	0	0	0	not significant
5085	CHEOK_RESPONSE_TO_MERCAPTOPURINE_AND_HD_MTX_DN	-0.77	0.09	0	0	0	not significant
5086	CHR9Q32	-0.77	0.09	0	0	0	not significant
5087	GO_MRNA_SPLICE_SITE_SELECTION	-0.77	0.09	0	0	0	not significant
5088	GO_POSITIVE_REGULATION_OF_CILIUM_ASSEMBLY	-0.77	0.09	0	0	0	not significant
5089	REACTOME_NEPHRIN_FAMILY_INTERACTIONS	-0.77	0.09	0	0	0	not significant
5090	BIOCARTA_STRESS_PATHWAY	-0.77	0.08	0	0	0	not significant
5091	GO_REGULATION_OF_CILIUM_ASSEMBLY	-0.77	0.07	0	0	0	not significant
5092	GO_INORGANIC_ION_IMPORT_ACROSS_PLASMA_MEMBRANE	-0.77	0.06	0	0	0	not significant
5093	GO_REGULATION_OF_RESPONSE_TO_DRUG	-0.77	0.06	0	0	0	not significant
5094	GO_MORPHOGENESIS_OF_EMBRYONIC_EPITHELIUM	-0.77	0.04	0	0	0	not significant
5095	JAZAG_TGFB1_SIGNALING_VIA_SMAD4_UP	-0.77	0.04	0	0	0	not significant
5096	GO_CENTRAL_NERVOUS_SYSTEM_NEURON_DIFFERENTIATION	-0.77	0.03	0	0	0	not significant
5097	SIRNA_EIF4GI_DN	-0.77	0.03	0	0	0	not significant
5098	GO_REGULATION_OF_HYALURONAN_BIOSYNTHETIC_PROCESS	-0.76	0.13	0	0	0	not significant
5099	INAMURA_LUNG_CANCER_SCC_SUBTYPES_UP	-0.76	0.13	0	0	0	not significant
5100	GO_CONDENSIN_COMPLEX	-0.76	0.12	0	0	0	not significant
5101	GO_MRNA_3_END_PROCESSING_BY_STEM_LOOP_BINDING_AND_CLEAVAGE	-0.76	0.12	0	0	0	not significant
5102	GO_NEGATIVE_REGULATION_OF_ATPASE_ACTIVITY	-0.76	0.12	0	0	0	not significant
5103	GO_NEGATIVE_REGULATION_OF_VIRAL_ENTRY_INTO_HOST_CELL	-0.76	0.12	0	0	0	not significant
5104	GO_PHOSPHOFRUCTOKINASE_ACTIVITY	-0.76	0.12	0	0	0	not significant
5105	REACTOME_ACTIVATION_OF_RAC1	-0.76	0.12	0	0	0	not significant
5106	REACTOME_NEF_MEDIATED_CD8_DOWN_REGULATION	-0.76	0.12	0	0	0	not significant
5107	ZEMBUTSU_SENSITIVITY_TO_CISPLATIN	-0.76	0.12	0	0	0	not significant
5108	BIOCARTA_PCAF_PATHWAY	-0.76	0.11	0	0	0	not significant
5109	GO_HEPATOCYTE_APOPTOTIC_PROCESS	-0.76	0.11	0	0	0	not significant
5110	GO_NUCLEAR_RETENTION_OF_PRE_MRNA_AT_THE_SITE_OF_TRANSCRIPTION	-0.76	0.11	0	0	0	not significant
5111	GO_REFLEX	-0.76	0.11	0	0	0	not significant

5112	GO_SMALL_RIBOSOMAL_SUBUNIT_RRNA_BINDING	-0.76	0.11	0	0	0	not significant
5113	BIOCARTA_PKC_PATHWAY	-0.76	0.1	0	0	0	not significant
5114	GO_CYTOCHROME_B5_REDUCTASE_ACTIVITY_ACTING_ON_NAD_P_H	-0.76	0.1	0	0	0	not significant
5115	GO_EPIBOLY	-0.76	0.1	0	0	0	not significant
5116	GO_POSITIVE_REGULATION_OF_LIPID_CATABOLIC_PROCESS	-0.76	0.1	0	0	0	not significant
5117	GO_POSTSYNAPTIC_SPECIALIZATION_ASSEMBLY	-0.76	0.1	0	0	0	not significant
5118	GO_REGULATION_OF_NUCLEAR_TRANSCRIBED_MRNA_CATABOLIC_PROCESS_DE/	-0.76	0.1	0	0	0	not significant
5119	GO_RRNA_BASE_METHYLATION	-0.76	0.1	0	0	0	not significant
5120	REACTOME_SYNTHESIS_OF_DIPHTHAMIDE_EEF2	-0.76	0.1	0	0	0	not significant
5121	WIKMAN_ASBESTOS_LUNG_CANCER_UP	-0.76	0.1	0	0	0	not significant
5122	GO_PLATELET_DENSE_GRANULE	-0.76	0.09	0	0	0	not significant
5123	REACTOME_GABA_RECEPTOR_ACTIVATION	-0.76	0.09	0	0	0	not significant
5124	GO_PHOTOTRANSDUCTION	-0.76	0.07	0	0	0	not significant
5125	GO_REGULATION_OF_RECEPTOR_INTERNALIZATION	-0.76	0.07	0	0	0	not significant
5126	EPPERT_LSC_R	-0.76	0.06	0	0	0	not significant
5127	GO_MICROBODY_LUMEN	-0.76	0.06	0	0	0	not significant
5128	GO_RESPONSE_TO_DSRNA	-0.76	0.06	0	0	0	not significant
5129	GO_VESICLE_DOCKING_INVOLVED_IN_EXOCYTOSIS	-0.76	0.06	0	0	0	not significant
5130	GO_POSITIVE_REGULATION_OF_ACTIN_FILAMENT_BUNDLE_ASSEMBLY	-0.76	0.05	0	0	0	not significant
5131	GO_POSITIVE_REGULATION_OF_STRESS_FIBER_ASSEMBLY	-0.76	0.05	0	0	0	not significant
5132	GO_REGULATION_OF_CARDIAC_MUSCLE_TISSUE_DEVELOPMENT	-0.76	0.05	0	0	0	not significant
5133	GO_MITOCHONDRIAL_ELECTRON_TRANSPORT_NADH_TO_UBIQUINONE	-0.76	0.04	0	0	0	not significant
5134	GO_POSITIVE_REGULATION_OF_ENDOCYTOSIS	-0.76	0.04	0	0	0	not significant
5135	REACTOME_E3_UBIQUITIN_LIGASES_UBIQUITINATE_TARGET_PROTEINS	-0.76	0.04	0	0	0	not significant
5136	REACTOME_P75_NTR_RECEPTOR_MEDIATED_SIGNALLING	-0.76	0.03	0	0	0	not significant
5137	GO_RESPIRASOME	-0.76	0.02	0	0	0	not significant
5138	GO_SMALL_RIBOSOMAL_SUBUNIT	-0.76	0.02	0	0	0	not significant
5139	REACTOME_RESPIRATORY_ELECTRON_TRANSPORT_ATP_SYNTHESIS_BY_CHEMIC	-0.76	0.02	0	0	0	not significant
5140	GO_GAMMA_AMINOBUTYRIC_ACID_TRANSPORT	-0.75	0.12	0	0	0	not significant
5141	GO_TUBULIN_COMPLEX_ASSEMBLY	-0.75	0.12	0	0	0	not significant
5142	GO_REGULATION_OF_ASPARTIC_TYPE_ENDOPEPTIDASE_ACTIVITY_INVOLVED_IN	-0.75	0.11	0	0	0	not significant
5143	GO_REGULATION_OF_ER_ASSOCIATED_UBIQUITIN_DEPENDENT_PROTEIN_CATABC	-0.75	0.11	0	0	0	not significant
5144	GO_REGULATION_OF_PROTEIN_FOLDING	-0.75	0.11	0	0	0	not significant
5145	GO_REGULATION_OF_RESPIRATORY_GASEOUS_EXCHANGE	-0.75	0.11	0	0	0	not significant
5146	GO_SOMATIC_DIVERSIFICATION_OF_T_CELL_RECEPTOR_GENES	-0.75	0.11	0	0	0	not significant
5147	MIKKELSEN_IPS_ICP_WITH_H3K27ME3	-0.75	0.11	0	0	0	not significant
5148	WILLIAMS_ESR2_TARGETS_DN	-0.75	0.11	0	0	0	not significant
5149	BIOCARTA_PS1_PATHWAY	-0.75	0.1	0	0	0	not significant
5150	CHR4Q23	-0.75	0.1	0	0	0	not significant
5151	CHRXQ23	-0.75	0.1	0	0	0	not significant
5152	GO_AEROBIC_ELECTRON_TRANSPORT_CHAIN	-0.75	0.1	0	0	0	not significant
5153	GO_ESTABLISHMENT_OR_MAINTENANCE_OF_ACTIN_CYTOSKELETON_POLARITY	-0.75	0.1	0	0	0	not significant
5154	GO_FOREBRAIN_NEURON_DIFFERENTIATION	-0.75	0.1	0	0	0	not significant
5155	GO_MITOTIC_NUCLEAR_ENVELOPE_REASSEMBLY	-0.75	0.1	0	0	0	not significant
5156	REACTOME_MYOCLONIC_EPILEPSY_OF_LAFORA	-0.75	0.1	0	0	0	not significant
5157	REACTOME_REGULATION_OF_KIT_SIGNALING	-0.75	0.1	0	0	0	not significant
5158	REACTOME_STING_MEDIATED_INDUCTION_OF_HOST_IMMUNE_RESPONSES	-0.75	0.1	0	0	0	not significant
5159	REACTOME_TRANSPORT_OF_NUCLEOTIDE_SUGARS	-0.75	0.1	0	0	0	not significant
5160	WEINMANN_ADAPTATION_TO_HYPOXIA_UP	-0.75	0.1	0	0	0	not significant
5161	GO_CELL_PROLIFERATION_INVOLVED_IN_HEART_MORPHOGENESIS	-0.75	0.09	0	0	0	not significant
5162	GO_ENDOPLASMIC_RETICULUM_TUBULAR_NETWORK_ORGANIZATION	-0.75	0.09	0	0	0	not significant
5163	GO_GASTRULATION_WITH_MOUTH_FORMING_SECOND	-0.75	0.09	0	0	0	not significant
5164	GO_OXIDOREDUCTASE_ACTIVITY_ACTING_ON_A_HEME_GROUP_OF_DONORS	-0.75	0.09	0	0	0	not significant
5165	GO_SECRETORY_GRANULE_ORGANIZATION	-0.75	0.09	0	0	0	not significant
5166	PARK_HSC_VS_MULTIPOTENT_PROGENITORS_UP	-0.75	0.09	0	0	0	not significant
5167	GO_POSITIVE_REGULATION_OF_PROTEIN_AUTOPHOSPHORYLATION	-0.75	0.08	0	0	0	not significant
5168	KIM_ALL_DISORDERS_OLIGODENDROCYTE_NUMBER_CORR_DN	-0.75	0.08	0	0	0	not significant
5169	GO_RAC_PROTEIN_SIGNAL_TRANSDUCTION	-0.75	0.07	0	0	0	not significant
5170	GO_DETERMINATION_OF_HEART_LEFT_RIGHT_ASYMMETRY	-0.75	0.06	0	0	0	not significant
5171	GO_METALLOENDOPEPTIDASE_ACTIVITY	-0.75	0.06	0	0	0	not significant
5172	GO_REGULATION_OF_DEFENSE_RESPONSE_TO_VIRUS_BY_VIRUS	-0.75	0.06	0	0	0	not significant
5173	GO_RNA_POLYMERASE_II_TRANSCRIPTION_FACTOR_BINDING	-0.75	0.06	0	0	0	not significant
5174	REACTOME_MTOR_SIGNALLING	-0.75	0.06	0	0	0	not significant
5175	FAELT_B_CLL_WITH_VH_REARRANGEMENTS_DN	-0.75	0.05	0	0	0	not significant
5176	GO_APICAL_JUNCTION_ASSEMBLY	-0.75	0.05	0	0	0	not significant
5177	GO_EMBRYONIC_HEART_TUBE_DEVELOPMENT	-0.75	0.05	0	0	0	not significant
5178	REACTOME_ENDOSOMAL_SORTING_COMPLEX_REQUIRED_FOR_TRANSPORT_ESC	-0.75	0.05	0	0	0	not significant
5179	GO_ACTOMYOSIN	-0.75	0.03	0	0	0	not significant
5180	REACTOME_RAB_GEFS_EXCHANGE_GTP_FOR_GDP_ON_RABS	-0.75	0.03	0	0	0	not significant
5181	KEGG_PARKINSONS_DISEASE	-0.75	0.02	0	0	0	not significant
5182	GO_GAMMA_AMINOBUTYRIC_ACID_SECRETION	-0.74	0.12	0	0	0	not significant
5183	GO_NEGATIVE_REGULATION_OF_RECEPTOR_BINDING	-0.74	0.12	0	0	0	not significant
5184	BAFNA_MUC4_TARGETS_UP	-0.74	0.11	0	0	0	not significant
5185	BIOCARTA_HSWI_SNF_PATHWAY	-0.74	0.1	0	0	0	not significant
5186	FIGUEROA_AML_METHYLATION_CLUSTER_5_UP	-0.74	0.1	0	0	0	not significant
5187	GO_EOSINOPHIL_ACTIVATION	-0.74	0.1	0	0	0	not significant
5188	GO_KERATINIZATION	-0.74	0.1	0	0	0	not significant
5189	GO_MAINTENANCE_OF_SYNAPSE_STRUCTURE	-0.74	0.1	0	0	0	not significant
5190	GO_MEDIUM_CHAIN_FATTY_ACID_COA_LIGASE_ACTIVITY	-0.74	0.1	0	0	0	not significant
5191	GO_POSITIVE_REGULATION_OF_DEOXYRIBONUCLEASE_ACTIVITY	-0.74	0.1	0	0	0	not significant
5192	GO_PROTEIN_LOCALIZATION_TO_BASOLATERAL_PLASMA_MEMBRANE	-0.74	0.1	0	0	0	not significant
5193	GO_TRANSMEMBRANE_RECEPTOR_PROTEIN_SERINE_THREONINE_KINASE_ACTIVIT	-0.74	0.1	0	0	0	not significant
5194	HOLLERN_ADENOMYOEPITHELIAL_BREAST_TUMOR	-0.74	0.1	0	0	0	not significant
5195	MIKHAYLOVA_OXIDATIVE_STRESS_RESPONSE_VIA_VHL_DN	-0.74	0.1	0	0	0	not significant
5196	REACTOME_ARYL_HYDROCARBON_RECEPTOR_SIGNALLING	-0.74	0.1	0	0	0	not significant
5197	CHR14Q13	-0.74	0.09	0	0	0	not significant
5198	GO_REGULATION_OF_AMINO_ACID_TRANSPORT	-0.74	0.09	0	0	0	not significant
5199	GO_TITIN_BINDING	-0.74	0.09	0	0	0	not significant
5200	MYLLYKANGAS_AMPLIFICATION_HOT_SPOT_15	-0.74	0.09	0	0	0	not significant
5201	GO_COPI_VESICLE_COAT	-0.74	0.08	0	0	0	not significant
5202	GO_PHOTOTRANSDUCTION_VISIBLE_LIGHT	-0.74	0.08	0	0	0	not significant
5203	GO_POSITIVE_REGULATION_OF_CALCIUM_ION_TRANSPORT_INTO_CYTOSOL	-0.74	0.08	0	0	0	not significant
5204	GO_POSITIVE_REGULATION_OF_RELEASE_OF_SEQUESTERED_CALCIUM_ION_INTC	-0.74	0.08	0	0	0	not significant
5205	NIKOLSKY_BREAST_CANCER_5P15_AMPLICON	-0.74	0.08	0	0	0	not significant
5206	REACTOME_RAF_INDEPENDENT_MAPK1_3_ACTIVATION	-0.74	0.08	0	0	0	not significant
5207	REACTOME_SIGNALING_BY_NTRK3_TRKC	-0.74	0.08	0	0	0	not significant
5208	ZHAN_MULTIPLE_MYELOMA_MF_UP	-0.74	0.08	0	0	0	not significant
5209	CHR5P13	-0.74	0.07	0	0	0	not significant
5210	GO_INOSITOL_PHOSPHATE_CATABOLIC_PROCESS	-0.74	0.07	0	0	0	not significant
5211	GO_KERATINOCYTE_PROLIFERATION	-0.74	0.07	0	0	0	not significant
5212	GO_PODOSOME	-0.74	0.07	0	0	0	not significant
5213	GO_ARP2_3_COMPLEX_MEDIATED_ACTIN_NUCLEATION	-0.74	0.06	0	0	0	not significant
5214	GO_CARBOHYDRATE_PHOSPHORYLATION	-0.74	0.06	0	0	0	not significant
5215	NAKAMURA_METASTASIS_MODEL_UP	-0.74	0.06	0	0	0	not significant
5216	BURTON_ADIPOGENESIS_7	-0.74	0.05	0	0	0	not significant
5217	GO_CELL_CELL_CONTACT_ZONE	-0.74	0.04	0	0	0	not significant
5218	JOHNSTONE_PARVB_TARGETS_1_DN	-0.74	0.04	0	0	0	not significant
5219	GO_SITE_OF_DOUBLE_STRAND_BREAK	-0.74	0.03	0	0	0	not significant
5220	GO_ARYL_HYDROCARBON_RECEPTOR_BINDING	-0.73	0.11	0	0	0	not significant

5221	GO_ENDOCARDIUM_DEVELOPMENT	-0.73	0.11	0	0	0	not significant
5222	GO_RESPONSE_TO_MACROPHAGE_COLONY_STIMULATING_FACTOR	-0.73	0.11	0	0	0	not significant
5223	GARGALOVIC_RESPONSE_TO_OXIDIZED_PHOSPHOLIPIDS_BLACK_DN	-0.73	0.1	0	0	0	not significant
5224	GO_SODIUM_ION_IMPORT_ACROSS_PLASMA_MEMBRANE	-0.73	0.1	0	0	0	not significant
5225	MYLLYKANGAS_AMPLIFICATION_HOT_SPOT_25	-0.73	0.1	0	0	0	not significant
5226	REACTOME_IRF3_MEDIATED_INDUCTION_OF_TYPE_I_IFN	-0.73	0.1	0	0	0	not significant
5227	RIZ_ERYTHROID_DIFFERENTIATION_6HR	-0.73	0.1	0	0	0	not significant
5228	GO_ANATOMICAL_STRUCTURE_REGRESSION	-0.73	0.09	0	0	0	not significant
5229	GO_COMPLEMENT_BINDING	-0.73	0.09	0	0	0	not significant
5230	GO_CYTOPLASMIC_SEQUESTERING_OF_NF_KAPPAB	-0.73	0.09	0	0	0	not significant
5231	GO_DOPAMINERGIC_NEURON_DIFFERENTIATION	-0.73	0.09	0	0	0	not significant
5232	GO_EPITHELIAL_TO_MESENCHYMAL_TRANSITION_INVOLVED_IN_ENDOCARDIAL_CU	-0.73	0.09	0	0	0	not significant
5233	GO_NEGATIVE_REGULATION_OF_GLUCOSE_IMPORT	-0.73	0.09	0	0	0	not significant
5234	GO_POSITIVE_REGULATION_OF_GRANULOCYTE_MACROPHAGE_COLONY_STIMULA	-0.73	0.09	0	0	0	not significant
5235	GO_REGULATION_OF_RAC_PROTEIN_SIGNAL_TRANSDUCTION	-0.73	0.09	0	0	0	not significant
5236	GO_UV_DAMAGE_EXCISION_REPAIR	-0.73	0.09	0	0	0	not significant
5237	NEBEN_AML_WITH_FLT3_OR_NRAS_DN	-0.73	0.09	0	0	0	not significant
5238	GO_AMINO_SUGAR_METABOLIC_PROCESS	-0.73	0.08	0	0	0	not significant
5239	GO_DEATH_RECEPTOR_BINDING	-0.73	0.08	0	0	0	not significant
5240	GO_VOLTAGE_GATED_CALCIUM_CHANNEL_COMPLEX	-0.73	0.08	0	0	0	not significant
5241	CHR21Q21	-0.73	0.07	0	0	0	not significant
5242	GO_H4_HISTONE_ACETYLTRANSFERASE_ACTIVITY	-0.73	0.07	0	0	0	not significant
5243	GO_WW_DOMAIN_BINDING	-0.73	0.07	0	0	0	not significant
5244	REACTOME_GLYOXYLATE_METABOLISM_AND_GLYCINE_DEGRADATION	-0.73	0.07	0	0	0	not significant
5245	REACTOME_METAL_ION_SLC_TRANSPORTERS	-0.73	0.07	0	0	0	not significant
5246	GO_EXOSOME_RNASE_COMPLEX_	-0.73	0.06	0	0	0	not significant
5247	GO_PROTEIN_TRANSPORT_WITHIN_LIPID_BILAYER	-0.73	0.06	0	0	0	not significant
5248	GO_REGULATION_OF_ACTIN_CYTOSKELETON_REORGANIZATION	-0.73	0.06	0	0	0	not significant
5249	GO_REGULATION_OF_COAGULATION	-0.73	0.05	0	0	0	not significant
5250	GO_REGULATION_OF_TRANSCRIPTION_REGULATORY_REGION_DNA_BINDING	-0.73	0.05	0	0	0	not significant
5251	GO_RUFFLE_ASSEMBLY	-0.73	0.05	0	0	0	not significant
5252	CHR14Q23	-0.73	0.04	0	0	0	not significant
5253	NIKOLSKY_BREAST_CANCER_8P12_P11_AMPLICON	-0.73	0.04	0	0	0	not significant
5254	REACTOME_FORMATION_OF_INCISION_COMPLEX_IN_GG_NER	-0.73	0.04	0	0	0	not significant
5255	GARGALOVIC_RESPONSE_TO_OXIDIZED_PHOSPHOLIPIDS_BLUE_DN	-0.73	0.03	0	0	0	not significant
5256	GO_NEUROMUSCULAR_PROCESS	-0.73	0.03	0	0	0	not significant
5257	REACTOME_CELL_DEATH_SIGNALLING_VIA_NRAGE_NRIF_AND_NADE	-0.73	0.03	0	0	0	not significant
5258	REACTOME_PEROXISOMAL_PROTEIN_IMPORT	-0.73	0.02	0	0	0	not significant
5259	GO_APICAL_JUNCTION_COMPLEX	-0.73	0.01	0	0	0	not significant
5260	GO_RUFFLE	-0.73	0.01	0	0	0	not significant
5261	GO_SYNAPTIC_VESICLE_LOCALIZATION	-0.73	0.01	0	0	0	not significant
5262	GO_MODULATION_OF_AGE_RELATED_BEHAVIORAL_DECLINE	-0.72	0.1	0	0	0	not significant
5263	GO_NEGATIVE_REGULATION_OF_AMINE_TRANSPORT	-0.72	0.1	0	0	0	not significant
5264	GO_POSITIVE_REGULATION_OF_CELL_SIZE	-0.72	0.1	0	0	0	not significant
5265	GO_REGULATION_OF_CLATHRIN_DEPENDENT_ENDOCYTOSIS	-0.72	0.1	0	0	0	not significant
5266	ITO_PTTG1_TARGETS_UP	-0.72	0.1	0	0	0	not significant
5267	REACTOME_LIPOPHAGY	-0.72	0.1	0	0	0	not significant
5268	GO_ACTIVATION_OF_JNKK_ACTIVITY	-0.72	0.09	0	0	0	not significant
5269	GO_CELLULAR_ANION_HOMEOSTASIS	-0.72	0.09	0	0	0	not significant
5270	GO_CENTRIOLE_CENTRIOLE_COHESION	-0.72	0.09	0	0	0	not significant
5271	GO_EMBRYONIC_FORELIMB_MORPHOGENESIS	-0.72	0.09	0	0	0	not significant
5272	GO_ESTABLISHMENT_OF_VIRAL_LATENCY	-0.72	0.09	0	0	0	not significant
5273	GO_MITOCHONDRIAL_RESPIRATORY_CHAIN_COMPLEX_IV	-0.72	0.09	0	0	0	not significant
5274	GO_PROTEIN_INSERTION_INTO_MITOCHONDRIAL_INNER_MEMBRANE	-0.72	0.09	0	0	0	not significant
5275	GO_TYPE_B_PANCREATIC_CELL_APOPTOTIC_PROCESS	-0.72	0.09	0	0	0	not significant
5276	KUMAMOTO_RESPONSE_TO_NUTLIN_3A_UP	-0.72	0.09	0	0	0	not significant
5277	MOOTHA_PYR	-0.72	0.09	0	0	0	not significant
5278	RIZ_ERYTHROID_DIFFERENTIATION_APOBEC2	-0.72	0.09	0	0	0	not significant
5279	SHANK_TAL1_TARGETS_DN	-0.72	0.09	0	0	0	not significant
5280	BIOCARTA_41BB_PATHWAY	-0.72	0.08	0	0	0	not significant
5281	GO_ADAPTATION_OF_SIGNALING_PATHWAY	-0.72	0.08	0	0	0	not significant
5282	GO_CCR4_NOT_COMPLEX	-0.72	0.08	0	0	0	not significant
5283	GO_CELLULAR_RESPONSE_TO_LEPTIN_STIMULUS	-0.72	0.08	0	0	0	not significant
5284	GO_CORTICAL_ENDOPLASMIC_RETICULUM	-0.72	0.08	0	0	0	not significant
5285	GO_MUSCLE_HYPERTROPHY_IN_RESPONSE_TO_STRESS	-0.72	0.08	0	0	0	not significant
5286	GO_NEGATIVE_REGULATION_OF_CHROMATIN_SILENCING	-0.72	0.08	0	0	0	not significant
5287	GO_OXIDOREDUCTASE_ACTIVITY_ACTING_ON_THE_CH_NH_GROUP_OF_DONORS_	-0.72	0.08	0	0	0	not significant
5288	ROZANOV_MMP14_TARGETS_SUBSET	-0.72	0.08	0	0	0	not significant
5289	GO_CARBOHYDRATE_KINASE_ACTIVITY	-0.72	0.07	0	0	0	not significant
5290	MATTIOLI_MGUS_VS_MULTIPLE_MYELOMA	-0.72	0.07	0	0	0	not significant
5291	REACTOME_SIGNAL_TRANSDUCTION_BY_L1	-0.72	0.07	0	0	0	not significant
5292	RUAN_RESPONSE_TO_TROGLITAZONE_UP	-0.72	0.07	0	0	0	not significant
5293	VIETOR_IFRD1_TARGETS	-0.72	0.07	0	0	0	not significant
5294	GO_MITOCHONDRIAL_FUSION	-0.72	0.06	0	0	0	not significant
5295	REACTOME_DOWNREGULATION_OF_ERBB2_SIGNALING	-0.72	0.06	0	0	0	not significant
5296	WATANABE_COLON_CANCER_MSI_VS_MSS_UP	-0.72	0.06	0	0	0	not significant
5297	GO_FEAR_RESPONSE	-0.72	0.05	0	0	0	not significant
5298	GO_RESPONSE_TO_AMYLOID_BETA	-0.72	0.05	0	0	0	not significant
5299	HOLLEMAN_ASPARAGINASE_RESISTANCE_ALL_UP	-0.72	0.05	0	0	0	not significant
5300	REACTOME_DAP12_SIGNALING	-0.72	0.05	0	0	0	not significant
5301	GO_ESTABLISHMENT_OR_MAINTENANCE_OF_BIPOLAR_CELL_POLARITY	-0.72	0.04	0	0	0	not significant
5302	BURTON_ADIPOGENESIS_11	-0.72	0.03	0	0	0	not significant
5303	YAO_TEMPORAL_RESPONSE_TO_PROGESTERONE_CLUSTER_13	-0.72	0	0	0	0	not significant
5304	GO_NON_MEMBRANE_SPANNING_PROTEIN_TYROSINE_PHOSPHATASE_ACTIVITY	-0.71	0.09	0	0	0	not significant
5305	GO_POSITIVE_REGULATION_OF_RNA_BINDING	-0.71	0.09	0	0	0	not significant
5306	GO_POSITIVE_REGULATION_OF_TORC1_SIGNALING	-0.71	0.09	0	0	0	not significant
5307	GO_REGULATION_OF_MRNA_EXPORT_FROM_NUCLEUS	-0.71	0.09	0	0	0	not significant
5308	SABATES_COLORECTAL_ADENOMA_SIZE_DN	-0.71	0.09	0	0	0	not significant
5309	BIOCARTA_EPONFKB_PATHWAY	-0.71	0.08	0	0	0	not significant
5310	DACOSTA_LOW_DOSE_UV_RESPONSE_VIA_ERCC3_XPCS_DN	-0.71	0.08	0	0	0	not significant
5311	GO_KERATINOCYTE_DEVELOPMENT	-0.71	0.08	0	0	0	not significant
5312	GO_MICROFILAMENT_MOTOR_ACTIVITY	-0.71	0.08	0	0	0	not significant
5313	GO_PROTEIN_KINASE_A_CATALYTIC_SUBUNIT_BINDING	-0.71	0.08	0	0	0	not significant
5314	GO_REGULATION_OF_UNSATURATED_FATTY_ACID_BIOSYNTHETIC_PROCESS	-0.71	0.08	0	0	0	not significant
5315	GO_CYTOKINE_SECRETION_INVOLVED_IN_IMMUNE_RESPONSE	-0.71	0.07	0	0	0	not significant
5316	GO_HYDROGEN_PEROXIDE_CATABOLIC_PROCESS	-0.71	0.07	0	0	0	not significant
5317	GO_NEGATIVE_REGULATION_OF_ENDOTHELIAL_CELL_PROLIFERATION	-0.71	0.07	0	0	0	not significant
5318	GO_NEUROMUSCULAR_SYNAPTIC_TRANSMISSION	-0.71	0.07	0	0	0	not significant
5319	GO_NUCLEOTIDE_TRANSMEMBRANE_TRANSPORTER_ACTIVITY	-0.71	0.07	0	0	0	not significant
5320	GO_RESPONSE_TO_LEPTIN	-0.71	0.07	0	0	0	not significant
5321	GO_SEQUENCE_SPECIFIC_SINGLE_STRANDED_DNA_BINDING	-0.71	0.07	0	0	0	not significant
5322	OXFORD_RALA_AND_RALB_TARGETS_UP	-0.71	0.07	0	0	0	not significant
5323	GO_FORMATION_OF_EXTRACHROMOSOMAL_CIRCULAR_DNA	-0.71	0.06	0	0	0	not significant
5324	GO_REGULATION_OF_PRODUCTION_OF_SMALL_RNA_INVOLVED_IN_GENE_SILENCI	-0.71	0.06	0	0	0	not significant
5325	REACTOME_G_PROTEIN_BETA:GAMMA_SIGNALLING	-0.71	0.05	0	0	0	not significant
5326	CHRXQ26	-0.71	0.04	0	0	0	not significant
5327	LIEN_BREAST_CARCINOMA_METAPLASTIC_VS_DUCTAL_UP	-0.71	0.04	0	0	0	not significant
5328	GO_BASE_CONVERSION_OR_SUBSTITUTION_EDITING	-0.70	0.08	0	0	0	not significant
5329	GO_GAMMA_AMINOBUTYRIC_ACID_SIGNALING_PATHWAY	-0.70	0.08	0	0	0	not significant

5330	GO_INTERFERON_ALPHA_BIOSYNTHETIC_PROCESS	-0.70	0.08	0	0	0	not significant
5331	GO_LATE_ENDOSOME_TO_LYSOSOME_TRANSPORT	-0.70	0.08	0	0	0	not significant
5332	GO_METALLOCHAPERONE_ACTIVITY	-0.70	0.08	0	0	0	not significant
5333	GO_MYOBLAST_MIGRATION	-0.70	0.08	0	0	0	not significant
5334	HOLLEMAN_PREDNISOLONE_RESISTANCE_ALL_DN	-0.70	0.08	0	0	0	not significant
5335	REACTOME_GRB2:SOS_PROVIDES_LINKAGE_TO_MAPK_SIGNALING_FOR_INTEGRIN	-0.70	0.08	0	0	0	not significant
5336	GO_EMBRYONIC_HEMOPOIESIS	-0.70	0.07	0	0	0	not significant
5337	GO_HISTONE_H3_K9_TRIMETHYLATION	-0.70	0.07	0	0	0	not significant
5338	GO_NEGATIVE_REGULATION_OF_CHOLESTEROL_BIOSYNTHETIC_PROCESS	-0.70	0.07	0	0	0	not significant
5339	GO_NEGATIVE_REGULATION_OF_GLUCONEOGENESIS	-0.70	0.07	0	0	0	not significant
5340	GO_REGULATION_OF_DEOXYRIBONUCLEASE_ACTIVITY	-0.70	0.07	0	0	0	not significant
5341	GO_RESPONSE_TO_SALT_STRESS	-0.70	0.07	0	0	0	not significant
5342	MANN_RESPONSE_TO_AMIFOSTINE_UP	-0.70	0.07	0	0	0	not significant
5343	SILIGAN_TARGETS_OF_EWS_FLI1_FUSION_UP	-0.70	0.07	0	0	0	not significant
5344	ZEMBUTSU_SENSITIVITY_TO_CYCLOPHOSPHAMIDE	-0.70	0.07	0	0	0	not significant
5345	BIOCARTA_PTDINS_PATHWAY	-0.70	0.06	0	0	0	not significant
5346	CHR8Q13	-0.70	0.06	0	0	0	not significant
5347	GO_POSITIVE_REGULATION_OF_EXTRACELLULAR_MATRIX_ORGANIZATION	-0.70	0.06	0	0	0	not significant
5348	GO_POSITIVE_REGULATION_OF_FOCAL_ADHESION_ASSEMBLY	-0.70	0.06	0	0	0	not significant
5349	GO_SET1C_COMPASS_COMPLEX	-0.70	0.06	0	0	0	not significant
5350	HOLLEMAN_PREDNISOLONE_RESISTANCE_ALL_UP	-0.70	0.06	0	0	0	not significant
5351	MARCHINI_TRABECTEDIN_RESISTANCE_UP	-0.70	0.06	0	0	0	not significant
5352	NIKOLSKY_BREAST_CANCER_12Q24_AMPLICON	-0.70	0.06	0	0	0	not significant
5353	REACTOME_P75NTR_SIGNALS_VIA_NF_KB	-0.70	0.06	0	0	0	not significant
5354	WEINMANN_ADAPTATION_TO_HYPOXIA_DN	-0.70	0.06	0	0	0	not significant
5355	GO_DORSAL_VENTRAL_NEURAL_TUBE_PATTERNING	-0.70	0.05	0	0	0	not significant
5356	GO_NUCLEOTIDE_TRANSPORT	-0.70	0.05	0	0	0	not significant
5357	LEE_CALORIE_RESTRICTION_MUSCLE_UP	-0.70	0.05	0	0	0	not significant
5358	GO_FLEMMING_BODY	-0.70	0.04	0	0	0	not significant
5359	GO_NEGATIVE_REGULATION_OF_CALCIUM_ION_TRANSPORT	-0.70	0.04	0	0	0	not significant
5360	CHR8P11	-0.70	0.03	0	0	0	not significant
5361	GO_PROTEIN_CONTAINING_COMPLEX_SCAFFOLD_ACTIVITY	-0.70	0.02	0	0	0	not significant
5362	GO_NEGATIVE_REGULATION_OF_FIBROBLAST_APOPTOTIC_PROCESS	-0.69	0.09	0	0	0	not significant
5363	GO_UDP_N_ACETYLGLUCOSAMINE_METABOLIC_PROCESS	-0.69	0.09	0	0	0	not significant
5364	GO_MESSENGER_RIBONUCLEOPROTEIN_COMPLEX	-0.69	0.08	0	0	0	not significant
5365	GO_NEGATIVE_REGULATION_OF_CAMP_DEPENDENT_PROTEIN_KINASE_ACTIVITY	-0.69	0.08	0	0	0	not significant
5366	GO_NEGATIVE_REGULATION_OF_SODIUM_ION_TRANSMEMBRANE_TRANSPORT	-0.69	0.08	0	0	0	not significant
5367	GO_REGULATION_OF_MACROPHAGE_APOPTOTIC_PROCESS	-0.69	0.08	0	0	0	not significant
5368	GO_RS_DOMAIN_BINDING	-0.69	0.08	0	0	0	not significant
5369	GO_SEQUESTERING_OF_TRIGLYCERIDE	-0.69	0.08	0	0	0	not significant
5370	MATZUK_MALE_REPRODUCTION_SERTOLI	-0.69	0.08	0	0	0	not significant
5371	GO_AXON_ENSHEATHMENT_IN_CENTRAL_NERVOUS_SYSTEM	-0.69	0.07	0	0	0	not significant
5372	GO_FORELIMB_MORPHOGENESIS	-0.69	0.07	0	0	0	not significant
5373	GO_GOLGI_DISASSEMBLY	-0.69	0.07	0	0	0	not significant
5374	GO_PLATELET_DENSE_TUBULAR_NETWORK	-0.69	0.07	0	0	0	not significant
5375	GO_POSITIVE_REGULATION_OF_AUTOPHAGOSOME_ASSEMBLY	-0.69	0.07	0	0	0	not significant
5376	REACTOME_TIGHT_JUNCTION_INTERACTIONS	-0.69	0.07	0	0	0	not significant
5377	REACTOME_WNT_LIGAND_BIOGENESIS_AND_TRAFFICKING	-0.69	0.07	0	0	0	not significant
5378	WACKER_HYPOXIA_TARGETS_OF_VHL	-0.69	0.07	0	0	0	not significant
5379	GO_NUCLEOTIDE_DIPHOSPHATASE_ACTIVITY	-0.69	0.06	0	0	0	not significant
5380	GO_REGULATION_OF_NEURON_PROJECTION_REGENERATION	-0.69	0.06	0	0	0	not significant
5381	GO_SINGLE_STRANDED_DNA_ENDODEOXYRIBONUCLEASE_ACTIVITY	-0.69	0.06	0	0	0	not significant
5382	NABA_BASEMENT_MEMBRANES	-0.69	0.06	0	0	0	not significant
5383	PID_SYNDECAN_1_PATHWAY	-0.69	0.06	0	0	0	not significant
5384	WU_HBX_TARGETS_2_UP	-0.69	0.06	0	0	0	not significant
5385	GO_CELLULAR_PROTEIN_CONTAINING_COMPLEX_LOCALIZATION	-0.69	0.05	0	0	0	not significant
5386	GO_CYTOPLASMIC_EXOSOME_RNASE_COMPLEX_	-0.69	0.05	0	0	0	not significant
5387	REACTOME_REGULATION_OF_INNATE_IMMUNE_RESPONSES_TO_CYTOSOLIC_DNA	-0.69	0.05	0	0	0	not significant
5388	FIGUEROA_AML_METHYLATION_CLUSTER_5_DN	-0.69	0.04	0	0	0	not significant
5389	GO_LOW_DENSITY_LIPOPROTEIN_PARTICLE_CLEARANCE	-0.69	0.04	0	0	0	not significant
5390	GO_PROTEIN_TRANSPORT_WITHIN_PLASMA_MEMBRANE	-0.69	0.04	0	0	0	not significant
5391	KEGG_GALACTOSE_METABOLISM	-0.69	0.04	0	0	0	not significant
5392	GO_REGULATION_OF_AUTOPHAGOSOME_ASSEMBLY	-0.69	0.03	0	0	0	not significant
5393	LEE_LIVER_CANCER_DENA_DN	-0.69	0.03	0	0	0	not significant
5394	GO_MITOCHONDRIAL_RESPIRATORY_CHAIN_COMPLEX_I	-0.69	0.02	0	0	0	not significant
5395	GO_PLATELET_DEGRANULATION	-0.69	0.01	0	0	0	not significant
5396	NIKOLSKY_BREAST_CANCER_8Q12_Q22_AMPLICON	-0.69	0.01	0	0	0	not significant
5397	GO_FATTY_ACID_ALPHA_OXIDATION	-0.68	0.08	0	0	0	not significant
5398	GO_POLAR_MICROTUBULE	-0.68	0.08	0	0	0	not significant
5399	GO_CEREBRAL_CORTEX_NEURON_DIFFERENTIATION	-0.68	0.07	0	0	0	not significant
5400	GO_INOSITOL_METABOLIC_PROCESS	-0.68	0.07	0	0	0	not significant
5401	GO_MEMBRANOUS_SEPTUM_MORPHOGENESIS	-0.68	0.07	0	0	0	not significant
5402	GO_NUCLEAR_NCRNA_SURVEILLANCE	-0.68	0.07	0	0	0	not significant
5403	GO_POSITIVE_REGULATION_OF_SMOOTH_MUSCLE_CONTRACTION	-0.68	0.07	0	0	0	not significant
5404	GO_REGULATION_OF_BICELLULAR_TIGHT_JUNCTION_ASSEMBLY	-0.68	0.07	0	0	0	not significant
5405	REACTOME_DCC_MEDIATED_ATTRACTIVE_SIGNALING	-0.68	0.07	0	0	0	not significant
5406	WANG_RESPONSE_TO_FORSKOLIN_DN	-0.68	0.07	0	0	0	not significant
5407	BIOCARTA_MALATEX_PATHWAY	-0.68	0.06	0	0	0	not significant
5408	CHR2P14	-0.68	0.06	0	0	0	not significant
5409	GO_INNER_CELL_MASS_CELL_PROLIFERATION	-0.68	0.06	0	0	0	not significant
5410	GO_NEGATIVE_REGULATION_OF_ACTIVATED_T_CELL_PROLIFERATION	-0.68	0.06	0	0	0	not significant
5411	GO_POSITIVE_REGULATION_OF_ENDOPLASMIC_RETICULUM_UNFOLDED_PROTEIN_	-0.68	0.06	0	0	0	not significant
5412	REACTOME_REGULATION_OF_GENE_EXPRESSION_IN_LATE_STAGE_BRANCHING_M	-0.68	0.06	0	0	0	not significant
5413	REACTOME_TICAM1_TRAF6_DEPENDENT_INDUCTION_OF_TAK1_COMPLEX	-0.68	0.06	0	0	0	not significant
5414	GAUSSMANN_MLL_AF4_FUSION_TARGETS_D_UP	-0.68	0.05	0	0	0	not significant
5415	GO_CARDIAC_MUSCLE_CELL_MEMBRANE_REPOLARIZATION	-0.68	0.05	0	0	0	not significant
5416	GO_PRP19_COMPLEX	-0.68	0.05	0	0	0	not significant
5417	GO_QUINONE_BINDING	-0.68	0.05	0	0	0	not significant
5418	BIOCARTA_CERAMIDE_PATHWAY	-0.68	0.04	0	0	0	not significant
5419	GO_NEURAL_TUBE_PATTERNING	-0.68	0.04	0	0	0	not significant
5420	GO_PRC1_COMPLEX	-0.68	0.04	0	0	0	not significant
5421	GO_CALCIUM_ION_IMPORT	-0.68	0.03	0	0	0	not significant
5422	GO_HYDRO_LYASE_ACTIVITY	-0.68	0.03	0	0	0	not significant
5423	GO_HYDROLASE_ACTIVITY_ACTING_ON_CARBON_NITROGEN_BUT_NOT_PEPTIDE_	-0.68	0.03	0	0	0	not significant
5424	CHR1P32	-0.68	0.02	0	0	0	not significant
5425	PID_INSULIN_PATHWAY	-0.68	0.02	0	0	0	not significant
5426	REACTOME_G_ALPHA_12_13_SIGNALLING_EVENTS	-0.68	0.02	0	0	0	not significant
5427	GO_IRON_ION_HOMEOSTASIS	-0.68	0.01	0	0	0	not significant
5428	GO_BCL_2_FAMILY_PROTEIN_COMPLEX	-0.67	0.08	0	0	0	not significant
5429	GO_NEURON_INTRINSIC_APOPTOTIC_SIGNALING_PATHWAY_IN_RESPONSE_TO_OX	-0.67	0.08	0	0	0	not significant
5430	GO_DNA_METHYLTRANSFERASE_ACTIVITY	-0.67	0.07	0	0	0	not significant
5431	GO_EMBRYONIC_DIGESTIVE_TRACT_DEVELOPMENT	-0.67	0.07	0	0	0	not significant
5432	GO_MYOSIN_FILAMENT_ORGANIZATION	-0.67	0.07	0	0	0	not significant
5433	GO_REGULATION_OF_RESPONSE_TO_TUMOR_CELL	-0.67	0.07	0	0	0	not significant
5434	NOUSHMEHR_GBM_SOMATIC_MUTATED	-0.67	0.07	0	0	0	not significant
5435	REACTOME_MITOCHONDRIAL_FATTY_ACID_BETA_OXIDATION_OF_UNSATURATED_	-0.67	0.07	0	0	0	not significant
5436	GO_ADENOSINE_DEAMINASE_ACTIVITY	-0.67	0.06	0	0	0	not significant
5437	GO_POSITIVE_REGULATION_OF_PROTEIN_LOCALIZATION_TO_EARLY_ENDOSOME	-0.67	0.06	0	0	0	not significant
5438	GO_RNA_POLYMERASE_I_PREINITIATION_COMPLEX_ASSEMBLY	-0.67	0.06	0	0	0	not significant

5439	NIKOLSKY_BREAST_CANCER_1Q32_AMPLICON	-0.67	0.06	0	0	0	not significant
5440	PID_LPA4_PATHWAY	-0.67	0.06	0	0	0	not significant
5441	GO_ALDO_KETO_REDUCTASE_NADP_ACTIVITY	-0.67	0.05	0	0	0	not significant
5442	GO_PROTEIN_CONTAINING_COMPLEX_REMODELING	-0.67	0.05	0	0	0	not significant
5443	NIKOLSKY_OVERCONNECTED_IN_BREAST_CANCER	-0.67	0.05	0	0	0	not significant
5444	GO_REGULATION_OF_CELL_ADHESION_MEDIATED_BY_INTEGRIN	-0.67	0.04	0	0	0	not significant
5445	GO_REGULATION_OF_EXECUTION_PHASE_OF_APOPTOSIS	-0.67	0.04	0	0	0	not significant
5446	GO_ACETYL_COA_BIOSYNTHETIC_PROCESS	-0.67	0.03	0	0	0	not significant
5447	GO_DETECTION_OF_LIGHT_STIMULUS	-0.67	0.03	0	0	0	not significant
5448	GO_POSITIVE_REGULATION_OF_CELL_MATRIX_ADHESION	-0.67	0.03	0	0	0	not significant
5449	GO_HISTONE_LYSINE_N_METHYLTRANSFERASE_ACTIVITY	-0.67	0.02	0	0	0	not significant
5450	GO_4_IRON_4_SULFUR_CLUSTER_BINDING	-0.67	0.01	0	0	0	not significant
5451	GO_POSITIVE_REGULATION_OF_CALCIUM_ION_TRANSMEMBRANE_TRANSPORT	-0.67	0.01	0	0	0	not significant
5452	GO_POSITIVE_REGULATION_OF_CALCIUM_ION_TRANSPORT	-0.67	0.01	0	0	0	not significant
5453	GO_ESTABLISHMENT_OF_GOLGI_LOCALIZATION	-0.66	0.07	0	0	0	not significant
5454	GO_HYPOTHALAMUS_DEVELOPMENT	-0.66	0.07	0	0	0	not significant
5455	PID_CONE_PATHWAY	-0.66	0.07	0	0	0	not significant
5456	BIOCARTA_CDC25_PATHWAY	-0.66	0.06	0	0	0	not significant
5457	GO_CALCIUM_IMPORT_INTO_THE_MITOCHONDRION	-0.66	0.06	0	0	0	not significant
5458	GO_HETEROCHROMATIN_ASSEMBLY	-0.66	0.06	0	0	0	not significant
5459	GO_INTERFERON_BETA_BIOSYNTHETIC_PROCESS	-0.66	0.06	0	0	0	not significant
5460	GO_NEGATIVE_REGULATION_OF_CELLULAR_RESPONSE_TO_DRUG	-0.66	0.06	0	0	0	not significant
5461	GO_NEGATIVE_REGULATION_OF_DIGESTIVE_SYSTEM_PROCESS	-0.66	0.06	0	0	0	not significant
5462	GO_POSITIVE_REGULATION_OF_PEROXISOME_PROLIFERATOR_ACTIVATED_RECE	-0.66	0.06	0	0	0	not significant
5463	GO_REGULATION_OF_SEQUESTERING_OF_TRIGLYCERIDE	-0.66	0.06	0	0	0	not significant
5464	GO_RNA_POLYMERASE_I_REGULATORY_REGION_DNA_BINDING	-0.66	0.06	0	0	0	not significant
5465	WATANABE_ULCERATIVE_COLITIS_WITH_CANCER_UP	-0.66	0.06	0	0	0	not significant
5466	GO_3_UTR_MEDIATED_MRNA_DESTABILIZATION	-0.66	0.05	0	0	0	not significant
5467	GO_PHOSPHOTRANSFERASE_ACTIVITY_FOR_OTHER_SUBSTITUTED_PHOSPHATE_	-0.66	0.05	0	0	0	not significant
5468	GO_REGULATION_OF_MEMBRANE_REPOLARIZATION	-0.66	0.05	0	0	0	not significant
5469	REACTOME_RHO_GTPASES_ACTIVATE_ROCKS	-0.66	0.05	0	0	0	not significant
5470	YIH_RESPONSE_TO_ARSENITE_C2	-0.66	0.05	0	0	0	not significant
5471	GO_DOSAGE_COMPENSATION	-0.66	0.04	0	0	0	not significant
5472	REACTOME_PURINE_SALVAGE	-0.66	0.04	0	0	0	not significant
5473	GO_ADENYLATE_CYCLASE_INHIBITING_G_PROTEIN_COUPLED_RECEPTOR_SIGNAL	-0.66	0.03	0	0	0	not significant
5474	GO_EPHRIN_RECEPTOR_BINDING	-0.66	0.03	0	0	0	not significant
5475	GO_PHOSPHATIDYLINOSITOL_PHOSPHATE_PHOSPHATASE_ACTIVITY	-0.66	0.03	0	0	0	not significant
5476	KEGG_SNARE_INTERACTIONS_IN_VESICULAR_TRANSPORT	-0.66	0.03	0	0	0	not significant
5477	REACTOME_DETOXIFICATION_OF_REACTIVE_OXYGEN_SPECIES	-0.66	0.03	0	0	0	not significant
5478	BIOCARTA_IL1R_PATHWAY	-0.66	0.02	0	0	0	not significant
5479	EBAUER_TARGETS_OF_PAX3_FOXO1_FUSION_DN	-0.66	0.02	0	0	0	not significant
5480	GO_MYOFIBRIL_ASSEMBLY	-0.66	0.02	0	0	0	not significant
5481	GO_NUCLEOTIDE_EXCISION_REPAIR_PREINCISION_COMPLEX_ASSEMBLY	-0.66	0.02	0	0	0	not significant
5482	GO_TRANSITION_METAL_ION_TRANSMEMBRANE_TRANSPORTER_ACTIVITY	-0.66	0.02	0	0	0	not significant
5483	GO_POSITIVE_REGULATION_OF_TOR_SIGNALING	-0.66	0.01	0	0	0	not significant
5484	REACTOME_EPH_EPHRIN_SIGNALING	-0.66	0.01	0	0	0	not significant
5485	CHEBOTAEV_GR_TARGETS_DN	-0.66	0	0	0	0	not significant
5486	GO_CALCIUM_ION_REGULATED_EXOCYTOSIS	-0.66	0	0	0	0	not significant
5487	GO_DNA_CYTOSINE_DEAMINATION	-0.65	0.06	0	0	0	not significant
5488	GO_DOPAMINE_METABOLIC_PROCESS	-0.65	0.06	0	0	0	not significant
5489	GO_HIGH_DENSITY_LIPOPROTEIN_PARTICLE_REMODELING	-0.65	0.06	0	0	0	not significant
5490	GO_MACROPHAGE_APOPTOTIC_PROCESS	-0.65	0.06	0	0	0	not significant
5491	GO_PROSTANOID_RECEPTOR_ACTIVITY	-0.65	0.06	0	0	0	not significant
5492	GO_SPERMATOPROTEASOME_COMPLEX	-0.65	0.06	0	0	0	not significant
5493	GO_VOCALIZATION_BEHAVIOR	-0.65	0.06	0	0	0	not significant
5494	GLI1_UP.V1_DN	-0.65	0.05	0	0	0	not significant
5495	GO_ESTABLISHMENT_OF_CENTROSOME_LOCALIZATION	-0.65	0.05	0	0	0	not significant
5496	GO_IRON_ION_TRANSMEMBRANE_TRANSPORT	-0.65	0.05	0	0	0	not significant
5497	GO_MEIOTIC_CHROMOSOME_CONDENSATION	-0.65	0.05	0	0	0	not significant
5498	GO_PHOTORECEPTOR_OUTER_SEGMENT_MEMBRANE	-0.65	0.05	0	0	0	not significant
5499	GO_PROTEIN_TAG	-0.65	0.05	0	0	0	not significant
5500	GO_REGULATION_OF_SECONDARY_HEART_FIELD_CARDIOBLAST_PROLIFERATION	-0.65	0.05	0	0	0	not significant
5501	REACTOME_NITRIC_OXIDE_STIMULATES_GUANYLATE_CYCLASE	-0.65	0.05	0	0	0	not significant
5502	REACTOME_REGULATION_OF_BETA_CELL_DEVELOPMENT	-0.65	0.05	0	0	0	not significant
5503	REACTOME_RUNX2_REGULATES_GENES_INVOLVED_IN_CELL_MIGRATION	-0.65	0.05	0	0	0	not significant
5504	PID_TOLL_ENDOGENOUS_PATHWAY	-0.65	0.04	0	0	0	not significant
5505	REACTOME_ZINC_TRANSPORTERS	-0.65	0.04	0	0	0	not significant
5506	GO_ACTIN_CORTICAL_PATCH	-0.65	0.03	0	0	0	not significant
5507	KANG_GIST_WITH_PDGFRA_UP	-0.65	0.03	0	0	0	not significant
5508	REACTOME_NUCLEOTIDE_SALVAGE	-0.65	0.03	0	0	0	not significant
5509	GO_PEPTIDYL_LYSINE_TRIMETHYLATION	-0.65	0.02	0	0	0	not significant
5510	GO_REGULATION_OF_CENTRIOLE_REPLICATION	-0.65	0.02	0	0	0	not significant
5511	GO_REGULATION_OF_EPIDERMAL_CELL_DIFFERENTIATION	-0.65	0.02	0	0	0	not significant
5512	REACTOME_MICROAUTOPHAGY	-0.65	0.02	0	0	0	not significant
5513	GO_CHONDROITIN_SULFATE_BINDING	-0.64	0.06	0	0	0	not significant
5514	GO_LATE_ENDOSOME_TO_VACUOLE_TRANSPORT_VIA_MULTIVESICULAR_BODY_S	-0.64	0.06	0	0	0	not significant
5515	GO_G_PROTEIN_BETA_GAMMA_SUBUNIT_COMPLEX_BINDING	-0.64	0.05	0	0	0	not significant
5516	GO_PALMITOYL_COA_HYDROLASE_ACTIVITY	-0.64	0.05	0	0	0	not significant
5517	GO_POSTSYNAPTIC_MEMBRANE_ASSEMBLY	-0.64	0.05	0	0	0	not significant
5518	GO_RESPONSE_TO_CAFFEINE	-0.64	0.05	0	0	0	not significant
5519	GO_SEQUESTERING_OF_ACTIN_MONOMERS	-0.64	0.05	0	0	0	not significant
5520	REACTOME_APOBEC3G_MEDIATED_RESISTANCE_TO_HIV_1_INFECTION	-0.64	0.05	0	0	0	not significant
5521	REACTOME_FREE_FATTY_ACIDS_REGULATE_INSULIN_SECRETION	-0.64	0.05	0	0	0	not significant
5522	FIGUEROA_AML_METHYLATION_CLUSTER_6_DN	-0.64	0.04	0	0	0	not significant
5523	GO_CHONDROCYTE_DIFFERENTIATION_INVOLVED_IN_ENDOCHONDRAL_BONE_MO	-0.64	0.04	0	0	0	not significant
5524	GO_NEUROMUSCULAR_PROCESS_CONTROLLING_POSTURE	-0.64	0.04	0	0	0	not significant
5525	GO_PIRNA_METABOLIC_PROCESS	-0.64	0.04	0	0	0	not significant
5526	GO_REGULATION_OF_URINE_VOLUME	-0.64	0.04	0	0	0	not significant
5527	MCMURRAY_TP53_HRAS_COOPERATION_RESPONSE_UP	-0.64	0.04	0	0	0	not significant
5528	BIOCARTA_TGFB_PATHWAY	-0.64	0.03	0	0	0	not significant
5529	GO_CELLULAR_RESPONSE_TO_DSRNA	-0.64	0.03	0	0	0	not significant
5530	GO_COPI_COATED_VESICLE	-0.64	0.03	0	0	0	not significant
5531	GO_CHONDROCYTE_DIFFERENTIATION	-0.64	0.01	0	0	0	not significant
5532	GO_REGULATION_OF_MUSCLE_HYPERTROPHY	-0.64	0.01	0	0	0	not significant
5533	HOFFMANN_IMMATURE_TO_MATURE_B_LYMPHOCYTE_DN	-0.64	0.01	0	0	0	not significant
5534	LEE_LIVER_CANCER_E2F1_DN	-0.64	0.01	0	0	0	not significant
5535	GO_PIGMENTATION	-0.64	0	0	0	0	not significant
5536	GO_CORECEPTOR_ACTIVITY_INVOLVED_IN_WNT_SIGNALING_PATHWAY	-0.63	0.06	0	0	0	not significant
5537	GO_CYTIDINE_DEAMINASE_ACTIVITY	-0.63	0.05	0	0	0	not significant
5538	GO_HISTONE_THREONINE_KINASE_ACTIVITY	-0.63	0.05	0	0	0	not significant
5539	GO_POST_EMBRYONIC_EYE_MORPHOGENESIS	-0.63	0.05	0	0	0	not significant
5540	GO_THIOREDOXIN_PEROXIDASE_ACTIVITY	-0.63	0.05	0	0	0	not significant
5541	INAMURA_LUNG_CANCER_SCC_DN	-0.63	0.05	0	0	0	not significant
5542	CHR1P12	-0.63	0.04	0	0	0	not significant
5543	GO_CIRCADIAN_SLEEP_WAKE_CYCLE	-0.63	0.04	0	0	0	not significant
5544	GO_HISTONE_ACETYLTRANSFERASE_ACTIVITY_H4_K5_SPECIFIC_	-0.63	0.04	0	0	0	not significant
5545	GO_PHOSPHATIDYLINOSITOL_PHOSPHATE_4_PHOSPHATASE_ACTIVITY	-0.63	0.04	0	0	0	not significant
5546	GO_POSITIVE_REGULATION_OF_ALCOHOL_BIOSYNTHETIC_PROCESS	-0.63	0.04	0	0	0	not significant
5547	GO_POSTSYNAPSE_ASSEMBLY	-0.63	0.04	0	0	0	not significant

5548	HALLMARK_PANCREAS_BETA_CELLS	-0.63	0.03	0	0	0	not significant
5549	PAPASPYRIDONOS_UNSTABLE_ATEROSCLEROTIC_PLAQUE_DN	-0.63	0.03	0	0	0	not significant
5550	REACTOME_VXPX_CARGO_TARGETING_TO_CILIUM	-0.63	0.03	0	0	0	not significant
5551	TERAMOTO_OPN_TARGETS_CLUSTER_6	-0.63	0.03	0	0	0	not significant
5552	XU_GH1_EXOGENOUS_TARGETS_UP	-0.63	0.02	0	0	0	not significant
5553	KEGG_OXIDATIVE_PHOSPHORYLATION	-0.63	0	0	0	0	not significant
5554	REACTOME_BLOOD_GROUP_SYSTEMS_BIOSYNTHESIS	-0.62	0.06	0	0	0	not significant
5555	GO_ATP_DEPENDENT_MICROTUBULE_MOTOR_ACTIVITY_MINUS_END_DIRECTED	-0.62	0.05	0	0	0	not significant
5556	GO_CHROMO_SHADOW_DOMAIN_BINDING	-0.62	0.05	0	0	0	not significant
5557	GO_G_PROTEIN_COUPLED_ACETYLCHOLINE_RECEPTOR_SIGNALING_PATHWAY	-0.62	0.05	0	0	0	not significant
5558	GO_HEPATOCYTE_GROWTH_FACTOR_RECEPTOR_SIGNALING_PATHWAY	-0.62	0.05	0	0	0	not significant
5559	GO_HISTONE_THREONINE_PHOSPHORYLATION	-0.62	0.05	0	0	0	not significant
5560	GO_MONOCYTE_ACTIVATION	-0.62	0.05	0	0	0	not significant
5561	GO_PROTEIN_INSERTION_INTO_ER_MEMBRANE	-0.62	0.05	0	0	0	not significant
5562	MOTAMED_RESPONSE_TO_ANDROGEN_DN	-0.62	0.05	0	0	0	not significant
5563	SETLUR_PROSTATE_CANCER_TMPRSS2_ERG_FUSION_DN	-0.62	0.05	0	0	0	not significant
5564	CERVERA_SDHB_TARGETS_1_DN	-0.62	0.04	0	0	0	not significant
5565	GO_MAMMARY_GLAND_ALVEOLUS_DEVELOPMENT	-0.62	0.04	0	0	0	not significant
5566	GO_NEURON_CELL_CELL_ADHESION	-0.62	0.04	0	0	0	not significant
5567	GO_POSITIVE_REGULATION_OF_AUTOPHAGOSOME_MATURATION	-0.62	0.04	0	0	0	not significant
5568	GO_POSITIVE_REGULATION_OF_MYELOID_CELL_APOPTOTIC_PROCESS	-0.62	0.04	0	0	0	not significant
5569	GO_REGULATION_OF_MYOSIN_LIGHT_CHAIN_PHOSPHATASE_ACTIVITY	-0.62	0.04	0	0	0	not significant
5570	GO_RESPONSE_TO_HERBICIDE	-0.62	0.04	0	0	0	not significant
5571	GO_REVERSE_CHOLESTEROL_TRANSPORT	-0.62	0.04	0	0	0	not significant
5572	GO_DEOXYRIBONUCLEOSIDE_TRIPHOSPHATE_BIOSYNTHETIC_PROCESS	-0.62	0.03	0	0	0	not significant
5573	VALK_AML_CLUSTER_5	-0.62	0.03	0	0	0	not significant
5574	BIOCARTA_RHO_PATHWAY	-0.62	0.02	0	0	0	not significant
5575	GO_POSITIVE_REGULATION_OF_AXON_EXTENSION	-0.62	0.01	0	0	0	not significant
5576	GO_OXIDOREDUCTASE_ACTIVITY_ACTING_ON_NAD_P_H	-0.62	0	0	0	0	not significant
5577	GO_ACTIN_CORTICAL_PATCH_LOCALIZATION	-0.61	0.05	0	0	0	not significant
5578	GO_DETECTION_OF_MECHANICAL_STIMULUS_INVOLVED_IN_SENSORY_PERCEPTIC	-0.61	0.05	0	0	0	not significant
5579	GO_OXIDOREDUCTASE_ACTIVITY_ACTING_ON_PAIRED_DONORS_WITH_INCORPOR	-0.61	0.05	0	0	0	not significant
5580	GO_POSITIVE_REGULATION_OF_INTERFERON_GAMMA_BIOSYNTHETIC_PROCESS	-0.61	0.05	0	0	0	not significant
5581	GO_PROGRAMMED_CELL_DEATH_INVOLVED_IN_CELL_DEVELOPMENT	-0.61	0.05	0	0	0	not significant
5582	GO_SPANNING_COMPONENT_OF_MEMBRANE	-0.61	0.05	0	0	0	not significant
5583	GO_CIRCADIAN_SLEEP_WAKE_CYCLE_SLEEP	-0.61	0.04	0	0	0	not significant
5584	GO_L_PHENYLALANINE_CATABOLIC_PROCESS	-0.61	0.04	0	0	0	not significant
5585	GO_LIPID_DROPLET_ORGANIZATION	-0.61	0.04	0	0	0	not significant
5586	GO_NEGATIVE_REGULATION_OF_SYNAPSE_ORGANIZATION	-0.61	0.04	0	0	0	not significant
5587	OXFORD_RALA_TARGETS_DN	-0.61	0.04	0	0	0	not significant
5588	GO_REGULATION_OF_CELL_GROWTH_INVOLVED_IN_CARDIAC_MUSCLE_CELL_DE\	-0.61	0.03	0	0	0	not significant
5589	GO_REGULATION_OF_RECEPTOR_BINDING	-0.61	0.03	0	0	0	not significant
5590	CHR2Q23	-0.61	0.02	0	0	0	not significant
5591	GO_CHONDROCYTE_DEVELOPMENT	-0.61	0.02	0	0	0	not significant
5592	GO_REGULATION_OF_RESPONSE_TO_INTERFERON_GAMMA	-0.61	0.01	0	0	0	not significant
5593	TBK1.DN.48HRS_DN	-0.61	0.01	0	0	0	not significant
5594	GO_AMINO_ACID_BETAINE_METABOLIC_PROCESS	-0.60	0.05	0	0	0	not significant
5595	GO_ADULT_HEART_DEVELOPMENT	-0.60	0.04	0	0	0	not significant
5596	GO_C4_DICARBOXYLATE_TRANSMEMBRANE_TRANSPORTER_ACTIVITY	-0.60	0.04	0	0	0	not significant
5597	GO_CALCIUM_ION_IMPORT_INTO_CYTOSOL	-0.60	0.04	0	0	0	not significant
5598	GO_FEMALE_GENITALIA_DEVELOPMENT	-0.60	0.04	0	0	0	not significant
5599	GO_POST_EMBRYONIC_ANIMAL_ORGAN_MORPHOGENESIS	-0.60	0.04	0	0	0	not significant
5600	KANG_GIST_WITH_PDGFRA_DN	-0.60	0.04	0	0	0	not significant
5601	REACTOME_PHENYLALANINE_AND_TYROSINE_METABOLISM	-0.60	0.04	0	0	0	not significant
5602	GO_POST_EMBRYONIC_ANIMAL_MORPHOGENESIS	-0.60	0.03	0	0	0	not significant
5603	GO_PROTON_TRANSPORTING_TWO_SECTOR_ATPASE_COMPLEX_ASSEMBLY	-0.60	0.03	0	0	0	not significant
5604	SINGH_NFE2L2_TARGETS	-0.60	0.03	0	0	0	not significant
5605	GO_CRANIAL_NERVE_DEVELOPMENT	-0.60	0.02	0	0	0	not significant
5606	GO_NEGATIVE_REGULATION_OF_NEURAL_PRECURSOR_CELL_PROLIFERATION	-0.60	0.02	0	0	0	not significant
5607	GO_RESPONSE_TO_INCREASED_OXYGEN_LEVELS	-0.60	0.02	0	0	0	not significant
5608	REACTOME_THE_PHOTOTRANSDUCTION_CASCADE	-0.60	0.02	0	0	0	not significant
5609	GO_NUCLEAR_HETEROCHROMATIN	-0.60	0.01	0	0	0	not significant
5610	GO_EYE_MORPHOGENESIS	-0.60	0	0	0	0	not significant
5611	GO_INTERFERON_GAMMA_BIOSYNTHETIC_PROCESS	-0.59	0.05	0	0	0	not significant
5612	GO_EXTRACELLULAR_MATRIX_CONSTITUENT_CONFERRING_ELASTICITY	-0.59	0.04	0	0	0	not significant
5613	GO_NEGATIVE_REGULATION_OF_CYSTEINE_TYPE_ENDOPEPTIDASE_ACTIVITY_INV	-0.59	0.04	0	0	0	not significant
5614	GO_PROTEIN_PHOSPHATASE_2B_BINDING	-0.59	0.04	0	0	0	not significant
5615	GO_SPHINGOMYELIN_BIOSYNTHETIC_PROCESS	-0.59	0.04	0	0	0	not significant
5616	GO_SYNAPTIC_VESICLE_MATURATION	-0.59	0.04	0	0	0	not significant
5617	GO_CELLULAR_RESPONSE_TO_ATP	-0.59	0.03	0	0	0	not significant
5618	GO_OXIDOREDUCTASE_ACTIVITY_ACTING_ON_THE_CH_NH_GROUP_OF_DONORS	-0.59	0.03	0	0	0	not significant
5619	GO_PEPTIDE_DISULFIDE_OXIDOREDUCTASE_ACTIVITY	-0.59	0.03	0	0	0	not significant
5620	GO_POSITIVE_REGULATION_OF_HEART_RATE	-0.59	0.03	0	0	0	not significant
5621	GO_REGULATION_OF_POSTSYNAPTIC_NEUROTRANSMITTER_RECEPTOR_INTERNA	-0.59	0.03	0	0	0	not significant
5622	MATZUK_POSTIMPLANTATION_AND_POSTPARTUM	-0.59	0.03	0	0	0	not significant
5623	SA_PROGRAMMED_CELL_DEATH	-0.59	0.03	0	0	0	not significant
5624	BANDRES_RESPONSE_TO_CARMUSTIN_MGMT_24HR_DN	-0.59	0.02	0	0	0	not significant
5625	MATZUK_POST-IMPLANTATION_AND_POST-PARTUM	-0.59	0.02	0	0	0	not significant
5626	GO_PRESYNAPTIC_ACTIVE_ZONE_MEMBRANE	-0.59	0.01	0	0	0	not significant
5627	GO_REGULATION_OF_SMOOTH_MUSCLE_CONTRACTION	-0.59	0.01	0	0	0	not significant
5628	BENPORATH_ES_CORE_NINE	-0.58	0.03	0	0	0	not significant
5629	GO_EXTRACELLULAR_MATRIX_STRUCTURAL_CONSTITUENT_CONFERRING_COMPF	-0.58	0.03	0	0	0	not significant
5630	GO_FERRIC_IRON_BINDING	-0.58	0.03	0	0	0	not significant
5631	GO_POSITIVE_REGULATION_OF_STEM_CELL_DIFFERENTIATION	-0.58	0.03	0	0	0	not significant
5632	GO_S_ADENOSYLMETHIONINE_CYCLE	-0.58	0.03	0	0	0	not significant
5633	YAO_HOXA10_TARGETS_VIA_PROGESTERONE_DN	-0.58	0.03	0	0	0	not significant
5634	BIOCARTA_MCALPAIN_PATHWAY	-0.58	0.02	0	0	0	not significant
5635	GO_DNA_UNWINDING_INVOLVED_IN_DNA_REPLICATION	-0.58	0.02	0	0	0	not significant
5636	GO_GRANULOCYTE_MACROPHAGE_COLONY_STIMULATING_FACTOR_PRODUCTIOI	-0.58	0.02	0	0	0	not significant
5637	GO_REGULATION_OF_ADENYLATE_CYCLASE_ACTIVITY	-0.58	0.02	0	0	0	not significant
5638	GO_REGULATION_OF_CARDIAC_MUSCLE_CELL_DIFFERENTIATION	-0.58	0.02	0	0	0	not significant
5639	GO_REGULATION_OF_METHYLATION_DEPENDENT_CHROMATIN_SILENCING	-0.58	0.02	0	0	0	not significant
5640	GO_RESPONSE_TO_FOOD	-0.58	0.02	0	0	0	not significant
5641	GO_THO_COMPLEX	-0.58	0.02	0	0	0	not significant
5642	KYNG_NORMAL_AGING_DN	-0.58	0.02	0	0	0	not significant
5643	MIKKELSEN_MEF_LCP_WITH_H3K27ME3	-0.58	0.02	0	0	0	not significant
5644	GO_FOREBRAIN_GENERATION_OF_NEURONS	-0.58	0.01	0	0	0	not significant
5645	GO_REGULATION_OF_EPIDERMIS_DEVELOPMENT	-0.58	0.01	0	0	0	not significant
5646	REACTOME_G_BETA:GAMMA_SIGNALLING_THROUGH_PI3KGAMMA	-0.58	0.01	0	0	0	not significant
5647	XU_CREBBP_TARGETS_DN	-0.58	0.01	0	0	0	not significant
5648	GO_PEROXISOMAL_TRANSPORT	-0.58	0	0	0	0	not significant
5649	BIOCARTA_FREE_PATHWAY	-0.57	0.03	0	0	0	not significant
5650	GO_1_PHOSPHATIDYLINOSITOL_4_PHOSPHATE_3_KINASE_ACTIVITY	-0.57	0.03	0	0	0	not significant
5651	GO_HISTONE_H3_K36_DEMETHYLATION	-0.57	0.03	0	0	0	not significant
5652	GO_IMMUNE_RESPONSE_TO_TUMOR_CELL	-0.57	0.03	0	0	0	not significant
5653	GO_NEGATIVE_REGULATION_OF_HISTONE_H3_K4_METHYLATION	-0.57	0.03	0	0	0	not significant
5654	GO_POSITIVE_REGULATION_OF_HEAT_GENERATION	-0.57	0.03	0	0	0	not significant
5655	GO_PROTEIN_LOCALIZATION_TO_NON_MOTILE_CILIUM	-0.57	0.03	0	0	0	not significant
5656	GO_REGULATION_OF_MDA_5_SIGNALING_PATHWAY	-0.57	0.03	0	0	0	not significant

5657	GO_VITAMIN_D_METABOLIC_PROCESS	-0.57	0.03	0	0	0	not significant
5658	KUWANO_RNA_STABILIZED_BY_NO	-0.57	0.03	0	0	0	not significant
5659	GO_MELANOCYTE_DIFFERENTIATION	-0.57	0.02	0	0	0	not significant
5660	GO_NEGATIVE_REGULATION_OF_SINGLE_STRANDED_VIRAL_RNA_REPLICATION_VI	-0.57	0.02	0	0	0	not significant
5661	GO_ORGANELLE_INHERITANCE	-0.57	0.02	0	0	0	not significant
5662	GO_REGULATION_OF_TRANSPOSITION	-0.57	0.02	0	0	0	not significant
5663	GO_RESPONSE_TO_GRANULOCYTE_MACROPHAGE_COLONY_STIMULATING_FACT	-0.57	0.02	0	0	0	not significant
5664	GO_REGULATION_OF_CARDIOCYTE_DIFFERENTIATION	-0.57	0.01	0	0	0	not significant
5665	GO_RESPONSE_TO_ELECTRICAL_STIMULUS	-0.57	0.01	0	0	0	not significant
5666	KANG_CISPLATIN_RESISTANCE_UP	-0.57	0.01	0	0	0	not significant
5667	GO_REGULATION_OF_MEGAKARYOCYTE_DIFFERENTIATION	-0.57	0	0	0	0	not significant
5668	GO_NURF_COMPLEX	-0.56	0.04	0	0	0	not significant
5669	GO_POSITIVE_REGULATION_OF_KILLING_OF_CELLS_OF_OTHER_ORGANISM	-0.56	0.03	0	0	0	not significant
5670	GO_PYRIMIDINE_RIBONUCLEOSIDE_CATABOLIC_PROCESS	-0.56	0.03	0	0	0	not significant
5671	REACTOME_PI5P_REGULATES_TP53_ACETYLATION	-0.56	0.03	0	0	0	not significant
5672	BIOCARTA_CTBP1_PATHWAY	-0.56	0.02	0	0	0	not significant
5673	GO_CALCIUM_TRANSPORTING_ATPASE_ACTIVITY	-0.56	0.02	0	0	0	not significant
5674	GO_DEFINITIVE_HEMOPOIESIS	-0.56	0.02	0	0	0	not significant
5675	GO_HISTONE_DEMETHYLASE_ACTIVITY_H3_K36_SPECIFIC_	-0.56	0.02	0	0	0	not significant
5676	KOBAYASHI_EGFR_SIGNALING_6HR_DN	-0.56	0.02	0	0	0	not significant
5677	REACTOME_DECTIN_2_FAMILY	-0.56	0.02	0	0	0	not significant
5678	REACTOME_RNF_MUTANTS_SHOW_ENHANCED_WNT_SIGNALING_AND_PROLIFERA	-0.56	0.02	0	0	0	not significant
5679	GO_5_3_EXONUCLEASE_ACTIVITY	-0.56	0.01	0	0	0	not significant
5680	GO_GENETIC_IMPRINTING	-0.56	0.01	0	0	0	not significant
5681	GO_MITOCHONDRIAL_CALCIUM_ION_HOMEOSTASIS	-0.56	0.01	0	0	0	not significant
5682	GO_NEGATIVE_REGULATION_OF_SIGNAL_TRANSDUCTION_IN_ABSENCE_OF_LIGAN	-0.56	0	0	0	0	not significant
5683	GO_ZINC_ION_HOMEOSTASIS	-0.56	0	0	0	0	not significant
5684	GO_FIBROBLAST_GROWTH_FACTOR_PRODUCTION	-0.55	0.03	0	0	0	not significant
5685	GO_POSITIVE_REGULATION_OF_ACTIN_FILAMENT_DEPOLYMERIZATION	-0.55	0.03	0	0	0	not significant
5686	GO_PURINE_RIBONUCLEOSIDE_SALVAGE	-0.55	0.03	0	0	0	not significant
5687	GO_UBIQUITIN_DEPENDENT_ENDOCYTOSIS	-0.55	0.03	0	0	0	not significant
5688	PLASARI_NFIC_TARGETS_BASAL_UP	-0.55	0.03	0	0	0	not significant
5689	ALONSO_METASTASIS_NEURAL_UP	-0.55	0.02	0	0	0	not significant
5690	BIOCARTA_SAM68_PATHWAY	-0.55	0.02	0	0	0	not significant
5691	GO_CELLULAR_RESPONSE_TO_COLD	-0.55	0.02	0	0	0	not significant
5692	GO_NEGATIVE_REGULATION_OF_PEPTIDYL_CYSTEINE_S_NITROSYLATION	-0.55	0.02	0	0	0	not significant
5693	GO_NUCLEAR_POLYADENYLATION_DEPENDENT_TRNA_CATABOLIC_PROCESS	-0.55	0.02	0	0	0	not significant
5694	GO_PROTEASOME_CORE_COMPLEX_BETA_SUBUNIT_COMPLEX	-0.55	0.02	0	0	0	not significant
5695	ANDERSEN_CHOLANGIOCARCINOMA_CLASS1	-0.55	0.01	0	0	0	not significant
5696	FINETTI_BREAST_CANCER_KINOME_GREEN	-0.55	0.01	0	0	0	not significant
5697	GO_GABA_ERGIC_SYNAPSE	-0.55	0.01	0	0	0	not significant
5698	GO_REGULATION_OF_ACTIN_NUCLEATION	-0.55	0.01	0	0	0	not significant
5699	GO_CELLULAR_METABOLIC_COMPOUND_SALVAGE	-0.55	0	0	0	0	not significant
5700	GO_PHOSPHATIDYLINOSITOL_BISPHOSPHATE_PHOSPHATASE_ACTIVITY	-0.55	0	0	0	0	not significant
5701	GO_REGULATION_OF_PEPTIDYL_CYSTEINE_S_NITROSYLATION	-0.54	0.03	0	0	0	not significant
5702	REACTOME_SODIUM_PROTON_EXCHANGERS	-0.54	0.03	0	0	0	not significant
5703	GO_CAMP_DEPENDENT_PROTEIN_KINASE_INHIBITOR_ACTIVITY	-0.54	0.02	0	0	0	not significant
5704	GO_CRANIAL_NERVE_MORPHOGENESIS	-0.54	0.02	0	0	0	not significant
5705	GO_DNA_METHYLATION_INVOLVED_IN_GAMETE_GENERATION	-0.54	0.02	0	0	0	not significant
5706	GO_POSITIVE_REGULATION_OF_LYASE_ACTIVITY	-0.54	0.02	0	0	0	not significant
5707	GO_TOOTH_MINERALIZATION	-0.54	0.02	0	0	0	not significant
5708	SHI_SPARC_TARGETS_DN	-0.54	0.02	0	0	0	not significant
5709	TERAMOTO_OPN_TARGETS_CLUSTER_3	-0.54	0.02	0	0	0	not significant
5710	GO_EXTRACELLULAR_VESICLE_BIOGENESIS	-0.54	0.01	0	0	0	not significant
5711	GO_NEGATIVE_REGULATION_OF_INTERLEUKIN_8_SECRETION	-0.54	0.01	0	0	0	not significant
5712	REACTOME_BETA_OXIDATION_OF_DECANOYL_COA_TO_OCTANOYL_COA_COA	-0.54	0.01	0	0	0	not significant
5713	GO_PEROXISOME_ORGANIZATION	-0.54	0	0	0	0	not significant
5714	GO_XENOBIOTIC_CATABOLIC_PROCESS	-0.53	0.03	0	0	0	not significant
5715	CHR3Q24	-0.53	0.02	0	0	0	not significant
5716	GO_NEGATIVE_REGULATION_OF_GLIAL_CELL_DIFFERENTIATION	-0.53	0.02	0	0	0	not significant
5717	GO_POSITIVE_REGULATION_OF_ADENYLATE_CYCLASE_ACTIVITY	-0.53	0.02	0	0	0	not significant
5718	GO_REGULATION_OF_EXOSOMAL_SECRETION	-0.53	0.02	0	0	0	not significant
5719	HUMMERICH_BENIGN_SKIN_TUMOR_UP	-0.53	0.02	0	0	0	not significant
5720	MEISSNER_BRAIN_HCP_WITH_H3K4ME2	-0.53	0.02	0	0	0	not significant
5721	REACTOME_EICOSANOID_LIGAND_BINDING_RECEPTORS	-0.53	0.02	0	0	0	not significant
5722	REACTOME_RUNX3_REGULATES_BCL2L11_BIM_TRANSCRIPTION	-0.53	0.02	0	0	0	not significant
5723	GO_HEART_FORMATION	-0.53	0.01	0	0	0	not significant
5724	GO_MEMBRANE_REPOLARIZATION	-0.53	0.01	0	0	0	not significant
5725	GO_METHIONINE_BIOSYNTHETIC_PROCESS	-0.52	0.02	0	0	0	not significant
5726	GO_POSITIVE_REGULATION_OF_PROTEIN_GLYCOSYLATION	-0.52	0.02	0	0	0	not significant
5727	HANSON_HRAS_SIGNALING_VIA_NFKB	-0.52	0.02	0	0	0	not significant
5728	WHITESIDE_CISPLATIN_RESISTANCE_UP	-0.52	0.02	0	0	0	not significant
5729	CHR8Q12	-0.52	0.01	0	0	0	not significant
5730	GO_CAMP_DEPENDENT_PROTEIN_KINASE_REGULATOR_ACTIVITY	-0.52	0.01	0	0	0	not significant
5731	GO_INHIBITORY_POSTSYNAPTIC_POTENTIAL	-0.52	0.01	0	0	0	not significant
5732	NABA_PROTEOGLYCANS	-0.52	0.01	0	0	0	not significant
5733	CHR2P21	-0.52	0	0	0	0	not significant
5734	GO_REGULATION_OF_COENZYME_METABOLIC_PROCESS	-0.52	0	0	0	0	not significant
5735	REACTOME_ACTIVATED_PKN1_STIMULATES_TRANSCRIPTION_OF_AR_ANDROGEN_	-0.52	0	0	0	0	not significant
5736	GO_1_PHOSPHATIDYLINOSITOL_4_PHOSPHATE_5_KINASE_ACTIVITY	-0.51	0.01	0	0	0	not significant
5737	GO_CHAPERONE_MEDIATED_PROTEIN_TRANSPORT	-0.51	0.01	0	0	0	not significant
5738	GO_NEGATIVE_REGULATION_OF_ADENYLATE_CYCLASE_ACTIVITY	-0.51	0.01	0	0	0	not significant
5739	GO_NEGATIVE_REGULATION_OF_PROTEIN_LOCALIZATION_TO_CELL_SURFACE	-0.51	0.01	0	0	0	not significant
5740	GO_POSITIVE_REGULATION_OF_ACTIN_NUCLEATION	-0.51	0.01	0	0	0	not significant
5741	GO_PYRIMIDINE_NUCLEOSIDE_BIOSYNTHETIC_PROCESS	-0.51	0.01	0	0	0	not significant
5742	GO_REGULATION_OF_ACYL_COA_BIOSYNTHETIC_PROCESS	-0.51	0.01	0	0	0	not significant
5743	GO_REGULATION_OF_MITOCHONDRIAL_FUSION	-0.51	0.01	0	0	0	not significant
5744	GO_RIBONUCLEOSIDE_CATABOLIC_PROCESS	-0.51	0.01	0	0	0	not significant
5745	PARK_TRETINOIN_RESPONSE	-0.51	0.01	0	0	0	not significant
5746	REACTOME_SEMA3A_PLEXIN_REPULSION_SIGNALING_BY_INHIBITING_INTEGRIN_ADI	-0.51	0.01	0	0	0	not significant
5747	RODRIGUES_NTN1_TARGETS_UP	-0.51	0.01	0	0	0	not significant
5748	GO_POSITIVE_REGULATION_OF_SIGNALING_RECEPTOR_ACTIVITY	-0.51	0	0	0	0	not significant
5749	GO_REGULATION_OF_EXTRINSIC_APOPTOTIC_SIGNALING_PATHWAY_IN_ABSENCE	-0.51	0	0	0	0	not significant
5750	NAKAYAMA_SOFT_TISSUE_TUMORS_PCA2_DN	-0.51	0	0	0	0	not significant
5751	REACTOME_DNA_METHYLATION	-0.51	0	0	0	0	not significant
5752	GO_ADENOSINE_TO_INOSINE_EDITING	-0.50	0.01	0	0	0	not significant
5753	GO_RENAL_ABSORPTION	-0.50	0.01	0	0	0	not significant
5754	GO_VITAMIN_CATABOLIC_PROCESS	-0.50	0.01	0	0	0	not significant
5755	ZHAN_MULTIPLE_MYELOMA_DN	-0.50	0	0	0	0	not significant
5756	BIOCARTA_HES_PATHWAY	-0.49	0.01	0	0	0	not significant
5757	GO_BH_DOMAIN_BINDING	-0.49	0.01	0	0	0	not significant
5758	GO_HOMOCYSTEINE_METABOLIC_PROCESS	-0.49	0.01	0	0	0	not significant
5759	GO_PYRIMIDINE_NUCLEOTIDE_SALVAGE	-0.49	0.01	0	0	0	not significant
5760	GO_REGULATION_OF_KERATINOCYTE_PROLIFERATION	-0.49	0.01	0	0	0	not significant
5761	HALLMARK_COAGULATION	-0.49	0	0	0	0	not significant
5762	GO_VINCULIN_BINDING	-0.48	0.01	0	0	0	not significant
5763	GO_G_PROTEIN_COUPLED_PURINERGIC_RECEPTOR_SIGNALING_PATHWAY	-0.48	0	0	0	0	not significant
5764	GO_PHOSPHATIDYLINOSITOL_PHOSPHATE_KINASE_ACTIVITY	-0.48	0	0	0	0	not significant
5765	GO_HISTAMINE_TRANSPORT	-0.47	0.01	0	0	0	not significant

5766	GO_STRESS_INDUCED_PREMATURE_SENESCENCE	-0.47	0.01	0	0	0	not significant
5767	BIOCARTA_CYTOKINE_PATHWAY	-0.47	0	0	0	0	not significant
5768	IIZUKA_LIVER_CANCER_PROGRESSION_G2_G3_UP	-0.47	0	0	0	0	not significant
5769	KEGG_STEROID_HORMONE_BIOSYNTHESIS	-0.47	0	0	0	0	not significant
5770	GO_ADP_RIBOSYLATION_FACTOR_BINDING	-0.46	0.01	0	0	0	not significant
5771	GO_NEUROPEPTIDE_RECEPTOR_ACTIVITY	-0.46	0.01	0	0	0	not significant
5772	GO_PRIMITIVE_HEMOPOIESIS	-0.46	0.01	0	0	0	not significant
5773	GO_BLOOD_VESSEL_ENDOTHELIAL_CELL_DIFFERENTIATION	-0.46	0	0	0	0	not significant
5774	GO_CD4_RECEPTOR_BINDING	-0.46	0	0	0	0	not significant
5775	GO_DIGESTIVE_TRACT_MORPHOGENESIS	-0.46	0	0	0	0	not significant
5776	GO_INTRACILIARY_TRANSPORT_PARTICLE_B	-0.46	0	0	0	0	not significant
5777	GO_L_ASCORBIC_ACID_METABOLIC_PROCESS	-0.46	0	0	0	0	not significant
5778	BOYAULT_LIVER_CANCER_SUBCLASS_G12_DN	-0.45	0	0	0	0	not significant
5779	CHAN_INTERFERON_PRODUCING_DENDRITIC_CELL	-0.45	0	0	0	0	not significant
5780	GO_OXYGEN_BINDING	-0.45	0	0	0	0	not significant
5781	GO_PROTON_EXPORTING_ATPASE_ACTIVITY_PHOSPHORYLATIVE_MECHANISM	-0.45	0	0	0	0	not significant
5782	GO_RENAL_SODIUM_ION_TRANSPORT	-0.45	0	0	0	0	not significant
5783	GO_ADENOSINE_RECEPTOR_SIGNALING_PATHWAY	-0.44	0.01	0	0	0	not significant
5784	GO_DNA_PROTECTION	-0.44	0.01	0	0	0	not significant
5785	GO_VESICLE_FUSION_WITH_ENDOPLASMIC_RETICULUM_GOLGI_INTERMEDIATE_CC	-0.44	0.01	0	0	0	not significant
5786	GO_NEGATIVE_REGULATION_OF_MITOCHONDRIAL_FUSION	-0.44	0	0	0	0	not significant
5787	GO_NEGATIVE_REGULATION_OF_VASCULAR_ENDOTHELIAL_GROWTH_FACTOR_RI	-0.44	0	0	0	0	not significant
5788	GO_POSITIVE_REGULATION_OF_T_CELL_DIFFERENTIATION_IN_THYMUS	-0.44	0	0	0	0	not significant
5789	TUOMISTO_TUMOR_SUPPRESSION_BY_COL13A1_UP	-0.43	0.01	0	0	0	not significant
5790	GO_REGULATION_OF_CALCIUM_ION_IMPORT	-0.43	0	0	0	0	not significant
5791	GO_REGULATION_OF_REMOVAL_OF_SUPEROXIDE_RADICALS	-0.43	0	0	0	0	not significant
5792	GO_BETA_2_ADRENERGIC_RECEPTOR_BINDING	-0.42	0	0	0	0	not significant
5793	REACTOME_TICAM1_DEPENDENT_ACTIVATION_OF_IRF3_IRF7	-0.42	0	0	0	0	not significant
5794	GO_DYNEIN_LIGHT_CHAIN_BINDING	-0.41	0	0	0	0	not significant
5795	KUROKAWA_LIVER_CANCER_EARLY_RECURRENCE_UP	-0.41	0	0	0	0	not significant
5796	GO_BLOC_1_COMPLEX	-0.40	0	0	0	0	not significant
5797	GO_MEMBRANE_RAFT_ASSEMBLY	-0.40	0	0	0	0	not significant
5798	GO_REGULATION_OF_LYASE_ACTIVITY	-0.40	0	0	0	0	not significant
5799	GO_MUCOPOLYSACCHARIDE_METABOLIC_PROCESS	-0.39	0	0	0	0	not significant
5800	GO_PARAXIAL_MESODERM_DEVELOPMENT	-0.39	0	0	0	0	not significant
5801	GO_ENDOPLASMIC_RETICULUM_LOCALIZATION	-0.37	0	0	0	0	not significant
5802	GO_CALCINEURIN_COMPLEX	-0.34	0	0	0	0	not significant
5803	GO_NEURAL_RETINA_DEVELOPMENT	0.33	0	0	0	0	not significant
5804	GO_CHEMOSENSORY_BEHAVIOR	0.37	0	0	0	0	not significant
5805	GO_COENZYME_A_BIOSYNTHETIC_PROCESS	0.37	0	0	0	0	not significant
5806	PID_RHODOPSIN_PATHWAY	0.37	0	0	0	0	not significant
5807	CHR11P14	0.38	0	0	0	0	not significant
5808	GO_FACIAL_NERVE_MORPHOGENESIS	0.38	0	0	0	0	not significant
5809	GO_N_ACETYLGLUCOSAMINE_METABOLIC_PROCESS	0.38	0	0	0	0	not significant
5810	GO_POSITIVE_REGULATION_OF_GLUTAMATE_SECRETION	0.38	0	0	0	0	not significant
5811	GO_REGULATION_OF_GLUTAMATE_SECRETION	0.38	0	0	0	0	not significant
5812	GO_GLYCINE_BINDING	0.39	0	0	0	0	not significant
5813	GO_REGULATION_OF_HISTONE_H3_K9_TRIMETHYLATION	0.39	0	0	0	0	not significant
5814	GO_S_ADENOSYLMETHIONINE_METABOLIC_PROCESS	0.39	0	0	0	0	not significant
5815	GO_UV_PROTECTION	0.39	0	0	0	0	not significant
5816	REACTOME_BETA_OXIDATION_OF_OCTANOYL_COA_TO_HEXANOYL_COA	0.39	0	0	0	0	not significant
5817	GO_LIPOPOLYSACCHARIDE_BINDING	0.40	0	0	0	0	not significant
5818	REACTOME_NOTCH4_INTRACELLULAR_DOMAIN_REGULATES_TRANSCRIPTION	0.40	0	0	0	0	not significant
5819	CHR4P11	0.41	0	0	0	0	not significant
5820	GO_DICHOTOMOUS_SUBDIVISION_OF_AN_EPITHELIAL_TERMINAL_UNIT	0.41	0	0	0	0	not significant
5821	GO_PARASYMPATHETIC_NERVOUS_SYSTEM_DEVELOPMENT	0.41	0	0	0	0	not significant
5822	REACTOME_NOTCH2_INTRACELLULAR_DOMAIN_REGULATES_TRANSCRIPTION	0.41	0	0	0	0	not significant
5823	GO_COA_HYDROLASE_ACTIVITY	0.42	0	0	0	0	not significant
5824	GO_H3_HISTONE_ACETYLTRANSFERASE_COMPLEX	0.42	0	0	0	0	not significant
5825	GO_MORPHOGENESIS_OF_AN_ENDOTHELIUM	0.42	0	0	0	0	not significant
5826	HASLINGER_B_CLL_WITH_6Q21_DELETION	0.42	0	0	0	0	not significant
5827	REACTOME_GLYCEROPHOSPHOLIPID_CATABOLISM	0.42	0	0	0	0	not significant
5828	GO_REGULATION_OF_KILLING_OF_CELLS_OF_OTHER_ORGANISM	0.43	0.01	0	0	0	not significant
5829	GO_REGULATION_OF_WNT_SIGNALING_PATHWAY_PLANAR_CELL_POLARITY_PATH	0.43	0.01	0	0	0	not significant
5830	GO_SPHINGOID_BIOSYNTHETIC_PROCESS	0.43	0.01	0	0	0	not significant
5831	GO_OXIDOREDUCTASE_ACTIVITY_ACTING_ON_NAD_P_H_HEME_PROTEIN_AS_ACCI	0.43	0	0	0	0	not significant
5832	GO_PROTEIN_IMPORT_INTO_PEROXISOME_MEMBRANE	0.43	0	0	0	0	not significant
5833	KEGG_PANTOTHENATE_AND_COA_BIOSYNTHESIS	0.43	0	0	0	0	not significant
5834	SUMI_HNF4A_TARGETS	0.43	0	0	0	0	not significant
5835	GO_CARBONATE_DEHYDRATASE_ACTIVITY	0.44	0.01	0	0	0	not significant
5836	GO_NEURAL_PLATE_PATTERN_SPECIFICATION	0.44	0.01	0	0	0	not significant
5837	GO_OLIGOSACCHARIDE_BIOSYNTHETIC_PROCESS	0.44	0.01	0	0	0	not significant
5838	GO_ATPASE_ACTIVITY_COUPLED_TO_TRANSMEMBRANE_MOVEMENT_OF_IONS_PH	0.44	0	0	0	0	not significant
5839	GO_MYELIN_MAINTENANCE	0.45	0.01	0	0	0	not significant
5840	HUMMERICH_MALIGNANT_SKIN_TUMOR_UP	0.45	0.01	0	0	0	not significant
5841	KRAS.50_UP.V1_UP	0.45	0.01	0	0	0	not significant
5842	EBAUER_MYOGENIC_TARGETS_OF_PAX3_FOXO1_FUSION	0.45	0	0	0	0	not significant
5843	GO_BETA_CATENIN_TCF_COMPLEX	0.45	0	0	0	0	not significant
5844	LI_PROSTATE_CANCER_EPIGENETIC	0.45	0	0	0	0	not significant
5845	REACTOME_VITAMIN_C_ASCORBATE_METABOLISM	0.45	0	0	0	0	not significant
5846	YANG_BREAST_CANCER_ESR1_DN	0.45	0	0	0	0	not significant
5847	GO_RAC_GUANYL_NUCLEOTIDE_EXCHANGE_FACTOR_ACTIVITY	0.46	0.01	0	0	0	not significant
5848	GO_DETERMINATION_OF_DORSAL_VENTRAL_ASYMMETRY	0.46	0	0	0	0	not significant
5849	SA_TRKA_RECEPTOR	0.46	0	0	0	0	not significant
5850	BIOCARTA_SUMO_PATHWAY	0.47	0.01	0	0	0	not significant
5851	GO_BRANCHING_INVOLVED_IN_SALIVARY_GLAND_MORPHOGENESIS	0.47	0.01	0	0	0	not significant
5852	GO_ESTROGEN_METABOLIC_PROCESS	0.47	0.01	0	0	0	not significant
5853	GO_LIPOPROTEIN_METABOLIC_PROCESS	0.47	0.01	0	0	0	not significant
5854	GO_POLYPEPTIDE_N_ACETYLGALACTOSAMINYLTRANSFERASE_ACTIVITY	0.47	0.01	0	0	0	not significant
5855	GO_POTASSIUM_CHANNEL_INHIBITOR_ACTIVITY	0.47	0.01	0	0	0	not significant
5856	IGLESIAS_E2F_TARGETS_DN	0.47	0.01	0	0	0	not significant
5857	REACTOME_DISSOLUTION_OF_FIBRIN_CLOT	0.47	0.01	0	0	0	not significant
5858	VERNOCHET_ADIPOGENESIS	0.47	0.01	0	0	0	not significant
5859	CHR3Q12	0.47	0	0	0	0	not significant
5860	GO_EMBRYONIC_DIGIT_MORPHOGENESIS	0.47	0	0	0	0	not significant
5861	GO_EMBRYONIC_EYE_MORPHOGENESIS	0.47	0	0	0	0	not significant
5862	GO_REGULATION_OF_CYCLASE_ACTIVITY	0.47	0	0	0	0	not significant
5863	TORCHIA_TARGETS_OF_EWSR1_FLI1_FUSION_TOP20_UP	0.47	0	0	0	0	not significant
5864	GO_ACTIN_FILAMENT_BASED_TRANSPORT	0.48	0.01	0	0	0	not significant
5865	GO_CELLULAR_SODIUM_ION_HOMEOSTASIS	0.48	0.01	0	0	0	not significant
5866	GO_POSITIVE_REGULATION_OF_HEART_CONTRACTION	0.48	0.01	0	0	0	not significant
5867	GO_POTASSIUM_ION_ANTIPORTER_ACTIVITY	0.48	0.01	0	0	0	not significant
5868	GO_REGULATION_OF_HEART_MORPHOGENESIS	0.48	0.01	0	0	0	not significant
5869	GO_REGULATION_OF_LYMPHOID_PROGENITOR_CELL_DIFFERENTIATION	0.48	0.01	0	0	0	not significant
5870	GO_SODIUM_ION_EXPORT_ACROSS_PLASMA_MEMBRANE	0.48	0.01	0	0	0	not significant
5871	CHEOK_RESPONSE_TO_MERCAPTOPURINE_AND_LD_MTX_UP	0.49	0.01	0	0	0	not significant
5872	GO_DETECTION_OF_MECHANICAL_STIMULUS_INVOLVED_IN_SENSORY_PERCEPTIC	0.49	0.01	0	0	0	not significant
5873	GO_FILOPODIUM_MEMBRANE	0.49	0.01	0	0	0	not significant
5874	GO_POSITIVE_REGULATION_OF_TRANSCRIPTION_FROM_RNA_POLYMERASE_II_PR	0.49	0.01	0	0	0	not significant

5875	KEGG_HISTIDINE_METABOLISM	0.49	0.01	0	0	0	not significant
5876	REACTOME_SUMO_IS_CONJUGATED_TO_E1_UBA2:SAE1	0.49	0.01	0	0	0	not significant
5877	BAUS_TFF2_TARGETS_UP	0.49	0	0	0	0	not significant
5878	GO_PIGMENT_GRANULE_ORGANIZATION	0.49	0	0	0	0	not significant
5879	GO_ZINC_ION_TRANSPORT	0.49	0	0	0	0	not significant
5880	GO_CELLULAR_RESPONSE_TO_ALDEHYDE	0.50	0.02	0	0	0	not significant
5881	GO_NUCLEOSIDE_TRIPHOSPHATE_CATABOLIC_PROCESS	0.50	0.02	0	0	0	not significant
5882	GO_PURINE_NUCLEOSIDE_CATABOLIC_PROCESS	0.50	0.02	0	0	0	not significant
5883	GO_CALMODULIN_DEPENDENT_PROTEIN_PHOSPHATASE_ACTIVITY	0.50	0.01	0	0	0	not significant
5884	GO_CELLULAR_RESPONSE_TO_ZINC_ION	0.50	0.01	0	0	0	not significant
5885	GO_POSITIVE_REGULATION_OF_CYCLASE_ACTIVITY	0.50	0.01	0	0	0	not significant
5886	GO_PROTEIN_BINDING_BRIDGING_INVOLVED_IN_SUBSTRATE_RECOGNITION_FOR_	0.50	0.01	0	0	0	not significant
5887	GO_INTRACILIARY_TRANSPORT_PARTICLE	0.50	0	0	0	0	not significant
5888	DEBOSSCHER_NFKB_TARGETS_REPRESSED_BY_GLUCOCORTICOIDS	0.51	0.02	0	0	0	not significant
5889	GO_CATION_TRANSPORTING_ATPASE_COMPLEX	0.51	0.02	0	0	0	not significant
5890	GO_MITOCHONDRIAL_DNA_REPAIR	0.51	0.02	0	0	0	not significant
5891	GO_PROTEIN_GERANYLGERANYLTRANSFERASE_ACTIVITY	0.51	0.02	0	0	0	not significant
5892	GO_REGULATION_OF_CELL_CELL_ADHESION_MEDIATED_BY_CADHERIN	0.51	0.02	0	0	0	not significant
5893	GO_REPLICATIVE_CELL_AGING	0.51	0.02	0	0	0	not significant
5894	GO_SODIUM_ION_BINDING	0.51	0.02	0	0	0	not significant
5895	PIEPOLI_LGI1_TARGETS_UP	0.51	0.02	0	0	0	not significant
5896	DUNNE_TARGETS_OF_AML1_MTG8_FUSION_DN	0.51	0.01	0	0	0	not significant
5897	GO_CAMP_BINDING	0.51	0.01	0	0	0	not significant
5898	GO_NADPLUS_BINDING	0.51	0.01	0	0	0	not significant
5899	OZANNE_AP1_TARGETS_UP	0.51	0.01	0	0	0	not significant
5900	REACTOME_BH3_ONLY_PROTEINS_ASSOCIATE_WITH_AND_INACTIVATE_ANTI_APOI	0.51	0.01	0	0	0	not significant
5901	REACTOME_CRMPS_IN_SEMA3A_SIGNALING	0.51	0.01	0	0	0	not significant
5902	REACTOME_PEROXISOMAL_LIPID_METABOLISM	0.51	0.01	0	0	0	not significant
5903	REACTOME_REGULATION_OF_TP53_ACTIVITY_THROUGH_ASSOCIATION_WITH_CO	0.51	0.01	0	0	0	not significant
5904	GO_PYRIMIDINE_NUCLEOSIDE_MONOPHOSPHATE_BIOSYNTHETIC_PROCESS	0.51	0	0	0	0	not significant
5905	GRADE_COLON_CANCER_DN	0.51	0	0	0	0	not significant
5906	GO_LENS_MORPHOGENESIS_IN_CAMERA_TYPE_EYE	0.52	0.02	0	0	0	not significant
5907	GO_PERIPHERAL_NERVOUS_SYSTEM_MYELIN_MAINTENANCE	0.52	0.02	0	0	0	not significant
5908	GO_POSITIVE_REGULATION_OF_VACUOLE_ORGANIZATION	0.52	0.02	0	0	0	not significant
5909	GO_PROTEIN_GERANYLGERANYLATION	0.52	0.02	0	0	0	not significant
5910	GO_RELAXATION_OF_SMOOTH_MUSCLE	0.52	0.02	0	0	0	not significant
5911	GO_POSITIVE_REGULATION_OF_HISTONE_H3_K4_METHYLATION	0.52	0.01	0	0	0	not significant
5912	GO_SERINE_TYPE_EXOPEPTIDASE_ACTIVITY	0.52	0.01	0	0	0	not significant
5913	REACTOME_MOLECULES_ASSOCIATED_WITH_ELASTIC_FIBRES	0.52	0.01	0	0	0	not significant
5914	REACTOME_PENTOSE_PHOSPHATE_PATHWAY	0.52	0.01	0	0	0	not significant
5915	GO_ESTABLISHMENT_OF_EPITHELIAL_CELL_POLARITY	0.52	0	0	0	0	not significant
5916	PICCALUGA_ANGIOIMMUNOBLASTIC_LYMPHOMA_UP	0.52	0	0	0	0	not significant
5917	REACTOME_ION_TRANSPORT_BY_P_TYPE_ATPASES	0.52	0	0	0	0	not significant
5918	GO_DEATH_DOMAIN_BINDING	0.53	0.03	0	0	0	not significant
5919	GO_GENITALIA_MORPHOGENESIS	0.53	0.03	0	0	0	not significant
5920	GO_REGULATION_OF_GROWTH_RATE	0.53	0.03	0	0	0	not significant
5921	SPIELMAN_LYMPHOBLAST_EUROPEAN_VS_ASIAN_2FC_UP	0.53	0.03	0	0	0	not significant
5922	GO_BLOC_COMPLEX	0.53	0.02	0	0	0	not significant
5923	GO_REGULATION_OF_BONE_DEVELOPMENT	0.53	0.02	0	0	0	not significant
5924	GO_RRNA_3_END_PROCESSING	0.53	0.02	0	0	0	not significant
5925	GO_SPHINGOLIPID_TRANSPORTER_ACTIVITY	0.53	0.02	0	0	0	not significant
5926	LEE_NAIVE_T_LYMPHOCYTE	0.53	0.02	0	0	0	not significant
5927	SANDERSON_PPARA_TARGETS	0.53	0.02	0	0	0	not significant
5928	CHR18Q12	0.53	0.01	0	0	0	not significant
5929	GO_GTP_DEPENDENT_PROTEIN_BINDING	0.53	0.01	0	0	0	not significant
5930	GO_REGULATION_OF_GENE_EXPRESSION_BY_GENETIC_IMPRINTING	0.53	0.01	0	0	0	not significant
5931	GO_RELAXATION_OF_MUSCLE	0.53	0.01	0	0	0	not significant
5932	REACTOME_SIGNALING_BY_NOTCH1_HD_DOMAIN_MUTANTS_IN_CANCER	0.53	0.01	0	0	0	not significant
5933	GO_CARDIAC_MUSCLE_CELL_ACTION_POTENTIAL	0.53	0	0	0	0	not significant
5934	GO_LATERAL_PLASMA_MEMBRANE	0.53	0	0	0	0	not significant
5935	REACTOME_PROTEIN_PROTEIN_INTERACTIONS_AT_SYNAPSES	0.53	0	0	0	0	not significant
5936	REACTOME_RUNX1_REGULATES_GENES_INVOLVED_IN_MEGAKARYOCYTE_DIFFER	0.53	0	0	0	0	not significant
5937	GO_HISTONE_METHYLTRANSFERASE_ACTIVITY_H3_K27_SPECIFIC_	0.54	0.03	0	0	0	not significant
5938	GO_REGULATION_OF_CATECHOLAMINE_METABOLIC_PROCESS	0.54	0.03	0	0	0	not significant
5939	GO_MRNA_EDITING_COMPLEX	0.54	0.02	0	0	0	not significant
5940	GO_NEGATIVE_REGULATION_OF_IMMUNOGLOBULIN_PRODUCTION	0.54	0.02	0	0	0	not significant
5941	GO_NEGATIVE_REGULATION_OF_LYASE_ACTIVITY	0.54	0.02	0	0	0	not significant
5942	GO_POSITIVE_REGULATION_OF_CARDIAC_MUSCLE_CELL_DIFFERENTIATION	0.54	0.02	0	0	0	not significant
5943	GO_PROTEIN_PRENYLTRANSFERASE_ACTIVITY	0.54	0.02	0	0	0	not significant
5944	GO_PULMONARY_VALVE_DEVELOPMENT	0.54	0.02	0	0	0	not significant
5945	OXFORD_RALB_TARGETS_DN	0.54	0.02	0	0	0	not significant
5946	REACTOME_OLFACTORY_SIGNALING_PATHWAY	0.54	0.02	0	0	0	not significant
5947	GO_EMBRYONIC_CAMERA_TYPE_EYE_MORPHOGENESIS	0.54	0.01	0	0	0	not significant
5948	GO_PHENOL_CONTAINING_COMPOUND_METABOLIC_PROCESS	0.54	0.01	0	0	0	not significant
5949	GO_POSITIVE_REGULATION_OF_STRIATED_MUSCLE_CONTRACTION	0.55	0.04	0	0	0	not significant
5950	CHR4P13	0.55	0.03	0	0	0	not significant
5951	GO_COPPER_CHAPERONE_ACTIVITY	0.55	0.03	0	0	0	not significant
5952	GO_PULMONARY_VALVE_MORPHOGENESIS	0.55	0.03	0	0	0	not significant
5953	REACTOME_BETA_OXIDATION_OF_PRISTANOYL_COA	0.55	0.03	0	0	0	not significant
5954	BIOCARTA_EPHA4_PATHWAY	0.55	0.02	0	0	0	not significant
5955	GO_ACTIVATION_OF_MAPKKK_ACTIVITY	0.55	0.02	0	0	0	not significant
5956	GO_CELLULAR_RESPONSE_TO_OXIDISED_LOW_DENSITY_LIPOPROTEIN_PARTICLE	0.55	0.02	0	0	0	not significant
5957	GO_FATTY_ACID_DERIVATIVE_CATABOLIC_PROCESS	0.55	0.02	0	0	0	not significant
5958	GO_INTRACELLULAR_MRNA_LOCALIZATION	0.55	0.02	0	0	0	not significant
5959	GO_LONG_CHAIN_FATTY_ACID_BINDING	0.55	0.02	0	0	0	not significant
5960	GO_NUCLEOTIDE_TRANSMEMBRANE_TRANSPORT	0.55	0.02	0	0	0	not significant
5961	GO_POSITIVE_REGULATION_OF_CARDIOCYTE_DIFFERENTIATION	0.55	0.02	0	0	0	not significant
5962	GO_REGULATION_OF_DEVELOPMENTAL_PIGMENTATION	0.55	0.02	0	0	0	not significant
5963	GO_FAT_SOLUBLE_VITAMIN_METABOLIC_PROCESS	0.55	0.01	0	0	0	not significant
5964	GO_REGULATION_OF_NEURAL_PRECURSOR_CELL_PROLIFERATION	0.55	0	0	0	0	not significant
5965	GO_REGULATION_OF_POSTSYNAPTIC_MEMBRANE_NEUROTRANSMITTER_RECEPT	0.55	0	0	0	0	not significant
5966	PID_AMB2_NEUTROPHILS_PATHWAY	0.55	0	0	0	0	not significant
5967	GO_EXTRACELLULAR_MATRIX_COMPONENT	0.56	0.03	0	0	0	not significant
5968	GO_FATTY_ACID_DERIVATIVE_METABOLIC_PROCESS	0.56	0.03	0	0	0	not significant
5969	GO_MITOCHONDRIAL_INTERMEMBRANE_SPACE_PROTEIN_TRANSPORTER_COMPLI	0.56	0.03	0	0	0	not significant
5970	GO_PENTOSE_PHOSPHATE_SHUNT_NON_OXIDATIVE_BRANCH	0.56	0.03	0	0	0	not significant
5971	GO_PRENYLATION	0.56	0.03	0	0	0	not significant
5972	MIKKELSEN_IPS_LCP_WITH_H3K27ME3	0.56	0.03	0	0	0	not significant
5973	BIOCARTA_HCMV_PATHWAY	0.56	0.02	0	0	0	not significant
5974	GO_ER_ASSOCIATED_MISFOLDED_PROTEIN_CATABOLIC_PROCESS	0.56	0.02	0	0	0	not significant
5975	GO_HETEROCHROMATIN_ORGANIZATION	0.56	0.02	0	0	0	not significant
5976	GO_INNATE_IMMUNE_RESPONSE_IN_MUCOSA	0.56	0.02	0	0	0	not significant
5977	GO_PROTEIN_MATURATION_BY_PROTEIN_FOLDING	0.56	0.02	0	0	0	not significant
5978	GO_REGULATION_OF_MACROPHAGE_DERIVED_FOAM_CELL_DIFFERENTIATION	0.56	0.02	0	0	0	not significant
5979	GO_RNA_POLYMERASE_III_REGULATORY_REGION_DNA_BINDING	0.56	0.02	0	0	0	not significant
5980	GO_TYPE_B_PANCREATIC_CELL_DEVELOPMENT	0.56	0.02	0	0	0	not significant
5981	GO_TYPE_I_INTERFERON_BIOSYNTHETIC_PROCESS	0.56	0.02	0	0	0	not significant
5982	GO_ZINC_ION_TRANSMEMBRANE_TRANSPORT	0.56	0.02	0	0	0	not significant
5983	LIM_MAMMARY_LUMINAL_PROGENITOR_DN	0.56	0.02	0	0	0	not significant

5984	GO_FOREBRAIN_CELL_MIGRATION	0.56	0.01	0	0	0	not significant
5985	MIKKELSEN_NPC_LCP_WITH_H3K4ME3	0.56	0.01	0	0	0	not significant
5986	REACTOME_ENERGY_DEPENDENT_REGULATION_OF_MTOR_BY_LKB1_AMPK	0.56	0.01	0	0	0	not significant
5987	REACTOME_IKK_COMPLEX_RECRUITMENT_MEDIATED_BY_RIP1	0.56	0.01	0	0	0	not significant
5988	REACTOME_NEUREXINS_AND_NEUROLIGINS	0.56	0.01	0	0	0	not significant
5989	WANG_RESPONSE_TO_BEXAROTENE_DN	0.56	0.01	0	0	0	not significant
5990	GO_PERIPHERAL_NERVOUS_SYSTEM_DEVELOPMENT	0.56	0	0	0	0	not significant
5991	GO_PURINE_NUCLEOSIDE_TRIPHOSPHATE_CATABOLIC_PROCESS	0.57	0.04	0	0	0	not significant
5992	KIM_ALL_DISORDERS_DURATION_CORR_UP	0.57	0.04	0	0	0	not significant
5993	BIOCARTA_CFTR_PATHWAY	0.57	0.03	0	0	0	not significant
5994	GO_ATP_TRANSPORT	0.57	0.03	0	0	0	not significant
5995	GO_EKC_KEOPS_COMPLEX	0.57	0.03	0	0	0	not significant
5996	GO_ESTABLISHMENT_OR_MAINTENANCE_OF_TRANSMEMBRANE_ELECTROCHEMICA	0.57	0.03	0	0	0	not significant
5997	GO_RAGE_RECEPTOR_BINDING	0.57	0.03	0	0	0	not significant
5998	REACTOME_BETA_OXIDATION_OF_VERY_LONG_CHAIN_FATTY_ACIDS	0.57	0.03	0	0	0	not significant
5999	REACTOME_TRP_CHANNELS	0.57	0.03	0	0	0	not significant
6000	REACTOME_VITAMIN_D_CALCIFEROL_METABOLISM	0.57	0.03	0	0	0	not significant
6001	GO_APOPTOTIC_PROCESS_INVOLVED_IN_MORPHOGENESIS	0.57	0.02	0	0	0	not significant
6002	GO_EPITHELIAL_CELL_MORPHOGENESIS	0.57	0.02	0	0	0	not significant
6003	GO_GLUCOSE_CATABOLIC_PROCESS	0.57	0.02	0	0	0	not significant
6004	GO_MDA_5_SIGNALING_PATHWAY	0.57	0.02	0	0	0	not significant
6005	GO_MLL3_4_COMPLEX	0.57	0.02	0	0	0	not significant
6006	GO_OSTEOBLAST_DEVELOPMENT	0.57	0.02	0	0	0	not significant
6007	GO_RESPIRATORY_CHAIN_COMPLEX_IV	0.57	0.02	0	0	0	not significant
6008	GO_SPHINGOLIPID_BINDING	0.57	0.02	0	0	0	not significant
6009	GO_TRACHEA_FORMATION	0.57	0.02	0	0	0	not significant
6010	KEGG_FATTY_ACID_METABOLISM	0.57	0.01	0	0	0	not significant
6011	REACTOME_VITAMIN_B5_PANTOTHENATE_METABOLISM	0.57	0.01	0	0	0	not significant
6012	GO_LYSINE_N_METHYLTRANSFERASE_ACTIVITY	0.57	0	0	0	0	not significant
6013	GO_POSITIVE_REGULATION_OF_SYNAPTIC_TRANSMISSION	0.57	0	0	0	0	not significant
6014	GO_REGULATION_OF_NEUROTRANSMITTER_SECRETION	0.57	0	0	0	0	not significant
6015	GO_COENZYME_TRANSPORT	0.58	0.05	0	0	0	not significant
6016	GO_REGULATION_OF_MICROVILLUS_ORGANIZATION	0.58	0.05	0	0	0	not significant
6017	GO_PEPTIDYL_SERINE_AUTOPHOSPHORYLATION	0.58	0.04	0	0	0	not significant
6018	GO_POSITIVE_REGULATION_OF_METHYLATION_DEPENDENT_CHROMATIN_SILENCII	0.58	0.04	0	0	0	not significant
6019	GO_POSTSYNAPTIC_ACTIN_CYTOSKELETON_ORGANIZATION	0.58	0.04	0	0	0	not significant
6020	GO_POSTSYNAPTIC_CYTOSKELETON_ORGANIZATION	0.58	0.04	0	0	0	not significant
6021	GO_ELASTIC_FIBER_ASSEMBLY	0.58	0.03	0	0	0	not significant
6022	GO_METHIONINE_METABOLIC_PROCESS	0.58	0.03	0	0	0	not significant
6023	GO_MRNA_3_UTR_AU_RICH_REGION_BINDING	0.58	0.03	0	0	0	not significant
6024	GO_PONS_DEVELOPMENT	0.58	0.03	0	0	0	not significant
6025	GO_PROTEIN_OXIDATION	0.58	0.03	0	0	0	not significant
6026	GO_REGULATION_OF_ENDOTHELIAL_CELL_DEVELOPMENT	0.58	0.03	0	0	0	not significant
6027	GO_REGULATION_OF_RESPONSE_TO_FOOD	0.58	0.03	0	0	0	not significant
6028	GO_THIOESTERASE_BINDING	0.58	0.03	0	0	0	not significant
6029	GO_ZINC_ION_TRANSMEMBRANE_TRANSPORTER_ACTIVITY	0.58	0.03	0	0	0	not significant
6030	REACTOME_NF_KB_IS_ACTIVATED_AND_SIGNALS_SURVIVAL	0.58	0.03	0	0	0	not significant
6031	GO_GTPASE_ACTIVATING_PROTEIN_BINDING	0.58	0.02	0	0	0	not significant
6032	GO_NEURON_CELLULAR_HOMEOSTASIS	0.58	0.02	0	0	0	not significant
6033	NIELSEN_MALIGNAT_FIBROUS_HISTIOCYTOMA_DN	0.58	0.02	0	0	0	not significant
6034	GO_FIBROBLAST_MIGRATION	0.58	0.01	0	0	0	not significant
6035	GO_REGULATION_OF_MUSCLE_ADAPTATION	0.58	0.01	0	0	0	not significant
6036	GO_LAMELLIPODIUM	0.58	0	0	0	0	not significant
6037	REACTOME_SIRT1_NEGATIVELY_REGULATES_RRNA_EXPRESSION	0.58	0	0	0	0	not significant
6038	CHR13Q33	0.59	0.04	0	0	0	not significant
6039	CHR8P22	0.59	0.04	0	0	0	not significant
6040	GO_ATP_TRANSMEMBRANE_TRANSPORTER_ACTIVITY	0.59	0.04	0	0	0	not significant
6041	GO_CALCIUM_CATION_ANTIPORTER_ACTIVITY	0.59	0.04	0	0	0	not significant
6042	GO_CARDIAC_LEFT_VENTRICLE_MORPHOGENESIS	0.59	0.04	0	0	0	not significant
6043	GO_NEGATIVE_REGULATION_OF_ACTIN_NUCLEATION	0.59	0.04	0	0	0	not significant
6044	GO_OLIGOPEPTIDE_BINDING	0.59	0.04	0	0	0	not significant
6045	BIOCARTA_INFLAM_PATHWAY	0.59	0.03	0	0	0	not significant
6046	GO_EXCRETION	0.59	0.03	0	0	0	not significant
6047	GO_G_RICH_STRAND_TELOMERIC_DNA_BINDING	0.59	0.03	0	0	0	not significant
6048	GO_POSITIVE_REGULATION_OF_BLOOD_PRESSURE	0.59	0.03	0	0	0	not significant
6049	RUAN_RESPONSE_TO_TNF_TROGLITAZONE_UP	0.59	0.03	0	0	0	not significant
6050	GO_BARBED_END_ACTIN_FILAMENT_CAPPING	0.59	0.02	0	0	0	not significant
6051	GO_CYCLIC_NUCLEOTIDE_BINDING	0.59	0.02	0	0	0	not significant
6052	GO_GLYCOPROTEIN_COMPLEX	0.59	0.02	0	0	0	not significant
6053	GO_HISTONE_H3_K4_TRIMETHYLATION	0.59	0.02	0	0	0	not significant
6054	GO_NEGATIVE_REGULATION_OF_EPITHELIAL_CELL_DIFFERENTIATION	0.59	0.02	0	0	0	not significant
6055	GO_SARCOMERE_ORGANIZATION	0.59	0.02	0	0	0	not significant
6056	GO_TELOMERE_MAINTENANCE_VIA_RECOMBINATION	0.59	0.02	0	0	0	not significant
6057	GO_UBIQUITIN_DEPENDENT_PROTEIN_CATABOLIC_PROCESS_VIA_THE_MULTIVESI(	0.59	0.02	0	0	0	not significant
6058	HUANG_FOXA2_TARGETS_DN	0.59	0.02	0	0	0	not significant
6059	REACTOME_EPHRIN_SIGNALING	0.59	0.02	0	0	0	not significant
6060	VALK_AML_WITH_FLT3_ITD	0.59	0.02	0	0	0	not significant
6061	KEEN_RESPONSE_TO_ROSIGLITAZONE_UP	0.59	0.01	0	0	0	not significant
6062	TURASHVILI_BREAST_LOBULAR_CARCINOMA_VS_LOBULAR_NORMAL_UP	0.59	0.01	0	0	0	not significant
6063	GO_EXTRACELLULAR_MATRIX_STRUCTURAL_CONSTITUENT_CONFERRING_TENSIL	0.60	0.05	0	0	0	not significant
6064	GO_INTRINSIC_APOPTOTIC_SIGNALING_PATHWAY_IN_RESPONSE_TO_HYDROGEN_	0.60	0.05	0	0	0	not significant
6065	GO_REGULATION_OF_TYPE_B_PANCREATIC_CELL_DEVELOPMENT	0.60	0.05	0	0	0	not significant
6066	GO_HISTONE_DEMETHYLASE_ACTIVITY_H3_K4_SPECIFIC_	0.60	0.04	0	0	0	not significant
6067	GO_REGULATION_OF_CHROMATIN_ASSEMBLY_OR_DISASSEMBLY	0.60	0.04	0	0	0	not significant
6068	REACTOME_MAPK3_ERK1_ACTIVATION	0.60	0.04	0	0	0	not significant
6069	GO_CELL_CELL_SIGNALING_INVOLVED_IN_CARDIAC_CONDUCTION	0.60	0.03	0	0	0	not significant
6070	GO_HAIR_CELL_DIFFERENTIATION	0.60	0.03	0	0	0	not significant
6071	GO_INTRINSIC_COMPONENT_OF_POSTSYNAPTIC_DENSITY_MEMBRANE	0.60	0.03	0	0	0	not significant
6072	GO_POSITIVE_REGULATION_OF_ARP2_3_COMPLEX_MEDIATED_ACTIN_NUCLEATIO	0.60	0.03	0	0	0	not significant
6073	GO_PROTECTION_FROM_NON_HOMOLOGOUS_END_JOINING_AT_TELOMERE	0.60	0.03	0	0	0	not significant
6074	GO_REGULATION_OF_VASCULAR_ENDOTHELIAL_GROWTH_FACTOR_RECEPTOR_S	0.60	0.03	0	0	0	not significant
6075	LU_TUMOR_VASCULATURE_DN	0.60	0.03	0	0	0	not significant
6076	VERRECCHIA_RESPONSE_TO_TGFB1_C3	0.60	0.03	0	0	0	not significant
6077	DING_LUNG_CANCER_MUTATED_SIGNIFICANTLY	0.60	0.02	0	0	0	not significant
6078	GO_DIVALENT_INORGANIC_CATION_TRANSMEMBRANE_TRANSPORTER_ACTIVITY	0.60	0.02	0	0	0	not significant
6079	GO_INTRINSIC_COMPONENT_OF_PEROXISOMAL_MEMBRANE	0.60	0.02	0	0	0	not significant
6080	GO_POSITIVE_REGULATION_OF_AUTOPHAGY_OF_MITOCHONDRION	0.60	0.02	0	0	0	not significant
6081	GO_REGULATION_OF_PLATELET_DERIVED_GROWTH_FACTOR_RECEPTOR_SIGNA	0.60	0.02	0	0	0	not significant
6082	GO_DIENCEPHALON_DEVELOPMENT	0.60	0.01	0	0	0	not significant
6083	GO_NON_MOTILE_CILIUM_ASSEMBLY	0.60	0.01	0	0	0	not significant
6084	GO_REGULATION_OF_CELLULAR_RESPONSE_TO_DRUG	0.60	0.01	0	0	0	not significant
6085	REACTOME_DEACTIVATION_OF_THE_BETA_CATENIN_TRANSACTIVATING_COMPLE:	0.60	0.01	0	0	0	not significant
6086	GO_ACTIN_FILAMENT_BINDING	0.60	0	0	0	0	not significant
6087	GO_POSITIVE_REGULATION_OF_ION_TRANSMEMBRANE_TRANSPORT	0.60	0	0	0	0	not significant
6088	GO_REGULATION_OF_NEUROTRANSMITTER_RECEPTOR_LOCALIZATION_TO_POST	0.61	0.06	0	0	0	not significant
6089	GO_APICAL_DENDRITE	0.61	0.05	0	0	0	not significant
6090	GO_FATTY_ACYL_COA_METABOLIC_PROCESS	0.61	0.05	0	0	0	not significant
6091	GO_LENS_FIBER_CELL_DEVELOPMENT	0.61	0.05	0	0	0	not significant
6092	GO_NEGATIVE_REGULATION_OF_DNA_ENDOREDUPLICATION	0.61	0.05	0	0	0	not significant

6093	DASU_IL6_SIGNALING_SCAR_DN	0.61	0.04	0	0	0	not significant
6094	DAZARD_UV_RESPONSE_CLUSTER_G5	0.61	0.04	0	0	0	not significant
6095	GO_CELLULAR_RESPONSE_TO_NITROGEN_STARVATION	0.61	0.04	0	0	0	not significant
6096	GO_NEGATIVE_REGULATION_OF_OXIDATIVE_STRESS_INDUCED_INTRINSIC_APOPT	0.61	0.04	0	0	0	not significant
6097	GO_POSITIVE_REGULATION_OF_CHONDROCYTE_DIFFERENTIATION	0.61	0.04	0	0	0	not significant
6098	GO_POSITIVE_REGULATION_OF_DOUBLE_STRAND_BREAK_REPAIR_VIA_NONHOMO	0.61	0.04	0	0	0	not significant
6099	GO_PROTEIN_ACTIVATION_CASCADE	0.61	0.04	0	0	0	not significant
6100	GO_REGULATION_OF_PHOSPHOLIPID_BIOSYNTHETIC_PROCESS	0.61	0.04	0	0	0	not significant
6101	IIZUKA_LIVER_CANCER_PROGRESSION_L0_L1_UP	0.61	0.04	0	0	0	not significant
6102	REACTOME_COLLAGEN_CHAIN_TRIMERIZATION	0.61	0.04	0	0	0	not significant
6103	GO_DEOXYRIBONUCLEOSIDE_TRIPHOSPHATE_CATABOLIC_PROCESS	0.61	0.03	0	0	0	not significant
6104	GO_NEGATIVE_REGULATION_OF_TUMOR_NECROSIS_FACTOR_MEDIATED_SIGNALI	0.61	0.03	0	0	0	not significant
6105	GO_POSITIVE_REGULATION_OF_CHROMATIN_SILENCING	0.61	0.03	0	0	0	not significant
6106	GO_REGULATION_OF_DNA_DAMAGE_CHECKPOINT	0.61	0.03	0	0	0	not significant
6107	NABA_COLLAGENS	0.61	0.03	0	0	0	not significant
6108	REACTOME_TRANSLOCATION_OF_ZAP_70_TO_IMMUNOLOGICAL_SYNAPSE	0.61	0.03	0	0	0	not significant
6109	CHR2Q24	0.61	0.02	0	0	0	not significant
6110	GO_LYSINE_ACETYLATED_HISTONE_BINDING	0.61	0.02	0	0	0	not significant
6111	GO_POSITIVE_REGULATION_OF_CELL_JUNCTION_ASSEMBLY	0.61	0.02	0	0	0	not significant
6112	GO_PROTEIN_QUALITY_CONTROL_FOR_MISFOLDED_OR_INCOMPLETELY_SYNTHE	0.61	0.02	0	0	0	not significant
6113	GO_POSITIVE_REGULATION_OF_DNA_BINDING	0.61	0.01	0	0	0	not significant
6114	GO_PRIMARY_ALCOHOL_METABOLIC_PROCESS	0.61	0.01	0	0	0	not significant
6115	ZHAN_MULTIPLE_MYELOMA_CD1_VS_CD2_DN	0.61	0.01	0	0	0	not significant
6116	GO_CHANGES_TO_DNA_METHYLATION_INVOLVED_IN_EMBRYO_DEVELOPMENT	0.62	0.06	0	0	0	not significant
6117	GO_ICOSANOID_RECEPTOR_ACTIVITY	0.62	0.06	0	0	0	not significant
6118	REACTOME_BETA_OXIDATION_OF_HEXANOYL_COA_TO_BUTANOYL_COA	0.62	0.06	0	0	0	not significant
6119	REACTOME_CROSSLINKING_OF_COLLAGEN_FIBRILS	0.62	0.06	0	0	0	not significant
6120	REACTOME_INTRINSIC_PATHWAY_OF_FIBRIN_CLOT_FORMATION	0.62	0.06	0	0	0	not significant
6121	CASTELLANO_HRAS_AND_NRAS_TARGETS_DN	0.62	0.05	0	0	0	not significant
6122	GO_EATING_BEHAVIOR	0.62	0.05	0	0	0	not significant
6123	GO_HISTONE_H2A_K119_MONOUBIQUITINATION	0.62	0.05	0	0	0	not significant
6124	GO_ENDOSOME_TO_LYSOSOME_TRANSPORT_VIA_MULTIVESICULAR_BODY_SORT	0.62	0.04	0	0	0	not significant
6125	GO_MALE_MEIOSIS_I	0.62	0.04	0	0	0	not significant
6126	GO_NEGATIVE_REGULATION_OF_HISTONE_H3_K9_METHYLATION	0.62	0.04	0	0	0	not significant
6127	GO_RNA_SPLICING_VIA_ENDONUCLEOLYTIC_CLEAVAGE_AND_LIGATION	0.62	0.04	0	0	0	not significant
6128	CLASPER_LYMPHATIC_VESSELS_DURING_METASTASIS_DN	0.62	0.03	0	0	0	not significant
6129	GO_MAST_CELL_GRANULE	0.62	0.03	0	0	0	not significant
6130	GO_PURINE_CONTAINING_COMPOUND_SALVAGE	0.62	0.03	0	0	0	not significant
6131	CHR3P24	0.62	0.02	0	0	0	not significant
6132	GO_ENDOPLASMIC_RETICULUM_CALCIUM_ION_HOMEOSTASIS	0.62	0.02	0	0	0	not significant
6133	GO_NUCLEAR_MIGRATION	0.62	0.02	0	0	0	not significant
6134	GO_PEPTIDYL_CYSTEINE_MODIFICATION	0.62	0.02	0	0	0	not significant
6135	YU_BAP1_TARGETS	0.62	0.02	0	0	0	not significant
6136	GO_RHO_GUANYL_NUCLEOTIDE_EXCHANGE_FACTOR_ACTIVITY	0.62	0.01	0	0	0	not significant
6137	GO_METALLOPEPTIDASE_ACTIVITY	0.62	0	0	0	0	not significant
6138	GO_REGULATION_OF_NEUROTRANSMITTER_TRANSPORT	0.62	0	0	0	0	not significant
6139	GO_C_TERMINAL_PROTEIN_LIPIDATION	0.63	0.06	0	0	0	not significant
6140	GO_CARNITINE_O_ACYLTRANSFERASE_ACTIVITY	0.63	0.06	0	0	0	not significant
6141	GO_COENZYME_TRANSMEMBRANE_TRANSPORTER_ACTIVITY	0.63	0.06	0	0	0	not significant
6142	GO_FIBROBLAST_ACTIVATION	0.63	0.06	0	0	0	not significant
6143	GO_NEGATIVE_REGULATION_OF_HELICASE_ACTIVITY	0.63	0.06	0	0	0	not significant
6144	GO_THALAMUS_DEVELOPMENT	0.63	0.06	0	0	0	not significant
6145	IIZUKA_LIVER_CANCER_PROGRESSION_G1_G2_UP	0.63	0.06	0	0	0	not significant
6146	REACTOME_FCGR_ACTIVATION	0.63	0.06	0	0	0	not significant
6147	GO_BLOOD_COAGULATION_INTRINSIC_PATHWAY	0.63	0.05	0	0	0	not significant
6148	GO_BRANCHING_INVOLVED_IN_LABYRINTHINE_LAYER_MORPHOGENESIS	0.63	0.05	0	0	0	not significant
6149	GO_DIPHOSPHOTRANSFERASE_ACTIVITY	0.63	0.05	0	0	0	not significant
6150	GO_NK_T_CELL_DIFFERENTIATION	0.63	0.05	0	0	0	not significant
6151	GO_REGULATION_OF_POTASSIUM_ION_IMPORT	0.63	0.05	0	0	0	not significant
6152	GO_REGULATION_OF_SKELETAL_MUSCLE_CELL_DIFFERENTIATION	0.63	0.05	0	0	0	not significant
6153	GO_REGULATION_OF_T_CELL_CHEMOTAXIS	0.63	0.05	0	0	0	not significant
6154	GO_SULFUR_AMINO_ACID_CATABOLIC_PROCESS	0.63	0.05	0	0	0	not significant
6155	REACTOME_ACETYLCHOLINE_REGULATES_INSULIN_SECRETION	0.63	0.05	0	0	0	not significant
6156	STEGER_ADIPOGENESIS_DN	0.63	0.05	0	0	0	not significant
6157	STEGMEIER_PRE-MITOTIC_CELL_CYCLE_REGULATORS	0.63	0.05	0	0	0	not significant
6158	GO_CHEMOKINE_RECEPTOR_BINDING	0.63	0.04	0	0	0	not significant
6159	GO_FATTY_ACID_BETA_OXIDATION_USING_ACYL_COA_OXIDASE	0.63	0.04	0	0	0	not significant
6160	GO_NEGATIVE_REGULATION_OF_EMBRYONIC_DEVELOPMENT	0.63	0.04	0	0	0	not significant
6161	GO_TUMOR_NECROSIS_FACTOR_SECRETION	0.63	0.04	0	0	0	not significant
6162	KYNG_NORMAL_AGING_UP	0.63	0.04	0	0	0	not significant
6163	CHR12Q12	0.63	0.03	0	0	0	not significant
6164	GO_DNA_REPLICATION_ORIGIN_BINDING	0.63	0.03	0	0	0	not significant
6165	GO_NEGATIVE_REGULATION_OF_MUSCLE_HYPERTROPHY	0.63	0.03	0	0	0	not significant
6166	GO_POSITIVE_REGULATION_OF_BMP_SIGNALING_PATHWAY	0.63	0.03	0	0	0	not significant
6167	GO_PROTEIN_LOCALIZATION_TO_GOLGI_APPARATUS	0.63	0.03	0	0	0	not significant
6168	MEISSNER_BRAIN_ICP_WITH_H3K4ME3	0.63	0.03	0	0	0	not significant
6169	REACTOME_HDMS_DEMETHYLATE_HISTONES	0.63	0.03	0	0	0	not significant
6170	REACTOME_NEGATIVE_REGULATION_OF_MET_ACTIVITY	0.63	0.03	0	0	0	not significant
6171	REACTOME_SYNTHESIS_OF_PIPS_AT_THE_GOLGI_MEMBRANE	0.63	0.03	0	0	0	not significant
6172	GO_BRAIN_MORPHOGENESIS	0.63	0.02	0	0	0	not significant
6173	GO_CELLULAR_RESPONSE_TO_RETINOIC_ACID	0.63	0.02	0	0	0	not significant
6174	GO_EMBRYONIC_SKELETAL_SYSTEM_DEVELOPMENT	0.63	0.01	0	0	0	not significant
6175	GO_VESICLE_MEDIATED_TRANSPORT_IN_SYNAPSE	0.63	0	0	0	0	not significant
6176	GO_NEGATIVE_REGULATION_OF_GLYCOGEN_METABOLIC_PROCESS	0.64	0.07	0	0	0	not significant
6177	GO_CALYX_OF_HELD	0.64	0.06	0	0	0	not significant
6178	GO_MYELIN_SHEATH_ABAXONAL_REGION	0.64	0.06	0	0	0	not significant
6179	GO_POSITIVE_REGULATION_OF_MONOCYTE_DIFFERENTIATION	0.64	0.06	0	0	0	not significant
6180	GO_POSITIVE_REGULATION_OF_NON_CANONICAL_WNT_SIGNALING_PATHWAY	0.64	0.06	0	0	0	not significant
6181	GO_SCAR_COMPLEX	0.64	0.06	0	0	0	not significant
6182	GO_VCP_NPL4_UFD1_AAA_ATPASE_COMPLEX	0.64	0.06	0	0	0	not significant
6183	REACTOME_SYNTHESIS_OF_PG	0.64	0.06	0	0	0	not significant
6184	GO_MITOTIC_DNA_REPLICATION_CHECKPOINT	0.64	0.05	0	0	0	not significant
6185	GO_POSITIVE_REGULATION_OF_FIBROBLAST_APOPTOTIC_PROCESS	0.64	0.05	0	0	0	not significant
6186	GO_REGULATION_OF_PODOSOME_ASSEMBLY	0.64	0.05	0	0	0	not significant
6187	GO_WNT_SIGNALING_PATHWAY_INVOLVED_IN_MIDBRAIN_DOPAMINERGIC_NEURON	0.64	0.05	0	0	0	not significant
6188	LEIN_LOCALIZED_TO_DISTAL_AND_PROXIMAL_DENDRITES	0.64	0.05	0	0	0	not significant
6189	REACTOME_EPHA_MEDIATED_GROWTH_CONE_COLLAPSE	0.64	0.05	0	0	0	not significant
6190	REACTOME_THE_CANONICAL_RETINOID_CYCLE_IN_RODS_TWILIGHT_VISION	0.64	0.05	0	0	0	not significant
6191	STEGMEIER_PREMITOTIC_CELL_CYCLE_REGULATORS	0.64	0.05	0	0	0	not significant
6192	GO_CHAPERONE_MEDIATED_AUTOPHAGY	0.64	0.04	0	0	0	not significant
6193	GO_CYCLIC_NUCLEOTIDE_PHOSPHODIESTERASE_ACTIVITY	0.64	0.04	0	0	0	not significant
6194	POS_RESPONSE_TO_HISTAMINE_UP	0.64	0.04	0	0	0	not significant
6195	REACTOME_EARLY_PHASE_OF_HIV_LIFE_CYCLE	0.64	0.04	0	0	0	not significant
6196	REACTOME_O_GLYCOSYLATION_OF_TSR_DOMAIN_CONTAINING_PROTEINS	0.64	0.04	0	0	0	not significant
6197	MCCABE_HOXC6_TARGETS_CANCER_DN	0.64	0.03	0	0	0	not significant
6198	PID_EPHA_FWDPATHWAY	0.64	0.03	0	0	0	not significant
6199	GO_REGULATION_OF_BMP_SIGNALING_PATHWAY	0.64	0.02	0	0	0	not significant
6200	GO_REGULATION_OF_CENTROSOME_DUPLICATION	0.64	0.02	0	0	0	not significant
6201	CHR3Q13	0.64	0.01	0	0	0	not significant

6202	CHR7P22	0.64	0.01	0	0	0	not significant
6203	GO_ANDROGEN_RECEPTOR_BINDING	0.64	0.01	0	0	0	not significant
6204	MIKKELSEN_ES_ICP_WITH_H3K27ME3	0.65	0.07	0	0	0	not significant
6205	REACTOME_UNBLOCKING_OF_NMDA_RECEPTORS_GLUTAMATE_BINDING_AND_AC	0.65	0.07	0	0	0	not significant
6206	GO_ACETYLCHOLINE_GATED_CHANNEL_COMPLEX	0.65	0.06	0	0	0	not significant
6207	GO_CEREBELLAR_PURKINJE_CELL_LAYER_FORMATION	0.65	0.06	0	0	0	not significant
6208	GO_FIBRINOLYSIS	0.65	0.06	0	0	0	not significant
6209	GO_GAMMA_CATENIN_BINDING	0.65	0.06	0	0	0	not significant
6210	GO_NEGATIVE_REGULATION_OF_OSTEOBLAST_PROLIFERATION	0.65	0.06	0	0	0	not significant
6211	GO_POSITIVE_REGULATION_OF_EXECUTION_PHASE_OF_APOPTOSIS	0.65	0.06	0	0	0	not significant
6212	GO_REGULATION_OF_MITOTIC_RECOMBINATION	0.65	0.06	0	0	0	not significant
6213	GO_REGULATION_OF_NEUTROPHIL_DEGRANULATION	0.65	0.06	0	0	0	not significant
6214	HASINA_NOL7_TARGETS_DN	0.65	0.06	0	0	0	not significant
6215	CHR16Q13	0.65	0.05	0	0	0	not significant
6216	GO_POSITIVE_REGULATION_OF_RECEPTOR_BIOSYNTHETIC_PROCESS	0.65	0.05	0	0	0	not significant
6217	GO_REGULATION_OF_APOPTOTIC_DNA_FRAGMENTATION	0.65	0.05	0	0	0	not significant
6218	GO_SEMINIFEROUS_TUBULE_DEVELOPMENT	0.65	0.05	0	0	0	not significant
6219	NAKAJIMA_EOSINOPHIL	0.65	0.05	0	0	0	not significant
6220	GO_REGULATION_OF_FIBROBLAST_MIGRATION	0.65	0.04	0	0	0	not significant
6221	PID_BETA_CATENIN_DEG_PATHWAY	0.65	0.04	0	0	0	not significant
6222	REACTOME_BUTYRATE_RESPONSE_FACTOR_1_BRF1_BINDS_AND_DESTABILIZES_	0.65	0.04	0	0	0	not significant
6223	REACTOME_ROLE_OF_PHOSPHOLIPIDS_IN_PHAGOCYTOSIS	0.65	0.04	0	0	0	not significant
6224	WALLACE_JAK2_TARGETS_UP	0.65	0.04	0	0	0	not significant
6225	CHANGOLKAR_H2AFY_TARGETS_UP	0.65	0.03	0	0	0	not significant
6226	GO_H4_H2A_HISTONE_ACETYLTRANSFERASE_COMPLEX	0.65	0.03	0	0	0	not significant
6227	GO_HISTONE_DEMETHYLASE_ACTIVITY	0.65	0.03	0	0	0	not significant
6228	GO_LONG_TERM_SYNAPTIC_POTENTIATION	0.65	0.03	0	0	0	not significant
6229	GO_NEGATIVE_REGULATION_OF_ORGANELLE_ASSEMBLY	0.65	0.03	0	0	0	not significant
6230	GO_POSITIVE_REGULATION_OF_MITOCHONDRIAL_OUTER_MEMBRANE_PERMEABIL	0.65	0.03	0	0	0	not significant
6231	KEGG_REGULATION_OF_AUTOPHAGY	0.65	0.03	0	0	0	not significant
6232	CHR8Q22	0.65	0.02	0	0	0	not significant
6233	GO_CELL_REDOX_HOMEOSTASIS	0.65	0.02	0	0	0	not significant
6234	GO_EXTRINSIC_COMPONENT_OF_CYTOPLASMIC_SIDE_OF_PLASMA_MEMBRANE	0.65	0.01	0	0	0	not significant
6235	GO_SPECIFICATION_OF_SYMMETRY	0.65	0.01	0	0	0	not significant
6236	GO_SYNAPTIC_VESICLE_EXOCYTOSIS	0.65	0.01	0	0	0	not significant
6237	GO_MELANOSOME_MEMBRANE	0.66	0.08	0	0	0	not significant
6238	GO_GROWTH_PLATE_CARTILAGE_CHONDROCYTE_DIFFERENTIATION	0.66	0.07	0	0	0	not significant
6239	GO_POSITIVE_REGULATION_OF_GLIAL_CELL_MIGRATION	0.66	0.07	0	0	0	not significant
6240	GO_SYNAPTIC_VESICLE_DOCKING	0.66	0.07	0	0	0	not significant
6241	DE_YY1_TARGETS_UP	0.66	0.06	0	0	0	not significant
6242	GO_AUTOPHAGY_OF_PEROXISOME	0.66	0.06	0	0	0	not significant
6243	GO_MIDBRAIN_DOPAMINERGIC_NEURON_DIFFERENTIATION	0.66	0.06	0	0	0	not significant
6244	GO_NEGATIVE_REGULATION_OF_RIG_I_SIGNALING_PATHWAY	0.66	0.06	0	0	0	not significant
6245	GO_NEGATIVE_REGULATION_OF_TRANSCRIPTION_REGULATORY_REGION_DNA_BI	0.66	0.06	0	0	0	not significant
6246	GO_NEGATIVE_REGULATION_OF_VIRAL_INDUCED_CYTOPLASMIC_PATTERN_RECO	0.66	0.06	0	0	0	not significant
6247	GO_POSITIVE_REGULATION_OF_MITOCHONDRIAL_CALCIUM_ION_CONCENTRATION	0.66	0.06	0	0	0	not significant
6248	KAMIKUBO_MYELOID_MN1_NETWORK	0.66	0.06	0	0	0	not significant
6249	MYLLYKANGAS_AMPLIFICATION_HOT_SPOT_13	0.66	0.06	0	0	0	not significant
6250	REACTOME_PROTON_COUPLED_MONOCARBOXYLATE_TRANSPORT	0.66	0.06	0	0	0	not significant
6251	CHR20P12	0.66	0.05	0	0	0	not significant
6252	CHR2P24	0.66	0.05	0	0	0	not significant
6253	CHR6Q16	0.66	0.05	0	0	0	not significant
6254	GO_CELL_FATE_COMMITMENT_INVOLVED_IN_FORMATION_OF_PRIMARY_GERM_LA	0.66	0.05	0	0	0	not significant
6255	GO_MITOCHONDRIA_ASSOCIATED_ENDOPLASMIC_RETICULUM_MEMBRANE	0.66	0.05	0	0	0	not significant
6256	GO_NUCLEOSIDE_KINASE_ACTIVITY	0.66	0.05	0	0	0	not significant
6257	MADAN_DPPA4_TARGETS	0.66	0.05	0	0	0	not significant
6258	ZHANG_RESPONSE_TO_CANTHARIDIN_UP	0.66	0.05	0	0	0	not significant
6259	CHR12P11	0.66	0.04	0	0	0	not significant
6260	GO_AU_RICH_ELEMENT_BINDING	0.66	0.04	0	0	0	not significant
6261	GO_ERBB2_SIGNALING_PATHWAY	0.66	0.04	0	0	0	not significant
6262	GO_GAMMA_TUBULIN_BINDING	0.66	0.04	0	0	0	not significant
6263	GO_POLYSACCHARIDE_CATABOLIC_PROCESS	0.66	0.04	0	0	0	not significant
6264	GO_SWR1_COMPLEX	0.66	0.04	0	0	0	not significant
6265	KEGG_PROTEIN_EXPORT	0.66	0.04	0	0	0	not significant
6266	REACTOME_GENERATION_OF_SECOND_MESSENGER_MOLECULES	0.66	0.04	0	0	0	not significant
6267	GO_GOLGI_TO_PLASMA_MEMBRANE_PROTEIN_TRANSPORT	0.66	0.03	0	0	0	not significant
6268	GO_NEURON_PROJECTION_REGENERATION	0.66	0.03	0	0	0	not significant
6269	GO_CELLULAR_PIGMENTATION	0.66	0.02	0	0	0	not significant
6270	GO_NEGATIVE_REGULATION_OF_TOR_SIGNALING	0.66	0.02	0	0	0	not significant
6271	GO_RNA_POLYMERASE_II_REPRESSING_TRANSCRIPTION_FACTOR_BINDING	0.66	0.02	0	0	0	not significant
6272	GO_SNARE_COMPLEX	0.66	0.02	0	0	0	not significant
6273	GO_ATPASE_COMPLEX	0.66	0.01	0	0	0	not significant
6274	GO_TUBE_FORMATION	0.66	0.01	0	0	0	not significant
6275	BENPORATH_NOS_TARGETS	0.66	0	0	0	0	not significant
6276	GO_ADHERENS_JUNCTION_ORGANIZATION	0.66	0	0	0	0	not significant
6277	CHANG_POU5F1_TARGETS_UP	0.67	0.08	0	0	0	not significant
6278	GO_HISTONE_H3_K4_DEMETHYLATION	0.67	0.08	0	0	0	not significant
6279	GO_3_HYDROXYACYL_COA_DEHYDROGENASE_ACTIVITY	0.67	0.07	0	0	0	not significant
6280	GO_CORPUS_CALLOSUM_MORPHOGENESIS	0.67	0.07	0	0	0	not significant
6281	GO_LOCOMOTORY_EXPLORATION_BEHAVIOR	0.67	0.07	0	0	0	not significant
6282	GO_NEGATIVE_REGULATION_OF_HEMATOPOIETIC_PROGENITOR_CELL_DIFFEREN	0.67	0.07	0	0	0	not significant
6283	GO_POSITIVE_REGULATION_OF_TUMOR_NECROSIS_FACTOR_BIOSYNTHETIC_PRO	0.67	0.07	0	0	0	not significant
6284	GO_RAP_PROTEIN_SIGNAL_TRANSDUCTION	0.67	0.07	0	0	0	not significant
6285	GO_RELAXATION_OF_CARDIAC_MUSCLE	0.67	0.07	0	0	0	not significant
6286	REACTOME_TRIGLYCERIDE_BIOSYNTHESIS	0.67	0.07	0	0	0	not significant
6287	GO_3_5_CYCLIC_GMP_PHOSPHODIESTERASE_ACTIVITY	0.67	0.06	0	0	0	not significant
6288	GO_CELLULAR_RESPONSE_TO_THYROID_HORMONE_STIMULUS	0.67	0.06	0	0	0	not significant
6289	GO_NEGATIVE_REGULATION_OF_HEART_CONTRACTION	0.67	0.06	0	0	0	not significant
6290	GO_POSITIVE_REGULATION_OF_EXCITATORY_POSTSYNAPTIC_POTENTIAL	0.67	0.06	0	0	0	not significant
6291	GO_POSITIVE_REGULATION_OF_MITOCHONDRIAL_FISSION	0.67	0.06	0	0	0	not significant
6292	GO_POSITIVE_REGULATION_OF_PODOSOME_ASSEMBLY	0.67	0.06	0	0	0	not significant
6293	GO_POSITIVE_REGULATION_OF_TRIGLYCERIDE_BIOSYNTHETIC_PROCESS	0.67	0.06	0	0	0	not significant
6294	GO_REGULATION_OF_TOLERANCE_INDUCTION	0.67	0.06	0	0	0	not significant
6295	BOYAULT_LIVER_CANCER_SUBCLASS_G3_DN	0.67	0.05	0	0	0	not significant
6296	FRASOR_RESPONSE_TO_SERM_OR_FULVESTRANT_UP	0.67	0.05	0	0	0	not significant
6297	GO_COPI_COATED_VESICLE_MEMBRANE	0.67	0.05	0	0	0	not significant
6298	GO_NEGATIVE_REGULATION_OF_ENDOPLASMIC_RETICULUM_STRESS_INDUCED_IN	0.67	0.05	0	0	0	not significant
6299	GO_PENTOSE_PHOSPHATE_SHUNT	0.67	0.05	0	0	0	not significant
6300	NIELSEN_LIPOSARCOMA_DN	0.67	0.05	0	0	0	not significant
6301	REACTOME_TICAM1_RIP1_MEDIATED_IKK_COMPLEX_RECRUITMENT	0.67	0.05	0	0	0	not significant
6302	CHR14Q21	0.67	0.04	0	0	0	not significant
6303	GO_ACETYLGALACTOSAMINYLTRANSFERASE_ACTIVITY	0.67	0.04	0	0	0	not significant
6304	GO_INTRACELLULAR_LIPID_TRANSPORT	0.67	0.04	0	0	0	not significant
6305	GO_POSITIVE_REGULATION_OF_NEURAL_PRECURSOR_CELL_PROLIFERATION	0.67	0.04	0	0	0	not significant
6306	GO_SEMAPHORIN_PLEXIN_SIGNALING_PATHWAY	0.67	0.04	0	0	0	not significant
6307	GO_DORSAL_VENTRAL_PATTERN_FORMATION	0.67	0.03	0	0	0	not significant
6308	GO_REGULATION_OF_ERYTHROCYTE_DIFFERENTIATION	0.67	0.03	0	0	0	not significant
6309	GO_MUSCLE_ADAPTATION	0.67	0.02	0	0	0	not significant
6310	GO_NEGATIVE_REGULATION_OF_DNA_REPLICATION	0.67	0.02	0	0	0	not significant

6311	GO_POSITIVE_REGULATION_OF_CELL_CYCLE_G1_S_PHASE_TRANSITION	0.67	0.02	0	0	0	not significant
6312	BOUDOUKHA_BOUND_BY_IGF2BP2	0.67	0.01	0	0	0	not significant
6313	GO_REGULATION_OF_CILIUM_MOVEMENT	0.68	0.09	0	0	0	not significant
6314	KANG_GLIS3_TARGETS	0.68	0.09	0	0	0	not significant
6315	GO_POSITIVE_REGULATION_OF_TUMOR_NECROSIS_FACTOR_SECRETION	0.68	0.08	0	0	0	not significant
6316	GO_REGULATION_OF_CARDIAC_MUSCLE_CELL_MEMBRANE_REPOLARIZATION	0.68	0.08	0	0	0	not significant
6317	GO_SYNAPTIC_TRANSMISSION_CHOLINERGIC	0.68	0.08	0	0	0	not significant
6318	BYSTRYKH_HEMATOPOIESIS_STEM_CELL_FLI1	0.68	0.07	0	0	0	not significant
6319	CHR7P21	0.68	0.07	0	0	0	not significant
6320	GO_ASPARTATE_TRANSMEMBRANE_TRANSPORT	0.68	0.07	0	0	0	not significant
6321	GO_ENTRY_OF_BACTERIUM_INTO_HOST_CELL	0.68	0.07	0	0	0	not significant
6322	GO_POSITIVE_REGULATION_OF_HETEROTYPIC_CELL_CELL_ADHESION	0.68	0.07	0	0	0	not significant
6323	GO_REGULATION_OF_CILIUM_BEAT_FREQUENCY	0.68	0.07	0	0	0	not significant
6324	BIOCARTA_SPPA_PATHWAY	0.68	0.06	0	0	0	not significant
6325	GO_INNER_EAR_AUDITORY_RECEPTOR_CELL_DIFFERENTIATION	0.68	0.06	0	0	0	not significant
6326	GO_SYNTAXIN_1_BINDING	0.68	0.06	0	0	0	not significant
6327	PUJANA_BREAST_CANCER_WITH_BRCA1_MUTATED_DN	0.68	0.06	0	0	0	not significant
6328	REACTOME_PECAM1_INTERACTIONS	0.68	0.06	0	0	0	not significant
6329	DARWICHE_PAPILLOMA_RISK_HIGH_VS_LOW_DN	0.68	0.05	0	0	0	not significant
6330	GO_INTERCALATED_DISC	0.68	0.05	0	0	0	not significant
6331	MEISSNER_NPC_ICP_WITH_H3K4ME3	0.68	0.05	0	0	0	not significant
6332	CHR4Q35	0.68	0.04	0	0	0	not significant
6333	GO_POSITIVE_REGULATION_OF_CALCIUM_MEDIATED_SIGNALING	0.68	0.04	0	0	0	not significant
6334	PETRETTO_CARDIAC_HYPERTROPHY	0.68	0.04	0	0	0	not significant
6335	BALDWIN_PRKCI_TARGETS_UP	0.68	0.03	0	0	0	not significant
6336	CHRXQ22	0.68	0.03	0	0	0	not significant
6337	GO_DISULFIDE_OXIDOREDUCTASE_ACTIVITY	0.68	0.03	0	0	0	not significant
6338	GO_HOMOTYPIC_CELL_CELL_ADHESION	0.68	0.03	0	0	0	not significant
6339	PID_PI3KCI_PATHWAY	0.68	0.03	0	0	0	not significant
6340	GO_ESTABLISHMENT_OF_PROTEIN_LOCALIZATION_TO_PLASMA_MEMBRANE	0.68	0.02	0	0	0	not significant
6341	GO_SITE_OF_DNA_DAMAGE	0.68	0.02	0	0	0	not significant
6342	IWANAGA_CARCINOGENESIS_BY_KRAS_UP	0.68	0.01	0	0	0	not significant
6343	NOTCH_DN.V1_UP	0.68	0.01	0	0	0	not significant
6344	CHR8Q23	0.69	0.09	0	0	0	not significant
6345	GO_MACROPHAGE_CYTOKINE_PRODUCTION	0.69	0.09	0	0	0	not significant
6346	GO_NEGATIVE_REGULATION_OF_AUTOPHAGY_OF_MITOCHONDRION	0.69	0.09	0	0	0	not significant
6347	GO_NOTOCHORD_DEVELOPMENT	0.69	0.09	0	0	0	not significant
6348	GO_PLATELET_DENSE_GRANULE_LUMEN	0.69	0.09	0	0	0	not significant
6349	GO_ANTEROGRADE_AXONAL_PROTEIN_TRANSPORT	0.69	0.08	0	0	0	not significant
6350	GO_EARLY_PHAGOSOME	0.69	0.08	0	0	0	not significant
6351	GO_EMBRYONIC_BRAIN_DEVELOPMENT	0.69	0.08	0	0	0	not significant
6352	GO_NEGATIVE_REGULATION_OF_PROTEIN_K63_LINKED_UBIQUITINATION	0.69	0.08	0	0	0	not significant
6353	GO_POSITIVE_REGULATION_OF_NEUROBLAST_PROLIFERATION	0.69	0.08	0	0	0	not significant
6354	GO_REGULATION_OF_ARP2_3_COMPLEX_MEDIATED_ACTIN_NUCLEATION	0.69	0.08	0	0	0	not significant
6355	HESSON_TUMOR_SUPPRESSOR_CLUSTER_3P21_3	0.69	0.08	0	0	0	not significant
6356	MATZUK_EARLY_ANTRAL_FOLLICLE	0.69	0.08	0	0	0	not significant
6357	REACTOME_SYNTHESIS_OF_PIPS_AT_THE_ER_MEMBRANE	0.69	0.08	0	0	0	not significant
6358	ELLWOOD_MYC_TARGETS_UP	0.69	0.07	0	0	0	not significant
6359	GO_3_PHOSPHOADENOSINE_5_PHOSPHOSULFATE_METABOLIC_PROCESS	0.69	0.07	0	0	0	not significant
6360	GO_CUL2_RING_UBIQUITIN_LIGASE_COMPLEX	0.69	0.07	0	0	0	not significant
6361	GO_HISTONE_MRNA_CATABOLIC_PROCESS	0.69	0.07	0	0	0	not significant
6362	GO_NEGATIVE_REGULATION_OF_GLIOGENESIS	0.69	0.07	0	0	0	not significant
6363	GO_NEGATIVE_REGULATION_OF_PROTEIN_ACETYLATION	0.69	0.07	0	0	0	not significant
6364	GO_PATTERN_RECOGNITION_RECEPTOR_ACTIVITY	0.69	0.07	0	0	0	not significant
6365	GO_POSITIVE_REGULATION_OF_CENTROSOME_CYCLE	0.69	0.07	0	0	0	not significant
6366	GO_RNA_DNA_HYBRID_RIBONUCLEASE_ACTIVITY	0.69	0.07	0	0	0	not significant
6367	REACTOME_RA_BIOSYNTHESIS_PATHWAY	0.69	0.07	0	0	0	not significant
6368	WANG_NFKB_TARGETS	0.69	0.07	0	0	0	not significant
6369	CHR11Q22	0.69	0.06	0	0	0	not significant
6370	GO_ACTIVIN_RECEPTOR_SIGNALING_PATHWAY	0.69	0.06	0	0	0	not significant
6371	GO_FOAM_CELL_DIFFERENTIATION	0.69	0.06	0	0	0	not significant
6372	GO_GLUCOSYLTRANSFERASE_ACTIVITY	0.69	0.06	0	0	0	not significant
6373	GO_MITOCHONDRIAL_PROTEIN_PROCESSING	0.69	0.06	0	0	0	not significant
6374	GO_NECROPTOTIC_SIGNALING_PATHWAY	0.69	0.06	0	0	0	not significant
6375	GO_NEGATIVE_REGULATION_OF_REGULATED_SECRETORY_PATHWAY	0.69	0.06	0	0	0	not significant
6376	GO_PHOSPHATIDYLCHOLINE_BIOSYNTHETIC_PROCESS	0.69	0.06	0	0	0	not significant
6377	GO_PTERIDINE_CONTAINING_COMPOUND_BIOSYNTHETIC_PROCESS	0.69	0.06	0	0	0	not significant
6378	GRAHAM_CML_QUIESCENT_VS_NORMAL_QUIESCENT_DN	0.69	0.06	0	0	0	not significant
6379	REACTOME_ACTIVATED_NOTCH1_TRANSMITS_SIGNAL_TO_THE_NUCLEUS	0.69	0.06	0	0	0	not significant
6380	REACTOME_METABOLISM_OF_PORPHYRINS	0.69	0.06	0	0	0	not significant
6381	REACTOME_REGULATION_OF_IFNA_SIGNALING	0.69	0.06	0	0	0	not significant
6382	GO_MULTICELLULAR_ORGANISM_AGING	0.69	0.05	0	0	0	not significant
6383	GO_POSITIVE_REGULATION_OF_G1_S_TRANSITION_OF_MITOTIC_CELL_CYCLE	0.69	0.05	0	0	0	not significant
6384	GO_PROTEIN_LOCALIZATION_TO_MICROTUBULE_ORGANIZING_CENTER	0.69	0.05	0	0	0	not significant
6385	MARTIN_NFKB_TARGETS_UP	0.69	0.05	0	0	0	not significant
6386	LIANG_HEMATOPOIESIS_STEM_CELL_NUMBER_SMALL_VS_HUGE_UP	0.69	0.04	0	0	0	not significant
6387	ULE_SPLICING_VIA_NOVA2	0.69	0.04	0	0	0	not significant
6388	GO_CELLULAR_RESPONSE_TO_MECHANICAL_STIMULUS	0.69	0.03	0	0	0	not significant
6389	REACTOME_PKMTS_METHYLATE_HISTONE_LYSINES	0.69	0.03	0	0	0	not significant
6390	GO_CELL_SUBSTRATE_JUNCTION_ASSEMBLY	0.69	0.02	0	0	0	not significant
6391	GO_NUCLEOSIDE_PHOSPHATE_CATABOLIC_PROCESS	0.69	0.02	0	0	0	not significant
6392	GO_PML_BODY	0.69	0.02	0	0	0	not significant
6393	GO_SH3_DOMAIN_BINDING	0.69	0.02	0	0	0	not significant
6394	ACEVEDO_LIVER_CANCER_WITH_H3K27ME3_DN	0.69	0.01	0	0	0	not significant
6395	GO_GUANYL_NUCLEOTIDE_EXCHANGE_FACTOR_ACTIVITY	0.69	0	0	0	0	not significant
6396	GO_AXON_INITIAL_SEGMENT	0.70	0.1	0	0	0	not significant
6397	GO_FACIAL_NERVE_STRUCTURAL_ORGANIZATION	0.70	0.1	0	0	0	not significant
6398	GO_PURINE_DEOXYRIBONUCLEOTIDE_CATABOLIC_PROCESS	0.70	0.1	0	0	0	not significant
6399	GO_EUKARYOTIC_INITIATION_FACTOR_4E_BINDING	0.70	0.09	0	0	0	not significant
6400	GO_FRUCTOSE_1_6_BISPHOSPHATE_METABOLIC_PROCESS	0.70	0.09	0	0	0	not significant
6401	GO_MIRNA_LOADING_ONTO_RISC_INVOLVED_IN_GENE_SILENCING_BY_MIRNA	0.70	0.09	0	0	0	not significant
6402	GO_NEGATIVE_REGULATION_OF_MITOPHAGY	0.70	0.09	0	0	0	not significant
6403	GO_WHITE_FAT_CELL_DIFFERENTIATION	0.70	0.09	0	0	0	not significant
6404	KOINUMA_COLON_CANCER_MSI_UP	0.70	0.09	0	0	0	not significant
6405	PID_S1P_S1P4_PATHWAY	0.70	0.09	0	0	0	not significant
6406	GO_CELLULAR_RESPONSE_TO_TESTOSTERONE_STIMULUS	0.70	0.08	0	0	0	not significant
6407	GO_DENDRITIC_SHAFT	0.70	0.08	0	0	0	not significant
6408	GO_POSITIVE_REGULATION_OF_GLYCOLYTIC_PROCESS	0.70	0.08	0	0	0	not significant
6409	GO_REGULATION_OF_EXIT_FROM_MITOSIS	0.70	0.08	0	0	0	not significant
6410	GO_RETINOL_DEHYDROGENASE_ACTIVITY	0.70	0.08	0	0	0	not significant
6411	REACTOME_ETHANOL_OXIDATION	0.70	0.08	0	0	0	not significant
6412	GO_APICAL_PROTEIN_LOCALIZATION	0.70	0.07	0	0	0	not significant
6413	GO_CYCLIC_NUCLEOTIDE_CATABOLIC_PROCESS	0.70	0.07	0	0	0	not significant
6414	GO_GABA_RECEPTOR_BINDING	0.70	0.07	0	0	0	not significant
6415	GO_POSITIVE_REGULATION_OF_RESPONSE_TO_DRUG	0.70	0.07	0	0	0	not significant
6416	GO_POSITIVE_REGULATION_OF_VASCULAR_ENDOTHELIAL_GROWTH_FACTOR_PR	0.70	0.07	0	0	0	not significant
6417	GO_REGULATION_OF_BILE_ACID_METABOLIC_PROCESS	0.70	0.07	0	0	0	not significant
6418	GO_REGULATION_OF_ENDOCRINE_PROCESS	0.70	0.07	0	0	0	not significant
6419	GO_TELOMERE_MAINTENANCE_IN_RESPONSE_TO_DNA_DAMAGE	0.70	0.07	0	0	0	not significant

6420	HE_PTEN_TARGETS_UP	0.70	0.07	0	0	0	not significant
6421	MIKKELSEN_ES_HCP_WITH_H3_UNMETHYLATED	0.70	0.07	0	0	0	not significant
6422	REACTOME_NEF_MEDIATED_DOWNREGULATION_OF_MHC_CLASS_I_COMPLEX_CE	0.70	0.07	0	0	0	not significant
6423	REACTOME_RUNX2_REGULATES_OSTEOBLAST_DIFFERENTIATION	0.70	0.07	0	0	0	not significant
6424	REACTOME_SMALL_INTERFERING_RNA_SIRNA_BIOGENESIS	0.70	0.07	0	0	0	not significant
6425	VALK_AML_CLUSTER_1	0.70	0.07	0	0	0	not significant
6426	CHR7P13	0.70	0.06	0	0	0	not significant
6427	GO_CARDIAC_MUSCLE_CELL_CONTRACTION	0.70	0.06	0	0	0	not significant
6428	GO_HISTONE_H2A_UBIQUITINATION	0.70	0.06	0	0	0	not significant
6429	GO_NUCLEOTIDE_EXCISION_REPAIR_PREINCISION_COMPLEX_STABILIZATION	0.70	0.06	0	0	0	not significant
6430	GO_TRANSPOSITION	0.70	0.06	0	0	0	not significant
6431	REACTOME_TP53_REGULATES_TRANSCRIPTION_OF_ADDITIONAL_CELL_CYCLE_G	0.70	0.06	0	0	0	not significant
6432	YAP1_DN	0.70	0.06	0	0	0	not significant
6433	BRIDEAU_IMPRINTED_GENES	0.70	0.05	0	0	0	not significant
6434	GO_NUCLEAR_TRANSCRIBED_MRNA_POLY_A_TAIL_SHORTENING	0.70	0.05	0	0	0	not significant
6435	GO_REGULATION_OF_HUMORAL_IMMUNE_RESPONSE	0.70	0.05	0	0	0	not significant
6436	GO_SCHAFFER_COLLATERAL__CA1_SYNAPSE	0.70	0.05	0	0	0	not significant
6437	KRAS.AMP.LUNG_UP.V1_UP	0.70	0.05	0	0	0	not significant
6438	BOYLAN_MULTIPLE_MYELOMA_C_UP	0.70	0.04	0	0	0	not significant
6439	GO_POSITIVE_REGULATION_OF_CELL_SUBSTRATE_ADHESION	0.70	0.04	0	0	0	not significant
6440	GAZDA_DIAMOND_BLACKFAN_ANEMIA_PROGENITOR_DN	0.70	0.03	0	0	0	not significant
6441	GO_ACTION_POTENTIAL	0.70	0.03	0	0	0	not significant
6442	GO_HISTONE_H4_ACETYLATION	0.70	0.03	0	0	0	not significant
6443	GO_RETINA_DEVELOPMENT_IN_CAMERA_TYPE_EYE	0.70	0.02	0	0	0	not significant
6444	TAKAO_RESPONSE_TO_UVB_RADIATION_UP	0.70	0.01	0	0	0	not significant
6445	CREIGHTON_ENDOCRINE_THERAPY_RESISTANCE_2	0.70	0	0	0	0	not significant
6446	GO_CELL_LEADING_EDGE	0.70	0	0	0	0	not significant
6447	GO_CRANIAL_NERVE_STRUCTURAL_ORGANIZATION	0.71	0.1	0	0	0	not significant
6448	GO_NADP_METABOLIC_PROCESS	0.71	0.1	0	0	0	not significant
6449	GO_NEGATIVE_REGULATION_OF_PROTEIN_POLYUBIQUITINATION	0.71	0.1	0	0	0	not significant
6450	GO_PINOSOME	0.71	0.1	0	0	0	not significant
6451	GO_POTASSIUM_TRANSPORTING_ATPASE_ACTIVITY	0.71	0.1	0	0	0	not significant
6452	GO_RNA_POLYMERASE_III_GENERAL_TRANSCRIPTION_INITIATION_FACTOR_ACTIVI	0.71	0.1	0	0	0	not significant
6453	CHEOK_RESPONSE_TO_MERCAPTOPURINE_AND_LD_MTX_DN	0.71	0.09	0	0	0	not significant
6454	DELACROIX_RAR_TARGETS_DN	0.71	0.09	0	0	0	not significant
6455	GO_ALCOHOL_DEHYDROGENASE_NADPPLUS_ACTIVITY	0.71	0.09	0	0	0	not significant
6456	GO_CALCIUM_DEPENDENT_PROTEIN_SERINE_THREONINE_PHOSPHATASE_ACTIVIT	0.71	0.09	0	0	0	not significant
6457	GO_POSITIVE_REGULATION_OF_ER_ASSOCIATED_UBIQUITIN_DEPENDENT_PROTEI	0.71	0.09	0	0	0	not significant
6458	GO_PROTEIN_HORMONE_RECEPTOR_ACTIVITY	0.71	0.09	0	0	0	not significant
6459	GO_SODIUM_POTASSIUM_EXCHANGING_ATPASE_COMPLEX	0.71	0.09	0	0	0	not significant
6460	PID_ALK2_PATHWAY	0.71	0.09	0	0	0	not significant
6461	REACTOME_SEMA4D_MEDIATED_INHIBITION_OF_CELL_ATTACHMENT_AND_MIGRAT	0.71	0.09	0	0	0	not significant
6462	SMID_BREAST_CANCER_RELAPSE_IN_LUNG_UP	0.71	0.09	0	0	0	not significant
6463	BIOCARTA_PLC_PATHWAY	0.71	0.08	0	0	0	not significant
6464	GO_CELLULAR_RESPONSE_TO_ETHANOL	0.71	0.08	0	0	0	not significant
6465	GO_FATTY_ACID_LIGASE_ACTIVITY	0.71	0.08	0	0	0	not significant
6466	GO_I_SMAD_BINDING	0.71	0.08	0	0	0	not significant
6467	GO_POSITIVE_REGULATION_OF_ASPARTIC_TYPE_PEPTIDASE_ACTIVITY	0.71	0.08	0	0	0	not significant
6468	GO_POSITIVE_REGULATION_OF_EXTRINSIC_APOPTOTIC_SIGNALING_PATHWAY_IN	0.71	0.08	0	0	0	not significant
6469	GO_PROTEIN_LOCALIZATION_TO_PRESYNAPSE	0.71	0.08	0	0	0	not significant
6470	GO_SINGLE_STRAND_BREAK_REPAIR	0.71	0.08	0	0	0	not significant
6471	HAN_JNK_SINGALING_DN	0.71	0.08	0	0	0	not significant
6472	PID_ECADHERIN_KERATINOCYTE_PATHWAY	0.71	0.08	0	0	0	not significant
6473	REACTOME_TRANSCRIPTION_OF_E2F_TARGETS_UNDER_NEGATIVE_CONTROL_BY	0.71	0.08	0	0	0	not significant
6474	SA_PTEN_PATHWAY	0.71	0.08	0	0	0	not significant
6475	ZEMBUTSU_SENSITIVITY_TO_METHOTREXATE	0.71	0.08	0	0	0	not significant
6476	GARGALOVIC_RESPONSE_TO_OXIDIZED_PHOSPHOLIPIDS_CYAN_UP	0.71	0.07	0	0	0	not significant
6477	GO_MICROTUBULE_ANCHORING_AT_MICROTUBULE_ORGANIZING_CENTER	0.71	0.07	0	0	0	not significant
6478	GO_RECEPTOR_SIGNALING_COMPLEX_SCAFFOLD_ACTIVITY	0.71	0.07	0	0	0	not significant
6479	KEGG_PENTOSE_PHOSPHATE_PATHWAY	0.71	0.07	0	0	0	not significant
6480	VALK_AML_CLUSTER_8	0.71	0.07	0	0	0	not significant
6481	GO_INTRINSIC_COMPONENT_OF_POSTSYNAPTIC_SPECIALIZATION_MEMBRANE	0.71	0.06	0	0	0	not significant
6482	GO_NUCLEAR_OUTER_MEMBRANE	0.71	0.06	0	0	0	not significant
6483	BIOCARTA_EDG1_PATHWAY	0.71	0.05	0	0	0	not significant
6484	CHR15Q21	0.71	0.05	0	0	0	not significant
6485	EHLERS_ANEUPLOIDY_UP	0.71	0.05	0	0	0	not significant
6486	GO_MULTICELLULAR_ORGANISMAL_RESPONSE_TO_STRESS	0.71	0.05	0	0	0	not significant
6487	GO_POSITIVE_REGULATION_OF_WOUND_HEALING	0.71	0.05	0	0	0	not significant
6488	GO_RENAL_SYSTEM_PROCESS	0.71	0.05	0	0	0	not significant
6489	NIKOLSKY_BREAST_CANCER_7P22_AMPLICON	0.71	0.05	0	0	0	not significant
6490	GO_DENDRITIC_SPINE_DEVELOPMENT	0.71	0.04	0	0	0	not significant
6491	GO_MOLTING_CYCLE	0.71	0.04	0	0	0	not significant
6492	GO_PCG_PROTEIN_COMPLEX	0.71	0.04	0	0	0	not significant
6493	GO_ROOF_OF_MOUTH_DEVELOPMENT	0.71	0.04	0	0	0	not significant
6494	GO_SENSORY_PERCEPTION_OF_PAIN	0.71	0.04	0	0	0	not significant
6495	GO_VESICLE_DOCKING	0.71	0.04	0	0	0	not significant
6496	HUTTMANN_B_CLL_POOR_SURVIVAL_DN	0.71	0.04	0	0	0	not significant
6497	GO_ACTIVATION_OF_GTPASE_ACTIVITY	0.71	0.03	0	0	0	not significant
6498	GO_OXIDOREDUCTASE_COMPLEX	0.71	0.03	0	0	0	not significant
6499	GO_REGULATION_OF_INTRACELLULAR_STEROID_HORMONE_RECEPTOR_SIGNALIN	0.71	0.03	0	0	0	not significant
6500	GO_RUFFLE_MEMBRANE	0.71	0.03	0	0	0	not significant
6501	GO_HISTONE_METHYLATION	0.71	0.02	0	0	0	not significant
6502	GO_REGULATION_OF_CELL_SHAPE	0.71	0.02	0	0	0	not significant
6503	GO_REGULATION_OF_MEMBRANE_POTENTIAL	0.71	0	0	0	0	not significant
6504	GO_ACETYLCHOLINE_GATED_CATION_SELECTIVE_CHANNEL_ACTIVITY	0.72	0.11	0	0	0	not significant
6505	GO_DIPEPTIDYL_PEPTIDASE_ACTIVITY	0.72	0.1	0	0	0	not significant
6506	GO_ETHANOL_OXIDATION	0.72	0.1	0	0	0	not significant
6507	GO_NEGATIVE_REGULATION_OF_TRANSCRIPTION_ELONGATION_FROM_RNA_POLY	0.72	0.1	0	0	0	not significant
6508	GO_PEPTIDYL_LYSINE_HYDROXYLATION	0.72	0.1	0	0	0	not significant
6509	GO_POSITIVE_REGULATION_OF_METALLOPEPTIDASE_ACTIVITY	0.72	0.1	0	0	0	not significant
6510	TAGHAVI_NEOPLASTIC_TRANSFORMATION	0.72	0.1	0	0	0	not significant
6511	GO_CELLULAR_RESPONSE_TO_INTERFERON_BETA	0.72	0.09	0	0	0	not significant
6512	GO_PRENYLTRANSFERASE_ACTIVITY	0.72	0.09	0	0	0	not significant
6513	GO_REGULATION_OF_ISOTYPE_SWITCHING_TO_IGE_ISOTYPES	0.72	0.09	0	0	0	not significant
6514	GO_REGULATION_OF_MICROTUBULE_DEPOLYMERIZATION	0.72	0.09	0	0	0	not significant
6515	GO_REGULATION_OF_PEROXISOME_PROLIFERATOR_ACTIVATED_RECEPTOR_SIG	0.72	0.09	0	0	0	not significant
6516	GO_WNT_SIGNALOSOME	0.72	0.09	0	0	0	not significant
6517	ZHOU_CELL_CYCLE_GENES_IN_IR_RESPONSE_2HR	0.72	0.09	0	0	0	not significant
6518	GO_ANTIMICROBIAL_HUMORAL_IMMUNE_RESPONSE_MEDIATED_BY_ANTIMICROBIAL	0.72	0.08	0	0	0	not significant
6519	GO_PYRUVATE_METABOLIC_PROCESS	0.72	0.08	0	0	0	not significant
6520	GO_REGULATION_OF_HETEROTYPIC_CELL_CELL_ADHESION	0.72	0.08	0	0	0	not significant
6521	GO_TRANSFORMING_GROWTH_FACTOR_BETA_BINDING	0.72	0.08	0	0	0	not significant
6522	HASLINGER_B_CLL_WITH_11Q23_DELETION	0.72	0.08	0	0	0	not significant
6523	REACTOME_DISEASES_ASSOCIATED_WITH_N_GLYCOSYLATION_OF_PROTEINS	0.72	0.08	0	0	0	not significant
6524	REACTOME_NUCLEAR_ENVELOPE_REASSEMBLY	0.72	0.08	0	0	0	not significant
6525	CHR6Q22	0.72	0.07	0	0	0	not significant
6526	GO_AXON_REGENERATION	0.72	0.07	0	0	0	not significant
6527	GO_DNA_REPLICATION_DEPENDENT_NUCLEOSOME_ORGANIZATION	0.72	0.07	0	0	0	not significant
6528	GO_POSITIVE_REGULATION_OF_GLYCOPROTEIN_BIOSYNTHETIC_PROCESS	0.72	0.07	0	0	0	not significant

6529	GO_PROTEIN_LOCALIZATION_TO_POSTSYNAPTIC_MEMBRANE	0.72	0.07	0	0	0	not significant
6530	KORKOLA_YOLK_SAC_TUMOR	0.72	0.07	0	0	0	not significant
6531	GO_HISTONE_H3_K9_METHYLATION	0.72	0.06	0	0	0	not significant
6532	GO_LYSOSOME_LOCALIZATION	0.72	0.06	0	0	0	not significant
6533	GO_OUTFLOW_TRACT_MORPHOGENESIS	0.72	0.06	0	0	0	not significant
6534	KYNG_DNA_DAMAGE_BY_4NQO_OR_UV	0.72	0.06	0	0	0	not significant
6535	PID_HIV_NEF_PATHWAY	0.72	0.06	0	0	0	not significant
6536	COATES_MACROPHAGE_M1_VS_M2_UP	0.72	0.05	0	0	0	not significant
6537	GO_VIRION_ASSEMBLY	0.72	0.05	0	0	0	not significant
6538	SIG_BCR_SIGNALING_PATHWAY	0.72	0.05	0	0	0	not significant
6539	CHR2Q11	0.72	0.04	0	0	0	not significant
6540	GO_BONE_MORPHOGENESIS	0.72	0.04	0	0	0	not significant
6541	GO_CYSTEINE_TYPE_ENDOPEPTIDASE_ACTIVITY	0.72	0.04	0	0	0	not significant
6542	GO_AEROBIC_RESPIRATION	0.72	0.03	0	0	0	not significant
6543	GO_INDOLE_CONTAINING_COMPOUND_METABOLIC_PROCESS	0.73	0.12	0	0	0	not significant
6544	GO_ALPHA_CATENIN_BINDING	0.73	0.11	0	0	0	not significant
6545	GO_FOREBRAIN_REGIONALIZATION	0.73	0.11	0	0	0	not significant
6546	GO_NEGATIVE_REGULATION_OF_MEMBRANE_POTENTIAL	0.73	0.11	0	0	0	not significant
6547	GO_REGULATION_OF_DEVELOPMENT_HETEROCHRONIC	0.73	0.11	0	0	0	not significant
6548	GO_TYPE_I_TRANSFORMING_GROWTH_FACTOR_BETA_RECEPTOR_BINDING	0.73	0.11	0	0	0	not significant
6549	BIOCARTA_HBX_PATHWAY	0.73	0.1	0	0	0	not significant
6550	CHRXQ25	0.73	0.1	0	0	0	not significant
6551	GO_FATTY_ACID_BETA_OXIDATION_USING_ACYL_COA_DEHYDROGENASE	0.73	0.1	0	0	0	not significant
6552	GO_NEGATIVE_REGULATION_OF_ALPHA_BETA_T_CELL_DIFFERENTIATION	0.73	0.1	0	0	0	not significant
6553	GO_POSITIVE_REGULATION_OF_CYTOPLASMIC_MRNA_PROCESSING_BODY_ASSEI	0.73	0.1	0	0	0	not significant
6554	GO_POSITIVE_REGULATION_OF_RECEPTOR_BINDING	0.73	0.1	0	0	0	not significant
6555	GO_REGULATION_OF_DOUBLE_STRAND_BREAK_REPAIR_VIA_NONHOMOLOGOUS_	0.73	0.1	0	0	0	not significant
6556	GO_REGULATION_OF_SENSORY_PERCEPTION	0.73	0.1	0	0	0	not significant
6557	GO_SYNAPTIC_CLEFT	0.73	0.1	0	0	0	not significant
6558	GO_CUL4A_RING_E3_UBIQUITIN_LIGASE_COMPLEX	0.73	0.09	0	0	0	not significant
6559	GO_MICROTUBULE_MINUS_END	0.73	0.09	0	0	0	not significant
6560	GO_NEGATIVE_REGULATION_OF_HISTONE_H3_K9_TRIMETHYLATION	0.73	0.09	0	0	0	not significant
6561	GO_NUCLEOTIDE_EXCISION_REPAIR_COMPLEX	0.73	0.09	0	0	0	not significant
6562	GO_PROTEIN_KINASE_A_SIGNALING	0.73	0.09	0	0	0	not significant
6563	GO_PROTEIN_LOCALIZATION_TO_ENDOSOME	0.73	0.09	0	0	0	not significant
6564	GO_REGULATION_OF_PROTEIN_K63_LINKED_UBIQUITINATION	0.73	0.09	0	0	0	not significant
6565	GO_REGULATION_OF_VITAMIN_METABOLIC_PROCESS	0.73	0.09	0	0	0	not significant
6566	GO_UBIQUITIN_LIKE_MODIFIER_ACTIVATING_ENZYME_ACTIVITY	0.73	0.09	0	0	0	not significant
6567	CHR12Q14	0.73	0.08	0	0	0	not significant
6568	GO_BETA_CATENIN_DESTRUCTION_COMPLEX_DISASSEMBLY	0.73	0.08	0	0	0	not significant
6569	GO_DETERMINATION_OF_ADULT_LIFESPAN	0.73	0.08	0	0	0	not significant
6570	GO_PHOTORECEPTOR_CONNECTING_CILIUM	0.73	0.08	0	0	0	not significant
6571	GO_POSITIVE_REGULATION_OF_NEUROTRANSMITTER_TRANSPORT	0.73	0.08	0	0	0	not significant
6572	GO_PROTEIN_SERINE_THREONINE_KINASE_INHIBITOR_ACTIVITY	0.73	0.08	0	0	0	not significant
6573	GO_QUATERNARY_AMMONIUM_GROUP_BINDING	0.73	0.08	0	0	0	not significant
6574	JIANG_AGING_CEREBRAL_CORTEX_UP	0.73	0.08	0	0	0	not significant
6575	PID_EPHA2_FWD_PATHWAY	0.73	0.08	0	0	0	not significant
6576	REACTOME_ADVANCED_GLYCOSYLATION_ENDPRODUCT_RECEPTOR_SIGNALING	0.73	0.08	0	0	0	not significant
6577	REACTOME_E2F_MEDIATED_REGULATION_OF_DNA_REPLICATION	0.73	0.08	0	0	0	not significant
6578	REACTOME_SYNTHESIS_OF_IP3_AND_IP4_IN_THE_CYTOSOL	0.73	0.08	0	0	0	not significant
6579	REACTOME_TRANSCRIPTION_OF_E2F_TARGETS_UNDER_NEGATIVE_CONTROL_BY	0.73	0.08	0	0	0	not significant
6580	DIRMEIER_LMP1_RESPONSE_LATE_DN	0.73	0.07	0	0	0	not significant
6581	GO_CEREBRAL_CORTEX_CELL_MIGRATION	0.73	0.07	0	0	0	not significant
6582	GO_POSTSYNAPTIC_DENSITY_MEMBRANE	0.73	0.07	0	0	0	not significant
6583	GO_REGULATION_OF_VACUOLE_ORGANIZATION	0.73	0.07	0	0	0	not significant
6584	GO_VASCULAR_ENDOTHELIAL_GROWTH_FACTOR_PRODUCTION	0.73	0.07	0	0	0	not significant
6585	GO_VENTRICULAR_SEPTUM_DEVELOPMENT	0.73	0.07	0	0	0	not significant
6586	GO_VOLTAGE_GATED_POTASSIUM_CHANNEL_ACTIVITY	0.73	0.07	0	0	0	not significant
6587	REACTOME_MITOCHONDRIAL_FATTY_ACID_BETA_OXIDATION	0.73	0.07	0	0	0	not significant
6588	RIGGINS_TAMOXIFEN_RESISTANCE_UP	0.73	0.07	0	0	0	not significant
6589	CHR18P11	0.73	0.06	0	0	0	not significant
6590	GO_MITOCHONDRIAL_FISSION	0.73	0.06	0	0	0	not significant
6591	GO_NEGATIVE_REGULATION_OF_ACTIN_FILAMENT_POLYMERIZATION	0.73	0.06	0	0	0	not significant
6592	GO_TRICARBOXYLIC_ACID_CYCLE	0.73	0.06	0	0	0	not significant
6593	BROWNE_HCMV_INFECTION_20HR_DN	0.73	0.05	0	0	0	not significant
6594	GO_MYELOID_CELL_DEVELOPMENT	0.73	0.05	0	0	0	not significant
6595	GO_REGULATION_OF_DENDRITIC_SPINE_DEVELOPMENT	0.73	0.05	0	0	0	not significant
6596	GO_SEQUESTERING_OF_CALCIUM_ION	0.73	0.04	0	0	0	not significant
6597	PID_MET_PATHWAY	0.73	0.04	0	0	0	not significant
6598	GINESTIER_BREAST_CANCER_20Q13_AMPLIFICATION_UP	0.73	0.03	0	0	0	not significant
6599	GO_CARDIAC_SEPTUM_DEVELOPMENT	0.73	0.03	0	0	0	not significant
6600	GO_CARTILAGE_DEVELOPMENT	0.73	0.03	0	0	0	not significant
6601	GO_TRANSITION_METAL_ION_TRANSPORT	0.73	0.03	0	0	0	not significant
6602	REACTOME_MUSCLE_CONTRACTION	0.73	0.03	0	0	0	not significant
6603	GO_RAS_GUANYL_NUCLEOTIDE_EXCHANGE_FACTOR_ACTIVITY	0.73	0.02	0	0	0	not significant
6604	GO_ACTIN_FILAMENT_POLYMERIZATION	0.73	0.01	0	0	0	not significant
6605	GO_ACTIN_POLYMERIZATION_OR_DEPOLYMERIZATION	0.73	0.01	0	0	0	not significant
6606	GO_INOSITOL_TRISPHOSPHATE_PHOSPHATASE_ACTIVITY	0.74	0.12	0	0	0	not significant
6607	GO_VESICLE_UNCOATING	0.74	0.12	0	0	0	not significant
6608	GO_FILOPODIUM_TIP	0.74	0.11	0	0	0	not significant
6609	GO_HISTONE_METHYLTRANSFERASE_ACTIVITY_H3_K9_SPECIFIC_	0.74	0.11	0	0	0	not significant
6610	GO_NADP_RETINOL_DEHYDROGENASE_ACTIVITY	0.74	0.11	0	0	0	not significant
6611	GO_NODAL_SIGNALING_PATHWAY	0.74	0.11	0	0	0	not significant
6612	GO_REGULATION_OF_CYTOPLASMIC_TRANSLATIONAL_INITIATION	0.74	0.11	0	0	0	not significant
6613	GO_REGULATION_OF_PROTEIN_GLYCOSYLATION	0.74	0.11	0	0	0	not significant
6614	BIOCARTA_CTLA4_PATHWAY	0.74	0.1	0	0	0	not significant
6615	CHR19Q12	0.74	0.1	0	0	0	not significant
6616	GO_CATECHOLAMINE_SECRETION	0.74	0.1	0	0	0	not significant
6617	GO_DEATH_INDUCING_SIGNALING_COMPLEX_ASSEMBLY	0.74	0.1	0	0	0	not significant
6618	GO_HISTONE_DEMETHYLASE_ACTIVITY_H3_K9_SPECIFIC_	0.74	0.1	0	0	0	not significant
6619	GO_NEURONAL_ION_CHANNEL_CLUSTERING	0.74	0.1	0	0	0	not significant
6620	GO_NPBAF_COMPLEX	0.74	0.1	0	0	0	not significant
6621	GO_REGULATION_OF_METALLOENDOPEPTIDASE_ACTIVITY	0.74	0.1	0	0	0	not significant
6622	REACTOME_VOLTAGE_GATED_POTASSIUM_CHANNELS	0.74	0.1	0	0	0	not significant
6623	GO_COLLECTING_DUCT_DEVELOPMENT	0.74	0.09	0	0	0	not significant
6624	GO_ENERGY_HOMEOSTASIS	0.74	0.09	0	0	0	not significant
6625	GO_ENTEROENDOCRINE_CELL_DIFFERENTIATION	0.74	0.09	0	0	0	not significant
6626	GO_INACTIVATION_OF_MAPK_ACTIVITY	0.74	0.09	0	0	0	not significant
6627	GO_METALLOAMINOPEPTIDASE_ACTIVITY	0.74	0.09	0	0	0	not significant
6628	GO_MICROTUBULE_SEVERING	0.74	0.09	0	0	0	not significant
6629	GO_NAD_METABOLIC_PROCESS	0.74	0.09	0	0	0	not significant
6630	GO_NEGATIVE_REGULATION_OF_CD4_POSITIVE_ALPHA_BETA_T_CELL_DIFFEREN	0.74	0.09	0	0	0	not significant
6631	GO_ORGANOPHOSPHATE_ESTER_TRANSMEMBRANE_TRANSPORTER_ACTIVITY	0.74	0.09	0	0	0	not significant
6632	GO_REGULATION_OF_EARLY_ENDOSOME_TO_LATE_ENDOSOME_TRANSPORT	0.74	0.09	0	0	0	not significant
6633	HAHTOLA_MYCOSIS_FUNGOIDES_DN	0.74	0.09	0	0	0	not significant
6634	REACTOME_SUMOYLATION_OF_TRANSCRIPTION_FACTORS	0.74	0.09	0	0	0	not significant
6635	GO_NEGATIVE_REGULATION_OF_COLD_INDUCED_THERMOGENESIS	0.74	0.08	0	0	0	not significant
6636	GO_RESPONSE_TO_X_RAY	0.74	0.08	0	0	0	not significant
6637	LEIN_LOCALIZED_TO_PROXIMAL_DENDRITES	0.74	0.08	0	0	0	not significant

6638	PID_ER_NONGENOMIC_PATHWAY	0.74	0.08	0	0	0	not significant
6639	REACTOME_BRANCHED_CHAIN_AMINO_ACID_CATABOLISM	0.74	0.08	0	0	0	not significant
6640	REACTOME_TAK1_ACTIVATES_NFKB_BY_PHOSPHORYLATION_AND_ACTIVATION_O	0.74	0.08	0	0	0	not significant
6641	HEIDENBLAD_AMPLICON_8Q24_DN	0.74	0.07	0	0	0	not significant
6642	REACTOME_ECM_PROTEOGLYCANS	0.74	0.07	0	0	0	not significant
6643	WOOD_EBV_EBNA1_TARGETS_DN	0.74	0.07	0	0	0	not significant
6644	GO_KINASE_INHIBITOR_ACTIVITY	0.74	0.06	0	0	0	not significant
6645	GO_PROTEIN_LOCALIZATION_TO_CELL_SURFACE	0.74	0.06	0	0	0	not significant
6646	GO_REGULATION_OF_CALCIUM_ION_TRANSPORT_INTO_CYTOSOL	0.74	0.06	0	0	0	not significant
6647	GO_TRANSFERASE_ACTIVITY_TRANSFERRING_PENTOSYL_GROUPS	0.74	0.06	0	0	0	not significant
6648	HEIDENBLAD_AMPLICON_8Q24_UP	0.74	0.06	0	0	0	not significant
6649	GO_POSITIVE_REGULATION_OF_PROTEIN_BINDING	0.74	0.05	0	0	0	not significant
6650	GO_MOTILE_CILIUM	0.74	0.04	0	0	0	not significant
6651	GO_NEGATIVE_REGULATION_OF_CELLULAR_PROTEIN_LOCALIZATION	0.74	0.04	0	0	0	not significant
6652	GO_NEGATIVE_REGULATION_OF_CYTOSKELETON_ORGANIZATION	0.74	0.03	0	0	0	not significant
6653	GO_UBIQUITIN_LIKE_PROTEIN_LIGASE_ACTIVITY	0.74	0.02	0	0	0	not significant
6654	GO_PROTEIN_TARGETING	0.74	0	0	0	0	not significant
6655	GO_AUDITORY_RECEPTOR_CELL_MORPHOGENESIS	0.75	0.13	0	0	0	not significant
6656	GO_ACTIN_POLYMERIZATION_DEPENDENT_CELL_MOTILITY	0.75	0.12	0	0	0	not significant
6657	GO_CELL_SURFACE_RECEPTOR_SIGNALING_PATHWAY_INVOLVED_IN_HEART_DEV	0.75	0.12	0	0	0	not significant
6658	GO_CLATHRIN_HEAVY_CHAIN_BINDING	0.75	0.12	0	0	0	not significant
6659	GO_REGULATION_OF_MICROTUBULE_NUCLEATION	0.75	0.12	0	0	0	not significant
6660	GO_RNA_POLYMERASE_III_TYPE_1_PROMOTER_DNA_BINDING	0.75	0.12	0	0	0	not significant
6661	IKEDA_MIR30_TARGETS_DN	0.75	0.12	0	0	0	not significant
6662	BUDHU_LIVER_CANCER_METASTASIS_UP	0.75	0.11	0	0	0	not significant
6663	FARMER_BREAST_CANCER_CLUSTER_4	0.75	0.11	0	0	0	not significant
6664	GO_CELL_DIFFERENTIATION_IN_HINDBRAIN	0.75	0.11	0	0	0	not significant
6665	GO_CHLORIDE_ION_BINDING	0.75	0.11	0	0	0	not significant
6666	GO_ENDOCARDIAL_CUSHION_MORPHOGENESIS	0.75	0.11	0	0	0	not significant
6667	GO_INTRACILIARY_TRANSPORT_PARTICLE_A	0.75	0.11	0	0	0	not significant
6668	GO_NEGATIVE_REGULATION_OF_ENDOPLASMIC_RETICULUM_CALCIUM_ION_CONC	0.75	0.11	0	0	0	not significant
6669	GO_NEGATIVE_REGULATION_OF_RHO_PROTEIN_SIGNAL_TRANSDUCTION	0.75	0.11	0	0	0	not significant
6670	GO_REGULATION_OF_ANIMAL_ORGAN_FORMATION	0.75	0.11	0	0	0	not significant
6671	GO_REGULATION_OF_STEM_CELL_POPULATION_MAINTENANCE	0.75	0.11	0	0	0	not significant
6672	GO_TRANSLATIONAL_READTHROUGH	0.75	0.11	0	0	0	not significant
6673	GO_VESICLE_TETHERING	0.75	0.11	0	0	0	not significant
6674	REACTOME_CD209_DC_SIGN_SIGNALING	0.75	0.11	0	0	0	not significant
6675	REACTOME_CELLULAR_HEXOSE_TRANSPORT	0.75	0.11	0	0	0	not significant
6676	REACTOME_DOWNREGULATION_OF_ERBB2:ERBB3_SIGNALING	0.75	0.11	0	0	0	not significant
6677	REACTOME_TRIF_MEDIATED_PROGRAMMED_CELL_DEATH	0.75	0.11	0	0	0	not significant
6678	BIOCARTA_PRC2_PATHWAY	0.75	0.1	0	0	0	not significant
6679	BIOCARTA_PTEN_PATHWAY	0.75	0.1	0	0	0	not significant
6680	GO_AMINO_SUGAR_BIOSYNTHETIC_PROCESS	0.75	0.1	0	0	0	not significant
6681	GO_COLLAGEN_FIBRIL_ORGANIZATION	0.75	0.1	0	0	0	not significant
6682	GO_DEOXYRIBOSE_PHOSPHATE_BIOSYNTHETIC_PROCESS	0.75	0.1	0	0	0	not significant
6683	GO_ESTABLISHMENT_OF_PROTEIN_LOCALIZATION_TO_POSTSYNAPTIC_MEMBRAN	0.75	0.1	0	0	0	not significant
6684	GO_MESENCHYME_MORPHOGENESIS	0.75	0.1	0	0	0	not significant
6685	GO_MITOTIC_DNA_REPLICATION	0.75	0.1	0	0	0	not significant
6686	GO_RESPONSE_TO_ATP	0.75	0.1	0	0	0	not significant
6687	REACTOME_PHASE_0_RAPID_DEPOLARISATION	0.75	0.1	0	0	0	not significant
6688	CHR4P14	0.75	0.09	0	0	0	not significant
6689	GO_NCRNA_CATABOLIC_PROCESS	0.75	0.09	0	0	0	not significant
6690	GO_NUCLEOSIDE_SALVAGE	0.75	0.09	0	0	0	not significant
6691	GO_PHOSPHATIDIC_ACID_BIOSYNTHETIC_PROCESS	0.75	0.09	0	0	0	not significant
6692	GO_REGULATION_OF_NEUROBLAST_PROLIFERATION	0.75	0.09	0	0	0	not significant
6693	REACTOME_ELASTIC_FIBRE_FORMATION	0.75	0.09	0	0	0	not significant
6694	GO_G2_DNA_DAMAGE_CHECKPOINT	0.75	0.08	0	0	0	not significant
6695	GO_MYD88_INDEPENDENT_TOLL_LIKE_RECEPTOR_SIGNALING_PATHWAY	0.75	0.08	0	0	0	not significant
6696	GO_REGULATION_OF_LIPID_CATABOLIC_PROCESS	0.75	0.08	0	0	0	not significant
6697	GO_RESPONSE_TO_EPIDERMAL_GROWTH_FACTOR	0.75	0.08	0	0	0	not significant
6698	HUMMEL_BURKITTS_LYMPHOMA_UP	0.75	0.08	0	0	0	not significant
6699	MARTIN_VIRAL_GPCR_SIGNALING_DN	0.75	0.08	0	0	0	not significant
6700	REACTOME_REGULATION_OF_RUNX1_EXPRESSION_AND_ACTIVITY	0.75	0.08	0	0	0	not significant
6701	GO_MESODERM_MORPHOGENESIS	0.75	0.07	0	0	0	not significant
6702	GO_POSITIVE_REGULATION_OF_NEURON_APOPTOTIC_PROCESS	0.75	0.07	0	0	0	not significant
6703	GO_REGULATION_OF_RELEASE_OF_SEQUESTERED_CALCIUM_ION_INTO_CYTOSO	0.75	0.07	0	0	0	not significant
6704	CHR3P22	0.75	0.06	0	0	0	not significant
6705	PKCA_DN.V1_UP	0.75	0.06	0	0	0	not significant
6706	GO_HISTONE_METHYLTRANSFERASE_COMPLEX	0.75	0.04	0	0	0	not significant
6707	GO_MEMBRANE_FUSION	0.75	0.03	0	0	0	not significant
6708	REACTOME_G2_M_DNA_DAMAGE_CHECKPOINT	0.75	0.03	0	0	0	not significant
6709	CHR3P21	0.75	0.02	0	0	0	not significant
6710	GO_UBIQUITIN_LIKE_PROTEIN_TRANSFERASE_ACTIVITY	0.75	0.01	0	0	0	not significant
6711	GO_ANCHORING_JUNCTION	0.75	0	0	0	0	not significant
6712	GO_NEUROTRANSMITTER_RECEPTOR_LOCALIZATION_TO_POSTSYNAPTIC_SPECIA	0.76	0.14	0	0	0	not significant
6713	GO_CELLULAR_RESPONSE_TO_INCREASED_OXYGEN_LEVELS	0.76	0.13	0	0	0	not significant
6714	GO_REGULATION_OF_CILIUM_BEAT_FREQUENCY_INVOLVED_IN_CILIARY_MOTILITY	0.76	0.13	0	0	0	not significant
6715	GO_REGULATION_OF_PROTEIN_COMPLEX_STABILITY	0.76	0.13	0	0	0	not significant
6716	GO_VENTRICULAR_CARDIAC_MUSCLE_CELL_MEMBRANE_REPOLARIZATION	0.76	0.13	0	0	0	not significant
6717	PIEPOLI_LGI1_TARGETS_DN	0.76	0.13	0	0	0	not significant
6718	GO_C4_DICARBOXYLATE_TRANSPORT	0.76	0.12	0	0	0	not significant
6719	GO_CALCIUM_ION_REGULATED_EXOCYTOSIS_OF_NEUROTRANSMITTER	0.76	0.12	0	0	0	not significant
6720	GO_MULTI_ORGANISM_MEMBRANE_ORGANIZATION	0.76	0.12	0	0	0	not significant
6721	GO_POSITIVE_REGULATION_OF_STEROID_BIOSYNTHETIC_PROCESS	0.76	0.12	0	0	0	not significant
6722	GO_PRO_B_CELL_DIFFERENTIATION	0.76	0.12	0	0	0	not significant
6723	GO_REGULATION_OF_CARDIAC_MUSCLE_ADAPTATION	0.76	0.12	0	0	0	not significant
6724	GO_TELOMERE_LOCALIZATION	0.76	0.12	0	0	0	not significant
6725	MCGARVEY_SILENCED_BY_METHYLATION_IN_COLON_CANCER	0.76	0.12	0	0	0	not significant
6726	SHIRAISHI_PLZF_TARGETS_DN	0.76	0.12	0	0	0	not significant
6727	VANDESLUIS_COMMD1_TARGETS_GROUP_3_DN	0.76	0.12	0	0	0	not significant
6728	GO_C_TERMINAL_PROTEIN_AMINO_ACID_MODIFICATION	0.76	0.11	0	0	0	not significant
6729	GO_PHOSPHATIDYLINOSITOL_3_KINASE_COMPLEX_CLASS_I	0.76	0.11	0	0	0	not significant
6730	GO_POSITIVE_REGULATION_OF_POTASSIUM_ION_TRANSMEMBRANE_TRANSPORTE	0.76	0.11	0	0	0	not significant
6731	GO_POSITIVE_REGULATION_OF_STEROID_METABOLIC_PROCESS	0.76	0.11	0	0	0	not significant
6732	GO_TRANSCRIPTION_COACTIVATOR_BINDING	0.76	0.11	0	0	0	not significant
6733	LI_ADIPOGENESIS_BY_ACTIVATED_PPARG	0.76	0.11	0	0	0	not significant
6734	MOOTHA_ROS	0.76	0.11	0	0	0	not significant
6735	REACTOME_DISEASES_OF_MISMATCH_REPAIR_MMR	0.76	0.11	0	0	0	not significant
6736	WONG_ENDMETRIUM_CANCER_DN	0.76	0.11	0	0	0	not significant
6737	BIOCARTA_ATM_PATHWAY	0.76	0.1	0	0	0	not significant
6738	GO_BRANCHED_CHAIN_AMINO_ACID_CATABOLIC_PROCESS	0.76	0.1	0	0	0	not significant
6739	GO_MICROTUBULE_ANCHORING	0.76	0.1	0	0	0	not significant
6740	GO_MODIFICATION_OF_SYNAPTIC_STRUCTURE	0.76	0.1	0	0	0	not significant
6741	GO_POSITIVE_REGULATION_OF_NOTCH_SIGNALING_PATHWAY	0.76	0.1	0	0	0	not significant
6742	KEGG_ALDOSTERONE_REGULATED_SODIUM_REABSORPTION	0.76	0.1	0	0	0	not significant
6743	PID_ARF_3PATHWAY	0.76	0.1	0	0	0	not significant
6744	REACTOME_AKT_PHOSPHORYLATES_TARGETS_IN_THE_CYTOSOL	0.76	0.1	0	0	0	not significant
6745	REACTOME_CD28_DEPENDENT_VAV1_PATHWAY	0.76	0.1	0	0	0	not significant
6746	REACTOME_TRANSCRIPTIONAL_REGULATION_BY_THE_AP_2_TFAP2_FAMILY_OF_T	0.76	0.1	0	0	0	not significant

6747	DELASERNA_MYOD_TARGETS_DN	0.76	0.09	0	0	0	not significant
6748	GO_ENDOSOME_TRANSPORT_VIA_MULTIVESICULAR_BODY_SORTING_PATHWAY	0.76	0.09	0	0	0	not significant
6749	HASLINGER_B_CLL_WITH_CHROMOSOME_12_TRISOMY	0.76	0.09	0	0	0	not significant
6750	MATTIOLI_MULTIPLE_MYELOMA_WITH_14Q32_TRANSLOCATIONS	0.76	0.09	0	0	0	not significant
6751	REACTOME_INTERLEUKIN_10_SIGNALING	0.76	0.09	0	0	0	not significant
6752	GO_CENTRIOLE_ASSEMBLY	0.76	0.08	0	0	0	not significant
6753	GO_DEMETHYLASE_ACTIVITY	0.76	0.08	0	0	0	not significant
6754	GO_ENDODERM_FORMATION	0.76	0.08	0	0	0	not significant
6755	GO_SEX_CHROMOSOME	0.76	0.08	0	0	0	not significant
6756	CHR11P11	0.76	0.07	0	0	0	not significant
6757	GO_ACTIVATION_OF_CYSTEINE_TYPE_ENDOPEPTIDASE_ACTIVITY_INVOLVED_IN_A	0.76	0.07	0	0	0	not significant
6758	GO_NEGATIVE_REGULATION_OF_PEPTIDYL_TYROSINE_PHOSPHORYLATION	0.76	0.07	0	0	0	not significant
6759	PID_TXA2PATHWAY	0.76	0.07	0	0	0	not significant
6760	GINESTIER_BREAST_CANCER_ZNF217_AMPLIFIED_UP	0.76	0.06	0	0	0	not significant
6761	GO_ACTIN_FILAMENT	0.76	0.06	0	0	0	not significant
6762	GO_SNARE_BINDING	0.76	0.05	0	0	0	not significant
6763	GO_CYTOSOLIC_CALCIUM_ION_TRANSPORT	0.76	0.04	0	0	0	not significant
6764	GO_SKIN_DEVELOPMENT	0.76	0.04	0	0	0	not significant
6765	KEGG_FOCAL_ADHESION	0.76	0.04	0	0	0	not significant
6766	GO_TISSUE_HOMEOSTASIS	0.76	0.03	0	0	0	not significant
6767	GO_PROTEIN_METHYLATION	0.76	0.02	0	0	0	not significant
6768	HOLLMANN_APOPTOSIS_VIA_CD40_UP	0.76	0.02	0	0	0	not significant
6769	ABBUD_LIF_SIGNALING_2_UP	0.77	0.13	0	0	0	not significant
6770	EHRLICH_ICF_SYNDROM_DN	0.77	0.13	0	0	0	not significant
6771	GO_AUDITORY_RECEPTOR_CELL_DEVELOPMENT	0.77	0.13	0	0	0	not significant
6772	GO_MULTI_ORGANISM_MEMBRANE_FUSION	0.77	0.13	0	0	0	not significant
6773	GO_REGULATION_OF_DENDRITIC_CELL_CHEMOTAXIS	0.77	0.13	0	0	0	not significant
6774	GO_REGULATION_OF_FATTY_ACID_BETA_OXIDATION	0.77	0.13	0	0	0	not significant
6775	GO_REGULATION_OF_HEART_RATE_BY_CHEMICAL_SIGNAL	0.77	0.13	0	0	0	not significant
6776	GO_REGULATION_OF_SYNAPTIC_VESICLE_ENDOCYTOSIS	0.77	0.13	0	0	0	not significant
6777	GO_SPHINGOSINE_N_ACYLTRANSFERASE_ACTIVITY	0.77	0.13	0	0	0	not significant
6778	GO_SURFACTANT_HOMEOSTASIS	0.77	0.13	0	0	0	not significant
6779	GO_VASCULAR_ENDOTHELIAL_GROWTH_FACTOR_RECEPTOR_2_BINDING	0.77	0.13	0	0	0	not significant
6780	REACTOME_INTERLEUKIN_6_SIGNALING	0.77	0.13	0	0	0	not significant
6781	GO_AUDITORY_RECEPTOR_CELL_STEREOCILIUM_ORGANIZATION	0.77	0.12	0	0	0	not significant
6782	GO_DNA_N_GLYCOSYLASE_ACTIVITY	0.77	0.12	0	0	0	not significant
6783	GO_PEPTIDYL_PROLINE_4_DIOXYGENASE_ACTIVITY	0.77	0.12	0	0	0	not significant
6784	GO POSITIVE REGULATION OF CYTOKINE SECRETION INVOLVED IN IMMUNE RE	0.77	0.12	0	0	0	not significant
6785	GO POSITIVE REGULATION OF LIPOPOLYSACCHARIDE MEDIATED SIGNALING PA	0.77	0.12	0	0	0	not significant
6786	GO_REGULATION_OF_BILE_ACID_BIOSYNTHETIC_PROCESS	0.77	0.12	0	0	0	not significant
6787	GO_REGULATION_OF_CYTOPLASMIC_TRANSPORT	0.77	0.12	0	0	0	not significant
6788	GO_REPLACEMENT_OSSIFICATION	0.77	0.12	0	0	0	not significant
6789	GO_RNA_SURVEILLANCE	0.77	0.12	0	0	0	not significant
6790	KEGG_RETINOL_METABOLISM	0.77	0.12	0	0	0	not significant
6791	MAEKAWA_ATF2_TARGETS	0.77	0.12	0	0	0	not significant
6792	MYLLYKANGAS_AMPLIFICATION_HOT_SPOT_9	0.77	0.12	0	0	0	not significant
6793	STARK_BRAIN_22Q11_DELETION	0.77	0.12	0	0	0	not significant
6794	BIOCARTA_HER2_PATHWAY	0.77	0.11	0	0	0	not significant
6795	GO_LATE_ENDOSOME_TO_VACUOLE_TRANSPORT	0.77	0.11	0	0	0	not significant
6796	GO_LUNG_ALVEOLUS_DEVELOPMENT	0.77	0.11	0	0	0	not significant
6797	GO_MICROVILLUS_ORGANIZATION	0.77	0.11	0	0	0	not significant
6798	GO_OLIGODENDROCYTE_DEVELOPMENT	0.77	0.11	0	0	0	not significant
6799	GO_OLIGOSACCHARIDE_LIPID_INTERMEDIATE_BIOSYNTHETIC_PROCESS	0.77	0.11	0	0	0	not significant
6800	GO_PEROXISOME_FISSION	0.77	0.11	0	0	0	not significant
6801	GO_PHOSPHATIDYLGLYCEROL_ACYL_CHAIN_REMODELING	0.77	0.11	0	0	0	not significant
6802	GO_PHOSPHATIDYLINOSITOL_3_KINASE_ACTIVITY	0.77	0.11	0	0	0	not significant
6803	GO_REGULATION_OF_HISTONE_H3_K9_METHYLATION	0.77	0.11	0	0	0	not significant
6804	GO_VASODILATION	0.77	0.11	0	0	0	not significant
6805	WANG_BARRETTS_ESOPHAGUS_DN	0.77	0.11	0	0	0	not significant
6806	BIOCARTA_CSK_PATHWAY	0.77	0.1	0	0	0	not significant
6807	GO_FAD_BINDING	0.77	0.1	0	0	0	not significant
6808	GO_NEGATIVE_REGULATION_OF_LEUKOCYTE_APOPTOTIC_PROCESS	0.77	0.1	0	0	0	not significant
6809	GO_NEGATIVE_REGULATION_OF_PEPTIDYL_LYSINE_ACETYLATION	0.77	0.1	0	0	0	not significant
6810	GO_POSITIVE_REGULATION_OF_EMBRYONIC_DEVELOPMENT	0.77	0.1	0	0	0	not significant
6811	GO_PROLINE_RICH_REGION_BINDING	0.77	0.1	0	0	0	not significant
6812	GO_REGULATION_OF_LONG_TERM_SYNAPTIC_POTENTIATION	0.77	0.1	0	0	0	not significant
6813	GO_TRANS_GOLGI_NETWORK_TRANSPORT_VESICLE	0.77	0.1	0	0	0	not significant
6814	REACTOME INSERTION OF TAIL ANCHORED PROTEINS INTO THE ENDOPLASMIC	0.77	0.1	0	0	0	not significant
6815	REACTOME_THE_ROLE_OF_NEF_IN_HIV_1_REPLICATION_AND_DISEASE_PATHOGE	0.77	0.1	0	0	0	not significant
6816	SIG_IL4RECEPTOR_IN_B_LYPHOCYTES	0.77	0.1	0	0	0	not significant
6817	CHR9Q21	0.77	0.09	0	0	0	not significant
6818	GO_TERTIARY_GRANULE_MEMBRANE	0.77	0.09	0	0	0	not significant
6819	KEGG_ANTIGEN_PROCESSING_AND_PRESENTATION	0.77	0.09	0	0	0	not significant
6820	CHRXQ28	0.77	0.08	0	0	0	not significant
6821	GO NEGATIVE REGULATION OF CELL MORPHOGENESIS INVOLVED IN DIFFEREN	0.77	0.08	0	0	0	not significant
6822	GO_PROTEIN_TARGETING_TO_VACUOLE	0.77	0.08	0	0	0	not significant
6823	GO_RAC_GTPASE_BINDING	0.77	0.08	0	0	0	not significant
6824	GO_REGULATION_OF_T_CELL_RECEPTOR_SIGNALING_PATHWAY	0.77	0.08	0	0	0	not significant
6825	KEGG_PPAR_SIGNALING_PATHWAY	0.77	0.08	0	0	0	not significant
6826	LIN_APC_TARGETS	0.77	0.08	0	0	0	not significant
6827	GO_CORTICAL_ACTIN_CYTOSKELETON	0.77	0.07	0	0	0	not significant
6828	KEGG_APOPTOSIS	0.77	0.07	0	0	0	not significant
6829	GO_CALCIUM_ION_TRANSMEMBRANE_IMPORT_INTO_CYTOSOL	0.77	0.06	0	0	0	not significant
6830	GO_CORTICAL_CYTOSKELETON	0.77	0.06	0	0	0	not significant
6831	GO_EPITHELIAL_CELL_DEVELOPMENT	0.77	0.06	0	0	0	not significant
6832	KEGG_FC_EPSILON_RI_SIGNALING_PATHWAY	0.77	0.06	0	0	0	not significant
6833	XU_GH1_AUTOCRINE_TARGETS_UP	0.77	0.05	0	0	0	not significant
6834	LINDGREN_BLADDER_CANCER_CLUSTER_2A_DN	0.77	0.04	0	0	0	not significant
6835	GO_HEART_DEVELOPMENT	0.77	0.02	0	0	0	not significant
6836	GO_NUCLEOSIDE_TRIPHOSPHATASE_REGULATOR_ACTIVITY	0.77	0.01	0	0	0	not significant
6837	IVANOVA_HEMATOPOIESIS_LATE_PROGENITOR	0.77	0.01	0	0	0	not significant
6838	GO_COENZYME_A_METABOLIC_PROCESS	0.78	0.14	0	0	0	not significant
6839	GO_PLATELET_DERIVED_GROWTH_FACTOR_RECEPTOR_BINDING	0.78	0.14	0	0	0	not significant
6840	GO_RNA_POLYMERASE_III_PREINITIATION_COMPLEX_ASSEMBLY	0.78	0.14	0	0	0	not significant
6841	GO_SUGAR_TRANSMEMBRANE_TRANSPORTER_ACTIVITY	0.78	0.14	0	0	0	not significant
6842	BIOCARTA_HSP27_PATHWAY	0.78	0.13	0	0	0	not significant
6843	GO_ATP_DEPENDENT_3_5_DNA_HELICASE_ACTIVITY	0.78	0.13	0	0	0	not significant
6844	GO_DENDRITE_MEMBRANE	0.78	0.13	0	0	0	not significant
6845	GO_MALE_SEX_DETERMINATION	0.78	0.13	0	0	0	not significant
6846	GO_MITOGEN_ACTIVATED_PROTEIN_KINASE_P38_BINDING	0.78	0.13	0	0	0	not significant
6847	GO POSITIVE REGULATION OF MAMMARY GLAND EPITHELIAL CELL PROLIFERA	0.78	0.13	0	0	0	not significant
6848	GO_PYRIMIDINE_DIMER_REPAIR	0.78	0.13	0	0	0	not significant
6849	GO_REGULATION_OF_AMYLOID_PRECURSOR_PROTEIN_CATABOLIC_PROCESS	0.78	0.13	0	0	0	not significant
6850	GO_REGULATION_OF_NEUTROPHIL_ACTIVATION	0.78	0.13	0	0	0	not significant
6851	GO_RESPONSE_TO_DIETARY_EXCESS	0.78	0.13	0	0	0	not significant
6852	HAHTOLA_CTCL_CUTANEOUS	0.78	0.13	0	0	0	not significant
6853	REACTOME FATTY ACIDS BOUND TO GPR40 FFAR1 REGULATE INSULIN SECRE	0.78	0.13	0	0	0	not significant
6854	REACTOME_MITOCHONDRIAL_FATTY_ACID_BETA_OXIDATION_OF_SATURATED_FA	0.78	0.13	0	0	0	not significant
6855	WU_HBX_TARGETS_1_UP	0.78	0.13	0	0	0	not significant

6856	BIOCARTA_IL12_PATHWAY	0.78	0.12	0	0	0	not significant
6857	BIOCARTA_SHH_PATHWAY	0.78	0.12	0	0	0	not significant
6858	CORRE_MULTIPLE_MYELOMA_UP	0.78	0.12	0	0	0	not significant
6859	GO_DYNACTIN_COMPLEX	0.78	0.12	0	0	0	not significant
6860	GO POSITIVE REGULATION OF INTRACELLULAR ESTROGEN RECEPTOR SIGNAL	0.78	0.12	0	0	0	not significant
6861	GO_POSITIVE_REGULATION_OF_MULTICELLULAR_ORGANISM_GROWTH	0.78	0.12	0	0	0	not significant
6862	GO_PROSTANOID_BIOSYNTHETIC_PROCESS	0.78	0.12	0	0	0	not significant
6863	KYNG_ENVIRONMENTAL_STRESS_RESPONSE_NOT_BY_UV_IN_WS	0.78	0.12	0	0	0	not significant
6864	PARK_TRETINOIN_RESPONSE_AND_RARA_PLZF_FUSION	0.78	0.12	0	0	0	not significant
6865	REACTOME_ELEVATION_OF_CYTOSOLIC_CA2PLUS_LEVELS	0.78	0.12	0	0	0	not significant
6866	CHR7P15	0.78	0.11	0	0	0	not significant
6867	DAZARD_UV_RESPONSE_CLUSTER_G2	0.78	0.11	0	0	0	not significant
6868	FERRANDO_LYL1_NEIGHBORS	0.78	0.11	0	0	0	not significant
6869	GO_CELLULAR_RESPONSE_TO_ALKALOID	0.78	0.11	0	0	0	not significant
6870	GO_COPPER_ION_BINDING	0.78	0.11	0	0	0	not significant
6871	GO_PEROXISOMAL_MEMBRANE_TRANSPORT	0.78	0.11	0	0	0	not significant
6872	GO_PITUITARY_GLAND_DEVELOPMENT	0.78	0.11	0	0	0	not significant
6873	GO_PROTEIN_K48_LINKED_DEUBIQUITINATION	0.78	0.11	0	0	0	not significant
6874	GO_PYRIMIDINE_CONTAINING_COMPOUND_CATABOLIC_PROCESS	0.78	0.11	0	0	0	not significant
6875	MARIADASON_RESPONSE_TO_BUTYRATE_SULINDAC_4	0.78	0.11	0	0	0	not significant
6876	YANG_BREAST_CANCER_ESR1_BULK_UP	0.78	0.11	0	0	0	not significant
6877	CHR1Q31	0.78	0.1	0	0	0	not significant
6878	GO_ANTIBIOTIC_METABOLIC_PROCESS	0.78	0.1	0	0	0	not significant
6879	GO_PIGMENT_METABOLIC_PROCESS	0.78	0.1	0	0	0	not significant
6880	GO_PYRIDINE_CONTAINING_COMPOUND_METABOLIC_PROCESS	0.78	0.1	0	0	0	not significant
6881	KUNINGER_IGF1_VS_PDGFB_TARGETS_DN	0.78	0.1	0	0	0	not significant
6882	CHR11Q23	0.78	0.09	0	0	0	not significant
6883	GO_PIGMENT_BIOSYNTHETIC_PROCESS	0.78	0.09	0	0	0	not significant
6884	GO_PLATELET_AGGREGATION	0.78	0.09	0	0	0	not significant
6885	GO_VESICLE_MEDIATED_TRANSPORT_BETWEEN_ENDOSOMAL_COMPARTMENTS	0.78	0.09	0	0	0	not significant
6886	LEE_CALORIE_RESTRICTION_NEOCORTEX_UP	0.78	0.09	0	0	0	not significant
6887	LINDSTEDT_DENDRITIC_CELL_MATURATION_D	0.78	0.09	0	0	0	not significant
6888	PETROVA_PROX1_TARGETS_DN	0.78	0.09	0	0	0	not significant
6889	REACTOME_ONCOGENE_INDUCED_SENESCENCE	0.78	0.09	0	0	0	not significant
6890	GO_FORMATION_OF_PRIMARY_GERM_LAYER	0.78	0.08	0	0	0	not significant
6891	GO_REGULATION_OF_OXIDOREDUCTASE_ACTIVITY	0.78	0.08	0	0	0	not significant
6892	REACTOME_ACTIVATION_OF_THE_MRNA_UPON_BINDING_OF_THE_CAP_BINDING_C	0.78	0.08	0	0	0	not significant
6893	SASSON_RESPONSE_TO_GONADOTROPHINS_DN	0.78	0.08	0	0	0	not significant
6894	GO_LIPID_PHOSPHORYLATION	0.78	0.07	0	0	0	not significant
6895	GO_PROTEIN_DEPOLYMERIZATION	0.78	0.07	0	0	0	not significant
6896	GO_REGULATION_OF_SYNAPTIC_VESICLE_CYCLE	0.78	0.07	0	0	0	not significant
6897	GO_CYTOSOLIC_RIBOSOME	0.78	0.06	0	0	0	not significant
6898	GO_REGULATION_OF_VIRAL_LIFE_CYCLE	0.78	0.06	0	0	0	not significant
6899	MIYAGAWA_TARGETS_OF_EWSR1_ETS_FUSIONS_DN	0.78	0.06	0	0	0	not significant
6900	GO_ACTIN_FILAMENT_ORGANIZATION	0.78	0.02	0	0	0	not significant
6901	YAGI_AML_WITH_INV_16_TRANSLOCATION	0.78	0.02	0	0	0	not significant
6902	GO_4_HYDROXYPROLINE_METABOLIC_PROCESS	0.79	0.15	0	0	0	not significant
6903	GO_ALPHA_BETA_T_CELL_RECEPTOR_COMPLEX	0.79	0.15	0	0	0	not significant
6904	GO_EPHRIN_RECEPTOR_ACTIVITY	0.79	0.15	0	0	0	not significant
6905	GO_NEGATIVE_REGULATION_OF_PROTEIN_KINASE_ACTIVITY_BY_REGULATION_OF	0.79	0.15	0	0	0	not significant
6906	GO_PEPTIDYL_PROLINE_HYDROXYLATION_TO_4_HYDROXY_L_PROLINE	0.79	0.15	0	0	0	not significant
6907	ISHIKAWA_STING_SIGNALING	0.79	0.15	0	0	0	not significant
6908	BIOCARTA_MTA3_PATHWAY	0.79	0.14	0	0	0	not significant
6909	FRIDMAN_SENESCENCE_DN	0.79	0.14	0	0	0	not significant
6910	GO_AORTA_MORPHOGENESIS	0.79	0.14	0	0	0	not significant
6911	GO_G1_TO_G0_TRANSITION	0.79	0.14	0	0	0	not significant
6912	GO_METANEPHRIC_COLLECTING_DUCT_DEVELOPMENT	0.79	0.14	0	0	0	not significant
6913	GO_MOTOR_BEHAVIOR	0.79	0.14	0	0	0	not significant
6914	GO_MUTLALPHA_COMPLEX	0.79	0.14	0	0	0	not significant
6915	GO_NEGATIVE_REGULATION_OF_PROTEIN_HOMOOLIGOMERIZATION	0.79	0.14	0	0	0	not significant
6916	GO_PHOSPHOLIPASE_BINDING	0.79	0.14	0	0	0	not significant
6917	GO_REGULATION_OF_CYTOLYSIS	0.79	0.14	0	0	0	not significant
6918	GO_REGULATION_OF_OVARIAN_FOLLICLE_DEVELOPMENT	0.79	0.14	0	0	0	not significant
6919	REACTOME NEGATIVE REGULATION OF NMDA RECEPTOR MEDIATED NEURONA	0.79	0.14	0	0	0	not significant
6920	RICKMAN_HEAD_AND_NECK_CANCER_C	0.79	0.14	0	0	0	not significant
6921	RUNNE_GENDER_EFFECT_UP	0.79	0.14	0	0	0	not significant
6922	XU_AKT1_TARGETS_48HR	0.79	0.14	0	0	0	not significant
6923	BIOCARTA_RANBP2_PATHWAY	0.79	0.13	0	0	0	not significant
6924	CHEN_LVAD_SUPPORT_OF_FAILING_HEART_DN	0.79	0.13	0	0	0	not significant
6925	CHR8P12	0.79	0.13	0	0	0	not significant
6926	GHO_ATF5_TARGETS_UP	0.79	0.13	0	0	0	not significant
6927	GO_C3HC4_TYPE_RING_FINGER_DOMAIN_BINDING	0.79	0.13	0	0	0	not significant
6928	GO_DIACYLGLYCEROL_BINDING	0.79	0.13	0	0	0	not significant
6929	GO_GOLGI_RIBBON_FORMATION	0.79	0.13	0	0	0	not significant
6930	GO_INTEGRIN_ACTIVATION	0.79	0.13	0	0	0	not significant
6931	GO_NEUROPEPTIDE_RECEPTOR_BINDING	0.79	0.13	0	0	0	not significant
6932	GO_PODOSOME_ASSEMBLY	0.79	0.13	0	0	0	not significant
6933	GO_SEX_CHROMATIN	0.79	0.13	0	0	0	not significant
6934	GO_U6_SNRNP	0.79	0.13	0	0	0	not significant
6935	GREENBAUM_E2A_TARGETS_DN	0.79	0.13	0	0	0	not significant
6936	KEGG_PRION_DISEASES	0.79	0.13	0	0	0	not significant
6937	LI_CISPLATIN_RESISTANCE_DN	0.79	0.13	0	0	0	not significant
6938	MANTOVANI_NFKB_TARGETS_UP	0.79	0.13	0	0	0	not significant
6939	MARKS_HDAC_TARGETS_DN	0.79	0.13	0	0	0	not significant
6940	REACTOME_NICOTINAMIDE_SALVAGING	0.79	0.13	0	0	0	not significant
6941	VALK_AML_CLUSTER_15	0.79	0.13	0	0	0	not significant
6942	WANG_NEOPLASTIC_TRANSFORMATION_BY_CCND1_MYC	0.79	0.13	0	0	0	not significant
6943	WELCH_GATA1_TARGETS	0.79	0.13	0	0	0	not significant
6944	BANDRES_RESPONSE_TO_CARMUSTIN_WITHOUT_MGMT_24HR_UP	0.79	0.12	0	0	0	not significant
6945	BIOCARTA_ERK5_PATHWAY	0.79	0.12	0	0	0	not significant
6946	BYSTRYKH_HEMATOPOIESIS_STEM_CELL_SCP2_QTL_TRANS	0.79	0.12	0	0	0	not significant
6947	GO_L_ASCORBIC_ACID_BINDING	0.79	0.12	0	0	0	not significant
6948	GO_NEGATIVE_REGULATION_OF_PROTEIN_LOCALIZATION_TO_NUCLEUS	0.79	0.12	0	0	0	not significant
6949	GO_POLYAMINE_METABOLIC_PROCESS	0.79	0.12	0	0	0	not significant
6950	GO_REGULATION_OF_CHROMATIN_SILENCING	0.79	0.12	0	0	0	not significant
6951	GO_REGULATION_OF_NON_CANONICAL_WNT_SIGNALING_PATHWAY	0.79	0.12	0	0	0	not significant
6952	ICHIBA_GRAFT_VERSUS_HOST_DISEASE_35D_DN	0.79	0.12	0	0	0	not significant
6953	KORKOLA_CORRELATED_WITH_POU5F1	0.79	0.12	0	0	0	not significant
6954	LIU_LIVER_CANCER	0.79	0.12	0	0	0	not significant
6955	REACTOME TP53 REGULATES TRANSCRIPTION OF GENES INVOLVED IN CYTOC	0.79	0.12	0	0	0	not significant
6956	GO_FATTY_ACID_TRANSMEMBRANE_TRANSPORT	0.79	0.11	0	0	0	not significant
6957	GO_POSITIVE_REGULATION_OF_ERYTHROCYTE_DIFFERENTIATION	0.79	0.11	0	0	0	not significant
6958	GO REGULATION OF MITOCHONDRIAL OUTER MEMBRANE PERMEABILIZATION IN	0.79	0.11	0	0	0	not significant
6959	KAAB_FAILED_HEART_ATRIUM_UP	0.79	0.11	0	0	0	not significant
6960	KYNG_RESPONSE_TO_H2O2_VIA_ERCC6_DN	0.79	0.11	0	0	0	not significant
6961	GO_CYTOPLASMIC_MICROTUBULE_ORGANIZATION	0.79	0.1	0	0	0	not significant
6962	GO_MEMBRANE_DEPOLARIZATION	0.79	0.1	0	0	0	not significant
6963	GO_MULTI_ORGANISM_CELLULAR_PROCESS	0.79	0.1	0	0	0	not significant
6964	GO_POLY_PYRIMIDINE_TRACT_BINDING	0.79	0.1	0	0	0	not significant

6965	XU_HGF_TARGETS_REPRESSED_BY_AKT1_DN	0.79	0.1	0	0	0	not significant
6966	GUO_HEX_TARGETS_UP	0.79	0.09	0	0	0	not significant
6967	GO_DETECTION_OF_ABIOTIC_STIMULUS	0.79	0.08	0	0	0	not significant
6968	GO_DNA_MODIFICATION	0.79	0.08	0	0	0	not significant
6969	ROESSLER_LIVER_CANCER_METASTASIS_UP	0.79	0.08	0	0	0	not significant
6970	REACTOME_GLOBAL_GENOME_NUCLEOTIDE_EXCISION_REPAIR_GG_NER	0.79	0.07	0	0	0	not significant
6971	IVANOVA_HEMATOPOIESIS_STEM_CELL_LONG_TERM	0.79	0.06	0	0	0	not significant
6972	GO_REGULATION_OF_PROTEIN_LOCALIZATION_TO_MEMBRANE	0.79	0.05	0	0	0	not significant
6973	GO_EPITHELIAL_TUBE_MORPHOGENESIS	0.79	0.04	0	0	0	not significant
6974	GO_REGULATION_OF_TRANS_SYNAPTIC_SIGNALING	0.79	0.04	0	0	0	not significant
6975	GO_MUSCLE_SYSTEM_PROCESS	0.79	0.03	0	0	0	not significant
6976	GO_PROTEIN_ACYLATION	0.79	0.03	0	0	0	not significant
6977	GO_EMBRYONIC_MORPHOGENESIS	0.79	0.02	0	0	0	not significant
6978	GO_VESICLE_ORGANIZATION	0.79	0.02	0	0	0	not significant
6979	GO_PROTEIN_LOCALIZATION_TO_MEMBRANE	0.79	0.01	0	0	0	not significant
6980	CHR2Q34	0.80	0.16	0	0	0	not significant
6981	GO_COPPER_ION_TRANSMEMBRANE_TRANSPORT	0.80	0.16	0	0	0	not significant
6982	GO_LIPOXYGENASE_PATHWAY	0.80	0.16	0	0	0	not significant
6983	REACTOME_SYNTHESIS_OF_DOLICHYL_PHOSPHATE	0.80	0.16	0	0	0	not significant
6984	CHR7P11	0.80	0.15	0	0	0	not significant
6985	GHANDHI_BYSTANDER_IRRADIATION_DN	0.80	0.15	0	0	0	not significant
6986	GO_POSITIVE_REGULATION_OF_INTRACELLULAR_STEROID_HORMONE_RECEPTOF	0.80	0.15	0	0	0	not significant
6987	GO_POSITIVE_REGULATION_OF_PROTEIN_POLYUBIQUITINATION	0.80	0.15	0	0	0	not significant
6988	GO_PROTEIN_PHOSPHATASE_TYPE_1_COMPLEX	0.80	0.15	0	0	0	not significant
6989	GO_REGULATION_OF_NUCLEAR_CELL_CYCLE_DNA_REPLICATION	0.80	0.15	0	0	0	not significant
6990	GO_SORTING_ENDOSOME	0.80	0.15	0	0	0	not significant
6991	GO_TRIGLYCERIDE_CATABOLIC_PROCESS	0.80	0.15	0	0	0	not significant
6992	GO_VERY_LONG_CHAIN_FATTY_ACID_COA_LIGASE_ACTIVITY	0.80	0.15	0	0	0	not significant
6993	HOLLEMAN_DAUNORUBICIN_ALL_UP	0.80	0.15	0	0	0	not significant
6994	LE_SKI_TARGETS_UP	0.80	0.15	0	0	0	not significant
6995	NIKOLSKY_BREAST_CANCER_6P24_P22_AMPLICON	0.80	0.15	0	0	0	not significant
6996	REACTOME_HEME_BIOSYNTHESIS	0.80	0.15	0	0	0	not significant
6997	YANG_MUC2_TARGETS_DUODENUM_3MO_DN	0.80	0.15	0	0	0	not significant
6998	GO_CHONDROCYTE_DEVELOPMENT_INVOLVED_IN_ENDOCHONDRAL_BONE_MORP	0.80	0.14	0	0	0	not significant
6999	GO_DIAPEDESIS	0.80	0.14	0	0	0	not significant
7000	GO_EQUATORIAL_MICROTUBULE_ORGANIZING_CENTER	0.80	0.14	0	0	0	not significant
7001	GO_NEGATIVE_REGULATION_OF_REACTIVE_OXYGEN_SPECIES_BIOSYNTHETIC_PR	0.80	0.14	0	0	0	not significant
7002	GO_PALMITOYLTRANSFERASE_COMPLEX	0.80	0.14	0	0	0	not significant
7003	GO_REGULATION_OF_MAST_CELL_ACTIVATION_INVOLVED_IN_IMMUNE_RESPONSE	0.80	0.14	0	0	0	not significant
7004	GO_RETINAL_ROD_CELL_DEVELOPMENT	0.80	0.14	0	0	0	not significant
7005	GO_T_CELL_RECEPTOR_COMPLEX	0.80	0.14	0	0	0	not significant
7006	REACTOME_POST_TRANSCRIPTIONAL_SILENCING_BY_SMALL_RNAS	0.80	0.14	0	0	0	not significant
7007	REACTOME_SIGNALING_BY_BMP	0.80	0.14	0	0	0	not significant
7008	WORSCHECH_TUMOR_REJECTION_DN	0.80	0.14	0	0	0	not significant
7009	ZHENG_FOXP3_TARGETS_DN	0.80	0.14	0	0	0	not significant
7010	BREDEMEYER_RAG_SIGNALING_VIA_ATM_NOT_VIA_NFKB_DN	0.80	0.13	0	0	0	not significant
7011	GO_FUCOSE_METABOLIC_PROCESS	0.80	0.13	0	0	0	not significant
7012	GO_INNER_EAR_RECEPTOR_CELL_STEREOCILIUM_ORGANIZATION	0.80	0.13	0	0	0	not significant
7013	GO_INTERMEMBRANE_LIPID_TRANSFER	0.80	0.13	0	0	0	not significant
7014	GO_LONG_TERM_SYNAPTIC_DEPRESSION	0.80	0.13	0	0	0	not significant
7015	GO_MISMATCH_REPAIR_COMPLEX	0.80	0.13	0	0	0	not significant
7016	GO_NEGATIVE_REGULATION_OF_STEM_CELL_DIFFERENTIATION	0.80	0.13	0	0	0	not significant
7017	GO_OXIDOREDUCTASE_ACTIVITY_ACTING_ON_THE_ALDEHYDE_OR_OXO_GROUP_	0.80	0.13	0	0	0	not significant
7018	GO_PHOSPHATIDYLINOSITOL_3_PHOSPHATE_BIOSYNTHETIC_PROCESS	0.80	0.13	0	0	0	not significant
7019	GO_REGULATION_OF_INTRACELLULAR_ESTROGEN_RECEPTOR_SIGNALING_PATHV	0.80	0.13	0	0	0	not significant
7020	GO_REGULATION_OF_T_CELL_TOLERANCE_INDUCTION	0.80	0.13	0	0	0	not significant
7021	GO_U1_SNRNP	0.80	0.13	0	0	0	not significant
7022	GO_ZYMOGEN_ACTIVATION	0.80	0.13	0	0	0	not significant
7023	PID_A6B1_A6B4_INTEGRIN_PATHWAY	0.80	0.13	0	0	0	not significant
7024	REACTOME_RUNX2_REGULATES_BONE_DEVELOPMENT	0.80	0.13	0	0	0	not significant
7025	REACTOME_SPHINGOLIPID_DE_NOVO_BIOSYNTHESIS	0.80	0.13	0	0	0	not significant
7026	REACTOME_TRANSFERRIN_ENDOCYTOSIS_AND_RECYCLING	0.80	0.13	0	0	0	not significant
7027	ZWANG_CLASS_2_TRANSIENTLY_INDUCED_BY_EGF	0.80	0.13	0	0	0	not significant
7028	BECKER_TAMOXIFEN_RESISTANCE_DN	0.80	0.12	0	0	0	not significant
7029	CHEN_HOXA5_TARGETS_9HR_DN	0.80	0.12	0	0	0	not significant
7030	GO_MITOCHONDRIAL_GENOME_MAINTENANCE	0.80	0.12	0	0	0	not significant
7031	HU_GENOTOXIC_DAMAGE_24HR	0.80	0.12	0	0	0	not significant
7032	NAKAYAMA_SOFT_TISSUE_TUMORS_PCA1_DN	0.80	0.12	0	0	0	not significant
7033	SU_PANCREAS	0.80	0.12	0	0	0	not significant
7034	YAMASHITA_LIVER_CANCER_STEM_CELL_DN	0.80	0.12	0	0	0	not significant
7035	BIOCARTA_ALK_PATHWAY	0.80	0.11	0	0	0	not significant
7036	GO_FILOPODIUM_ASSEMBLY	0.80	0.11	0	0	0	not significant
7037	REACTOME_G_ALPHA_S_SIGNALLING_EVENTS	0.80	0.11	0	0	0	not significant
7038	REACTOME_PRC2_METHYLATES_HISTONES_AND_DNA	0.80	0.11	0	0	0	not significant
7039	REACTOME_TRANSPORT_OF_BILE_SALTS_AND_ORGANIC_ACIDS_METAL_IONS_AN	0.80	0.11	0	0	0	not significant
7040	GO_NEGATIVE_REGULATION_OF_ION_TRANSPORT	0.80	0.1	0	0	0	not significant
7041	JAZAG_TGFB1_SIGNALING_UP	0.80	0.1	0	0	0	not significant
7042	LEE_LIVER_CANCER_MYC_TGFA_UP	0.80	0.1	0	0	0	not significant
7043	REACTOME_DISEASES_OF_METABOLISM	0.80	0.09	0	0	0	not significant
7044	GO_NEGATIVE_REGULATION_OF_NEURON_PROJECTION_DEVELOPMENT	0.80	0.08	0	0	0	not significant
7045	PKCA_DN.V1_DN	0.80	0.08	0	0	0	not significant
7046	PLASARI_TGFB1_SIGNALING_VIA_NFIC_1HR_DN	0.80	0.08	0	0	0	not significant
7047	REACTOME_CHROMOSOME_MAINTENANCE	0.80	0.08	0	0	0	not significant
7048	SMID_BREAST_CANCER_LUMINAL_B_UP	0.80	0.08	0	0	0	not significant
7049	TIEN_INTESTINE_PROBIOTICS_2HR_DN	0.80	0.08	0	0	0	not significant
7050	MULLIGHAN_NPM1_MUTATED_SIGNATURE_1_DN	0.80	0.07	0	0	0	not significant
7051	MULLIGHAN_NPM1_SIGNATURE_3_DN	0.80	0.07	0	0	0	not significant
7052	GAUSSMANN_MLL_AF4_FUSION_TARGETS_A_UP	0.80	0.06	0	0	0	not significant
7053	GO_APPENDAGE_DEVELOPMENT	0.80	0.06	0	0	0	not significant
7054	GO_REGULATION_OF_EXOCYTOSIS	0.80	0.06	0	0	0	not significant
7055	GO_RHO_GTPASE_BINDING	0.80	0.06	0	0	0	not significant
7056	NOUZOVA_TRETINOIN_AND_H4_ACETYLATION	0.80	0.06	0	0	0	not significant
7057	GO_REGULATION_OF_MACROAUTOPHAGY	0.80	0.05	0	0	0	not significant
7058	GO_SYNAPSE	0.80	0.01	0	0	0	not significant
7059	GO_POSITIVE_REGULATION_OF_VESICLE_FUSION	0.81	0.17	0	0	0	not significant
7060	FRASOR_TAMOXIFEN_RESPONSE_DN	0.81	0.16	0	0	0	not significant
7061	GO_AXIS_ELONGATION	0.81	0.16	0	0	0	not significant
7062	GO_LYSOSOMAL_PROTEIN_CATABOLIC_PROCESS	0.81	0.16	0	0	0	not significant
7063	GO_MAINTENANCE_OF_DNA_METHYLATION	0.81	0.16	0	0	0	not significant
7064	GO_POSITIVE_REGULATION_OF_TUMOR_NECROSIS_FACTOR_MEDIATED_SIGNALIN	0.81	0.16	0	0	0	not significant
7065	GO_REGULATION_OF_EXTRACELLULAR_MATRIX_DISASSEMBLY	0.81	0.16	0	0	0	not significant
7066	MAGRANGEAS_MULTIPLE_MYELOMA_IGG_VS_IGA_UP	0.81	0.16	0	0	0	not significant
7067	SHIRAISHI_PLZF_TARGETS_UP	0.81	0.16	0	0	0	not significant
7068	TSUDA_ALVEOLAR_SOFT_PART_SARCOMA	0.81	0.16	0	0	0	not significant
7069	GO_AMYLOID_BETA_CLEARANCE_BY_TRANSCYTOSIS	0.81	0.15	0	0	0	not significant
7070	GO_CENTRIOLE_ELONGATION	0.81	0.15	0	0	0	not significant
7071	GO_CROSSOVER_JUNCTION_ENDODEOXYRIBONUCLEASE_ACTIVITY	0.81	0.15	0	0	0	not significant
7072	GO_DOLICHYL_DIPHOSPHATE_BIOSYNTHETIC_PROCESS	0.81	0.15	0	0	0	not significant
7073	GO_FRUCTOSE_6_PHOSPHATE_METABOLIC_PROCESS	0.81	0.15	0	0	0	not significant

7074	GO_GLUTAMATE_SECRETION	0.81	0.15	0	0	0	not significant
7075	GO_HINDBRAIN_RADIAL_GLIA_GUIDED_CELL_MIGRATION	0.81	0.15	0	0	0	not significant
7076	GO_JUN_KINASE_BINDING	0.81	0.15	0	0	0	not significant
7077	GO_MICROTUBULE_ANCHORING_AT_CENTROSOME	0.81	0.15	0	0	0	not significant
7078	GO_NUCLEOTIDASE_ACTIVITY	0.81	0.15	0	0	0	not significant
7079	REACTOME_SOS_MEDIATED_SIGNALLING	0.81	0.15	0	0	0	not significant
7080	REACTOME_SUMO_IS_TRANSFERRED_FROM_E1_TO_E2_UBE2I_UBC9	0.81	0.15	0	0	0	not significant
7081	REACTOME_SYNTHESIS_OF_5_EICOSATETRAENOIC_ACIDS	0.81	0.15	0	0	0	not significant
7082	SESTO_RESPONSE_TO_UV_C4	0.81	0.15	0	0	0	not significant
7083	TCGA_GLIOBLASTOMA_MUTATED	0.81	0.15	0	0	0	not significant
7084	CHR2P16	0.81	0.14	0	0	0	not significant
7085	GO_EYE_PHOTORECEPTOR_CELL_DEVELOPMENT	0.81	0.14	0	0	0	not significant
7086	GO_MISMATCHED_DNA_BINDING	0.81	0.14	0	0	0	not significant
7087	GO_NEGATIVE_REGULATION_OF_MYOSIN_LIGHT_CHAIN_PHOSPHATASE_ACTIVITY	0.81	0.14	0	0	0	not significant
7088	GO_POSITIVE_REGULATION_OF_FILOPODIUM_ASSEMBLY	0.81	0.14	0	0	0	not significant
7089	GO_POSITIVE_REGULATION_OF_INSULIN_SECRETION_INVOLVED_IN_CELLULAR_RE	0.81	0.14	0	0	0	not significant
7090	GO_PROTON_TRANSPORTING_TWO_SECTOR_ATPASE_COMPLEX	0.81	0.14	0	0	0	not significant
7091	GO_REGULATION_OF_MAST_CELL_ACTIVATION	0.81	0.14	0	0	0	not significant
7092	GOERING_BLOOD_HDL_CHOLESTEROL_QTL_CIS	0.81	0.14	0	0	0	not significant
7093	HALLMARK_ANGIOGENESIS	0.81	0.14	0	0	0	not significant
7094	HENDRICKS_SMARCA4_TARGETS_DN	0.81	0.14	0	0	0	not significant
7095	MIZUSHIMA_AUTOPHAGOSOME_FORMATION	0.81	0.14	0	0	0	not significant
7096	ONGUSAHA_BRCA1_TARGETS_UP	0.81	0.14	0	0	0	not significant
7097	REACTOME_GDP_FUCOSE_BIOSYNTHESIS	0.81	0.14	0	0	0	not significant
7098	YAO_TEMPORAL_RESPONSE_TO_PROGESTERONE_CLUSTER_3	0.81	0.14	0	0	0	not significant
7099	BOYLAN_MULTIPLE_MYELOMA_D_CLUSTER_UP	0.81	0.13	0	0	0	not significant
7100	CHR1Q24	0.81	0.13	0	0	0	not significant
7101	GO_CELL_MIGRATION_IN_HINDBRAIN	0.81	0.13	0	0	0	not significant
7102	GO_ENDOCRINE_HORMONE_SECRETION	0.81	0.13	0	0	0	not significant
7103	GO_O_GLYCAN_PROCESSING	0.81	0.13	0	0	0	not significant
7104	GO_SRP_DEPENDENT_COTRANSLATIONAL_PROTEIN_TARGETING_TO_MEMBRANE	0.81	0.13	0	0	0	not significant
7105	STEARMAN_LUNG_CANCER_EARLY_VS_LATE_DN	0.81	0.13	0	0	0	not significant
7106	GO_MULTIVESICULAR_BODY_SORTING_PATHWAY	0.81	0.12	0	0	0	not significant
7107	GO_NEGATIVE_REGULATION_OF_HISTONE_MODIFICATION	0.81	0.12	0	0	0	not significant
7108	GO_SH3_SH2_ADAPTOR_ACTIVITY	0.81	0.12	0	0	0	not significant
7109	KENNY_CTNNB1_TARGETS_DN	0.81	0.12	0	0	0	not significant
7110	REACTOME_LYSOSOME_VESICLE_BIOGENESIS	0.81	0.12	0	0	0	not significant
7111	SHETH_LIVER_CANCER_VS_TXNIP_LOSS_PAM5	0.81	0.12	0	0	0	not significant
7112	WOO_LIVER_CANCER_RECURRENCE_DN	0.81	0.12	0	0	0	not significant
7113	GO_IRON_ION_TRANSPORT	0.81	0.11	0	0	0	not significant
7114	GO_RECEPTOR_TYROSINE_KINASE_BINDING	0.81	0.11	0	0	0	not significant
7115	GO_REGULATION_OF_MITOCHONDRIAL_MEMBRANE_POTENTIAL	0.81	0.11	0	0	0	not significant
7116	GO_SYNTAXIN_BINDING	0.81	0.11	0	0	0	not significant
7117	HEDENFALK_BREAST_CANCER_HEREDITARY_VS_SPORADIC	0.81	0.11	0	0	0	not significant
7118	KEGG_CARDIAC_MUSCLE_CONTRACTION	0.81	0.11	0	0	0	not significant
7119	KEGG_PANCREATIC_CANCER	0.81	0.11	0	0	0	not significant
7120	CHR10Q26	0.81	0.1	0	0	0	not significant
7121	GO_EXONUCLEASE_ACTIVITY	0.81	0.1	0	0	0	not significant
7122	KAYO_CALORIE_RESTRICTION_MUSCLE_DN	0.81	0.1	0	0	0	not significant
7123	KEGG_PEROXISOME	0.81	0.1	0	0	0	not significant
7124	GO_CILIARY_BASAL_BODY	0.81	0.09	0	0	0	not significant
7125	GO_NEGATIVE_REGULATION_OF_PROTEIN_CATABOLIC_PROCESS	0.81	0.09	0	0	0	not significant
7126	GO_POSITIVE_REGULATION_OF_CYSTEINE_TYPE_ENDOPEPTIDASE_ACTIVITY	0.81	0.09	0	0	0	not significant
7127	MORI_SMALL_PRE_BII_LYMPHOCYTE_DN	0.81	0.09	0	0	0	not significant
7128	GO_PIGMENT_GRANULE	0.81	0.08	0	0	0	not significant
7129	GO_ACTOMYOSIN_STRUCTURE_ORGANIZATION	0.81	0.07	0	0	0	not significant
7130	WAMUNYOKOLI_OVARIAN_CANCER_GRADES_1_2_UP	0.81	0.07	0	0	0	not significant
7131	BONOME_OVARIAN_CANCER_SURVIVAL_OPTIMAL_DEBULKING	0.81	0.06	0	0	0	not significant
7132	RIGGINS_TAMOXIFEN_RESISTANCE_DN	0.81	0.06	0	0	0	not significant
7133	GO_SKELETAL_SYSTEM_DEVELOPMENT	0.81	0.05	0	0	0	not significant
7134	GO_POSITIVE_REGULATION_OF_CATABOLIC_PROCESS	0.81	0.03	0	0	0	not significant
7135	GO_POSTSYNAPSE	0.81	0.03	0	0	0	not significant
7136	GO_SENSORY_PERCEPTION_OF_SMELL	0.82	0.18	0	0	0	not significant
7137	CUI_TCF21_TARGETS_UP	0.82	0.17	0	0	0	not significant
7138	GO_CELLULAR_POTASSIUM_ION_HOMEOSTASIS	0.82	0.17	0	0	0	not significant
7139	GO_REGULATION_OF_DENDRITE_EXTENSION	0.82	0.17	0	0	0	not significant
7140	GO_SEMAPHORIN_RECEPTOR_ACTIVITY	0.82	0.17	0	0	0	not significant
7141	GO_UDP_N_ACETYLGLUCOSAMINE_BIOSYNTHETIC_PROCESS	0.82	0.17	0	0	0	not significant
7142	GO_CHEMOATTRACTANT_ACTIVITY	0.82	0.16	0	0	0	not significant
7143	GO_DOPAMINE_SECRETION	0.82	0.16	0	0	0	not significant
7144	GO_EXTRACELLULAR_MATRIX_ASSEMBLY	0.82	0.16	0	0	0	not significant
7145	GO_FAT_SOLUBLE_VITAMIN_BIOSYNTHETIC_PROCESS	0.82	0.16	0	0	0	not significant
7146	GO_INTERMEMBRANE_LIPID_TRANSFER_ACTIVITY	0.82	0.16	0	0	0	not significant
7147	GO_KERATAN_SULFATE_BIOSYNTHETIC_PROCESS	0.82	0.16	0	0	0	not significant
7148	GO_POSITIVE_REGULATION_OF_T_CELL_RECEPTOR_SIGNALING_PATHWAY	0.82	0.16	0	0	0	not significant
7149	GO_SEMAPHORIN_PLEXIN_SIGNALING_PATHWAY_INVOLVED_IN_NEURON_PROJECT	0.82	0.16	0	0	0	not significant
7150	GO_SWI_SNF_COMPLEX	0.82	0.16	0	0	0	not significant
7151	RICKMAN_HEAD_AND_NECK_CANCER_E	0.82	0.16	0	0	0	not significant
7152	YANG_MUC2_TARGETS_DUODENUM_6MO_DN	0.82	0.16	0	0	0	not significant
7153	BIOCARTA_BIOPEPTIDES_PATHWAY	0.82	0.15	0	0	0	not significant
7154	BIOCARTA_DICER_PATHWAY	0.82	0.15	0	0	0	not significant
7155	CHR18Q11	0.82	0.15	0	0	0	not significant
7156	CHR5Q21	0.82	0.15	0	0	0	not significant
7157	CROMER_TUMORIGENESIS_UP	0.82	0.15	0	0	0	not significant
7158	GAUSSMANN_MLL_AF4_FUSION_TARGETS_F_DN	0.82	0.15	0	0	0	not significant
7159	GO_HISTONE_H2A_MONOUBIQUITINATION	0.82	0.15	0	0	0	not significant
7160	GO_MIDBODY_ABSCISSION	0.82	0.15	0	0	0	not significant
7161	GO_RIG_I_SIGNALING_PATHWAY	0.82	0.15	0	0	0	not significant
7162	GO_WNT_SIGNALING_PATHWAY_CALCIUM_MODULATING_PATHWAY	0.82	0.15	0	0	0	not significant
7163	MEISSNER_ES_ICP_WITH_H3K4ME3	0.82	0.15	0	0	0	not significant
7164	OXFORD_RALA_OR_RALB_TARGETS_DN	0.82	0.15	0	0	0	not significant
7165	PARK_TRETINOIN_RESPONSE_AND_PML_RARA_FUSION	0.82	0.15	0	0	0	not significant
7166	REACTOME_P75NTR_REGULATES_AXONOGENESIS	0.82	0.15	0	0	0	not significant
7167	REACTOME_SYNTHESIS_OF_UDP_N_ACETYL_GLUCOSAMINE	0.82	0.15	0	0	0	not significant
7168	REACTOME_TRAF6_MEDIATED_NF_KB_ACTIVATION	0.82	0.15	0	0	0	not significant
7169	BIOCARTA_PPARA_PATHWAY	0.82	0.14	0	0	0	not significant
7170	BOHN_PRIMARY_IMMUNODEFICIENCY_SYNDROM_DN	0.82	0.14	0	0	0	not significant
7171	GO_HSP70_PROTEIN_BINDING	0.82	0.14	0	0	0	not significant
7172	GO_INTERLEUKIN_6_MEDIATED_SIGNALING_PATHWAY	0.82	0.14	0	0	0	not significant
7173	GO_MIRNA_MEDIATED_INHIBITION_OF_TRANSLATION	0.82	0.14	0	0	0	not significant
7174	GO_NUCLEOSIDE_METABOLIC_PROCESS	0.82	0.14	0	0	0	not significant
7175	GO_PROTON_EXPORTING_ATPASE_ACTIVITY	0.82	0.14	0	0	0	not significant
7176	GO_REGULATION_OF_ENDOTHELIAL_CELL_DIFFERENTIATION	0.82	0.14	0	0	0	not significant
7177	KEGG_INOSITOL_PHOSPHATE_METABOLISM	0.82	0.14	0	0	0	not significant
7178	NIKOLSKY_BREAST_CANCER_20Q11_AMPLICON	0.82	0.14	0	0	0	not significant
7179	REACTOME_POTASSIUM_CHANNELS	0.82	0.14	0	0	0	not significant
7180	ENGELMANN_CANCER_PROGENITORS_DN	0.82	0.13	0	0	0	not significant
7181	GO_HISTONE_H3_ACETYLATION	0.82	0.13	0	0	0	not significant
7182	GO_NUCLEOTIDE_EXCISION_REPAIR_DNA_INCISION	0.82	0.13	0	0	0	not significant

7183	GO_PRESYNAPTIC_ENDOCYTOSIS	0.82	0.13	0	0	0	not significant
7184	GO_TRANSCRIPTION_COFACTOR_BINDING	0.82	0.13	0	0	0	not significant
7185	SCIAN_INVERSED_TARGETS_OF_TP53_AND_TP73_DN	0.82	0.13	0	0	0	not significant
7186	BCAT_BILD_ET_AL_DN	0.82	0.12	0	0	0	not significant
7187	FONTAINE_PAPILLARY_THYROID_CARCINOMA_UP	0.82	0.12	0	0	0	not significant
7188	GOLUB_ALL_VS_AML_UP	0.82	0.12	0	0	0	not significant
7189	HOLLERN_EMT_BREAST_TUMOR_DN	0.82	0.12	0	0	0	not significant
7190	GO_CELL_DEATH_IN_RESPONSE_TO_OXIDATIVE_STRESS	0.82	0.11	0	0	0	not significant
7191	GO_ENDOCRINE_SYSTEM_DEVELOPMENT	0.82	0.11	0	0	0	not significant
7192	GO_EXECUTION_PHASE_OF_APOPTOSIS	0.82	0.11	0	0	0	not significant
7193	GO_NEGATIVE_REGULATION_OF_VIRAL_PROCESS	0.82	0.11	0	0	0	not significant
7194	KEGG_PHOSPHATIDYLINOSITOL_SIGNALING_SYSTEM	0.82	0.11	0	0	0	not significant
7195	RB_P130_DN.V1_DN	0.82	0.1	0	0	0	not significant
7196	ZHANG_BREAST_CANCER_PROGENITORS_DN	0.82	0.1	0	0	0	not significant
7197	GO_NEURON_PROJECTION_EXTENSION	0.82	0.09	0	0	0	not significant
7198	GO_POSITIVE_REGULATION_OF_ION_TRANSPORT	0.82	0.09	0	0	0	not significant
7199	GO_TOR_SIGNALING	0.82	0.09	0	0	0	not significant
7200	HALLMARK_DNA_REPAIR	0.82	0.09	0	0	0	not significant
7201	GO_REGULATION_OF_CARBOHYDRATE_METABOLIC_PROCESS	0.82	0.08	0	0	0	not significant
7202	PROVENZANI_METASTASIS_UP	0.82	0.08	0	0	0	not significant
7203	GO_POSITIVE_REGULATION_OF_CELL_PROJECTION_ORGANIZATION	0.82	0.05	0	0	0	not significant
7204	CONRAD_GERMLINE_STEM_CELL	0.83	0.18	0	0	0	not significant
7205	GO_REGULATION_OF_T_HELPER_17_TYPE_IMMUNE_RESPONSE	0.83	0.18	0	0	0	not significant
7206	REACTOME_GRB2_EVENTS_IN_ERBB2_SIGNALING	0.83	0.18	0	0	0	not significant
7207	GO_CAVEOLIN_MEDIATED_ENDOCYTOSIS	0.83	0.17	0	0	0	not significant
7208	GO_COLLAGEN_TRIMER	0.83	0.17	0	0	0	not significant
7209	GO_I_KAPPAB_PHOSPHORYLATION	0.83	0.17	0	0	0	not significant
7210	GO_LONG_CHAIN_FATTY_ACID_IMPORT_INTO_CELL	0.83	0.17	0	0	0	not significant
7211	GO_NEGATIVE_REGULATION_OF_HISTONE_ACETYLATION	0.83	0.17	0	0	0	not significant
7212	GO_PLATELET_DERIVED_GROWTH_FACTOR_RECEPTOR_BETA_SIGNALING_PATH\	0.83	0.17	0	0	0	not significant
7213	GO_PROTEIN_LIPID_COMPLEX_SUBUNIT_ORGANIZATION	0.83	0.17	0	0	0	not significant
7214	GO_PROTEIN_LOCALIZATION_TO_ENDOPLASMIC_RETICULUM_EXIT_SITE	0.83	0.17	0	0	0	not significant
7215	GO_REGULATION_OF_MACROPHAGE_CHEMOTAXIS	0.83	0.17	0	0	0	not significant
7216	GO_REGULATION_OF_SYNAPTIC_TRANSMISSION_DOPAMINERGIC	0.83	0.17	0	0	0	not significant
7217	PIONTEK_PKD1_TARGETS_UP	0.83	0.17	0	0	0	not significant
7218	REACTOME_FERTILIZATION	0.83	0.17	0	0	0	not significant
7219	REACTOME_KERATAN_SULFATE_BIOSYNTHESIS	0.83	0.17	0	0	0	not significant
7220	STEINER_ERYTHROCYTE_MEMBRANE_GENES	0.83	0.17	0	0	0	not significant
7221	BANDRES_RESPONSE_TO_CARMUSTIN_WITHOUT_MGMT_24HR_DN	0.83	0.16	0	0	0	not significant
7222	BIOCARTA_CARM_ER_PATHWAY	0.83	0.16	0	0	0	not significant
7223	BIOCARTA_MITOCHONDRIA_PATHWAY	0.83	0.16	0	0	0	not significant
7224	BIOCARTA_RB_PATHWAY	0.83	0.16	0	0	0	not significant
7225	GO_COHESIN_COMPLEX	0.83	0.16	0	0	0	not significant
7226	GO_FIBROBLAST_GROWTH_FACTOR_BINDING	0.83	0.16	0	0	0	not significant
7227	GO_HEME_BIOSYNTHETIC_PROCESS	0.83	0.16	0	0	0	not significant
7228	GO_LSM2_8_COMPLEX	0.83	0.16	0	0	0	not significant
7229	GO_LYS63_SPECIFIC_DEUBIQUITINASE_ACTIVITY	0.83	0.16	0	0	0	not significant
7230	GO_NEGATIVE_REGULATION_OF_RECEPTOR_MEDIATED_ENDOCYTOSIS	0.83	0.16	0	0	0	not significant
7231	GO_RECEPTOR_SERINE_THREONINE_KINASE_BINDING	0.83	0.16	0	0	0	not significant
7232	GO_REGULATION_OF_B_CELL_RECEPTOR_SIGNALING_PATHWAY	0.83	0.16	0	0	0	not significant
7233	GO_REGULATION_OF_GOLGI_ORGANIZATION	0.83	0.16	0	0	0	not significant
7234	GO_SEMAPHORIN_RECEPTOR_COMPLEX	0.83	0.16	0	0	0	not significant
7235	GO_SMC5_SMC6_COMPLEX	0.83	0.16	0	0	0	not significant
7236	GO_VENOUS_BLOOD_VESSEL_MORPHOGENESIS	0.83	0.16	0	0	0	not significant
7237	GOLUB_ALL_VS_AML_DN	0.83	0.16	0	0	0	not significant
7238	KANG_AR_TARGETS_UP	0.83	0.16	0	0	0	not significant
7239	PARK_APL_PATHOGENESIS_UP	0.83	0.16	0	0	0	not significant
7240	REACTOME_PURINE_CATABOLISM	0.83	0.16	0	0	0	not significant
7241	BIOCARTA_PAR1_PATHWAY	0.83	0.15	0	0	0	not significant
7242	GO_CELL_FATE_SPECIFICATION	0.83	0.15	0	0	0	not significant
7243	GO_DNA_CATABOLIC_PROCESS_ENDONUCLEOLYTIC	0.83	0.15	0	0	0	not significant
7244	GO_REGULATION_OF_EXTRACELLULAR_MATRIX_ORGANIZATION	0.83	0.15	0	0	0	not significant
7245	GO_RESPONSE_TO_INTERFERON_BETA	0.83	0.15	0	0	0	not significant
7246	GO_VOLTAGE_GATED_CALCIUM_CHANNEL_ACTIVITY	0.83	0.15	0	0	0	not significant
7247	KAAB_FAILED_HEART_VENTRICLE_DN	0.83	0.15	0	0	0	not significant
7248	KEGG_NOTCH_SIGNALING_PATHWAY	0.83	0.15	0	0	0	not significant
7249	REACTOME_SIGNALING_BY_HIPPO	0.83	0.15	0	0	0	not significant
7250	SHETH_LIVER_CANCER_VS_TXNIP_LOSS_PAM6	0.83	0.15	0	0	0	not significant
7251	XU_HGF_SIGNALING_NOT_VIA_AKT1_6HR	0.83	0.15	0	0	0	not significant
7252	ANDERSEN_LIVER_CANCER_KRT19_UP	0.83	0.14	0	0	0	not significant
7253	BREDEMEYER_RAG_SIGNALING_NOT_VIA_ATM_UP	0.83	0.14	0	0	0	not significant
7254	GO_CELL_CELL_ADHESION_MEDIATOR_ACTIVITY	0.83	0.14	0	0	0	not significant
7255	GO_NEGATIVE_REGULATION_OF_CHROMATIN_ORGANIZATION	0.83	0.14	0	0	0	not significant
7256	GO_NUCLEOBASE_CONTAINING_COMPOUND_KINASE_ACTIVITY	0.83	0.14	0	0	0	not significant
7257	GO_REGULATION_OF_SODIUM_ION_TRANSMEMBRANE_TRANSPORT	0.83	0.14	0	0	0	not significant
7258	GO_RESPONSE_TO_NICOTINE	0.83	0.14	0	0	0	not significant
7259	PID_CASPASE_PATHWAY	0.83	0.14	0	0	0	not significant
7260	REACTOME_G1_PHASE	0.83	0.14	0	0	0	not significant
7261	GO_ENERGY_RESERVE_METABOLIC_PROCESS	0.83	0.13	0	0	0	not significant
7262	GO_EXOCYTIC_PROCESS	0.83	0.13	0	0	0	not significant
7263	GO_GLYCOSYL_COMPOUND_METABOLIC_PROCESS	0.83	0.13	0	0	0	not significant
7264	GO_GRANULOCYTE_MIGRATION	0.83	0.13	0	0	0	not significant
7265	ZHONG_SECRETOME_OF_LUNG_CANCER_AND_MACROPHAGE	0.83	0.13	0	0	0	not significant
7266	GO_ACTIN_FILAMENT_BASED_MOVEMENT	0.83	0.12	0	0	0	not significant
7267	GO_MICROBODY_PART	0.83	0.12	0	0	0	not significant
7268	GO_NEGATIVE_REGULATION_OF_CYSTEINE_TYPE_ENDOPEPTIDASE_ACTIVITY	0.83	0.12	0	0	0	not significant
7269	PID_CMYB_PATHWAY	0.83	0.12	0	0	0	not significant
7270	CHR13Q14	0.83	0.11	0	0	0	not significant
7271	GO_CHROMATIN_ORGANIZATION_INVOLVED_IN_REGULATION_OF_TRANSCRIPTION	0.83	0.1	0	0	0	not significant
7272	KEGG_CALCIUM_SIGNALING_PATHWAY	0.83	0.1	0	0	0	not significant
7273	WATANABE_RECTAL_CANCER_RADIOTHERAPY_RESPONSIVE_UP	0.83	0.1	0	0	0	not significant
7274	ESC_V6.5_UP_EARLY.V1_UP	0.83	0.09	0	0	0	not significant
7275	GO_CELL_MATRIX_ADHESION	0.83	0.09	0	0	0	not significant
7276	GO_HISTONE_BINDING	0.83	0.08	0	0	0	not significant
7277	MULLIGHAN_NPM1_MUTATED_SIGNATURE_1_UP	0.83	0.08	0	0	0	not significant
7278	GO_POSITIVE_REGULATION_OF_NEURON_DIFFERENTIATION	0.83	0.06	0	0	0	not significant
7279	GO_IN_UTERO_EMBRYONIC_DEVELOPMENT	0.83	0.05	0	0	0	not significant
7280	GO_SYNAPTIC_SIGNALING	0.83	0.05	0	0	0	not significant
7281	GO_CELL_PROJECTION_ASSEMBLY	0.83	0.04	0	0	0	not significant
7282	BIOCARTA_PGC1A_PATHWAY	0.84	0.19	0	0	0	not significant
7283	FERRARI_RESPONSE_TO_FENRETINIDE_UP	0.84	0.18	0	0	0	not significant
7284	GO_NADPH_BINDING	0.84	0.18	0	0	0	not significant
7285	GO_NEGATIVE_REGULATION_OF_MONOOXYGENASE_ACTIVITY	0.84	0.18	0	0	0	not significant
7286	GO_PHOSPHATIDYLGLYCEROL_BINDING	0.84	0.18	0	0	0	not significant
7287	GO_POSITIVE_REGULATION_OF_STEM_CELL_PROLIFERATION	0.84	0.18	0	0	0	not significant
7288	GO_REGULATION_OF_CYTOPLASMIC_MRNA_PROCESSING_BODY_ASSEMBLY	0.84	0.18	0	0	0	not significant
7289	GO_REGULATION_OF_TUBULIN_DEACETYLATION	0.84	0.18	0	0	0	not significant
7290	KALMA_E2F1_TARGETS	0.84	0.18	0	0	0	not significant
7291	BERTUCCI_INVASIVE_CARCINOMA_DUCTAL_VS_LOBULAR_DN	0.84	0.17	0	0	0	not significant

7292	GO_DNA_REPLICATION_FACTOR_A_COMPLEX	0.84	0.17	0	0	0	not significant
7293	GO_KERATAN_SULFATE_CATABOLIC_PROCESS	0.84	0.17	0	0	0	not significant
7294	GO_MHC_CLASS_II_BIOSYNTHETIC_PROCESS	0.84	0.17	0	0	0	not significant
7295	GO_NEGATIVE_REGULATION_OF_BMP_SIGNALING_PATHWAY	0.84	0.17	0	0	0	not significant
7296	GO_NEGATIVE_REGULATION_OF_LONG_TERM_SYNAPTIC_POTENTIATION	0.84	0.17	0	0	0	not significant
7297	GO_OMEGA_PEPTIDASE_ACTIVITY	0.84	0.17	0	0	0	not significant
7298	GO_POSITIVE_REGULATION_OF_MEIOTIC_CELL_CYCLE	0.84	0.17	0	0	0	not significant
7299	GO_POSITIVE_REGULATION_OF_NAD_P_H_OXIDASE_ACTIVITY	0.84	0.17	0	0	0	not significant
7300	GO_PRESYNAPTIC_ACTIVE_ZONE_CYTOPLASMIC_COMPONENT	0.84	0.17	0	0	0	not significant
7301	GO_REGULATION_OF_AMYLOID_BETA_FORMATION	0.84	0.17	0	0	0	not significant
7302	GO_RESPONSE_TO_ANGIOTENSIN	0.84	0.17	0	0	0	not significant
7303	GO_WNT_ACTIVATED_RECEPTOR_ACTIVITY	0.84	0.17	0	0	0	not significant
7304	NELSON_RESPONSE_TO_ANDROGEN_DN	0.84	0.17	0	0	0	not significant
7305	REACTOME_ATF4_ACTIVATES_GENES_IN_RESPONSE_TO_ENDOPLASMIC_RETICUL	0.84	0.17	0	0	0	not significant
7306	REACTOME_IRAK1_RECRUITS_IKK_COMPLEX	0.84	0.17	0	0	0	not significant
7307	BALLIF_DEVELOPMENTAL_DISABILITY_P16_P12_DELETION	0.84	0.16	0	0	0	not significant
7308	BIOCARTA_RACC_PATHWAY	0.84	0.16	0	0	0	not significant
7309	CHR11Q21	0.84	0.16	0	0	0	not significant
7310	GO_CALCIUM_DEPENDENT_CYSTEINE_TYPE_ENDOPEPTIDASE_ACTIVITY	0.84	0.16	0	0	0	not significant
7311	GO_CEREBRAL_CORTEX_RADIALLY_ORIENTED_CELL_MIGRATION	0.84	0.16	0	0	0	not significant
7312	GO_COLLAGEN_BIOSYNTHETIC_PROCESS	0.84	0.16	0	0	0	not significant
7313	GO_GALACTOSYLTRANSFERASE_ACTIVITY	0.84	0.16	0	0	0	not significant
7314	GO_POSITIVE_REGULATION_OF_LIGASE_ACTIVITY	0.84	0.16	0	0	0	not significant
7315	GO_PROTEIN_LIPID_COMPLEX_ASSEMBLY	0.84	0.16	0	0	0	not significant
7316	GO_REGULATION_OF_ESTABLISHMENT_OR_MAINTENANCE_OF_CELL_POLARITY	0.84	0.16	0	0	0	not significant
7317	GO_REGULATION_OF_NAD_P_H_OXIDASE_ACTIVITY	0.84	0.16	0	0	0	not significant
7318	GO_T_CELL_TOLERANCE_INDUCTION	0.84	0.16	0	0	0	not significant
7319	HOWLIN_CITED1_TARGETS_1_DN	0.84	0.16	0	0	0	not significant
7320	LUI_TARGETS_OF_PAX8_PPARG_FUSION	0.84	0.16	0	0	0	not significant
7321	REACTOME_REGULATION_OF_PTEN_MRNA_TRANSLATION	0.84	0.16	0	0	0	not significant
7322	REACTOME_TLR3_MEDIATED_TICAM1_DEPENDENT_PROGRAMMED_CELL_DEATH	0.84	0.16	0	0	0	not significant
7323	REICHERT_G1S_REGULATORS_AS_PI3K_TARGETS	0.84	0.16	0	0	0	not significant
7324	STANELLE_E2F1_TARGETS	0.84	0.16	0	0	0	not significant
7325	WANG_METASTASIS_OF_BREAST_CANCER_ESR1_DN	0.84	0.16	0	0	0	not significant
7326	CASTELLANO_NRAS_TARGETS_UP	0.84	0.15	0	0	0	not significant
7327	CHR11P12	0.84	0.15	0	0	0	not significant
7328	CHR3Q26	0.84	0.15	0	0	0	not significant
7329	FIGUEROA_AML_METHYLATION_CLUSTER_3_DN	0.84	0.15	0	0	0	not significant
7330	GO_CUL3_RING_UBIQUITIN_LIGASE_COMPLEX	0.84	0.15	0	0	0	not significant
7331	GO_METALLOEXOPEPTIDASE_ACTIVITY	0.84	0.15	0	0	0	not significant
7332	GO_MYOSIN_BINDING	0.84	0.15	0	0	0	not significant
7333	GO_PURINE_NUCLEOSIDE_METABOLIC_PROCESS	0.84	0.15	0	0	0	not significant
7334	GO_REGULATION_OF_ANTIGEN_RECEPTOR_MEDIATED_SIGNALING_PATHWAY	0.84	0.15	0	0	0	not significant
7335	GO_REGULATION_OF_ASPARTIC_TYPE_PEPTIDASE_ACTIVITY	0.84	0.15	0	0	0	not significant
7336	GO_REGULATION_OF_VIRAL_INDUCED_CYTOPLASMIC_PATTERN_RECOGNITION_RI	0.84	0.15	0	0	0	not significant
7337	LEE_AGING_CEREBELLUM_DN	0.84	0.15	0	0	0	not significant
7338	RASHI_RESPONSE_TO_IONIZING_RADIATION_3	0.84	0.15	0	0	0	not significant
7339	RIZ_ERYTHROID_DIFFERENTIATION_CCNE1	0.84	0.15	0	0	0	not significant
7340	RUAN_RESPONSE_TO_TNF_TROGLITAZONE_DN	0.84	0.15	0	0	0	not significant
7341	BHAT_ESR1_TARGETS_NOT_VIA_AKT1_DN	0.84	0.14	0	0	0	not significant
7342	GO_MOLTING_CYCLE_PROCESS	0.84	0.14	0	0	0	not significant
7343	GO_NEGATIVE_REGULATION_OF_SMALL_GTPASE_MEDIATED_SIGNAL_TRANSDUCT	0.84	0.14	0	0	0	not significant
7344	GO_NEUROMUSCULAR_PROCESS_CONTROLLING_BALANCE	0.84	0.14	0	0	0	not significant
7345	GO_NEURON_PROJECTION_ORGANIZATION	0.84	0.14	0	0	0	not significant
7346	REACTOME_DEFECTS_IN_VITAMIN_AND_COFACTOR_METABOLISM	0.84	0.14	0	0	0	not significant
7347	SONG_TARGETS_OF_IE86_CMV_PROTEIN	0.84	0.14	0	0	0	not significant
7348	GO_CELL_ADHESION_MEDIATED_BY_INTEGRIN	0.84	0.13	0	0	0	not significant
7349	GO_CELL_RECOGNITION	0.84	0.13	0	0	0	not significant
7350	QI_HYPOXIA_TARGETS_OF_HIF1A_AND_FOXA2	0.84	0.13	0	0	0	not significant
7351	GO_FC_RECEPTOR_MEDIATED_STIMULATORY_SIGNALING_PATHWAY	0.84	0.12	0	0	0	not significant
7352	GO_REGULATION_OF_VIRAL_GENOME_REPLICATION	0.84	0.12	0	0	0	not significant
7353	ASTON_MAJOR_DEPRESSIVE_DISORDER_DN	0.84	0.11	0	0	0	not significant
7354	GO_REGULATION_OF_SYNAPSE_STRUCTURE_OR_ACTIVITY	0.84	0.11	0	0	0	not significant
7355	GO_GASTRULATION	0.84	0.1	0	0	0	not significant
7356	GO_POSTSYNAPTIC_MEMBRANE	0.84	0.1	0	0	0	not significant
7357	GO_CELL_JUNCTION_ORGANIZATION	0.84	0.09	0	0	0	not significant
7358	GO_REGULATION_OF_CELLULAR_RESPONSE_TO_GROWTH_FACTOR_STIMULUS	0.84	0.09	0	0	0	not significant
7359	GO_REGULATION_OF_METAL_ION_TRANSPORT	0.84	0.09	0	0	0	not significant
7360	CHR1P36	0.84	0.07	0	0	0	not significant
7361	GO_VIRAL_LIFE_CYCLE	0.84	0.07	0	0	0	not significant
7362	TBK1.DF_UP	0.84	0.07	0	0	0	not significant
7363	REACTOME_METABOLISM_OF_AMINO_ACIDS_AND_DERIVATIVES	0.84	0.06	0	0	0	not significant
7364	KIM_BIPOLAR_DISORDER_OLIGODENDROCYTE_DENSITY_CORR_UP	0.84	0.03	0	0	0	not significant
7365	CHR13Q13	0.85	0.2	0	0	0	not significant
7366	REACTOME_KERATAN_SULFATE_DEGRADATION	0.85	0.2	0	0	0	not significant
7367	GO_NEGATIVE_REGULATION_OF_PROTEIN_FOLDING	0.85	0.19	0	0	0	not significant
7368	GO_OXIDOREDUCTASE_ACTIVITY_ACTING_ON_A_SULFUR_GROUP_OF_DONORS_D	0.85	0.19	0	0	0	not significant
7369	GO_POST_EMBRYONIC_CAMERA_TYPE_EYE_DEVELOPMENT	0.85	0.19	0	0	0	not significant
7370	GO_REGULATION_OF_PERK_MEDIATED_UNFOLDED_PROTEIN_RESPONSE	0.85	0.19	0	0	0	not significant
7371	GO_RETINOL_METABOLIC_PROCESS	0.85	0.19	0	0	0	not significant
7372	CHR15Q23	0.85	0.18	0	0	0	not significant
7373	CHR16Q12	0.85	0.18	0	0	0	not significant
7374	CHR18Q22	0.85	0.18	0	0	0	not significant
7375	GO_APOPTOTIC_PROCESS_INVOLVED_IN_DEVELOPMENT	0.85	0.18	0	0	0	not significant
7376	GO_CYCLIN_DEPENDENT_PROTEIN_SERINE_THREONINE_KINASE_INHIBITOR_ACTIVI	0.85	0.18	0	0	0	not significant
7377	GO_HISTONE_H2A_ACETYLATION	0.85	0.18	0	0	0	not significant
7378	GO_NEGATIVE_REGULATION_OF_MICROTUBULE_DEPOLYMERIZATION	0.85	0.18	0	0	0	not significant
7379	GO_POSITIVE_REGULATION_OF_ENDOPLASMIC_RETICULUM_STRESS_INDUCED_INT	0.85	0.18	0	0	0	not significant
7380	GO_PROTEIN_INSERTION_INTO_MITOCHONDRIAL_MEMBRANE_INVOLVED_IN_APOPT	0.85	0.18	0	0	0	not significant
7381	GO_REGULATION_OF_EXTRACELLULAR_MATRIX_ASSEMBLY	0.85	0.18	0	0	0	not significant
7382	GO_RESPONSE_TO_COBALT_ION	0.85	0.18	0	0	0	not significant
7383	GO_SPERM_MIDPIECE	0.85	0.18	0	0	0	not significant
7384	GO_T_CELL_CHEMOTAXIS	0.85	0.18	0	0	0	not significant
7385	IWANAGA_E2F1_TARGETS_NOT_INDUCED_BY_SERUM	0.85	0.18	0	0	0	not significant
7386	JACKSON_DNMT1_TARGETS_DN	0.85	0.18	0	0	0	not significant
7387	REACTOME_DISPLACEMENT_OF_DNA_GLYCOSYLASE_BY_APEX1	0.85	0.18	0	0	0	not significant
7388	BIOCARTA_TOB1_PATHWAY	0.85	0.17	0	0	0	not significant
7389	GO_CALCIUM_CHANNEL_REGULATOR_ACTIVITY	0.85	0.17	0	0	0	not significant
7390	GO_CALCIUM_RELEASE_CHANNEL_ACTIVITY	0.85	0.17	0	0	0	not significant
7391	GO_DNA_POLYMERASE_BINDING	0.85	0.17	0	0	0	not significant
7392	GO_ENDOPLASMIC_RETICULUM_CHAPERONE_COMPLEX	0.85	0.17	0	0	0	not significant
7393	GO_HEPARAN_SULFATE_PROTEOGLYCAN_METABOLIC_PROCESS	0.85	0.17	0	0	0	not significant
7394	GO_K63_LINKED_POLYUBIQUITIN_MODIFICATION_DEPENDENT_PROTEIN_BINDING	0.85	0.17	0	0	0	not significant
7395	GO_MICROTUBULE_SEVERING_ATPASE_ACTIVITY	0.85	0.17	0	0	0	not significant
7396	GO_MITOCHONDRIAL_DEPOLARIZATION	0.85	0.17	0	0	0	not significant
7397	GO_NEGATIVE_REGULATION_OF_GLIAL_CELL_PROLIFERATION	0.85	0.17	0	0	0	not significant
7398	GO_NEGATIVE_REGULATION_OF_HISTONE_METHYLATION	0.85	0.17	0	0	0	not significant
7399	GO_NUCLEOTIDE_SUGAR_METABOLIC_PROCESS	0.85	0.17	0	0	0	not significant
7400	GO_REGULATION_OF_COLLAGEN_BIOSYNTHETIC_PROCESS	0.85	0.17	0	0	0	not significant

7401	GO_REGULATION_OF_VIRAL_ENTRY_INTO_HOST_CELL	0.85	0.17	0	0	0	not significant
7402	GO_U1_SNRNA_BINDING	0.85	0.17	0	0	0	not significant
7403	HOLLERN_PAPILLARY_BREAST_TUMOR	0.85	0.17	0	0	0	not significant
7404	LIANG_HEMATOPOIESIS_STEM_CELL_NUMBER_SMALL_VS_HUGE_DN	0.85	0.17	0	0	0	not significant
7405	MA_PITUITARY_FETAL_VS_ADULT_UP	0.85	0.17	0	0	0	not significant
7406	MANTOVANI_VIRAL_GPCR_SIGNALING_DN	0.85	0.17	0	0	0	not significant
7407	GO_ACTIVE_ION_TRANSMEMBRANE_TRANSPORTER_ACTIVITY	0.85	0.16	0	0	0	not significant
7408	GO_G_PROTEIN_ALPHA_SUBUNIT_BINDING	0.85	0.16	0	0	0	not significant
7409	GO_LOW_DENSITY_LIPOPROTEIN_RECEPTOR_PARTICLE_METABOLIC_PROCESS	0.85	0.16	0	0	0	not significant
7410	GO_POLYAMINE_BIOSYNTHETIC_PROCESS	0.85	0.16	0	0	0	not significant
7411	GO_POSITIVE_REGULATION_OF_GLUCOSE_TRANSMEMBRANE_TRANSPORT	0.85	0.16	0	0	0	not significant
7412	GO_REGULATION_OF_VASCULAR_SMOOTH_MUSCLE_CELL_PROLIFERATION	0.85	0.16	0	0	0	not significant
7413	JIANG_HYPOXIA_VIA_VHL	0.85	0.16	0	0	0	not significant
7414	REACTOME_DAP12_INTERACTIONS	0.85	0.16	0	0	0	not significant
7415	REACTOME_NCAM_SIGNALING_FOR_NEURITE_OUT_GROWTH	0.85	0.16	0	0	0	not significant
7416	REACTOME_TRANSPORT_OF_VITAMINS_NUCLEOSIDES_AND_RELATED_MOLECULE	0.85	0.16	0	0	0	not significant
7417	YAMASHITA_LIVER_CANCER_WITH_EPCAM_UP	0.85	0.16	0	0	0	not significant
7418	CHENG_IMPRINTED_BY_ESTRADIOL	0.85	0.15	0	0	0	not significant
7419	DELASERNA_MYOD_TARGETS_UP	0.85	0.15	0	0	0	not significant
7420	GO_CARDIAC_VENTRICLE_MORPHOGENESIS	0.85	0.15	0	0	0	not significant
7421	GO_GLAND_MORPHOGENESIS	0.85	0.15	0	0	0	not significant
7422	GO_POTASSIUM_CHANNEL_COMPLEX	0.85	0.15	0	0	0	not significant
7423	JIANG_TIP30_TARGETS_UP	0.85	0.15	0	0	0	not significant
7424	REACTOME_INTERLEUKIN_3_INTERLEUKIN_5_AND_GM_CSF_SIGNALING	0.85	0.15	0	0	0	not significant
7425	CHR4P16	0.85	0.14	0	0	0	not significant
7426	GO_MICROBODY_MEMBRANE	0.85	0.14	0	0	0	not significant
7427	GO_REGULATION_OF_PHOSPHATIDYLINOSITOL_3_KINASE_SIGNALING	0.85	0.14	0	0	0	not significant
7428	KEGG_B_CELL_RECEPTOR_SIGNALING_PATHWAY	0.85	0.14	0	0	0	not significant
7429	PRC2_SUZ12_UP.V1_DN	0.85	0.14	0	0	0	not significant
7430	GO_ERYTHROCYTE_HOMEOSTASIS	0.85	0.13	0	0	0	not significant
7431	GO_REGULATION_OF_ESTABLISHMENT_OF_PROTEIN_LOCALIZATION_TO_MITOCH(	0.85	0.13	0	0	0	not significant
7432	GO_NOTCH_SIGNALING_PATHWAY	0.85	0.12	0	0	0	not significant
7433	GO_ORGANELLE_FUSION	0.85	0.12	0	0	0	not significant
7434	GO_PROTEIN_KINASE_B_SIGNALING	0.85	0.11	0	0	0	not significant
7435	REACTOME_SELENOAMINO_ACID_METABOLISM	0.85	0.11	0	0	0	not significant
7436	WAMUNYOKOLI_OVARIAN_CANCER_LMP_UP	0.85	0.11	0	0	0	not significant
7437	GO_OUTER_MEMBRANE	0.85	0.1	0	0	0	not significant
7438	BERTUCCI_MEDULLARY_VS_DUCTAL_BREAST_CANCER_UP	0.85	0.09	0	0	0	not significant
7439	BROWNE_HCMV_INFECTION_4HR_DN	0.85	0.09	0	0	0	not significant
7440	IWANAGA_CARCINOGENESIS_BY_KRAS_PTEN_DN	0.85	0.09	0	0	0	not significant
7441	GO_NEGATIVE_REGULATION_OF_TRANSFERASE_ACTIVITY	0.85	0.08	0	0	0	not significant
7442	UDAYAKUMAR_MED1_TARGETS_DN	0.85	0.08	0	0	0	not significant
7443	BAELDE_DIABETIC_NEPHROPATHY_DN	0.85	0.07	0	0	0	not significant
7444	GO_REGULATION_OF_CELL_PROJECTION_ORGANIZATION	0.85	0.06	0	0	0	not significant
7445	HSIAO_HOUSEKEEPING_GENES	0.85	0.06	0	0	0	not significant
7446	BUYTAERT_PHOTODYNAMIC_THERAPY_STRESS_DN	0.85	0.04	0	0	0	not significant
7447	KIM_ALL_DISORDERS_OLIGODENDROCYTE_NUMBER_CORR_UP	0.85	0.04	0	0	0	not significant
7448	BONOME_OVARIAN_CANCER_POOR_SURVIVAL_DN	0.86	0.2	0	0	0	not significant
7449	COLIN_PILOCYTIC_ASTROCYTOMA_VS_GLIOBLASTOMA_UP	0.86	0.2	0	0	0	not significant
7450	GO_POSITIVE_REGULATION_OF_MONOCYTE_CHEMOTAXIS	0.86	0.2	0	0	0	not significant
7451	GO_REGULATION_OF_LYSOSOMAL_PROTEIN_CATABOLIC_PROCESS	0.86	0.2	0	0	0	not significant
7452	BOYAULT_LIVER_CANCER_SUBCLASS_G2	0.86	0.19	0	0	0	not significant
7453	CHR1P21	0.86	0.19	0	0	0	not significant
7454	CLIMENT_BREAST_CANCER_COPY_NUMBER_UP	0.86	0.19	0	0	0	not significant
7455	GHANDHI_DIRECT_IRRADIATION_DN	0.86	0.19	0	0	0	not significant
7456	GO_CELLULAR_LIPID_BIOSYNTHETIC_PROCESS	0.86	0.19	0	0	0	not significant
7457	GO_CGMP_MEDIATED_SIGNALING	0.86	0.19	0	0	0	not significant
7458	GO_CHLORIDE_CHANNEL_REGULATOR_ACTIVITY	0.86	0.19	0	0	0	not significant
7459	GO_INWARD_RECTIFIER_POTASSIUM_CHANNEL_ACTIVITY	0.86	0.19	0	0	0	not significant
7460	GO_NEGATIVE_REGULATION_OF_PROTEIN_LOCALIZATION_TO_MICROTUBULE	0.86	0.19	0	0	0	not significant
7461	GO_PHOSPHODIESTERASE_I_ACTIVITY	0.86	0.19	0	0	0	not significant
7462	GO_POTASSIUM_ION_IMPORT_ACROSS_PLASMA_MEMBRANE	0.86	0.19	0	0	0	not significant
7463	GO_PROTEIN_PHOSPHATASE_1_BINDING	0.86	0.19	0	0	0	not significant
7464	GO_PROTOPORPHYRINOGEN_IX_BIOSYNTHETIC_PROCESS	0.86	0.19	0	0	0	not significant
7465	GO_TROPHOBLAST_GIANT_CELL_DIFFERENTIATION	0.86	0.19	0	0	0	not significant
7466	KIM_LIVER_CANCER_POOR_SURVIVAL_DN	0.86	0.19	0	0	0	not significant
7467	LUI_THYROID_CANCER_CLUSTER_2	0.86	0.19	0	0	0	not significant
7468	PID_IL2_STAT5_PATHWAY	0.86	0.19	0	0	0	not significant
7469	WATANABE_COLON_CANCER_MSI_VS_MSS_DN	0.86	0.19	0	0	0	not significant
7470	ZIRN_TRETINOIN_RESPONSE_WT1_DN	0.86	0.19	0	0	0	not significant
7471	BYSTROEM_CORRELATED_WITH_IL5_UP	0.86	0.18	0	0	0	not significant
7472	CHEN_ETV5_TARGETS_TESTIS	0.86	0.18	0	0	0	not significant
7473	CROONQUIST_NRAS_SIGNALING_UP	0.86	0.18	0	0	0	not significant
7474	EINAV_INTERFERON_SIGNATURE_IN_CANCER	0.86	0.18	0	0	0	not significant
7475	ENGELMANN_CANCER_PROGENITORS_UP	0.86	0.18	0	0	0	not significant
7476	GO_ACTIVATION_OF_JANUS_KINASE_ACTIVITY	0.86	0.18	0	0	0	not significant
7477	GO_CERAMIDE_METABOLIC_PROCESS	0.86	0.18	0	0	0	not significant
7478	GO_INSULIN_LIKE_GROWTH_FACTOR_RECEPTOR_SIGNALING_PATHWAY	0.86	0.18	0	0	0	not significant
7479	GO_L_GLUTAMATE_TRANSMEMBRANE_TRANSPORT	0.86	0.18	0	0	0	not significant
7480	GO_NEGATIVE_REGULATION_OF_BIOMINERAL_TISSUE_DEVELOPMENT	0.86	0.18	0	0	0	not significant
7481	GO_NEGATIVE_REGULATION_OF_PLASMA_MEMBRANE_BOUNDED_CELL_PROJECTI	0.86	0.18	0	0	0	not significant
7482	GO_PEPTIDYL_TYROSINE_AUTOPHOSPHORYLATION	0.86	0.18	0	0	0	not significant
7483	GO_POSITIVE_REGULATION_OF_HUMORAL_IMMUNE_RESPONSE	0.86	0.18	0	0	0	not significant
7484	GO_REGULATION_OF_B_CELL_APOPTOTIC_PROCESS	0.86	0.18	0	0	0	not significant
7485	GO_REGULATION_OF_POSTSYNAPTIC_SPECIALIZATION_ASSEMBLY	0.86	0.18	0	0	0	not significant
7486	GO_RESPONSE_TO_MISFOLDED_PROTEIN	0.86	0.18	0	0	0	not significant
7487	GO_TELOMERIC_LOOP_DISASSEMBLY	0.86	0.18	0	0	0	not significant
7488	GO_TRANSFERRIN_TRANSPORT	0.86	0.18	0	0	0	not significant
7489	KYNG_DNA_DAMAGE_BY_GAMMA_RADIATION	0.86	0.18	0	0	0	not significant
7490	PID_REELIN_PATHWAY	0.86	0.18	0	0	0	not significant
7491	GARGALOVIC_RESPONSE_TO_OXIDIZED_PHOSPHOLIPIDS_LIGHTYELLOW_UP	0.86	0.17	0	0	0	not significant
7492	GO_3_PHOSPHOADENOSINE_5_PHOSPHOSULFATE_BIOSYNTHETIC_PROCESS	0.86	0.17	0	0	0	not significant
7493	GO_ADIPONECTIN_ACTIVATED_SIGNALING_PATHWAY	0.86	0.17	0	0	0	not significant
7494	GO_AGGRESOME	0.86	0.17	0	0	0	not significant
7495	GO_MUCUS_SECRETION	0.86	0.17	0	0	0	not significant
7496	GO_REGULATION_OF_CAMP_MEDIATED_SIGNALING	0.86	0.17	0	0	0	not significant
7497	GO_REGULATION_OF_PROTEIN_TARGETING_TO_MITOCHONDRION	0.86	0.17	0	0	0	not significant
7498	ROESSLER_LIVER_CANCER_METASTASIS_DN	0.86	0.17	0	0	0	not significant
7499	GO_AMINE_TRANSPORT	0.86	0.16	0	0	0	not significant
7500	GO_CELLULAR_RESPONSE_TO_GLUCOSE_STARVATION	0.86	0.16	0	0	0	not significant
7501	GO_ENDOSOME_TO_LYSOSOME_TRANSPORT	0.86	0.16	0	0	0	not significant
7502	GO_REGULATION_OF_LEUKOCYTE_DEGRANULATION	0.86	0.16	0	0	0	not significant
7503	GO_REGULATION_OF_PROTEIN_ACETYLATION	0.86	0.16	0	0	0	not significant
7504	GO_SENSORY_PERCEPTION_OF_LIGHT_STIMULUS	0.86	0.16	0	0	0	not significant
7505	GO_SIGNALING_ADAPTOR_ACTIVITY	0.86	0.16	0	0	0	not significant
7506	GO_VOLTAGE_GATED_CATION_CHANNEL_ACTIVITY	0.86	0.16	0	0	0	not significant
7507	LIN_SILENCED_BY_TUMOR_MICROENVIRONMENT	0.86	0.16	0	0	0	not significant
7508	CHR4Q31	0.86	0.15	0	0	0	not significant
7509	GO_ORGANELLE_DISASSEMBLY	0.86	0.15	0	0	0	not significant

7510	GO_PROTEIN_LOCALIZATION_TO_SYNAPSE	0.86	0.15	0	0	0	not significant
7511	FIGUEROA_AML_METHYLATION_CLUSTER_4_UP	0.86	0.14	0	0	0	not significant
7512	GO_EXTRINSIC_COMPONENT_OF_PLASMA_MEMBRANE	0.86	0.14	0	0	0	not significant
7513	GO_PURINE_CONTAINING_COMPOUND_METABOLIC_PROCESS	0.86	0.14	0	0	0	not significant
7514	GO_REGULATION_OF_INTRINSIC_APOPTOTIC_SIGNALING_PATHWAY	0.86	0.13	0	0	0	not significant
7515	KIM_ALL_DISORDERS_DURATION_CORR_DN	0.86	0.13	0	0	0	not significant
7516	STEARMAN_LUNG_CANCER_EARLY_VS_LATE_UP	0.86	0.13	0	0	0	not significant
7517	GO_NEGATIVE_REGULATION_OF_KINASE_ACTIVITY	0.86	0.12	0	0	0	not significant
7518	GO_REGULATION_OF_CHROMATIN_ORGANIZATION	0.86	0.12	0	0	0	not significant
7519	GO_SEX_DIFFERENTIATION	0.86	0.12	0	0	0	not significant
7520	DAVICIONI_MOLECULAR_ARMS_VS_ERMS_UP	0.86	0.11	0	0	0	not significant
7521	GO_NEURON_TO_NEURON_SYNAPSE	0.86	0.11	0	0	0	not significant
7522	GO_POSITIVE_REGULATION_OF_PROTEIN_SERINE_THREONINE_KINASE_ACTIVITY	0.86	0.11	0	0	0	not significant
7523	GO_TRANSFERASE_ACTIVITY_TRANSFERRING_ACYL_GROUPS	0.86	0.11	0	0	0	not significant
7524	LOCKWOOD_AMPLIFIED_IN_LUNG_CANCER	0.86	0.11	0	0	0	not significant
7525	GO_POSITIVE_REGULATION_OF_GTPASE_ACTIVITY	0.86	0.1	0	0	0	not significant
7526	GRUETZMANN_PANCREATIC_CANCER_UP	0.86	0.1	0	0	0	not significant
7527	NIKOLSKY_BREAST_CANCER_17Q21_Q25_AMPLICON	0.86	0.1	0	0	0	not significant
7528	GO_ENDOMEMBRANE_SYSTEM_ORGANIZATION	0.86	0.09	0	0	0	not significant
7529	RAO_BOUND_BY_SALL4_ISOFORM_B	0.86	0.09	0	0	0	not significant
7530	GO_NEGATIVE_REGULATION_OF_PROTEIN_MODIFICATION_PROCESS	0.86	0.08	0	0	0	not significant
7531	GO_NUCLEOBASE_CONTAINING_SMALL_MOLECULE_METABOLIC_PROCESS	0.86	0.08	0	0	0	not significant
7532	REACTOME_DNA_REPAIR	0.86	0.08	0	0	0	not significant
7533	GO_SYNAPSE_PART	0.86	0.07	0	0	0	not significant
7534	GO_DNA_REPAIR	0.86	0.06	0	0	0	not significant
7535	GO_CELL_PROJECTION_PART	0.86	0.04	0	0	0	not significant
7536	GO_CONNECTIVE_TISSUE_REPLACEMENT	0.87	0.21	0	0	0	not significant
7537	GO_G_QUADRUPLEX_DNA_UNWINDING	0.87	0.21	0	0	0	not significant
7538	GO_REGULATION_OF_LIGASE_ACTIVITY	0.87	0.21	0	0	0	not significant
7539	KUROKAWA_LIVER_CANCER_CHEMOTHERAPY_UP	0.87	0.21	0	0	0	not significant
7540	GO_AMINE_BIOSYNTHETIC_PROCESS	0.87	0.2	0	0	0	not significant
7541	GO_BLEB_ASSEMBLY	0.87	0.2	0	0	0	not significant
7542	GO_NAD_BIOSYNTHETIC_PROCESS	0.87	0.2	0	0	0	not significant
7543	GO_POSITIVE_REGULATION_OF_MESONEPHROS_DEVELOPMENT	0.87	0.2	0	0	0	not significant
7544	GO_POSITIVE_REGULATION_OF_SIGNAL_TRANSDUCTION_BY_P53_CLASS_MEDIAT(	0.87	0.2	0	0	0	not significant
7545	GO_PYRIMIDINE_CONTAINING_COMPOUND_SALVAGE	0.87	0.2	0	0	0	not significant
7546	GO_REGULATION_OF_POSTSYNAPTIC_NEUROTRANSMITTER_RECEPTOR_ACTIVITY	0.87	0.2	0	0	0	not significant
7547	GO_TRANS_GOLGI_NETWORK_TRANSPORT_VESICLE_MEMBRANE	0.87	0.2	0	0	0	not significant
7548	REACTOME_ACYL_CHAIN_REMODELLING_OF_PG	0.87	0.2	0	0	0	not significant
7549	REACTOME_APEX1_INDEPENDENT_RESOLUTION_OF_AP_SITES_VIA_THE_SINGLE_I	0.87	0.2	0	0	0	not significant
7550	RIZ_ERYTHROID_DIFFERENTIATION_HEMGN	0.87	0.2	0	0	0	not significant
7551	SHARMA_PILOCYTIC_ASTROCYTOMA_LOCATION_UP	0.87	0.2	0	0	0	not significant
7552	TOMLINS_METASTASIS_UP	0.87	0.2	0	0	0	not significant
7553	WANG_BARRETTS_ESOPHAGUS_AND_ESOPHAGUS_CANCER_DN	0.87	0.2	0	0	0	not significant
7554	CLAUS_PGR_POSITIVE_MENINGIOMA_DN	0.87	0.19	0	0	0	not significant
7555	GO_BRCA1_A_COMPLEX	0.87	0.19	0	0	0	not significant
7556	GO_CYTOPLASMIC_SIDE_OF_ROUGH_ENDOPLASMIC_RETICULUM_MEMBRANE	0.87	0.19	0	0	0	not significant
7557	GO_LAGGING_STRAND_ELONGATION	0.87	0.19	0	0	0	not significant
7558	GO_PHARYNGEAL_SYSTEM_DEVELOPMENT	0.87	0.19	0	0	0	not significant
7559	GO_PIGMENT_GRANULE_LOCALIZATION	0.87	0.19	0	0	0	not significant
7560	GO_POSITIVE_REGULATION_OF_T_HELPER_1_TYPE_IMMUNE_RESPONSE	0.87	0.19	0	0	0	not significant
7561	GO_PURINE_CONTAINING_COMPOUND_CATABOLIC_PROCESS	0.87	0.19	0	0	0	not significant
7562	GO_REGULATION_OF_BONE_REMODELING	0.87	0.19	0	0	0	not significant
7563	GO_REGULATION_OF_PROTEIN_POLYUBIQUITINATION	0.87	0.19	0	0	0	not significant
7564	GO_TOR_COMPLEX	0.87	0.19	0	0	0	not significant
7565	KEGG_PORPHYRIN_AND_CHLOROPHYLL_METABOLISM	0.87	0.19	0	0	0	not significant
7566	LIANG_HEMATOPOIESIS_STEM_CELL_NUMBER_LARGE_VS_TINY_DN	0.87	0.19	0	0	0	not significant
7567	PAL_PRMT5_TARGETS_DN	0.87	0.19	0	0	0	not significant
7568	RAMJAUN_APOPTOSIS_BY_TGFB1_VIA_SMAD4_DN	0.87	0.19	0	0	0	not significant
7569	REACTOME_APOPTOTIC_CLEAVAGE_OF_CELLULAR_PROTEINS	0.87	0.19	0	0	0	not significant
7570	ZHAN_MULTIPLE_MYELOMA_MS_DN	0.87	0.19	0	0	0	not significant
7571	ZHOU_TNF_SIGNALING_4HR	0.87	0.19	0	0	0	not significant
7572	GILDEA_METASTASIS	0.87	0.18	0	0	0	not significant
7573	GO_DRUG_CATABOLIC_PROCESS	0.87	0.18	0	0	0	not significant
7574	GO_ENDOCYTIC_RECYCLING	0.87	0.18	0	0	0	not significant
7575	GO_HEPARAN_SULFATE_SULFOTRANSFERASE_ACTIVITY	0.87	0.18	0	0	0	not significant
7576	GO_NEGATIVE_REGULATION_OF_UBIQUITIN_PROTEIN_TRANSFERASE_ACTIVITY	0.87	0.18	0	0	0	not significant
7577	GO_RESPONSE_TO_OXYGEN_RADICAL	0.87	0.18	0	0	0	not significant
7578	GO_SYNAPTIC_TRANSMISSION_DOPAMINERGIC	0.87	0.18	0	0	0	not significant
7579	GO_X_CHROMOSOME	0.87	0.18	0	0	0	not significant
7580	KEGG_AMYOTROPHIC_LATERAL_SCLEROSIS_ALS	0.87	0.18	0	0	0	not significant
7581	KYNG_DNA_DAMAGE_BY_GAMMA_AND_UV_RADIATION	0.87	0.18	0	0	0	not significant
7582	LIANG_HEMATOPOIESIS_STEM_CELL_NUMBER_QTL	0.87	0.18	0	0	0	not significant
7583	PID_AR_TF_PATHWAY	0.87	0.18	0	0	0	not significant
7584	REACTOME_TRANSPORT_AND_SYNTHESIS_OF_PAPS	0.87	0.18	0	0	0	not significant
7585	GO_NEGATIVE_REGULATION_OF_METALLOPEPTIDASE_ACTIVITY	0.87	0.17	0	0	0	not significant
7586	GO_REGULATION_OF_VESICLE_FUSION	0.87	0.17	0	0	0	not significant
7587	HUMMERICH_SKIN_CANCER_PROGRESSION_DN	0.87	0.17	0	0	0	not significant
7588	ROSS_AML_OF_FAB_M7_TYPE	0.87	0.17	0	0	0	not significant
7589	VECCHI_GASTRIC_CANCER_ADVANCED_VS_EARLY_UP	0.87	0.17	0	0	0	not significant
7590	WU_CELL_MIGRATION	0.87	0.17	0	0	0	not significant
7591	CHR15Q15	0.87	0.16	0	0	0	not significant
7592	GO_ENDONUCLEASE_ACTIVITY	0.87	0.16	0	0	0	not significant
7593	GO_FILOPODIUM	0.87	0.16	0	0	0	not significant
7594	GO_REGULATION_OF_MUSCLE_ORGAN_DEVELOPMENT	0.87	0.16	0	0	0	not significant
7595	PID_CD8_TCR_PATHWAY	0.87	0.16	0	0	0	not significant
7596	REACTOME_BIOLOGICAL_OXIDATIONS	0.87	0.16	0	0	0	not significant
7597	BENPORATH_ES_CORE_NINE_CORRELATED	0.87	0.15	0	0	0	not significant
7598	GO_NEGATIVE_REGULATION_OF_DNA_BINDING_TRANSCRIPTION_FACTOR_ACTIVIT	0.87	0.15	0	0	0	not significant
7599	GO_REGULATION_OF_REGULATED_SECRETORY_PATHWAY	0.87	0.15	0	0	0	not significant
7600	CHR21Q22	0.87	0.14	0	0	0	not significant
7601	GO_CALCIUM_MEDIATED_SIGNALING	0.87	0.14	0	0	0	not significant
7602	GO_CYTOPLASMIC_SIDE_OF_MEMBRANE	0.87	0.14	0	0	0	not significant
7603	GO_NEURAL_TUBE_DEVELOPMENT	0.87	0.14	0	0	0	not significant
7604	GO_POSITIVE_REGULATION_OF_INTRACELLULAR_PROTEIN_TRANSPORT	0.87	0.14	0	0	0	not significant
7605	GO_REGULATION_OF_HISTONE_MODIFICATION	0.87	0.14	0	0	0	not significant
7606	BROWNE_HCMV_INFECTION_48HR_UP	0.87	0.13	0	0	0	not significant
7607	GO_PLATELET_ACTIVATION	0.87	0.13	0	0	0	not significant
7608	GO_REGULATION_OF_MYELOID_CELL_DIFFERENTIATION	0.87	0.13	0	0	0	not significant
7609	GO_ACTIN_BINDING	0.87	0.12	0	0	0	not significant
7610	GO_REGULATION_OF_DEVELOPMENTAL_GROWTH	0.87	0.12	0	0	0	not significant
7611	GO_GTPASE_REGULATOR_ACTIVITY	0.87	0.11	0	0	0	not significant
7612	GO_SENSORY_ORGAN_DEVELOPMENT	0.87	0.11	0	0	0	not significant
7613	GO_ESTABLISHMENT_OF_PROTEIN_LOCALIZATION_TO_MEMBRANE	0.87	0.1	0	0	0	not significant
7614	GO_REGULATION_OF_HEMOPOIESIS	0.87	0.09	0	0	0	not significant
7615	GO_CHROMATIN_BINDING	0.87	0.08	0	0	0	not significant
7616	BENPORATH_SOX2_TARGETS	0.87	0.07	0	0	0	not significant
7617	GO_SUPRAMOLECULAR_FIBER_ORGANIZATION	0.87	0.07	0	0	0	not significant
7618	GO_POSITIVE_REGULATION_OF_CALCIUM_ION_DEPENDENT_EXOCYTOSIS	0.88	0.22	0	0	0	not significant

7619	GO_REGULATION_OF_NECROTIC_CELL_DEATH	0.88	0.22	0	0	0	not significant
7620	BIOCARTA_PLK3_PATHWAY	0.88	0.21	0	0	0	not significant
7621	CHIANG_LIVER_CANCER_SUBCLASS_POLYSOMY7_DN	0.88	0.21	0	0	0	not significant
7622	GO_3_5_DNA_HELICASE_ACTIVITY	0.88	0.21	0	0	0	not significant
7623	GO_GLYOXYLATE_METABOLIC_PROCESS	0.88	0.21	0	0	0	not significant
7624	GO_NEGATIVE_REGULATION_OF_MITOCHONDRIAL_OUTER_MEMBRANE_PERMEABI	0.88	0.21	0	0	0	not significant
7625	GO_POSITIVE_REGULATION_OF_RECEPTOR_RECYCLING	0.88	0.21	0	0	0	not significant
7626	GO_PURINE_NUCLEOTIDE_TRANSMEMBRANE_TRANSPORTER_ACTIVITY	0.88	0.21	0	0	0	not significant
7627	GO_REGULATION_OF_LYMPHOCYTE_APOPTOTIC_PROCESS	0.88	0.21	0	0	0	not significant
7628	GO_REGULATION_OF_MESONEPHROS_DEVELOPMENT	0.88	0.21	0	0	0	not significant
7629	GO_REGULATION_OF_SODIUM_ION_TRANSMEMBRANE_TRANSPORTER_ACTIVITY	0.88	0.21	0	0	0	not significant
7630	GO_TRNA_CATABOLIC_PROCESS	0.88	0.21	0	0	0	not significant
7631	GO_ZYMOGEN_GRANULE	0.88	0.21	0	0	0	not significant
7632	PID_HEDGEHOG_2PATHWAY	0.88	0.21	0	0	0	not significant
7633	REACTOME_CASPASE_ACTIVATION_VIA_EXTRINSIC_APOPTOTIC_SIGNALLING_PATH	0.88	0.21	0	0	0	not significant
7634	ROYLANCE_BREAST_CANCER_16Q_COPY_NUMBER_DN	0.88	0.21	0	0	0	not significant
7635	SA_FAS_SIGNALING	0.88	0.21	0	0	0	not significant
7636	YOSHIOKA_LIVER_CANCER_EARLY_RECURRENCE_DN	0.88	0.21	0	0	0	not significant
7637	CHOW_RASSF1_TARGETS_UP	0.88	0.2	0	0	0	not significant
7638	GENTILE_UV_RESPONSE_CLUSTER_D7	0.88	0.2	0	0	0	not significant
7639	GO_DNA_DOUBLE_STRAND_BREAK_PROCESSING	0.88	0.2	0	0	0	not significant
7640	GO_ENDOCARDIAL_CUSHION_DEVELOPMENT	0.88	0.2	0	0	0	not significant
7641	GO_ENDOSOME_TO_PLASMA_MEMBRANE_PROTEIN_TRANSPORT	0.88	0.2	0	0	0	not significant
7642	GO_INOSITOL_PHOSPHATE_BIOSYNTHETIC_PROCESS	0.88	0.2	0	0	0	not significant
7643	GO_NEGATIVE_REGULATION_OF_EPITHELIAL_CELL_MIGRATION	0.88	0.2	0	0	0	not significant
7644	GO_POSITIVE_REGULATION_OF_MONONUCLEAR_CELL_MIGRATION	0.88	0.2	0	0	0	not significant
7645	GO_REGULATION_OF_ACTIVIN_RECEPTOR_SIGNALING_PATHWAY	0.88	0.2	0	0	0	not significant
7646	GO_REGULATION_OF_METALLOPEPTIDASE_ACTIVITY	0.88	0.2	0	0	0	not significant
7647	GO_REGULATION_OF_VASCULAR_SMOOTH_MUSCLE_CELL_DIFFERENTIATION	0.88	0.2	0	0	0	not significant
7648	GO_RESPONSE_TO_POTASSIUM_ION	0.88	0.2	0	0	0	not significant
7649	GO_VASCULAR_ENDOTHELIAL_GROWTH_FACTOR_RECEPTOR_BINDING	0.88	0.2	0	0	0	not significant
7650	REACTOME_TRANSCRIPTIONAL_REGULATION_OF_PLURIPOTENT_STEM_CELLS	0.88	0.2	0	0	0	not significant
7651	TONKS_TARGETS_OF_RUNX1_RUNX1T1_FUSION_ERYTHROCYTE_DN	0.88	0.2	0	0	0	not significant
7652	GO_ATP_HYDROLYSIS_COUPLED_CATION_TRANSMEMBRANE_TRANSPORT	0.88	0.19	0	0	0	not significant
7653	GO_BONE_MINERALIZATION	0.88	0.19	0	0	0	not significant
7654	GO_CYSTEINE_TYPE_ENDOPEPTIDASE_INHIBITOR_ACTIVITY_INVOLVED_IN_APOPT(	0.88	0.19	0	0	0	not significant
7655	GO_CYTOSOLIC_LARGE_RIBOSOMAL_SUBUNIT	0.88	0.19	0	0	0	not significant
7656	GO_DNA_RECOMBINASE_MEDIATOR_COMPLEX	0.88	0.19	0	0	0	not significant
7657	GO_FATTY_ACID_TRANSPORT	0.88	0.19	0	0	0	not significant
7658	GO_MULTIVESICULAR_BODY_ORGANIZATION	0.88	0.19	0	0	0	not significant
7659	GO_PROTEIN_SELF_ASSOCIATION	0.88	0.19	0	0	0	not significant
7660	GO_VASCULAR_SMOOTH_MUSCLE_CELL_DIFFERENTIATION	0.88	0.19	0	0	0	not significant
7661	HERNANDEZ_MITOTIC_ARREST_BY_DOCETAXEL_1_DN	0.88	0.19	0	0	0	not significant
7662	HOWLIN_CITED1_TARGETS_1_UP	0.88	0.19	0	0	0	not significant
7663	LABBE_TARGETS_OF_TGFB1_AND_WNT3A_DN	0.88	0.19	0	0	0	not significant
7664	LANDIS_ERBB2_BREAST_TUMORS_65_DN	0.88	0.19	0	0	0	not significant
7665	REACTOME_DISEASES_OF_CARBOHYDRATE_METABOLISM	0.88	0.19	0	0	0	not significant
7666	REACTOME_PREGNENOLONE_BIOSYNTHESIS	0.88	0.19	0	0	0	not significant
7667	ATM_DN.V1_DN	0.88	0.18	0	0	0	not significant
7668	BURTON_ADIPOGENESIS_8	0.88	0.18	0	0	0	not significant
7669	CHR15Q24	0.88	0.18	0	0	0	not significant
7670	GO_CALCIUM_ION_TRANSMEMBRANE_TRANSPORTER_ACTIVITY	0.88	0.18	0	0	0	not significant
7671	GO_FEMALE_SEX_DIFFERENTIATION	0.88	0.18	0	0	0	not significant
7672	GO_NEGATIVE_REGULATION_OF_RESPONSE_TO_BIOTIC_STIMULUS	0.88	0.18	0	0	0	not significant
7673	GO_PROTEIN_TYROSINE_KINASE_ACTIVITY	0.88	0.18	0	0	0	not significant
7674	GO_REGULATION_OF_OXIDATIVE_STRESS_INDUCED_CELL_DEATH	0.88	0.18	0	0	0	not significant
7675	KEGG_COLORECTAL_CANCER	0.88	0.18	0	0	0	not significant
7676	PID_ANGIOPOIETIN_RECEPTOR_PATHWAY	0.88	0.18	0	0	0	not significant
7677	PID_HDAC_CLASSI_PATHWAY	0.88	0.18	0	0	0	not significant
7678	VANDESLUIS_COMMD1_TARGETS_GROUP_3_UP	0.88	0.18	0	0	0	not significant
7679	CHUNG_BLISTER_CYTOTOXICITY_UP	0.88	0.17	0	0	0	not significant
7680	FONTAINE_PAPILLARY_THYROID_CARCINOMA_DN	0.88	0.17	0	0	0	not significant
7681	GO_ADAPTIVE_THERMOGENESIS	0.88	0.17	0	0	0	not significant
7682	GO_DEMETHYLATION	0.88	0.17	0	0	0	not significant
7683	GO_DIOXYGENASE_ACTIVITY	0.88	0.17	0	0	0	not significant
7684	GO_HISTONE_H3_K4_METHYLATION	0.88	0.17	0	0	0	not significant
7685	GO_REGULATION_OF_EXTENT_OF_CELL_GROWTH	0.88	0.17	0	0	0	not significant
7686	MIKKELSEN_MCV6_HCP_WITH_H3K27ME3	0.88	0.17	0	0	0	not significant
7687	REACTOME_MEIOTIC_SYNAPSIS	0.88	0.17	0	0	0	not significant
7688	BOSCO_ALLERGEN_INDUCED_TH2_ASSOCIATED_MODULE	0.88	0.16	0	0	0	not significant
7689	GO_HISTONE_DEACETYLASE_COMPLEX	0.88	0.16	0	0	0	not significant
7690	GO_NEGATIVE_REGULATION_OF_CELL_PROJECTION_ORGANIZATION	0.88	0.16	0	0	0	not significant
7691	REACTOME_G_ALPHA_Q_SIGNALLING_EVENTS	0.88	0.16	0	0	0	not significant
7692	GO_RESPONSE_TO_TRANSFORMING_GROWTH_FACTOR_BETA	0.88	0.15	0	0	0	not significant
7693	GO_MUSCLE_CONTRACTION	0.88	0.14	0	0	0	not significant
7694	GO_REGULATION_OF_CELLULAR_COMPONENT_SIZE	0.88	0.12	0	0	0	not significant
7695	GO_REPRODUCTIVE_SYSTEM_DEVELOPMENT	0.88	0.11	0	0	0	not significant
7696	BLALOCK_ALZHEIMERS_DISEASE_INCIPIENT_UP	0.88	0.1	0	0	0	not significant
7697	GO_REGULATION_OF_GROWTH	0.88	0.1	0	0	0	not significant
7698	GO_RESPONSE_TO_WOUNDING	0.88	0.09	0	0	0	not significant
7699	GO_NEURON_DIFFERENTIATION	0.88	0.06	0	0	0	not significant
7700	GO_POSITIVE_REGULATION_OF_DENDRITIC_SPINE_MORPHOGENESIS	0.89	0.23	0	0	0	not significant
7701	GO_REGULATION_OF_SMOOTH_MUSCLE_CELL_DIFFERENTIATION	0.89	0.23	0	0	0	not significant
7702	CAFFAREL_RESPONSE_TO_THC_24HR_3_UP	0.89	0.22	0	0	0	not significant
7703	GO_DNA_INSERTION_OR_DELETION_BINDING	0.89	0.22	0	0	0	not significant
7704	GO_ENDOCARDIAL_CUSHION_FORMATION	0.89	0.22	0	0	0	not significant
7705	GO_MONOCARBOXYLIC_ACID_TRANSMEMBRANE_TRANSPORTER_ACTIVITY	0.89	0.22	0	0	0	not significant
7706	GO_NEGATIVE_REGULATION_OF_EPIDERMAL_GROWTH_FACTOR_ACTIVATED_RE(	0.89	0.22	0	0	0	not significant
7707	GO_PINOCYTOSIS	0.89	0.22	0	0	0	not significant
7708	GO_PRESYNAPTIC_MODULATION_OF_CHEMICAL_SYNAPTIC_TRANSMISSION	0.89	0.22	0	0	0	not significant
7709	GO_PURINE_NUCLEOTIDE_TRANSPORT	0.89	0.22	0	0	0	not significant
7710	GO_SYNAPTIC_MEMBRANE_ADHESION	0.89	0.22	0	0	0	not significant
7711	KORKOLA_TERATOMA_UP	0.89	0.22	0	0	0	not significant
7712	LIANG_HEMATOPOIESIS_STEM_CELL_NUMBER_LARGE_VS_TINY_UP	0.89	0.22	0	0	0	not significant
7713	PID_S1P_S1P3_PATHWAY	0.89	0.22	0	0	0	not significant
7714	REACTOME_NRIF_SIGNALS_CELL_DEATH_FROM_THE_NUCLEUS	0.89	0.22	0	0	0	not significant
7715	SUZUKI_RESPONSE_TO_TSA_AND_DECITABINE_1A	0.89	0.22	0	0	0	not significant
7716	CERIBELLI_GENES_INACTIVE_AND_BOUND_BY_NFY	0.89	0.21	0	0	0	not significant
7717	GO_BASEMENT_MEMBRANE_ORGANIZATION	0.89	0.21	0	0	0	not significant
7718	GO_DNA_REPLICATION_SYNTHESIS_OF_RNA_PRIMER	0.89	0.21	0	0	0	not significant
7719	GO_MAST_CELL_MEDIATED_IMMUNITY	0.89	0.21	0	0	0	not significant
7720	GO_NEPHRON_EPITHELIUM_DEVELOPMENT	0.89	0.21	0	0	0	not significant
7721	GO_NEURON_PROJECTION_MAINTENANCE	0.89	0.21	0	0	0	not significant
7722	GO_PYRIMIDINE_DEOXYRIBONUCLEOTIDE_BIOSYNTHETIC_PROCESS	0.89	0.21	0	0	0	not significant
7723	GO_RECEPTOR_CATABOLIC_PROCESS	0.89	0.21	0	0	0	not significant
7724	GO_SUBSTRATE_DEPENDENT_CELL_MIGRATION	0.89	0.21	0	0	0	not significant
7725	GO_VITAMIN_B6_BINDING	0.89	0.21	0	0	0	not significant
7726	RIZ_ERYTHROID_DIFFERENTIATION_HBZ	0.89	0.21	0	0	0	not significant
7727	WATTEL_AUTONOMOUS_THYROID_ADENOMA_UP	0.89	0.21	0	0	0	not significant

7728	CEBALLOS_TARGETS_OF_TP53_AND_MYC_DN	0.89	0.2	0	0	0	not significant
7729	GO_ACROSOMAL_VESICLE	0.89	0.2	0	0	0	not significant
7730	GO_BODY_FLUID_SECRETION	0.89	0.2	0	0	0	not significant
7731	GO_CENTROSOME_DUPLICATION	0.89	0.2	0	0	0	not significant
7732	GO_ER_TO_GOLGI_TRANSPORT_VESICLE_MEMBRANE	0.89	0.2	0	0	0	not significant
7733	GO_MECHANORECEPTOR_DIFFERENTIATION	0.89	0.2	0	0	0	not significant
7734	GO_NEGATIVE_REGULATION_OF_ANTIGEN_RECEPTOR_MEDIATED_SIGNALING_PAT	0.89	0.2	0	0	0	not significant
7735	GO_POSITIVE_REGULATION_OF_DNA_CATABOLIC_PROCESS	0.89	0.2	0	0	0	not significant
7736	GO_REGULATION_OF_DNA_CATABOLIC_PROCESS	0.89	0.2	0	0	0	not significant
7737	GO_REGULATION_OF_TISSUE_REMODELING	0.89	0.2	0	0	0	not significant
7738	GO_SMAD_PROTEIN_COMPLEX	0.89	0.2	0	0	0	not significant
7739	GO_TETRAPYRROLE_BINDING	0.89	0.2	0	0	0	not significant
7740	GO_TETRAPYRROLE_BIOSYNTHETIC_PROCESS	0.89	0.2	0	0	0	not significant
7741	GO_TRAIL_ACTIVATED_APOPTOTIC_SIGNALING_PATHWAY	0.89	0.2	0	0	0	not significant
7742	KUROKAWA_LIVER_CANCER_EARLY_RECURRENCE_DN	0.89	0.2	0	0	0	not significant
7743	REACTOME_SIGNALING_BY_ERBB4	0.89	0.2	0	0	0	not significant
7744	TCGA_GLIOBLASTOMA_COPY_NUMBER_DN	0.89	0.2	0	0	0	not significant
7745	WESTON_VEGFA_TARGETS_12HR	0.89	0.2	0	0	0	not significant
7746	ZHAN_MULTIPLE_MYELOMA_MF_DN	0.89	0.2	0	0	0	not significant
7747	BIOCARTA_CIRCADIAN_PATHWAY	0.89	0.19	0	0	0	not significant
7748	GO_CARBOHYDRATE_TRANSMEMBRANE_TRANSPORT	0.89	0.19	0	0	0	not significant
7749	GO_FLAVIN_ADENINE_DINUCLEOTIDE_BINDING	0.89	0.19	0	0	0	not significant
7750	GO_GLUCOSIDASE_ACTIVITY	0.89	0.19	0	0	0	not significant
7751	GO_LEADING_EDGE_MEMBRANE	0.89	0.19	0	0	0	not significant
7752	GO_LEUKOCYTE_APOPTOTIC_PROCESS	0.89	0.19	0	0	0	not significant
7753	GO_NEGATIVE_REGULATION_OF_DEPHOSPHORYLATION	0.89	0.19	0	0	0	not significant
7754	GO_NEGATIVE_REGULATION_OF_PROTEIN_COMPLEX_DISASSEMBLY	0.89	0.19	0	0	0	not significant
7755	GO_POSITIVE_REGULATION_OF_NIK_NF_KAPPAB_SIGNALING	0.89	0.19	0	0	0	not significant
7756	REACTOME_LONG_TERM_POTENTIATION	0.89	0.19	0	0	0	not significant
7757	REACTOME_SIGNALING_BY_INSULIN_RECEPTOR	0.89	0.19	0	0	0	not significant
7758	REACTOME_TP53_REGULATES_TRANSCRIPTION_OF_DNA_REPAIR_GENES	0.89	0.19	0	0	0	not significant
7759	SMID_BREAST_CANCER_RELAPSE_IN_BRAIN_DN	0.89	0.19	0	0	0	not significant
7760	GO_ACTIN_CYTOSKELETON_REORGANIZATION	0.89	0.18	0	0	0	not significant
7761	GO_APOPTOTIC_MITOCHONDRIAL_CHANGES	0.89	0.18	0	0	0	not significant
7762	GO_EXTRINSIC_APOPTOTIC_SIGNALING_PATHWAY_VIA_DEATH_DOMAIN_RECEPTO	0.89	0.18	0	0	0	not significant
7763	GO_NEGATIVE_REGULATION_OF_ION_TRANSMEMBRANE_TRANSPORT	0.89	0.18	0	0	0	not significant
7764	DARWICHE_SQUAMOUS_CELL_CARCINOMA_DN	0.89	0.17	0	0	0	not significant
7765	GO_BONE_DEVELOPMENT	0.89	0.17	0	0	0	not significant
7766	GO_EMBRYONIC_ORGAN_MORPHOGENESIS	0.89	0.17	0	0	0	not significant
7767	GO_NUCLEASE_ACTIVITY	0.89	0.17	0	0	0	not significant
7768	GO_REGULATION_OF_CALCIUM_ION_TRANSPORT	0.89	0.17	0	0	0	not significant
7769	GO_TISSUE_REMODELING	0.89	0.17	0	0	0	not significant
7770	GO_POSITIVE_REGULATION_OF_NEURON_PROJECTION_DEVELOPMENT	0.89	0.16	0	0	0	not significant
7771	KYNG_DNA_DAMAGE_UP	0.89	0.16	0	0	0	not significant
7772	GO_REGULATION_OF_RESPONSE_TO_DNA_DAMAGE_STIMULUS	0.89	0.15	0	0	0	not significant
7773	GO_EMBRYONIC_ORGAN_DEVELOPMENT	0.89	0.14	0	0	0	not significant
7774	GO_IMMUNE_RESPONSE_REGULATING_CELL_SURFACE_RECEPTOR_SIGNALING_P/	0.89	0.14	0	0	0	not significant
7775	GO_PRESYNAPSE	0.89	0.14	0	0	0	not significant
7776	GO_CELL_CYCLE_G1_S_PHASE_TRANSITION	0.89	0.13	0	0	0	not significant
7777	GO_MYELOID_CELL_DIFFERENTIATION	0.89	0.13	0	0	0	not significant
7778	SWEET_LUNG_CANCER_KRAS_UP	0.89	0.12	0	0	0	not significant
7779	GO_REGULATION_OF_KINASE_ACTIVITY	0.89	0.1	0	0	0	not significant
7780	GO_NEGATIVE_REGULATION_OF_RNA_BIOSYNTHETIC_PROCESS	0.89	0.08	0	0	0	not significant
7781	GO_REGULATION_OF_CELLULAR_LOCALIZATION	0.89	0.08	0	0	0	not significant
7782	ACEVEDO_LIVER_CANCER_UP	0.89	0.07	0	0	0	not significant
7783	GO_IDENTICAL_PROTEIN_BINDING	0.89	0.06	0	0	0	not significant
7784	CHR13Q31	0.90	0.24	0	0	0	not significant
7785	GO_NEGATIVE_REGULATION_OF_MORPHOGENESIS_OF_AN_EPITHELIUM	0.90	0.24	0	0	0	not significant
7786	GO_POSITIVE_REGULATION_OF_CYTOPLASMIC_TRANSPORT	0.90	0.24	0	0	0	not significant
7787	GO_PROGESTERONE_RECEPTOR_SIGNALING_PATHWAY	0.90	0.24	0	0	0	not significant
7788	GO_TRANSMEMBRANE_RECEPTOR_PROTEIN_TYROSINE_KINASE_ADAPTOR_ACTIV	0.90	0.24	0	0	0	not significant
7789	PID_GLYPICAN_1PATHWAY	0.90	0.24	0	0	0	not significant
7790	CHR10P11	0.90	0.23	0	0	0	not significant
7791	DAVICIONI_PAX_FOXO1_SIGNATURE_IN_ARMS_DN	0.90	0.23	0	0	0	not significant
7792	GO_AROMATIC_AMINO_ACID_FAMILY_METABOLIC_PROCESS	0.90	0.23	0	0	0	not significant
7793	GO_ESTABLISHMENT_OF_PIGMENT_GRANULE_LOCALIZATION	0.90	0.23	0	0	0	not significant
7794	GO_NEGATIVE_REGULATION_OF_NECROTIC_CELL_DEATH	0.90	0.23	0	0	0	not significant
7795	GO_NEGATIVE_REGULATION_OF_TELOMERE_CAPPING	0.90	0.23	0	0	0	not significant
7796	GO_RAB_GUANYL_NUCLEOTIDE_EXCHANGE_FACTOR_ACTIVITY	0.90	0.23	0	0	0	not significant
7797	BANDRES_RESPONSE_TO_CARMUSTIN_MGMT_48HR_UP	0.90	0.22	0	0	0	not significant
7798	CHR5Q15	0.90	0.22	0	0	0	not significant
7799	CROMER_TUMORIGENESIS_DN	0.90	0.22	0	0	0	not significant
7800	DANG_MYC_TARGETS_DN	0.90	0.22	0	0	0	not significant
7801	GAUSSMANN_MLL_AF4_FUSION_TARGETS_A_DN	0.90	0.22	0	0	0	not significant
7802	GO_AMMONIUM_TRANSMEMBRANE_TRANSPORT	0.90	0.22	0	0	0	not significant
7803	GO_CELLULAR_BIOGENIC_AMINE_METABOLIC_PROCESS	0.90	0.22	0	0	0	not significant
7804	GO_DNA_METHYLATION_OR_DEMETHYLATION	0.90	0.22	0	0	0	not significant
7805	GO_ISOCITRATE_DEHYDROGENASE_ACTIVITY	0.90	0.22	0	0	0	not significant
7806	GO_MEMBRANE_REPOLARIZATION_DURING_ACTION_POTENTIAL	0.90	0.22	0	0	0	not significant
7807	GO_NEGATIVE_REGULATION_OF_LYMPHOCYTE_APOPTOTIC_PROCESS	0.90	0.22	0	0	0	not significant
7808	GO_NEGATIVE_REGULATION_OF_NEUROTRANSMITTER_TRANSPORT	0.90	0.22	0	0	0	not significant
7809	GO_PHOSPHOTRANSFERASE_ACTIVITY_PHOSPHATE_GROUP_AS_ACCEPTOR	0.90	0.22	0	0	0	not significant
7810	GO_POSITIVE_REGULATION_OF_FAT_CELL_DIFFERENTIATION	0.90	0.22	0	0	0	not significant
7811	GO_POSITIVE_REGULATION_OF_PROTEIN_EXIT_FROM_ENDOPLASMIC_RETICULUM	0.90	0.22	0	0	0	not significant
7812	GO_PROTEIN_AUTOUBIQUITINATION	0.90	0.22	0	0	0	not significant
7813	GO_PROTEIN_IMPORT_INTO_PEROXISOME_MATRIX	0.90	0.22	0	0	0	not significant
7814	GO_REGULATION_OF_BEHAVIOR	0.90	0.22	0	0	0	not significant
7815	GO_TRAPP_COMPLEX	0.90	0.22	0	0	0	not significant
7816	GO_UDP_GALACTOSYLTRANSFERASE_ACTIVITY	0.90	0.22	0	0	0	not significant
7817	GO_VIRAL_RNA_GENOME_REPLICATION	0.90	0.22	0	0	0	not significant
7818	HEIDENBLAD_AMPLICON_12P11_12_DN	0.90	0.22	0	0	0	not significant
7819	KIM_GLIS2_TARGETS_UP	0.90	0.22	0	0	0	not significant
7820	REACTOME_PRESYNAPTIC_DEPOLARIZATION_AND_CALCIUM_CHANNEL_OPENING	0.90	0.22	0	0	0	not significant
7821	REACTOME_RHO_GTPASES_ACTIVATE_CIT	0.90	0.22	0	0	0	not significant
7822	VETTER_TARGETS_OF_PRKCA_AND_ETS1_UP	0.90	0.22	0	0	0	not significant
7823	CHR14Q12	0.90	0.21	0	0	0	not significant
7824	DOANE_BREAST_CANCER_CLASSES_DN	0.90	0.21	0	0	0	not significant
7825	GO_CELL_DIVISION_SITE	0.90	0.21	0	0	0	not significant
7826	GO_ETHER_METABOLIC_PROCESS	0.90	0.21	0	0	0	not significant
7827	GO_GROWTH_FACTOR_RECEPTOR_BINDING	0.90	0.21	0	0	0	not significant
7828	GO_LIPID_GLYCOSYLATION	0.90	0.21	0	0	0	not significant
7829	GO_NEGATIVE_REGULATION_OF_ERK1_AND_ERK2_CASCADE	0.90	0.21	0	0	0	not significant
7830	GO_NEGATIVE_REGULATION_OF_UBIQUITIN_PROTEIN_LIGASE_ACTIVITY	0.90	0.21	0	0	0	not significant
7831	GO_NEURAL_CREST_CELL_MIGRATION	0.90	0.21	0	0	0	not significant
7832	GO_PERICENTRIOLAR_MATERIAL	0.90	0.21	0	0	0	not significant
7833	GO_POSITIVE_REGULATION_OF_SYNAPTIC_TRANSMISSION_GLUTAMATERGIC	0.90	0.21	0	0	0	not significant
7834	GO_POSTREPLICATION_REPAIR	0.90	0.21	0	0	0	not significant
7835	GO_REGULATION_OF_MITOCHONDRIAL_FISSION	0.90	0.21	0	0	0	not significant
7836	GO_REGULATION_OF_PROTEIN_DEACETYLATION	0.90	0.21	0	0	0	not significant

7837	GO_REPLICATION_FORK_PROTECTION_COMPLEX	0.90	0.21	0	0	0	not significant
7838	GO_TAT_PROTEIN_BINDING	0.90	0.21	0	0	0	not significant
7839	LANDIS_BREAST_CANCER_PROGRESSION_DN	0.90	0.21	0	0	0	not significant
7840	LASTOWSKA_COAMPLIFIED_WITH_MYCN	0.90	0.21	0	0	0	not significant
7841	LUI_THYROID_CANCER_PAX8_PPARG_DN	0.90	0.21	0	0	0	not significant
7842	MARIADASON_RESPONSE_TO_BUTYRATE_SULINDAC_6	0.90	0.21	0	0	0	not significant
7843	SCIBETTA_KDM5B_TARGETS_UP	0.90	0.21	0	0	0	not significant
7844	CAFFAREL_RESPONSE_TO_THC_24HR_5_DN	0.90	0.2	0	0	0	not significant
7845	GO_ASTROCYTE_CELL_MIGRATION	0.90	0.2	0	0	0	not significant
7846	GO_GLYCOSPHINGOLIPID_BIOSYNTHETIC_PROCESS	0.90	0.2	0	0	0	not significant
7847	GO_NEGATIVE_REGULATION_OF_PROTEIN_MODIFICATION_BY_SMALL_PROTEIN_C	0.90	0.2	0	0	0	not significant
7848	GO_NUCLEOBASE_CONTAINING_COMPOUND_TRANSMEMBRANE_TRANSPORTER_A	0.90	0.2	0	0	0	not significant
7849	GO_POSITIVE_REGULATION_OF_MEMBRANE_PERMEABILITY	0.90	0.2	0	0	0	not significant
7850	GO_POSITIVE_REGULATION_OF_RESPONSE_TO_WOUNDING	0.90	0.2	0	0	0	not significant
7851	KAUFFMANN_MELANOMA_RELAPSE_DN	0.90	0.2	0	0	0	not significant
7852	KEGG_ADIPOCYTOKINE_SIGNALING_PATHWAY	0.90	0.2	0	0	0	not significant
7853	KEGG_CHRONIC_MYELOID_LEUKEMIA	0.90	0.2	0	0	0	not significant
7854	KIM_WT1_TARGETS_12HR_UP	0.90	0.2	0	0	0	not significant
7855	MIKKELSEN_MEF_LCP_WITH_H3K4ME3	0.90	0.2	0	0	0	not significant
7856	VARELA_ZMPSTE24_TARGETS_DN	0.90	0.2	0	0	0	not significant
7857	GO_CHORION_DEVELOPMENT	0.90	0.19	0	0	0	not significant
7858	GO_GLUCAN_METABOLIC_PROCESS	0.90	0.19	0	0	0	not significant
7859	GO_POSITIVE_REGULATION_OF_AUTOPHAGY	0.90	0.19	0	0	0	not significant
7860	GO_POSITIVE_REGULATION_OF_TRANSMEMBRANE_TRANSPORT	0.90	0.19	0	0	0	not significant
7861	GO_REGULATION_OF_MYELOID_LEUKOCYTE_DIFFERENTIATION	0.90	0.19	0	0	0	not significant
7862	GO_REGULATION_OF_RELEASE_OF_CYTOCHROME_C_FROM_MITOCHONDRIA	0.90	0.19	0	0	0	not significant
7863	GO_RENAL_TUBULE_DEVELOPMENT	0.90	0.19	0	0	0	not significant
7864	GO_SITE_OF_POLARIZED_GROWTH	0.90	0.19	0	0	0	not significant
7865	GO_VITAMIN_METABOLIC_PROCESS	0.90	0.19	0	0	0	not significant
7866	KRAS.DF.V1_DN	0.90	0.19	0	0	0	not significant
7867	MIKKELSEN_MEF_HCP_WITH_H3K27ME3	0.90	0.19	0	0	0	not significant
7868	GO_NEGATIVE_REGULATION_OF_CELLULAR_AMIDE_METABOLIC_PROCESS	0.90	0.18	0	0	0	not significant
7869	PECE_MAMMARY_STEM_CELL_UP	0.90	0.18	0	0	0	not significant
7870	GO_RECEPTOR_METABOLIC_PROCESS	0.90	0.17	0	0	0	not significant
7871	GREGORY_SYNTHETIC_LETHAL_WITH_IMATINIB	0.90	0.17	0	0	0	not significant
7872	MARKEY_RB1_ACUTE_LOF_DN	0.90	0.17	0	0	0	not significant
7873	RAF_UP.V1_DN	0.90	0.17	0	0	0	not significant
7874	GO_JNK_CASCADE	0.90	0.16	0	0	0	not significant
7875	GO_REGULATION_OF_NEURON_PROJECTION_DEVELOPMENT	0.90	0.15	0	0	0	not significant
7876	GO_REGULATION_OF_GTPASE_ACTIVITY	0.90	0.14	0	0	0	not significant
7877	GO_CILIUM_ORGANIZATION	0.90	0.13	0	0	0	not significant
7878	GO_ACTIN_FILAMENT_BASED_PROCESS	0.90	0.11	0	0	0	not significant
7879	GO_NEURON_DEVELOPMENT	0.90	0.1	0	0	0	not significant
7880	GO_CELLULAR_COMPONENT_MORPHOGENESIS	0.90	0.09	0	0	0	not significant
7881	GO_RETINAL_GANGLION_CELL_AXON_GUIDANCE	0.91	0.25	0	0	0	not significant
7882	WONG_IFNA2_RESISTANCE_DN	0.91	0.25	0	0	0	not significant
7883	BIOCARTA_D4GDI_PATHWAY	0.91	0.24	0	0	0	not significant
7884	DACOSTA_UV_RESPONSE_VIA_ERCC3_XPCS_UP	0.91	0.24	0	0	0	not significant
7885	GO_ATRIAL_CARDIAC_MUSCLE_CELL_TO_AV_NODE_CELL_SIGNALING	0.91	0.24	0	0	0	not significant
7886	GO_CARDIAC_ATRIUM_DEVELOPMENT	0.91	0.24	0	0	0	not significant
7887	GO_CELL_DIFFERENTIATION_INVOLVED_IN_EMBRYONIC_PLACENTA_DEVELOPMEN	0.91	0.24	0	0	0	not significant
7888	GO_CLEAVAGE_FURROW	0.91	0.24	0	0	0	not significant
7889	GO_NEGATIVE_REGULATION_OF_INTERLEUKIN_12_PRODUCTION	0.91	0.24	0	0	0	not significant
7890	GO_POSTSYNAPTIC_DENSITY_INTRACELLULAR_COMPONENT	0.91	0.24	0	0	0	not significant
7891	GO_POSTSYNAPTIC_SPECIALIZATION_ORGANIZATION	0.91	0.24	0	0	0	not significant
7892	GO_RECEPTOR_LOCALIZATION_TO_SYNAPSE	0.91	0.24	0	0	0	not significant
7893	KYNG_WERNER_SYNDROM_UP	0.91	0.24	0	0	0	not significant
7894	OUELLET_OVARIAN_CANCER_INVASIVE_VS_LMP_DN	0.91	0.24	0	0	0	not significant
7895	REACTOME_CARBOXYTERMINAL_POST_TRANSLATIONAL_MODIFICATIONS_OF_TUE	0.91	0.24	0	0	0	not significant
7896	SASSON_RESPONSE_TO_FORSKOLIN_UP	0.91	0.24	0	0	0	not significant
7897	BIOCARTA_ACH_PATHWAY	0.91	0.23	0	0	0	not significant
7898	CASTELLANO_NRAS_TARGETS_DN	0.91	0.23	0	0	0	not significant
7899	CHRXQ24	0.91	0.23	0	0	0	not significant
7900	GO_ANGIOTENSIN_RECEPTOR_BINDING	0.91	0.23	0	0	0	not significant
7901	GO_CHONDROITIN_SULFATE_BIOSYNTHETIC_PROCESS	0.91	0.23	0	0	0	not significant
7902	GO_CONVERGENT_EXTENSION	0.91	0.23	0	0	0	not significant
7903	GO_ESCRT_COMPLEX	0.91	0.23	0	0	0	not significant
7904	GO_N_TERMINAL_PROTEIN_LIPIDATION	0.91	0.23	0	0	0	not significant
7905	GO_ORGAN_OR_TISSUE_SPECIFIC_IMMUNE_RESPONSE	0.91	0.23	0	0	0	not significant
7906	GO_POSITIVE_REGULATION_OF_MEMBRANE_PROTEIN_ECTODOMAIN_PROTEOLYS	0.91	0.23	0	0	0	not significant
7907	GO_REGULATION_OF_GLUCOSE_METABOLIC_PROCESS	0.91	0.23	0	0	0	not significant
7908	GO_REGULATION_OF_PROTEIN_DEPOLYMERIZATION	0.91	0.23	0	0	0	not significant
7909	GO_SERINE_TYPE_CARBOXYPEPTIDASE_ACTIVITY	0.91	0.23	0	0	0	not significant
7910	GO_SPECIFIC_GRANULE_LUMEN	0.91	0.23	0	0	0	not significant
7911	GO_TELOMERIC_D_LOOP_DISASSEMBLY	0.91	0.23	0	0	0	not significant
7912	KEGG_RENIN_ANGIOTENSIN_SYSTEM	0.91	0.23	0	0	0	not significant
7913	ONDER_CDH1_TARGETS_3_DN	0.91	0.23	0	0	0	not significant
7914	REACTOME_DISEASES_ASSOCIATED_WITH_GLYCOSYLATION_PRECURSOR_BIOSYI	0.91	0.23	0	0	0	not significant
7915	REACTOME_DNA_REPLICATION_INITIATION	0.91	0.23	0	0	0	not significant
7916	REACTOME_PI3K_AKT_ACTIVATION	0.91	0.23	0	0	0	not significant
7917	RUAN_RESPONSE_TO_TROGLITAZONE_DN	0.91	0.23	0	0	0	not significant
7918	TSENG_ADIPOGENIC_POTENTIAL_DN	0.91	0.23	0	0	0	not significant
7919	UEDA_CENTRAL_CLOCK	0.91	0.23	0	0	0	not significant
7920	ZHENG_RESPONSE_TO_ARSENITE_DN	0.91	0.23	0	0	0	not significant
7921	BOYAULT_LIVER_CANCER_SUBCLASS_G1_DN	0.91	0.22	0	0	0	not significant
7922	GARGALOVIC_RESPONSE_TO_OXIDIZED_PHOSPHOLIPIDS_TAN_DN	0.91	0.22	0	0	0	not significant
7923	GO_CARD_DOMAIN_BINDING	0.91	0.22	0	0	0	not significant
7924	GO_CERAMIDE_BIOSYNTHETIC_PROCESS	0.91	0.22	0	0	0	not significant
7925	GO_EXTRAEMBRYONIC_MEMBRANE_DEVELOPMENT	0.91	0.22	0	0	0	not significant
7926	GO_IRE1_MEDIATED_UNFOLDED_PROTEIN_RESPONSE	0.91	0.22	0	0	0	not significant
7927	GO_MEMBRANE_LIPID_CATABOLIC_PROCESS	0.91	0.22	0	0	0	not significant
7928	GO_NEGATIVE_REGULATION_OF_DNA_METABOLIC_PROCESS	0.91	0.22	0	0	0	not significant
7929	GO_NEURON_DEATH_IN_RESPONSE_TO_OXIDATIVE_STRESS	0.91	0.22	0	0	0	not significant
7930	GO_PHOSPHATIDYLINOSITOL_3_5_BISPHOSPHATE_BINDING	0.91	0.22	0	0	0	not significant
7931	GO_PHOSPHOLIPASE_INHIBITOR_ACTIVITY	0.91	0.22	0	0	0	not significant
7932	GO_REGULATION_OF_CELLULAR_CARBOHYDRATE_METABOLIC_PROCESS	0.91	0.22	0	0	0	not significant
7933	GO_REGULATION_OF_INTERLEUKIN_2_SECRETION	0.91	0.22	0	0	0	not significant
7934	GO_REGULATION_OF_PROTEIN_CATABOLIC_PROCESS_IN_THE_VACUOLE	0.91	0.22	0	0	0	not significant
7935	GUTIERREZ_WALDENSTROEMS_MACROGLOBULINEMIA_1_UP	0.91	0.22	0	0	0	not significant
7936	HAHTOLA_SEZARY_SYNDROM_UP	0.91	0.22	0	0	0	not significant
7937	KAYO_CALORIE_RESTRICTION_MUSCLE_UP	0.91	0.22	0	0	0	not significant
7938	KIM_GERMINAL_CENTER_T_HELPER_UP	0.91	0.22	0	0	0	not significant
7939	MARTINEZ_RESPONSE_TO_TRABECTEDIN_UP	0.91	0.22	0	0	0	not significant
7940	MIKKELSEN_MEF_ICP_WITH_H3K27ME3	0.91	0.22	0	0	0	not significant
7941	PARK_APL_PATHOGENESIS_DN	0.91	0.22	0	0	0	not significant
7942	REACTOME_NEGATIVE_REGULATORS_OF_DDX58_IFIH1_SIGNALING	0.91	0.22	0	0	0	not significant
7943	SCHEIDEREIT_IKK_INTERACTING_PROTEINS	0.91	0.22	0	0	0	not significant
7944	BIOCARTA_EICOSANOID_PATHWAY	0.91	0.21	0	0	0	not significant
7945	COATES_MACROPHAGE_M1_VS_M2_DN	0.91	0.21	0	0	0	not significant

7946	GO_AP_1_ADAPTOR_COMPLEX_BINDING	0.91	0.21	0	0	0	not significant
7947	GO_MORPHOGENESIS_OF_A_BRANCHING_STRUCTURE	0.91	0.21	0	0	0	not significant
7948	GO_SYNAPTIC_VESICLE_RECYCLING	0.91	0.21	0	0	0	not significant
7949	HOLLERN_MICROACINAR_BREAST_TUMOR_DN	0.91	0.21	0	0	0	not significant
7950	HUI_MAPK14_TARGETS_UP	0.91	0.21	0	0	0	not significant
7951	PID_ERBB1_DOWNSTREAM_PATHWAY	0.91	0.21	0	0	0	not significant
7952	GO_GLIAL_CELL_DIFFERENTIATION	0.91	0.19	0	0	0	not significant
7953	GO_GOLGI_ORGANIZATION	0.91	0.19	0	0	0	not significant
7954	GO_HORMONE_RECEPTOR_BINDING	0.91	0.19	0	0	0	not significant
7955	GO_REGULATION_OF_CYSTEINE_TYPE_ENDOPEPTIDASE_ACTIVITY	0.91	0.19	0	0	0	not significant
7956	HAHTOLA_MYCOSIS_FUNGOIDES_SKIN_UP	0.91	0.19	0	0	0	not significant
7957	REACTOME_RHO_GTPASE_CYCLE	0.91	0.19	0	0	0	not significant
7958	RODWELL_AGING_KIDNEY_DN	0.91	0.19	0	0	0	not significant
7959	GO_LIPID_MODIFICATION	0.91	0.18	0	0	0	not significant
7960	KEGG_ENDOCYTOSIS	0.91	0.18	0	0	0	not significant
7961	QI_PLASMACYTOMA_UP	0.91	0.18	0	0	0	not significant
7962	GO_NUCLEOSIDE_PHOSPHATE_METABOLIC_PROCESS	0.91	0.17	0	0	0	not significant
7963	AGUIRRE_PANCREATIC_CANCER_COPY_NUMBER_UP	0.91	0.16	0	0	0	not significant
7964	GO_DENDRITIC_TREE	0.91	0.16	0	0	0	not significant
7965	GO_REGULATION_OF_AUTOPHAGY	0.91	0.16	0	0	0	not significant
7966	GO_REGULATION_OF_BINDING	0.91	0.16	0	0	0	not significant
7967	GO_REGULATION_OF_INTRACELLULAR_PROTEIN_TRANSPORT	0.91	0.16	0	0	0	not significant
7968	KOYAMA_SEMA3B_TARGETS_DN	0.91	0.16	0	0	0	not significant
7969	LOPEZ_MBD_TARGETS	0.91	0.12	0	0	0	not significant
7970	GO_CELL_PROJECTION_ORGANIZATION	0.91	0.1	0	0	0	not significant
7971	GO_NEGATIVE_REGULATION_OF_MOLECULAR_FUNCTION	0.91	0.1	0	0	0	not significant
7972	BONOME_OVARIAN_CANCER_POOR_SURVIVAL_UP	0.92	0.26	0	0	0	not significant
7973	GO_REGULATION_OF_PROTEIN_TARGETING_TO_MEMBRANE	0.92	0.26	0	0	0	not significant
7974	REACTOME_ZBP1_DAI_MEDIATED_INDUCTION_OF_TYPE_I_IFNS	0.92	0.26	0	0	0	not significant
7975	STARK_HYPPOCAMPUS_22Q11_DELETION_DN	0.92	0.26	0	0	0	not significant
7976	GO_ACYL_COA_DEHYDROGENASE_ACTIVITY	0.92	0.25	0	0	0	not significant
7977	GO_HIGH_DENSITY_LIPOPROTEIN_PARTICLE_ASSEMBLY	0.92	0.25	0	0	0	not significant
7978	GO_MAST_CELL_ACTIVATION	0.92	0.25	0	0	0	not significant
7979	GO_NEGATIVE_REGULATION_OF_CD4_POSITIVE_ALPHA_BETA_T_CELL_ACTIVATIC	0.92	0.25	0	0	0	not significant
7980	GO_NUCLEOTIDE_SUGAR_TRANSMEMBRANE_TRANSPORTER_ACTIVITY	0.92	0.25	0	0	0	not significant
7981	GO_OLIGOSACCHARIDE_BINDING	0.92	0.25	0	0	0	not significant
7982	GO_POLY_A_SPECIFIC_RIBONUCLEASE_ACTIVITY	0.92	0.25	0	0	0	not significant
7983	MOOTHA_FFA_OXYDATION	0.92	0.25	0	0	0	not significant
7984	NICK_RESPONSE_TO_PROC_TREATMENT_DN	0.92	0.25	0	0	0	not significant
7985	NOUSHMEHR_GBM_SILENCED_BY_METHYLATION	0.92	0.25	0	0	0	not significant
7986	STREICHER_LSM1_TARGETS_DN	0.92	0.25	0	0	0	not significant
7987	VALK_AML_CLUSTER_9	0.92	0.25	0	0	0	not significant
7988	CHR10Q25	0.92	0.24	0	0	0	not significant
7989	CHR8Q11	0.92	0.24	0	0	0	not significant
7990	GO_CARDIAC_MUSCLE_CELL_PROLIFERATION	0.92	0.24	0	0	0	not significant
7991	GO_CELLULAR_RESPONSE_TO_GAMMA_RADIATION	0.92	0.24	0	0	0	not significant
7992	GO_INNER_EAR_RECEPTOR_CELL_DEVELOPMENT	0.92	0.24	0	0	0	not significant
7993	GO_LAMELLAR_BODY	0.92	0.24	0	0	0	not significant
7994	GO_NEGATIVE_REGULATION_OF_ERAD_PATHWAY	0.92	0.24	0	0	0	not significant
7995	GO_NEGATIVE_REGULATION_OF_TRANSLATION_IN_RESPONSE_TO_STRESS	0.92	0.24	0	0	0	not significant
7996	GO_NEGATIVE_REGULATION_OF_TYPE_2_IMMUNE_RESPONSE	0.92	0.24	0	0	0	not significant
7997	GO_NEPHRON_MORPHOGENESIS	0.92	0.24	0	0	0	not significant
7998	GO_PORE_COMPLEX_ASSEMBLY	0.92	0.24	0	0	0	not significant
7999	GO_POSITIVE_REGULATION_OF_ANTIGEN_RECEPTOR_MEDIATED_SIGNALING_PAT	0.92	0.24	0	0	0	not significant
8000	GO_POSITIVE_REGULATION_OF_POTASSIUM_ION_TRANSPORT	0.92	0.24	0	0	0	not significant
8001	GO_POSITIVE_REGULATION_OF_PROTEIN_MATURATION	0.92	0.24	0	0	0	not significant
8002	GO_POSITIVE_REGULATION_OF_TRANSCRIPTION_FROM_RNA_POLYMERASE_II_PR	0.92	0.24	0	0	0	not significant
8003	GO_PURINERGIC_RECEPTOR_ACTIVITY	0.92	0.24	0	0	0	not significant
8004	HASLINGER_B_CLL_WITH_MUTATED_VH_GENES	0.92	0.24	0	0	0	not significant
8005	IRITANI_MAD1_TARGETS_DN	0.92	0.24	0	0	0	not significant
8006	NGUYEN_NOTCH1_TARGETS_UP	0.92	0.24	0	0	0	not significant
8007	REACTOME_CLASS_I_PEROXISOMAL_MEMBRANE_PROTEIN_IMPORT	0.92	0.24	0	0	0	not significant
8008	REACTOME_NEF_MEDIATES_DOWN_MODULATION_OF_CELL_SURFACE_RECEPTOI	0.92	0.24	0	0	0	not significant
8009	REACTOME_RIP_MEDIATED_NFKB_ACTIVATION_VIA_ZBP1	0.92	0.24	0	0	0	not significant
8010	WIKMAN_ASBESTOS_LUNG_CANCER_DN	0.92	0.24	0	0	0	not significant
8011	ALCALAY_AML_BY_NPM1_LOCALIZATION_UP	0.92	0.23	0	0	0	not significant
8012	GO_BRANCHING_MORPHOGENESIS_OF_AN_EPITHELIAL_TUBE	0.92	0.23	0	0	0	not significant
8013	GO_DYSTROGLYCAN_BINDING	0.92	0.23	0	0	0	not significant
8014	GO_LEUKOCYTE_AGGREGATION	0.92	0.23	0	0	0	not significant
8015	GO_LYMPHOCYTE_APOPTOTIC_PROCESS	0.92	0.23	0	0	0	not significant
8016	GO_NECROTIC_CELL_DEATH	0.92	0.23	0	0	0	not significant
8017	GO_NEGATIVE_REGULATION_OF_EPIDERMIS_DEVELOPMENT	0.92	0.23	0	0	0	not significant
8018	GO_NEGATIVE_REGULATION_OF_HEART_RATE	0.92	0.23	0	0	0	not significant
8019	GO_NEGATIVE_REGULATION_OF_PROTEIN_IMPORT_INTO_NUCLEUS	0.92	0.23	0	0	0	not significant
8020	GO_NEGATIVE_REGULATION_OF_STRESS_ACTIVATED_PROTEIN_KINASE_SIGNALIN	0.92	0.23	0	0	0	not significant
8021	GO_ORGANELLE_MEMBRANE_FUSION	0.92	0.23	0	0	0	not significant
8022	GO_PHOSPHATIDYLINOSITOL_3_PHOSPHATE_BINDING	0.92	0.23	0	0	0	not significant
8023	GO_POSITIVE_REGULATION_OF_DEACETYLASE_ACTIVITY	0.92	0.23	0	0	0	not significant
8024	GO_POSITIVE_REGULATION_OF_DNA_REPAIR	0.92	0.23	0	0	0	not significant
8025	GO_PROTEIN_LOCALIZATION_TO_VACUOLE	0.92	0.23	0	0	0	not significant
8026	GO_RNA_PHOSPHODIESTER_BOND_HYDROLYSIS_EXONUCLEOLYTIC	0.92	0.23	0	0	0	not significant
8027	GO_VASCULAR_ENDOTHELIAL_GROWTH_FACTOR_RECEPTOR_SIGNALING_PATHW	0.92	0.23	0	0	0	not significant
8028	GO_WNT_PROTEIN_BINDING	0.92	0.23	0	0	0	not significant
8029	HEDVAT_ELF4_TARGETS_UP	0.92	0.23	0	0	0	not significant
8030	HOOI_ST7_TARGETS_UP	0.92	0.23	0	0	0	not significant
8031	MAYBURD_RESPONSE_TO_L663536_DN	0.92	0.23	0	0	0	not significant
8032	REACTOME_RAB_GERANYLGERANYLATION	0.92	0.23	0	0	0	not significant
8033	WARTERS_IR_RESPONSE_5GY	0.92	0.23	0	0	0	not significant
8034	GO_CELL_CELL_ADHERENS_JUNCTION	0.92	0.22	0	0	0	not significant
8035	GO_DEVELOPMENT_OF_PRIMARY_FEMALE_SEXUAL_CHARACTERISTICS	0.92	0.22	0	0	0	not significant
8036	GO_LIPASE_INHIBITOR_ACTIVITY	0.92	0.22	0	0	0	not significant
8037	GO_MEMBRANE_BIOGENESIS	0.92	0.22	0	0	0	not significant
8038	GO_MYELOID_CELL_HOMEOSTASIS	0.92	0.22	0	0	0	not significant
8039	GO_POSITIVE_REGULATION_OF_CHROMATIN_ORGANIZATION	0.92	0.22	0	0	0	not significant
8040	GO_REGULATION_OF_PROTEIN_LOCALIZATION_TO_CELL_SURFACE	0.92	0.22	0	0	0	not significant
8041	GO_SARCOLEMMA	0.92	0.22	0	0	0	not significant
8042	CHR20Q13	0.92	0.21	0	0	0	not significant
8043	GO_CELL_JUNCTION_ASSEMBLY	0.92	0.21	0	0	0	not significant
8044	GO_CONNECTIVE_TISSUE_DEVELOPMENT	0.92	0.21	0	0	0	not significant
8045	GO_POSITIVE_REGULATION_OF_CELL_MORPHOGENESIS_INVOLVED_IN_DIFFERENT	0.92	0.21	0	0	0	not significant
8046	REACTOME_G_ALPHA_I_SIGNALLING_EVENTS	0.92	0.21	0	0	0	not significant
8047	GO_NEURON_DEATH	0.92	0.2	0	0	0	not significant
8048	GO_REGULATION_OF_HORMONE_SECRETION	0.92	0.2	0	0	0	not significant
8049	GO_VIRAL_GENOME_REPLICATION	0.92	0.2	0	0	0	not significant
8050	GO_HOMEOSTASIS_OF_NUMBER_OF_CELLS	0.92	0.19	0	0	0	not significant
8051	MARTINEZ_RB1_AND_TP53_TARGETS_DN	0.92	0.16	0	0	0	not significant
8052	GO_GROWTH	0.92	0.14	0	0	0	not significant
8053	GO_NEGATIVE_REGULATION_OF_CATALYTIC_ACTIVITY	0.92	0.14	0	0	0	not significant
8054	GRAESSMANN_APOPTOSIS_BY_DOXORUBICIN_DN	0.92	0.08	0	0	0	not significant

8055	GO_GLUTAMATE_METABOLIC_PROCESS	0.93	0.27	0	0	0	not significant
8056	GO_NEGATIVE_REGULATION_OF_OSTEOBLAST_DIFFERENTIATION	0.93	0.27	0	0	0	not significant
8057	GO_REGULATION_OF_SPHINGOLIPID_BIOSYNTHETIC_PROCESS	0.93	0.27	0	0	0	not significant
8058	GO_RESPONSE_TO_HYDROPEROXIDE	0.93	0.27	0	0	0	not significant
8059	GO_T_CELL_APOPTOTIC_PROCESS	0.93	0.27	0	0	0	not significant
8060	PID_WNT_SIGNALING_PATHWAY	0.93	0.27	0	0	0	not significant
8061	REACTOME_LDL_CLEARANCE	0.93	0.27	0	0	0	not significant
8062	BOGNI_TREATMENT_RELATED_MYELOID_LEUKEMIA_UP	0.93	0.26	0	0	0	not significant
8063	CAFFAREL_RESPONSE_TO_THC_DN	0.93	0.26	0	0	0	not significant
8064	CHR12Q22	0.93	0.26	0	0	0	not significant
8065	GO_ALPHA_AMINO_ACID_CATABOLIC_PROCESS	0.93	0.26	0	0	0	not significant
8066	GO_POSITIVE_REGULATION_OF_EARLY_ENDOSOME_TO_LATE_ENDOSOME_TRAN	0.93	0.26	0	0	0	not significant
8067	GO_RESPONSE_TO_FUNGUS	0.93	0.26	0	0	0	not significant
8068	HATADA_METHYLATED_IN_LUNG_CANCER_DN	0.93	0.26	0	0	0	not significant
8069	REACTOME_NCAM1_INTERACTIONS	0.93	0.26	0	0	0	not significant
8070	REACTOME_NONSENSE_MEDIATED_DECAY_NMD_INDEPENDENT_OF_THE_EXON_JU	0.93	0.26	0	0	0	not significant
8071	REACTOME_SIGNALING_BY_NOTCH1	0.93	0.26	0	0	0	not significant
8072	BIOCARTA_NFKB_PATHWAY	0.93	0.25	0	0	0	not significant
8073	BIOCARTA_SPRY_PATHWAY	0.93	0.25	0	0	0	not significant
8074	FIGUEROA_AML_METHYLATION_CLUSTER_1_DN	0.93	0.25	0	0	0	not significant
8075	GO_CALCIUM_DEPENDENT_PROTEIN_BINDING	0.93	0.25	0	0	0	not significant
8076	GO_EXORIBONUCLEASE_ACTIVITY	0.93	0.25	0	0	0	not significant
8077	GO_LYMPHANGIOGENESIS	0.93	0.25	0	0	0	not significant
8078	GO_MYOTUBE_CELL_DEVELOPMENT	0.93	0.25	0	0	0	not significant
8079	GO_NEGATIVE_REGULATION_OF_DNA_BINDING	0.93	0.25	0	0	0	not significant
8080	GO_NEGATIVE_REGULATION_OF_ENDOCYTOSIS	0.93	0.25	0	0	0	not significant
8081	GO_NEGATIVE_REGULATION_OF_MUSCLE_CONTRACTION	0.93	0.25	0	0	0	not significant
8082	GO_NEGATIVE_REGULATION_OF_PROGRAMMED_NECROTIC_CELL_DEATH	0.93	0.25	0	0	0	not significant
8083	GO_OVARIAN_FOLLICLE_DEVELOPMENT	0.93	0.25	0	0	0	not significant
8084	GO_PEPTIDYL_THREONINE_DEPHOSPHORYLATION	0.93	0.25	0	0	0	not significant
8085	GO_PHOSPHATIDYLINOSITOL_3_4_BISPHOSPHATE_BINDING	0.93	0.25	0	0	0	not significant
8086	GO_PLASMA_MEMBRANE_TUBULATION	0.93	0.25	0	0	0	not significant
8087	GO_POSITIVE_REGULATION_OF_ACUTE_INFLAMMATORY_RESPONSE	0.93	0.25	0	0	0	not significant
8088	GO_POSITIVE_REGULATION_OF_LEUKOCYTE_CHEMOTAXIS	0.93	0.25	0	0	0	not significant
8089	GO_REGULATION_OF_FATTY_ACID_METABOLIC_PROCESS	0.93	0.25	0	0	0	not significant
8090	GO_REGULATION_OF_LYMPHOCYTE_CHEMOTAXIS	0.93	0.25	0	0	0	not significant
8091	KEGG_SULFUR_METABOLISM	0.93	0.25	0	0	0	not significant
8092	LIU_TOPBP1_TARGETS	0.93	0.25	0	0	0	not significant
8093	REACTOME_ACTIVATED_TAK1_MEDIATES_P38_MAPK_ACTIVATION	0.93	0.25	0	0	0	not significant
8094	REACTOME_LYSINE_CATABOLISM	0.93	0.25	0	0	0	not significant
8095	REACTOME_PI3K_EVENTS_IN_ERBB2_SIGNALING	0.93	0.25	0	0	0	not significant
8096	REACTOME_TP53_REGULATES_TRANSCRIPTION_OF_CELL_DEATH_GENES	0.93	0.25	0	0	0	not significant
8097	BOQUEST_STEM_CELL_CULTURED_VS_FRESH_DN	0.93	0.24	0	0	0	not significant
8098	GARGALOVIC_RESPONSE_TO_OXIDIZED_PHOSPHOLIPIDS_GREY_DN	0.93	0.24	0	0	0	not significant
8099	GO_KIDNEY_EPITHELIUM_DEVELOPMENT	0.93	0.24	0	0	0	not significant
8100	GO_LYMPHOCYTE_HOMEOSTASIS	0.93	0.24	0	0	0	not significant
8101	GO_MICROTUBULE_NUCLEATION_BY_INTERPHASE_MICROTUBULE_ORGANIZING_CE	0.93	0.24	0	0	0	not significant
8102	GO_MUSCLE_ALPHA_ACTININ_BINDING	0.93	0.24	0	0	0	not significant
8103	GO_ORGANOPHOSPHATE_CATABOLIC_PROCESS	0.93	0.24	0	0	0	not significant
8104	GO_PLACENTA_DEVELOPMENT	0.93	0.24	0	0	0	not significant
8105	GO_POLYUBIQUITIN_MODIFICATION_DEPENDENT_PROTEIN_BINDING	0.93	0.24	0	0	0	not significant
8106	GO_POSITIVE_REGULATION_OF_DENDRITE_MORPHOGENESIS	0.93	0.24	0	0	0	not significant
8107	GO_REGULATION_OF_AUTOPHAGY_OF_MITOCHONDRION	0.93	0.24	0	0	0	not significant
8108	GO_REGULATION_OF_SKELETAL_MUSCLE_TISSUE_DEVELOPMENT	0.93	0.24	0	0	0	not significant
8109	GO_SIGNAL_TRANSDUCTION_INVOLVED_IN_REGULATION_OF_GENE_EXPRESSION	0.93	0.24	0	0	0	not significant
8110	GO_TRANSLATION_ELONGATION_FACTOR_ACTIVITY	0.93	0.24	0	0	0	not significant
8111	KEGG_ONE_CARBON_POOL_BY_FOLATE	0.93	0.24	0	0	0	not significant
8112	REACTOME_METABOLISM_OF_WATER_SOLUBLE_VITAMINS_AND_COFACTORS	0.93	0.24	0	0	0	not significant
8113	SEMBA_FHIT_TARGETS_UP	0.93	0.24	0	0	0	not significant
8114	DASU_IL6_SIGNALING_UP	0.93	0.23	0	0	0	not significant
8115	GO_GLYCOLIPID_TRANSPORT	0.93	0.23	0	0	0	not significant
8116	GO_HYDROXYMETHYL__FORMYL_AND_RELATED_TRANSFERASE_ACTIVITY	0.93	0.23	0	0	0	not significant
8117	GO_MYELOID_LEUKOCYTE_DIFFERENTIATION	0.93	0.23	0	0	0	not significant
8118	GO_REGULATION_OF_MEMBRANE_PERMEABILITY	0.93	0.23	0	0	0	not significant
8119	GO_SEMI_LUNAR_VALVE_DEVELOPMENT	0.93	0.23	0	0	0	not significant
8120	GO_SMOOTH_MUSCLE_CONTRACTION	0.93	0.23	0	0	0	not significant
8121	GO_SMOOTHENED_SIGNALING_PATHWAY	0.93	0.23	0	0	0	not significant
8122	GO_SYNAPTIC_VESICLE_CYCLE	0.93	0.23	0	0	0	not significant
8123	GO_TRANSFERASE_ACTIVITY_TRANSFERRING_ALKYL_OR_ARYL_OTHER_THAN_ME	0.93	0.23	0	0	0	not significant
8124	ONDER_CDH1_TARGETS_2_UP	0.93	0.23	0	0	0	not significant
8125	REACTOME_PYRIMIDINE_SALVAGE	0.93	0.23	0	0	0	not significant
8126	YAN_ESCAPE_FROM_ANOIKIS	0.93	0.23	0	0	0	not significant
8127	BOQUEST_STEM_CELL_UP	0.93	0.22	0	0	0	not significant
8128	GO_CYTOSOLIC_SMALL_RIBOSOMAL_SUBUNIT	0.93	0.22	0	0	0	not significant
8129	GO_EPSILON_DNA_POLYMERASE_COMPLEX	0.93	0.22	0	0	0	not significant
8130	GO_GERM_CELL_DEVELOPMENT	0.93	0.22	0	0	0	not significant
8131	GO_POSITIVE_REGULATION_OF_DNA_BINDING_TRANSCRIPTION_FACTOR_ACTIVITY	0.93	0.22	0	0	0	not significant
8132	GO_REGULATION_OF_TRANSPORTER_ACTIVITY	0.93	0.22	0	0	0	not significant
8133	GO_SENSORY_ORGAN_MORPHOGENESIS	0.93	0.22	0	0	0	not significant
8134	GO_SIGNAL_RELEASE_FROM_SYNAPSE	0.93	0.22	0	0	0	not significant
8135	HALLMARK_FATTY_ACID_METABOLISM	0.93	0.22	0	0	0	not significant
8136	MARTORIATI_MDM4_TARGETS_FETAL_LIVER_UP	0.93	0.22	0	0	0	not significant
8137	AGUIRRE_PANCREATIC_CANCER_COPY_NUMBER_DN	0.93	0.21	0	0	0	not significant
8138	DARWICHE_PAPILLOMA_RISK_HIGH_DN	0.93	0.21	0	0	0	not significant
8139	GO_COENZYME_METABOLIC_PROCESS	0.93	0.21	0	0	0	not significant
8140	GO_VESICLE_LUMEN	0.93	0.21	0	0	0	not significant
8141	STARK_PREFRONTAL_CORTEX_22Q11_DELETION_UP	0.93	0.21	0	0	0	not significant
8142	GO_CELL_CELL_JUNCTION	0.93	0.2	0	0	0	not significant
8143	GO_DEVELOPMENT_OF_PRIMARY_SEXUAL_CHARACTERISTICS	0.93	0.2	0	0	0	not significant
8144	REACTOME_INTERFERON_SIGNALING	0.93	0.2	0	0	0	not significant
8145	GO_NEGATIVE_REGULATION_OF_INTRACELLULAR_SIGNAL_TRANSDUCTION	0.93	0.17	0	0	0	not significant
8146	GO_ORGANOPHOSPHATE_BIOSYNTHETIC_PROCESS	0.93	0.17	0	0	0	not significant
8147	GO_ACTIVATION_OF_IMMUNE_RESPONSE	0.93	0.16	0	0	0	not significant
8148	WAKABAYASHI_ADIPOGENESIS_PPARG_BOUND_8D	0.93	0.16	0	0	0	not significant
8149	GO_FATTY_ACYL_COA_BINDING	0.94	0.28	0	0	0	not significant
8150	GO_FOUR_WAY_JUNCTION_DNA_BINDING	0.94	0.28	0	0	0	not significant
8151	GO_MICROVILLUS_MEMBRANE	0.94	0.28	0	0	0	not significant
8152	GO_NEGATIVE_REGULATION_OF_B_CELL_ACTIVATION	0.94	0.28	0	0	0	not significant
8153	GO_POSITIVE_REGULATION_OF_PROTEIN_DEACETYLATION	0.94	0.28	0	0	0	not significant
8154	GO_TRANSFORMING_GROWTH_FACTOR_BETA_ACTIVATION	0.94	0.28	0	0	0	not significant
8155	BIOCARTA_AHSP_PATHWAY	0.94	0.27	0	0	0	not significant
8156	GO_CILIARY_TRANSITION_ZONE	0.94	0.27	0	0	0	not significant
8157	GO_FATTY_ACID_HOMEOSTASIS	0.94	0.27	0	0	0	not significant
8158	GO_GALACTOSE_CATABOLIC_PROCESS	0.94	0.27	0	0	0	not significant
8159	GO_HOMOLOGOUS_CHROMOSOME_SEGREGATION	0.94	0.27	0	0	0	not significant
8160	GO_MYOSIN_COMPLEX	0.94	0.27	0	0	0	not significant
8161	GO_NEGATIVE_REGULATION_OF_CELL_CYCLE_G1_S_PHASE_TRANSITION	0.94	0.27	0	0	0	not significant
8162	GO_NEGATIVE_REGULATION_OF_OXIDATIVE_STRESS_INDUCED_NEURON_DEATH	0.94	0.27	0	0	0	not significant
8163	GO_NERVE_DEVELOPMENT	0.94	0.27	0	0	0	not significant

8164	GO_POSITIVE_REGULATION_OF_ESTABLISHMENT_OF_PROTEIN_LOCALIZATION_TC	0.94	0.27	0	0	0	not significant
8165	GO_POSITIVE_REGULATION_OF_LIPID_STORAGE	0.94	0.27	0	0	0	not significant
8166	GO_POSITIVE_REGULATION_OF_P38MAPK_CASCADE	0.94	0.27	0	0	0	not significant
8167	GO_POSITIVE_REGULATION_OF_T_HELPER_CELL_DIFFERENTIATION	0.94	0.27	0	0	0	not significant
8168	GO_PROTEIN_PHOSPHATASE_2A_BINDING	0.94	0.27	0	0	0	not significant
8169	GRAESSMANN_RESPONSE_TO_MC_AND_SERUM_DEPRIVATION_DN	0.94	0.27	0	0	0	not significant
8170	PID_WNT_NONCANONICAL_PATHWAY	0.94	0.27	0	0	0	not significant
8171	REACTOME_NETRIN_1_SIGNALING	0.94	0.27	0	0	0	not significant
8172	BILANGES_SERUM_SENSITIVE_VIA_TSC1	0.94	0.26	0	0	0	not significant
8173	GO_CUL4B_RING_E3_UBIQUITIN_LIGASE_COMPLEX	0.94	0.26	0	0	0	not significant
8174	GO_HISTONE_DEUBIQUITINATION	0.94	0.26	0	0	0	not significant
8175	GO_INTERCELLULAR_BRIDGE	0.94	0.26	0	0	0	not significant
8176	GO_INVADOPODIUM	0.94	0.26	0	0	0	not significant
8177	GO_LYMPHOCYTE_COSTIMULATION	0.94	0.26	0	0	0	not significant
8178	GO_MESONEPHRIC_TUBULE_MORPHOGENESIS	0.94	0.26	0	0	0	not significant
8179	GO_METHYLATED_HISTONE_BINDING	0.94	0.26	0	0	0	not significant
8180	GO_MITOTIC_RECOMBINATION	0.94	0.26	0	0	0	not significant
8181	GO_NEGATIVE_REGULATION_OF_TOLL_LIKE_RECEPTOR_SIGNALING_PATHWAY	0.94	0.26	0	0	0	not significant
8182	GO_PARTURITION	0.94	0.26	0	0	0	not significant
8183	GO_POSITIVE_REGULATION_OF_PROTEIN_TARGETING_TO_MITOCHONDRION	0.94	0.26	0	0	0	not significant
8184	GO_REGULATION_OF_CALCIUM_ION_TRANSMEMBRANE_TRANSPORT	0.94	0.26	0	0	0	not significant
8185	GO_REGULATION_OF_HEART_RATE_BY_CARDIAC_CONDUCTION	0.94	0.26	0	0	0	not significant
8186	GO_REGULATION_OF_MITOCHONDRIAL_MEMBRANE_PERMEABILITY_INVOLVED_IN_	0.94	0.26	0	0	0	not significant
8187	GO_REGULATION_OF_PROTEIN_SUMOYLATION	0.94	0.26	0	0	0	not significant
8188	GO_REGULATION_OF_WOUND_HEALING	0.94	0.26	0	0	0	not significant
8189	GO_SMOOTH_MUSCLE_CELL_DIFFERENTIATION	0.94	0.26	0	0	0	not significant
8190	JACKSON_DNMT1_TARGETS_UP	0.94	0.26	0	0	0	not significant
8191	KAMMINGA_SENESCENCE	0.94	0.26	0	0	0	not significant
8192	KYNG_ENVIRONMENTAL_STRESS_RESPONSE_NOT_BY_GAMMA_IN_WS	0.94	0.26	0	0	0	not significant
8193	LEE_LIVER_CANCER_MYC_UP	0.94	0.26	0	0	0	not significant
8194	MEISSNER_BRAIN_HCP_WITH_H3_UNMETHYLATED	0.94	0.26	0	0	0	not significant
8195	NOUZOVA_METHYLATED_IN_APL	0.94	0.26	0	0	0	not significant
8196	PID_INTEGRIN_A4B1_PATHWAY	0.94	0.26	0	0	0	not significant
8197	PID_INTEGRIN5_PATHWAY	0.94	0.26	0	0	0	not significant
8198	REACTOME_GABA_SYNTHESIS_RELEASE_REUPTAKE_AND_DEGRADATION	0.94	0.26	0	0	0	not significant
8199	RODRIGUES_THYROID_CARCINOMA_UP	0.94	0.26	0	0	0	not significant
8200	YAP1_UP	0.94	0.26	0	0	0	not significant
8201	BIOCARTA_SODD_PATHWAY	0.94	0.25	0	0	0	not significant
8202	CHR11Q25	0.94	0.25	0	0	0	not significant
8203	CHR17P11	0.94	0.25	0	0	0	not significant
8204	CROONQUIST_STROMAL_STIMULATION_DN	0.94	0.25	0	0	0	not significant
8205	GO_ALDEHYDE_LYASE_ACTIVITY	0.94	0.25	0	0	0	not significant
8206	GO_LAMELLIPODIUM_MEMBRANE	0.94	0.25	0	0	0	not significant
8207	GO_MALE_SEX_DIFFERENTIATION	0.94	0.25	0	0	0	not significant
8208	GO_NEGATIVE_REGULATION_OF_INTRACELLULAR_TRANSPORT	0.94	0.25	0	0	0	not significant
8209	GO_NEGATIVE_REGULATION_OF_PROTEIN_AUTOPHOSPHORYLATION	0.94	0.25	0	0	0	not significant
8210	GO_NOTCH_BINDING	0.94	0.25	0	0	0	not significant
8211	GO_POSITIVE_REGULATION_OF_LYMPHOCYTE_CHEMOTAXIS	0.94	0.25	0	0	0	not significant
8212	GO_REGULATION_OF_SECONDARY_METABOLIC_PROCESS	0.94	0.25	0	0	0	not significant
8213	GO_REGULATION_OF_T_HELPER_CELL_DIFFERENTIATION	0.94	0.25	0	0	0	not significant
8214	KAUFFMANN_MELANOMA_RELAPSE_UP	0.94	0.25	0	0	0	not significant
8215	RAY_TUMORIGENESIS_BY_ERBB2_CDC25A_UP	0.94	0.25	0	0	0	not significant
8216	ROSS_AML_WITH_PML_RARA_FUSION	0.94	0.25	0	0	0	not significant
8217	SIG_PIP3_SIGNALING_IN_B_LYMPHOCYTES	0.94	0.25	0	0	0	not significant
8218	GO_HIGH_VOLTAGE_GATED_CALCIUM_CHANNEL_ACTIVITY	0.94	0.24	0	0	0	not significant
8219	GO_NEGATIVE_REGULATION_OF_SUPRAMOLECULAR_FIBER_ORGANIZATION	0.94	0.24	0	0	0	not significant
8220	GO_REGULATION_OF_EPIDERMAL_GROWTH_FACTOR_ACTIVATED_RECEPTOR_AC	0.94	0.24	0	0	0	not significant
8221	GO_REGULATION_OF_RETINOIC_ACID_RECEPTOR_SIGNALING_PATHWAY	0.94	0.24	0	0	0	not significant
8222	GO_TORC2_COMPLEX	0.94	0.24	0	0	0	not significant
8223	KEGG_ERBB_SIGNALING_PATHWAY	0.94	0.24	0	0	0	not significant
8224	REACTOME_DEX_H_BOX_HELICASES_ACTIVATE_TYPE_I_IFN_AND_INFLAMMATORY	0.94	0.24	0	0	0	not significant
8225	AMUNDSON_POOR_SURVIVAL_AFTER_GAMMA_RADIATION_8G	0.94	0.23	0	0	0	not significant
8226	GO_HORMONE_TRANSPORT	0.94	0.23	0	0	0	not significant
8227	GO_LIGASE_ACTIVITY_FORMING_PHOSPHORIC_ESTER_BONDS	0.94	0.23	0	0	0	not significant
8228	GO_POSITIVE_REGULATION_OF_STRIATED_MUSCLE_CELL_APOPTOTIC_PROCESS	0.94	0.23	0	0	0	not significant
8229	POOLA_INVASIVE_BREAST_CANCER_DN	0.94	0.23	0	0	0	not significant
8230	PUIFFE_INVASION_INHIBITED_BY_ASCITES_DN	0.94	0.23	0	0	0	not significant
8231	GO_NUCLEAR_MEMBRANE	0.94	0.21	0	0	0	not significant
8232	DEURIG_T_CELL_PROLYMPHOCYTIC_LEUKEMIA_UP	0.94	0.2	0	0	0	not significant
8233	GO_DEVELOPMENTAL_PROCESS_INVOLVED_IN_REPRODUCTION	0.94	0.2	0	0	0	not significant
8234	GO_REGULATION_OF_VESICLE_MEDIATED_TRANSPORT	0.94	0.2	0	0	0	not significant
8235	GO_DEVELOPMENTAL_GROWTH	0.94	0.19	0	0	0	not significant
8236	NUYTTEN_EZH2_TARGETS_UP	0.94	0.16	0	0	0	not significant
8237	GO_CELLULAR_RESPONSE_TO_DNA_DAMAGE_STIMULUS	0.94	0.15	0	0	0	not significant
8238	GO_DNA_BINDING_TRANSCRIPTION_FACTOR_ACTIVITY	0.94	0.14	0	0	0	not significant
8239	GO_NEGATIVE_REGULATION_OF_BIOSYNTHETIC_PROCESS	0.94	0.13	0	0	0	not significant
8240	GO_DIGESTIVE_SYSTEM_PROCESS	0.95	0.3	0	0	0	not significant
8241	AMIT_EGF_RESPONSE_20_MCF10A	0.95	0.29	0	0	0	not significant
8242	GO_PROGRAMMED_NECROTIC_CELL_DEATH	0.95	0.29	0	0	0	not significant
8243	BIOCARTA_MAPK_PATHWAY	0.95	0.28	0	0	0	not significant
8244	CHIANG_LIVER_CANCER_SUBCLASS_UNANNOTATED_UP	0.95	0.28	0	0	0	not significant
8245	CHR6Q27	0.95	0.28	0	0	0	not significant
8246	DER_IFN_GAMMA_RESPONSE_DN	0.95	0.28	0	0	0	not significant
8247	GO_BONE_RESORPTION	0.95	0.28	0	0	0	not significant
8248	GO_CILIARY_MEMBRANE	0.95	0.28	0	0	0	not significant
8249	GO_COFACTOR_CATABOLIC_PROCESS	0.95	0.28	0	0	0	not significant
8250	GO_ENDOCRINE_PANCREAS_DEVELOPMENT	0.95	0.28	0	0	0	not significant
8251	GO_MYELOID_CELL_APOPTOTIC_PROCESS	0.95	0.28	0	0	0	not significant
8252	GO_POSITIVE_REGULATION_OF_ALPHA_BETA_T_CELL_DIFFERENTIATION	0.95	0.28	0	0	0	not significant
8253	GO_POSITIVE_REGULATION_OF_PROTEIN_KINASE_B_SIGNALING	0.95	0.28	0	0	0	not significant
8254	GO_REGULATION_OF_T_CELL_APOPTOTIC_PROCESS	0.95	0.28	0	0	0	not significant
8255	GO_REGULATION_OF_TYPE_2_IMMUNE_RESPONSE	0.95	0.28	0	0	0	not significant
8256	GO_RESPONSE_TO_ISCHEMIA	0.95	0.28	0	0	0	not significant
8257	KYNG_DNA_DAMAGE_BY_UV	0.95	0.28	0	0	0	not significant
8258	PID_ARF6_PATHWAY	0.95	0.28	0	0	0	not significant
8259	REACTOME_O_LINKED_GLYCOSYLATION	0.95	0.28	0	0	0	not significant
8260	REACTOME_SULFUR_AMINO_ACID_METABOLISM	0.95	0.28	0	0	0	not significant
8261	SARTIPY_BLUNTED_BY_INSULIN_RESISTANCE_DN	0.95	0.28	0	0	0	not significant
8262	BCAT_GDS748_UP	0.95	0.27	0	0	0	not significant
8263	CHR18Q23	0.95	0.27	0	0	0	not significant
8264	DACOSTA_LOW_DOSE_UV_RESPONSE_VIA_ERCC3_XPCS_UP	0.95	0.27	0	0	0	not significant
8265	GO_ACTIN_FILAMENT_SEVERING	0.95	0.27	0	0	0	not significant
8266	GO_AXON_EXTENSION	0.95	0.27	0	0	0	not significant
8267	GO_BRANCH_ELONGATION_OF_AN_EPITHELIUM	0.95	0.27	0	0	0	not significant
8268	GO_HIPPO_SIGNALING	0.95	0.27	0	0	0	not significant
8269	GO_MAP_KINASE_SCAFFOLD_ACTIVITY	0.95	0.27	0	0	0	not significant
8270	GO_MITOCHONDRIAL_OUTER_MEMBRANE_PERMEABILIZATION	0.95	0.27	0	0	0	not significant
8271	GO_NEGATIVE_REGULATION_OF_CELLULAR_RESPONSE_TO_GROWTH_FACTOR_S	0.95	0.27	0	0	0	not significant
8272	GO_NEGATIVE_REGULATION_OF_EXTRINSIC_APOPTOTIC_SIGNALING_PATHWAY	0.95	0.27	0	0	0	not significant

8273	GO_NEGATIVE_REGULATION_OF_RESPONSE_TO_EXTRACELLULAR_STIMULUS	0.95	0.27	0	0	0	not significant
8274	GO_OVULATION	0.95	0.27	0	0	0	not significant
8275	GO_OXIDOREDUCTASE_ACTIVITY_ACTING_ON_A_SULFUR_GROUP_OF_DONORS_C	0.95	0.27	0	0	0	not significant
8276	GO_POSITIVE_REGULATION_OF_VASCULAR_ENDOTHELIAL_GROWTH_FACTOR_RE	0.95	0.27	0	0	0	not significant
8277	GO_REGULATION_OF_PEPTIDYL_LYSINE_ACETYLATION	0.95	0.27	0	0	0	not significant
8278	GO_TOXIN_METABOLIC_PROCESS	0.95	0.27	0	0	0	not significant
8279	GO_TRANSFERASE_ACTIVITY_TRANSFERRING_NITROGENOUS_GROUPS	0.95	0.27	0	0	0	not significant
8280	HOEGERKORP_CD44_TARGETS_TEMPORAL_DN	0.95	0.27	0	0	0	not significant
8281	IL15_UP.V1_UP	0.95	0.27	0	0	0	not significant
8282	PID_P53_DOWNSTREAM_PATHWAY	0.95	0.27	0	0	0	not significant
8283	REACTOME_TNFS_BIND_THEIR_PHYSIOLOGICAL_RECEPTORS	0.95	0.27	0	0	0	not significant
8284	SESTO_RESPONSE_TO_UV_C8	0.95	0.27	0	0	0	not significant
8285	GO_ANKYRIN_BINDING	0.95	0.26	0	0	0	not significant
8286	GO_AUTOLYSOSOME	0.95	0.26	0	0	0	not significant
8287	GO_CARBOHYDRATE_TRANSMEMBRANE_TRANSPORTER_ACTIVITY	0.95	0.26	0	0	0	not significant
8288	GO_CARBOHYDRATE_TRANSPORT	0.95	0.26	0	0	0	not significant
8289	GO_CELL_MATURATION	0.95	0.26	0	0	0	not significant
8290	GO_CELLULAR_RESPONSE_TO_X_RAY	0.95	0.26	0	0	0	not significant
8291	GO_DISTAL_AXON	0.95	0.26	0	0	0	not significant
8292	GO_G_QUADRUPLEX_RNA_BINDING	0.95	0.26	0	0	0	not significant
8293	GO_GLYCEROLIPID_BIOSYNTHETIC_PROCESS	0.95	0.26	0	0	0	not significant
8294	GO_INTRA_S_DNA_DAMAGE_CHECKPOINT	0.95	0.26	0	0	0	not significant
8295	GO_NEGATIVE_REGULATION_OF_INTRACELLULAR_PROTEIN_TRANSPORT	0.95	0.26	0	0	0	not significant
8296	GO_NEGATIVE_REGULATION_OF_RESPONSE_TO_OXIDATIVE_STRESS	0.95	0.26	0	0	0	not significant
8297	GO_PERK_MEDIATED_UNFOLDED_PROTEIN_RESPONSE	0.95	0.26	0	0	0	not significant
8298	GO_POSITIVE_REGULATION_OF_COLD_INDUCED_THERMOGENESIS	0.95	0.26	0	0	0	not significant
8299	GO_POSITIVE_REGULATION_OF_PROTEIN_ACETYLATION	0.95	0.26	0	0	0	not significant
8300	GO_PROTEIN_AUTOPROCESSING	0.95	0.26	0	0	0	not significant
8301	GO_PROTON_TRANSMEMBRANE_TRANSPORT	0.95	0.26	0	0	0	not significant
8302	GO_PROTON_TRANSPORTING_V_TYPE_ATPASE_COMPLEX	0.95	0.26	0	0	0	not significant
8303	GO_REGULATION_OF_DENDRITE_MORPHOGENESIS	0.95	0.26	0	0	0	not significant
8304	GO_RNA_POLYMERASE_II_C_TERMINAL_DOMAIN_BINDING	0.95	0.26	0	0	0	not significant
8305	GO_SMOOTH_ENDOPLASMIC_RETICULUM	0.95	0.26	0	0	0	not significant
8306	GO_VERY_LONG_CHAIN_FATTY_ACID_METABOLIC_PROCESS	0.95	0.26	0	0	0	not significant
8307	HERNANDEZ_MITOTIC_ARREST_BY_DOCETAXEL_2_UP	0.95	0.26	0	0	0	not significant
8308	MELLMAN_TUT1_TARGETS_DN	0.95	0.26	0	0	0	not significant
8309	MISHRA_CARCINOMA_ASSOCIATED_FIBROBLAST_DN	0.95	0.26	0	0	0	not significant
8310	NIKOLSKY_BREAST_CANCER_11Q12_Q14_AMPLICON	0.95	0.26	0	0	0	not significant
8311	PID_PDGFRB_PATHWAY	0.95	0.26	0	0	0	not significant
8312	REACTOME_ASPARTATE_AND_ASPARAGINE_METABOLISM	0.95	0.26	0	0	0	not significant
8313	WOOD_EBV_EBNA1_TARGETS_UP	0.95	0.26	0	0	0	not significant
8314	ZHANG_GATA6_TARGETS_UP	0.95	0.26	0	0	0	not significant
8315	GAL_LEUKEMIC_STEM_CELL_UP	0.95	0.25	0	0	0	not significant
8316	GAUSSMANN_MLL_AF4_FUSION_TARGETS_C_UP	0.95	0.25	0	0	0	not significant
8317	GO_CELLULAR_RESPONSE_TO_RADIATION	0.95	0.25	0	0	0	not significant
8318	GO_CYTOKINE_RECEPTOR_BINDING	0.95	0.25	0	0	0	not significant
8319	GO_DEVELOPMENTAL_GROWTH_INVOLVED_IN_MORPHOGENESIS	0.95	0.25	0	0	0	not significant
8320	GO_GPI_ANCHOR_TRANSAMIDASE_ACTIVITY	0.95	0.25	0	0	0	not significant
8321	GO_HEART_MORPHOGENESIS	0.95	0.25	0	0	0	not significant
8322	GO_LYSINE_CATABOLIC_PROCESS	0.95	0.25	0	0	0	not significant
8323	GO_NEGATIVE_REGULATION_OF_VASOCONSTRICTION	0.95	0.25	0	0	0	not significant
8324	GO_RECEPTOR_ANTAGONIST_ACTIVITY	0.95	0.25	0	0	0	not significant
8325	GO_TRIGLYCERIDE_LIPASE_ACTIVITY	0.95	0.25	0	0	0	not significant
8326	HALLMARK_COMPLEMENT	0.95	0.25	0	0	0	not significant
8327	LIM_MAMMARY_STEM_CELL_UP	0.95	0.25	0	0	0	not significant
8328	PETRETTO_BLOOD_PRESSURE_DN	0.95	0.25	0	0	0	not significant
8329	REACTOME_SIGNALING_BY_NOTCH2	0.95	0.25	0	0	0	not significant
8330	GO_PEPTIDYL_LYSINE_METHYLATION	0.95	0.24	0	0	0	not significant
8331	GO_REGULATION_OF_CELL_MORPHOGENESIS_INVOLVED_IN_DIFFERENTIATION	0.95	0.24	0	0	0	not significant
8332	REN_ALVEOLAR_RHABDOMYOSARCOMA_DN	0.95	0.24	0	0	0	not significant
8333	SWEET_LUNG_CANCER_KRAS_DN	0.95	0.24	0	0	0	not significant
8334	GO_REGULATION_OF_CELL_MORPHOGENESIS	0.95	0.23	0	0	0	not significant
8335	BONOME_OVARIAN_CANCER_SURVIVAL_SUBOPTIMAL_DEBULKING	0.95	0.21	0	0	0	not significant
8336	GO_HYDROLASE_ACTIVITY_ACTING_ON_ESTER_BONDS	0.95	0.2	0	0	0	not significant
8337	GO_TRANSCRIPTION_FACTOR_BINDING	0.95	0.2	0	0	0	not significant
8338	GO_CELL_JUNCTION	0.95	0.19	0	0	0	not significant
8339	BOYLAN_MULTIPLE_MYELOMA_PCA3_DN	0.96	0.31	0	0	0	not significant
8340	GO_CYTOPLASMIC_PATTERN_RECOGNITION_RECEPTOR_SIGNALING_PATHWAY_IN	0.96	0.31	0	0	0	not significant
8341	GO_PEPTIDYL_L_CYSTEINE_S_PALMITOYLATION	0.96	0.3	0	0	0	not significant
8342	GO_POSITIVE_REGULATION_OF_PROTEIN_TARGETING_TO_MEMBRANE	0.96	0.3	0	0	0	not significant
8343	GO_TRANSCRIPTION_COREPRESSOR_BINDING	0.96	0.3	0	0	0	not significant
8344	GO_TRANSLATION_REPRESSOR_ACTIVITY	0.96	0.3	0	0	0	not significant
8345	KEGG_VALINE_LEUCINE_AND_ISOLEUCINE_DEGRADATION	0.96	0.3	0	0	0	not significant
8346	PID_PI3K_PLC_TRK_PATHWAY	0.96	0.3	0	0	0	not significant
8347	ZHENG_GLIOBLASTOMA_PLASTICITY_DN	0.96	0.3	0	0	0	not significant
8348	DER_IFN_BETA_RESPONSE_UP	0.96	0.29	0	0	0	not significant
8349	GO_ARMADILLO_REPEAT_DOMAIN_BINDING	0.96	0.29	0	0	0	not significant
8350	GO_ESTABLISHMENT_OF_PROTEIN_LOCALIZATION_TO_VACUOLE	0.96	0.29	0	0	0	not significant
8351	GO_G1_DNA_DAMAGE_CHECKPOINT	0.96	0.29	0	0	0	not significant
8352	GO_GOLGI_TO_ENDOSOME_TRANSPORT	0.96	0.29	0	0	0	not significant
8353	GO_KETONE_BIOSYNTHETIC_PROCESS	0.96	0.29	0	0	0	not significant
8354	GO_MICROVILLUS	0.96	0.29	0	0	0	not significant
8355	GO_NEGATIVE_REGULATION_OF_GTPASE_ACTIVITY	0.96	0.29	0	0	0	not significant
8356	GO_NEGATIVE_REGULATION_OF_TYPE_I_INTERFERON_MEDIATED_SIGNALING_PAT	0.96	0.29	0	0	0	not significant
8357	GO_REGULATION_OF_CELLULAR_EXTRAVASATION	0.96	0.29	0	0	0	not significant
8358	GO_REGULATION_OF_DNA_REPLICATION	0.96	0.29	0	0	0	not significant
8359	KEGG_AXON_GUIDANCE	0.96	0.29	0	0	0	not significant
8360	LI_WILMS_TUMOR_VS_FETAL_KIDNEY_2_DN	0.96	0.29	0	0	0	not significant
8361	RUAN_RESPONSE_TO_TNF_DN	0.96	0.29	0	0	0	not significant
8362	SCHUETZ_BREAST_CANCER_DUCTAL_INVASIVE_DN	0.96	0.29	0	0	0	not significant
8363	ZHAN_MULTIPLE_MYELOMA_CD1_DN	0.96	0.29	0	0	0	not significant
8364	BANDRES_RESPONSE_TO_CARMUSTIN_MGMT_48HR_DN	0.96	0.28	0	0	0	not significant
8365	BROWNE_HCMV_INFECTION_2HR_DN	0.96	0.28	0	0	0	not significant
8366	CHEOK_RESPONSE_TO_MERCAPTOPURINE_DN	0.96	0.28	0	0	0	not significant
8367	GAVIN_FOXP3_TARGETS_CLUSTER_P4	0.96	0.28	0	0	0	not significant
8368	GO_ATPASE_ACTIVITY_COUPLED_TO_TRANSMEMBRANE_MOVEMENT_OF_IONS_R	0.96	0.28	0	0	0	not significant
8369	GO_EPIDERMIS_MORPHOGENESIS	0.96	0.28	0	0	0	not significant
8370	GO_HOPS_COMPLEX	0.96	0.28	0	0	0	not significant
8371	GO_INFLAMMATORY_CELL_APOPTOTIC_PROCESS	0.96	0.28	0	0	0	not significant
8372	GO_INTRACELLULAR_STEROL_TRANSPORT	0.96	0.28	0	0	0	not significant
8373	GO_MAGNESIUM_DEPENDENT_PROTEIN_SERINE_THREONINE_PHOSPHATASE_ACTI	0.96	0.28	0	0	0	not significant
8374	GO_MONOSACCHARIDE_BINDING	0.96	0.28	0	0	0	not significant
8375	GO_NEGATIVE_REGULATION_OF_AXONOGENESIS	0.96	0.28	0	0	0	not significant
8376	GO_NEGATIVE_REGULATION_OF_DNA_DEPENDENT_DNA_REPLICATION	0.96	0.28	0	0	0	not significant
8377	GO_NEGATIVE_REGULATION_OF_T_HELPER_CELL_DIFFERENTIATION	0.96	0.28	0	0	0	not significant
8378	GO_NUCLEOSIDE_CATABOLIC_PROCESS	0.96	0.28	0	0	0	not significant
8379	GO_PHAGOSOME_ACIDIFICATION	0.96	0.28	0	0	0	not significant
8380	GO_PHOSPHATIDYLINOSITOL_BIOSYNTHETIC_PROCESS	0.96	0.28	0	0	0	not significant
8381	GO_POSITIVE_REGULATION_OF_PHOSPHATIDYLINOSITOL_3_KINASE_SIGNALING	0.96	0.28	0	0	0	not significant

8382	GO_REGULATION_OF_AUTOPHAGY_OF_MITOCHONDRION_IN_RESPONSE_TO_MITC	0.96	0.28	0	0	0	not significant
8383	GO_REGULATION_OF_RESPONSE_TO_WOUNDING	0.96	0.28	0	0	0	not significant
8384	HOFMANN_MYELODYSPLASTIC_SYNDROM_HIGH_RISK_DN	0.96	0.28	0	0	0	not significant
8385	KEGG_RIG_I_LIKE_RECEPTOR_SIGNALING_PATHWAY	0.96	0.28	0	0	0	not significant
8386	KESHELAVA_MULTIPLE_DRUG_RESISTANCE	0.96	0.28	0	0	0	not significant
8387	MARSON_FOXP3_TARGETS_STIMULATED_UP	0.96	0.28	0	0	0	not significant
8388	REACTOME_COMPETING_ENDOGENOUS_RNAS_CERNAS_REGULATE_PTEN_TRANS	0.96	0.28	0	0	0	not significant
8389	REACTOME_PROCESSING_OF_DNA_DOUBLE_STRAND_BREAK_ENDS	0.96	0.28	0	0	0	not significant
8390	STAEGE_EWING_FAMILY_TUMOR	0.96	0.28	0	0	0	not significant
8391	WENG_POR_TARGETS_LIVER_DN	0.96	0.28	0	0	0	not significant
8392	CHRYQ11	0.96	0.27	0	0	0	not significant
8393	GO_ALPHA_DNA_POLYMERASE_PRIMASE_COMPLEX	0.96	0.27	0	0	0	not significant
8394	GO_APICOLATERAL_PLASMA_MEMBRANE	0.96	0.27	0	0	0	not significant
8395	GO_ASTROCYTE_DEVELOPMENT	0.96	0.27	0	0	0	not significant
8396	GO_ESTABLISHMENT_OF_ENDOTHELIAL_BARRIER	0.96	0.27	0	0	0	not significant
8397	GO_NEUROTRANSMITTER_TRANSPORT	0.96	0.27	0	0	0	not significant
8398	GO_OXIDIZED_DNA_BINDING	0.96	0.27	0	0	0	not significant
8399	GO_POSITIVE_REGULATION_OF_SYNAPTIC_VESICLE_RECYCLING	0.96	0.27	0	0	0	not significant
8400	GO_POSITIVE_REGULATION_OF_VIRAL_ENTRY_INTO_HOST_CELL	0.96	0.27	0	0	0	not significant
8401	GO_PROTEIN_HYDROXYLATION	0.96	0.27	0	0	0	not significant
8402	GO_REGULATION_OF_APPETITE	0.96	0.27	0	0	0	not significant
8403	GO_REGULATION_OF_DEACETYLASE_ACTIVITY	0.96	0.27	0	0	0	not significant
8404	GO_RESPONSE_TO_FOLIC_ACID	0.96	0.27	0	0	0	not significant
8405	GO_SECONDARY_LYSOSOME	0.96	0.27	0	0	0	not significant
8406	KEGG_PROSTATE_CANCER	0.96	0.27	0	0	0	not significant
8407	LIEN_BREAST_CARCINOMA_METAPLASTIC	0.96	0.27	0	0	0	not significant
8408	REACTOME_DOWNSTREAM_SIGNAL_TRANSDUCTION	0.96	0.27	0	0	0	not significant
8409	STEGER_ADIPOGENESIS_UP	0.96	0.27	0	0	0	not significant
8410	VANHARANTA_UTERINE_FIBROID_WITH_7Q_DELETION_DN	0.96	0.27	0	0	0	not significant
8411	BASSO_HAIRY_CELL_LEUKEMIA_UP	0.96	0.26	0	0	0	not significant
8412	GO_G_PROTEIN_COUPLED_RECEPTOR_SIGNALING_PATHWAY_INVOLVED_IN_HEAR	0.96	0.26	0	0	0	not significant
8413	GO_HEPARIN_BIOSYNTHETIC_PROCESS	0.96	0.26	0	0	0	not significant
8414	GO_MAP_KINASE_PHOSPHATASE_ACTIVITY	0.96	0.26	0	0	0	not significant
8415	GO_MONOSACCHARIDE_BIOSYNTHETIC_PROCESS	0.96	0.26	0	0	0	not significant
8416	GO_NEGATIVE_REGULATION_OF_MAST_CELL_ACTIVATION	0.96	0.26	0	0	0	not significant
8417	GO_NEGATIVE_REGULATION_OF_PROTEOLYSIS	0.96	0.26	0	0	0	not significant
8418	GO_NUCLEAR_HORMONE_RECEPTOR_BINDING	0.96	0.26	0	0	0	not significant
8419	GO_PHOSPHORYLASE_KINASE_COMPLEX	0.96	0.26	0	0	0	not significant
8420	GO_POSITIVE_REGULATION_OF_PROTEIN_LOCALIZATION_TO_CELL_SURFACE	0.96	0.26	0	0	0	not significant
8421	GO_POSITIVE_THYMIC_T_CELL_SELECTION	0.96	0.26	0	0	0	not significant
8422	GO_REGULATION_OF_FERTILIZATION	0.96	0.26	0	0	0	not significant
8423	OKUMURA_INFLAMMATORY_RESPONSE_LPS	0.96	0.26	0	0	0	not significant
8424	GO_GPI_ANCHOR_TRANSAMIDASE_COMPLEX	0.96	0.25	0	0	0	not significant
8425	SCHAEFFER_PROSTATE_DEVELOPMENT_48HR_DN	0.96	0.25	0	0	0	not significant
8426	GO_NUCLEAR_ENVELOPE	0.96	0.24	0	0	0	not significant
8427	GO_POSITIVE_REGULATION_OF_MUSCLE_ADAPTATION	0.96	0.24	0	0	0	not significant
8428	REACTOME_CYTOKINE_SIGNALING_IN_IMMUNE_SYSTEM	0.96	0.22	0	0	0	not significant
8429	GO_KINASE_BINDING	0.96	0.21	0	0	0	not significant
8430	GO_POSITIVE_REGULATION_OF_RNA_BIOSYNTHETIC_PROCESS	0.96	0.19	0	0	0	not significant
8431	GO_VISUAL_BEHAVIOR	0.97	0.32	0	0	0	not significant
8432	BEGUM_TARGETS_OF_PAX3_FOXO1_FUSION_DN	0.97	0.31	0	0	0	not significant
8433	CHR10P12	0.97	0.31	0	0	0	not significant
8434	GO_CYSTEINE_TYPE_ENDOPEPTIDASE_REGULATOR_ACTIVITY_INVOLVED_IN_APOI	0.97	0.31	0	0	0	not significant
8435	GO_INTRAMOLECULAR_OXIDOREDUCTASE_ACTIVITY	0.97	0.31	0	0	0	not significant
8436	GO_REGULATION_OF_LEUKOCYTE_APOPTOTIC_PROCESS	0.97	0.31	0	0	0	not significant
8437	GO_REGULATION_OF_POTASSIUM_ION_TRANSPORT	0.97	0.31	0	0	0	not significant
8438	GO_RESPONSE_TO_MITOCHONDRIAL_DEPOLARISATION	0.97	0.31	0	0	0	not significant
8439	REACTOME_RAB_REGULATION_OF_TRAFFICKING	0.97	0.31	0	0	0	not significant
8440	REACTOME_SIGNALING_BY_SCF_KIT	0.97	0.31	0	0	0	not significant
8441	BAE_BRCA1_TARGETS_DN	0.97	0.3	0	0	0	not significant
8442	CHR13Q34	0.97	0.3	0	0	0	not significant
8443	CHR5Q12	0.97	0.3	0	0	0	not significant
8444	GO_1_PHOSPHATIDYLINOSITOL_BINDING	0.97	0.3	0	0	0	not significant
8445	GO_CYSTEINE_TYPE_ENDOPEPTIDASE_ACTIVATOR_ACTIVITY_INVOLVED_IN_APOP	0.97	0.3	0	0	0	not significant
8446	GO_LABYRINTHINE_LAYER_DEVELOPMENT	0.97	0.3	0	0	0	not significant
8447	GO_NEGATIVE_REGULATION_OF_SIGNALING_RECEPTOR_ACTIVITY	0.97	0.3	0	0	0	not significant
8448	GO_POSITIVE_REGULATION_OF_LIPID_KINASE_ACTIVITY	0.97	0.3	0	0	0	not significant
8449	GO_POSITIVE_REGULATION_OF_PHOSPHOPROTEIN_PHOSPHATASE_ACTIVITY	0.97	0.3	0	0	0	not significant
8450	GO_RECEPTOR_MEDIATED_ENDOCYTOSIS	0.97	0.3	0	0	0	not significant
8451	ICHIBA_GRAFT_VERSUS_HOST_DISEASE_D7_DN	0.97	0.3	0	0	0	not significant
8452	RAMPON_ENRICHED_LEARNING_ENVIRONMENT_LATE_UP	0.97	0.3	0	0	0	not significant
8453	REACTOME_CYTOSOLIC_SULFONATION_OF_SMALL_MOLECULES	0.97	0.3	0	0	0	not significant
8454	WANG_CLASSIC_ADIPOGENIC_TARGETS_OF_PPARG	0.97	0.3	0	0	0	not significant
8455	ACEVEDO_LIVER_CANCER_WITH_H3K9ME3_UP	0.97	0.29	0	0	0	not significant
8456	BENPORATH_MYC_TARGETS_WITH_EBOX	0.97	0.29	0	0	0	not significant
8457	CHR2Q31	0.97	0.29	0	0	0	not significant
8458	GO_DNA_INTEGRITY_CHECKPOINT	0.97	0.29	0	0	0	not significant
8459	GO_DNA_SECONDARY_STRUCTURE_BINDING	0.97	0.29	0	0	0	not significant
8460	GO_IKAPPAB_KINASE_COMPLEX	0.97	0.29	0	0	0	not significant
8461	GO_LONG_CHAIN_FATTY_ACID_BIOSYNTHETIC_PROCESS	0.97	0.29	0	0	0	not significant
8462	GO_MAMMARY_GLAND_EPITHELIAL_CELL_PROLIFERATION	0.97	0.29	0	0	0	not significant
8463	GO_NEGATIVE_REGULATION_OF_PROTEIN_OLIGOMERIZATION	0.97	0.29	0	0	0	not significant
8464	GO_PHOSPHATIDYLINOSITOL_KINASE_ACTIVITY	0.97	0.29	0	0	0	not significant
8465	GO_PHOSPHATIDYLINOSITOL_PHOSPHATE_5_PHOSPHATASE_ACTIVITY	0.97	0.29	0	0	0	not significant
8466	GO_POSITIVE_REGULATION_OF_ORGANIC_ACID_TRANSPORT	0.97	0.29	0	0	0	not significant
8467	GO_REGULATION_OF_CELL_CYCLE_ARREST	0.97	0.29	0	0	0	not significant
8468	GO_REGULATION_OF_DEFENSE_RESPONSE_TO_VIRUS	0.97	0.29	0	0	0	not significant
8469	GO_RIPOPTOSOME	0.97	0.29	0	0	0	not significant
8470	GO_SIGNAL_RECOGNITION_PARTICLE	0.97	0.29	0	0	0	not significant
8471	GROSS_ELK3_TARGETS_DN	0.97	0.29	0	0	0	not significant
8472	ICHIBA_GRAFT_VERSUS_HOST_DISEASE_D7_UP	0.97	0.29	0	0	0	not significant
8473	KEGG_NATURAL_KILLER_CELL_MEDIATED_CYTOTOXICITY	0.97	0.29	0	0	0	not significant
8474	REACTOME_INHIBITION_OF_REPLICATION_INITIATION_OF_DAMAGED_DNA_BY_RB1_	0.97	0.29	0	0	0	not significant
8475	REACTOME_RUNX3_REGULATES_CDKN1A_TRANSCRIPTION	0.97	0.29	0	0	0	not significant
8476	REACTOME_SIALIC_ACID_METABOLISM	0.97	0.29	0	0	0	not significant
8477	RODWELL_AGING_KIDNEY_NO_BLOOD_DN	0.97	0.29	0	0	0	not significant
8478	SASSON_RESPONSE_TO_FORSKOLIN_DN	0.97	0.29	0	0	0	not significant
8479	VETTER_TARGETS_OF_PRKCA_AND_ETS1_DN	0.97	0.29	0	0	0	not significant
8480	ZHANG_PROLIFERATING_VS_QUIESCENT	0.97	0.29	0	0	0	not significant
8481	CHR6P23	0.97	0.28	0	0	0	not significant
8482	GAUSSMANN_MLL_AF4_FUSION_TARGETS_B_UP	0.97	0.28	0	0	0	not significant
8483	GO_ATTACHMENT_OF_GPI_ANCHOR_TO_PROTEIN	0.97	0.28	0	0	0	not significant
8484	GO_GDP_DISSOCIATION_INHIBITOR_ACTIVITY	0.97	0.28	0	0	0	not significant
8485	GO_GLYCOSPHINGOLIPID_BINDING	0.97	0.28	0	0	0	not significant
8486	GO_MODIFICATION_DEPENDENT_PROTEIN_BINDING	0.97	0.28	0	0	0	not significant
8487	GO_NEGATIVE_REGULATION_OF_B_CELL_APOPTOTIC_PROCESS	0.97	0.28	0	0	0	not significant
8488	GO_NEGATIVE_REGULATION_OF_ERBB_SIGNALING_PATHWAY	0.97	0.28	0	0	0	not significant
8489	GO_NEGATIVE_REGULATION_OF_MYELOID_CELL_APOPTOTIC_PROCESS	0.97	0.28	0	0	0	not significant
8490	GO_REGULATION_OF_MULTICELLULAR_ORGANISM_GROWTH	0.97	0.28	0	0	0	not significant

8491	IIZUKA_LIVER_CANCER_PROGRESSION_L0_L1_DN	0.97	0.28	0	0	0	not significant
8492	REACTOME_CYTOSOLIC_IRON_SULFUR_CLUSTER_ASSEMBLY	0.97	0.28	0	0	0	not significant
8493	VALK_AML_WITH_CEBPA	0.97	0.28	0	0	0	not significant
8494	WOTTON_RUNX_TARGETS_UP	0.97	0.28	0	0	0	not significant
8495	ZHAN_EARLY_DIFFERENTIATION_GENES_DN	0.97	0.28	0	0	0	not significant
8496	BIOCARTA_STAT3_PATHWAY	0.97	0.27	0	0	0	not significant
8497	GO_CELL_MORPHOGENESIS_INVOLVED_IN_DIFFERENTIATION	0.97	0.27	0	0	0	not significant
8498	GO_NEGATIVE_REGULATION_OF_LEUKOCYTE_DEGRANULATION	0.97	0.27	0	0	0	not significant
8499	GO_NEGATIVE_REGULATION_OF_MAST_CELL_ACTIVATION_INVOLVED_IN_IMMUNE	0.97	0.27	0	0	0	not significant
8500	GO_PARAXIAL_MESODERM_MORPHOGENESIS	0.97	0.27	0	0	0	not significant
[truncated: 2,252,922 more chars]
